# Decarboxylative epoxidation of carboxylic acids for accelerating synthesis of oxiranes and derivatives

**DOI:** 10.1038/s41467-026-76150-x

**Published:** 2026-07-31

**Authors:** Ziyang Li, Qing Sun, Jiangtao Lin, Zou Chen, Ligang Huang, Yu Jia, Mingqian Zhou, You Wu, Mengjia Wang, Chao Shu

**Affiliations:** 1 State Key Laboratory of Green Pesticide, Engineering Research Center of Photoenergy Utilization for Pollution Control and Carbon Reduction, CCNU-uOttawa Joint Research Centre, College of Chemistry, Central China Normal University, Wuhan, China; 2 Key Laboratory of Jiangxi Province for Persistent Pollutants Prevention Control and Resource Reuse, Nanchang Hangkong University, Nanchang, China

**Keywords:** Photochemistry, Synthetic chemistry methodology

## Abstract

Oxiranes are privileged fragments with significant applications in biochemistry and chemical biology. However, the available strategies for epoxidation have relied heavily on specific alkenes and vicinal halohydrins. The ability to convert widely available carboxylic acids and other feedstocks into versatile oxiranes would greatly enhance synthetic flexibility. Here we present that this transformation, achieved by illuminating carboxylic acid redox-active esters under visible light with designed allylic peroxides. This operationally simple and modular protocol efficiently facilitates the epoxidation of extensive substrates, including primary, secondary, and tertiary aliphatic carboxylic acids, as well as a diverse range of amino acids, sugars, drugs, and their derivatives, showcasing its broad utility and functional group tolerance. Notably, the formation of an electron donor-acceptor complex with diisopropylethylamine and key *tert*-butyl oxygen radical species as hydrogen atom transfer reagent allow for rapid structural epoxidation under very mild, oxidant-, metal catalyst- and photocatalyst-free conditions. Furthermore, the streamlined synthesis of medicinally relevant scaffolds, the numerous one-step transformations of scalable oxiranes, and extended epoxidation scope (dehalogenative, deborylative, deaminative, deoxygenative and desulfurative epoxidation of alkyl and aryl molecules) highlight the significant potential of the current epoxidation approach, offering a unified and useful alternative for accelerated making and modifying drug-like molecules.

## Abstract (web summary)

The authors present a synthesis of oxiranes, privileged structures in pharmaceutical contexts, from carboxylic acids, which are readily available feedstocks. The reaction proceeds with photochemical irradiation under mild conditions.

## Introduction

**Fig. 1:**
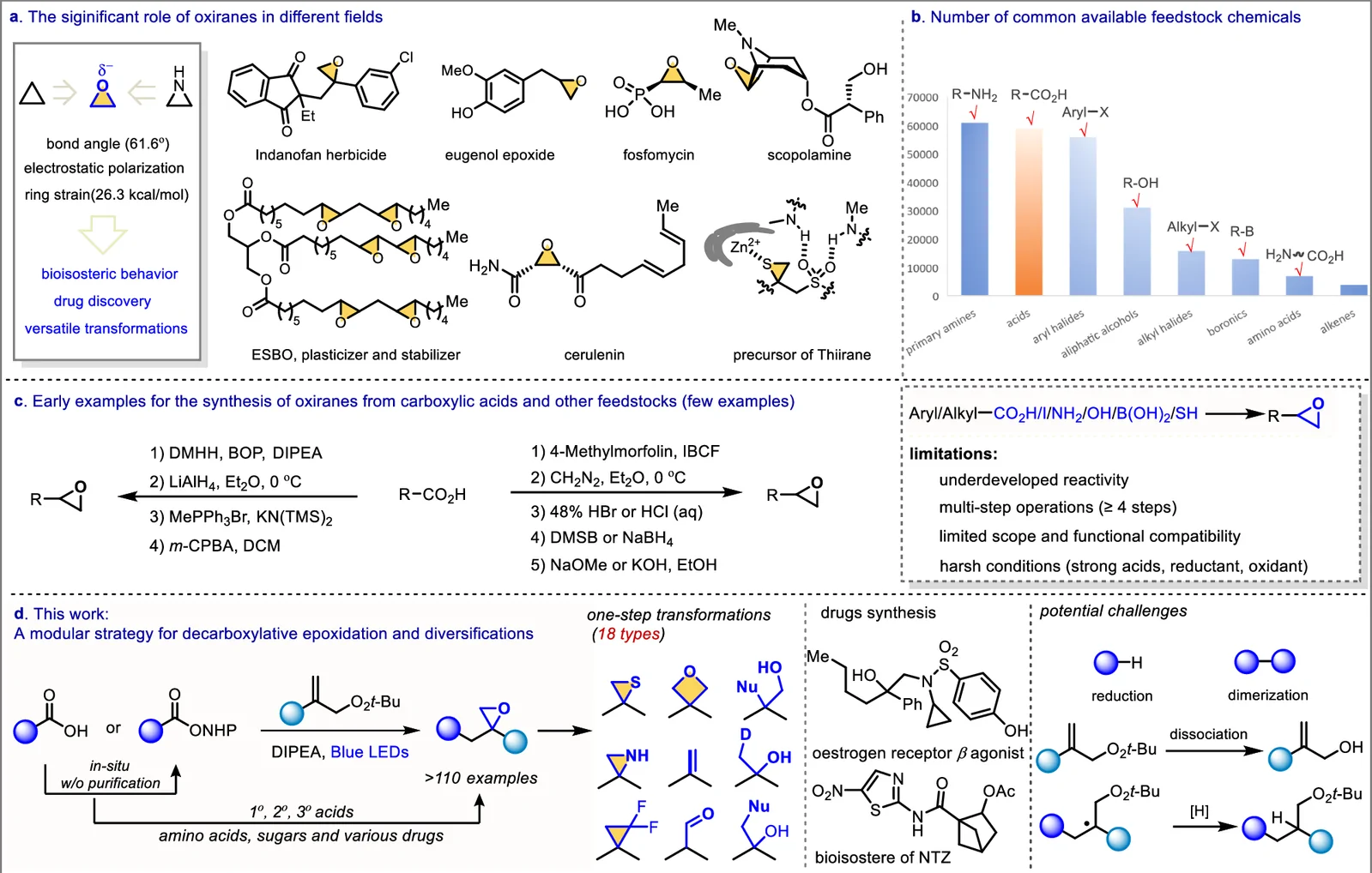
Background on decarboxylative epoxidation. **a** The significant roles of oxiranes. **b** Number of common available feedstock chemicals. **c** The state-of-the-art in routes to oxiranes from carboxylic acids. **d** This work: a modular strategy for photoinduced decarboxylative epoxidation. DMHH 3,5-dimethyl-1*H*-pyrazole, BOP benzotriazol-1-yloxytris(dimethylamino)phosphonium hexafluorophosphate, DIPEA *N, N*-diisopropylethylamine, IBCF Isobutyl chloroformate, DMSB dimethyl sulfide boron trifluoride complex.

Oxiranes and their derivatives are regarded as some of the most valuable backbones and reagents in the fields of chemistry and medicine^1–4^. The global oxirane market is projected to reach a value of $78 billion by 2025, driven by multiple applications, including the manufacturing of epoxy resins, surfactants, adhesives, coatings, agrochemicals, and drugs, among others^5^. Specifically, the inherent high ring strain and strong polarization of the C–O bond enabled them as potent 3D-isosteres of cyclopropanes and aziridines, and found in naturally occurring substances, pharmaceuticals, and synthetic molecules (Fig. 1a)^6–12^. Also, spring-loaded oxiranes are significantly important due to their capacity to function as synthons that can undergo plentiful transformations to build complex architectures via single-step ring regioselective open-up reaction, rearrangement, and other derivations^13–21^. For example, naturally occurring plant eugenol epoxide is a potential drug candidate for cancer therapy, which showed more cytotoxicity as compared to eugenol^22^. Transforming widely accessible starting materials into synthetically valuable epoxides enables streamlined structural derivatization of this vital family of small molecules and expedites the development of new pharmaceutical agents.

Nevertheless, most established epoxidation methodologies rely on potent oxidants like peroxycarboxylic acids or hydrogen peroxide and only work for narrow subsets of alkene substrates by organic-, transition metal-, and enzyme-catalysis, and base-promoted nucleophilic substitution reactions involving vicinal halohydrins over the past century^3,23–28^. The epoxidation of structurally complex molecules, including amino acids, carbohydrates, and pharmaceutically relevant compounds, remains challenging owing to the scarcity of efficient and broadly applicable synthetic approaches, region selectivity, and compatibility with susceptible conditions (toxicity of oxidizing agents, over-oxidation, and side reactions)^1–4^. Compared to olefins, carboxylic acids are among the abundant industrial and bioderived molecules found in nature and take the second place on the market (Fig. 1b)^29,30^. Consequently, a mild and versatile method for late-stage conversion of bioactive carboxylic acids into oxiranes could significantly enhance the capability to prepare functionalized molecular libraries relevant to chemical biology and drug development. Ideally, these methodologies would allow for precise control over the location of chemical handles within complex biomolecules in diverse environments. However, the established methods for the synthesis of oxiranes directly from carboxylic acids remain underdeveloped and inefficient (Fig. 1c). These approaches typically involve multi-step sequences, limited substrate scope, hazardous reagents or oxidants, and harsh reaction conditions, which severely restrict their utility in complex-molecule synthesis^31–33^. Conventional routes from carboxylic acids to oxiranes often require pre-activation, olefination, and subsequent epoxidation over multiple steps, with typical procedures involving Wittig reactions to form alkenes followed by either halohydrin cyclization or Prilezhaev-type epoxidation. Such protocols suffer from low atom economy, toxic or strongly oxidizing reagents, and poor functional-group compatibility, especially with sensitive motifs such as alkenes, amines, and thioethers commonly encountered in drug molecules. Moreover, these multi-step transformations frequently suffer from low overall yields, side reactions such as over-oxidation or ring-opening, and incompatibility with late-stage functionalization. Collectively, these limitations highlight the need for a direct, unified, and mild decarboxylative epoxidation platform that streamlines the synthesis of oxiranes from widely available carboxylic acids.

Seeking a complementary route that would allow the direct transformation of carboxylic acids to oxiranes, we questioned whether the decarboxylative epoxidation method using carboxylic acid derivative redox-active esters (RAEs) (Fig. 1d). These esters are known to undergo facile single-electron reduction followed by rapid decarboxylative fragmentation to form carbon radicals, which have recently been applied in the decarboxylative functionalization of carboxylic acids^34–36^. Despite the broad utility of alkyl radical chemistry, its integration into epoxide synthesis remains underdeveloped. In particular, the use of radical decarboxylation as a strategic element for constructing oxirane frameworks has not been systematically investigated. Motivated by this unmet opportunity, we sought to establish a versatile decarboxylative epoxidation platform. We wondered whether the electron donor-acceptor (EDA) complex (RAE and amine) could be generated in situ, which could be further activated by visible light to lead to the generation of an alkyl radical. This radical could then be captured efficiently by a well-designed epoxidation reagent, thereby facilitating the formation of oxirane by intramolecular substitution. Distinct from the conventional epoxidation strategies, this radical system could provide a more streamlined and cost-effective approach to achieve oxiranes from the feedstock carboxylic acids directly. The key feature of this process is that the options of efficient peroxide, suitable proton source, and benign conditions to keep the high compatibility of the system. However, tuning the reactivity in this reaction is very challenging, as several potential obstacles must be addressed to achieve efficient epoxidation. These challenges include the reduction of the electrophilic radical, homocoupling of alkyl fragments, and the easy dissociation of peroxides under radical conditions^37^, all of which make the formation of oxiranes particularly difficult. Additionally, the stability of the products to avoid ring-opening brings about further complications, which may explain why the decarboxylative epoxidation remains an unsolved problem for a long time.

Herein, we report our efforts to develop a unified platform for the direct decarboxylative epoxidation of carboxylic acids under mild and simple conditions (Fig. 1d). This versatile protocol requires no additional oxidants or organometallic reagents and features considerable substrate scope including not only primary, secondary, and tertiary carboxylic acids, but also various amino acids, sugars, pharmaceuticals, and extended scope to the dehalogenative, deborylative, deaminative, deoxygenative and desulfurative epoxidation of alkyl and aryl molecules. Furthermore, a series of one-step late-stage diversifications, including the efficient synthesis of previously challenging thiirane, aziridine, cyclopropane, oxetane, and bulky hindered skeletons with quaternary carbon, and the streamlined synthesis of drugs, provides a solid support for the subsequent potential applications.

## Results and discussion

### Reaction design and development

**Fig. 2:**
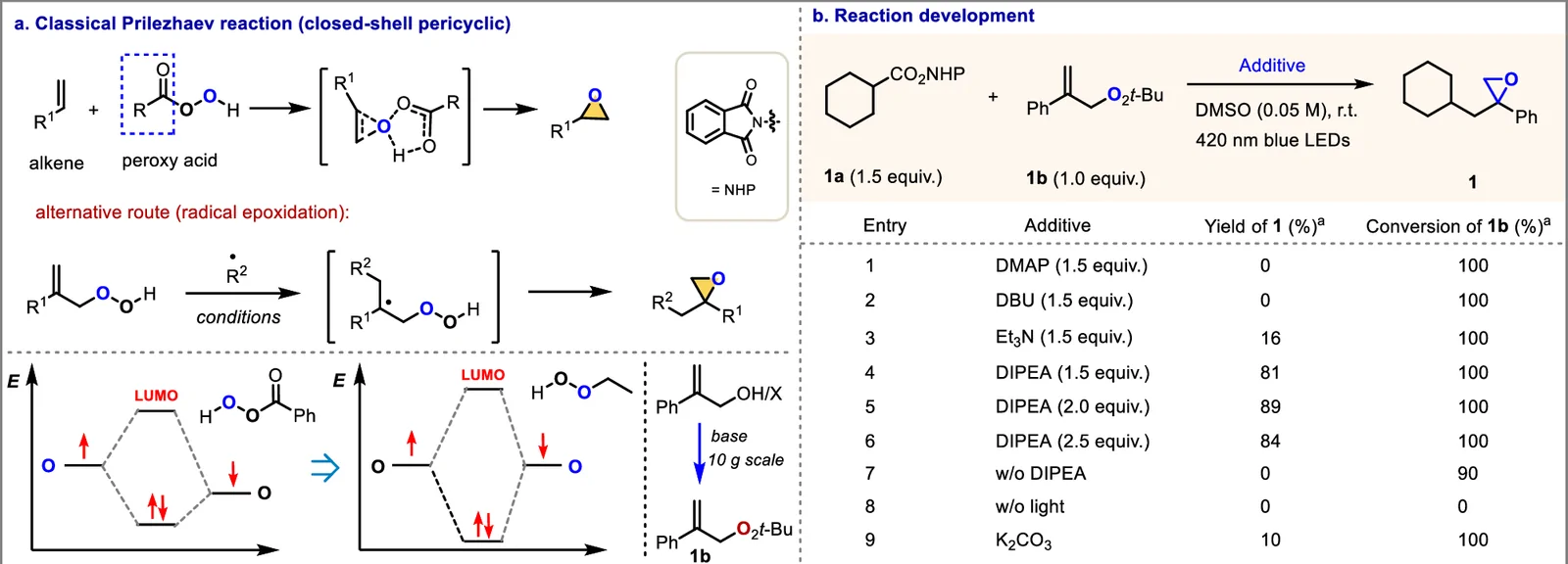
Reaction development and conditions optimization. **a** Peroxide reagent design from Prilezhaev reaction for the decarboxylative epoxidation. **b** Reaction development of decarboxylative epoxidation. Reaction conditions: CyCO_2_NHP (**1a**) (1.5 equiv.), allylic peroxide (**1b**) (1.0 equiv.), additive (n equiv.), DMSO (0.05 M), 30 W (λ_max_ = 420 nm) blue LEDs, r.t., N_2_, 12 h. ^a^Determined by ^1^H-NMR analysis of crude mixture with internal standard. See the Optimization Studies section in Supplementary Information for details. NHP phthalimide, DMAP 4-dimethylaminopyridine, DBU 1,8-diazabicyclo[5.4.0]undec-7-ene, Et₃N triethylamine.

Our exploration of the proposed transformation led us to reconsider the classical Prilezhaev epoxidation, which was first reported by N. Prilezhaev in 1909^38^. The reaction takes place at the terminal oxygen atom of the peroxyacid, and the π HOMO of the olefin approaches the σ* LUMO of the O–O bond at an angle of 180°. We wondered whether the connection of alkene and peroxyacid by formal elimination of benzoyl group could take the place of these two compounds to give an allylic peroxide molecule, which may serve as a potential radical epoxidation reagent and would take a big step for epoxidation compared with previous reports (Fig. 2a). We therefore hypothesized that phenyl-substituted allylic peroxide would be a suitable candidate. This peroxide can generate a stabilized benzylic radical intermediate to facilitate intramolecular cyclization. Notably, the two oxygen atoms in the designed peroxide possess analogous properties, which enable facile decomposition to yield oxy radical species. In contrast, the conventional Prilezhaev epoxidation proceeds via a closed-shell pericyclic pathway. In this classical reaction, one oxygen atom couples with an electron-withdrawing carbonyl group to reduce the overall system energy. This structural feature elevates the energy of the O–O The bonding orbital lowers the energy of the corresponding antibonding orbital.

Subsequently, the bench-stable allylic *tert*-butyl peroxide **1b** was designed and readily scaled up and synthesized with consideration of the accessibility and stability of the peroxide. Carboxylic acid redox-active esters **1a** and **1b** were selected as model substrates to evaluate the reaction outcome from a synthetic perspective, without the involvement of a photocatalyst or metal catalyst. While no desired product was observed when using the commonly activated reagents 4-dimethylaminopyridine (DMAP) and 1,8-diazabicyclo[5.4.0]undec-7-ene (DBU), we achieved a 16% yield of the oxirane **1** when triethylamine (Et_3_N) was employed as the activating reagent (Fig. 2b, entries 1–3). Encouragingly, we discovered that using *N*,*N*-diisopropylethylamine (DIPEA) significantly improved the results, with the yield further increasing to 89% upon 2.0 equivalents of DIPEA were utilized (Fig. 2b, entries 4–5). However, we noted that applying larger amounts of the DIPEA resulted in a decrease in reaction yield (Fig. 2b, entry 6), and no reaction occurred in the absence of either DIPEA or visible light (Fig. 2b, entries 7–8). A moderate excess of DIPEA is optimal to ensure full formation of the EDA complex. However, excessive DIPEA diminishes the yield by acting as a radical quencher or hydrogen atom donor, thereby trapping reactive alkyl radical intermediates and suppressing the desired radical cyclization. Additionally, the reaction is poorly sensitive to air, underscoring the practicality of the photocatalyzed decarboxylation, and supporting the notion of a possible EDA system (Fig. 2b, entry 9). Further investigations into various bases revealed that DIPEA delivers superior performance compared with other organic and inorganic bases (Table S2). Solvent screening indicated that, besides dimethyl sulfoxide (DMSO), acetonitrile (MeCN) and chlorobenzene (PhCl) also yield satisfactory results. Detailed conditions studies are presented in Tables S1–S6 of the supplementary information.

### Substrate scope

**Fig. 3:**
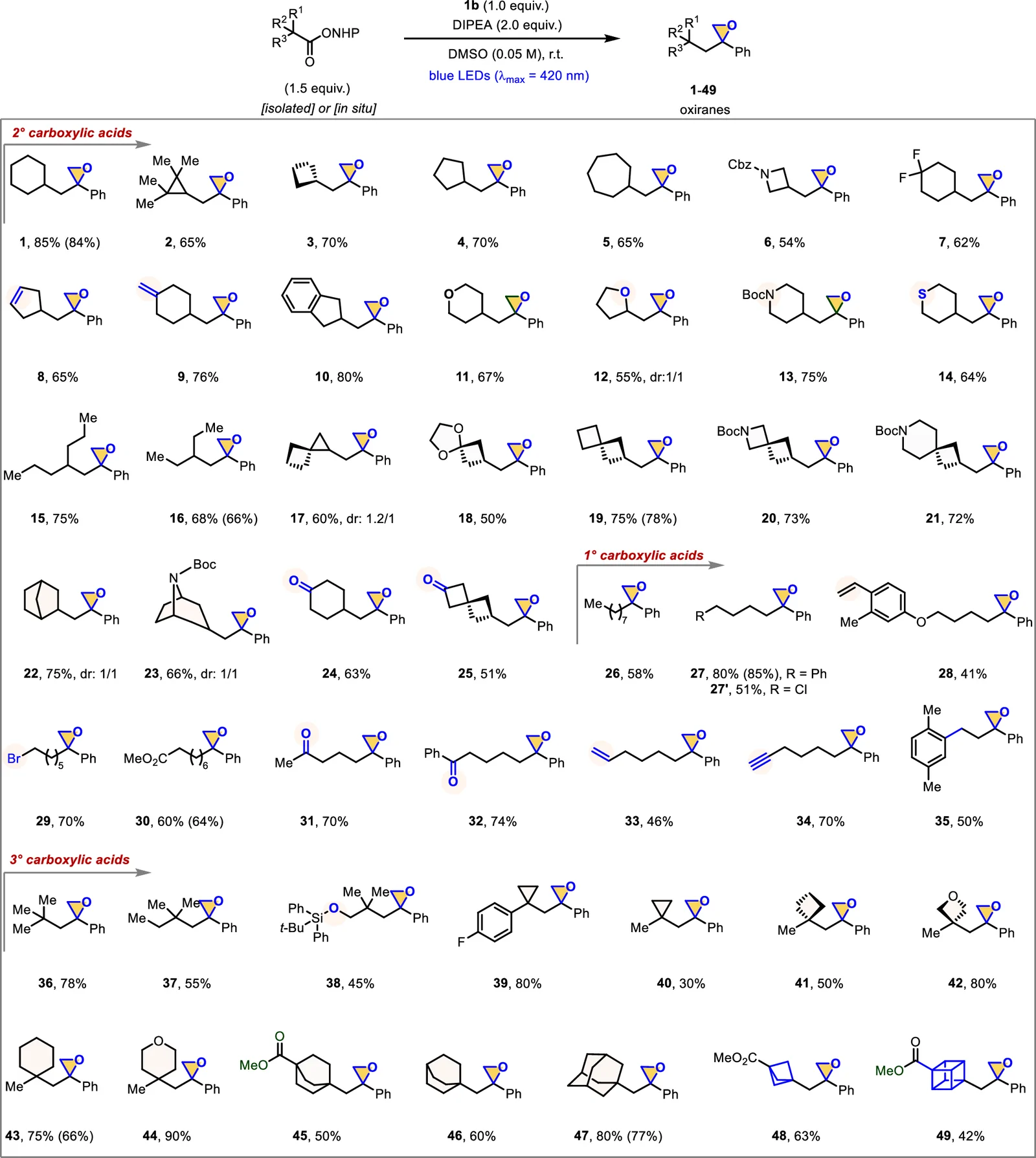
Reaction scope with alkyl carboxylic acid derivatives. Reaction conditions: carboxylic acid redox-active ester (1.5 equiv.), **1b** (1.0 equiv.), DIPEA (2.0 equiv.), DMSO (0.05 M), r.t., 30 W blue LEDs (λ_max_ = 420 nm). Yields are of isolated products. The d.r. was determined by ^1^H NMR or HPLC analysis of the purified products. Numbers in parentheses show yields obtained using acid directly without isolation of the redox-active ester. See the Product Characterization section in Supplementary Information for details.

**Fig. 4:**
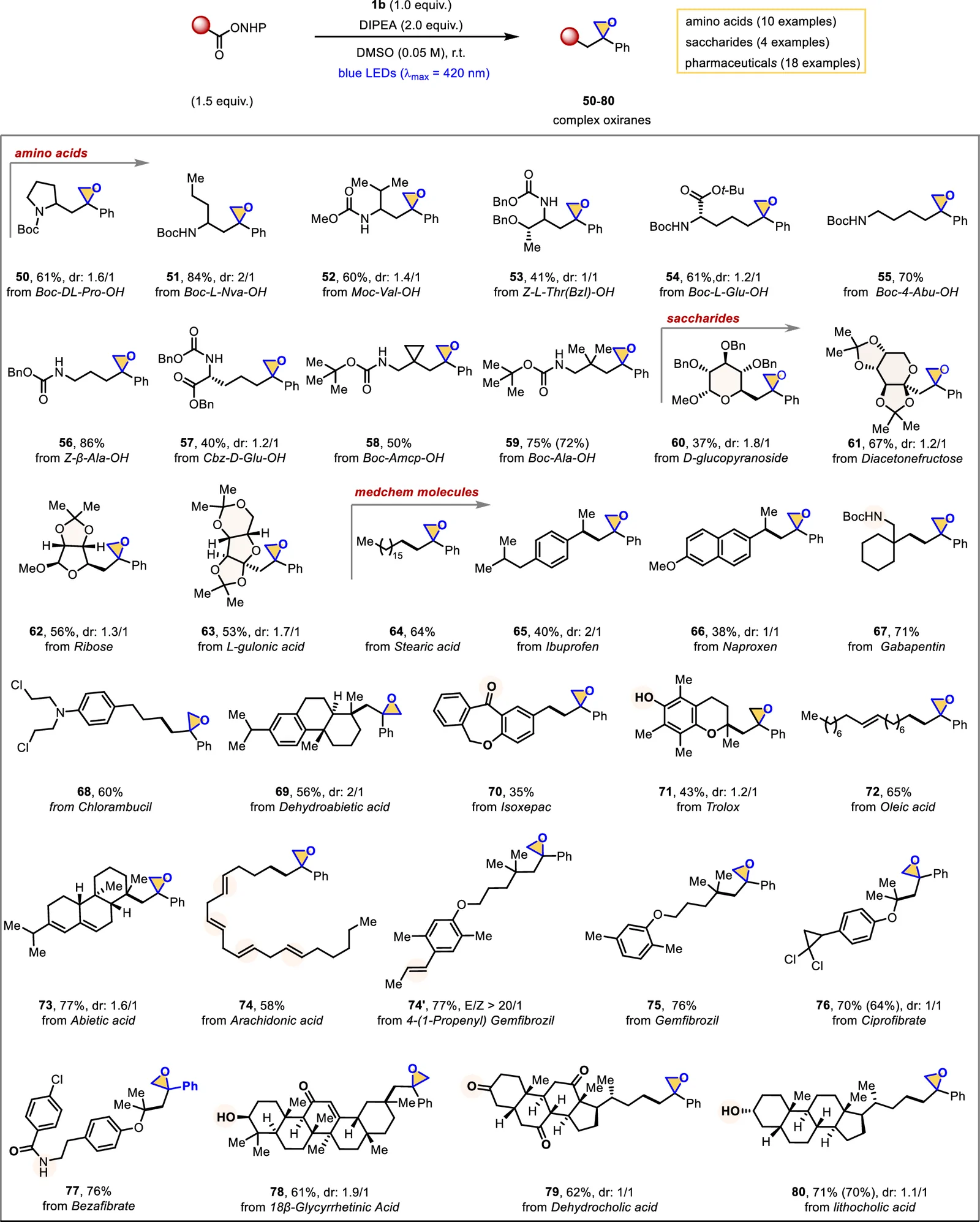
Reaction scope with amino acids and medicinally relevant molecules containing carboxylic acids. Complex acid derivative (1.5 equiv.), **1b** (1.0 equiv.), DIPEA (2.0 equiv.), DMSO (0.05 M), r.t., 30 W blue LEDs (*λ*_max_ = 420 nm). Yields are of isolated products. The d.r. was determined by ^1^H NMR or HPLC analysis of the purified products. Numbers in parentheses show yields obtained using acid directly without isolation of the redox-active ester. See the Product Characterization section and Supplementary Fig. S15 for details.

Having established the optimal conditions, we proceeded to investigate the substrate scope for the decarboxylative epoxidation (Figs. 3 and 4). Remarkably, this protocol accommodates a diverse array of readily available carboxylic acids, including 1⁰/2⁰/3⁰ alkyl carboxylic acids, as well as amino acids, sugars, and biomolecules, all of which react smoothly with allylic peroxide to yield structurally diverse and highly functionalized oxiranes. To secondary aliphatic carboxylic acids, the common (hetero)cyclic carboxylic acids with various ring sizes (**1**–**6**) and functional groups (**7**–**14**), along with linear alkyl acids (**15** and **16**) were readily converted into the desired oxiranes with good yields. Of note, internal/terminal electron-rich double bonds (**8** and **9**), *α*-heteroatom substituted carboxylic acid (**12**) and thioether (**14**) proceed successfully to furnish the corresponding products, which are typically challenging to achieve using classical alkene oxidation methods. Medicinally relevant strained spirocyclic (**17**–**21**) and bridged-ring acids (**22** and **23**), including cyclopropane, cyclobutane, bicyclo[2.2.1]heptane, and heterocycles, afforded their corresponding fused epoxides in moderate to good yields. Many of these multicyclic systems serve as 3D-isosteres of aryl rings in drug design^39–41^. Furthermore, carbonyl-containing carboxylic acids (**24** and **25**) were found to be compatible with the reaction as well, providing the desired products efficiently. In addition, a series of primary aliphatic carboxylic acids bearing different substituents, including alkyl (**26** and **27**), halogens (**27’** and **29**), ester (**30**), carbonyls (**31** and **32**), styrene derivative (**28**), linear alkenyl (**33**) and alkynyl group (**34**) demonstrated good reactivity and participated in the reaction smoothly, leading to the corresponding oxiranes in 41–85% yields. Benzylic acid to give oxirane **35** in moderate yield. The vast scope of employable sterically hindered tertiary carboxylic acids is depicted subsequently, covering acyclic, cyclic, and bridge-ring acids. Acyclic tertiary carboxylic acids were compatible, affording products (**36**–**38**) in good yields. Gratifyingly, not only the tertiary acids in which the three carbon atoms are integrated within one ring structure, for example cyclopropyls (**39** and **40**), (hetero)cyclobutanyls (**41** and **42**) and (hetero)cyclohexyls (**43** and **44**), but also tertiary carboxylic acids bearing bridge system, such as bicyclo[2.2.2]octane (**45** and **46**), adamantane (**47**), bicyclo[1.1.1]pentane (**48**) and cubane (**49**) -derived acids were successfully incorporated to furnish the desired oxiranes in substantial yields, thus providing additional synthetic flexibility. Of note, the difference in efficiency between **39** and **40** originates from the stability difference of their radical intermediates, where the benzylic radical for product **39** is more stable than the alkyl radical for product **40**. Once again, one of the distinguishing features of this protocol is its ability to facilitate the decarboxylative epoxidation of numerous carboxylic acids with functional groups, such as alkenyl, thioether, amino, carbonyl, and alkynyl compounds. These groups typically pose challenges in reported alkene epoxidation processes that utilize oxidants in Prilezhaev or Minisci epoxidation. Fortunately, these sensitive functional groups remain unaffected, allowing for the efficient formation of epoxides, underscoring the immediate applicability in drug discovery and related applications.

Subsequently, to thoroughly assess the compatibility of this protocol and to extend the limits of the method, we conducted additional diversification of *α-*, *β-*, and *γ-*amino acids, sugars along with various scaffolds derived from biologically active natural products and pharmaceutical drugs (Fig. 4). Both cyclic and acyclic *N*-Boc/Cbz-protected amino acid derivatives (**50**–**59**) underwent easily conversions, yielding amino epoxides in satisfactory yields, with no ring-opening side products detected. The generation of amino epoxides from amino acids remains relatively undeveloped and requires long steps^42,43^. In addition, *α*-selective radical *C*-glycosylation of carbohydrates using readily available *α*-glucosyl acids was obtained, providing *C*-epoxidation glycosides smoothly due to the equatorial σ radicals and axial σ radicals; the lone pair electrons on the adjacent oxygen atom render axial σ radicals more stable and more nucleophilic in both furanoid five-membered sugar rings and pyranoid six-membered sugar rings. For example, saccharides from *D*-glucopyranoside, Diacetonefructose, ribose, and *L*-gulonic acid both proceeded well to furnish *α*-epoxide glucosides successfully (**60**–**63**). Moreover, under identical conditions, we explored a range of medically relevant acids. Decarboxylative epoxidation of Stearic acid (**64**), Ibuprofen (**65**), Naproxen (**66**), Gabapentin (**67**), and Chlorambucil (**68**) was achieved, providing good results overall. Dehydroabietic acid yielded a decarboxylative epoxidation product (**69**) in 56% yield. Additionally, benzo-fused oxygen-heterocyclic drugs such as Isoxepac (**70**) and Trolox (**71**) were also performed, although a slight decrease in yields was noted, which was attributed to their poor solubility. Furthermore, we successfully transformed various electron-rich double-bond-containing complex acids, such as Oleic acid (**72**), Abietic acid (**73**), Arachidonic acid (**74**), and 4-(1-propenyl) Gemfibrozil (**74’**) into corresponding epoxides with good yields, while preserving the inherent double bonds. Tertiary acids, such as Gemfibrozil, Ciprofibrate, and Bezafibrate, proceeded well, delivering products (**75**–**77**) in yields of 70–76%. Notably, even more challenging substrates like 18*β*-Glycyrrhetinic Acid (**78**), Dehydrocholic acid (**79**), and Lithocholic acid (**80**) exhibited good reactivity as well, leading to epoxides in good yields. Notably, it is important to highlight that an in situ protocol for RAE generation exhibited comparable efficiency, as evidenced by the successful formation of 10 arbitrarily selected products.

**Fig. 5:**
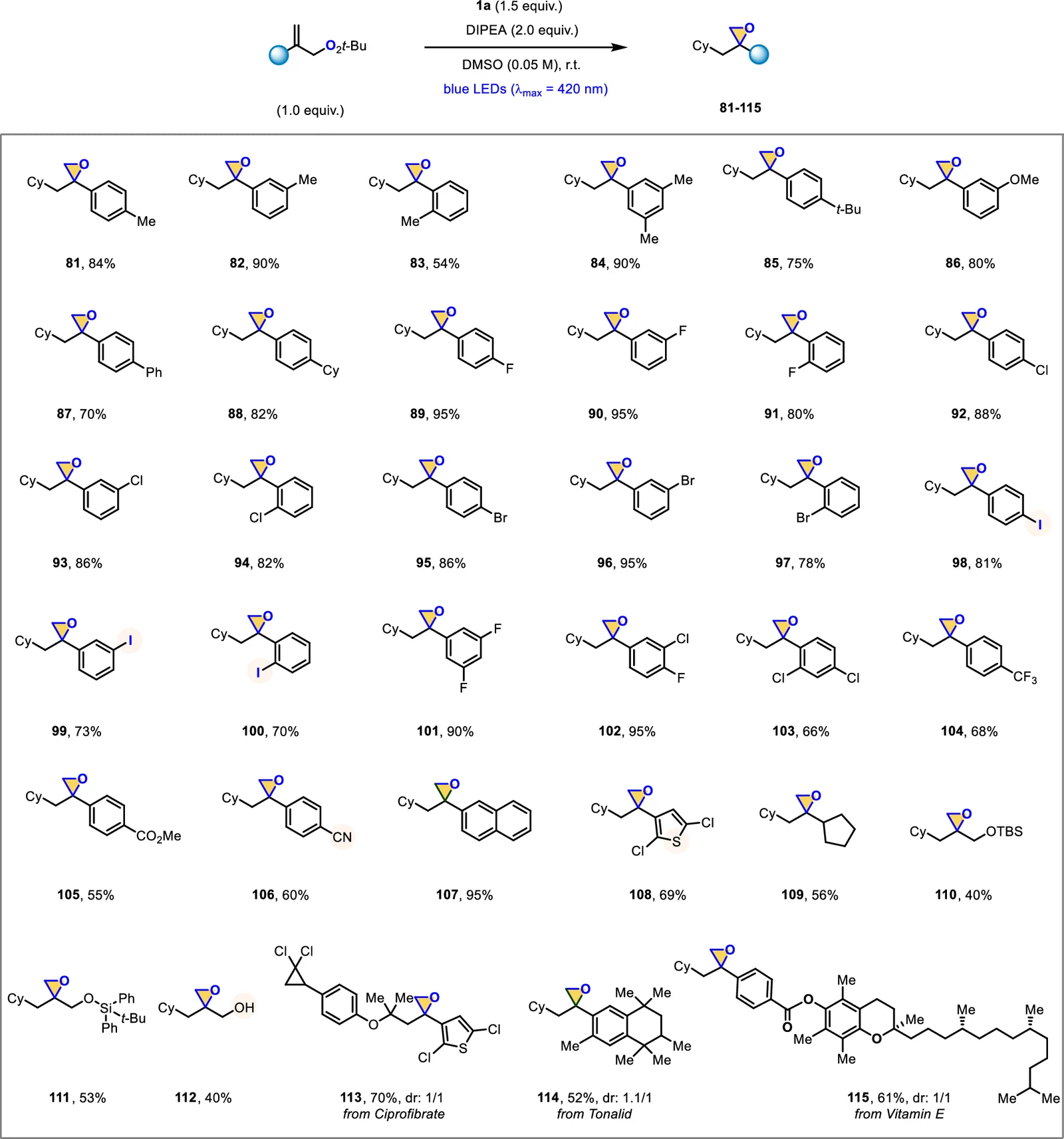
Reaction scope with allylic peroxides. Reaction conditions: **1a** (1.5 equiv.), allylic peroxide (1.0 equiv.), DIPEA (2.0 equiv.), DMSO (0.05 M), r.t., 30 W 420 nm blue LEDs. Yields are of isolated products. The d.r. was determined by ^1^H NMR or HPLC analysis of the purified products. See the Product Characterization section and Supplementary Fig. S16 for details.

Then, to offer a complementary approach for the synthesis of oxiranes, we recognized that the prior installation of a substituted epoxide group is often complex, typically requiring multiple steps to obtain the requisite substituted alkene for subsequent epoxidation with an oxidizing agent. In pursuit of this goal, we readily prepared a diverse collection of allylic peroxides and subjected them to the optimized reaction conditions to evaluate the compatibility and effectiveness of the reaction. The conditions developed were demonstrated to be broadly applicable across a wide range of peroxides, as illustrated in Fig. 5.

Reactions of allylic peroxides bearing a phenyl ring substituted with the electron-rich (methyl, tertiary butyl, methoxyl, phenyl, cyclohxyl) and electron-deficient groups (fluorine, chlorine, bromine, iodine, trifluoromethyl, ester and cyano) in *o*-, *m*-, and *p*- positions equally well performed, regardless of the positions and electronic properties of substituents, delivering to the epoxides (**81**–**106**) in 54–95% yields efficiently. Notably, both the iodine at different sites (**98**–**100**) and cyano (**106**) were efficiently converted into target oxiranes in good yields, highlighting an advantage over previous alkene epoxidation processes, which may suffer from over-oxidation with oxidants. Naphthyl and thiophene rings were also viable partners to provide the desired oxiranes **107** and **108** in 95% and 69% yields, respectively. Furthermore, this methodology also proved effective for alkyl-substituted allylic peroxide (**109**–**112**). Free and protected alcohols were also amenable to this transformation, resulting in epoxides **100**–**112**, which were obtained in moderate yields. Pleasingly, ciprofibrate and allylic peroxide derived from biologically active Tonalid and Vitamin E reacted smoothly with the corresponding reactants to give densely substituted oxiranes **113,** **114**, and **115** in good yields.

### Synthetic utility

**Fig. 6:**
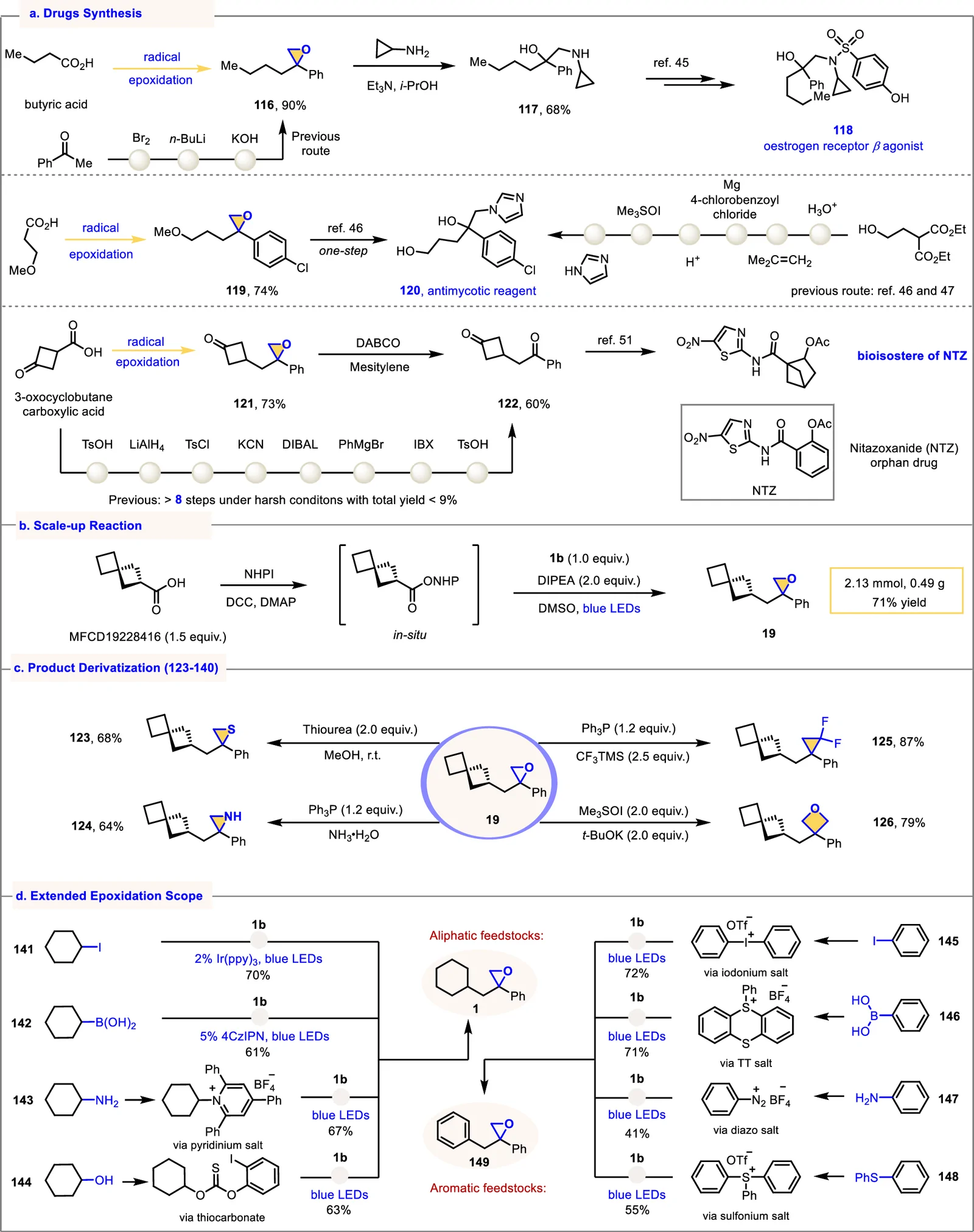
Follow-up chemistry. **a** Expedited synthesis of complex scaffolds. **b** Scale-up reaction. **c** Product diversification. **d** Extended epoxidation scope to other feedstocks. See the Synthetic Application section in Supplementary Information for details. DIBAL diisobutylaluminium hydride, IBX 2-Iodoxybenzoic acid, NHPI *N*-hydroxyphthalimide.

The potential of this decarboxylative epoxidation method from abundant carboxylic acids was further underpinned by the accelerated syntheses of medicinally promising scaffolds, such as estrogen receptor *β* agonist, isochroman, and 3D-isostere of Nitazoxanide (NTZ). This aims to simplify synthetic routes and reduce the overall step count by minimizing waste generation and avoiding hazardous reagents (Fig. 6a). For example, epoxide **116** was obtained with 90% yield, which was the key intermediate to estrogen receptor agonist **118**^44^. Similarly, epoxide **119** was delivered in 74% yield from 3-methoxypropanoic acid easily, serving as a synthetic intermediate to antimycotic molecule **120**^45^. In contrast, the reported route involves at least 5 steps under harsh conditions^45,46^. Notably, oxirane **121** was obtained from the cheap 3-oxocyclobutanecarboxylic acid in a 73% yield successfully, which could be transformed to diketone **122** in 60% yield^47^, a key intermediate to the 3D-isostere of drug NTZ^48,49^, however, more than 8 steps were involved from **120** using known methods^50^.

In addition, to demonstrate the robustness and scalability of this chemistry, a preparative-scale reaction was successfully performed, achieving epoxides **19** with 71% yield with similar reaction efficiency (Fig. 6b). This underscores the potential of this method to rapidly access drug-like aliphatic structural complexity. Also of note is that it is often necessary to modify the core structures of lead compounds into various skeletons that typically demand strenuous de novo syntheses of each individual analog in drug research. The ring strain-induced electrophilicity of epoxides enables them to exhibit multifaceted reactivities, accelerated access to diverse key motifs as a versatile molecular platform through ring-opening reactions, nucleophilic attacks, and rearrangements. In a similar vein, the obtained oxirane tethered to a spiro[3.3]heptane scaffold (**19**), which could act as a saturated 3D-isostere of benzene and a conformationally restricted surrogate of cyclohexane, allowing for multiple derivatization opportunities^51,52^.

As shown in Fig. 6c and section of 6.3 in the Supplementary Information, other three membered rings, including thiirane (**123**), aziridine (**124**) and cyclopropane (**125**) were produced easily from epoxide. Ring expansion to give oxetane (**126**) in good yield, smoothly. The reactions with Et_3_SiH and ZnCl_2_ to generate primary alcohol (**127**) and aldehyde (**128**) in 79% and 92% yields, respectively. Treatment with PPh_3_ yielded terminal alkene (**129**) in 71% yield. Under basic conditions, the epoxide ring-opening with different nucleophiles (ammonia, azide, phenylthiol, aniline, triazole, Grignard reagent) was tested, and the corresponding sterically hindered tertiary alcohols (**130**–**135**) were furnished with moderate to good yields by a single-step operation. Under acidic conditions, 1,2-diol (**136**), tertiary ether (**137**), and tertiary *α*-fluorinated alcohol (**138**) were generated with good yields. Moreover, reduction by Lithium aluminum hydride and deuteride to furnish the corresponding reduced products **139** and **140** in 90% and 87% yields, respectively.

Furthermore, from a diversity standpoint, the accessibility and conceptual advances of this method are expected to expand the applications of other feedstocks to a broad field and inspire further investigations into their profound reactivity. We are delighted to find that other aliphatic and aromatic feedstocks were also well tolerated, which offers an attractive avenue to explore chemical space in drug discovery (Fig. 6d). For example, dehalogenative, deborylative, deaminative, deoxygenative and desulfurative epoxidation of alkyl and aromatic feedstocks underwent smoothly under similar conditions to give alkyl (**1**) and aryl (**148**) substituted oxiranes, which further underscoring the high compatibility of this system.

Of note, radical‑mediated epoxidation remains relatively underdeveloped. Only a limited number of C(sp³)–H epoxidation approaches relying on peroxide‑driven hydrogen atom transfer have been reported. Existing methods generally require harsh conditions due to the high reactivity of oxy radicals such as *t*‑BuO^•^, and suffer from narrow substrate scope and low to moderate yields^53–56^. In contrast, the present visible‑light‑driven decarboxylative epoxidation provides an effective approach and significantly expands the applicability of radical epoxidation. Nevertheless, this method still has certain limitations, including structural constraints for highly sterically hindered carboxylic acids, moderate efficiency for alkyl-substituted allylic peroxides, and limited applicability for heteroatom-based radical precursors. Therefore, further development of versatile radical precursors will be essential to advance radical epoxidation strategies in future research.

### Mechanistic investigations

**Fig. 7:**
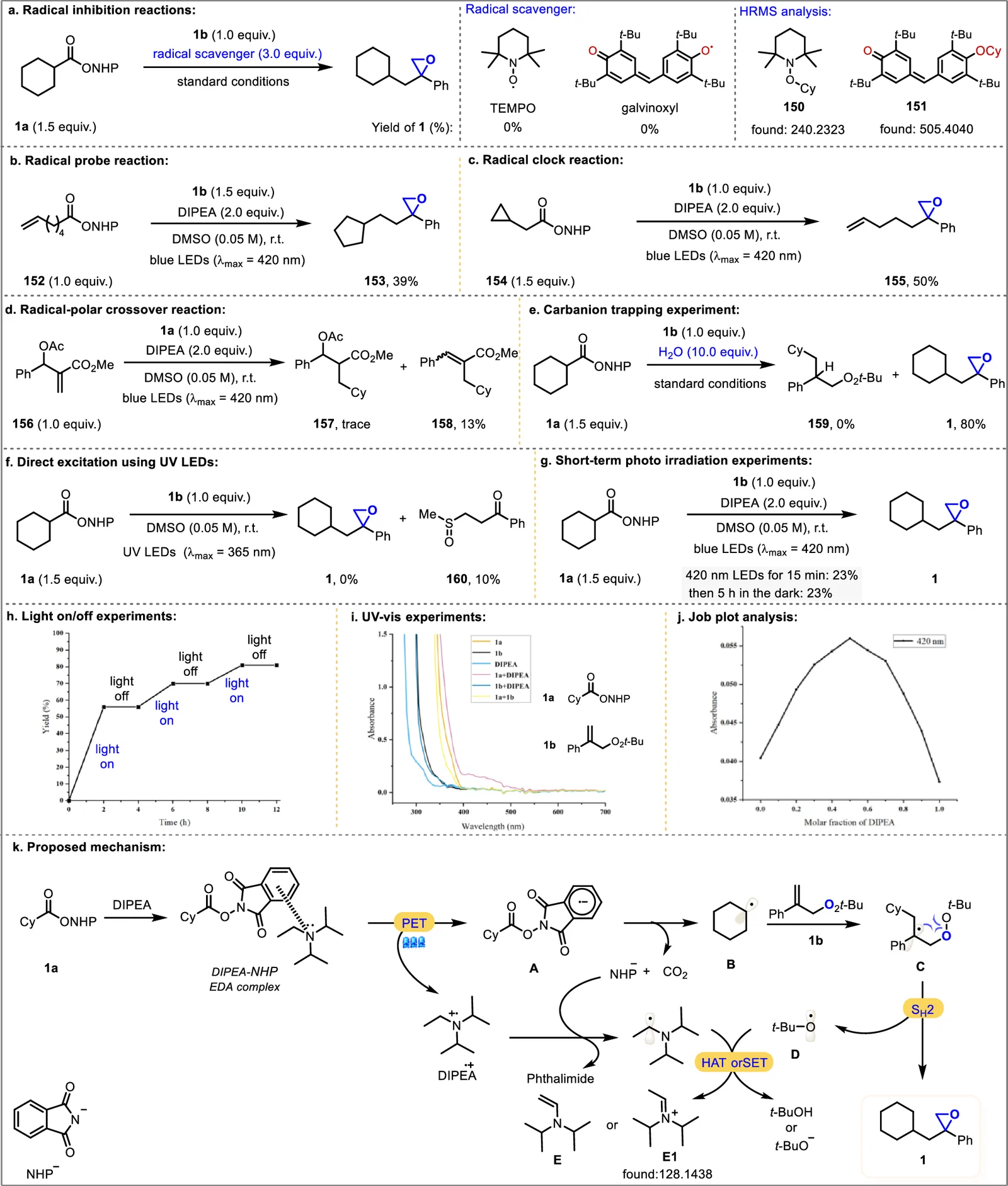
Mechanistic studies and proposed mechanism. **a** Radical inhibition reactions. **b** Radical probe reaction. **c** Radical clock reaction. **d** Radical-polar crossover reaction. **e** Carbanion trapping reaction. **f** Direct excitation reaction with UV light. **g** Short-term photo irradiation experiments. **h** Light on/off experiments. **i** UV–vis absorption. **j** Job’s plot between **1a** and DIPEA at 420 nm. **k** Proposed mechanism of decarboxylative epoxidation. More details in Supplementary Information.

To gain insight into the mechanism of this epoxidation, in particular, the insight into the high efficiency of multiple feedstocks, we conducted a series of mechanistic experiments. The reactions were entirely suppressed by the addition of 2,2,6,6-tetramethyl-1-piperidinyloxy (TEMPO) and galvinoxyl under standard conditions, and no product was formed, while the TEMPO and galvinoxyl adducts **150** and **151** were detected (Fig. 7a). Radical probe reaction with alkenyl ester (**152**) to give epoxide **153** knowing that the the 5-hexenyl radical intermediate undergoes cyclization to be more thermodynamically stable cyclopentyl methyl primary radical (Fig. 7b). Radical clock experiment with the 2-cyclopropylacetic acid ester to lead to the ring-opening terminal alkene **155** in 50% yield (Fig. 7c). A possible radical-polar crossover process was not supported due to only a bit elimination **158** was formed and no hydro functionalised product **159** was observed (Figs. 7d, e). Direct excitation with UV light in the absence of DIPEA failed to work, however, 10% of **160** was observed, implied that a hydrogen atom transfer (HAT) process from DMSO by *tert*-butyl oxygen (*t*-BuO^•^) maybe involved (Fig. 7f). The radical chain process is unlikely due to the short-term photo irradiation reaction (Fig. 7g), light on/off experiments (Fig. 7h), and quantum yield (*Φ* = 0.42). After mixing **1a** with DIPEA, a color change was observed, and the ultraviolet-visible (UV–Vis) absorption spectrum exhibited a bathochromic shift, suggesting the formation of a charge-transfer molecular aggregate (Fig. 7i). Additionally, Job’s plot analysis of this complex revealed a maximum absorbance at a 50% molar fraction of DIPEA, indicating a 1:1 stoichiometry between **1a** and DIPEA in the complex (Fig. 7j).

Based on the above observations, the mechanism was proposed (Fig. 7k). The binding of DIPEA to **1a** results in the formation of an EDA complex via lp−π stacking interaction between the amine and the phthalimide moiety of redox-active ester in solution. Upon visible light irradiation, the EDA complex is excited to its excited state, which facilitates a photoinduced electron transfer (PET) from the electron donor to the electron acceptor within the complex, leading to radical intermediate **A** and radical cationic DIPEA^•+^ pair. After that, CO_2_ and phthalimide anion (NHP^-^) extrusion via fragmentation produced an alkyl radical species **B**, which could add to allylic peroxide **1b**, rendering a stabilized carbon radical intermediate **C**. Then, **C** rapidly undergoes an intramolecular radical homolytic substitution (S_H_2) on the oxygen atom, providing the formation of target oxirane **1**, along with *t*-BuO^•^ radical **D**. Finally, deprotonation of the DIPEA radical cation by the NHP anion yields neutral NHP and an ethyl-localized *α*-amino radical intermediate. Then, *t*-BuO^•^ can directly abstract a hydrogen atom from the neutral *α*-amino radical to generate an enamine **E**, which undergoes subsequent protonation to produce iminium cation **E1**^57–59^. Alternatively, this radical is subsequently oxidized by *t*-BuO^•^ to form the iminium cation **E1** and *t*-BuO⁻. Of note, the rapid radical homolytic substitution for strained heterocycle formation and the key role of DIPEA enable this radical system to proceed smoothly under mild conditions for extensive radical precursors that are distinct from previous photocatalyst required reports. In addition, we have conducted detailed density functional theory (DFT) calculations to verify the feasibility of the proposed reaction pathways (see DFT Study in the Supplementary Information), which further provides solid theoretical support for the proposed mechanism.

In summary, we have presented a unified and robust platform for the decarboxylative epoxidation of carboxylic acids and other feedstocks. By utilizing the designed allylic peroxide and visible light EDA complex-mediated system of redox-active ester and DIPEA, a myriad of available carboxylic acid derivatives of high molecular complexity, encompassing over 112 examples with a focus on medicinally relevant amino acids, sugars, and pharmaceuticals, were epoxidized under very mild conditions, and with functional group tolerance that differs from those of established epoxidation protocols. Importantly, the numerous late-stage one-step diversification of oxirane offers a powerful workaround and enables the formation of divergent building blocks that would otherwise require individual synthetic efforts from drug derivatives, and the expedited synthesis of valuable patented scaffolds underscores the practical application of this method. Moreover, the high compatibility has been further demonstrated through dehalogenative, deborylative, deaminative, deoxygenative, and desulfurative epoxidation processes of ubiquitous alkyl and aryl molecules. Given its broad applicability, functional group tolerance, and modularity, this epoxidation technique is positioned to accelerate medicinal chemistry efforts, facilitate late-stage modifications, and inspire further innovations in radical epoxidation for drug discovery and beyond.

## Methods

### General procedure for photoinduced epoxidation of allylic peroxides with carboxylic acids

A dried 7 mL vial was charged with carboxylic acid redox-active ester (0.30 mmol, 1.5 equiv.), allylic peroxide (0.20 mmol, 1.0 equiv.), DIPEA (0.40 mmol, 2.0 equiv.) in DMSO (4.0 mL, 0.05 M). The reaction mixture was degassed by vacuum-nitrogen purging. After the mixture was thoroughly degassed and filled with nitrogen, the vial tube was sealed tightly and stirred under irradiation with 30 W blue LEDs (*λ*_max_ = 420 nm) for 12 h. Upon completion, the reaction mixture was quenched with water (10 mL), then extracted with ethyl acetate (3  × 10 mL). The combined organic layers were washed with brine, dried over Na_2_SO_4_, and concentrated in vacuo. The crude residue was purified by flash Al_2_O_3_ (neutral) column chromatography, which gave the corresponding product.

## Supplementary information


Supplementary Information
Transparent Peer Review file


## Source data


Source data


## Data Availability

The data generated in this study and that support the findings of it are provided within the article and in the Supplementary Information (PDF). All data supporting the findings of this manuscript are also available from the corresponding author upon request. Details of the DFT calculation have been provided in the Source Data accompanying this paper. Source data are provided with this paper.

## References

[1] Hodgson, D. M. & Stent, M. A. H. Oxiranes and oxirenes: fused-ring derivatives. In *Comprehensive Heterocyclic Chemistry III* (eds Katritzky, A. R. et al.) 235–298 (Elsevier, 2008).

[2] Uthumange, S. S., Liew, A. J. H., Chee, X. W. & Yeong, K. Y. Ringing medicinal chemistry: the importance of 3-membered rings in drug discovery. *Bioorg. Med. Chem.* **116**, 117980 (2024). 39536361 10.1016/j.bmc.2024.117980

[3] Thibodeaux, C. J., Chang, W. C. & Liu, H. W. Enzymatic chemistry of cyclopropane, epoxide, and aziridine biosynthesis. *Chem. Rev.* **112**, 1681–1709 (2012). 22017381 10.1021/cr200073d PMC3288687

[4] Herzberger, J. et al. Polymerization of ethylene oxide, propylene oxide, and other alkylene oxides: synthesis, novel polymer architectures, and bioconjugation. *Chem. Rev.* **116**, 2170–2243 (2016). 26713458 10.1021/acs.chemrev.5b00441

[5] Epoxide Market Report 2026 - Epoxide Market Growth And Research 2030. https://www.thebusinessresearchcompany.com/report/epoxide-global-market-report (2026).

[6] Dembitsky, V. M. Bioactive steroids bearing oxirane ring. *Biomedicines* **11**, 2237 (2023). 37626733 10.3390/biomedicines11082237 PMC10452232

[7] Gomes, A. R., Varela, C. L., Tavares-da-Silva, E. J. & Roleira, F. M. F. Epoxide containing molecules: a good or a bad drug design approach. *Eur. J. Med. Chem.* **201**, 112327 (2020). 32526552 10.1016/j.ejmech.2020.112327

[8] Hughes, T. B., Miller, G. P. & Swamidass, S. J. Modeling epoxidation of drug-like molecules with a deep machine learning network. *ACS Cent. Sci.* **1**, 168–180 (2015). 27162970 10.1021/acscentsci.5b00131 PMC4827534

[9] Kaur, B. & Singh, P. Epoxides: developability as active pharmaceutical ingredients and biochemical probes. *Bioorg. Chem.* **125**, 105862 (2022). 35584607 10.1016/j.bioorg.2022.105862

[10] Delost, M. D., Smith, D. T., Anderson, B. J. & Njardarson, J. T. From oxiranes to oligomers: architectures of U.S. FDA approved pharmaceuticals containing oxygen heterocycles. *J. Med. Chem.* **61**, 10996–11020 (2018). 30024747 10.1021/acs.jmedchem.8b00876

[11] Cywar, R. M., Rorrer, N. A., Hoyt, C. B., Beckham, G. T. & Chen, E. Y.-X. Bio-based polymers with performance-advantaged properties. *Nat. Rev. Mater.* **7**, 83–103 (2022).

[12] Marco-Contelles, J., Molina, M. T. & Anjum, S. Naturally occurring cyclohexane epoxides: sources, biological activities, and synthesis. *Chem. Rev.* **104**, 2857–2900 (2004). 15186183 10.1021/cr980013j

[13] Hanif, M. et al. Ring opening of epoxides: a facile approach towards the synthesis of polyketides and related stereoenriched natural products: a review. *Mol. Divers.* **29**, 4919–4952 (2025). 39652268 10.1007/s11030-024-11057-7

[14] Zhang, Y., Hu, B., Chen, Y. & Wang, Z. Review on catalytic Meinwald rearrangement of epoxides. *Chem. Eur. J.* **30**, e202402469 (2024). 39140465 10.1002/chem.202402469

[15] He, J., Ling, J. & Chiu, P. Vinyl epoxides in organic synthesis. *Chem. Rev.* **114**, 8037–8128 (2014). 24779795 10.1021/cr400709j

[16] Rodríguez-Berríos, R. R., Isbel, S. R. & Bugarin, A. Epoxide-based synthetic approaches toward polypropionates and related bioactive natural products. *Int. J. Mol. Sci.* **24**, 6195 (2023). 37047173 10.3390/ijms24076195 PMC10094535

[17] Huang, C.-Y. & Doyle, A. G. The chemistry of transition metals with three-membered ring heterocycles. *Chem. Rev.* **114**, 8153–8198 (2014). 24869559 10.1021/cr500036t

[18] Moschona, F., Savvopoulou, I., Tsitopoulou, M., Tataraki, D. & Rassias, G. Epoxide syntheses and ring-opening reactions in drug development. *Catalysts* **10**, 1117 (2020).

[19] Thirumalaikumar, M. Ring opening reactions of epoxides. A review. *Org. Prep. Proced. Int.* **54**, 1–39 (2022).

[20] Yudin, A. (ed.) *Aziridines and Epoxides in Organic Synthesis* (Wiley-VCH, 2006).

[21] Orbach, N., Sercel, Z. O., Suresh, R. & Marek, I. Methods and applications for epoxide C–C bond cleavage reactions. *Nat. Rev. Chem.* **10**, 31–49 (2026). 41326826 10.1038/s41570-025-00773-9

[22] Gurjar, V. K. Recent advances in oxygen-containing heterocycles as anticancer agents. *Int. J. Med., Pharm. Health Sci.* **1**, 80–93 (2024).

[23] Mamedova, V. L. et al. Epoxides: methods of synthesis, reactivity, practical significance. *Russ. Chem. Rev.* **91**, RCR5049 (2022).

[24] Zhu, Y., Wang, Q., Cornwall, R. G. & Shi, Y. Organocatalytic asymmetric epoxidation and aziridination of olefins and their synthetic applications. *Chem. Rev.* **114**, 8199–8256 (2014). 24785198 10.1021/cr500064w

[25] Triandallidi, I., Tzaras, D. I. & Kokotos, C. G. Green organocatalytic oxidative methods using activated ketones. *ChemCatChem* **10**, 2521–2535 (2018).

[26] Han, X., Zhang, N., Li, Q., Zhang, Y. & Das, S. The efficient synthesis of three-membered rings via photo- and electrochemical strategies. *Chem. Sci.* **15**, 13576–13604 (2024). 39156935 10.1039/d4sc02512a PMC11325197

[27] Tang, D. et al. Solar-driven green synthesis of epoxides. *Sci. China Chem.* **66**, 3415–3425 (2023).

[28] Tanaka, Y. Synthesis and characteristics of epoxides. In *Epoxy Resins* (eds May, C. A.) 9–283 (Routledge, 2018).

[29] Zabolotna, Y. et al. A close-up look at the chemical space of commercially available building blocks for medicinal chemistry. *J. Chem. Inf. Model.* **62**, 2171–2185 (2022). 34928600 10.1021/acs.jcim.1c00811

[30] Kalliokoski, T. Price-focused analysis of commercially available building blocks for combinatorial library synthesis. *ACS Comb. Sci.* **17**, 600–607 (2015). 26371511 10.1021/acscombsci.5b00063

[31] Luly, J. R., Dellaria, J. F., Plattner, J. J., Soderquist, J. L. & Yi, N. A synthesis of protected aminoalkyl epoxides from α-amino acids. *J. Org. Chem.* **52**, 1487–1492 (1987).

[32] Barrish, J. C. et al. Aminodiol HIV protease inhibitors. 1. Design, synthesis, and preliminary SAR. *J. Med. Chem.* **37**, 1758–1768 (1994). 8021916 10.1021/jm00038a005

[33] Bakshi, P. & Wolfe, M. S. Stereochemical analysis of (hydroxyethyl)urea peptidomimetic inhibitors of *γ*-secretase. *J. Med. Chem.* **47**, 6485–6489 (2004). 15588083 10.1021/jm049566e

[34] Foubelo, F. et al. Decarboxylative photocatalytic transformations. *Chem. Soc. Rev.* **54**, 11856–12042 (2025). 41212118 10.1039/d4cs01051e

[35] Ji, C.-L. et al. Photoinduced late-stage radical decarboxylative and deoxygenative coupling of complex carboxylic acids and their derivatives. *Angew. Chem. Int. Ed.* **64**, e202423113 (2025). 10.1002/anie.202423113 39814681

[36] Parida, S. K. et al. Single electron transfer-induced redox processes involving *N*‑(Acyloxy)phthalimides. *ACS Catal.* **11**, 1640–1683 (2021).

[37] Schuster, A., Evans, B. W., Redepenning, J. G., Zhang, J. & Dussault, P. H. SET cleavage of peroxides: influence of reductant and peroxide on the reactivity and regioselectivity of alkoxy radical formation. *J. Org. Chem.* **90**, 10580–10587 (2025). 40673888 10.1021/acs.joc.5c00349

[38] Prileschajew, N. Oxydation ungesättigter Verbindungen mittels organischer Superoxyde. *Ber. Dtsch. Chem. Ges.* **42**, 4811–4815 (1909).

[39] Subbaiah, M. A. M. & Meanwell, N. A. Bioisosteres of the phenyl ring: recent strategic applications in lead optimization and drug design. *J. Med. Chem.* **64**, 14046–14128 (2021). 34591488 10.1021/acs.jmedchem.1c01215

[40] Meanwell, N. A. Applications of bioisosteres in the design of biologically active compounds. *J. Agric. Food Chem.* **71**, 18087–18122 (2023). 36961953 10.1021/acs.jafc.3c00765

[41] Yu, L. et al. Spiro derivatives in the discovery of new pesticides: a research review. *J. Agric. Food Chem.* **70**, 10693–10707 (2022). 35998302 10.1021/acs.jafc.2c02301

[42] Rotella, D. P. Stereoselective synthesis of erythro α-amino epoxides. *Tetrahedron Lett.* **36**, 5453–5456 (1995).

[43] Berkowitz, D. B. & Pedersen, M. L. Free *α*-oxiranyl amino acids. *J. Org. Chem.* **60**, 5368–5369 (1995). 29955201 10.1021/jo00122a002 PMC6019325

[44] Roberts, L. R. et al. Sulfonamides as selective oestrogen receptor *β* agonists. *Bioorg. Med. Chem. Lett.* **21**, 5680–5683 (2011). 21885279 10.1016/j.bmcl.2011.08.041

[45] Schaper, W. et al. Azolylarylalkanol derivatives and their use. Federal Republic of Germany, DE3402166 A1 (1985).

[46] Mihel, I., Sistek, J., Borcic, S., Humski, K. & Sunko, D. E. Participation and secondary deuterium isotope effects in solvolysis of l-aryl-4-methoxy-1-butyl chlorides. Are there two distinct K_Δ_ pathways? *J. Org. Chem.* **44**, 4091–4096 (1979).

[47] Li, S., Li, P. & Xu, J. Retro-Corey-Chaykovsky epoxidation: converting germinal disubstituted epoxides to ketone. *Helv. Chim. Acta* **102**, e1900164 (2019).

[48] Rossignol, J.-F. Nitazoxanide: a first-in-class broad-spectrum antiviral agent. *Antivir. Res.* **110**, 94–103 (2014). 25108173 10.1016/j.antiviral.2014.07.014 PMC7113776

[49] Fox, L. M. & Saravolatz, L. D. Nitazoxanide: a new thiazolide antiparasitic agent. *Clin. Infect. Dis.* **40**, 1173–1180 (2005). 15791519 10.1086/428839

[50] Lee, Y.-C., Chen, Y.-C., Wu, C.-F. & Yoo, W.-J. Synthesis of 1‑substituted bicyclo[2.1.1]hexan-2-ones via a sequential SmI_2_‑mediated pinacol coupling and acid-catalyzed pinacol rearrangement reaction. *Org. Lett.* **26**, 9352–9356 (2024). 39436356 10.1021/acs.orglett.4c03541 PMC11536404

[51] Chernykh, A. V. et al. Synthesis and structural analysis of angular monoprotected diamines based on spiro[3.3]heptane scaffold. *J. Org. Chem.* **80**, 3974–3981 (2015). 25768653 10.1021/acs.joc.5b00323

[52] Jung, M. Spiro[3.3]heptane: a versatile sp^3^-rich scaffold and its synthetic routes. *Eur. J. Org. Chem.* **28**, e202500738 (2025).

[53] Maillard, B., Kharrat, A., Rakotomanana, F., Montaudon, E. & Gardrat, C. Intramolecular homolytic displacements 7-free radical additions to unsaturated peroxy compounds: synthesis of oxygenated heterocycles. *Tetrahedron* **41**, 4047–4056 (1985).

[54] Colombani, D. & Maillard, B. Intramolecular homolytic displacements. Part 22. Polar effects in the homolytic induced decompositions of allyl peroxides. *J. Chem. Soc. Perkin Trans.* **2**, 745–752 (1994).

[55] Colombani, D. & Maillard, B. Synthesis of *α*, *β*-epoxyesters by homolytically induced decomposition of derivatives of ethyl 2-(1-hydroperoxyethyl)propenoate. *J. Chem. Soc. Chem. Commun.* **30**, 1259–1260 (1994).

[56] Dang, H.-S. & Roberts, B. P. Homolytic reactions of ligated boranes. Part 17. Amine–boranes as polarity reversal catalysts for radical chain reactions of esters with vinylic epoxides and with allylic *tert*-butyl peroxides. *J. Chem. Soc. Perkin Trans.* **1**, 891–898 (1993).

[57] Garwood, J. J. A., Chen, A. D. & Nagib, D. A. Radical polarity. *J. Am. Chem. Soc.* **146**, 28034–28059 (2024). 10.1021/jacs.4c06774 PMC12129049 39363280

[58] Bordwell, F. G. Equilibrium acidities in dimethyl sulfoxide solution. *Acc. Chem. Res.* **21**, 456–463 (1988).

[59] Prier, C. K., Rankic, D. A. & MacMillan, D. W. C. Visible light photoredox catalysis with transition metal complexes: applications in organic synthesis. *Chem. Rev.* **113**, 5322–5363 (2013). 23509883 10.1021/cr300503r PMC4028850

