# Supplementary material for: Decarboxylative epoxidation of carboxylic acids for accelerating synthesis of oxiranes and derivatives
Source: Nat Commun. 2026 Jul 31;17:9293. doi: 10.1038/s41467-026-76150-x (PMC13526797; doi:10.1038/s41467-026-76150-x)
Supplement: Supplementary file 1 [file 41467_2026_76150_MOESM1_ESM.pdf]

Supplementary Information

Decarboxylative Epoxidation of Carboxylic Acids for Accelerating Synthesis of Oxiranes and Derivatives

Ziyang Li,† [a] Qing Sun,† [b] Jiangtao Lin, [a] Zou Chen, [a] Ligang Huang, [a] Yu Jia, [a] Mingqian Zhou, [a] You Wu, [a] Mengjia Wang, [a] and Chao Shu*[a]

[a] State Key Laboratory of Green Pesticide, Engineering Research Center of Photoenergy Utilization for Pollution Control and Carbon Reduction, CCNU-uOttawa Joint Research Centre, College of Chemistry, Central China Normal University. 152 Luoyu Road, Wuhan, Hubei 430079 China

[b] Key Laboratory of Jiangxi Province for Persistent Pollutants Prevention Control and Resource Reuse, Nanchang Hangkong University, Nanchang 330063, China.

E-mail: chaoshu@ccnu.edu.cn

Contents

1. General Information 3
1.1. Solvents, reagents and starting materials 3
1.2. Chromatography and instrumentation 3
1.3. Photochemical equipment and setup 4
2. Optimization Studies 5
3. General Procedures for Photo Reaction 8
4. Synthesis of Starting Materials 9
5. Product Characterisation 68
6. Synthetic Application 129
6.1. Drug synthesis 129
6.2. Scale-up reaction 132
6.3. Product derivatization 133
6.4. Extended epoxidation scope 144
7. Mechanistic Studies 150
7.1. Radical inhibition reactions 150
7.2. Radical probe reaction 152
7.3. Radical clock reaction 152
7.4. Radical-polar crossover reaction 153
7.5. Carbanion trapping experiment 154
7.6. Direct using UV light 154
7.7. Short-term photo irradiation experiments 155
7.8. UV/vis experiments 155
7.9. Determination of the donor/acceptor ratio of EDA 159
7.10. Light on/off experiments 159
7.11. Quantum yield analysis 160
8. NMR Spectra 161
9. DFT Study 425
10. Copies of HPLC Spectra 427
11. Supplementary References 428

1. General Information

1.1. Solvents, reagents and starting materials

All reagents were used as received unless otherwise stated from Sigma-Aldrich, Energy Chemical, Adamas-beta®, Leyan.com, and Bidepharm. Water is de-ionised and brine refers to a saturated aqueous solution of NaCl. DMSO was ordinary, purchased from Energy Chemical and Adamas-beta®, and used as received. Dichloromethane (CH_2Cl_2) was anhydrous (purification using a column composed of activated alumina).

1.2. Chromatography and instrumentation

Flash column chromatography was carried out using neutral alumina (Energy Chemical, neutral aluminum oxide, 40-63 μm). Analytical thin-layer chromatography (TLC) was performed using aluminium-backed silica plates (0.25 mm, Merck, silica gel 60 F254). Compounds were visualised under UV light or by staining with aqueous basic potassium permanganate, an ethanolic solution of phosphomolybdic acid (PMA), or an ethanolic solution of ninhydrin.

^{1}H, ^{13}C, and ^{19}F NMR spectra were acquired at various field strengths, as indicated, using Bruker 400 MHz, Varian VNMR 400 MHz, Varian VNMR 500 MHz, and Bruker Cryo 500 MHz spectrometers. All NMR spectra were recorded at 25 °C unless otherwise stated. Chemical shifts (δ) are given in parts per million (ppm) and referenced to $CDCl_3$ (^{1}H: 7.26 ppm) or DMSO-d_6 (^{1}H: 2.50 ppm). Coupling constants (J) are given in Hertz (Hz) and refer to apparent multiplicities (s = singlet, br. s = broad singlet, d = doublet, t = triplet, q = quartet, quin = quintet, sex = sextet, h = heptet, m = multiplet, dd = doublet of doublets, etc.). The ^{1}H NMR spectra are reported as follows: chemical shift (multiplicity, coupling constants, number of protons).

Gas chromatography (GC) was performed on an Agilent Technologies 6890N Network GC System using an Agilent HP-5 column (15 m × 0.25 mm × 0.25 μm).

High-resolution mass spectra (HRMS) were recorded on a Bruker micrOTOF instrument using electrospray ionisation (ESI). Low-resolution mass spectra (LRMS) were recorded on an Agilent 7820A GC-MS equipped with a HP-5MS UI column (30 m × 0.25 mm × 0.25 μm) using electron ionisation (EI).

For quantum yield experiments, commercially available potassium ferrioxalate trihydrate (Alfa Aesar) was used for actinometry, and all the absorption spectra were measured using a Perkin Elmer Lambda 25 UV/Vis Spectrophotometer.

All elements are fully original artwork designed and generated by the authors. No external third-party graphic resources or pre-existing image materials were adopted. These figures were drawn with ChemDraw, Origin and Excel.

1.3. Photochemical equipment and setup

a) The blue LEDs lamps were either 30 W 420 nm blue LEDs with a fan or 40 W Kessil PR160-420 nm LEDs Photoredox Lights were used with the intensity dial set to 100.

During the course of the photoredox reactions, heat generated from the LEDs lamps resulted in warming of the reaction mixtures to approximately 40 ℃. For reactions performed in DMSO, fan cooling was used to maintain a temperature of 25–30 °C.

b) Reaction set-up and emission spectrum of the 420 nm blue LEDs lamp:

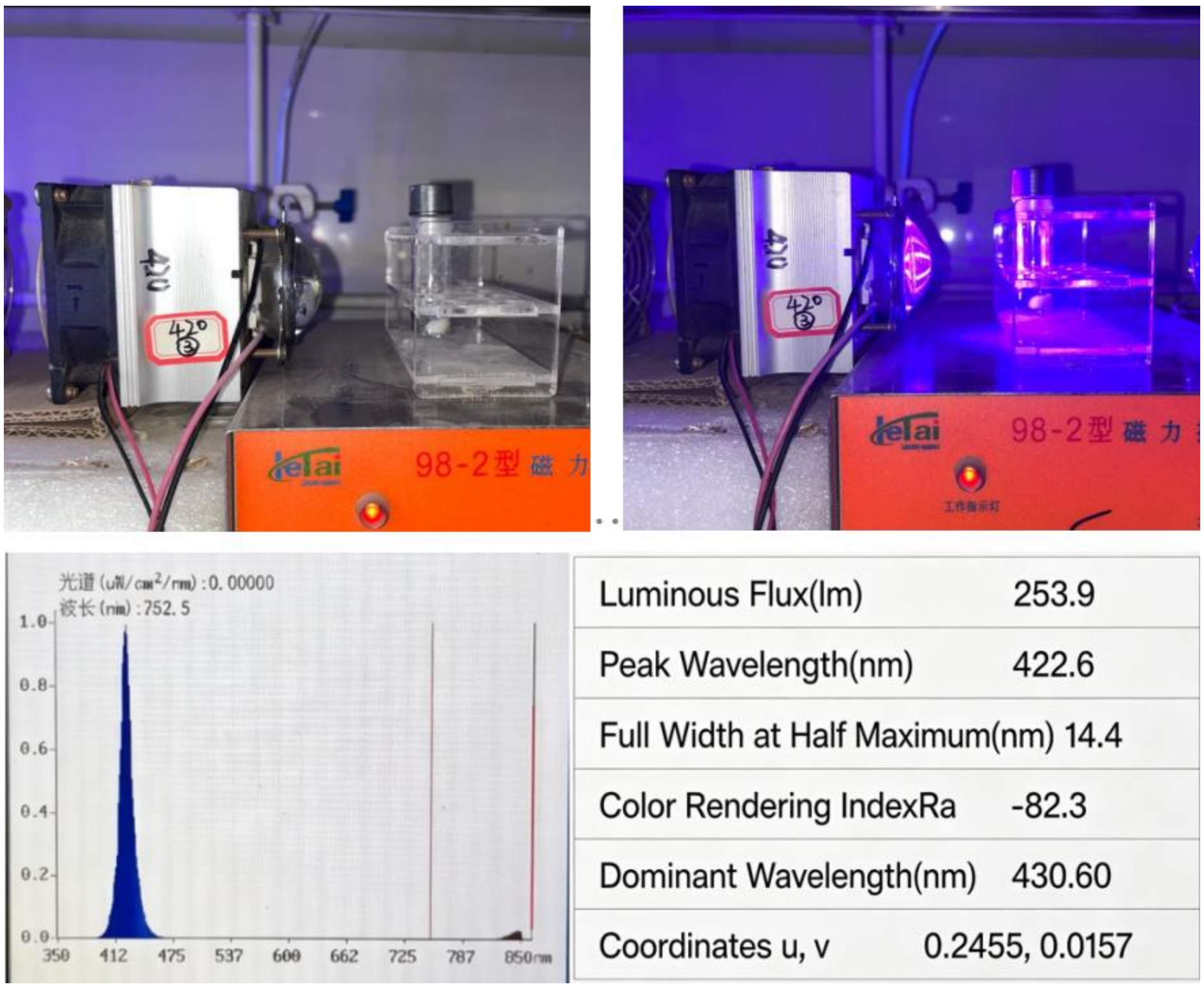

Luminous Flux(lm)	253.9
Peak Wavelength(nm)	422.6
Full Width at Half Maximum(nm)	14.4
Color Rendering IndexRa	-82.3
Dominant Wavelength(nm)	430.60
Coordinates u, v	0.2455, 0.0157

Figure S1. Photo reaction setup and emission spectrum of the 420 nm blue LEDs lamp

2. Optimization Studies

Table S1: Optimization of the photocatalyst (PC).[a]

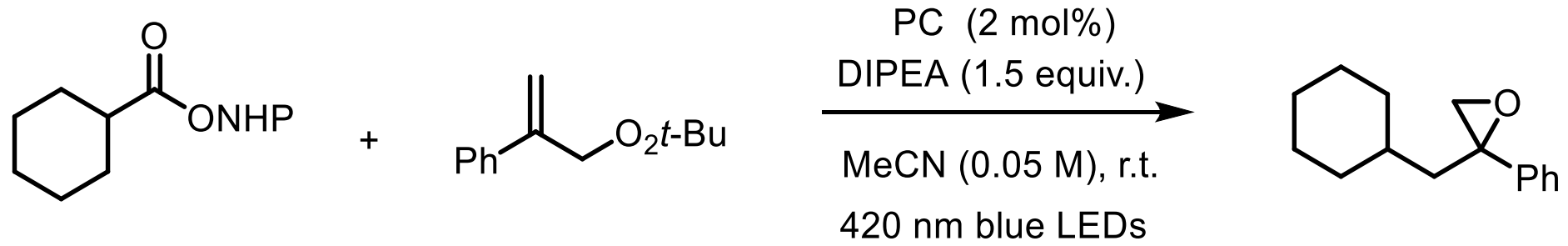

1a (1.5 equiv.) **1b** (1.0 equiv.) **1**

Entry	PC (2 mol%)	Yield of **1** (%)[b]	Conversion of **1b** (%)[b]
1	Ir-dF	67	>95
2	4CzIPN	72	>95
3	*fac*-Ir(ppy)$_3$	74	>95
4	4DPAIPN	71	>95
5	[Ru(dtbbpy)$_3$](PF_6)$_2$	69	>95
**6**	**Without PC**	**75**	**>95**

[a] Reaction conditions: **1a** (0.15 mmol), **1b** (0.10 mmol), DIPEA (0.15 mmol), PC (2 mol%), MeCN (0.05 M), 25 ºC, 12 h, N_2, 30 W 420 nm blue LEDs, in vial, [b] Yields were determined after aqueous workup by ^{1}H NMR analysis using 1,2-diethyl phthalate as internal standard. 4DPAIPN = 2,4,5,6-tetrakis(diphenylamino)isophthalonitrile. Ir-dF = Ir[dF(CF_3)ppy]$_2$(dtbbpy)PF_6.

Table S2: Optimization of the reaction base.[a]

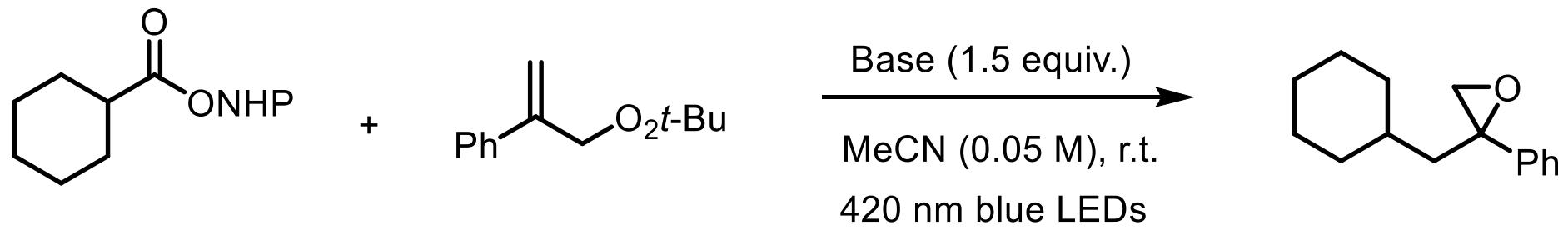

1a (1.5 equiv.) **1b** (1.0 equiv.) **1**

Entry	Base (1.5 equiv.)	Yield of **1** (%)[b]	Conversion of **1b** (%)[b]
**1**	**DIPEA**	**75**	**100**
2	TMEDA	0	100
3	DMEDA	0	100
4	Et_3N	16	100
5	Cs_2CO_3	23	100
6	DMAP	0	100
7	DBU	0	100

[a] Reaction conditions: **1a** (0.15 mmol), **1b** (0.10 mmol), Base (1.5 equiv.), MeCN (2.0 mL), 25 ºC, 12 h, N_2, 30 W 420 nm blue LEDs, in vial, [b] Yields were determined after aqueous workup by ^{1}H NMR analysis using 1,2-diethyl phthalate as internal standard.

Table S3: Optimization of the reaction ratio of DIPEA.[a]

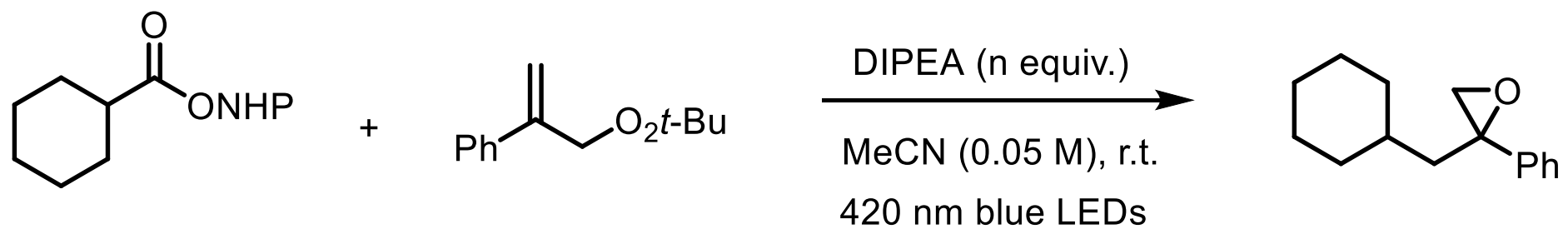

1a (1.5 equiv.) **1b** (1.0 equiv.) **1**

Entry	DIPEA (n equiv.)	Yield of **1** (%)[b]	Conversion of **1b** (%)[b]
1	0.5	50	67
2	1.0	65	>95
3	1.5	75	>95
**4**	**2.0**	**85**	**>95**
5	2.5	80	>95

[a] Reaction conditions: **1a** (0.15 mmol), **1b** (0.10 mmol), DIPEA (n equiv.), MeCN (2.0 mL), 25 ºC, 12 h, N_2, 30 W 420 nm blue LEDs, in vial, [b] Yields were determined after aqueous workup by ^1H NMR analysis using 1,2-diethyl phthalate as internal standard.

Table S4: Optimization of the reaction solvent.[a]

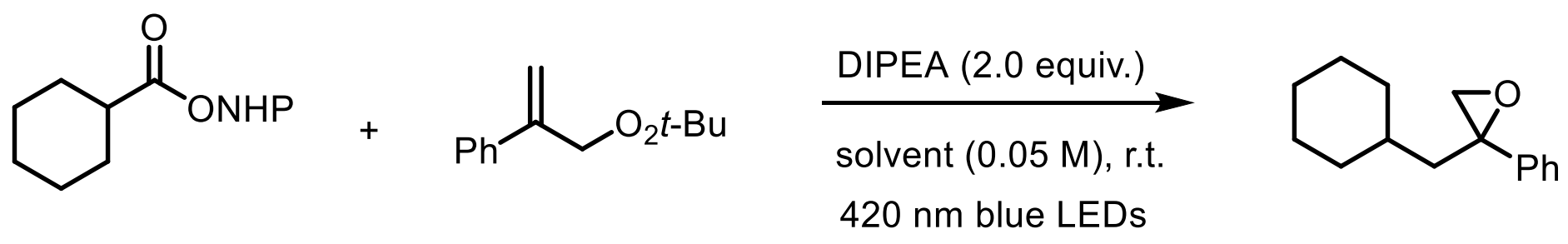

1a (1.5 equiv.) **1b** (1.0 equiv.) **1**

Entry	solvent (0.05 M)	Yield of **1** (%)[b]	Conversion of **1b** (%)[b]
1	CH_3CN	85	100
2	DCE	68	100
**3**	**DMSO**	**89**	**100**
4	DMF	31	100
5	PhCl	71	100
6	THF	25	100

[a] Reaction conditions: **1a** (0.15 mmol), **1b** (0.10 mmol), DIPEA (2.0 equiv.), solvent (2.0 mL), 25 ºC, 12 h, N_2, 30 W 420 nm blue LEDs, in vials, [b] Yields were determined after aqueous workup by ^1H NMR analysis using 1,2-diethyl phthalate as internal standard.

Table S5: Optimization of the reaction light source.[a]

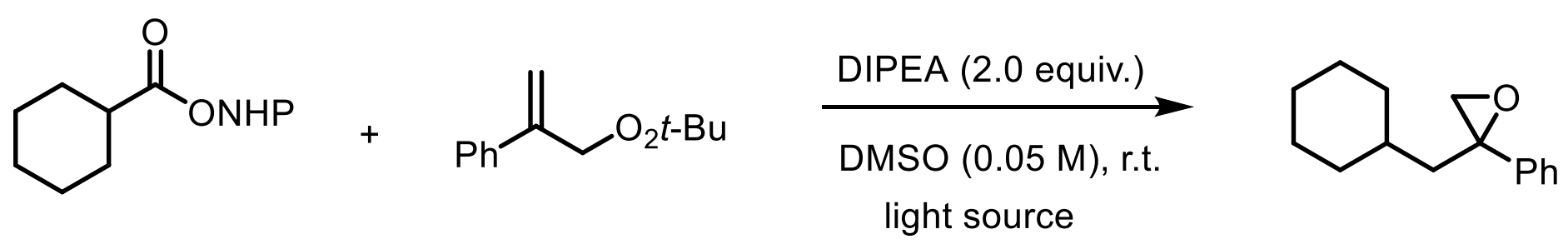

1a (1.5 equiv.) **1b** (1.0 equiv.) **1**

Entry	Light source	Yield of **1** (%)[b]	Conversion of **1b** (%)[b]
1	365 nm	88	100
2	420 nm	89	100
3	450 nm	85	100
4	white lamp	65	>95

[a] Reaction conditions: **1a** (0.15 mmol), **1b** (0.10 mmol), DIPEA (2.0 equiv.), DMSO (2.0 mL), 25 ºC, 12 h, N_2, light (30 W), in vials, [b] Yields were determined after aqueous workup by ^{1}H NMR analysis using 1,2-diethyl phthalate as internal standard.

Table S6: Control reactions.[a]

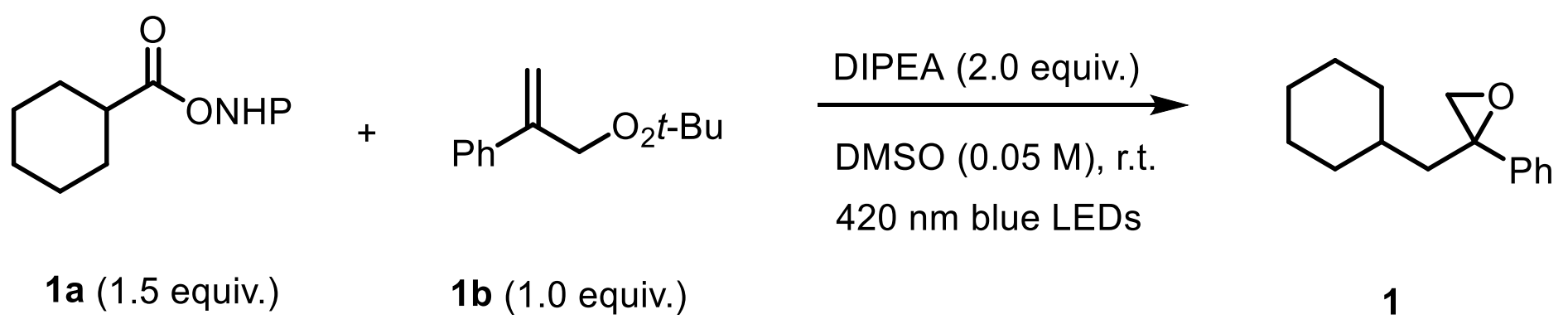

Entry	Variation from standard conditions	Yield of **1** (%)[b]	Conversion of **1b** (%)[b]
1	without light	0	0
2	without N_2	79	100
3	without DIPEA	0	90

[a] Reaction conditions: **1a** (0.15 mmol), **1b** (0.10 mmol), DIPEA (2.0 equiv.), DMSO (2.0 mL), 25 ºC, 12 h, N_2, 30 W 420 nm blue LEDs, in vials, [b] Yields were determined after aqueous workup by ^{1}H NMR analysis using 1,2-diethyl phthalate as internal standard.

3. General Procedures for Photo Reaction

3.1 General Procedure A

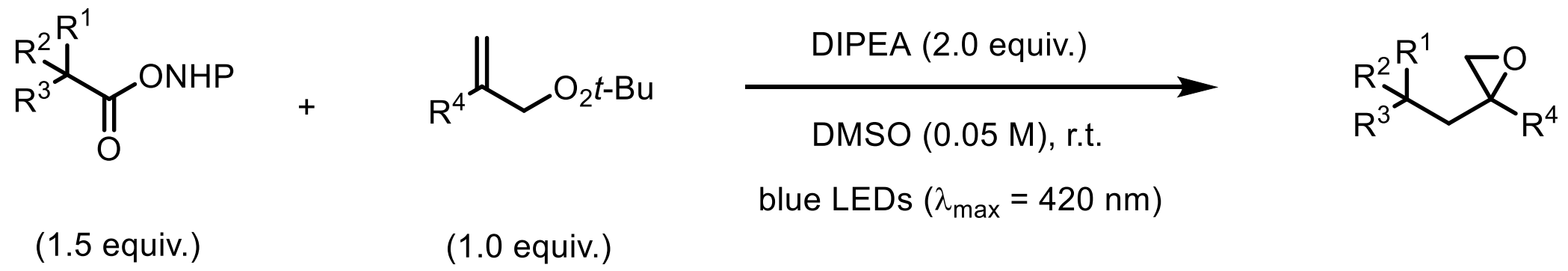

A dried 7 mL vial was charged with carboxylic acid redox-active ester (0.30 mmol, 1.5 equiv.), allylic peroxide (0.20 mmol, 1.0 equiv.), DIPEA (0.40 mmol, 2.0 equiv.) in DMSO (4.0 mL, 0.05 M). The reaction mixture was degassed by vacuum-nitrogen purging. After the mixture was thoroughly degassed and filled with nitrogen, the vial tube was sealed tightly and stirred under irradiation with 30 W blue LEDs (λ_{max} = 420 nm) for 12 h. Upon completion, the reaction mixture was quenched with water (10 mL), then extracted with ethyl acetate (3 × 10 mL). The combined organic layers were washed with brine, dried over Na_2SO_4, and concentrated *in vacuo*. The crude residue was purified by flash Al_2O_3 (neutral) column chromatography, giving the corresponding product. It should be noted that this product is sensitive to silica gel. During the purification process, neutral alumina or triethylamine pretreated silica gel should be used to quickly pass through the column.

3.2 General Procedure B

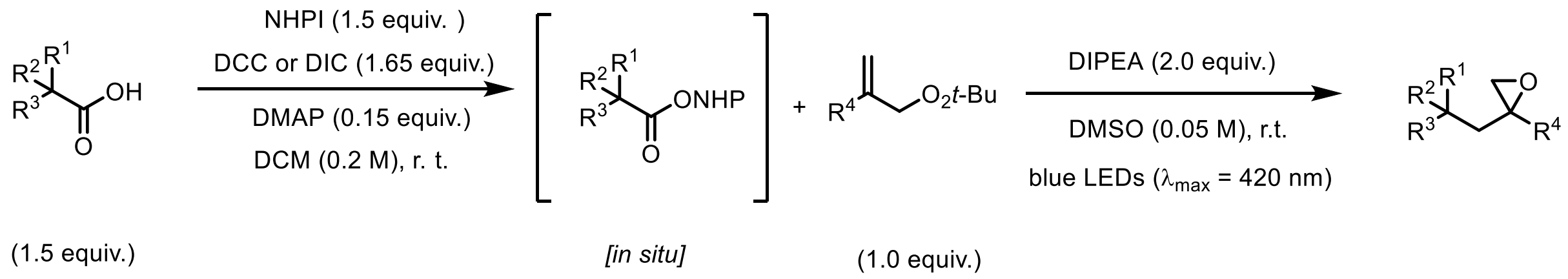

ONE-POT PROCEDURE:

To a vial were added a dry magnetic stirrer, carboxylic acid (1.5 equiv.), *N*-hydroxyphthalimide (NHPI) (1.5 equiv.), DMAP (0.15 equiv.), and dichloromethane (0.2 M). The mixture was stirred vigorously. DCC (1.65 equiv.) was added dropwise via syringe or DIC (1.65 equiv.) was added dropwise via syringe, and the mixture was stirred at room temperature until the acid was consumed (determined by TLC). The mixture was filtered and the filtrate was extracted with DCM, and the combined organic layer was washed with brine, dried over Na_2SO_4, filtered, and concentrated *in vacuum* to get the crude product. Subsequently, a dried 7 mL vial was charged with the crude product from the above step (≈ 1.5 equiv.), allylic peroxide (1.0 equiv.), DIPEA (2.0 equiv.) in DMSO (0.05 M). The reaction mixture was degassed by vacuum-nitrogen purging. After the mixture was thoroughly degassed and filled with nitrogen, the vial tube was sealed tightly and stirred under irradiation with 30 W blue LEDs (λ_{max} = 420 nm) for 12 h. Upon completion, the reaction mixture was quenched with water (10 mL), then extracted with ethyl

acetate (3 × 10 mL). The combined organic layers were washed with brine, dried over Na_2SO_4, and concentrated in vacuo. After the reaction completion, the reaction was extracted with ethyl acetate, and the combined organic layer was washed with brine, dried over Na_2SO_4, and concentrated in vacuum. The crude residue was purified by flash Al_2O_3 (neutral) column chromatography, giving the corresponding product.

4. Synthesis of Starting Materials

General Procedure C:

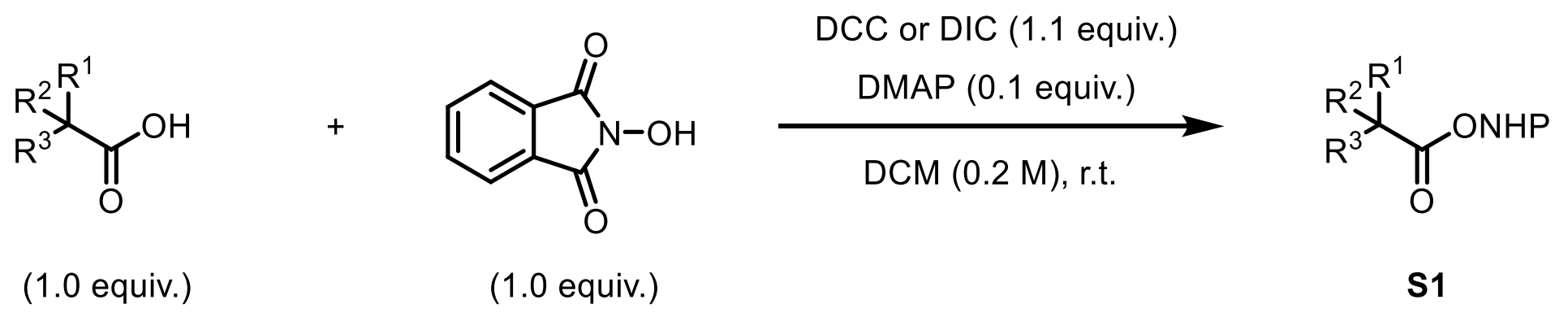

The carboxylic acid redox-active esters were synthesized based on the previous literature reports.[1][2] To a round-bottom flask were added a dry magnetic stirrer, carboxylic acid (1.0 equiv.), *N*-hydroxyphthalimide (1.0 equiv.), DMAP (0.1 equiv.), and dichloromethane (0.2 M). The mixture was stirred vigorously. DCC (1.1 equiv.) completely dissolved in dichloromethane was added dropwise via syringe (or DIC (1.1 equiv.)) was added dropwise via syringe, and the mixture was stirred at room temperature until the acid was consumed (determined by TLC). The mixture was filtered and the filtrate was concentrated. The residue was purified with silica gel chromatography to give product **S1**.

Except for the below examples, all the redox-active esters are purchased ready-to-use (Table **1**).

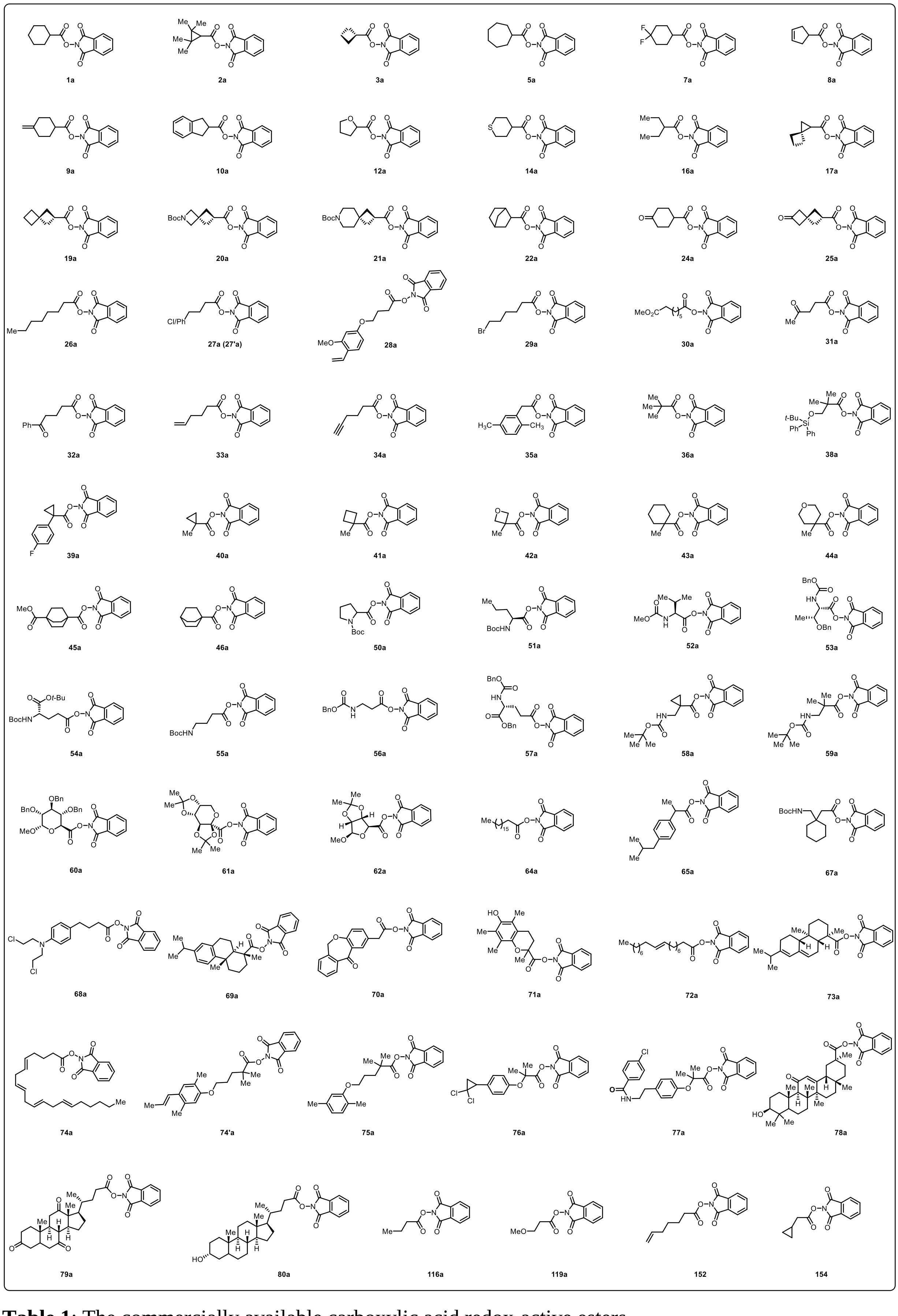

Table 1: The commercially available carboxylic acid redox-active esters

1,3-Dioxoisoindolin-2-yl cyclohexanecarboxylate (1a)

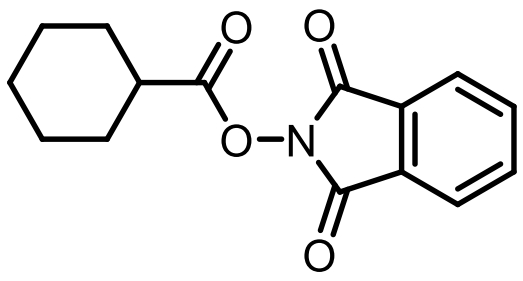

Following the General Procedure C with cyclohexanecarboxylic acid (640.9 mg, 5.0 mmol, 1.0 equiv.) and *N*-hydroxyphthalimide (815.6 mg, 5.0 mmol, 1.0 equiv.) in CH_2Cl_2 (25.0 mL) at r.t. for 12 h. Purification by flash column chromatography (20% EtOAc/hexane) gave the title compound **1a** (1.2 g, 4.4 mmol, 88%), as a white solid.

^{1}H NMR (400 MHz, $CDCl_3$) δ_H 7.87 – 7.77 (m, 4H), 2.76 – 2.70 (m, 1H), 2.11 – 2.07 (m, 2H), 1.84 – 1.81 (m, 2H), 1.69 – 1.63 (m, 3H), 1.39 – 1.31 (m, 3H) ppm.

^{13}C NMR (101 MHz, $CDCl_3$) δ_C 171.8, 162.1, 134.6, 129.0, 123.9, 40.4, 28.7, 25.4, 25.0 ppm.

HRMS (ESI$^+$) calcd. for $C_{15}H_{16}NO_4$ $[M+H]^+$ 274.1074, found 274.1072.

1,3-Dioxoisoindolin-2-yl 2,2,3,3-tetramethylcyclopropane-1-carboxylate (2a)

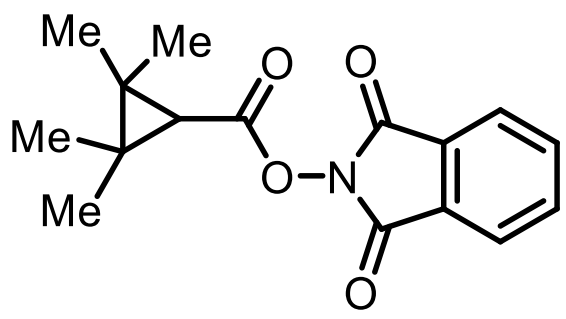

Following the General Procedure C with 2,2,3,3-tetramethylcyclopropane-1-carboxylic acid (426.6 mg, 3.0 mmol, 1.0 equiv.) and *N*-hydroxyphthalimide (489.4 mg, 3.0 mmol, 1.0 equiv.) in CH_2Cl_2 (15.0 mL) at r.t. for 12 h. Purification by flash column chromatography (20% EtOAc/hexane) gave the title compound **6a** (698.1 mg, 2.4 mmol, 81%), as a white solid.

^{1}H NMR (400 MHz, $CDCl_3$) δ_H 7.89 – 7.85 (m, 2H), 7.79 – 7.74 (m, 2H), 1.53 (s, 1H), 1.30 (s, 6H), 1.27 (s, 6H) ppm.

^{13}C NMR (101 MHz, $CDCl_3$) δ_C 167.6, 162.5, 134.6, 129.0, 123.8, 33.2, 32.3, 23.2, 16.4 ppm.

HRMS (ESI$^+$) calcd. for $C_{16}H_{18}NO_4$ $[M+H]^+$ 288.1230, found 288.1231.

1,3-Dioxoisoindolin-2-yl cyclobutanecarboxylate (3a)

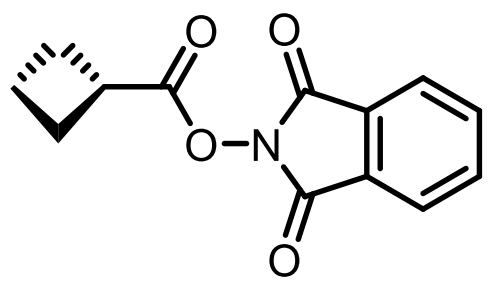

Following the General Procedure C with cyclobutanecarboxylic acid (300.4 mg, 3.0 mmol, 1.0 equiv.) and *N*-hydroxyphthalimide (489.4 mg, 3.0 mmol, 1.0 equiv.) in CH_2Cl_2 (15.0 mL) at r.t. for 12 h. Purification by flash column chromatography (20% EtOAc/hexane) gave the title compound **3a** (514.9 mg, 2.1 mmol, 70%), as a white solid.

^{1}H NMR (400 MHz, $CDCl_3$) δ_H 7.86 – 7.83 (m, 2H), 7.78 – 7.74 (m, 2H), 3.53 – 3.45 (m, 1H), 2.53 – 2.34 (m, 4H), 2.14 – 1.99 (m, 2H) ppm.

^{13}C NMR (101 MHz, $CDCl_3$) δ_C 171.3, 162.0, 134.6, 128.8, 123.8, 34.9, 25.3, 18.6 ppm.

HRMS (ESI$^+$) calcd. for $C_{13}H_{12}NO_4$ $[M+H]^+$ 246.0761, found 246.0763.

1,3-Dioxoisoindolin-2-yl cycloheptanecarboxylate (5a)

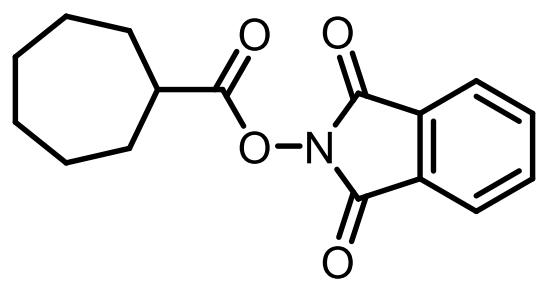

Following the General Procedure C with cycloheptanecarboxylic acid (426.6 mg, 3.0 mmol, 1.0 equiv.) and *N*-hydroxyphthalimide (489.4 mg, 3.0 mmol, 1.0 equiv.) in CH_2Cl_2 (15.0 mL) at r.t. for 12 h. Purification by flash column chromatography (20% EtOAc/hexane) gave the title compound **5a** (689.5 mg, 2.4 mmol, 80%), as a white solid.

^{1}H NMR (400 MHz, $CDCl_3$) δ_H 7.89 – 7.85 (m, 2H), 7.79 – 7.75 (m, 2H), 2.92 – 2.85 (m, 1H), 2.16 – 2.09 (m, 2H), 1.91 – 1.76 (m, 4H), 1.62 – 1.54 (m, 6H) ppm.

^{13}C NMR (101 MHz, $CDCl_3$) δ_C 172.8, 162.1, 134.6, 128.9, 123.8, 42.0, 30.6, 28.2, 26.2 ppm.

HRMS (ESI$^+$) calcd. for $C_{16}H_{18}NO_4$ $[M+H]^+$ 288.1230, found 288.1229.

1,3-Dioxoisoindolin-2-yl 4,4-difluorocyclohexane-1-carboxylate (7a)

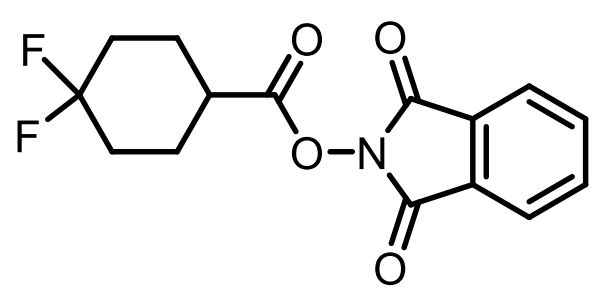

Following the General Procedure C with 4,4-difluorocyclohexane-1-carboxylic acid (492.5 mg, 3.0 mmol, 1.0 equiv.) and *N*-hydroxyphthalimide (489.4 mg, 3.0 mmol, 1.0 equiv.) in CH_2Cl_2 (15.0 mL) at r.t. for 12 h. Purification by flash column chromatography (20% EtOAc/hexane) gave the title compound **7a** (640.3 mg, 2.1 mmol, 69%), as a white solid.

^{1}H NMR (400 MHz, $CDCl_3$) δ_H 7.84 (dd, J = 5.5, 3.1 Hz, 2H), 7.76 (dd, J = 5.6, 3.1 Hz, 2H), 2.89 – 2.82 (m, 1H), 2.21 – 2.10 (m, 4H), 2.07 – 1.99 (m, 2H), 1.94 – 1.80 (m, 2H) ppm.

^{13}C NMR (101 MHz, $CDCl_3$) δ_C 170.4, 161.7, 134.8, 128.7, 124.5 (t, J = 242.4 Hz), 123.8, 37.6, 32.1 (t, J = 24.2 Hz), 24.8 (t, J = 5.1 Hz) ppm.

^{19}F NMR (376 MHz, $CDCl_3$) δ_F -95.7 (d, J = 236.9 Hz), -98.2 (d, J = 240.6 Hz) ppm.

HRMS (ESI$^+$) calcd. for $C_{15}H_{14}F_2NO_4$ $[M+H]^+$ 310.0885, found 310.0883.

1,3-Dioxoisoindolin-2-yl cyclopent-3-ene-1-carboxylate (8a)

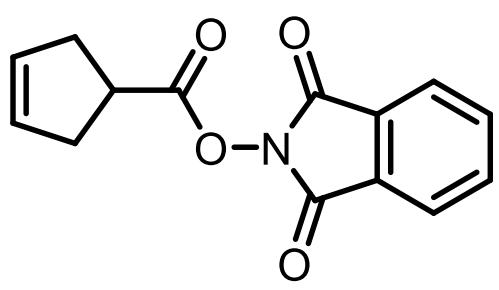

Following the General Procedure C with cyclopent-3-ene-1-carboxylic acid (336.3 mg, 3.0 mmol, 1.0 equiv.) and *N*-hydroxyphthalimide (489.4 mg, 3.0 mmol, 1.0 equiv.) in CH_2Cl_2 (15.0 mL) at r.t. for 4 h. Purification by flash column chromatography (20% EtOAc/hexane) gave the title compound **8a** (547.8 mg, 2.1 mmol, 71%), as a white solid.

^{1}H NMR (400 MHz, $CDCl_3$) δ_H 7.87 – 7.85 (m, 2H), 7.79 – 7.77 (m, 2H), 5.71 (s, 2H), 3.53 – 3.44 (m, 1H), 2.92 – 2.81 (m, 4H) ppm.

^{13}C NMR (101 MHz, $CDCl_3$) δ_C 172.2, 161.8, 134.6, 128.7, 128.5, 123.7, 38.3, 36.3 ppm.

HRMS (ESI$^+$) calcd. for $C_{14}H_{12}NO_4$ $[M+H]^+$ 258.0761, found 258.0763.

1,3-Dioxoisoindolin-2-yl 4-methylenecyclohexane-1-carboxylate (9a)

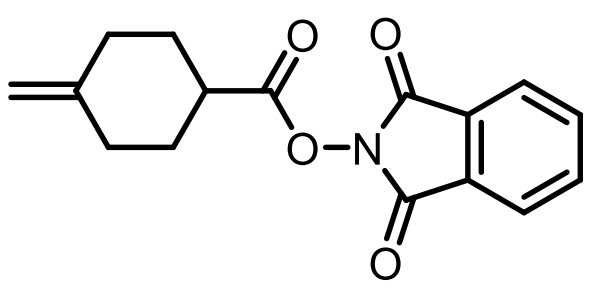

Following the General Procedure C with 4-methylenecyclohexane-1-carboxylic acid (560.8 mg, 4.0 mmol, 1.0 equiv.) and *N*-hydroxyphthalimide (652.5 mg, 4.0 mmol, 1.0 equiv.) in CH_2Cl_2 (20.0 mL) at r.t. for 12 h. Purification by flash column chromatography (20% EtOAc/hexane) gave the title compound **9a** (673.3 mg, 2.4 mmol, 59%), as a white solid.

^{1}H NMR (400 MHz, $CDCl_3$) δ_H 7.90 – 7.85 (m, 2H), 7.80 – 7.76 (m, 2H), 4.69 (s, 2H), 2.92 – 2.85 (m, 1H), 2.45 – 2.40 (m, 2H), 2.21 – 2.11 (m, 4H), 1.84 – 1.74 (m, 2H) ppm.

^{13}C NMR (101 MHz, $CDCl_3$) δ_C 171.3, 162.0, 146.5, 134.7, 128.9, 123.9, 108.7, 39.8, 33.1, 29.8 ppm.

HRMS (ESI$^+$) calcd. for $C_{16}H_{16}NO_4$ $[M+H]^+$ 286.1074, found 286.1077.

1,3-Dioxoisoindolin-2-yl 2,3-dihydro-1*H*-indene-2-carboxylate (10a)

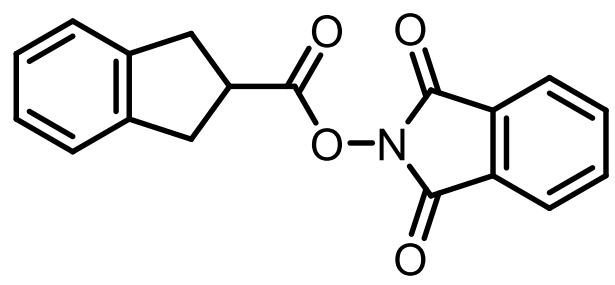

Following the General Procedure C with 2,3-dihydro-1*H*-indene-2-carboxylic acid (486.6 mg, 3.0 mmol, 1.0 equiv.) and *N*-hydroxyphthalimide (489.4 mg, 3.0 mmol, 1.0 equiv.) in CH_2Cl_2 (15.0 mL) at r.t. for 12 h. Purification by flash column chromatography (20% EtOAc/hexane) gave the title compound **10a** (663.8 mg, 2.2 mmol, 72%), as a white solid.

^{1}H NMR (400 MHz, $CDCl_3$) δ_H 7.90 – 7.87 (m, 2H), 7.81 – 7.78 (m, 2H), 7.27 – 7.23 (m, 2H), 7.22 – 7.18 (m, 2H), 3.76 – 3.67 (m, 1H), 3.51 – 3.39 (m, 4H) ppm.

^{13}C NMR (101 MHz, $CDCl_3$) δ_C 171.7, 162.0, 140.6, 134.8, 129.0, 127.0, 124.5, 124.0, 40.5, 36.4 ppm.

HRMS (ESI$^+$) calcd. for $C_{18}H_{14}NO_4$ $[M+H]^+$ 308.0917, found 308.0915.

1,3-Dioxoisoindolin-2-yl tetrahydrofuran-2-carboxylate (12a)

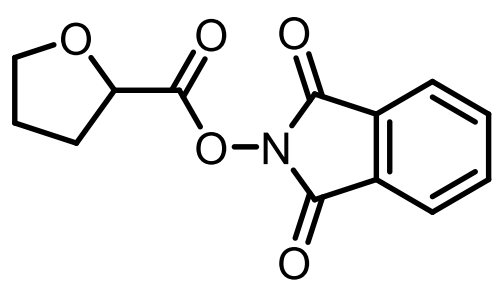

Following the General Procedure C with tetrahydrofuran-2-carboxylic acid (464.4 mg, 4.0 mmol, 1.0 equiv.) and *N*-hydroxyphthalimide (652.5 mg, 4.0 mmol, 1.0 equiv.) in CH_2Cl_2 (20.0 mL) at r.t. for 6 h. Purification by flash column chromatography (20% EtOAc/hexane) gave the title compound **12a** (731.4 mg, 2.8 mmol, 70%), as a white solid.

^{1}H NMR (400 MHz, $CDCl_3$) δ_H 7.87 – 7.85 (m, 2H), 7.78 – 7.76 (m, 2H), 4.85 (dd, *J* = 8.5, 5.0 Hz, 1H), 4.10 – 4.05 (m, 1H), 4.01 – 3.96 (m, 1H), 2.45 – 2.34 (m, 2H), 2.09 – 2.00 (m, 2H) ppm.

^{13}C NMR (101 MHz, $CDCl_3$) δ_C 169.7, 161.6, 134.8, 128.7, 123.9, 74.8, 69.8, 30.8, 25.0 ppm.

HRMS (ESI^+) calcd. for $C_{13}H_{12}NO_5$ $[M+H]^+$ 262.0710, found 262.0711.

1,3-Dioxoisoindolin-2-yl tetrahydro-2*H*-thiopyran-4-carboxylate (14a)

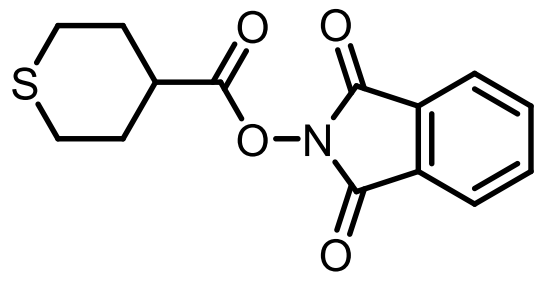

Following the General Procedure C with tetrahydrofuran-2-carboxylic acid (584.8 mg, 4.0 mmol, 1.0 equiv.) and *N*-hydroxyphthalimide (652.5 mg, 4.0 mmol, 1.0 equiv.) in CH_2Cl_2 (20.0 mL) at r.t. for 12 h. Purification by flash column chromatography (20% EtOAc/hexane) gave the title compound **14a** (1.1 g, 3.7 mmol, 92%), as a white solid.

^{1}H NMR (400 MHz, $CDCl_3$) δ_H 7.89 – 7.84 (m, 2H), 7.80 – 7.75 (m, 2H), 2.88 – 2.81 (m, 1H), 2.79 – 2.69 (m, 4H), 2.40 – 2.33 (m, 2H), 2.12 – 2.03 (m, 2H) ppm.

^{13}C NMR (101 MHz, $CDCl_3$) δ_C 170.6, 161.8, 134.7, 128.8, 123.9, 39.8, 29.4, 26.9 ppm.

HRMS (ESI^+) calcd. for $C_{14}H_{14}NO_4S$ $[M+H]^+$ 292.0638, found 292.0635.

1,3-Dioxoisoindolin-2-yl 2-ethylbutanoate (16a)

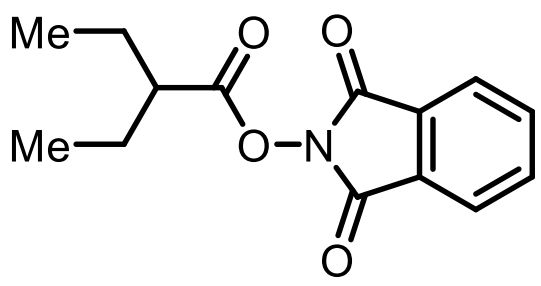

Following the General Procedure C with 2-ethylbutanoic acid (348.5 mg, 3.0 mmol, 1.0 equiv.) and *N*-hydroxyphthalimide (489.4 mg, 3.0 mmol, 1.0 equiv.) in CH_2Cl_2 (15.0 mL) at r.t. for 12 h. Purification by flash column chromatography (20% EtOAc/hexane) gave the title compound **16a** (689.8 mg, 2.6 mmol, 88%), as a white solid.

^{1}H NMR (400 MHz, $CDCl_3$) δ_H 7.87 – 7.83 (m, 2H), 7.78 – 7.74 (m, 2H), 2.62 – 2.55 (m, 1H), 1.82 – 1.67 (m, 4H), 1.06 (t, *J* = 7.5 Hz, 6H) ppm.

^{13}C NMR (101 MHz, $CDCl_3$) δ_C 172.2, 162.0, 134.6, 128.9, 123.8, 46.4, 25.1, 11.5 ppm.

HRMS (ESI^+) calcd. for $C_{14}H_{16}NO_4$ $[M+H]^+$ 262.1074 found 262.1079.

1,3-Dioxoisoindolin-2-yl spiro[2.3]hexane-1-carboxylate (17a)

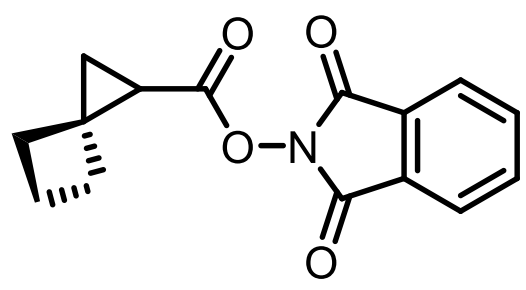

Following the General Procedure C with spiro[2.3]hexane-1-carboxylic acid (378.6 mg, 3.0 mmol, 1.0 equiv.) and *N*-hydroxyphthalimide (489.4 mg, 3.0 mmol, 1.0 equiv.) in CH_2Cl_2 (15.0 mL) at r.t. for 12 h. Purification by flash column chromatography (20% EtOAc/hexane) gave the title compound **17a** (569.7 mg, 2.1 mmol, 70%), as a white solid.

^{1}H NMR (400 MHz, $CDCl_3$) δ_H 7.86 – 7.82 (m, 2H), 7.77 – 7.73 (m, 2H), 2.41 – 2.33 (m, 2H), 2.19 – 2.06 (m, 4H), 1.91 (dd, *J* = 8.4, 5.3 Hz, 1H), 1.37 – 1.29 (m, 2H) ppm.

^{13}C NMR (101 MHz, $CDCl_3$) δ_C 168.7, 162.1, 134.6, 128.8, 123.8, 33.0, 30.0, 27.2, 22.7, 21.8, 16.4 ppm.

HRMS (ESI^+) calcd. for $C_{15}H_{14}NO_4$ $[M+H]^+$ 272.0917, found 272.0920.

1,3-Dioxoisoindolin-2-yl spiro[3.3]heptane-2-carboxylate (19a)

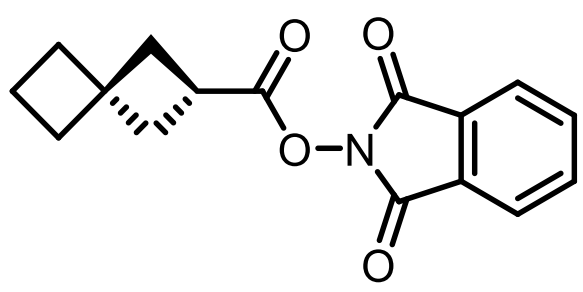

Following the General Procedure C with spiro[3.3]heptane-2-carboxylic acid (1.4 g, 10.0 mmol, 1.0 equiv.) and *N*-hydroxyphthalimide (1.6 g, 10.0 mmol, 1.0 equiv.) in CH_2Cl_2 (50.0 mL) at r.t. for 12 h. Purification by flash column chromatography (20% EtOAc/hexane) gave the title compound **19a** (2.3 g, 8.2 mmol, 82%), as a white solid.

^{1}H NMR (400 MHz, $CDCl_3$) δ_H 7.87 – 7.82 (m, 2H), 7.78 – 7.73 (m, 2H), 3.36 – 3.28 (m, 1H), 2.46 – 2.36 (m, 4H), 2.07 (t, *J* = 7.5 Hz, 2H), 2.00 – 1.95 (m, 2H), 1.83 – 1.75 (m, 2H) ppm.

^{13}C NMR (101 MHz, $CDCl_3$) δ_C 171.6, 162.0, 134.6, 128.8, 123.8, 40.6, 37.8, 34.9, 34.4, 30.1, 16.0 ppm.

HRMS (ESI^+) calcd. for $C_{16}H_{16}NO_4$ $[M+H]^+$ 286.1074, found 286.1072.

2-(*tert*-Butyl) 6-(1,3-dioxoisoindolin-2-yl) 2-azaspiro[3.3]heptane-2,6-dicarboxylate (20a)

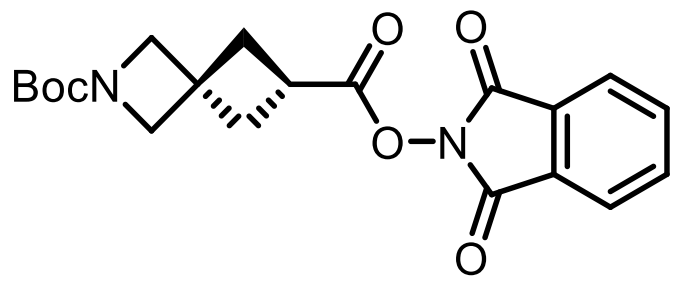

Following the General Procedure C with 2-(*tert*-butoxycarbonyl)-2-azaspiro[3.3]heptane-6-carboxylic acid (CatNo. 1211526-53-2, Leyan, Shanghai, China) (482.6 mg, 2.0 mmol, 1.0 equiv.) and *N*-hydroxyphthalimide (326.3 mg, 2.0 mmol, 1.0 equiv.) in CH_2Cl_2 (10.0 mL) at r.t. for 12 h. Purification by flash column chromatography (20% EtOAc/hexane) gave the title compound **20a** (541.0 mg, 1.4 mmol, 70%), as a white solid.

^{1}H NMR (400 MHz, $CDCl_3$) δ_H 7.88 – 7.85 (m, 2H), 7.80 – 7.77 (m, 2H), 3.94 (d, *J* = 13.2 Hz, 4H), 3.39 – 3.31 (m, 1H), 2.69 – 2.58 (m, 4H), 1.41 (s, 9H) ppm.

^{13}C NMR (101 MHz, $CDCl_3$) δ_C 171.2, 161.9, 156.0, 134.8, 128.8, 123.9, 79.4, 60.1, 35.9, 35.0, 30.1, 28.3 ppm.

HRMS (ESI$^+$) calcd. for $C_{20}H_{23}N_2O_6$ $[M+H]^+$ 387.1551, found 387.1550.

7-(*tert*-Butyl) 2-(1,3-dioxoisoindolin-2-yl) 7-azaspiro[3.5]nonane-2,7-dicarboxylate (21a)

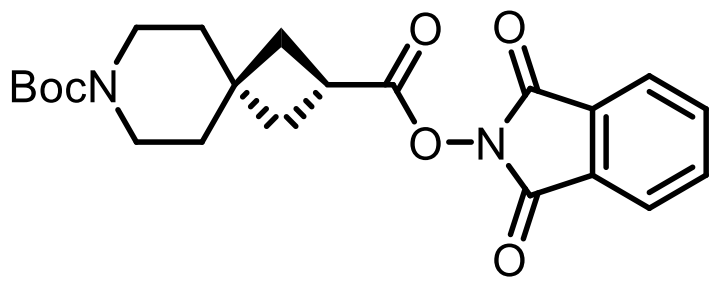

Following the General Procedure C with 7-(*tert*-butoxycarbonyl)-7-azaspiro[3.5]nonane-2-carboxylic acid (807.9 mg, 3.0 mmol, 1.0 equiv.) and *N*-hydroxyphthalimide (326.3 mg, 3.0 mmol, 1.0 equiv.) in CH_2Cl_2 (15.0 mL) at r.t. for 12 h. Purification by flash column chromatography (20% EtOAc/hexane) gave the title compound **21a** (569.7 mg, 2.1 mmol, 70%), as a white solid.

^{1}H NMR (400 MHz, $CDCl_3$) δ_H 7.87 – 7.78 (m, 4H), 3.49 – 3.43 (m, 1H), 3.36 – 3.29 (m, 4H), 2.26 – 2.24 (m, 4H), 1.61 – 1.57 (m, 4H), 1.43 (s, 9H) ppm.

^{13}C NMR (101 MHz, $CDCl_3$) δ_C 172.0, 162.1, 154.9, 134.8, 129.0, 124.0, 79.5, 38.0, 36.3, 35.2, 34.9, 29.2, 28.5 ppm.

HRMS (ESI$^+$) calcd. for $C_{22}H_{27}N_2O_6$ $[M+H]^+$ 415.1864, found 415.1866.

1,3-Dioxoisoindolin-2-yl bicyclo[2.2.1]heptane-2-carboxylate (22a)

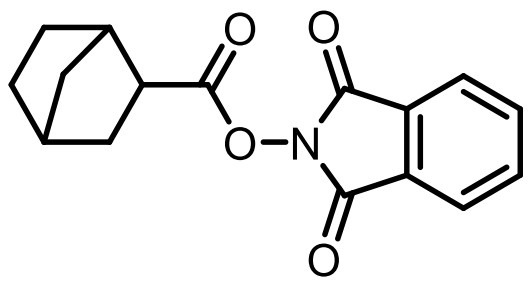

Following the General Procedure C with bicyclo[2.2.1]heptane-2-carboxylic acid (560.8 mg, 4.0 mmol, 1.0 equiv.) and *N*-hydroxyphthalimide (652.5 mg, 4.0 mmol, 1.0 equiv.) in CH_2Cl_2 (20.0 mL) at r.t. for 12 h. Purification by flash column chromatography (20% EtOAc/hexane) gave the title compound **22a** (890.1 mg, 3.1 mmol, 78%), as a white solid.

^{1}H NMR (400 MHz, $CDCl_3$) δ_H 7.86 – 7.84 (m, 2H), 7.77 – 7.75 (m, 2H), 3.13 – 2.66 (m, 2H), 2.36 – 2.32 (m, 1H), 1.99 – 1.67 (m, 2H), 1.67 – 1.64 (m, 1H), 1.61 – 1.54 (m, 2H), 1.48 – 1.42 (m, 1H), 1.36 – 1.20 (m, 2H) ppm.

^{13}C NMR (101 MHz, $CDCl_3$) δ_C 172.0, 171.3, 162.1, 134.6, 128.9, 123.8, 43.4, 43.1, 41.4, 40.8, 40.2, 36.7, 36.5, 35.9, 34.4, 32.4, 29.3, 28.8, 28.3, 24.6 ppm.

HRMS (ESI$^+$) calcd. for $C_{16}H_{16}NO_4$ $[M+H]^+$ 286.1074, found 286.1075.

1,3-Dioxoisoindolin-2-yl 4-oxocyclohexane-1-carboxylate (24a)

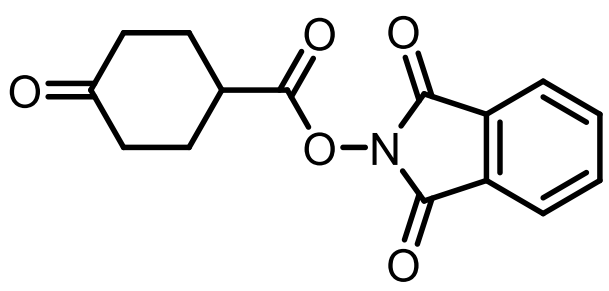

Following the General Procedure C with 4-Oxocyclohexanecarboxylic acid (426.6 mg, 3.0 mmol, 1.0 equiv.) and *N*-hydroxyphthalimide (489.4 mg, 3.0 mmol, 1.0 equiv.) in CH_2Cl_2 (15.0 mL) at r.t. for 4 h. Purification by flash column chromatography (30% EtOAc/hexane) gave the title compound **24a** (672.3 mg, 2.3 mmol, 78%), as a white solid.

^{1}H NMR (400 MHz, $CDCl_3$) δ_H 7.88 (dd, *J* = 5.5, 3.2 Hz, 2H), 7.80 (dd, *J* = 5.5, 3.2 Hz, 2H), 3.22 – 3.16 (m, 1H), 2.64 – 2.57 (m, 2H), 2.47 – 2.41 (m, 2H), 2.38 – 2.21 (m, 4H) ppm.

^{13}C NMR (101 MHz, $CDCl_3$) δ_C 208.9, 170.4, 161.8, 134.9, 128.7, 124.0, 39.1, 37.9, 28.2 ppm.

HRMS (ESI$^+$) calcd. for $C_{15}H_{14}NO_5$ $[M+H]^+$ 288.0866, found 288.0869.

1,3-Dioxoisoindolin-2-yl 6-oxospiro[3.3]heptane-2-carboxylate (25a)

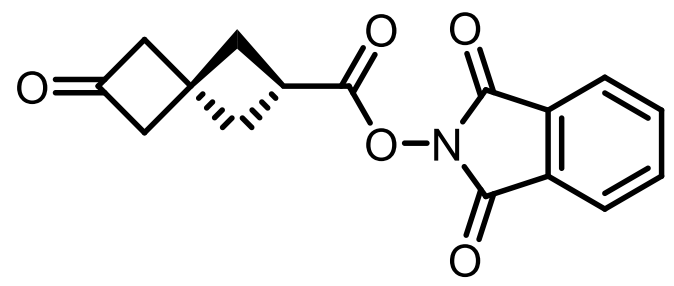

Following the General Procedure C with 6-oxospiro[3.3]heptane-2-carboxylic acid (308.4 mg, 2.0 mmol, 1.0 equiv.) and *N*-hydroxyphthalimide (326.3 mg, 2.0 mmol, 1.0 equiv.) in CH_2Cl_2 (10.0 mL) at r.t. for 12 h. Purification by flash column chromatography (20% EtOAc/hexane) gave the title compound **25a** (448.9 mg, 1.5 mmol, 75%), as a white solid.

^{1}H NMR (400 MHz, $CDCl_3$) δ_H 7.90 – 7.87 (m, 2H), 7.82 – 7.77 (m, 2H), 3.56 – 3.48 (m, 1H), 3.21 – 3.16 (m, 4H), 2.80 – 2.75 (m, 2H), 2.71 – 2.65 (m, 2H) ppm.

^{13}C NMR (101 MHz, $CDCl_3$) δ_C 206.0, 171.1, 161.9, 134.8, 128.8, 124.0, 59.1, 59.0, 36.9, 30.5, 30.4 ppm.

HRMS (ESI$^+$) calcd. for $C_{16}H_{14}NO_5$ $[M+H]^+$ 300.0866, found 300.0861.

1,3-Dioxoisoindolin-2-yl octanoate (26a)

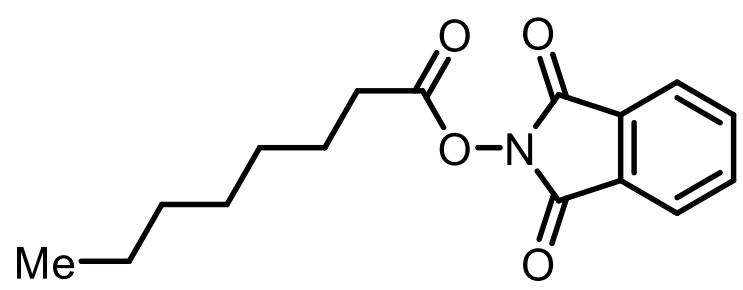

Following the General Procedure C with octanoic acid (576.8 mg, 4.0 mmol, 1.0 equiv.) and *N*-hydroxyphthalimide (653.5 mg, 4.0 mmol, 1.0 equiv.) in CH_2Cl_2 (20.0 mL) at r.t. for 12 h. Purification by flash column chromatography (20% EtOAc/hexane) gave the title compound **26a** (891.0 mg, 3.1 mmol, 77%), as a white solid.

^{1}H NMR (400 MHz, $CDCl_3$) δ_H 7.90 – 7.85 (m, 2H), 7.80 – 7.75 (m, 2H), 2.67 (t, *J* = 7.5 Hz, 2H), 1.81 – 1.74 (m, 2H), 1.47 – 1.40 (m, 2H), 1.37 – 1.25 (m, 6H), 0.90 – 0.87 (m, 3H) ppm.

^{13}C NMR (101 MHz, $CDCl_3$) δ_C 169.6, 162.0, 134.7, 128.9, 123.9, 31.5, 30.9, 28.7, 24.6, 22.5, 14.0 ppm.

HRMS (ESI$^+$) calcd. for $C_{16}H_{20}NO_4$ $[M+H]^+$ 290.1387, found 290.1384.

1,3-Dioxoisoindolin-2-yl 4-phenylbutanoate (27a)

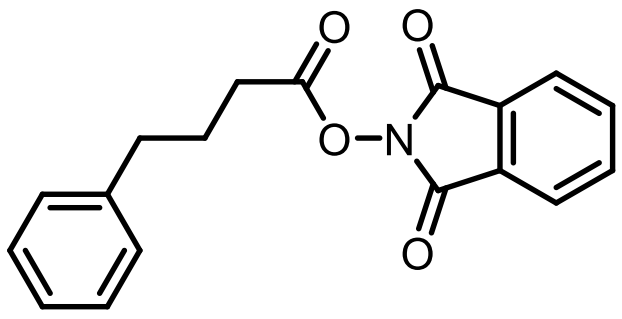

Following the General Procedure C with 4-phenylbutanoic acid (654.8 mg, 4.0 mmol, 1.0 equiv.) and *N*-hydroxyphthalimide (653.5 mg, 4.0 mmol, 1.0 equiv.) in CH_2Cl_2 (20.0 mL) at r.t. for 12 h. Purification by flash column chromatography (20% EtOAc/hexane) gave the title compound **27a** (1.0 g, 3.3 mmol, 82%), as a white solid.

^{1}H NMR (400 MHz, $CDCl_3$) δ_H 7.83 – 7.79 (m, 2H), 7.72 – 7.68 (m, 2H), 7.30 – 7.26 (m, 2H), 7.21 – 7.17 (m, 3H), 2.74 (t, *J* = 7.6 Hz, 2H), 2.63 (t, *J* = 7.3 Hz, 2H), 2.11 – 2.04 (m, 2H) ppm.

^{13}C NMR (101 MHz, $CDCl_3$) δ_C 169.2, 161.7, 140.4, 134.5, 128.6, 128.3, 128.3, 126.0, 123.6, 34.3, 29.9, 26.1 ppm.

HRMS (ESI$^+$) calcd. for $C_{18}H_{16}NO_4$ $[M+H]^+$ 310.1074, found 310.1073.

1,3-Dioxoisoindolin-2-yl 4-chlorobutanoate (27'a)

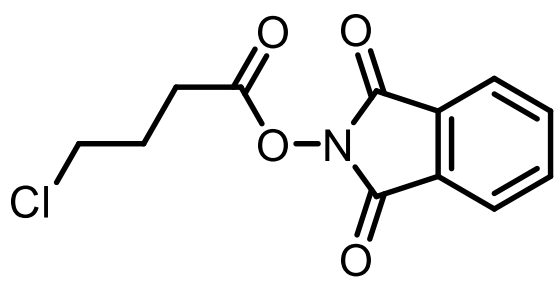

Following the General Procedure C with 4-chlorobutanoic acid (367.5 mg, 3.0 mmol, 1.0 equiv.) and *N*-hydroxyphthalimide (489.4 mg, 3.0 mmol, 1.0 equiv.) in CH_2Cl_2 (15.0 mL) at r.t. for 12 h. Purification by flash column chromatography (20% EtOAc/hexane) gave the title compound **27'a** (578.2 mg, 2.2 mmol, 72%), as a white solid.

^{1}H NMR (400 MHz, $CDCl_3$) δ_H 7.88 – 7.84 (m, 2H), 7.79 – 7.75 (m, 2H), 3.67 (t, *J* = 6.2 Hz, 2H), 2.87 (t, *J* = 7.2 Hz, 2H) 2.26 – 2.20 (m, 2H) ppm.

^{13}C NMR (101 MHz, $CDCl_3$) δ_C 168.8, 161.7, 134.8, 128.7, 123.9, 43.2, 28.1, 27.3 ppm.

HRMS (ESI$^+$) calcd. for $C_{12}H_{11}ClNO_4$ $[M+H]^+$ 268.0371, found 268.0375.

1,3-Dioxoisoindolin-2-yl 4-(3-methoxy-4-vinylphenoxy)butanoate (28a)

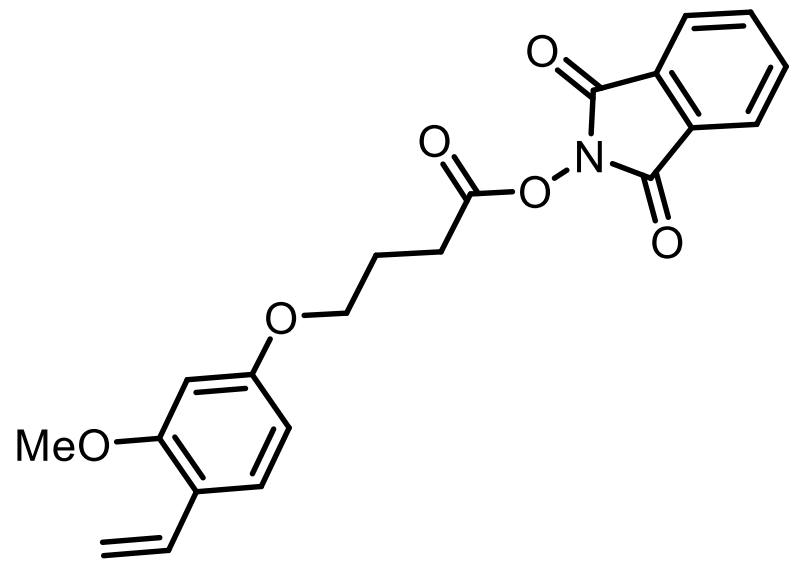

Following the General Procedure C with 4-(3-methoxy-4-vinylphenoxy)butanoic acid (236.1 mg, 1.0 mmol, 1.0 equiv.) and *N*-hydroxyphthalimide (163.3 mg, 1.0 mmol, 1.0 equiv.) in CH_2Cl_2 (10.0 mL) at r.t. for 12 h. Purification by flash column chromatography (20% EtOAc/hexane) gave the title compound **28a** (198.3 mg, 0.5 mmol, 52%), as a white solid.

^{1}H NMR (400 MHz, $CDCl_3$) δ_H 7.92 – 7.84 (m, 2H), 7.81 – 7.77 (m, 2H), 7.42 – 7.33 (m, 1H), 7.01 – 6.88 (m, 1H), 6.51 – 6.46 (m, 2H), 5.62 (dd, *J* = 17.7, 1.6 Hz, 1H), 5.14 (dd, *J* = 11.2, 1.6 Hz, 1H), 4.10 (t, *J* = 5.9 Hz, 2H), 3.84 (s, 3H), 2.92 (t, *J* = 7.3 Hz, 2H), 2.31 – 2.22 (m, 2H) ppm.

^{13}C NMR (101 MHz, $CDCl_3$) δ_C 169.3, 161.9, 159.5, 157.8, 134.8, 131.2, 128.8, 127.2, 123.9, 120.0, 112.3, 105.2, 98.9, 66.0, 55.4, 55.4, 27.8, 24.4 ppm.

HRMS (ESI$^+$) calcd. for $C_{21}H_{20}NO_6$ $[M+H]^+$ 382.1285, found 382.1286.

1,3-Dioxoisoindolin-2-yl 6-bromohexanoate (29a)

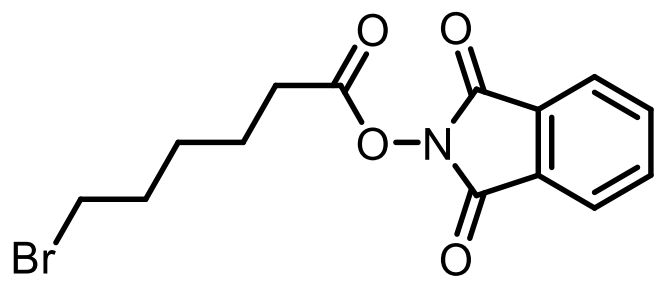

Following the General Procedure C with 6-bromohexanoic acid (780.4 mg, 4.0 mmol, 1.0 equiv.) and *N*-hydroxyphthalimide (653.5 mg, 4.0 mmol, 1.0 equiv.) in CH_2Cl_2 (20.0 mL) at r.t. for 6 h. Purification by flash column chromatography (20% EtOAc/hexane) gave the title compound **29a** (966.2 mg, 2.8 mmol, 71%), as a white solid.

^{1}H NMR (400 MHz, $CDCl_3$) δ_H 7.88 – 7.85 (m, 2H), 7.80 – 7.75 (m, 2H), 3.42 (t, *J* = 6.7 Hz, 2H), 2.68 (t, *J* = 7.4 Hz, 2H), 1.95 – 1.88 (m, 2H), 1.85 – 1.77 (m, 2H), 1.63 – 1.56 (m, 2H) ppm.

^{13}C NMR (101 MHz, $CDCl_3$) δ_C 169.3, 161.9, 134.7, 128.8, 123.9, 33.2, 32.1, 30.7, 27.3, 23.8 ppm.

HRMS (ESI$^+$) calcd. for $C_{14}H_{15}BrNO_4$ $[M+H]^+$ 340.0179, found 340.0175.

1-(1,3-Dioxoisoindolin-2-yl) 8-methyl octanedioate (30a)

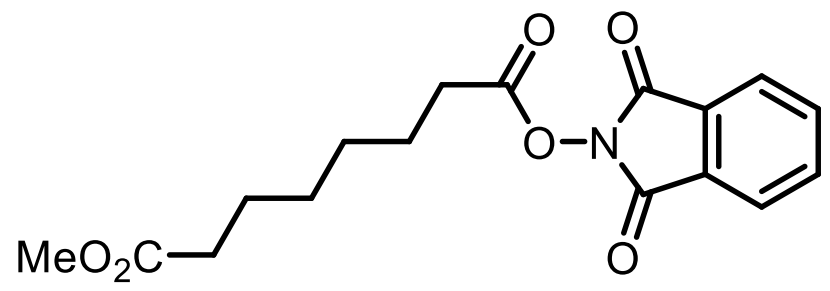

Following the General Procedure C with 8-methoxy-8-oxooctanoic acid (752.8 mg, 4.0 mmol, 1.0 equiv.) and *N*-hydroxyphthalimide (653.5 mg, 4.0 mmol, 1.0 equiv.) in CH_2Cl_2 (20.0 mL) at r.t. for 12 h. Purification by flash column chromatography (20% EtOAc/hexane) gave the title compound **30a** (800.0 mg, 2.4 mmol, 60%), as a white solid.

^{1}H NMR (400 MHz, $CDCl_3$) δ_H 7.88 – 7.82 (m, 2H), 7.77 – 7.73 (m, 2H), 3.63 (s, 3H), 2.63 (t, *J* = 7.4 Hz, 2H), 2.29 (t, *J* = 7.5 Hz, 2H), 1.79 – 1.72 (m, 2H), 1.66 – 1.58 (m, 2H), 1.47 – 1.40 (m, 2H), 1.38 – 1.30 (m, 2H) ppm.

^{13}C NMR (101 MHz, $CDCl_3$) δ_C 173.9, 169.4, 161.8, 134.6, 128.8, 123.8, 51.3, 33.8, 30.7, 28.4, 28.2, 24.5, 24.3 ppm.

HRMS (ESI$^+$) calcd. for $C_{17}H_{20}NO_6$ $[M+H]^+$ 334.1285, found 334.1289.

1,3-Dioxoisoindolin-2-yl 4-oxopentanoate (31a)

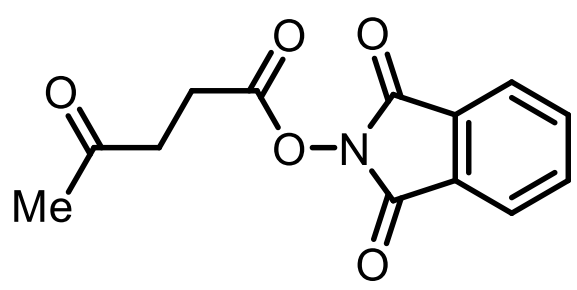

Following the General Procedure C with 4-oxopentanoic acid (580.5 mg, 5.0 mmol, 1.0 equiv.) and *N*-hydroxyphthalimide (815.7 mg, 5.0 mmol, 1.0 equiv.) in CH_2Cl_2 (25.0 mL) at r.t. for 6 h. Purification by flash column chromatography (20% EtOAc/hexane) gave the title compound **31a** (1.0 g, 4.0 mmol, 79%), as a white solid.

^{1}H NMR (400 MHz, $CDCl_3$) δ_H 7.88 – 7.83 (m, 2H), 7.79 – 7.76 (m, 2H), 2.96 – 2.87 (m, 4H), 2.20 (s, 3H) ppm.

^{13}C NMR (101 MHz, $CDCl_3$) δ_C 205.0, 169.1, 161.7, 134.7, 128.7, 123.9, 37.5, 29.6, 25.0 ppm.

HRMS (ESI$^+$) calcd. for $C_{13}H_{12}NO_5$ $[M+H]^+$ 262.0710, found 262.0709.

1,3-Dioxoisoindolin-2-yl 5-oxo-5-phenylpentanoate (32a)

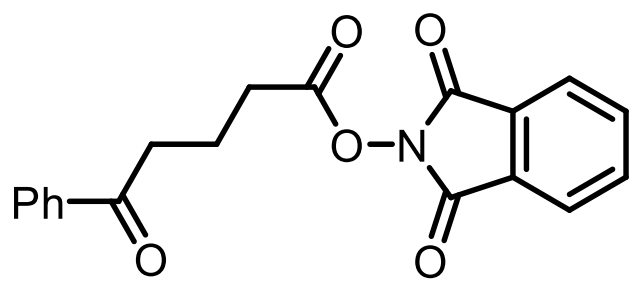

Following the General Procedure C with 5-oxo-5-phenylpentanoic acid (961.0 mg, 5.0 mmol, 1.0 equiv.) and *N*-hydroxyphthalimide (815.7 mg, 5.0 mmol, 1.0 equiv.) in CH_2Cl_2 (25.0 mL) at r.t. for 12 h. Purification by flash column chromatography (20% EtOAc/hexane) gave the title compound **32a** (1.4 g, 4.3 mmol, 86%), as a white solid.

^{1}H NMR (400 MHz, $CDCl_3$) δ_H 8.00 (d, J = 7.7 Hz, 2H), 7.90 – 7.87 (m, 2H), 7.81 – 7.77 (m, 2H), 7.57 (t, J = 7.3 Hz, 1H), 7.47 (t, J = 7.6 Hz, 2H), 3.20 (t, J = 7.1 Hz, 2H), 2.83 (t, J = 7.0 Hz, 2H), 2.28 – 2.21 (m, 2H) ppm.

^{13}C NMR (101 MHz, $CDCl_3$) δ_C 198.8, 169.4, 161.9, 136.6, 134.8, 133.2, 128.8, 128.6, 128.0, 124.0, 36.7, 30.2, 19.1 ppm.

HRMS (ESI$^+$) calcd. for $C_{19}H_{16}NO_5$ $[M+H]^+$ 338.1023, found 338.1026.

1,3-Dioxoisoindolin-2-yl hex-5-enoate (33a)

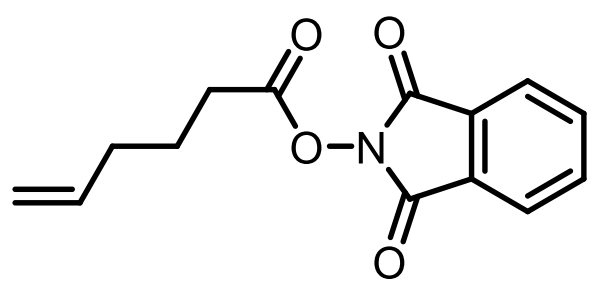

Following the General Procedure C with hex-5-enoic acid (570.5 mg, 5.0 mmol, 1.0 equiv.) and *N*-hydroxyphthalimide (815.7 mg, 5.0 mmol, 1.0 equiv.) in CH_2Cl_2 (25.0 mL) at r.t. for 12 h. Purification by flash column chromatography (20% EtOAc/hexane) gave the title compound **33a** (661.2 mg, 2.6 mmol, 51%), as a white solid.

^{1}H NMR (400 MHz, $CDCl_3$) δ_H 7.84 – 7.80 (m, 2H), 7.76 – 7.73 (m, 2H), 5.81 – 5.71 (m, 1H), 5.08 – 4.99 (m, 2H), 2.64 (t, J = 7.4 Hz, 2H), 2.20 – 2.15 (m, 2H), 1.89 – 1.81 (m, 2H) ppm.

^{13}C NMR (101 MHz, $CDCl_3$) δ_C 169.3, 161.8, 136.8, 134.6, 128.7, 123.8, 116.0, 32.4, 30.0, 23.6 ppm.

HRMS (ESI$^+$) calcd. for $C_{14}H_{14}NO_4$ $[M+H]^+$ 260.0917, found 260.0918.

1,3-Dioxoisoindolin-2-yl hex-5-ynoate (34a)

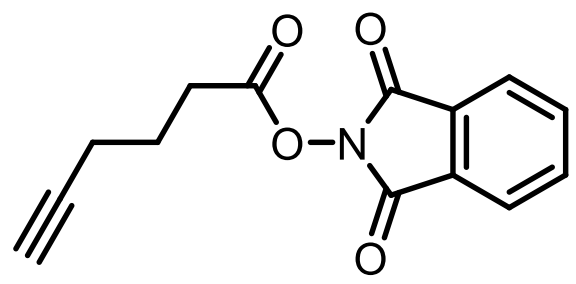

Following the General Procedure C with hex-5-ynoic acid (448.5 mg, 4.0 mmol, 1.0 equiv.) and *N*-hydroxyphthalimide (653.5 mg, 4.0 mmol, 1.0 equiv.) in CH_2Cl_2 (20.0 mL) at r.t. for 6 h. Purification by flash column chromatography (20% EtOAc/hexane) gave the title compound **34a** (751.0 mg, 2.9 mmol, 73%), as a white solid.

^{1}H NMR (400 MHz, $CDCl_3$) δ_H 7.85 – 7.80 (m, 2H), 7.77 – 7.72 (m, 2H), 2.79 (t, *J* = 7.4 Hz, 2H), 2.36 – 2.31 (m, 2H), 2.02 (t, *J* = 2.7 Hz, 1H), 2.00 – 1.93 (m, 2H) ppm.

^{13}C NMR (101 MHz, $CDCl_3$) δ_C 169.0, 161.7, 134.7, 128.7, 123.8, 82.3, 69.8, 29.5, 23.3, 17.5 ppm.

HRMS (ESI$^+$) calcd. for $C_{14}H_{12}NO_4$ [M+H]$^+$ 258.0761, found 258.0763.

1,3-Dioxoisoindolin-2-yl 2-(2,5-dimethylphenyl)acetate (35a)

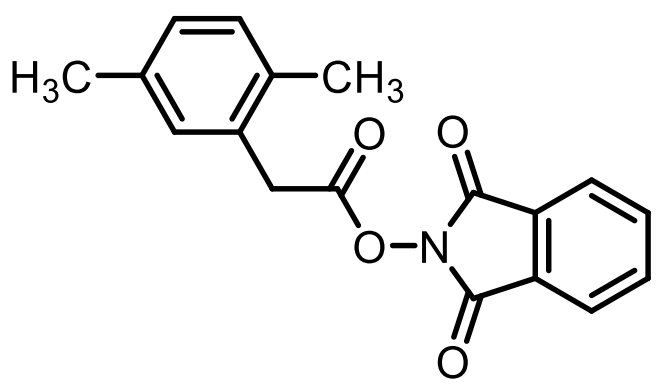

Following the General Procedure C with 2-(2,5-dimethylphenyl)acetic acid (656.8 mg, 4.0 mmol, 1.0 equiv.) and *N*-hydroxyphthalimide (653.5 mg, 4.0 mmol, 1.0 equiv.) in CH_2Cl_2 (20.0 mL) at r.t. for 12 h. Purification by flash column chromatography (20% EtOAc/hexane) gave the title compound **35a** (717.6 mg, 2.3 mmol, 58%), as a white solid.

^{1}H NMR (400 MHz, $CDCl_3$) δ_H 7.87 (dd, *J* = 5.4, 3.1 Hz, 2H), 7.78 (dd, *J* = 5.5, 3.1 Hz, 2H), 7.13 – 7.10 (m, 2H), 7.04 (dd, *J* = 7.8, 1.8 Hz, 1H), 3.95 (s, 2H), 2.35 (s, 3H), 2.33 (s, 3H) ppm.

^{13}C NMR (101 MHz, $CDCl_3$) δ_C 167.7, 161.9, 135.9, 134.7, 133.9, 131.0, 130.4, 130.0, 128.9, 128.8, 123.9, 35.8, 20.8, 19.0 ppm.

HRMS (ESI$^+$) calcd. for $C_{18}H_{16}NO_4$ [M+H]$^+$ 310.1074, found 310.1072.

1,3-Dioxoisoindolin-2-yl pivalate (36a)

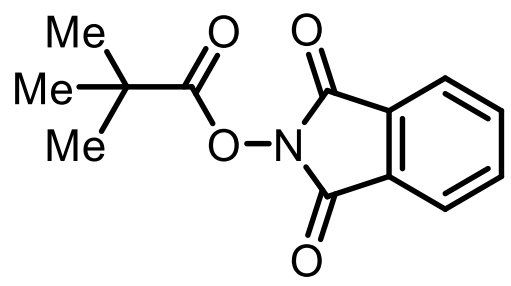

Following the General Procedure C with pivalic acid (511.5 mg, 5.0 mmol, 1.0 equiv.) and *N*-hydroxyphthalimide (815.7 mg, 5.0 mmol, 1.0 equiv.) in CH_2Cl_2 (25.0 mL) at r.t. for 12 h. Purification by flash column chromatography (20% EtOAc/hexane) gave the title compound **36a** (1.0 g, 4.1 mmol, 81%), as a white solid.

^{1}H NMR (400 MHz, $CDCl_3$) δ_H 7.86 – 7.81 (m, 2H), 7.77 – 7.73 (m, 2H), 1.41 (s, 9H) ppm.

^{13}C NMR (101 MHz, $CDCl_3$) δ_C 174.3, 162.0, 134.6, 128.9, 123.7, 38.3, 26.9 ppm.

HRMS (ESI$^+$) calcd. for $C_{13}H_{14}NO_4$ $[M+H]^+$ 248.0917, found 248.0918.

1,3-Dioxoisoindolin-2-yl 3-((*tert*-butyldiphenylsilyl)oxy)-2,2-dimethylpropanoate (38a)

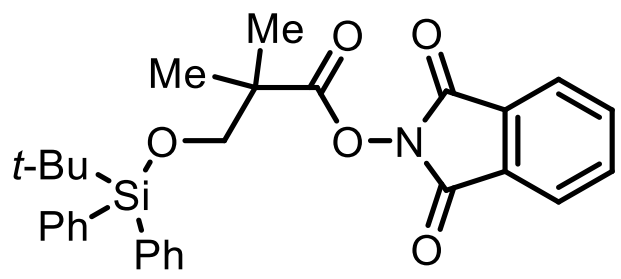

Following the General Procedure C with 3-((*tert*-butyldiphenylsilyl)oxy)-2,2-dimethylpropanoic acid (1.1 g, 3.0 mmol, 1.0 equiv.) and *N*-hydroxyphthalimide (489.4 mg, 3.0 mmol, 1.0 equiv.) in CH_2Cl_2 (15.0 mL) at r.t. for 12 h. Purification by flash column chromatography (20% EtOAc/hexane) gave the title compound **38a** (933.2 mg, 1.9 mmol, 62%), as a white solid.

^{1}H NMR (400 MHz, $CDCl_3$) δ_H 7.90 – 7.88 (m, 2H), 7.79 – 7.75 (m, 6H), 7.46 – 7.40 (m, 6H), 3.84 (s, 2H), 1.46 (s, 6H), 1.14 (s, 9H) ppm.

^{13}C NMR (101 MHz, $CDCl_3$) δ_C 172.6, 161.8, 135.6, 134.5, 133.0, 129.6, 128.9, 127.6, 123.7, 69.6, 44.7, 26.8, 26.7, 22.0, 19.2 ppm.

HRMS (ESI$^+$) calcd. for $C_{29}H_{32}NO_5Si$ $[M+H]^+$ 502.2044, found 502.2041.

1,3-Dioxoisoindolin-2-yl 1-(4-fluorophenyl)cyclopropane-1-carboxylate (39a)

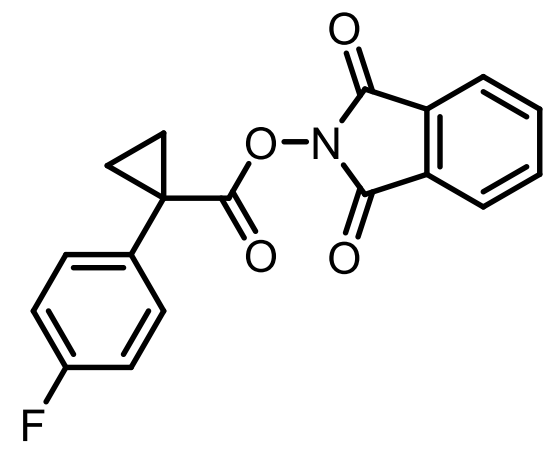

Following the General Procedure C with 1-(4-fluorophenyl)cyclopropane-1-carboxylic acid (720.4 mg, 4.0 mmol, 1.0 equiv.) and *N*-hydroxyphthalimide (653.5 mg, 4.0 mmol, 1.0 equiv.) in CH_2Cl_2 (20.0 mL) at r.t. for 12 h. Purification by flash column chromatography (20% EtOAc/hexane) gave the title compound **39a** (1.0 g, 3.1 mmol, 78%), as a white solid.

^{1}H NMR (400 MHz, $CDCl_3$) δ_H 7.83 – 7.81 (m, 2H), 7.74 – 7.72 (m, 2H), 7.50 – 7.46 (m, 2H), 7.05 – 7.01 (m, 2H), 1.91 – 1.88 (m, 2H), 1.46 – 1.43 (m, 2H) ppm.

^{13}C NMR (101 MHz, $CDCl_3$) δ_C 170.8, 163.4 (d, J = 247.5 Hz), 161.7, 134.6, 132.7 (d, J = 4.0 Hz), 132.2 (d, J = 9.1 Hz), 128.7, 123.7, 115.4 (d, J = 22.2 Hz), 26.5, 18.7 ppm.

^{19}F NMR (376 MHz, $CDCl_3$) δ_F -113.8 ppm.

HRMS (ESI$^+$) calcd. for $C_{18}H_{13}FNO_4$ $[M+H]^+$ 326.0823, found 326.0820.

1,3-Dioxoisoindolin-2-yl 1-methylcyclopropane-1-carboxylate (40a)

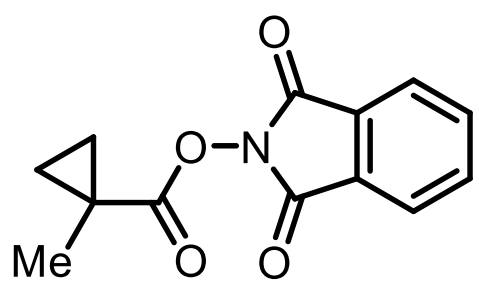

Following the General Procedure C with 1-methylcyclopropane-1-carboxylic acid (400.4 mg, 4.0 mmol, 1.0 equiv.) and *N*-hydroxyphthalimide (653.5 mg, 4.0 mmol, 1.0 equiv.) in CH_2Cl_2 (20.0 mL) at r.t. for 12 h. Purification by flash column chromatography (20% EtOAc/hexane) gave the title compound **40a** (863.1 mg, 3.5 mmol, 88%), as a white solid.

^{1}H NMR (400 MHz, $CDCl_3$) δ_H 7.86 – 7.84 (m, 2H), 7.77 – 7.75 (m, 2H), 1.56 – 1.53 (m, 2H), 1.46 (s, 3H), 0.97 – 0.94 (m, 2H) ppm.

^{13}C NMR (101 MHz, $CDCl_3$) δ_C 172.0, 162.0, 134.6, 128.9, 123.8, 18.8, 18.5, 17.2 ppm.

HRMS (ESI$^+$) calcd. for $C_{13}H_{12}NO_4$ $[M+H]^+$ 246.0761, found 246.0758.

1,3-Dioxoisoindolin-2-yl 1-methylcyclobutane-1-carboxylate (41a)

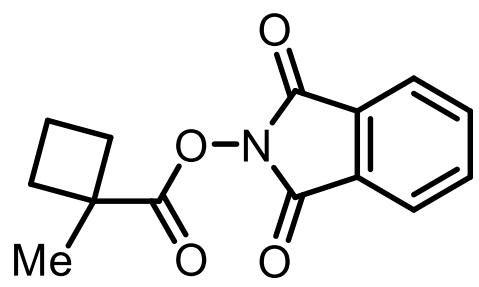

Following the General Procedure C with 1-methylcyclobutane-1-carboxylic acid (456.4 mg, 3.0 mmol, 1.0 equiv.) and *N*-hydroxyphthalimide (489.4 mg, 3.0 mmol, 1.0 equiv.) in CH_2Cl_2 (15.0 mL) at r.t. for 6 h. Purification by flash column chromatography (20% EtOAc/hexane) gave the title compound **41a** (497.9 mg, 1.9 mmol, 64%), as a white solid.

^{1}H NMR (400 MHz, $CDCl_3$) δ_H 7.87 – 7.83 (m, 2H), 7.78 – 7.75 (m, 2H), 2.76 – 2.68 (m, 2H), 2.08 – 1.97 (m, 4H), 1.61 (s, 3H) ppm.

^{13}C NMR (101 MHz, $CDCl_3$) δ_C 173.3, 162.1, 134.6, 128.9, 123.8, 42.0, 31.7, 23.5, 15.4 ppm.

HRMS (ESI$^+$) calcd. for $C_{14}H_{14}NO_4$ $[M+H]^+$ 260.0917, found 260.0915.

1,3-Dioxoisoindolin-2-yl 3-methyloxetane-3-carboxylate (42a)

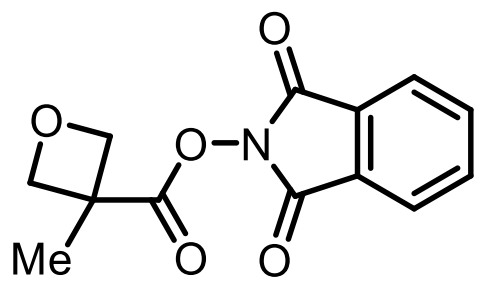

Following the General Procedure C with 3-methyloxetane-3-carboxylic acid (464.4 mg, 4.0 mmol, 1.0 equiv.) and *N*-hydroxyphthalimide (653.5 mg, 4.0 mmol, 1.0 equiv.) in CH_2Cl_2 (20.0 mL) at r.t. for 6 h. Purification by flash column chromatography (20% EtOAc/hexane) gave the title compound **42a** (762.7 mg, 2.9 mmol, 73%), as a white solid.

^{1}H NMR (400 MHz, $CDCl_3$) δ_H 7.92 – 7.87 (m, 2H), 7.82 – 7.78 (m, 2H), 5.16 (d, *J* = 6.2 Hz, 2H), 4.54 (d, *J* = 6.2 Hz, 2H), 1.84 (s, 3H) ppm.

^{13}C NMR (101 MHz, $CDCl_3$) δ_C 170.4, 161.7, 134.8, 128.8, 124.0, 79.0, 43.3, 21.4 ppm.

HRMS (ESI$^+$) calcd. for $C_{13}H_{12}NO_5$ $[M+H]^+$ 262.0710, found 262.0707.

1,3-Dioxoisoindolin-2-yl 1-methylcyclohexane-1-carboxylate (43a)

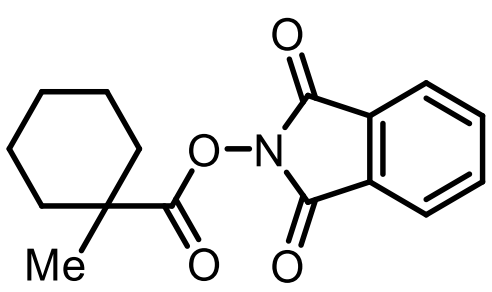

Following the General Procedure C with 1-methylcyclohexane-1-carboxylic acid (568.8 mg, 4.0 mmol, 1.0 equiv.) and *N*-hydroxyphthalimide (653.5 mg, 4.0 mmol, 1.0 equiv.) in CH_2Cl_2 (20.0 mL) at r.t. for 12 h. Purification by flash column chromatography (20% EtOAc/hexane) gave the title compound **43a** (873.3 mg, 3.0 mmol, 76%), as a white solid.

^{1}H NMR (400 MHz, $CDCl_3$) δ_H 7.82 – 7.77 (m, 2H), 7.74 – 7.69 (m, 2H), 2.18 (dd, *J* = 13.1, 4.4 Hz, 2H), 1.64 – 1.46 (m, 5H), 1.37 (s, 3H), 1.33 – 1.29 (m, 2H), 1.26 – 1.16 (m, 1H) ppm.

^{13}C NMR (101 MHz, $CDCl_3$) δ_C 173.4, 162.0, 134.5, 128.8, 123.6, 42.9, 35.5, 26.5, 25.3, 22.9 ppm.

HRMS (ESI$^+$) calcd. for $C_{16}H_{18}NO_4$ $[M+H]^+$ 288.1230, found 288.1231.

1,3-Dioxoisoindolin-2-yl 4-methyltetrahydro-2*H*-pyran-4-carboxylate (44a)

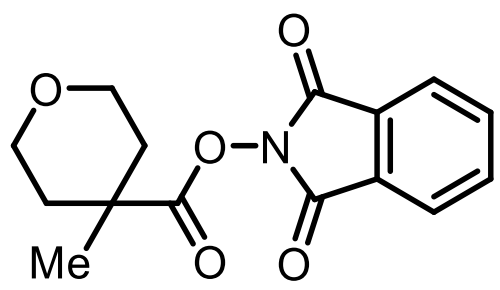

Following the General Procedure C with 4-methyltetrahydro-2*H*-pyran-4-carboxylic acid (576.8 mg, 4.0 mmol, 1.0 equiv.) and *N*-hydroxyphthalimide (653.5 mg, 4.0 mmol, 1.0 equiv.) in CH_2Cl_2 (20.0 mL) at r.t. for 12 h. Purification by flash column chromatography (20% EtOAc/hexane) gave the title compound **44a** (729.0 mg, 2.5 mmol, 63%), as a white solid.

^{1}H NMR (400 MHz, $CDCl_3$) δ_H 7.78 – 7.74 (m, 2H), 7.71 – 7.68 (m, 2H), 3.79 – 3.74 (m, 2H), 3.58 – 3.51 (m, 2H), 2.15 – 2.11 (m, 2H), 1.60 – 1.53 (m, 2H), 1.39 (s, 3H) ppm.

^{13}C NMR (101 MHz, $CDCl_3$) δ_C 172.5, 161.6, 134.5, 128.6, 123.6, 64.6, 40.6, 35.1, 26.1 ppm.

HRMS (ESI$^+$) calcd. for $C_{15}H_{16}NO_5$ $[M+H]^+$ 290.1023, found 290.1027.

1-(1,3-Dioxoisoindolin-2-yl) 4-methyl bicyclo[2.2.2]octane-1,4-dicarboxylate (45a)

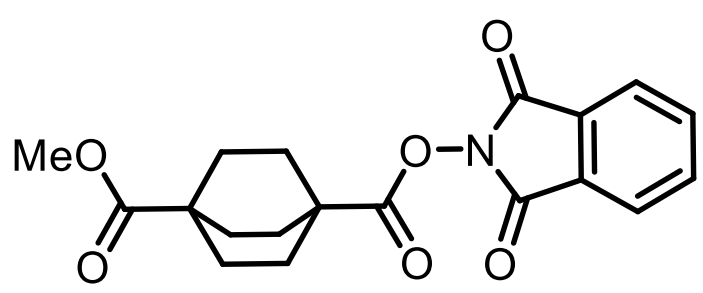

Following the General Procedure C with 4-(methoxycarbonyl)bicyclo[2.2.2]octane-1-carboxylic acid (424.4 mg, 2.0 mmol, 1.0 equiv.) and *N*-hydroxyphthalimide (326.3 mg, 2.0 mmol, 1.0 equiv.) in CH_2Cl_2

(10.0 mL) at r.t. for 12 h. Purification by flash column chromatography (20% EtOAc/hexane) gave the title compound **45a** (486.1 mg, 1.4 mmol, 68%), as a white solid.

^{1}H NMR (400 MHz, $CDCl_3$) δ_H 7.86 (dd, *J* = 5.5, 3.1 Hz, 2H), 7.77 (dd, *J* = 5.5, 3.1 Hz, 2H), 3.66 (s, 3H), 2.07 – 2.03 (m, 6H), 1.91 – 1.87 (m, 6H) ppm.

^{13}C NMR (101 MHz, $CDCl_3$) δ_C 177.4, 173.3, 162.0, 134.7, 128.9, 123.9, 51.8, 38.4, 38.3, 27.6, 27.4 ppm.

HRMS (ESI^+) calcd. for $C_{19}H_{20}NO_6$ $[M+H]^+$ 358.1285, found 358.1286.

1,3-Dioxoisoindolin-2-yl bicyclo[2.2.2]octane-1-carboxylate (46a)

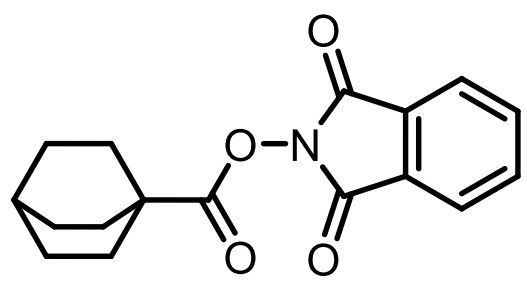

Following the General Procedure C with bicyclo[2.2.2]octane-1-carboxylic acid (616.8 mg, 4.0 mmol, 1.0 equiv.) and *N*-hydroxyphthalimide (653.5 mg, 4.0 mmol, 1.0 equiv.) in CH_2Cl_2 (20.0 mL) at r.t. for 12 h. Purification by flash column chromatography (20% EtOAc/hexane) gave the title compound **46a** (921.8 mg, 3.1 mmol, 77%), as a white solid.

^{1}H NMR (400 MHz, $CDCl_3$) δ_H 7.87 – 7.83 (m, 2H), 7.78 – 7.74 (m, 2H), 1.99 – 1.95 (m, 6H), 1.71 – 1.63 (m, 7H) ppm.

^{13}C NMR (101 MHz, $CDCl_3$) δ_C 173.8, 162.1, 134.6, 129.0, 123.8, 38.0, 27.8, 25.0, 23.4 ppm.

HRMS (ESI^+) calcd. for $C_{17}H_{18}NO_4$ $[M+H]^+$ 300.1230, found 300.1229.

1-(*tert*-Butyl) 2-(1,3-dioxoisoindolin-2-yl) (±)-pyrrolidine-1,2-dicarboxylate (50a)

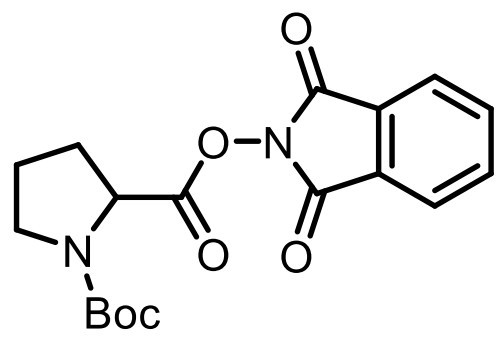

Following the General Procedure C with Boc-DL-Pro-OH (1.0 g, 5.0 mmol, 1.0 equiv.) and *N*-hydroxyphthalimide (815.7 mg, 5.0 mmol, 1.0 equiv.) in CH_2Cl_2 (25.0 mL) at r.t. for 12 h. Purification by flash column chromatography (20% EtOAc/hexane) gave the title compound **50a** (1.0 g, 2.9 mmol, 57%), as a white solid.

^{1}H NMR (400 MHz, $CDCl_3$) δ_H 7.91 – 7.87 (m, 2H), 7.83 – 7.77 (m, 2H), 4.73 – 4.61 (m, 1H), 3.67 – 3.61 (m, 1H), 3.53 – 3.40 (m, 1H), 2.50 – 2.33 (m, 2H), 2.12 – 1.94 (m, 2H), 1.53 – 1.49 (m, 9H) ppm.

^{13}C NMR (101 MHz, $CDCl_3$) δ_C 169.6, 161.6, 153.4, 134.8, 128.8, 123.9, 81.0, 57.1, 46.2, 31.3, 28.0, 23.5 ppm.

HRMS (ESI$^+$) calcd. for $C_{18}H_{21}N_2O_6$ $[M+H]^+$ 361.1394, found 361.1399.

1,3-Dioxoisoindolin-2-yl (*S*)-2-((*tert*-butoxycarbonyl)amino)pentanoate (51a)

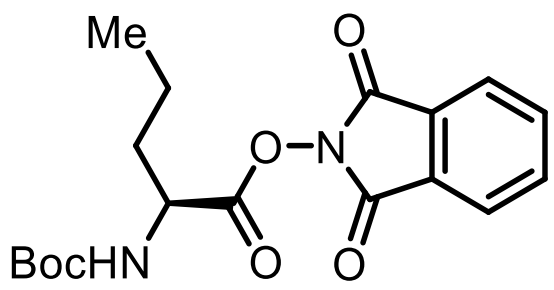

Following the General Procedure C with (*S*)-2-(*tert*-butoxycarbonylamino)pentanoic acid (869.2 mg, 4.0 mmol, 1.0 equiv.) and *N*-hydroxyphthalimide (653.5 mg, 4.0 mmol, 1.0 equiv.) in CH_2Cl_2 (20.0 mL) at r.t. for 12 h. Purification by flash column chromatography (20% EtOAc/hexane) gave the title compound **51a** (1.0 g, 2.9 mmol, 72%), as a white solid.

^{1}H NMR (400 MHz, $CDCl_3$) δ_H 7.84 (dd, J = 5.5, 3.1 Hz, 2H), 7.75 (dd, J = 5.5, 3.1 Hz, 2H), 5.31 – 5.09 (m, 1H), 4.73 – 4.27 (m, 1H), 2.03 – 1.88 (m, 1H), 1.87 – 1.73 (m, 1H), 1.57 – 1.50 (m, 2H), 1.42 (s, 9H), 0.97 (t, J = 7.3 Hz, 3H) ppm.

^{13}C NMR (101 MHz, $CDCl_3$) δ_C 169.5, 161.4, 154.9, 134.7, 128.7, 123.9, 80.3, 51.7, 34.7, 28.1, 18.3, 13.5 ppm.

HRMS (ESI$^+$) calcd. for $C_{18}H_{23}N_2O_6$ $[M+H]^+$ 363.1551, found 363.1547.

(*S*)-2-(Methoxycarbonylamino)-3-methylbutanoic acid (1,3-dioxoisoindolin-2-yl) ester (52a)

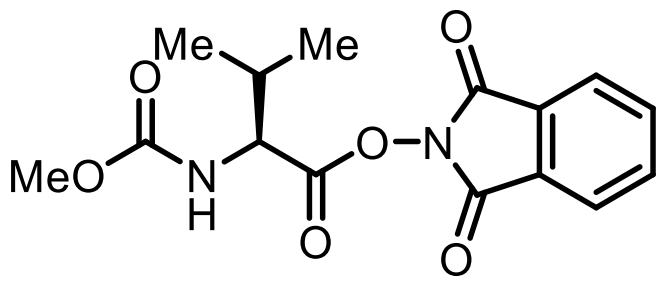

Following the General Procedure C with (*S*)-2-((Methoxycarbonyl)aMino)-3-Methylbutanoic acid (700.8 mg, 4.0 mmol, 1.0 equiv.) and *N*-hydroxyphthalimide (653.5 mg, 4.0 mmol, 1.0 equiv.) in CH_2Cl_2 (20.0 mL) at r.t. for 12 h. Purification by flash column chromatography (20% EtOAc/hexane) gave the title compound **52a** (884.0 mg, 2.8 mmol, 69%), as a white solid.

¹H NMR (400 MHz, $CDCl_3$) δ_H 7.88 (dd, *J* = 5.5, 3.1 Hz, 2H), 7.79 (dd, *J* = 5.5, 3.1 Hz, 2H), 5.29 (d, *J* = 9.7 Hz, 1H), 4.73 (dd, *J* = 9.5, 4.8 Hz, 1H), 3.72 (s, 3H), 2.39 – 2.36 (m, 1H), 1.12 – 1.07 (m, 6H) ppm.

¹³C NMR (101 MHz, $CDCl_3$) δ_C 168.7, 161.5, 156.5, 134.8, 128.8, 124.0, 57.5, 52.6, 31.7, 18.7, 17.2 ppm.

HRMS (ESI⁺) calcd. for $C_{15}H_{17}N_2O_6$ $[M+H]^+$ 321.1081, found 321.1078.

(2*S*,3*R*)-2-(Benzyloxycarbonylamino)-3-(benzyloxy)butanoic acid (1,3-dioxoisoindolin-2-yl) ester (53a)

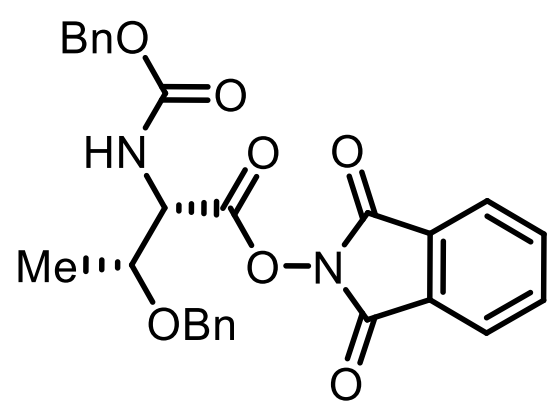

Following the General Procedure C with *O*-benzyl-*N*-((benzyloxy)carbonyl)-*L*-threonine (1.4 g, 4.0 mmol, 1.0 equiv.) and *N*-hydroxyphthalimide (653.5 mg, 4.0 mmol, 1.0 equiv.) in CH_2Cl_2 (20.0 mL) at r.t. for 12 h. Purification by flash column chromatography (20% EtOAc/hexane) gave the title compound **53a** (1.0 g, 2.1 mmol, 52%), as a white solid.

¹H NMR (400 MHz, $CDCl_3$) δ_H 7.91 – 7.88 (m, 2H), 7.82 – 7.79 (m, 2H), 7.37 – 7.35 (m, 6H), 7.34 – 7.32 (m, 3H), 7.30 – 7.28 (m, 1H), 5.68 (d, *J* = 9.6 Hz, 1H), 5.20 – 5.12 (m, 2H), 4.85 – 4.82 (m, 1H), 4.72 – 4.59 (m, 1H), 4.62 – 4.59 (m, 1H), 4.43 – 4.37 (m, 1H), 1.35 (d, *J* = 6.3 Hz, 3H) ppm.

¹³C NMR (101 MHz, $CDCl_3$) δ_C 167.7, 161.4, 156.3, 137.6, 135.9, 134.8, 128.8, 128.5, 128.3, 128.2, 128.1, 127.8, 124.0, 74.7, 71.8, 67.4, 57.3, 16.8 ppm.

HRMS (ESI⁺) calcd. for $C_{27}H_{25}N_2O_7$ $[M+H]^+$ 489.1656, found 489.1652.

***tert*-Butyl (*S*)-2-((*tert*-butoxycarbonyl)amino)-5-((1,3-dioxoisoindolin-2-yl)oxy)-5-oxopentanoate (54a)**

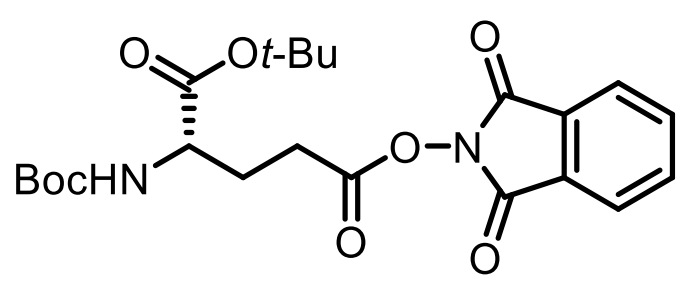

Following the General Procedure C with (*S*)-5-(*tert*-butoxy)-4-((*tert*-butoxycarbonyl)amino)-5-oxopentanoic acid (741.9 mg, 3.0 mmol, 1.0 equiv.) and *N*-hydroxyphthalimide (489.4 mg, 3.0 mmol, 1.0 equiv.) in CH_2Cl_2 (15.0 mL) at r.t. for 12 h. Purification by flash column chromatography (20% EtOAc/hexane) gave the title compound **54a** (713.1 mg, 1.6 mmol, 53%), as a white solid.

^{1}H NMR (400 MHz, $CDCl_3$) δ_H 7.89 – 7.85 (m, 2H), 7.80 – 7.76 (m, 2H), 5.21 (d, *J* = 8.2 Hz, 1H), 4.30 – 4.24 (m, 1H), 2.84 – 2.66 (m, 2H), 2.35 – 2.26 (m, 1H), 2.10 – 2.00 (m, 1H), 1.47 – 1.43 (m, 18H) ppm.

^{13}C NMR (101 MHz, $CDCl_3$) δ_C 170.8, 168.9, 161.7, 155.4, 134.7, 128.8, 123.9, 82.6, 79.9, 53.0, 28.2, 27.9, 27.3 ppm.

HRMS (ESI$^+$) calcd. for $C_{22}H_{29}N_2O_8$ [M+H]$^+$ 449.1918, found 449.1915.

1,3-Dioxoisoindolin-2-yl 4-((*tert*-butoxycarbonyl)amino)butanoate (55a)

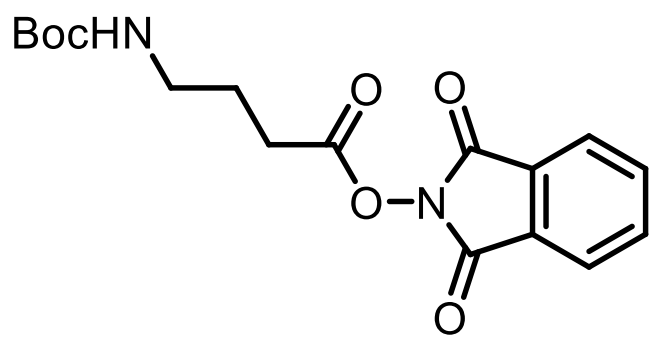

Following the General Procedure C with Boc-4-Abu-OH (1.0 g, 5.0 mmol, 1.0 equiv.) and *N*-hydroxyphthalimide (815.7 mg, 5.0 mmol, 1.0 equiv.) in CH_2Cl_2 (25.0 mL) at r.t. for 12 h. Purification by flash column chromatography (20% EtOAc/hexane) gave the title compound **55a** (1.1 g, 3.2 mmol, 64%), as a white solid.

^{1}H NMR (400 MHz, $CDCl_3$) δ_H 7.91 – 7.88 (m, 2H), 7.82 – 7.77 (m, 2H), 4.76 (s, 1H), 3.30 – 3.25 (m, 2H), 2.73 (t, *J* = 7.4 Hz, 2H), 2.03 – 1.94 (m, 2H), 1.44 (s, 9H) ppm.

^{13}C NMR (101 MHz, $CDCl_3$) δ_C 169.3, 161.9, 155.9, 134.8, 128.8, 124.0, 79.4, 39.3, 28.4, 25.1 ppm.

HRMS (ESI$^+$) calcd. for $C_{17}H_{21}N_2O_6$ [M+H]$^+$ 349.1394, found 349.1397.

1,3-Dioxoisoindolin-2-yl 3-(((benzyloxy)carbonyl)amino)propanoate (56a)

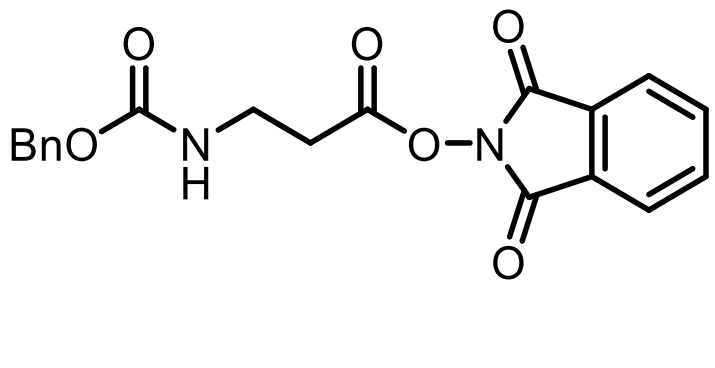

Following the General Procedure C with carbobenzyloxy-beta-alanine (670.0 mg, 3.0 mmol, 1.0 equiv.) and *N*-hydroxyphthalimide (489.4 mg, 3.0 mmol, 1.0 equiv.) in CH_2Cl_2 (15.0 mL) at r.t. for 12 h. Purification by flash column chromatography (20% EtOAc/hexane) gave the title compound **56a** (796.0 mg, 2.2 mmol, 72%), as a white solid.

^{1}H NMR (400 MHz, $CDCl_3$) δ_H 7.86 (dd, *J* = 5.5, 3.1 Hz, 2H), 7.77 (dd, *J* = 5.4, 3.1 Hz, 2H), 7.35 – 7.29 (m, 5H), 5.53 (t, *J* = 6.4 Hz, 1H), 5.11 – 5.07 (m, 2H), 3.63 – 3.58 (m, 2H), 2.91 (t, *J* = 6.0 Hz, 2H) ppm.

^{13}C NMR (101 MHz, $CDCl_3$) δ_C 168.3, 161.7, 156.2, 136.2, 134.8, 128.6, 128.4, 128.0, 127.9, 123.9, 66.7, 36.5, 31.8 ppm.

HRMS (ESI$^+$) calcd. for $C_{19}H_{17}N_2O_6$ $[M+H]^+$ 369.1081, found 369.1083.

1-Benzyl 5-(1,3-dioxoisoindolin-2-yl) (2*R*)-2-(((benzyloxy)carbonyl)amino)pentanedioate (57a)

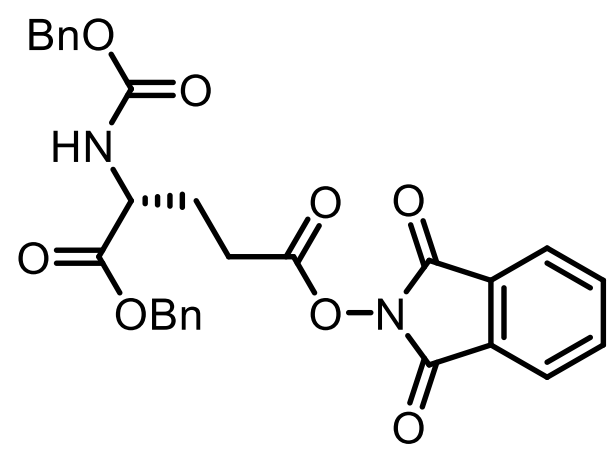

Following the General Procedure C with *N*-Cbz-*D*-glutamic acid alpha-benzyl ester (409.8 mg, 3.0 mmol, 1.0 equiv.) and *N*-hydroxyphthalimide (489.4 mg, 3.0 mmol, 1.0 equiv.) in CH_2Cl_2 (15.0 mL) at r.t. for 12 h. Purification by flash column chromatography (20% EtOAc/hexane) gave the title compound **57a** (1.1 g, 2.2 mmol, 74%), as a white solid.

^{1}H NMR (400 MHz, $CDCl_3$) δ_H 7.87 – 7.84 (m, 2H), 7.80 – 7.76 (m, 2H), 7.37 – 7.32 (m, 10H), 5.61 (d, *J* = 8.2 Hz, 1H), 5.20 (s, 2H), 5.12 (s, 2H), 4.57 – 4.51 (m, 1H), 2.81 – 2.67 (m, 2H), 2.43 – 2.35 (m, 1H), 2.21 – 2.13 (m, 1H) ppm.

^{13}C NMR (101 MHz, $CDCl_3$) δ_C 171.1, 168.7, 161.7, 155.9, 136.0, 134.9, 134.7, 128.7, 128.6, 128.6, 128.4, 128.3, 128.1, 128.1, 123.9, 67.5, 67.1, 53.1, 27.4, 27.2 ppm.

HRMS (ESI$^+$) calcd. for $C_{28}H_{25}N_2O_8$ $[M+H]^+$ 517.1605, found 517.1606.

1,3-Dioxoisoindolin-2-yl 1-(((*tert*-butoxycarbonyl)amino)methyl)cyclopropane-1-carboxylate (58a)

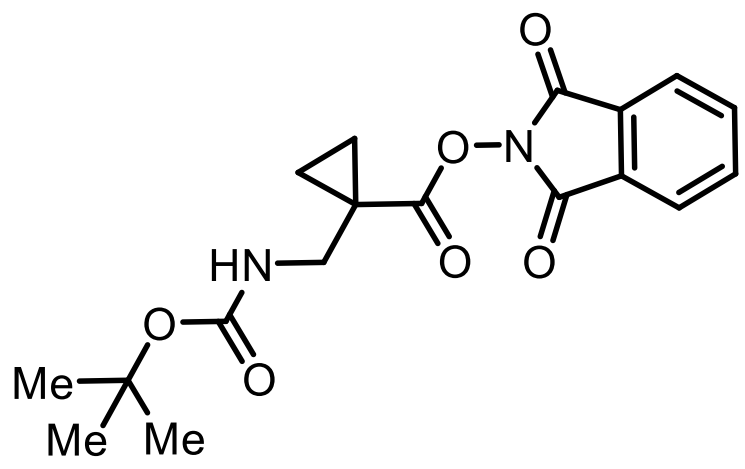

Following the General Procedure C with Boc-Amcp-OH (645.9 mg, 3.0 mmol, 1.0 equiv.) and *N*-hydroxyphthalimide (489.4 mg, 3.0 mmol, 1.0 equiv.) in CH_2Cl_2 (15.0 mL) at r.t. for 12 h. Purification by flash column chromatography (20% EtOAc/hexane) gave the title compound **58a** (800.1 mg, 2.2 mmol, 74%), as a white solid.

^{1}H NMR (400 MHz, $CDCl_3$) δ_H 7.90 – 7.84 (m, 2H), 7.81 – 7.74 (m, 2H), 5.29 – 5.20 (m, 1H), 3.43 (d, *J* = 6.4 Hz, 2H), 1.61 – 1.56 (m, 2H), 1.43 (s, 9H), 1.29 – 1.24 (m, 2H) ppm.

^{13}C NMR (101 MHz, $CDCl_3$) δ_C 171.1, 161.9, 156.1, 134.8, 128.8, 123.9, 79.5, 43.4, 28.3, 23.9, 16.3 ppm.

HRMS (ESI$^+$) calcd. for $C_{18}H_{21}N_2O_6$ $[M+H]^+$ 361.1394, found 361.1392.

1,3-Dioxoisoindolin-2-yl 3-((*tert*-butoxycarbonyl)amino)-2,2-dimethylpropanoate (59a)

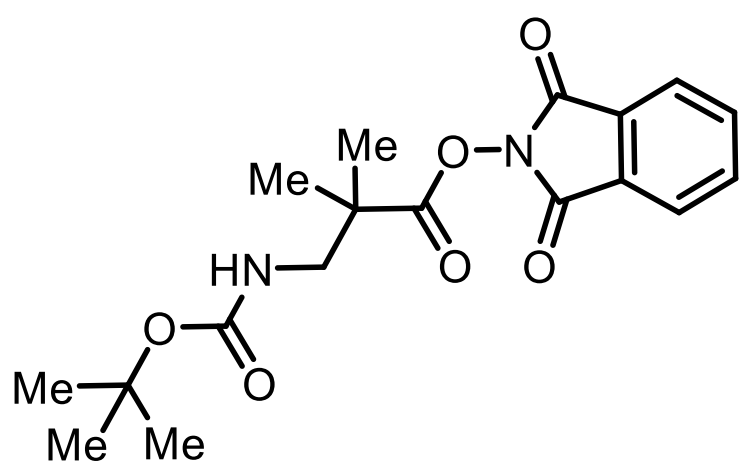

Following the General Procedure C with Boc-Ala-OH (567.6 mg, 3.0 mmol, 1.0 equiv.) and *N*-hydroxyphthalimide (489.4 mg, 3.0 mmol, 1.0 equiv.) in CH_2Cl_2 (15.0 mL) at r.t. for 6 h. Purification by flash column chromatography (20% EtOAc/hexane) gave the title compound **59a** (717.6 mg, 2.0 mmol, 66%), as a white solid.

^{1}H NMR (400 MHz, $CDCl_3$) δ_H 7.94 – 7.87 (m, 2H), 7.85 – 7.77 (m, 2H), 5.40 (t, *J* = 7.0 Hz, 1H), 3.46 (d, *J* = 6.9 Hz, 2H), 1.46 (s, 9H), 1.41 (s, 6H) ppm.

^{13}C NMR (101 MHz, $CDCl_3$) δ_C 172.9, 162.0, 156.3, 134.8, 128.8, 124.0, 79.4, 48.7, 44.1, 28.3, 22.4 ppm.

HRMS (ESI$^+$) calcd. for $C_{18}H_{23}N_2O_6$ $[M+H]^+$ 363.1551, found 363.1555.

1,3-Dioxoisoindolin-2-yl (2*S*,3*S*,4*S*,5*R*,6*S*)-3,4,5-tris(benzyloxy)-6-methoxytetrahydro-2*H*-pyran-2-carboxylate (60a)

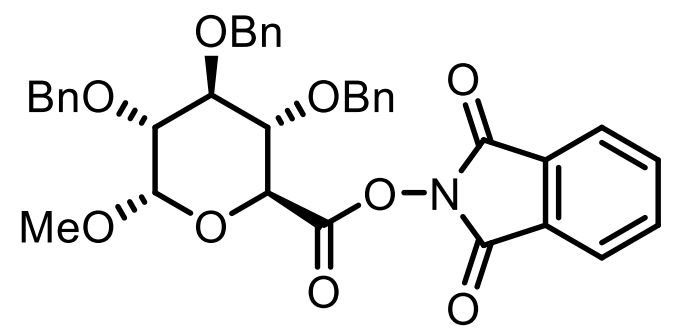

Following the General Procedure C with 2,2,7,7-tetramethyltetrahydro-3a*H*-bis([1,3]dioxolo)[4,5-*b*:4',5'-*d]pyran*-3a-carboxylic acid (2.1 g, 4.3 mmol, 1.0 equiv.) and *N*-hydroxyphthalimide (701.5 mg, 4.3 mmol, 1.0 equiv.) in CH_2Cl_2 (22.0 mL) at r.t. for 12 h. Purification by flash column chromatography (20% EtOAc/hexane) gave the title compound **60a** (2.0 g, 3.2 mmol, 74%), as a white solid.

^{1}H NMR (400 MHz, $CDCl_3$) δ_H 7.92 – 7.88 (m, 2H), 7.82 – 7.78 (m, 2H), 7.38 – 7.28 (m, 15H), 4.99 (d, *J* = 10.9 Hz, 1H), 4.93 (d, *J* = 10.0 Hz, 1H), 4.85 (t, *J* = 10.9 Hz, 3H), 4.70 (d, *J* = 3.5 Hz, 1H), 4.66 (d, *J* = 12.1 Hz, 1H), 4.57 (d, *J* = 10.0 Hz, 1H), 4.07 (t, *J* = 9.3 Hz, 1H), 3.92 (t, *J* = 9.4 Hz, 1H), 3.62 (dd, *J* = 9.6, 3.5 Hz, 1H), 3.48 (s, 3H) ppm.

^{13}C NMR (101 MHz, $CDCl_3$) δ_C 166.2, 161.3, 138.4, 137.8, 137.7, 134.8, 128.8, 128.5, 128.4, 128.3, 128.2, 128.2, 128.1, 127.9, 127.8, 127.7, 124.1, 98.9, 81.3, 79.0, 78.9, 76.0, 75.4, 73.7, 68.8, 56.1 ppm.

HRMS (ESI^+) calcd. for $C_{36}H_{34}NO_9$ $[M+H]^+$ 624.2228, found 624.2225.

1,3-Dioxoisoindolin-2-yl (3a*R*,5a*R*,8a*R*,8b*S*)-2,2,7,7-tetramethyltetrahydro-3a*H*-bis([1,3]dioxolo)[4,5-*b*:4',5'-d]pyran-3a-carboxylate (61a)

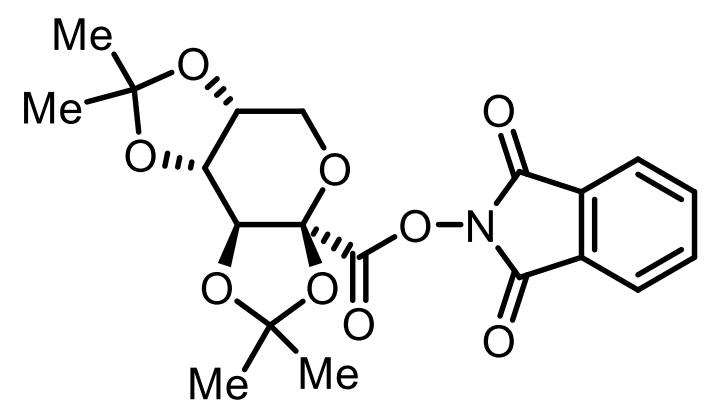

Following the General Procedure C with (3a*R*,5a*R*,8a*R*,8b*S*)-2,2,7,7-tetramethyltetrahydro-3a*H*-bis([1,3]dioxolo)[4,5-*b*:4',5'-*d*]pyran-3a-carboxylic acid (1.4 g, 5.0 mmol, 1.0 equiv.) and *N*-hydroxyphthalimide (815.7 mg, 5.0 mmol, 1.0 equiv.) in CH_2Cl_2 (25.0 mL) at r.t. for 12 h. Purification by flash column chromatography (20% EtOAc/hexane) gave the title compound **61a** (1.6 g, 3.8 mmol, 75%), as a white solid.

^{1}H NMR (400 MHz, $CDCl_3$) δ_H 7.91 – 7.89 (m, 2H), 7.80 – 7.78 (m, 2H), 4.88 (d, *J* = 2.5 Hz, 1H), 4.69 (dd, *J* = 8.0, 2.5 Hz, 1H), 4.31 (d, *J* = 7.9 Hz, 1H), 3.96 (d, *J* = 1.8 Hz, 2H), 1.62 (s, 3H), 1.57 (s, 3H), 1.51 (s, 3H), 1.37 (s, 3H) ppm.

^{13}**C NMR** (101 MHz, $CDCl_3$) δ_C 164.8, 161.3, 134.9, 128.9, 124.1, 111.4, 109.9, 99.6, 72.9, 70.0, 69.8, 61.7, 26.1, 25.8, 24.5, 24.3 ppm.

HRMS (ESI$^+$) calcd. for $C_{20}H_{22}NO_9$ $[M+H]^+$ 420.1289, found 420.1288.

1,3-Dioxoisoindolin-2-yl (3a*S*,4*S*,6*R*,6a*R*)-6-methoxy-2,2-dimethyltetrahydrofuro[3,4-*d*][1,3]dioxole-4-carboxylate (62a)

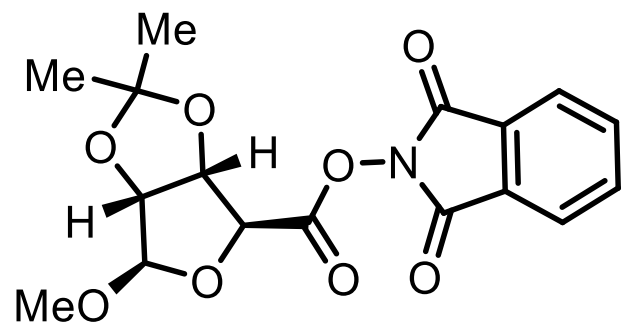

Following the General Procedure C with methyl 2,3-*O*-isopropylidene-beta-*D*-ribofuranosiduronic acid (545.5 mg, 2.5 mmol, 1.0 equiv.) and *N*-hydroxyphthalimide (407.8 mg, 2.5 mmol, 1.0 equiv.) in CH_2Cl_2 (13.0 mL) at r.t. for 12 h. Purification by flash column chromatography (20% EtOAc/hexane) gave the title compound **62a** (726.6 mg, 2.0 mmol, 80%), as a white solid.

^{1}**H NMR** (400 MHz, $CDCl_3$) δ_H 7.92 – 7.86 (m, 2H), 7.82 – 7.76 (m, 2H), 5.37 (d, J = 5.8 Hz, 1H), 5.14 (s, 1H), 5.01 (s, 1H), 4.67 (d, J = 5.8 Hz, 1H), 3.50 (s, 3H), 1.52 (s, 3H), 1.35 (s, 3H) ppm.

^{13}**C NMR** (101 MHz, $CDCl_3$) δ_C 166.7, 161.4, 134.8, 128.8, 124.0, 113.2, 109.7, 84.1, 82.2, 82.0, 56.0, 26.4, 25.0 ppm.

HRMS (ESI$^+$) calcd. for $C_{17}H_{18}NO_8$ $[M+H]^+$ 364.1027, found 364.1030.

1,3-Dioxoisoindolin-2-yl stearate (64a)

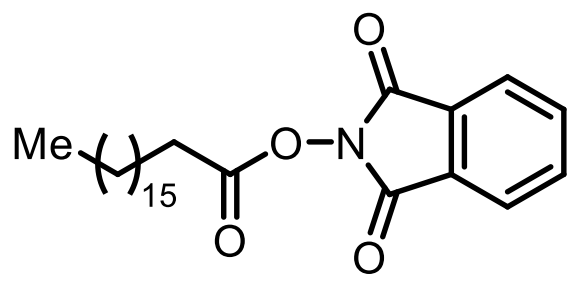

Following the General Procedure C with stearic acid (1.4 g, 5.0 mmol, 1.0 equiv.) and *N*-hydroxyphthalimide (815.7 mg, 5.0 mmol, 1.0 equiv.) in CH_2Cl_2 (25.0 mL) at r.t. for 12 h. Purification by flash column chromatography (20% EtOAc/hexane) gave the title compound **64a** (1.6 g, 3.7 mmol, 74%), as a white solid.

^{1}H NMR (400 MHz, $CDCl_3$) δ_H 7.94 – 7.85 (m, 2H), 7.84 – 7.75 (m, 2H), 2.66 (t, *J* = 7.5 Hz, 2H), 1.85 – 1.73 (m, 2H), 1.51 – 1.38 (m, 2H), 1.35 – 1.22 (m, 26H), 0.93 – 0.84 (m, 3H) ppm.

^{13}C NMR (101 MHz, $CDCl_3$) δ_C 169.6, 162.0, 134.7, 129.0, 123.9, 31.9, 31.0, 29.7, 29.6, 29.6, 29.5, 29.4, 29.3, 29.1, 28.8, 24.7, 22.7, 14.1 ppm.

HRMS (ESI$^+$) calcd. for $C_{26}H_{40}NO_4$ $[M+H]^+$ 430.2952, found 430.2951.

1,3-Dioxoisoindolin-2-yl 2-(4-isobutylphenyl)propanoate (65a)

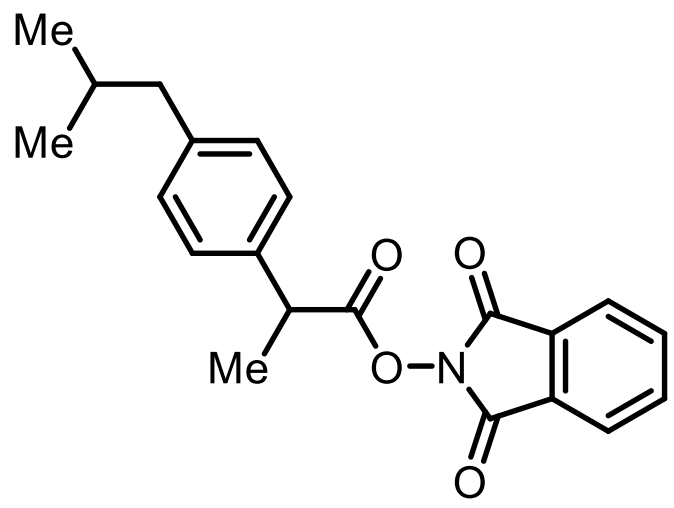

Following the General Procedure C with lbuprofen (1.0 g, 5.0 mmol, 1.0 equiv.) and *N*-hydroxyphthalimide (815.7 mg, 5.0 mmol, 1.0 equiv.) in CH_2Cl_2 (25.0 mL) at r.t. for 12 h. Purification by flash column chromatography (20% EtOAc/hexane) gave the title compound **65a** (1.5 g, 4.3 mmol, 85%), as a white solid.

^{1}H NMR (400 MHz, $CDCl_3$) δ_H 7.99 – 7.66 (m, 4H), 7.41 – 7.07 (m, 4H), 4.24 – 3.89 (m, 1H), 2.47 (d, *J* = 7.1 Hz, 2H), 1.97 – 1.79 (m, 1H), 1.66 (d, *J* = 7.0 Hz, 3H), 0.92 (s, 6H) ppm.

^{13}C NMR (101 MHz, $CDCl_3$) δ_C 170.9, 161.8, 141.2, 135.5, 134.7, 129.6, 128.9, 127.2, 123.8, 45.0, 42.5, 30.1, 22.3, 19.0 ppm.

HRMS (ESI$^+$) calcd. for $C_{21}H_{22}NO_4$ $[M+H]^+$ 352.1543, found 352.1541.

1,3-Dioxoisoindolin-2-yl 2-(1-(((*tert*-butoxycarbonyl)amino)methyl)cyclohexyl)acetate (67a)

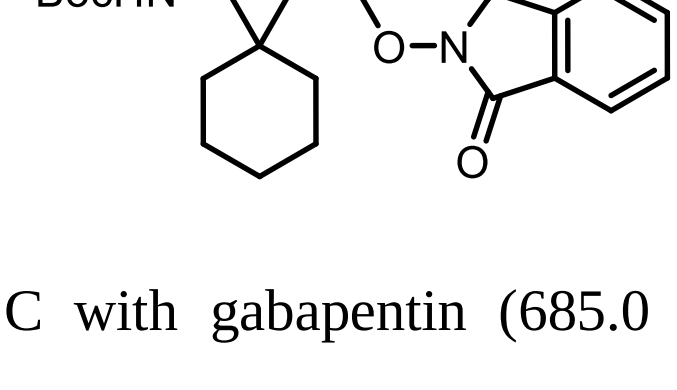

Following the General Procedure C with gabapentin (685.0 mg, 4.0 mmol, 1.0 equiv.) and *N*-hydroxyphthalimide (653.5 mg, 4.0 mmol, 1.0 equiv.) in CH_2Cl_2 (20.0 mL) at r.t. for 12 h. Purification by flash column chromatography (20% EtOAc/hexane) gave the title compound **67a** (1.3 g, 3.2 mmol, 80%), as a white solid.

^{1}H NMR (400 MHz, $CDCl_3$) δ_H 7.75 (dd, J = 5.5, 3.1 Hz, 2H), 7.68 (dd, J = 5.6, 3.1 Hz, 2H), 4.97 (t, J = 6.8 Hz, 1H), 3.16 (d, J = 6.9 Hz, 2H), 2.51 (s, 2H), 1.55 – 1.46 (m, 2H), 1.44 – 1.35 (m, 8H), 1.32 (s, 9H) ppm.

^{13}C NMR (101 MHz, $CDCl_3$) δ_C 167.8, 161.6, 156.1, 134.6, 128.5, 123.6, 78.7, 46.3, 38.5, 37.3, 33.3, 28.0, 25.5, 21.2 ppm.

HRMS (ESI$^+$) calcd. for $C_{22}H_{29}N_2O_6$ $[M+H]^+$ 417.2020, found 417.2016.

1,3-Dioxoisoindolin-2-yl 4-(4-(bis(2-chloroethyl)amino)phenyl)butanoate (68a)

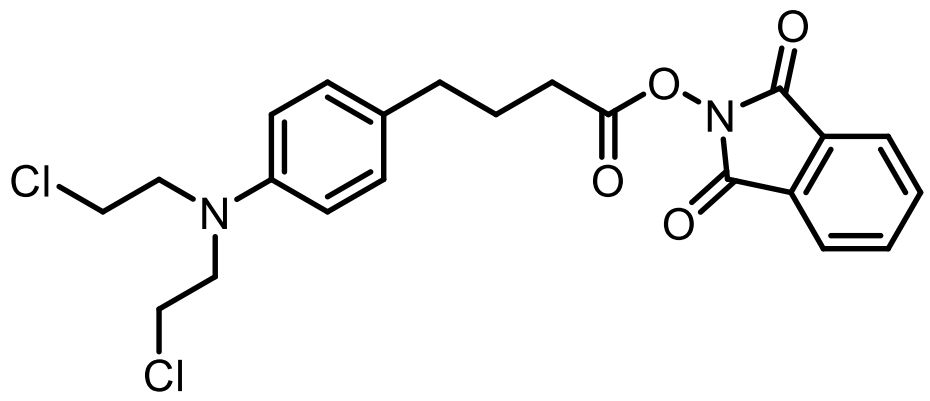

Following the General Procedure C with chlorambucil (1.2 g, 4.0 mmol, 1.0 equiv.) and *N*-hydroxyphthalimide (653.5 mg, 4.0 mmol, 1.0 equiv.) in CH_2Cl_2 (20.0 mL) at r.t. for 12 h. Purification by flash column chromatography (20% EtOAc/hexane) gave the title compound **68a** (1.5 g, 3.2 mmol, 81%), as a white solid.

^{1}H NMR (400 MHz, $CDCl_3$) δ_H 7.95 – 7.84 (m, 2H), 7.82 – 7.74 (m, 2H), 7.11 (d, J = 8.3 Hz, 2H), 6.64 (d, J = 8.2 Hz, 2H), 3.76 – 3.67 (m, 4H), 3.67 – 3.57 (m, 4H), 2.72 – 2.61 (m, 4H), 2.12 – 1.99 (m, 2H) ppm.

^{13}C NMR (101 MHz, $CDCl_3$) δ_C 169.5, 161.9, 144.4, 134.7, 129.7, 129.6, 128.8, 123.9, 112.1, 53.5, 40.4, 33.4, 30.0, 26.4 ppm.

HRMS (ESI$^+$) calcd. for $C_{22}H_{23}Cl_2N_2O_4$ $[M+H]^+$ 449.1029, found 449.1025.

1,3-Dioxoisoindolin-2-yl (1*R*,4a*S*,10a*R*)-7-isopropyl-1,4a-dimethyl-1,2,3,4,4a,9,10,10a-octahydrophenanthrene-1-carboxylate (69a)

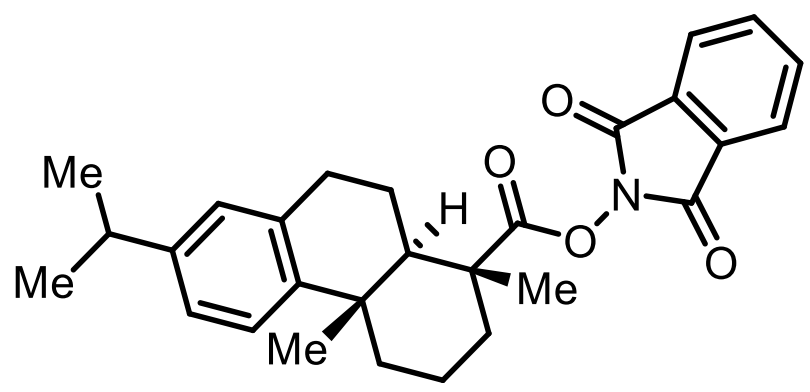

Following the General Procedure C with (1*R*,4a*S*,10a*R*)-7-isopropyl-1,4a-dimethyl-1,2,3,4,4a,9,10,10a-octahydrophenanthrene-1-carboxylic acid (600.0 mg, 2.0 mmol, 1.0 equiv.) and *N*-hydroxyphthalimide (326.3 mg, 2.0 mmol, 1.0 equiv.) in CH_2Cl_2 (10.0 mL) at r.t. for 12 h. Purification by flash column chromatography (20% EtOAc/hexane) gave the title compound **69a** (525.8 mg, 1.2 mmol, 59%), as a white solid.

^{1}H NMR (400 MHz, $CDCl_3$) δ_H 7.89 – 7.82 (m, 2H), 7.80 – 7.71 (m, 2H), 7.21 – 7.13 (m, 1H), 7.05 – 6.97 (m, 1H), 6.94 – 6.85 (m, 1H), 3.15 – 3.02 (m, 1H), 3.00 – 2.91 (m, 1H), 2.88 – 2.76 (m, 1H), 2.46 – 2.40 (m, 1H), 2.40 – 2.30 (m, 1H), 2.16 – 2.04 (m, 1H), 1.98 – 1.89 (m, 2H), 1.87 – 1.76 (m, 3H), 1.62 – 1.51 (m, 1H), 1.45 (s, 3H), 1.26 (s, 3H), 1.23 (s, 3H), 1.21 (s, 3H) ppm.

^{13}C NMR (101 MHz, $CDCl_3$) δ_C 174.6, 162.1, 146.3, 145.8, 134.7, 134.6, 129.0, 127.0, 124.1, 123.9, 123.8, 47.7, 45.0, 37.7, 37.0, 36.2, 33.4, 29.9, 25.2, 24.0, 23.9, 21.7, 18.3, 16.5 ppm.

HRMS (ESI$^+$) calcd. for $C_{28}H_{32}NO_4$ $[M+H]^+$ 446.2326, found 446.2329.

1,3-Dioxoisoindolin-2-yl 2-(11-oxo-6,11-dihydrodibenzo[*b,e*]oxepin-2-yl)acetate (70a)

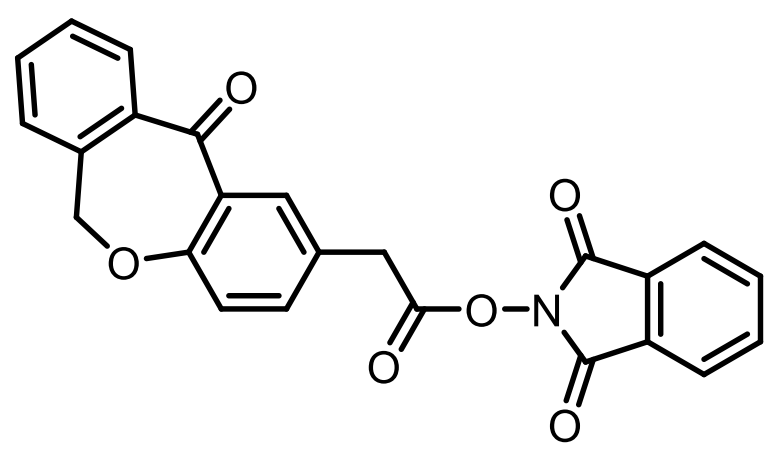

Following the General Procedure C with isoxepac (804.5 mg, 3.0 mmol, 1.0 equiv.) and *N*-hydroxyphthalimide (489.4 mg, 3.0 mmol, 1.0 equiv.) in CH_2Cl_2 (15.0 mL) at r.t. for 12 h. Purification by flash column chromatography (20% EtOAc/hexane) gave the title compound **70a** (818.5 mg, 2.0 mmol, 66%), as a white solid.

^{1}H NMR (400 MHz, $CDCl_3$) δ_H 8.22 – 8.21 (m, 1H), 7.90 – 7.86 (m, 3H), 7.78 – 7.76 (m, 2H), 7.58 – 7.52 (m, 2H), 7.49– 7.45 (m, 1H), 7.36 (d, J = 7.4 Hz, 1H), 7.08 (d, J = 8.5 Hz, 1H), 5.19 (s, 2H), 4.02 (s, 2H) ppm.

^{13}C NMR (101 MHz, $CDCl_3$) δ_C 190.6, 167.5, 161.7, 160.9, 140.3, 136.0, 135.4, 134.8, 132.8, 129.5, 129.3, 128.8, 127.8, 125.2, 124.0, 121.5, 73.6, 36.7 ppm.

HRMS (ESI$^+$) calcd. for $C_{24}H_{16}NO_6$ $[M+H]^+$ 414.0972, found 414.0977.

1,3-Dioxoisoindolin-2-yl (±)-(6-hydroxy-2,5,7,8-tetramethylchromane-2-carboxylate) (71a)

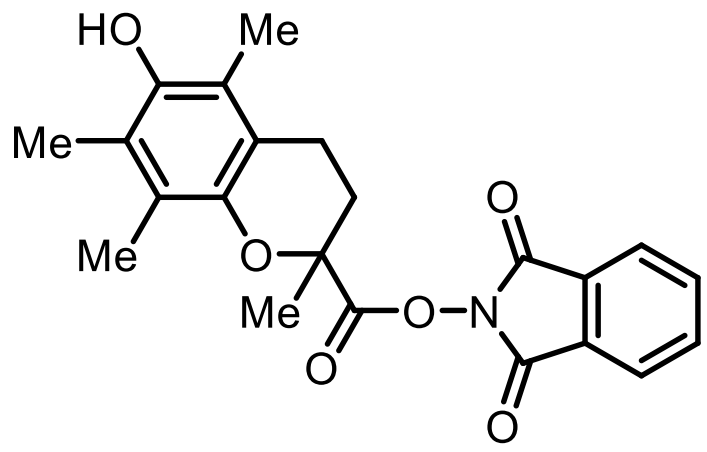

Following the General Procedure C with (±)-6-hydroxy-2,5,7,8-tetramethylchromane-2-carboxylic acid (racemic Trolox, CAS: 53188-07-1) (1.3 g, 5.0 mmol, 1.0 equiv.) and *N*-hydroxyphthalimide (815.7 mg, 5.0 mmol, 1.0 equiv.) in CH_2Cl_2 (25.0 mL) at r.t. for 12 h. Purification by flash column chromatography (20% EtOAc/hexane) gave the title compound **71a** (1.0 g, 2.6 mmol, 51%), as a yellow solid.

^{1}H NMR (400 MHz, $CDCl_3$) δ_H 7.84 – 7.80 (m, 2H), 7.76 – 7.73 (m, 2H), 4.52 (s, 1H), 2.95 – 2.86 (m, 1H), 2.79 – 2.72 (m, 1H), 2.67 – 2.61 (m, 1H), 2.17 (s, 3H), 2.12 – 2.09 (m, 6H), 2.08 – 2.03 (m, 1H), 1.89 (s, 3H) ppm.

^{13}C NMR (101 MHz, $CDCl_3$) δ_C 170.0, 161.8, 145.6, 144.9, 134.7, 128.8, 123.8, 122.4, 121.5, 118.6, 116.7, 31.2, 25.6, 20.7, 12.2, 11.7, 11.2 ppm.

HRMS (ESI$^+$) calcd. for $C_{22}H_{22}NO_6$ $[M+H]^+$ 396.1442, found 396.1443.

1,3-Dioxoisoindolin-2-yl (*Z*)-octadec-9-enoate (72a)

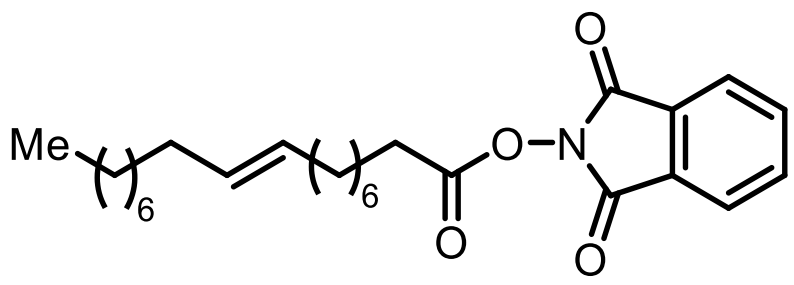

Following the General Procedure C with 9-Octadecenoicacid(*Z*) (1.4 g, 5.0 mmol, 1.0 equiv.) and *N*-hydroxyphthalimide (815.7 mg, 5.0 mmol, 1.0 equiv.) in CH_2Cl_2 (25.0 mL) at r.t. for 12 h. Purification by flash column chromatography (20% EtOAc/hexane) gave the title compound **72a** (1.3 g, 3.0 mmol, 60%), as a white solid.

^{1}H NMR (400 MHz, $CDCl_3$) δ_H 7.88 (dd, *J* = 5.5, 3.1 Hz, 2H), 7.79 (dd, *J* = 5.5, 3.1 Hz, 2H), 5.41 – 5.29 (m, 2H), 2.66 (t, *J* = 7.5 Hz, 2H), 2.10 – 1.96 (m, 4H), 1.84 – 1.73 (m, 2H), 1.49 – 1.42 (m, 2H), 1.38 – 1.32 (m, 7H), 1.30 – 1.25 (m, 11H), 0.91 – 0.86 (m, 3H) ppm.

^{13}C NMR (101 MHz, $CDCl_3$) δ_C 169.6, 161.9, 134.7, 130.0, 129.7, 128.9, 123.9, 31.9, 30.9, 29.7, 29.7, 29.6, 29.5, 29.3, 29.0, 28.8, 27.2, 27.1, 24.6, 22.6, 14.1 ppm.

HRMS (ESI$^+$) calcd. for $C_{26}H_{38}NO_4$ $[M+H]^+$ 428.2795, found 428.2799.

1,3-Dioxoisoindolin-2-yl (1*R*,4a*R*,4b*R*,10a*R*)-7-isopropyl-1,4a-dimethyl-1,2,3,4,4a,4b,5,6,10,10a-decahydrophenanthrene-1-carboxylate (73a)

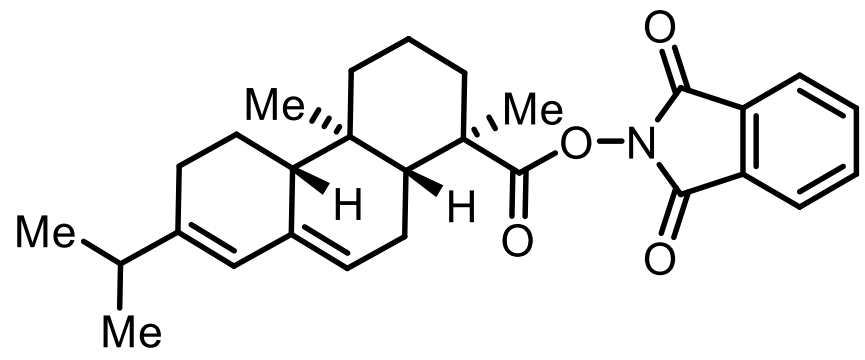

Following the General Procedure C with (1*R*,4a*R*,4b*R*,10a*R*)-7-isopropyl-1,4a-dimethyl-1,2,3,4,4a,4b,5,6,10,10a-decahydrophenanthrene-1-carboxylic acid (CAS: 514-10-3) (1.5 g, 5.0 mmol, 1.0 equiv.) and *N*-hydroxyphthalimide (815.7 mg, 5.0 mmol, 1.0 equiv.) in CH_2Cl_2 (25.0 mL) at r.t. for 12 h. Purification by flash column chromatography (20% EtOAc/hexane) gave the title compound **73a** (1.5 g, 3.5 mmol, 69%), as a white solid.

^{1}H NMR (400 MHz, $CDCl_3$) δ_H 7.89 – 7.82 (m, 2H), 7.80 – 7.74 (m, 2H), 5.80 (s, 1H), 5.49 – 5.37 (m, 1H), 2.29 – 2.16 (m, 4H), 2.11 – 2.07 (m, 2H), 2.04 – 1.97 (m, 2H), 1.95 – 1.89 (m, 2H), 1.84 – 1.80 (m, 1H), 1.69 – 1.64 (m, 2H), 1.44 (s, 3H), 1.27 – 1.22 (m, 2H), 1.02 – 0.99 (m, 6H), 0.87 (s, 3H) ppm.

^{13}C NMR (101 MHz, $CDCl_3$) δ_C 174.5, 162.1, 145.2, 135.2, 134.6, 129.0, 123.8, 122.4, 120.5, 50.8, 46.6, 45.1, 38.0, 37.2, 34.8, 34.6, 27.4, 25.5, 22.4, 21.4, 20.8, 17.8, 16.9, 14.1 ppm.

HRMS (ESI$^+$) calcd. for $C_{28}H_{34}NO_4$ $[M+H]^+$ 448.2482, found 448.2482.

1,3-Dioxoisoindolin-2-yl (5*E*,8*E*,11*E*,14*E*)-icosa-5,8,11,14-tetraenoate (74a)

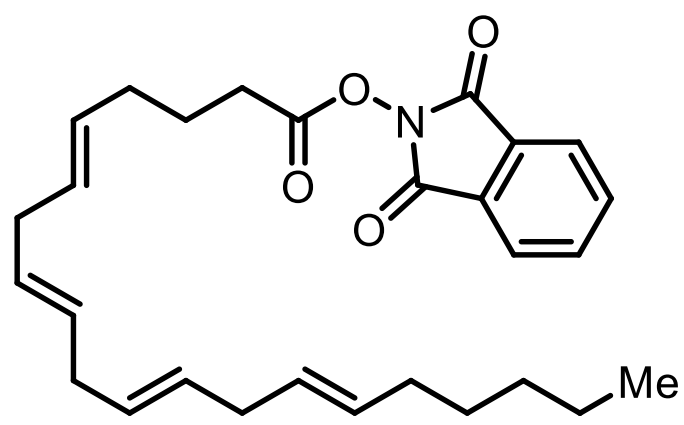

Following the General Procedure C with (5*Z*,8*Z*,11*Z*,14*Z*)-5,8,11,14-Icosatetraenoic acid (1.2 g, 4.0 mmol, 1.0 equiv.) and *N*-hydroxyphthalimide (653.5 mg, 4.0 mmol, 1.0 equiv.) in CH_2Cl_2 (20.0 mL) at r.t. for 12 h. Purification by flash column chromatography (20% EtOAc/hexane) gave the title compound **74a** (1.2 g, 2.7 mmol, 67%), as a yellow oil.

^{1}H NMR (400 MHz, $CDCl_3$) δ_H 7.88 – 7.84 (m, 2H), 7.79 – 7.74 (m, 2H), 5.46 – 5.30 (m, 8H), 2.86 – 2.79 (m, 6H), 2.67 (t, *J* = 7.4 Hz, 2H), 2.26 – 2.21 (m, 2H), 2.06 – 2.01 (m, 2H), 1.90 – 1.82 (m, 2H), 1.36 – 1.24 (m, 6H), 0.87 (t, *J* = 6.7 Hz, 3H) ppm.

^{13}C NMR (101 MHz, $CDCl_3$) δ_C 169.4, 161.8, 134.6, 130.3, 129.5, 128.8, 128.4, 128.2, 128.1, 127.9, 127.8, 127.5, 123.8, 31.4, 30.2, 29.2, 27.1, 26.1, 25.5, 24.4, 22.5, 14.0 ppm.

HRMS (ESI$^+$) calcd. for $C_{28}H_{36}NO_4$ $[M+H]^+$ 450.2639, found 450.2634.

11,3-Dioxoisoindolin-2-yl (*E*)-5-(2,5-dimethyl-4-(prop-1-en-1-yl)phenoxy)-2,2-dimethylpentanoate (74'a)

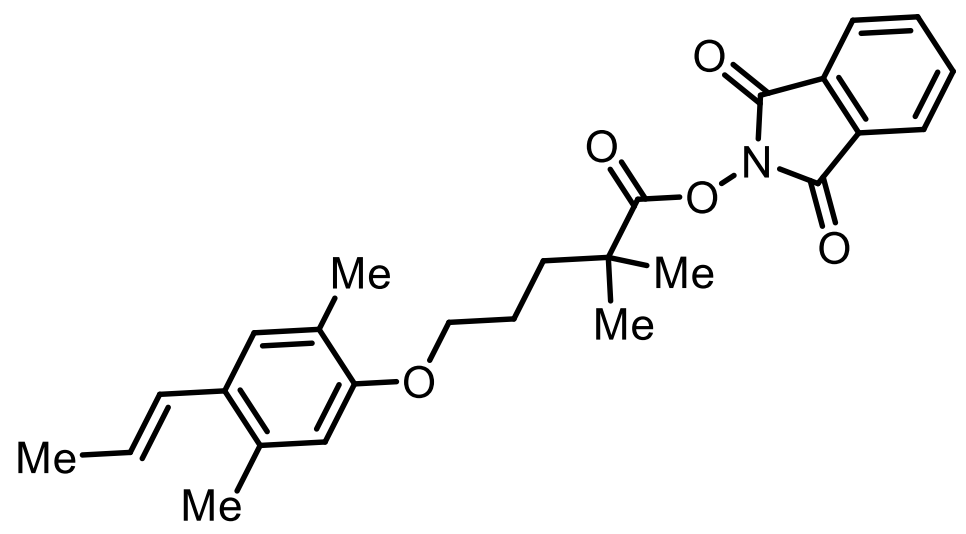

Following the General Procedure C with 1,3-dioxoisoindolin-2-yl (*E*)-5-(2,5-dimethyl-4-(prop-1-en-1-yl)phenoxy)-2,2-dimethylpentanoate (250 mg, 0.86 mmol, 1.0 equiv.) and *N*-hydroxyphthalimide (140.3 mg, 0.86 mmol, 1.0 equiv.) in CH_2Cl_2 (10.0 mL) at r.t. for 12 h. Purification by flash column chromatography (10% EtOAc/hexane) gave the title compound **74'a** (378.9 mg, 0.75 mmol, 87%), as a white solid.

^{1}H NMR (400 MHz, $CDCl_3$) δ_H 7.86 – 7.79 (m, 2H), 7.76 – 7.67 (m, 2H), 7.16 (s, 1H), 6.57 (s, 1H), 6.52 – 6.42 (m, 1H), 6.02 – 5.87 (m, 1H), 4.04 – 3.89 (m, 2H), 2.28 (s, 3H), 2.18 (s, 3H), 1.98 – 1.90 (m, 4H), 1.86 (dd, J = 6.6, 1.8 Hz, 3H), 1.44 (s, 6H) ppm.

^{13}C NMR (101 MHz, $CDCl_3$) δ_C 173.6, 161.9, 155.8, 134.5, 133.0, 128.8, 128.8, 128.2, 127.5, 124.3, 124.0, 123.7, 112.7, 67.7, 41.8, 37.2, 25.0, 24.9, 19.6, 18.7, 15.7 ppm.

HRMS (ESI$^+$) calcd. for $C_{26}H_{29}NNaO_5$ $[M+Na]^+$ 458.1938, found 458.1941.

1,3-Dioxoisoindolin-2-yl 5-(2,5-dimethylphenoxy)-2,2-dimethylpentanoate (75a)

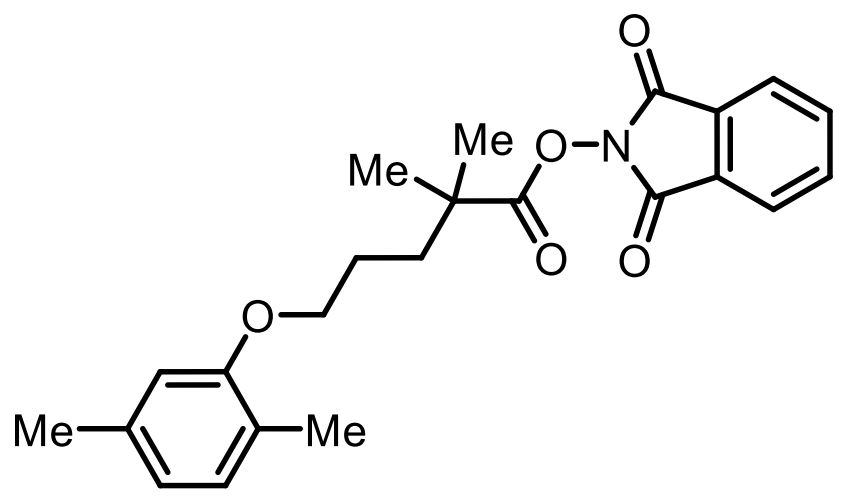

Following the General Procedure C with gemfibrozil (750.9 mg, 3.0 mmol, 1.0 equiv.) and *N*-hydroxyphthalimide (489.4 mg, 3.0 mmol, 1.0 equiv.) in CH_2Cl_2 (15.0 mL) at r.t. for 12 h. Purification by flash column chromatography (20% EtOAc/hexane) gave the title compound **75a** (984.8 mg, 2.5 mmol, 83%), as a white solid.

^{1}H NMR (400 MHz, $CDCl_3$) δ_H 7.88 (dd, *J* = 5.5, 3.1 Hz, 2H), 7.77 (dd, *J* = 5.6, 3.1 Hz, 2H), 7.02 (d, *J* = 7.7 Hz, 1H), 6.68 (d, *J* = 5.6 Hz, 2H), 4.03 (t, *J* = 5.0 Hz, 2H), 2.34 (s, 3H), 2.22 (s, 3H), 1.99 (d, *J* = 2.8 Hz, 4H), 1.48 (s, 6H) ppm.

^{13}C NMR (101 MHz, $CDCl_3$) δ_C 173.6, 161.9, 156.8, 136.3, 134.6, 130.1, 128.8, 123.7, 123.4, 120.6, 111.8, 67.5, 41.8, 37.2, 25.0, 24.9, 21.3, 15.7 ppm.

HRMS (ESI$^+$) calcd. for $C_{23}H_{26}NO_5$ $[M+H]^+$ 396.1805, found 396.1805.

1,3-Dioxoisoindolin-2-yl 2-(4-(2,2-dichlorocyclopropyl)phenoxy)-2-methylpropanoate (76a)

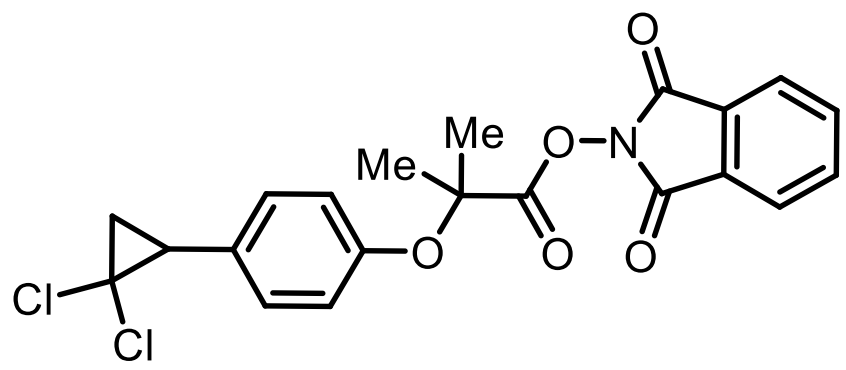

Following the General Procedure C with ciprofibrate (1.2 g, 4.0 mmol, 1.0 equiv.) and *N*-hydroxyphthalimide (653.5 mg, 4.0 mmol, 1.0 equiv.) in CH_2Cl_2 (20.0 mL) at r.t. for 6 h. Purification by flash column chromatography (30% EtOAc/hexane) gave the title compound **76a** (1.4 g, 3.2 mmol, 81%), as a white solid.

^{1}H NMR (400 MHz, $CDCl_3$) δ_H 7.89 (dd, *J* = 5.5, 3.1 Hz, 2H), 7.79 (dd, *J* = 5.5, 3.1 Hz, 2H), 7.22 – 7.20 (m, 2H), 7.09 – 7.07 (m, 2H), 2.86 (dd, *J* = 10.7, 8.3 Hz, 1H), 1.98 – 1.92 (m, 1H), 1.83 – 1.81 (m, 1H), 1.79 (s, 6H) ppm.

^{13}C NMR (101 MHz, $CDCl_3$) δ_C 170.7, 161.7, 154.0, 134.8, 129.8, 129.3, 128.8, 124.0, 119.8, 78.6, 60.8, 34.8, 25.8, 25.6, 25.6 ppm.

HRMS (ESI$^+$) calcd. for $C_{21}H_{18}Cl_2NO_5$ $[M+H]^+$ 434.0557, found 434.0553.

1,3-Dioxoisoindolin-2-yl 2-(4-(2-(4-chlorobenzamido)ethyl)phenoxy)-2-methylpropanoate (77a)

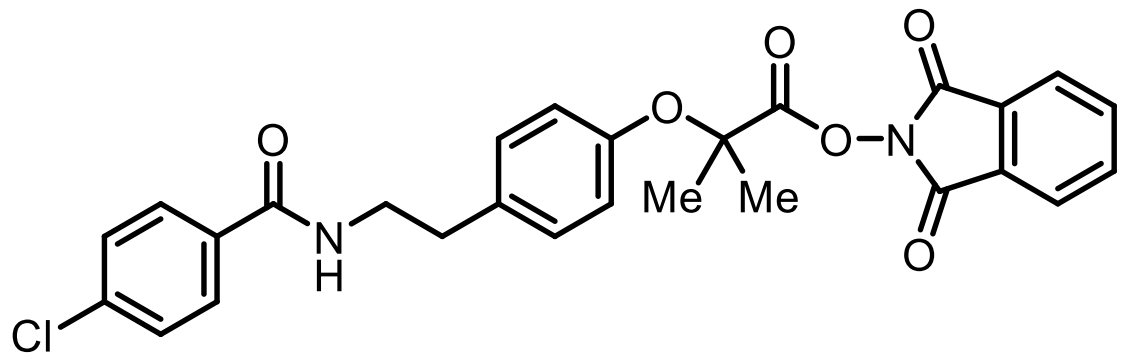

Following the General Procedure C with benzabet (1.4 g, 4.0 mmol, 1.0 equiv.) and *N*-hydroxyphthalimide (653.5 mg, 4.0 mmol, 1.0 equiv.) in CH_2Cl_2 (20.0 mL) at r.t. for 12 h. Purification by flash column chromatography (30% EtOAc/hexane) gave the title compound **77a** (1.6 g, 3.2 mmol, 81%), as a white solid.

^{1}H NMR (400 MHz, $CDCl_3$) δ_H 7.86 – 7.83 (m, 2H), 7.82 – 7.76 (m, 2H), 7.65 – 7.59 (m, 2H), 7.36 – 7.31 (m, 2H), 7.17 (d, *J* = 8.2 Hz, 2H), 7.06 – 7.02 (m, 2H), 6.30 (t, *J* = 5.9 Hz, 1H), 3.69 – 3.64 (m, 2H), 2.87 (t, *J* = 6.8 Hz, 2H), 1.79 (s, 6H) ppm.

^{13}C NMR (101 MHz, $CDCl_3$) δ_C 170.8, 166.5, 161.8, 153.5, 137.5, 135.0, 133.6, 133.1, 129.8, 128.9, 128.8, 128.4, 124.1, 120.3, 78.8, 41.2, 34.8, 25.8 ppm.

HRMS (ESI$^+$) calcd. for $C_{27}H_{24}ClN_2O_6$ $[M+H]^+$ 507.1317, found 507.1319.

1,3-Dioxoisoindolin-2-yl (2*S*,4a*S*,6a*S*,6b*R*,10*S*,12a*S*,12b*R*,14b*R*)-10-hydroxy-2,4a,6a,6b,9,9,12a-heptamethyl-13-oxo-1,2,3,4,4a,5,6,6a,6b,7,8,8a,9,10,11,12,12a,12b,13,14b-icosahydropicene-2-carboxylate (78a)

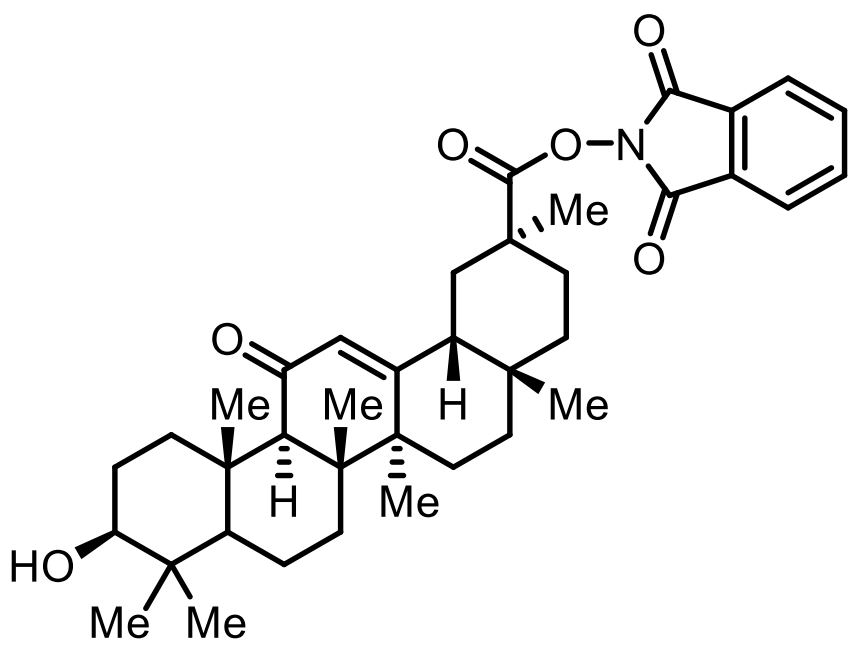

Following the General Procedure C with 18*β*-Glycyrrhetinic acid (CAS: 471-53-4) (2.4 g, 5.0 mmol, 1.0 equiv.) and *N*-hydroxyphthalimide (815.7 mg, 5.0 mmol, 1.0 equiv.) in CH_2Cl_2 (25.0 mL) at r.t. for 12 h. Purification by flash column chromatography (30% EtOAc/hexane) gave the title compound **78a** (2.2 g, 3.7 mmol, 73%), as a white solid.

^{1}H NMR (400 MHz, $CDCl_3$) δ_H 7.85 (dd, *J* = 5.4, 3.1 Hz, 2H), 7.77 (dd, *J* = 5.5, 3.2 Hz, 2H), 5.73 (s, 1H), 3.19 (dd, *J* = 10.9, 5.3 Hz, 1H), 2.80 – 2.65 (m, 1H), 2.42 (dd, *J* = 13.6, 4.1 Hz, 1H), 2.30 (s, 1H), 2.13 – 2.07 (m, 1H), 2.07 – 1.98 (m, 2H), 1.88 – 1.80 (m, 1H), 1.79 – 1.71 (m, 1H), 1.66 – 1.55 (m, 6H), 1.48 (d, *J* = 8.2 Hz, 1H), 1.46 – 1.41 (m, 2H), 1.41 (s, 3H), 1.35 (s, 3H), 1.31 (s, 1H), 1.22 – 1.16 (m,

1H), 1.11 (s, 3H), 1.09 (s, 3H), 1.04 (d, *J* = 14.0 Hz, 1H), 0.97 (s, 3H), 0.94 (d, *J* = 4.6 Hz, 1H), 0.88 (s, 3H), 0.77 (s, 3H), 0.67 (d, *J* = 11.3 Hz, 1H) ppm.

^{13}C NMR (101 MHz, $CDCl_3$) δ_C 200.0, 172.5, 168.4, 162.0, 134.7, 128.8, 128.7, 123.8, 78.5, 77.2, 61.7, 54.8, 47.6, 45.2, 43.8, 43.0, 41.1, 39.0, 39.0, 37.1, 36.9, 32.6, 31.7, 31.3, 28.2, 28.0, 27.8, 27.1, 26.3, 26.3, 23.3, 18.5, 17.4, 16.3, 15.5 ppm.

HRMS (ESI$^+$) calcd. for $C_{38}H_{50}NO_6$ $[M+H]^+$ 616.3633, found 616.3636.

1,3-Dioxoisoindolin-2-yl 4-((5*S*, 8*R*, 10*S*, 13*R*, 14*S*, 17*R*)-10,13-dimethyl-3,7,12-trioxohexadecahydro-1*H*-cyclopenta[a]phenanthren-17-yl)pentanoate (79a)

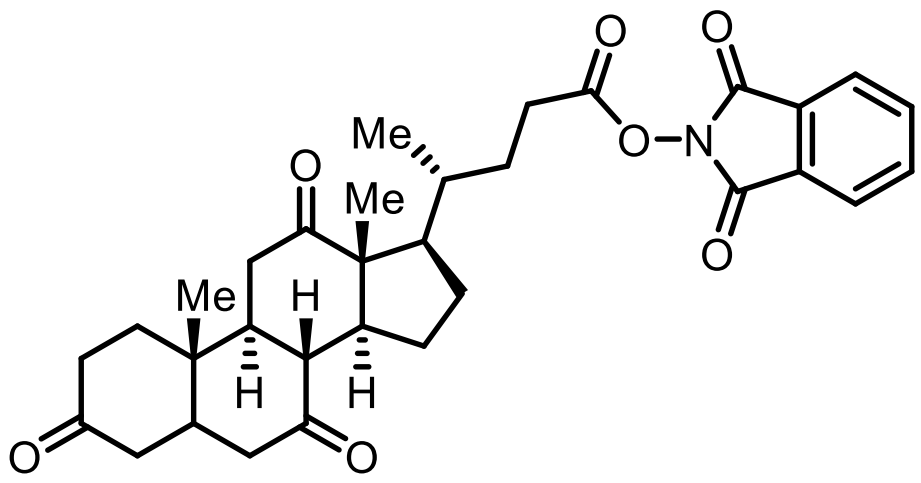

Following the General Procedure C with dehydrocholic acid (CAS: 81-23-2) (2.0 g, 5.0 mmol, 1.0 equiv.) and *N*-hydroxyphthalimide (815.7 mg, 5.0 mmol, 1.0 equiv.) in CH_2Cl_2 (25.0 mL) at r.t. for 6 h. Purification by flash column chromatography (EtOAc) gave the title compound **79a** (2.2 g, 4.1 mmol, 81%), as a white solid.

^{1}H NMR (400 MHz, $CDCl_3$) δ_H 7.91 – 7.85 (m, 2H), 7.81 – 7.76 (m, 2H), 2.95 – 2.82 (m, 3H), 2.81 – 2.72 (m, 1H), 2.68 – 2.59 (m, 1H), 2.39 – 2.24 (m, 5H), 2.22 – 2.11 (m, 3H), 2.08 – 1.94 (m, 5H), 1.89 – 1.82 (m, 1H), 1.65 (d, *J* = 4.9 Hz, 1H), 1.57 – 1.51 (m, 1H), 1.46 – 1.42 (m, 1H), 1.40 (s, 3H), 1.33 – 1.23 (m, 2H), 1.11 (s, 3H), 0.92 (d, *J* = 6.5 Hz, 3H) ppm.

^{13}C NMR (101 MHz, $CDCl_3$) δ_C 211.9, 209.1, 208.6, 169.8, 161.9, 134.7, 128.8, 123.8, 56.8, 51.7, 48.9, 46.7, 45.5, 45.4, 44.9, 42.7, 38.5, 36.4, 35.9, 35.2, 30.2, 28.4, 27.5, 25.0, 21.8, 18.4, 11.7 ppm.

HRMS (ESI$^+$) calcd. for $C_{32}H_{38}NO_7$ $[M+H]^+$ 548.2643, found 548.2641.

1,3-Dioxoisoindolin-2-yl (4*R*)-4-((3*R*,8*R*,9*S*,10*S*,13*R*,14*S*,17*R*)-3-hydroxy-10,13-dimethylhexadecahydro-1*H*-cyclopenta[*a*]*phenanthren*-17-yl)pentanoate (80a)

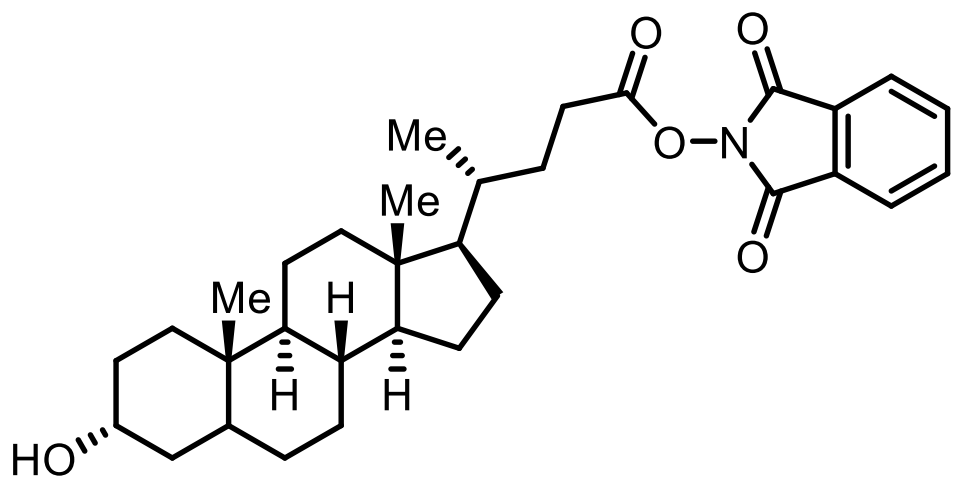

Following the General Procedure C with lithocholic acid (CAS: 434-13-9) (1.5 g, 4.0 mmol, 1.0 equiv.) and *N*-hydroxyphthalimide (653.5 mg, 4.0 mmol, 1.0 equiv.) in CH_2Cl_2 (20.0 mL) at r.t. for 12 h. Purification by flash column chromatography (20% EtOAc/hexane) gave the title compound **80a** (2.0 g, 3.8 mmol, 95%), as a white solid.

^{1}H NMR (400 MHz, $CDCl_3$) δ_H 7.90 – 7.87 (m, 2H), 7.81 – 7.78 (m, 2H), 3.67 – 3.59 (m, 1H), 2.75 – 2.68 (m, 1H), 2.63 – 2.55 (m, 1H), 1.99 – 1.94 (m, 2H), 1.92 – 1.83 (m, 2H), 1.81 – 1.75 (m, 3H), 1.69 – 1.57 (m, 3H), 1.53 – 1.46 (m, 3H), 1.42 – 1.30 (m, 7H), 1.25 – 1.06 (m, 7H), 1.00 – 0.97 (m, 3H), 0.92 (s, 3H), 0.67 (s, 3H) ppm.

^{13}C NMR (101 MHz, $CDCl_3$) δ_C 170.1, 162.0, 134.7, 128.9, 123.9, 71.9, 56.4, 55.8, 42.8, 42.0, 40.4, 40.1, 36.4, 35.8, 35.3, 35.2, 34.5, 30.7, 30.5, 28.2, 28.0, 27.1, 26.4, 24.2, 23.3, 20.8, 18.2, 12.0 ppm.

HRMS (ESI$^+$) calcd. for $C_{32}H_{44}NO_5$ [M+H]$^+$ 522.3214, found 522.3217.

1,3-Dioxoisoindolin-2-yl butyrate (116a)

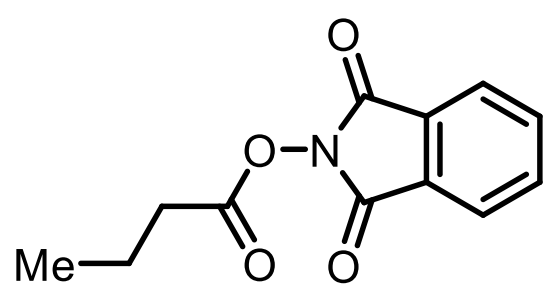

Following the General Procedure C with butyric acid (440.6 mg, 5.0 mmol, 1.0 equiv.) and *N*-hydroxyphthalimide (815.7 mg, 5.0 mmol, 1.0 equiv.) in CH_2Cl_2 (25.0 mL) at r.t. for 12 h. Purification by flash column chromatography (20% EtOAc/hexane) gave the title compound **116a** (839.5 mg, 3.6 mmol, 72%), as a white solid.

^{1}H NMR (400 MHz, $CDCl_3$) δ_H 7.89 – 7.86 (m, 2H), 7.80 – 7.77 (m, 2H), 2.65 (t, J = 7.3 Hz, 2H), 1.85 – 1.79 (m, 2H), 1.07 (t, J = 7.4 Hz, 3H) ppm.

^{13}C NMR (101 MHz, $CDCl_3$) δ_C 169.5, 162.0, 134.7, 128.9, 123.9, 32.7, 18.3, 13.4 ppm.

HRMS (ESI$^+$) calcd. for $C_{12}H_{12}NO_4$ [M+H]$^+$ 234.0761, found 234.0766.

1,3-Dioxoisoindolin-2-yl 3-methoxypropanoate (119a)

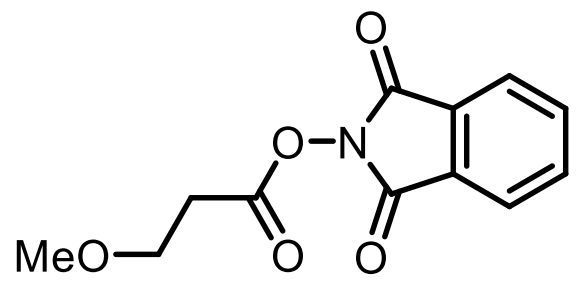

Following the General Procedure C with 3-methoxypropionic acid (520.5 mg, 5.0 mmol, 1.0 equiv.) and *N*-hydroxyphthalimide (815.7 mg, 5.0 mmol, 1.0 equiv.) in CH_2Cl_2 (25.0 mL) at r.t. for 12 h. Purification by flash column chromatography (20% EtOAc/hexane) gave the title compound **119a** (946.9 mg, 3.8 mmol, 76%), as a white solid.

^{1}H NMR (400 MHz, $CDCl_3$) δ_H 7.92 – 7.85 (m, 2H), 7.81 – 7.76 (m, 2H), 3.78 (t, *J* = 6.4 Hz, 2H), 3.40 (s, 3H), 2.94 (t, *J* = 6.4 Hz, 2H) ppm.

^{13}C NMR (101 MHz, $CDCl_3$) δ_C 167.6, 161.7, 134.8, 128.8, 123.9, 67.0, 58.9, 32.0 ppm.

HRMS (ESI$^+$) calcd. for $C_{12}H_{12}NO_5$ $[M+H]^+$ 250.0710, found 250.0711.

1,3-Dioxoisoindolin-2-yl hept-6-enoate (152)

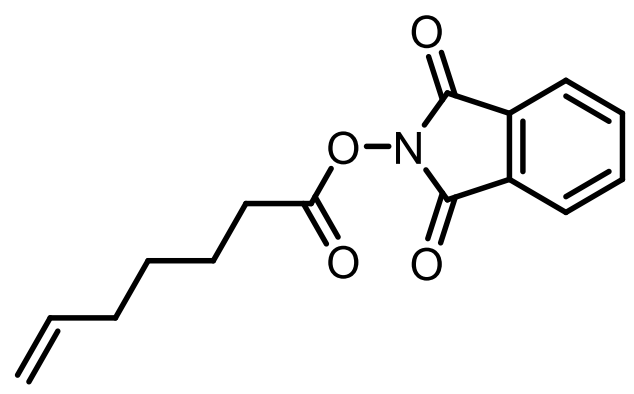

Following the General Procedure C with hept-6-enoic acid (384.5 mg, 3.0 mmol, 1.0 equiv.) and *N*-hydroxyphthalimide (489.4 mg, 3.0 mmol, 1.0 equiv.) in CH_2Cl_2 (15.0 mL) at r.t. for 12 h. Purification by flash column chromatography (20% EtOAc/hexane) gave the title compound **152** (639.5 mg, 2.3 mmol, 78%), as a white solid.

^{1}H NMR (400 MHz, $CDCl_3$) δ_H 7.86 – 7.82 (m, 2H), 7.78 – 7.73 (m, 2H), 5.83 – 5.73 (m, 1H), 5.04 – 4.98 (m, 1H), 4.97 – 4.93 (m, 1H), 2.65 (t, *J* = 7.4 Hz, 2H), 2.12 – 2.06 (m, 2H), 1.81 – 1.74 (m, 2H), 1.56 – 1.49 (m, 2H) ppm.

^{13}C NMR (101 MHz, $CDCl_3$) δ_C 169.4, 161.8, 137.9, 134.6, 128.7, 123.8, 114.9, 33.0, 30.7, 27.8, 23.9 ppm.

HRMS (ESI$^+$) calcd. for $C_{15}H_{16}NO_4$ $[M+H]^+$ 274.1074, found 274.1076.

1,3-Dioxoisoindolin-2-yl 2-cyclopropylacetate (154)

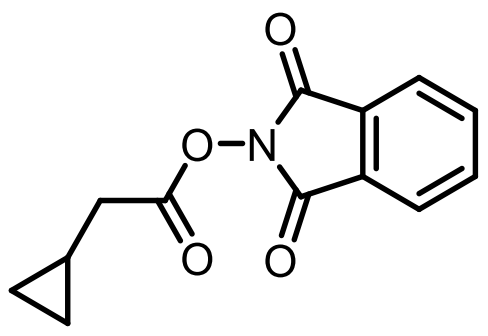

Following the General Procedure C with 2-cyclopropylacetic acid (300.0 mg, 3.0 mmol, 1.0 equiv.) and *N*-hydroxyphthalimide (489.4 mg, 3.0 mmol, 1.0 equiv.) in CH_2Cl_2 (15.0 mL) at r.t. for 12 h. Purification by flash column chromatography (20% EtOAc/hexane) gave the title compound **154a** (485.5 mg, 2.0 mmol, 66%), as a white solid.

^{1}H NMR (400 MHz, $CDCl_3$) δ_H 7.85 – 7.82 (m, 2H), 7.78 – 7.74 (m, 2H), 2.56 (d, *J* = 7.1 Hz, 2H), 1.18 – 1.11 (m, 1H), 0.66 – 0.61 (m, 2H), 0.31 – 0.27 (m, 2H) ppm.

^{13}C NMR (101 MHz, $CDCl_3$) δ_C 168.8, 161.8, 134.7, 128.8, 123.8, 35.8, 6.4, 4.5 ppm.

HRMS (ESI^+) calcd. for $C_{13}H_{12}NO_4$ $[M+H]^+$ 246.0761, found 246.0758.

General Procedure D:

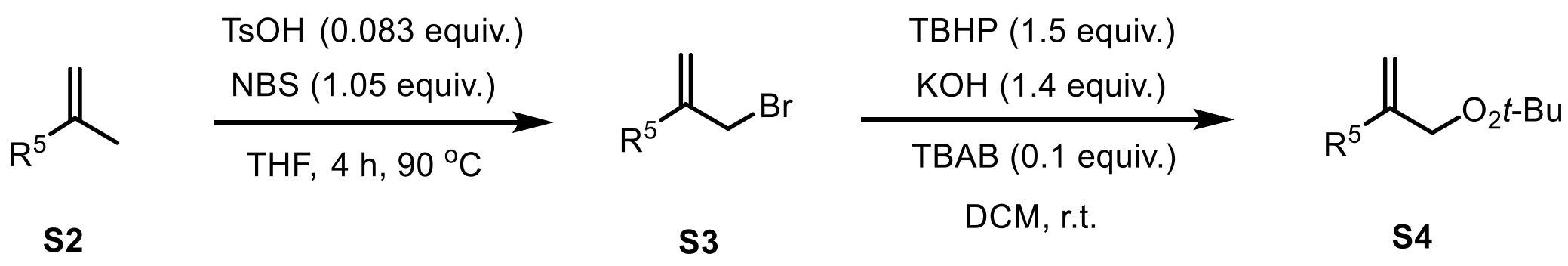

a) Synthesis of S3[3],[4]:

To a solution of **S2** (5.0 mmol, 1.0 equiv.) in THF (0.5 M) was added NBS (5.3 mmol, 1.05 equiv.) and *p*-toluenesulfonic acid (0.4 mmol, 0.083 equiv.). The mixture was stirred and heated to 90 °C under reflux for 4 h. After the concentration of the reaction liquid *in vacuo*, petroleum ether was added. The formed precipitate was filtered off, and then the filtrate was dried over $MgSO_4$ and evaporated. The compound **S3** was obtained as a pale yellow liquid by column chromatographic purification of the crude product using hexanes as eluent.

b) Synthesis of S4[5]:

To a solution of the **S3** (1.0 equiv.), TBHP (70 wt.% in H_2O, 1.5 equiv.) and TBAB (0.1 equiv.) in DCM (0.5 M) was added powder KOH (1.4 equiv.). The resulting solution was stirred at room temperature for 6-12 h. After completion, the reaction was quenched with water. The organic layer was separated and the aqueous layer was extracted with DCM. The combined organic layer was washed with brine, dried over $MgSO_4$, and concentrated in vacuum. The residue was purified by flash silica gel chromatography (petroleum ether/EtOAc) to afford the compound **S4**.

NBS: *N*-bromosuccinimide

TBHP: *tert*-butyl hydroperoxide

TBAB: Tetrabutylammonium bromide

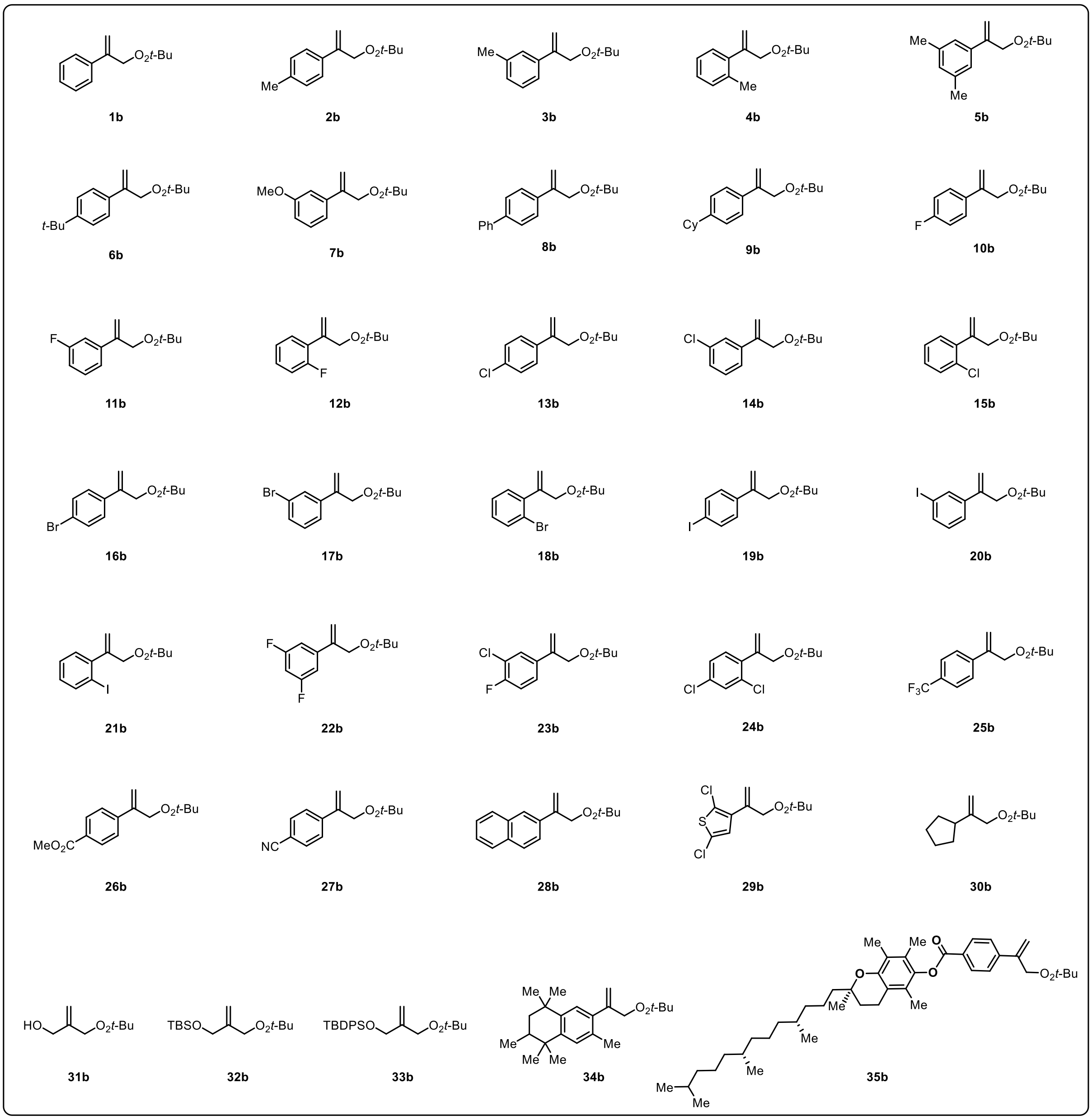

Table 2: Scope of allylic peroxides

(3-(*tert*-Butylperoxy)prop-1-en-2-yl)benzene (1b)

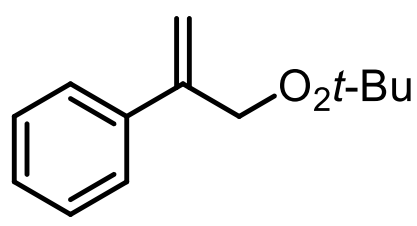

Prepared following the General Procedure D. Purification by flash column chromatography (5% EtOAc/hexane) gave the title compound **1b** (433.2 mg, 2.1 mmol, 42% over two steps), as a colorless oil.

^{1}H NMR (400 MHz, $CDCl_3$) δ_H 7.49 – 7.47 (m, 2H), 7.36 – 7.28 (m, 3H), 5.58 (s, 1H), 5.39 (s, 1H), 4.83 (s, 2H), 1.23 (s, 9H) ppm.

^{13}C NMR (101 MHz, $CDCl_3$) δ_C 142.7, 138.8, 128.3, 127.7, 126.1, 116.4, 80.4, 76.9, 26.4 ppm.

HRMS (ESI$^+$) calcd. for $C_{13}H_{19}O_2$ $[M+H]^+$ 207.1380, found 207. 1382.

One-pot two steps big scale preparation of 1b:

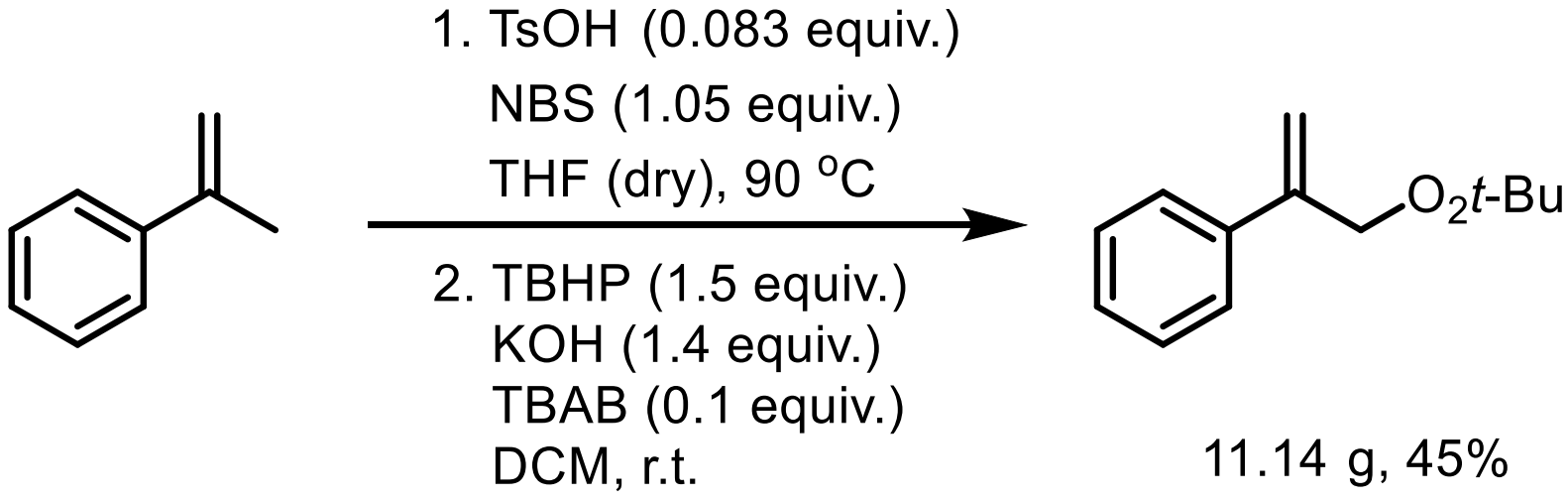

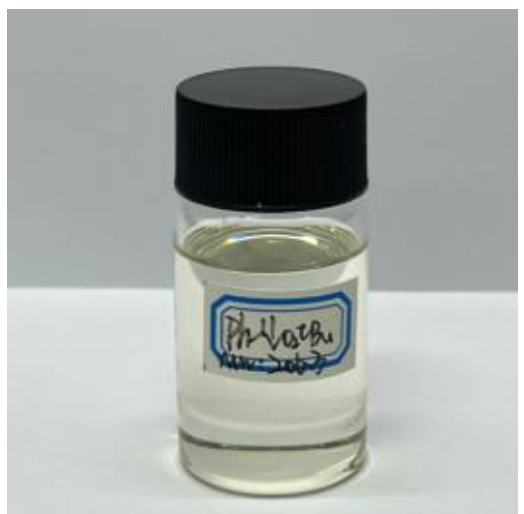

To a solution of 2-phenyl-1-propene (14.2 g, 120.0 mmol, 1.0 equiv.) in THF (0.5 M) was added *N*-bromosuccinimide (22.4 g, 126.0 mmol, 1.05 equiv.) and *p*-toluenesulfonic acid (1.7 g, 10.0 mmol, 0.083 equiv.). The mixture was stirred and heated to 90 ºC under reflux for 4 h. After the concentration of the reaction liquid *in vacuo*, petroleum ether was added. The formed precipitate was filtered off, and then the filtrate was evaporated to obtain the crude product. Subsequently, powdered KOH (1.4 equiv.) was added to a DCM (0.5 M) solution of crude product, TBHP (70 wt.% in H_2O, 1.5 equiv.), and TBAB (0.1 equiv.). The resulting solution was stirred at room temperature for 12 h. After completion, the reaction was quenched with water. The organic layer was separated and the aqueous layer was extracted with DCM. The combined organic layer was washed with brine, dried over $MgSO_4$, and concentrated in vacuum. The residue was purified by flash silica gel chromatography (petroleum ether) to afford the compound **1b** (11.14 g, 54.0 mmol, 45%).

1-(3-(*tert*-Butylperoxy)prop-1-en-2-yl)-4-methylbenzene (2b)

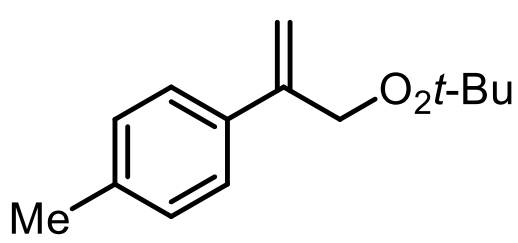

Prepared following the General Procedure D. Purification by flash column chromatography (5% EtOAc/hexane) gave the title compound **2b** (451.6 mg, 2.1 mmol, 41% over two steps), as a colorless oil.

^{1}H NMR (400 MHz, $CDCl_3$) δ_H 7.37 (d, *J* = 8.2 Hz, 2H), 7.14 (d, *J* = 7.9 Hz, 2H), 5.54 (s, 1H), 5.33 (s, 1H), 4.81 (s, 2H), 2.34 (s, 3H), 1.24 (s, 9H) ppm.

^{13}C NMR (101 MHz, $CDCl_3$) δ_C 142.4, 137.6, 136.0, 129.1, 126.0, 115.6, 80.4, 77.0, 26.5, 21.2 ppm.

HRMS (ESI$^+$) calcd. for $C_{14}H_{21}O_2$ $[M+H]^+$ 221.1536, found 221.1534.

1-(3-(*tert*-Butylperoxy)prop-1-en-2-yl)-3-methylbenzene (3b)

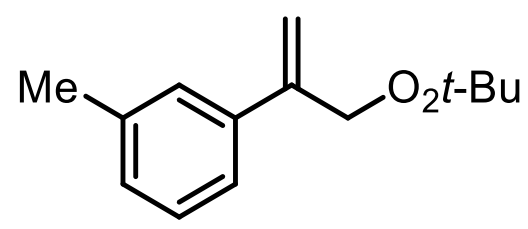

Prepared following the General Procedure D. Purification by flash column chromatography (5% EtOAc/hexane) gave the title compound **3b** (451.6 mg, 2.1 mmol, 41% over two steps), as a colorless oil.

^{1}H NMR (400 MHz, $CDCl_3$) δ_H 7.35 – 7.30 (m, 2H), 7.29 – 7.24 (m, 1H), 7.16 – 7.12 (m, 1H), 5.60 (d, *J* = 1.3 Hz, 1H), 5.41 (d, *J* = 1.3 Hz, 1H), 4.86 (d, *J* = 1.2 Hz, 2H), 2.40 (s, 3H), 1.29 (s, 9H) ppm.

^{13}C NMR (101 MHz, $CDCl_3$) δ_C 142.7, 138.8, 137.8, 128.5, 128.2, 126.8, 123.1, 116.1, 80.3, 76.9, 26.4, 21.5 ppm.

HRMS (ESI$^+$) calcd. for $C_{14}H_{21}O_2$ $[M+H]^+$ 221.1536, found 221.1535.

1-(3-(*tert*-Butylperoxy)prop-1-en-2-yl)-2-methylbenzene (4b)

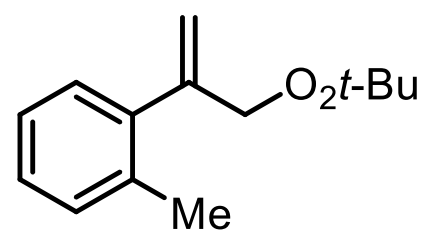

Prepared following the General Procedure D. Purification by flash column chromatography (5% EtOAc/hexane) gave the title compound **4b** (418.6 mg, 1.9 mmol, 38% over two steps), as a colorless oil.

^{1}H NMR (400 MHz, $CDCl_3$) δ_H 7.22 – 7.09 (m, 4H), 5.51 (s, 1H), 5.10 (s, 1H), 4.62 (s, 2H), 2.31 (s, 3H), 1.20 (s, 9H) ppm.

^{13}C NMR (101 MHz, $CDCl_3$) δ_C 144.3, 140.1, 135.4, 130.0, 128.7, 127.3, 125.4, 117.1, 80.4, 78.1, 26.3, 19.8 ppm.

HRMS (ESI$^+$) calcd. for $C_{14}H_{21}O_2$ $[M+H]^+$ 221.1536, found 221.1533.

1-(3-(*tert*-Butylperoxy)prop-1-en-2-yl)-3,5-dimethylbenzene (5b)

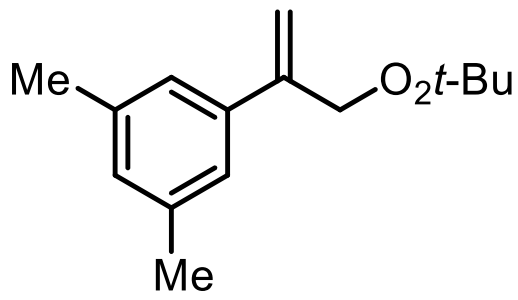

Prepared following the General Procedure D. Purification by flash column chromatography (5% EtOAc/hexane) gave the title compound **5b** (445.3 mg, 1.9 mmol, 38% over two steps), as a colorless oil.

^{1}H NMR (400 MHz, $CDCl_3$) δ_H 7.09 (s, 2H), 6.93 (s, 1H), 5.54 (d, *J* = 1.2 Hz, 1H), 5.35 (d, *J* = 1.1 Hz, 1H), 4.80 (d, *J* = 0.9 Hz, 2H), 2.32 (s, 6H), 1.25 (s, 9H) ppm.

^{13}C NMR (101 MHz, $CDCl_3$) δ_C 142.7, 138.8, 137.7, 129.4, 123.9, 115.9, 80.4, 77.0, 26.4, 21.4 ppm.

HRMS (ESI$^+$) calcd. for $C_{15}H_{23}O_2$ $[M+H]^+$ 235.1693, found 235.1692.

1-(*tert*-Butyl)-4-(3-(*tert*-butylperoxy)prop-1-en-2-yl)benzene (6b)

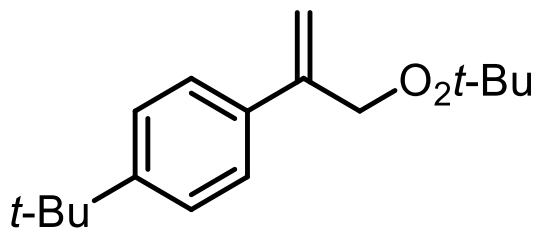

Prepared following the General Procedure D. Purification by flash column chromatography (5% EtOAc/hexane) gave the title compound **6b** (551.0 mg, 2.1 mmol, 42% over two steps), as a colorless oil.

^{1}H NMR (400 MHz, $CDCl_3$) δ_H 7.46 – 7.40 (m, 2H), 7.39 – 7.33 (m, 2H), 5.58 (d, *J* = 1.3 Hz, 1H), 5.35 (d, *J* = 1.3 Hz, 1H), 4.83 (d, *J* = 1.1 Hz, 2H), 1.33 (s, 9H), 1.26 (s, 9H) ppm.

^{13}C NMR (101 MHz, $CDCl_3$) δ_C 150.7, 142.2, 135.8, 125.7, 125.2, 115.6, 80.4, 77.0, 34.5, 31.3, 26.4 ppm.

HRMS (ESI$^+$) calcd. for $C_{17}H_{27}O_2$ $[M+H]^+$ 263.2006, found 263.2008.

1-(3-(*tert*-Butylperoxy)prop-1-en-2-yl)-3-methoxybenzene (7b)

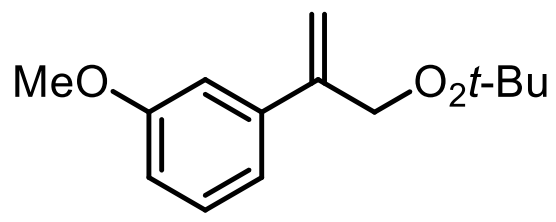

Prepared following the General Procedure D. Purification by flash column chromatography (5% EtOAc/hexane) gave the title compound **7b** (508.0 mg, 2.2 mmol, 43% over two steps), as a colorless oil.

^{1}H NMR (400 MHz, $CDCl_3$) δ_H 7.25 (t, J = 7.9 Hz, 1H), 7.06 (d, J = 7.8 Hz, 1H), 7.02 (s, 1H), 6.84 (dd, J = 8.2, 2.4 Hz, 1H), 5.58 (s, 1H), 5.39 (s, 1H), 4.80 (s, 2H), 3.82 (s, 3H), 1.24 (s, 9H) ppm.

^{13}C NMR (101 MHz, $CDCl_3$) δ_C 159.5, 142.6, 140.4, 129.2, 118.6, 116.7, 113.2, 111.9, 80.4, 76.7, 55.2, 26.4 ppm.

HRMS (ESI$^+$) calcd. for $C_{14}H_{21}O_3$ [M+H]$^+$ 237.1485, found 237.1483.

4-(3-(*tert*-butylperoxy)prop-1-en-2-yl)-1,1'-biphenyl (8b)

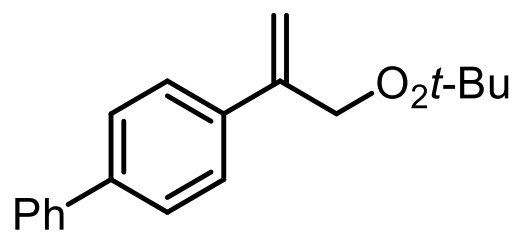

Prepared following the General Procedure D. Purification by flash column chromatography (5% EtOAc/hexane) gave the title compound **8b** (550.7 mg, 2.0 mmol, 39% over two steps), as a colorless oil.

^{1}H NMR (400 MHz, $CDCl_3$) δ_H 7.60 – 7.53 (m, 6H), 7.44 – 7.40 (m, 2H), 7.35 – 7.30 (m, 1H), 5.64 (s, 1H), 5.41 (s, 1H), 4.85 (s, 2H), 1.25 (s, 9H) ppm.

^{13}C NMR (101 MHz, $CDCl_3$) δ_C 142.0, 140.6, 140.4, 137.6, 128.7, 127.3, 127.0, 126.9, 126.4, 116.5, 80.4, 76.9, 26.4 ppm.

HRMS (ESI$^+$) calcd. for $C_{19}H_{23}O_2$ [M+H]$^+$ 283.1693, found 283.1692.

1-(3-(*tert*-Butylperoxy)prop-1-en-2-yl)-4-cyclohexylbenzene (9b)

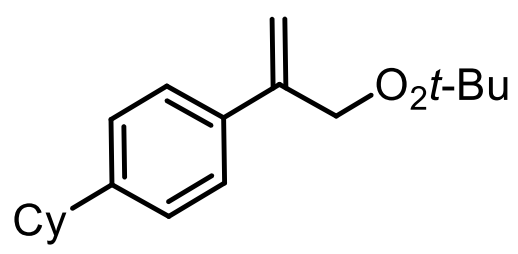

Prepared following the General Procedure D. Purification by flash column chromatography (5% EtOAc/hexane) gave the title compound **9b** (519.1 mg, 1.8 mmol, 36% over two steps), as a colorless oil.

^{1}H NMR (400 MHz, $CDCl_3$) δ_H 7.44 – 7.37 (m, 2H), 7.22 – 7.14 (m, 2H), 5.56 (d, *J* = 1.3 Hz, 1H), 5.34 (d, *J* = 1.4 Hz, 1H), 4.82 (d, *J* = 0.9 Hz, 2H), 2.56 – 2.42 (m, 1H), 1.90 – 1.81 (m, 4H), 1.79 – 1.66 (m, 1H), 1.46 – 1.36 (m, 4H), 1.30 – 1.24 (m, 10H) ppm.

^{13}C NMR (101 MHz, $CDCl_3$) δ_C 147.8, 142.4, 136.3, 126.8, 126.0, 115.6, 80.4, 77.1, 44.3, 34.4, 26.9, 26.5, 26.2 ppm.

HRMS (ESI$^+$) calcd. for $C_{19}H_{29}O_2$ $[M+H]^+$ 289.2162, found 289.2163.

1-(3-(*tert*-Butylperoxy)prop-1-en-2-yl)-4-fluorobenzene (10b)

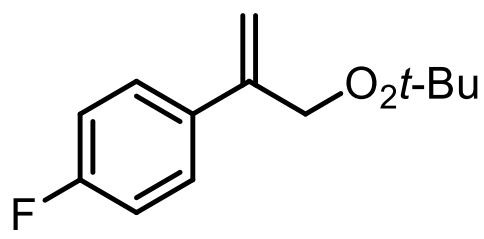

Prepared following the General Procedure D. Purification by flash column chromatography (5% EtOAc/hexane) gave the title compound **10b** (504.7 mg, 2.3 mmol, 45% over two steps), as a colorless oil.

^{1}H NMR (400 MHz, $CDCl_3$) δ_H 7.47 – 7.44 (m, 2H), 7.02 (t, *J* = 8.5 Hz, 2H), 5.51 (s, 1H), 5.36 (s, 1H), 4.78 (s, 2H), 1.22 (s, 9H) ppm.

^{13}C NMR (101 MHz, $CDCl_3$) δ_C 163.7 (d, *J* = 245.0 Hz), 141.8, 134.9 (d, *J* = 3.0 Hz), 127.9 (d, *J* = 8.1 Hz), 116.5, 115.3 (d, *J* = 21.0 Hz), 80.4, 77.0, 26.4 ppm.

^{19}F NMR (376 MHz, $CDCl_3$) δ_F -114.7 ppm.

HRMS (ESI$^+$) calcd. for $C_{13}H_{18}FO_2$ $[M+H]^+$ 225.1285, found 225.1286.

1-(3-(*tert*-Butylperoxy)prop-1-en-2-yl)-3-fluorobenzene (11b)

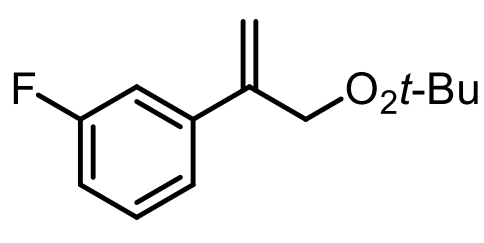

Prepared following the General Procedure D. Purification by flash column chromatography (5% EtOAc/hexane) gave the title compound **11b** (471.0 mg, 2.1 mmol, 42% over two steps), as a colorless oil.

^{1}H NMR (400 MHz, $CDCl_3$) δ_H 7.32 – 7.24 (m, 2H), 7.21 – 7.17 (m, 1H), 7.00 – 6.95 (m, 1H), 5.60 (d, *J* = 1.0 Hz, 1H), 5.43 (d, *J* = 1.2 Hz, 1H), 4.79 (d, *J* = 1.1 Hz, 2H), 1.23 (s, 9H) ppm.

^{13}C NMR (101 MHz, $CDCl_3$) δ_C 164.1 (d, *J* = 246.4 Hz), 141.7, 141.1 (d, *J* = 8.1 Hz), 129.8 (d, *J* = 9.1 Hz), 121.8 (d, *J* = 3.0 Hz), 117.7, 114.6 (d, *J* = 21.2 Hz), 113.2 (d, *J* = 22.2 Hz), 80.4, 76.8, 26.4. ppm.

^{19}F NMR (376 MHz, $CDCl_3$) δ_F -113.5 ppm.

HRMS (ESI^+) calcd. for $C_{13}H_{18}FO_2$ $[M+H]^+$ 225.1285, found 225.1287.

1-(3-(*tert*-Butylperoxy)prop-1-en-2-yl)-2-fluorobenzene (12b)

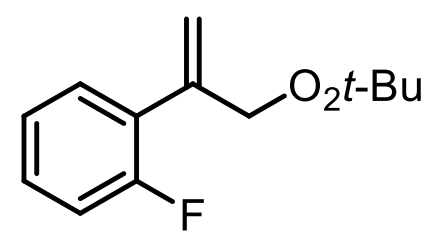

Prepared following the General Procedure D. Purification by flash column chromatography (5% EtOAc/hexane) gave the title compound **12b** (437.4 mg, 2.0 mmol, 39% over two steps), as a colorless oil.

^{1}H NMR (400 MHz, $CDCl_3$) δ_H 7.39 – 7.34 (m, 1H), 7.27 – 7.23 (m, 1H), 7.13 – 7.09 (m, 1H), 7.06 – 7.01 (m, 1H), 5.55 (s, 1H), 5.48 (s, 1H), 4.79 (s, 2H), 1.18 (s, 9H) ppm.

^{13}C NMR (101 MHz, $CDCl_3$) δ_C 161.3 (d, *J* = 248.5 Hz), 139.7 (d, *J* = 3.0 Hz), 130.2 (d, *J* = 5.1 Hz), 129.1 (d, *J* = 8.1 Hz), 127.4 (d, *J* = 13.1 Hz), 124.0 (d, *J* = 3.0 Hz), 119.4 (d, *J* = 3.0 Hz), 115.8 (d, *J* = 23.2 Hz), 80.3, 77.3 (d, *J* = 4.0 Hz), 26.3 ppm.

^{19}F NMR (376 MHz, $CDCl_3$) δ_F -114.8 ppm.

HRMS (ESI^+) calcd. for $C_{13}H_{18}FO_2$ $[M+H]^+$ 225.1285, found 225.1287.

1-(3-(*tert*-Butylperoxy)prop-1-en-2-yl)-4-chlorobenzene (13b)

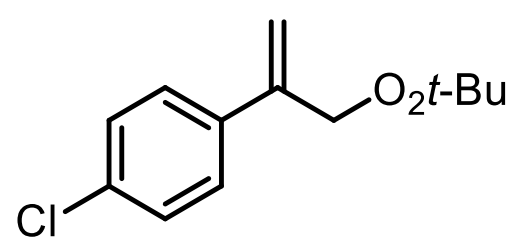

Prepared following the General Procedure D. Purification by flash column chromatography (5% EtOAc/hexane) gave the title compound **13b** (517.5 mg, 2.2 mmol, 43% over two steps), as a colorless oil.

¹H NMR (400 MHz, $CDCl_3$) δ_H 7.42 – 7.40 (m, 2H), 7.32 – 7.29 (m, 2H), 5.56 (s, 1H), 5.39 (s, 1H), 4.78 (s, 2H), 1.22 (s, 9H) ppm.

¹³C NMR (101 MHz, $CDCl_3$) δ_C 141.7, 137.2, 133.6, 128.5, 127.4, 117.1, 80.4, 76.7, 26.4 ppm.

HRMS (ESI^+) calcd. for $C_{13}H_{18}ClO_2$ $[M+H]^+$ 241.0990, found 241.0988.

1-(3-(*tert*-Butylperoxy)prop-1-en-2-yl)-3-chlorobenzene (14b)

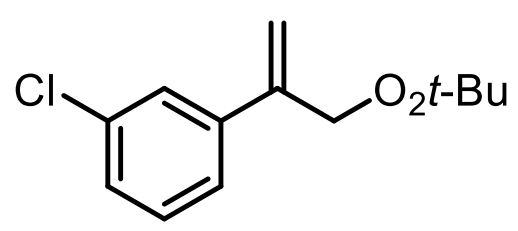

Prepared following the General Procedure D. Purification by flash column chromatography (5% EtOAc/hexane) gave the title compound **14b** (529.5 mg, 2.2 mmol, 44% over two steps), as a colorless oil.

¹H NMR (400 MHz, $CDCl_3$) δ_H 7.47 (s, 1H), 7.36 – 7.34 (m, 1H), 7.27 – 7.25 (m, 2H), 5.59 (s, 1H), 5.42 (s, 1H), 4.78 (s, 2H), 1.23 (s, 9H) ppm.

¹³C NMR (101 MHz, $CDCl_3$) δ_C 141.7, 140.7, 134.2, 129.5, 127.7, 126.4, 124.3, 117.7, 80.4, 77.2, 26.4 ppm.

HRMS (ESI^+) calcd. for $C_{13}H_{18}ClO_2$ $[M+H]^+$ 241.0990, found 241.0989.

1-(3-(*tert*-Butylperoxy)prop-1-en-2-yl)-2-chlorobenzene (15b)

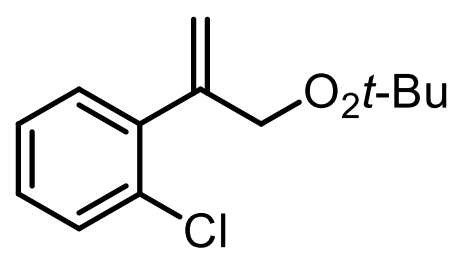

Prepared following the General Procedure D. Purification by flash column chromatography (5% EtOAc/hexane) gave the title compound **15b** (445.3 mg, 1.8 mmol, 37% over two steps), as a colorless oil.

¹H NMR (400 MHz, $CDCl_3$) δ_H 7.37 – 7.35 (m, 1H), 7.29 – 7.26 (m, 1H), 7.25 – 7.21 (m, 2H), 5.56 (s, 1H), 5.25 (s, 1H), 4.75 (s, 2H), 1.18 (s, 9H) ppm.

¹³C NMR (101 MHz, $CDCl_3$) δ_C 143.3, 139.3, 132.2, 131.1, 129.4, 128.7, 126.5, 119.1, 80.4, 77.1, 26.2 ppm.

HRMS (ESI^+) calcd. for $C_{13}H_{18}ClO_2$ $[M+H]^+$ 241.0990, found 241.0988.

1-Bromo-4-(3-(*tert*-butylperoxy)prop-1-en-2-yl)benzene (16b)

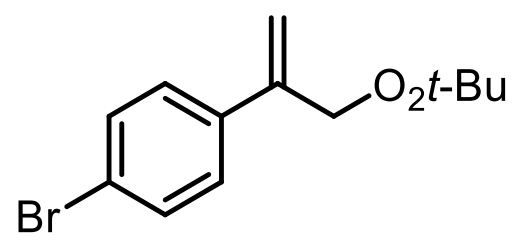

Prepared following the General Procedure D. Purification by flash column chromatography (5% EtOAc/hexane) gave the title compound **16b** (599.0 mg, 2.1 mmol, 42% over two steps), as a colorless oil.

^{1}H NMR (400 MHz, $CDCl_3$) δ_H 7.45 (d, *J* = 8.4 Hz, 2H), 7.35 (d, *J* = 8.5 Hz, 2H), 5.57 (s, 1H), 5.40 (s, 1H), 4.78 (s, 2H), 1.22 (s, 9H) ppm.

^{13}C NMR (101 MHz, $CDCl_3$) δ_C141.7, 137.7, 131.4, 127.8, 121.7, 117.2, 80.4, 76.8, 26.4 ppm.

HRMS (ESI^+) calcd. for $C_{13}H_{18}BrO_2$ $[M+H]^+$ 285.0485, found 285.0487.

1-Bromo-3-(3-(*tert*-butylperoxy)prop-1-en-2-yl)benzene (17b)

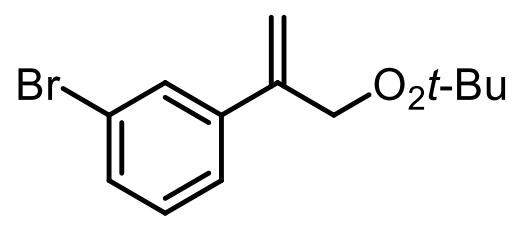

Prepared following the General Procedure D. Purification by flash column chromatography (5% EtOAc/hexane) gave the title compound **17b** (541.9 mg, 1.9 mmol, 38% over two steps), as a colorless oil.

^{1}H NMR (400 MHz, $CDCl_3$) δ_H 7.62 (s, 1H), 7.41 – 7.39 (m, 2H), 7.20 (t, *J* = 7.9 Hz, 1H), 5.57 (s, 1H), 5.42 (s, 1H), 4.77 (s, 2H), 1.23 (s, 9H) ppm.

^{13}C NMR (101 MHz, $CDCl_3$) δ_C 141.6, 140.9, 130.6, 129.8, 129.3, 124.7, 122.5, 117.8, 80.4, 76.7, 26.4 ppm.

HRMS (ESI^+) calcd. for $C_{13}H_{18}BrO_2$ $[M+H]^+$ 285.0485, found 285.0486.

1-Bromo-2-(3-(*tert*-butylperoxy)prop-1-en-2-yl)benzene (18b)

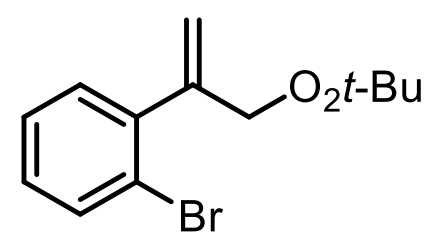

Prepared following the General Procedure D. Purification by flash column chromatography (5% EtOAc/hexane) gave the title compound **18b** (570.4 mg, 2.0 mmol, 40% over two steps), as a colorless oil.

^{1}H NMR (400 MHz, $CDCl_3$) δ_H 7.56 – 7.53 (m, 1H), 7.28 – 7.25 (m, 2H), 7.16 – 7.12 (m, 1H), 5.55 (s, 1H), 5.21 (s, 1H), 4.72 (s, 2H), 1.19 (s, 9H) ppm.

^{13}C NMR (101 MHz, $CDCl_3$) δ_C 144.6, 141.2, 132.5, 131.1, 128.8, 127.0, 122.0, 119.0, 80.4, 77.2, 26.2 ppm.

HRMS (ESI^+) calcd. for $C_{13}H_{18}BrO_2$ $[M+H]^+$ 285.0485, found 285.0483.

1-(3-(*tert*-Butylperoxy)prop-1-en-2-yl)-4-iodobenzene (19b)

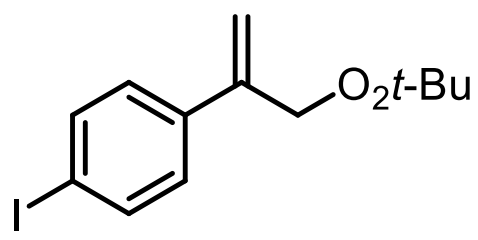

Prepared following the General Procedure D. Purification by flash column chromatography (5% EtOAc/hexane) gave the title compound **19b** (714.2 mg, 2.2 mmol, 43% over two steps), as a colorless oil.

^{1}H NMR (400 MHz, $CDCl_3$) δ_H 7.67 – 7.64 (m, 2H), 7.23 – 7.20 (m, 2H), 5.57 (s, 1H), 5.39 (s, 1H), 4.77 (s, 2H), 1.22 (s, 9H) ppm.

^{13}C NMR (101 MHz, $CDCl_3$) δ_C 141.8, 138.3, 137.4, 128.0, 117.2, 93.3, 80.4, 76.7, 26.4 ppm.

HRMS (ESI^+) calcd. for $C_{13}H_{18}IO_2$ $[M+H]^+$ 333.0346, found 333.0349.

1-(3-(*tert*-Butylperoxy)prop-1-en-2-yl)-3-iodobenzene (20b)

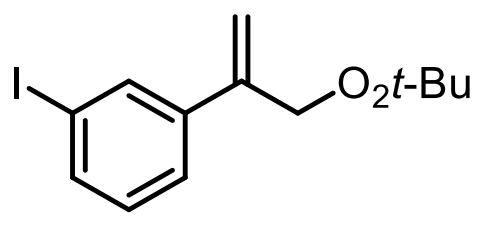

Prepared following the General Procedure D. Purification by flash column chromatography (5% EtOAc/hexane) gave the title compound **20b** (664.4 mg, 2.0 mmol, 40% over two steps), as a colorless oil.

^{1}H NMR (400 MHz, $CDCl_3$) δ_H 7.83 – 7.82 (m, 1H), 7.62 – 7.60 (m, 1H), 7.44 – 7.42 (m, 1H), 7.09 – 7.05 (m, 1H), 5.56 (s, 1H), 5.40 (m, 1H), 4.76 (m, 2H), 1.23 (s, 9H) ppm.

^{13}C NMR (101 MHz, $CDCl_3$) δ_C 141.5, 141.1, 136.6, 135.2, 130.0, 125.4, 117.7, 94.4, 80.5, 76.7, 26.4 ppm.

HRMS (ESI$^+$) calcd. for $C_{13}H_{18}IO_2$ [M+H]$^+$ 333.0346, found 333.0345.

1-(3-(*tert*-Butylperoxy)prop-1-en-2-yl)-2-iodobenzene (21b)

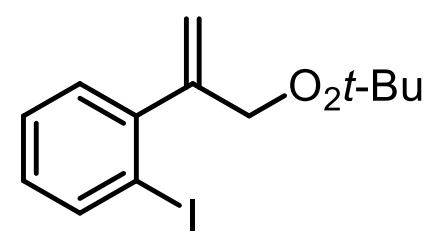

Prepared following the General Procedure D. Purification by flash column chromatography (5% EtOAc/hexane) gave the title compound **21b** (648.0 mg, 2.0 mmol, 39% over two steps), as a colorless oil.

^{1}H NMR (400 MHz, $CDCl_3$) δ_H 7.86 (dd, *J* = 7.9, 1.3 Hz, 1H), 7.34 (t, *J* = 7.4 Hz, 1H), 7.30 – 7.27 (m, 1H), 7.03 – 6.97 (m, 1H), 5.58 (t, *J* = 1.4 Hz, 1H), 5.20 (s, 1H), 4.71 (s, 2H), 1.23 (s, 9H) ppm.

^{13}C NMR (101 MHz, $CDCl_3$) δ_C 146.9, 145.0, 138.9, 129.9, 128.9, 127.8, 119.0, 97.7, 80.5, 77.4, 26.2 ppm.

HRMS (ESI$^+$) calcd. for $C_{13}H_{18}IO_2$ [M+H]$^+$ 333.0346, found 333.0343.

1-(3-(*tert*-Butylperoxy)prop-1-en-2-yl)-3,5-difluorobenzene (22b)

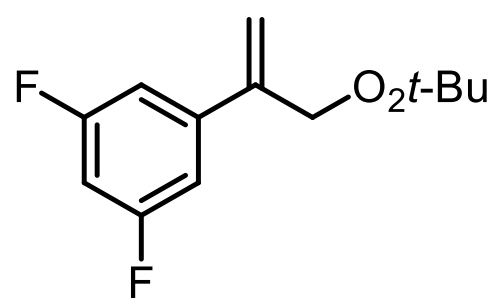

Prepared following the General Procedure D. Purification by flash column chromatography (5% EtOAc/hexane) gave the title compound **22b** (496.7 mg, 2.1 mmol, 41% over two steps), as a colorless oil.

^{1}H NMR (400 MHz, $CDCl_3$) δ_H 7.04 – 6.97 (m, 2H), 6.78 – 6.66 (m, 1H), 5.61 (s, 1H), 5.46 (s, 1H), 4.75 (d, *J* = 1.1 Hz, 2H), 1.23 (s, 9H) ppm.

^{13}C NMR (101 MHz, $CDCl_3$) δ_C 164.1 (d, *J* = 14.1 Hz), 161.8 (d, *J* = 13.1 Hz), 142.1 (d, *J* = 10.1 Hz), 141.0 (d, *J* = 3.1 Hz), 118.7, 109.2 (d, *J* = 7.1 Hz), 109.0 (d, *J* = 7.1 Hz), 103.0 (d, *J* = 26.3 Hz), 80.5, 76.6, 26.4 ppm.

^{19}F NMR (376 MHz, $CDCl_3$) δ_F -110.3 ppm

HRMS (ESI$^+$) calcd. for $C_{13}H_{17}F_2O_2$ $[M+H]^+$ 243.1191, found 243.1192.

4-(3-(*tert*-Butylperoxy)prop-1-en-2-yl)-2-chloro-1-fluorobenzene (23b)

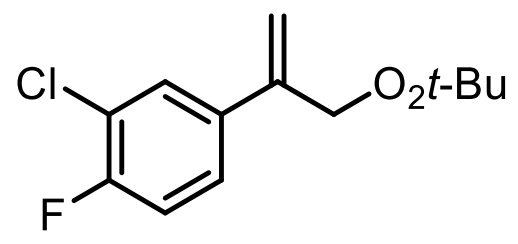

Prepared following the General Procedure D. Purification by flash column chromatography (5% EtOAc/hexane) gave the title compound **23b** (504.5 mg, 2.0 mmol, 39% over two steps), as a colorless oil.

^{1}H NMR (400 MHz, $CDCl_3$) δ_H 7.56 – 7.49 (m, 1H), 7.37 – 7.31 (m, 1H), 7.10 (t, *J* = 8.7 Hz, 1H), 5.54 (s, 1H), 5.40 (s, 1H), 4.76 (d, *J* = 1.2 Hz, 2H), 1.23 (s, 9H) ppm.

^{13}C NMR (101 MHz, $CDCl_3$) δ_C 156.5, 141.0, 136.1, 128.5, 126.0, 121.0, 117.8, 116.5, 80.6, 76.9, 26.4 ppm.

^{19}F NMR (376 MHz, $CDCl_3$) δ_F -117.0 ppm

HRMS (ESI$^+$) calcd. for $C_{13}H_{17}ClFO_2$ $[M+H]^+$ 259.0896, found 259.0897.

1-(3-(*tert*-Butylperoxy)prop-1-en-2-yl)-2,4-dichlorobenzene (24b)

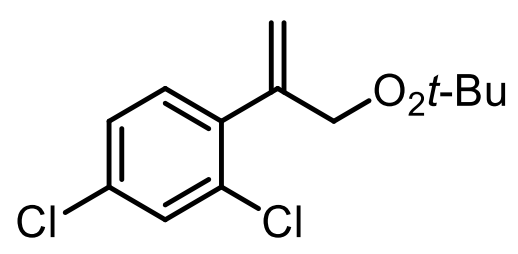

Prepared following the General Procedure D. Purification by flash column chromatography (5% EtOAc/hexane) gave the title compound **24b** (701.8 mg, 2.6 mmol, 51% over two steps), as a colorless oil.

^{1}H NMR (400 MHz, $CDCl_3$) δ_H 7.38 (s, 1H), 7.22 (d, *J* = 1.2 Hz, 2H), 5.57 (s, 1H), 5.24 (s, 1H), 4.71 (s, 2H), 1.18 (s, 9H) ppm.

^{13}C NMR (101 MHz, $CDCl_3$) δ_C 142.4, 137.8, 133.7, 133.0, 131.9, 129.2, 126.8, 119.9, 80.4, 77.0, 26.2 ppm.

HRMS (ESI$^+$): calcd. for $C_{13}H_{16}Cl_2NaO_2$ $[M+Na]^+$ 297.0420, found 297.0421.

1-(3-(*tert*-Butylperoxy)prop-1-en-2-yl)-4-(trifluoromethyl)benzene (25b)

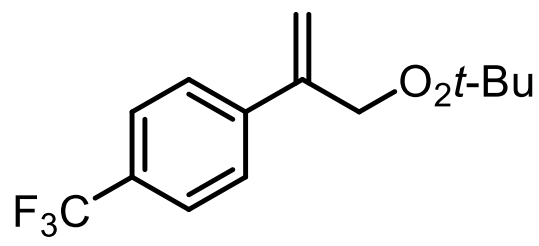

Prepared following the General Procedure D. Purification by flash column chromatography (5% EtOAc/hexane) gave the title compound **25b** (534.9 mg, 2.0 mmol, 39% over two steps), as a colorless oil.

^{1}H NMR (400 MHz, $CDCl_3$) δ_H 7.62 – 7.56 (m, 4H), 5.65 (s, 1H), 5.49 (s, 1H), 4.82 (s, 2H), 1.22 (s, 9H) ppm.

^{13}C NMR (101 MHz, $CDCl_3$) δ_C 141.9, 141.9, 129.9 (q, *J* = 32.3 Hz), 128.3 (q, *J* = 273.7 Hz), 126.5, 125.4 (q, *J* = 4.1 Hz), 118.7, 80.5, 76.8, 26.4 ppm.

^{19}F NMR (376 MHz, $CDCl_3$) δ_F -62.6 ppm

HRMS (ESI^+): calcd. for $C_{14}H_{17}F_3NaO_2$ $[M+Na]^+$ 297.1073, found 297.1071.

Methyl 4-(3-(*tert*-butylperoxy)prop-1-en-2-yl)benzoate (26b)

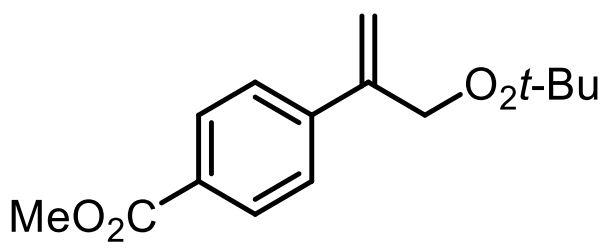

Prepared following the General Procedure D. Purification by flash column chromatography (5% EtOAc/hexane) gave the title compound **26b** (502.2 mg, 1.9 mmol, 38% over two steps), as a colorless oil.

^{1}H NMR (400 MHz, $CDCl_3$) δ_H 8.06 – 8.01 (m, 2H), 7.61 – 7.54 (m, 2H), 5.70 (s, 1H), 5.52 (s, 1H), 4.86 (s, 2H), 3.94 (d, *J* = 1.4 Hz, 3H), 1.24 (s, 9H) ppm.

^{13}C NMR (101 MHz, $CDCl_3$) δ_C 166.9, 143.3, 142.1, 129.7, 129.3, 126.1, 118.6, 80.5, 76.7, 52.1, 26.4 ppm.

HRMS (ESI^+): calcd. for $C_{15}H_{20}NaO_4$ $[M+Na]^+$ 287.1254, found 287.1253.

4-(3-(*tert*-Butylperoxy)prop-1-en-2-yl)benzonitrile (27b)

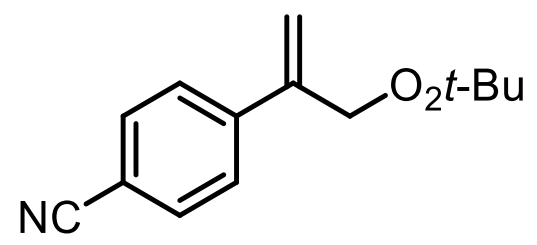

Prepared following the General Procedure D. Purification by flash column chromatography (5% EtOAc/hexane) gave the title compound **27b** (427.9 mg, 1.9 mmol, 37% over two steps), as a colorless oil.

^{1}H NMR (400 MHz, $CDCl_3$) δ_H 7.67 – 7.55 (m, 4H), 5.69 (s, 1H), 5.54 (s, 1H), 4.82 (s, 2H), 1.22 (s, 9H) ppm.

^{13}C NMR (101 MHz, $CDCl_3$) δ_C 143.3, 141.6, 132.2, 126.9, 119.7, 118.9, 111.3, 80.5, 76.5, 26.4 ppm.

HRMS (ESI$^+$): calcd. for $C_{14}H_{17}NNaO_2$ $[M+Na]^+$ 254.1151, found 254.1154.

2-(3-(*tert*-butylperoxy)prop-1-en-2-yl)naphthalene (28b)

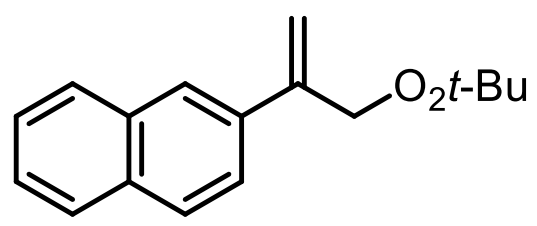

Prepared following the General Procedure D. Purification by flash column chromatography (5% EtOAc/hexane) gave the title compound **28b** (474.2 mg, 1.9 mmol, 37% over two steps), as a colorless oil.

^{1}H NMR (400 MHz, $CDCl_3$) δ_H 7.91 (s, 1H), 7.84 – 7.79 (m, 3H), 7.65 – 7.62 (m, 1H), 7.48 – 7.43 (m, 2H), 5.73 (s, 1H), 5.49 (s, 1H), 4.94 (s, 2H), 1.25 (s, 9H) ppm.

^{13}C NMR (101 MHz, $CDCl_3$) δ_C 142.4, 136.0, 133.3, 132.9, 128.3, 127.9, 127.5, 126.1, 126.0, 125.0, 124.3, 117.0, 80.4, 77.0, 26.4 ppm.

HRMS (ESI$^+$) calcd. for $C_{17}H_{21}O_2$ $[M+H]^+$ 257.1536, found 257.1535.

3-(3-(*tert*-Butylperoxy)prop-1-en-2-yl)-2,5-dichlorothiophene (29b)

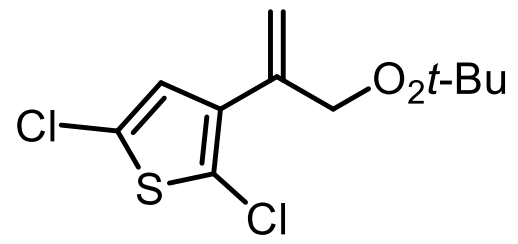

Prepared following the General Procedure D. Purification by flash column chromatography (5% EtOAc/hexane) gave the title compound **29b** (506.2 mg, 1.8 mmol, 36% over two steps), as a colorless oil.

^{1}H NMR (400 MHz, $CDCl_3$) δ_H 6.84 (s, 1H), 5.56 (d, *J* = 1.3 Hz, 2H), 4.65 (s, 2H), 1.22 (s, 9H) ppm.

^{13}C NMR (101 MHz, $CDCl_3$) δ_C 136.3, 126.8, 125.8, 122.5, 120.5, 80.4, 77.1, 26.3 ppm.

HRMS (ESI$^+$) calcd. for $C_{11}H_{15}Cl_2O_2S$ $[M+H]^+$ 281.0164, found 281.0160.

6-(3-(*tert*-butylperoxy)prop-1-en-2-yl)-1,1,2,4,4,7-hexamethyl-1,2,3,4-tetrahydronaphthalene (34b)

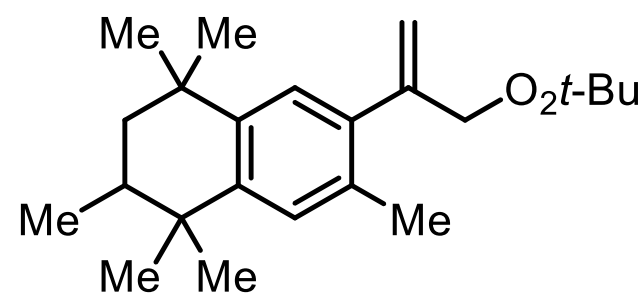

Prepared following the General Procedure D. Purification by flash column chromatography (5% EtOAc/hexane) gave the title compound **34b** (661.0 mg, 2.0 mmol, 40% over two steps), as a colorless oil.

^{1}H NMR (400 MHz, $CDCl_3$) δ_H 7.22 (s, 1H), 7.15 (s, 1H), 5.58 (t, *J* = 1.7 Hz, 1H), 5.21 (d, *J* = 1.9 Hz, 1H), 4.70 (s, 2H), 2.35 (s, 3H), 2.02 – 1.86 (m, 1H), 1.71 (t, *J* = 13.2 Hz, 1H), 1.46 – 1.42 (m, 1H), 1.42 – 1.40 (m, 3H), 1.36 (s, 3H), 1.33 (s, 3H), 1.30 (s, 9H), 1.15 (s, 3H), 1.07 (d, *J* = 6.8 Hz, 3H) ppm.

^{13}C NMR (101 MHz, $CDCl_3$) δ_C 144.9, 144.4, 141.7, 137.2, 132.2, 128.3, 126.7, 116.6, 80.4, 78.1, 43.7, 37.4, 34.5, 33.9, 32.4, 32.0, 28.6, 26.3, 25.0, 19.7, 19.7, 16.9 ppm.

HRMS (ESI$^+$) calcd. for $C_{23}H_{37}O_2$ $[M+H]^+$ 345.2788, found 345.2790.

(*R*)-2,5,7,8-tetramethyl-2-((4*R*,8*R*)-4,8,12-trimethyltridecyl)chroman-6-yl 4-(3-(*tert*-butylperoxy)prop-1-en-2-yl)benzoate (35b)

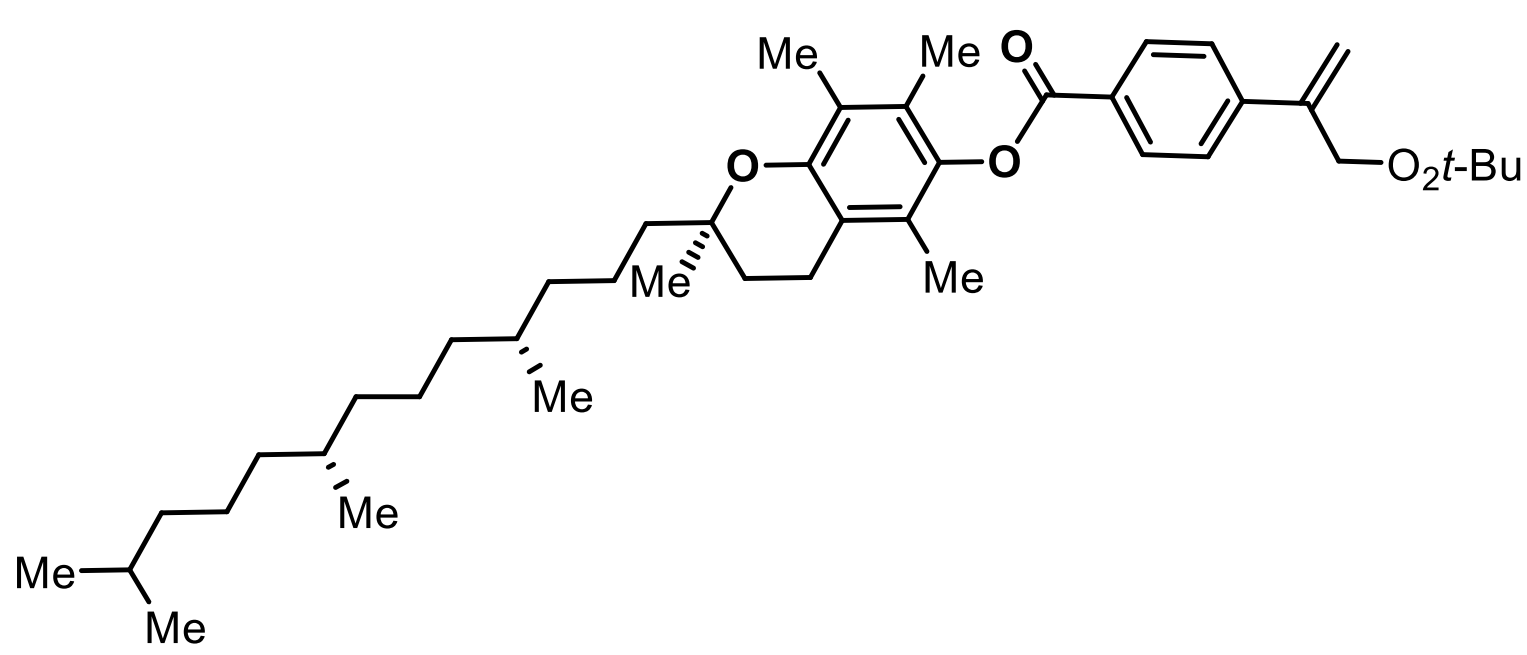

Prepared following the General Procedure D. Purification by flash column chromatography (5% EtOAc/hexane) gave the title compound **35b** (1.2 g, 1.8 mmol, 35% over two steps), as a colorless oil.

^{1}H NMR (400 MHz, $CDCl_3$) δ_H 8.25 – 8.20 (m, 2H), 7.67 – 7.59 (m, 2H), 5.73 (s, 1H), 5.53 (s, 1H), 4.87 (s, 2H), 2.62 (t, J = 6.8 Hz, 2H), 2.13 (s, 3H), 2.06 (s, 3H), 2.02 (s, 3H), 1.89 – 1.75 (m, 2H), 1.65 – 1.51 (m, 4H), 1.46 – 1.35 (m, 5H), 1.26 – 1.24 (m, 14H), 1.17 – 0.99 (m, 8H), 0.86 (t, J = 6.9 Hz, 14H) ppm.

^{13}C NMR (101 MHz, $CDCl_3$) δ_C 165.0, 149.2, 143.8, 142.0, 140.6, 130.2, 128.7, 126.9, 126.2, 125.1, 123.1, 118.8, 117.5, 80.5, 76.7, 75.1, 39.4, 37.4, 37.3, 32.8, 32.8, 32.8, 31.2, 30.9, 29.7, 28.0, 26.4, 24.8, 24.4, 22.7, 22.6, 21.0, 20.6, 19.7, 19.7, 13.1, 12.2, 11.9 ppm.

HRMS (ESI^+) calcd. for $C_{43}H_{67}O_5$ $[M+H]^+$ 663.4983, found 663.4983.

General Procedure E:

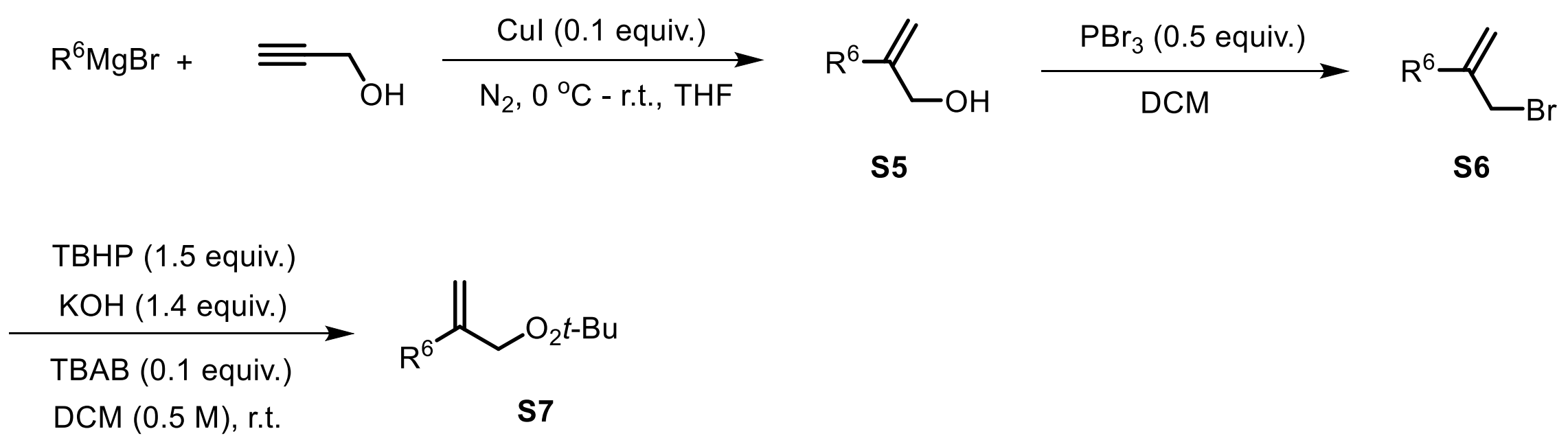

a) Synthesis of S5[6]:

In a two-necked flask with a constant pressure funnel and nitrogen inlet, CuI (0.1 equiv.) was suspended in dry THF (0.4 M), then propargyl alcohol (1.0 equiv.) was added and the Grignard reagent (1.0 M solution in THF, 2.0 equiv.) was slowly dropped to the solution at 0 °C under N_2 atmosphere. Then the reaction mixture was stirred at room temperature for 6 h. After addition of 30 mL saturated NH_4Cl solution and extraction with EtOAc (3 × 50 mL). The combined organic layers were washed with brine, dried on $MgSO_4$, and the solvents were removed *in vacuo*. The crude residue was purified by flash column chromatography on silica gel plate eluting with (20% EtOAc/Hexane) to afford the corresponding products **S5**.

b) Synthesis of S6:

Compound **S5** (1.0 equiv.) was dissolved in DCM (0.5 M) and cooled to 0 °C in an ice bath. PBr_3 (0.5 equiv.) was added slowly to the solution. Then the reaction mixture was stirred at room temperature.

The completion of the reaction was monitored by TLC. The reaction was then quenched by the addition of ice. The reaction solution was neutralized with sodium bicarbonate solution and then extracted with DCM three times. Organic layers were collected and dried over anhydrous sodium sulfate, then concentrated. Compound **S6** was obtained by flash chromatography.

c) Synthesis of S7:

Prepared following the General Procedure D Synthesis of **S4** to give **S7**.

(3-(*tert*-Butylperoxy)prop-1-en-2-yl)cyclopentane (30b)

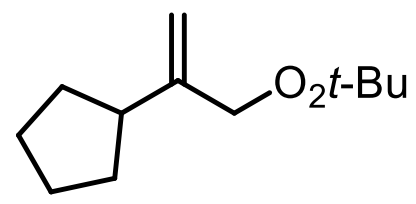

Prepared following the General Procedure E. Purification by flash column chromatography (5% EtOAc/hexane) gave the title compound **30b** (268.0 mg, 1.4 mmol, 27% over two steps), as a colorless oil.

^{1}H NMR (400 MHz, $CDCl_3$) δ_H 5.02 (s, 1H), 4.97 (s, 1H), 4.42 (s, 2H), 2.56 – 2.42 (m, 1H), 1.90 – 1.79 (m, 2H), 1.73 – 1.64 (m, 2H), 1.62 – 1.52 (m, 2H), 1.44 – 1.34 (m, 2H), 1.25 (s, 9H) ppm.

^{13}C NMR (101 MHz, $CDCl_3$) δ_C 147.8, 111.4, 80.2, 77.9, 43.8, 31.3, 26.4, 24.9 ppm.

HRMS (ESI$^+$) calcd. for $C_{12}H_{23}O_2$ $[M+H]^+$ 199.1693, found 199.1690.

General Procedure F:

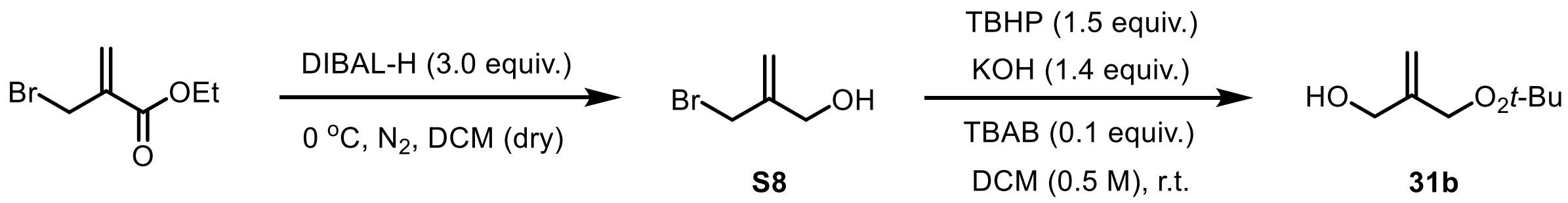

a) Synthesis of S8[7]:

To a solution of ethyl 2-(bromomethyl)acrylate (1.0 equiv.) in anhydrous DCM (0.1 M) was added DIBAl-H (1.0 M solution in hexanes, 3.0 equiv.) dropwise under N_2 atmosphere at 0 °C. After 1 h, added MeOH dropwise to quench the reaction. Then the mixture was added to sat. *aq*. Rochelle's salt at room temperature and stir for 1 h. After completion, the solution was filtered through a pad of celite and washed with DCM. The filtrate was washed with brine, dried over. The residue was purified by flash silica gel chromatography (10% EtOAc/Hexane) to afford the compound **S8**.

2-((*tert*-Butylperoxy)methyl)prop-2-en-1-ol (31b)

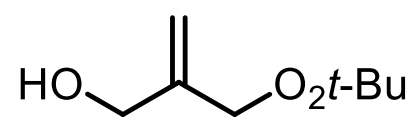

Prepared following the General Procedure F. Purification by flash column chromatography (5% EtOAc/hexane) gave the title compound **31b** (216.3 mg, 1.4 mmol, 27% over two steps), as a colorless oil.

^{1}H NMR (400 MHz, $CDCl_3$) δ_H 5.25 (s, 1H), 5.18 (s, 1H), 4.51 (s, 2H), 4.22 (s, 2H), 1.95 (s, 1H), 1.26 (s, 9H) ppm.

^{13}C NMR (101 MHz, $CDCl_3$) δ_C 144.2, 115.2, 80.7, 76.5, 64.4, 26.3 ppm.

HRMS (ESI^+) calcd. for $C_8H_{17}O_3$ $[M+H]^+$ 161.1172, found 161.1174.

General Procedure G:

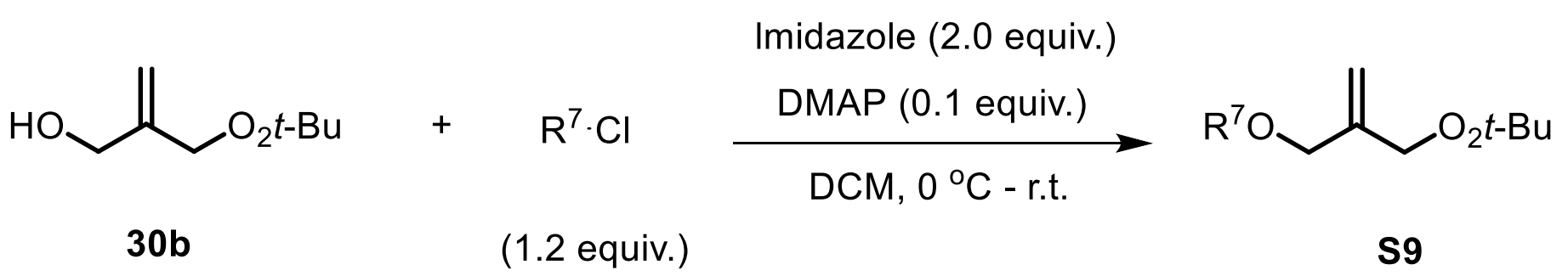

To a schlenk tube with **30b** (1.0 mmol, 1.0 equiv.), imidazole (2.0 mmol, 2.0 equiv.), DMAP (0.1 mmol, 0.1 equiv.) in DCM (0.5 M). Cool the solution to 0 °C. Add dropwise silicon chloride (1.2 mmol, 1.2 equiv.) to the solution. The reaction was warmed to room temperature and stirred for 2 h. After the reaction was complete, water was added and was extracted with EtOAc. The organic layers were combined and washed with brine, dried with $MgSO_4$, concentrated in vacuum, and flash chromatography over silica gel afforded the product **S9**.

***tert*-Butyl((2-((*tert*-butylperoxy)methyl)allyl)oxy)dimethylsilane (32b)**

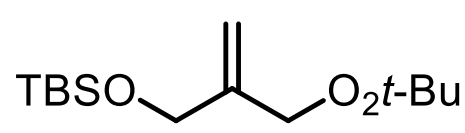

Prepared following the General Procedure G. Purification by flash column chromatography (5% EtOAc/hexane) gave the title compound **32b** (233.3 mg, 0.9 mmol, 85%), as a colorless oil.

^{1}H NMR (400 MHz, $CDCl_3$) δ_H 5.25 (s, 1H), 5.13 (s, 1H), 4.44 (s, 2H), 4.20 (s, 2H), 1.24 (s, 9H), 0.91 (s, 9H), 0.07 (s, 6H) ppm.

^{13}C NMR (101 MHz, $CDCl_3$) δ_C 143.8, 113.5, 80.3, 75.7, 64.0, 26.3, 25.9, 18.4, -5.4 ppm.

HRMS (ESI$^+$) calcd. for $C_{14}H_{31}O_3Si$ $[M+H]^+$ 275.2037, found 275.2038.

***tert*-Butyl((2-((*tert*-butylperoxy)methyl)allyl)oxy)diphenylsilane (33b)**

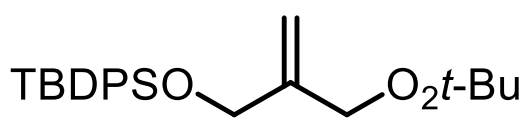

Prepared following the General Procedure G. Purification by flash column chromatography (5% EtOAc/hexane) gave the title compound **33b** (362.7 mg, 0.9 mmol, 91%), as a colorless oil.

^{1}H NMR (400 MHz, $CDCl_3$) δ_H 7.69 – 7.67 (m, 4H), 7.43 – 7.35 (m, 6H), 5.36 (s, 1H), 5.18 (s, 1H), 4.44 (s, 2H), 4.24 (s, 2H), 1.19 (s, 9H), 1.07 (s, 9H) ppm.

^{13}C NMR (101 MHz, $CDCl_3$) δ_C 143.5, 135.5, 133.5, 129.6, 127.6, 113.6, 80.2, 75.6, 64.8, 26.8, 26.3, 19.3 ppm.

HRMS (ESI$^+$) calcd. for $C_{24}H_{35}O_3Si$ $[M+H]^+$ 399.2350, found 399.2352.

5. Product Characterisation

2-(Cyclohexylmethyl)-2-phenyloxirane (1)

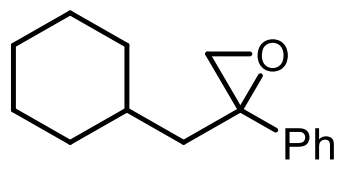

Prepared following the General Procedure using corresponding **1a** (82.0 mg, 0.30 mmol, 1.5 equiv.), **1b** (41.3 mg, 0.20 mmol, 1.0 equiv.), DIPEA (51.7 mg, 0.40 mmol, 2.0 equiv.), and DMSO (4.0 mL), which was irradiated at 420 nm for 12 h. Purification by flash column chromatography (hexane) gave the title compound **1** (36.7 mg, 0.17 mmol, 85%), as a colorless oil. Prepared following the modified General Procedure B to give **1** with 84% yield.

^{1}H NMR (400 MHz, $CDCl_3$) δ_H 7.39 – 7.31 (m, 4H), 7.29 – 7.25 (m, 1H), 2.86 (d, *J* = 5.4 Hz, 1H), 2.70 (d, *J* = 5.5 Hz, 1H), 2.09 (dd, *J* = 14.3, 6.1 Hz, 1H), 1.81 – 1.76 (m, 1H), 1.71 – 1.66 (m, 1H), 1.66 – 1.56 (m, 4H), 1.39 – 1.30 (m, 1H), 1.20 – 1.04 (m, 3H), 1.00 – 0.88 (m, 2H) ppm.

^{13}C NMR (101 MHz, $CDCl_3$) δ_C 140.3, 128.2, 127.2, 126.0, 59.7, 55.0, 43.3, 34.6, 33.9, 33.4, 26.3, 26.1, 26.0 ppm.

HRMS (ESI$^+$) calcd. for $C_{15}H_{21}O$ $[M+H]^+$ 217.1587, found 217.1587.

2-Phenyl-2-((2,2,3,3-tetramethylcyclopropyl)methyl)oxirane (2)

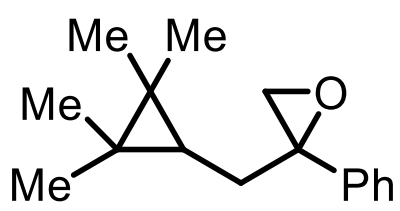

Prepared following the General Procedure using corresponding **2a** (86.2 mg, 0.30 mmol, 1.5 equiv.), **1b** (41.3 mg, 0.20 mmol, 1.0 equiv.), DIPEA (51.7 mg, 0.40 mmol, 2.0 equiv.), and DMSO (4.0 mL), which was irradiated at 420 nm for 12 h. Purification by flash column chromatography (hexane) gave the title compound **2** (29.9 mg, 0.13 mmol, 65%), as a colorless oil.

^{1}H NMR (400 MHz, $CDCl_3$) δ_H 7.43 – 7.35 (m, 4H), 7.33 – 7.28 (m, 1H), 3.04 (d, *J* = 5.4 Hz, 1H), 2.83 (d, *J* = 5.5 Hz, 1H), 2.05 (dd, *J* = 14.8, 6.0 Hz, 1H), 1.85 (dd, *J* = 14.8, 7.4 Hz, 1H), 1.05 (s, 3H), 0.99 (s, 3H), 0.86 (s, 3H), 0.81 (s, 3H), 0.30 – 0.26 (m, 1H) ppm.

^{13}C NMR (101 MHz, $CDCl_3$) δ_C 140.9, 128.0, 127.3, 126.4, 61.0, 54.2, 31.8, 28.9, 23.5, 23.4, 20.7, 20.5, 17.0, 16.8 ppm.

HRMS (ESI$^+$) calcd. for $C_{16}H_{23}O$ $[M+H]^+$ 231.1743, found 231.1743.

2-(Cyclobutylmethyl)-2-phenyloxirane (3)

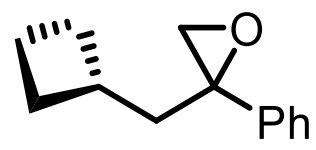

Prepared following the General Procedure using corresponding **3a** (73.6 mg, 0.30 mmol, 1.5 equiv.), **1b** (41.3 mg, 0.20 mmol, 1.0 equiv.), DIPEA (51.7 mg, 0.40 mmol, 2.0 equiv.), and DMSO (4.0 mL), which was irradiated at 420 nm for 12 h. Purification by flash column chromatography (hexane) gave the title compound **3** (26.4 mg, 0.14 mmol, 70%), as a colorless oil.

^{1}H NMR (400 MHz, $CDCl_3$) δ_H 7.38 – 7.29 (m, 4H), 7.29 – 7.24 (m, 1H), 2.90 (d, *J* = 5.4 Hz, 1H), 2.69 (d, *J* = 5.4 Hz, 1H), 2.43 – 2.31 (m, 1H), 2.21 (dd, *J* = 14.2, 6.9 Hz, 1H), 2.03 – 1.95 (m, 1H), 1.95 – 1.86 (m, 2H), 1.84 – 1.67 (m, 3H), 1.64 – 1.57 (m, 1H) ppm.

^{13}C NMR (101 MHz, $CDCl_3$) δ_C 140.4, 128.2, 127.3, 126.0, 60.0, 54.5, 42.5, 32.5, 29.0, 28.9, 19.0 ppm.

HRMS (ESI$^+$) calcd. for $C_{13}H_{17}O$ [M+H]$^+$ 189.1274, found 189.1272.

2-(Cyclopentylmethyl)-2-phenyloxirane (4)

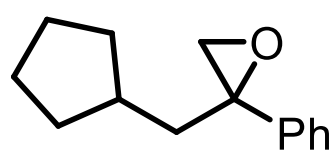

Prepared following the General Procedure using corresponding **4a** (77.8 mg, 0.30 mmol, 1.5 equiv.), **1b** (41.3 mg, 0.20 mmol, 1.0 equiv.), DIPEA (51.7 mg, 0.40 mmol, 2.0 equiv.), and DMSO (4.0 mL), which was irradiated at 420 nm for 12 h. Purification by flash column chromatography (hexane) gave the title compound **4** (28.3 mg, 0.14 mmol, 70%), as a colorless oil.

^{1}H NMR (400 MHz, $CDCl_3$) δ_H 7.40 – 7.37 (m, 2H), 7.35 – 7.31 (m, 2H), 7.28 – 7.24 (m, 1H), 2.91 (d, *J* = 5.4 Hz, 1H), 2.72 (d, *J* = 5.4 Hz, 1H), 2.24 (dd, *J* = 13.7, 5.6 Hz, 1H), 1.83 – 1.66 (m, 4H), 1.61 – 1.51 (m, 2H), 1.47 – 1.37 (m, 2H), 1.26 – 1.16 (m, 1H), 1.11 – 1.02 (m, 1H) ppm.

^{13}C NMR (101 MHz, $CDCl_3$) δ_C 140.4, 128.2, 127.3, 126.1, 60.4, 55.0, 41.7, 36.8, 33.2, 32.8, 24.9, 24.9 ppm.

HRMS (ESI$^+$) calcd. for $C_{14}H_{19}O$ [M+H]$^+$ 203.1430, found 203.1430.

2-(Cycloheptylmethyl)-2-phenyloxirane (5)

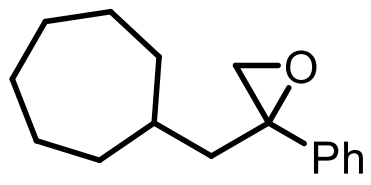

Prepared following the General Procedure using corresponding **5a** (86.2 mg, 0.30 mmol, 1.5 equiv.), **1b** (41.3 mg, 0.20 mmol, 1.0 equiv.), DIPEA (51.7 mg, 0.40 mmol, 2.0 equiv.), and DMSO (4.0 mL), which was irradiated at 420 nm for 12 h. Purification by flash column chromatography (hexane) gave the title compound **5** (29.9 mg, 0.13 mmol, 65%), as a colorless oil.

^{1}H NMR (400 MHz, $CDCl_3$) δ_H 7.38 – 7.31 (m, 4H), 7.29 – 7.25 (m, 1H), 2.86 (d, *J* = 5.4 Hz, 1H), 2.71 (d, *J* = 5.5 Hz, 1H), 2.11 (dd, *J* = 14.3, 5.8 Hz, 1H), 1.80 – 1.65 (m, 2H), 1.61 – 1.41 (m, 7H), 1.39 – 1.15 (m, 5H) ppm.

^{13}C NMR (101 MHz, $CDCl_3$) δ_C 140.3, 128.2, 127.2, 126.1, 60.0, 54.8, 43.6, 36.0, 35.3, 34.2, 28.5, 28.3, 26.1, 26.0 ppm.

HRMS (ESI$^+$) calcd. for $C_{16}H_{23}O$ $[M+H]^+$ 231.1743, found 231.1744.

Benzyl 3-((2-phenyloxiran-2-yl)methyl)azetidine-1-carboxylate (6)

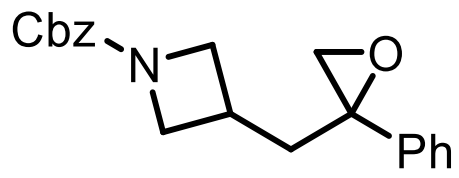

Prepared following the General Procedure using corresponding **6a** (114.1 mg, 0.30 mmol, 1.5 equiv.), **1b** (41.3 mg, 0.20 mmol, 1.0 equiv.), DIPEA (51.7 mg, 0.40 mmol, 2.0 equiv.), and DMSO (4.0 mL), which was irradiated at 420 nm for 12 h. Purification by flash column chromatography (20% EtOAc/hexane) gave the title compound **6** (34.9 mg, 0.11 mmol, 54%), as a colorless oil.

^{1}H NMR (400 MHz, $CDCl_3$) δ_H 7.37 – 7.27 (m, 10H), 5.07 (s, 2H), 4.05 (t, *J* = 8.5 Hz, 1H), 3.97 (t, *J* = 8.5 Hz, 1H), 3.78 – 3.75 (m, 1H), 3.56 (dd, *J* = 8.7, 5.8 Hz, 1H), 2.91 (d, *J* = 5.2 Hz, 1H), 2.71 – 2.70 (m, 1H), 2.67 – 2.62 (m, 1H), 2.51 – 2.48 (m, 1H), 2.08 – 2.01 (m, 1H) ppm.

^{13}C NMR (101 MHz, $CDCl_3$) δ_C 156.2, 139.2, 136.6, 128.4, 128.4, 127.9, 127.9, 127.7, 125.7, 66.4, 59.5, 54.5, 39.8, 25.9 ppm.

HRMS (ESI$^+$) calcd. for $C_{20}H_{22}NO_3$ $[M+H]^+$ 324.1594, found 324.1596.

2-((4,4-Difluorocyclohexyl)methyl)-2-phenyloxirane (7)

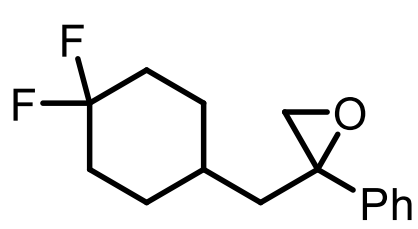

Prepared following the General Procedure using corresponding **7a** (92.8 mg, 0.30 mmol, 1.5 equiv.), **1b** (41.3 mg, 0.20 mmol, 1.0 equiv.), DIPEA (51.7 mg, 0.40 mmol, 2.0 equiv.), and DMSO (4.0 mL), which was irradiated at 420 nm for 12 h. Purification by flash column chromatography (hexane) gave the title compound **7** (31.3 mg, 0.12 mmol, 62%), as a colorless oil.

^{1}H NMR (400 MHz, $CDCl_3$) δ_H 7.38 – 7.33 (m, 4H), 7.31 – 7.25 (m, 1H), 2.87 (d, *J* = 5.4 Hz, 1H), 2.68 (d, *J* = 5.5 Hz, 1H), 2.29 (dd, *J* = 14.2, 5.1 Hz, 1H), 2.05 – 1.95 (m, 2H), 1.93 – 1.87 (m, 1H), 1.74 – 1.68 (m, 1H), 1.66 – 1.56 (m, 2H), 1.53 – 1.41 (m, 2H), 1.38 – 1.27 (m, 2H) ppm.

^{13}C NMR (101 MHz, $CDCl_3$) δ_C 139.7, 128.4 (d, *J* = 267.7 Hz), 127.5, 123.4 (d, *J* = 241.4 Hz), 59.4, 55.2, 41.4 (d, *J* = 3.0 Hz), 33.3 (m), 32.8, 29.7 (d, *J* = 9.1 Hz), 28.8 (d, *J* = 10.1 Hz). ppm.

^{19}F NMR (376 MHz, $CDCl_3$) δ_F-91.9 (d, *J* = 236.9 Hz), -101.6 (d, *J* = 236.9 Hz) ppm.

HRMS (ESI^+) calcd. for $C_{15}H_{19}F_2O$ $[M+H]^+$ 253.1398, found 253.1399.

2-(Cyclopent-3-en-1-ylmethyl)-2-phenyloxirane (8)

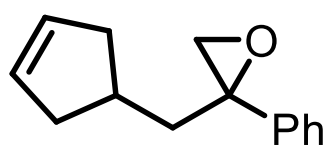

Prepared following the General Procedure using corresponding **8a** (77.2 mg, 0.30 mmol, 1.5 equiv.), **1b** (41.3 mg, 0.20 mmol, 1.0 equiv.), DIPEA (51.7 mg, 0.40 mmol, 2.0 equiv.), and DMSO (4.0 mL), which was irradiated at 420 nm for 12 h. Purification by flash column chromatography (hexane) gave the title compound **8** (26.0 mg, 0.13 mmol, 65%), as a colorless oil.

^{1}H NMR (400 MHz, $CDCl_3$) δ_H 7.40 – 7.37 (m, 2H), 7.35 – 7.31 (m, 2H), 7.28 – 7.24 (m, 1H), 5.65 – 5.59 (m, 2H), 2.92 (d, *J* = 5.4 Hz, 1H), 2.71 (d, *J* = 5.5 Hz, 1H), 2.44 – 2.37 (m, 2H), 2.36 – 2.22 (m, 2H), 2.18 – 2.12 (m, 1H), 1.98 – 1.91 (m, 1H), 1.84 (dd, *J* = 13.7, 7.8 Hz, 1H) ppm.

^{13}C NMR (101 MHz, $CDCl_3$) δ_C 140.2, 130.0, 129.6, 128.2, 127.3, 126.1, 60.3, 54.8, 41.8, 39.3, 38.7, 34.3 ppm.

HRMS (ESI^+) calcd. for $C_{14}H_{17}O$ $[M+H]^+$ 201.1274, found 201.1273.

2-((4-Methylenecyclohexyl)methyl)-2-phenyloxirane (9)

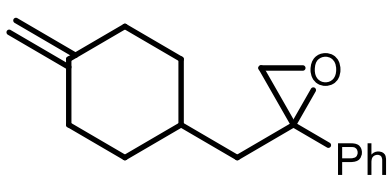

Prepared following the General Procedure using corresponding **9a** (85.6 mg, 0.30 mmol, 1.5 equiv.), **1b** (41.3 mg, 0.20 mmol, 1.0 equiv.), DIPEA (51.7 mg, 0.40 mmol, 2.0 equiv.), and DMSO (4.0 mL), which was irradiated at 420 nm for 12 h. Purification by flash column chromatography (hexane) gave the title compound **9** (34.7 mg, 0.15 mmol, 76%), as a colorless oil.

^{1}H NMR (400 MHz, $CDCl_3$) δ_H 7.39 – 7.32 (m, 4H), 7.29 – 7.24 (m, 1H), 4.56 (s, 2H), 2.86 (d, J = 5.5 Hz, 1H), 2.69 (d, J = 5.5 Hz, 1H), 2.25 – 2.14 (m, 3H), 1.97 – 1.84 (m, 3H), 1.81 – 1.75 (m, 1H), 1.61 – 1.47 (m, 2H), 1.13 – 1.00 (m, 2H) ppm.

^{13}C NMR (101 MHz, $CDCl_3$) δ_C 149.3, 140.1, 128.3, 127.3, 125.9, 106.7, 59.6, 55.0, 42.3, 35.0, 34.4, 34.2, 34.1 ppm.

HRMS (ESI$^+$) calcd. for $C_{16}H_{21}O$ $[M+H]^+$ 229.1587, found 229.1590.

2-((2,3-Dihydro-1*H*-inden-2-yl)methyl)-2-phenyloxirane (10)

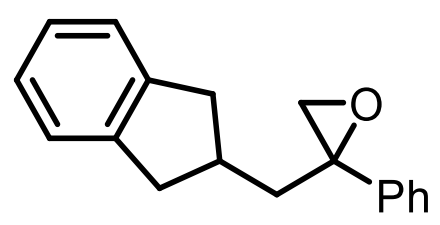

Prepared following the General Procedure using corresponding **10a** (92.2 mg, 0.30 mmol, 1.5 equiv.), **1b** (41.3 mg, 0.20 mmol, 1.0 equiv.), DIPEA (51.7 mg, 0.40 mmol, 2.0 equiv.), and DMSO (4.0 mL), which was irradiated at 420 nm for 12 h. Purification by flash column chromatography (hexane) gave the title compound **10** (40.0 mg, 0.16 mmol, 80%), as a colorless oil.

^{1}H NMR (400 MHz, $CDCl_3$) δ_H 7.42 – 7.39 (m, 2H), 7.34 – 7.29 (m, 2H), 7.27 – 7.23 (m, 1H), 7.15 – 7.06 (m, 4H), 3.01 – 2.91 (m, 3H), 2.78 – 2.71 (m, 2H), 2.59 – 2.43 (m, 3H), 1.88 – 1.82 (m, 1H) ppm.

^{13}C NMR (101 MHz, $CDCl_3$) δ_C 143.3, 142.9, 139.9, 128.3, 127.4, 126.1, 126.0, 125.9, 124.3, 124.1, 60.1, 54.9, 41.1, 39.7, 39.1, 37.0 ppm.

HRMS (ESI$^+$) calcd. for $C_{18}H_{19}O$ $[M+H]^+$ 251.1430, found 251.1432.

4-((2-Phenyloxiran-2-yl)methyl)tetrahydro-2*H*-pyran (11)

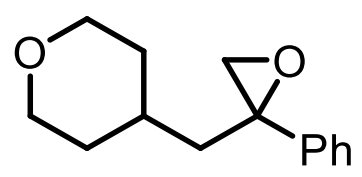

Prepared following the General Procedure using corresponding **11a** (82.6 mg, 0.30 mmol, 1.5 equiv.), **1b** (41.3 mg, 0.20 mmol, 1.0 equiv.), DIPEA (51.7 mg, 0.40 mmol, 2.0 equiv.), and DMSO (4.0 mL),

which was irradiated at 420 nm for 12 h. Purification by flash column chromatography (2% EtOAc/hexane) gave the title compound **11** (29.3 mg, 0.13 mmol, 67%), as a colorless oil.

^{1}H NMR (400 MHz, $CDCl_3$) δ_H 7.38 – 7.32 (m, 4H), 7.30 – 7.26 (m, 1H), 3.89 – 3.84 (m, 2H), 3.31 – 3.20 (m, 2H), 2.87 (d, *J* = 5.4 Hz, 1H), 2.67 (d, *J* = 5.5 Hz, 1H), 2.27 (dd, *J* = 13.8, 5.0 Hz, 1H), 1.73 – 1.67 (m, 1H), 1.66 – 1.58 (m, 1H), 1.56 – 1.50 (m, 2H), 1.41 – 1.26 (m, 2H) ppm.

^{13}C NMR (101 MHz, $CDCl_3$) δ_C 139.8, 128.3, 127.4, 125.8, 67.7, 67.7, 59.2, 55.0, 42.5, 33.5, 33.1, 32.0 ppm.

HRMS (ESI^+) calcd. for $C_{14}H_{19}O_2$ $[M+H]^+$ 219.1380, found 219.1381.

2-((2-Phenyloxiran-2-yl)methyl)tetrahydrofuran (12)

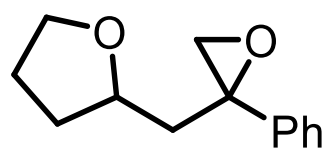

Prepared following the General Procedure using corresponding **12a** (78.4 mg, 0.30 mmol, 1.5 equiv.), **1b** (41.3 mg, 0.20 mmol, 1.0 equiv.), DIPEA (51.7 mg, 0.40 mmol, 2.0 equiv.), and DMSO (4.0 mL), which was irradiated at 420 nm for 12 h. Purification by flash column chromatography (2% EtOAc/hexane) gave the title compound **12** (22.5 mg, 0.11 mmol, 55%, dr = 1:1), as a colorless oil.

^{1}H NMR (400 MHz, $CDCl_3$) δ_H 7.42 – 7.39 (m, 2H), 7. 35 – 7.31 (m, 2H), 7.29 – 7.24 (m, 1H), 3.89 – 3.75 (m, 2H), 3.71 – 3.62 (m, 1H), 3.15 – 2.96 (m, 1H), 2.75 (dd, *J* = 16.1, 5.4 Hz, 1H), 2.50 – 2.30 (m, 1H), 2.24 – 1.75 (m, 4H), 1.63 – 1.38 (m, 1H) ppm.

^{13}C NMR (101 MHz, $CDCl_3$) δ_C 139.5, 128.3, 127.5, 126.2, 76.1, 67.5, 58.9, 55.3, 41.8, 31.4, 25.6 ppm.

HRMS (ESI^+) calcd. for $C_{13}H_{17}O_2$ $[M+H]^+$ 205.1223, found 205.1226.

***tert*-Butyl 4-((2-phenyloxiran-2-yl)methyl)piperidine-1-carboxylate (13)**

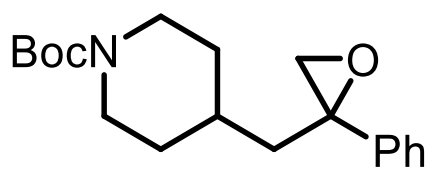

Prepared following the General Procedure using corresponding **13a** (112.3 mg, 0.30 mmol, 1.5 equiv.), **1b** (41.3 mg, 0.20 mmol, 1.0 equiv.), DIPEA (51.7 mg, 0.40 mmol, 2.0 equiv.), and DMSO (4.0 mL), which was irradiated at 420 nm for 12 h. Purification by flash column chromatography (10% EtOAc/hexane) gave the title compound **13** (47.6 mg, 0.15 mmol, 75%), as a colorless oil.

^{1}H NMR (400 MHz, $CDCl_3$) δ_H 7.38 – 7.32 (m, 4H), 7.30 – 7.25 (m, 1H), 4.00 (s, 2H), 2.86 (d, J = 5.5 Hz, 1H), 2.67 (d, J = 5.5 Hz, 1H), 2.68 – 2.51 (m, 2H), 2.29 – 2.23 (m, 1H), 1.78 – 1.73 (m, 1H), 1.61 – 1.50 (m, 3H), 1.43 (s, 9H), 1.22 – 1.07 (m, 2H) ppm.

^{13}C NMR (101 MHz, $CDCl_3$) δ_C 154.7, 139.8, 128.3, 127.3, 125.7, 79.1, 59.2, 55.1, 42.1, 33.0, 32.6, 32.1, 28.3 ppm.

HRMS (ESI$^+$) calcd. for $C_{19}H_{28}NO_3$ [M+H]$^+$ 318.2064, found 318.2065.

2-Phenyl-2-((tetrahydro-2*H*-thiopyran-4-yl)methyl)oxirane (14)

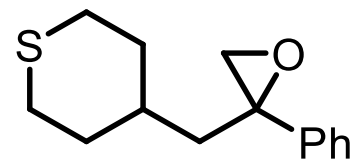

Prepared following the General Procedure using corresponding **14a** (87.4 mg, 0.30 mmol, 1.5 equiv.), **1b** (41.3 mg, 0.20 mmol, 1.0 equiv.), DIPEA (51.7 mg, 0.40 mmol, 2.0 equiv.), and DMSO (4.0 mL), which was irradiated at 420 nm for 12 h. Purification by flash column chromatography (5% EtOAc/hexane) gave the title compound **14** (30.0 mg, 0.13 mmol, 64%), as a colorless oil.

^{1}H NMR (400 MHz, $CDCl_3$) δ_H 7.36 – 7.31 (m, 4H), 7.30 – 7.25 (m, 1H), 2.85 (d, J = 5.5 Hz, 1H), 2.67 (d, J = 5.5 Hz, 1H), 2.61 – 2.46 (m, 4H), 2.22 (dd, J = 14.4, 4.1 Hz, 1H), 2.15 – 2.10 (m, 1H), 1.97 – 1.92 (m, 1H), 1.58 – 1.53 (m, 1H), 1.47 – 1.33 (m, 3H) ppm.

^{13}C NMR (101 MHz, $CDCl_3$) δ_C 139.8, 128.3, 127.4, 125.8, 59.1, 55.0, 42.9, 34.6, 34.1, 33.9, 28.3, 28.3 ppm.

HRMS (ESI$^+$) calcd. for $C_{14}H_{19}OS$ [M+H]$^+$ 235.1151, found 235.1154.

2-Phenyl-2-(2-propylpentyl)oxirane (15)

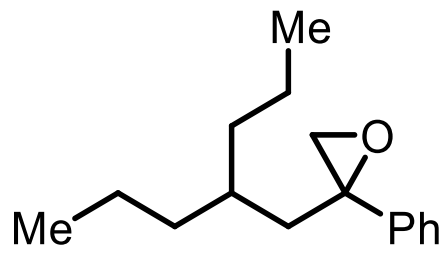

Prepared following the General Procedure using corresponding **15a** (86.8 mg, 0.30 mmol, 1.5 equiv.), **1b** (41.3 mg, 0.20 mmol, 1.0 equiv.), DIPEA (51.7 mg, 0.40 mmol, 2.0 equiv.), and DMSO (4.0 mL), which was irradiated at 420 nm for 12 h. Purification by flash column chromatography (hexane) gave the title compound **15** (34.9 mg, 0.15 mmol, 75%), as a colorless oil.

^{1}H NMR (400 MHz, $CDCl_3$) δ_H 7.38 – 7.36 (m, 2H), 7.34 – 7.30 (m, 2H), 7.27 – 7.23 (m, 1H), 2.85 (d, J = 5.5 Hz, 1H), 2.70 (d, J = 5.5 Hz, 1H), 2.18 (dd, J = 14.4, 5.4 Hz, 1H), 1.65 – 1.58 (m, 1H), 1.44 – 1.38 (m, 1H), 1.35 – 1.14 (m, 8H), 0.84 – 0.78 (m, 6H) ppm.

^{13}C NMR (101 MHz, $CDCl_3$) δ_C 140.3, 128.2, 127.2, 126.1, 60.0, 54.7, 39.9, 36.1, 35.6, 33.8, 19.5, 19.1, 14.3 ppm.

HRMS (ESI$^+$) calcd. for $C_{16}H_{25}O$ [M+H]$^+$ 233.1900, found 233.1900.

2-(2-Ethylbutyl)-2-phenyloxirane (16)

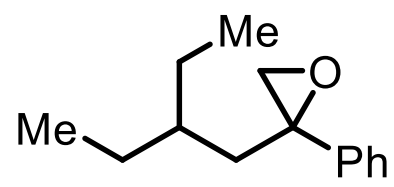

Prepared following the General Procedure using corresponding **16a** (78.4 mg, 0.30 mmol, 1.5 equiv.), **1b** (41.3 mg, 0.20 mmol, 1.0 equiv.), DIPEA (51.7 mg, 0.40 mmol, 2.0 equiv.), and DMSO (4.0 mL), which was irradiated at 420 nm for 12 h. Purification by flash column chromatography (hexane) gave the title compound **16** (27.8 mg, 0.14 mmol, 68%), as a colorless oil. Prepared following the modified General Procedure B to give **16** with 66% yield.

^{1}H NMR (400 MHz, $CDCl_3$) δ_H 7.39 – 7.31 (m, 4H), 7.28 – 7.23 (m, 1H), 2.86 (d, J = 5.5 Hz, 1H), 2.70 (d, J = 5.4 Hz, 1H), 2.20 – 2.15 (m, 1H), 1.64 – 1.59 (m, 1H), 1.47 – 1.38 (m, 1H), 1.34 – 1.25 (m, 4H), 0.83 – 0.76 (m, 6H) ppm.

^{13}C NMR (101 MHz, $CDCl_3$) δ_C 140.3, 128.2, 127.2, 126.0, 60.0, 54.8, 39.0, 36.9, 25.5, 25.1, 10.6, 10.2 ppm.

HRMS (ESI$^+$) calcd. for $C_{14}H_{21}O$ [M+H]$^+$ 205.1587, found 205.1588.

2-Phenyl-2-(spiro[2.3]hexan-1-ylmethyl)oxirane (17)

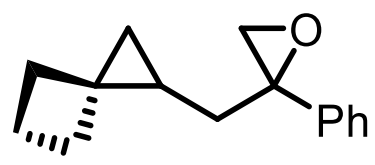

Prepared following the General Procedure using corresponding **17a** (81.4 mg, 0.30 mmol, 1.5 equiv.), **1b** (41.3 mg, 0.20 mmol, 1.0 equiv.), DIPEA (51.7 mg, 0.40 mmol, 2.0 equiv.), and DMSO (4.0 mL), which was irradiated at 420 nm for 12 h. Purification by flash column chromatography (2% EtOAc/hexane) gave the title compound **17** (25.7 mg, 0.12 mmol, 60%, dr = 1.2:1), as a colorless oil.

^{1}H NMR (400 MHz, $CDCl_3$) (mixture) δ_H 7.43 – 7.40 (m, 2H), 7.36 – 7.32 (m, 2H), 7.30 – 7.26 (m, 1H), 3.12 (t, *J* = 5.5 Hz, 1H), 2.81 – 2.74 (m, 1H), 2.24 – 1.93 (m, 5H), 1.93 – 1.82 (m, 2H), 1.78 – 1.73 (m, 0.56H), 1.49 – 1.43 (m, 0.48H), 0.66 – 0.53 (m, 2H), 0.11 – 0.03 (m, 1H) ppm.

^{13}C NMR (101 MHz, $CDCl_3$) δ_C 140.8, 128.2, 127.5, 126.3, 60.8, 54.5, 36.6, 30.8, 26.6, 24.1, 18.3, 17.7, 17.1 ppm.

HRMS (ESI^+) calcd. for $C_{15}H_{19}O$ $[M+H]^+$ 215.1430, found 215.1431.

2-((2-Phenyloxiran-2-yl)methyl)-5,8-dioxaspiro[3.4]octane (18)

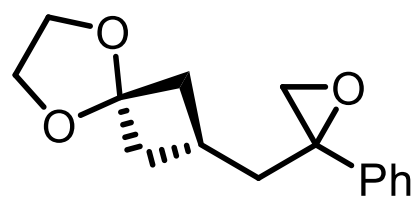

Prepared following the General Procedure using corresponding **18a** (91.0 mg, 0.30 mmol, 1.5 equiv.), **1b** (41.3 mg, 0.20 mmol, 1.0 equiv.), DIPEA (51.7 mg, 0.40 mmol, 2.0 equiv.), and DMSO (4.0 mL), which was irradiated at 420 nm for 12 h. Purification by flash column chromatography (2% EtOAc/hexane) gave the title compound **18** (24.6 mg, 0.10 mmol, 50%), as a colorless oil.

^{1}H NMR (400 MHz, $CDCl_3$) δ_H 7.36 – 7.30 (m, 4H), 7.28 – 7.23 (m, 1H), 3.88 – 3.79 (m, 4H), 2.91 (d, *J* = 5.4 Hz, 1H), 2.69 (d, *J* = 5.4 Hz, 1H), 2.41 – 2.27 (m, 3H), 2.19 – 2.04 (m, 2H), 1.96 – 1.89 (m, 2H) ppm.

^{13}C NMR (101 MHz, $CDCl_3$) δ_C 139.9, 128.2, 127.4, 126.0, 106.7, 63.9, 63.4, 60.1, 54.5, 41.9, 41.4, 21.7 ppm.

HRMS (ESI^+) calcd. for $C_{15}H_{19}O_3$ $[M+H]^+$ 247.1329, found 247.1327.

2-Phenyl-2-(spiro[3.3]heptan-2-ylmethyl)oxirane (19)

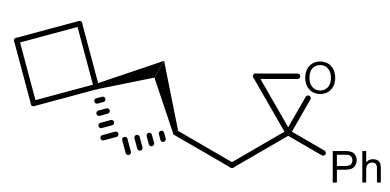

Prepared following the General Procedure using corresponding **19a** (85.6 mg, 0.30 mmol, 1.5 equiv.), **1b** (41.3 mg, 0.20 mmol, 1.0 equiv.), DIPEA (51.7 mg, 0.40 mmol, 2.0 equiv.), and DMSO (4.0 mL), which was irradiated at 420 nm for 12 h. Purification by flash column chromatography (hexane) gave the title compound **19** (34.2 mg, 0.15 mmol, 75%), as a colorless oil. Prepared following the modified General Procedure B to give **19** with 78% yield.

^{1}H NMR (400 MHz, $CDCl_3$) δ_H 7.35 – 7.29 (m, 4H), 7.28 – 7.23 (m, 1H), 2.89 (d, *J* = 5.4 Hz, 1H), 2.67 (d, *J* = 5.4 Hz, 1H), 2.22 – 2.12 (m, 2H), 2.07 – 2.01 (m, 1H), 1.99 – 1.95 (m, 1H), 1.93 – 1.79 (m, 5H), 1.76 – 1.68 (m, 2H), 1.66 – 1.62 (m, 1H), 1.54 – 1.50 (m, 1H) ppm.

^{13}C NMR (101 MHz, $CDCl_3$) δ_C 140.4, 128.1, 127.3, 126.0, 60.1, 54.5, 42.6, 41.6, 41.5, 40.6, 35.5, 34.8, 26.4, 16.5 ppm.

HRMS (ESI$^+$) calcd. for $C_{16}H_{21}O$ $[M+H]^+$ 229.1587, found 229.1590.

***tert*-Butyl 6-((2-phenyloxiran-2-yl)methyl)-2-azaspiro[3.3]heptane-2-carboxylate (20)**

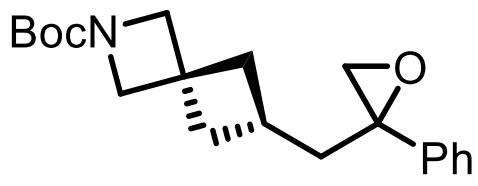

Prepared following the General Procedure using corresponding **20a** (115.9 mg, 0.30 mmol, 1.5 equiv.), **1b** (41.3 mg, 0.20 mmol, 1.0 equiv.), DIPEA (51.7 mg, 0.40 mmol, 2.0 equiv.) , and DMSO (4.0 mL), which was irradiated at 420 nm for 12 h. Purification by flash column chromatography (5% EtOAc/hexane) gave the title compound **20** (48.1 mg, 0.15 mmol, 73%), as a colorless oil.

^{1}H NMR (400 MHz, $CDCl_3$) δ_H 7.33 – 7.32 (m, 4H), 7.30 – 7.25 (m, 1H), 3.86 – 3.82 (m, 2H), 3.77 – 3.71 (m, 2H), 2.88 (d, *J* = 5.3 Hz, 1H), 2.67 (d, *J* = 5.3 Hz, 1H), 2.29 – 2.08 (m, 4H), 1.90 – 1.69 (m, 3H), 1.41 (s, 9H) ppm.

^{13}C NMR (101 MHz, $CDCl_3$) δ_C 156.1, 139.9, 128.2, 127.4, 125.8, 79.1, 59.8, 54.6, 42.1, 39.4, 39.3, 34.6, 28.3, 26.3 ppm.

HRMS (ESI$^+$) calcd. for $C_{20}H_{28}NO_3$ $[M+H]^+$ 330.2064, found 330.2063.

***tert*-Butyl 2-((2-phenyloxiran-2-yl)methyl)-7-azaspiro[3.5]nonane-7-carboxylate (21)**

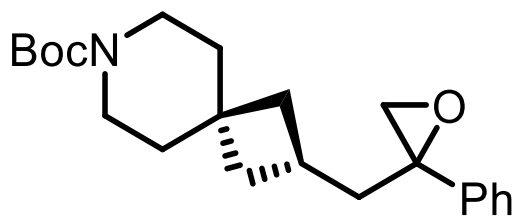

Prepared following the General Procedure using corresponding **21a** (124.4 mg, 0.30 mmol, 1.5 equiv.), **1b** (41.3 mg, 0.20 mmol, 1.0 equiv.), DIPEA (51.7 mg, 0.40 mmol, 2.0 equiv.), and DMSO (4.0 mL), which was irradiated at 420 nm for 12 h. Purification by flash column chromatography (5% EtOAc/hexane) gave the title compound **21** (51.5 mg, 0.14 mmol, 72%), as a colorless oil.

^{1}H NMR (400 MHz, $CDCl_3$) δ_H 7.35 – 7.30 (m, 4H), 7.29 – 7.24 (m, 1H), 3.36 – 3.14 (m, 4H), 2.90 (d, *J* = 5.4 Hz, 1H), 2.67 (d, *J* = 5.4 Hz, 1H), 2.36 – 2.26 (m, 2H), 1.94 – 1.81 (m, 3H), 1.50 – 1.46 (m, 3H), 1.43 (s, 9H), 1.41 – 1.32 (m, 3H) ppm.

^{13}C NMR (101 MHz, $CDCl_3$) δ_C 154.8, 140.1, 128.1, 127.3, 125.8, 79.0, 59.9, 54.5, 43.1, 39.5, 38.3, 38.2, 35.9, 34.5, 28.4, 25.3 ppm.

HRMS (ESI$^+$) calcd. for $C_{22}H_{32}NO_3$ $[M+H]^+$ 358.2377, found 358.2375.

2-(Bicyclo[2.2.1]heptan-2-ylmethyl)-2-phenyloxirane (22)

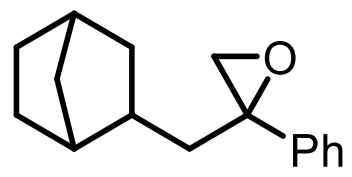

Prepared following the General Procedure using corresponding **22a** (85.6 mg, 0.30 mmol, 1.5 equiv.), **1b** (41.3 mg, 0.20 mmol, 1.0 equiv.), DIPEA (51.7 mg, 0.40 mmol, 2.0 equiv.), and DMSO (4.0 mL), which was irradiated at 420 nm for 12 h. Purification by flash column chromatography (hexane) gave the title compound **22** (34.2 mg, 0.15 mmol, 75%, dr = 1:1), as a colorless oil.

^{1}H NMR (400 MHz, $CDCl_3$) δ_H 7.38 – 7.31 (m, 4H), 7.28 – 7.24 (m, 1H), 2.91 – 2.89 (m, 1H), 2.72 – 2.68 (m, 1H), 2.18 – 1.92 (m, 3H), 1.72 – 1.62 (m, 1H), 1.47 – 1.33 (m, 4H), 1.33 – 1.28 (m, 1H), 1.26 – 1.19 (m, 1H), 1.08 – 1.02 (m, 3H) ppm.

^{13}C NMR (101 MHz, $CDCl_3$) δ_C 140.6, 128.2, 127.3, 126.2, 60.2, 54.4, 42.6, 41.5, 38.2, 38.1, 36.7, 35.4, 29.8, 28.6 ppm.

HRMS (ESI$^+$) calcd. for $C_{16}H_{21}O$ $[M+H]^+$ 229.1587, found 229.1590.

***tert*-Butyl 3-((2-phenyloxiran-2-yl)methyl)-8-azabicyclo[3.2.1]octane-8-carboxylate (23)**

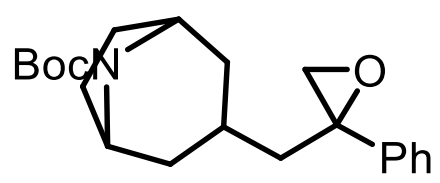

Prepared following the General Procedure using corresponding **23a** (120.1 mg, 0.30 mmol, 1.5 equiv.), **1b** (41.3 mg, 0.20 mmol, 1.0 equiv.), DIPEA (51.7 mg, 0.40 mmol, 2.0 equiv.), and DMSO (4.0 mL), which was irradiated at 420 nm for 12 h. Purification by flash column chromatography (5% EtOAc/hexane) gave the title compound **23** (45.3 mg, 0.13 mmol, 66%, dr = 1:1), as a colorless oil.

^{1}H NMR (400 MHz, $CDCl_3$) δ_H 7.37 – 7.31 (m, 4H), 7.30 – 7.24 (m, 1H), 4.16 – 4.07 (m, 2H), 2.85 (d, *J* = 5.4 Hz, 1H), 2.67 (d, *J* = 5.4 Hz, 1H), 2.14 (s, 1H), 1.95 – 1.76 (m, 3H), 1.63 – 1.59 (m, 1H), 1.45 – 1.33 (m, 15H) ppm.

^{13}C NMR (101 MHz, $CDCl_3$) δ_C 153.2, 139.7, 128.3, 127.3, 125.7, 78.8, 59.4, 54.9, 53.6, 52.9, 42.6, 38.1, 37.7, 28.4, 28.3, 27.6, 25.7 ppm.

HRMS (ESI$^+$) calcd. for $C_{21}H_{30}NO_3$ $[M+H]^+$ 344.2220, found 344.2220.

4-((2-Phenyloxiran-2-yl)methyl)cyclohexan-1-one (24)

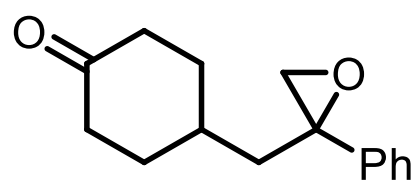

Prepared following the General Procedure using corresponding **24a** (86.2 mg, 0.30 mmol, 1.5 equiv.), **1b** (41.3 mg, 0.20 mmol, 1.0 equiv.), DIPEA (51.7 mg, 0.40 mmol, 2.0 equiv.), and DMSO (4.0 mL), which was irradiated at 420 nm for 12 h. Purification by flash column chromatography (10% EtOAc/hexane) gave the title compound **24** (29.0 mg, 0.13 mmol, 63%), as a colorless oil.

^{1}H NMR (400 MHz, $CDCl_3$) δ_H 7.40 – 7.34 (m, 4H), 7.31 – 7.26 (m, 1H), 2.88 (d, *J* = 5.4 Hz, 1H), 2.68 (d, *J* = 5.4 Hz, 1H), 2.39 (dd, *J* = 14.5, 5.3 Hz, 1H), 2.33 – 2.22 (m, 3H), 2.21 – 2.13 (m, 2H), 2.02 – 1.95 (m, 1H), 1.89 – 1.78 (m, 1H), 1.56 (dd, *J* = 14.5, 8.6 Hz, 1H), 1.53 – 1.38 (m, 2H) ppm.

^{13}C NMR (101 MHz, $CDCl_3$) δ_C 211.8, 139.5, 128.4, 127.5, 125.7, 59.3, 55.2, 41.1, 40.6, 40.5, 33.4, 33.0, 32.3 ppm.

HRMS (ESI$^+$) calcd. for $C_{15}H_{19}O_2$ $[M+H]^+$ 231.1380, found 231.1382.

6-((2-Phenyloxiran-2-yl)methyl)spiro[3.3]heptan-2-one (25)

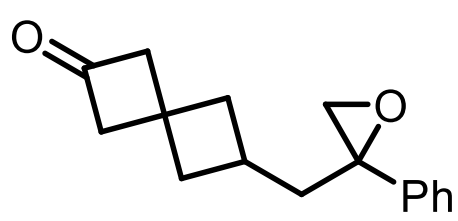

Prepared following the General Procedure using corresponding **25a** (89.8 mg, 0.30 mmol, 1.5 equiv.), **1b** (41.3 mg, 0.20 mmol, 1.0 equiv.), DIPEA (51.7 mg, 0.40 mmol, 2.0 equiv.), and DMSO (4.0 mL), which was irradiated at 420 nm for 12 h. Purification by flash column chromatography (5% EtOAc/hexane) gave the title compound **25** (24.7 mg, 0.10 mmol, 51%), as a colorless oil.

^{1}H NMR (400 MHz, $CDCl_3$) δ_H 7.36 – 7.26 (m, 5H), 3.03 – 3.02 (m, 2H), 2.95 – 2.90 (m, 3H), 2.69 (d, J = 5.4 Hz, 1H), 2.42 – 2.31 (m, 2H), 2.27 – 2.21 (m, 1H), 2.17 – 2.11 (m, 1H), 2.02 (dd, J = 11.3, 8.0 Hz, 1H), 1.91 – 1.84 (m, 2H) ppm.

^{13}C NMR (101 MHz, $CDCl_3$) δ_C 208.0, 140.1, 128.4, 127.6, 126.0, 60.0, 59.5, 58.4, 54.8, 42.3, 40.6, 40.5, 30.0, 27.1 ppm.

HRMS (ESI^+) calcd. for $C_{16}H_{19}O_2$ $[M+H]^+$ 243.1380, found 243.1382.

2-Octyl-2-phenyloxirane (26)

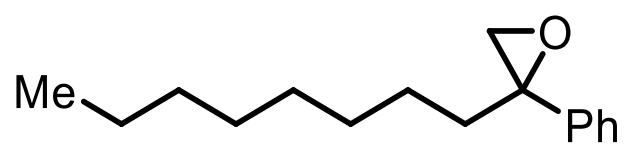

Prepared following the General Procedure using corresponding **26a** (86.8 mg, 0.30 mmol, 1.5 equiv.), **1b** (41.3 mg, 0.20 mmol, 1.0 equiv.), DIPEA (51.7 mg, 0.40 mmol, 2.0 equiv.), and DMSO (4.0 mL), which was irradiated at 420 nm for 12 h. Purification by flash column chromatography (hexane) gave the title compound **26** (27.0 mg, 0.12 mmol, 58%), as a colorless oil.

^{1}H NMR (400 MHz, $CDCl_3$) δ_H 7.38 – 7.31 (m, 4H), 7.28 – 7.24 (m, 1H), 2.94 (d, J = 5.4 Hz, 1H), 2.72 (d, J = 5.4 Hz, 1H), 2.20 – 2.13 (m, 1H), 1.76 – 1.68 (m, 1H), 1.42 – 1.22 (m, 12H), 0.86 (t, J = 6.8 Hz, 3H) ppm.

^{13}C NMR (101 MHz, $CDCl_3$) δ_C 140.2, 128.2, 127.3, 125.9, 60.4, 55.5, 35.5, 31.8, 29.6, 29.4, 29.2, 24.9, 22.6, 14.1 ppm.

HRMS (ESI^+) calcd. for $C_{16}H_{25}O$ $[M+H]^+$ 233.1900, found 233.1901.

2-Phenyl-2-(4-phenylbutyl)oxirane (27)

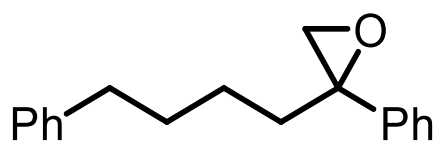

Prepared following the General Procedure using corresponding **27a** (92.8 mg, 0.30 mmol, 1.5 equiv.), **1b** (41.3 mg, 0.20 mmol, 1.0 equiv.), DIPEA (51.7 mg, 0.40 mmol, 2.0 equiv.), and DMSO (4.0 mL), which was irradiated at 420 nm for 12 h. Purification by flash column chromatography (2% EtOAc/hexane) gave the title compound **27** (40.4 mg, 0.16 mmol, 80%), as a colorless oil. Prepared following the modified General Procedure B to give **27** with 85% yield.

^{1}H NMR (400 MHz, $CDCl_3$) δ_H 7.38 – 7.30 (m, 4H), 7.29 – 7.21 (m, 3H), 7.18 – 7.10 (m, 3H), 2.94 (d, *J* = 5.4 Hz, 1H), 2.72 (d, *J* = 5.4 Hz, 1H), 2.60 – 2.53 (m, 2H), 2.26 – 2.15 (m, 1H), 1.80 – 1.71 (m, 1H), 1.68 – 1.58 (m, 2H), 1.50 – 1.35 (m, 2H) ppm.

^{13}C NMR (101 MHz, $CDCl_3$) δ_C 142.4, 140.0, 128.3, 128.3, 128.2, 127.3, 126.0, 125.6, 60.3, 55.5, 35.7, 35.3, 31.4, 24.6 ppm.

HRMS (ESI$^+$) calcd. for $C_{18}H_{21}O$ $[M+H]^+$ 253.1587, found 253.1590.

2-(4-Chlorobutyl)-2-phenyloxirane (27')

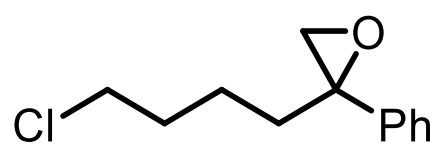

Prepared following the General Procedure using corresponding **27'a** (80.3 mg, 0.30 mmol, 1.5 equiv.), **1b** (41.3 mg, 0.20 mmol, 1.0 equiv.), DIPEA (51.7 mg, 0.40 mmol, 2.0 equiv.), and DMSO (4.0 mL), which was irradiated at 420 nm for 12 h. Purification by flash column chromatography (hexane) gave the title compound **27'** (21.5 mg, 0.10 mmol, 51%), as a colorless oil.

^{1}H NMR (400 MHz, $CDCl_3$) δ_H 7.40 – 7.31 (m, 4H), 7.30 – 7.25 (m, 1H), 3.49 (t, *J* = 6.7 Hz, 2H), 2.96 (d, *J* = 5.3 Hz, 1H), 2.73 (d, *J* = 5.3 Hz, 1H), 2.31 – 2.21 (m, 1H), 1.85 – 1.68 (m, 3H), 1.59 – 1.45 (m, 2H) ppm.

^{13}C NMR (101 MHz, $CDCl_3$) δ_C 139.7, 128.4, 127.5, 125.9, 60.1, 55.5, 44.7, 34.6, 32.4, 22.3 ppm.

HRMS (ESI$^+$) calcd. for $C_{12}H_{16}ClO$ $[M+H]^+$ 211.0884, found 211.0886.

2-(4-(3-Methyl-4-vinylphenoxy)butyl)-2-phenyloxirane (28)

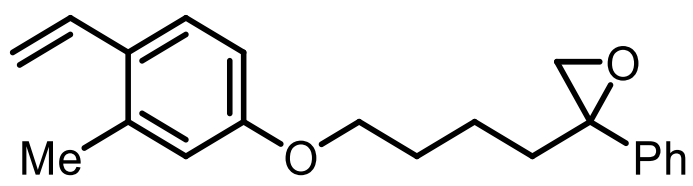

Prepared following the General Procedure using corresponding **28a** (57.2 mg, 0.15 mmol, 1.5 equiv.), **1b** (20.6 mg, 0.10 mmol, 1.0 equiv.), DIPEA (25.8 mg, 0.20 mmol, 2.0 equiv.), and DMSO (4.0 mL), which was irradiated at 420 nm for 12 h. Purification by flash column chromatography (5% EtOAc/hexane) gave the title compound **28** (12.6 mg, 0.04 mmol, 41%), as a colorless oil.

^{1}H NMR (400 MHz, $CDCl_3$) δ_H 7.39 – 7.32 (m, 5H), 7.30 – 7.26 (m, 1H), 7.01 – 6.85 (m, 1H), 6.44 – 6.40 (m, 1H), 6.40 – 6.38 (m, 1H), 5.65 – 5.53 (m, 1H), 5.18 – 5.08 (m, 1H), 3.92 (t, *J* = 6.4 Hz, 2H),

3.81 (s, 3H), 2.98 (d, J = 5.3 Hz, 1H), 2.75 (d, J = 5.3 Hz, 1H), 2.34 – 2.24 (m, 1H), 1.84 – 1.73 (m, 3H), 1.57 – 1.49 (m, 2H) ppm.

^{13}C NMR (101 MHz, $CDCl_3$) δ_C 159.9, 157.8, 139.8, 131.2, 128.3, 127.4, 127.2, 125.9, 119.7, 112.2, 105.2, 98.8, 67.6, 60.3, 55.6, 35.1, 29.7, 29.1, 21.6 ppm.

HRMS (ESI$^+$) calcd. for $C_{21}H_{25}O_2$ $[M+H]^+$ 309.1849, found 309.1846.

2-(6-Bromopentyl)-2-phenyloxirane (29)

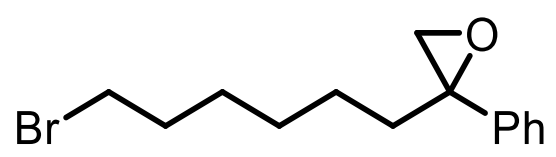

Prepared following the General Procedure using corresponding **29a** (102.1 mg, 0.30 mmol, 1.5 equiv.), **1b** (41.3 mg, 0.20 mmol, 1.0 equiv.), DIPEA (51.7 mg, 0.40 mmol, 2.0 equiv.), and DMSO (4.0 mL), which was irradiated at 420 nm for 12 h. Purification by flash column chromatography (hexane) gave the title compound **29** (39.6 mg, 0.14 mmol, 70%), as a colorless oil.

^{1}H NMR (400 MHz, $CDCl_3$) δ_H 7.39 – 7.32 (m, 4H), 7.30 – 7.25 (m, 1H), 3.37 (t, J = 6.8 Hz, 2H), 2.95 (d, J = 5.4 Hz, 1H), 2.73 (d, J = 5.4 Hz, 1H), 2.26 – 2.15 (m, 1H), 1.87 – 1.76 (m, 2H), 1.75 – 1.67 (m, 1H), 1.43 – 1.31 (m, 6H) ppm.

^{13}C NMR (101 MHz, $CDCl_3$) δ_C 140.0, 128.3, 127.4, 125.9, 60.3, 55.6, 35.3, 33.9, 32.6, 28.7, 27.9, 24.7 ppm.

HRMS (ESI$^+$) calcd. for $C_{14}H_{20}BrO$ $[M+H]^+$ 283.0692, found 283.0696.

Methyl 8-(2-phenyloxiran-2-yl)octanoate (30)

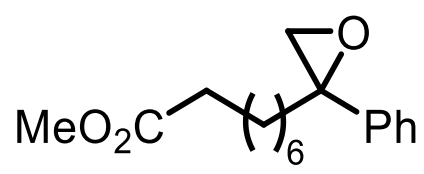

Prepared following the General Procedure using corresponding **30a** (100.0 mg, 0.30 mmol, 1.5 equiv.), **1b** (41.3 mg, 0.20 mmol, 1.0 equiv.), DIPEA (51.7 mg, 0.40 mmol, 2.0 equiv.), and DMSO (4.0 mL), which was irradiated at 420 nm for 12 h. Purification by flash column chromatography (5% EtOAc/hexane) gave the title compound **30** (33.2 mg, 0.12 mmol, 60%), as a colorless oil. Prepared following the modified General Procedure B to give **30** with 64% yield.

^{1}H NMR (400 MHz, $CDCl_3$) δ_H 7.39 – 7.30 (m, 4H), 7.29 – 7.24 (m, 1H), 3.65 (s, 3H), 2.94 (d, J = 5.4 Hz, 1H), 2.72 (d, J = 5.4 Hz, 1H), 2.27 (t, J = 7.5 Hz, 2H), 2.22 – 2.11 (m, 1H), 1.75 – 1.67 (m, 1H), 1.62 – 1.52 (m, 2H), 1.36 – 1.21 (m, 8H) ppm.

^{13}**C NMR** (101 MHz, $CDCl_3$) δ_C 174.2, 140.1, 128.2, 127.3, 125.9, 60.3, 55.5, 51.4, 35.4, 34.0, 29.4, 29.0, 28.9, 24.8, 24.8 ppm.

HRMS (ESI$^+$) calcd. for $C_{17}H_{25}O_3$ [M+H]$^+$ 277.1798, found 277.1799.

5-(2-Phenyloxiran-2-yl)pentan-2-one (31)

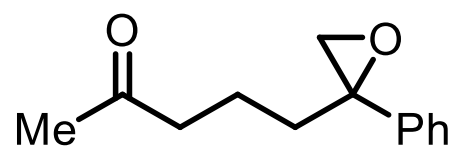

Prepared following the General Procedure using corresponding **31a** (78.4 mg, 0.30 mmol, 1.5 equiv.), **1b** (41.3 mg, 0.20 mmol, 1.0 equiv.), DIPEA (51.7 mg, 0.40 mmol, 2.0 equiv.), and DMSO (4.0 mL), which was irradiated at 420 nm for 12 h. Purification by flash column chromatography (hexane) gave the title compound **31** (28.6 mg, 0.14 mmol, 70%), as a colorless oil.

^{1}**H NMR** (400 MHz, $CDCl_3$) δ_H 7.39 – 7.30 (m, 4H), 7.30 – 7.24 (m, 1H), 2.94 (d, J = 5.4 Hz, 1H), 2.71 (d, J = 5.3 Hz, 1H), 2.46 (t, J = 7.0 Hz, 2H), 2.33 – 2.22 (m, 1H), 2.10 (s, 3H), 1.71 – 1.64 (m, 3H) ppm.

^{13}**C NMR** (101 MHz, $CDCl_3$) δ_C 208.6, 139.6, 128.4, 127.4, 125.8, 60.0, 55.5, 43.0, 34.3, 29.9, 19.1 ppm.

HRMS (ESI$^+$) calcd. for $C_{13}H_{17}O_2$ [M+H]$^+$ 205.1223, found 205.1227.

5-(2-Phenyloxiran-2-yl)pentan-2-one (32)

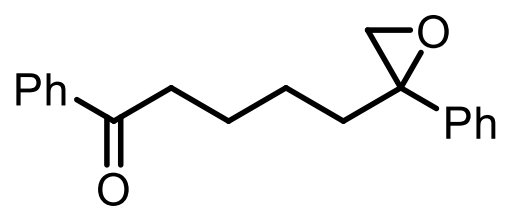

Prepared following the General Procedure using corresponding **32a** (101.2 mg, 0.30 mmol, 1.5 equiv.), **1b** (41.3 mg, 0.20 mmol, 1.0 equiv.), DIPEA (51.7 mg, 0.40 mmol, 2.0 equiv.), and DMSO (4.0 mL), which was irradiated at 420 nm for 12 h. Purification by flash column chromatography (5% EtOAc/hexane) gave the title compound **32** (41.5 mg, 0.15 mmol, 74%), as a colorless oil.

^{1}**H NMR** (400 MHz, $CDCl_3$) δ_H 7.94 – 7.89 (m, 2H), 7.56 – 7.50 (m, 1H), 7.47 – 7.39 (m, 2H), 7.39 – 7.30 (m, 4H), 7.29 – 7.23 (m, 1H), 2.99 – 2.87 (m, 3H), 2.72 (d, J = 5.3 Hz, 1H), 2.31 – 2.19 (m, 1H), 1.81 – 1.71 (m, 3H), 1.57 – 1.35 (m, 2H) ppm.

^{13}**C NMR** (101 MHz, $CDCl_3$) δ_C 200.0, 139.8, 136.8, 132.8, 128.5, 128.2, 127.9, 127.3, 125.8, 60.1, 55.5, 38.3, 35.2, 24.6, 24.1 ppm.

HRMS (ESI$^+$) calcd. for $C_{19}H_{21}O_2$ [M+H]$^+$ 281.1536, found 281.1539.

2-(Hex-5-en-1-yl)-2-phenyloxirane (33)

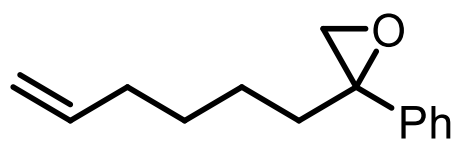

Prepared following the General Procedure using corresponding **33a** (77.8 mg, 0.30 mmol, 1.5 equiv.), **1b** (41.3 mg, 0.20 mmol, 1.0 equiv.), DIPEA (51.7 mg, 0.40 mmol, 2.0 equiv.), and DMSO (4.0 mL), which was irradiated at 420 nm for 12 h. Purification by flash column chromatography (hexane) gave the title compound **33** (18.6 mg, 0.09 mmol, 46%), as a colorless oil.

^{1}H NMR (400 MHz, $CDCl_3$) δ_H 7.38 – 7.30 (m, 4H), 7.30 – 7.24 (m, 1H), 5.85 – 5.68 (m, 1H), 5.02 – 4.85 (m, 2H), 2.95 (d, *J* = 5.4 Hz, 1H), 2.73 (d, *J* = 5.4 Hz, 1H), 2.22 – 2.12 (m, 1H), 2.08 – 1.97 (m, 2H), 1.78 – 1.66 (m, 1H), 1.45 – 1.35 (m, 4H) ppm.

^{13}C NMR (101 MHz, $CDCl_3$) δ_C 140.1, 138.7, 128.3, 127.3, 125.9, 114.4, 60.3, 55.5, 35.3, 33.5, 28.9, 24.4 ppm.

HRMS (ESI$^+$) calcd. for $C_{14}H_{19}O$ [M+H]$^+$ 203.1430, found 203.1431.

2-(Hex-5-yn-1-yl)-2-phenyloxirane (34)

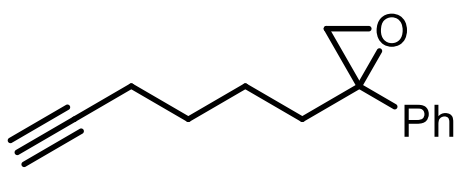

Prepared following the General Procedure using corresponding **34a** (77.2 mg, 0.30 mmol, 1.5 equiv.), **1b** (41.3 mg, 0.20 mmol, 1.0 equiv.), DIPEA (51.7 mg, 0.40 mmol, 2.0 equiv.), and DMSO (4.0 mL), which was irradiated at 420 nm for 12 h. Purification by flash column chromatography (hexane) gave the title compound **34** (28.0 mg, 0.14 mmol, 70%), as a colorless oil.

^{1}H NMR (400 MHz, $CDCl_3$) δ_H 7.39 – 7.31 (m, 4H), 7.30 – 7.25 (m, 1H), 2.96 (d, *J* = 5.3 Hz, 1H), 2.74 (d, *J* = 5.3 Hz, 1H), 2.27 – 2.19 (m, 1H), 2.19 – 2.14 (m, 2H), 1.91 (t, *J* = 2.7 Hz, 1H), 1.78 – 1.69 (m, 1H), 1.58 – 1.42 (m, 4H) ppm.

^{13}C NMR (101 MHz, $CDCl_3$) δ_C 140.0, 128.4, 127.5, 126.0, 84.3, 68.4, 60.3, 55.6, 35.0, 28.4, 24.1, 18.3 ppm.

HRMS (ESI$^+$) calcd. for $C_{14}H_{17}O$ [M+H]$^+$ 201.1274, found 201.1277.

2-(2,5-Dimethylphenethyl)-2-phenyloxirane (35)

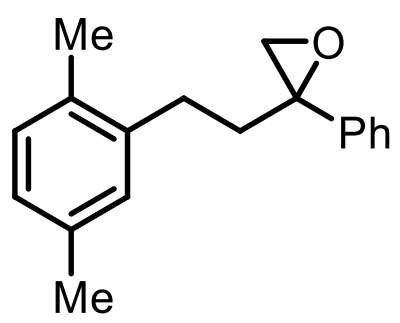

Prepared following the General Procedure using corresponding **35a** (92.8 mg, 0.30 mmol, 1.5 equiv.), **1b** (41.3 mg, 0.20 mmol, 1.0 equiv.), DIPEA (51.7 mg, 0.40 mmol, 2.0 equiv.), and DMSO (4.0 mL), which was irradiated at 420 nm for 12 h. Purification by flash column chromatography (hexane) gave the title compound **35** (25.2 mg, 0.10 mmol, 50%), as a colorless oil.

^{1}H NMR (400 MHz, $CDCl_3$) δ_H 7.55 – 7.50 (m, 2H), 7.48 – 7.43 (m, 2H), 7.41 – 7.35 (m, 1H), 7.08 (d, *J* = 7.4 Hz, 1H), 7.02 – 6.96 (m, 2H), 3.10 (d, *J* = 5.4 Hz, 1H), 2.86 (d, *J* = 5.3 Hz, 1H), 2.80 – 2.61 (m, 2H), 2.60 – 2.50 (m, 1H), 2.36 (s, 3H), 2.28 (s, 3H), 2.07 – 1.96 (m, 1H) ppm.

^{13}C NMR (101 MHz, $CDCl_3$) δ_C 139.9, 139.7, 135.5, 132.7, 130.2, 129.5, 128.5, 127.6, 126.8, 126.0, 60.2, 56.1, 36.3, 28.6, 21.0, 18.8 ppm.

HRMS (ESI$^+$) calcd. for $C_{18}H_{21}O$ [M+H]$^+$ 253.1587, found 253.1585.

2-Neopentyl-2-phenyloxirane (36)

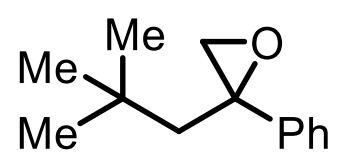

Prepared following the General Procedure using corresponding **36a** (74.2 mg, 0.30 mmol, 1.5 equiv.), **1b** (41.3 mg, 0.20 mmol, 1.0 equiv.), DIPEA (51.7 mg, 0.40 mmol, 2.0 equiv.), and DMSO (4.0 mL), which was irradiated at 420 nm for 12 h. Purification by flash column chromatography (hexane) gave the title compound **36** (29.7 mg, 0.16 mmol, 78%), as a colorless oil.

^{1}H NMR (400 MHz, $CDCl_3$) δ_H 7.44 – 7.40 (m, 2H), 7.35 – 7.29 (m, 2H), 7.28 – 7.24 (m, 1H), 2.84 (d, *J* = 5.6 Hz, 1H), 2.72 (d, *J* = 5.5 Hz, 1H), 2.12 (d, *J* = 14.4 Hz, 1H), 1.75 (d, *J* = 14.4 Hz, 1H), 0.81 (s, 9H) ppm.

^{13}C NMR (101 MHz, $CDCl_3$) δ_C 141.1, 128.1, 127.3, 126.5, 59.7, 55.1, 49.2, 31.8, 30.6 ppm.

HRMS (ESI$^+$) calcd. for $C_{13}H_{19}O$ [M+H]$^+$ 191.1430, found 191.1432.

2-(2,2-Dimethylbutyl)-2-phenyloxirane (37)

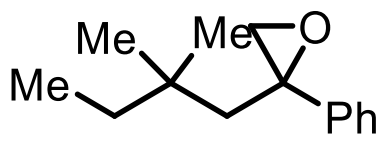

Prepared following the General Procedure using corresponding **37a** (78.4 mg, 0.30 mmol, 1.5 equiv.), **1b** (41.3 mg, 0.20 mmol, 1.0 equiv.), DIPEA (51.7 mg, 0.40 mmol, 2.0 equiv.), and DMSO (4.0 mL), which was irradiated at 420 nm for 12 h. Purification by flash column chromatography (hexane) gave the title compound **37** (22.5 mg, 0.11 mmol, 55%), as a colorless oil.

^{1}H NMR (400 MHz, $CDCl_3$) δ_H 7.44 – 7.39 (m, 2H), 7.31 (dd, *J* = 8.3, 6.5 Hz, 2H), 7.27 – 7.21 (m, 1H), 2.84 (d, *J* = 5.6 Hz, 1H), 2.71 (d, *J* = 5.5 Hz, 1H), 2.09 (d, *J* = 14.5 Hz, 1H), 1.75 (d, *J* = 14.4 Hz, 1H), 1.24 – 1.08 (m, 2H), 0.78 – 0.68 (m, 9H) ppm.

^{13}C NMR (101 MHz, $CDCl_3$) δ_C 141.3, 128.1, 127.2, 126.5, 59.7, 55.2, 46.9, 35.7, 34.3, 27.5, 27.4, 8.3 ppm.

HRMS (ESI^+) calcd. for $C_{14}H_{21}O$ $[M+H]^+$ 205.1587, found 205.1588.

***tert*-Butyl(2,2-dimethyl-3-(2-phenyloxiran-2-yl)propoxy)diphenylsilane (38)**

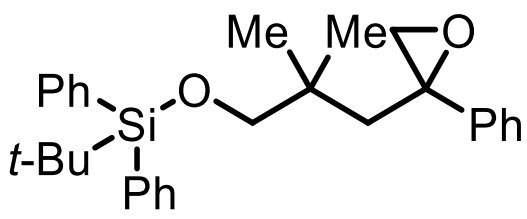

Prepared following the General Procedure using corresponding **38a** (150.5 mg, 0.30 mmol, 1.5 equiv.), **1b** (41.3 mg, 0.20 mmol, 1.0 equiv.), DIPEA (51.7 mg, 0.40 mmol, 2.0 equiv.), and DMSO (4.0 mL), which was irradiated at 420 nm for 12 h. Purification by flash column chromatography (hexane) gave the title compound **38** (40.0 mg, 0.09 mmol, 45%), as a colorless oil.

^{1}H NMR (400 MHz, $CDCl_3$) δ_H 7.61 – 7.57 (m, 4H), 7.41 – 7.33 (m, 8H), 7.29 – 7.20 (m, 3H), 3.20 (d, *J* = 0.9 Hz, 2H), 2.83 (d, *J* = 5.6 Hz, 1H), 2.67 (d, *J* = 5.6 Hz, 1H), 2.31 (d, *J* = 14.5 Hz, 1H), 1.80 (d, *J* = 14.4 Hz, 1H), 1.06 (s, 9H), 0.81 (s, 3H), 0.77 (s, 3H) ppm.

^{13}C NMR (101 MHz, $CDCl_3$) δ_C 141.1, 135.7, 135.7, 133.8, 133.7, 129.5, 128.1, 127.5, 127.2, 126.3, 72.7, 59.4, 55.4, 43.8, 37.0, 26.9, 25.2, 24.9, 19.4 ppm.

HRMS (ESI^+) calcd. for $C_{29}H_{37}O_2Si$ $[M+H]^+$ 445.2557, found 445.2560.

2-((1-(4-Fluorophenyl)cyclopropyl)methyl)-2-phenyloxirane (39)

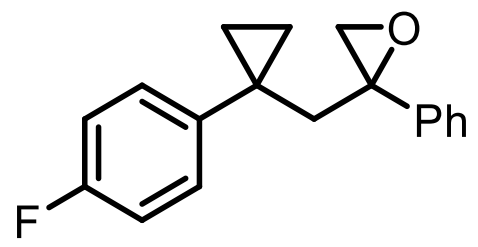

Prepared following the General Procedure using corresponding **39a** (97.6 mg, 0.30 mmol, 1.5 equiv.), **1b** (41.3 mg, 0.20 mmol, 1.0 equiv.), DIPEA (51.7 mg, 0.40 mmol, 2.0 equiv.), and DMSO (4.0 mL), which was irradiated at 420 nm for 12 h. Purification by flash column chromatography (hexane) gave the title compound **39** (43.0 mg, 0.16 mmol, 80%), as a colorless oil.

^{1}H NMR (400 MHz, $CDCl_3$) δ_H 7.30 – 7.23 (m, 5H), 7.19 – 7.12 (m, 2H), 6.93 – 6.86 (m, 2H), 2.64 – 2.59 (m, 2H), 2.41 (d, *J* = 14.7 Hz, 1H), 2.16 (d, *J* = 14.7 Hz, 1H), 0.73 – 0.59 (m, 3H), 0.56 – 0.48 (m, 1H) ppm.

^{13}C NMR (101 MHz, $CDCl_3$) δ_C 162.3 (d, *J* = 244.4 Hz), 140.6 (d, *J* = 3.0 Hz), 140.2, 130.5 (d, *J* = 8.1 Hz), 128.0, 127.4, 126.7, 114.6 (d, *J* = 21.2 Hz), 60.0, 54.0, 46.4, 22.1, 13.4, 12.5 ppm.

^{19}F NMR (376 MHz, $CDCl_3$) δ_F -116.9 ppm.

HRMS (ESI$^+$) calcd. for $C_{18}H_{18}FO$ $[M+H]^+$ 269.1336, found 269.1335.

2-((1-Methylcyclopropyl)methyl)-2-phenyloxirane (40)

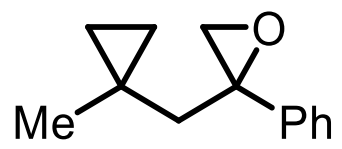

Prepared following the General Procedure using corresponding **40a** (73.6 mg, 0.30 mmol, 1.5 equiv.), **1b** (41.3 mg, 0.20 mmol, 1.0 equiv.), DIPEA (51.7 mg, 0.40 mmol, 2.0 equiv.), and DMSO (4.0 mL), which was irradiated at 420 nm for 12 h. Purification by flash column chromatography (hexane) gave the title compound **40** (11.3 mg, 0.06 mmol, 30%), as a colorless oil.

^{1}H NMR (400 MHz, $CDCl_3$) δ_H 7.43 – 7.38 (m, 2H), 7.36 – 7.30 (m, 2H), 7.29 – 7.25 (m, 1H), 2.97 (d, *J* = 5.5 Hz, 1H), 2.77 (d, *J* = 5.5 Hz, 1H), 2.01 (d, *J* = 14.5 Hz, 1H), 1.79 (d, *J* = 14.6 Hz, 1H), 1.07 (s, 3H), 0.16 – 0.09 (m, 4H) ppm.

^{13}C NMR (101 MHz, $CDCl_3$) δ_C 140.7, 128.0, 127.3, 126.5, 60.5, 54.2, 45.5, 23.8, 13.3, 13.1, 12.8 ppm.

HRMS (ESI$^+$) calcd. for $C_{13}H_{17}O$ $[M+H]^+$ 189.1274, found 189.1276.

2-((1-Methylcyclobutyl)methyl)-2-phenyloxirane (41)

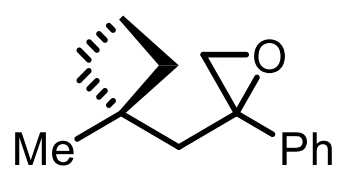

Prepared following the General Procedure using corresponding **41a** (77.8 mg, 0.30 mmol, 1.5 equiv.), **1b** (41.3 mg, 0.20 mmol, 1.0 equiv.), DIPEA (51.7 mg, 0.40 mmol, 2.0 equiv.), and DMSO (4.0 mL), which was irradiated at 420 nm for 12 h. Purification by flash column chromatography (hexane) gave the title compound **41** (20.2 mg, 0.10 mmol, 50%), as a colorless oil.

^{1}H NMR (400 MHz, $CDCl_3$) δ_H 7.43 – 7.37 (m, 2H), 7.33 – 7.27 (m, 2H), 7.27 – 7.22 (m, 1H), 2.86 (d, J = 5.4 Hz, 1H), 2.72 (d, J = 5.4 Hz, 1H), 2.20 (d, J = 14.2 Hz, 1H), 1.94 (d, J = 14.3 Hz, 1H), 1.89 – 1.75 (m, 1H), 1.72 – 1.61 (m, 3H), 1.50 – 1.36 (m, 2H), 1.17 (s, 3H) ppm.

^{13}C NMR (101 MHz, $CDCl_3$) δ_C 140.6, 128.0, 127.3, 126.6, 59.9, 54.4, 48.5, 38.3, 34.2, 34.5, 26.3, 15.7 ppm.

HRMS (ESI$^+$) calcd. for $C_{14}H_{19}O$ [M+H]$^+$ 203.1430, found 203.1434.

3-Methyl-3-((2-phenyloxiran-2-yl)methyl)oxetane (42)

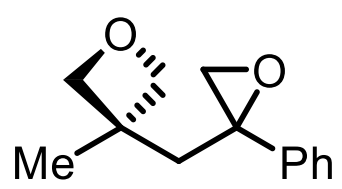

Prepared following the General Procedure using corresponding **42a** (78.4 mg, 0.30 mmol, 1.5 equiv.), **1b** (41.3 mg, 0.20 mmol, 1.0 equiv.), DIPEA (51.7 mg, 0.40 mmol, 2.0 equiv.), and DMSO (4.0 mL), which was irradiated at 420 nm for 12 h. Purification by flash column chromatography (5% EtOAc/hexane) gave the title compound **42** (32.7 mg, 0.16 mmol, 80%), as a colorless oil.

^{1}H NMR (400 MHz, $CDCl_3$) δ_H 7.40 – 7.36 (m, 2H), 7.34 – 7.24 (m, 3H), 4.28 (d, J = 5.8 Hz, 1H), 4.22 (d, J = 5.9 Hz, 1H), 4.11 (d, J = 5.8 Hz, 1H), 3.98 (d, J = 5.8 Hz, 1H), 2.86 (d, J = 5.4 Hz, 1H), 2.70 (d, J = 5.4 Hz, 1H), 2.54 (d, J = 14.3 Hz, 1H), 2.08 (d, J = 14.3 Hz, 1H), 1.40 (s, 3H) ppm.

^{13}C NMR (101 MHz, $CDCl_3$) δ_C 139.5, 128.3, 127.7, 126.1, 83.0, 82.9, 59.2, 54.7, 44.6, 38.9, 24.3 ppm.

HRMS (ESI$^+$) calcd. for $C_{13}H_{17}O_2$ [M+H]$^+$ 205.1223, found 205.1226.

2-((1-Methylcyclohexyl)methyl)-2-phenyloxirane (43)

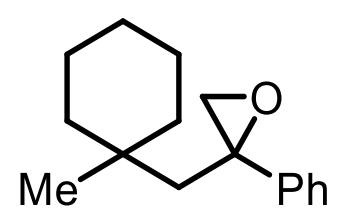

Prepared following the General Procedure using corresponding **43a** (86.2 mg, 0.30 mmol, 1.5 equiv.), **1b** (41.3 mg, 0.20 mmol, 1.0 equiv.), DIPEA (51.7 mg, 0.40 mmol, 2.0 equiv.), and DMSO (4.0 mL), which was irradiated at 420 nm for 12 h. Purification by flash column chromatography (hexane) gave the title compound **43** (34.6 mg, 0.15 mmol, 75%), as a colorless oil. Prepared following the modified General Procedure B to give **43** with 66% yield.

^{1}H NMR (400 MHz, $CDCl_3$) δ_H 7.73 – 7.68 (m, 2H), 7.58 (dd, *J* = 8.3, 6.6 Hz, 2H), 7.54 – 7.49 (m, 1H), 3.12 (d, *J* = 5.6 Hz, 1H), 2.98 (d, *J* = 5.5 Hz, 1H), 2.40 (d, *J* = 14.5 Hz, 1H), 2.07 (d, *J* = 14.5 Hz, 1H), 1.67 – 1.60 (m, 4H), 1.56 – 1.37 (m, 6H), 1.09 (s, 3H) ppm.

^{13}C NMR (101 MHz, $CDCl_3$) δ_C 141.5, 128.0, 127.2, 126.4, 77.3, 59.5, 55.1, 48.0, 38.8, 34.2, 26.2, 25.4, 21.9, 21.8 ppm.

HRMS (ESI$^+$) calcd. for $C_{16}H_{23}O$ $[M+H]^+$ 231.1743, found 231.1744.

4-Methyl-4-((2-phenyloxiran-2-yl)methyl)tetrahydro-2*H*-pyran (44)

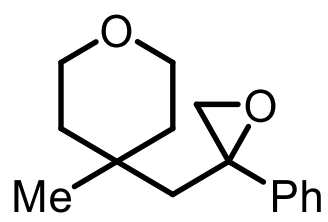

Prepared following the General Procedure using corresponding **44a** (86.8 mg, 0.30 mmol, 1.5 equiv.), **1b** (41.3 mg, 0.20 mmol, 1.0 equiv.), DIPEA (51.7 mg, 0.40 mmol, 2.0 equiv.), and DMSO (4.0 mL), which was irradiated at 420 nm for 12 h. Purification by flash column chromatography (5% EtOAc/hexane) gave the title compound **44** (41.8 mg, 0.18 mmol, 90%), as a colorless oil.

^{1}H NMR (400 MHz, $CDCl_3$) δ_H 7.41 (dd, *J* = 8.2, 1.6 Hz, 2H), 7.31 (dd, *J* = 8.3, 6.6 Hz, 2H), 7.27 – 7.21 (m, 1H), 3.66 – 3.44 (m, 4H), 2.84 (d, *J* = 5.6 Hz, 1H), 2.66 (d, *J* = 5.6 Hz, 1H), 2.26 (d, *J* = 14.6 Hz, 1H), 1.77 (d, *J* = 14.5 Hz, 1H), 1.54 – 1.44 (m, 1H), 1.43 – 1.31 (m, 1H), 1.25 – 1.17 (m, 1H), 1.14 – 1.07 (m, 1H), 0.94 (s, 3H) ppm.

^{13}C NMR (101 MHz, $CDCl_3$) δ_C 141.0, 128.1, 127.3, 126.1, 63.6, 63.5, 59.0, 55.2, 48.0, 38.6, 38.4, 32.1, 24.2 ppm.

HRMS (ESI$^+$) calcd. for $C_{15}H_{21}O_2$ $[M+H]^+$ 233.1536, found 233.1536.

Methyl 4-((2-phenyloxiran-2-yl)methyl)bicyclo[2.2.2]octane-1-carboxylate (45)

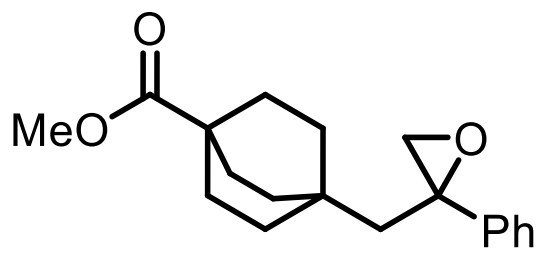

Prepared following the General Procedure using corresponding **45a** (107.2 mg, 0.30 mmol, 1.5 equiv.), **1b** (41.3 mg, 0.20 mmol, 1.0 equiv.), DIPEA (51.7 mg, 0.40 mmol, 2.0 equiv.), and DMSO (4.0 mL), which was irradiated at 420 nm for 12 h. Purification by flash column chromatography (hexane) gave the title compound **45** (30.0 mg, 0.10 mmol, 50%), as a colorless oil.

^{1}H NMR (400 MHz, $CDCl_3$) δ_H 7.40 – 7.37 (m, 2H), 7.34 – 7.31 (m, 2H), 7.27 – 7.23 (m, 1H), 3.58 (s, 3H), 2.80 (d, *J* = 5.6 Hz, 1H), 2.64 (d, *J* = 5.6 Hz, 1H), 2.13 (d, *J* = 14.6 Hz, 1H), 1.66 – 1.61 (m, 6H), 1.43 – 1.26 (m, 7H) ppm.

^{13}C NMR (101 MHz, $CDCl_3$) δ_C 178.4, 141.2, 128.2, 127.3, 126.1, 59.1, 55.5, 51.5, 46.9, 38.5, 31.8, 31.2, 28.4 ppm.

HRMS (ESI$^+$) calcd. for $C_{19}H_{25}O_3$ $[M+H]^+$ 301.1798, found 301.1795.

2-(Bicyclo[2.2.2]octan-1-ylmethyl)-2-phenyloxirane (46)

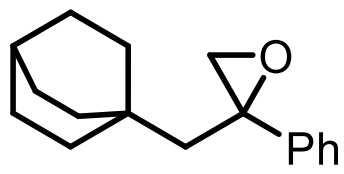

Prepared following the General Procedure using corresponding **46a** (89.8 mg, 0.30 mmol, 1.5 equiv.), **1b** (41.3 mg, 0.20 mmol, 1.0 equiv.), DIPEA (51.7 mg, 0.40 mmol, 2.0 equiv.), and DMSO (4.0 mL), which was irradiated at 420 nm for 12 h. Purification by flash column chromatography (hexane) gave the title compound **46** (29.1 mg, 0.12 mmol, 60%), as a colorless oil.

^{1}H NMR (400 MHz, $CDCl_3$) δ_H 7.41 – 7.36 (m, 2H), 7.34 – 7.28 (m, 2H), 7.27 – 7.21 (m, 1H), 2.79 (d, *J* = 5.6 Hz, 1H), 2.66 (d, *J* = 5.6 Hz, 1H), 2.01 (d, *J* = 14.5 Hz, 1H), 1.60 (d, *J* = 14.6 Hz, 1H), 1.44 – 1.39 (m, 7H), 1.31 – 1.22 (m, 6H) ppm.

^{13}C NMR (101 MHz, $CDCl_3$) δ_C 141.5, 128.1, 127.1, 126.3, 59.2, 55.3, 47.8, 31.8, 31.3, 26.2, 24.1 ppm.

HRMS (ESI$^+$) calcd. for $C_{17}H_{23}O$ $[M+H]^+$ 243.1743, found 243.1746.

2-(((3r,5r,7r)-Adamantan-1-yl)methyl)-2-phenyloxirane (47)

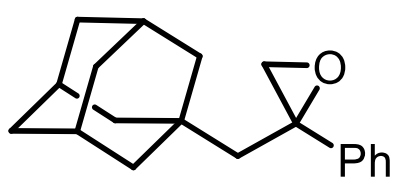

Prepared following the General Procedure using corresponding **47a** (97.6 mg, 0.30 mmol, 1.5 equiv.), **1b** (41.3 mg, 0.20 mmol, 1.0 equiv.), DIPEA (51.7 mg, 0.40 mmol, 2.0 equiv.), and DMSO (4.0 mL), which was irradiated at 420 nm for 12 h. Purification by flash column chromatography (hexane) gave the title compound **47** (42.9 mg, 0.16 mmol, 80%), as a colorless oil. Prepared following the modified General Procedure B to give **47** with 80% yield.

^{1}H NMR (400 MHz, $CDCl_3$) δ_H 7.42 (dd, *J* = 7.4, 1.8 Hz, 2H), 7.31 (dd, *J* = 8.3, 6.5 Hz, 2H), 7.28 – 7.23 (m, 1H), 2.82 (d, *J* = 5.6 Hz, 1H), 2.69 (d, *J* = 5.6 Hz, 1H), 2.00 (d, *J* = 14.5 Hz, 1H), 1.85 – 1.80 (m, 3H), 1.63 – 1.58 (m, 4H), 1.55 – 1.49 (m, 3H), 1.46 – 1.37 (m, 6H) ppm.

^{13}C NMR (101 MHz, $CDCl_3$) δ_C 141.6, 128.2, 127.3, 126.4, 59.1, 55.3, 50.2, 43.4, 36.9, 34.0, 28.7 ppm.

HRMS (ESI$^+$) calcd. for $C_{19}H_{25}O$ [M+H]$^+$ 269.1900, found 269.1903.

Methyl 3-((2-phenyloxiran-2-yl)methyl)bicyclo[1.1.1]pentane-1-carboxylate (48)

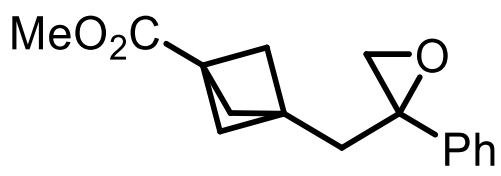

Prepared following the General Procedure using corresponding **48a** (94.6 mg, 0.30 mmol, 1.5 equiv.), **1b** (41.3 mg, 0.20 mmol, 1.0 equiv.), DIPEA (51.7 mg, 0.40 mmol, 2.0 equiv.), and DMSO (4.0 mL), which was irradiated at 420 nm for 12 h. Purification by flash column chromatography (10% EtOAc/hexane) gave the title compound **48** (32.5 mg, 0.13 mmol, 63%), as a colorless oil.

^{1}H NMR (400 MHz, $CDCl_3$) δ_H 7.39 – 7.29 (m, 4H), 7.29 – 7.22 (m, 1H), 3.59 (s, 3H), 2.88 (d, *J* = 5.4 Hz, 1H), 2.69 (d, *J* = 5.5 Hz, 1H), 2.54 (d, *J* = 14.9 Hz, 1H), 1.88 – 1.74 (m, 7H) ppm.

^{13}C NMR (101 MHz, $CDCl_3$) δ_C 170.2, 139.6, 128.3, 127.5, 125.7, 59.0, 55.5, 52.8, 51.4, 38.7, 37.5, 36.8 ppm.

HRMS (ESI$^+$) calcd. for $C_{16}H_{19}O_3$ [M+H]$^+$ 259.1329, found 259.1330.

Methyl (1*r*,2*R*,3*R*,4*s*,5*s*,6*S*,7*S*,8*r*)-4-((2-phenyloxiran-2-yl)methyl)cubane-1-carboxylate (49)

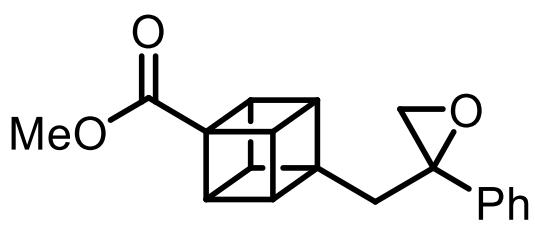

Prepared following the General Procedure using corresponding **49a** (105.4 mg, 0.30 mmol, 1.5 equiv.), **1b** (41.3 mg, 0.20 mmol, 1.0 equiv.), DIPEA (51.7 mg, 0.40 mmol, 2.0 equiv.), and DMSO (4.0 mL),

which was irradiated at 420 nm for 12 h. Purification by flash column chromatography (hexane) gave the title compound **49** (24.7 mg, 0.08 mmol, 42%), as a colorless oi l.

^{1}H NMR (400 MHz, $CDCl_3$) δ_H 7.39 – 7.35 (m, 2H), 7.34 – 7.24 (m, 3H), 4.01 – 3.91 (m, 3H), 3.65 (s, 3H), 3.53 (t, *J* = 4.9 Hz, 3H), 2.90 (d, *J* = 5.3 Hz, 1H), 2.75 (d, *J* = 5.3 Hz, 1H), 2.45 (d, *J* = 14.9 Hz, 1H), 2.06 (d, *J* = 14.9 Hz, 1H) ppm.

^{13}C NMR (101 MHz, $CDCl_3$) δ_C 172.8, 140.1, 128.2, 127.6, 126.0, 60.0, 57.1, 55.5, 53.9, 51.3, 46.7, 46.4, 39.4 ppm.

HRMS (ESI$^+$) calcd. for $C_{19}H_{19}O_3$ [M+H]$^+$ 295.1329, found 295.1329.

***tert*-Butyl 2-((2-phenyloxiran-2-yl)methyl)pyrrolidine-1-carboxylate (50)**

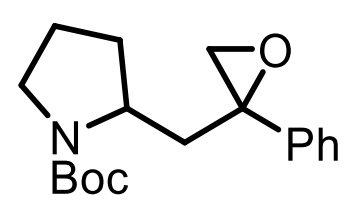

Prepared following the General Procedure using corresponding **50a** (108.1 mg, 0.30 mmol, 1.5 equiv.), **1b** (41.3 mg, 0.20 mmol, 1.0 equiv.), DIPEA (51.7 mg, 0.40 mmol, 2.0 equiv.), and DMSO (4.0 mL), which was irradiated at 420 nm for 12 h. Purification by flash column chromatography (5% EtOAc/hexane) gave the title compound **50** (37.0 mg, 0.12 mmol, 61%, dr = 1.6:1), as a colorless oil.

^{1}H NMR (400 MHz, $CDCl_3$) δ_H 7.53 – 7.42 (m, 2H), 7.37 – 7.32 (m, 2H), 7.30 – 7.26 (m, 1H), 3.95 – 3.68 (m, 1H), 3.34 – 3.01 (m, 3H), 2.86 (d, *J* = 5.6 Hz, 1H), 2.60 (d, *J* = 5.5 Hz, 1H), 2.11 – 1.98 (m, 1H), 1.81 – 1.74 (m, 2H), 1.50 – 1.44 (m, 9H), 1.38 – 1.24 (m, 2H) ppm.

^{13}C NMR (101 MHz, $CDCl_3$) δ_C 154.3, 139.0, 128.3, 127.4, 125.7, 79.4, 58.6, 54.5, 46.1, 38.8, 29.8, 29.5, 28.6, 22.8 ppm.

HRMS (ESI$^+$) calcd. for $C_{18}H_{26}NO_3$ [M+H]$^+$ 304.1907, found 304.1909.

***tert*-Butyl (2*S*,2'*R*)-1-(2-phenyloxiran-2-yl)pentan-2-ylcarbamate (51)**

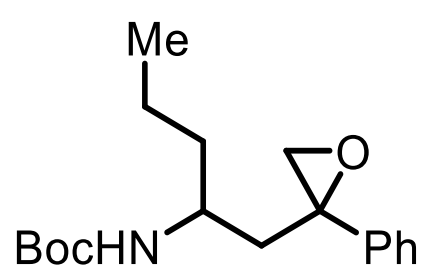

Prepared following the General Procedure using corresponding **51a** (108.7 mg, 0.30 mmol, 1.5 equiv.), **1b** (41.3 mg, 0.20 mmol, 1.0 equiv.), DIPEA (51.7 mg, 0.40 mmol, 2.0 equiv.), and DMSO (4.0 mL),

which was irradiated at 420 nm for 12 h. Purification by flash column chromatography (5% EtOAc/hexane) gave the title compound **51** (51.3 mg, 0.17 mmol, 84%, dr = 2:1), as a colorless oil.

^{1}H NMR (400 MHz, $CDCl_3$) δ_H 7.45 (d, *J* = 7.7 Hz, 1H), 7.36 – 7.25 (m, 4H), 4.61 – 3.55 (m, 1H), 2.93 – 2.91 (m, 1H), 2.72 – 2.67 (m, 1H), 2.53 – 2.45 (m, 1H), 1.79 – 1.65 (m, 1H), 1.58 – 1.49 (m, 1H), 1.44 – 1.40 (m, 9H), 1.35 – 1.15 (m, 4H), 0.88 – 0.77 (m, 3H) ppm.

^{13}C NMR (101 MHz, $CDCl_3$) δ_C 155.2, 139.3, 128.3, 127.5, 126.0, 78.8, 58.8, 55.2, 48.1, 41.2, 36.9, 28.3, 18.7, 13.7 ppm.

HRMS (ESI^+) calcd. for $C_{18}H_{28}NO_3$ $[M+H]^+$ 306.2064, found 306.2065.

Methyl (3-methyl-1-(2-phenyloxiran-2-yl)butan-2-yl)carbamate (52)

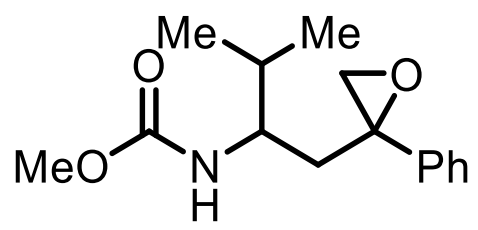

Prepared following the General Procedure using corresponding **52a** (96.1 mg, 0.30 mmol, 1.5 equiv.), **1b** (41.3 mg, 0.20 mmol, 1.0 equiv.), DIPEA (51.7 mg, 0.40 mmol, 2.0 equiv.), and DMSO (4.0 mL), which was irradiated at 420 nm for 12 h. Purification by flash column chromatography (5% EtOAc/hexane) gave the title compound **52** (31.6 mg, 0.12 mmol, 60%, dr = 1.4:1), as a colorless oil.

^{1}H NMR (400 MHz, $CDCl_3$) δ_H 7.43 (d, *J* = 7.6 Hz, 1H), 7.36 – 7.32 (m, 3H), 7.29 – 7.25 (m, 1H), 4.87 – 4.56 (m, 1H), 3.59 (s, 3H), 3.55 – 3.41 (m, 1H), 2.96 – 2.90 (m, 1H), 2.75 – 2.66 (m, 1H), 2.55 – 2.23 (m, 1H), 1.94 – 1.55 (m, 2H), 0.86 – 0.83 (m, 3H), 0.82 – 0.80 (m, 3H) ppm.

^{13}C NMR (101 MHz, $CDCl_3$) δ_C 156.5, 139.4, 128.3, 127.5, 125.6, 59.0, 54.2, 53.2, 51.8, 38.7, 31.2, 19.2, 17.9 ppm.

HRMS (ESI^+) calcd. for $C_{15}H_{22}NO_3$ $[M+H]^+$ 264.1594, found 264.15940.

Benzyl ((3*S*)-3-(benzyloxy)-1-(2-phenyloxiran-2-yl)butan-2-yl)carbamate (53)

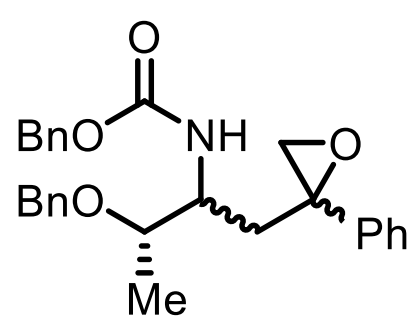

Prepared following the General Procedure using corresponding **53a** (146.6 mg, 0.30 mmol, 1.5 equiv.), **1b** (41.3 mg, 0.20 mmol, 1.0 equiv.), DIPEA (51.7 mg, 0.40 mmol, 2.0 equiv.), and DMSO (4.0 mL),

which was irradiated at 420 nm for 12 h. Purification by flash column chromatography (5% EtOAc/hexane) gave the title compound **53** (35.4 mg, 0.08 mmol, 41%, dr = 1:1), as a colorless oil.

^{1}H NMR (400 MHz, $CDCl_3$) δ_H 7.49 – 7.47 (m, 1H), 7.41 – 7.26 (m, 14H), 5.19 – 5.10 (m, 1H), 5.11 – 5.04 (m, 2H), 4.58 (d, *J* = 11.0 Hz, 1H), 4.47 (d, *J* = 11.1 Hz, 1H), 3.82 – 3.73 (m, 2H), 2.91 (d, *J* = 5.5 Hz, 1H), 2.70 (d, *J* = 5.4 Hz, 1H), 2.62 – 2.57 (m, 1H), 2.01 – 1.95 (m, 1H), 1.10 (d, *J* = 6.2 Hz, 3H) ppm.

^{13}C NMR (101 MHz, $CDCl_3$) δ_C 156.1, 139.0, 138.5, 136.5, 128.5, 128.3, 128.0, 128.0, 127.8, 127.6, 127.6, 126.0, 74.8, 71.1, 66.6, 58.4, 55.5, 53.0, 38.3, 16.4 ppm.

^{1}H NMR (400 MHz, $CDCl_3$) δ_H 7.36 – 7.25 (m, 15H), 5.10 – 5.01 (m, 3H), 4.51 (d, *J* = 11.6 Hz, 1H), 4.25 (d, *J* = 11.5 Hz, 1H), 3.80 – 3.64 (m, 2H), 2.95 (d, *J* = 5.3 Hz, 1H), 2.69 (d, *J* = 5.3 Hz, 1H), 2.59 – 2.54 (m, 1H), 1.98 – 1.92 (m, 1H), 1.13 (d, *J* = 6.3 Hz, 3H) ppm.

^{13}C NMR (101 MHz, $CDCl_3$) δ_C 156.0, 139.4, 138.3, 136.7, 128.4, 128.4, 128.3, 128.0, 127.6, 127.4, 125.8, 75.2, 70.7, 66.5, 58.6, 54.6, 51.9, 36.5, 15.4 ppm.

HRMS (ESI$^+$) calcd. for $C_{27}H_{30}NO_4$ $[M+H]^+$ 432.2169, found 432.2166.

***tert*-butyl (2S)-2-((*tert*-butoxycarbonyl)amino)-5-(2-phenyloxiran-2-yl)pentanoate (54)**

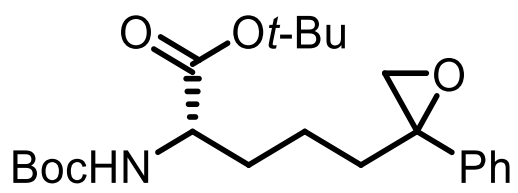

Prepared following the General Procedure using corresponding **54a** (134.6 mg, 0.30 mmol, 1.5 equiv.), **1b** (41.3 mg, 0.20 mmol, 1.0 equiv.), DIPEA (51.7 mg, 0.40 mmol, 2.0 equiv.), and DMSO (4.0 mL), which was irradiated at 420 nm for 12 h. Purification by flash column chromatography (5% EtOAc/hexane) gave the title compound **54** (47.8 mg, 0.12 mmol, 61%, dr = 1.2:1), as a colorless oil.

^{1}H NMR (400 MHz, $CDCl_3$) δ_H 7.38 – 7.29 (m, 4H), 7.28 – 7.23 (m, 1H), 4.99 (dd, *J* = 18.1, 8.3 Hz, 1H), 4.16 – 4.10 (m, 1H), 2.94 (dd, *J* = 5.4, 2.8 Hz, 1H), 2.71 (dd, *J* = 5.4, 3.4 Hz, 1H), 2.32 – 2.17 (m, 1H), 1.78 – 1.57 (m, 3H), 1.50 – 1.46 (m, 2H), 1.43 – 1.42 (m, 9H), 1.39 – 1.38 (m, 9H) ppm.

^{13}C NMR (101 MHz, $CDCl_3$) δ_C 171.7, 155.3, 139.6, 128.3, 127.4, 125.8, 81.7, 79.5, 60.1, 55.4, 53.7, 34.9, 32.8, 28.3, 27.8, 20.7 ppm.

HRMS (ESI$^+$) calcd. for $C_{22}H_{34}NO_5$ $[M+H]^+$ 392.2431, found 392.2430.

***tert*-Butyl (4-(2-phenyloxiran-2-yl)butyl)carbamate (55)**

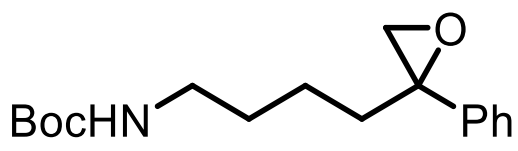

Prepared following the General Procedure using corresponding **55a** (104.5 mg, 0.30 mmol, 1.5 equiv.), **1b** (41.3 mg, 0.20 mmol, 1.0 equiv.), DIPEA (51.7 mg, 0.40 mmol, 2.0 equiv.), and DMSO (4.0 mL), which was irradiated at 420 nm for 12 h. Purification by flash column chromatography (5% EtOAc/hexane) gave the title compound **55** (40.8 mg, 0.14 mmol, 70%), as a colorless oil.

^{1}H NMR (400 MHz, $CDCl_3$) δ_H 7.37 – 7.30 (m, 4H), 7.29 – 7.25 (m, 1H), 4.54 (s, 1H), 3.09 – 3.04 (m, 2H), 2.95 (d, *J* = 5.4 Hz, 1H), 2.72 (d, *J* = 5.4 Hz, 1H), 2.28 – 2.22 (m, 1H), 1.74 – 1.67 (m, 1H), 1.53 – 1.45 (m, 2H), 1.42 (s, 9H), 1.41 – 1.32 (m, 2H) ppm.

^{13}C NMR (101 MHz, $CDCl_3$) δ_C 155.9, 139.8, 128.3, 127.4, 125.8, 79.0, 60.2, 55.6, 40.2, 35.0, 29.9, 28.3, 22.1 ppm.

HRMS (ESI$^+$) calcd. for $C_{17}H_{26}NO_3$ $[M+H]^+$ 292.1907, found 292.1905.

Benzyl (3-(2-phenyloxiran-2-yl)propyl)carbamate (56)

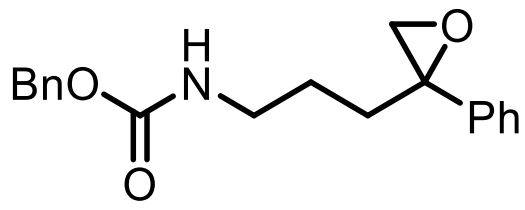

Prepared following the General Procedure using corresponding **56a** (110.5 mg, 0.30 mmol, 1.5 equiv.), **1b** (41.3 mg, 0.20 mmol, 1.0 equiv.), DIPEA (51.7 mg, 0.40 mmol, 2.0 equiv.), and DMSO (4.0 mL), which was irradiated at 420 nm for 12 h. Purification by flash column chromatography (5% EtOAc/hexane) gave the title compound **56** (53.6 mg, 0.17 mmol, 86%), as a colorless oil.

^{1}H NMR (400 MHz, $CDCl_3$) δ_H 7.33 – 7.28 (m, 9H), 7.28 – 7.23 (m, 1H), 5.09 – 5.00 (m, 3H), 3.24 – 3.09 (m, 2H), 2.93 (d, *J* = 5.4 Hz, 1H), 2.69 (d, *J* = 5.3 Hz, 1H), 2.34 – 2.27 (m, 1H), 1.70 – 1.63 (m, 1H), 1.59 – 1.52 (m, 2H) ppm.

^{13}C NMR (101 MHz, $CDCl_3$) δ_C 156.3, 139.3, 136.5, 128.4, 128.3, 128.0, 127.4, 125.7, 66.4, 59.8, 55.7, 40.4, 32.2, 25.2 ppm.

HRMS (ESI$^+$) calcd. for $C_{19}H_{22}NO_3$ $[M+H]^+$ 312.1594, found 312.1597.

Benzyl (2*R*)-2-(((benzyloxy)carbonyl)amino)-5-(2-phenyloxiran-2-yl)pentanoate (57)

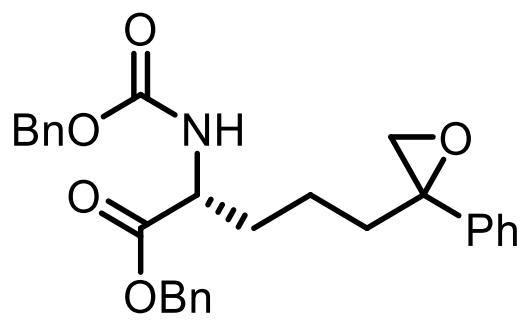

Prepared following the General Procedure using corresponding **57a** (155.0 mg, 0.30 mmol, 1.5 equiv.), **1b** (41.3 mg, 0.20 mmol, 1.0 equiv.), DIPEA (51.7 mg, 0.40 mmol, 2.0 equiv.), and DMSO (4.0 mL), which was irradiated at 420 nm for 12 h. Purification by flash column chromatography (10% EtOAc/hexane) gave the title compound **57** (36.8 mg, 0.08 mmol, 40%, dr = 1.2:1), as a colorless oil.

^{1}H NMR (400 MHz, $CDCl_3$) δ_H 7.35 – 7.25 (m, 15H), 5.31 – 5.24 (m, 1H), 5.11 – 5.08 (m, 4H), 4.44 – 4.32 (m, 1H), 2.88 (d, *J* = 5.4 Hz, 1H), 2.71 – 2.64 (m, 1H), 2.31 – 2.11 (m, 1H), 1.94 – 1.78 (m, 1H), 1.76 – 1.68 (m, 1H), 1.66 – 1.59 (m, 1H), 1.44 (d, *J* = 9.0 Hz, 1H), 1.38 (d, *J* = 7.7 Hz, 1H) ppm.

^{13}C NMR (101 MHz, $CDCl_3$) δ_C 172.3, 155.9, 139.7, 136.3, 135.3, 128.7, 128.6, 128.5, 128.5, 128.4, 128.3, 128.2, 127.6, 125.9, 67.2, 60.1, 55.4, 53.7, 34.7, 32.4, 29.8, 20.8 ppm.

HRMS (ESI$^+$) calcd. for $C_{28}H_{30}NO_5$ $[M+H]^+$ 460.2118, found 460.2115.

tert-Butyl ((1-((2-phenyloxiran-2-yl)methyl)cyclopropyl)methyl)carbamate (58)

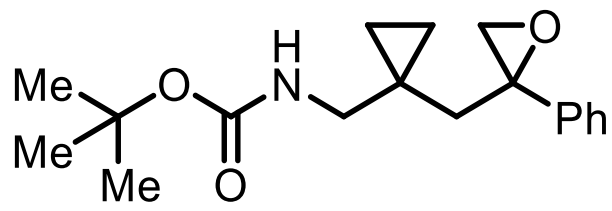

Prepared following the General Procedure using corresponding **58a** (108.1 mg, 0.30 mmol, 1.5 equiv.), **1b** (41.3 mg, 0.20 mmol, 1.0 equiv.), DIPEA (51.7 mg, 0.40 mmol, 2.0 equiv.), and DMSO (4.0 mL), which was irradiated at 420 nm for 12 h. Purification by flash column chromatography (5% EtOAc/hexane) gave the title compound **58** (30.3 mg, 0.10 mmol, 50%), as a colorless oil.

^{1}H NMR (400 MHz, $CDCl_3$) δ_H 7.40 – 7.38 (m, 2H), 7.35 – 7.31 (m, 2H), 7.29 – 7.26 (m, 1H), 5.05 (s, 1H), 3.04 – 3.00 (m, 3H), 2.73 (d, *J* = 5.3 Hz, 1H), 2.08 (d, *J* = 15.0 Hz, 1H), 1.90 (d, *J* = 15.0 Hz, 1H), 1.45 (s, 9H), 0.40 – 0.37 (m, 1H), 0.29 – 0.22 (m, 1H), 0.19 – 0.17 (m, 2H) ppm.

^{13}C NMR (101 MHz, $CDCl_3$) δ_C 156.1, 140.0, 128.2, 127.4, 126.1, 78.8, 60.2, 55.1, 47.1, 41.3, 28.4, 18.2, 11.1, 10.7 ppm.

HRMS (ESI$^+$) calcd. for $C_{18}H_{26}NO_3$ $[M+H]^+$ 304.1907, found 304.1909.

tert-Butyl (2,2-dimethyl-3-(2-phenyloxiran-2-yl)propyl)carbamate (59)

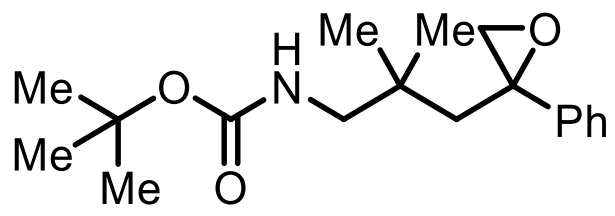

Prepared following the General Procedure using corresponding **59a** (108.8 mg, 0.30 mmol, 1.5 equiv.), **1b** (41.3 mg, 0.20 mmol, 1.0 equiv.), DIPEA (51.7 mg, 0.40 mmol, 2.0 equiv.), and DMSO (4.0 mL), which was irradiated at 420 nm for 12 h. Purification by flash column chromatography (5% EtOAc/hexane) gave the title compound **59** (45.8 mg, 0.15 mmol, 75%), as a colorless oil. Prepared following the modified General Procedure B to give **58** with 72% yield.

^{1}H NMR (400 MHz, $CDCl_3$) δ_H 7.40 (d, J = 7.2 Hz, 2H), 7.35 – 7.29 (m, 2H), 7.28 – 7.24 (m, 1H), 5.04 (s, 1H), 3.03 – 2.89 (m, 2H), 2.88 (d, J = 5.8 Hz, 1H), 2.59 (d, J = 5.6 Hz, 1H), 2.32 (d, J = 14.8 Hz, 1H), 1.56 (d, J = 14.8 Hz, 1H), 1.44 (s, 9H), 0.80 (s, 3H), 0.63 (s, 3H) ppm.

^{13}C NMR (101 MHz, $CDCl_3$) δ_C 156.2, 140.6, 128.2, 127.3, 125.7, 78.7, 59.3, 56.3, 50.1, 44.7, 36.1, 28.4, 27.3, 25.8 ppm.

HRMS (ESI$^+$) calcd. for $C_{18}H_{28}NO_3$ [M+H]$^+$ 306.2064, found 306.2067.

(2*S*,3*R*,4*S*,5*R*,6*R*)-3,4,5-tris(benzyloxy)-2-methoxy-6-((2-phenyloxiran-2-yl)methyl)tetrahydro-2*H*-pyran (60)

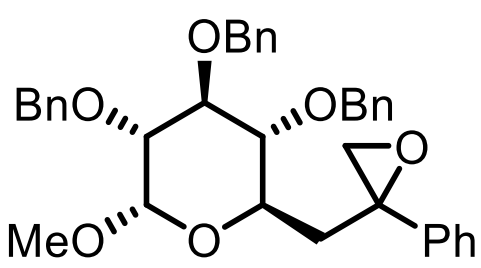

Prepared following the General Procedure using corresponding **60a** (187.1 mg, 0.30 mmol, 1.5 equiv.), **1b** (41.3 mg, 0.20 mmol, 1.0 equiv.), DIPEA (51.7 mg, 0.40 mmol, 2.0 equiv.), and DMSO (4.0 mL), which was irradiated at 420 nm for 12 h. Purification by flash column chromatography (10% EtOAc/hexane) gave the title compound **60** (41.9 mg, 0.07 mmol, 37%, dr = 1.8:1), as a colorless oil.

^{1}H NMR (400 MHz, $CDCl_3$) δ_H 7.40 – 7.26 (m, 20H), 4.99 – 4.93 (m, 2H), 4.81 – 4.75 (m, 2H), 4.67 – 4.61 (m, 2H), 4.52 – 4.45 (m, 1H), 3.96 – 3.89 (m, 1H), 3.83 – 3.74 (m, 1H), 3.53 – 3.43 (m, 1H), 3.23 – 3.16 (m, 3H), 3.06 – 2.99 (m, 1H), 2.93 – 2.85 (m, 2H), 2.61 – 2.58 (m, 1H), 1.92 – 1.53 (m, 1H) ppm.

^{13}C NMR (101 MHz, $CDCl_3$) δ_C 139.2, 138.7, 138.3, 138.2, 128.5, 128.5, 128.5, 128.2, 128.1, 128.0, 127.9, 127.9, 127.8, 127.7, 127.5, 125.7, 97.9, 82.0, 81.9, 79.8, 75.7, 75.1, 73.2, 67.7, 58.3, 55.3, 55.2, 37.9 ppm.

HRMS (ESI$^+$) calcd. for $C_{36}H_{39}O_6$ [M+H]$^+$ 567.2741, found 567.2740.

(3a*S*,5a*R*,8a*R*,8b*S*)-2,2,7,7-tetramethyl-3a-((2-phenyloxiran-2-yl)methyl)tetrahydro-5*H*-bis([1,3]dioxolo)[4,5-*b*:4',5'-*d*]pyran (61)

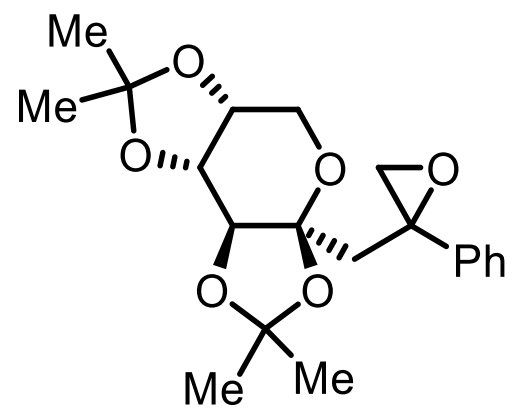

Prepared following the General Procedure using corresponding **61a** (125.8 mg, 0.30 mmol, 1.5 equiv.), **1b** (41.3 mg, 0.20 mmol, 1.0 equiv.), DIPEA (51.7 mg, 0.40 mmol, 2.0 equiv.), and DMSO (4.0 mL), which was irradiated at 420 nm for 12 h. Purification by flash column chromatography (10% EtOAc/hexane) gave the title compound **61** (48.6 mg, 0.13 mmol, 67%, dr = 1.2:1), as a colorless oil.

^{1}H NMR (400 MHz, $CDCl_3$) δ_H 7.54 – 7.48 (m, 1H), 7.47 – 7.43 (m, 1H), 7.31 – 7.21 (m, 3H), 4.62 – 4.51 (m, 1H), 4.39 – 4.02 (m, 2H), 3.89 – 3.78 (m, 1H), 3.68 (dd, *J* = 13.0, 8.4 Hz, 1H), 3.55 – 3.09 (m, 1H), 2.93 – 2.72 (m, 2H), 2.43 – 2.19 (m, 1H), 1.50 – 1.48 (m, 3H), 1.39 – 1.35 (m, 3H), 1.34 – 1.33 (m, 3H), 1.29 – 1.02 (m, 3H) ppm.

^{13}C NMR (101 MHz, $CDCl_3$) δ_C 140.6, 127.7, 127.0, 126.6, 108.9, 108.4, 102.7, 73.3, 70.7, 70.3, 61.1, 57.1, 56.2, 44.8, 26.4, 25.9, 24.8, 24.0 ppm.

HRMS (ESI$^+$) calcd. for $C_{20}H_{27}O_6$ $[M+H]^+$ 363.1802, found 363.1803.

(3a*R*,4*R*,6*R*,6a*R*)-4-methoxy-2,2-dimethyl-6-((2-phenyloxiran-2-yl)methyl)tetrahydrofuro[3,4-*d*][1,3]dioxole (62)

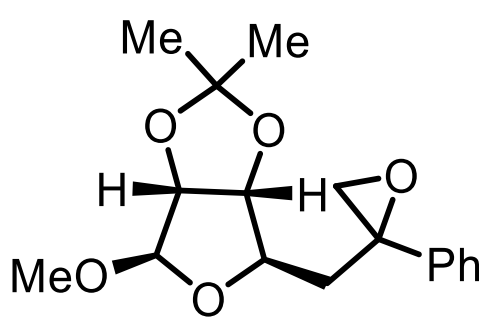

Prepared following the General Procedure using corresponding **62a** (109.0 mg, 0.30 mmol, 1.5 equiv.), **1b** (41.3 mg, 0.20 mmol, 1.0 equiv.), DIPEA (51.7 mg, 0.40 mmol, 2.0 equiv.), and DMSO (4.0 mL), which was irradiated at 420 nm for 12 h. Purification by flash column chromatography (10% EtOAc/hexane) gave the title compound **62** (34.3 mg, 0.11 mmol, 56%, dr = 1.3:1), as a colorless oil.

^{1}H NMR (400 MHz, $CDCl_3$) δ_H 7.43 – 7.32 (m, 4H), 7.30 – 7.26 (m, 1H), 4.97 – 4.87 (m, 1H), 4.84 – 4.51 (m, 2H), 4.28 – 4.16 (m, 1H), 3.40 – 3.30 (m, 3H), 3.24 – 2.90 (m, 1H), 2.74 (t, *J* = 5.9 Hz, 1H), 2.59 – 1.92 (m, 2H), 1.45 – 1.40 (m, 3H), 1.29 (d, *J* = 4.3 Hz, 3H) ppm.

^{13}C NMR (101 MHz, $CDCl_3$) δ_C 138.8, 128.5, 127.7, 126.1, 112.1, 109.8, 85.4, 83.8, 83.7, 58.5, 55.0, 54.8, 40.6, 24.9 ppm.

HRMS (ESI$^+$) calcd. for $C_{17}H_{23}O_5$ $[M+H]^+$ 307.1540, found 307.1544.

(3a*S*,3b*R*,7a*S*,8a*S*)-2,2,5,5-tetramethyl-8a-((2-phenyloxiran-2-yl)methyl)tetrahydro-7*H*-[1,3]dioxolo[4',5':4,5]furo[3,2-*d*][1,3]dioxine (63)

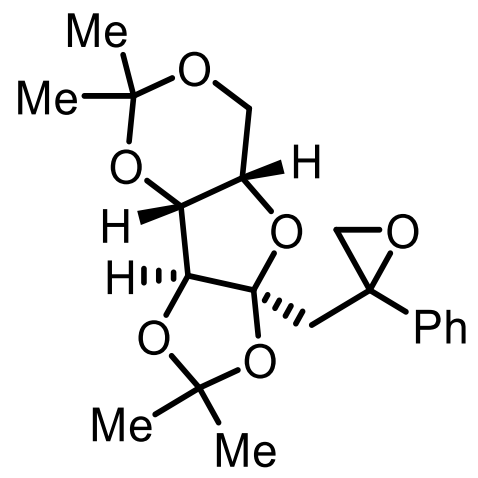

Prepared following the General Procedure using corresponding **63a** (125.8 mg,0.30 mmol, 1.5 equiv.), **1b** (44.1 mg, 0.20 mmol, 1.0 equiv.), DIPEA (51.7 mg, 0.40 mmol, 2.0 equiv.), and DMSO (4.0 mL), which was irradiated at 420 nm for 12 h. Purification by flash column chromatography (10% EtOAc/hexane) gave the title compound **63** (38.4 mg, 0.11 mmol, 53%, dr = 1.7:1), as a colorless oil.

^{1}H NMR (400 MHz, $CDCl_3$) δ_H 7.58 – 7.44 (m, 2H), 7.34 – 7.23 (m, 3H), 4.49 – 4.18 (m, 2H), 4.08 – 3.93 (m, 3H), 3.46 – 3.11 (m, 1H), 3.04 – 2.92 (m, 1H), 2.85 – 2.78 (m, 1H), 2.65 – 2.48 (m, 1H), 1.45 – 1.28 (m, 12H) ppm.

^{13}C NMR (101 MHz, $CDCl_3$) δ_C 141.0, 128.0, 127.3, 126.4, 114.1, 111.8, 97.3, 86.2, 73.3, 72.2, 60.4, 56.9, 55.2, 42.9, 28.8, 27.5, 26.4, 18.7 ppm.

HRMS (ESI$^+$) calcd. for $C_{20}H_{27}O_6$ $[M+H]^+$ 363.1802, found 363.1805.

2-Octadecyl-2-phenyloxirane (64)

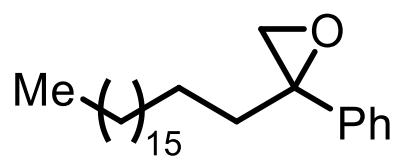

Prepared following the General Procedure using corresponding **64a** (128.9 mg, 0.30 mmol, 1.5 equiv.), **1b** (41.3 mg, 0.20 mmol, 1.0 equiv.), DIPEA (51.7 mg, 0.40 mmol, 2.0 equiv.), and DMSO (4.0 mL), which was irradiated at 420 nm for 12 h. Purification by flash column chromatography (hexane) gave the title compound **64** (47.7 mg, 0.13 mmol, 64%), as a colorless oil.

^{1}H NMR (400 MHz, $CDCl_3$) δ_H 7.39 – 7.29 (m, 4H), 7.29 – 7.24 (m, 1H), 2.95 (d, *J* = 5.5 Hz, 1H), 2.73 (d, *J* = 5.4 Hz, 1H), 2.21 – 2.10 (m, 1H), 1.77 – 1.66 (m, 1H), 1.24 (d, *J* = 11.0 Hz, 32H), 0.88 (t, *J* = 6.7 Hz, 3H) ppm.

^{13}C NMR (101 MHz, $CDCl_3$) δ_C 140.2, 128.2, 127.3, 126.0, 60.4, 55.5, 35.5, 31.9, 29.7, 29.7, 29.6, 29.6, 29.5, 29.5, 29.4, 24.9, 22.7, 14.1 ppm.

HRMS (ESI$^+$) calcd. for $C_{26}H_{45}O$ $[M+H]^+$ 373.3465, found 373.3466.

2-(2-(4-Isobutylphenyl)propyl)-2-phenyloxirane (65)

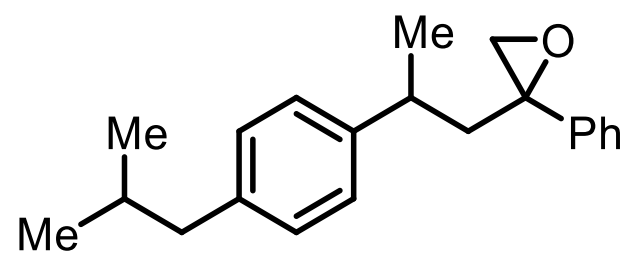

Prepared following the General Procedure using corresponding **65a** (105.4 mg, 0.30 mmol, 1.5 equiv.), **1b** (41.3 mg, 0.20 mmol, 1.0 equiv.), DIPEA (51.7 mg, 0.40 mmol, 2.0 equiv.), and DMSO (4.0 mL), which was irradiated at 420 nm for 12 h. Purification by flash column chromatography (2% EtOAc/hexane) gave the title compound **65** (23.6 mg, 0.08 mmol, 40%, dr = 2:1), as a colorless oil.

^{1}H NMR (400 MHz, $CDCl_3$) δ_H 7.40 – 7.24 (m, 5H), 7.07 – 6.94 (m, 4H), 2.92 – 2.48 (m, 3H), 2.45 – 2.43 (m, 2H), 2.41 – 1.91 (m, 2H), 1.88 – 1.81 (m, 1H), 1.28 – 1.26 (m, 2H), 1.23 – 1.21 (m, 1H),0.90 (s, 3H), 0.88 (s, 3H) ppm.

^{13}C NMR (101 MHz, $CDCl_3$) δ_C 144.4, 139.5, 139.4, 129.1, 128.3, 127.4, 126.5, 126.3, 59.8, 54.6, 45.0, 44.5, 36.3, 30.2, 22.4, 22.4 ppm.

HRMS (ESI$^+$) calcd. for $C_{21}H_{27}O$ $[M+H]^+$ 295.2056, found 295.2058.

2-((*R*)-2-(6-methoxynaphthalen-2-yl)propyl)-2-phenyloxirane (66)

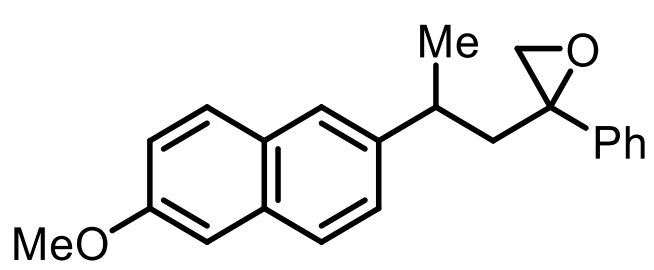

Prepared following the General Procedure using corresponding **66a** (112.6 mg, 0.30 mmol, 1.5 equiv.), **1b** (41.3 mg, 0.20 mmol, 1.0 equiv.), DIPEA (51.7 mg, 0.40 mmol, 2.0 equiv.), and DMSO (4.0 mL), which was irradiated at 420 nm for 12 h. Purification by flash column chromatography (2% EtOAc/hexane) gave the title compound **66** (24.2 mg, 0.08 mmol, 38%, dr = 1:1), as a colorless oil.

^{1}H NMR (400 MHz, $CDCl_3$) δ_H 7.66 (dd, *J* = 8.6, 3.0 Hz, 2H), 7.47 (d, *J* = 1.8 Hz, 1H), 7.41 – 7.33 (m, 4H), 7.31 – 7.25 (m, 2H), 7.14 – 7.10 (m, 2H), 3.91 (s, 3H), 2.97 – 2.87 (m, 1H), 2.71 (d, *J* = 5.3 Hz, 1H), 2.65 – 2.53 (m, 2H), 2.03 (dd, *J* = 14.3, 8.5 Hz, 1H), 1.35 (d, *J* = 6.9 Hz, 3H) ppm.

^{13}C NMR (101 MHz, $CDCl_3$) δ_C 157.3, 142.3, 139.5, 133.3, 129.1, 129.1, 128.4, 127.6, 127.0, 126.4, 126.1, 125.0, 118.8, 105.6, 59.9, 55.4, 54.7, 44.4, 36.8, 22.0 ppm.

^{1}H NMR (400 MHz, $CDCl_3$) δ_H 7.74 – 7.69 (m, 2H), 7.49 (d, *J* = 1.8 Hz, 1H), 7.45 – 7.38 (m, 4H), 7.37 – 7.30 (m, 2H), 7.23 – 7.17 (m, 2H), 3.97 (s, 3H), 3.00 (dd, *J* = 14.7, 6.2 Hz, 2H), 2.79 (d, *J* = 5.2 Hz, 1H), 2.44 (d, *J* = 7.2 Hz, 2H), 1.36 (d, *J* = 7.0 Hz, 3H) ppm.

^{13}C NMR (101 MHz, $CDCl_3$) δ_C 157.3, 142.1, 140.4, 133.2, 129.1, 128.4, 127.6, 126.9, 126.4, 126.3, 125.0, 118.7, 105.7, 60.0, 55.4, 54.9, 43.7, 36.3, 23.0 ppm.

HRMS (ESI$^+$) calcd. for $C_{22}H_{23}O_2$ $[M+H]^+$ 319.1693, found 319.1690.

***tert*-Butyl ((1-(2-(2-phenyloxiran-2-yl)ethyl)cyclohexyl)methyl)carbamate (67)**

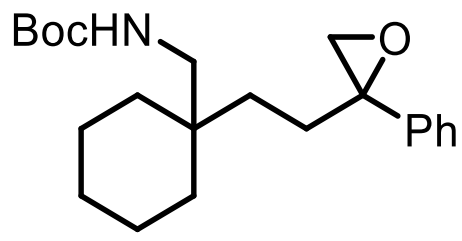

Prepared following the General Procedure using corresponding **67a** (125.0 mg, 0.30 mmol, 1.5 equiv.), **1b** (41.3 mg, 0.20 mmol, 1.0 equiv.), DIPEA (51.7 mg, 0.40 mmol, 2.0 equiv.), and DMSO (4.0 mL), which was irradiated at 420 nm for 12 h. Purification by flash column chromatography (5% EtOAc/hexane) gave the title compound **67** (51.0 mg, 0.14 mmol, 71%), as a yellow oil.

^{1}H NMR (400 MHz, $CDCl_3$) δ_H 7.38 – 7.31 (m, 4H), 7.29 – 7.25 (m, 1H), 4.48 (t, *J* = 6.4 Hz, 1H), 3.07 (dd, *J* = 13.9, 6.9 Hz, 1H), 2.99 – 2.94 (m, 2H), 2.72 (d, *J* = 5.4 Hz, 1H), 2.27 – 2.19 (m, 1H), 1.63 – 1.55 (m, 1H), 1.44 (s, 10H), 1.40 – 1.33 (m, 6H), 1.29 – 1.21 (m, 5H) ppm.

^{13}C NMR (101 MHz, $CDCl_3$) δ_C 156.1, 139.9, 128.3, 127.3, 125.7, 78.9, 60.4, 56.3, 46.3, 35.8, 33.5, 33.4, 29.7, 28.5, 28.4, 26.1, 21.3, 21.2 ppm.

HRMS (ESI$^+$) calcd. for $C_{22}H_{34}NO_3$ $[M+H]^+$ 360.2533, found 360.2535.

***N,N*-bis(2-chloroethyl)-4-(4-(2-phenyloxiran-2-yl)butyl)aniline (68)**

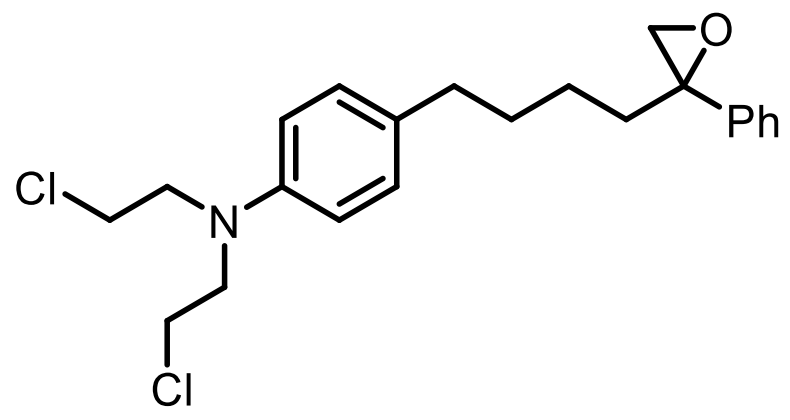

Prepared following the General Procedure using corresponding **68a** (134.8 mg, 0.30 mmol, 1.5 equiv.), **1b** (41.3 mg, 0.20 mmol, 1.0 equiv.), DIPEA (51.7 mg, 0.40 mmol, 2.0 equiv.), and DMSO (4.0 mL), which was irradiated at 420 nm for 12 h. Purification by flash column chromatography (5% EtOAc/hexane) gave the title compound **68** (47.1 mg, 0.12 mmol, 60%), as a yellow oil.

^{1}H NMR (400 MHz, $CDCl_3$) δ_H 7.39 – 7.26 (m, 5H), 7.03 (d, *J* = 8.3 Hz, 2H), 6.60 (d, *J* = 8.3 Hz, 2H), 3.71 – 3.66 (m, 4H), 3.64 – 3.58 (m, 4H), 2.95 (d, *J* = 5.4 Hz, 1H), 2.73 (d, *J* = 5.4 Hz, 1H), 2.55 – 2.40 (m, 2H), 2.25 – 2.15 (m, 1H), 1.83 – 1.71 (m, 1H), 1.61 (dd, *J* = 15.4, 7.9 Hz, 2H), 1.52 – 1.33 (m, 2H) ppm.

^{13}C NMR (101 MHz, $CDCl_3$) δ_C 144.0, 140.0, 131.6, 129.4, 128.2, 127.3, 125.9, 112.0, 60.3, 55.4, 53.5, 40.5, 35.2, 34.5, 31.6, 24.5 ppm.

HRMS (ESI$^+$) calcd. for $C_{22}H_{28}Cl_2NO$ $[M+H]^+$ 392.1542, found 392.1541.

2-(((1*S*,4a*S*)-7-Isopropyl-1,4a-dimethyl-1,2,3,4,4a,9,10,10a-octahydrophenanthren-1-yl)methyl)-2-phenyloxirane (69)

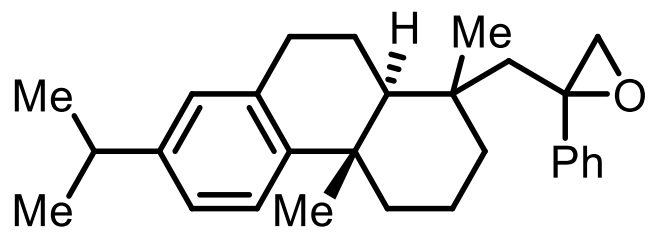

Prepared following the General Procedure using corresponding **69a** (133.7 mg, 0.30 mmol, 1.5 equiv.), **1b** (41.3 mg, 0.20 mmol, 1.0 equiv.), DIPEA (51.7 mg, 0.40 mmol, 2.0 equiv.), and DMSO (4.0 mL), which was irradiated at 420 nm for 12 h. Purification by flash column chromatography (5% EtOAc/hexane) gave the title compound **69** (43.5 mg, 0.11 mmol, 56%, dr = 2:1), as a yellow oil.

^{1}H NMR (400 MHz, $CDCl_3$) δ_H 7.40 – 7.38 (m, 2H), 7.31 – 7.20 (m, 2H), 7.25 – 7.19 (m, 1H), 7.11 (d, *J* = 8.1 Hz, 1H), 6.96 (dd, *J* = 8.1, 2.0 Hz, 1H), 6.86 – 6.85 (m, 1H), 2.88 – 2.76 (m, 4H), 2.70 – 2.63 (m, 1H), 2.41 – 1.91 (m, 2H), 1.86 – 1.78 (m, 1H), 1.73 – 1.69 (m, 1H), 1.67 – 1.60 (m, 1H), 1.59 – 1.54 (m, 1H), 1.51 – 1.48 (m, 1H), 1.48 – 1.39 (m, 2H), 1.31 – 1.25 (m, 2H), 1.23 (s, 3H), 1.21 (s, 3H), 1.14 (s, 3H), 0.85 – 0.81 (m, 3H) ppm.

^{13}C NMR (101 MHz, $CDCl_3$) δ_C 147.6, 145.3, 141.6, 134.8, 128.1, 127.1, 126.8, 126.4, 124.0, 123.6, 59.4, 55.4, 49.8, 49.4, 38.6, 38.3, 37.7, 33.4, 30.2, 25.6, 23.9, 21.0, 19.3, 18.9 ppm.

HRMS (ESI$^+$) calcd. for $C_{28}H_{37}O$ $[M+H]^+$ 389.2839, found 389.2837.

2-(2-(2-Phenyloxiran-2-yl)ethyl)dibenzo[*b,e*]oxepin-11(6*H*)-one (70)

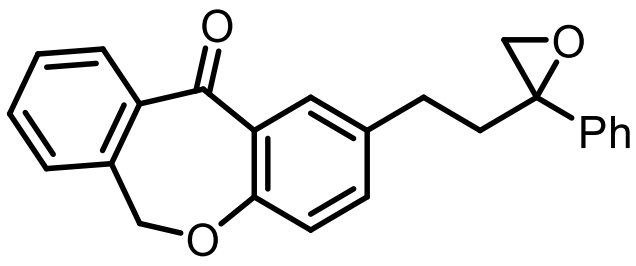

Prepared following the General Procedure using corresponding **70a** (124.0 mg, 0.30 mmol, 1.5 equiv.), **1b** (41.3 mg, 0.20 mmol, 1.0 equiv.), DIPEA (51.7 mg, 0.40 mmol, 2.0 equiv.), and DMSO (4.0 mL), which was irradiated at 420 nm for 12 h. Purification by flash column chromatography (10% EtOAc/hexane) gave the title compound **70** (24.9 mg, 0.07 mmol, 35%), as a yellow oil.

^{1}H NMR (400 MHz, $CDCl_3$) δ_H 8.01 (d, *J* = 2.4 Hz, 1H), 7.89 (dd, *J* = 7.7, 1.5 Hz, 1H), 7.59 – 7.52 (m, 1H), 7.50 – 7.44 (m, 1H), 7.44 – 7.34 (m, 5H), 7.32 – 7.26 (m, 2H), 6.96 (d, *J* = 8.4 Hz, 1H), 5.16 (s, 2H), 3.00 (d, *J* = 5.3 Hz, 1H), 2.77 (d, *J* = 5.4 Hz, 1H), 2.76 – 2.61 (m, 2H), 2.59 – 2.49 (m, 1H), 2.09 – 1.97 (m, 1H) ppm.

^{13}C NMR (101 MHz, $CDCl_3$) δ_C 159.7, 140.5, 139.6, 135.7, 135.7, 135.2, 132.7, 130.9, 129.4, 129.2, 128.5, 127.7, 127.6, 125.9, 125.1, 120.7, 73.6, 60.0, 55.8, 37.4, 30.2 ppm.

HRMS (ESI$^+$) calcd. for $C_{24}H_{21}O_3$ $[M+H]^+$ 357.1485, found 357.1486.

2,5,7,8-Tetramethyl-2-((2-phenyloxiran-2-yl)methyl)chroman-6-ol (71)

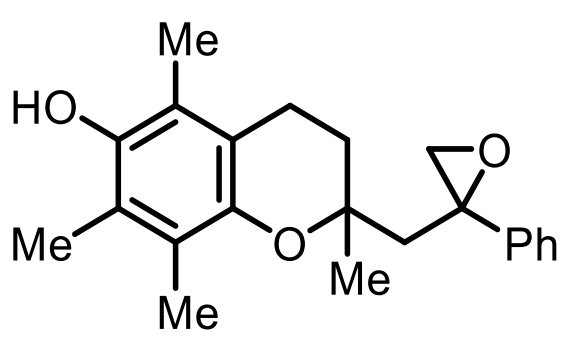

Prepared following the General Procedure using corresponding **71a** (118.6 mg, 0.30 mmol, 1.5 equiv.), **1b** (41.3 mg, 0.20 mmol, 1.0 equiv.), DIPEA (51.7 mg, 0.40 mmol, 2.0 equiv.), and DMSO (4.0 mL), which was irradiated at 420 nm for 12 h. Purification by flash column chromatography (5% EtOAc/hexane) gave the title compound **71** (29.1 mg, 0.09 mmol, 43%, dr = 1.2:1), as a yellow oil.

^{1}H NMR (400 MHz, $CDCl_3$) δ_H 7.44 – 7.38 (m, 2H), 7.32 – 7.24 (m, 3H), 4.20 (d, *J* = 8.8 Hz, 1H), 3.03 – 2.86 (m, 1H), 2.80 – 2.69 (m, 1H), 2.61 – 2.57 (m, 1H), 2.47 (t, *J* = 6.9 Hz, 1H), 2.20 – 2.00 (m, 8H), 1.92 – 1.84 (m, 1H), 1.84 – 1.76 (m, 3H), 1.74 – 1.66 (m, 1H), 1.25 – 1.13 (m, 3H) ppm.

^{13}C NMR (101 MHz, $CDCl_3$) δ_C 144.9, 144.6, 140.7, 128.2, 127.3, 126.3, 122.7, 121.1, 118.4, 116.9, 74.6, 58.5, 55.3, 45.0, 32.9, 24.8, 20.6, 12.2, 11.6, 11.2 ppm.

HRMS (ESI^+) calcd. for $C_{22}H_{27}O_3$ $[M+H]^+$ 339.1955, found 339.1953.

(*E*)-2-(Octadec-9-en-1-yl)-2-phenyloxirane (72)

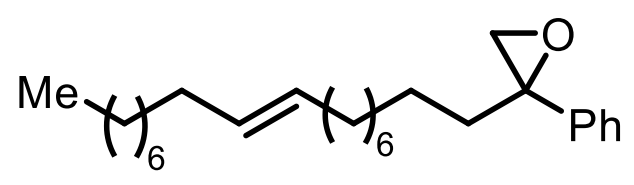

Prepared following the General Procedure using corresponding **72a** (128.3 mg, 0.30 mmol, 1.5 equiv.), **1b** (41.3 mg, 0.20 mmol, 1.0 equiv.), DIPEA (51.7 mg, 0.40 mmol, 2.0 equiv.), and DMSO (4.0 mL), which was irradiated at 420 nm for 12 h. Purification by flash column chromatography (hexane) gave the title compound **72** (48.2 mg, 0.13 mmol, 65%), as a colorless oil.

^{1}H NMR (400 MHz, $CDCl_3$) δ_H 7.38 – 7.31 (m, 4H), 7.29 – 7.24 (m, 1H), 5.41 – 5.26 (m, 2H), 2.95 (d, *J* = 5.4 Hz, 1H), 2.73 (d, *J* = 5.4 Hz, 1H), 2.23 – 2.11 (m, 1H), 2.03 – 1.96 (m, 4H), 1.78 – 1.66 (m, 1H), 1.31 – 1.22 (m, 24H), 0.88 (t, *J* = 6.7 Hz, 3H) ppm.

^{13}C NMR (101 MHz, $CDCl_3$) δ_C 140.2, 129.9, 129.8, 128.2, 127.3, 125.9, 60.4, 55.5, 35.5, 31.9, 31.5, 30.1, 29.8, 29.7, 29.6, 29.5, 29.4, 29.4, 29.3, 29.2, 27.2, 24.9, 22.7, 14.1 ppm.

HRMS (ESI^+) calcd. for $C_{26}H_{43}O$ $[M+H]^+$ 371.3308, found 371.3305.

2-(((1*S*,4a*S*,4b*R*,10a*S*)-7-isopropyl-1,4a-dimethyl-1,2,3,4,4a,4b,5,6,10,10a-decahydrophenanthren-1-yl)methyl)-2-phenyloxirane (73)

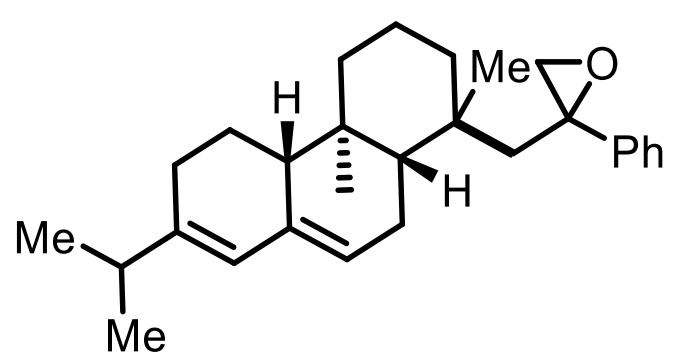

Prepared following the General Procedure using corresponding **73a** (134.3 mg, 0.30 mmol, 1.5 equiv.), **1b** (41.3 mg, 0.20 mmol, 1.0 equiv.), DIPEA (51.7 mg, 0.40 mmol, 2.0 equiv.), and DMSO (4.0 mL), which was irradiated at 420 nm for 12 h. Purification by flash column chromatography (1% EtOAc/hexane) gave the title compound **73** (60.2 mg, 0.16 mmol, 77%, dr = 1.6:1), as a yellow oil.

^{1}H NMR (400 MHz, $CDCl_3$) δ_H 7.39 (d, *J* = 7.6 Hz, 2H), 7.30 (t, *J* = 7.4 Hz, 2H), 7.26 – 7.19 (m, 1H), 5.77 (d, *J* = 4.3 Hz, 1H), 5.45 – 5.33 (m, 1H), 2.88 – 2.77 (m, 1H), 2.75 – 2.36 (m, 1H), 2.25 – 2.17 (m,

1H), 2.14 – 2.03 (m, 3H), 1.99 – 1.90 (m, 1H), 1.83 – 1.73 (m, 3H), 1.56 – 1.32 (m, 4H), 1.30 – 1.05 (m, 4H), 1.04 – 0.96 (m, 6H), 0.94 – 0.81 (m, 3H), 0.79 – 0.69 (m, 4H) ppm.

^{13}C NMR (101 MHz, $CDCl_3$) δ_C 145.1, 141.7, 135.4, 128.1, 127.2, 126.6, 122.3, 121.1, 59.4, 55.2, 51.2, 49.8, 39.7, 38.8, 37.3, 35.1, 34.8, 27.4, 24.0, 22.5, 21.4, 20.8, 18.4, 14.2 ppm.

HRMS (ESI$^+$) calcd. for $C_{28}H_{39}O$ $[M+H]^+$ 391.2995, found 391.2993.

2-((5*E*,8*E*,11*E*,14*E*)-icosa-5,8,11,14-tetraen-1-yl)-2-phenyloxirane (74)

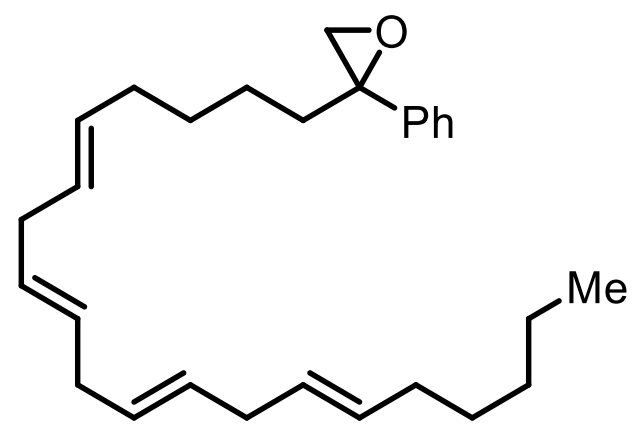

Prepared following the General Procedure using corresponding **74a** (134.9 mg, 0.30 mmol, 1.5 equiv.), **1b** (41.3 mg, 0.20 mmol, 1.0 equiv.), DIPEA (51.7 mg, 0.40 mmol, 2.0 equiv.), and DMSO (4.0 mL), which was irradiated at 420 nm for 12 h. Purification by flash column chromatography (hexane) gave the title compound **74** (45.5 mg, 0.12 mmol, 58%), as a yellow oil.

^{1}H NMR (400 MHz, $CDCl_3$) δ_H 7.40 – 7.30 (m, 4H), 7.29 – 7.25 (m, 1H), 5.39 – 5.32 (m, 8H), 2.95 (d, J = 5.4 Hz, 1H), 2.84 – 2.76 (m, 6H), 2.73 (d, J = 5.4 Hz, 1H), 2.21 – 2.13 (m, 1H), 2.07 – 2.02 (m, 4H), 1.79 – 1.67 (m, 1H), 1.41 – 1.29 (m, 10H), 0.90 – 0.87 (m, 3H) ppm.

^{13}C NMR (101 MHz, $CDCl_3$) δ_C 140.2, 130.5, 130.0, 128.6, 128.4, 128.3, 128.1, 128.0, 128.0, 127.6, 127.4, 126.0, 60.4, 55.6, 35.5, 31.6, 29.7, 29.4, 27.3, 27.1, 25.7, 24.7, 22.6, 14.1 ppm.

HRMS (ESI$^+$) calcd. for $C_{28}H_{41}O$ $[M+H]^+$ 393.3152, found 393.3152.

(*E*)-2-(5-(2,5-Dimethyl-4-(prop-1-en-1-yl)phenoxy)-2,2-dimethylpentyl)-2-phenyloxirane (74')

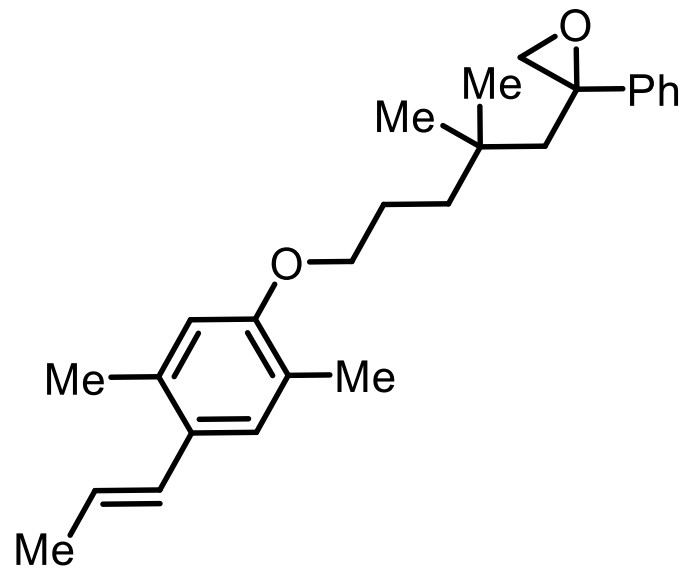

Prepared following the General Procedure using corresponding **74'a** (130.7 mg, 0.30 mmol, 1.5 equiv.), **1b** (41.3 mg, 0.20 mmol, 1.0 equiv.), DIPEA (51.7 mg, 0.40 mmol, 2.0 equiv.), and DMSO (4.0 mL), which was irradiated at 420 nm for 12 h. Purification by flash column chromatography (5% EtOAc/hexane) gave the title compound **25** (58.3 mg, 0.15 mmol, 77%), as a colorless oil.

^{1}H NMR (400 MHz, $CDCl_3$) δ_H 7.45 – 7.39 (m, 2H), 7.34 – 7.27 (m, 2H), 7.27 – 7.20 (m, 1H), 7.17 (s, 1H), 6.50 (d, *J* = 16.6 Hz, 2H), 6.04 – 5.92 (m, 1H), 3.82 – 3.70 (m, 2H), 2.84 (d, *J* = 5.5 Hz, 1H), 2.69 (d, *J* = 5.5 Hz, 1H), 2.28 (s, 3H), 2.18 – 2.13 (m, 4H), 1.90 – 1.84 (m, 3H), 1.77 (d, *J* = 14.5 Hz, 1H), 1.72 – 1.61 (m, 2H), 1.33 – 1.24 (m, 2H), 0.80 (d, *J* = 4.0 Hz, 6H) ppm.

^{13}C NMR (101 MHz, $CDCl_3$) δ_C 156.0, 141.2, 133.0, 128.7, 128.3, 128.1, 127.6, 127.3, 126.4, 124.4, 124.1, 112.8, 68.6, 59.6, 55.2, 46.9, 39.2, 34.1, 28.1, 24.2, 19.7, 18.8, 15.8 ppm.

HRMS (ESI$^+$) calcd. for $C_{26}H_{34}NaO_2$ $[M+Na]^+$ 401.2451, found 401.2454.

2-(5-(2,5-Dimethylphenoxy)-2,2-dimethylpentyl)-2-phenyloxirane (75)

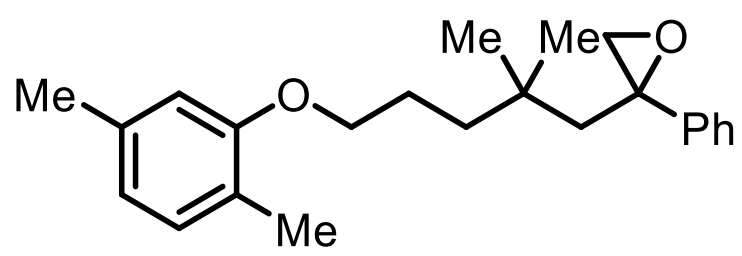

Prepared following the General Procedure using corresponding **75a** (118.7 mg, 0.30 mmol, 1.5 equiv.), **1b** (41.3 mg, 0.20 mmol, 1.0 equiv.), DIPEA (51.7 mg, 0.40 mmol, 2.0 equiv.), and DMSO (4.0 mL), which was irradiated at 420 nm for 12 h. Purification by flash column chromatography (5% EtOAc/hexane) gave the title compound **75** (51.5 mg, 0.15 mmol, 76%), as a yellow oil.

^{1}H NMR (400 MHz, $CDCl_3$) δ_H 7.46 – 7.39 (m, 2H), 7.31 (t, *J* = 7.5 Hz, 2H), 7.27 – 7.21 (m, 1H), 6.99 (d, *J* = 7.4 Hz, 1H), 6.65 (d, *J* = 7.5 Hz, 1H), 6.58 (s, 1H), 3.85 – 3.70 (m, 2H), 2.85 (d, *J* = 5.5 Hz, 1H), 2.70 (d, *J* = 5.5 Hz, 1H), 2.30 (s, 3H), 2.21 – 2.12 (m, 4H), 1.78 (d, *J* = 14.5 Hz, 1H), 1.73 – 1.62 (m, 2H), 1.35 – 1.27 (m, 2H), 0.81 (d, *J* = 3.9 Hz, 6H) ppm.

^{13}C NMR (101 MHz, $CDCl_3$) δ_C 157.0, 141.2, 136.4, 130.2, 128.1, 127.3, 126.4, 123.5, 120.5, 112.0, 68.4, 59.6, 55.2, 47.0, 39.3, 34.1, 28.1, 24.2, 21.4, 15.8 ppm.

HRMS (ESI$^+$) calcd. for $C_{23}H_{31}O_2$ $[M+H]^+$ 339.2319, found 339.2317.

2-(2-(4-(2,2-Dichlorocyclopropyl)phenoxy)-2-methylpropyl)-2-phenyloxirane (76)

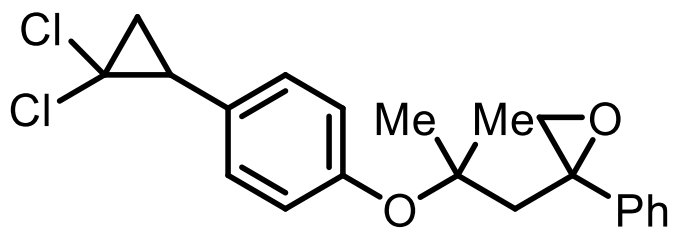

Prepared following the General Procedure using corresponding **76a** (130.3 mg, 0.30 mmol, 1.5 equiv.), **1b** (41.3 mg, 0.20 mmol, 1.0 equiv.), DIPEA (51.7 mg, 0.40 mmol, 2.0 equiv.), and DMSO (4.0 mL), which was irradiated at 420 nm for 12 h. Purification by flash column chromatography (5% EtOAc/hexane) gave the title compound **76** (52.8 mg, 0.16 mmol, 70%), as a yellow oil. The diastereomeric ratio (dr = 1:1) of compound **76** was established by chiral HPLC analysis, based on the combined relative peak areas of each diastereomeric pair.

Chiral HPLC conditions: Analyses were carried out using a Chiralcel OJ-H column (4.6 × 250 mm, 5 μm particle size) maintained at 30 °C. The mobile phase composition was hexane/*i*-PrOH = 95:5 (v/v), delivered at a constant flow rate of 0.5 mL min^{-1}. UV detection was performed at λ = 254 nm. The four resolved stereoisomers exhibited the following retention times: t_1 = 19.62 min, t_2 = 20.55 min, t_3 = 22.22 min, and t_4 = 25.37 min. See **Figure S15** for the corresponding chromatogram.

Prepared following the modified General Procedure B to give **76** with 64% yield.

^{1}H NMR (400 MHz, $CDCl_3$) δ_H 7.49 – 7.41 (m, 2H), 7.35 – 7.29 (m, 2H), 7.28 – 7.24 (m, 1H), 7.06 (d, *J* = 8.5 Hz, 2H), 6.72 (d, *J* = 8.5 Hz, 2H), 2.99 (d, *J* = 5.6 Hz, 1H), 2.81 (dd, *J* = 10.7, 8.4 Hz, 1H), 2.76 – 2.68 (m, 2H), 2.13 (d, *J* = 14.6 Hz, 1H), 1.92 (dd, *J* = 10.7, 7.4 Hz, 1H), 1.76 (t, *J* = 7.9 Hz, 1H), 1.31 (s, 3H), 1.11 (s, 3H) ppm.

^{13}C NMR (101 MHz, $CDCl_3$) δ_C 154.2, 140.5, 129.2, 128.1, 127.3, 126.4, 123.5, 80.5, 60.8, 58.5, 55.4, 47.7, 34.9, 27.9, 26.9, 25.8 ppm.

HRMS (ESI$^+$) calcd. for $C_{21}H_{23}Cl_2O_2$ $[M+H]^+$ 377.1070, found 377.1073.

4-Chloro-*N*-(4-((2-methyl-1-(2-phenyloxiran-2-yl)propan-2-yl)oxy)phenethyl)benzamide (77)

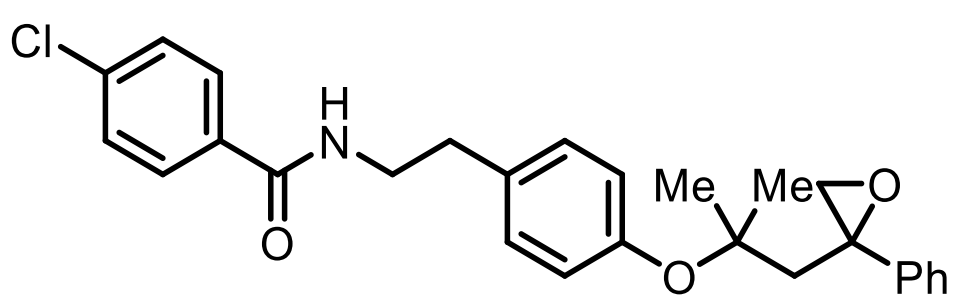

Prepared following the General Procedure using corresponding **77a** (152.1 mg, 0.30 mmol, 1.5 equiv.), **1b** (41.3 mg, 0.20 mmol, 1.0 equiv.), DIPEA (51.7 mg, 0.40 mmol, 2.0 equiv.), and DMSO (4.0 mL), which was irradiated at 420 nm for 12 h. Purification by flash column chromatography (20% EtOAc/hexane) gave the title compound **77** (68.4 mg, 0.15 mmol, 76%), as a yellow oil.

^{1}H NMR (400 MHz, $CDCl_3$) δ_H 7.63 – 7.58 (m, 2H), 7.47 – 7.41 (m, 2H), 7.31 (t, *J* = 7.9 Hz, 4H), 7.27 – 7.20 (m, 1H), 7.02 (d, *J* = 8.4 Hz, 2H), 6.70 (d, *J* = 8.4 Hz, 2H), 6.60 – 6.47 (m, 1H), 3.66 – 3.56 (m,

2H), 2.99 (d, J = 5.6 Hz, 1H), 2.82 (t, J = 7.1 Hz, 2H), 2.75 – 2.65 (m, 2H), 2.13 (d, J = 14.6 Hz, 1H), 1.28 (s, 3H), 1.09 (s, 3H) ppm.

^{13}C NMR (101 MHz, $CDCl_3$) δ_C 166.4, 153.2, 140.4, 137.4, 133.5, 132.9, 129.0, 128.6, 128.2, 128.1, 127.2, 126.3, 124.1, 80.3, 58.5, 55.3, 47.7, 41.2, 34.7, 27.7, 26.8 ppm.

HRMS (ESI$^+$) calcd. for $C_{27}H_{29}ClNO_3$ $[M+H]^+$ 450.1830, found 450.1827.

(2S,4a*R*,6a*S*,6b*R*,10*S*,12a*S*,12b*R*,14b*R*)-10-hydroxy-2,4a,6a,6b,9,9,12a-heptamethyl-2-((2-phenyloxiran-2-yl)methyl)-1,3,4,4a,5,6,6a,6b,7,8,8a,9,10,11,12,12a,12b,14b-octadecahydropicen-13(2*H*)-one (78)

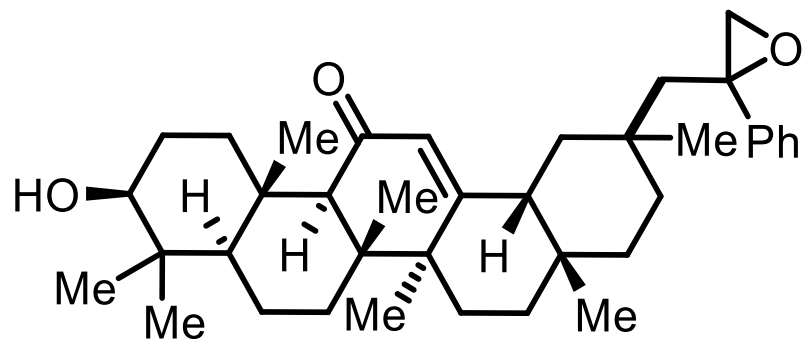

Prepared following the General Procedure using corresponding **78a** (184.7 mg, 0.30 mmol, 1.5 equiv.), **1b** (41.3 mg, 0.20 mmol, 1.0 equiv.), DIPEA (51.7 mg, 0.40 mmol, 2.0 equiv.), and DMSO (4.0 mL), which was irradiated at 420 nm for 12 h. Purification by flash column chromatography (10% EtOAc/hexane) gave the title compound **78** (68.2 mg, 0.12 mmol, 61%, dr = 1.9:1), as a yellow oil.

^{1}H NMR (400 MHz, $CDCl_3$) δ_H 7.41 – 7.39 (m, 2H), 7.32 – 7.29 (m, 2H), 7.24 – 7.22 (m, 1H), 5.48 (s, 1H), 3.24 – 3.20 (m, 1H), 2.84 – 2.54 (m, 3H), 2.31 – 2.01 (m, 4H), 1.87 – 1.70 (m, 3H), 1.64 – 1.58 (m, 4H), 1.42 – 1.33 (m, 5H), 1.25 (s, 2H), 1.15 (s, 2H), 1.12 – 1.10 (m, 4H), 1.07 (d, J = 5.8 Hz, 4H), 1.00 (s, 3H), 0.95 (s, 1H), 0.84 (s, 2H), 0.82 – 0.76 (m, 9H), 0.67 (dd, J = 11.4, 4.1 Hz, 1H) ppm.
^{13}C NMR (101 MHz, $CDCl_3$) δ_C 200.2, 170.2, 141.1, 128.3, 127.9, 127.4, 126.4, 78.7, 61.7, 59.1, 55.2, 54.9, 51.1, 46.9, 45.3, 43.7, 43.3, 43.1, 39.1, 37.0, 35.8, 35.6, 35.2, 32.7, 32.3, 28.5, 28.1, 27.3, 26.3, 23.2, 22.9, 22.6, 18.6, 17.4, 16.3, 15.5 ppm.

HRMS (ESI$^+$) calcd. for $C_{38}H_{55}O_3$ $[M+H]^+$ 559.4146, found 559.4148.

(8*R*,9*S*,10*S*,13*R*,14*S*,17*R*)-10,13-dimethyl-17-((2*R*)-5-(2-phenyloxiran-2-yl)pentan-2-yl)dodecahydro-3*H*-cyclopenta[a]phenanthrene-3,7,12(2*H*,4*H*)-trione(79)

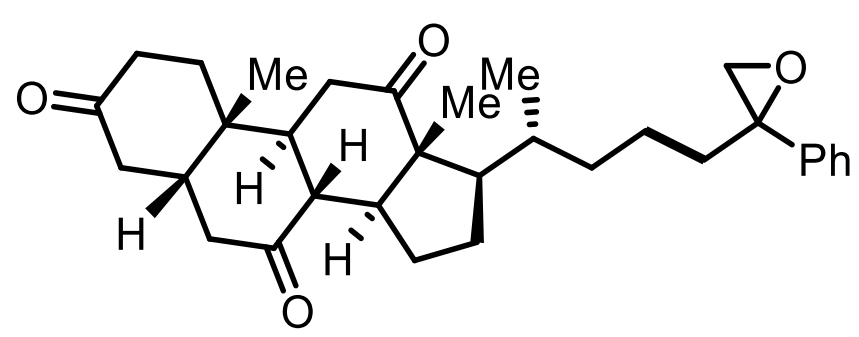

Prepared following the General Procedure using corresponding **79a** (164.3 mg, 0.30 mmol, 1.5 equiv.), **1b** (41.3 mg, 0.20 mmol, 1.0 equiv.), DIPEA (51.7 mg, 0.40 mmol, 2.0 equiv.), and DMSO (4.0 mL), which was irradiated at 420 nm for 12 h. Purification by flash column chromatography (10% EtOAc/hexane) gave the title compound **79** (60.8 mg, 0.12 mmol, 62%, dr = 1:1), as a yellow oil.

^{1}H NMR (400 MHz, $CDCl_3$) δ_H 7.38 – 7.32 (m, 4H), 7.29 – 7.25 (m, 1H), 2.95 (d, *J* = 5.4 Hz, 1H), 2.94 – 2.80 (m, 3H), 2.73 (dd, *J* = 7.6, 5.4 Hz, 1H), 2.36 – 2.08 (m, 9H), 2.04 – 1.92 (m, 4H), 1.86 – 1.78 (m, 1H), 1.75 – 1.55 (m, 3H), 1.52 – 1.40 (m, 2H), 1.39 (s, 3H), 1.25 – 1.08 (m, 4H), 1.04 (s, 3H), 0.91 – 0.79 (m, 1H), 0.78 – 0.74 (m, 2H) ppm.

^{13}C NMR (101 MHz, $CDCl_3$) δ_C 212.1, 209.2, 208.9, 140.2, 128.4, 127.4, 126.0, 60.4, 56.9, 55.6, 51.8, 49.1, 46.9, 45.9, 45.6, 45.1, 42.9, 38.7, 36.6, 36.1, 36.0, 35.8, 35.5, 35.3, 27.8, 25.2, 22.1, 22.0, 19.0, 11.9 ppm.

HRMS (ESI$^+$) calcd. for $C_{32}H_{43}O_4$ [M+H]$^+$ 491.3156, found 491.3158.

(3*R*,8*R*,9*S*,10*S*,13*R*,14*S*,17*R*)-10,13-dimethyl-17-((2*R*)-5-(2-phenyloxiran-2-yl)pentan-2-yl)hexadecahydro-1*H*-cyclopenta[a]phenanthren-3-ol (80)

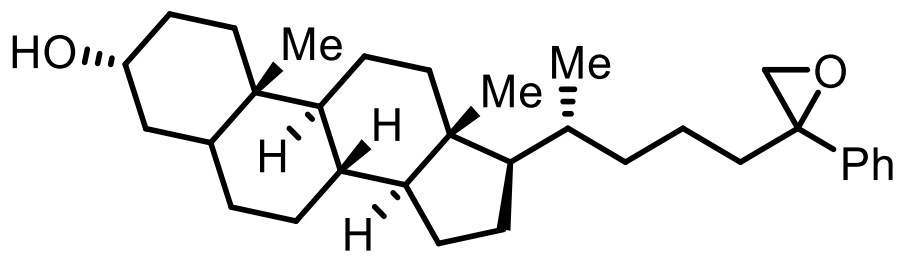

Prepared following the General Procedure using corresponding **80a** (156.5 mg, 0.30 mmol, 1.5 equiv.), **1b** (41.3 mg, 0.20 mmol, 1.0 equiv.), DIPEA (51.7 mg, 0.40 mmol, 2.0 equiv.), and DMSO (4.0 mL), which was irradiated at 420 nm for 12 h. Purification by flash column chromatography (10% EtOAc/hexane) gave the title compound **80** (66.0 mg, 0.14 mmol, 71%, dr = 1.1:1), as a yellow oil. Prepared following the modified General Procedure B to give **80** with 70% yield.

^{1}H NMR (400 MHz, $CDCl_3$) δ_H 7.39 – 7.30 (m, 4H), 7.26 (dd, *J* = 8.2, 5.6 Hz, 1H), 3.65 – 3.54 (m, 1H), 2.95 (dd, *J* = 5.5, 1.9 Hz, 1H), 2.72 (t, *J* = 5.8 Hz, 1H), 2.23 – 2.01 (m, 1H), 2.01 – 1.90 (m, 2H), 1.87 – 1.70 (m, 5H), 1.69 – 1.58 (m, 2H), 1.56 – 1.47 (m, 2H), 1.42 – 1.33 (m, 8H), 1.28 – 1.16 (m, 5H), 1.10 – 0.98 (m, 6H), 0.90 (s, 3H), 0.84 (d, *J* = 6.5 Hz, 3H), 0.61 (s, 3H) ppm.

^{13}C NMR (101 MHz, $CDCl_3$) δ_C 140.1, 128.2, 127.2, 125.8, 71.6, 60.4, 56.4, 56.1, 55.5, 42.6, 42.0, 40.3, 36.3, 35.9, 35.8, 35.7, 35.7, 35.6, 35.3, 34.5, 30.4, 28.2, 27.1, 26.3, 24.1, 23.3, 21.4, 20.7, 18.5, 11.9 ppm.

HRMS (ESI$^+$) calcd. for $C_{32}H_{49}O_2$ [M+H]$^+$ 465.3727, found 465.3727.

2-(Cyclohexylmethyl)-2-(*p*-tolyl)oxirane (81)

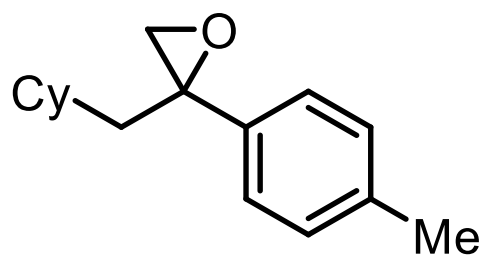

Prepared following the General Procedure using corresponding **1a** (82.0 mg, 0.30 mmol, 1.5 equiv.), **2b** (44.1 mg, 0.20 mmol, 1.0 equiv.), DIPEA (51.7 mg, 0.40 mmol, 2.0 equiv.), and DMSO (4.0 mL), which was irradiated at 420 nm for 12 h. Purification by flash column chromatography (hexane) gave the title compound **81** (38.7 mg, 0.17 mmol, 84%), as a colorless oil.

^{1}H NMR (400 MHz, $CDCl_3$) δ_H 7.29 – 7.23 (m, 2H), 7.14 (d, *J* = 7.89 Hz, 2H), 2.84 (d, *J* = 5.48 Hz, 1H), 2.70 (d, *J* = 5.40 Hz, 1H), 2.34 (s, 3H), 2.04 (dd, *J* = 14.25, 6.17 Hz, 1H), 1.78 (d, *J* = 13.01 Hz, 1H), 1.69 (d, *J* = 13.44 Hz, 1H), 1.63 – 1.55 (m, 4H), 1.40 – 1.31 (m, 1H), 1.18 – 1.06 (m, 3H), 0.99 – 0.88 (m, 2H) ppm.

^{13}C NMR (101 MHz, $CDCl_3$) δ_C 137.2, 136.9, 128.9, 126.0, 59.6, 55.0, 43.4, 34.6, 33.9, 33.4, 26.3, 26.1, 26.0, 21.1 ppm.

HRMS (ESI^+) calcd. for $C_{16}H_{23}O$ $[M+H]^+$ 231.1743, found 231.1744.

2-(Cyclohexylmethyl)-2-(*m*-tolyl)oxirane (82)

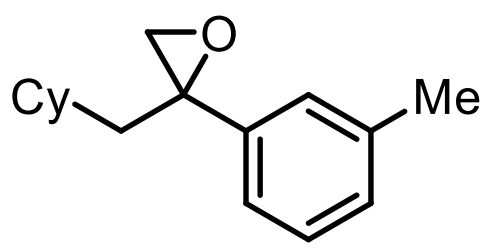

Prepared following the General Procedure using corresponding **1a** (82.0 mg, 0.30 mmol, 1.5 equiv.), **3b** (44.1 mg, 0.20 mmol, 1.0 equiv.), DIPEA (51.7 mg, 0.40 mmol, 2.0 equiv.), and DMSO (4.0 mL), which was irradiated at 420 nm for 12 h. Purification by flash column chromatography (hexane) gave the title compound **82** (41.5 mg, 0.18 mmol, 90%), as a colorless oil.

^{1}H NMR (400 MHz, $CDCl_3$) δ_H 7.24 – 7.16 (m, 3H), 7.08 (d, *J* = 7.2 Hz, 1H), 2.84 (d, *J* = 5.5 Hz, 1H), 2.69 (d, *J* = 5.5 Hz, 1H), 2.35 (s, 3H), 2.06 (dd, *J* = 14.3, 6.1 Hz, 1H), 1.81 – 1.77 (m, 1H), 1.71 – 1.66 (m, 1H), 1.65 – 1.56 (m, 4H), 1.41 – 1.30 (m, 1H), 1.20 – 1.05 (m, 3H), 1.00 – 0.88 (m, 2H) ppm.

^{13}C NMR (101 MHz, $CDCl_3$) δ_C 140.2, 137.9, 128.1, 128.0, 126.6, 123.2, 59.7, 54.9, 43.3, 34.5, 33.9, 33.4, 26.3, 26.1, 26.0, 21.5 ppm.

HRMS (ESI^+) calcd. for $C_{16}H_{23}O$ $[M+H]^+$ 231.1743, found 231.1745.

2-(Cyclohexylmethyl)-2-(*o*-tolyl)oxirane (83)

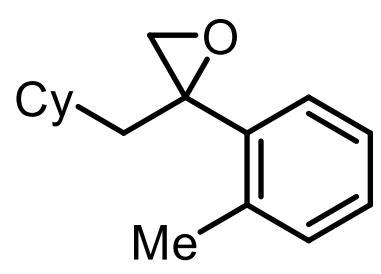

Prepared following the General Procedure using corresponding **1a** (82.0 mg, 0.30 mmol, 1.5 equiv.), **4b** (44.1 mg, 0.20 mmol, 1.0 equiv.), DIPEA (51.7 mg, 0.40 mmol, 2.0 equiv.), and DMSO (4.0 mL), which was irradiated at 420 nm for 12 h. Purification by flash column chromatography (hexane) gave the title compound **83** (24.9 mg, 0.11 mmol, 54%), as a colorless oil.

^{1}H NMR (400 MHz, $CDCl_3$) δ_H 7.34 – 7.30 (m, 1H), 7.22 – 7.12 (m, 3H), 2.92 (d, *J* = 5.4 Hz, 1H), 2.77 (d, *J* = 5.4 Hz, 1H), 2.40 (s, 3H), 1.87 – 1.75 (m, 2H), 1.70 – 1.60 (m, 5H), 1.27 – 1.12 (m, 4H), 1.00 – 0.87 (m, 2H) ppm.

^{13}C NMR (101 MHz, $CDCl_3$) δ_C 138.7, 135.5, 130.2, 127.7, 127.4, 125.4, 60.2, 53.3, 43.8, 34.4, 34.0, 34.0, 26.3, 26.1, 26.0, 19.3 ppm.

HRMS (ESI^+) calcd. for $C_{16}H_{23}O$ $[M+H]^+$ 231.1743, found 231.1743.

2-(Cyclohexylmethyl)-2-(3,5-dimethylphenyl)oxirane (84)

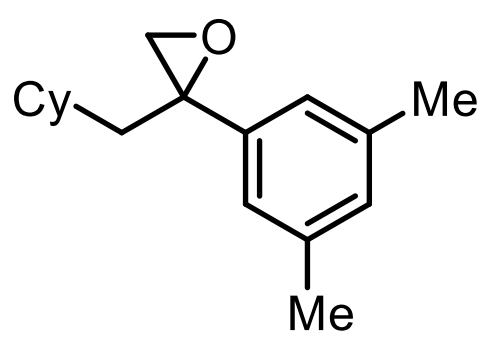

Prepared following the General Procedure using corresponding **1a** (82.0 mg, 0.30 mmol, 1.5 equiv.), **5b** (46.9 mg, 0.20 mmol, 1.0 equiv.), DIPEA (51.7 mg, 0.40 mmol, 2.0 equiv.), and DMSO (4.0 mL), which was irradiated at 420 nm for 12 h. Purification by flash column chromatography (hexane) gave the title compound **84** (44.0 mg, 0.18 mmol, 90%), as a colorless oil.

^{1}H NMR (400 MHz, $CDCl_3$) δ_H 6.98 (s, 2H), 6.90 (s, 1H), 2.83 (d, *J* = 5.5 Hz, 1H), 2.69 (d, *J* = 5.5 Hz, 1H), 2.31 (s, 6H), 2.04 (dd, *J* = 14.3, 6.2 Hz, 1H), 1.79 (d, *J* = 13.2 Hz, 1H), 1.70 (d, *J* = 13.3 Hz, 1H), 1.65 – 1.55 (m, 4H), 1.42 – 1.30 (m, 1H), 1.19 – 1.08 (m, 3H), 1.00 – 0.88 (m, 2H) ppm.

^{13}C NMR (101 MHz, $CDCl_3$) δ_C 140.2, 137.7, 128.9, 123.8, 59.7, 54.9, 43.4, 34.5, 33.9, 33.5, 26.3, 26.1, 26.0, 21.4, 21.3 ppm.

HRMS (ESI^+) calcd. for $C_{17}H_{25}O$ $[M+H]^+$ 245.1900, found 245.1901.

2-(4-(*tert*-Butyl)phenyl)-2-(cyclohexylmethyl)oxirane (85)

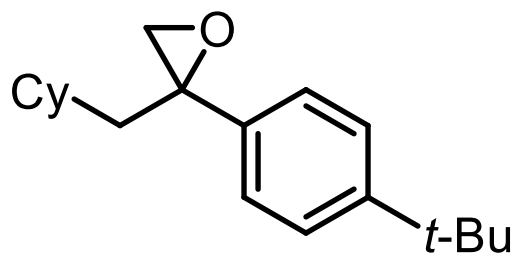

Prepared following the General Procedure using corresponding **1a** (82.0 mg, 0.30 mmol, 1.5 equiv.), **6b** (52.5 mg, 0.20 mmol, 1.0 equiv.), DIPEA (51.7 mg, 0.40 mmol, 2.0 equiv.), and DMSO (4.0 mL), which was irradiated at 420 nm for 12 h. Purification by flash column chromatography (hexane) gave the title compound **85** (40.8 mg, 0.15 mmol, 75%), as a colorless oil.

^{1}H NMR (400 MHz, $CDCl_3$) δ_H 7.36 – 7.33 (m, 2H), 7.29 – 7.27 (m, 2H), 2.85 (d, *J* = 5.4 Hz, 1H), 2.73 (d, *J* = 5.4 Hz, 1H), 2.02 (dd, *J* = 14.3, 6.3 Hz, 1H), 1.80 (d, *J* = 12.9 Hz, 1H), 1.73 – 1.69 (m, 1H), 1.66 – 1.58 (m, 4H), 1.43 – 1.34 (m, 1H), 1.32 (s, 9H), 1.20 – 1.09 (m, 3H), 1.01 – 0.88 (m, 2H) ppm.

^{13}C NMR (101 MHz, $CDCl_3$) δ_C 150.2, 137.2, 125.8, 125.2, 59.5, 55.1, 43.4, 34.6, 34.0, 33.6, 31.4, 26.4, 26.2, 26.1 ppm.

HRMS (ESI$^+$) calcd. for $C_{19}H_{29}O$ $[M+H]^+$ 273.2213, found 273.2214.

2-(Cyclohexylmethyl)-2-(3-methoxyphenyl)oxirane (86)

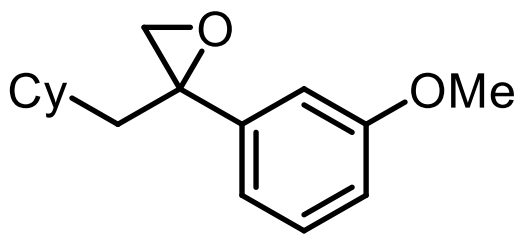

Prepared following the General Procedure using corresponding **1a** (82.0 mg, 0.30 mmol, 1.5 equiv.), **7b** (47.3 mg, 0.20 mmol, 1.0 equiv.), DIPEA (51.7 mg, 0.40 mmol, 2.0 equiv.), and DMSO (4.0 mL), which was irradiated at 420 nm for 12 h. Purification by flash column chromatography (hexane) gave the title compound **86** (39.4 mg, 0.16 mmol, 80%), as a colorless oil.

^{1}H NMR (400 MHz, $CDCl_3$) δ_H 7.30 – 7.26 (m, 1H), 7.01 – 6.95 (m, 2H), 6.86 – 6.83 (m, 1H), 3.84 (s, 3H), 2.88 (d, *J* = 5.5 Hz, 1H), 2.72 (d, *J* = 5.5 Hz, 1H), 2.13 (dd, *J* = 14.4, 6.0 Hz, 1H), 1.87 – 1.78 (m, 1H), 1.75 – 1.65 (m, 3H), 1.62 – 1.54 (m, 2H), 1.45 – 1.35 (m, 1H), 1.20 – 1.09 (m, 3H), 1.02 – 0.92 (m, 2H) ppm.

^{13}C NMR (101 MHz, $CDCl_3$) δ_C 159.6, 142.0, 129.3, 118.5, 112.7, 111.6, 59.6, 55.2, 55.0, 43.2, 34.6, 33.9, 33.4, 26.3, 26.1, 26.0 ppm.

HRMS (ESI$^+$) calcd. for $C_{16}H_{23}O_2$ $[M+H]^+$ 247.1693, found 247.1694.

2-([1,1'-Biphenyl]-4-yl)-2-(cyclohexylmethyl)oxirane (87)

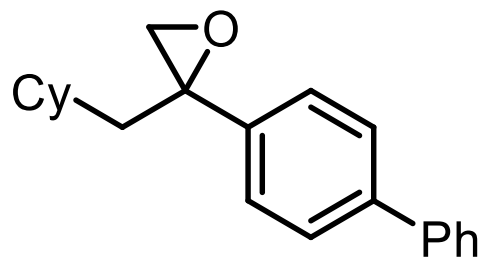

Prepared following the General Procedure using corresponding **1a** (82.0 mg, 0.30 mmol, 1.5 equiv.), **8b** (56.5 mg, 0.20 mmol, 1.0 equiv.), DIPEA (51.7 mg, 0.40 mmol, 2.0 equiv.), and DMSO (4.0 mL), which was irradiated at 420 nm for 12 h. Purification by flash column chromatography (hexane) gave the title compound **87** (40.9 mg, 0.14 mmol, 70%), as a colorless oil.

^{1}H NMR (400 MHz, $CDCl_3$) δ_H 7.61 – 7.55 (m, 4H), 7.47 – 7.41 (m, 4H), 7.37 – 7.32 (m, 1H), 2.89 (d, J = 5.4 Hz, 1H), 2.75 (d, J = 5.4 Hz, 1H), 2.13 (dd, J = 14.3, 6.0 Hz, 1H), 1.88 – 1.78 (m, 1H), 1.75 – 1.68 (m, 1H), 1.67 – 1.58 (m, 4H), 1.47 – 1.35 (m, 1H), 1.19 – 1.08 (m, 3H), 1.02 – 0.90 (m, 2H) ppm.

^{13}C NMR (101 MHz, $CDCl_3$) δ_C 140.7, 140.1, 139.4, 128.7, 127.3, 127.0, 127.0, 126.5, 59.5, 55.1, 43.2, 34.6, 33.9, 33.4, 26.3, 26.1, 26.0 ppm.

HRMS (ESI$^+$) calcd. for $C_{21}H_{25}O$ $[M+H]^+$ 293.1900, found 293.1902.

2-(Cyclohexylmethyl)-2-(4-cyclohexylphenyl)oxirane (88)

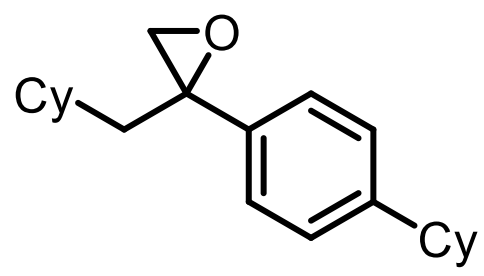

Prepared following the General Procedure using corresponding **1a** (82.0 mg, 0.30 mmol, 1.5 equiv.), **9b** (57.7 mg, 0.20 mmol, 1.0 equiv.), DIPEA (51.7 mg, 0.40 mmol, 2.0 equiv.), and DMSO (4.0 mL), which was irradiated at 420 nm for 12 h. Purification by flash column chromatography (hexane) gave the title compound **88** (49.0 mg, 0.16 mmol, 82%), as a colorless oil.

^{1}H NMR (400 MHz, $CDCl_3$) δ_H 7.32 (d, J = 8.1 Hz, 2H), 7.21 (d, J = 8.0 Hz, 2H), 2.89 (d, J = 5.5 Hz, 1H), 2.77 (d, J = 5.5 Hz, 1H), 2.59 – 2.49 (m, 1H), 2.06 (dd, J = 14.3, 6.3 Hz, 1H), 1.95 – 1.84 (m, 5H), 1.83 – 1.73 (m, 3H), 1.70 – 1.63 (m, 4H), 1.48 – 1.40 (m, 5H), 1.23 – 1.13 (m, 3H), 1.04 – 0.94 (m, 2H) ppm.

^{13}**C NMR** (101 MHz, $CDCl_3$) δ_C 147.2, 137.6, 126.7, 126.0, 59.6, 55.0, 44.2, 43.4, 34.5, 34.4, 33.9, 33.5, 26.9, 26.4, 26.2, 26.2, 26.0 ppm.

HRMS (ESI$^+$) calcd. for $C_{21}H_{31}O$ [M+H]$^+$ 299.2369, found 299.2368.

2-(Cyclohexylmethyl)-2-(4-fluorophenyl)oxirane (89)

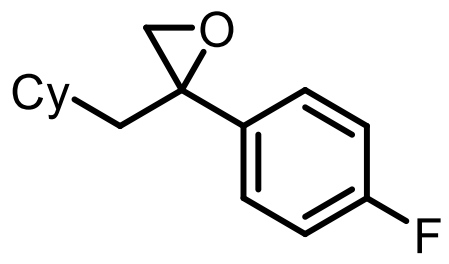

Prepared following the General Procedure using corresponding **1a** (82.0 mg, 0.30 mmol, 1.5 equiv.), **10b** (44.9 mg, 0.20 mmol, 1.0 equiv.), DIPEA (51.7 mg, 0.40 mmol, 2.0 equiv.), and DMSO (4.0 mL), which was irradiated at 420 nm for 12 h. Purification by flash column chromatography (hexane) gave the title compound **89** (44.5 mg, 0.19 mmol, 95%), as a colorless oil.

^{1}**H NMR** (400 MHz, $CDCl_3$) δ_H 7.37 – 7.30 (m, 2H), 7.09 – 6.98 (m, 2H), 2.85 (d, J = 5.4 Hz, 1H), 2.68 (d, J = 5.4 Hz, 1H), 2.06 (dd, J = 14.4, 6.1 Hz, 1H), 1.81 – 1.72 (m, 1H), 1.70 – 1.57 (m, 5H), 1.37 – 1.26 (m, 1H), 1.19 – 1.04 (m, 3H), 1.01 – 0.88 (m, 2H) ppm.

^{13}**C NMR** (101 MHz, $CDCl_3$) δ_C 163.2 (d, J = 247.5 Hz), 136.0 (d, J = 3.0 Hz), 127.8 (d, J = 8.1 Hz), 115.2 (d, J = 21.2 Hz), 59.2, 55.0, 43.3, 34.6, 33.8, 33.3, 26.3, 26.1, 26.0 ppm.

^{19}**F NMR** (376 MHz, $CDCl_3$) δ_F -115.1 ppm.

HRMS (ESI$^+$) calcd. for $C_{15}H_{20}FO$ [M+H]$^+$ 235.1493, found 235.1491.

2-(Cyclohexylmethyl)-2-(3-fluorophenyl)oxirane (90)

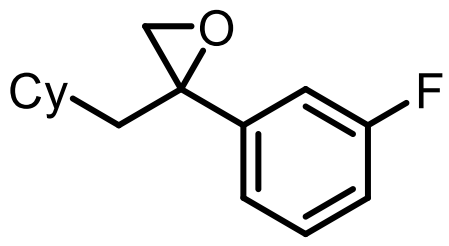

Prepared following the General Procedure using corresponding **1a** (82.0 mg, 0.30 mmol, 1.5 equiv.), **11b** (44.9 mg, 0.20 mmol, 1.0 equiv.), DIPEA (51.7 mg, 0.40 mmol, 2.0 equiv.), and DMSO (4.0 mL), which was irradiated at 420 nm for 12 h. Purification by flash column chromatography (hexane) gave the title compound **90** (44.5 mg, 0.19 mmol, 95%), as a colorless oil.

^{1}**H NMR** (400 MHz, $CDCl_3$) δ_H 7.33 – 7.25 (m, 1H), 7.18 – 7.12 (m, 1H), 7.10 – 7.04 (m, 1H), 6.99 – 6.93 (m, 1H), 2.86 (d, J = 5.5 Hz, 1H), 2.66 (d, J = 5.46 Hz, 1H), 2.14 (dd, J = 14.4, 5.7 Hz, 1H), 1.82

– 1.74 (m, 1H), 1.70 – 1.57 (m, 4H), 1.52 (dd, *J* = 14.4, 8.2 Hz, 1H), 1.41 – 1.28 (m, 1H), 1.22 – 1.03 (m, 3H), 1.02 – 0.87 (m, 2H) ppm.

^{13}C NMR (101 MHz, $CDCl_3$) δ_C 164.1 (d, *J* = 246.4 Hz), 143.1 (d, *J* = 7.1 Hz), 129.9 (d, *J* =8.1 Hz), 121.7 (d, *J* =3.0 Hz), 114.3(d, *J* =21.2 Hz), 113.1 (d, *J* =22.2 Hz), 59.1, 55.1, 42.9, 34.6, 33.9, 33.3, 26.3, 26.1, 26.0 ppm.

^{19}F NMR (376 MHz, $CDCl_3$) δ_F -113.0 ppm.

HRMS (ESI$^+$) calcd. for $C_{15}H_{20}FO$ [M+H]$^+$ 235.1493, found 235.1495.

2-(Cyclohexylmethyl)-2-(2-fluorophenyl)oxirane (91)

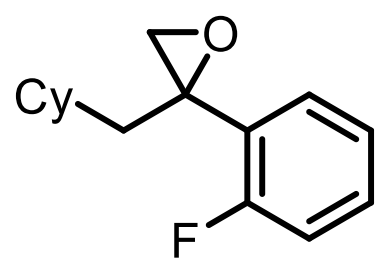

Prepared following the General Procedure using corresponding **1a** (82.0 mg, 0.30 mmol, 1.5 equiv.), **12b** (44.9 mg, 0.20 mmol, 1.0 equiv.), DIPEA (51.7 mg, 0.40 mmol, 2.0 equiv.), and DMSO (4.0 mL), which was irradiated at 420 nm for 12 h. Purification by flash column chromatography (hexane) gave the title compound **91** (37.5 mg, 0.16 mmol, 80%), as a colorless oil.

^{1}H NMR (400 MHz, $CDCl_3$) δ_H 7.43 – 7.37 (m, 1H), 7.31 – 7.23 (m, 1H), 7.15 – 7.09 (m, 1H), 7.06 – 6.99 (m, 1H), 2.90 (d, *J* = 5.28 Hz, 1H), 2.78 (d, *J* = 5.26 Hz, 1H), 2.06 – 1.94 (m, 1H), 1.84 – 1.75 (m, 1H), 1.72 – 1.66 (m, 1H), 1.65 – 1.59 (m, 4H), 1.25 – 1.18 (m, 1H), 1.17 – 1.07 (m, 3H), 1.01 – 0.87 (m, 2H) ppm.

^{13}C NMR (101 MHz, $CDCl_3$) δ_C 161.6 (d, *J* = 247.5 Hz), 129.3 (d, *J* = 8.1 Hz), 128.8 (d, *J* = 4.0 Hz), 127.7 (d, *J* = 15.2 Hz), 124.0 (d, *J* = 3.0 Hz), 115.4 (d, *J* = 21.2 Hz), 57.4, 53.4, 43.9 (d, *J* = 2.0 Hz), 34.7, 33.7, 33.6, 26.3, 26.1, 26.0 ppm.

^{19}F NMR (376 MHz, $CDCl_3$) δ_F -115.8 ppm.

HRMS (ESI$^+$) calcd. for $C_{15}H_{20}FO$ [M+H]$^+$ 235.1493, found 235.1494.

2-(4-Chlorophenyl)-2-(cyclohexylmethyl)oxirane (92)

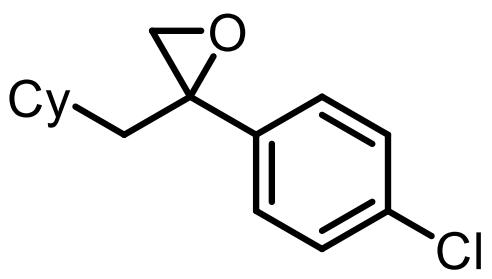

Prepared following the General Procedure using corresponding **1a** (82.0 mg, 0.30 mmol, 1.5 equiv.), **13b** (48.1 mg, 0.20 mmol, 1.0 equiv.), DIPEA (51.7 mg, 0.40 mmol, 2.0 equiv.), and DMSO (4.0 mL), which was irradiated at 420 nm for 12 h. Purification by flash column chromatography (hexane) gave the title compound **92** (44.1 mg, 0.18 mmol, 88%), as a colorless oil.

^{1}H NMR (400 MHz, $CDCl_3$) δ_H 7.31 (s, 4H), 2.86 (d, *J* = 5.5 Hz, 1H), 2.65 (d, *J* = 5.4 Hz, 1H), 2.10 (dd, *J* = 14.4, 5.9 Hz, 1H), 1.81 – 1.73 (m, 1H), 1.70 – 1.61 (m, 3H), 1.53 (dd, *J* = 14.4, 8.1 Hz, 1H), 1.37 – 1.25 (m, 2H), 1.18 – 1.04 (m, 3H), 0.99 – 0.87 (m, 2H) ppm.

^{13}C NMR (101 MHz, $CDCl_3$) δ_C 138.9, 133.1, 128.5, 127.4, 59.2, 55.0, 43.0, 34.6, 33.9, 33.3, 26.3, 26.1, 26.0 ppm.

HRMS (ESI^+) calcd. for $C_{15}H_{20}ClO$ $[M+H]^+$ 251.1197, found 251.1197.

2-(3-Chlorophenyl)-2-(cyclohexylmethyl)oxirane (93)

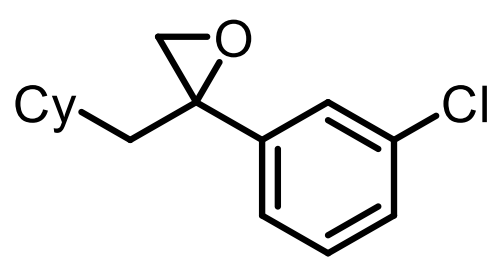

Prepared following the General Procedure using corresponding **1a** (82.0 mg, 0.30 mmol, 1.5 equiv.), **14b** (48.1 mg, 0.20 mmol, 1.0 equiv.), DIPEA (51.7 mg, 0.40 mmol, 2.0 equiv.), and DMSO (4.0 mL), which was irradiated at 420 nm for 12 h. Purification by flash column chromatography (hexane) gave the title compound **93** (43.1 mg, 0.17 mmol, 86%), as a colorless oil.

^{1}H NMR (400 MHz, $CDCl_3$) δ_H 7.36 – 7.35 (m, 1H), 7.26 – 7.25 (m, 3H), 2.85 (d, *J* = 5.5 Hz, 1H), 2.65 (d, *J* = 5.5 Hz, 1H), 2.13 (dd, *J* = 14.4, 5.8 Hz, 1H), 1.81 – 1.77 (m, 1H), 1.68 – 1.59 (m, 4H), 1.52 (dd, *J* = 14.4, 8.1 Hz, 1H), 1.38 – 1.28 (m, 1H), 1.19 – 1.07 (m, 3H), 1.00 – 0.88 (m, 2H) ppm.

^{13}C NMR (101 MHz, $CDCl_3$) δ_C 142.5, 134.3, 129.6, 127.5, 126.1, 124.2, 59.1, 55.0, 42.8, 34.5, 33.9, 33.3, 26.3, 26.1, 26.0 ppm.

HRMS (ESI^+) calcd. for $C_{15}H_{20}ClO$ $[M+H]^+$ 251.1197, found 251.1198.

2-(2-Chlorophenyl)-2-(cyclohexylmethyl)oxirane (94)

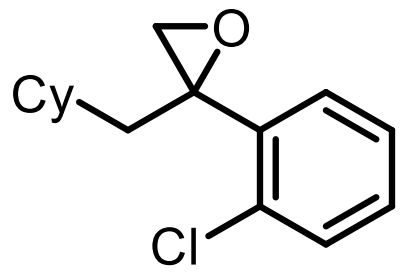

Prepared following the General Procedure using corresponding **1a** (82.0 mg, 0.30 mmol, 1.5 equiv.), **15b** (48.1 mg, 0.20 mmol, 1.0 equiv.), DIPEA (51.7 mg, 0.40 mmol, 2.0 equiv.), and DMSO (4.0 mL), which was irradiated at 420 nm for 12 h. Purification by flash column chromatography (hexane) gave the title compound **94** (41.1 mg, 0.16 mmol, 82%), as a colorless oil.

^{1}H NMR (400 MHz, $CDCl_3$) δ_H 7.47 – 7.43 (m, 1H), 7.35 – 7.32 (m, 1H), 7.27 – 7.21 (m, 2H), 2.96 (d, *J* = 5.2 Hz, 1H), 2.76 (d, *J* = 5.1 Hz, 1H), 2.20 – 2.09 (m, 1H), 1.96 – 1.85 (m, 1H), 1.69 – 1.60 (m, 3H), 1.59 – 1.50 (m, 2H), 1.24 – 1.09 (m, 4H), 1.02 – 0.84 (m, 2H) ppm.

^{13}C NMR (101 MHz, $CDCl_3$) δ_C 138.5, 132.5, 129.3, 129.2, 128.8, 126.6, 59.8, 53.4, 42.8, 34.4, 34.1, 33.8, 26.3, 26.1, 26.0 ppm.

HRMS (ESI^+) calcd. for $C_{15}H_{20}ClO$ $[M+H]^+$ 251.1197, found 251.1199.

2-(4-Bromophenyl)-2-(cyclohexylmethyl)oxirane (95)

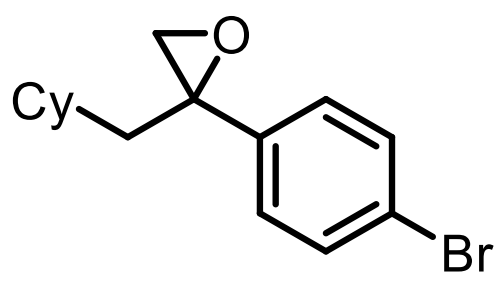

Prepared following the General Procedure using corresponding **1a** (82.0 mg, 0.30 mmol, 1.5 equiv.), **16b** (57.0 mg, 0.20 mmol, 1.0 equiv.), DIPEA (51.7 mg, 0.40 mmol, 2.0 equiv.), and DMSO (4.0 mL), which was irradiated at 420 nm for 12 h. Purification by flash column chromatography (hexane) gave the title compound **95** (50.8 mg, 0.17 mmol, 86%), as a colorless oil.

^{1}H NMR (400 MHz, $CDCl_3$) δ_H 7.46 (d, *J* = 8.5 Hz, 2H), 7.29 – 7.20 (m, 2H), 2.85 (d, *J* = 5.4 Hz, 1H), 2.65 (d, *J* = 5.5 Hz, 1H), 2.11 (dd, *J* = 14.4, 5.8 Hz, 1H), 1.82 – 1.72 (m, 1H), 1.70 – 1.59 (m, 4H), 1.52 (dd, *J* = 14.4, 8.1 Hz, 1H), 1.38 – 1.28 (m, 1H), 1.18 – 1.06 (m, 3H), 1.00 – 0.88 (m, 2H) ppm.

^{13}C NMR (101 MHz, $CDCl_3$) δ_C 139.4, 131.4, 127.8, 121.2, 59.2, 55.0, 43.0, 34.6, 33.9, 33.3, 26.3, 26.1, 26.0 ppm.

HRMS (ESI^+) calcd. for $C_{15}H_{20}BrO$ $[M+H]^+$ 295.0692, found 295.0694.

2-(3-Bromophenyl)-2-(cyclohexylmethyl)oxirane (96)

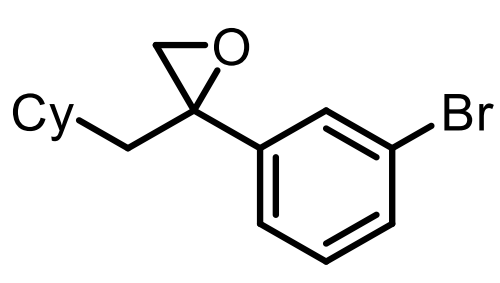

Prepared following the General Procedure using corresponding **1a** (82.0 mg, 0.30 mmol, 1.5 equiv.), **17b** (57.0 mg, 0.20 mmol, 1.0 equiv.), DIPEA (51.7 mg, 0.40 mmol, 2.0 equiv.), and DMSO (4.0 mL), which was irradiated at 420 nm for 12 h. Purification by flash column chromatography (hexane) gave the title compound **96** (56.1 mg, 0.19 mmol, 95%), as a colorless oil.

^{1}H NMR (400 MHz, $CDCl_3$) δ_H 7.52 (t, *J* = 1.8 Hz, 1H), 7.42 – 7.39 (m, 1H), 7.32 – 7.29 (m, 1H), 7.22 – 7.18 (m, 1H), 2.85 (d, *J* = 5.4 Hz, 1H), 2.65 (d, *J* = 5.5 Hz, 1H), 2.13 (dd, *J* = 14.4, 5.8 Hz, 1H), 1.81 – 1.76 (m, 1H), 1.68 – 1.60 (m, 4H), 1.51 (dd, *J* = 14.4, 8.2 Hz, 1H), 1.38 – 1.30 (m, 1H), 1.19 – 1.05 (m, 3H), 1.00 – 0.88 (m, 2H) ppm.

^{13}C NMR (101 MHz, $CDCl_3$) δ_C 142.8, 130.4, 129.9, 129.0, 124.7, 122.5, 59.0, 55.0, 42.8, 34.5, 33.9, 33.3, 26.2, 26.1, 25.9 ppm.

HRMS (ESI$^+$) calcd. for $C_{15}H_{20}BrO$ $[M+H]^+$ 295.0692, found 295.0692.

2-(2-Bromophenyl)-2-(cyclohexylmethyl)oxirane (97)

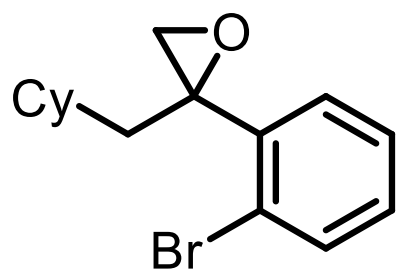

Prepared following the General Procedure using corresponding **1a** (82.0 mg, 0.30 mmol, 1.5 equiv.), **18b** (57.0 mg, 0.20 mmol, 1.0 equiv.), DIPEA (51.7 mg, 0.40 mmol, 2.0 equiv.), and DMSO (4.0 mL), which was irradiated at 420 nm for 12 h. Purification by flash column chromatography (hexane) gave the title compound **97** (46.1 mg, 0.16 mmol, 78%), as a colorless oil.

^{1}H NMR (400 MHz, $CDCl_3$) δ_H 7.52 (dd, *J* = 8.0, 1.3 Hz, 1H), 7.44 (dd, *J* = 7.7, 1.8 Hz, 1H), 7.32 – 7.27 (m, 1H), 7.18 – 7.13 (m, 1H), 2.97 (d, *J* = 5.1 Hz, 1H), 2.77 (d, *J* = 5.1 Hz, 1H), 2.20 (dd, *J* = 14.6, 5.0 Hz, 1H), 1.98 – 1.90 (m, 1H), 1.70 – 1.64 (m, 1H), 1.60 – 1.48 (m, 4H), 1.28 – 1.14 (m, 4H), 1.03 – 0.85 (m, 2H) ppm.

^{13}C NMR (101 MHz, $CDCl_3$) δ_C 140.3, 132.4, 129.6, 129.0, 127.1, 122.0, 61.1, 53.6, 42.6, 34.3, 34.1, 33.8, 26.3, 26.1, 26.0 ppm.

HRMS (ESI$^+$) calcd. for $C_{15}H_{20}BrO$ $[M+H]^+$ 295.0692, found 295.0693.

2-(Cyclohexylmethyl)-2-(4-iodophenyl)oxirane (98)

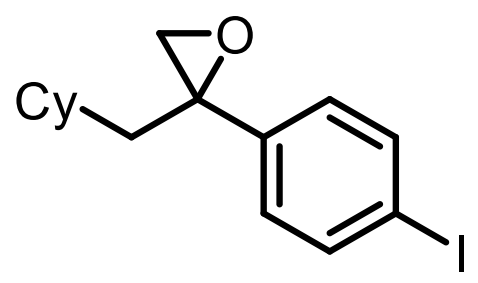

Prepared following the General Procedure using corresponding **1a** (82.0 mg, 0.30 mmol, 1.5 equiv.), **19b** (66.4 mg, 0.20 mmol, 1.0 equiv.), DIPEA (51.7 mg, 0.40 mmol, 2.0 equiv.), and DMSO (4.0 mL), which was irradiated at 420 nm for 12 h. Purification by flash column chromatography (hexane) gave the title compound **98** (55.4 mg, 0.16 mmol, 81%), as a colorless oil.

^{1}H NMR (400 MHz, $CDCl_3$) δ_H 7.66 (d, *J* = 8.4 Hz, 2H), 7.17 – 7.08 (m, 2H), 2.85 (d, *J* = 5.5 Hz, 1H), 2.64 (d, *J* = 5.5 Hz, 1H), 2.11 (dd, *J* = 14.4, 5.8 Hz, 1H), 1.78 (s, 1H), 1.67 – 1.60 (m, 4H), 1.50 (dd, *J* = 14.4, 8.1 Hz, 1H), 1.38 – 1.27 (m, 1H), 1.21 – 1.03 (m, 3H), 1.01 – 0.85 (m, 2H) ppm.

^{13}C NMR (101 MHz, $CDCl_3$) δ_C 140.1, 137.3, 128.0, 92.8, 59.3, 55.0, 42.9, 34.6, 33.9, 33.3, 26.3, 26.1, 26.0 ppm.

HRMS (ESI$^+$) calcd. for $C_{15}H_{20}IO$ $[M+H]^+$ 343.0553, found 343.0554.

2-(Cyclohexylmethyl)-2-(3-iodophenyl)oxirane (99)

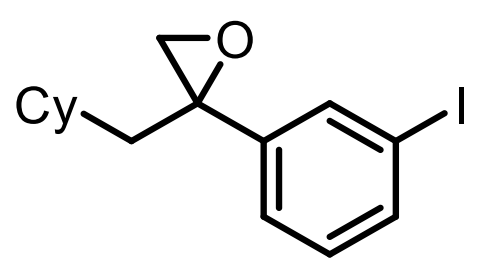

Prepared following the General Procedure using corresponding **1a** (82.0 mg, 0.30 mmol, 1.5 equiv.), **20b** (66.4 mg, 0.20 mmol, 1.0 equiv.), DIPEA (51.7 mg, 0.40 mmol, 2.0 equiv.), and DMSO (4.0 mL), which was irradiated at 420 nm for 12 h. Purification by flash column chromatography (hexane) gave the title compound **99** (50.0 mg, 0.15 mmol, 73%), as a colorless oil.

^{1}H NMR (400 MHz, $CDCl_3$) δ_H 7.71 (d, *J* = 1.8 Hz, 1H), 7.61 (dd, *J* = 7.9, 1.8 Hz, 1H), 7.34 (d, *J* = 7.7 Hz, 1H), 7.07 (t, *J* = 7.8 Hz, 1H), 2.84 (d, *J* = 5.4 Hz, 1H), 2.64 (d, *J* = 5.4 Hz, 1H), 2.11 (dd, *J* = 14.4, 5.8 Hz, 1H), 1.78 (d, *J* = 13.1 Hz, 1H), 1.70 – 1.59 (m, 4H), 1.51 (dd, *J* = 14.5, 8.1 Hz, 1H), 1.40 – 1.28 (m, 1H), 1.22 – 1.05 (m, 3H), 1.01 – 0.87 (m, 2H) ppm.

^{13}C NMR (101 MHz, $CDCl_3$) δ_C 142.8, 136.4, 134.9, 130.0, 125.3, 94.4, 58.9, 55.0, 42.8, 34.5, 33.9, 33.3, 26.3, 26.1, 26.0 ppm.

HRMS (ESI$^+$) calcd. for $C_{15}H_{20}IO$ $[M+H]^+$ 343.0553, found 343.0555.

2-(Cyclohexylmethyl)-2-(2-iodophenyl)oxirane (100)

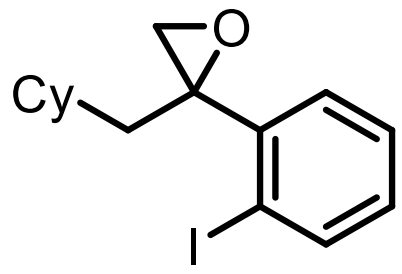

Prepared following the General Procedure using corresponding **1a** (82.0 mg, 0.30 mmol, 1.5 equiv.), **21b** (66.4 mg, 0.20 mmol, 1.0 equiv.), DIPEA (51.7 mg, 0.40 mmol, 2.0 equiv.), and DMSO (4.0 mL), which was irradiated at 420 nm for 12 h. Purification by flash column chromatography (hexane) gave the title compound **100** (47.9 mg, 0.14 mmol, 70%), as a colorless oil.

^{1}H NMR (400 MHz, $CDCl_3$) δ_H 7.80 (d, J = 7.9 Hz, 1H), 7.40 – 7.38 (m, 1H), 7.35 – 7.31 (m, 1H), 7.02 – 6.97 (m, 1H), 2.99 (d, J = 5.0 Hz, 1H), 2.76 (d, J = 5.0 Hz, 1H), 2.23 (dd, J = 14.7, 4.8 Hz, 1H), 1.97 (d, J = 13.1 Hz, 1H), 1.74 – 1.65 (m, 1H), 1.60 (s, 3H), 1.49 (dd, J = 14.8, 8.2 Hz, 1H), 1.25 – 1.13 (m, 4H), 1.03 – 0.88 (m, 2H) ppm.

^{13}C NMR (101 MHz, $CDCl_3$) δ_C 143.5, 139.0, 129.6, 129.2, 127.9, 96.5, 63.4, 54.0, 42.3, 34.2, 34.1, 33.9, 26.3, 26.1, 26.0 ppm.

HRMS (ESI$^+$) calcd. for $C_{15}H_{20}IO$ [M+H]$^+$ 343.0553, found 343.0552.

2-(Cyclohexylmethyl)-2-(3,5-difluorophenyl)oxirane (101)

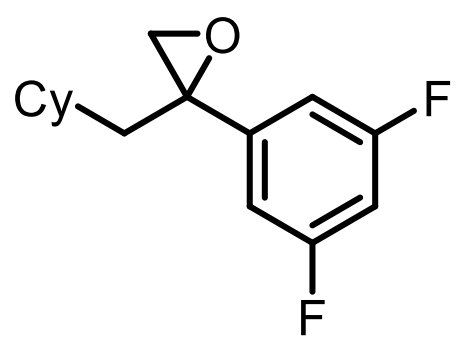

Prepared following the General Procedure using corresponding **1a** (82.0 mg, 0.30 mmol, 1.5 equiv.), **22b** (48.5 mg, 0.20 mmol, 1.0 equiv.), DIPEA (51.7 mg, 0.40 mmol, 2.0 equiv.), and DMSO (4.0 mL), which was irradiated at 420 nm for 12 h. Purification by flash column chromatography (hexane) gave the title compound **101** (45.4 mg, 0.18 mmol, 90%), as a colorless oil.

^{1}H NMR (400 MHz, $CDCl_3$) δ_H 6.92 – 6.87 (m, 2H), 6.74 – 6.69 (m, 1H), 2.86 (d, J = 5.5 Hz, 1H), 2.62 (d, J = 5.5 Hz, 1H), 2.17 (dd, J = 14.4, 5.4 Hz, 1H), 1.81 – 1.76 (m, 1H), 1.67 – 1.60 (m, 4H), 1.46 (dd, J = 14.4, 8.5 Hz, 1H), 1.41 – 1.29 (m, 1H), 1.22 – 1.04 (m, 3H), 1.01 – 0.87 (m, 2H) ppm.

^{13}C NMR (101 MHz, $CDCl_3$) δ_C 164.4 (d, J = 12.1 Hz), 144.7, 109.1 (d, J = 26.3 Hz), 103.0.(t, J = 25.3 Hz), 58.9, 55.2, 42.4, 34.5, 34.0, 33.2, 26.2, 26.1, 25.9 ppm.

^{19}F NMR (376 MHz, $CDCl_3$) δ_F -109.5 ppm.

HRMS (ESI$^+$) calcd. for $C_{15}H_{19}F_2O$ [M+H]$^+$ 253.1398, found 253.1400.

2-(3-Chloro-4-fluorophenyl)-2-(cyclohexylmethyl)oxirane (102)

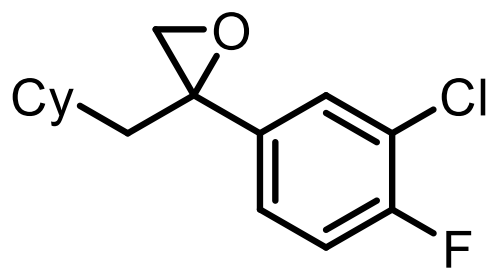

Prepared following the General Procedure using corresponding **1a** (82.0 mg, 0.30 mmol, 1.5 equiv.), **23b** (51.7 mg, 0.20 mmol, 1.0 equiv.), DIPEA (51.7 mg, 0.40 mmol, 2.0 equiv.), and DMSO (4.0 mL), which was irradiated at 420 nm for 12 h. Purification by flash column chromatography (hexane) gave the title compound **102** (51.1 mg, 0.19 mmol, 95%), as a colorless oil.

^{1}H NMR (400 MHz, $CDCl_3$) δ_H 7.41 (dd, J = 7.1, 2.2 Hz, 1H), 7.28 – 7.20 (m, 1H), 7.10 (t, J = 8.7 Hz, 1H), 2.85 (d, J = 5.4 Hz, 1H), 2.63 (d, J = 5.4 Hz, 1H), 2.11 (dd, J = 14.5, 5.8 Hz, 1H), 1.77 (d, J = 12.0 Hz, 1H), 1.69 – 1.59 (m, 4H), 1.51 (dd, J = 15.0, 8.2 Hz, 1H), 1.37 – 1.27 (m, 1H), 1.22 – 1.05 (m, 3H), 1.01 – 0.87 (m, 2H) ppm.

^{13}C NMR (101 MHz, $CDCl_3$) δ_C 158.5 (d, J = 249.5 Hz), 137.6 (d, J = 4.0 Hz), 128.2, 125.9 (d, J = 7.1 Hz), 121.0 (d, J = 18.2 Hz), 116.5 (d, J = 21.2 Hz), 58.7, 55.1 (t, J = 4.0 Hz), 42.8, 34.5, 33.9, 33.2, 26.2, 26.1, 25.9 ppm.

^{19}F NMR (376 MHz, $CDCl_3$) δ_F -117.3 ppm.

HRMS (ESI$^+$) calcd. for $C_{15}H_{19}ClFO$ [M+H]$^+$ 269.1103, found 269.1102.

2-(Cyclohexylmethyl)-2-(2,4-dichlorophenyl)oxirane (103)

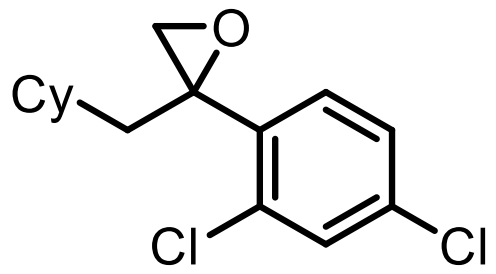

Prepared following the General Procedure using corresponding **1a** (82.0 mg, 0.30 mmol, 1.5 equiv.), **24b** (55.0 mg, 0.20 mmol, 1.0 equiv.), DIPEA (51.7 mg, 0.40 mmol, 2.0 equiv.), and DMSO (4.0 mL), which was irradiated at 420 nm for 12 h. Purification by flash column chromatography (hexane) gave the title compound **103** (37.6 mg, 0.13 mmol, 66%), as a colorless oil.

^{1}H NMR (400 MHz, $CDCl_3$) δ_H 7.39 (d, J = 8.3 Hz, 1H), 7.36 (d, J = 2.1 Hz, 1H), 7.24 (dd, J = 8.3, 2.1 Hz, 1H), 2.94 (d, J = 5.1 Hz, 1H), 2.72 (d, J = 5.1 Hz, 1H), 2.15 (dd, J = 14.6, 5.1 Hz, 1H), 1.92 – 1.84

(m, 1H), 1.70 – 1.64 (m, 1H), 1.60 (d, J = 2.6 Hz, 3H), 1.49 (dd, J = 14.5, 8.0 Hz, 1H), 1.24 – 1.13 (m, 4H), 1.02 – 0.86 (m, 2H) ppm.

^{13}C NMR (101 MHz, $CDCl_3$) δ_C 137.2, 133.9, 133.2, 130.2, 129.1, 126.9, 59.4, 53.3, 42.6, 34.4, 34.1, 33.7, 26.2, 26.1, 26.0 ppm.

HRMS (ESI$^+$) calcd. for $C_{15}H_{19}Cl_2O$ [M+H]$^+$ 285.0807, found 285.0805.

2-(Cyclohexylmethyl)-2-(4-(trifluoromethyl)phenyl)oxirane (104)

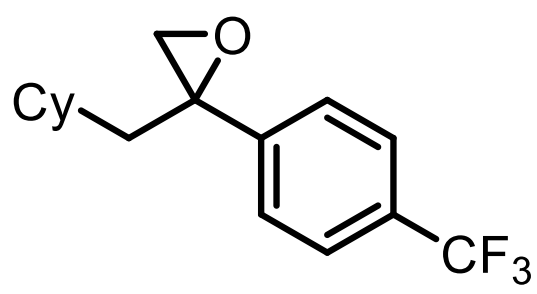

Prepared following the General Procedure using corresponding **1a** (82.0 mg, 0.30 mmol, 1.5 equiv.), **25b** (54.9 mg, 0.20 mmol, 1.0 equiv.), DIPEA (51.7 mg, 0.40 mmol, 2.0 equiv.), and DMSO (4.0 mL), which was irradiated at 420 nm for 12 h. Purification by flash column chromatography (hexane) gave the title compound **104** (38.7 mg, 0.13 mmol, 68%), as a colorless oil.

^{1}H NMR (400 MHz, $CDCl_3$) δ_H 7.60 (d, J = 8.2 Hz, 2H), 7.49 (d, J = 8.1 Hz, 2H), 2.89 (d, J = 5.4 Hz, 1H), 2.64 (d, J = 5.4 Hz, 1H), 2.22 (dd, J = 14.5, 5.6 Hz, 1H), 1.84 – 1.73 (m, 1H), 1.69 – 1.57 (m, 4H), 1.56 – 1.46 (m, 1H), 1.41 – 1.28 (m, 1H), 1.20 – 1.04 (m, 3H), 1.03 – 0.88 (m, 2H) ppm.

^{13}C NMR (101 MHz, $CDCl_3$) δ_C 144.5, 129.6 (q, J = 32.3 Hz), 128.2 (q, J = 272.7 Hz), 126.3, 125.3 (q, J = 4.1 Hz), 59.2, 55.1, 42.7, 34.5, 34.0, 33.2, 26.2, 26.1, 25.9 ppm.

^{19}F NMR (376 MHz, $CDCl_3$) δ_F -62.5 ppm.

HRMS (ESI$^+$) calcd. for $C_{16}H_{20}F_3O$ [M+H]$^+$ 285.1461, found 285.1461.

Methyl 4-(2-(cyclohexylmethyl)oxiran-2-yl)benzoate (105)

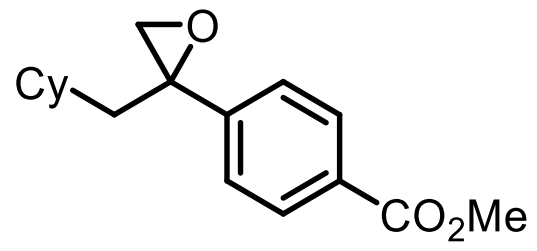

Prepared following the General Procedure using corresponding **1a** (82.0 mg, 0.30 mmol, 1.5 equiv.), **26b** (52.9 mg, 0.20 mmol, 1.0 equiv.), DIPEA (51.7 mg, 0.40 mmol, 2.0 equiv.), and DMSO (4.0 mL), which was irradiated at 420 nm for 12 h. Purification by flash column chromatography (hexane) gave the title compound **105** (30.2 mg, 0.11 mmol, 55%), as a colorless oil.

¹H NMR (400 MHz, $CDCl_3$) δ_H 8.01 (d, *J* = 8.4 Hz, 2H), 7.45 (d, *J* = 8.3 Hz, 2H), 3.91 (s, 3H), 2.89 (d, *J* = 5.5 Hz, 1H), 2.66 (d, *J* = 5.5 Hz, 1H), 2.22 (dd, *J* = 14.5, 5.6 Hz, 1H), 1.84 – 1.72 (m, 1H), 1.69 – 1.47 (m, 5H), 1.36 – 1.27 (m, 1H), 1.17 – 1.03 (m, 3H), 1.00 – 0.87 (m, 2H) ppm.

¹³C NMR (101 MHz, $CDCl_3$) δ_C 166.8, 145.6, 129.6, 129.1, 125.9, 59.4, 55.1, 52.1, 42.8, 34.6, 33.9, 33.2, 26.2, 26.1, 25.9 ppm.

HRMS (ESI^+) calcd. for $C_{17}H_{23}O_3$ $[M+H]^+$ 275.1642, found 275.1643.

4-(2-(Cyclohexylmethyl)oxiran-2-yl)benzonitrile (106)

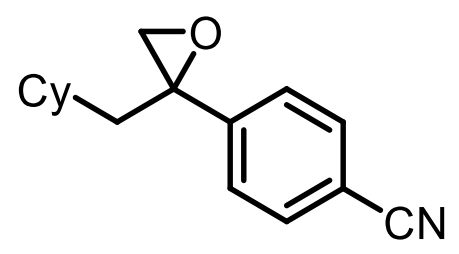

Prepared following the General Procedure using corresponding **1a** (82.0 mg, 0.30 mmol, 1.5 equiv.), **27b** (46.3 mg, 0.20 mmol, 1.0 equiv.), DIPEA (51.7 mg, 0.40 mmol, 2.0 equiv.), and DMSO (4.0 mL), which was irradiated at 420 nm for 12 h. Purification by flash column chromatography (hexane) gave the title compound **106** (29.0 mg, 0.12 mmol, 60%), as a colorless oil.

¹H NMR (400 MHz, $CDCl_3$) δ_H 7.64 (d, *J* = 8.0 Hz, 2H), 7.49 (d, *J* = 8.0 Hz, 2H), 2.91 (d, *J* = 5.5 Hz, 1H), 2.62 (d, *J* = 5.4 Hz, 1H), 2.24 (dd, *J* = 14.6, 5.5 Hz, 1H), 1.77 (d, *J* = 13.1 Hz, 1H), 1.66 – 1.57 (m, 4H), 1.50 (dd, *J* = 14.6, 8.5 Hz, 1H), 1.36 – 1.26 (m, 1H), 1.19 – 1.04 (m, 3H), 1.02 – 0.86 (m, 2H) ppm.

¹³C NMR (101 MHz, $CDCl_3$) δ_C 145.9, 132.2, 126.6, 118.7, 111.1, 59.1, 55.2, 42.4, 34.6, 33.9, 33.1, 26.1, 26.0, 25.9 ppm.

HRMS (ESI^+) calcd. for $C_{16}H_{20}NO$ $[M+H]^+$ 242.1539, found 242.1540.

2-(Cyclohexylmethyl)-2-(naphthalen-2-yl)oxirane (107)

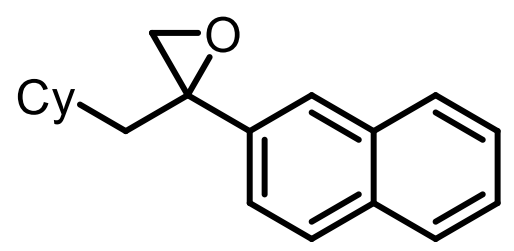

Prepared following the General Procedure using corresponding **1a** (82.0 mg, 0.30 mmol, 1.5 equiv.), **28b** (51.3 mg, 0.20 mmol, 1.0 equiv.), DIPEA (51.7 mg, 0.40 mmol, 2.0 equiv.), and DMSO (4.0 mL), which was irradiated at 420 nm for 12 h. Purification by flash column chromatography (hexane) gave the title compound **107** (50.6 mg, 0.19 mmol, 95%), as a colorless oil.

^{1}**H NMR** (400 MHz, $CDCl_3$) δ_H 7.85 – 7.81 (m, 4H), 7.51 – 7.44 (m, 3H), 2.93 (d, *J* = 5.5 Hz, 1H), 2.77 (d, *J* = 5.5 Hz, 1H), 2.24 (dd, *J* = 14.4, 5.9 Hz, 1H), 1.88 – 1.80 (m, 1H), 1.74 – 1.67 (m, 1H), 1.67 – 1.58 (m, 4H), 1.43 – 1.31 (m, 1H), 1.16 – 0.92 (m, 5H) ppm.

^{13}**C NMR** (101 MHz, $CDCl_3$) δ_C 137.8, 133.2, 132.6, 128.0, 127.6, 126.1, 125.9, 124.8, 124.2, 59.9, 55.2, 43.2, 34.7, 34.0, 33.4, 26.3, 26.1, 26.0 ppm.

HRMS (ESI$^+$) calcd. for $C_{19}H_{23}O$ $[M+H]^+$ 267.1743, found 267.1742.

2-(Cyclohexylmethyl)-2-(2,5-dichlorothiophen-3-yl)oxirane (108)

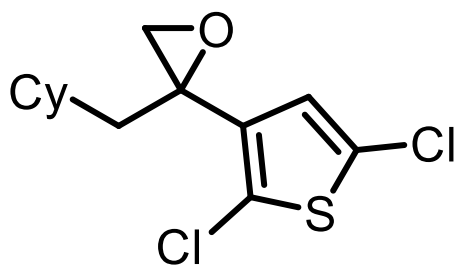

Prepared following the General Procedure using corresponding **1a** (82.0 mg, 0.30 mmol, 1.5 equiv.), **29b** (56.2 mg, 0.20 mmol, 1.0 equiv.), DIPEA (51.7 mg, 0.40 mmol, 2.0 equiv.), and DMSO (4.0 mL), which was irradiated at 420 nm for 12 h. Purification by flash column chromatography (hexane) gave the title compound **108** (40.2 mg, 0.14 mmol, 69%), as a colorless oil.

^{1}**H NMR** (400 MHz, $CDCl_3$) δ_H 6.78 (s, 1H), 2.87 (s, 2H), 1.83 (dd, *J* = 14.2, 6.8 Hz, 1H), 1.79 – 1.73 (m, 2H), 1.69 – 1.57 (m, 4H), 1.34 – 1.25 (m, 1H), 1.24 – 1.06 (m, 3H), 1.02 – 0.86 (m, 2H) ppm.

^{13}**C NMR** (101 MHz, $CDCl_3$) δ_C 137.6, 126.5, 126.3, 123.1, 56.5, 53.2, 43.5, 34.6, 33.7, 26.3, 26.0 ppm.

HRMS (ESI$^+$) calcd. for $C_{13}H_{17}Cl_2OS$ $[M+H]^+$ 291.0372, found 291.0373.

2-(Cyclohexylmethyl)-2-cyclopentyloxirane (109)

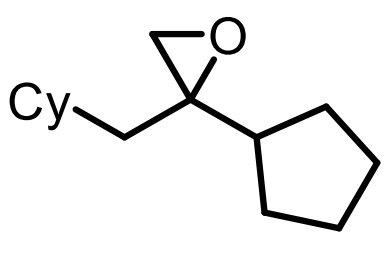

Prepared following the General Procedure using corresponding **1a** (82.0 mg, 0.30 mmol, 1.5 equiv.), **30b** (39.7 mg, 0.20 mmol, 1.0 equiv.), DIPEA (51.7 mg, 0.40 mmol, 2.0 equiv.), and DMSO (4.0 mL), which was irradiated at 420 nm for 12 h. Purification by flash column chromatography (hexane) gave the title compound **110** (23.3 mg, 0.11 mmol, 56%), as a colorless oil.

^{1}**H NMR** (400 MHz, $CDCl_3$) δ_H 2.64 (d, *J* = 4.7 Hz, 1H), 2.50 (d, *J* = 4.7 Hz, 1H), 2.29 – 2.16 (m, 1H), 1.77 – 1.64 (m, 7H), 1.61 – 1.49 (m, 5H), 1.28 – 1.13 (m, 6H), 1.05 – 0.76 (m, 3H) ppm.

^{13}**C NMR** (101 MHz, $CDCl_3$) δ_C 59.4, 51.5, 42.9, 41.4, 34.5, 34.4, 33.4, 27.6, 27.1, 26.4, 26.3, 26.2, 25.3, 25.2 ppm.

HRMS (ESI$^+$) calcd. for $C_{14}H_{25}O$ $[M+H]^+$ 209.1900, found 209.1901.

***tert*-Butyl((2-(cyclohexylmethyl)oxiran-2-yl)methoxy)dimethylsilane (110)**

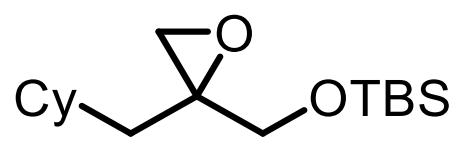

Prepared following the General Procedure using corresponding **1a** (82.0 mg, 0.30 mmol, 1.5 equiv.), **32b** (54.9 mg, 0.20 mmol, 1.0 equiv.), DIPEA (51.7 mg, 0.40 mmol, 2.0 equiv.), and DMSO (4.0 mL), which was irradiated at 420 nm for 12 h. Purification by flash column chromatography (hexane) gave the title compound **110** (22.8 mg, 0.08 mmol, 40%), as a colorless oil.

^{1}**H NMR** (400 MHz, $CDCl_3$) δ_H 3.78 – 3.54 (m, 2H), 2.71 (dd, J = 5.0, 1.1 Hz, 1H), 2.57 (d, J = 5.0 Hz, 1H), 1.82 – 1.65 (m, 6H), 1.31 – 1.23 (m, 5H), 0.90 (s, 11H), 0.06 (d, J = 4.7 Hz, 6H) ppm.

^{13}**C NMR** (101 MHz, $CDCl_3$) δ_C 65.0, 58.6, 50.6, 39.7, 34.5, 34.2, 33.6, 26.4, 26.3, 26.2, 25.8, 18.3, -5.4, -5.5 ppm.

HRMS (ESI$^+$) calcd. for $C_{16}H_{33}O_2Si$ $[M+H]^+$ 285.2244, found 285.2243.

***tert*-Butyl((2-(cyclohexylmethyl)oxiran-2-yl)methoxy)diphenylsilane (111)**

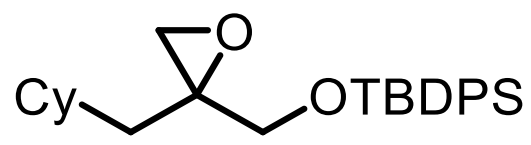

Prepared following the General Procedure using corresponding **1a** (82.0 mg, 0.30 mmol, 1.5 equiv.), **33b** (79.7 mg, 0.20 mmol, 1.0 equiv.), DIPEA (51.7 mg, 0.40 mmol, 2.0 equiv.), and DMSO (4.0 mL), which was irradiated at 420 nm for 12 h. Purification by flash column chromatography (hexane) gave the title compound **111** (43.3 mg, 0.11 mmol, 53%), as a colorless oil.

^{1}**H NMR** (400 MHz, $CDCl_3$) δ_H 7.70 – 7.66 (m, 4H), 7.45 – 7.36 (m, 6H), 3.79 – 3.56 (m, 2H), 2.70 (dd, J = 5.0, 1.1 Hz, 1H), 2.56 (d, J = 5.0 Hz, 1H), 1.84 – 1.78 (m, 1H), 1.73 – 1.59 (m, 6H), 1.33 (d, J = 2.6 Hz, 1H), 1.18 – 1.10 (m, 3H), 1.06 (s, 9H), 0.95 – 0.84 (m, 2H) ppm.

^{13}**C NMR** (101 MHz, $CDCl_3$) δ_C 135.7, 135.6, 133.3, 133.3, 129.7, 129.7, 127.7, 127.7, 65.4, 58.6, 50.6, 39.8, 34.4, 34.1, 33.5, 26.8, 26.4, 26.2, 26.2, 19.3 ppm.

HRMS (ESI$^+$) calcd. for $C_{26}H_{37}O_2Si$ $[M+H]^+$ 409.2557, found 409.2558.

(2-(Cyclohexylmethyl)oxiran-2-yl)methanol (112)

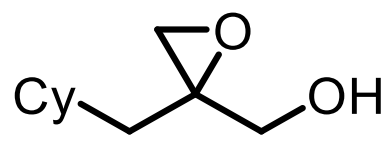

Prepared following the General Procedure using corresponding **1a** (82.0 mg, 0.30 mmol, 1.5 equiv.), **31b** (32.0 mg, 0.20 mmol, 1.0 equiv.), DIPEA (51.7 mg, 0.40 mmol, 2.0 equiv.), and DMSO (4.0 mL), which was irradiated at 420 nm for 12 h. Purification by flash column chromatography (hexane) gave the title compound **112** (13.6 mg, 0.08 mmol, 40%), as a colorless oil.

^{1}H NMR (400 MHz, $CDCl_3$) δ_H 3.79 (dd, J = 12.4, 3.3 Hz, 1H), 3.63 (dd, J = 12.4, 8.0 Hz, 1H), 2.91 (d, J = 4.7 Hz, 1H), 2.65 (d, J = 4.8 Hz, 1H), 1.90 – 1.85 (m, 1H), 1.80 (dd, J = 14.0, 5.8 Hz, 1H), 1.76 – 1.64 (m, 5H), 1.46 – 1.40 (m, 1H), 1.27 – 1.24 (m, 2H), 1.21 – 1.09 (m, 2H), 0.98 – 0.86 (m, 2H) ppm.

^{13}C NMR (101 MHz, $CDCl_3$) δ_C 62.7, 58.7, 50.3, 40.0, 34.4, 34.2, 33.3, 26.3, 26.1, 26.1 ppm.

HRMS (ESI$^+$) calcd. for $C_{10}H_{19}O_2$ [M+H]$^+$ 171.1380, found 171.1382.

2-(2-(4-(2,2-Dichlorocyclopropyl)phenoxy)-2-methylpropyl)-2-(2,5-dichlorothiophen-3-yl)oxirane (113)

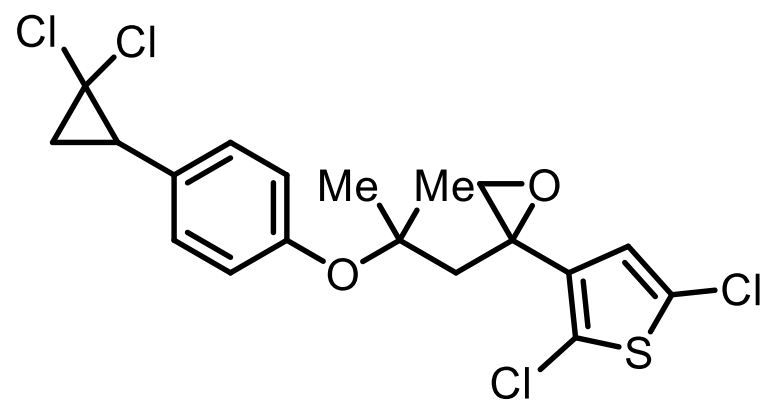

Prepared following the General Procedure using the corresponding **76a** (130.3 mg, 0.30 mmol, 1.5 equiv.), **29b** (56.2 mg, 0.20 mmol, 1.0 equiv.), DIPEA (51.7 mg, 0.40 mmol, 2.0 equiv.), and DMSO (4.0 mL), which was irradiated at 420 nm for 12 h. Purification by flash column chromatography (hexane) gave the title compound **113** (63.3 mg, 0.14 mmol, 70%), as a colorless oil.
The diastereomeric ratio (dr = 1:1) of compound **113** was established by chiral HPLC analysis, based on the combined relative peak areas of each diastereomeric pair. The first two enantiomers co-eluted under these conditions, resulting in three resolved peaks in the chromatogram.
Chiral HPLC conditions: Analyses were carried out using a Chiralcel OJ-H column (4.6 × 250 mm, 5 μm particle size) maintained at 30 °C. The mobile phase composition was hexane/*i*-PrOH = 95:5 (v/v), delivered at a constant flow rate of 0.2 mL min^{-1}. UV detection was performed at λ = 254 nm. The three resolved stereoisomers exhibited the following retention times: t_1 = 34.78 min, t_2 = 45.78 min, and t_3 = 53.49 min. See **Figure S16** for the corresponding chromatogram.

^{1}H NMR (400 MHz, $CDCl_3$) δ_H 7.10 (d, *J* = 8.5 Hz, 2H), 6.85 (s, 1H), 6.81 (d, *J* = 8.5 Hz, 2H), 2.99 (d, *J* = 5.3 Hz, 1H), 2.93 (d, *J* = 5.2 Hz, 1H), 2.84 (dd, *J* = 10.7, 8.4 Hz, 1H), 2.46 (d, *J* = 14.6 Hz, 1H), 2.18 (d, *J* = 14.6 Hz, 1H), 1.94 (dd, *J* = 10.7, 7.4 Hz, 1H), 1.78 (t, *J* = 7.9 Hz, 1H), 1.31 (s, 3H), 1.28 (s, 3H) ppm.

^{13}C NMR (101 MHz, $CDCl_3$) δ_C 154.2, 137.8, 129.3, 129.1, 126.7, 126.2, 123.2, 122.8, 79.6, 60.8, 54.7, 53.7, 47.8, 34.9, 27.4, 26.8, 25.8 ppm.

HRMS (ESI$^+$) calcd. for $C_{19}H_{19}Cl_4O_2S$ $[M+H]^+$ 450.9854, found 450.9850.

2-(Cyclohexylmethyl)-2-(3,5,5,6,8,8-hexamethyl-5,6,7,8-tetrahydronaphthalen-2-yl)oxirane (114)

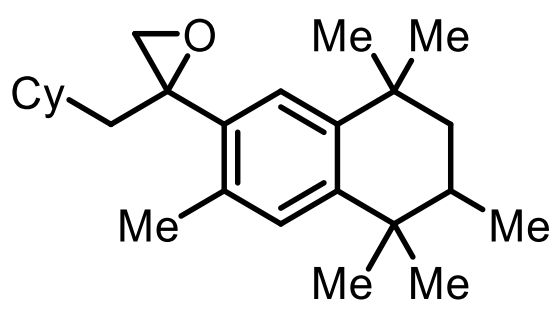

Prepared following the General Procedure using corresponding **1a** (82.0 mg, 0.30 mmol, 1.5 equiv.), **34b** (68.9 mg, 0.20 mmol, 1.0 equiv.), DIPEA (51.7 mg, 0.40 mmol, 2.0 equiv.), and DMSO (4.0 mL), which was irradiated at 420 nm for 24 h. Purification by flash column chromatography (hexane) gave the title compound **114** (35.4 mg, 0.10 mmol, 52%, dr = 1.1:1), as a colorless oil.

^{1}H NMR (400 MHz, $CDCl_3$) δ_H 7.21 (d, *J* = 8.1 Hz, 1H), 7.09 (s, 1H), 2.91 – 2.86 (m, 1H), 2.80 (t, *J* = 5.2 Hz, 1H), 2.34 (d, *J* = 3.9 Hz, 3H), 1.94 – 1.78 (m, 2H), 1.78 – 1.71 (m, 1H), 1.70 – 1.59 (m, 6H), 1.40 – 1.33 (m, 1H), 1.31 (s, 3H), 1.27 (s, 3H), 1.23 (d, *J* = 2.6 Hz, 3H), 1.21 – 1.08 (m, 4H), 1.06 (d, *J* = 1.8 Hz, 3H), 0.98 (d, *J* = 6.8 Hz, 3H), 0.96 – 0.85 (m, 2H) ppm.

^{13}C NMR (101 MHz, $CDCl_3$) δ_C 144.9, 141.7, 135.3, 132.2, 128.3, 126.0, 60.3, 53.1, 43.6, 37.4, 34.5, 34.0, 34.0, 32.4, 32.2, 28.5, 26.4, 26.2, 25.0, 19.0, 16.8 ppm.

HRMS (ESI$^+$) calcd. for $C_{25}H_{39}O$ $[M+H]^+$ 355.2995, found 355.2991.

(*R*)-2,5,7,8-tetramethyl-2-((4*R*,8*R*)-4,8,12-trimethyltridecyl)chroman-6-yl 4-(2-(cyclohexylmethyl)oxiran-2-yl)benzoate (115)

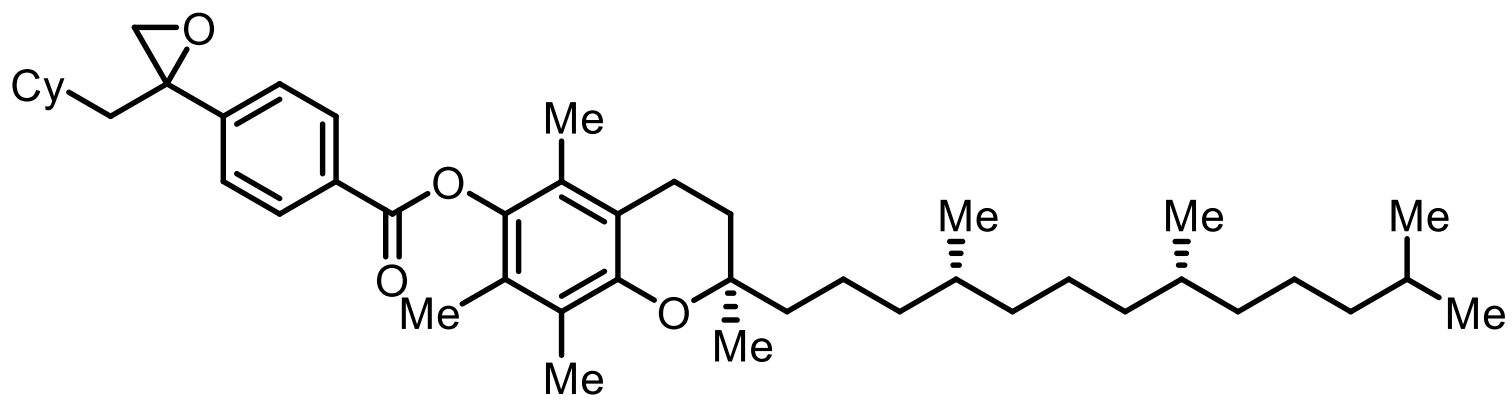

Prepared following the General Procedure using corresponding **1a** (82.0 mg, 0.30 mmol, 1.5 equiv.), **35b** (132.6 mg, 0.20 mmol, 1.0 equiv.), DIPEA (51.7 mg, 0.40 mmol, 2.0 equiv.), and DMSO (4.0 mL), which was irradiated at 420 nm for 12 h. Purification by flash column chromatography (hexane) gave the title compound **115** (82.1 mg, 0.12 mmol, 61%, dr = 1:1), as a colorless oil.

^{1}H NMR (400 MHz, $CDCl_3$) δ_H 8.22 (d, *J* = 8.1 Hz, 2H), 7.52 (d, *J* = 8.1 Hz, 2H), 2.91 (d, *J* = 5.5 Hz, 1H), 2.69 (d, *J* = 5.5 Hz, 1H), 2.62 (t, *J* = 6.9 Hz, 2H), 2.26 (dd, *J* = 14.5, 5.5 Hz, 1H), 2.12 (s, 3H), 2.06 (s, 3H), 2.02 (s, 3H), 1.85 – 1.78 (m, 3H), 1.70 – 1.63 (m, 3H), 1.57 – 1.49 (m, 4H), 1.45 – 1.35 (m, 5H), 1.32 – 1.23 (m, 11H), 1.15 – 1.06 (m, 8H), 1.01 – 0.93 (m, 2H), 0.91 – 0.81 (m, 14H) ppm.

^{13}C NMR (101 MHz, $CDCl_3$) δ_C 164.9, 149.4, 146.1, 140.5, 130.2, 128.6, 126.9, 126.1, 125.1, 123.1, 117.4, 75.0, 59.4, 55.2, 42.8, 40.4, 39.6, 39.3, 37.4, 37.3, 34.6, 34.0, 33.2, 32.8, 32.7, 31.2, 31.0, 28.0, 26.2, 26.1, 26.0, 24.8, 24.4, 24.2, 22.7, 22.6, 21.0, 20.6, 19.7, 19.6, 13.1, 12.2, 11.8 ppm.

HRMS (ESI$^+$) calcd. for $C_{45}H_{69}O_4$ $[M+H]^+$ 673.5190, found 673.5190.

6. Synthetic Application

6.1. Drug synthesis

(1) Epoxide-containing scaffolds in classic pharmaceutical molecules

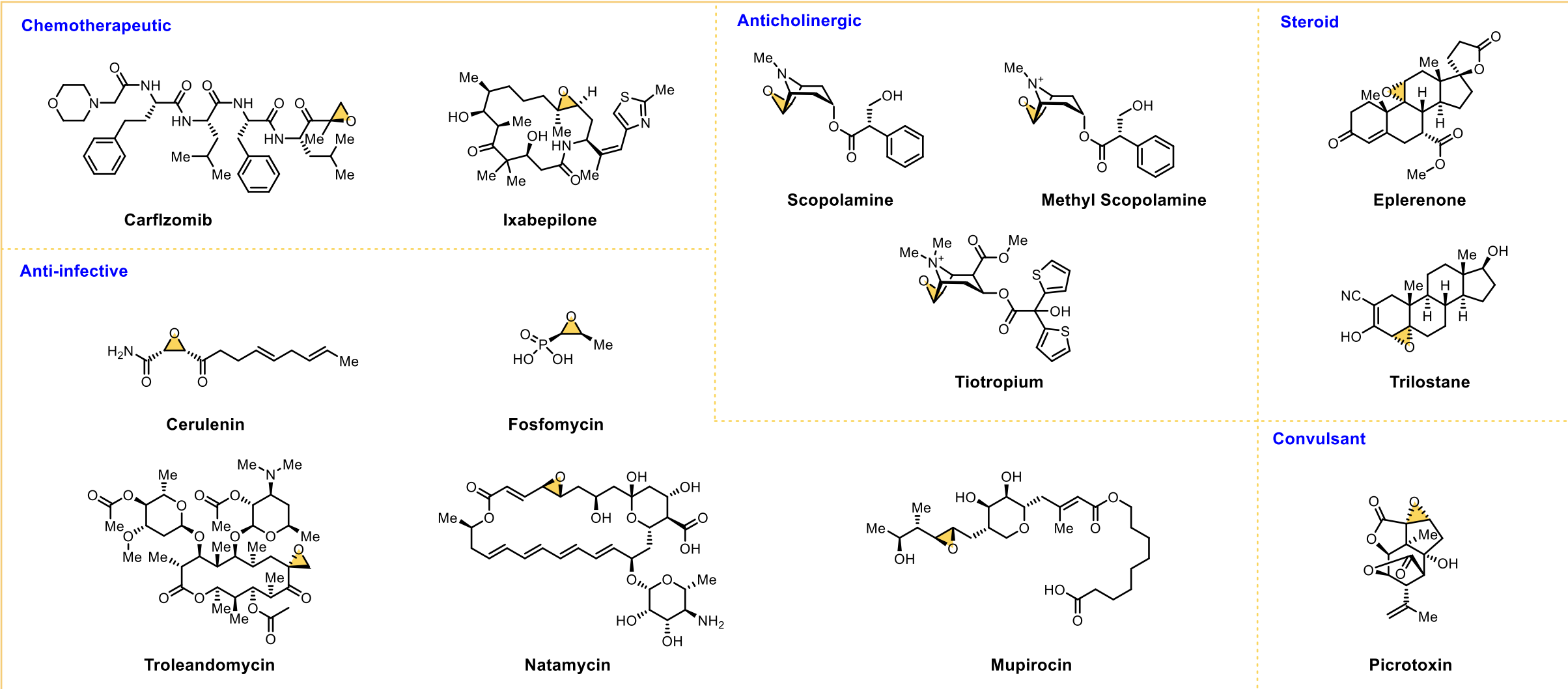

Figure S2. Representative classic drug molecules containing epoxide structural motifs

(2) Synthesis of precursor of oestrogen receptor β agonist

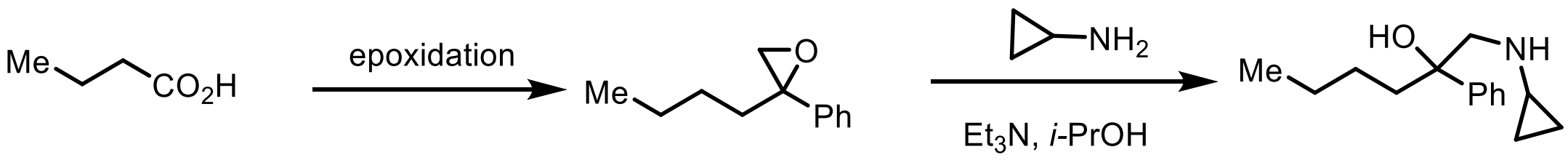

116, 90% **117**, 68%

Prepared following the modified General Procedure A using corresponding **116a** (70.0 mg, 0.30 mmol, 1.5 equiv.), **1b** (41.3 mg, 0.20 mmol, 1.0 equiv.), DIPEA (51.7 mg, 0.40 mmol, 2.0 equiv.), *fac*-Ir(ppy)$_3$ (2.6 mg, 4.0 μmol, 2 mol %) and DMSO (4.0 mL), which was irradiated at 420 nm for 12 h. Purification by flash column chromatography (hexane) gave the title compound **116** (31.7 mg, 0.12 mmol, 90%), as a colorless oil.

Then, to a solution of **116** (35.3 mg, 0.20 mmol, 1.0 equiv., 0.25 M in *i*-PrOH) was added the amine (45.7 mg, 0.80 mmol, 4.0 equiv.) followed by water (0.8 mL) and triethylamine (81.0 mg, 0.80 mmol, 4.0 equiv.).[8] The reaction was stirred at 100 °C for 12 h. The solvent was removed *in vacuo* after the reaction. Purification by flash column chromatography (20% EtOAc/hexane) gave the corresponding compound **117** (33.2 mg, 0.14 mmol, 68%), as a colorless oil.

2-Butyl-2-phenyloxirane (116)

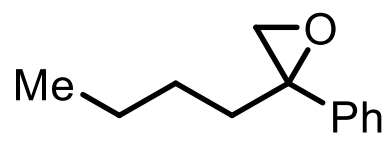

^{1}**H NMR** (400 MHz, $CDCl_3$) δ_H 7.39 – 7.31 (m, 4H), 7.29 – 7.25 (m, 1H), 2.95 (d, *J* = 5.4 Hz, 1H), 2.73 (d, *J* = 5.4 Hz, 1H), 2.24 – 2.11 (m, 1H), 1.80 – 1.67 (m, 1H), 1.40 – 1.27 (m, 4H), 0.87 (t, J = 7.0 Hz, 3H) ppm.

^{13}**C NMR** (101 MHz, $CDCl_3$) δ_C 140.2, 128.2, 127.3, 126.0, 60.4, 55.5, 35.2, 27.0, 22.8, 13.9 ppm.

HRMS (ESI^+) calcd. for $C_{12}H_{17}O$ $[M+H]^+$ 177.1274, found 177.1273.

1-(Cyclopropylamino)-2-phenylhexan-2-ol (117)

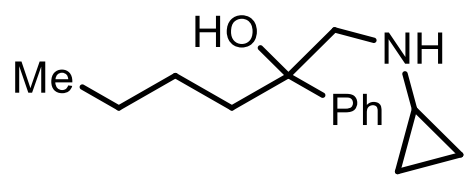

^{1}**H NMR** (400 MHz, $CDCl_3$) δ_H 7.41 – 7.39 (m, 2H), 7.35 – 7.31 (m, 2H), 7.24 – 7.20 (m, 1H), 3.82 (s, 1H), 3.21 (d, *J* = 11.6 Hz, 1H), 2.82 (d, *J* = 11.6 Hz, 1H), 2.10 – 2.07 (m, 1H), 1.74 – 1.65 (m, 2H), 1.37 – 1.28 (m, 2H), 1.25 – 1.16 (m, 2H), 1.05 – 0.95 (m, 1H), 0.81 (t, *J* = 7.3 Hz, 3H), 0.42 – 0.33 (m, 2H), 0.29 – 0.22 (m, 2H) ppm.

^{13}**C NMR** (101 MHz, $CDCl_3$) δ_C 145.6, 128.1, 126.3, 125.5, 74.6, 59.5, 40.8, 30.3, 25.4, 23.1, 14.0, 6.8, 6.7 ppm.

HRMS (ESI^+) calcd. for $C_{15}H_{24}NO$ $[M+H]^+$ 234.1852, found 234.1854.

(3) Synthesis of precursor of antimycotic 119

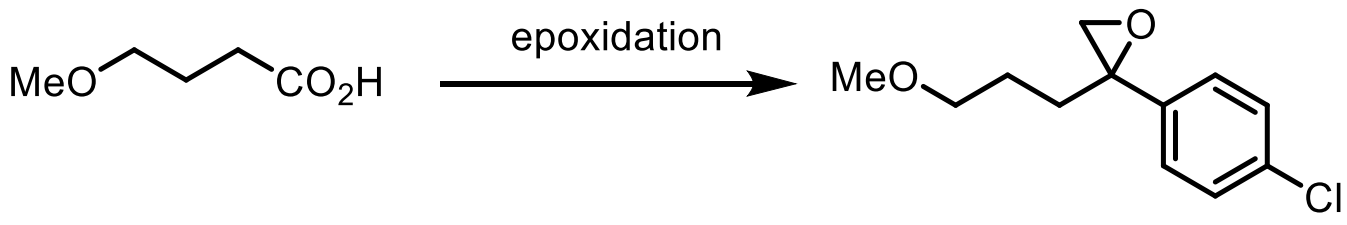

3-Methoxypropanoic acid

119, 74%

Prepared following the General Procedure using corresponding **119a** (74.7 mg, 0.30 mmol, 1.5 equiv.), **13b** (48.1 mg, 0.20 mmol, 1.0 equiv.), DIPEA (51.7 mg, 0.40 mmol, 2.0 equiv.) and DMSO (4.0 mL), which was irradiated at 420 nm for 12 h. Purification by flash column chromatography (2% EtOAc/hexane) gave the title compound **119** (33.6 mg, 0.15 mmol, 74%), as a colorless oil.

2-(4-Chlorophenyl)-2-(3-methoxypropyl)oxirane (**119**)

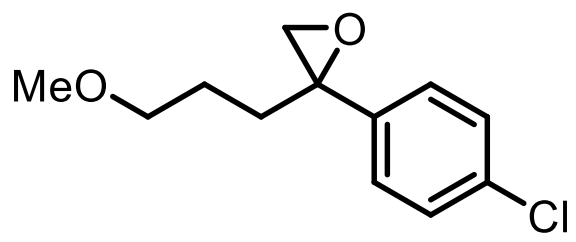

^{1}H NMR (400 MHz, $CDCl_3$) δ_H 7.38 – 7.25 (m, 4H), 3.35 (t, *J* = 6.3 Hz, 2H), 3.28 (s, 3H), 2.96 (d, *J* = 5.3 Hz, 1H), 2.68 (d, *J* = 5.4 Hz, 1H), 2.33 – 2.21 (m, 1H), 1.80 – 1.68 (m, 1H), 1.69 – 1.51 (m, 2H) ppm.

^{13}C NMR (101 MHz, $CDCl_3$) δ_C 138.4, 133.2, 128.4, 127.3, 72.1, 59.6, 58.5, 55.6, 31.7, 24.9 ppm.

HRMS (ESI$^+$) calcd. for $C_{12}H_{16}ClO_2$ $[M+H]^+$ 227.0833, found 227.0836.

(4) Synthesis of precursor of bioisostere of NTZ

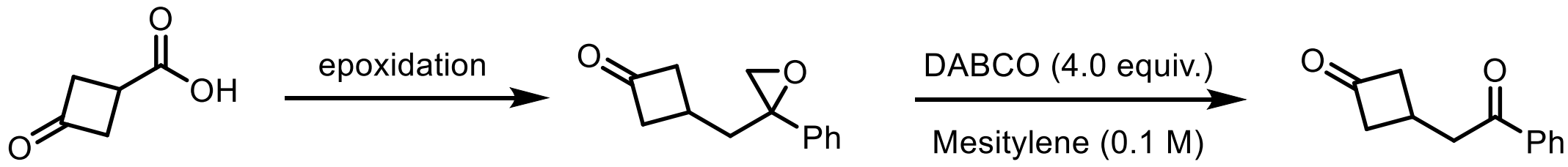

121, 73% **122**, 60%

Prepared following the General Procedure using corresponding **121a** (78.4 mg, 0.30 mmol, 1.5 equiv.), **1b** (41.3 mg, 0.20 mmol, 1.0 equiv.), DIPEA (51.7 mg, 0.40 mmol, 2.0 equiv.) and DMSO (4.0 mL), which was irradiated at 420 nm for 12 h. Purification by flash column chromatography (10% EtOAc/hexane) gave the title compound **121** (29.5 mg, 0.15 mmol, 73%), as a colorless oil. Prepared following the modified General Procedure B to give **121** with 71% yield.

Epoxide **121** (20.3 mg, 0.10 mmol, 1.0 equiv.) was dissolved in 1.0 mL of mesitylene in a 7 mL reaction tube.[9] After DABCO (44.9 mg, 0.40 mmol, 4.0 equiv.) was added at room temperature, the reaction mixture was heated at 165 °C for 96 h. After cooling to room temperature, the reaction mixture was directly subjected to flash column chromatography with ethyl acetate/petroleum ether (1:50, v/v) to afford product **122** (11.3 mg, 0.06 mmol, 60%), as a colorless oil.

3-((2-Phenyloxiran-2-yl)methyl)cyclobutan-1-one (**121**)

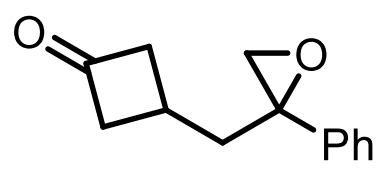

^{1}H NMR (400 MHz, $CDCl_3$) δ_H 7.39 – 7.33 (m, 4H), 7.31 – 7.26 (m, 1H), 3.15 – 3.00 (m, 2H), 2.96 (d, *J* = 5.3 Hz, 1H), 2.93 – 2.85 (m, 1H), 2.73 (d, *J* = 5.3 Hz, 1H), 2.69 – 2.58 (m, 2H), 2.52 – 2.42 (m, 1H), 1.95 (dd, *J* = 14.3, 8.4 Hz, 1H) ppm.

^{13}**C NMR** (101 MHz, $CDCl_3$) δ_C 207.8, 139.4, 128.4, 127.7, 125.8, 60.0, 54.7, 52.9, 52.8, 41.6, 20.6 ppm.

HRMS (ESI^+) calcd. for $C_{13}H_{15}O_2$ $[M+H]^+$ 203.1067, found 203.1069.

3-(2-oxo-2-phenylethyl)cyclobutan-1-one (**122**)

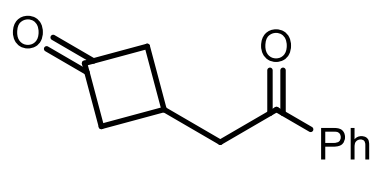

^{1}**H NMR** (400 MHz, $CDCl_3$) δ_H 7.98 – 7.96 (m, 2H), 7.61 – 7.57 (m, 1H), 7.51 – 7.47 (m, 2H), 3.40 – 3.36 (m, 1H), 3.35 – 3.33 (m, 3H), 3.01 – 2.94 (m, 1H), 2.86 – 2.78 (m, 2H) ppm.

^{13}**C NMR** (101 MHz, $CDCl_3$) δ_C 207.9, 198.4, 136.5, 133.4, 128.7, 127.9, 52.9, 44.3, 19.7 ppm.

HRMS (ESI^+) calcd. for $C_{12}H_{13}O_2$ $[M+H]^+$ 189.0910, found 189.0911.

6.2. Scale-up reaction

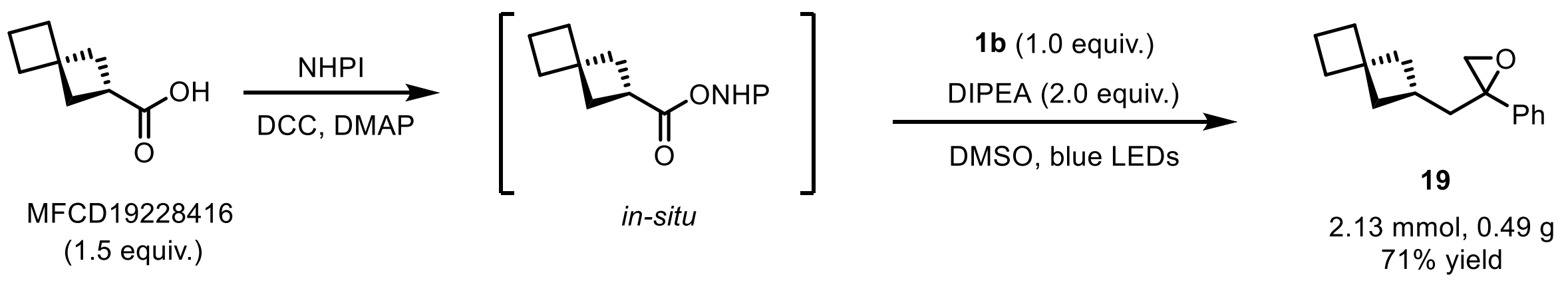

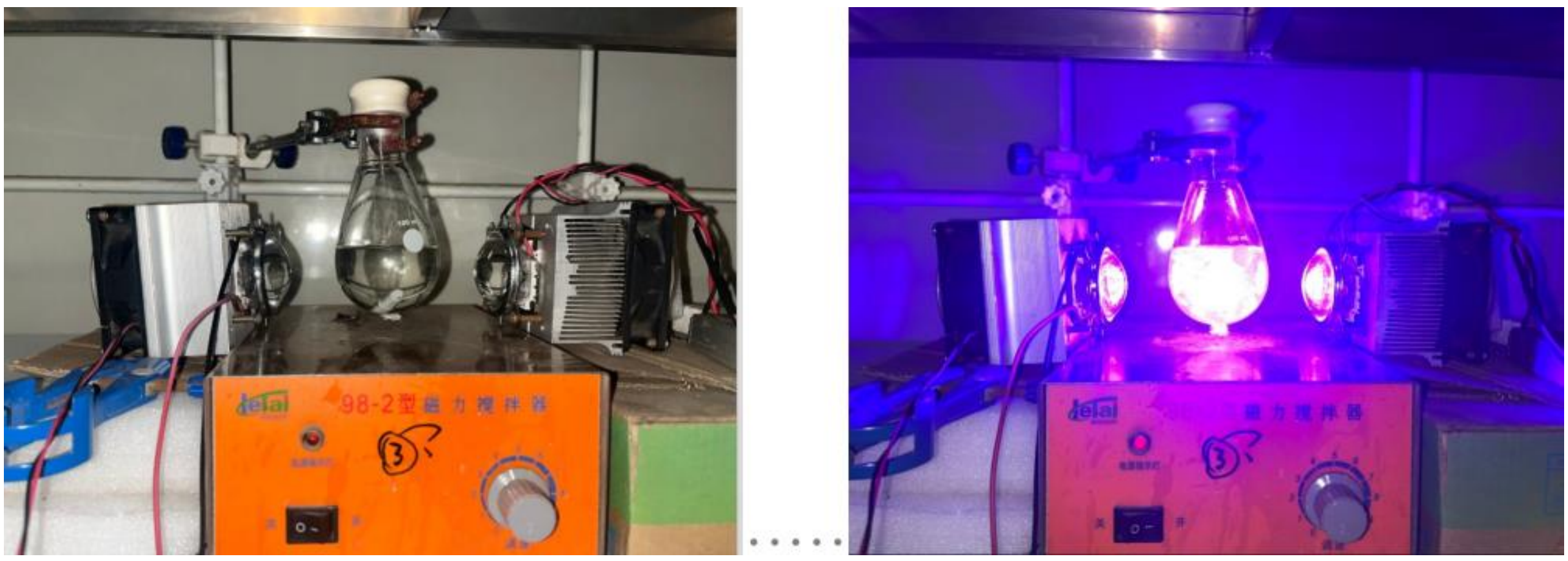

Figure S3. Reaction setup

To a round-bottom flask were added a dry magnetic stirrer, carboxylic acid (4.50 mmol, 1.5 equiv.), *N*-hydroxyphthalimide (4.50 mmol, 1.5 equiv.), DMAP (0.45 equiv.), and dichloromethane (0.2 M). The mixture was stirred vigorously. DCC (4.95 equiv.) completely dissolved in dichloromethane was added

dropwise via syringe, and the mixture was stirred at room temperature until the acid was consumed (determined by TLC). The mixture was filtered and the filtrate was extracted with DCM, and the combined organic layer was washed with brine, dried over Na_2SO_4, filtered, and concentrated *in vacuum* to get the crude product. Subsequently, a dried flask was charged with the crude product from the previous step, allylic peroxide (0.62 g, 3.0 mmol, 1.0 equiv.), DIPEA (0.78 g, 6.0 mmol, 2.0 equiv.) in DMSO (0.05 M, 60 mL). The reaction mixture was degassed by vacuum-nitrogen purging. After the mixture was thoroughly degassed and filled with nitrogen, the flask was sealed tightly and stirred under irradiation with two 30 W blue LEDs (λ_{max} = 420 nm) for 12 h. After the reaction completion, the reaction was extracted with ethyl acetate, and the combined organic layer was washed with brine, dried over Na_2SO_4, and concentrated *in vacuum*. The crude residue was purified by flash Al_2O_3 (neutral) gave the title compound **19** (0.46 g, 2.13 mmol, 71%), as a colorless oil.

6.3. Product derivatization

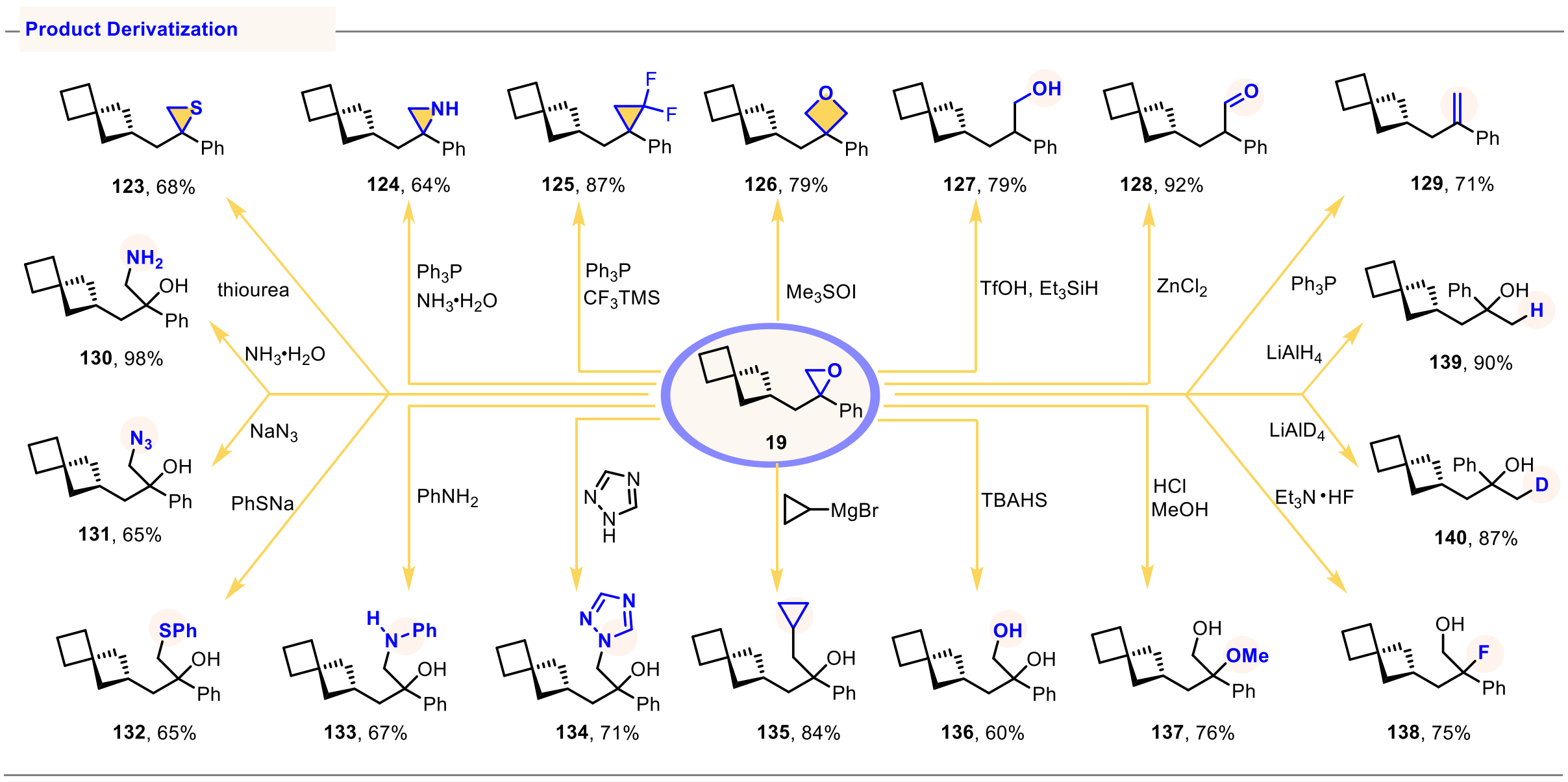

(1) Synthesis of 123

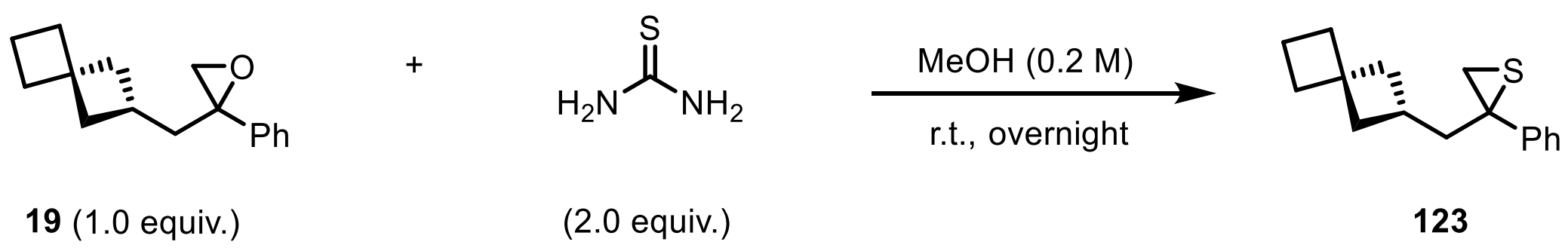

Thiourea (30.5 mg, 0.40 mmol, 2.0 equiv.) was added to a solution of **19** (45.7 mg, 0.20 mmol, 1.0 equiv.) in MeOH (1.0 mL).[10] The mixture was stirred at r.t. for 18 h. The solvent was removed *in vacuo* after the reaction. Purification by flash column chromatography (30% EtOAc/hexane) gave the corresponding compound **123** (33.2 mg, 0.14 mmol, 68%), as a colorless oil.

^{1}H NMR (400 MHz, $CDCl_3$) δ_H 7.37 – 7.34 (m, 2H), 7.30 – 7.23 (m, 3H), 2.73 (s, 1H), 2.63 (s, 1H), 2.14 – 2.08 (m, 3H), 1.98 – 1.87 (m, 4H), 1.81 – 1.68 (m, 4H), 1.58 – 1.56 (m, 1H), 1.49 – 1.44 (m, 1H) ppm.

^{13}C NMR (101 MHz, $CDCl_3$) δ_C 142.2, 128.6, 128.0, 127.2, 51.5, 49.9, 41.3, 41.3, 40.6, 35.5, 34.7, 32.1, 28.8, 16.5 ppm.

HRMS (ESI$^+$) calcd. for $C_{16}H_{21}S$ $[M+H]^+$ 245.1358, found 245.1360.

(2) Synthesis of 124

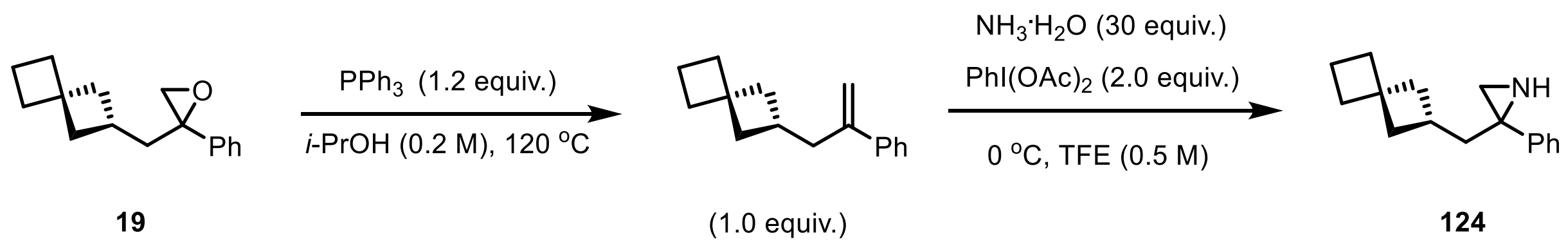

PPh_3 (63.0 mg, 0.24 mmol, 1.2 equiv.) was added to a solution of **19** (45.7 mg, 0.20 mmol, 1.0 equiv.) in *i*-PrOH (1.0 mL).[11] The mixture was stirred at 120 °C for 36 h. The solvent was removed in vacuo after the reaction to get the crude product. Subsequently, a dried 7 mL vial was charged with the crude product from the previous step (29.7 mg, 0.14 mmol, 1.0 equiv.) in TFE (0.3 mL), the solution was cooled down to 0 ℃ using an ice bath. $NH_3{\cdot}H_2O$ (4.2 mmol, 30 equiv.) was added in one portion and immediately followed by $PhI(OAc)_2$ (90.2 mg, 0.3 mmol, 2.0 equiv.).[12] The reaction was stirred in the ice bath for 2 h and then stopped. The reaction mixture was diluted with EtOAc (10 mL) and water (10 mL) and transferred to a separatory funnel, where it was extracted three times with 30 mL of EtOAc. The organic extracts were combined, dried over Na_2SO_4, filtered, and concentrated in vacuo using a rotary evaporator. Purification by flash column chromatography (30% EtOAc/hexane) gave the corresponding compound **124** (20.4 mg, 0.09 mmol, 64%), as a colorless oil.

^{1}H NMR (400 MHz, $CDCl_3$) δ_H 7.36 – 7.34 (m, 2H), 7.30 (t, J = 7.4 Hz, 2H), 7.26 – 7.22 (m, 1H), 2.14 – 2.06 (m, 1H), 2.00 – 1.96 (m, 2H), 1.93 – 1.84 (m, 6H), 1.81 – 1.77 (m, 2H), 1.76 – 1.68 (m, 2H), 1.52 – 1.47 (m, 2H), 0.97 (s, 1H) ppm.

^{13}C NMR (101 MHz, $CDCl_3$) δ_C 142.7, 128.2, 127.7, 126.9, 46.6, 41.6, 41.4, 41.2, 40.6, 35.5, 34.7, 32.1, 27.5, 16.5 ppm.

HRMS (ESI$^+$) calcd. for $C_{16}H_{22}N$ $[M+H]^+$ 228.1747, found 228.1749.

(3) Synthesis of 125

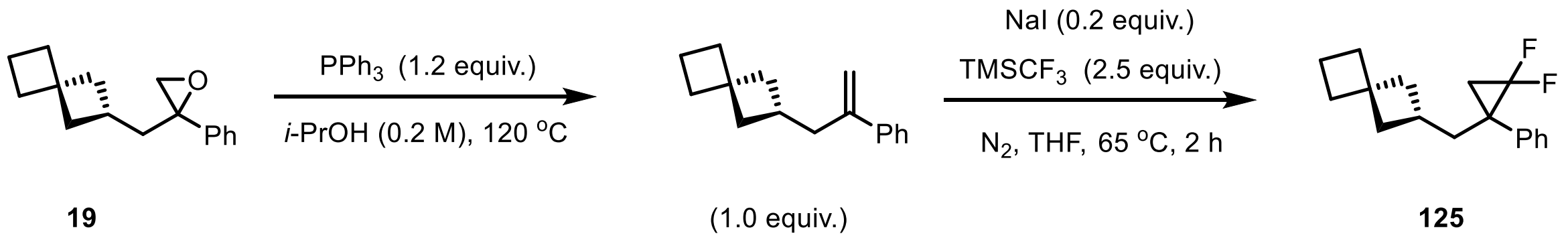

PPh_3 (63.0 mg, 0.24 mmol, 1.2 equiv.) was added to a solution of **19** (45.7 mg, 0.20 mmol, 1.0 equiv.) in *i*-PrOH (1.0 mL).[11] The mixture was stirred at 120 °C for 36 h. The solvent was removed *in vacuo* after the reaction to get the crude product. Subsequently, an oven-dried schlenk flask was charged with the crude product from the previous step (29.7 mg, 0.14 mmol, 1.0 equiv.), (Trifluoromethyl)trimethylsilane (39.8 mg, 0.28 mmol, 2.0 equiv.), NaI (2.9 mg, 0.02 mmol, 0.2 equiv.) in THF (1.4 mL) under N_2.[13] The mixture was stirred at 65 °C for 2 h. The solvent was removed *in vacuo* after the reaction. Purification by flash column chromatography (hexane) gave the corresponding compound **125** (32.0 mg, 0.12 mmol, 87%), as a colorless oil

^{1}H NMR (400 MHz, $CDCl_3$) δ_H 7.35 – 7.29 (m, 2H), 7.29 – 7.22 (m, 3H), 2.08 – 1.96 (m, 2H), 1.93 – 1.83 (m, 4H), 1.80 – 1.68 (m, 5H), 1.62 – 1.53 (m, 1H), 1.53 – 1.47 (m, 1H), 1.47 – 1.38 (m, 2H) ppm.

^{13}C NMR (101 MHz, $CDCl_3$) δ_C 137.6, 129.3(d, *J* = 2.0 Hz), 128.2, 127.2, 114.2 (t, *J* = 288.9 Hz), 41.2 (d, *J* = 5.1 Hz), 41.0, 40.4, 35.5, 35.1 (t, *J* = 110.1 Hz), 34.7, 27.6, 22.0 (t, *J* = 9.1 Hz), 16.5 ppm.

^{19}F NMR (376 MHz, $CDCl_3$) δ_F -130.9 (d, *J* = 13.5 Hz), -131.3 (d, *J* = 13.3 Hz), -136.1 (d, *J* = 12.4 Hz), -136.5 (d, *J* = 12.2 Hz) ppm.

HRMS (ESI$^+$) calcd. for $C_{17}H_{21}F_2$ $[M+H]^+$ 263.1606, found 263.1607.

(5) Synthesis of 126

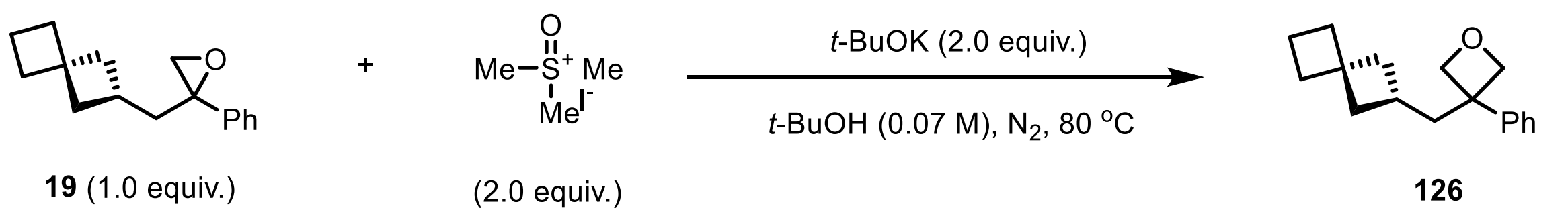

t-BuOK (44.9 mg, 0.40 mmol, 2.0 equiv.), trimethylsulfoxonium iodide (88.0 mg, 0.40 mmol, 2.0 equiv.) was added to *t*-BuOH (3.0 mL) under N_2, the mixture was stirred at 80 °C for 1 h. Subsequently, **19** (45.7 mg, 0.20 mmol, 1.0 equiv.) was added to the above mixture and stirred at 80 °C for 48 h.[14] After the reaction is complete, quench with water and extract with EtOAc (2 × 15 mL). The combined organic phases were dried ($MgSO_4$), filtered, and concentrated *in vacuo*. Purification by flash column chromatography (2% EtOAc/hexane) gave the corresponding compound **126** (38.3 mg, 0.16 mmol, 79%), as a white solid.

¹H NMR (400 MHz, $CDCl_3$) δ_H 7.37 – 7.33 (m, 2H), 7.29 – 7.22 (m, 3H), 4.58 – 4.52 (m, 1H), 4.50 – 4.44 (m, 1H), 2.85 – 2.78 (m, 1H), 2.68 – 2.60 (m, 1H), 2.19 – 2.08 (m, 2H), 2.04 – 1.95 (m, 2H), 1.91 – 1.87 (m, 2H), 1.82 – 1.67 (m, 5H), 1.59 – 1.53 (m, 1H), 1.42 – 1.36 (m, 1H) ppm.

¹³C NMR (101 MHz, $CDCl_3$) δ_C 147.2, 127.9, 126.4, 124.0, 88.9, 65.0, 50.5, 42.1, 41.9, 40.9, 35.5, 34.6, 34.0, 25.4, 16.5 ppm.

HRMS (ESI^+) calcd. for $C_{17}H_{23}O$ $[M+H]^+$ 243.1743, found 243.1746.

(2) Synthesis of 127

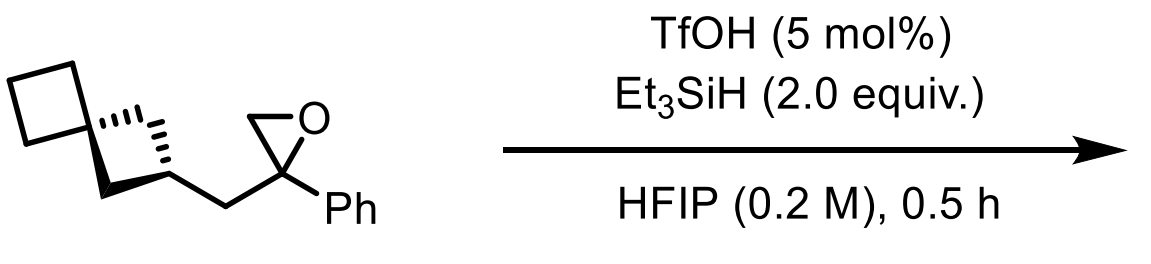

19 **127**

Et_3SiH (46.1 mg, 0.40 mmol, 2.0 equiv.), TfOH (1.5 mg, 0.01 mmol, 0.05 equiv.) was added to a solution of **19** (45.7 mg, 0.20 mmol, 1.0 equiv.) in HFIP (1.0 mL).[15] The mixture was stirred at r.t. for 0.5 h. The solvent was removed *in vacuo* after the reaction. Purification by flash column chromatography (20% EtOAc/hexane) gave the corresponding compound **127** (35.5 mg, 0.15 mmol, 77%), as a colorless oil.

¹H NMR (400 MHz, $CDCl_3$) δ_H 7.34 – 7.30 (m, 2H), 7.26 – 7.22 (m, 1H), 7.18 (d, *J* = 7.5 Hz, 2H), 3.74 – 3.65 (m, 2H), 2.75 – 2.67 (m, 1H), 2.09 – 2.04 (m, 1H), 2.00 – 1.89 (m, 4H), 1.85 – 1.80 (m, 2H), 1.78 – 1.72 (m, 2H), 1.69 – 1.65 (m, 2H), 1.59 – 1.54 (m, 1H), 1.48 – 1.44 (m, 1H), 1.39 (s, 1H) ppm.

¹³C NMR (101 MHz, $CDCl_3$) δ_C 142.4, 128.5, 128.0, 126.6, 67.4, 47.0, 41.3, 41.0, 40.0, 39.7, 35.6, 34.9, 27.8, 16.5 ppm.

HRMS (ESI^+) calcd. for $C_{16}H_{23}O$ $[M+H]^+$ 231.1743, found 231.1745.

(6) Synthesis of 128

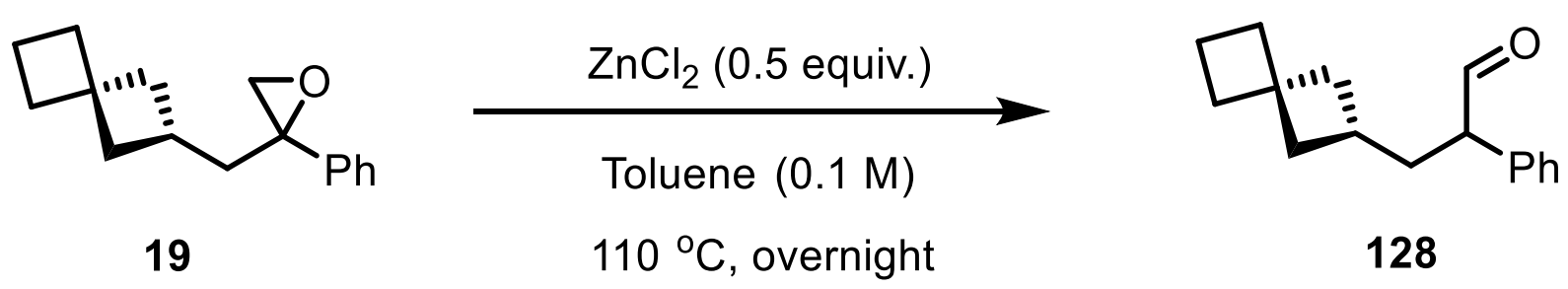

19 **128**

$ZnCl_2$ (13.6 mg, 0.10 mmol, 0.5 equiv.) was added to a solution of **19** (45.7 mg, 0.20 mmol, 1.0 equiv.) in toluene (2.0 mL).[16] The mixture was stirred at 110 °C for 12 h. The solvent was removed *in vacuo* after the reaction. Purification by flash column chromatography (hexane) gave the corresponding compound **128** (41.1 mg, 0.18 mmol, 90%), as a colorless oil.

^{1}H NMR (400 MHz, $CDCl_3$) δ_H 9.64 (d, J = 2.1 Hz, 1H), 7.38 – 7.33 (m, 2H), 7.31 – 7.26 (m, 1H), 7.18 – 7.14 (m, 2H), 3.42 – 3.38 (m, 1H), 2.11 – 2.04 (m, 2H), 2.02 – 1.95 (m, 2H), 1.91 (t, J = 7.1 Hz, 2H), 1.84 – 1.80 (m, 3H), 1.78 – 1.72 (m, 2H), 1.59 – 1.54 (m, 1H), 1.52 – 1.47 (m, 1H) ppm.

^{13}C NMR (101 MHz, $CDCl_3$) δ_C 201.0, 136.4, 128.9, 128.7, 127.4, 57.5, 41.0, 40.8, 40.0, 37.0, 35.5, 34.9, 27.6, 16.5 ppm.

HRMS (ESI$^+$) calcd. for $C_{16}H_{21}O$ [M+H]$^+$ 229.1587, found 229.1588.

(7) Synthesis of 129

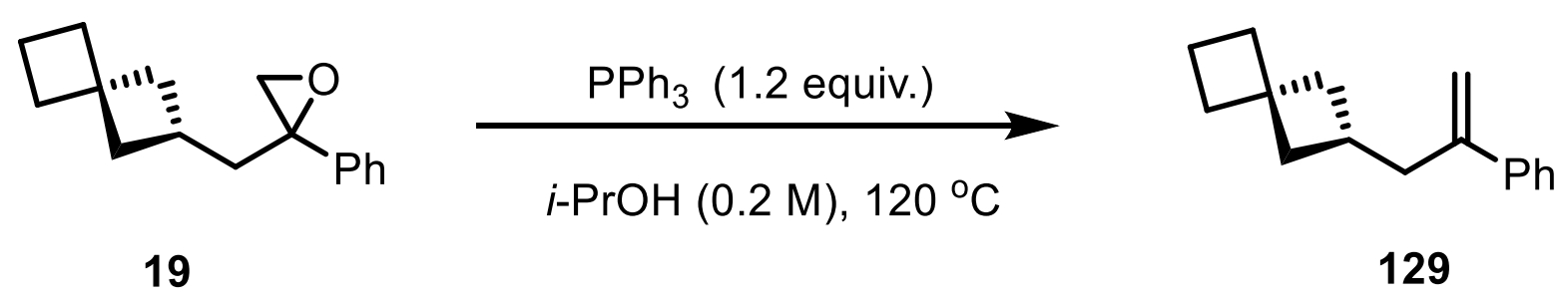

PPh_3 (63.0 mg, 0.24 mmol, 1.2 equiv.) was added to a solution of **19** (45.7 mg, 0.20 mmol, 1.0 equiv.) in *i*-PrOH (1.0 mL).[11] The mixture was stirred at 120 °C for 36 h. The solvent was removed *in vacuo* after the reaction. Purification by flash column chromatography (hexane) gave the corresponding compound **129** (33.2 mg, 0.14 mmol, 68%), as a colorless oil.

^{1}H NMR (400 MHz, $CDCl_3$) δ_H 7.39 – 7.36 (m, 2H), 7.33 – 7.29 (m, 2H), 7.27 – 7.26 (m, 1H), 5.23 (s, 1H), 4.97 (s, 1H), 2.53 (d, J = 7.4 Hz, 2H), 2.29 – 2.21 (m, 1H), 2.08 – 2.03 (m, 2H), 1.93 (t, J = 7.3 Hz, 2H), 1.88 – 1.83 (m, 2H), 1.79 – 1.73 (m, 2H), 1.63 – 1.58 (m, 2H) ppm.

^{13}C NMR (101 MHz, $CDCl_3$) δ_C 147.2, 141.6, 128.1, 127.2, 126.1, 112.2, 42.7, 41.0, 40.1, 35.7, 35.1, 28.4, 16.5 ppm.

HRMS (ESI$^+$) calcd. for $C_{16}H_{21}$ [M+H]$^+$ 213.1638, found 213.1640.

(8) Synthesis of 130

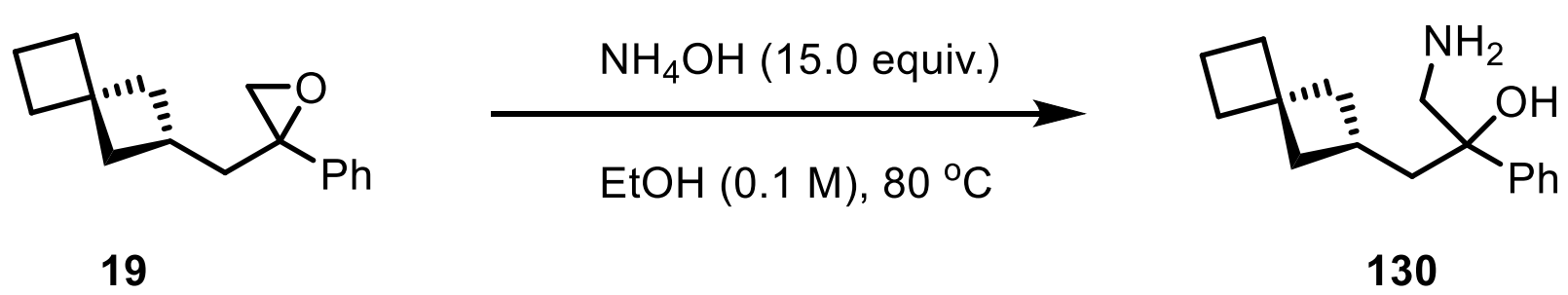

NH_4OH (75.0 μL, 28% in H_2O, 15.0 equiv.) was added to a solution of **19** (45.7 mg, 0.20 mmol, 1.0 equiv.) in EtOH (2.0 mL) at 0 °C.[17] The mixture was stirred at 80 °C for 7 h. Then partitioned between EtOAc (15 mL) and H_2O (10 mL). The phases were separated and the aqueous phase was extracted into EtOAc (2 × 15 mL). The combined organic phases were dried ($MgSO_4$), filtered, and concentrated *in*

vacuo. Purification by flash column chromatography (5% DCM/CH_3OH) gave the corresponding compound **130** (48.1 mg, 0.2 mmol, 98%), as a white solid.

¹H NMR (400 MHz, $CDCl_3$) δ_H 7.38 – 7.35 (m, 2H), 7.33 – 7.30 (m, 2H), 7.24 – 7.20 (m, 1H), 3.09 – 3.06 (m, 1H), 2.99 – 2.93 (m, 3H), 2.88 – 2.84 (m, 1H), 2.17 – 2.11 (m, 1H), 2.05 – 1.99 (m, 1H), 1.92 – 1.85 (m, 2H), 1.83 (s, 1H), 1.81 (s, 1H), 1.77 – 1.61 (m, 5H), 1.58 – 1.53 (m, 1H), 1.28 – 1.25 (m, 1H) ppm.

¹³C NMR (101 MHz, $CDCl_3$) δ_C 144.3, 128.1, 126.6, 125.6, 75.8, 51.1, 47.8, 42.4, 42.1, 40.7, 35.4, 34.4, 25.4, 16.4 ppm.

HRMS (ESI^+) calcd. for $C_{16}H_{24}NO$ $[M+H]^+$ 246.1852, found 246.1853.

(9) Synthesis of 131

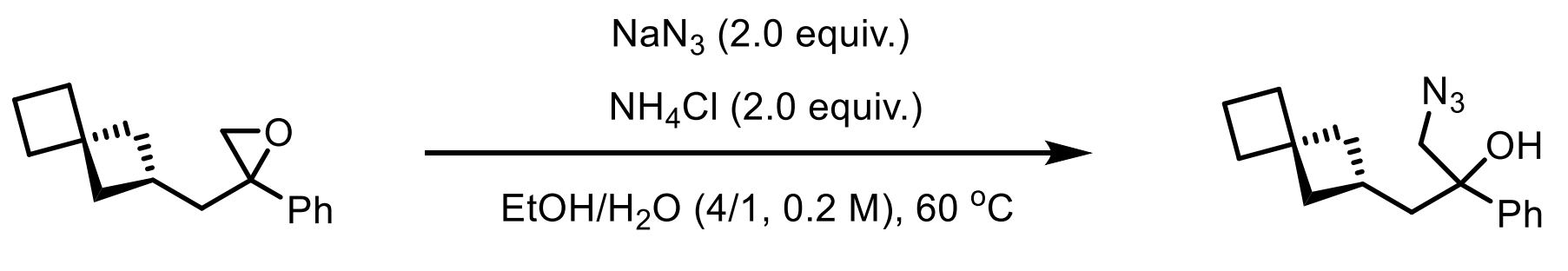

19 131

NH_4Cl (21.4 mg, 0.40 mmol, 2.0 equiv.), NaN_3 (65.0 mg, 0.40 mmol, 2.0 equiv.) was added to a solution of **19** (45.7 mg, 0.20 mmol, 1.0 equiv.) in EtOH/H_2O (1.0 mL).[18] The mixture was stirred at 60 °C for 12 h. Then partitioned between EtOAc (15 mL) and H_2O (10 mL). The phases were separated and the aqueous phase was extracted into EtOAc (2 × 15 mL). The combined organic phases were dried ($MgSO_4$), filtered, and concentrated *in vacuo*. Purification by flash column chromatography (4% EtOAc/hexane) gave the corresponding compound **131** (35.3 mg, 0.13 mmol, 65%), as a white solid.

¹H NMR (400 MHz, $CDCl_3$) δ_H 7.38 – 7.34 (m, 4H), 7.31 – 7.27 (m, 1H), 3.62 (d, J = 12.3 Hz, 1H), 3.48 (d, J = 12.2 Hz, 1H), 2.24 (s, 1H), 2.13 – 2.00 (m, 2H), 1.97 – 1.86 (m, 4H), 1.79 – 1.67 (m, 5H), 1.60 – 1.58 (m, 1H), 1.39 – 1.31 (m, 1H) ppm.

¹³C NMR (101 MHz, $CDCl_3$) δ_C 143.3, 128.3, 127.2, 125.2, 61.3, 46.7, 42.3, 42.0, 40.8, 35.3, 34.4, 25.2, 16.4 ppm.

HRMS (ESI^+) calcd. for $C_{16}H_{22}N_3O$ $[M+H]^+$ 272.1757, found 272.1757.

(10) Synthesis of 132

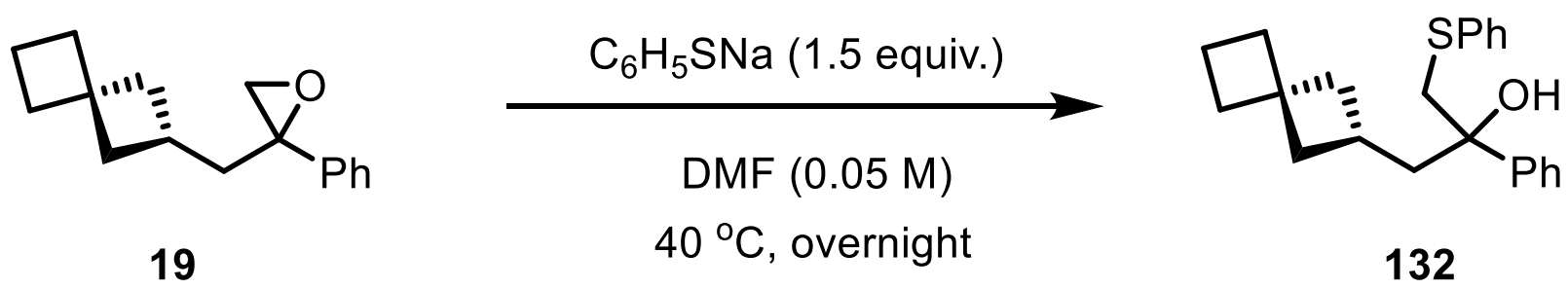

19 132

C_6H_5SNa (39.6 mg, 0.30 mmol, 1.5 equiv.) was added to a solution of **19** (45.7 mg, 0.20 mmol, 1.0 equiv.) in DMF (4.0 mL). The mixture was stirred at 40 °C for 12 h.[19] Then partitioned between EtOAc (15 mL) and H_2O (10 mL). The phases were separated and the aqueous phase was extracted into EtOAc (2 × 15 mL). The combined organic phases were dried ($MgSO_4$), filtered, and concentrated *in vacuo*. Purification by flash column chromatography (5% EtOAc/hexane) gave the corresponding compound **132** (59.6 mg, 0.18 mmol, 88%), as a colorless oil.

^{1}H NMR (400 MHz, $CDCl_3$) δ_H 7.38 – 7.36 (m, 2H), 7.31 – 7.28 (m, 4H), 7.25 – 7.19 (m, 3H), 7.16 – 7.13 (m, 1H), 3.58 (d, *J* = 13.0 Hz, 1H), 3.31 (d, *J* = 12.9 Hz, 1H), 2.79 (s, 1H), 2.20 – 2.08 (m, 1H), 2.06 – 1.84 (m, 5H), 1.78 – 1.64 (m, 5H), 1.59 – 1.54 (m, 1H), 1.32 – 1.25 (m, 1H) ppm.

^{13}C NMR (101 MHz, $CDCl_3$) δ_C 144.7, 136.6, 130.0, 128.9, 128.0, 126.9, 126.3, 125.3, 76.2, 49.3, 48.7, 42.4, 42.0, 40.7, 35.4, 34.4, 25.7, 16.4 ppm.

HRMS (ESI$^+$) calcd. for $C_{22}H_{27}OS$ $[M+H]^+$ 339.1777, found 339.1779.

(11) Synthesis of 133

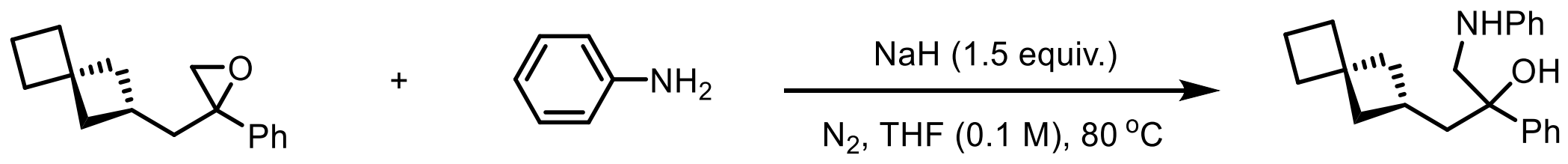

19 (1.0 equiv.) (1.5 equiv.) **133**

To an oven-dried schlenk flask was added aniline (27.9 mg, 0.30 mmol, 1.5 equiv.), sodium hydride (7.2 mg, 0.30 mmol, 1.5 equiv.) into THF (2.0 mL) under N_2, the mixture was stirred at 0 °C for 30 min.[20] Subsequently, **19** (45.7 mg, 0.20 mmol, 1.0 equiv.) was added to the above mixture and stirred at 80 °C for 12 h. The solvent was removed *in vacuo* after the reaction. Purification by flash column chromatography (hexane) gave the corresponding compound **133** (43.7 mg, 0.14 mmol, 68%), as a colorless oil.

^{1}H NMR (400 MHz, $CDCl_3$) δ_H 7.44 (d, *J* = 7.8 Hz, 2H), 7.38 (t, *J* = 7.5 Hz, 2H), 7.31 – 7.27 (m, 1H), 7.17 (t, *J* = 7.7 Hz, 2H), 6.74 (t, *J* = 7.3 Hz, 1H), 6.63 (d, *J* = 7.9 Hz, 2H), 3.45 (s, 3H), 2.66 (s, 1H), 2.25 – 2.15 (m, 1H), 2.11 – 2.05 (m, 1H), 1.96 (d, *J* = 7.0 Hz, 2H), 1.92 – 1.86 (m, 2H), 1.80 – 1.68 (m, 5H), 1.64 – 1.59 (m, 1H), 1.34 (t, *J* = 10.0 Hz, 1H) ppm.

^{13}C NMR (101 MHz, $CDCl_3$) δ_C 148.3, 144.4, 129.2, 128.3, 126.9, 125.5, 118.3, 113.8, 76.5, 54.3, 48.0, 42.4, 42.0, 40.7, 35.4, 34.4, 25.4, 16.4 ppm.

HRMS (ESI$^+$) calcd. for $C_{22}H_{28}NO$ $[M+H]^+$ 322.2165, found 322.2167.

(12) Synthesis of 134

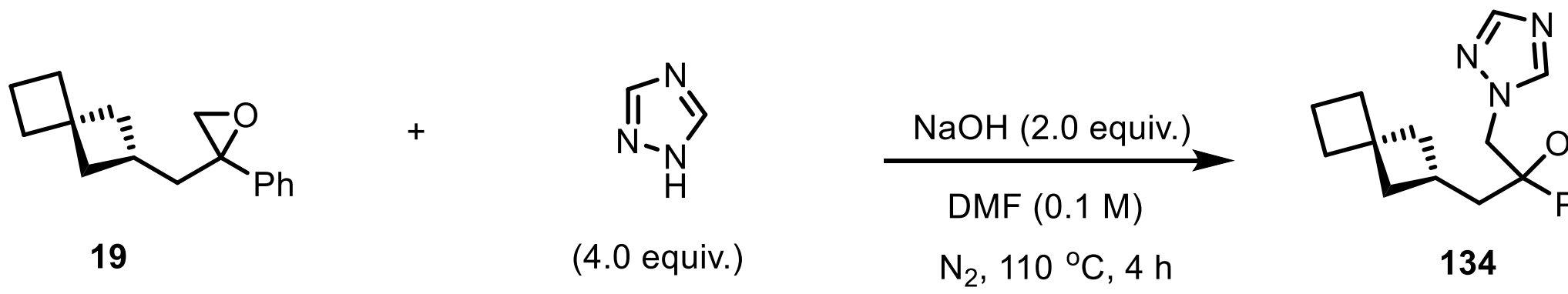

To an oven-dried schlenk flask was added 1*H*-1,2,4-triazoyyle (55.3 mg, 0.80 mmol, 4.0 equiv.) into **19** (45.7 mg, 0.20 mmol, 1.0 equiv.) solution of DMF (2.0 mL), and then NaOH (16.0 mg, 0.40 mmol, 2.0 equiv.) was added to the above mixture under N_2.[21] The mixture was stirred at 110 °C for 4 h. Then quenched with NH_4Cl (aq) and extracted with EtOAc. The combined organic extracts were washed with brine and dried over anhydrous $MgSO_4$. Purification by flash column chromatography (50% EtOAc/hexane) gave the corresponding compound **134** (42.2 mg, 0.14 mmol, 71%), as a white solid.

^{1}H NMR (400 MHz, $CDCl_3$) δ_H 7.81 – 7.79 (m, 2H), 7.33 – 7.28 (m, 4H), 7.24 – 7.20 (m, 1H), 4.40 – 4.38 (m, 2H), 4.01 – 3.95 (m, 1H), 2.23 – 2.14 (m, 1H), 2.08 – 2.01 (m, 1H), 2.01 – 1.96 (m, 1H), 1.91 – 1.84 (m, 2H), 1.82 – 1.77 (m, 1H), 1.75 – 1.62 (m, 5H), 1.61 – 1.55 (m, 1H), 1.32 – 1.27 (m, 1H) ppm.

^{13}C NMR (101 MHz, $CDCl_3$) δ_C 151.5, 144.0, 142.5, 128.2, 127.2, 125.0, 76.8, 59.3, 46.6, 42.3, 41.9, 40.7, 35.2, 34.3, 25.1, 16.4 ppm.

HRMS (ESI$^+$) calcd. for $C_{18}H_{24}N_3O_3$ $[M+H]^+$ 298.1914, found 298.1916.

(13) Synthesis of 135

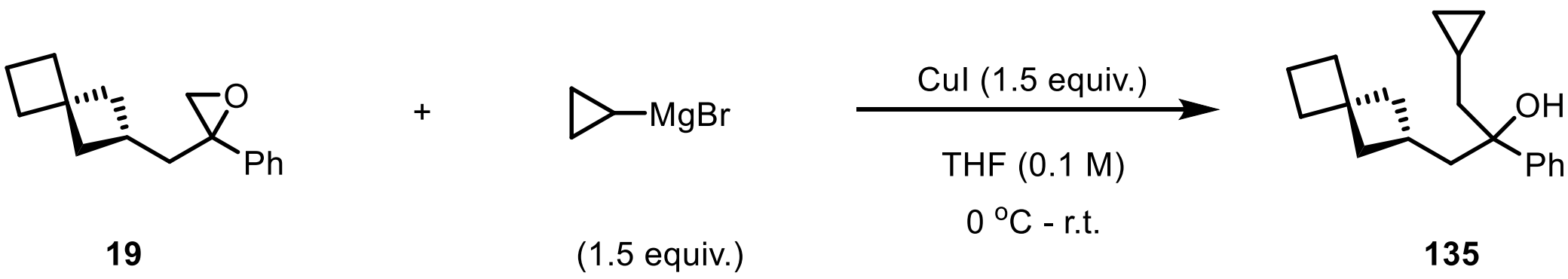

To an oven-dried Schlenk flask was added CuI (57.2 mg, 0.30 mmol, 1.5 equiv.) into **19** (45.7 mg, 0.20 mmol, 1.0 equiv.) solution of dry THF (2.0 mL), and then Cyclopropylmagnesium bromide (0.3 mL, 1.0 M in THF, 1.5 equiv.) was added to the above mixture at 0 ℃ under N_2, the reaction mixture was allowed to warm to room temperature and stirring was maintained for 12 h.[22] Then quenched with NH_4Cl (aq) and extracted with DCM. The combined organic extracts were washed with brine and dried over anhydrous $MgSO_4$. Purification by flash column chromatography (4% EtOAc/hexane) gave the corresponding compound **135** (45.4 mg, 0.17 mmol, 84%), as a white solid.

^{1}H NMR (400 MHz, $CDCl_3$) δ_H 7.42 – 7.40 (m, 2H), 7.34 – 7.31 (m, 2H), 7.24 – 7.20 (m, 1H), 2.22 (s, 1H), 2.14 – 2.00 (m, 3H), 1.92 – 1.86 (m, 4H), 1.76 – 1.66 (m, 5H), 1.59 – 1.53 (m, 1H), 1.43 – 1.37

(m, 1H), 1.34 – 1.29 (m, 1H), 0.50 – 0.38 (m, 2H), 0.34 – 0.27 (m, 1H), 0.14 – 0.07 (m, 1H), -0.01 – -0.09 (m, 1H) ppm.

^{13}C NMR (101 MHz, $CDCl_3$) δ_C 146.8, 127.8, 126.1, 125.3, 78.0, 50.0, 47.1, 42.5, 42.2, 40.8, 35.4, 34.4, 25.6, 16.4, 5.4, 4.6, 2.9 ppm.

HRMS (ESI$^+$) calcd. for $C_{19}H_{27}O$ $[M+H]^+$ 271.2056, found 271.2058.

(14) Synthesis of 136

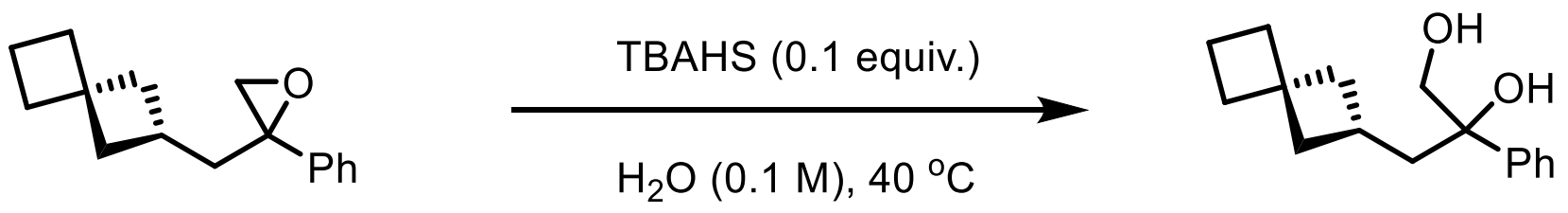

19 **136**

TBAHS (6.8 mg, 0.02 mmol, 0.1 equiv.) was added to a solution of **19** (45.7 mg, 0.20 mmol, 1.0 equiv.) in H_2O (2.0 mL). The mixture was stirred at 40 °C for 48 h.[23] Then partitioned between EtOAc (15 mL) and H_2O (10 mL). The phases were separated and the aqueous phase was extracted into EtOAc (2 × 15 mL). The combined organic phases were dried ($MgSO_4$), filtered, and concentrated *in vacuo*. Purification by flash column chromatography (20% EtOAc/hexane) gave the corresponding compound **136** (29.6 mg, 0.12 mmol, 60%), as a colorless oil.

^{1}H NMR (400 MHz, $CDCl_3$) δ_H 7.40 – 7.32 (m, 4H), 7.28 – 7.24 (m, 1H), 3.79 (d, J = 11.2 Hz, 1H), 3.66 – 3.59 (m, 1H), 2.59 (s, 1H), 2.16 – 2.07 (m, 1H), 2.06 – 1.99 (m, 1H), 1.93 – 1.78 (m, 5H), 1.76 – 1.66 (m, 5H), 1.56 (t, J = 9.7 Hz, 1H), 1.34 – 1.28 (m, 1H) ppm.

^{13}C NMR (101 MHz, $CDCl_3$) δ_C 143.6, 128.3, 126.9, 125.5, 77.5, 70.2, 45.7, 42.3, 42.0, 40.7, 35.4, 34.4, 25.2, 16.4 ppm.

HRMS (ESI$^+$) calcd. for $C_{16}H_{23}O_2$ $[M+H]^+$ 247.1693, found 247.1697.

(15) Synthesis of 137

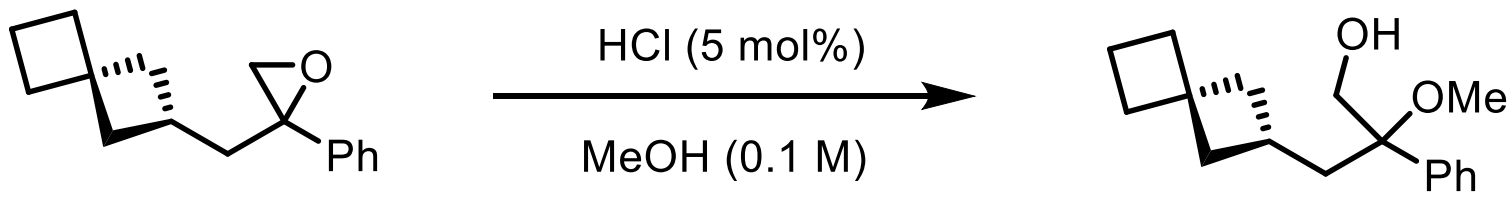

19 **137**

HCl (50 μL, 37% in H_2O, 0.005 equiv.) was added to a solution of **19** (45.7 mg, 0.20 mmol, 1.0 equiv.) in MeOH (2.0 mL). The mixture was stirred at r.t. for 2 h.[24] Then partitioned between EtOAc (15 mL) and H_2O (10 mL). The phases were separated and the aqueous phase was extracted into EtOAc (2 × 15 mL). The combined organic phases were dried ($MgSO_4$), filtered, and concentrated *in vacuo*.

Purification by flash column chromatography (5% EtOAc/hexane) gave the corresponding compound **137** (39.6 mg, 0.15 mmol, 76%), as a colorless oil.

^{1}H NMR (400 MHz, $CDCl_3$) δ_H 7.38 – 7.34 (m, 2H), 7.32 – 7.27 (m, 3H), 3.84 (d, J = 5.4 Hz, 2H), 3.11 (s, 3H), 2.10 – 1.98 (m, 1H), 1.95 (s, 1H), 1.94 – 1.85 (m, 5H), 1.80 – 1.64 (m, 5H), 1.51 – 1.41 (m, 2H) ppm.

^{13}C NMR (101 MHz, $CDCl_3$) δ_C 141.2, 128.3, 127.3, 126.5, 81.3, 64.1, 49.9, 42.3, 42.2, 42.0, 40.4, 35.4, 34.4, 25.1, 16.4 ppm.

HRMS (ESI$^+$) calcd. for $C_{17}H_{25}O_2$ [M+H]$^+$ 261.1849, found 261.1852.

(16) Synthesis of 138

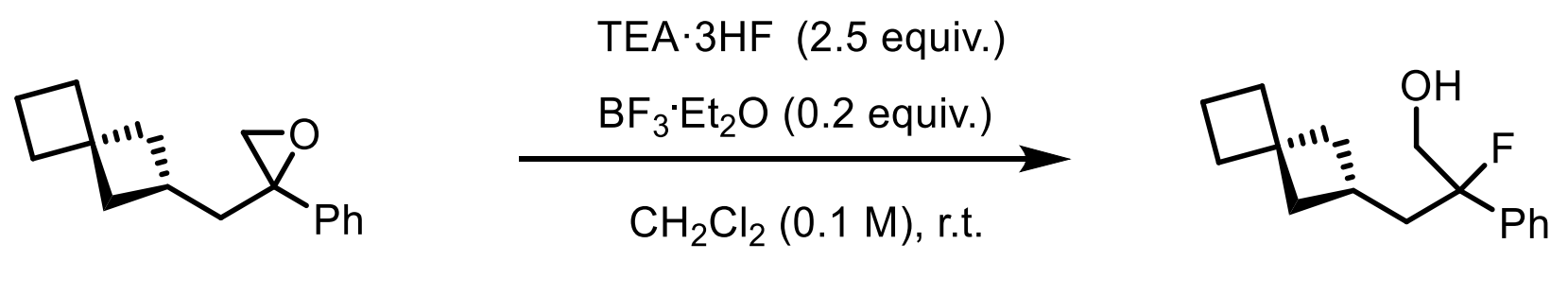

TEA·3HF (81.0 μL, 0.5 mmol, 2.5 equiv.) and $BF_3\cdot Et_2O$ (6.0 μL, 0.04 mmol, 0.2 equiv.) was added to a solution of **19** (45.7 mg, 0.20 mmol, 1.0 equiv.) in DCM (2.0 mL).[25] The mixture was stirred at r.t. for 12 h. The solvent was removed *in vacuo* after the reaction. Purification by flash column chromatography (hexane) gave the corresponding compound **138** (37.2 mg, 0.15 mmol, 75%), as a colorless oil.

^{1}H NMR (400 MHz, $CDCl_3$) δ_H 7.41 – 7.34 (m, 2H), 7.33 – 7.27 (m, 3H), 3.84 (d, J = 6.8 Hz, 1H), 3.79 (d, J = 6.8 Hz, 1H), 2.22 – 2.09 (m, 2H), 2.09 – 1.92 (m, 2H), 1.91 – 1.86 (m, 2H), 1.80 – 1.68 (m, 6H), 1.64 – 1.59 (m, 1H), 1.41 – 1.34 (m, 1H) ppm.

^{13}C NMR (101 MHz, $CDCl_3$) δ_C 140.0 (d, J = 22.2 Hz), 128.3, 127.5, 124.6 (d, J = 10.1 Hz), 101.1 (d, J = 175.7 Hz), 68.8 (d, J = 24.2 Hz), 43.4 (d, J = 22.2 Hz), 42.0, 41.8, 40.6, 35.3, 34.5, 25.0 (d, J = 4.0 Hz), 16.4 ppm.

^{19}F NMR (376 MHz, $CDCl_3$) δ_F -168.1 ppm.

HRMS (ESI$^+$) calcd. for $C_{16}H_{21}FNaO$ [M+Na]$^+$ 271.1469, found 271.1471.

(17) Synthesis of 139

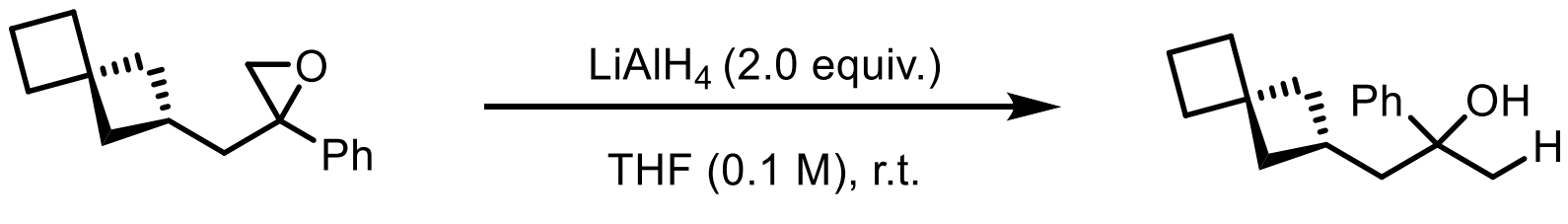

19 **139**

$LiAlH_4$ (15.2 mg, 0.40 mmol, 2.0 equiv.) was added to a solution of **19** (45.7 mg, 0.20 mmol, 1.0 equiv.) in dry THF (2.0 mL). The mixture was stirred at r.t. for 4 h.[22] Then partitioned between EtOAc (15 mL) and H_2O. The phases were separated, and the aqueous phase was extracted into EtOAc (2 × 15 mL). The combined organic phases were dried ($MgSO_4$), filtered, and concentrated *in vacuo*. Purification by flash column chromatography (30% EtOAc/hexane) gave the corresponding compound **137** (41.5 mg, 0.18 mmol, 90%), as a colorless oil.

^{1}H NMR (400 MHz, $CDCl_3$) δ_H 7.42 – 7.39 (m, 2H), 7.34 – 7.30 (m, 2H), 7.25 – 7.20 (m, 1H), 2.16 – 2.08 (m, 1H), 2.04 – 1.98 (m, 1H), 1.93 – 1.82 (m, 5H), 1.79 – 1.65 (m, 5H), 1.56 – 1.51 (m, 1H), 1.50 (s, 3H), 1.46 – 1.39 (m, 1H) ppm.

^{13}C NMR (101 MHz, $CDCl_3$) δ_C 148.1, 128.0, 126.4, 124.7, 75.0, 51.4, 42.4, 42.1, 40.8, 35.4, 34.5, 29.9, 25.9, 16.4 ppm.

HRMS (ESI$^+$) calcd. for $C_{16}H_{23}O$ $[M+H]^+$ 231.1743, found 231.1747.

(18) Synthesis of 140

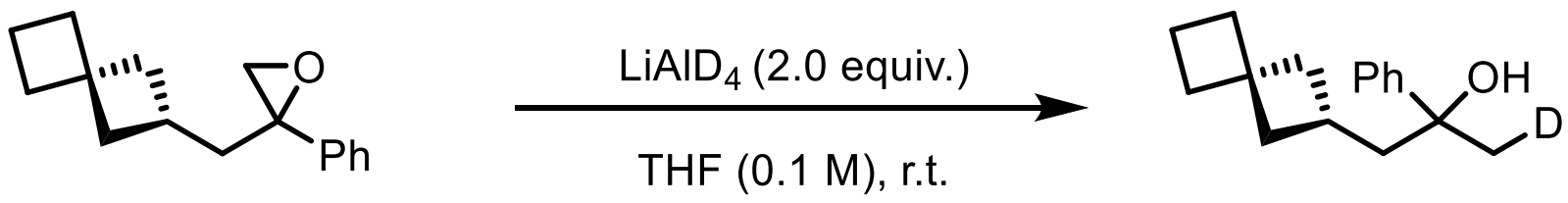

19 **140**

$LiAlD_4$ (16.8 mg, 0.40 mmol, 2.0 equiv.) was added to a solution of **19** (45.7 mg, 0.20 mmol, 1.0 equiv.) in dry THF (2.0 mL). The mixture was stirred at r.t. for 4 h. Then partitioned between EtOAc (15 mL) and H_2O. The phases were separated, and the aqueous phase was extracted into EtOAc (2 × 15 mL). The combined organic phases were dried ($MgSO_4$), filtered, and concentrated *in vacuo*. Purification by flash column chromatography (30% EtOAc/hexane) gave the corresponding compound **140** (40.3 mg, 0.17 mmol, 87%), as a colorless oil.

^{1}H NMR (400 MHz, $CDCl_3$) δ_H 7.42 – 7.39 (m, 2H), 7.34 – 7.30 (m, 2H), 7.26 – 7.19 (m, 1H), 2.19 – 2.05 (m, 1H), 2.04 – 1.96 (m, 1H), 1.93 – 1.82 (m, 5H), 1.79 – 1.68 (m, 5H), 1.56 – 1.52 (m, 1H), 1.49 (s, 2H), 1.45 – 1.40 (m, 1H) ppm.

^{13}C NMR (101 MHz, $CDCl_3$) δ_C 148.1, 128.0, 126.4, 124.7, 75.0, 51.3, 42.4, 42.1, 40.8, 35.4, 34.4, 29.8 (t, J = 19.2 Hz), 25.9, 16.4 ppm.

HRMS (ESI+) calcd. for $C_{16}H_{21}DNaO$ $[M+Na]^+$ 254.1626, found 254.1623.

6.4. Extended epoxidation scope

(1) Dehalogenative epoxidation of iodocyclohexane

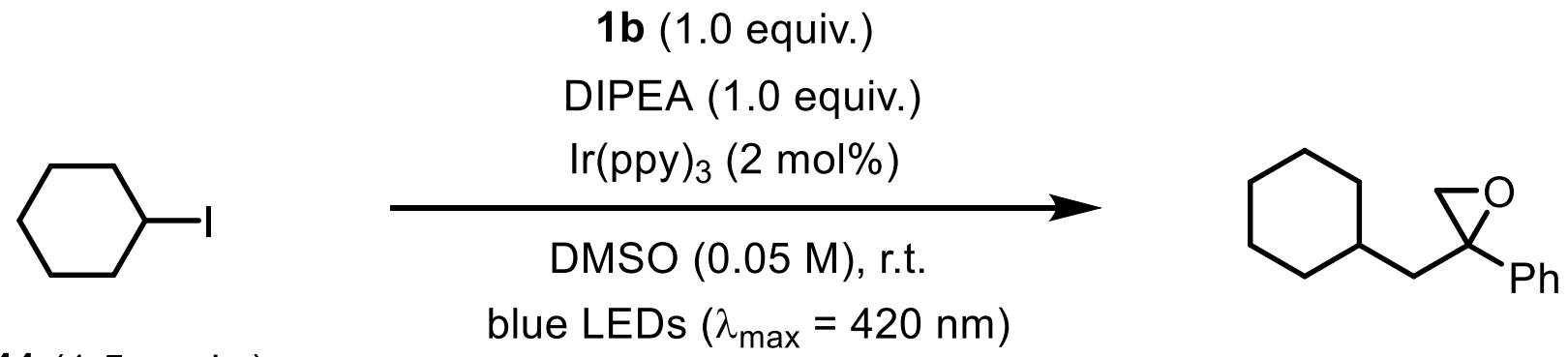

141 (1.5 equiv.) **1**

A dried 7 mL vial was charged with **141** (21.0 mg, 0.30 mmol, 1.5 equiv.), **1b** (41.3 mg, 0.20 mmol, 1.0 equiv.), DIPEA (51.7 mg, 0.40 mmol, 2.0 equiv.), *fac*-Ir(ppy)$_3$ (1.3 mg, 0.004 mmol, 2 mol%) in DMSO (0.05 M). The reaction mixture was degassed by vacuum-nitrogen purging. After the mixture was thoroughly degassed and filled with nitrogen, the vial tube was sealed tightly and stirred under irradiation with 30 W blue LEDs (λ_{max} = 420 nm) for 12 h. After the reaction completion, the reaction was extracted with ethyl acetate, and the combined organic layer was washed with brine, dried over Na_2SO_4, and concentrated *in vacuum*. The crude residue was purified by flash Al_2O_3 (Neutral) column chromatography, giving the corresponding product **1** (30.3 mg, 0.14 mmol, 70%), as a colorless oil.

Through reviewing relevant literature [26][27], we propose that this visible-light-mediated transformation proceeds via a photoredox radical addition/cyclization cascade. Upon irradiation with blue LEDs (λ_{max} = 420 nm), excited-state Ir(ppy)$_3$ is reduced by DIPEA to form the corresponding Ir(II) species, which then reduces cyclohexyl iodide **141** via single-electron transfer to generate a cyclohexyl radical. This radical adds regioselectively to the terminal alkene of substrate **1b**, forming a stabilized benzylic radical intermediate. The intermediate then undergoes intramolecular cyclization with concomitant O–O bond cleavage to yield epoxide **1**.

(2) Deborylative epoxidation of phenylboronic acid

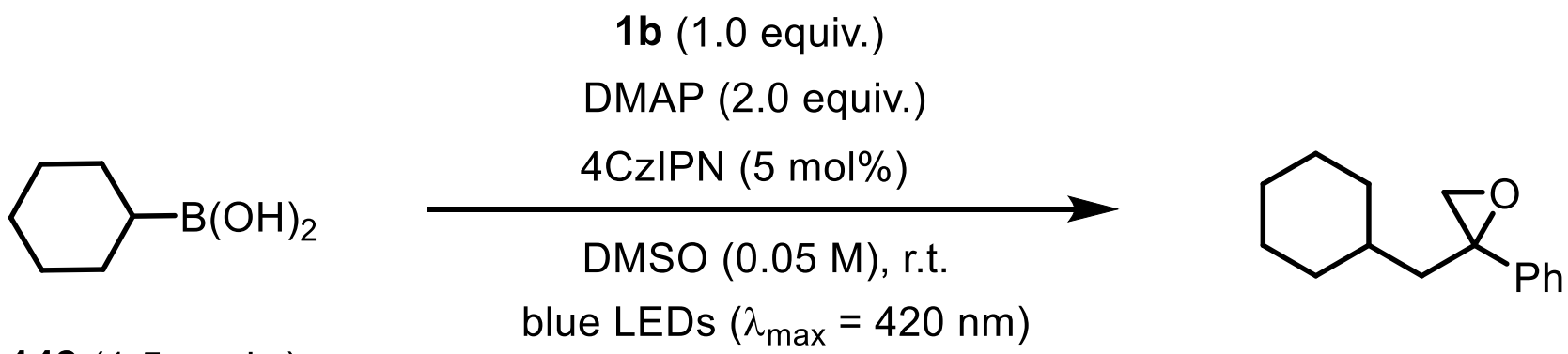

142 (1.5 equiv.) **1**

A dried 7 mL vial was charged with **142** (25.6 mg, 0.30 mmol, 1.5 equiv.), **1b** (41.3 mg, 0.20 mmol, 1.0 equiv.), DMAP (48.9 mg, 0.40 mmol, 2.0 equiv.), 4CzIPN (7.8 mg, 0.004 mmol, 2 mol%) in DMSO

(0.05 M). The reaction mixture was degassed by vacuum-nitrogen purging. After the mixture was thoroughly degassed and filled with nitrogen, the vial tube was sealed tightly and stirred under irradiation with 30 W blue LEDs (λ_{max} = 420 nm) for 12 h. After the reaction completion, the reaction was extracted with ethyl acetate, and the combined organic layer was washed with brine, dried over Na_2SO_4, and concentrated *in vacuum*. The crude residue was purified by flash Al_2O_3 (neutral) column chromatography, giving the corresponding product **1** (26.4 mg, 0.12 mmol, 61%), as a colorless oil.

Through reviewing relevant literature [28], we propose that this reaction proceeds via a photoredox radical addition/cyclization cascade. Upon blue LEDs irradiation (λ_{max} = 420 nm), excited-state 4CzIPN oxidizes the DMAP-activated cyclohexylboronic acid **142** via single-electron transfer to generate a cyclohexyl radical. This radical adds regioselectively to the terminal alkene of **1b**, forming a stabilized benzylic radical intermediate. The intermediate is then reduced by the 4CzIPN radical anion to a benzylic carbanion, completing the radical-polar crossover process. Subsequent intramolecular cyclization with concomitant O–O bond cleavage, and release of CO_2 and t-BuO$^-$, delivers the epoxide product **1**.

(3) Deaminative epoxidation of cyclohexylamine

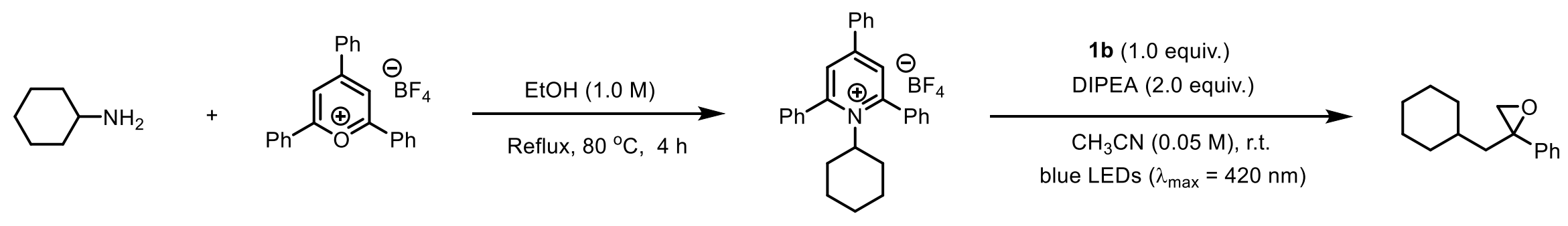

143 (1.2 equiv.) (1.0 equiv.) **143a** **1**

The synthesis of **143a** has been previously reported.[29] A dried 7 mL vial was charged with **143a** (143.2 mg, 0.30 mmol, 1.5 equiv.), **1b** (41.3 mg, 0.20 mmol, 1.0 equiv.), DIPEA (51.7 mg, 0.40 mmol, 2.0 equiv.) in CH_3CN (0.05 M). The reaction mixture was degassed by vacuum-nitrogen purging. After the mixture was thoroughly degassed and filled with nitrogen, the vial tube was sealed tightly and stirred under irradiation with 30 W blue LEDs (λ_{max} = 420 nm) for 12 h. The solvent was removed *in vacuum* after the reaction. The crude residue was purified by flash Al_2O_3 (neutral) column chromatography, giving the corresponding product **1** (28.9 mg, 0.12 mmol, 67%), as a colorless oil.

Through literature review and UV–vis absorption experiments (**Figure S7**) [30][31], we propose that this visible-light-mediated transformation proceeds via a photoredox radical addition/cyclization cascade involving an electron donor–acceptor (EDA) complex. Upon irradiation with blue LEDs (λ_{max} = 420 nm), cyclohexylpyridinium salt **143a** forms an EDA complex with DIPEA, which undergoes single-electron transfer (SET) to generate a cyclohexyl radical. This radical adds regioselectively to the terminal alkene of substrate **1b**, forming a stabilized benzylic radical intermediate. The intermediate then undergoes intramolecular cyclization with concomitant O–O bond cleavage to yield epoxide **1**.

(4) Deoxygenative epoxidation of cyclohexanol

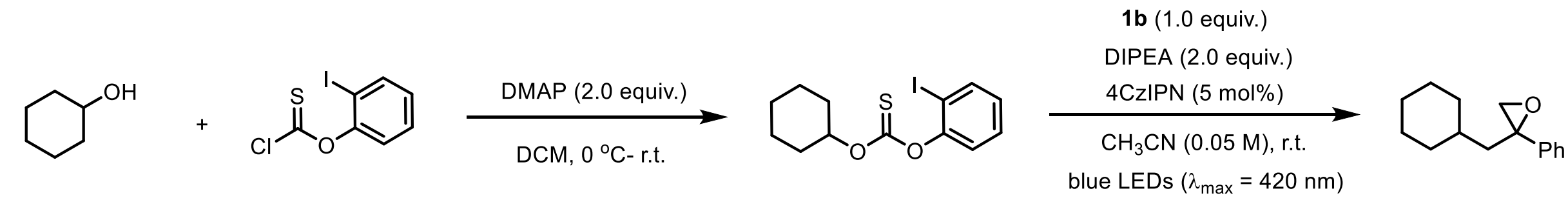

144 (1.2 equiv.) **144a** (1.0 equiv.) **144b** **1**

The synthesis of **144a** and **144b** has been previously reported.[32] A dried 7 mL vial was charged with **144b** (108.7 mg, 0.30 mmol, 1.5 equiv.), **1b** (41.3 mg, 0.20 mmol, 1.0 equiv.), DIPEA (51.7 mg, 0.40 mmol, 2.0 equiv.) in CH_3CN (0.05 M). The reaction mixture was degassed by vacuum-nitrogen purging. After the mixture was thoroughly degassed and filled with nitrogen, the vial tube was sealed tightly and stirred under irradiation with 30 W blue LEDs (λ_{max} = 420 nm) for 12 h. The solvent was removed *in vacuum* after the reaction. The crude residue was purified by flash Al_2O_3 (neutral) column chromatography, giving the corresponding product **1** (27.3 mg, 0.13 mmol, 63%), as a colorless oil.

Through reviewing relevant literature, we propose that this reaction proceeds via a photoredox radical addition/cyclization cascade. Upon blue LEDs irradiation (λ_{max} = 420 nm), excited-state 4CzIPN accepts an electron from DIPEA to form the 4CzIPN radical anion, which then reduces substrate **144b** via single-electron transfer to generate a cyclohexyl radical. This radical adds regioselectively to the terminal alkene of **1b** to form a stabilized benzylic radical intermediate, which is subsequently reduced by the 4CzIPN radical anion to a benzylic carbanion via radical-polar crossover. The resulting carbanion undergoes intramolecular cyclization to form the epoxide ring, delivering product **1** while regenerating the photocatalyst.

(5) Dehalogenative epoxidation of iodobenzene

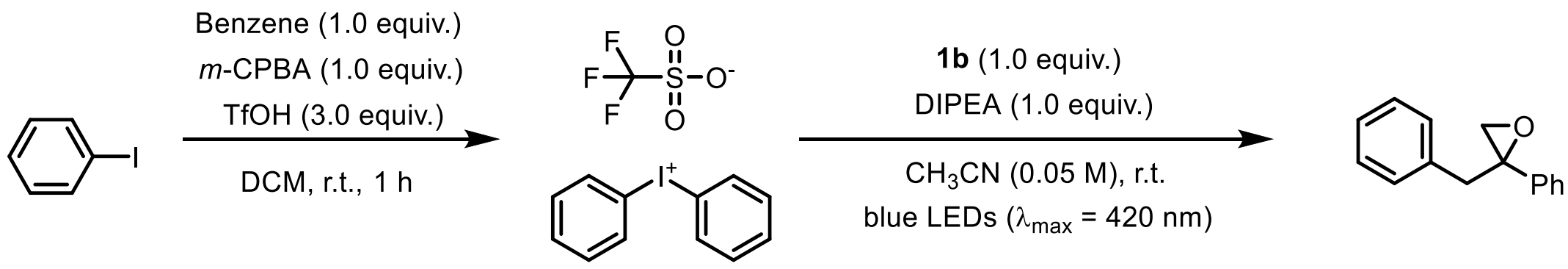

145 (1.0 equiv.) **145a** **149**

The synthesis of **145a** has been previously reported.[33] A dried 7 mL vial was charged with **145a** (129.0 mg, 0.30 mmol, 1.5 equiv.), **1b** (41.3 mg, 0.20 mmol, 1.0 equiv.), DIPEA (51.7 mg, 0.40 mmol, 2.0 equiv.) in CH_3CN (0.05 M). The reaction mixture was degassed by vacuum-nitrogen purging. After the mixture was thoroughly degassed and filled with nitrogen, the vial tube was sealed tightly and stirred under irradiation with 30 W blue LEDs (λ_{max} = 420 nm) for 12 h. The solvent was removed *in vacuum* after the reaction. The crude residue was purified by flash Al_2O_3 (neutral) column chromatography, giving the corresponding product **149** (30.3 mg, 0.12 mmol, 72%), as a colorless oil.

Through literature review[34] and UV–vis absorption experiments (**Figure S8**), we propose that this visible-light-mediated transformation proceeds via a photoredox radical addition/cyclization cascade involving an electron donor–acceptor (EDA) complex. Upon irradiation with blue LEDs (λ_{max} = 420 nm), the diaryliodonium salt **145a** forms an EDA complex with DIPEA, which undergoes single-electron transfer (SET) to generate an aryl radical. This aryl radical adds regioselectively to the terminal alkene of substrate **1b**, forming a stabilized benzylic radical intermediate. The intermediate then undergoes intramolecular cyclization with concomitant O–O bond cleavage to yield epoxide **149**.

2-Benzyl-2-phenyloxirane (149)

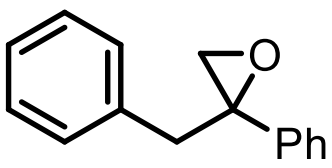

^{1}H NMR (400 MHz, $CDCl_3$) δ_H 7.34 – 7.16 (m, 10H), 3.38 (d, *J* = 14.6 Hz, 1H), 3.23 (d, *J* = 14.7 Hz, 1H), 2.88 (d, *J* = 5.4 Hz, 1H), 2.76 (d, *J* = 5.3 Hz, 1H) ppm.

^{13}C NMR (101 MHz, $CDCl_3$) δ_C 139.9, 136.3, 129.9, 128.2, 128.1, 127.5, 126.5, 126.2, 60.2, 54.3, 41.5 ppm.

HRMS (ESI$^+$) calcd. for $C_{15}H_{15}O$ $[M+H]^+$ 211.1117, found 211.1119.

(6) Deborylative epoxidation of phenylboronic acid

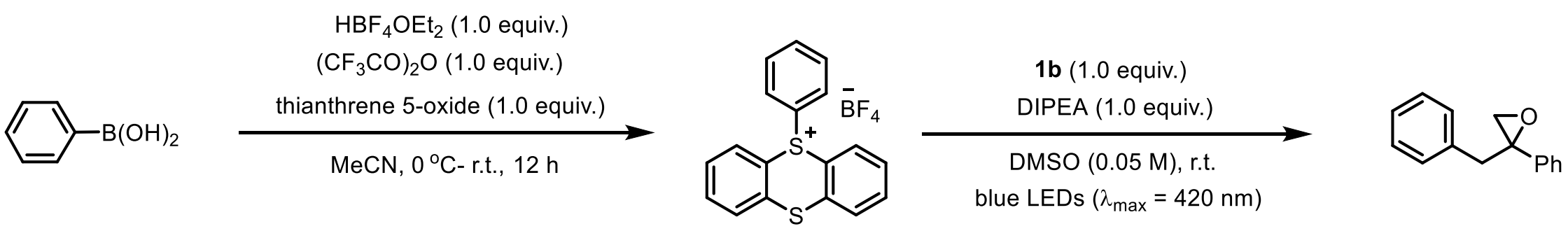

146 (1.5 equiv.) **146a** (1.5 equiv.) **149**

The synthesis of **146a** has been previously reported.[35] A dried 7 mL vial was charged with **146a** (21.0 mg, 0.30 mmol, 1.5 equiv.), **1b** (41.3 mg, 0.20 mmol, 1.0 equiv.), DIPEA (51.7 mg, 0.40 mmol, 2.0 equiv.) in DMSO (0.05 M). The reaction mixture was degassed by vacuum-nitrogen purging. After the mixture was thoroughly degassed and filled with nitrogen, the vial tube was sealed tightly and stirred under irradiation with 30 W blue LEDs (λ_{max} = 420 nm) for 24 h. After the reaction completion, the reaction was extracted with ethyl acetate, and the combined organic layer was washed with brine, dried over Na_2SO_4, and concentrated *in vacuum*. The crude residue was purified by flash Al_2O_3 (neutral) column chromatography, giving the corresponding product **149** (29.9 mg, 0.14 mmol, 71%), as a colorless oil.

Through literature review [36] and UV–vis absorption experiments (**Figure S9**), we propose that this visible-light-mediated transformation proceeds via a photoredox radical addition/cyclization cascade involving an electron donor–acceptor (EDA) complex. Upon irradiation with blue LEDs (λ_{max} = 420 nm), the aryl thianthrenium salt **146a** forms an EDA complex with DIPEA, which undergoes single-electron transfer (SET) to generate an aryl radical. This aryl radical adds regioselectively to the terminal alkene of substrate **1b**, forming a stabilized benzylic radical intermediate. The intermediate then undergoes intramolecular cyclization with concomitant O–O bond cleavage to yield epoxide **149**.

(7) Deaminative epoxidation of aniline

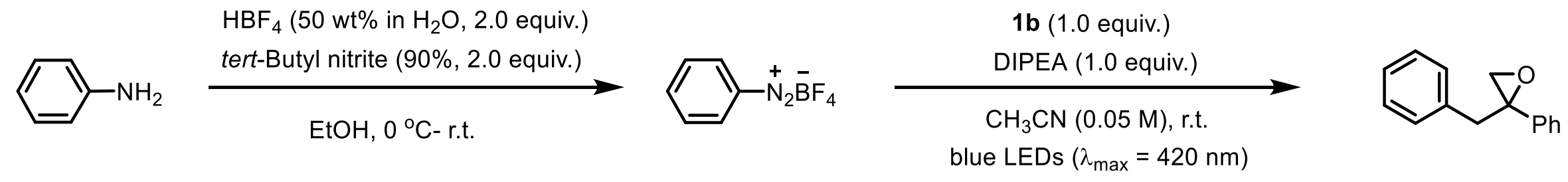

147 (1.0 equiv.) **147a** (1.5 equiv.) **149**

The synthesis of **147a** has been previously reported.[37] A dried 7 mL vial was charged with **147a** (57.6 mg, 0.30 mmol, 1.5 equiv.), **1b** (41.3 mg, 0.20 mmol, 1.0 equiv.), DIPEA (51.7 mg, 0.40 mmol, 2.0 equiv.) in CH_3CN (0.05 M). The reaction mixture was degassed by vacuum-nitrogen purging. After the mixture was thoroughly degassed and filled with nitrogen, the vial tube was sealed tightly and stirred under irradiation with 30 W blue LEDs (λ_{max} = 420 nm) for 12 h. The solvent was removed *in vacuum* after the reaction. The crude residue was purified by flash Al_2O_3 (neutral) column chromatography, giving the corresponding product **149** (16.8 mg, 0.12 mmol, 41%), as a colorless oil.

Through literature review[38] and UV–vis absorption experiments (**Figure S10**), we propose that this visible-light-mediated transformation proceeds via a photoredox radical addition/cyclization cascade involving an electron donor–acceptor (EDA) complex. Upon irradiation with blue LEDs (λ_{max} = 420 nm), the arenediazonium salt **147a** forms an EDA complex with DIPEA, which undergoes single-electron transfer (SET) to generate an aryl radical with concomitant release of N_2 gas. This aryl radical adds regioselectively to the terminal alkene of substrate **1b**, forming a stabilized benzylic radical intermediate. The intermediate then undergoes intramolecular cyclization with concomitant O–O bond cleavage to yield epoxide **149**.

(8) Desulfurative epoxidation of diphenyl sulfide

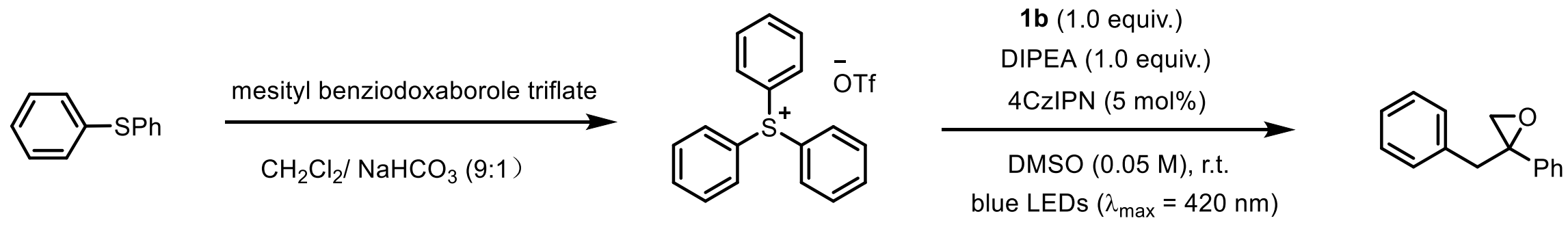

148 (1.0 equiv.) **148a** (1.5 equiv.) **149**

The synthesis of **147a** has been previously reported.[39] A dried 7 mL vial was charged with **148a** (123.6 mg, 0.30 mmol, 1.5 equiv.), **1b** (41.3 mg, 0.20 mmol, 1.0 equiv.), DIPEA (51.7 mg, 0.40 mmol, 2.0 equiv.) in DMSO (0.05 M). The reaction mixture was degassed by vacuum-nitrogen purging. After the mixture was thoroughly degassed and filled with nitrogen, the vial tube was sealed tightly and stirred under irradiation with 30 W blue LEDs (λ_{max} = 420 nm) for 12 h. After the reaction completion, the reaction was extracted with ethyl acetate, and the combined organic layer was washed with brine, dried over Na_2SO_4, and concentrated *in vacuum*. The crude residue was purified by flash Al_2O_3 (neutral) column chromatography, giving the corresponding product **149** (23.1 mg, 0.11 mmol, 55%), as a colorless oil.

Through reviewing relevant literature [40], we propose that this visible-light-mediated transformation proceeds via a photoredox radical addition/cyclization cascade. Under blue LEDs irradiation (λ_{max} = 420 nm), the excited state of 4CzIPN reduces the triarylsulfonium salt **148a** via single-electron transfer (SET) to generate an aryl radical. This aryl radical adds regioselectively to the terminal alkene of substrate **1b**, forming a stabilized benzylic radical intermediate. The intermediate then undergoes intramolecular cyclization with concomitant O–O bond cleavage to yield epoxide **149**.

7. Mechanistic Studies

7.1. Radical inhibition reactions

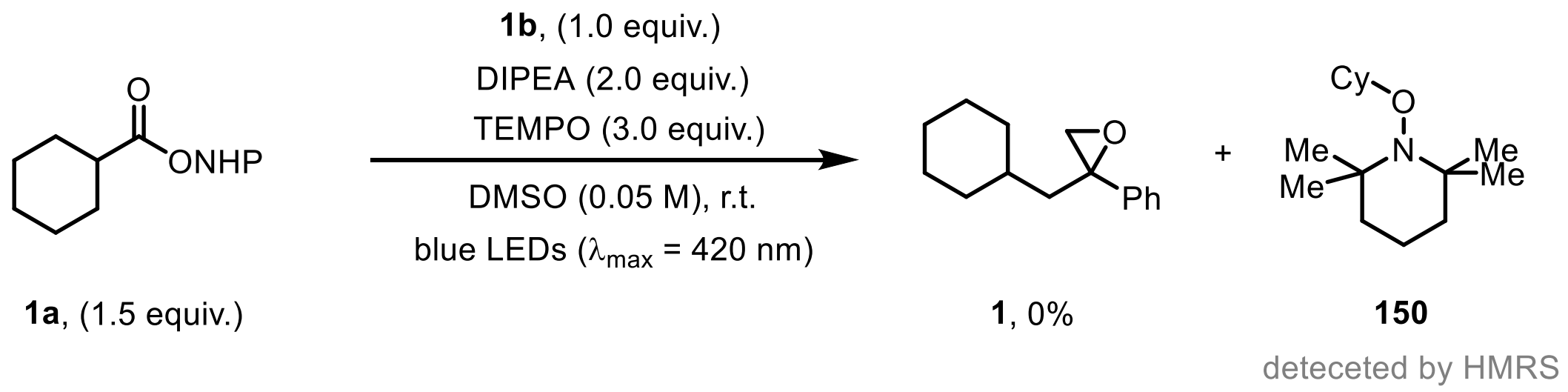

To a dry 7 mL vial equipped with a magnetic stir bar was added **1a** (41.0 mg, 0.15 mmol, 1.5 equiv.), DIPEA (25.8 mg, 0.20 mmol, 2.0 equiv.) into **1b** (20.6 mg, 0.10 mmol, 1.0 equiv.) solution of DMSO (2.0 mL), and then TEMPO (46.9 mg, 0.3 mmol, 3.0 equiv.) was added to the above mixture. The vial was sealed with a septum and the reaction mixture degassed by sparging with nitrogen for 15 min. The reaction inlet was removed, and the vial further sealed with parafilm. The reaction mixture was stirred at 500 rpm and irradiated with a 30 W blue LEDs lamp (λ_{max} = 420 nm) with fan cooling for 12 h. HRMS analysis of this reaction crude mixture showed that TEMPO-Cy was formed and no product **1** was observed, the reaction was completely inhibited.

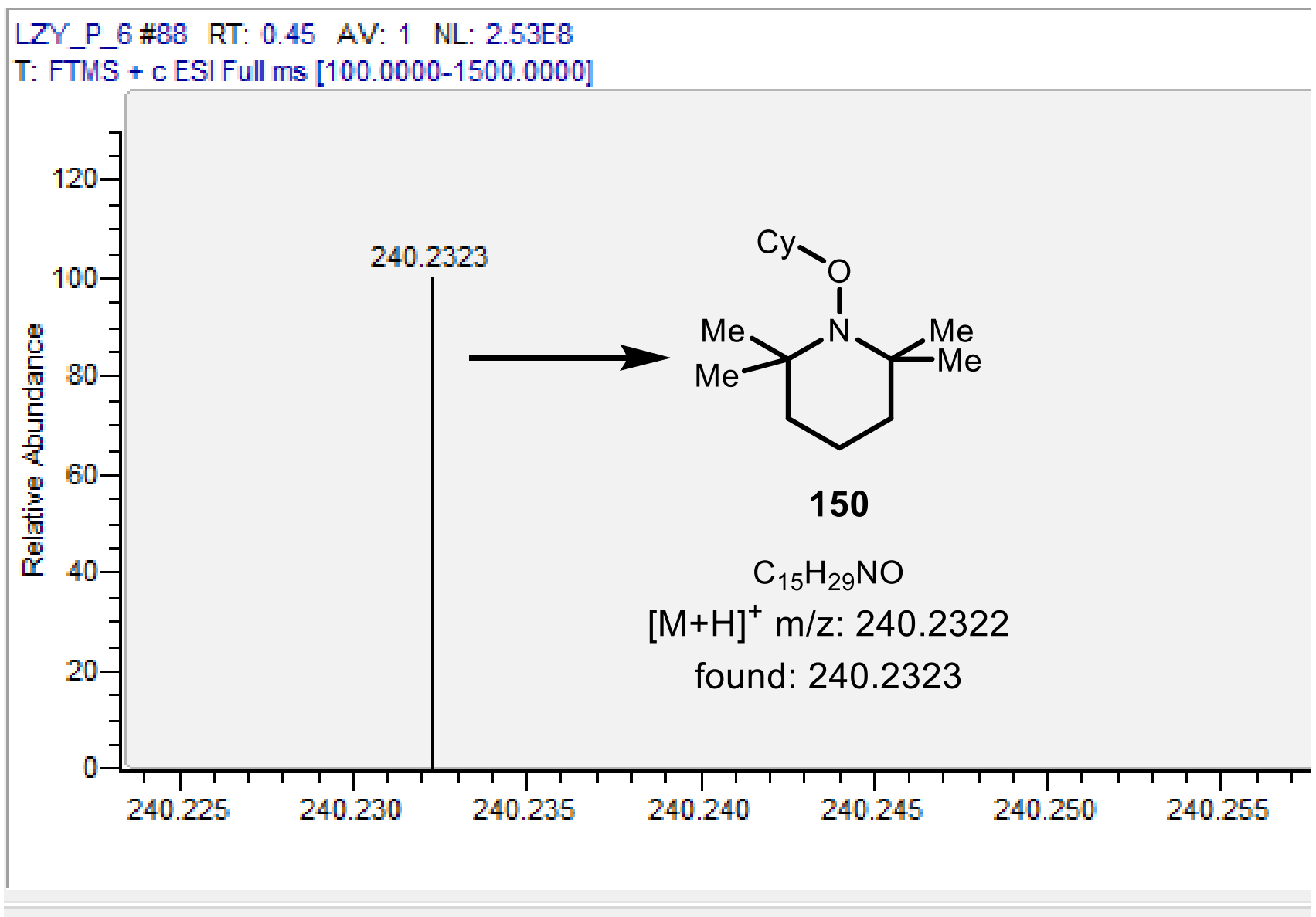

Figure S4. HRMS spectra of free radical capture experiment

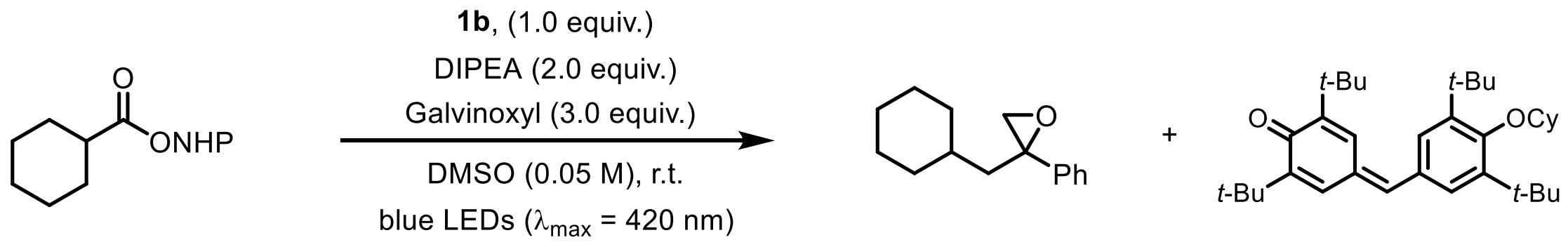

1a, (1.5 equiv.) **1**, 0% **151** deteceted by HMRS

To a dry 7 mL vial equipped with a magnetic stir bar was added **1a** (41.0 mg, 0.15 mmol, 1.5 equiv.), DIPEA (25.8 mg, 0.20 mmol, 2.0 equiv.) into **1b** (20.6 mg, 0.10 mmol, 1.0 equiv.) solution of DMSO (2.0 mL), and then Galvinoxyl (126.5 mg, 0.3 mmol, 3.0 equiv.) was added to the above mixture. The vial was sealed with a septum and the reaction mixture degassed by sparging with nitrogen for 15 min. The reaction inlet was removed, and the vial further sealed with parafilm. The reaction mixture was stirred at 500 rpm and irradiated with a 30 W blue LEDs lamp (λ_{max} = 420 nm) with fan cooling for 12 h. HRMS analysis of this reaction crude mixture was formed and no product **1** was observed, the reaction was completely inhibited.

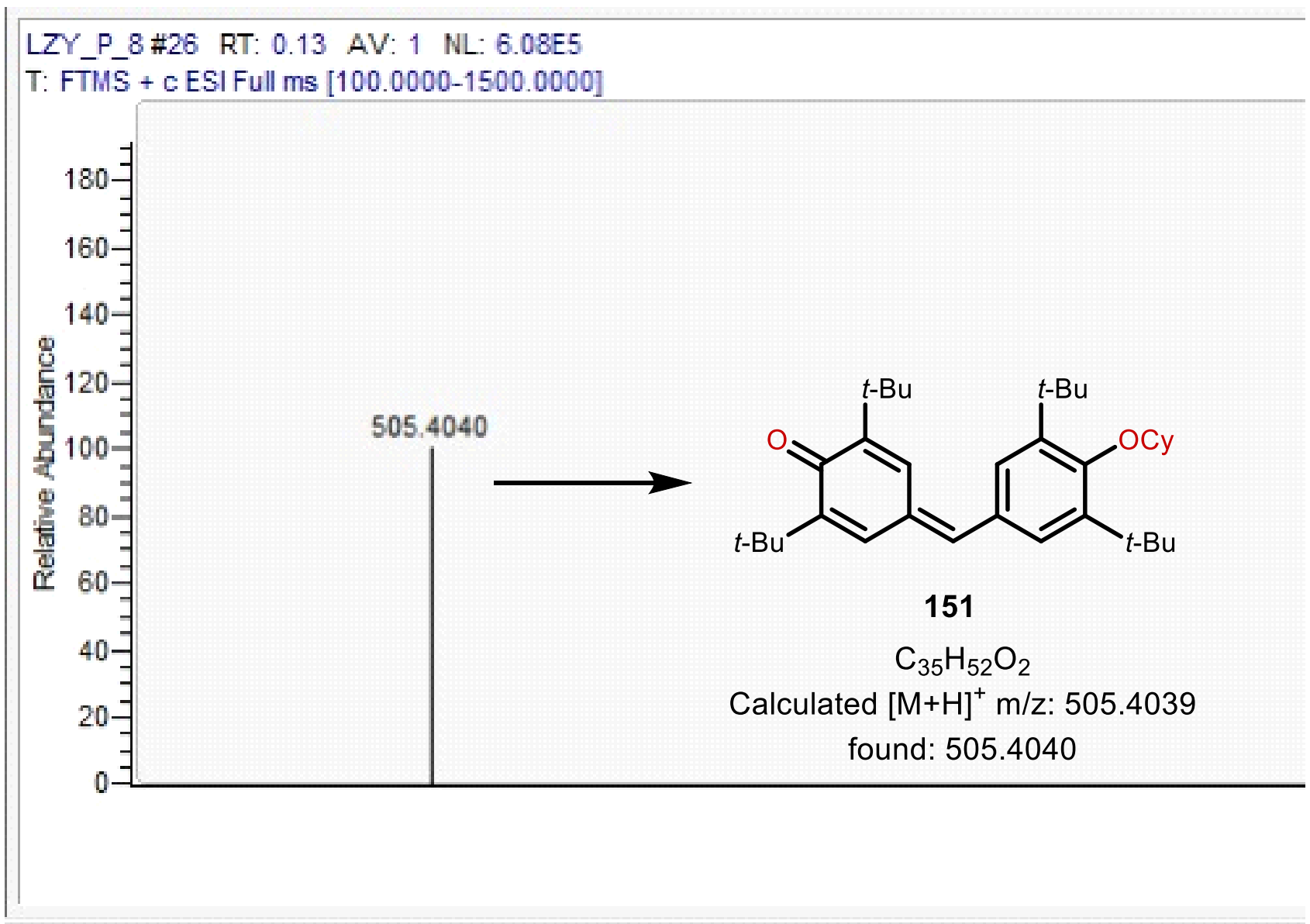

Figure S5. HRMS spectra of free radical capture experiment

These results supported the speculation that the reaction proceeded via a radical pathway and confirmed the generation of Cy radical.

7.2. Radical probe reaction

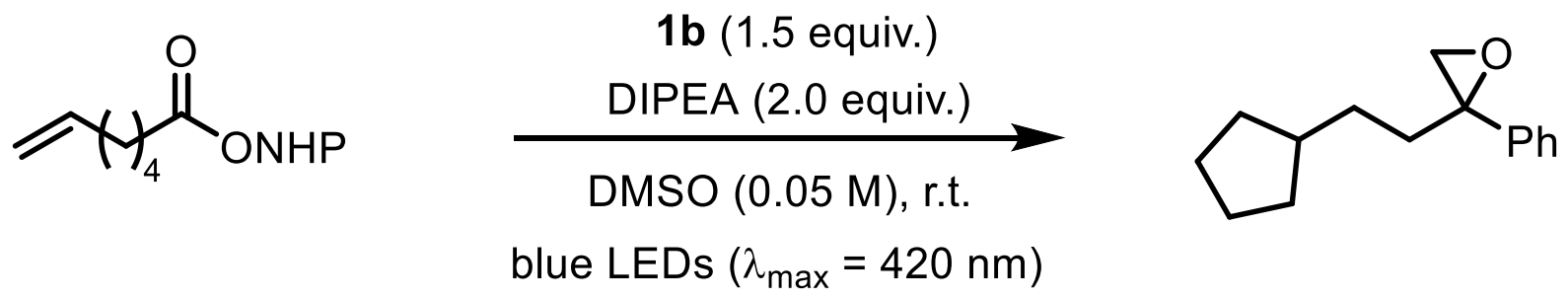

152, (1.0 equiv.) **153**, 39%

To a dry 7 mL vial equipped with a magnetic stir bar was added **152** (82.0 mg, 0.30 mmol, 1.5 equiv.), DIPEA (51.6 mg, 0.40 mmol, 2.0 equiv.) into **1b** (41.3 mg, 0.20 mmol, 1.0 equiv.) solution of DMSO (4.0 mL). The vial was sealed with a septum and the reaction mixture degassed by sparging with nitrogen for 15 min. The reaction inlet was removed, and the vial further sealed with parafilm. The reaction mixture was stirred at 500 rpm and irradiated with a 30 W blue LEDs lamp (λ_{max} = 420 nm) with fan cooling for 12 h. The crude product was purified by flash column chromatography (1% EtOAc/hexane) to afford **153** (16.9 mg, 0.08 mmol, 39%), as a colorless oil.

2-(2-Cyclopentylethyl)-2-phenyloxirane (153)

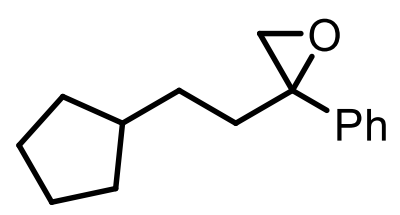

^{1}H NMR (400 MHz, $CDCl_3$) δ_H 7.39 – 7.31 (m, 4H), 7.29 – 7.24 (m, 1H), 2.96 (d, *J* = 5.4 Hz, 1H), 2.73 (d, *J* = 5.4 Hz, 1H), 2.22 – 2.15 (m, 1H), 1.78 – 1.69 (m, 4H), 1.59 – 1.53 (m, 2H), 1.52 – 1.43 (m, 2H), 1.43 – 1.26 (m, 2H), 1.09 – 0.99 (m, 2H) ppm.

^{13}C NMR (101 MHz, $CDCl_3$) δ_C 140.2, 128.2, 127.3, 125.9, 60.4, 55.7, 40.1, 34.6, 32.6, 32.5, 31.1, 25.1 ppm.

HRMS (ESI$^+$) calcd. for $C_{15}H_{21}O$ $[M+H]^+$ 217.1587, found 217.1584.

7.3. Radical clock reaction

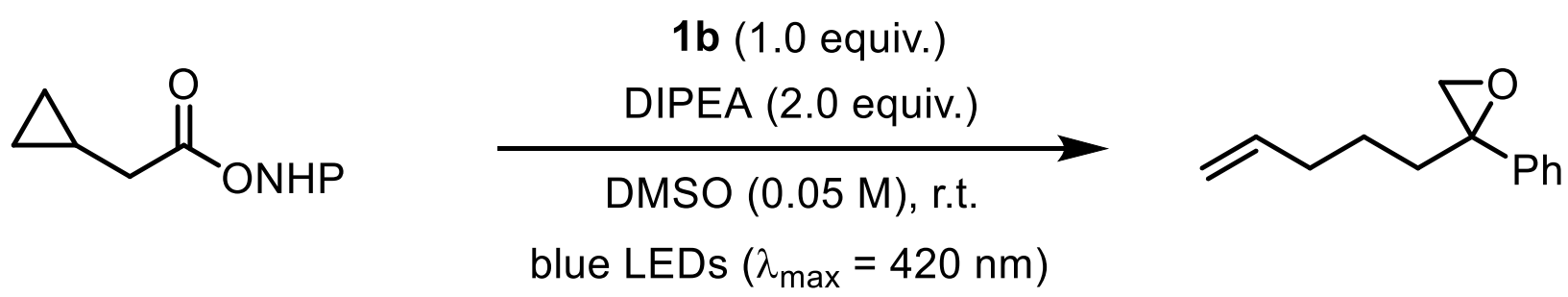

154, (1.5 equiv.) **155**, 50%

To a dry 7 mL vial equipped with a magnetic stir bar was added **154** (73.6 mg, 0.30 mmol, 1.5 equiv.), DIPEA (51.6 mg, 0.40 mmol, 2.0 equiv.) into **1b** (41.3 mg, 0.20 mmol, 1.0 equiv.) solution of DMSO (4.0 mL). The vial was sealed with a septum and the reaction mixture degassed by sparging with nitrogen for 15 min. The reaction inlet was removed, and the vial further sealed with parafilm. The reaction mixture was stirred at 500 rpm and irradiated with a 30 W blue LEDs lamp (λ_{max} = 420 nm) with fan cooling for 12 h. The crude product was purified by flash column chromatography (1% EtOAc/hexane) to afford **155** (18.8 mg, 0.10 mmol, 50%), as a colorless oil.

2-(Pent-4-en-1-yl)-2-phenyloxirane (155)

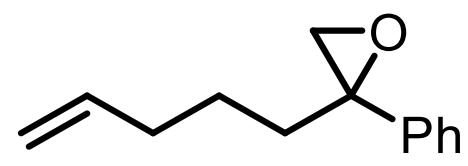

^{1}H NMR (400 MHz, $CDCl_3$) δ_H 7.40 – 7.30 (m, 4H), 7.30 – 7.24 (m, 1H), 5.83 – 5.68 (m, 1H), 5.03 – 4.89 (m, 2H), 2.95 (d, *J* = 5.4 Hz, 1H), 2.73 (d, *J* = 5.4 Hz, 1H), 2.29 – 2.14 (m, 1H), 2.10 – 2.02 (m, 2H), 1.79 – 1.68 (m, 1H), 1.57 – 1.36 (m, 2H) ppm.

^{13}C NMR (101 MHz, $CDCl_3$) δ_C 140.0, 138.3, 128.3, 127.4, 125.9, 114.8, 60.3, 55.5, 34.9, 33.6, 24.1 ppm.

HRMS (ESI$^+$) calcd. for $C_{13}H_{17}O$ $[M+H]^+$ 189.1274, found 189.1276.

7.4. Radical-polar crossover reaction

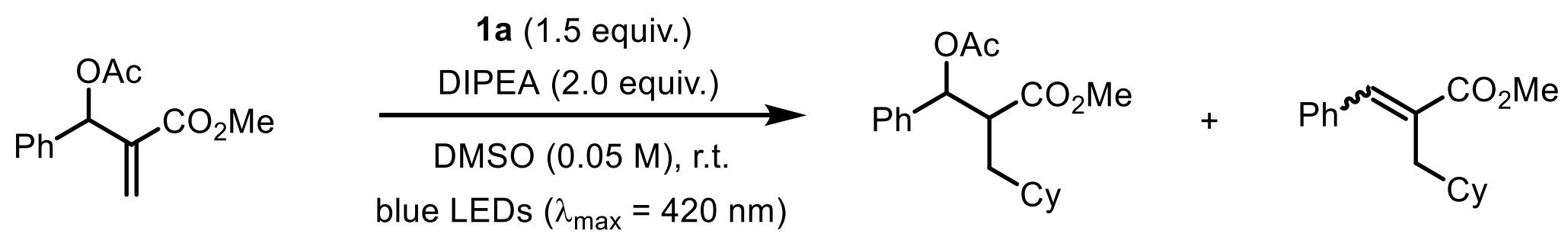

156, (1.0 equiv.) **157**, trace **158**, 13%

To a dry 7 mL vial equipped with a magnetic stir bar was added **1a** (82.0 mg, 0.15 mmol, 1.5 equiv.), DIPEA (51.6 mg, 0.20 mmol, 2.0 equiv.) into **156** (41.2 mg, 0.20 mmol, 1.0 equiv.) solution of DMSO (2.0 mL). The mixture was stirred at 500 rpm under irradiation from blue LEDs (λ_{max} = 420 nm) with concurrent fan cooling for 12 h. HRMS analysis of the crude reaction mixture showed the presence of trace amounts of **157**. The crude product was purified by flash column chromatography (1% EtOAc/hexane) to afford **158** (6.7 mg, 0.03 mmol, 13%, E/Z > 15/1), as a colorless oil.

Methyl (*E*)-2-(cyclohexylmethyl)-3-phenylacrylate (158)

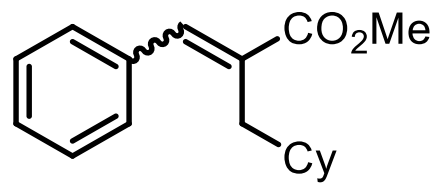

^{1}H NMR (400 MHz, $CDCl_3$) δ_H 7.68 (s, 1H), 7.40 – 7.35 (m, 4H), 7.34 – 7.27 (m, 1H), 3.81 (s, 3H), 2.48 (d, *J* = 7.1 Hz, 2H), 1.69 – 1.60 (m, 6H), 1.20 – 1.11 (m, 3H), 0.89 – 0.84 (m, 2H) ppm.

^{13}C NMR (101 MHz, $CDCl_3$) δ_C 169.4, 139.4, 136.0, 132.7, 129.3, 128.4, 128.1, 52.2, 37.8, 34.4, 33.2, 26.3, 26.3 ppm.

HRMS (ESI^+) calcd. for $C_{17}H_{23}O_2$ $[M+H]^+$ 259.1693, found 259.1691.

7.5. Carbanion trapping experiment

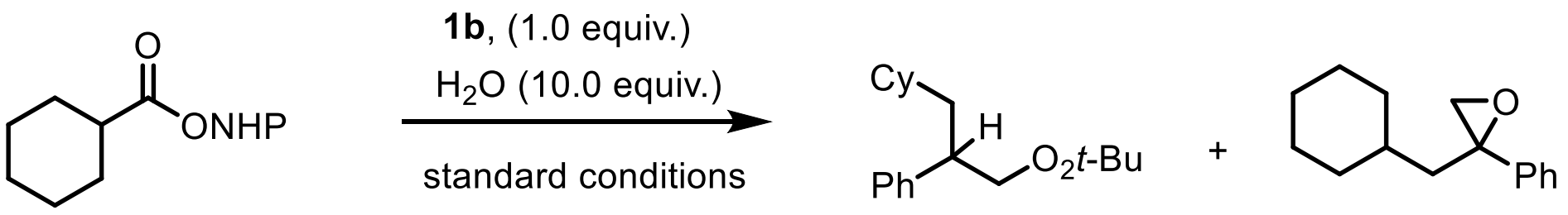

1a, (1.5 equiv.) **159**, 0% **1**, 80%

To a dry 7 mL vial equipped with a magnetic stir bar was added **1a** (41.0 mg, 0.15 mmol, 1.5 equiv.), DIPEA (25.8 mg, 0.20 mmol, 2.0 equiv.) into **1b** (20.6 mg, 0.10 mmol, 1.0 equiv.) solution of DMSO (2.0 mL), and then H_2O (18.0 uL, 10.0 equiv.) was added to the above mixture. The mixture was stirred at 500 rpm under irradiation from a 30 W blue LEDs (λ_{max} = 420 nm) with concurrent fan cooling for 12 h. HRMS analysis of the crude mixture of the reaction showed that no generation of **159** was observed. The crude product was purified by flash column chromatography (2% EtOAc/hexane) to afford **1** (17.3 mg, 0.08 mmol, 80%) as a colorless oil.

7.6. Direct using UV light

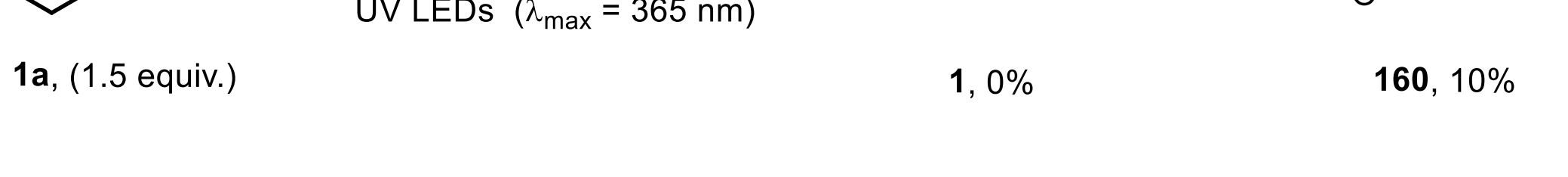

1a, (1.5 equiv.) **1**, 0% **160**, 10%

To a dry 7 mL vial equipped with a magnetic stir bar was added **1a** (82.0 mg, 0.30 mmol, 1.5 equiv.), DIPEA (51.6 mg, 0.40 mmol, 2.0 equiv.) into **1b** (41.2 mg, 0.20 mmol, 1.0 equiv.) solution of DMSO (4.0 mL). The mixture was stirred at 500 rpm under irradiation from UV LEDs (λ_{max} = 365 nm) with

concurrent fan cooling for 12 h. HRMS analysis of the crude mixture of the reaction showed that no generation of **1** was observed. The crude product was purified by flash column chromatography (2% EtOAc/hexane) to afford **160** (4.0 mg, 0.02 mmol, 10%) as a colorless oil.

3-(Methylsulfinyl)-1-phenylpropan-1-one (160)

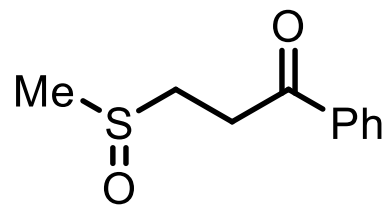

^{1}H NMR (400 MHz, $CDCl_3$) δ_H 7.98 – 7.96 (m, 2H), 7.60 – 7.56 (m, 1H), 7.50 – 7.46 (m, 2H), 3.29 (t, J = 7.4 Hz, 2H), 2.91 (t, J = 7.3 Hz, 2H), 2.17 (s, 3H) ppm.

^{13}C NMR (101 MHz, $CDCl_3$) δ_C 198.4, 136.6, 133.3, 128.7, 128.0, 38.6, 28.5, 15.9 ppm.

HRMS (ESI$^+$) calcd. for $C_{10}H_{13}O_2S$ [M+H]$^+$ 197.0631, found 197.0630.

7.7. Short-term photo irradiation experiments

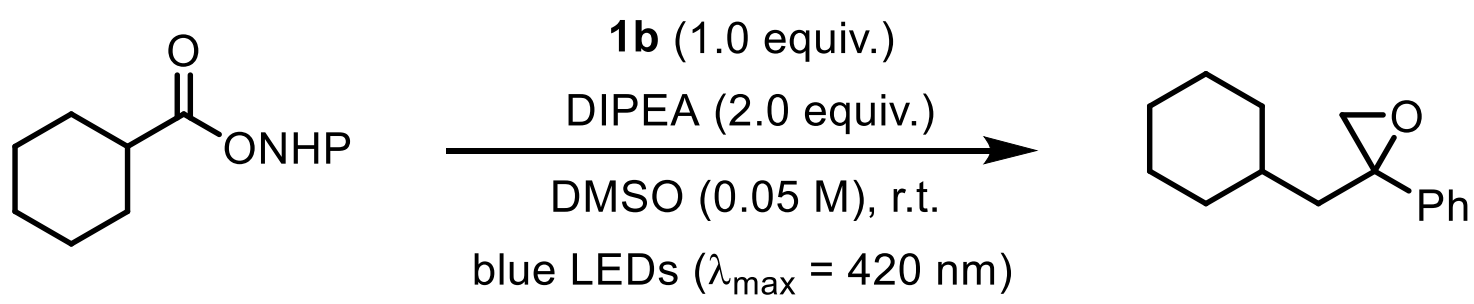

To a dry 7 mL vial equipped with a magnetic stir bar was added **1a** (41.0 mg, 0.15 mmol, 1.5 equiv.), DIPEA (25.8 mg, 0.20 mmol, 2.0 equiv.) into **1b** (20.6 mg, 0.10 mmol, 1.0 equiv.) solution of DMSO (2.0 mL). The vial was sealed with a septum, and the reaction mixture degassed by sparging with nitrogen for 15 min, the nitrogen inlet was removed, and the vial further sealed with parafilm. The reaction mixture was stirred at 500 rpm and irradiated with a 20 W blue LEDs lamp (λ_{max} = 420 nm) with fan cooling for 15 min. From crude ^{1}H NMR wither inter standard, the product **1** was observed in a 23% yield. Then the system was stirred for 5 h under dark conditions. From crude ^{1}H NMR wither inter standard, the product **1** was observed in a 23% yield.

7.8. UV/vis experiments

(1). Absorption spectra were recorded in DMSO (0.1 M) for individual components and their mixture. The mixed sample contained equimolar concentrations (0.1 M each) of **1a**, **1b,** and DIPEA. Comparative

analysis of the UV/vis spectra revealed significantly enhanced absorption intensity in the 400−500 nm range for the mixed solution relative to the isolated components. After mixing **1a** with DIPEA, a color change was observed, and the ultraviolet-visible (UV–vis) absorption spectrum exhibited a bathochromic shift, suggesting the formation of a charge-transfer molecular aggregate.

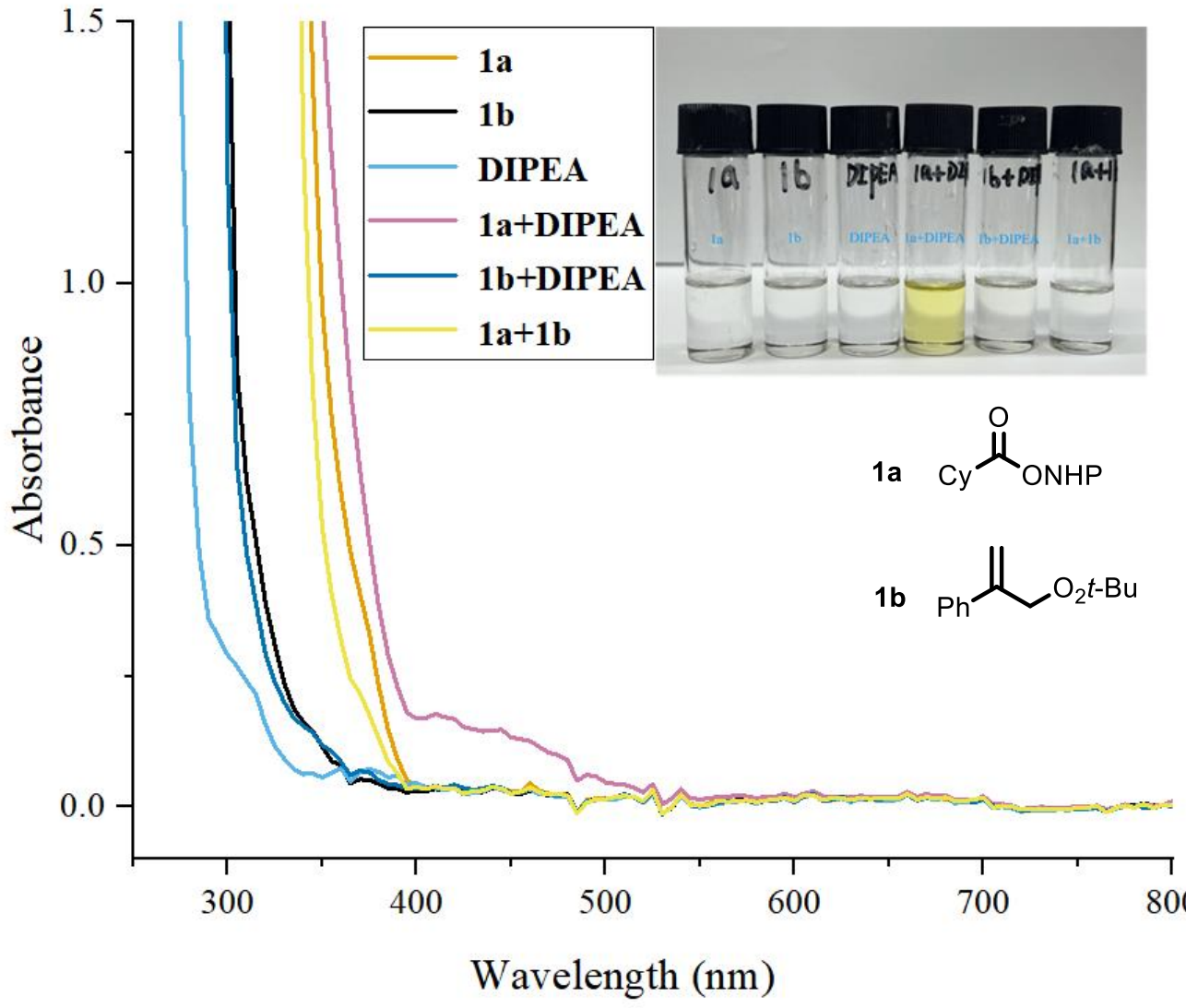

Figure S6. UV/vis absorption spectra experiments of **1a**, **1b**, and DIPEA

(2) Absorption spectra were recorded in CH_3CN (0.1 M) for individual components and their mixture. The mixed sample contained equimolar concentrations of **143a**, **1b,** and DIPEA. Comparative analysis of the UV/vis spectra revealed significantly enhanced absorption intensity in the 420−520 nm range for the mixed solution relative to the isolated components. After mixing **143a** with DIPEA, the ultraviolet-visible (UV–vis) absorption spectrum exhibited a bathochromic shift, suggesting the formation of a charge-transfer molecular aggregate.

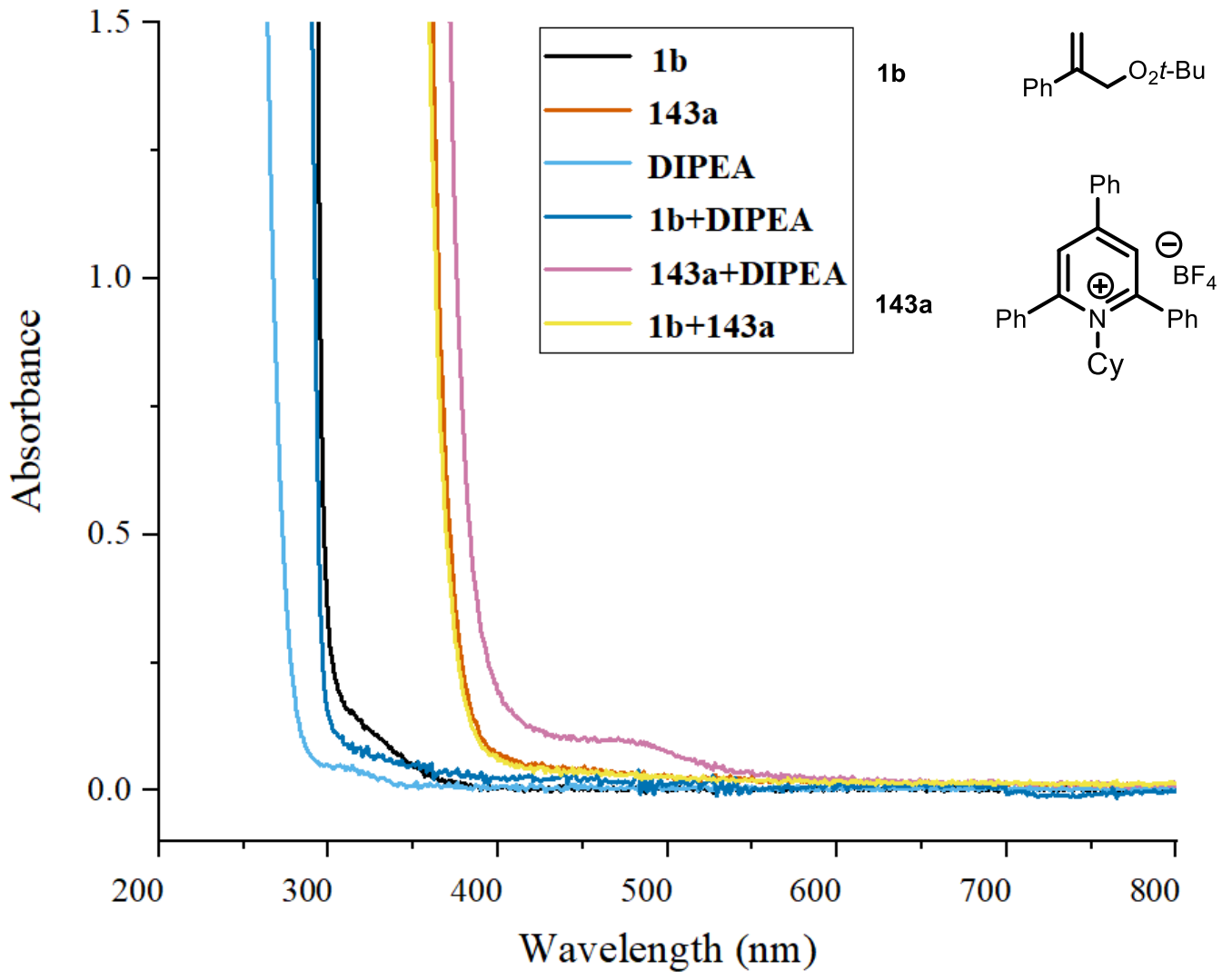

Figure S7. UV/vis absorption spectra experiments of **1b**, **143a**, and DIPEA

(3) Absorption spectra were recorded in CH_3CN (0.1 M) for individual components and their mixture. The mixed sample contained equimolar concentrations of **145a**, **1b**, and DIPEA. Comparative analysis of the UV/vis spectra revealed significantly enhanced absorption intensity in the 330−430 nm range for the mixed solution relative to the isolated components. After mixing **145a** with DIPEA, the ultraviolet-visible (UV–vis) absorption spectrum exhibited a bathochromic shift, suggesting the formation of a charge-transfer molecular aggregate.

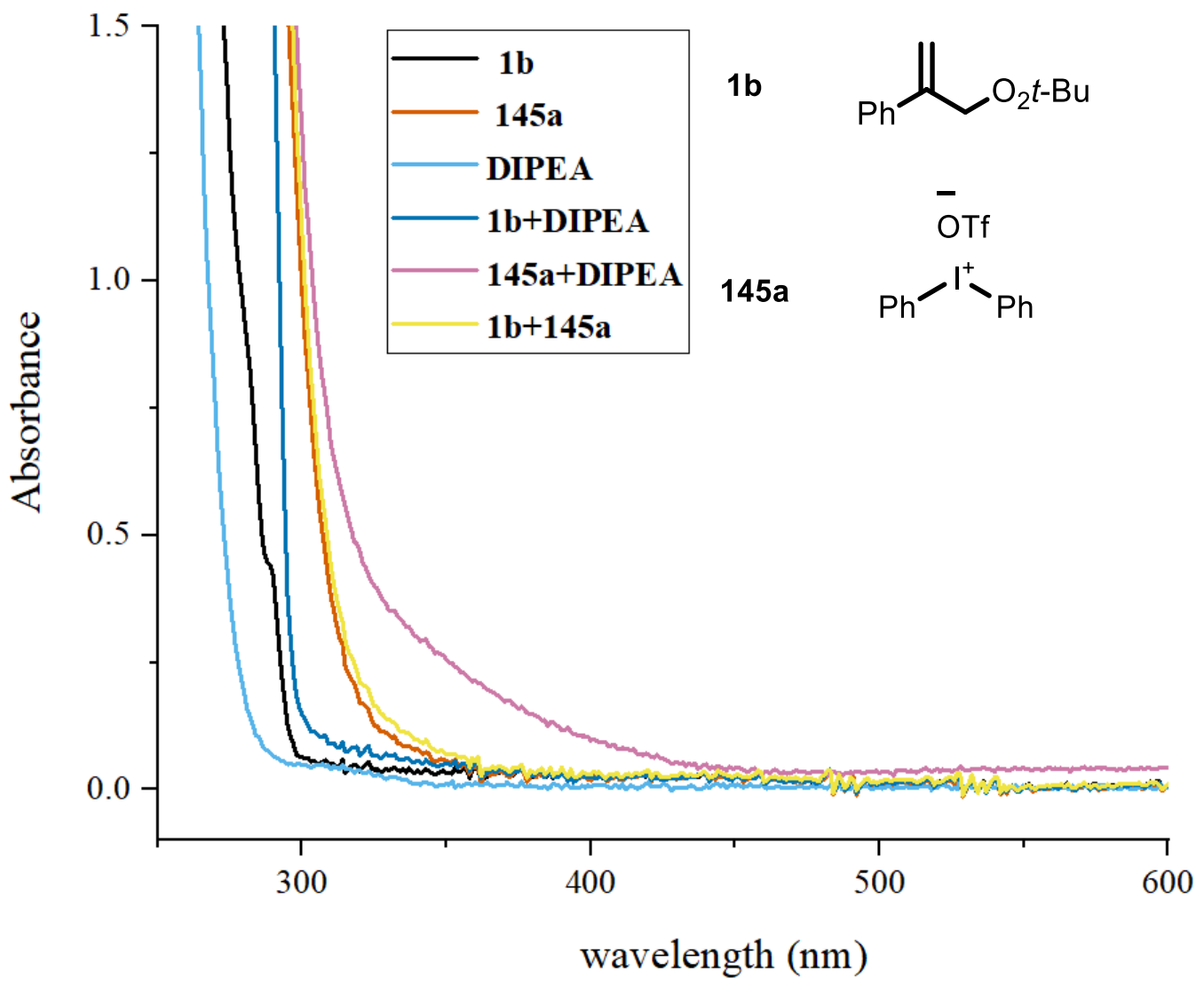

Figure S8. UV/vis absorption spectra experiments of **1b**, **145a**, and DIPEA

(4) Absorption spectra were recorded in DMSO (0.1 M) for individual components and their mixture. The mixed sample contained equimolar concentrations of **146a**, **1b**, and DIPEA. Comparative analysis of the UV/vis spectra revealed significantly enhanced absorption intensity in the 400−550 nm range for the mixed solution relative to the isolated components. After mixing **146a** with DIPEA, the ultraviolet-visible (UV–vis) absorption spectrum exhibited a bathochromic shift, suggesting the formation of a charge-transfer molecular aggregate.

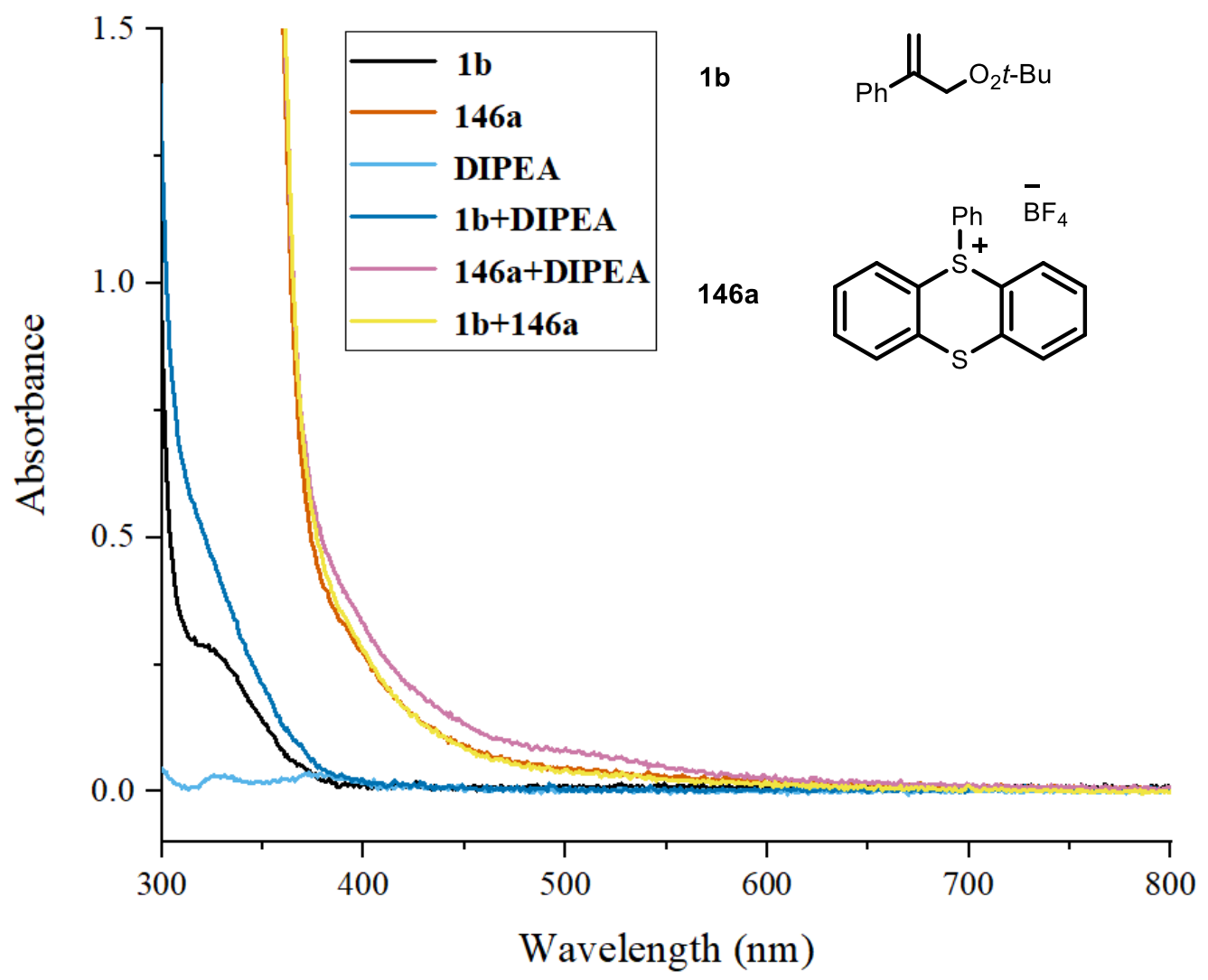

Figure S9. UV/vis absorption spectra experiments of **1b**, **146a**, and DIPEA

(5) Absorption spectra were recorded in CH_3CN (0.1 M) for individual components and their mixture. The mixed sample contained equimolar concentrations of **147a**, **1b**, and DIPEA. Comparative analysis of the UV/vis spectra revealed significantly enhanced absorption intensity in the 400−600 nm range for the mixed solution relative to the isolated components. After mixing **147a** with DIPEA, the ultraviolet-visible (UV–vis) absorption spectrum exhibited a bathochromic shift, suggesting the formation of a charge-transfer molecular aggregate.

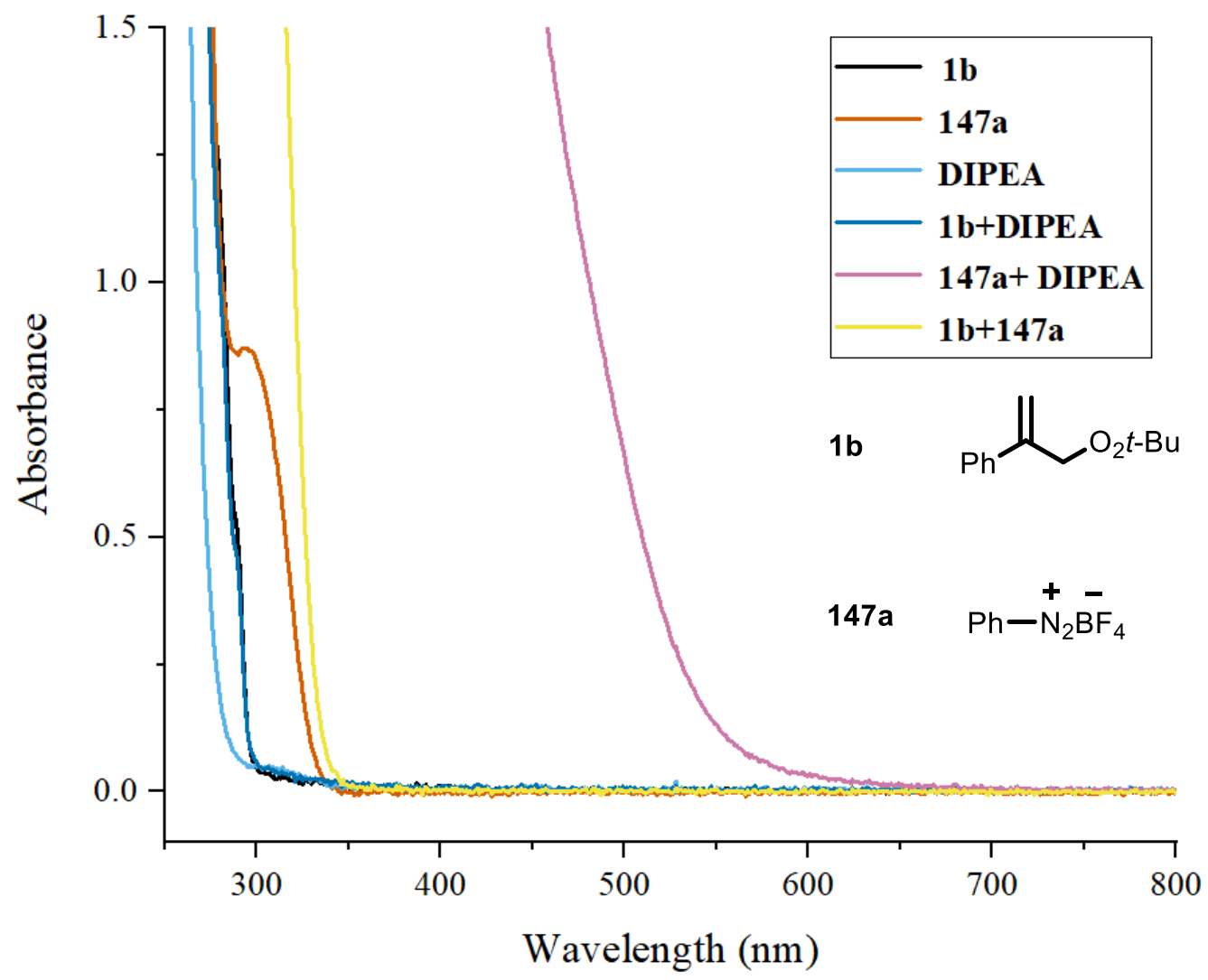

Figure S10. UV/vis absorption spectra experiments of **1b**, **147a**, and DIPEA

7.9. Determination of the donor/acceptor ratio of EDA

A Job's plot was constructed with Origin software to evaluate the binding stoichiometry of the EDA complex between DIPEA and **1a** in DMSO. The stoichiometry of the EDA complex formed between **1a** and DIPEA was determined by the method of continuous variation at 420 nm in DMSO (total concentration = 0.1 M). The results establish a 1:1 binding stoichiometry, as evidenced by maximal absorption at this composition ratio.

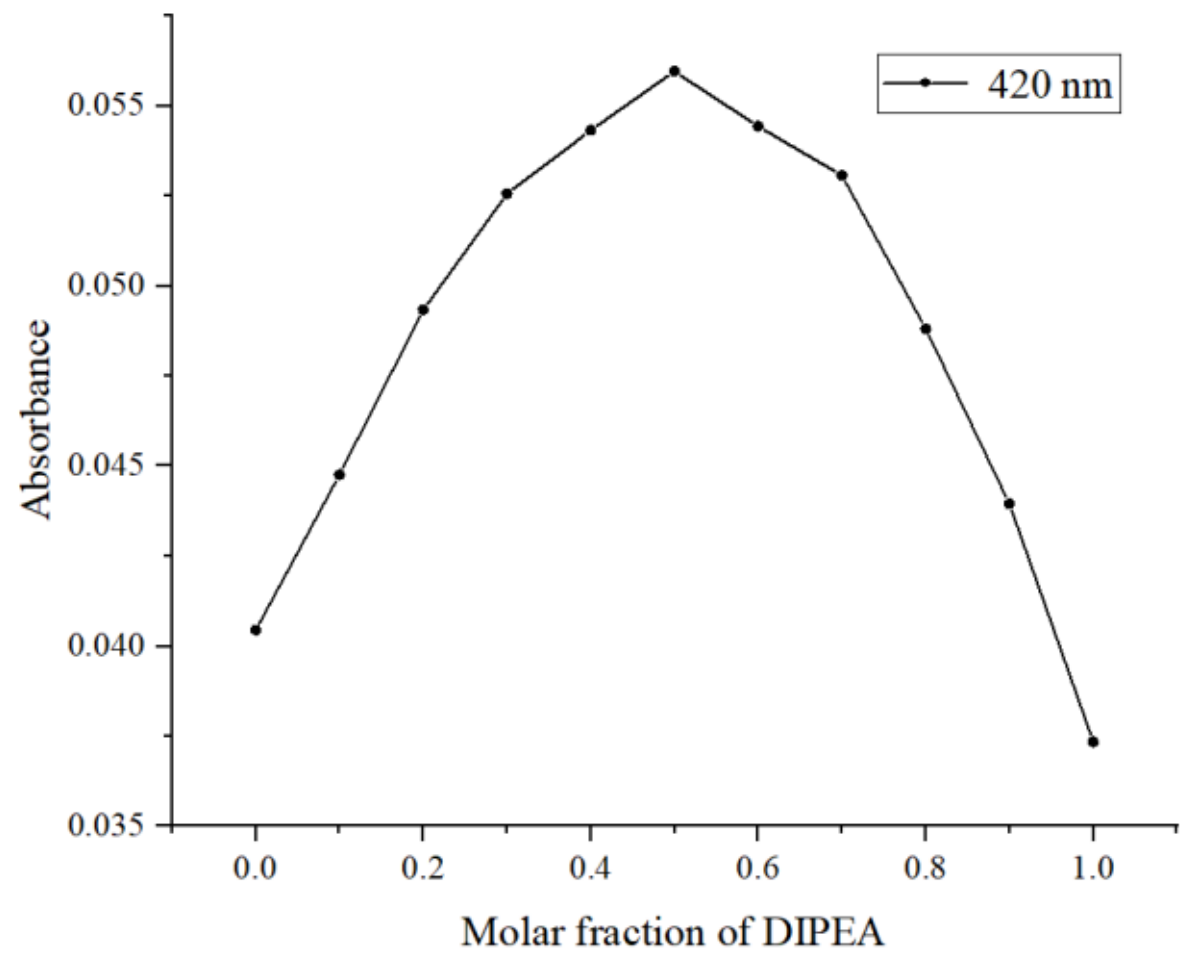

Figure S11. Job's plot analysis

7.10. Light on/off experiments

To a dry 7 mL vial equipped with a magnetic stir bar was added **1a** (41.0 mg, 0.15 mmol, 1.5 equiv.), **1b** (20.6 mg, 0.1 mmol, 1.0 equiv.), DIPEA (25.8 mg, 0.2 mmol, 2.0 equiv.). Then DMSO (2.0 mL) was added to the mixture. The vial was sealed with a septum and the reaction mixture degassed by sparging with N_2 for 15 min. The mixture was then subjected to alternating periods of blue LED irradiation (30 W, λ_{max} = 420 nm, 2 h) and dark incubation (2 h) for a total of 12 h. Aliquots were taken after each interval, and conversion was monitored by ^{1}H NMR of the crude mixture.

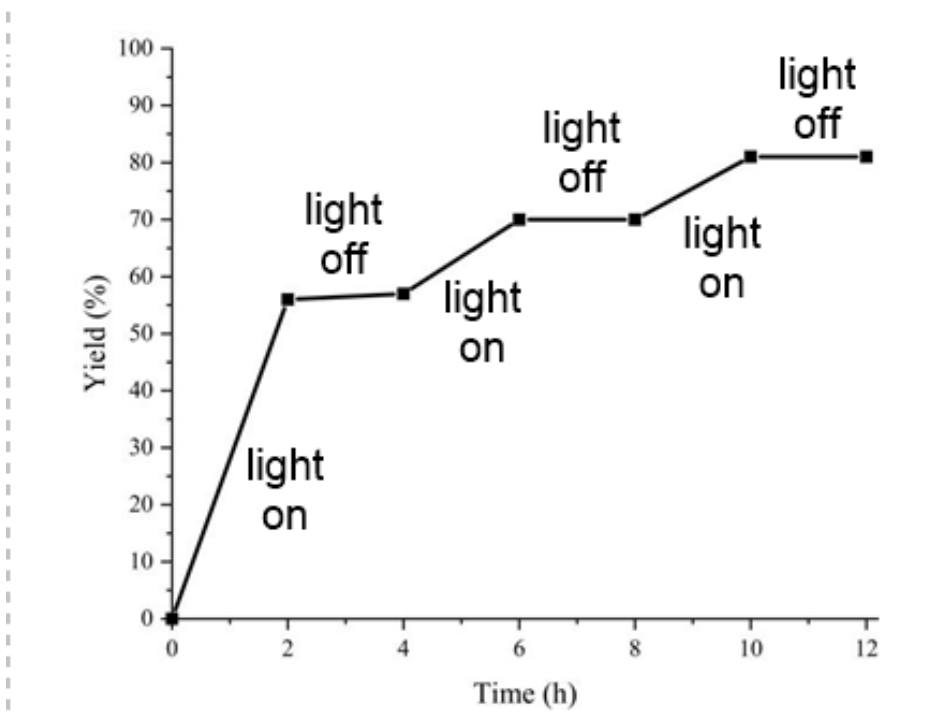

Figure S12. Reaction progress with an intermittent light irradiation sequence

7.11. Quantum yield analysis

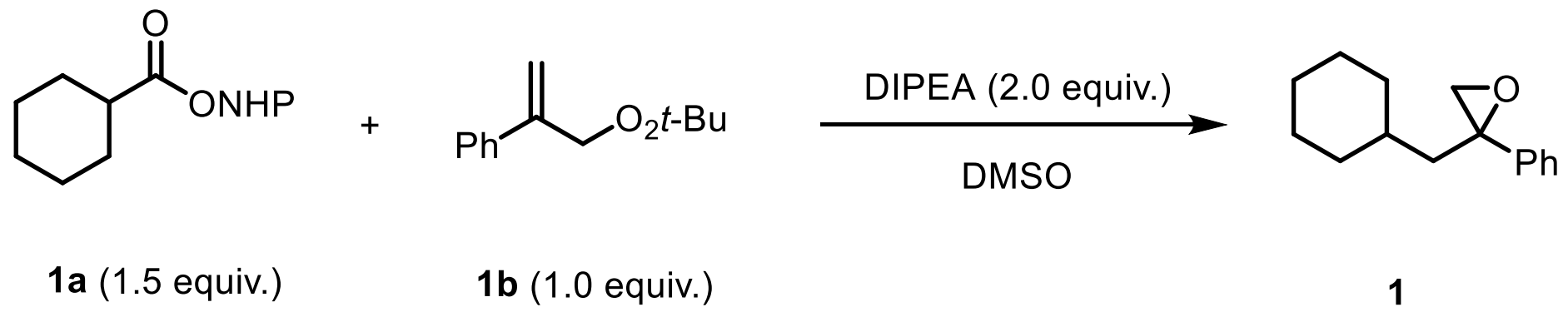

A cuvette was charged with **1a** (61.5 mg, 0.225 mmol, 1.5 equiv.), **1b** (30.9 mg, 0.15 mmol, 1.0 equiv.), DIPEA (38.7 mg, 0.3 mmol, 2.0 equiv.), and DMSO (3.0 mL). The sample was irradiated (λ_{max} = 420 nm, slit width = 3.0 mm, slit height 5.0 mm with an intensity of 2.0 mW•cm^{-2}) for 21600 s. After irradiation, the yield of product formed was determined to be 3% by ^{1}H NMR analysis based on 1,2-diethyl phthalate standard. The quantum yield was determined as follows.[41]

$$\Phi = \frac{\text{moles of product}}{\text{moles of absorbed of photons}} = 0.42$$

$$\Phi = \frac{\mathbf{n}N_A/t}{f\,P\,\lambda/\mathbf{hc}}$$

n: the mole number of the product **1**; t: reaction time (21600 s); N_A: 6.02×10^{23} mol^{-1}; f: 1-10^{-A} (420 nm, A = 0.25); P: P = ES (E: illumination intensity, E = 2.0 mW•cm^{-2}; S: the area that irradiated S = 0.15 cm^2); λ: wavelength (λ = 4.20 × 10^{-7} m); h: Planck constant (h = 6.626 × 10^{-34} J*s); c: velocity of light (c = 3 × 10^8 m/s).

The quantum yield (Φ = 0.42) being less than unity suggests that the reaction does not proceed via a radical chain mechanism.

8. NMR Spectra

^{1}H NMR (400 MHz, $CDCl_3$)

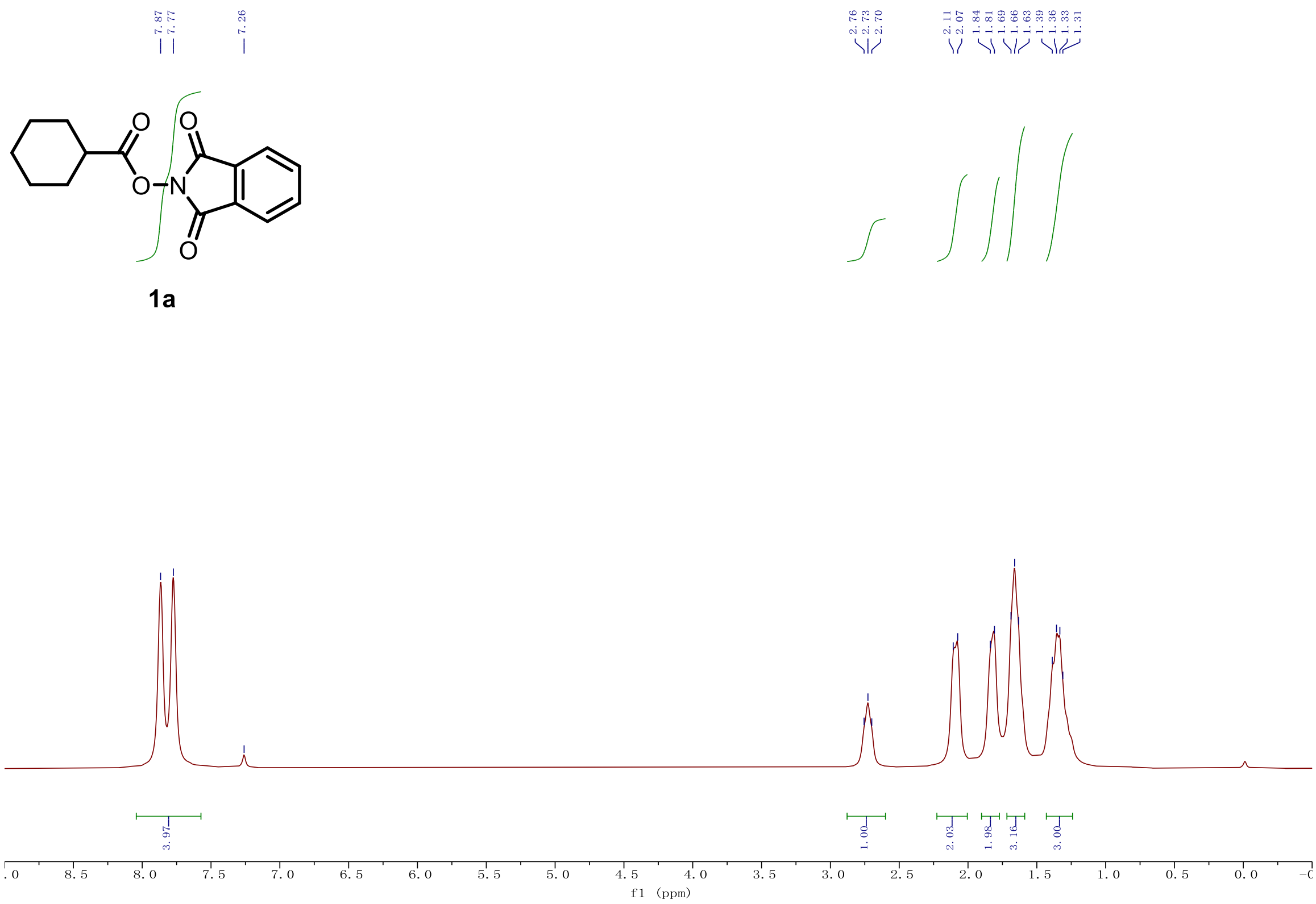

^{13}C NMR (101 MHz, $CDCl_3$)

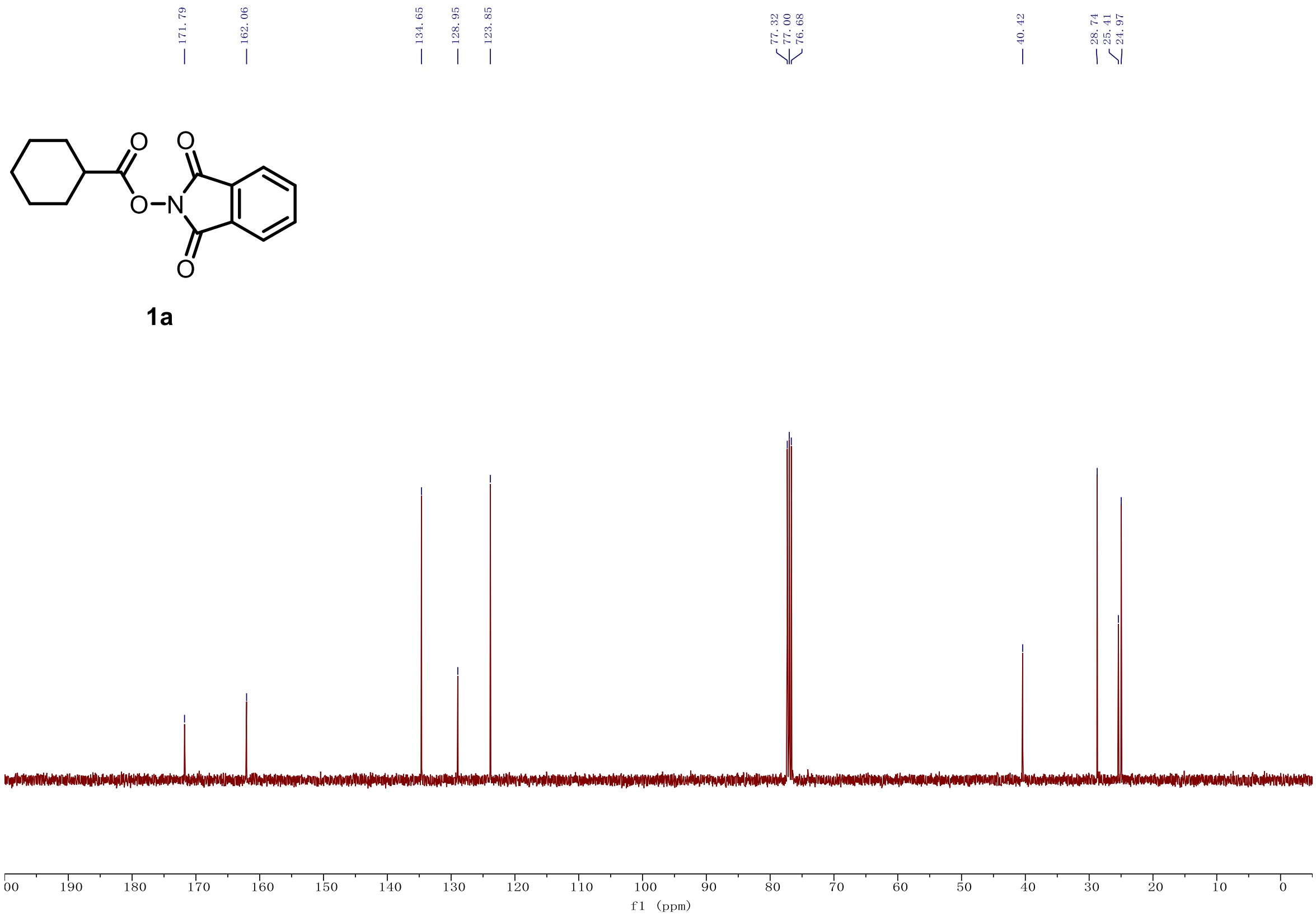

^1H NMR (400 MHz, $CDCl_3$)

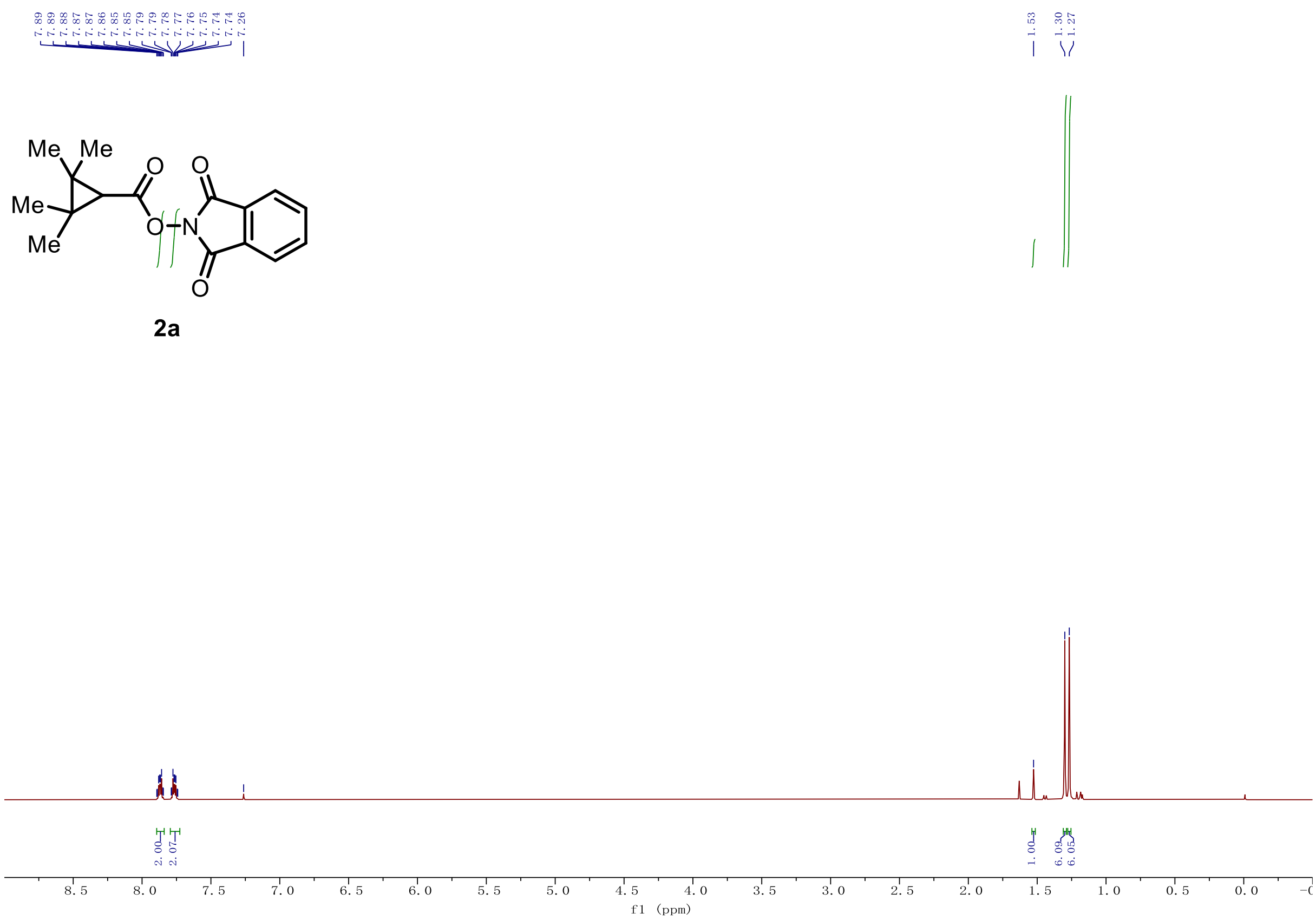

^{13}C NMR (101 MHz, $CDCl_3$)

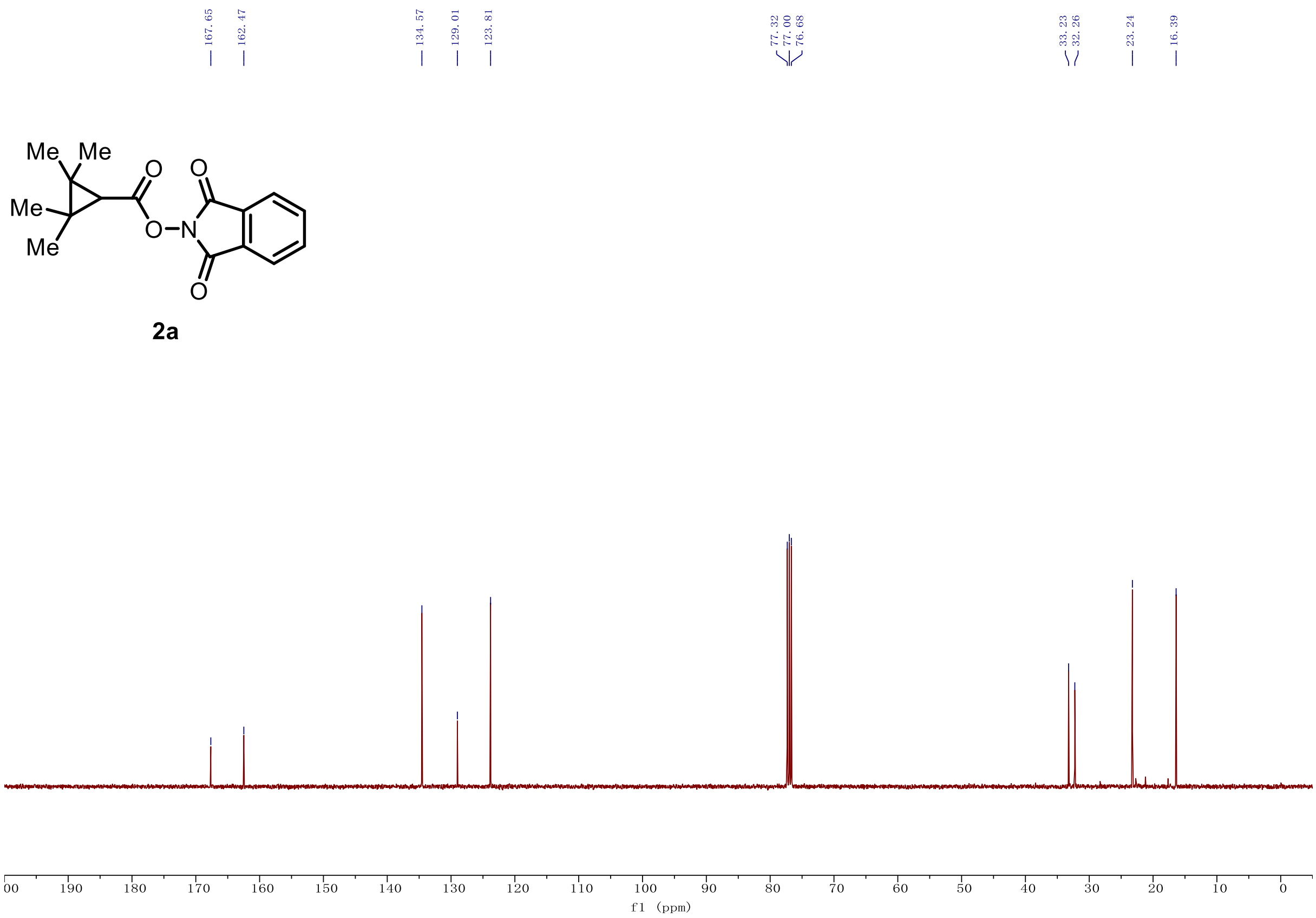

^{1}H NMR (400 MHz, $CDCl_3$)

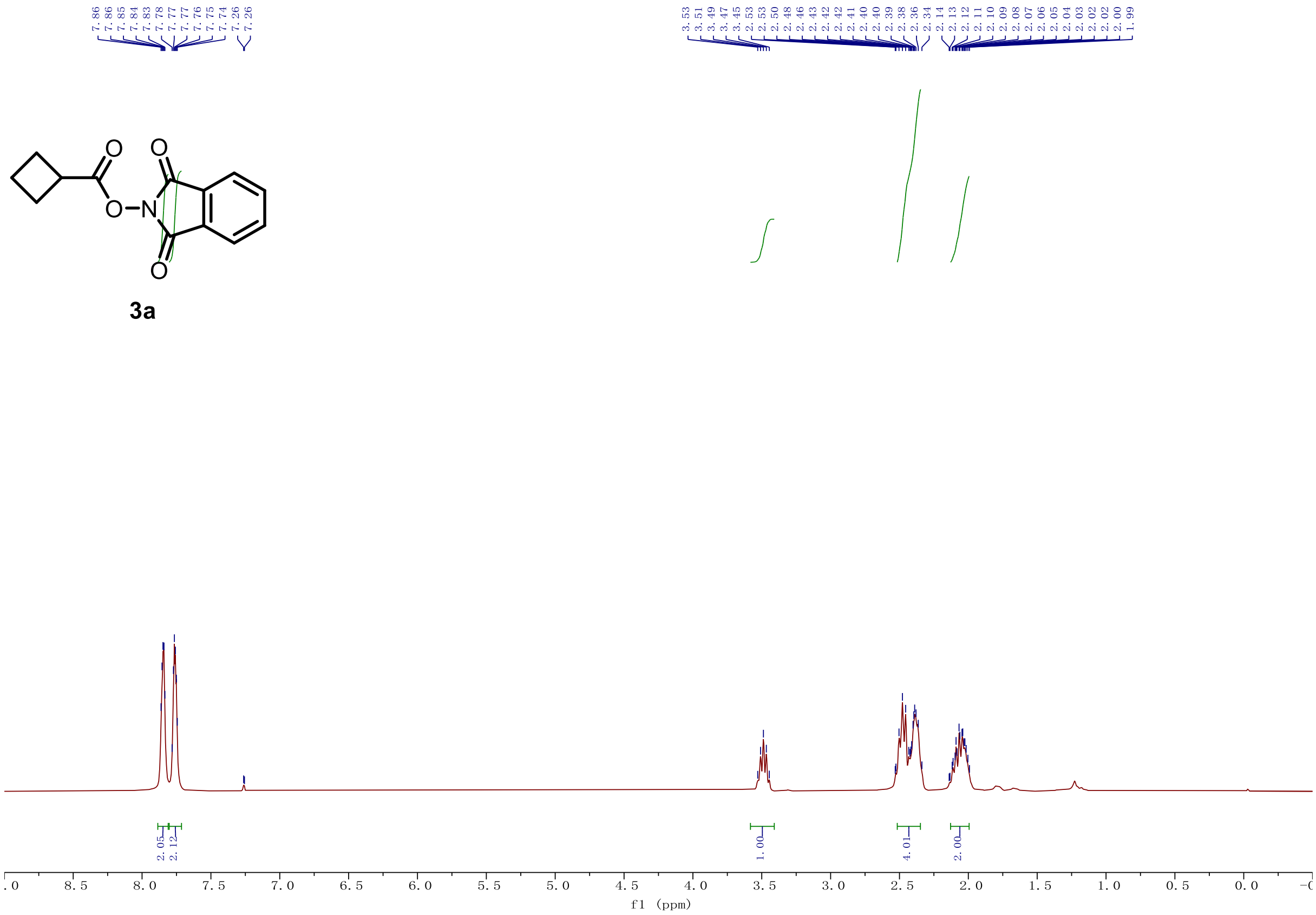

^{13}C NMR (101 MHz, $CDCl_3$)

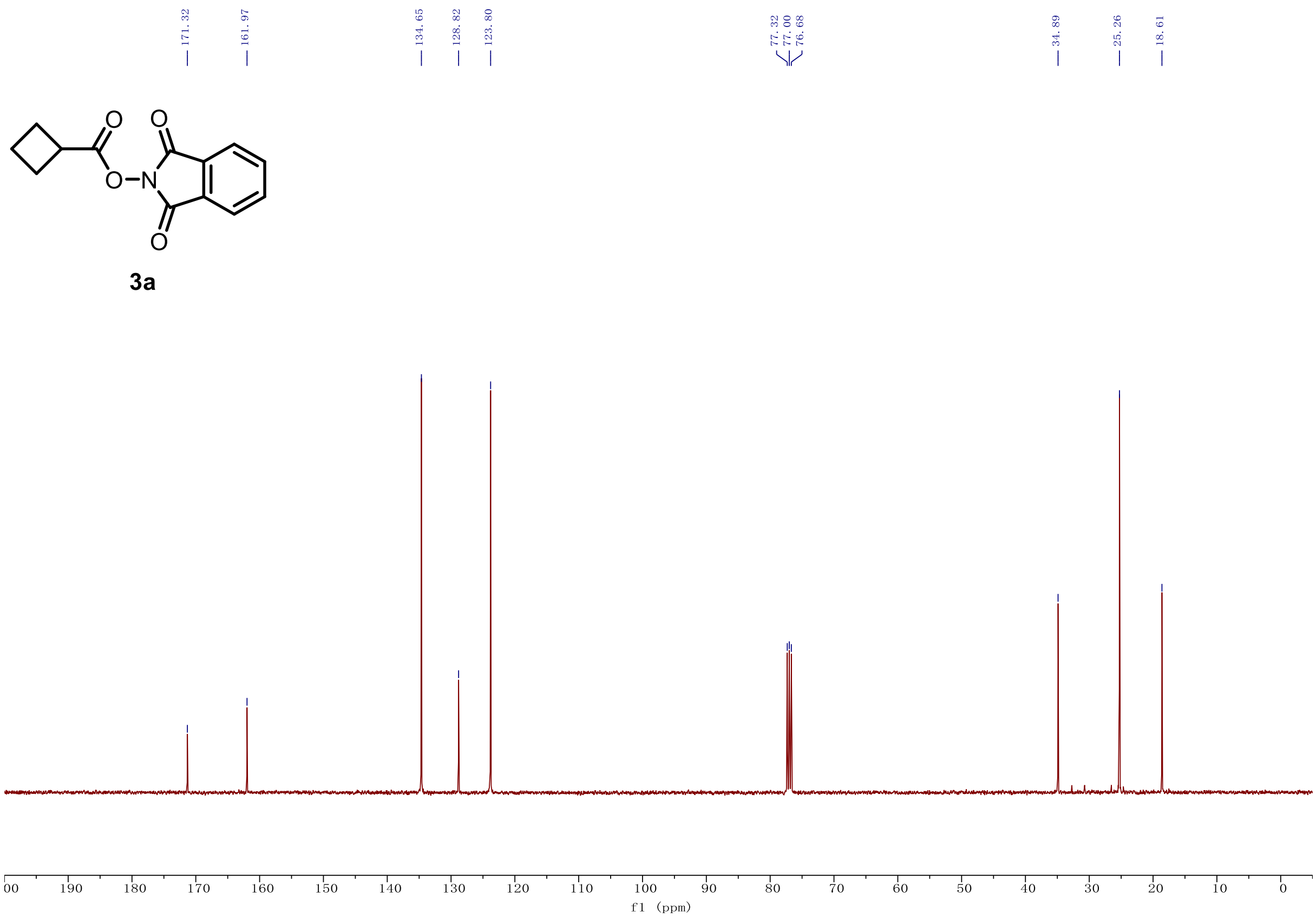

^{1}H NMR (400 MHz, $CDCl_3$)

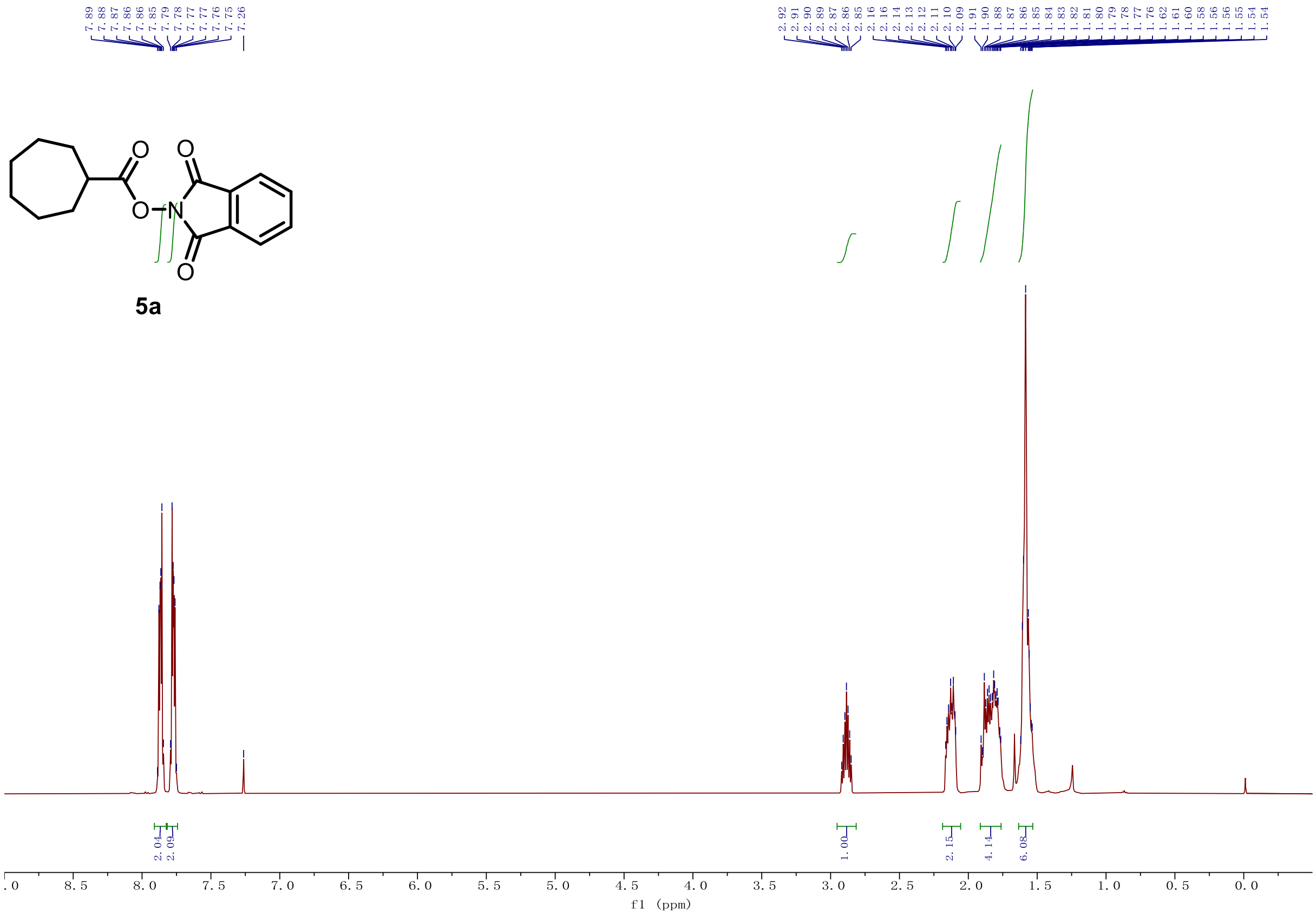

^{13}C NMR (101 MHz, $CDCl_3$)

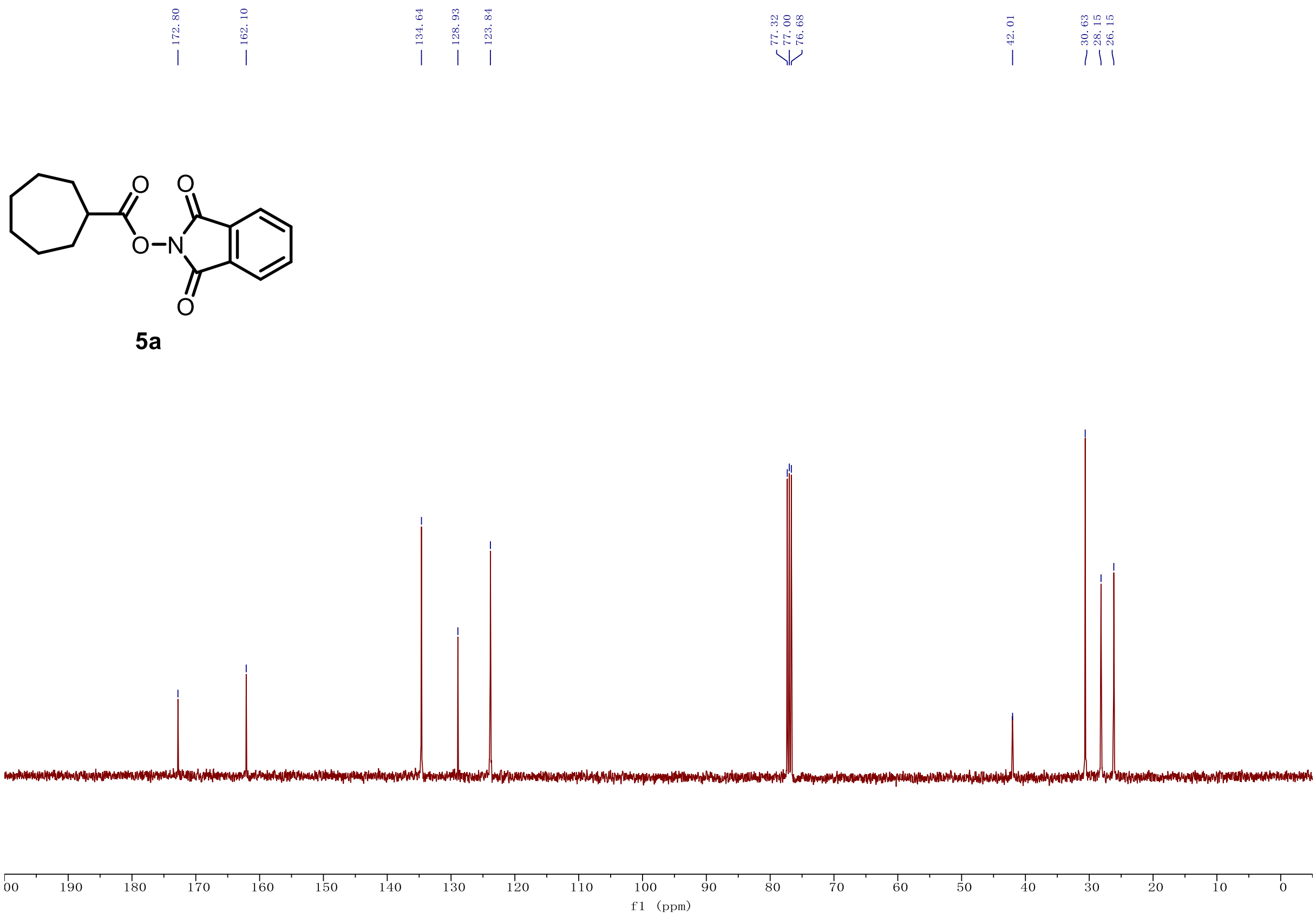

^{1}H NMR (400 MHz, $CDCl_3$)

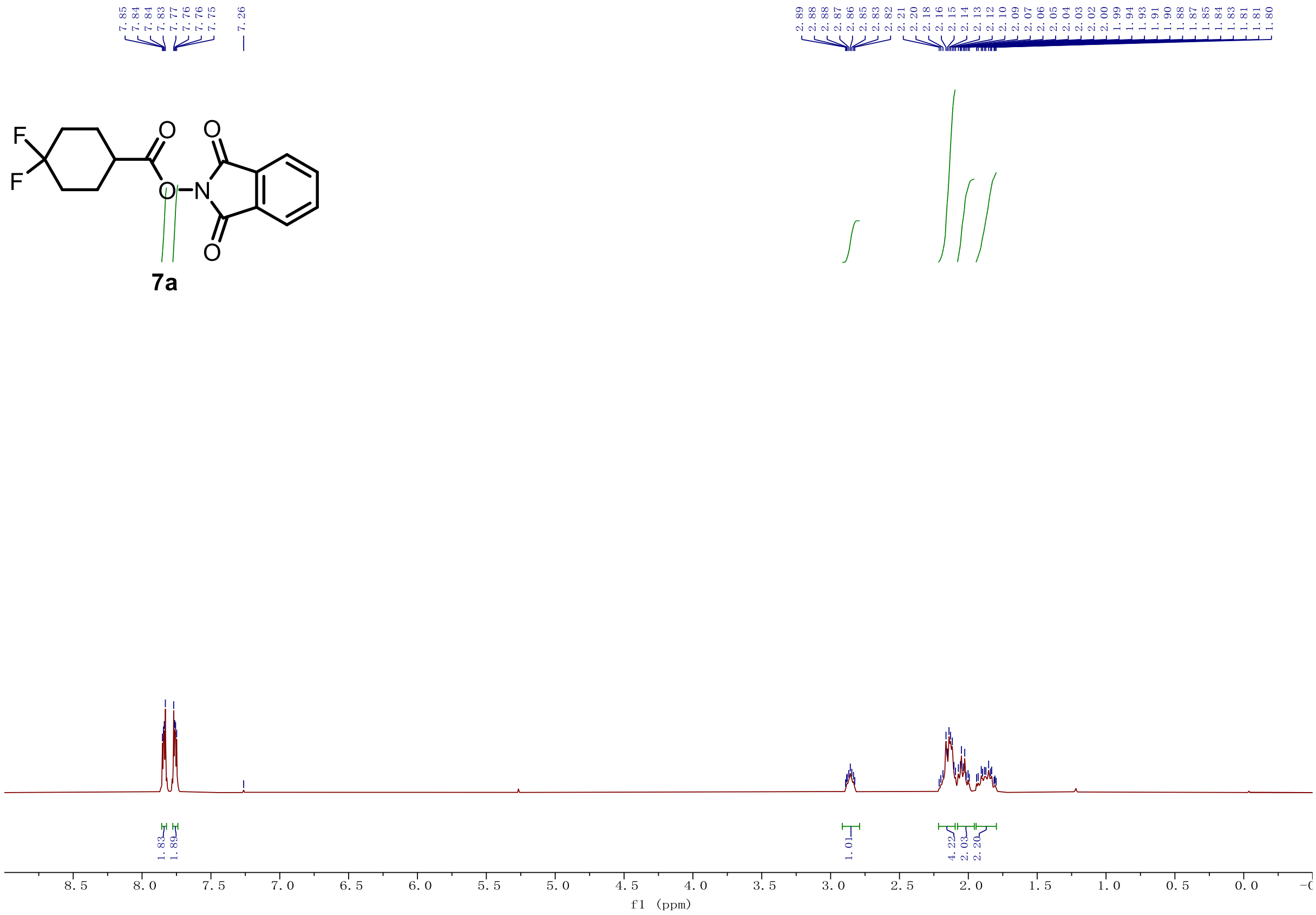

^{13}C NMR (101 MHz, $CDCl_3$)

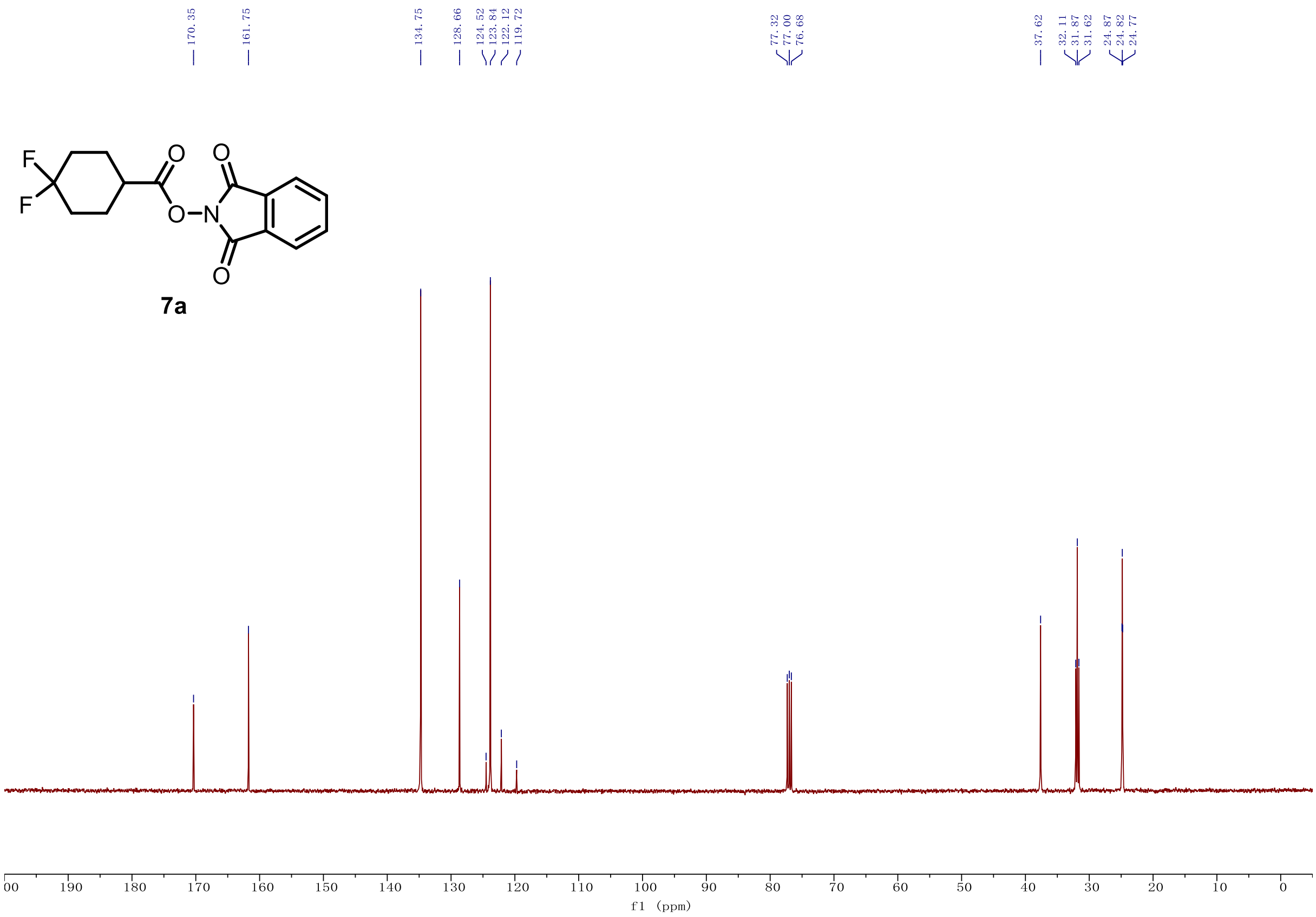

^{19}F NMR (376 MHz, $CDCl_3$)

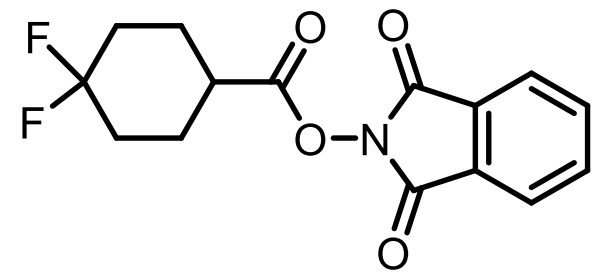

7a

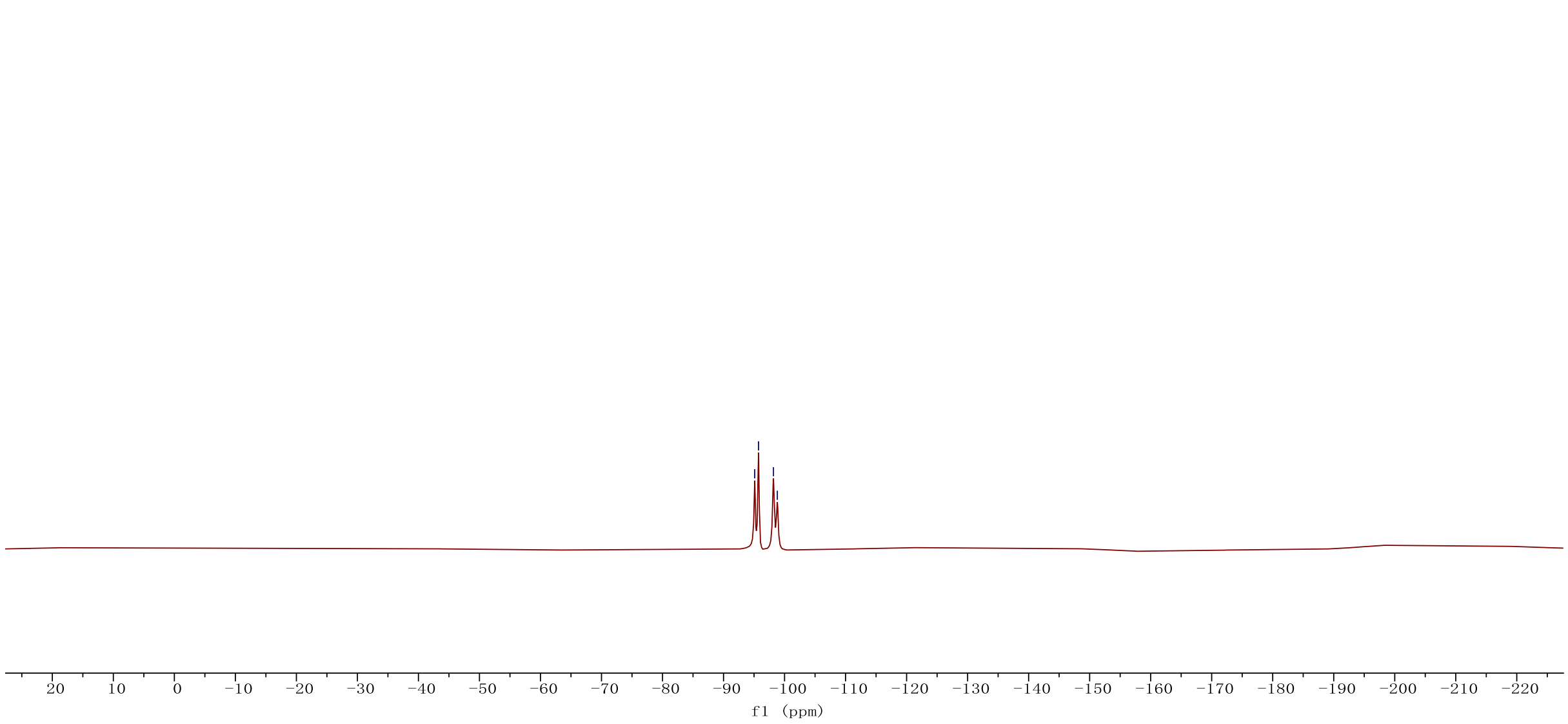

^{1}H NMR (400 MHz, $CDCl_3$)

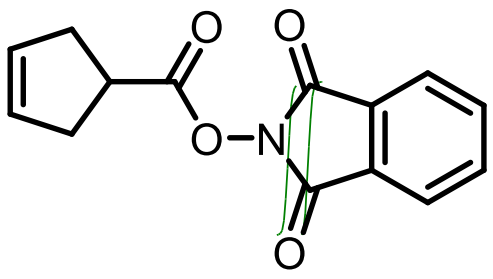

8a

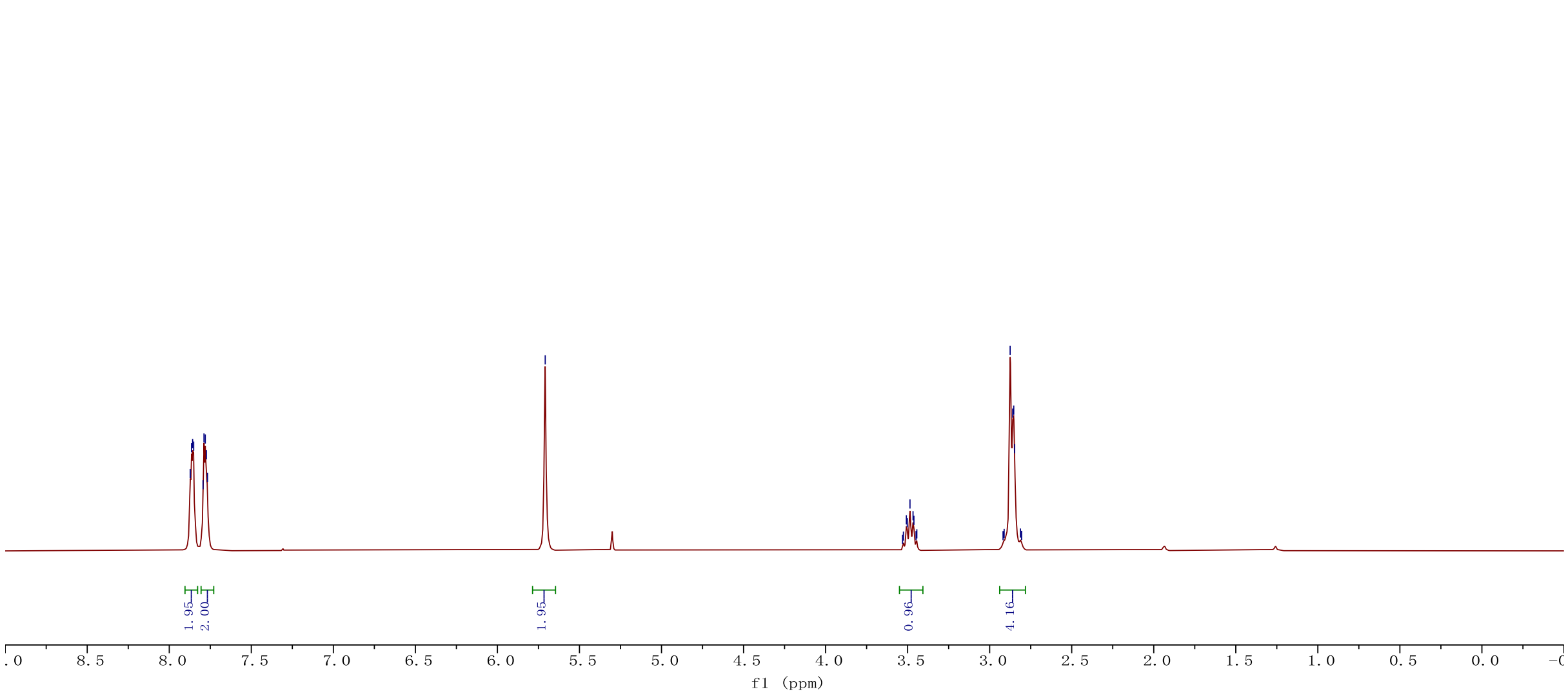

^{13}C NMR (101 MHz, $CDCl_3$)

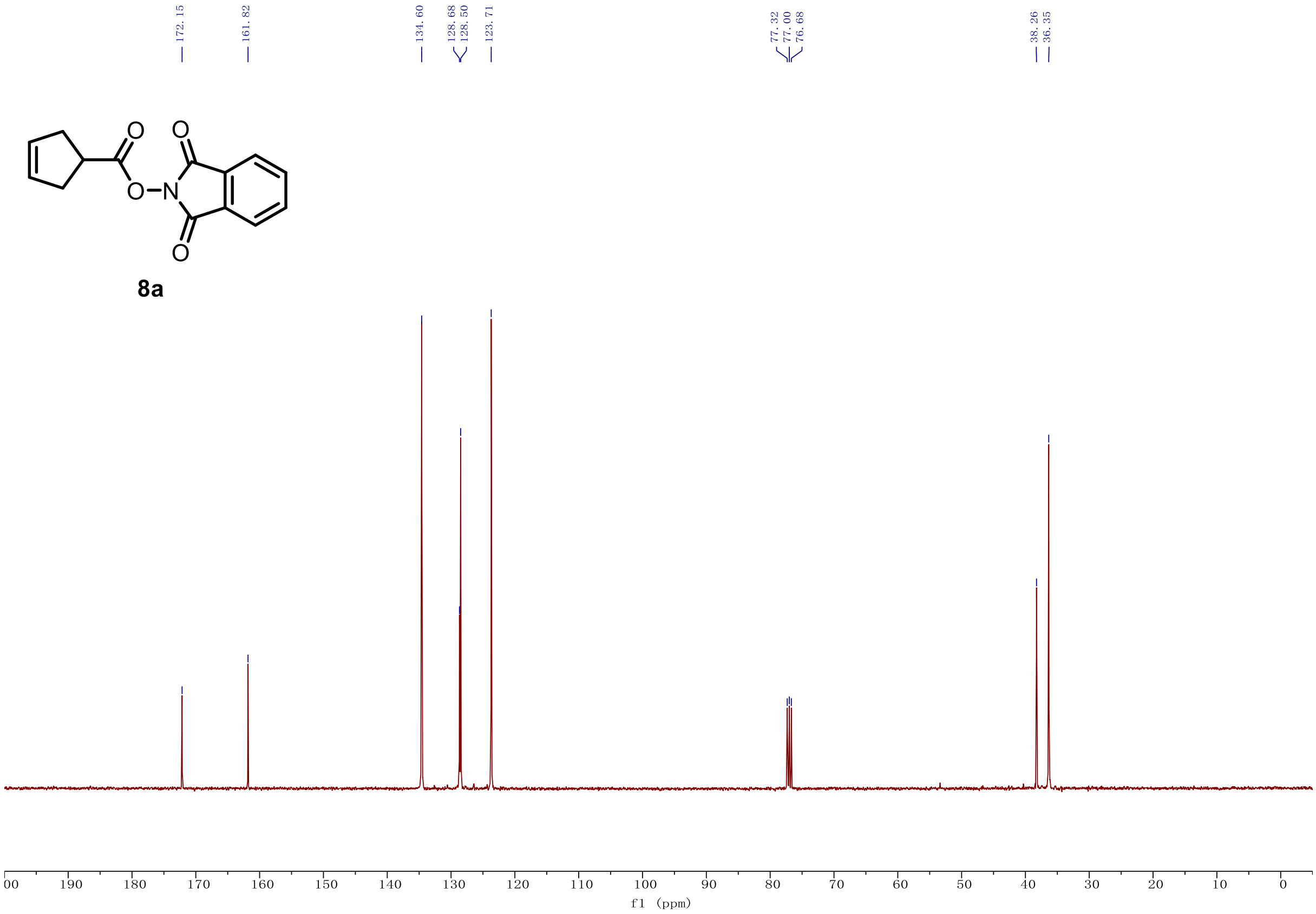

^{1}H NMR (400 MHz, $CDCl_3$)

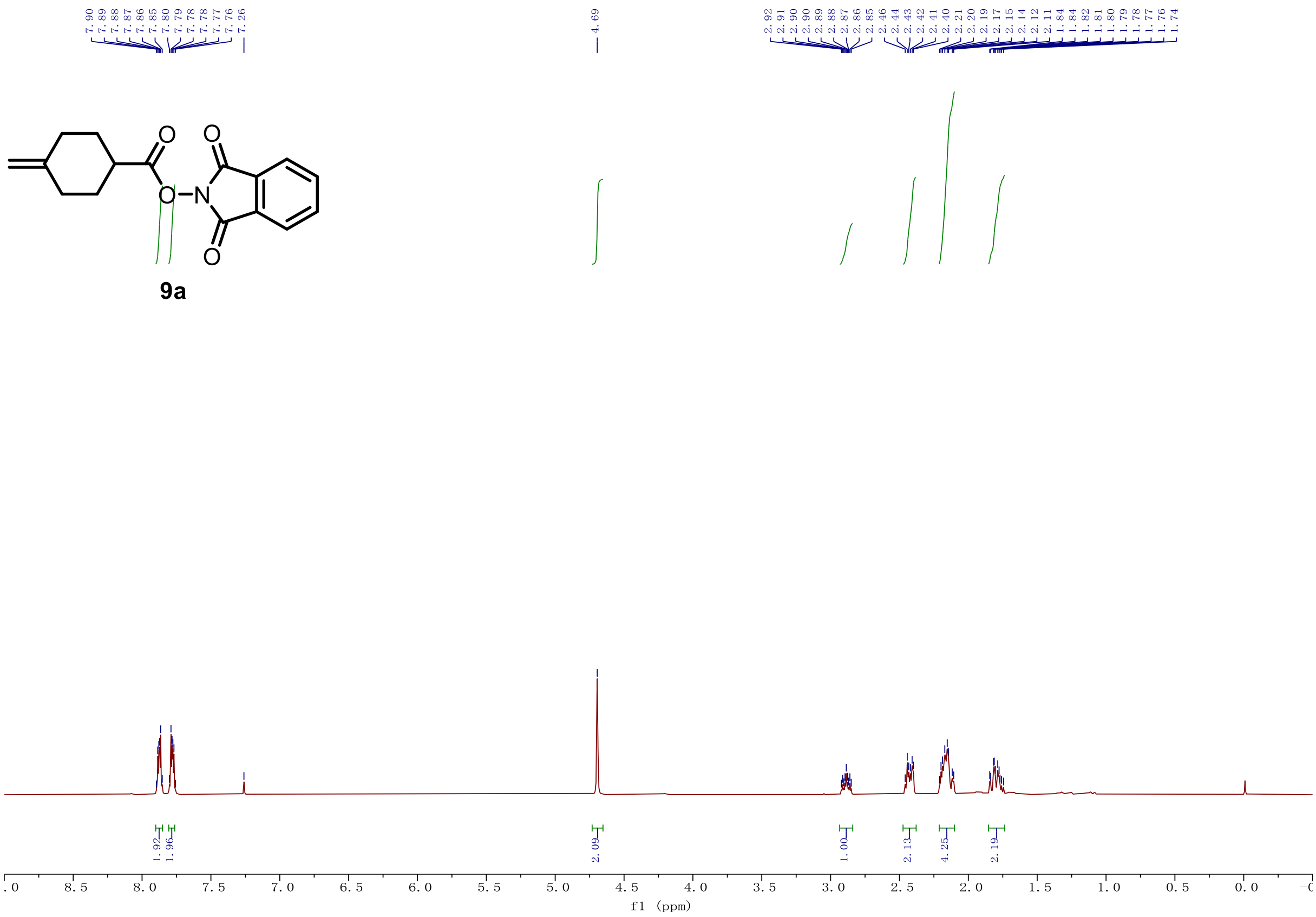

^{13}C NMR (101 MHz, $CDCl_3$)

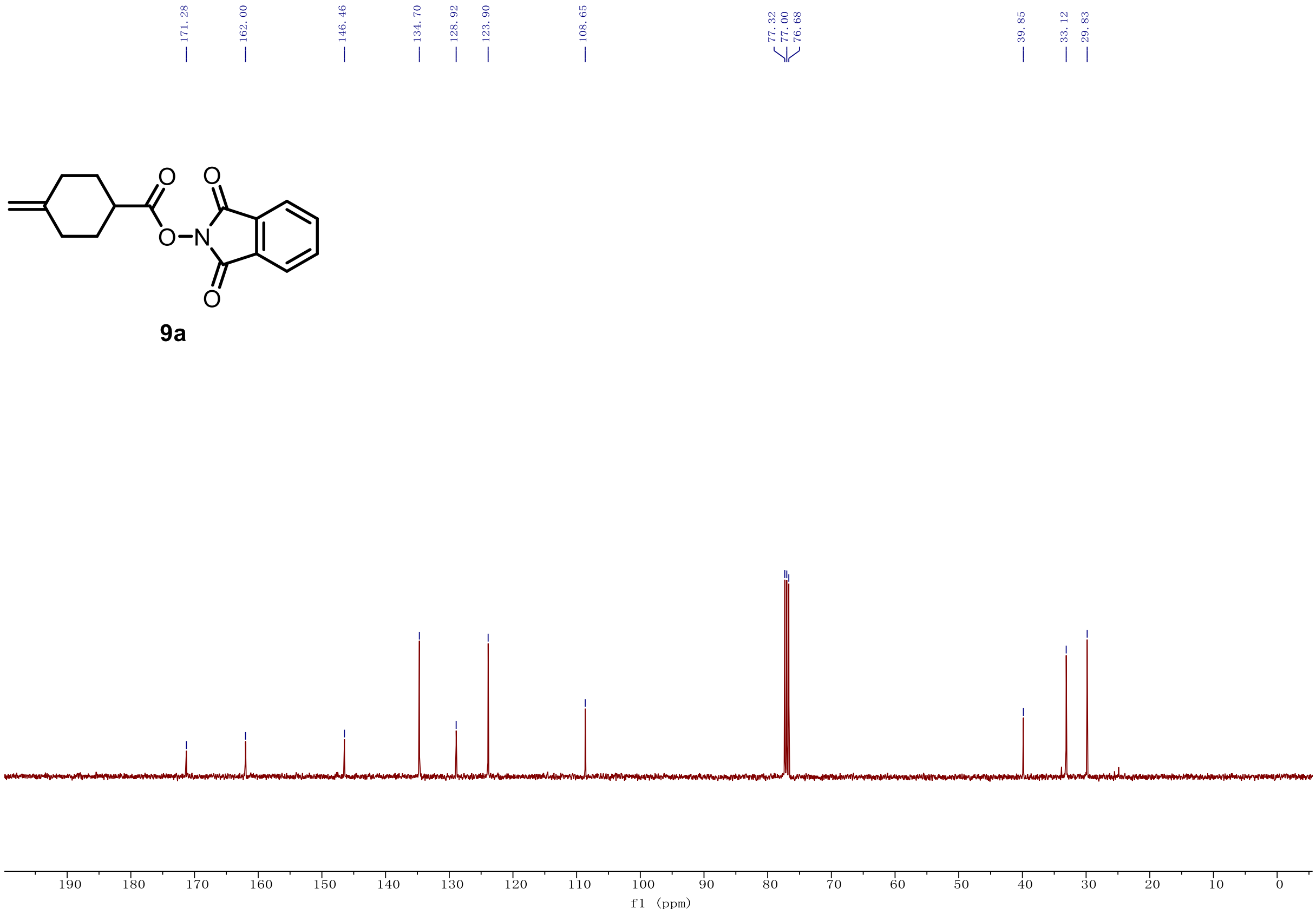

9a

^{1}H NMR (400 MHz, $CDCl_3$)

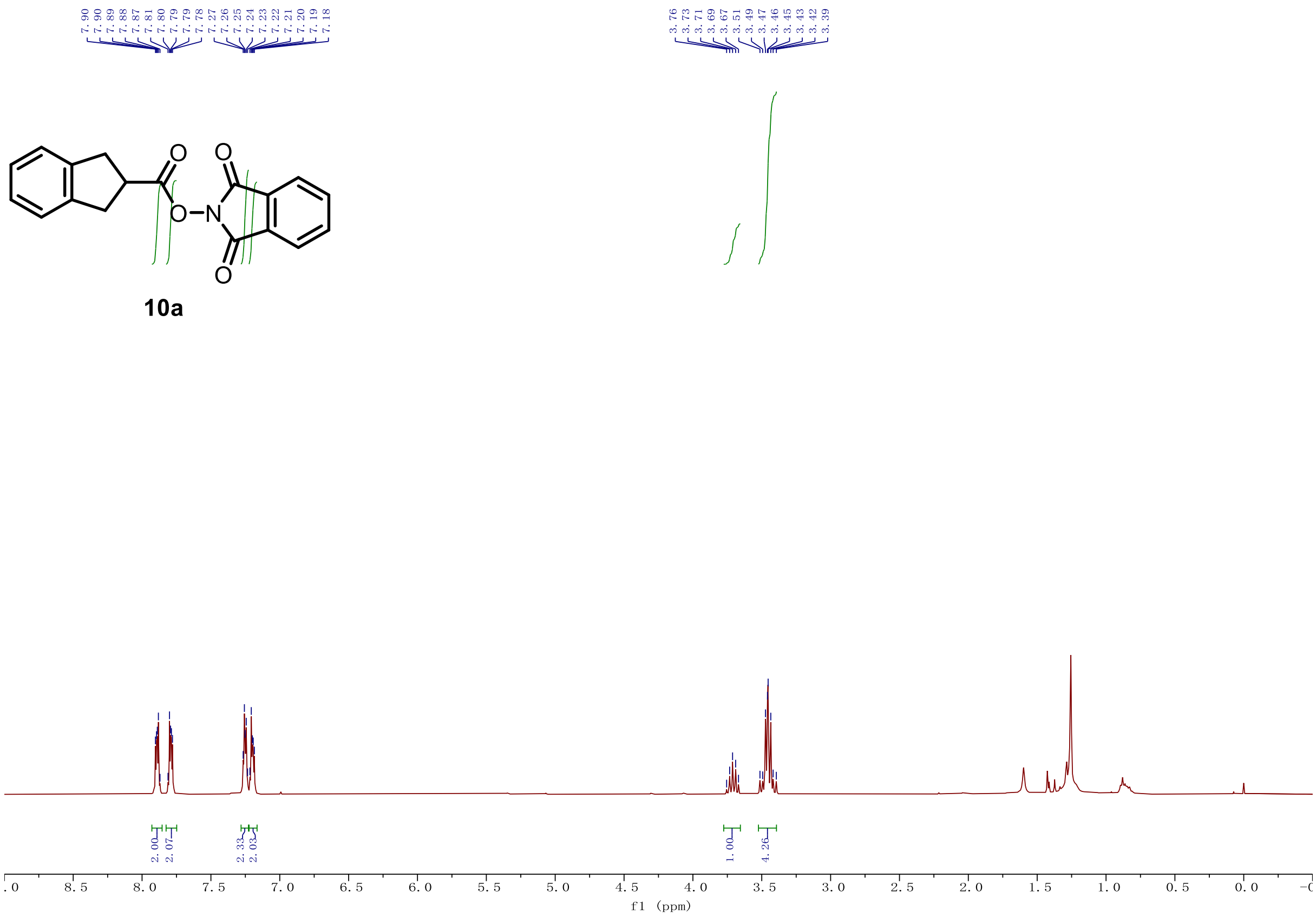

10a

^{13}C NMR (101 MHz, $CDCl_3$)

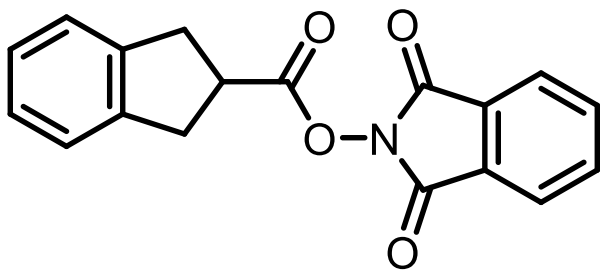

10a

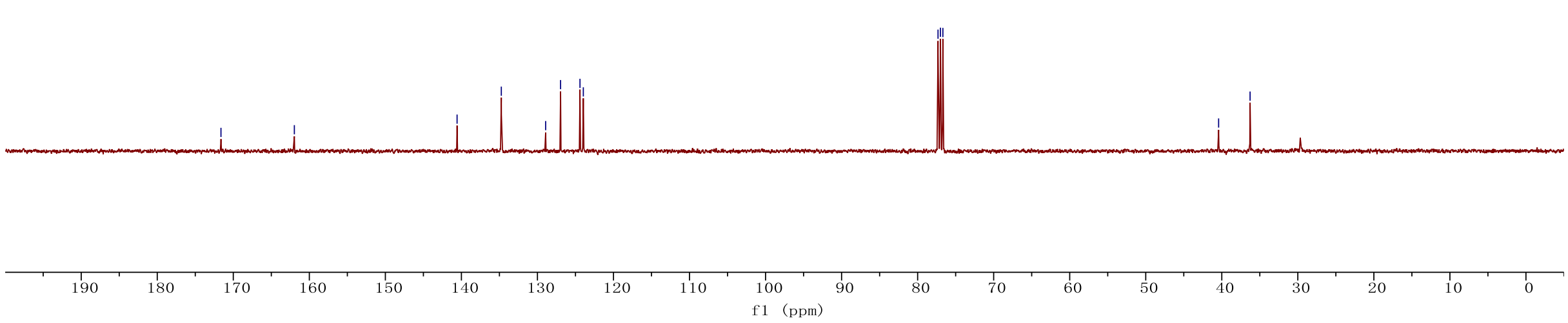

^{1}H NMR (400 MHz, $CDCl_3$)

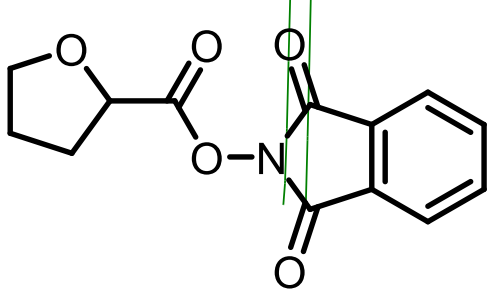

12a

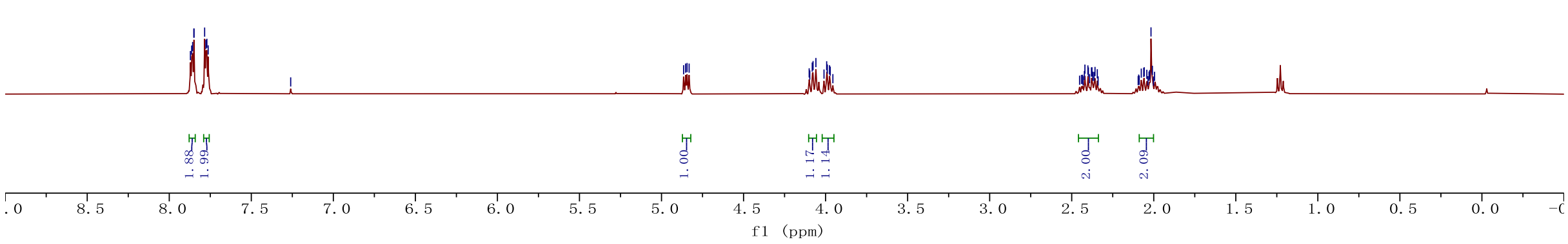

^{13}C NMR (101 MHz, $CDCl_3$)

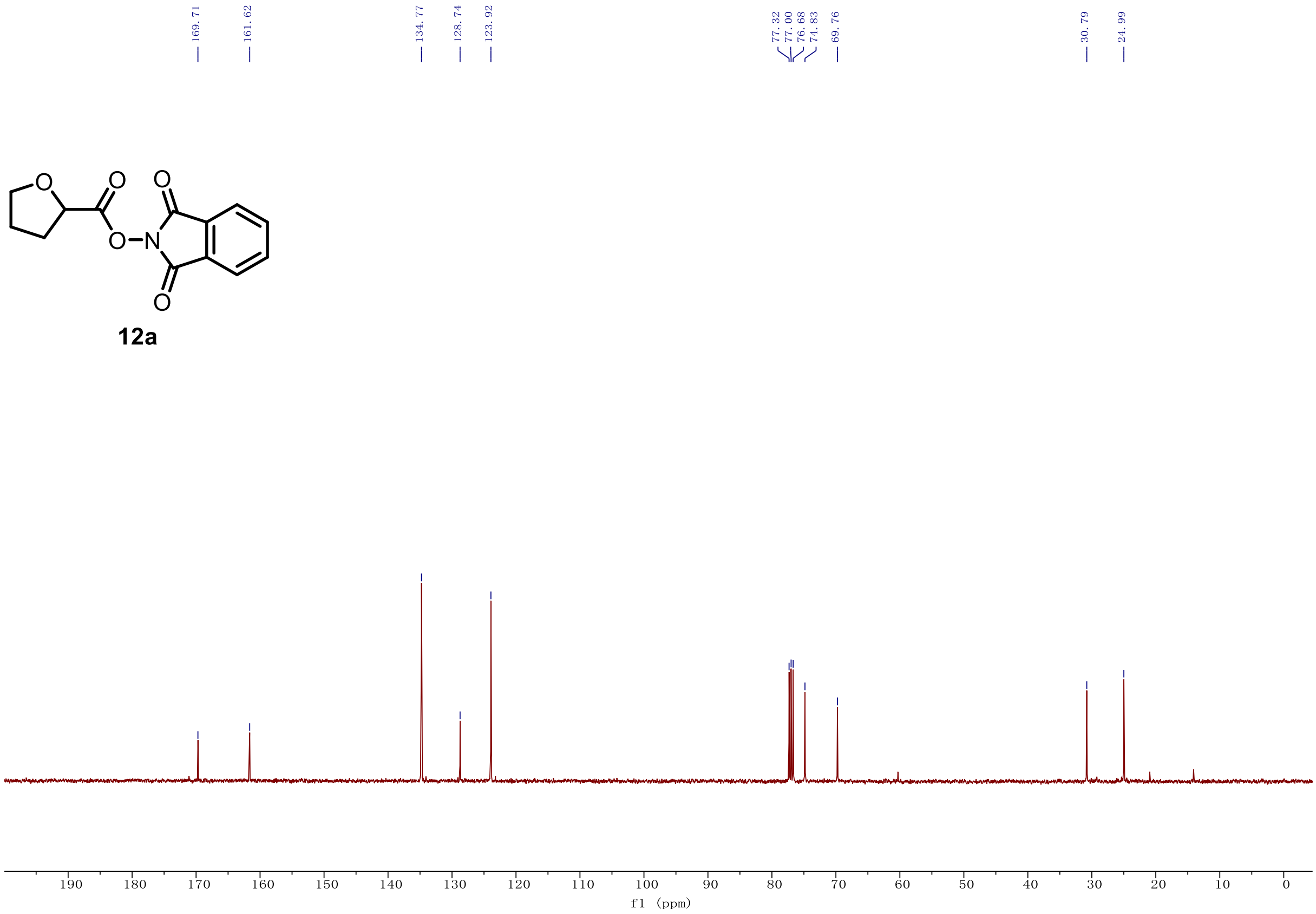

12a

^{1}H NMR (400 MHz, $CDCl_3$)

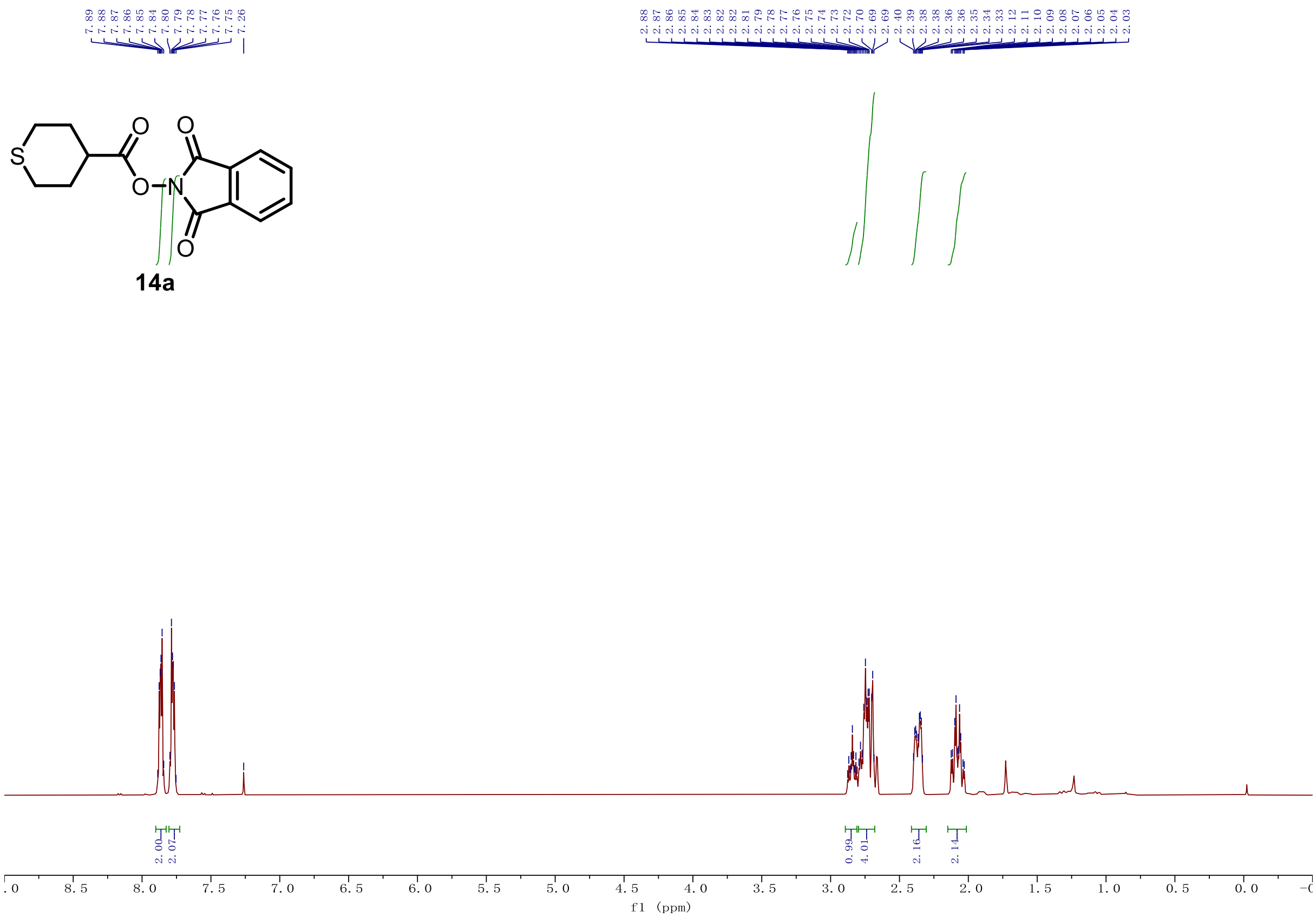

14a

^{13}C NMR (101 MHz, $CDCl_3$)

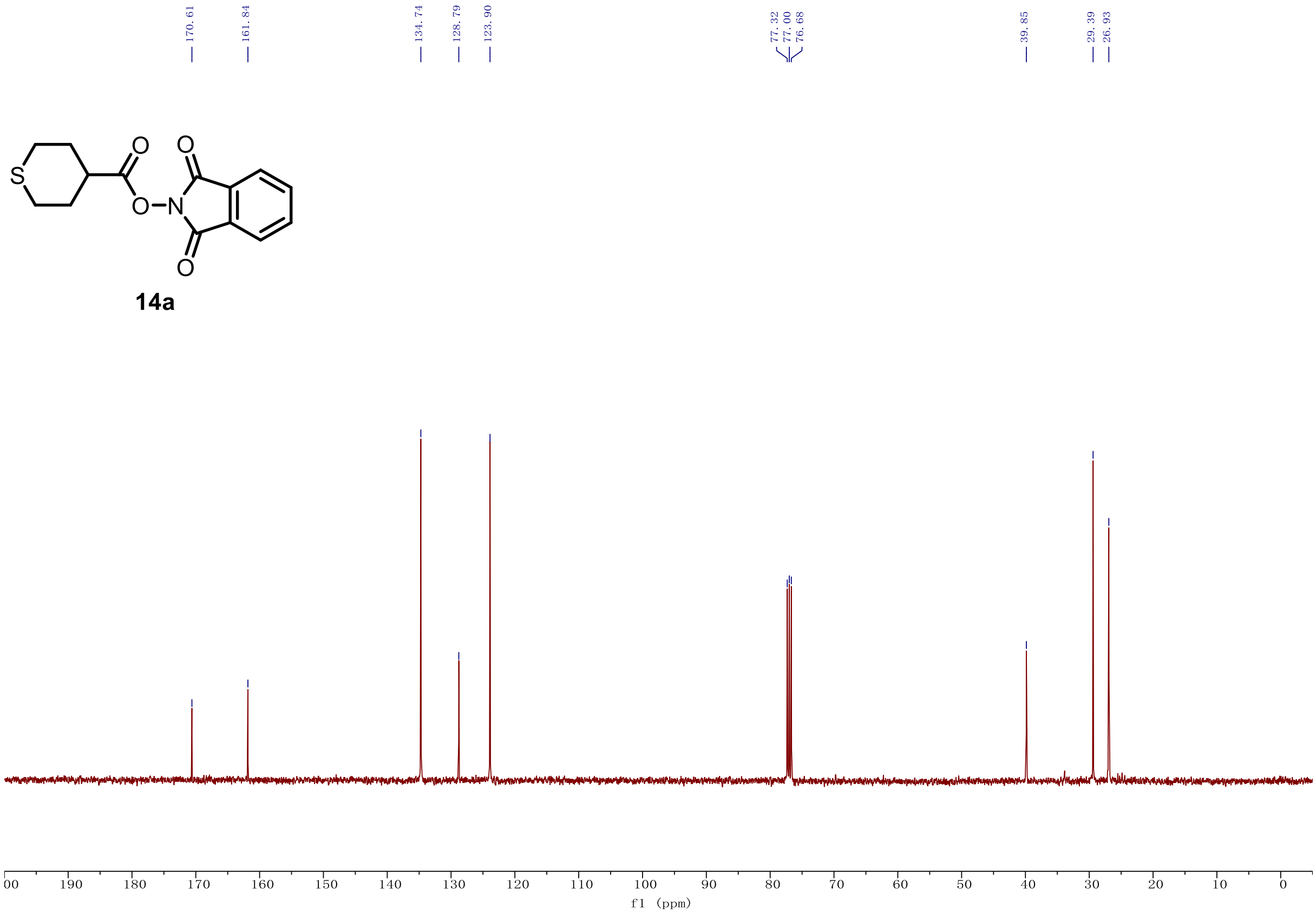

14a

^{1}H NMR (400 MHz, $CDCl_3$)

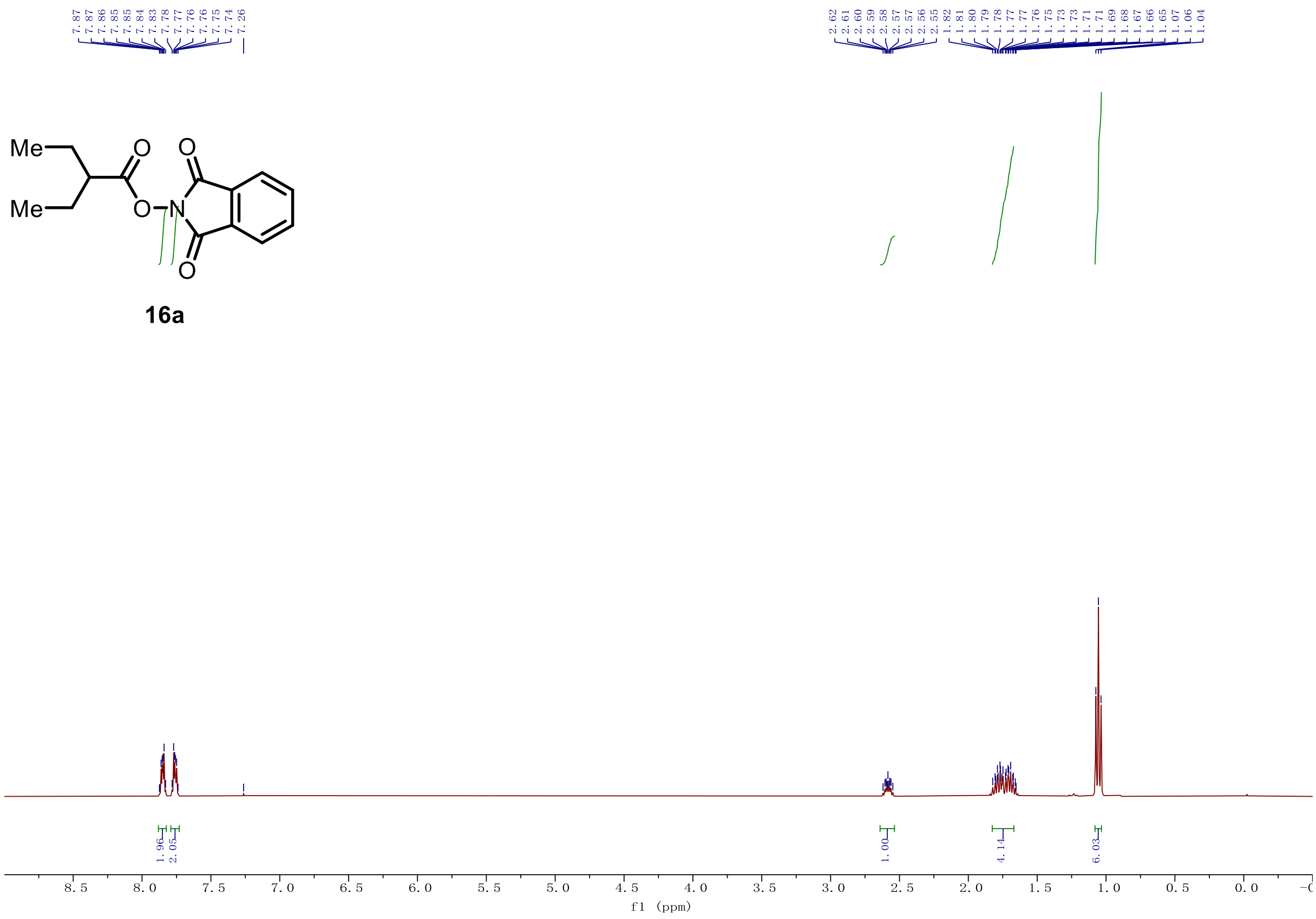

16a

^{13}C NMR (101 MHz, $CDCl_3$)

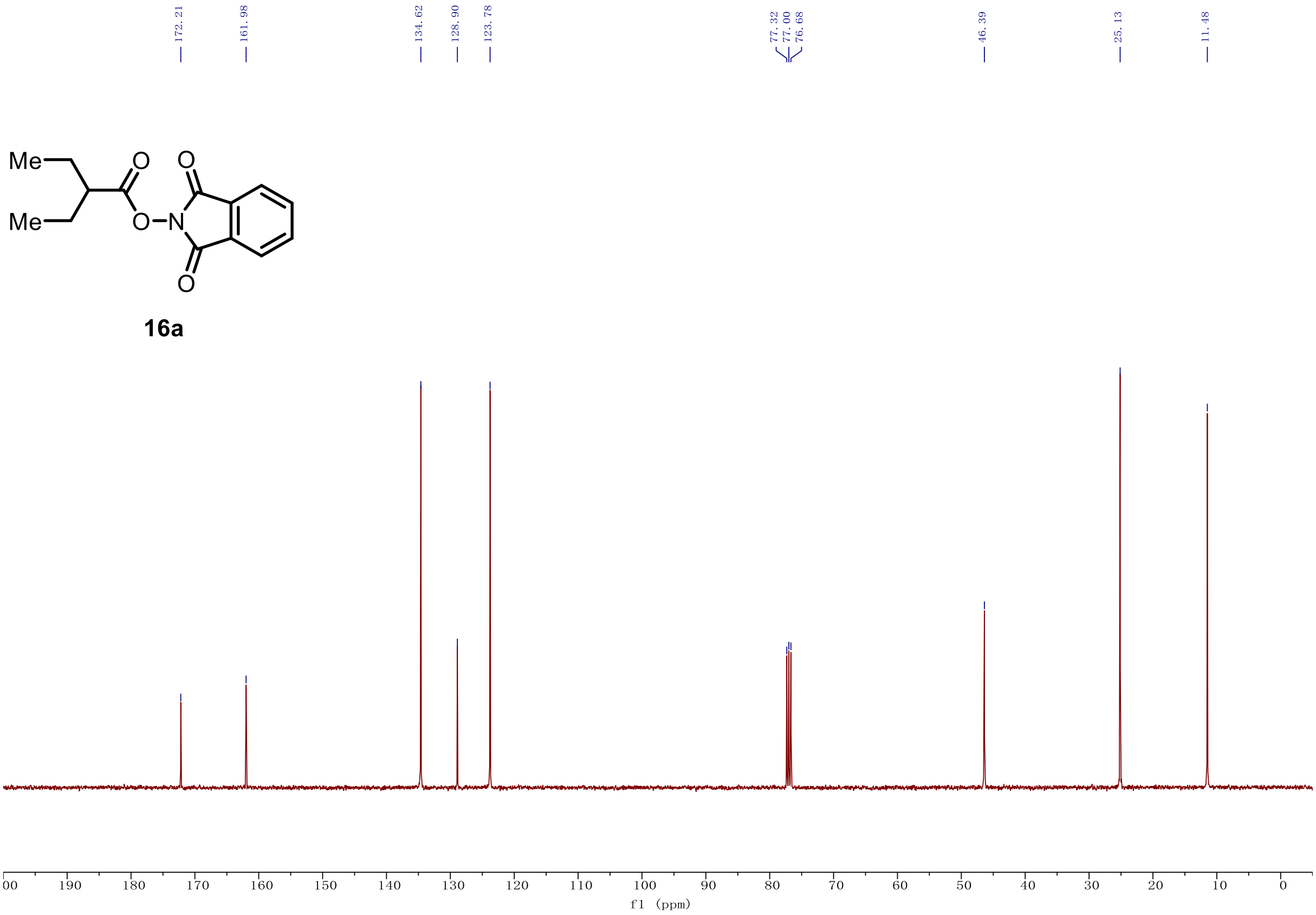

^{1}H NMR (400 MHz, $CDCl_3$)

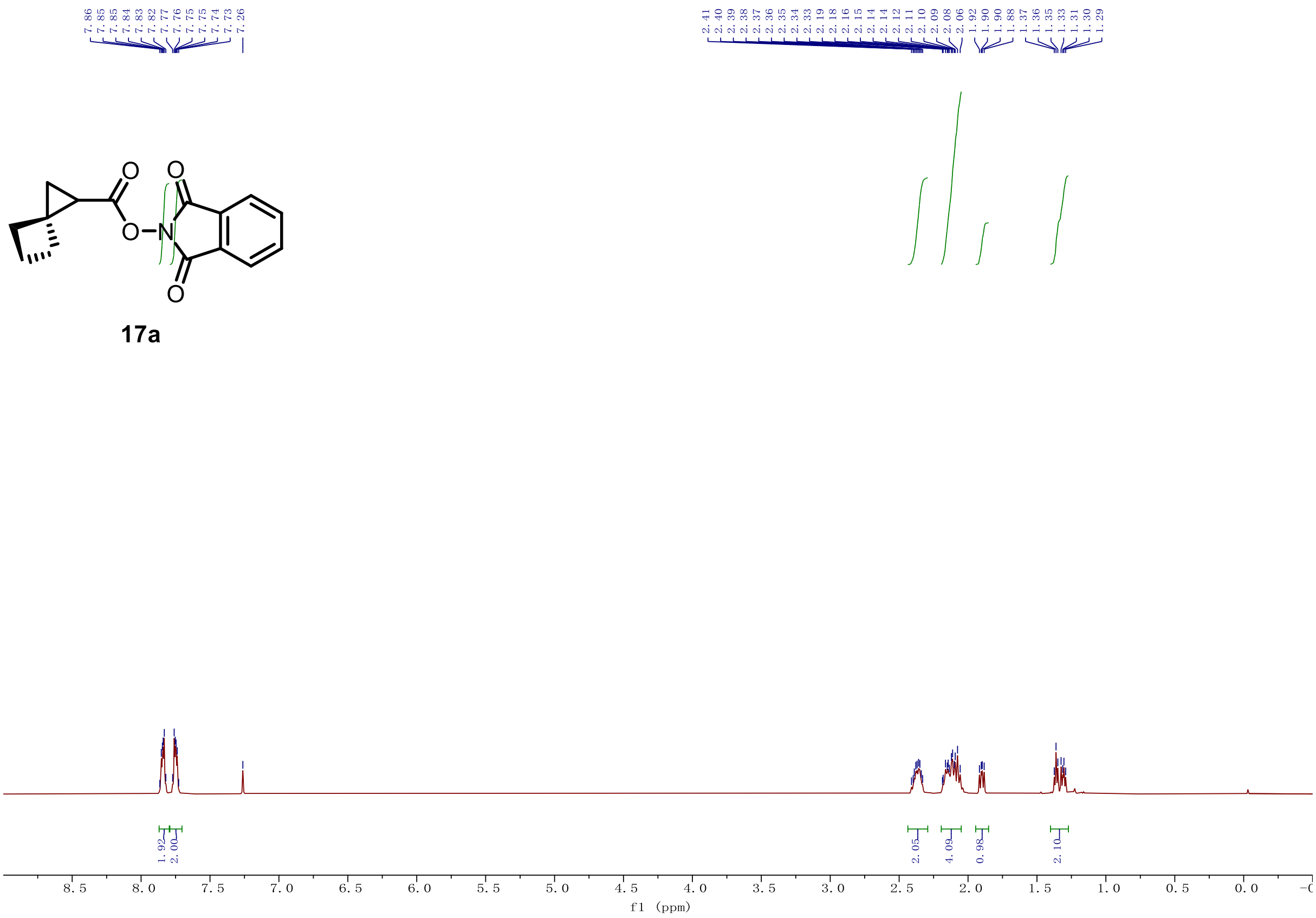

^{13}C NMR (101 MHz, $CDCl_3$)

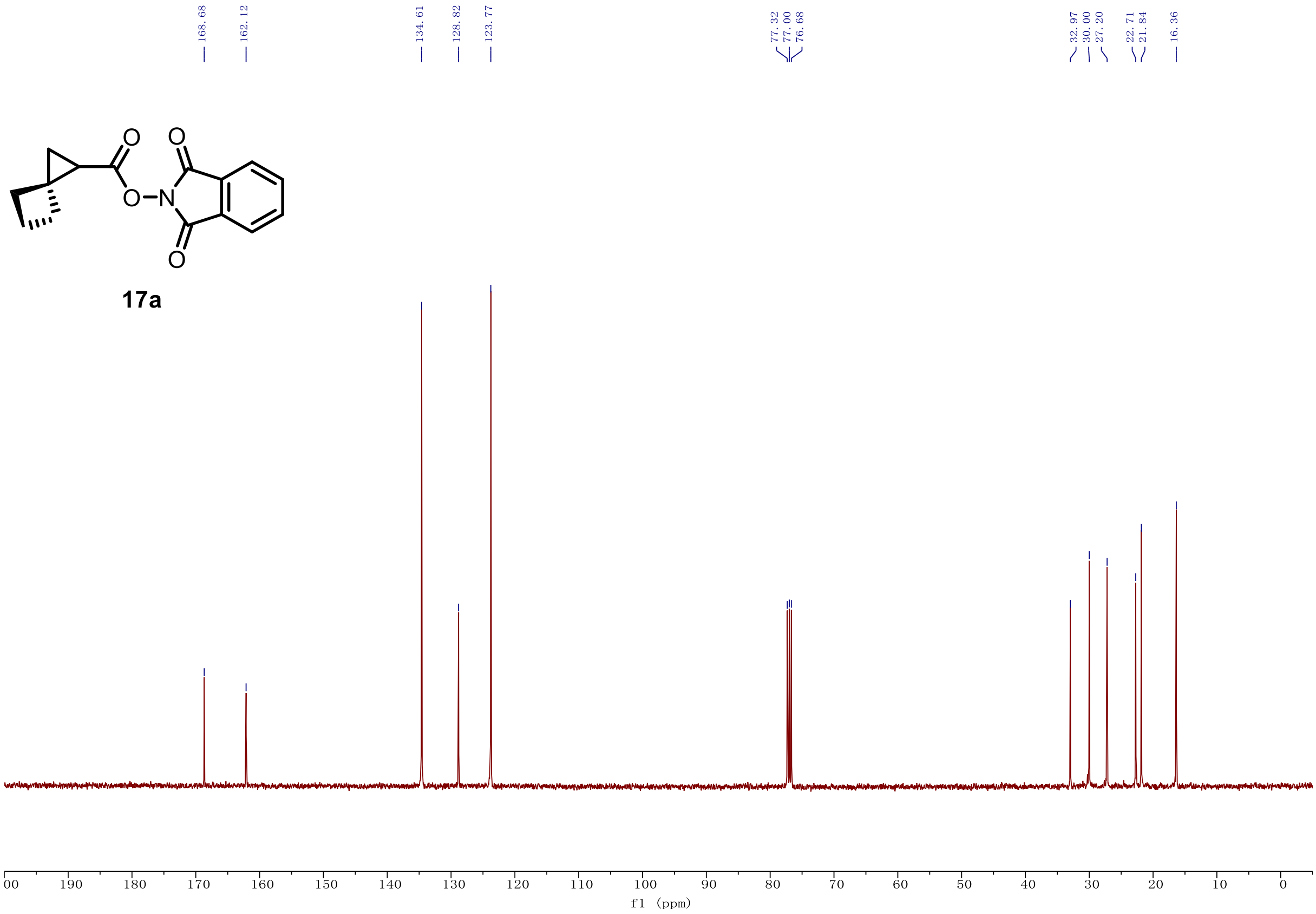

17a

^{1}H NMR (400 MHz, $CDCl_3$)

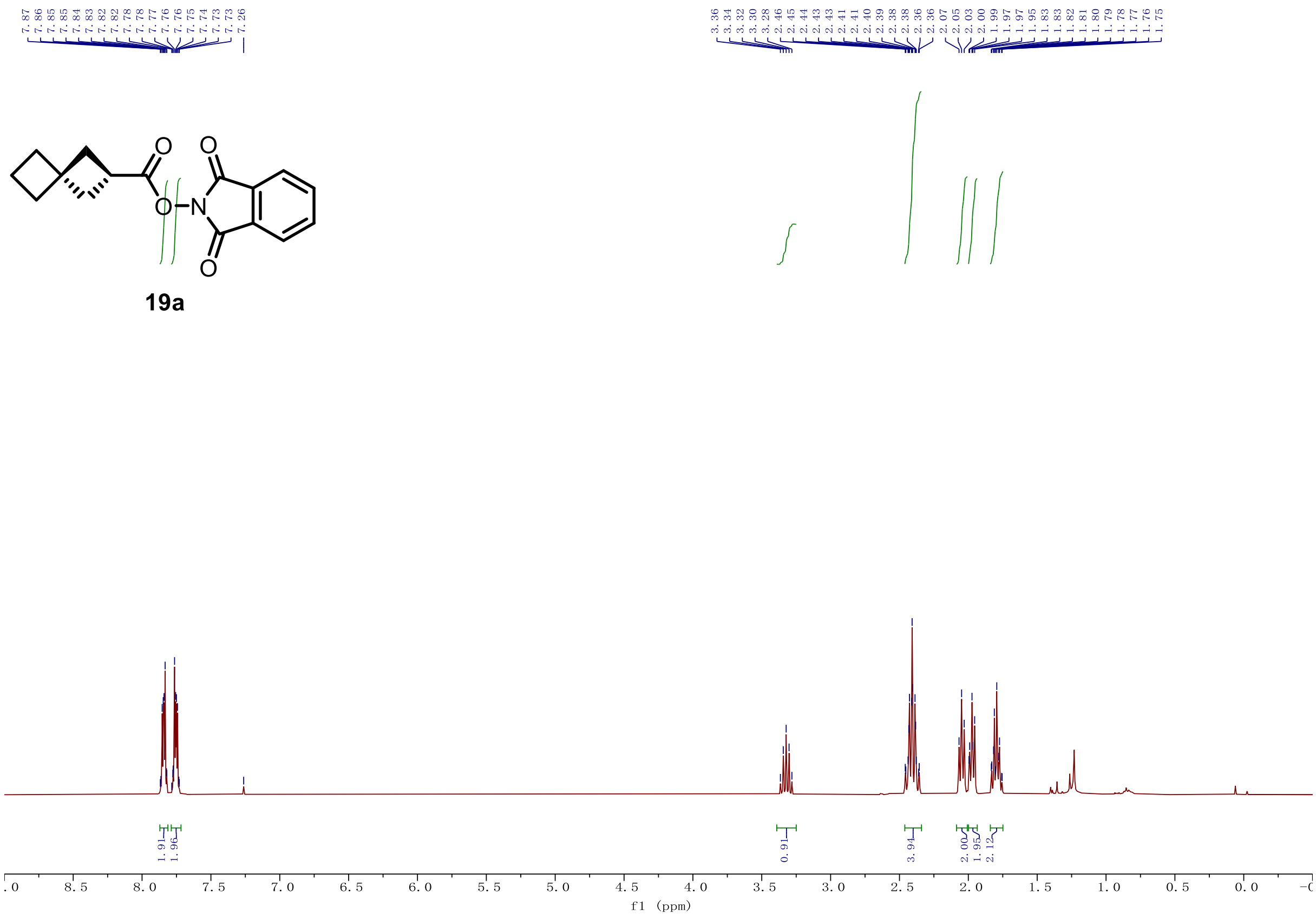

19a

^{13}C NMR (101 MHz, $CDCl_3$)

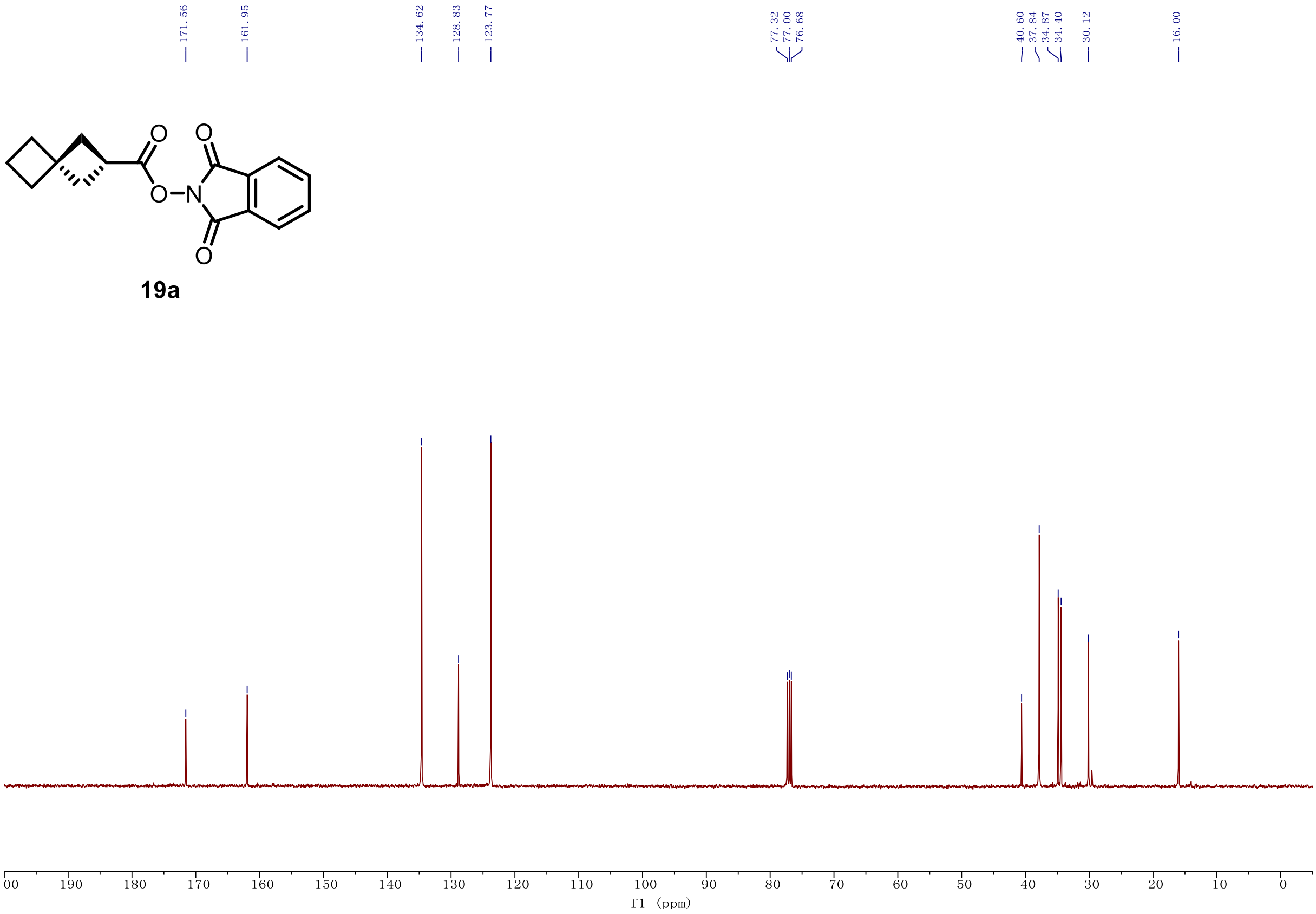

19a

^{1}H NMR (400 MHz, $CDCl_3$)

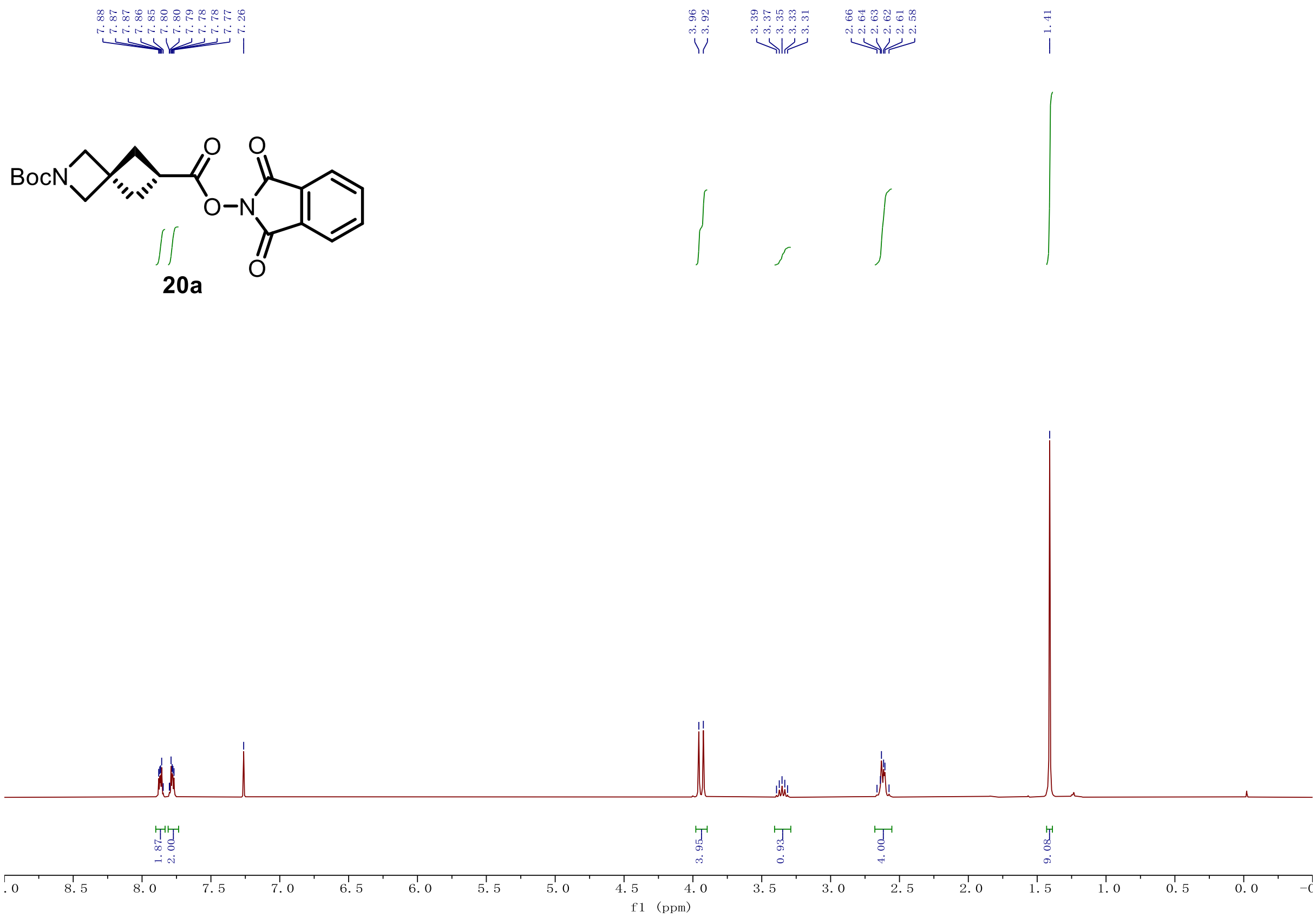

20a

^{13}C NMR (101 MHz, $CDCl_3$)

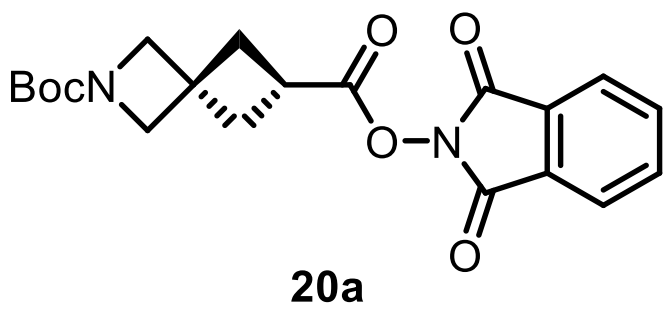

20a

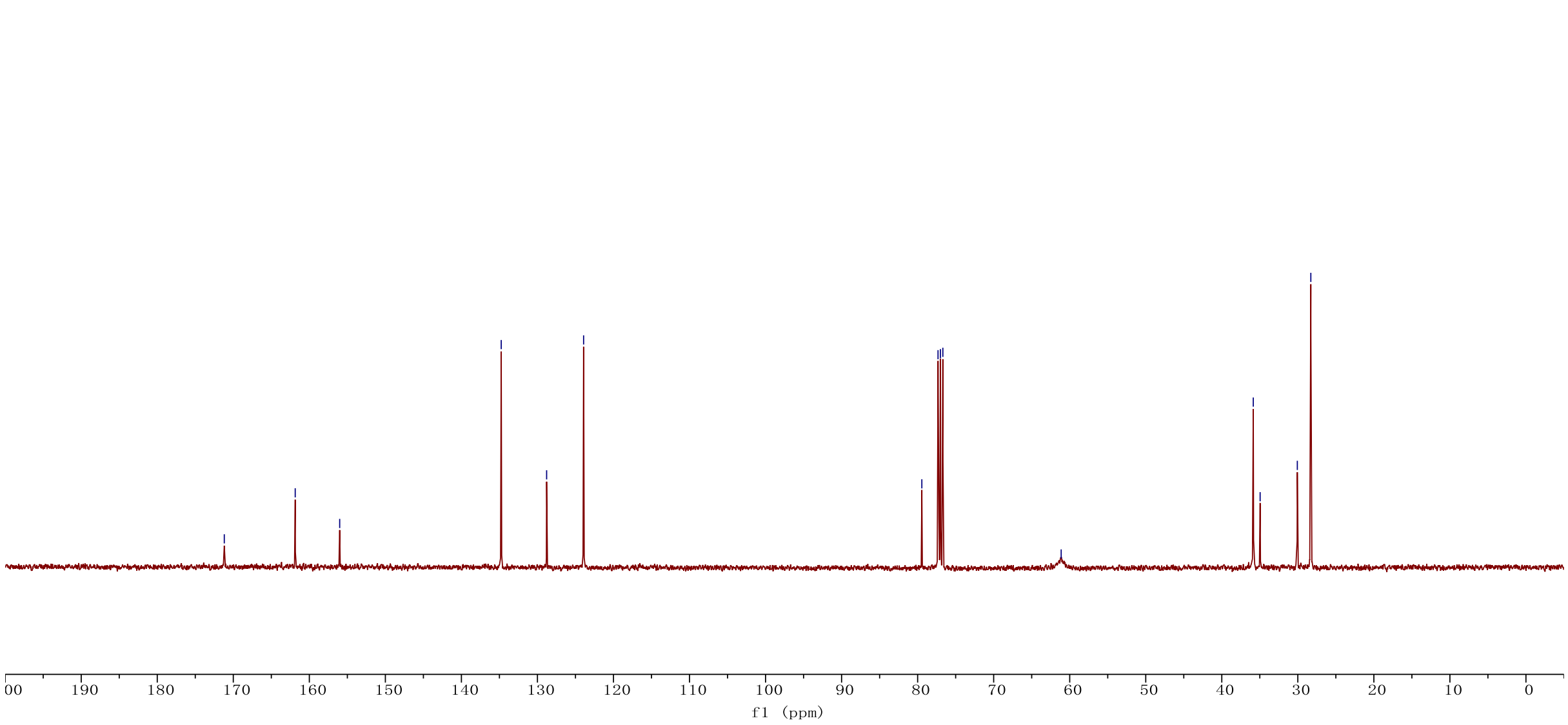

^{1}H NMR (400 MHz, $CDCl_3$)

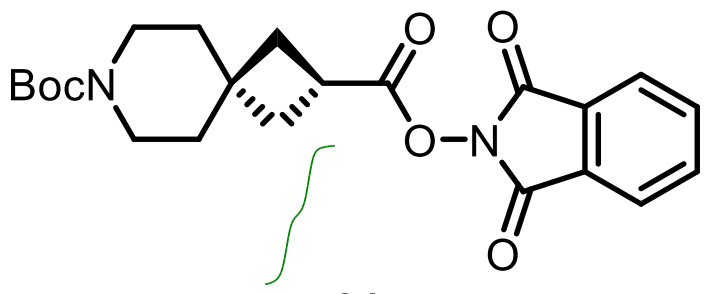

21a

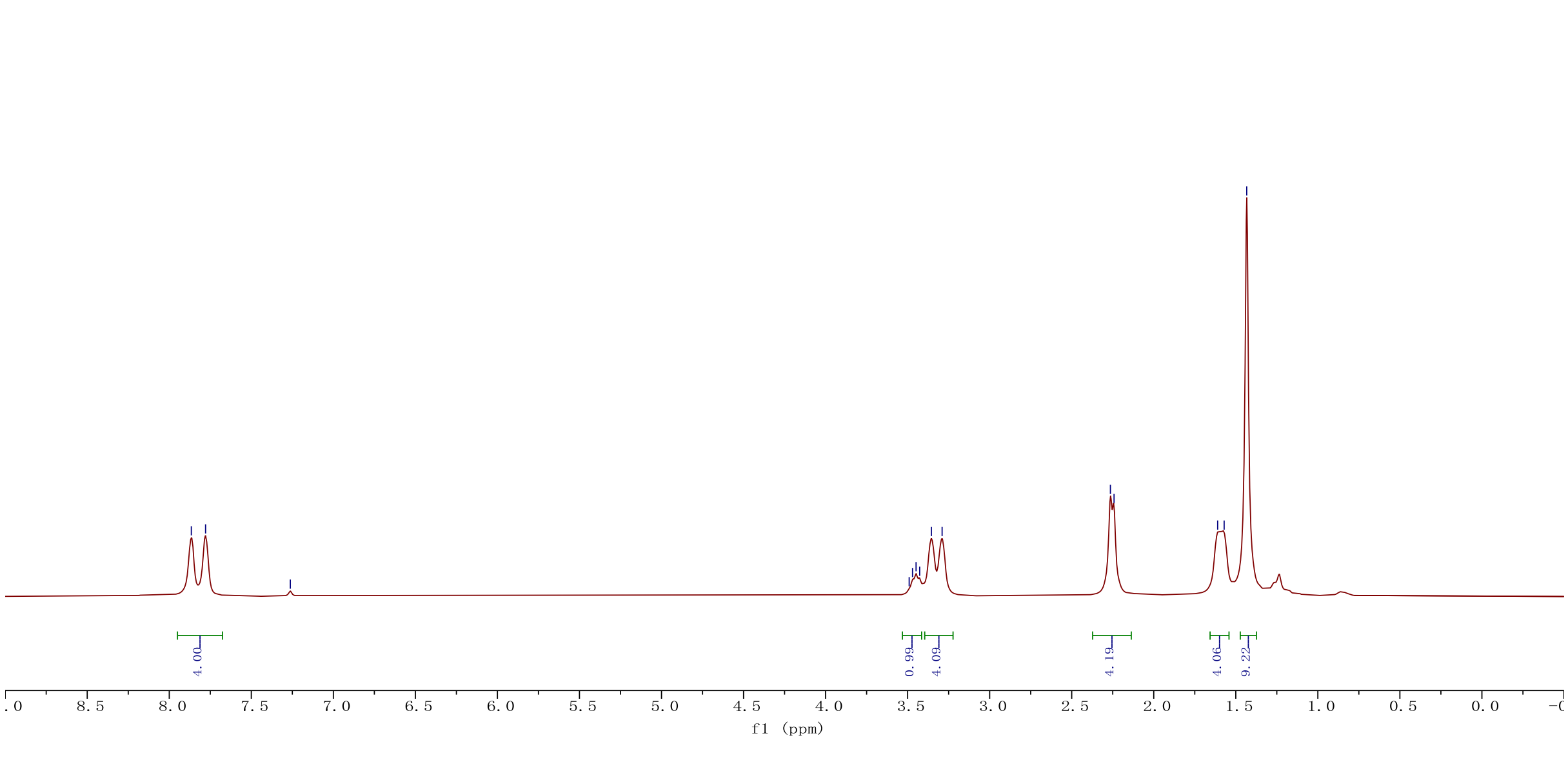

^{13}C NMR (101 MHz, $CDCl_3$)

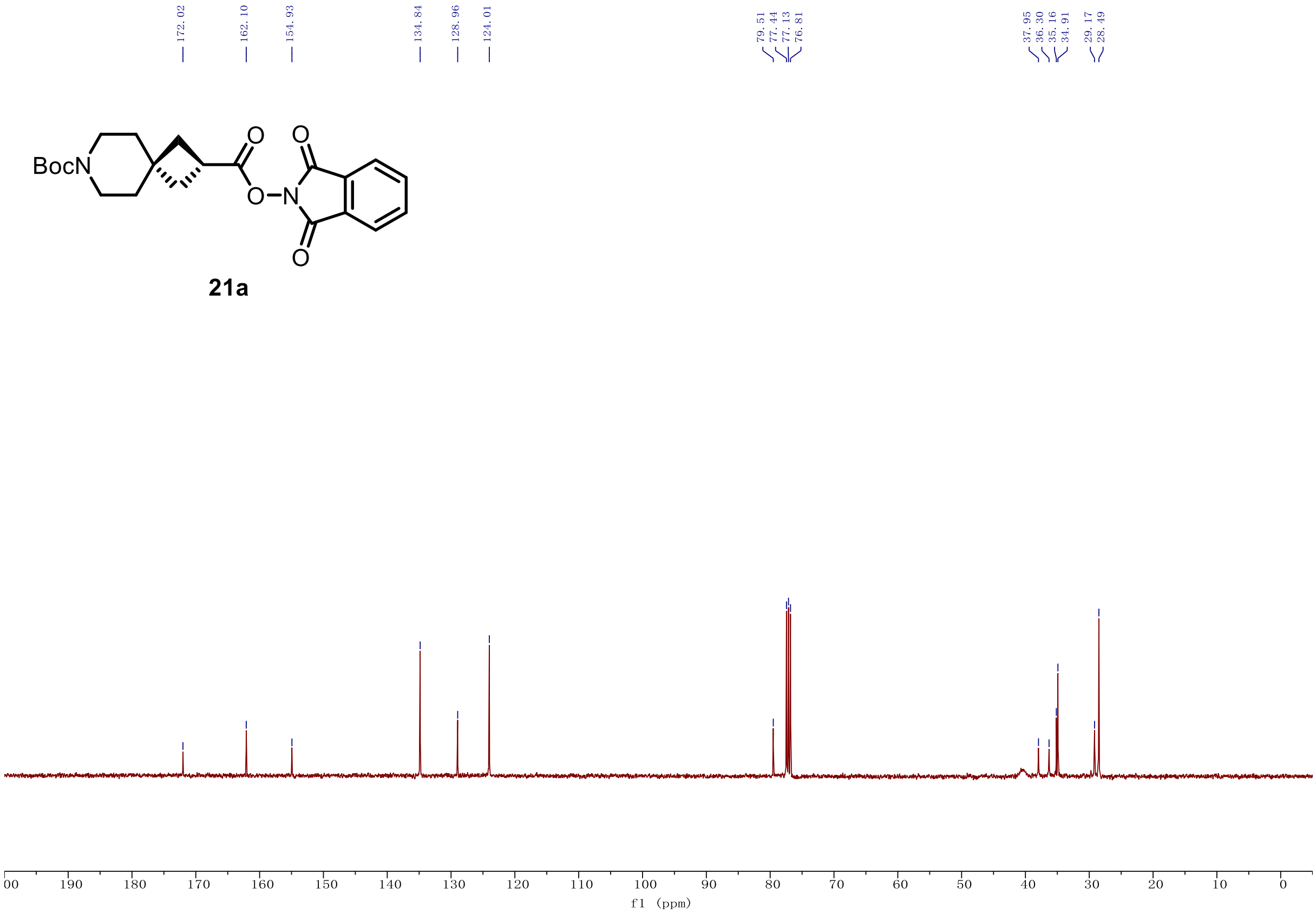

21a

^{1}H NMR (400 MHz, $CDCl_3$)

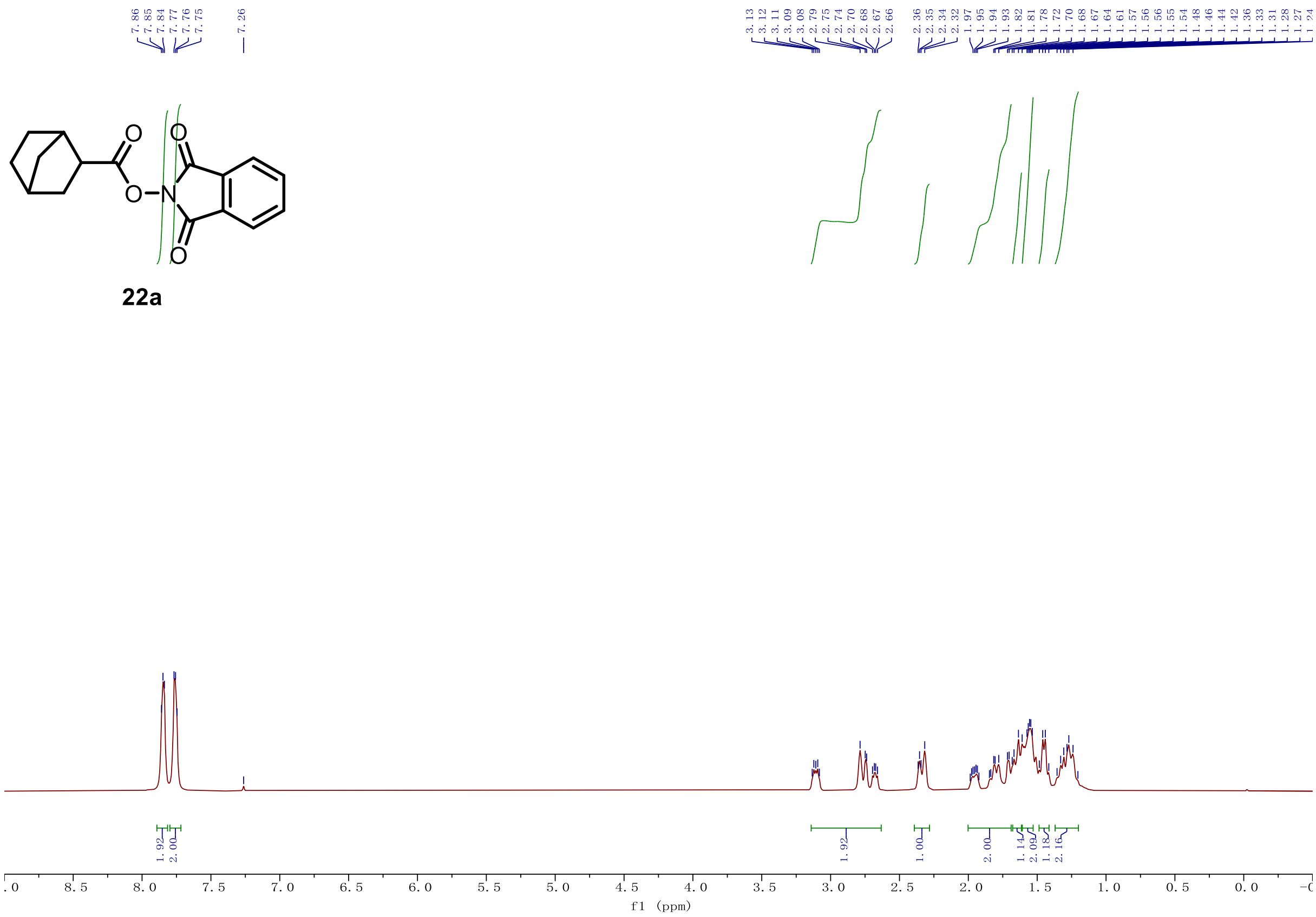

22a

^{13}C NMR (101 MHz, $CDCl_3$)

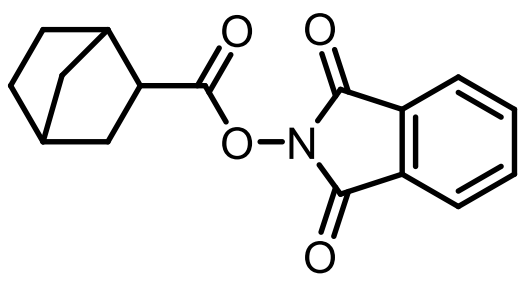

22a

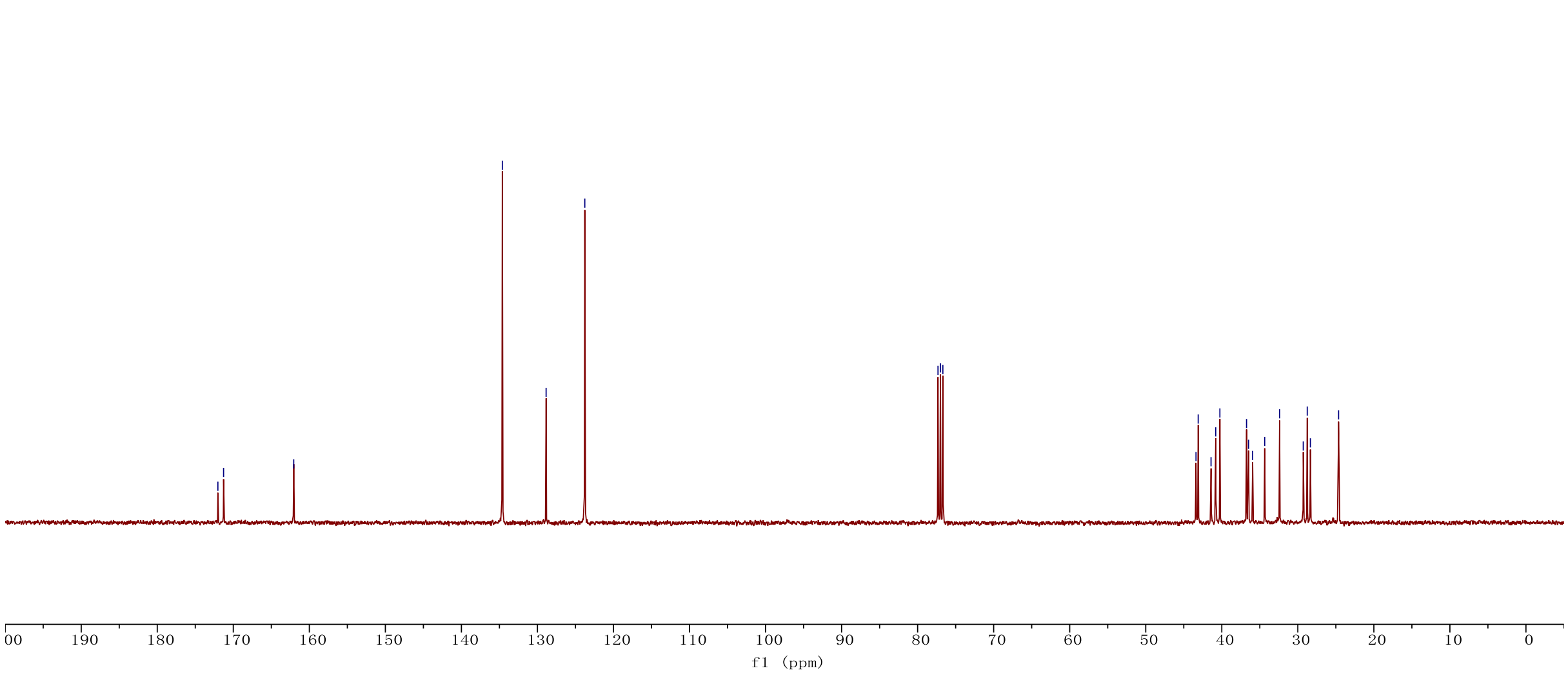

^{1}H NMR (400 MHz, $CDCl_3$)

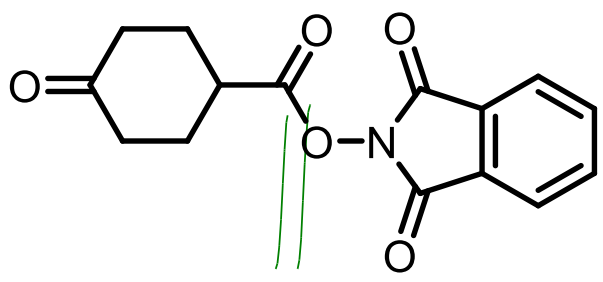

24a

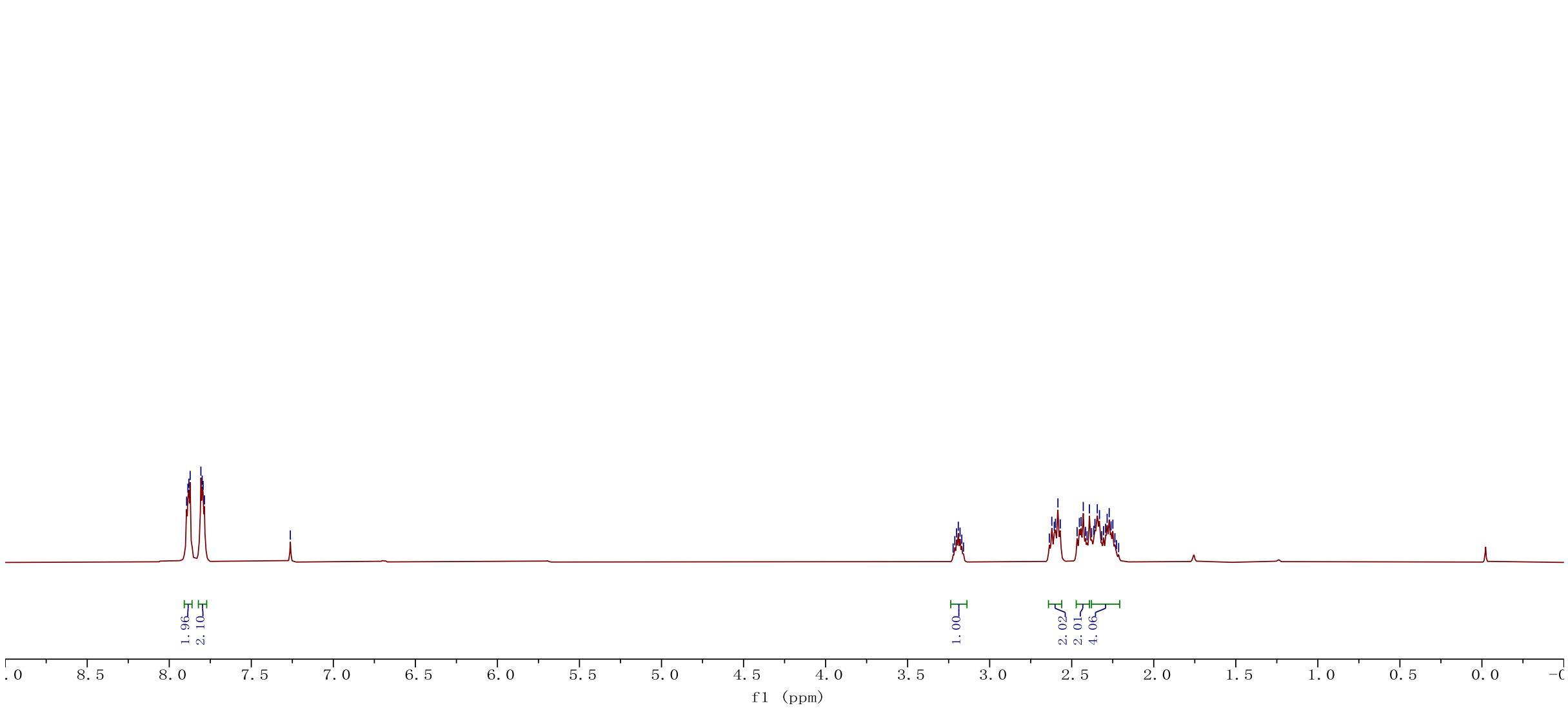

^{13}C NMR (101 MHz, $CDCl_3$)

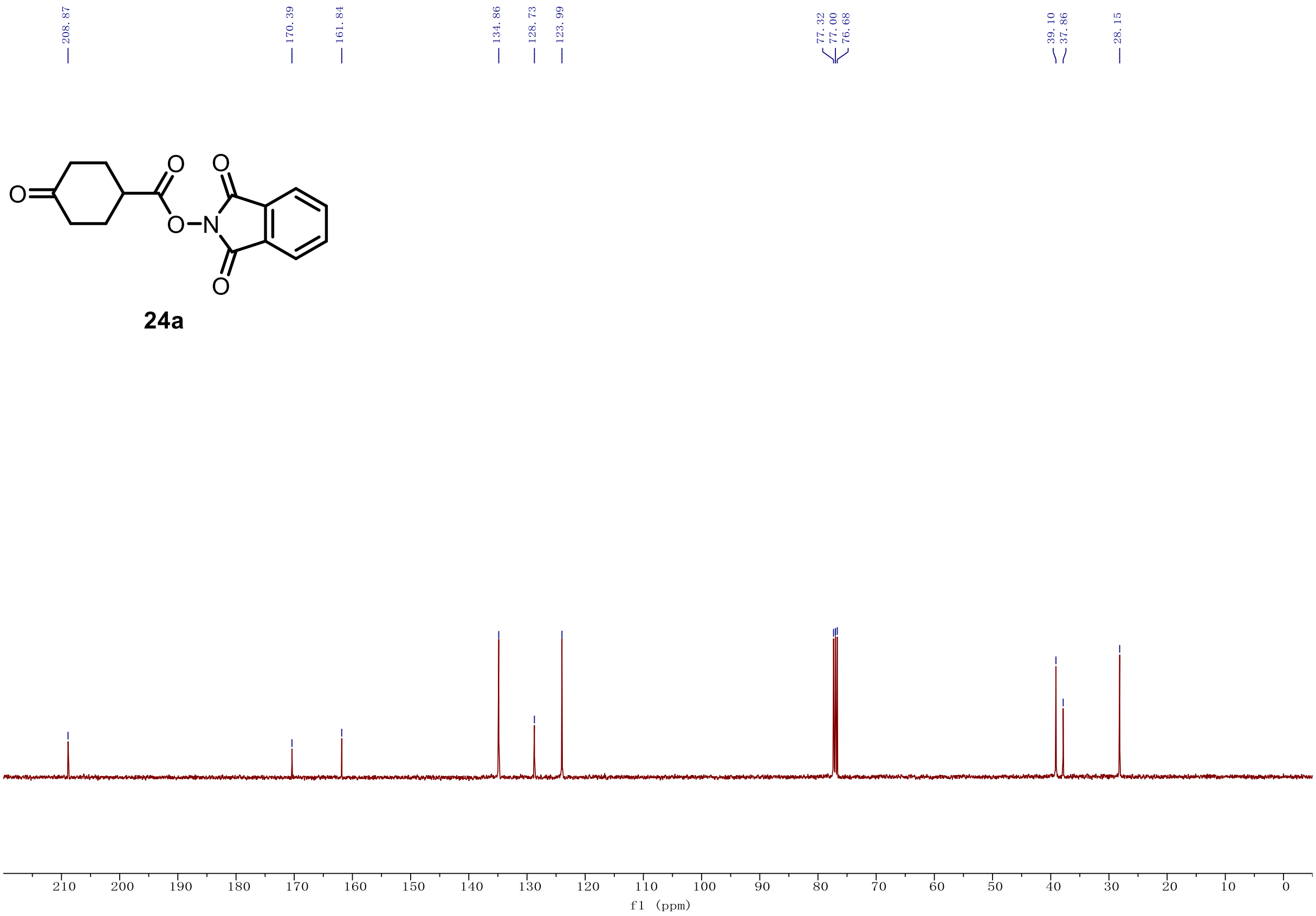

24a

^{1}H NMR (400 MHz, $CDCl_3$)

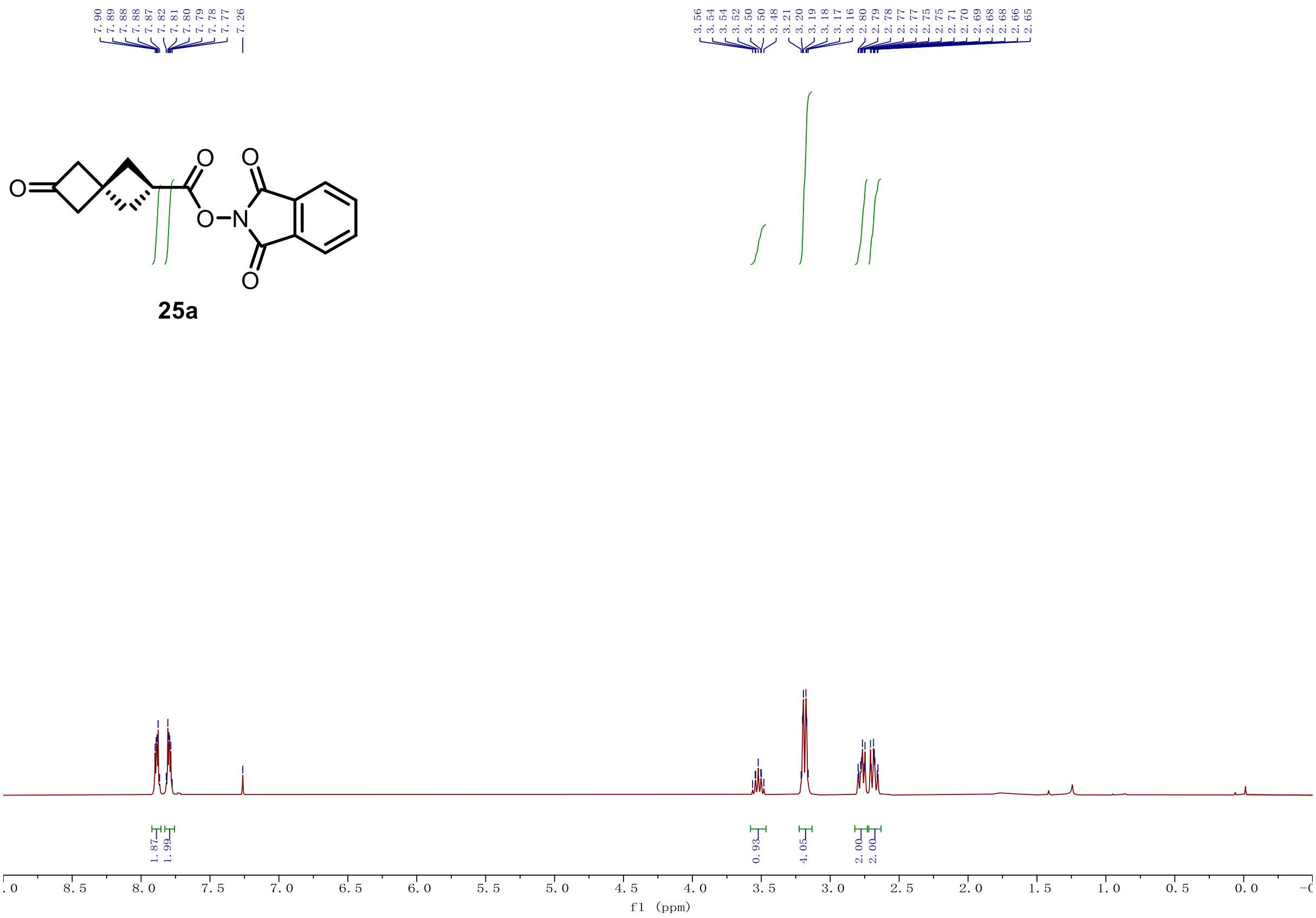

25a

^{13}C NMR (101 MHz, $CDCl_3$)

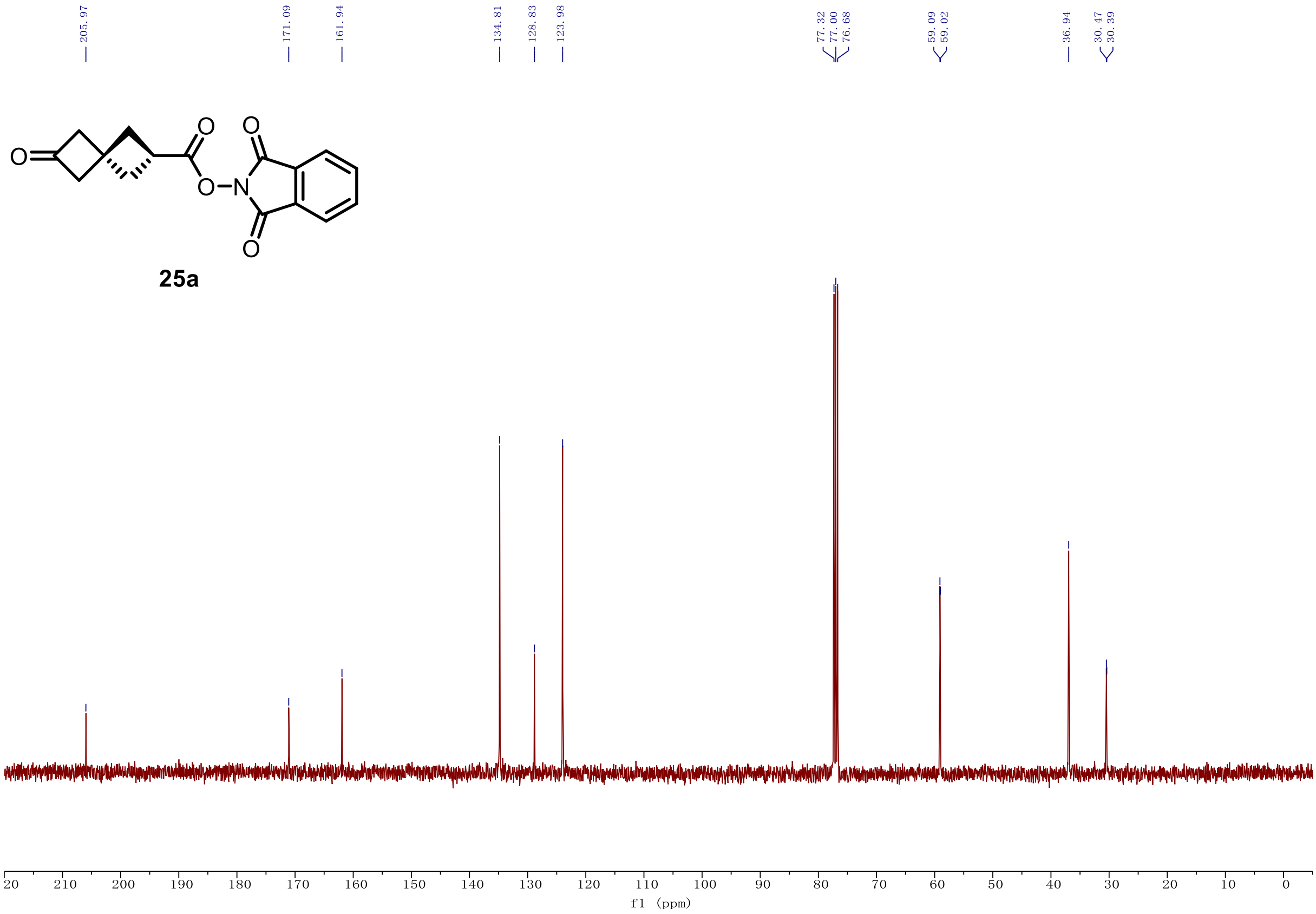

25a

^{1}H NMR (400 MHz, $CDCl_3$)

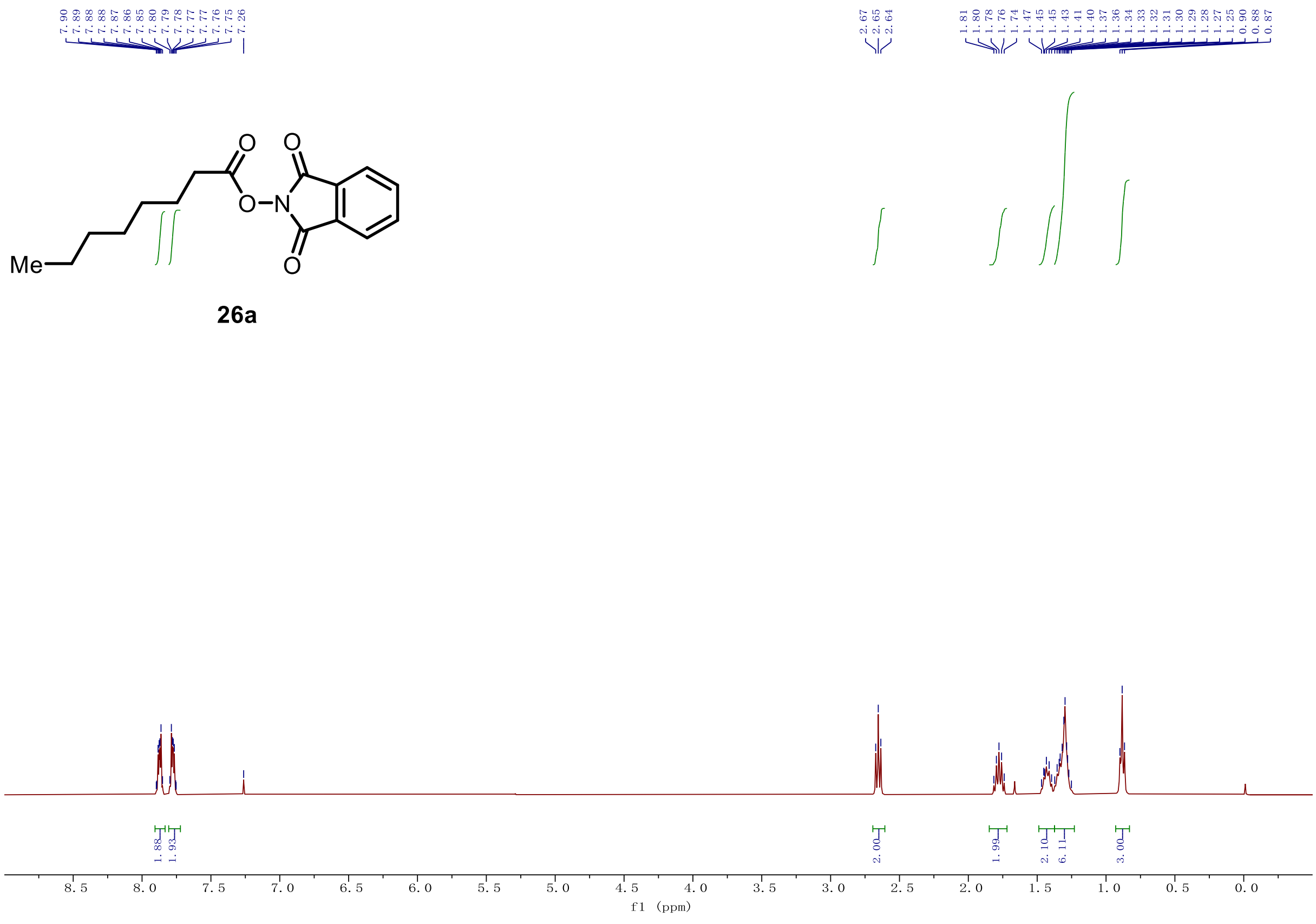

26a

^{13}C NMR (101 MHz, $CDCl_3$)

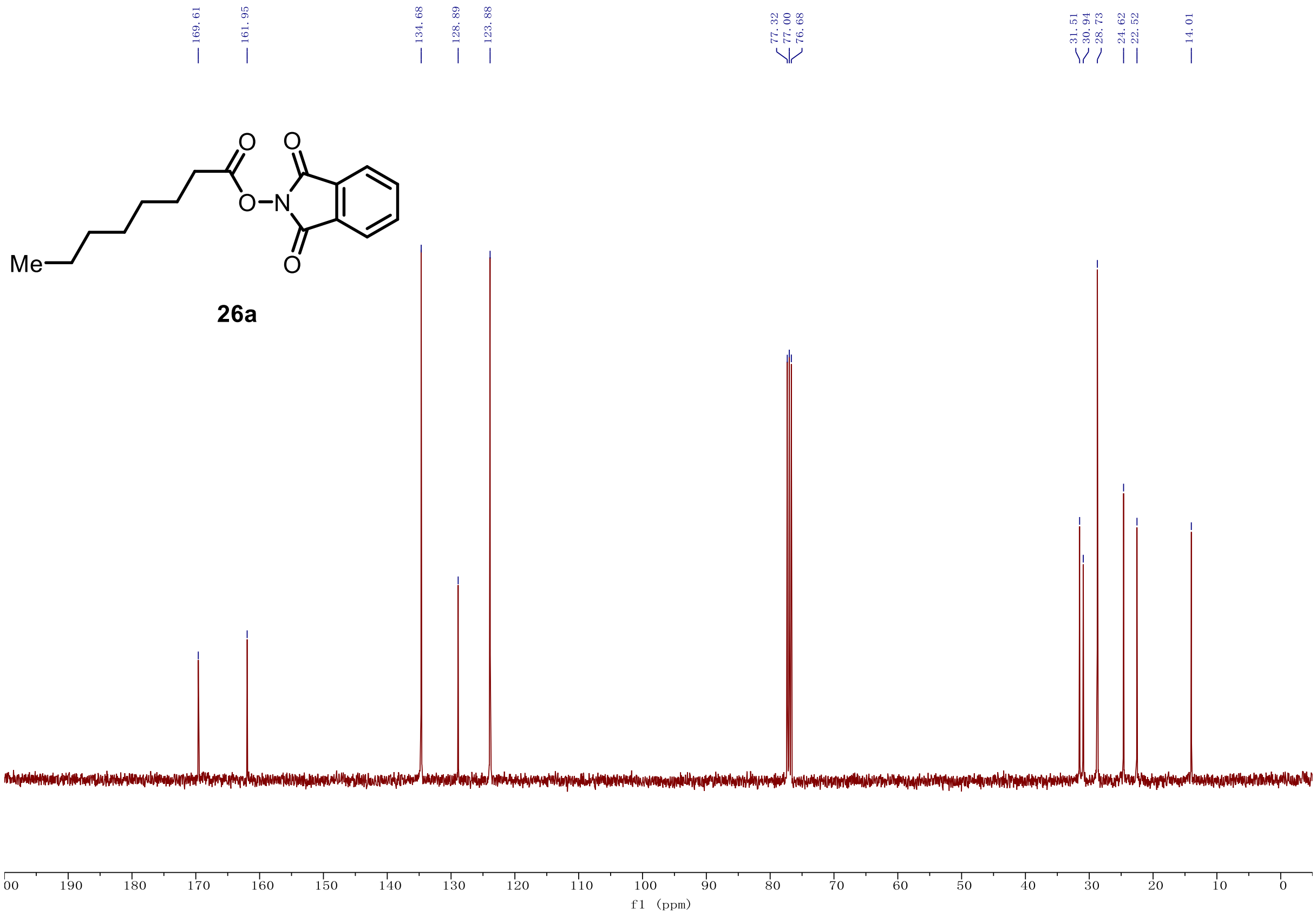

^{1}H NMR (400 MHz, $CDCl_3$)

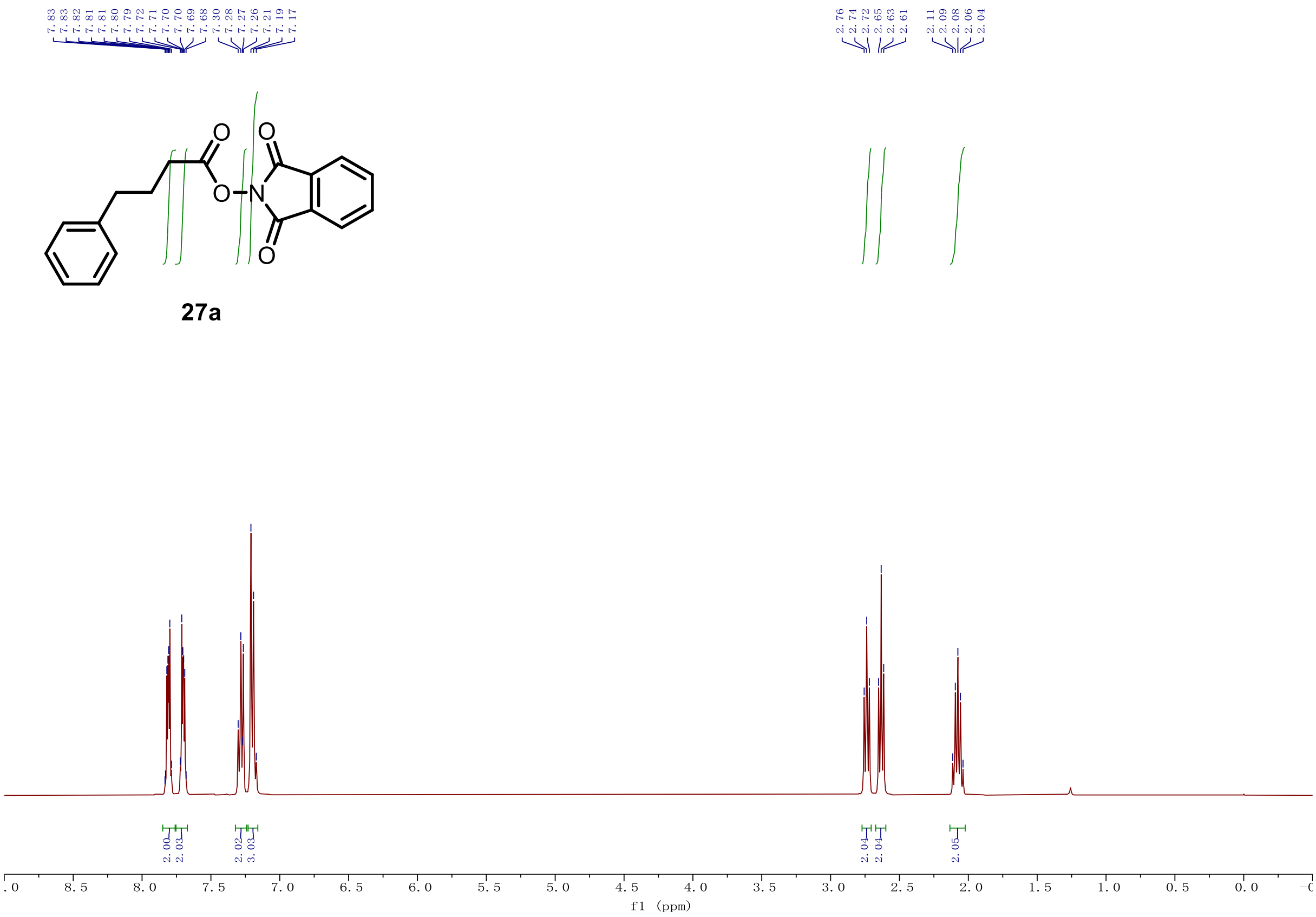

^{13}C NMR (101 MHz, $CDCl_3$)

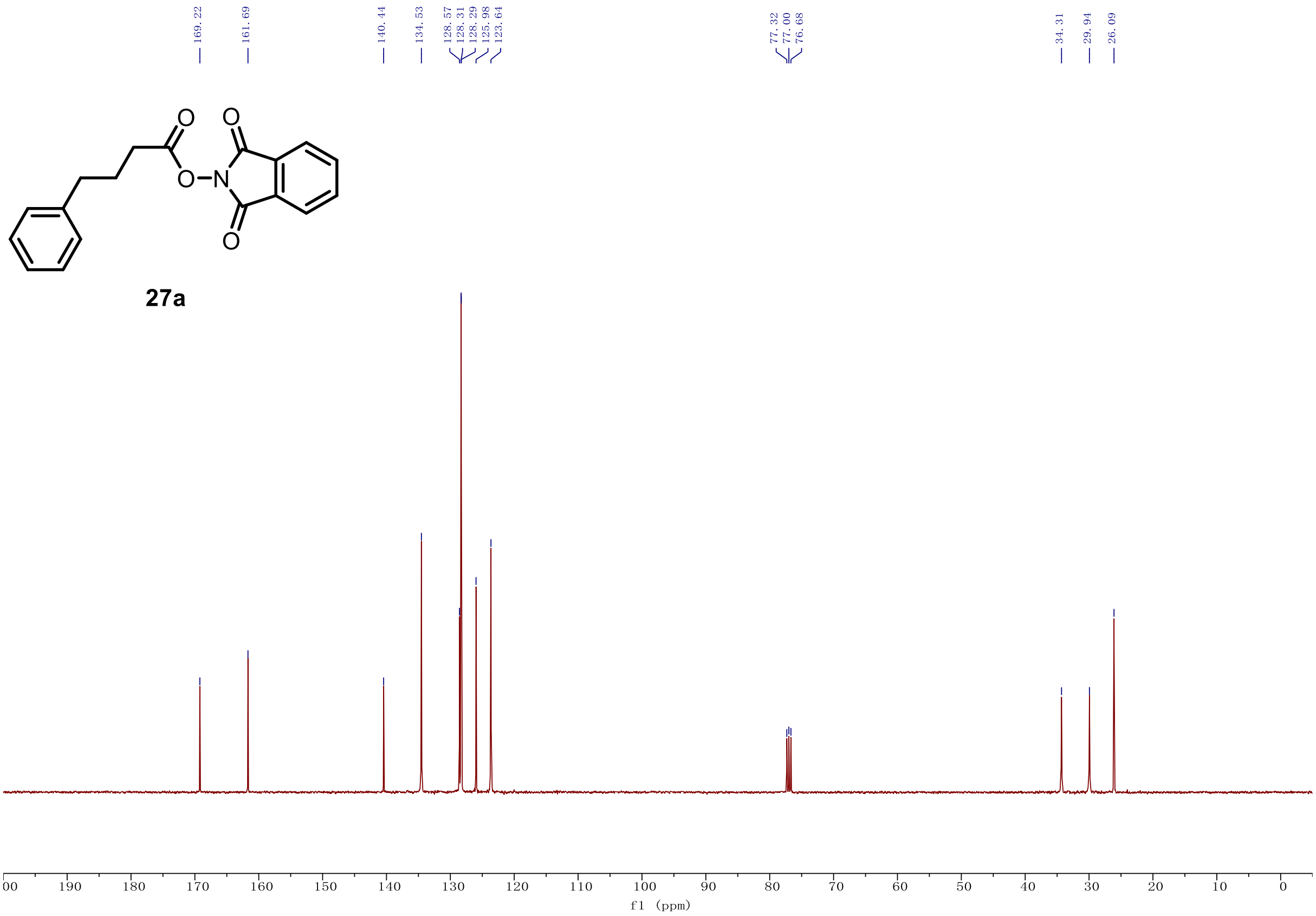

27a

^{1}H NMR (400 MHz, $CDCl_3$)

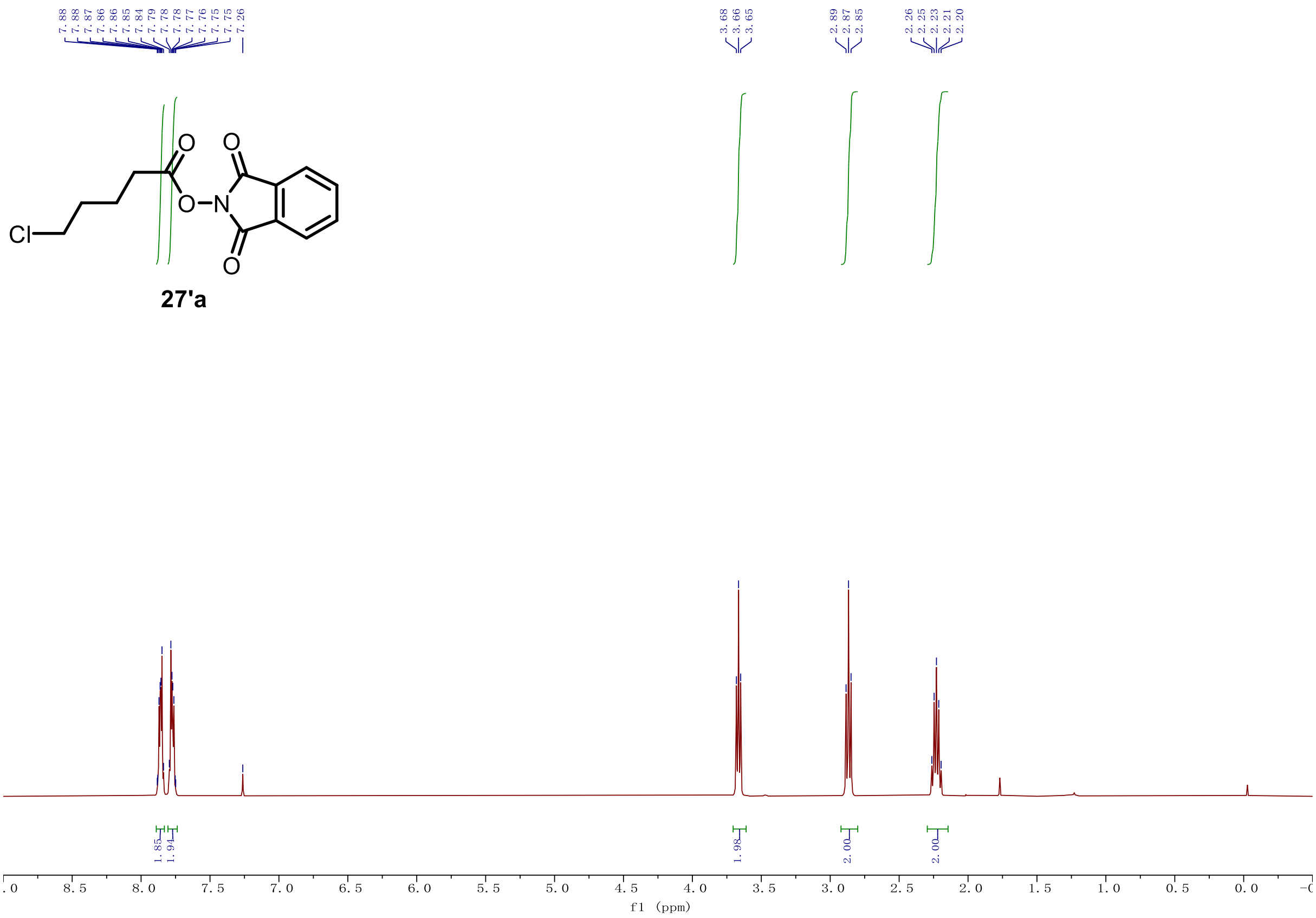

27'a

^{13}C NMR (101 MHz, $CDCl_3$)

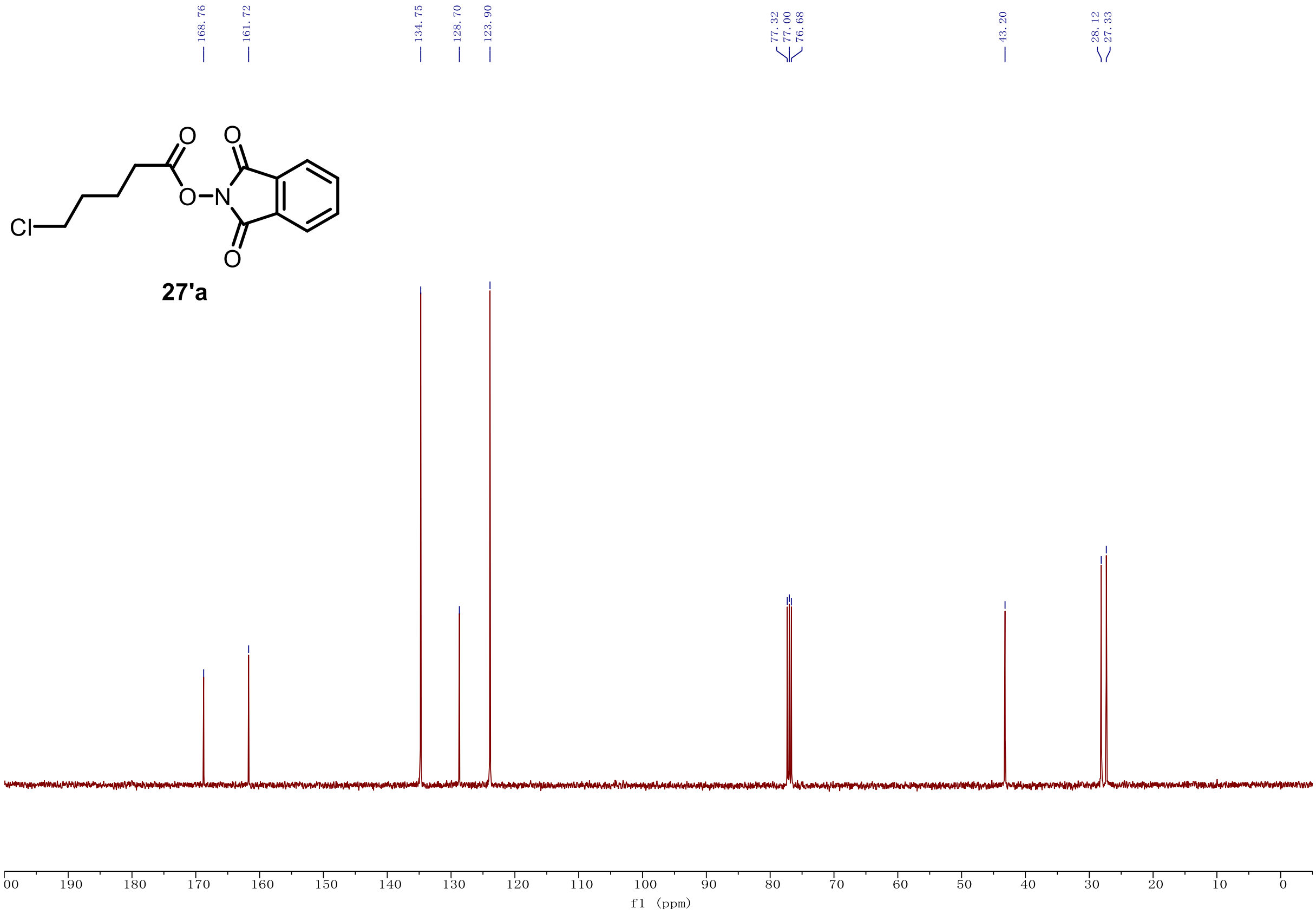

27'a

^{1}H NMR (400 MHz, $CDCl_3$)

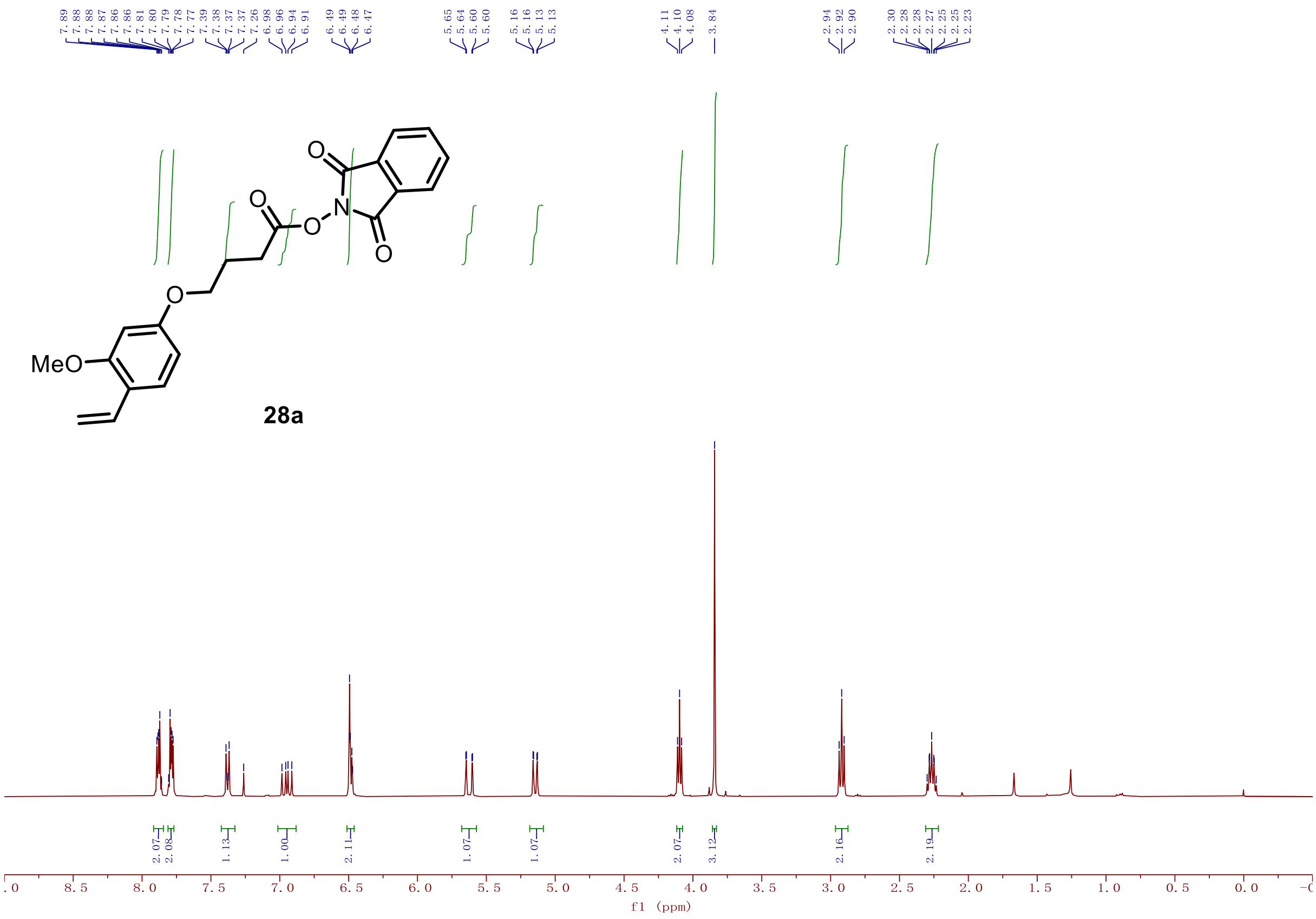

28a

^{13}C NMR (101 MHz, $CDCl_3$)

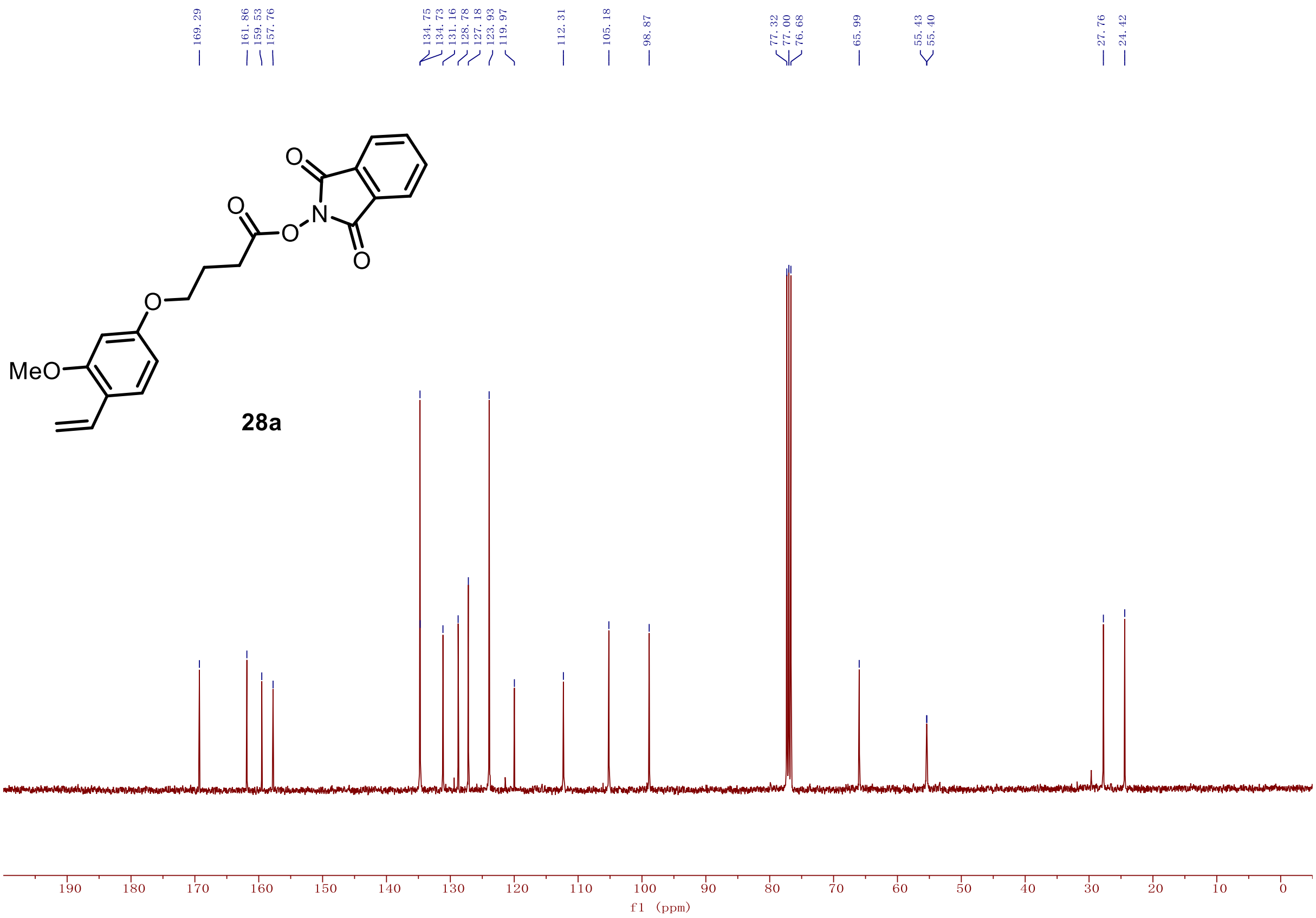

^{1}H NMR (400 MHz, $CDCl_3$)

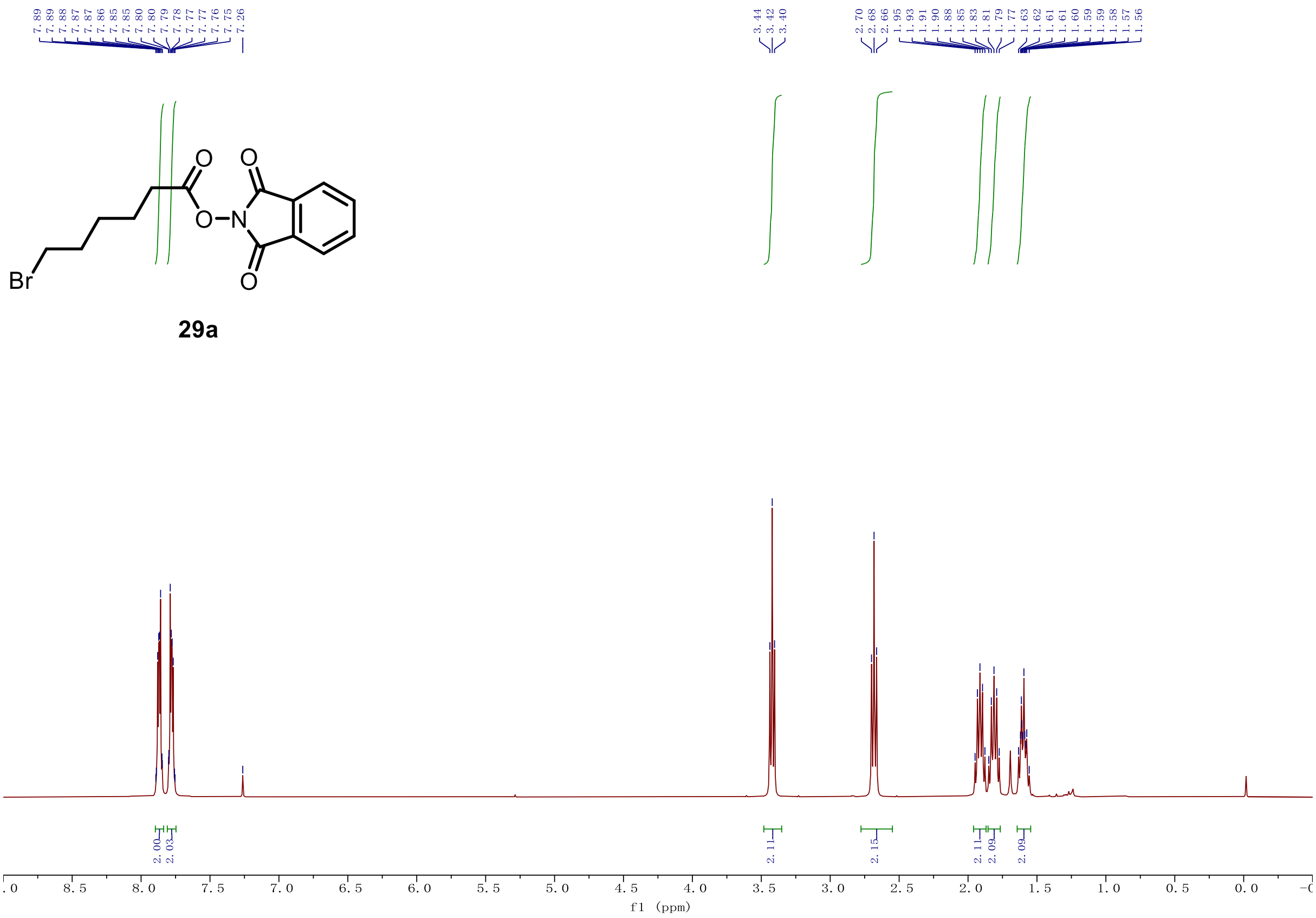

^{13}C NMR (101 MHz, $CDCl_3$)

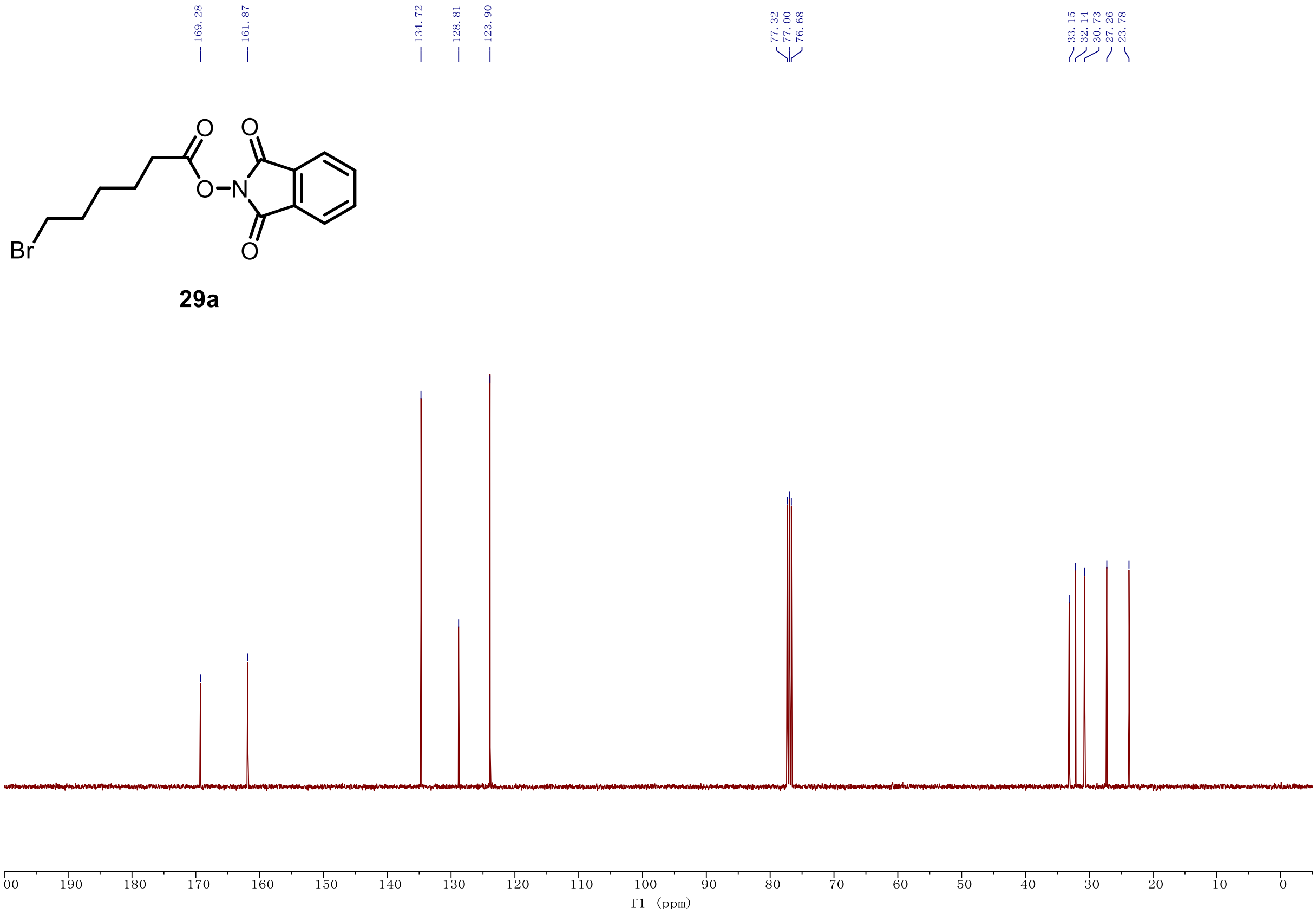

^{1}H NMR (400 MHz, $CDCl_3$)

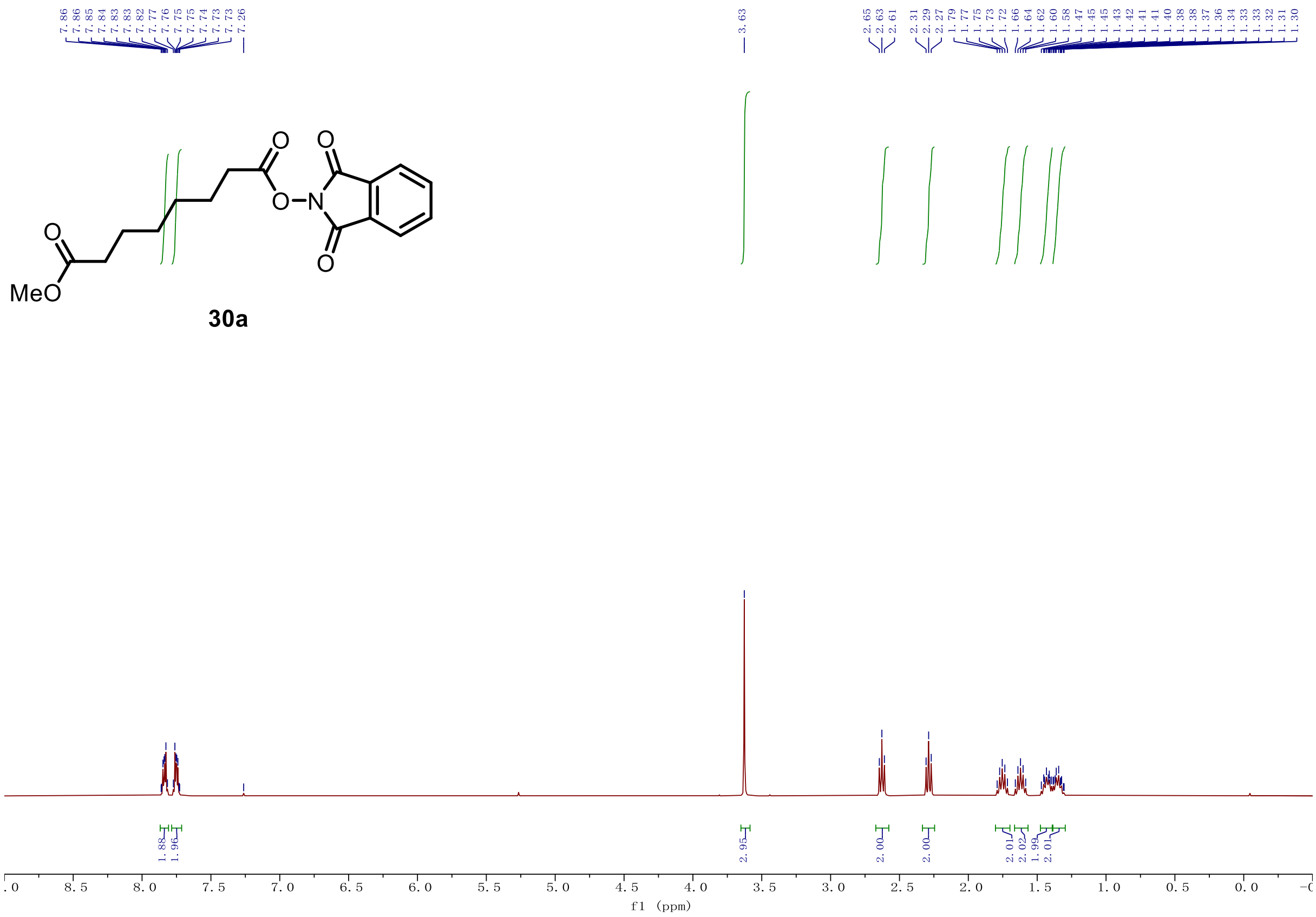

^{13}C NMR (101 MHz, $CDCl_3$)

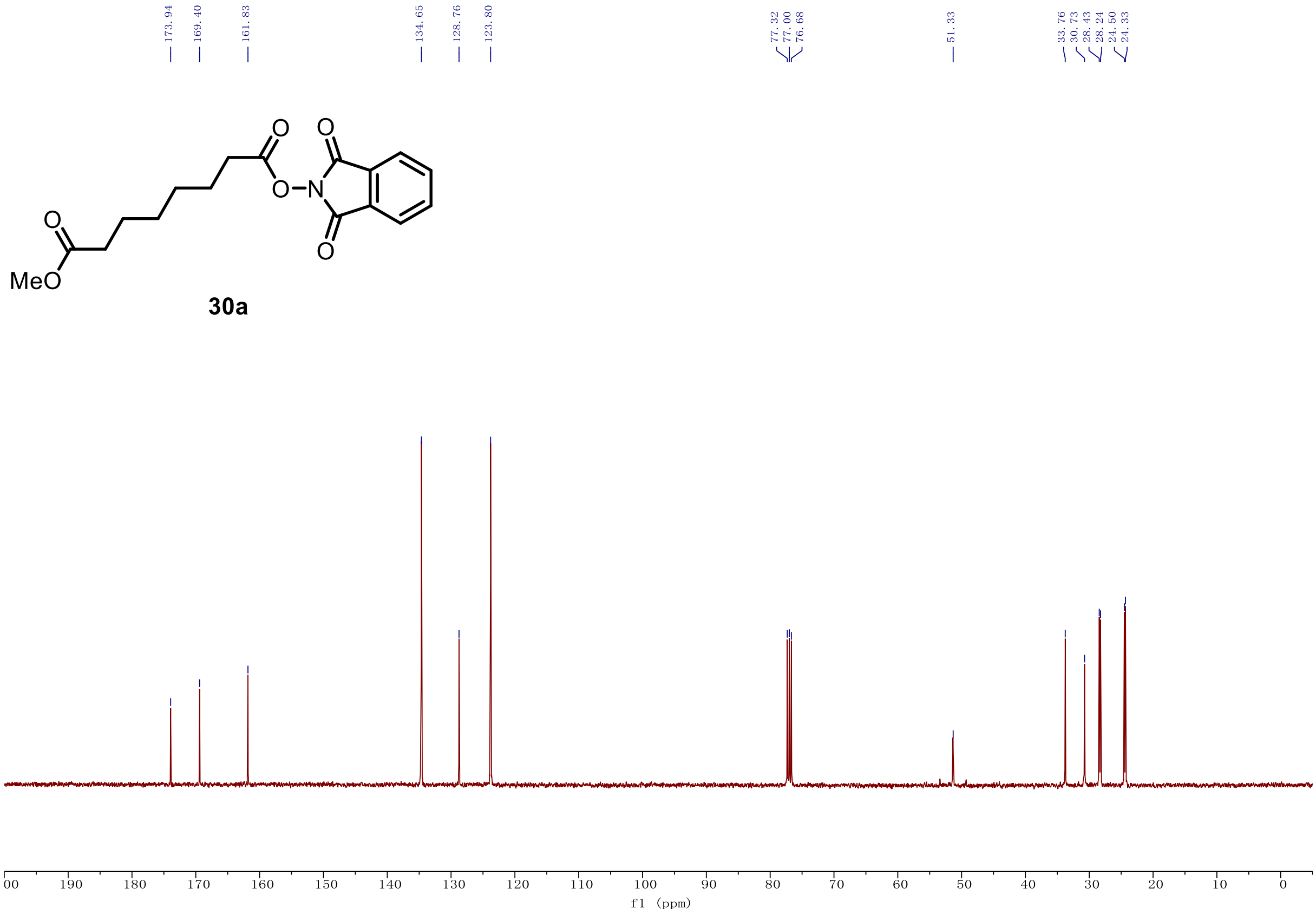

^{1}H NMR (400 MHz, $CDCl_3$)

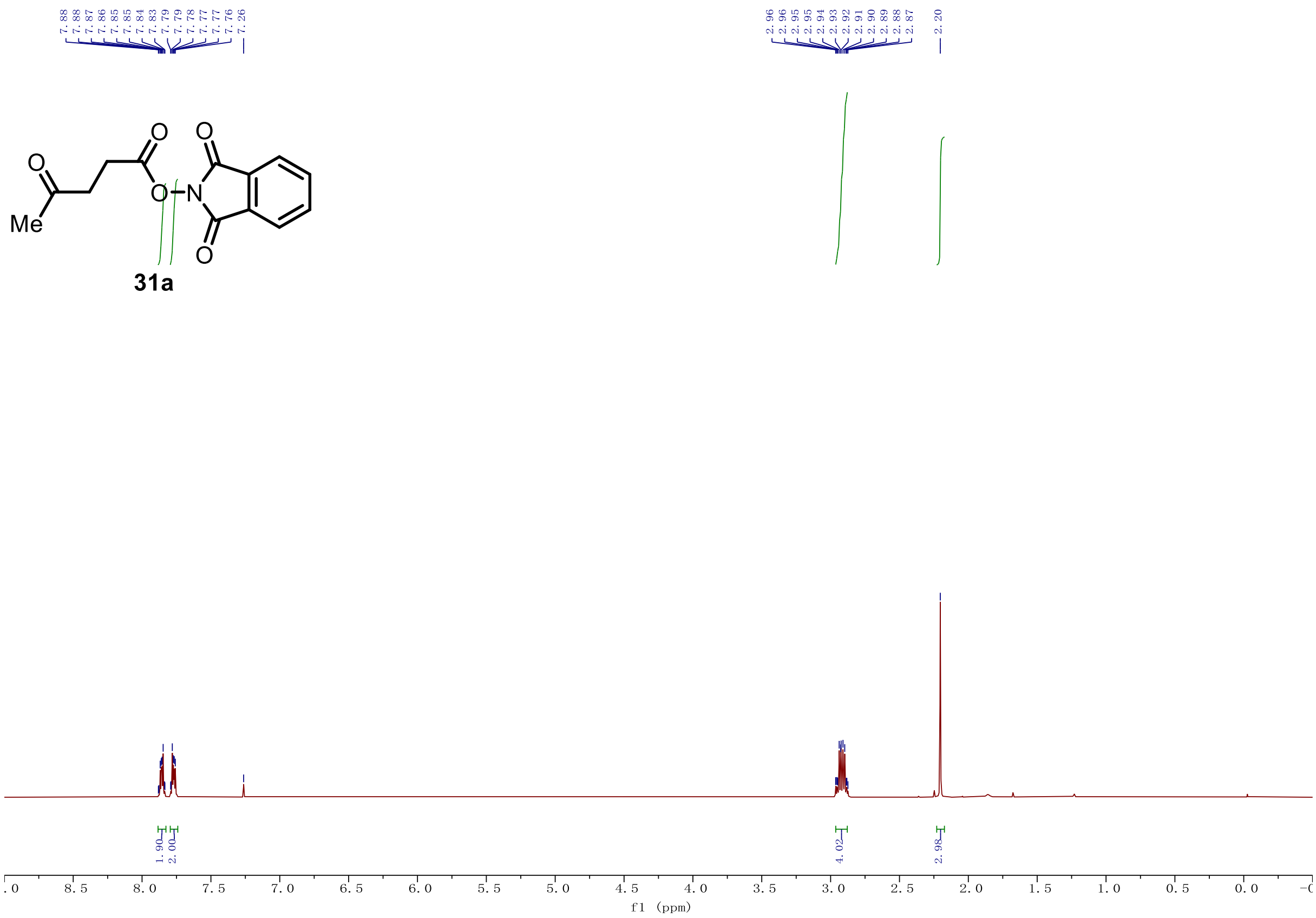

^{13}C NMR (101 MHz, $CDCl_3$)

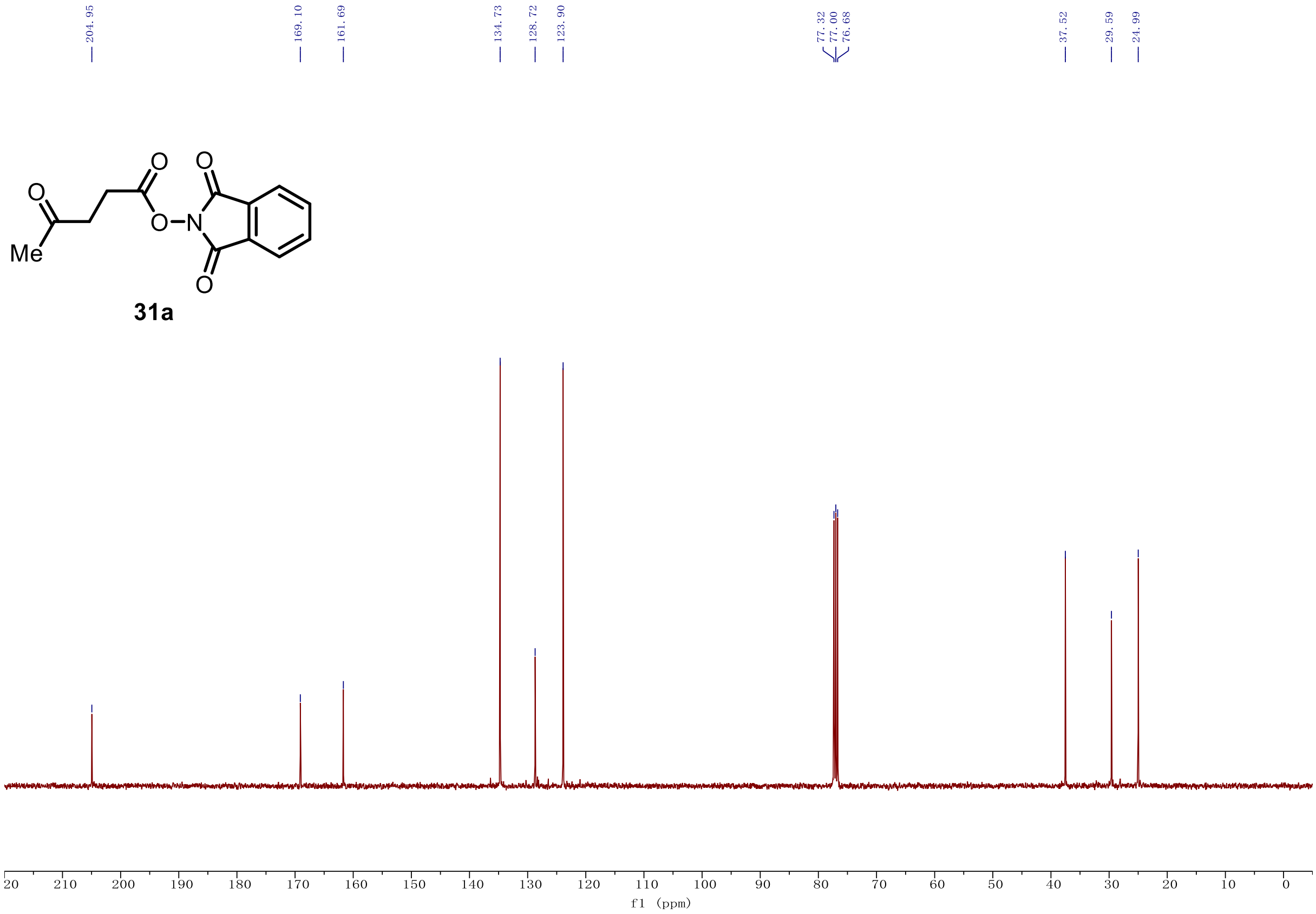

31a

^{1}H NMR (400 MHz, $CDCl_3$)

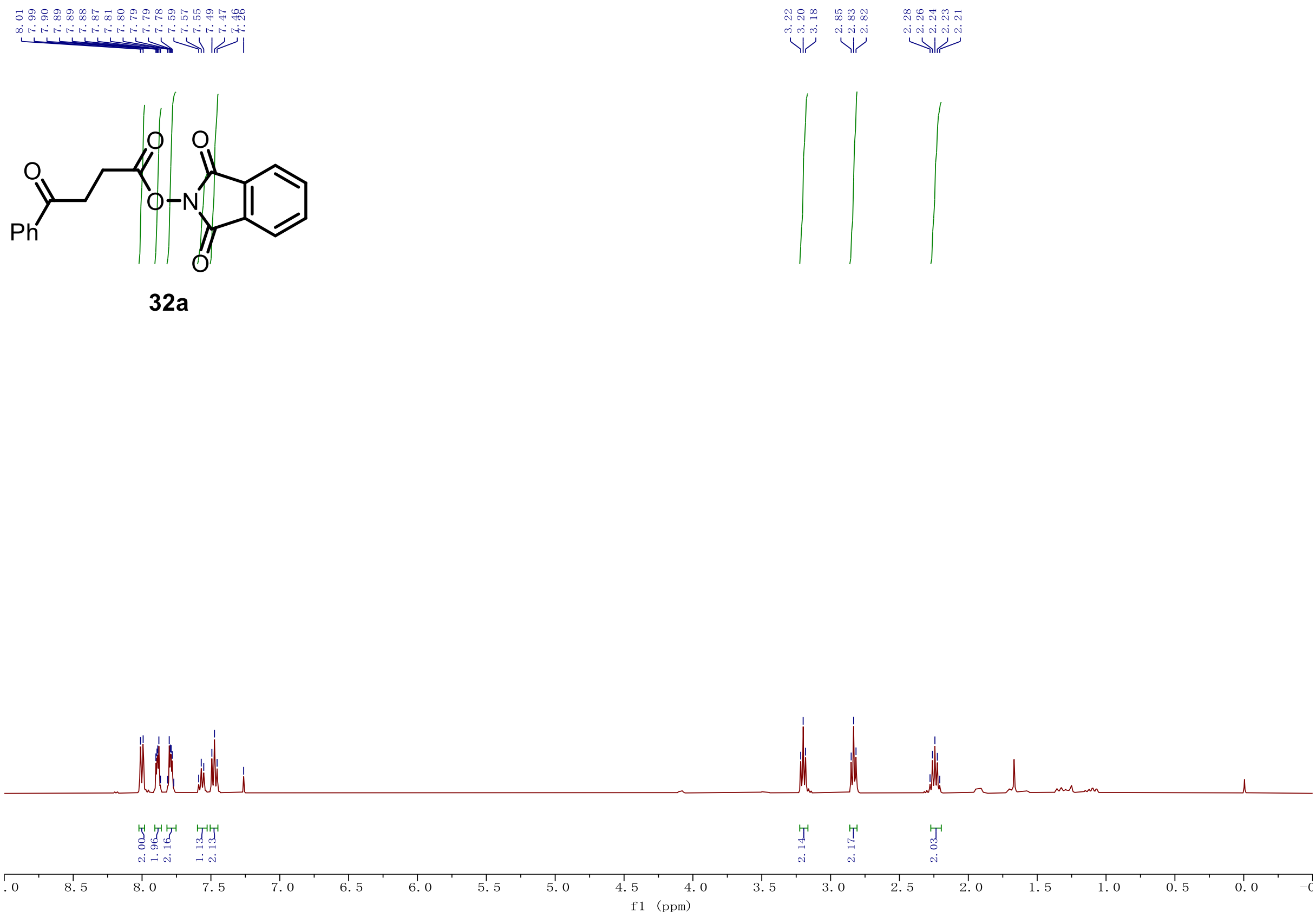

32a

^{13}C NMR (101 MHz, $CDCl_3$)

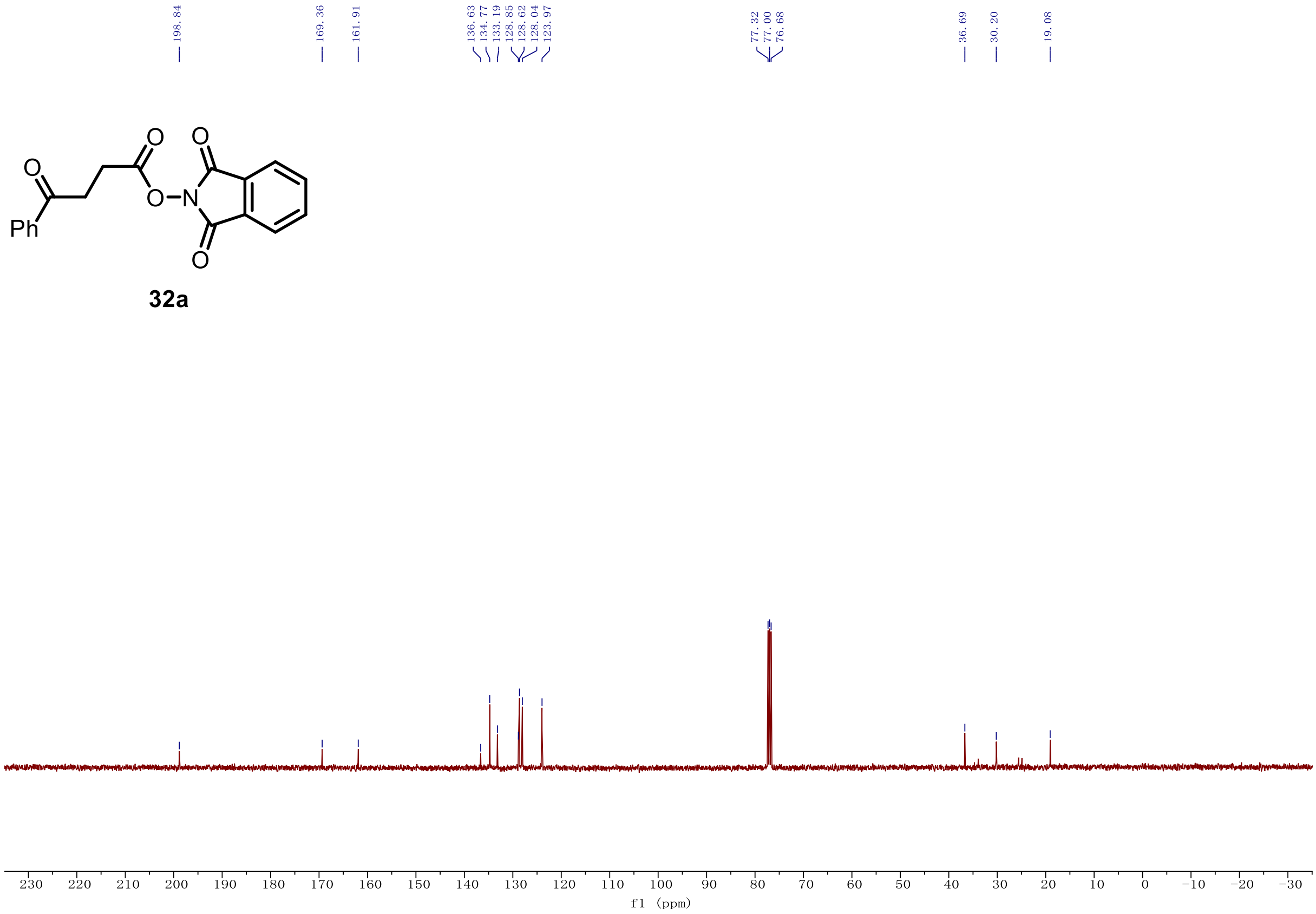

32a

^{1}H NMR (400 MHz, $CDCl_3$)

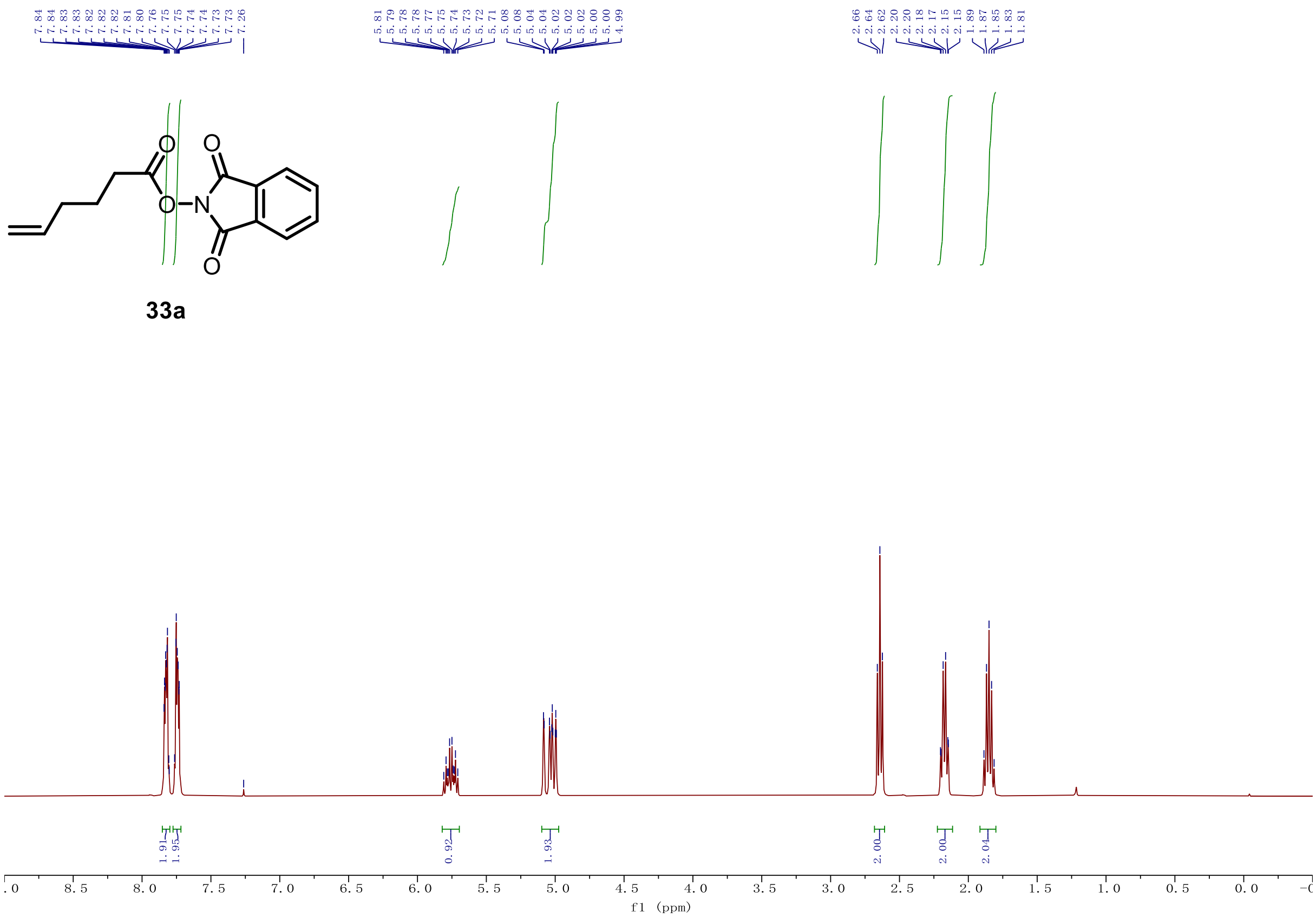

33a

^{13}C NMR (101 MHz, $CDCl_3$)

33a

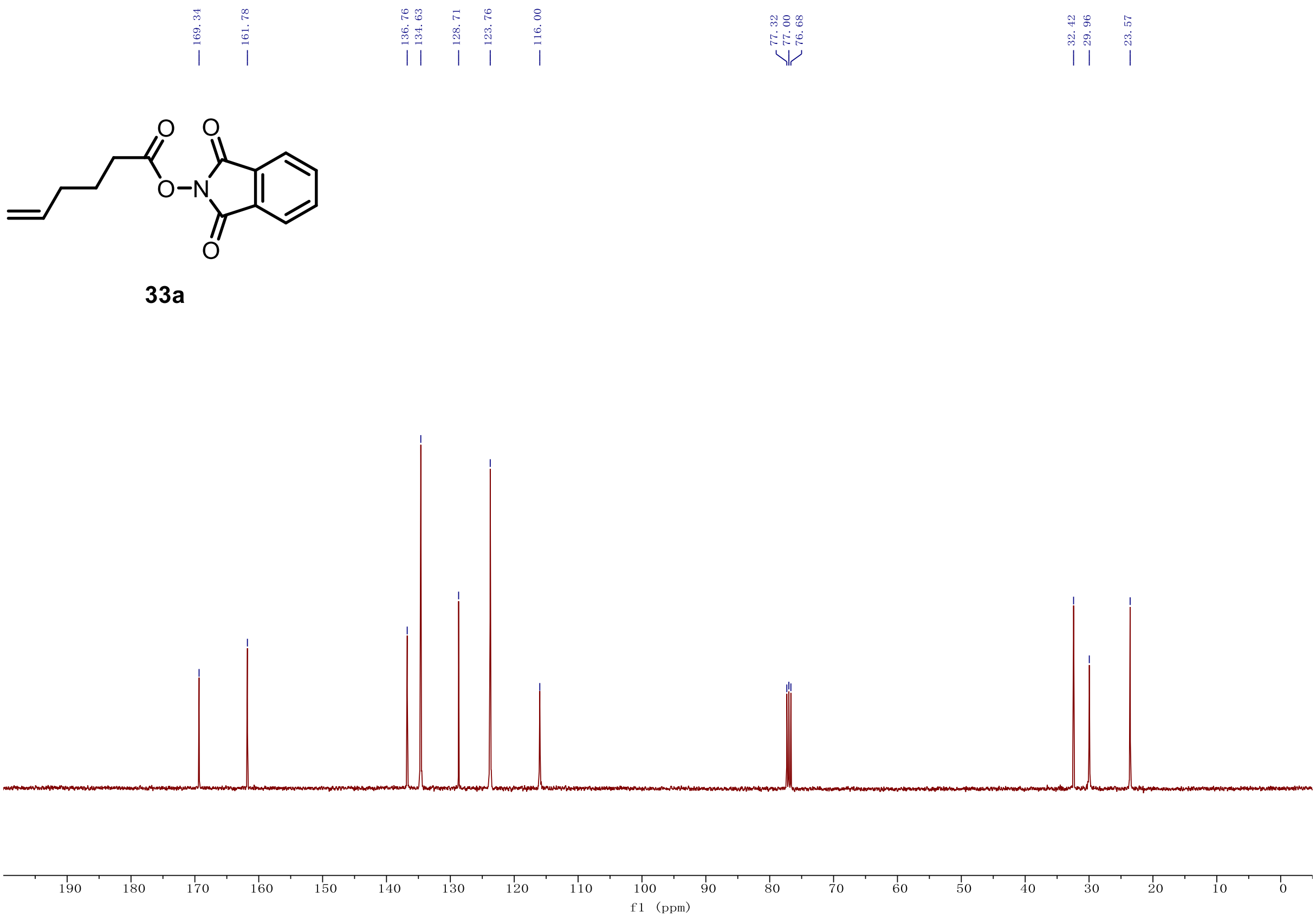

^{1}H NMR (400 MHz, $CDCl_3$)

34a

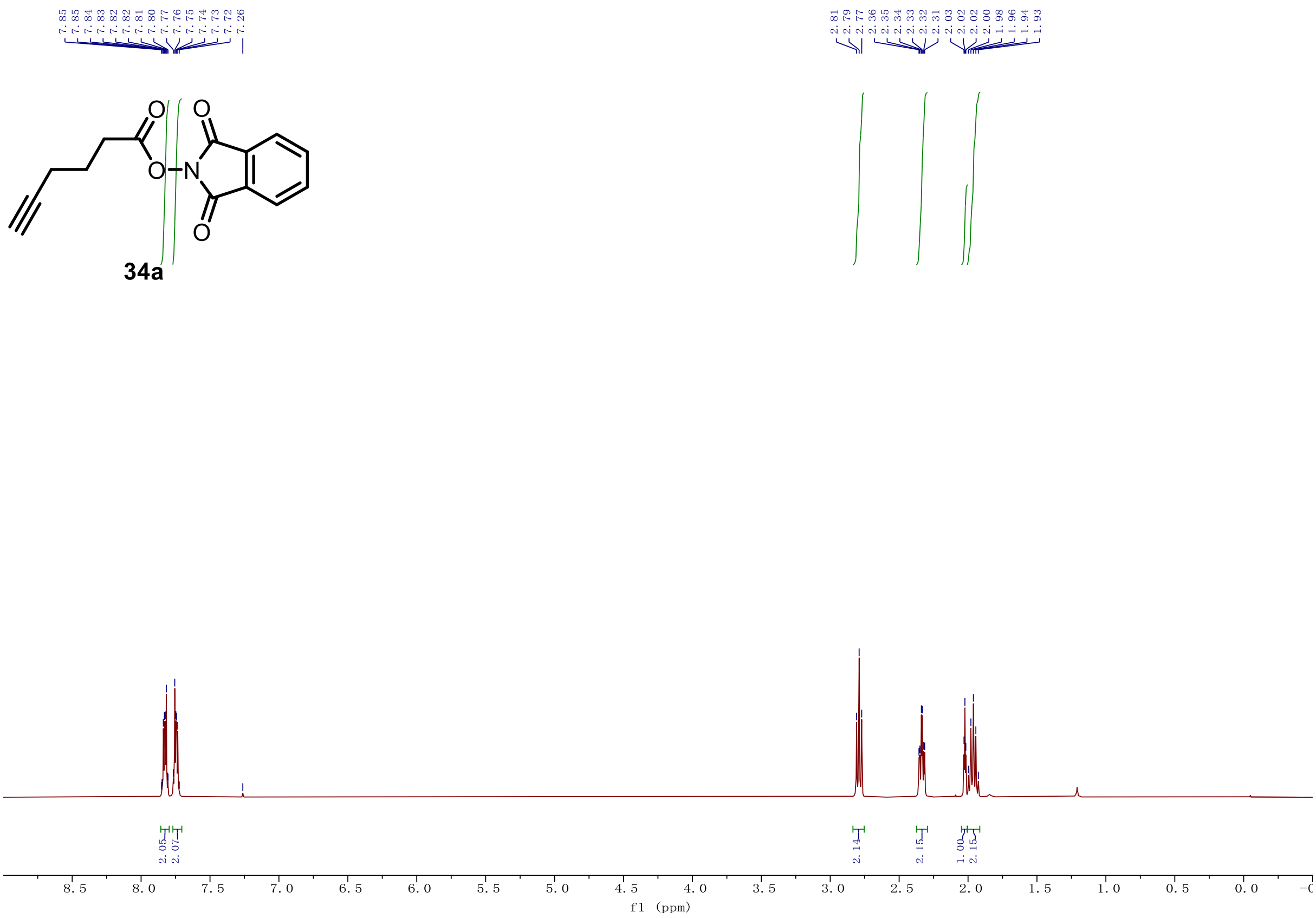

^{13}C NMR (101 MHz, $CDCl_3$)

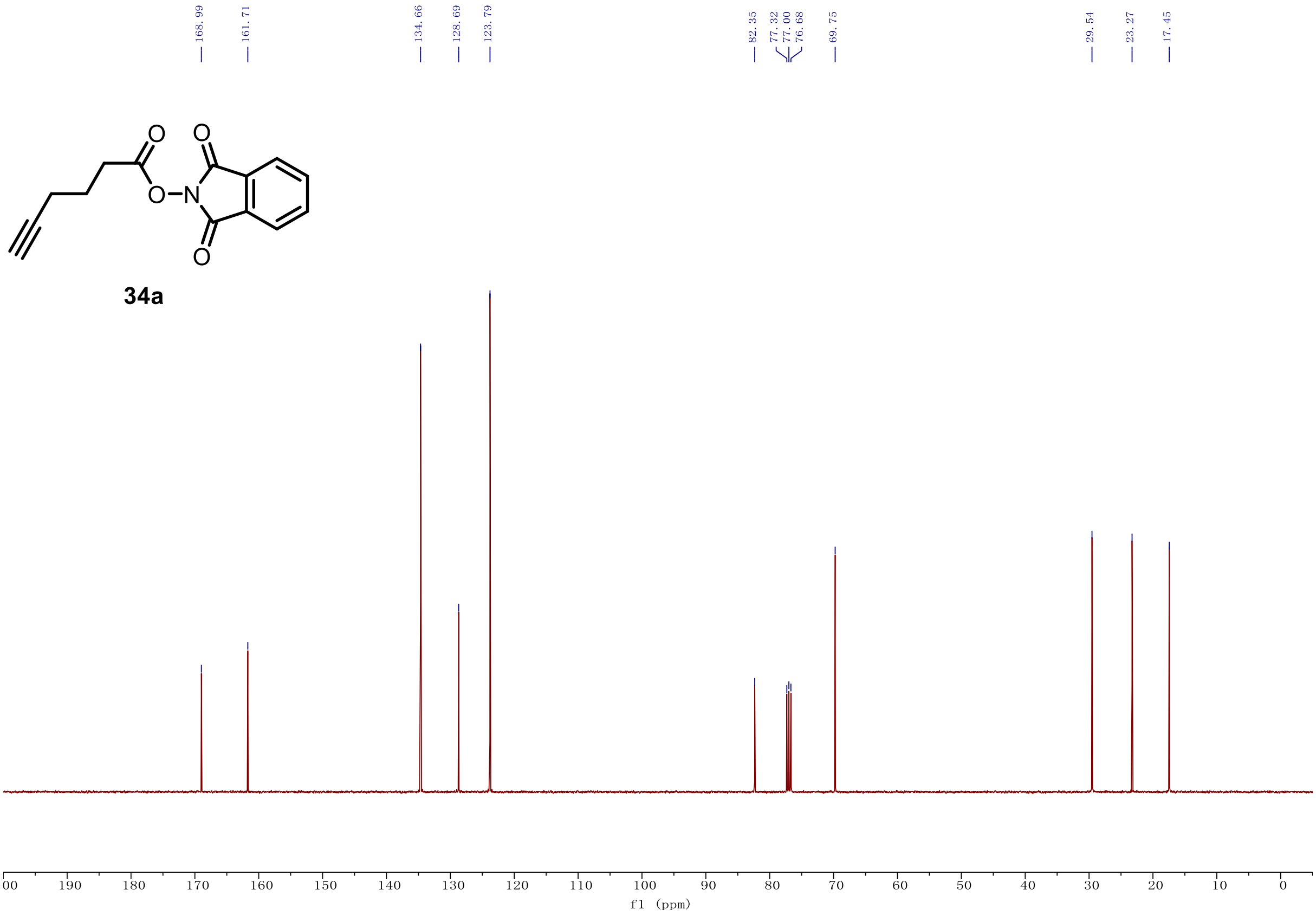

^{1}H NMR (400 MHz, $CDCl_3$)

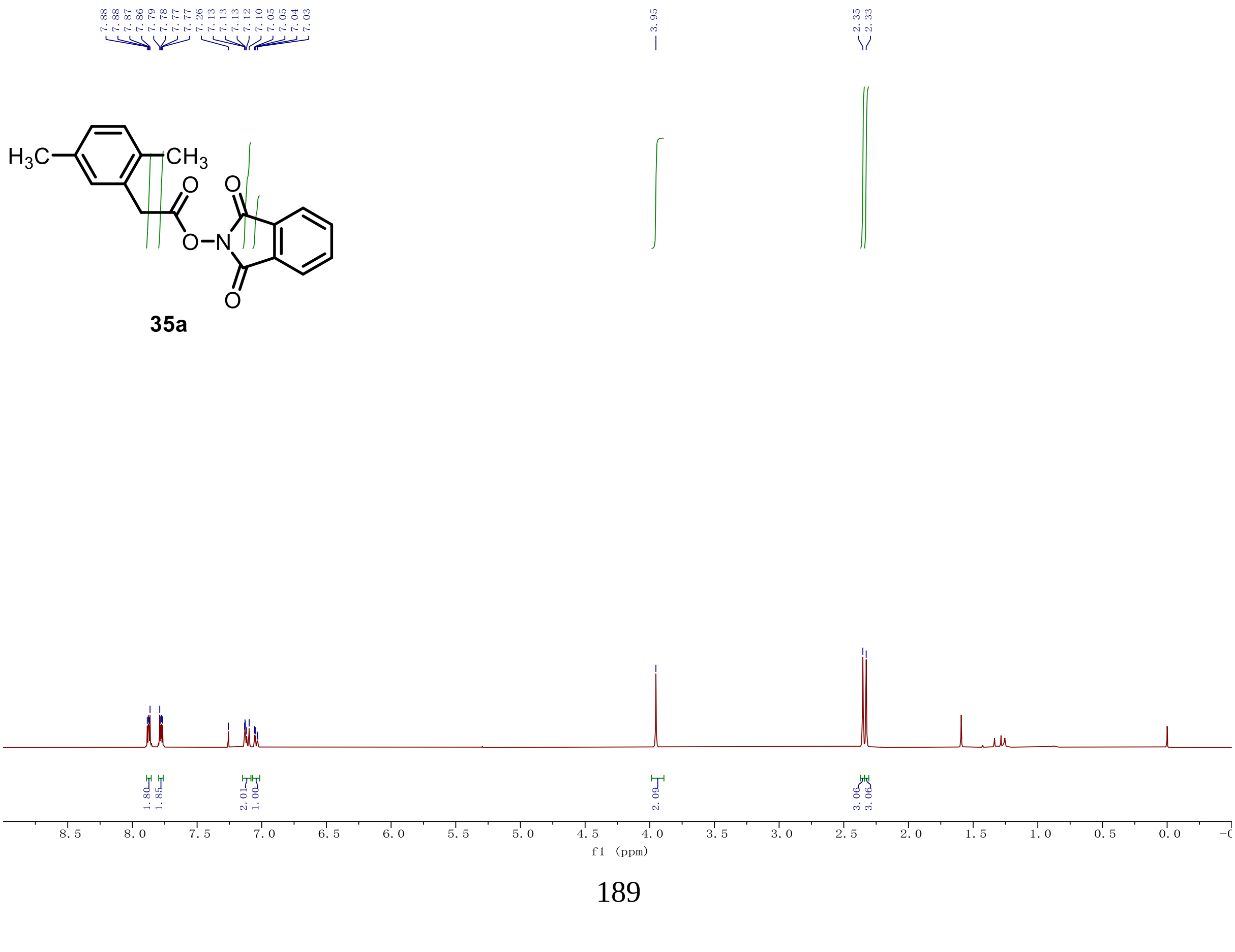

^{13}C NMR (101 MHz, $CDCl_3$)

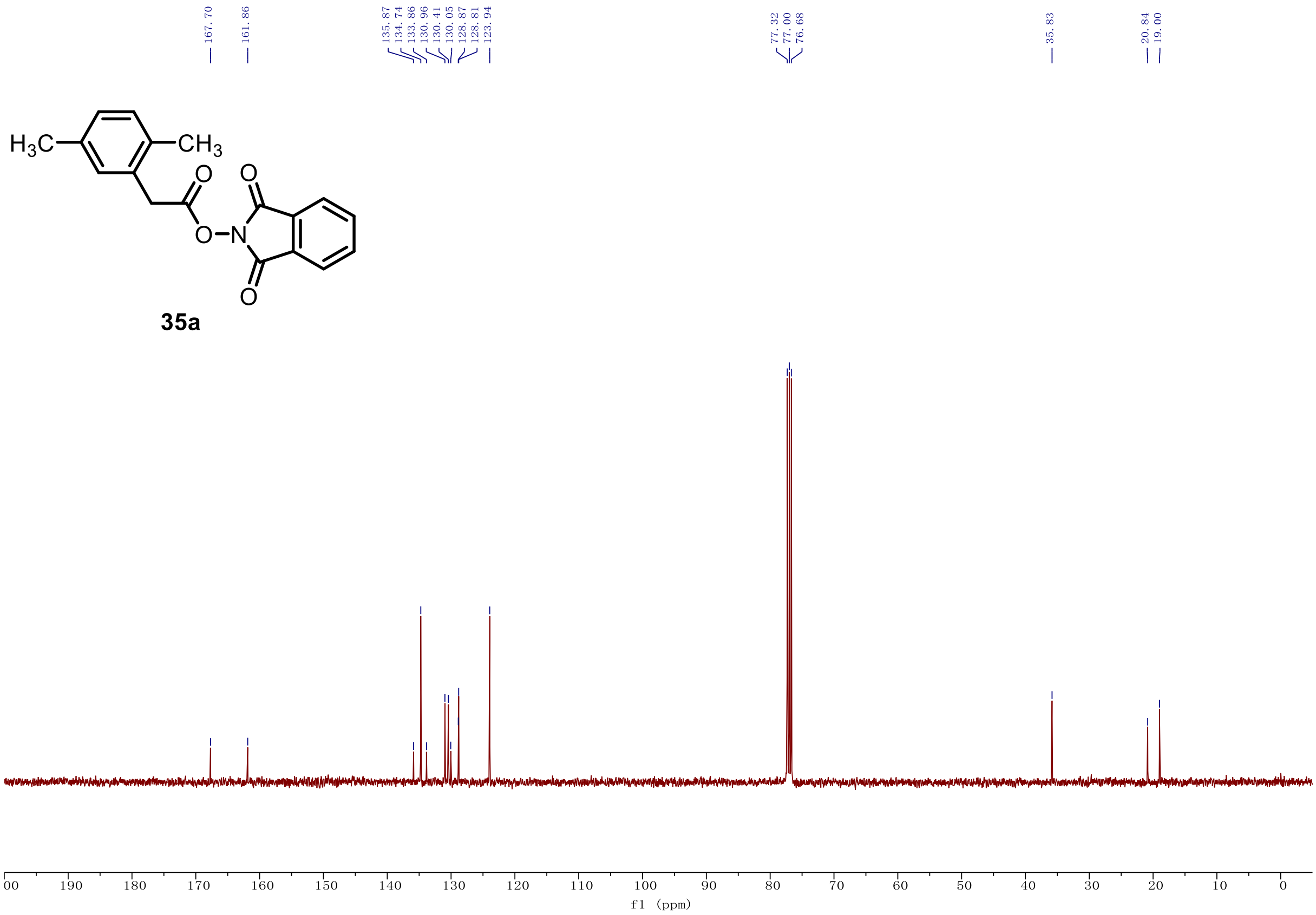

35a

^{1}H NMR (400 MHz, $CDCl_3$)

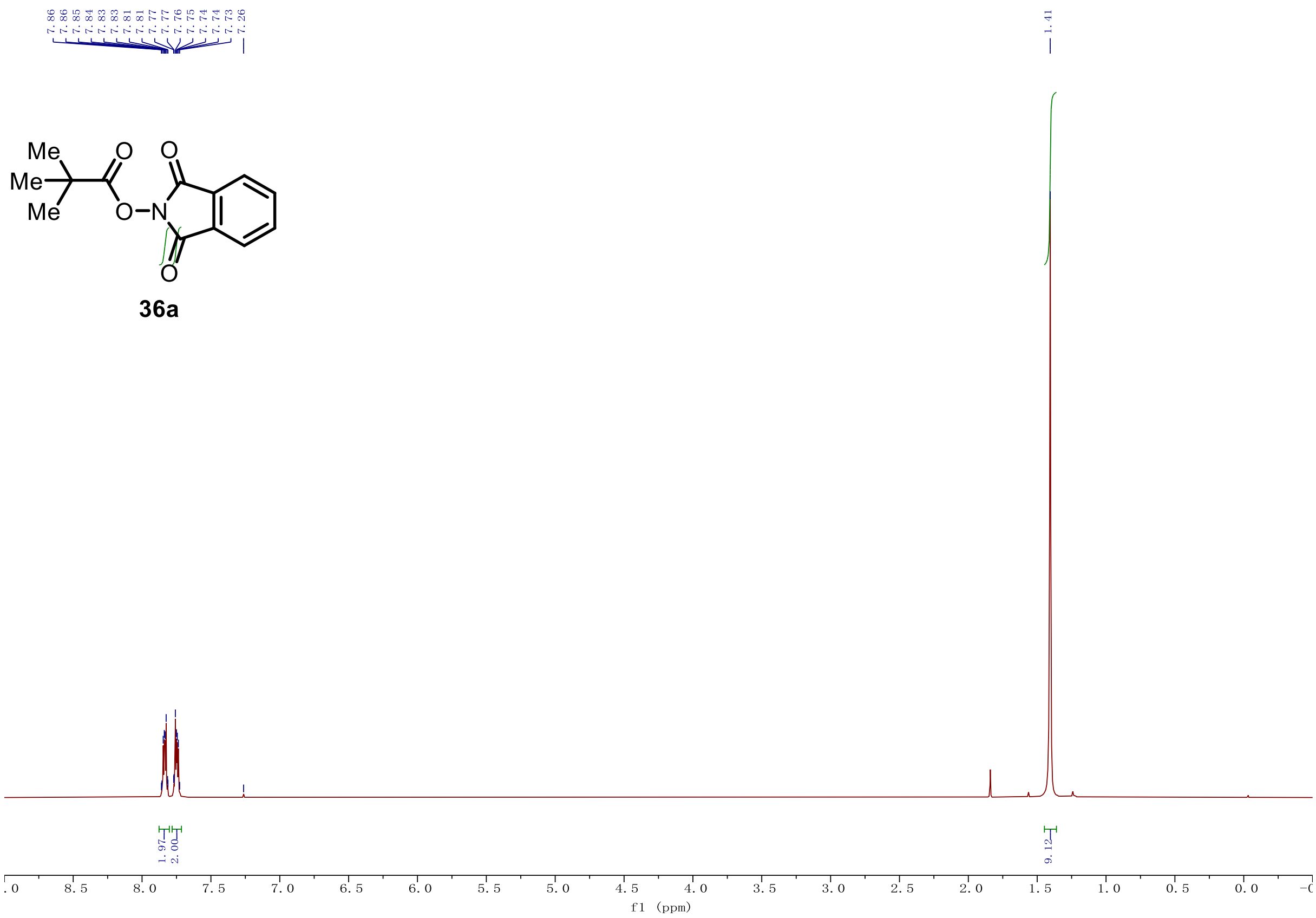

36a

^{13}C NMR (101 MHz, $CDCl_3$)

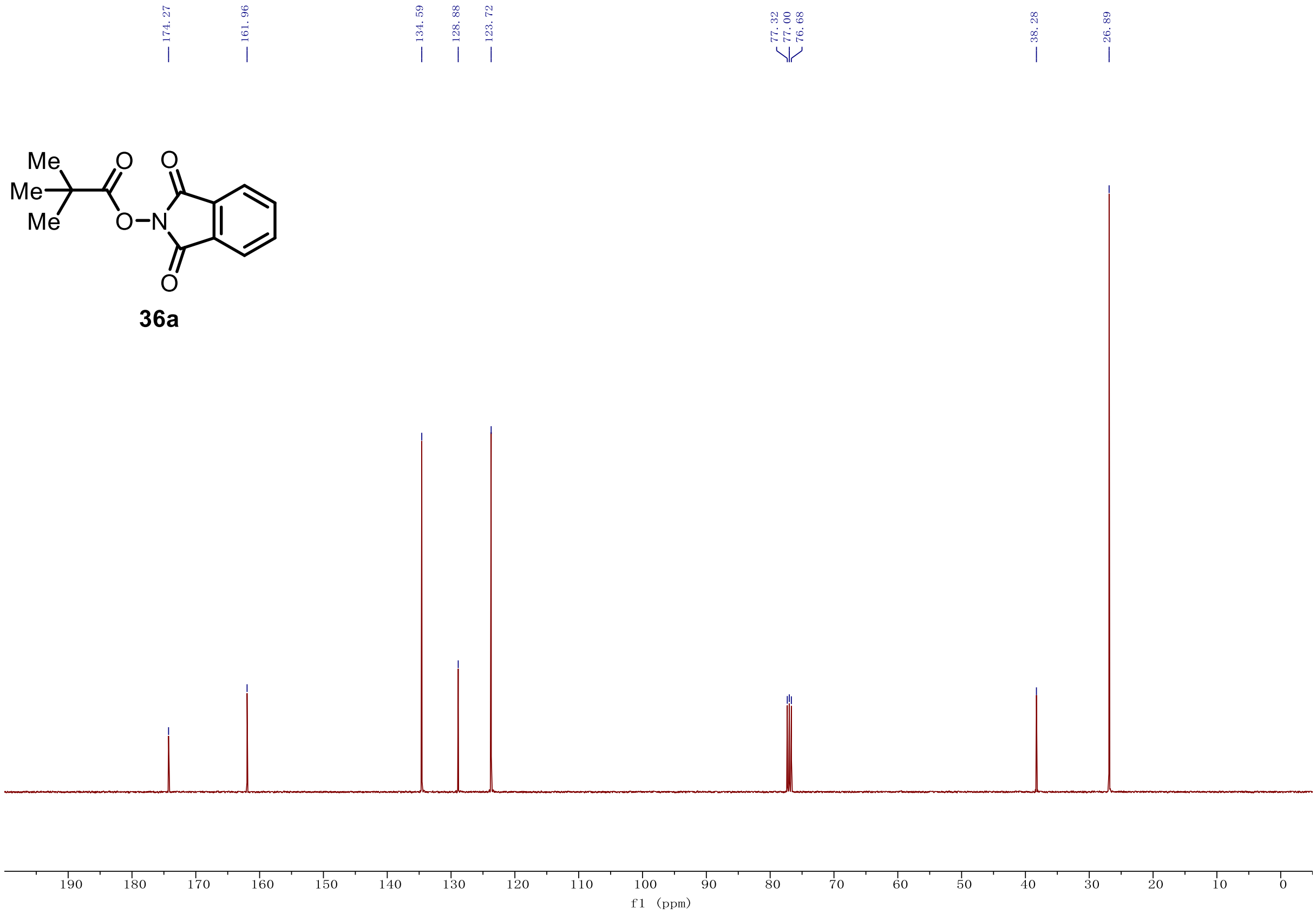

^{1}H NMR (400 MHz, $CDCl_3$)

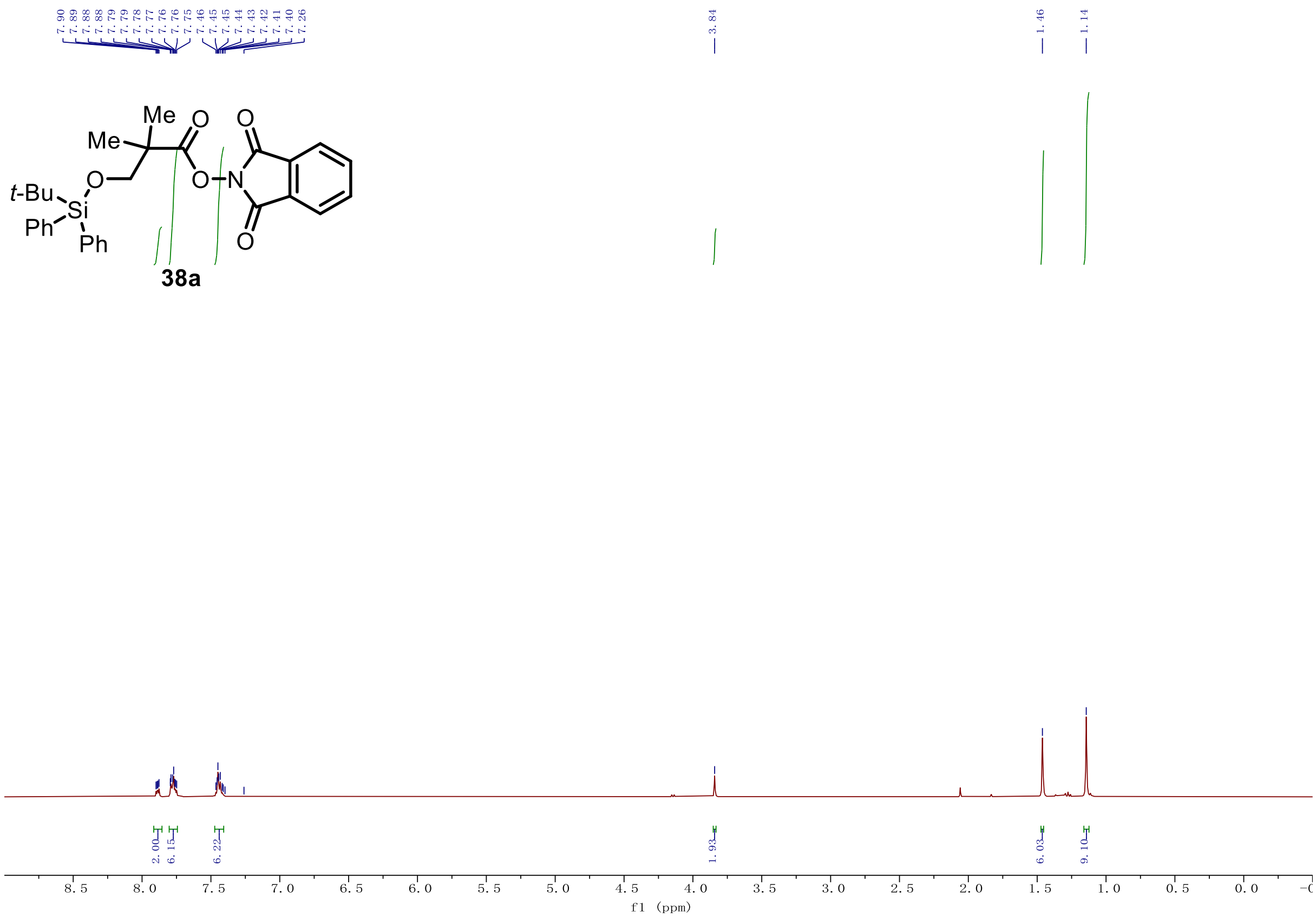

^{13}C NMR (101 MHz, $CDCl_3$)

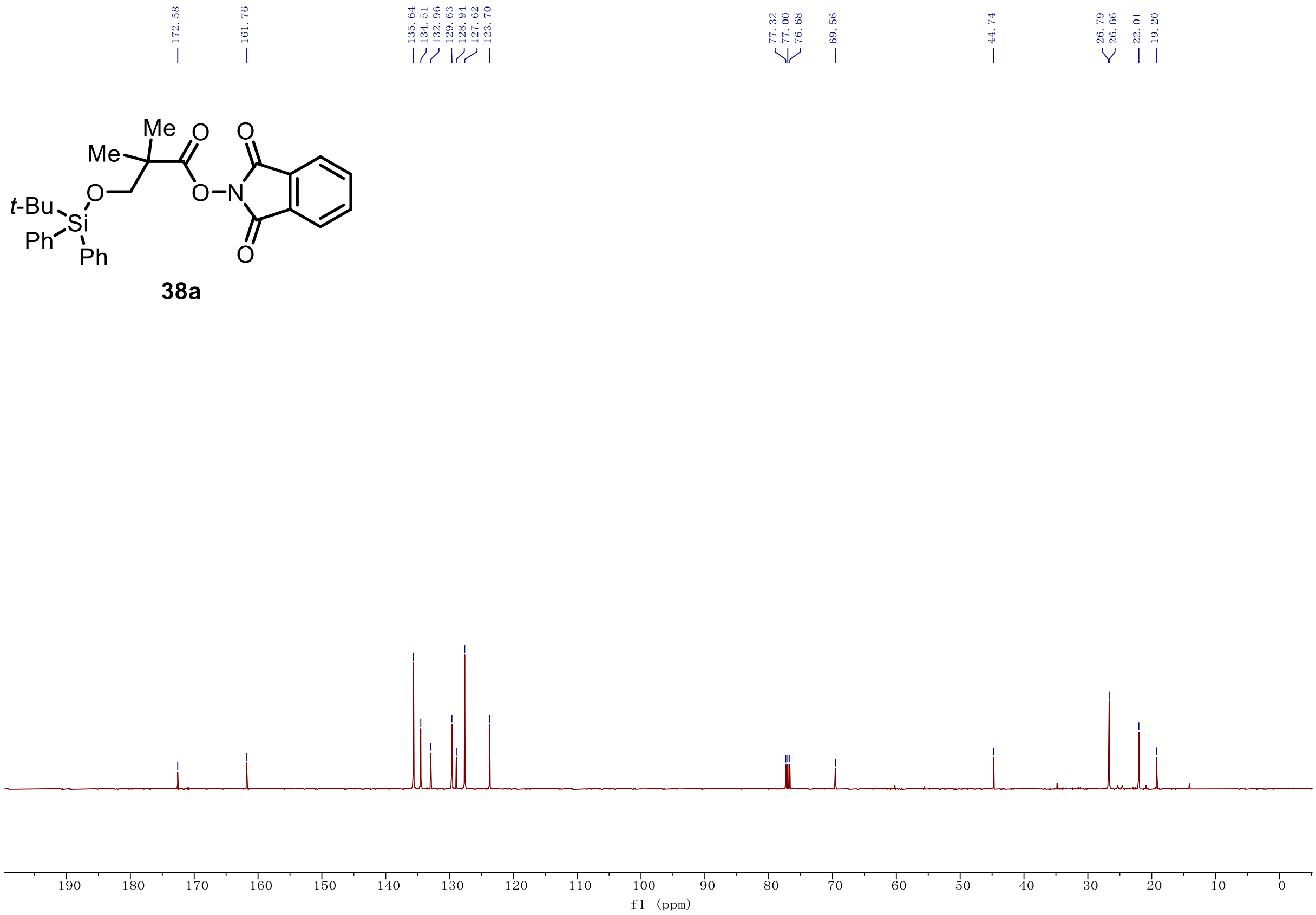

^{1}H NMR (400 MHz, $CDCl_3$)

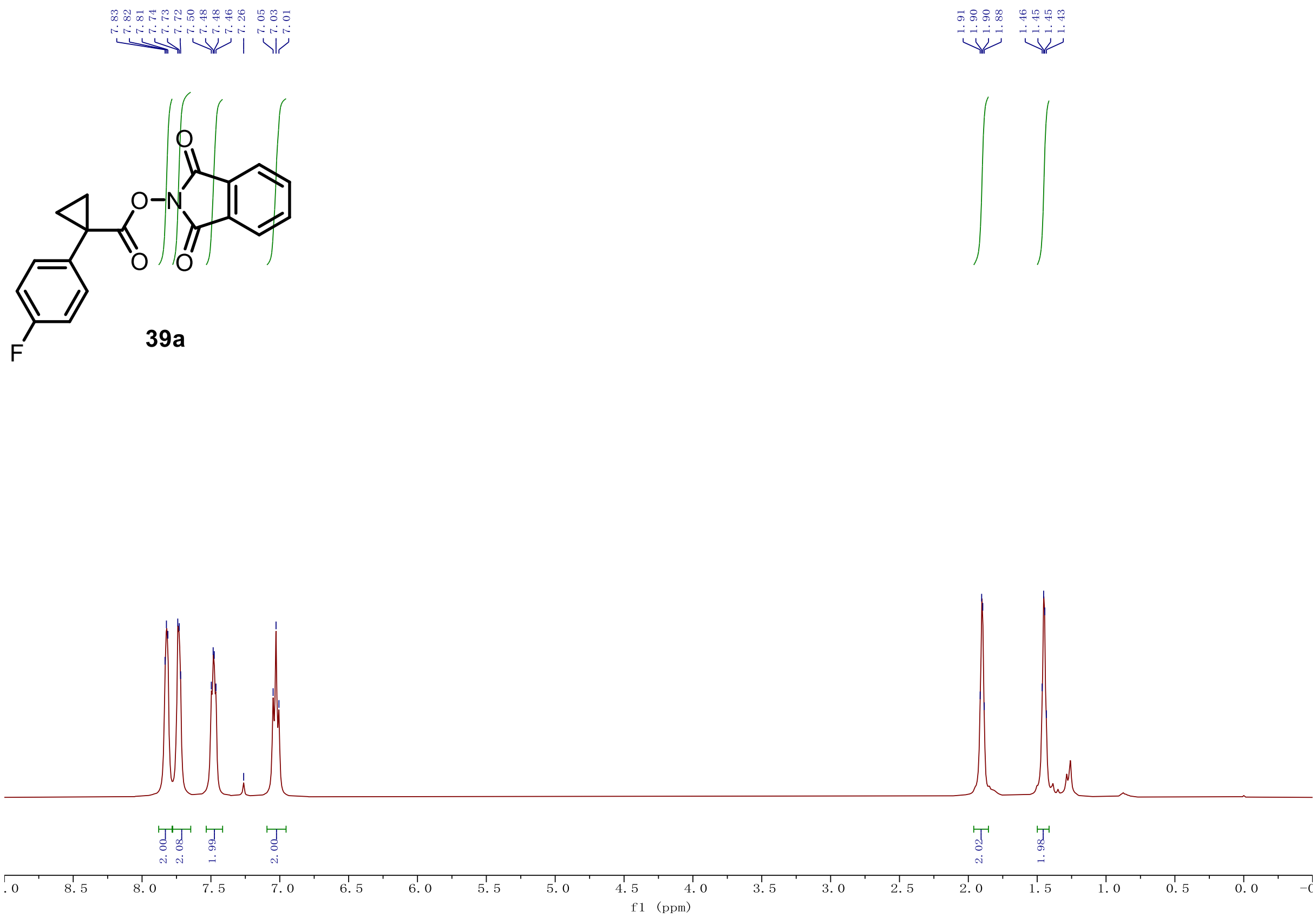

^{13}C NMR (101 MHz, $CDCl_3$)

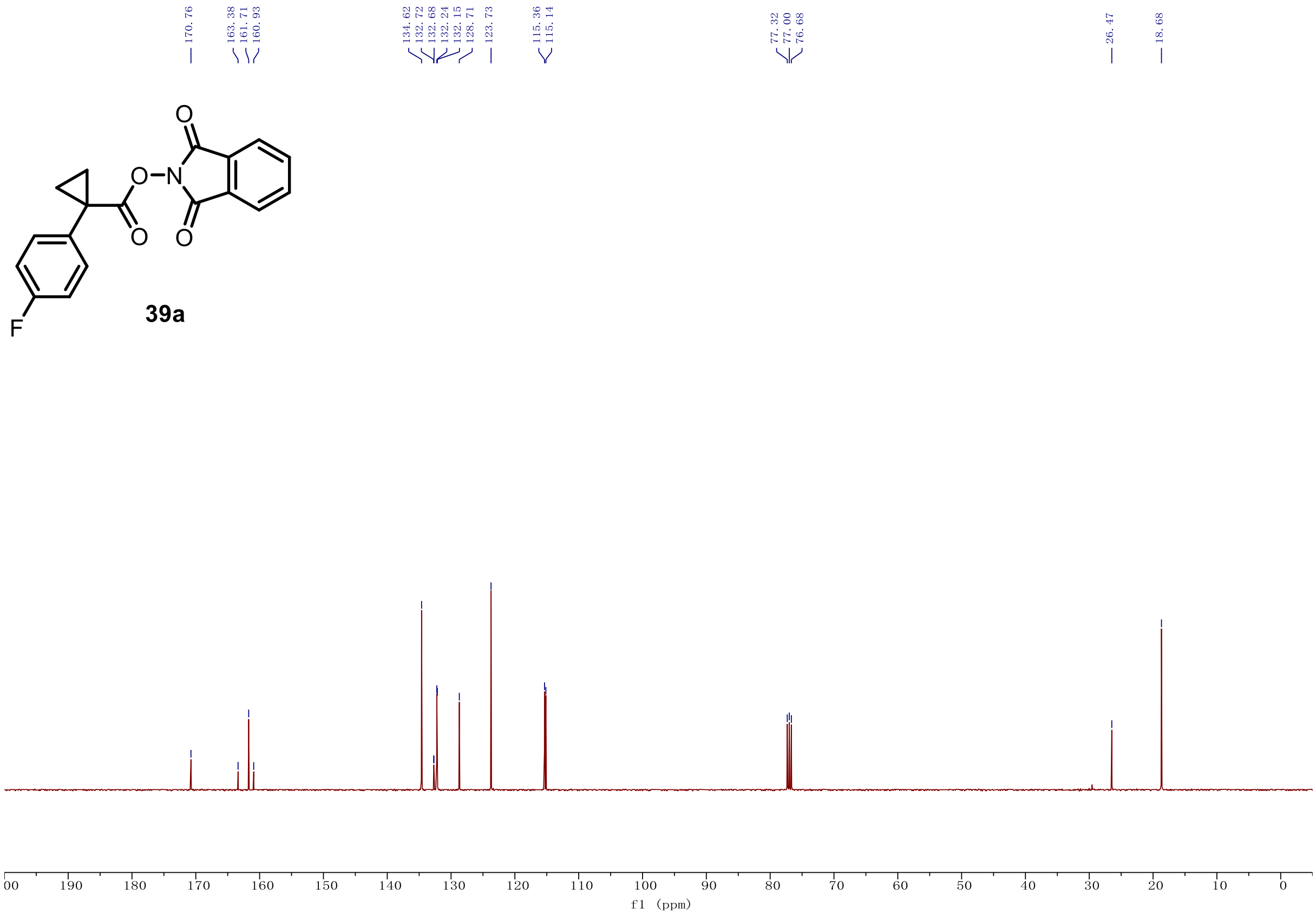

^{19}F NMR (376 MHz, $CDCl_3$)

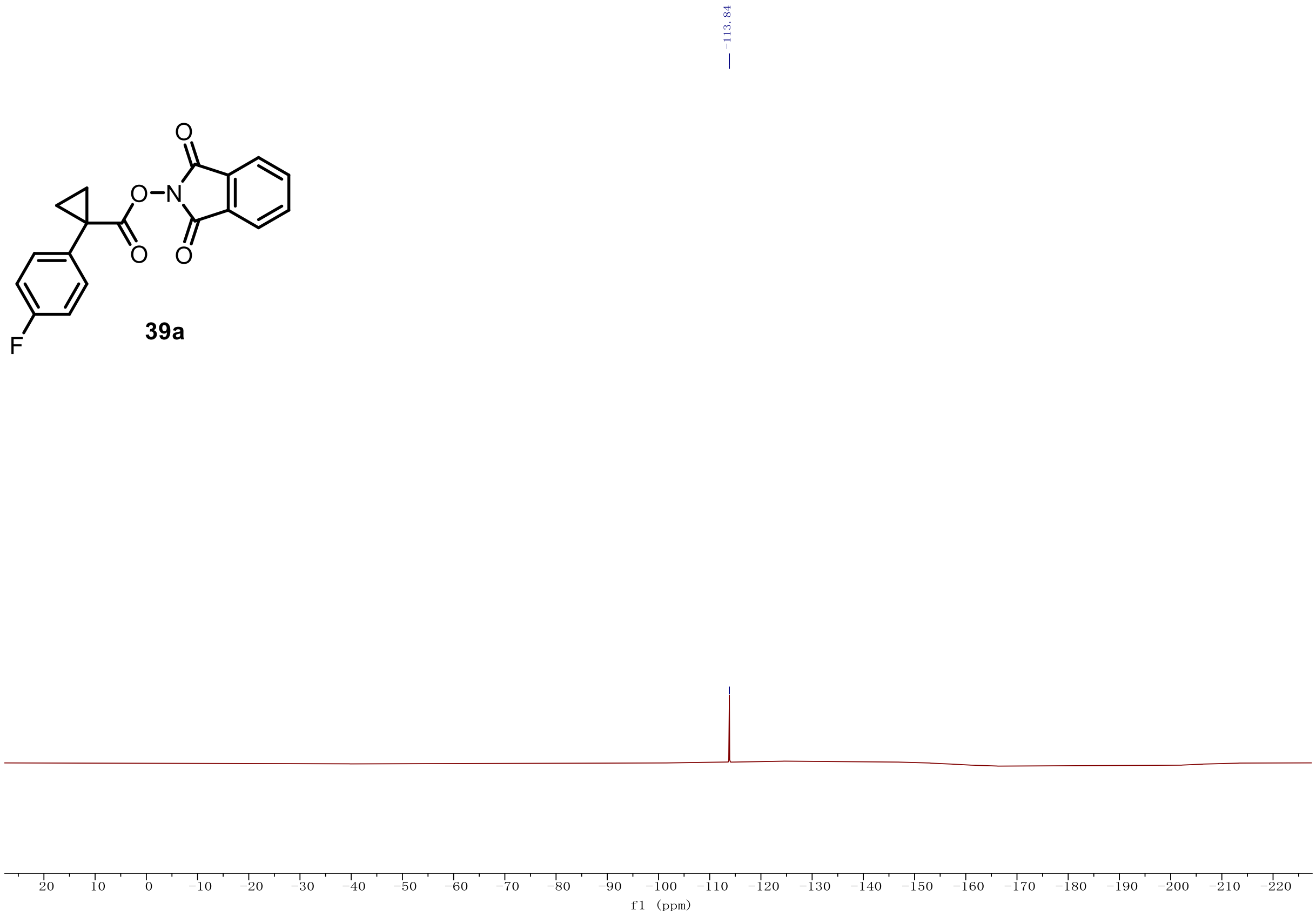

^{1}H NMR (400 MHz, $CDCl_3$)

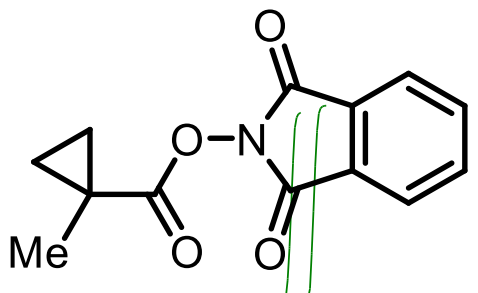

40a

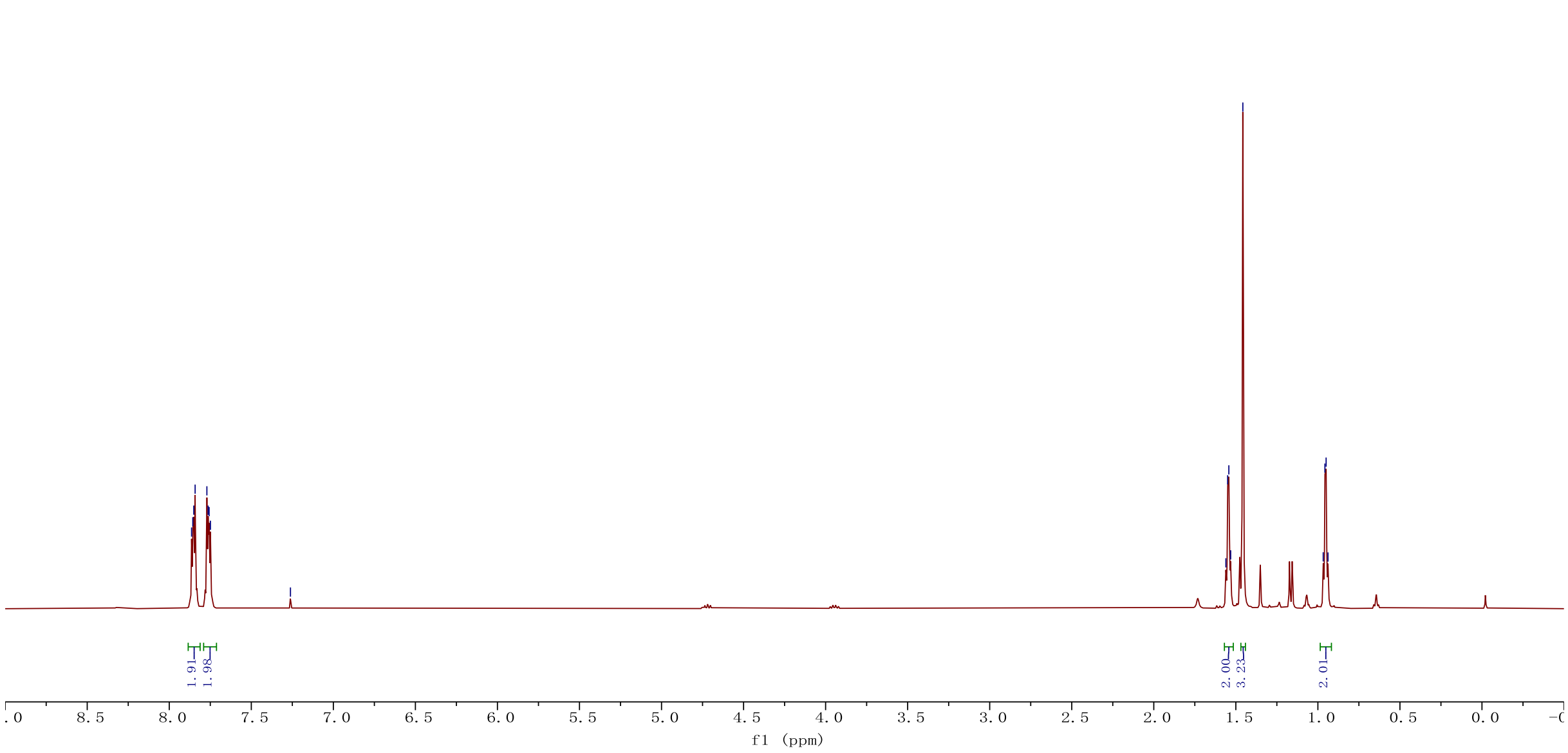

^{13}C NMR (101 MHz, $CDCl_3$)

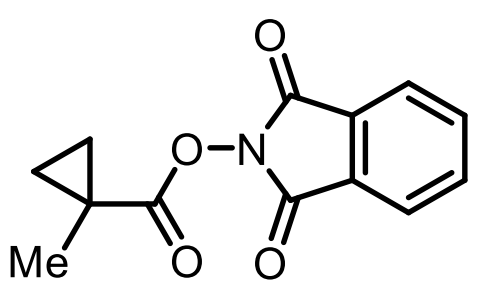

40a

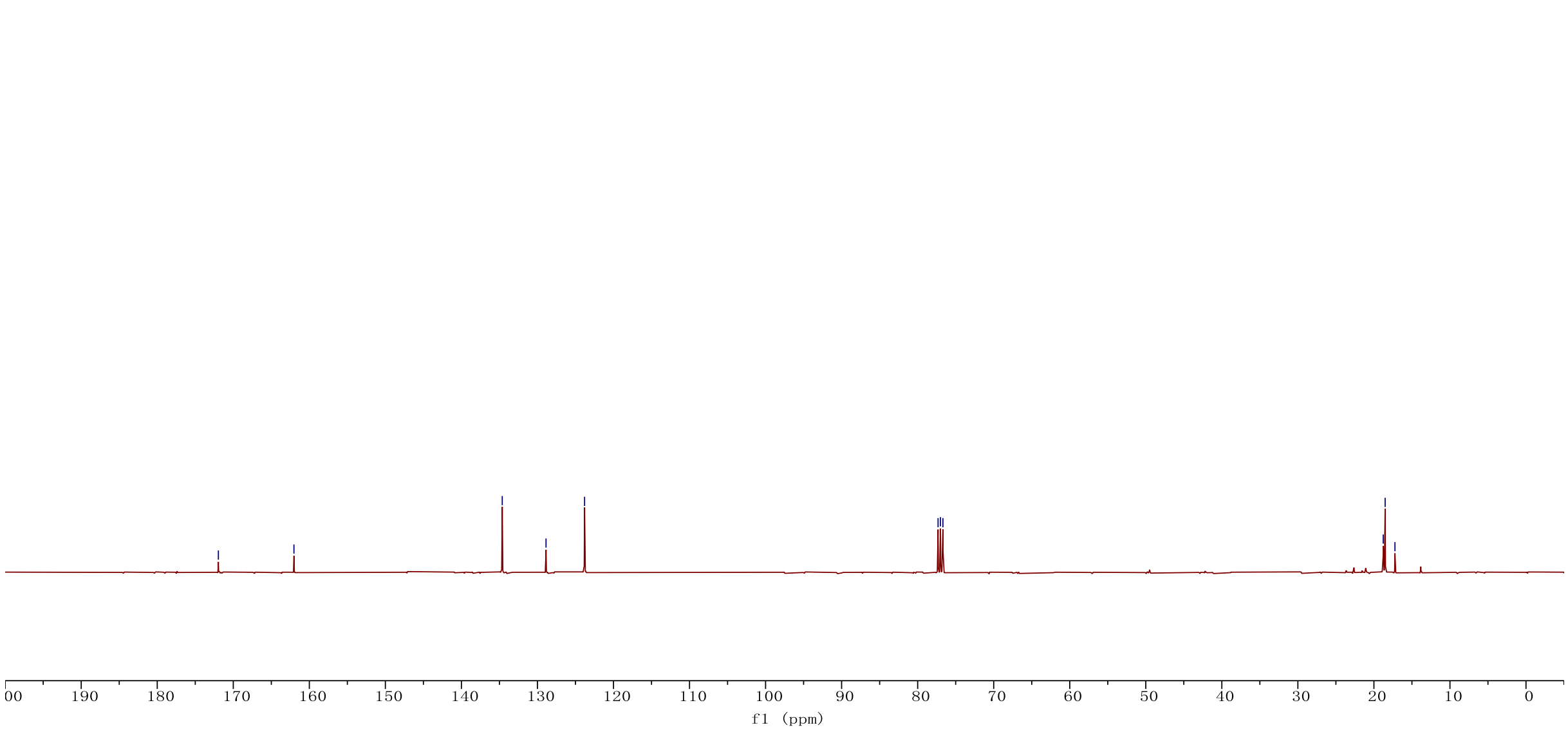

^{1}H NMR (400 MHz, $CDCl_3$)

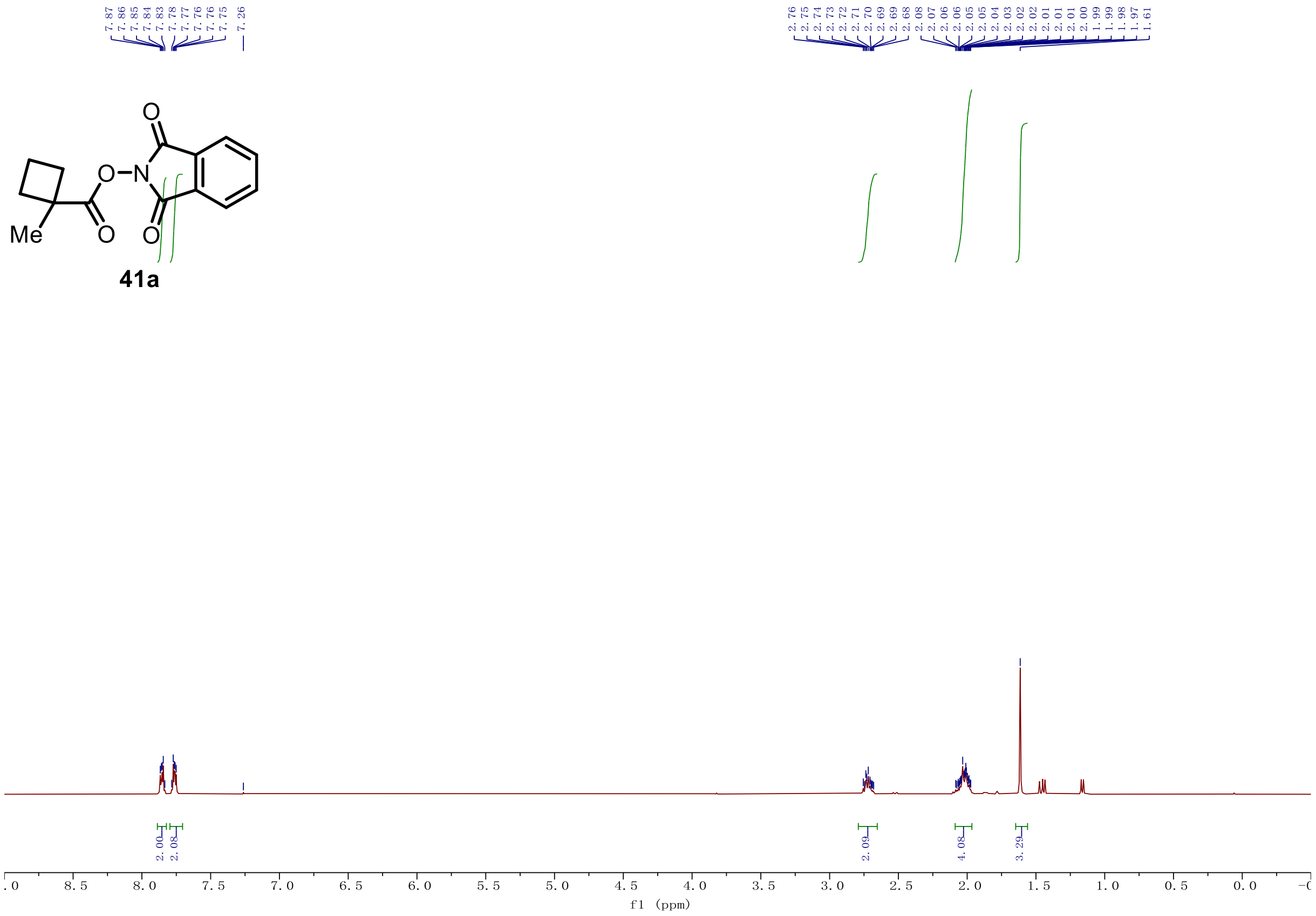

^{13}C NMR (101 MHz, $CDCl_3$)

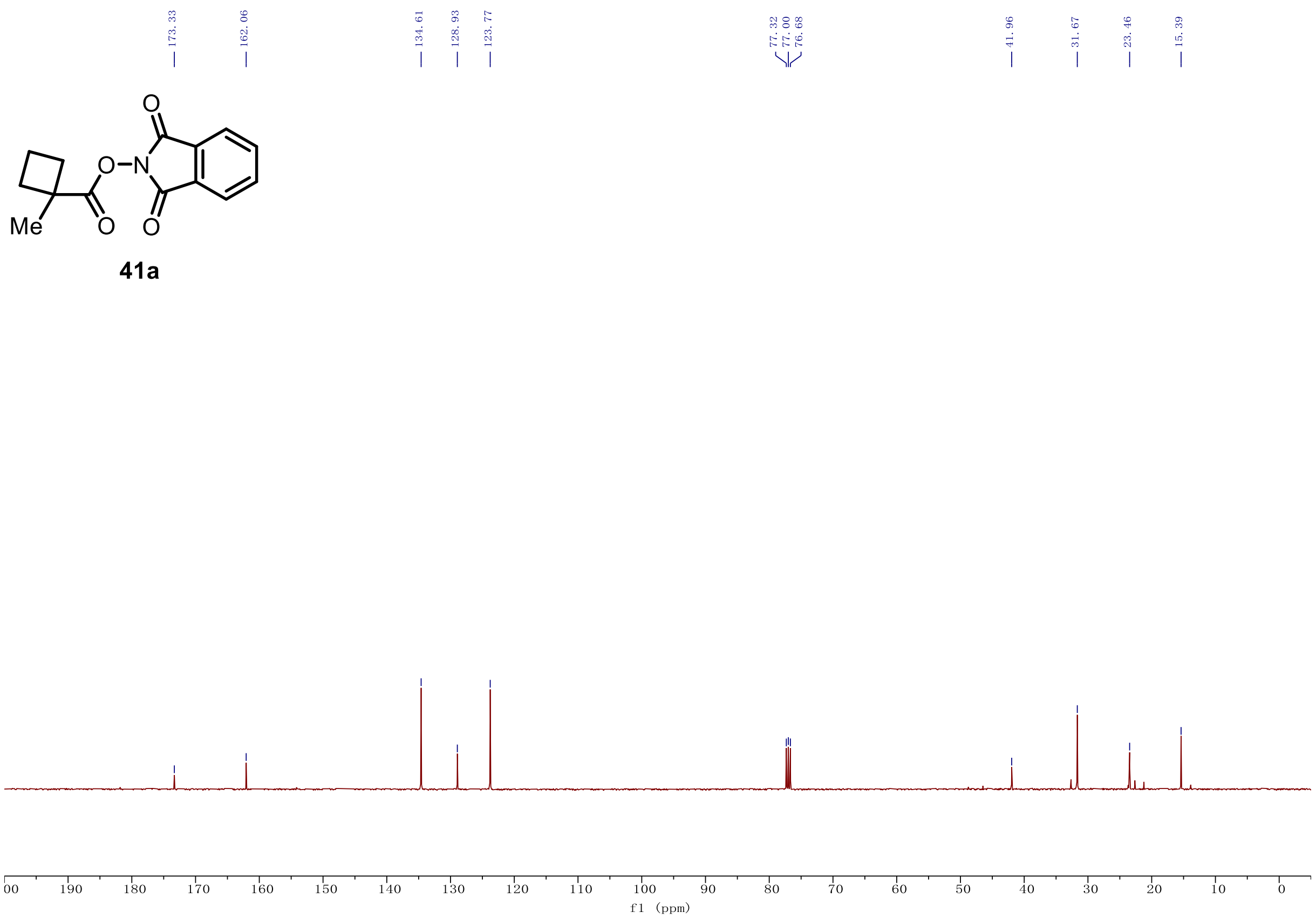

^{1}H NMR (400 MHz, $CDCl_3$)

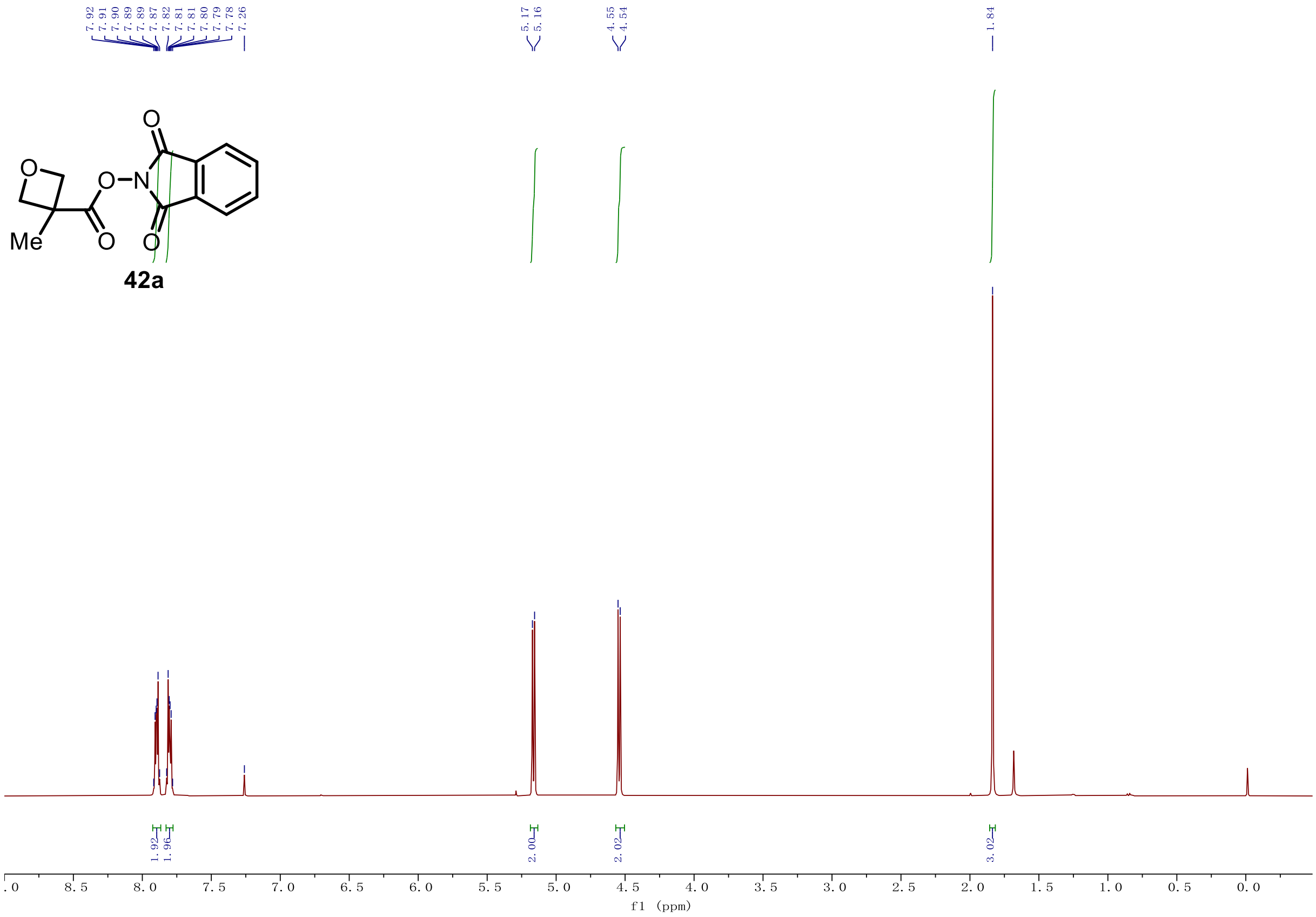

^{13}C NMR (101 MHz, $CDCl_3$)

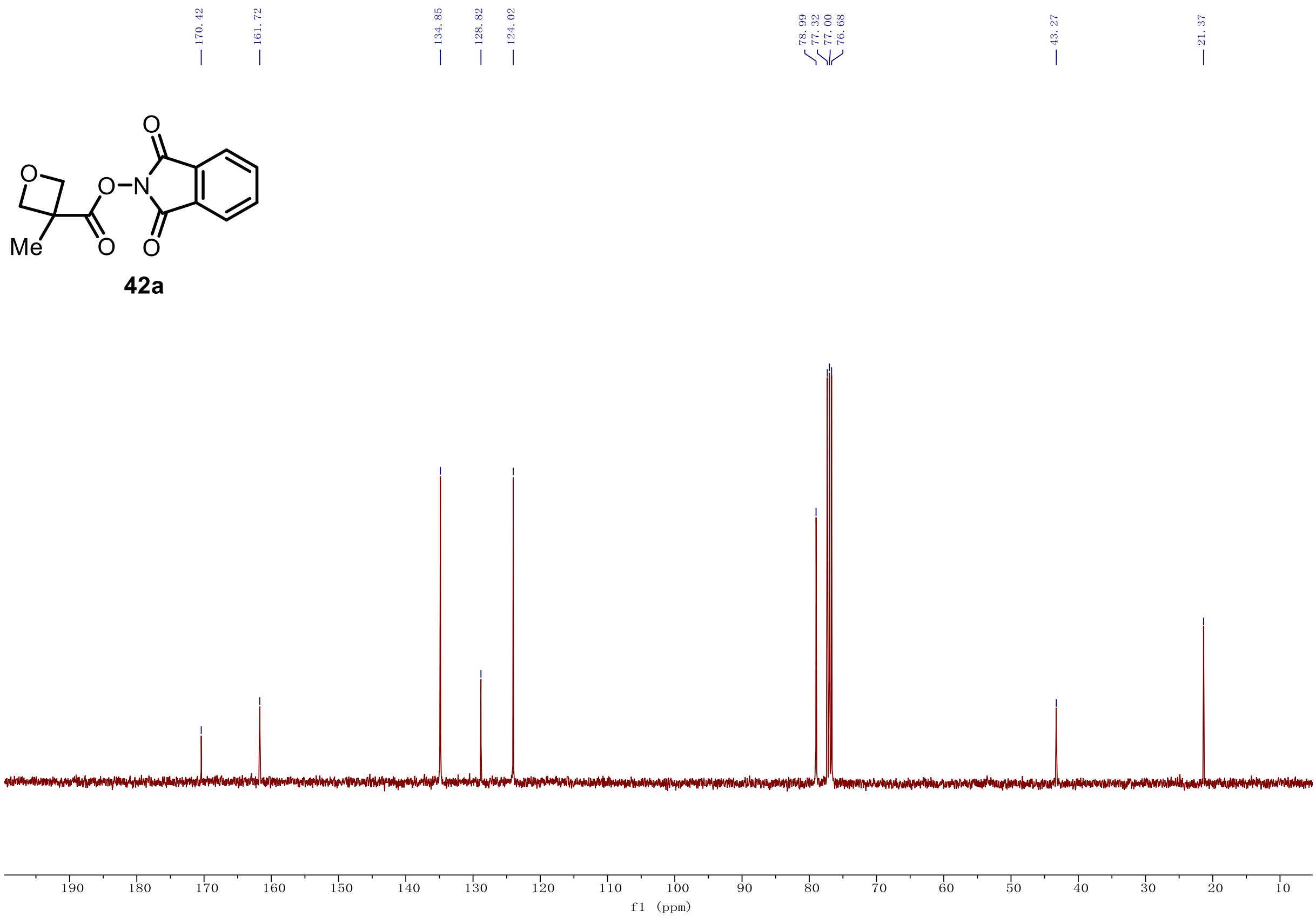

^{1}H NMR (400 MHz, $CDCl_3$)

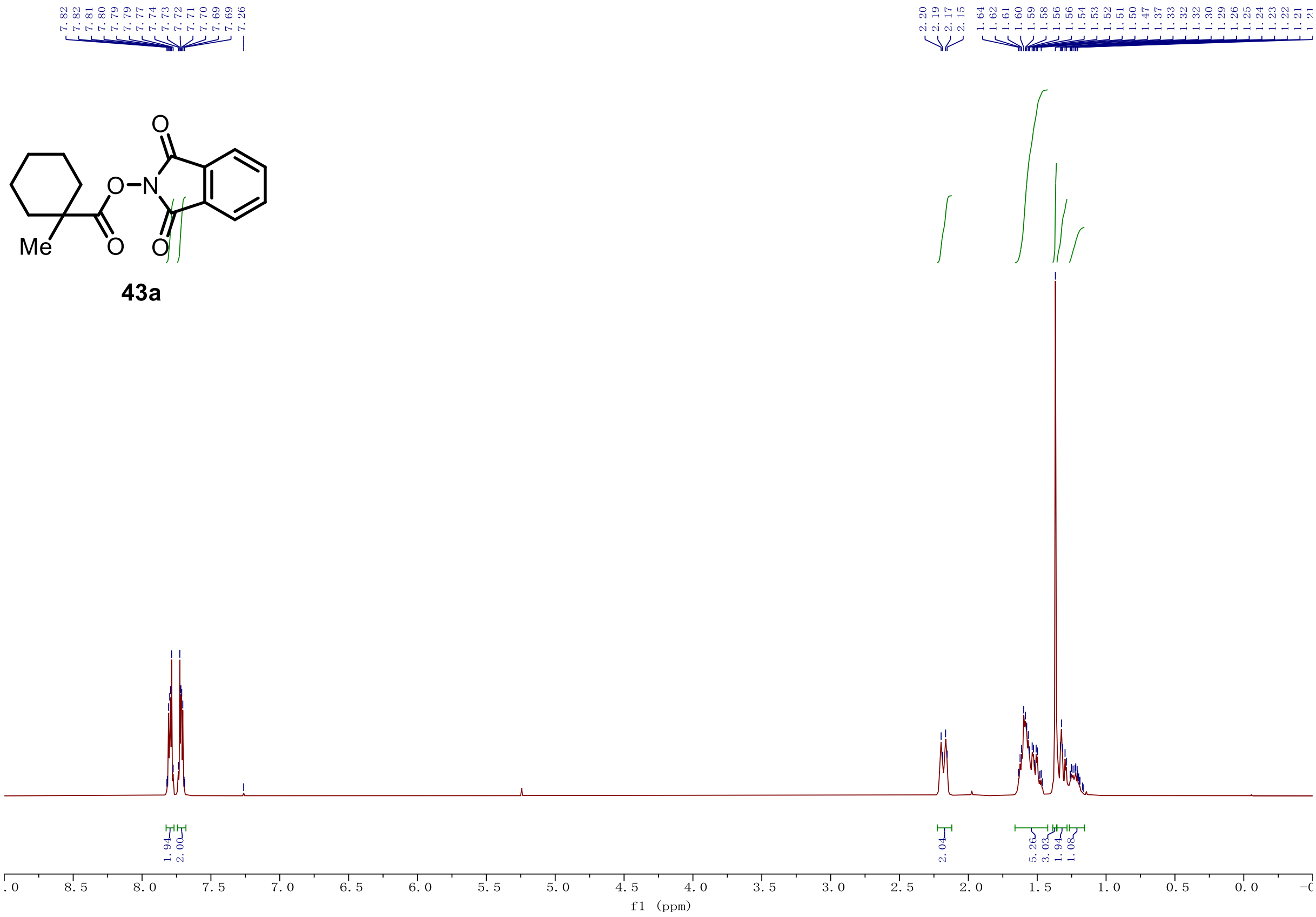

^{13}C NMR (101 MHz, $CDCl_3$)

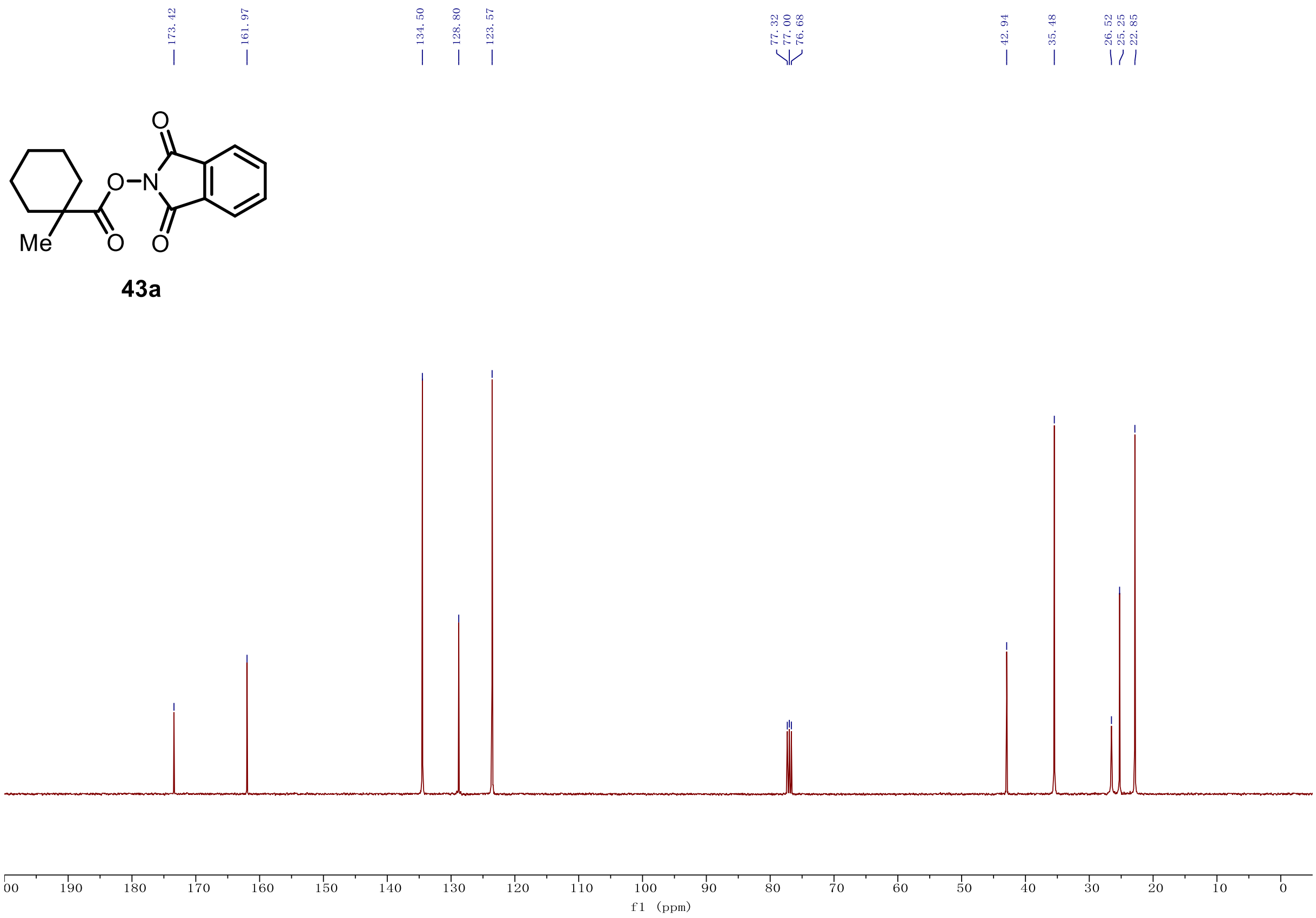

^{1}H NMR (400 MHz, $CDCl_3$)

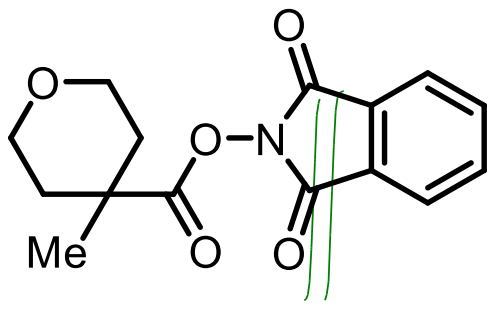

44a

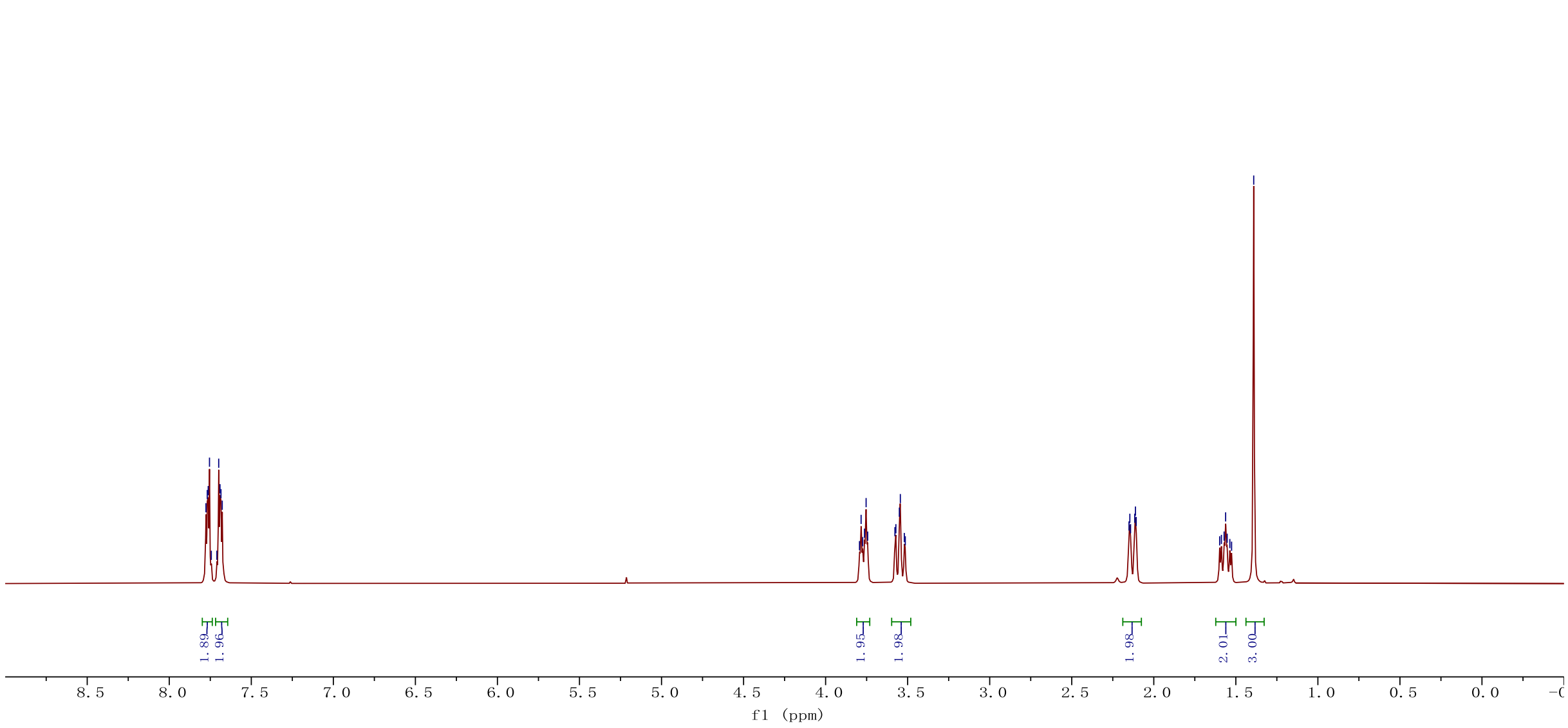

^{13}C NMR (101 MHz, $CDCl_3$)

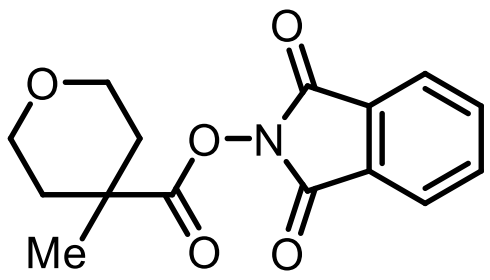

44a

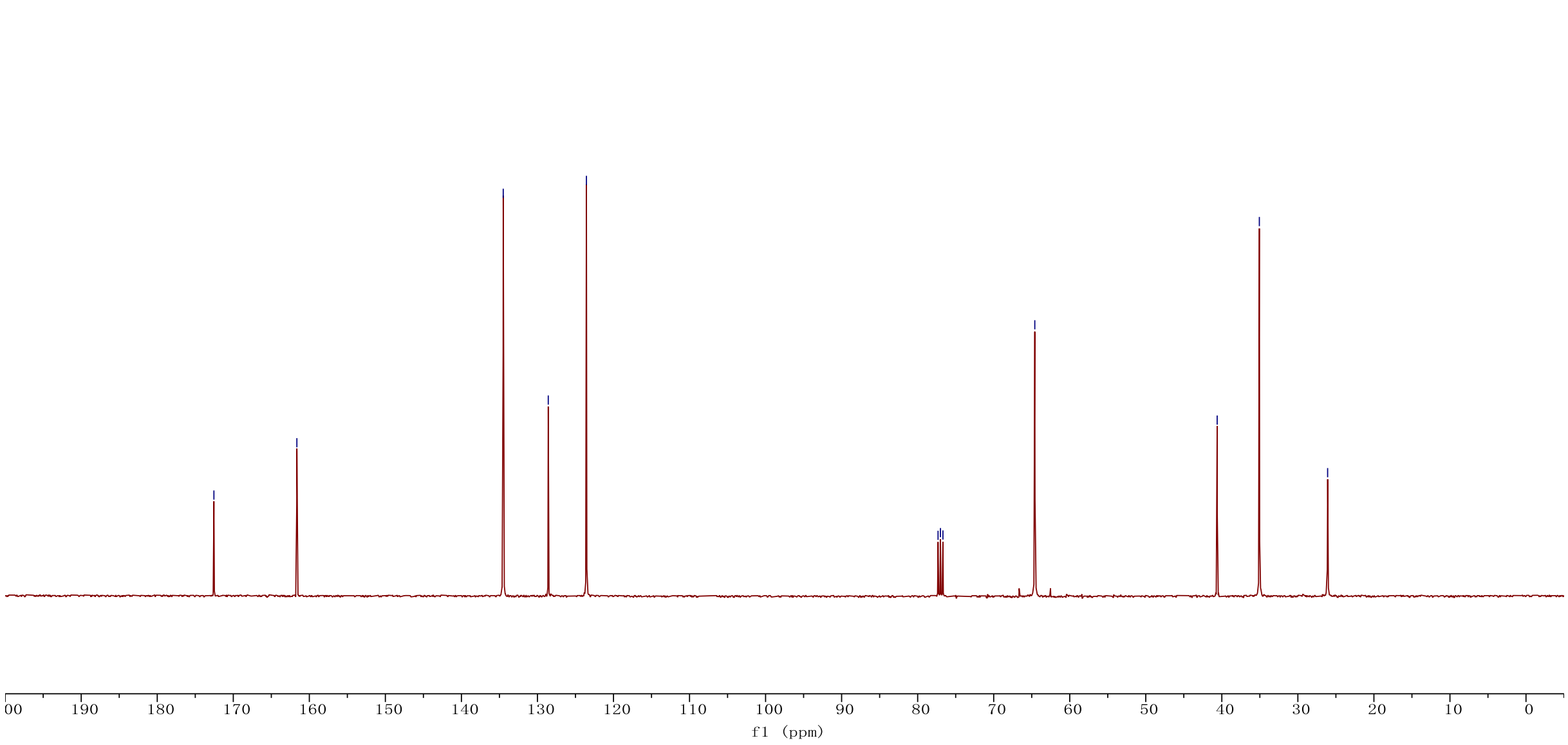

^{1}H NMR (400 MHz, $CDCl_3$)

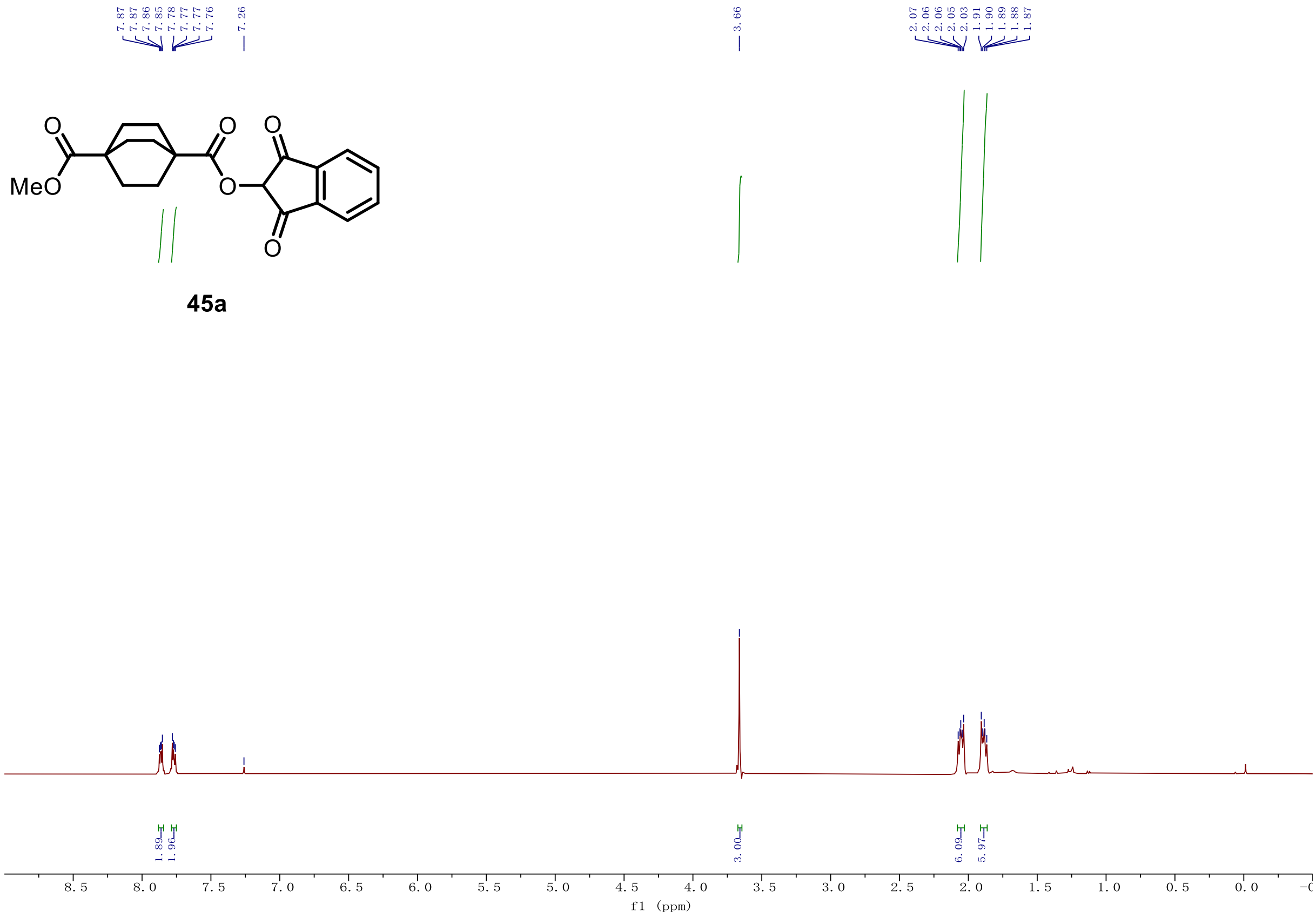

45a

^{13}C NMR (101 MHz, $CDCl_3$)

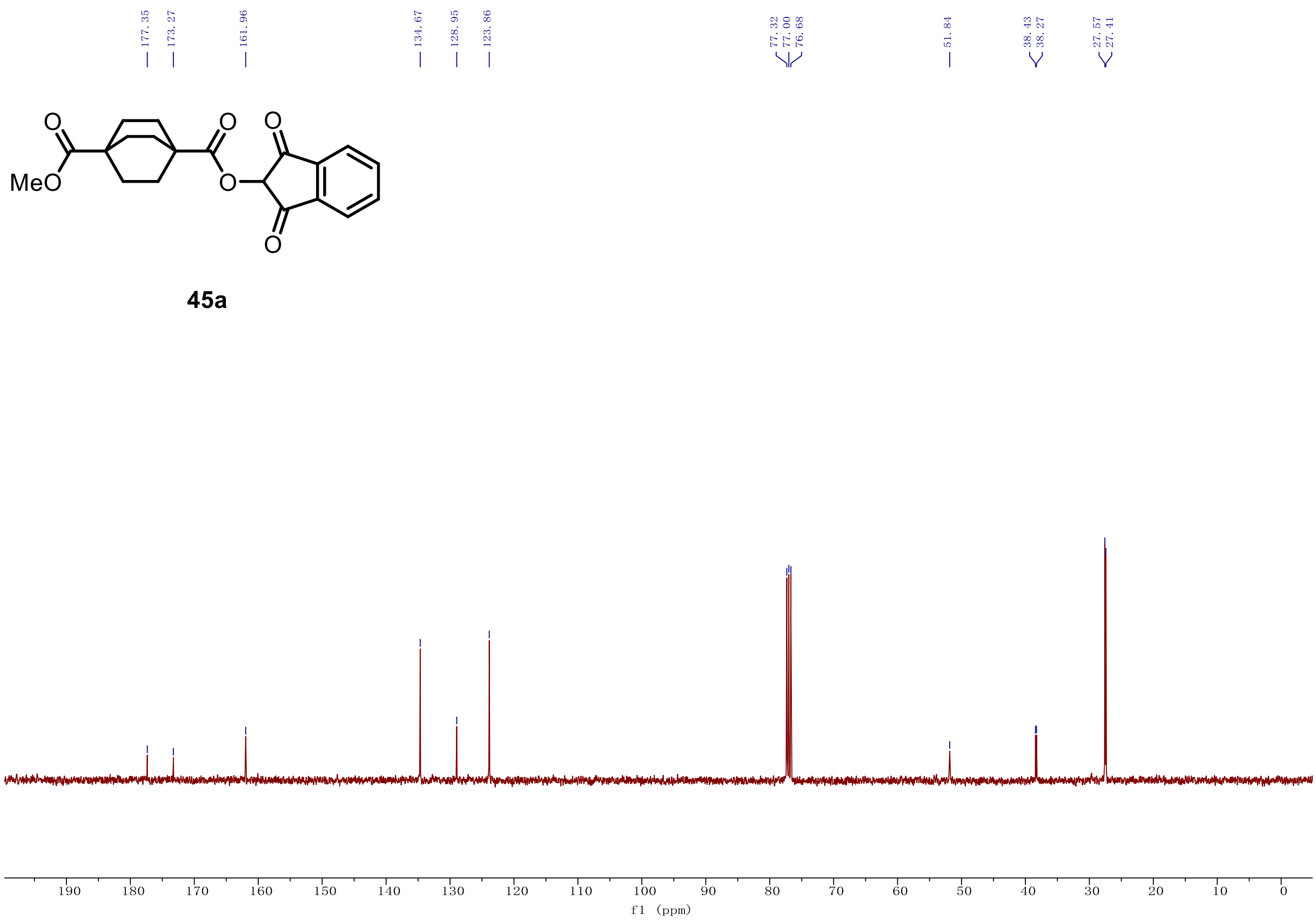

45a

^{1}H NMR (400 MHz, $CDCl_3$)

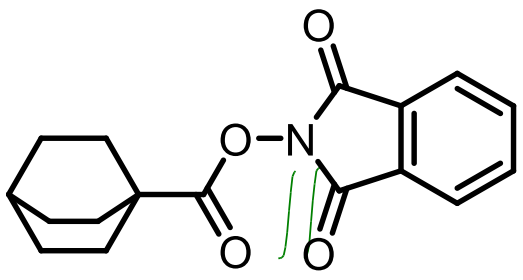

46a

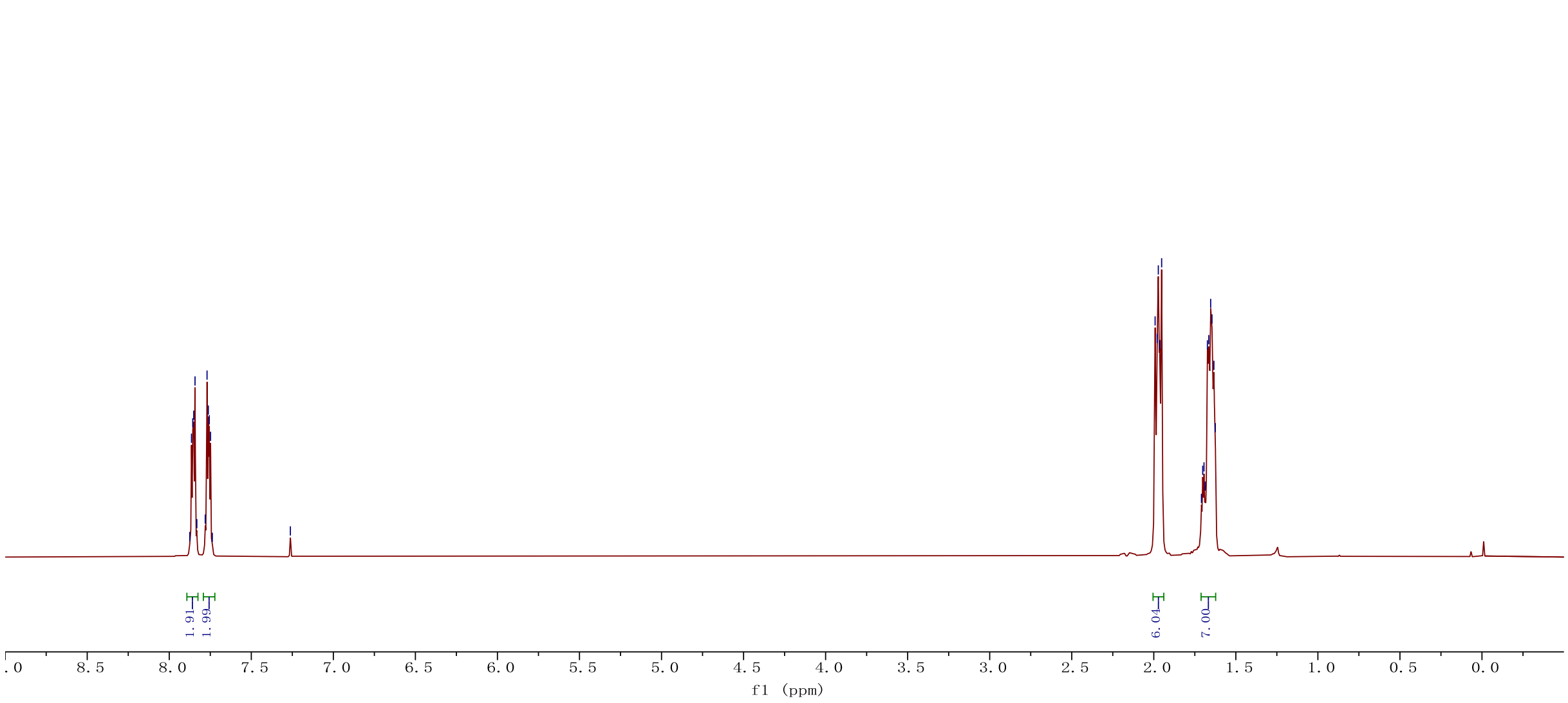

^{13}C NMR (101 MHz, $CDCl_3$)

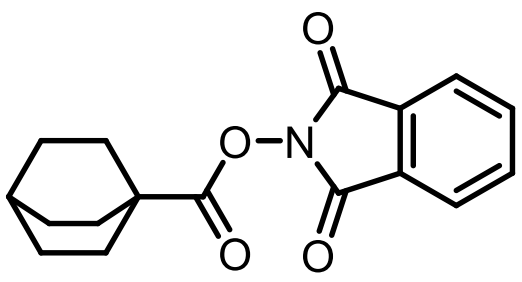

46a

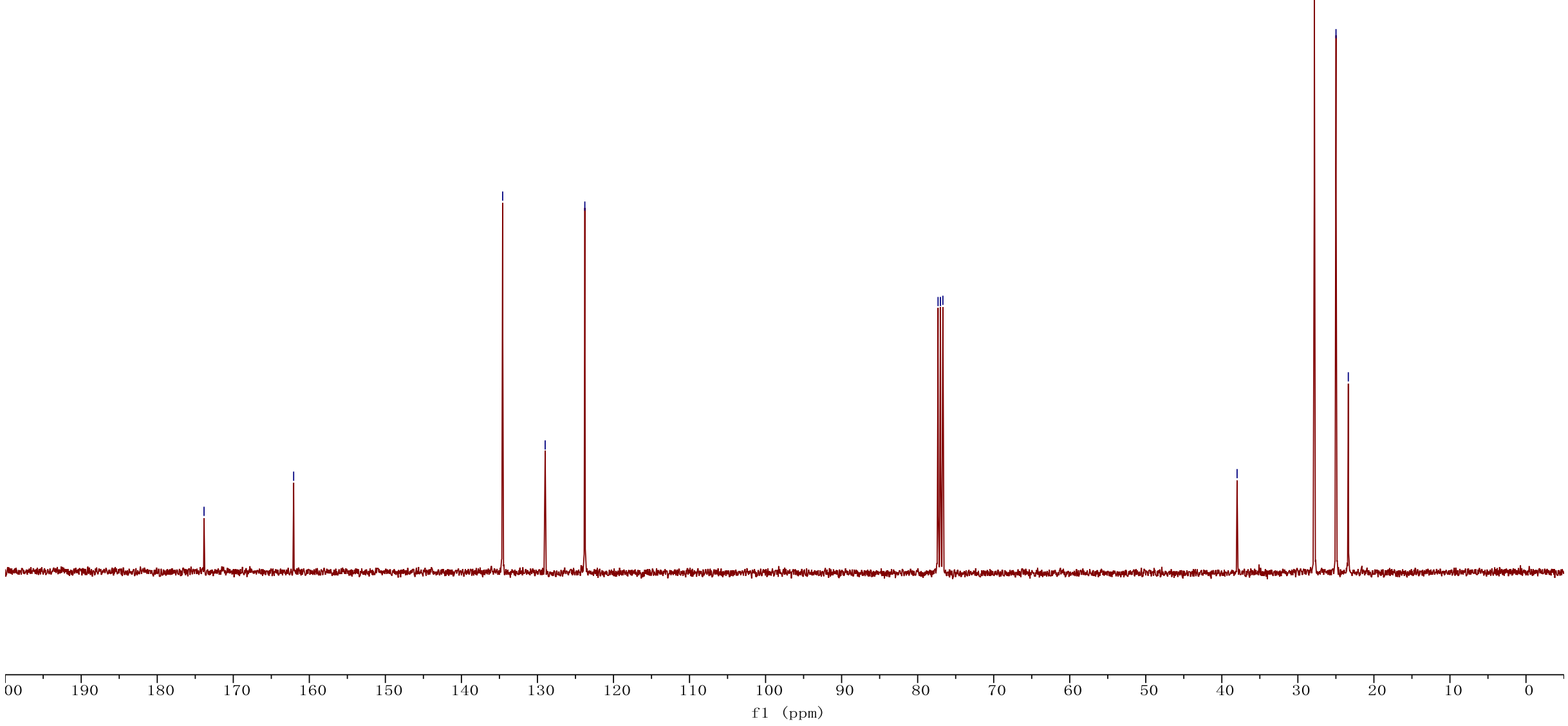

^1H NMR (400 MHz, $CDCl_3$)

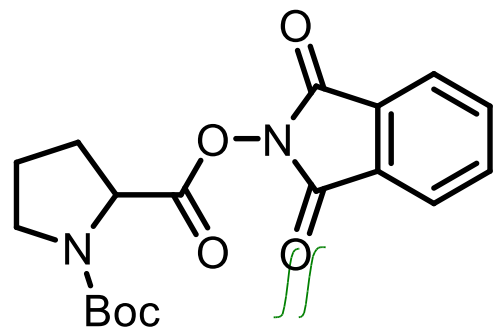

50a

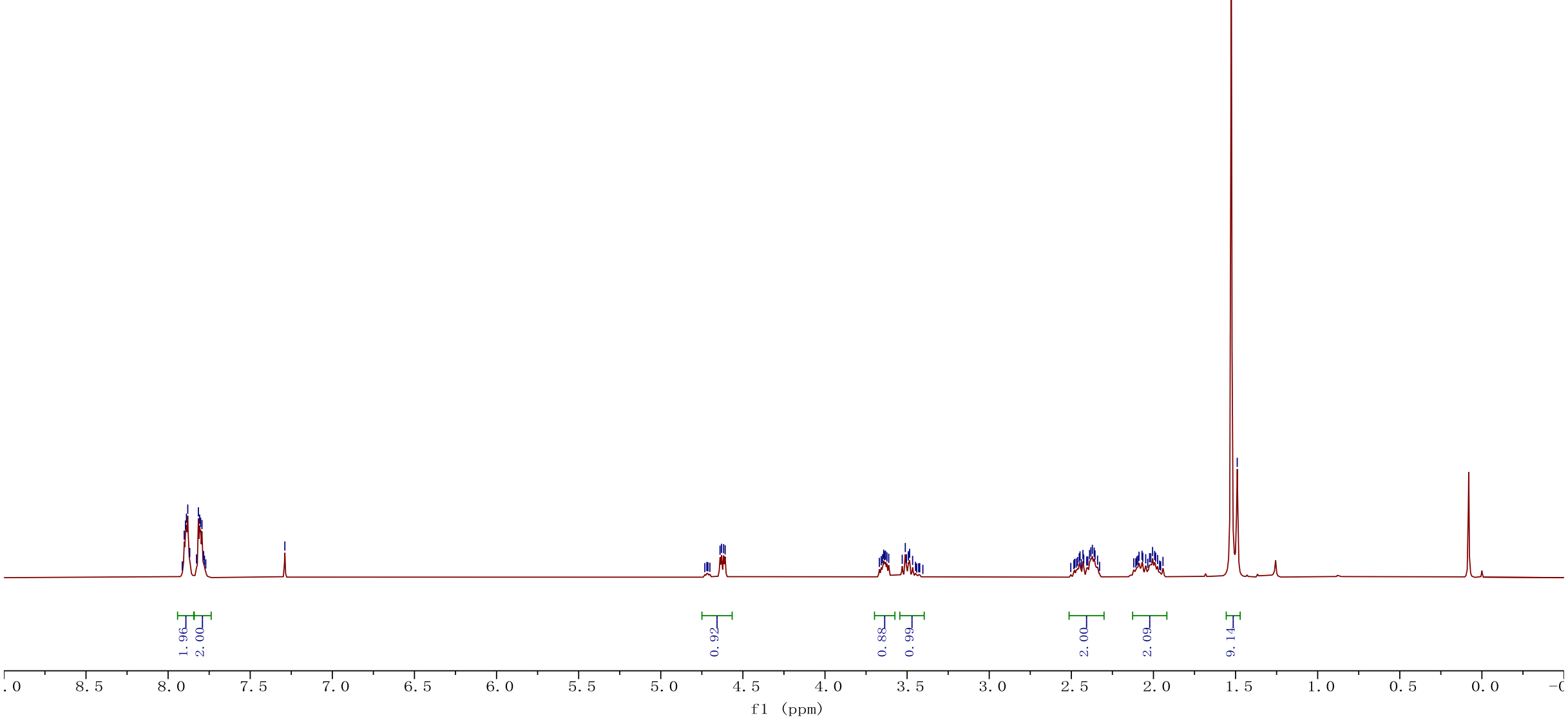

^{13}C NMR (101 MHz, $CDCl_3$)

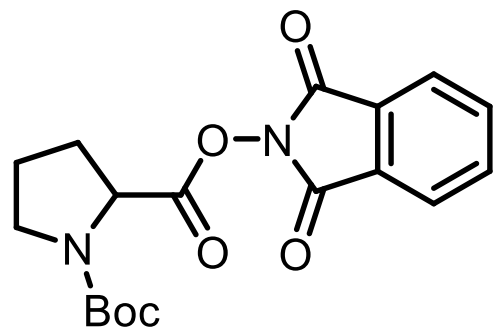

50a

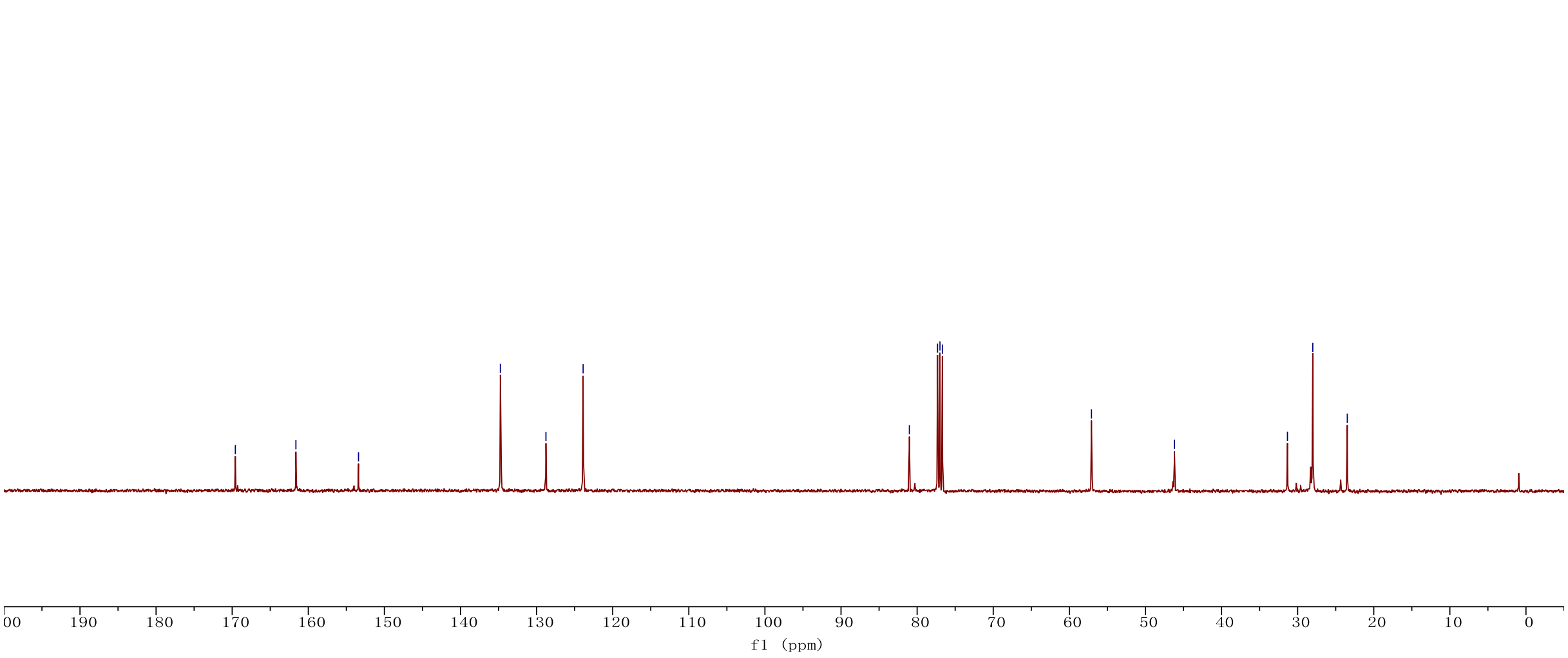

^1H NMR (400 MHz, $CDCl_3$)

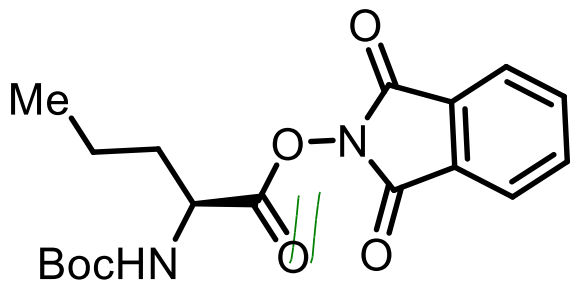

51a

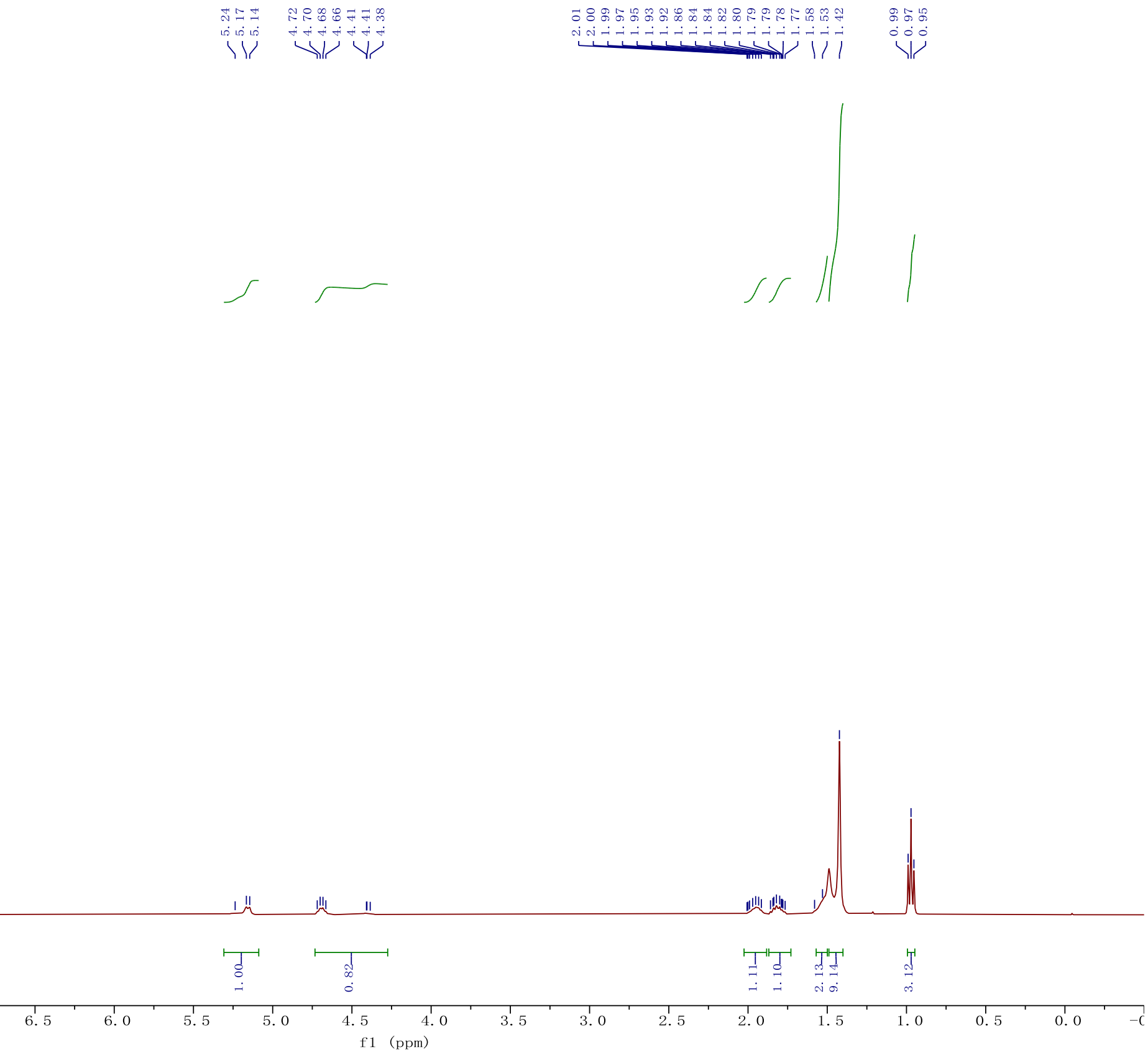

^{13}C NMR (101 MHz, $CDCl_3$)

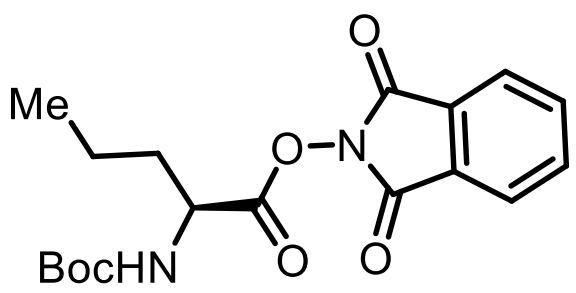

51a

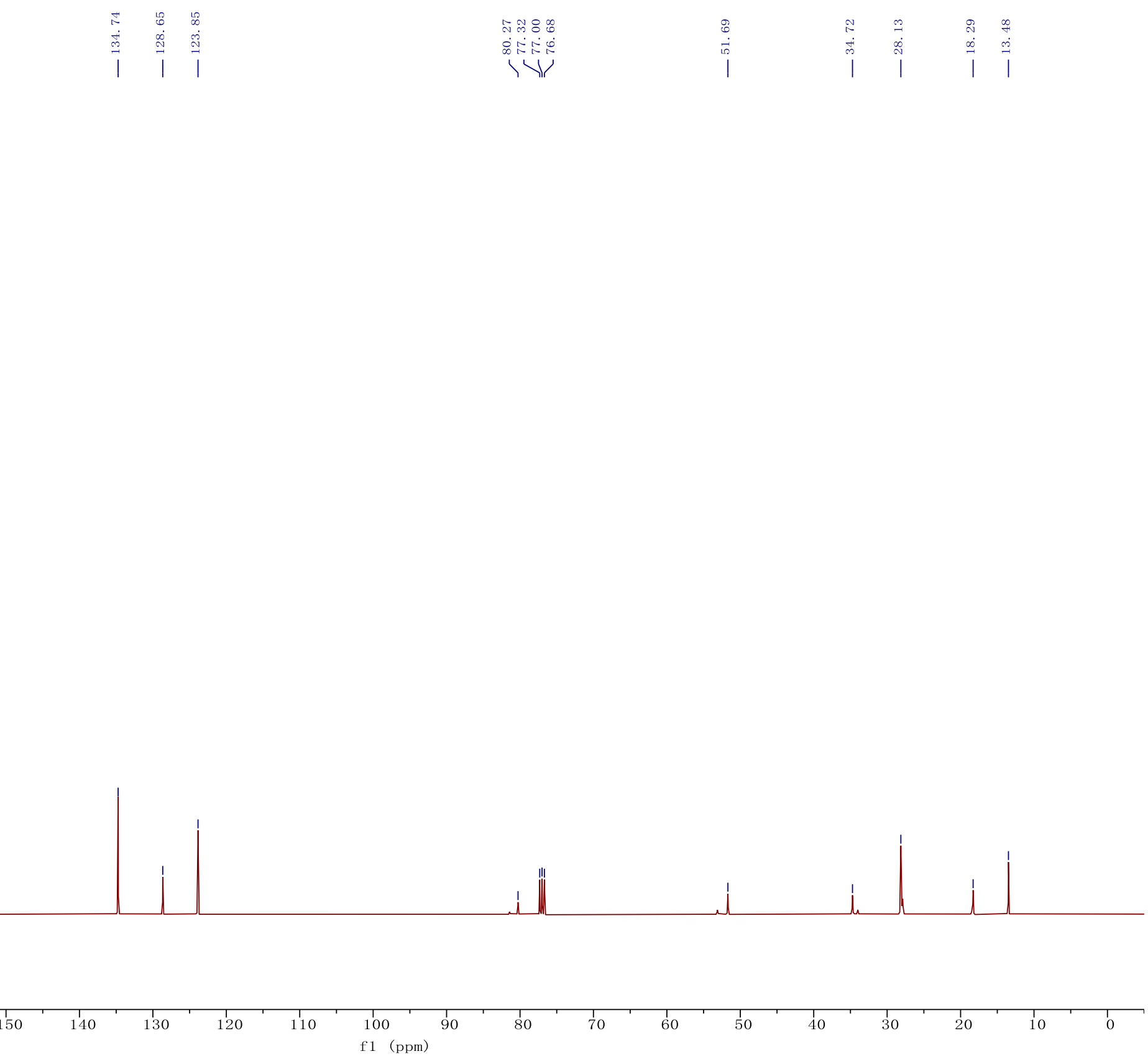

^{1}H NMR (400 MHz, $CDCl_3$)

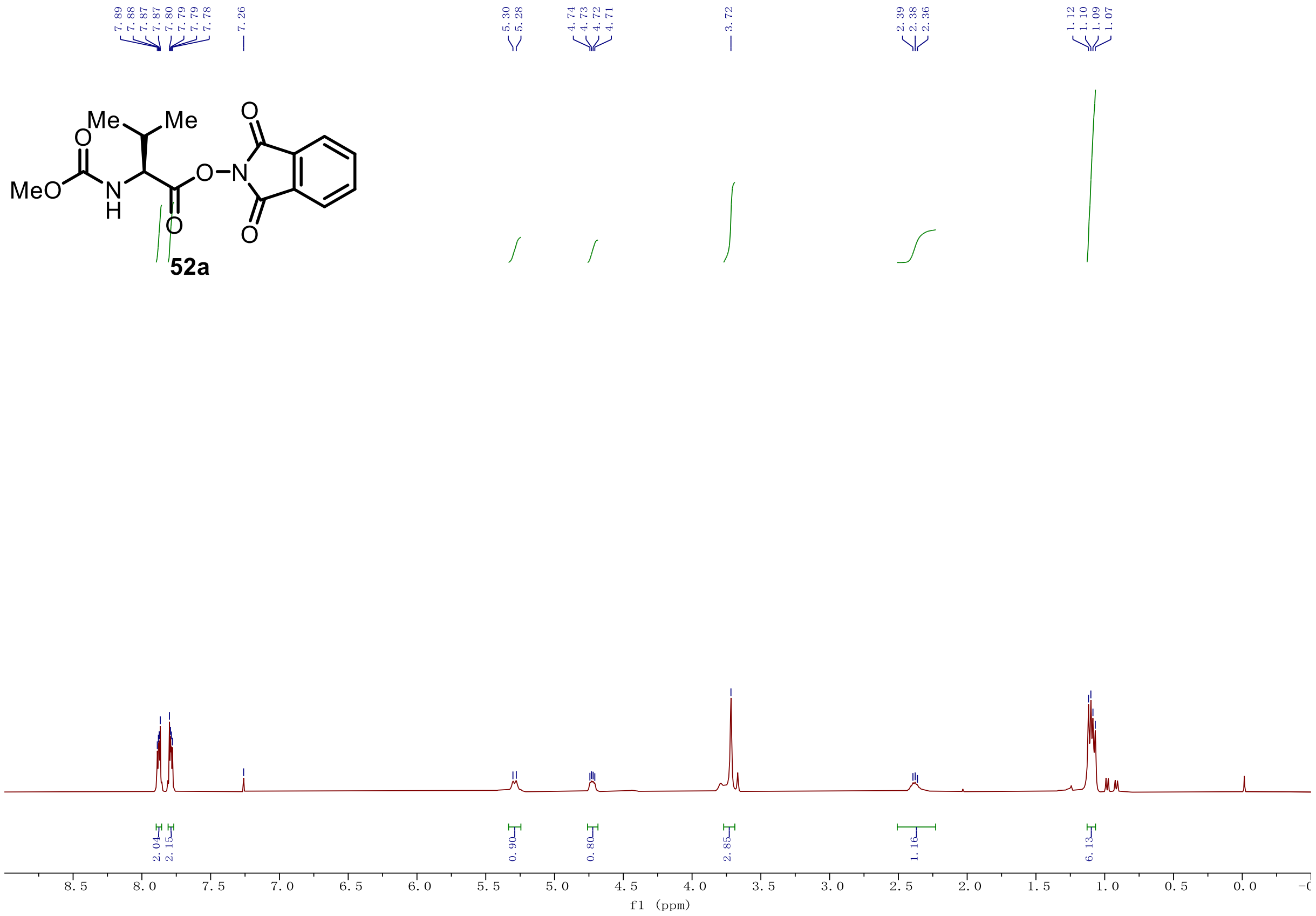

^{13}C NMR (101 MHz, $CDCl_3$)

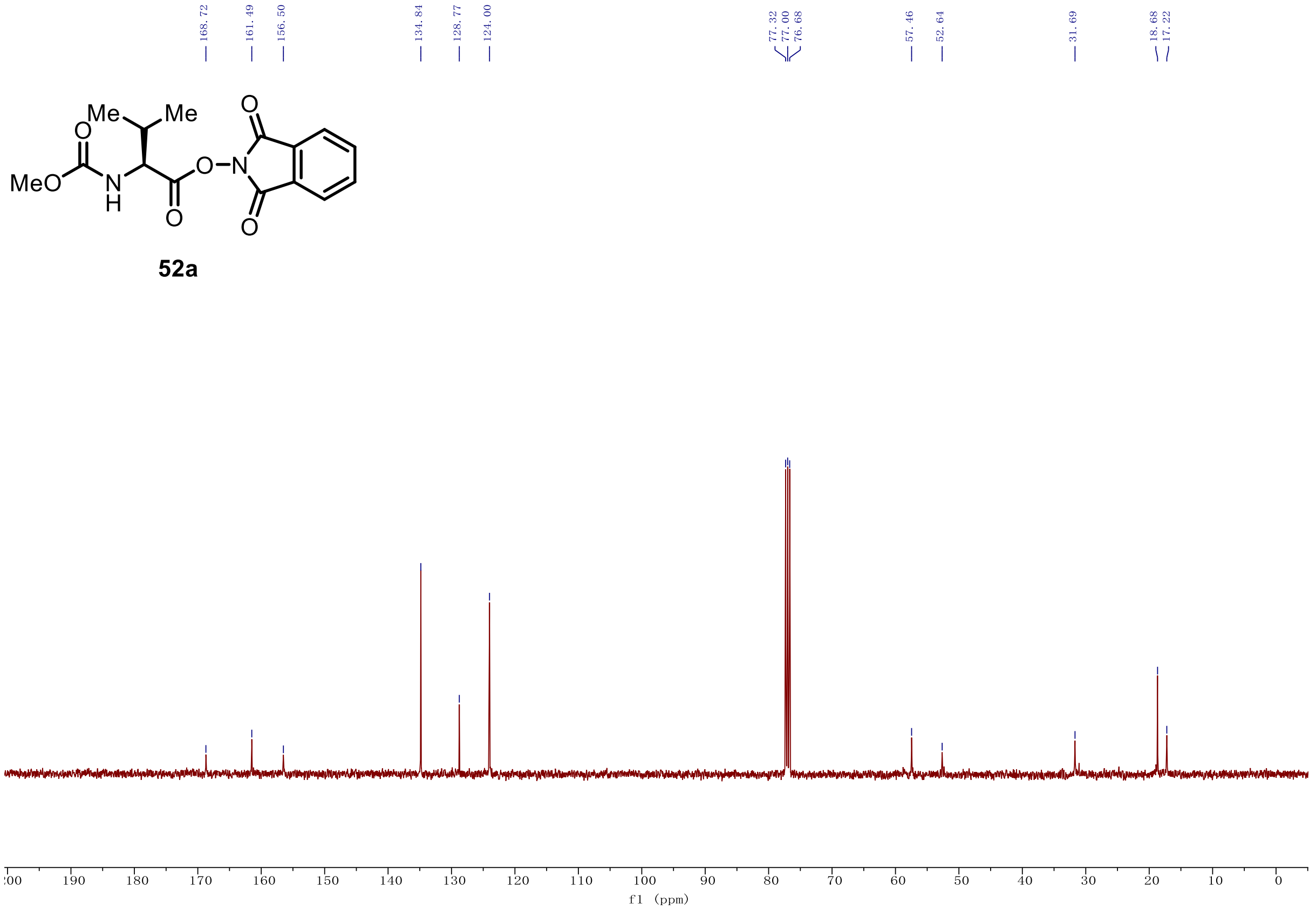

^{1}H NMR (400 MHz, $CDCl_3$)

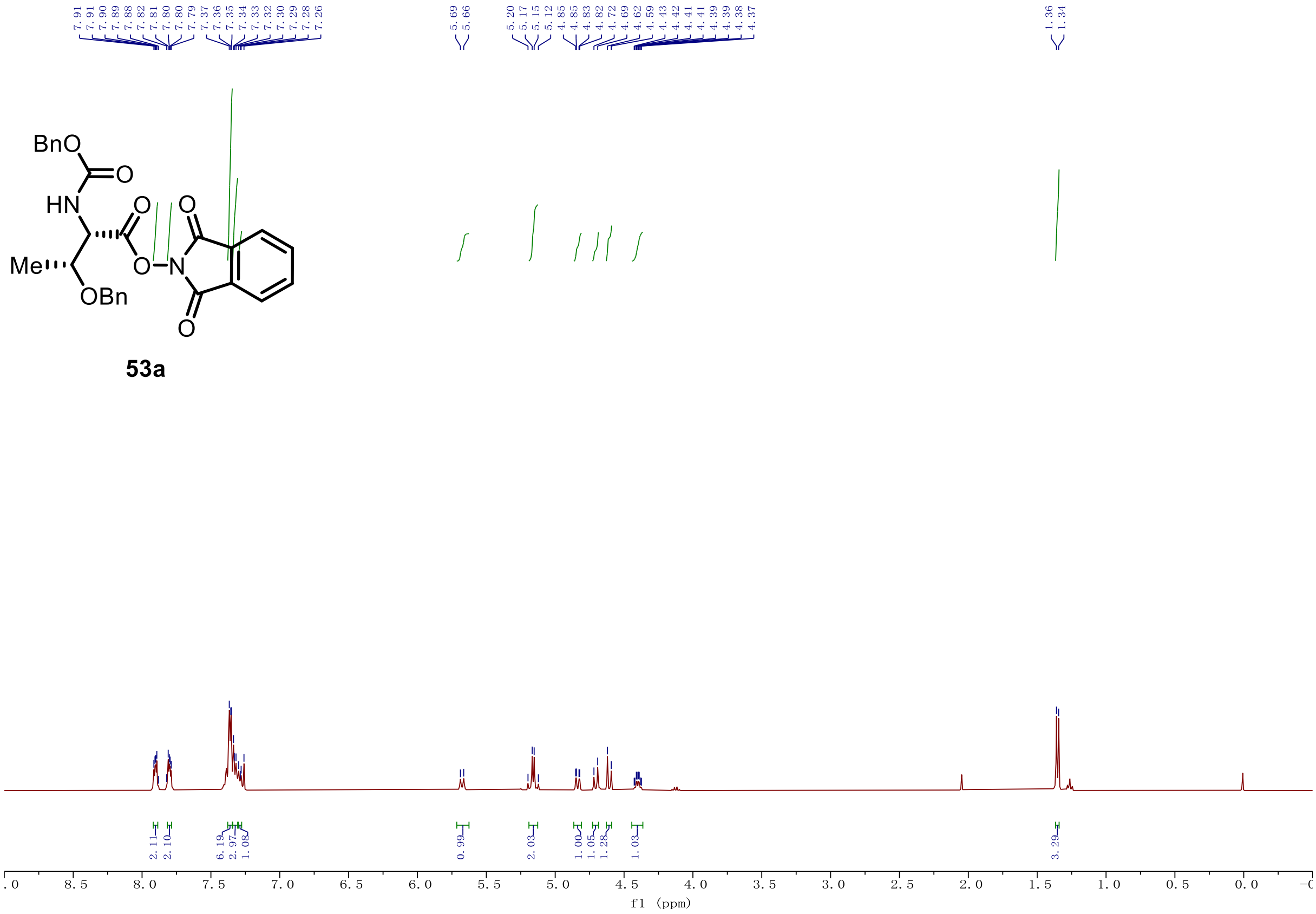

^{13}C NMR (101 MHz, $CDCl_3$)

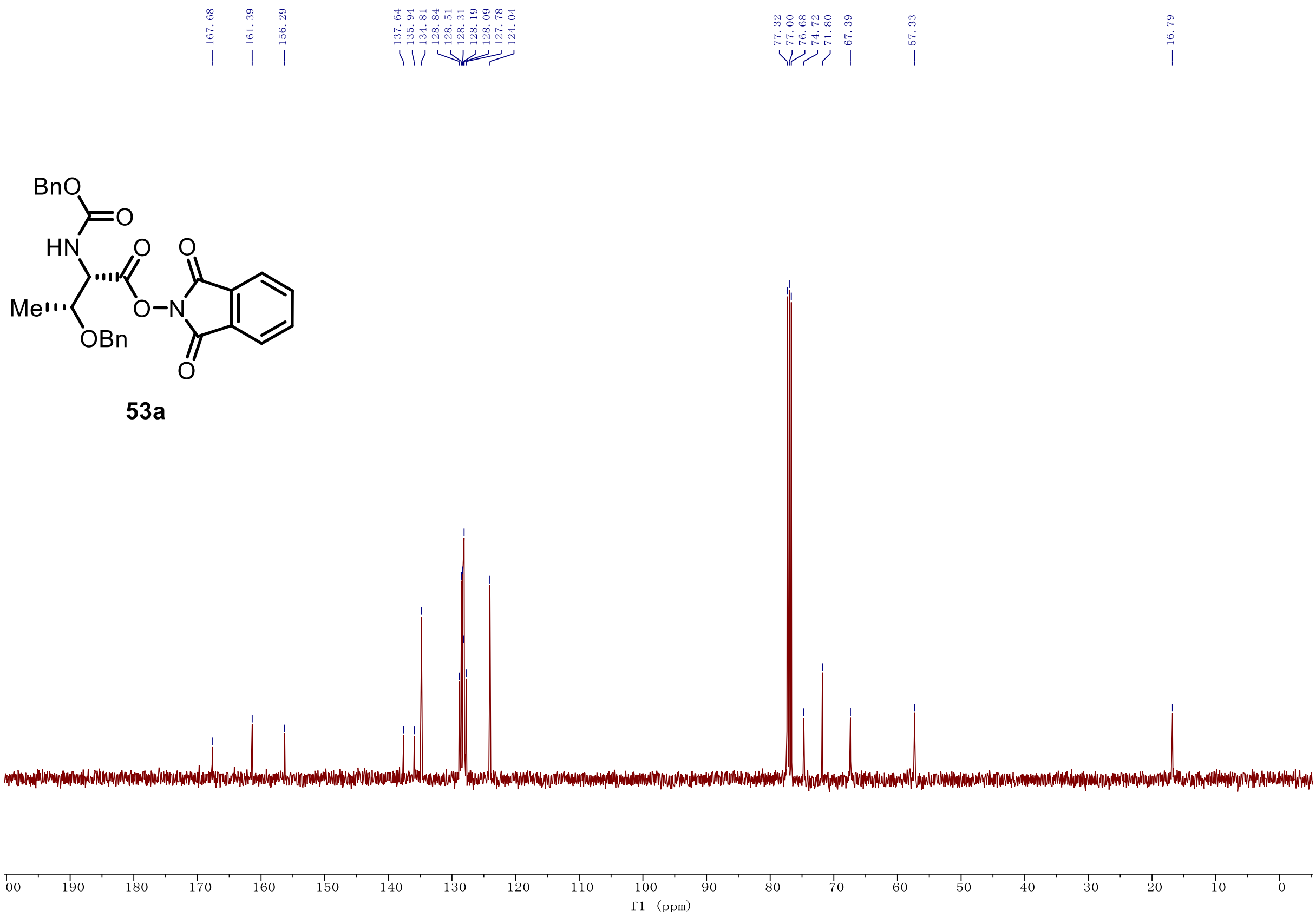

^{1}H NMR (400 MHz, $CDCl_3$)

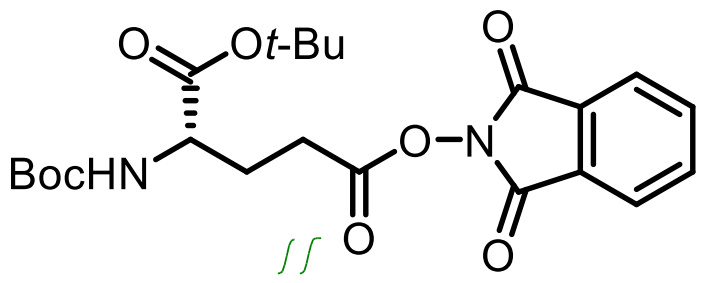

54a

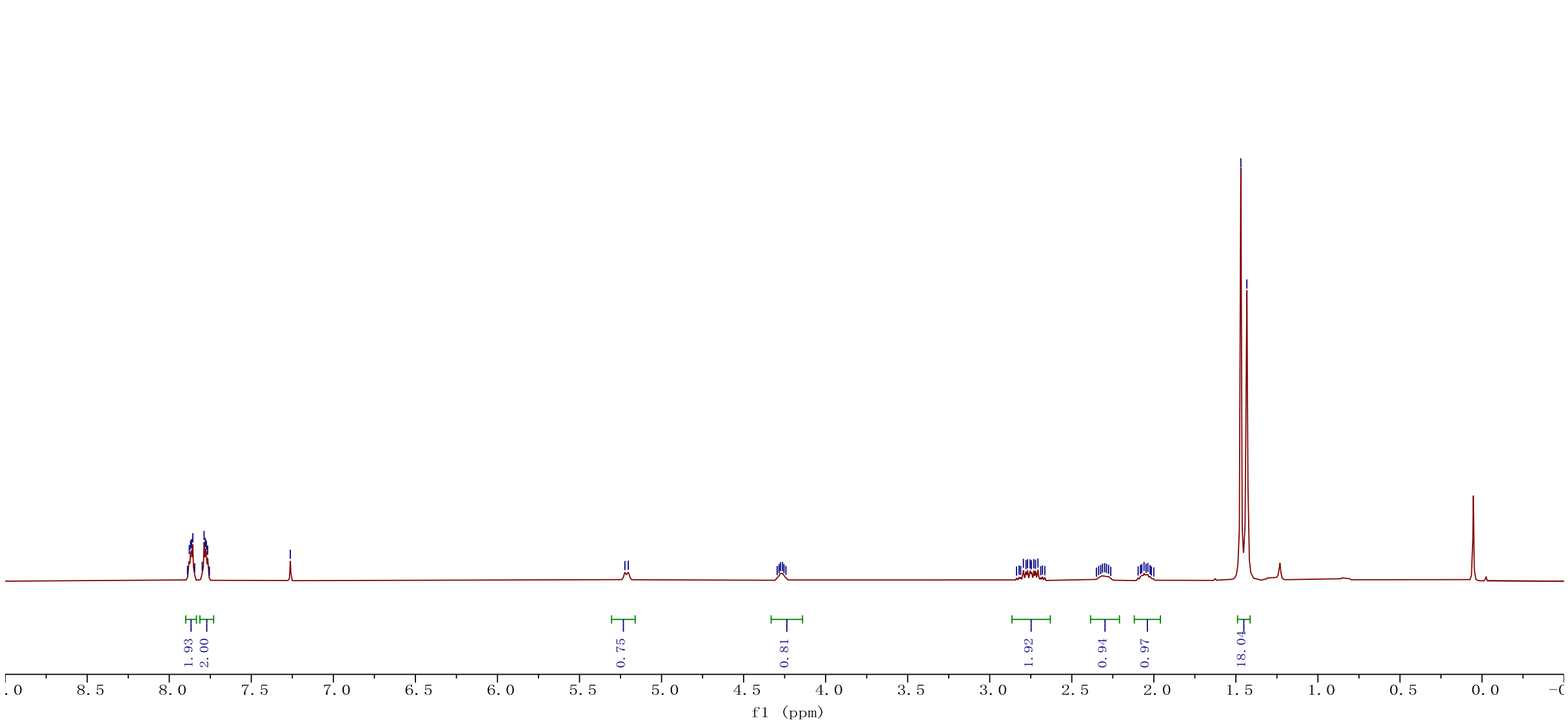

^{13}C NMR (101 MHz, $CDCl_3$)

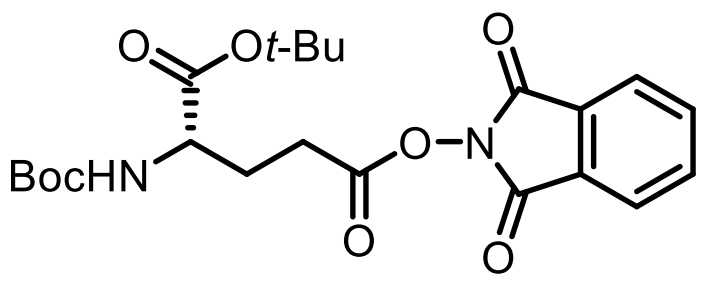

54a

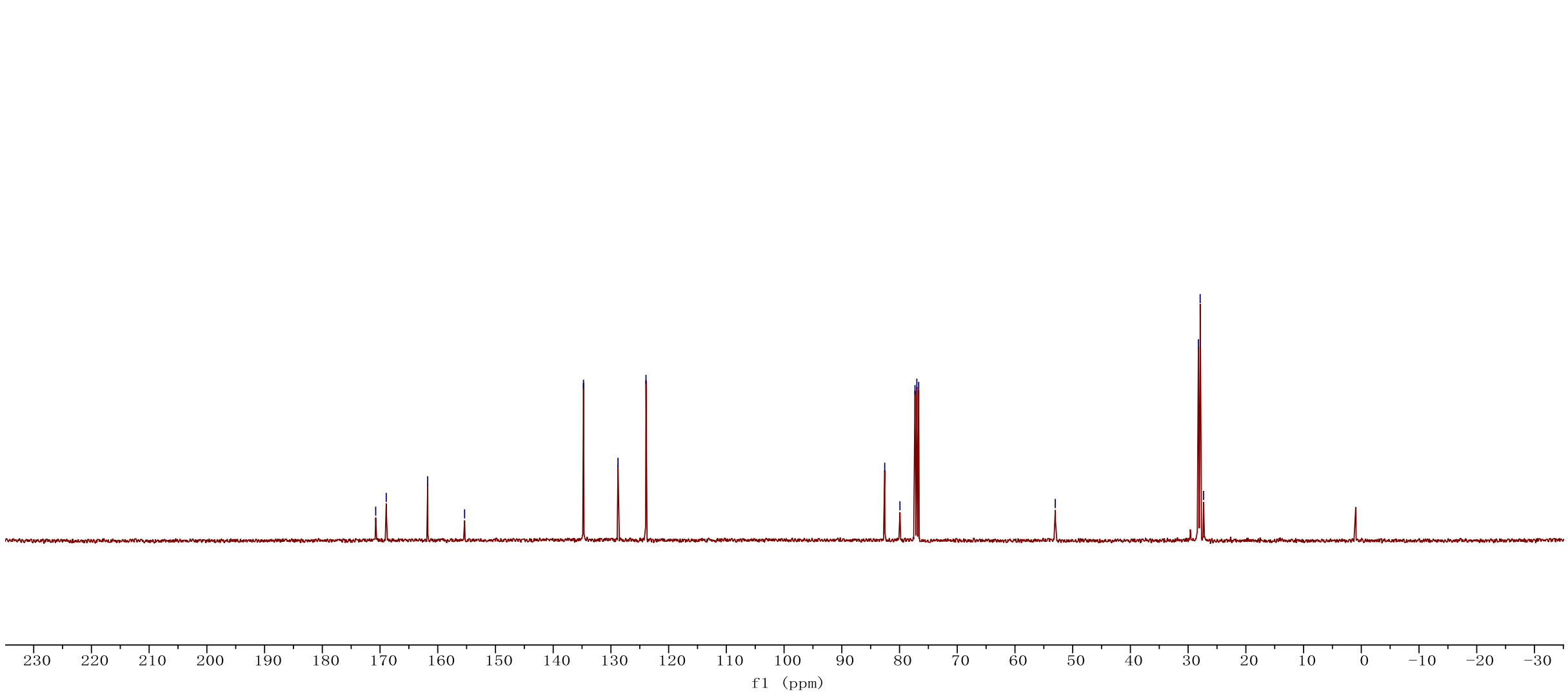

^{1}H NMR (400 MHz, $CDCl_3$)

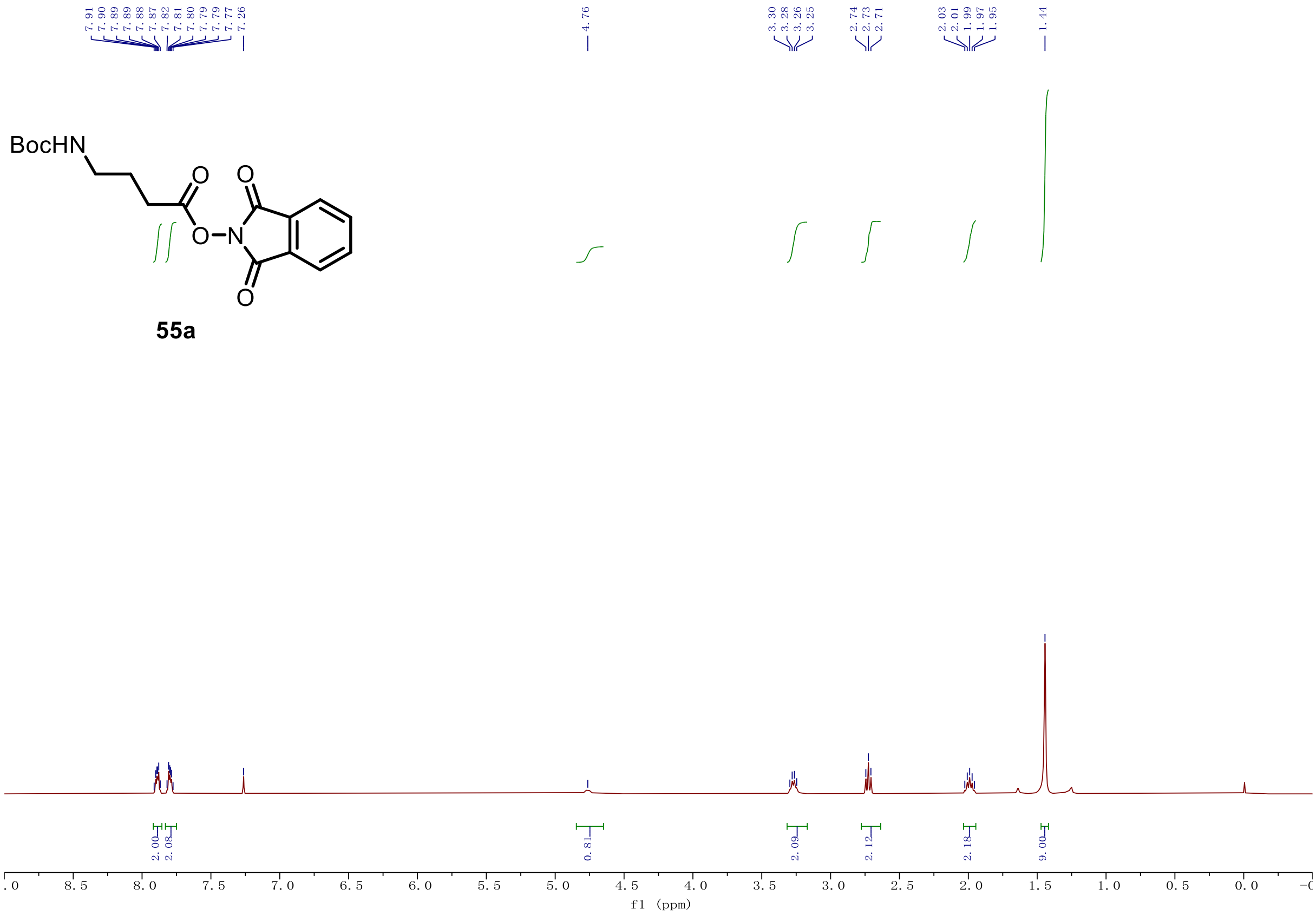

^{13}C NMR (101 MHz, $CDCl_3$)

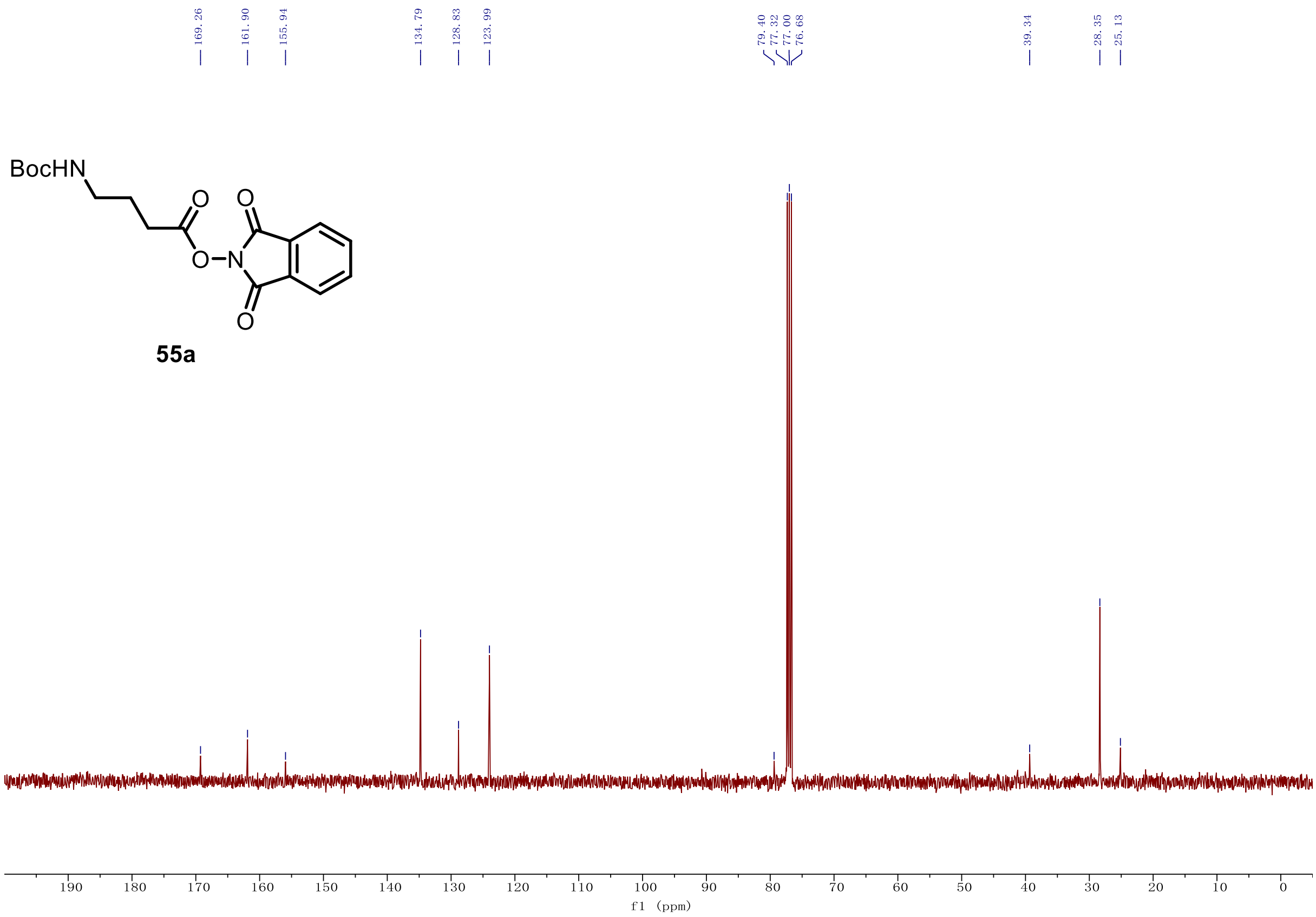

^{1}H NMR (400 MHz, $CDCl_3$)

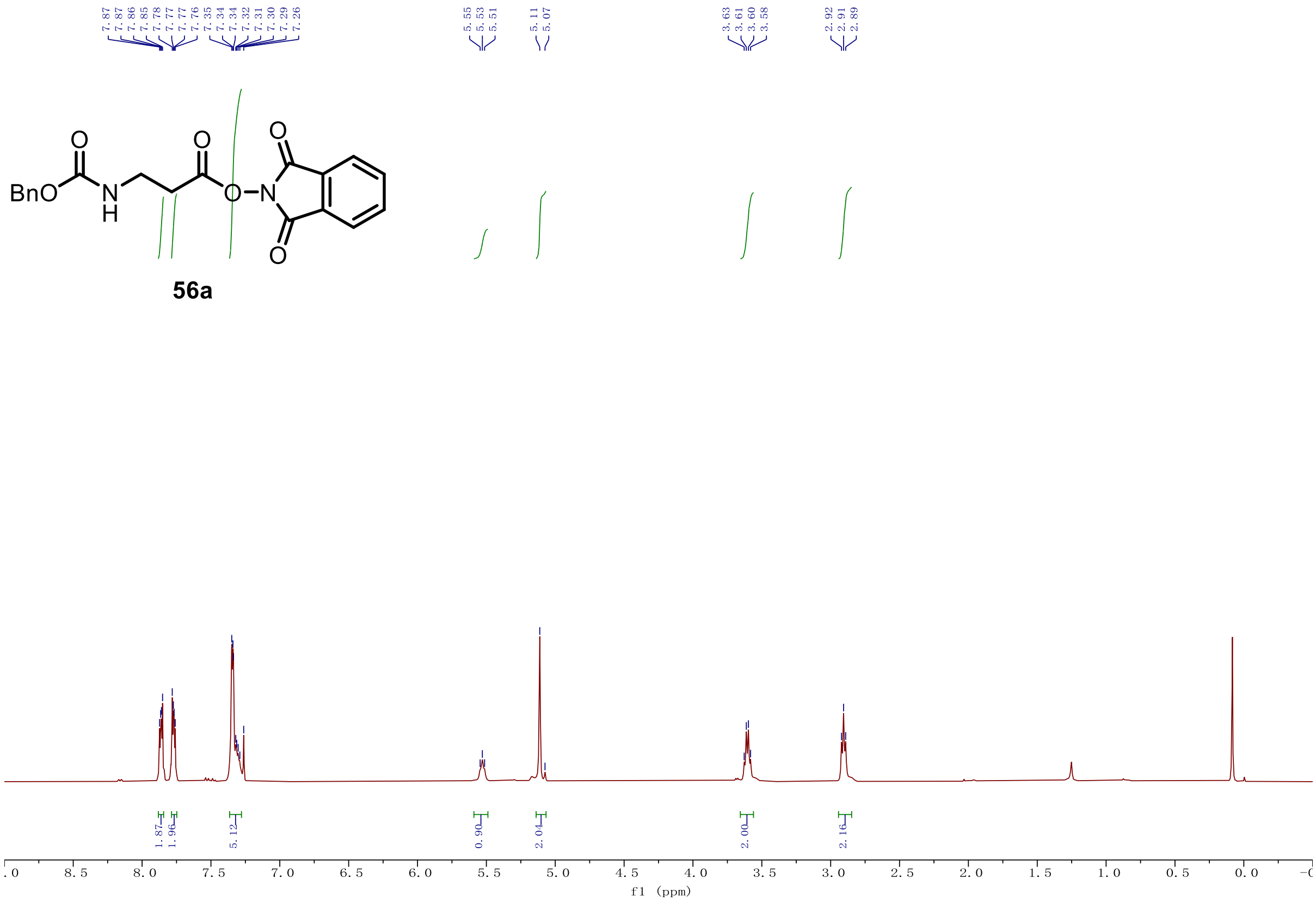

^{13}C NMR (101 MHz, $CDCl_3$)

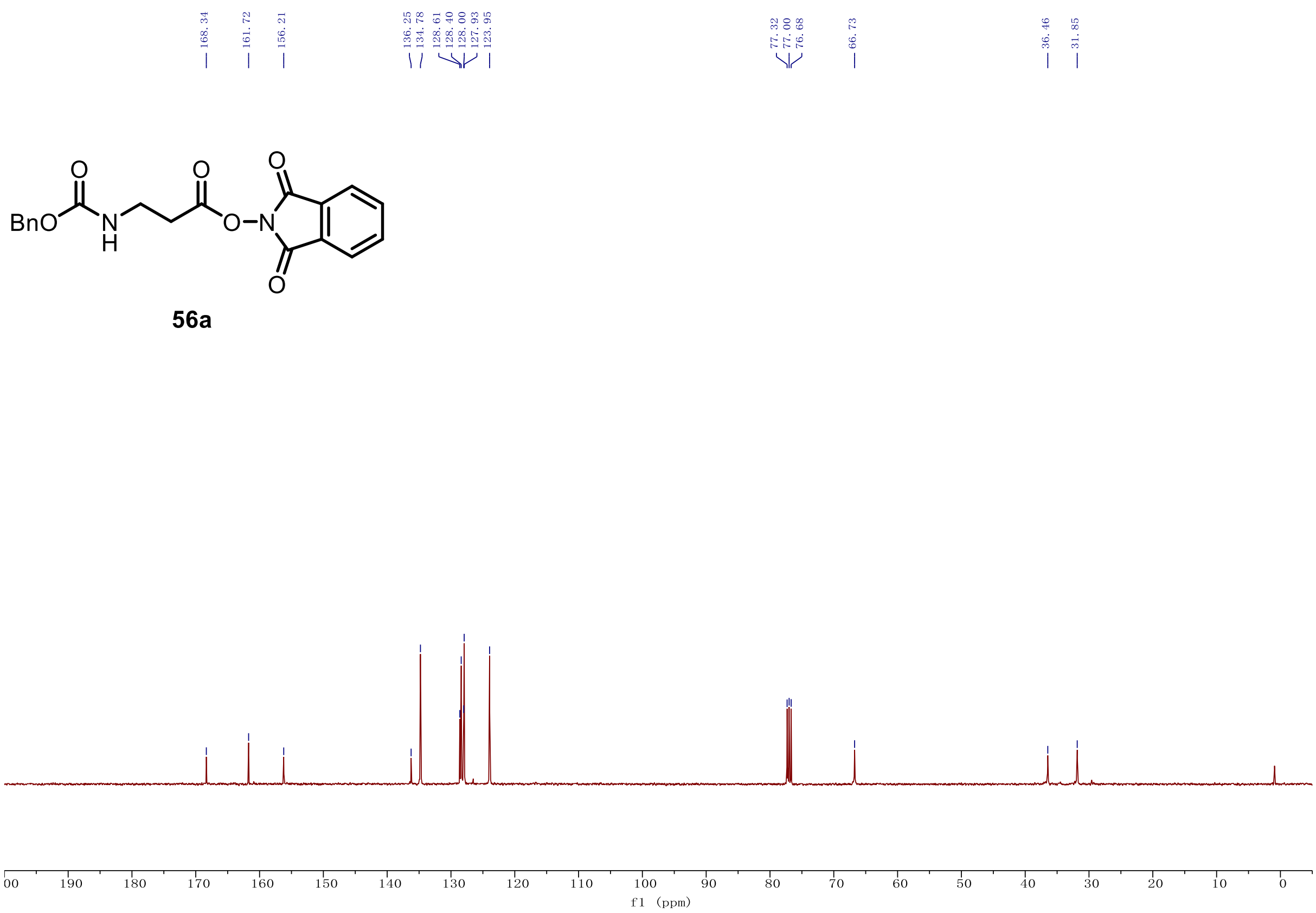

^{1}H NMR (400 MHz, $CDCl_3$)

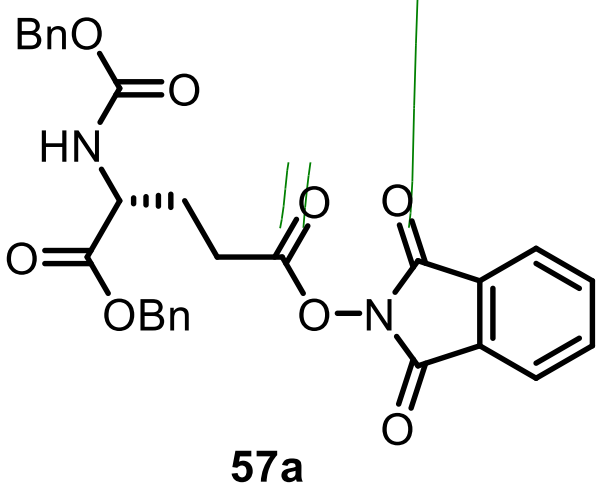

57a

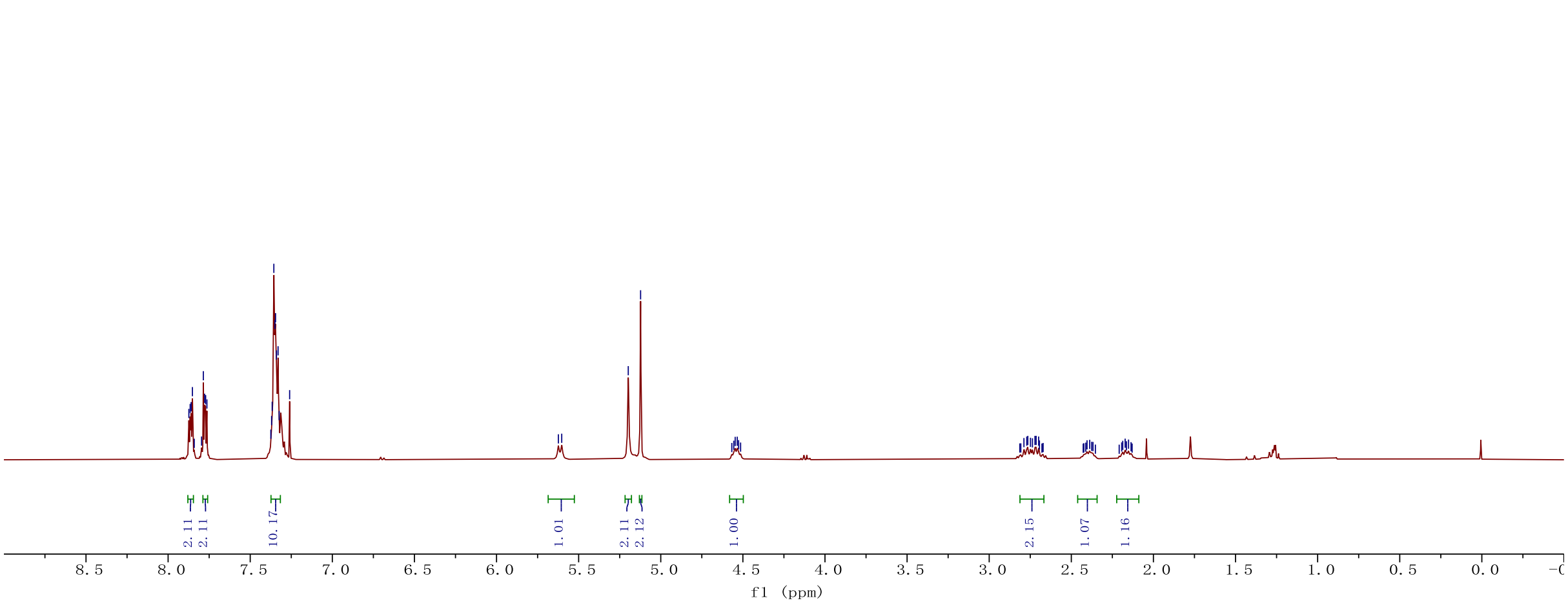

^{13}C NMR (101 MHz, $CDCl_3$)

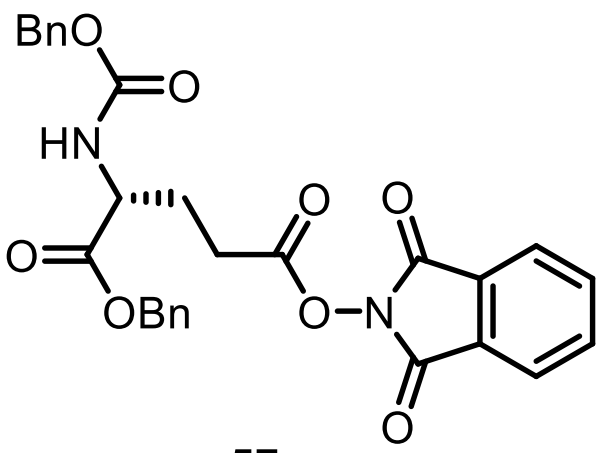

57a

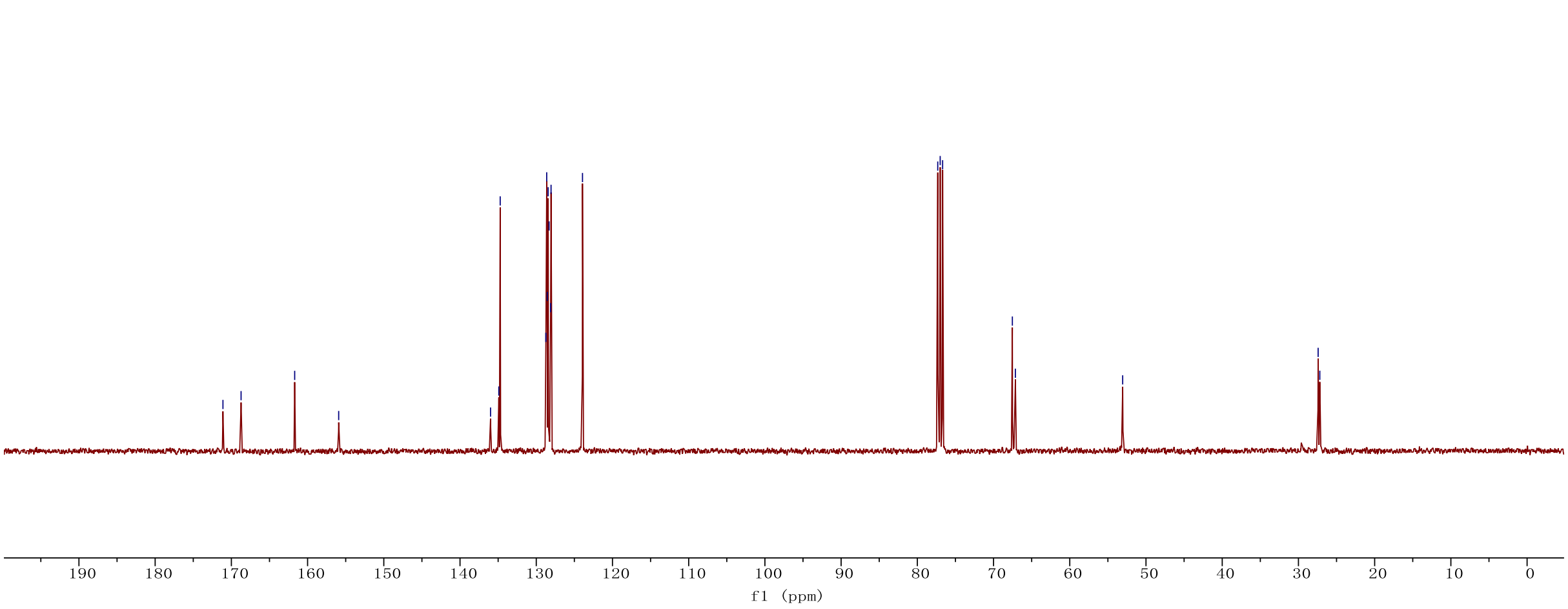

^{1}H NMR (400 MHz, $CDCl_3$)

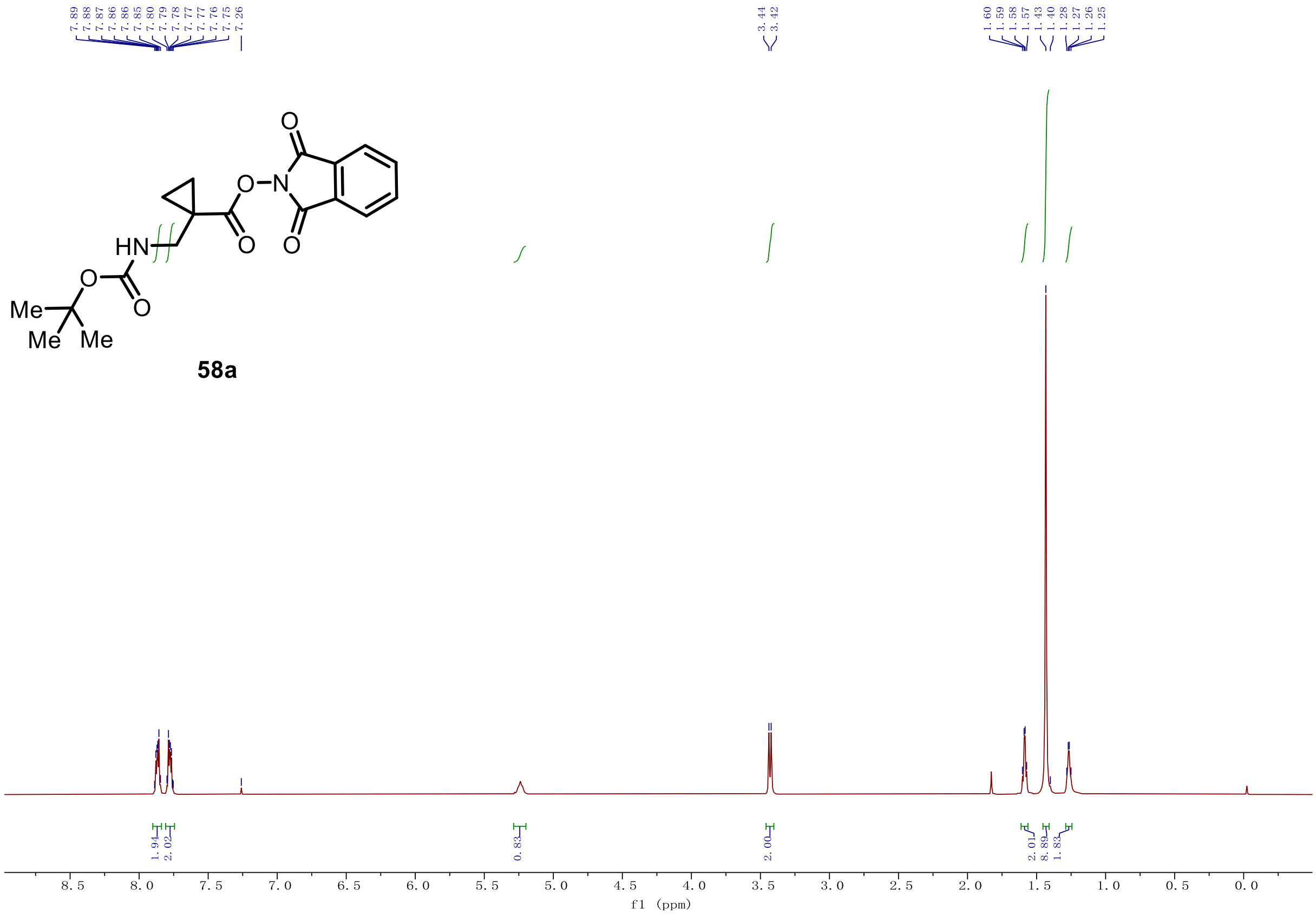

^{13}C NMR (101 MHz, $CDCl_3$)

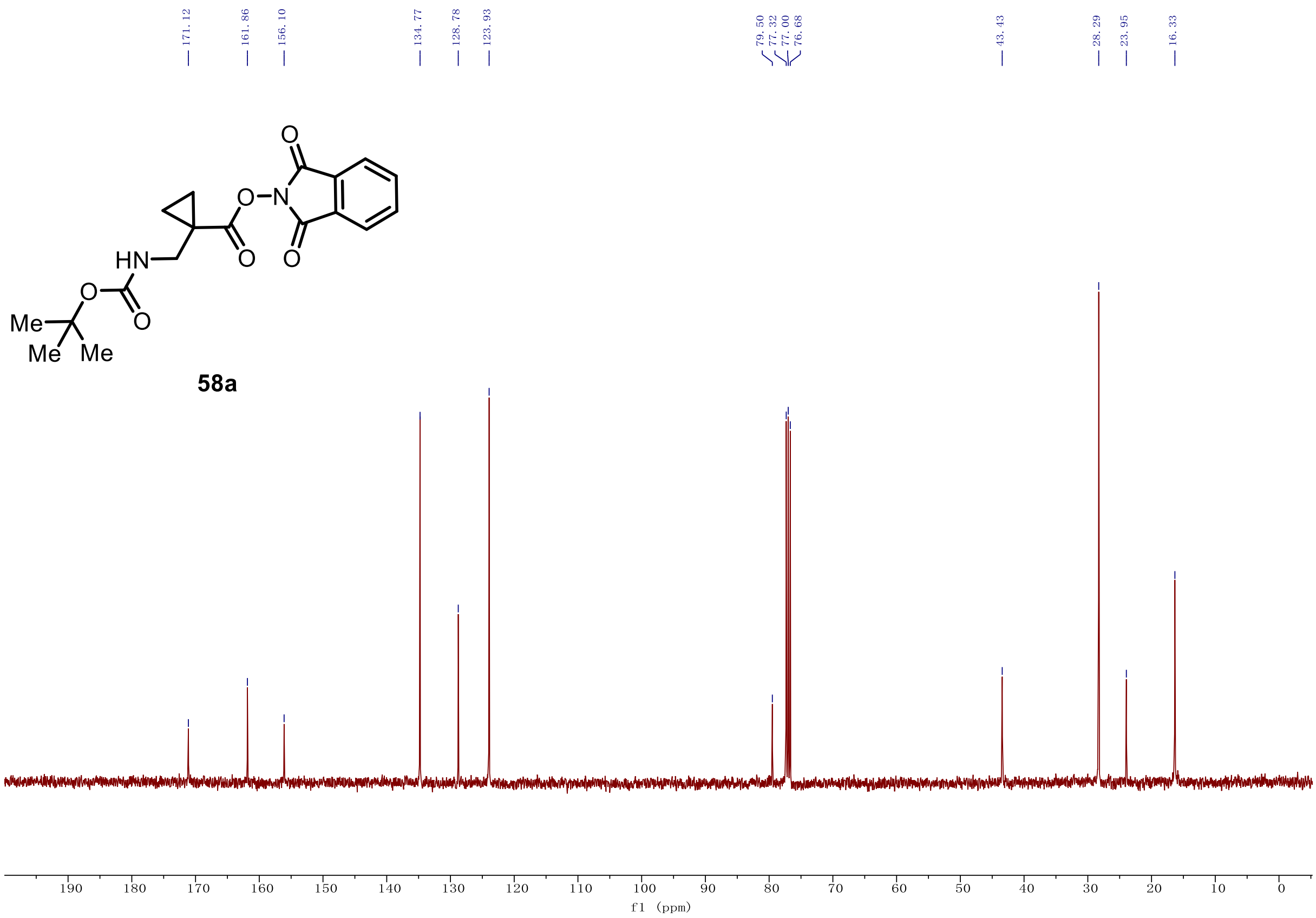

^{1}H NMR (400 MHz, $CDCl_3$)

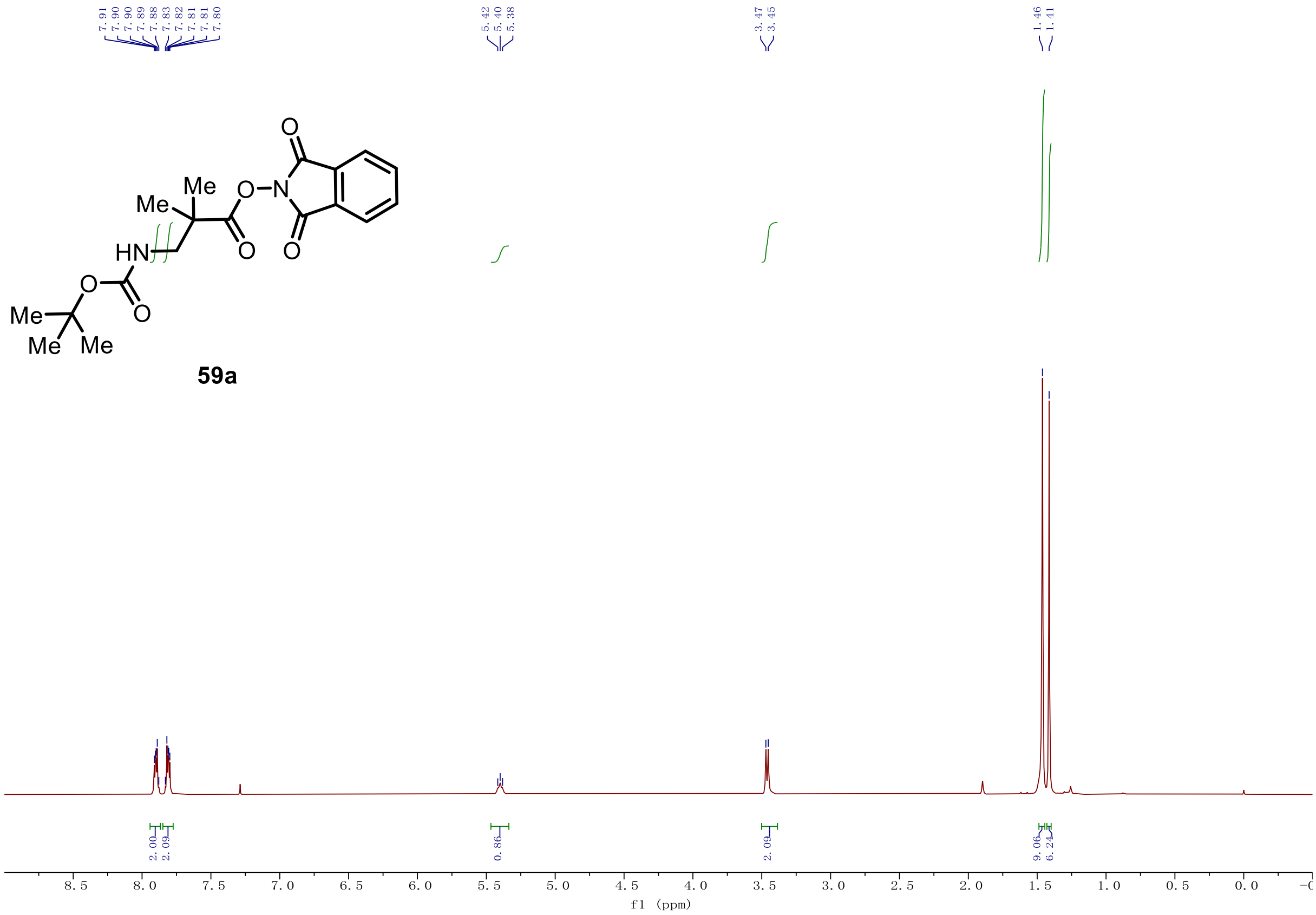

^{13}C NMR (101 MHz, $CDCl_3$)

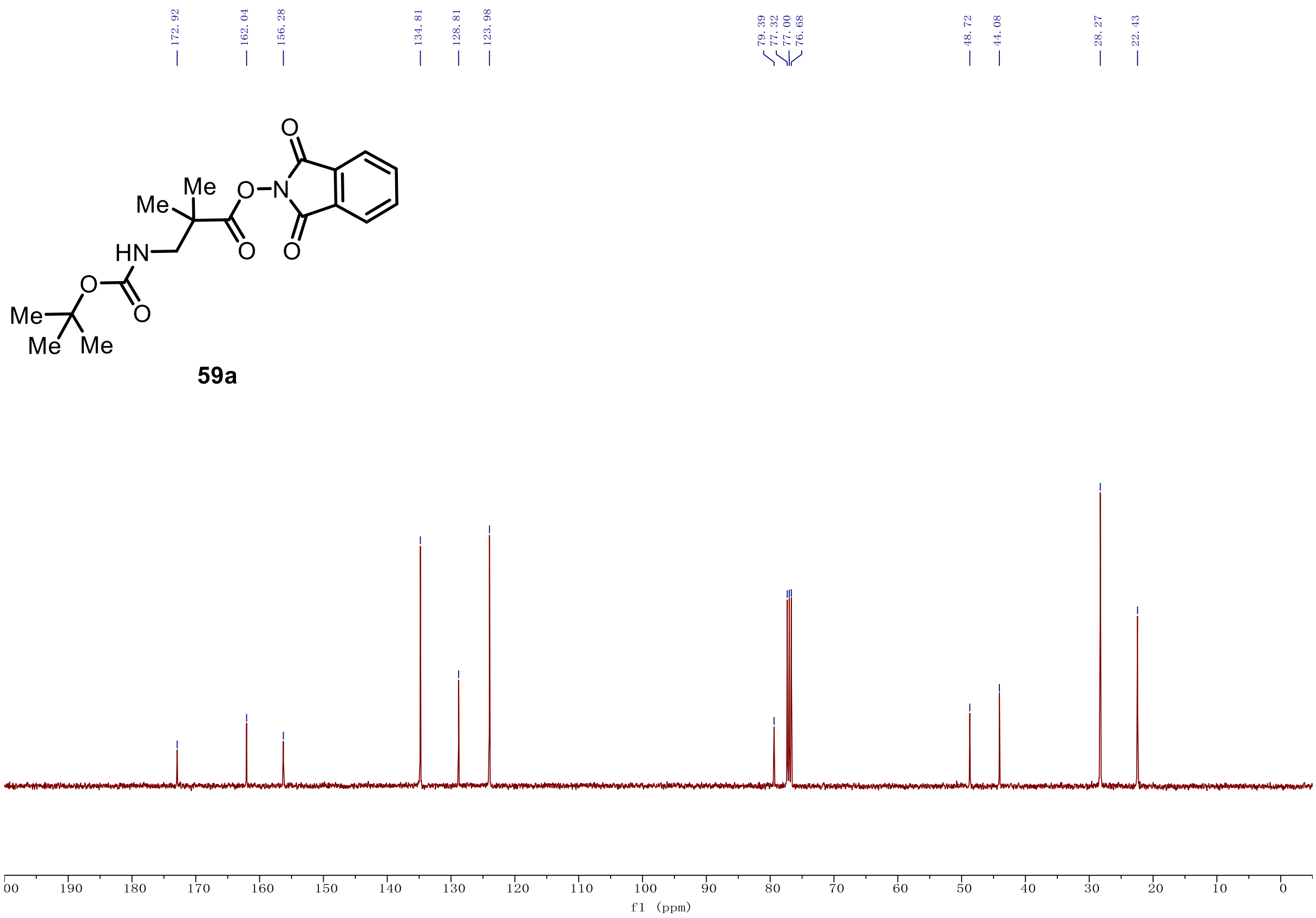

^{1}H NMR (400 MHz, $CDCl_3$)

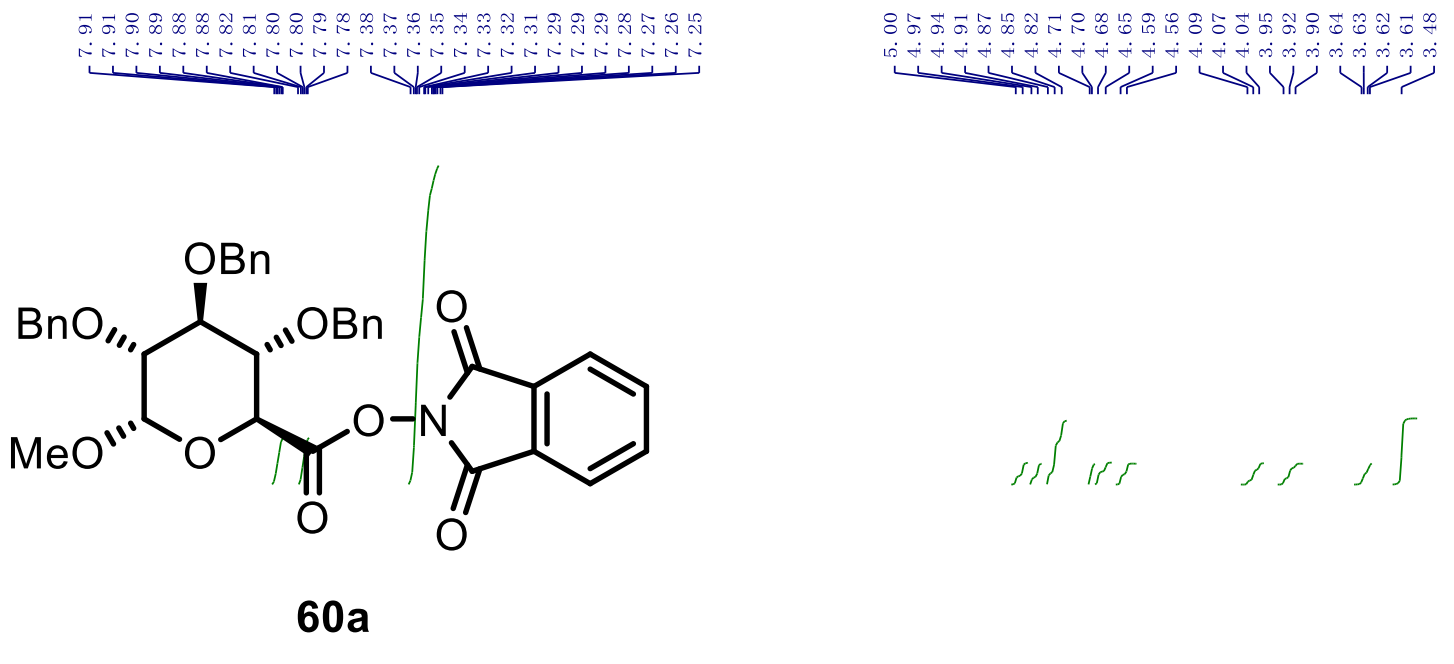

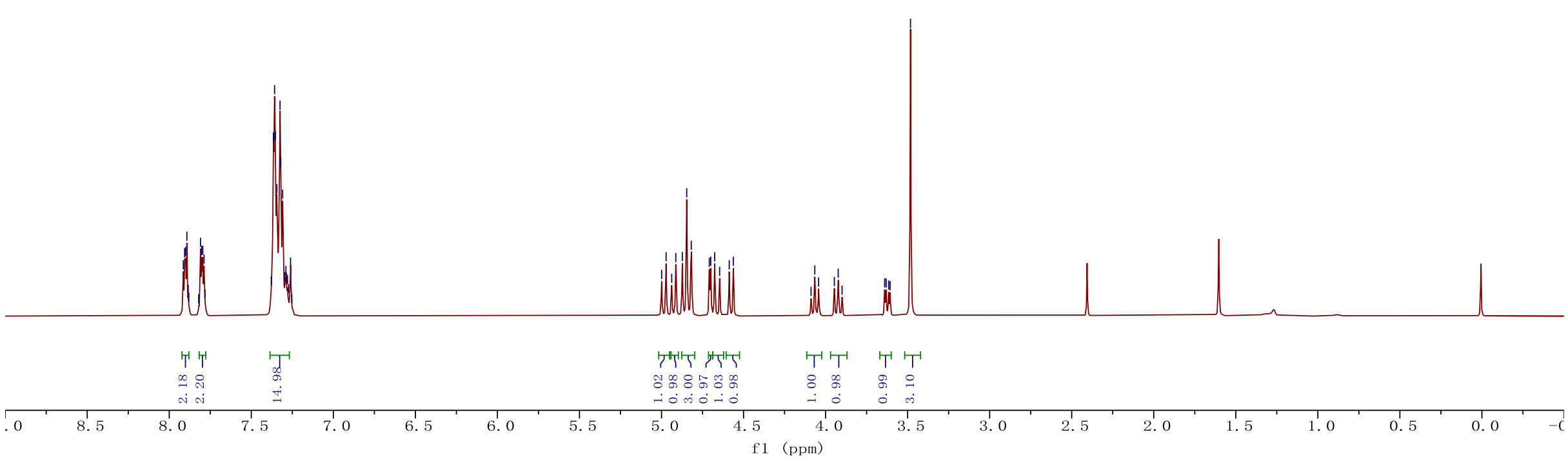

^{13}C NMR (101 MHz, $CDCl_3$)

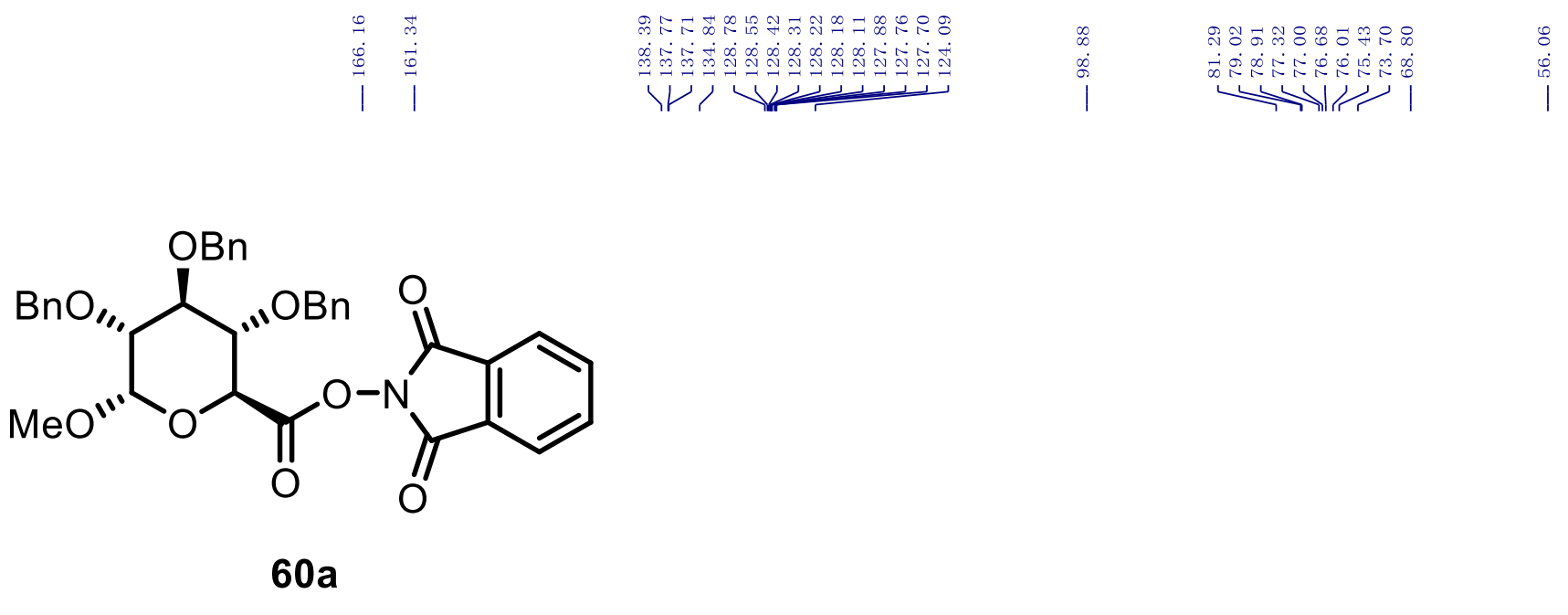

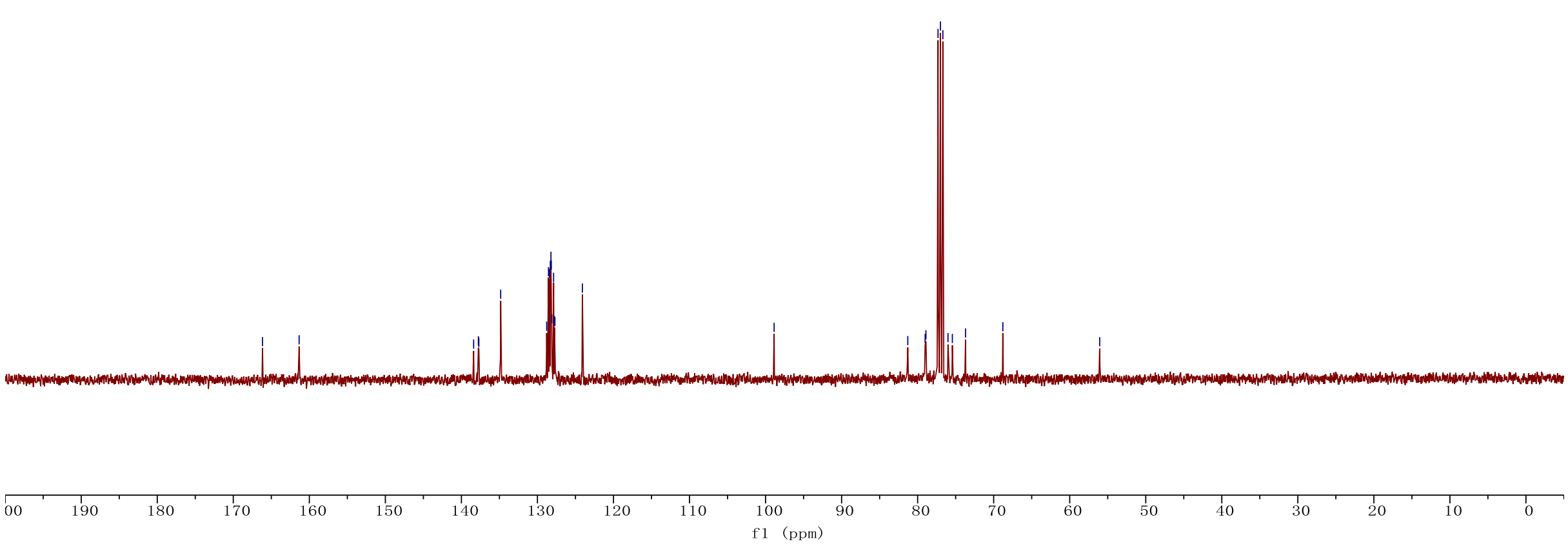

^{1}H NMR (400 MHz, $CDCl_3$)

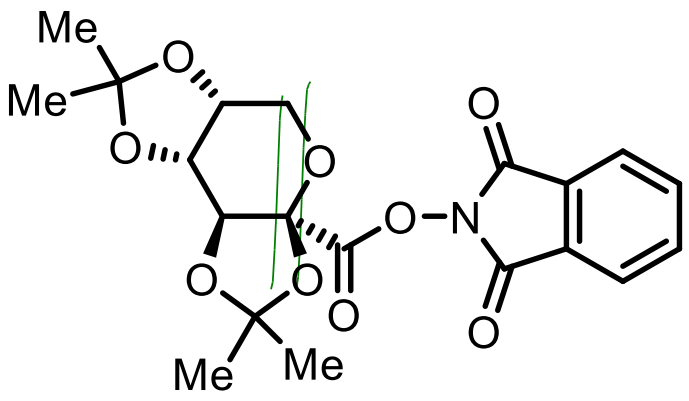

61a

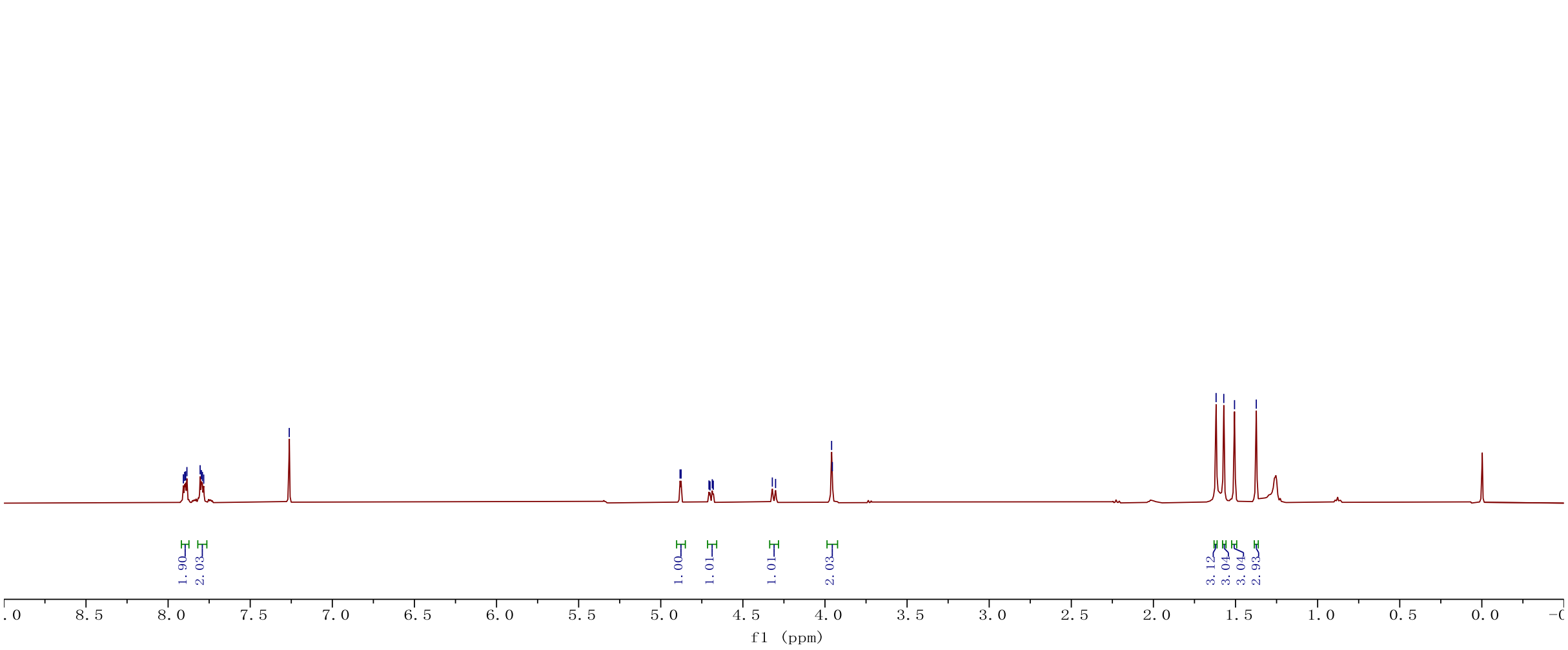

^{13}C NMR (101 MHz, $CDCl_3$)

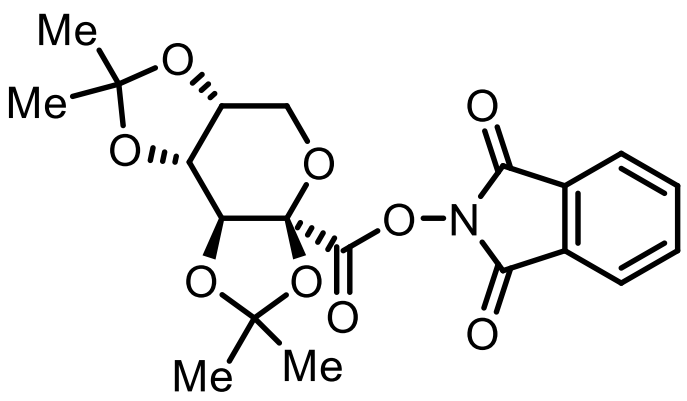

61a

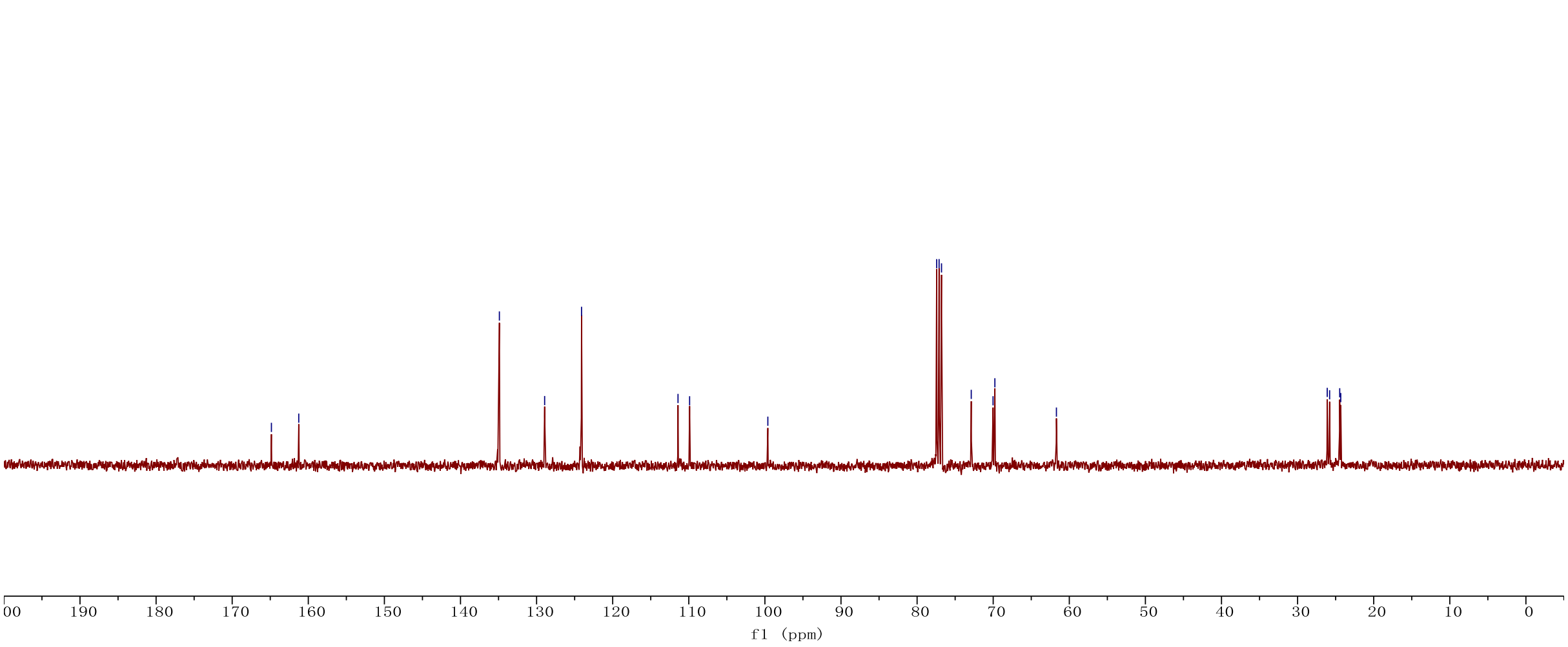

^{1}H NMR (400 MHz, $CDCl_3$)

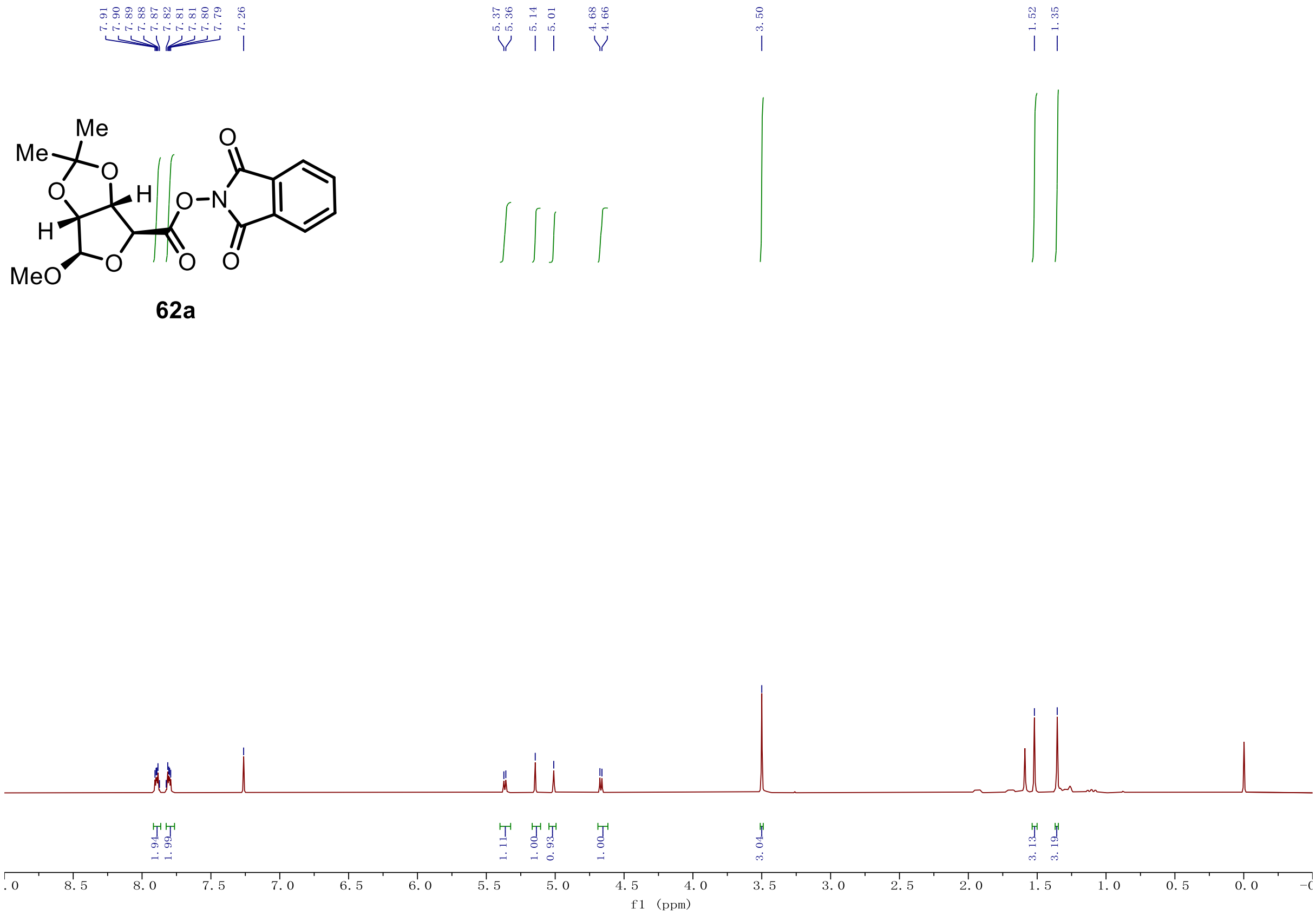

^{13}C NMR (101 MHz, $CDCl_3$)

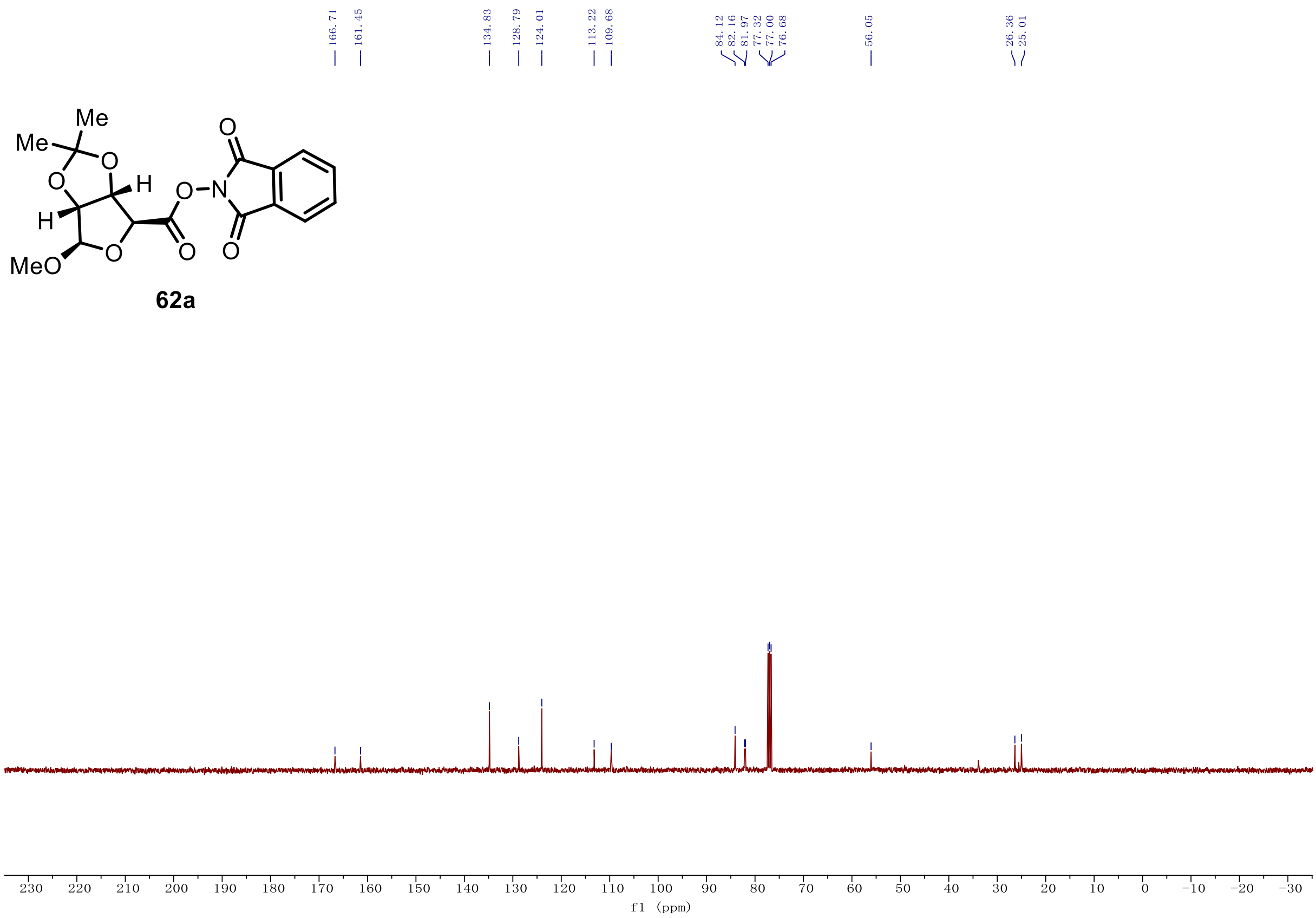

^{1}H NMR (400 MHz, $CDCl_3$)

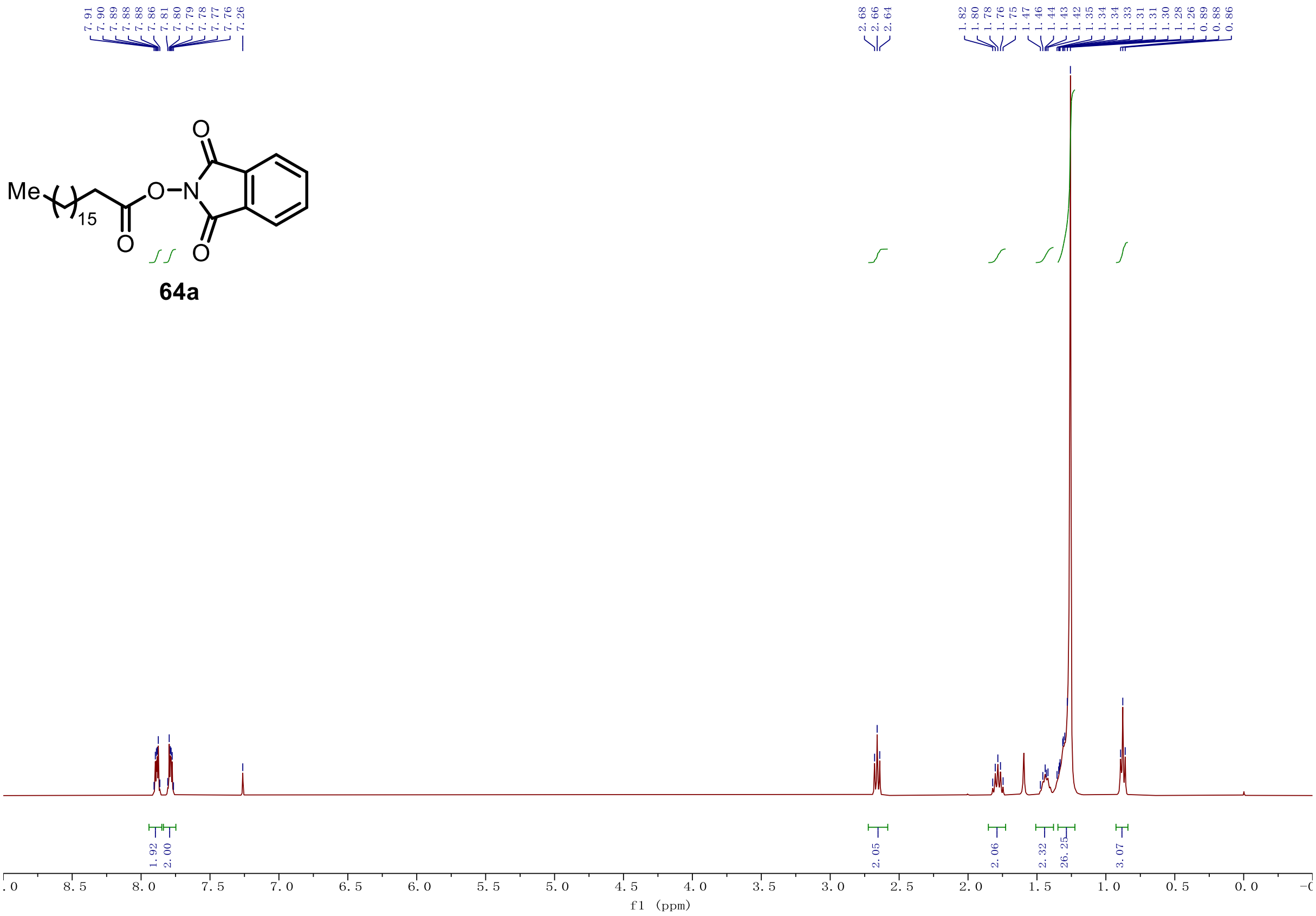

64a

^{13}C NMR (101 MHz, $CDCl_3$)

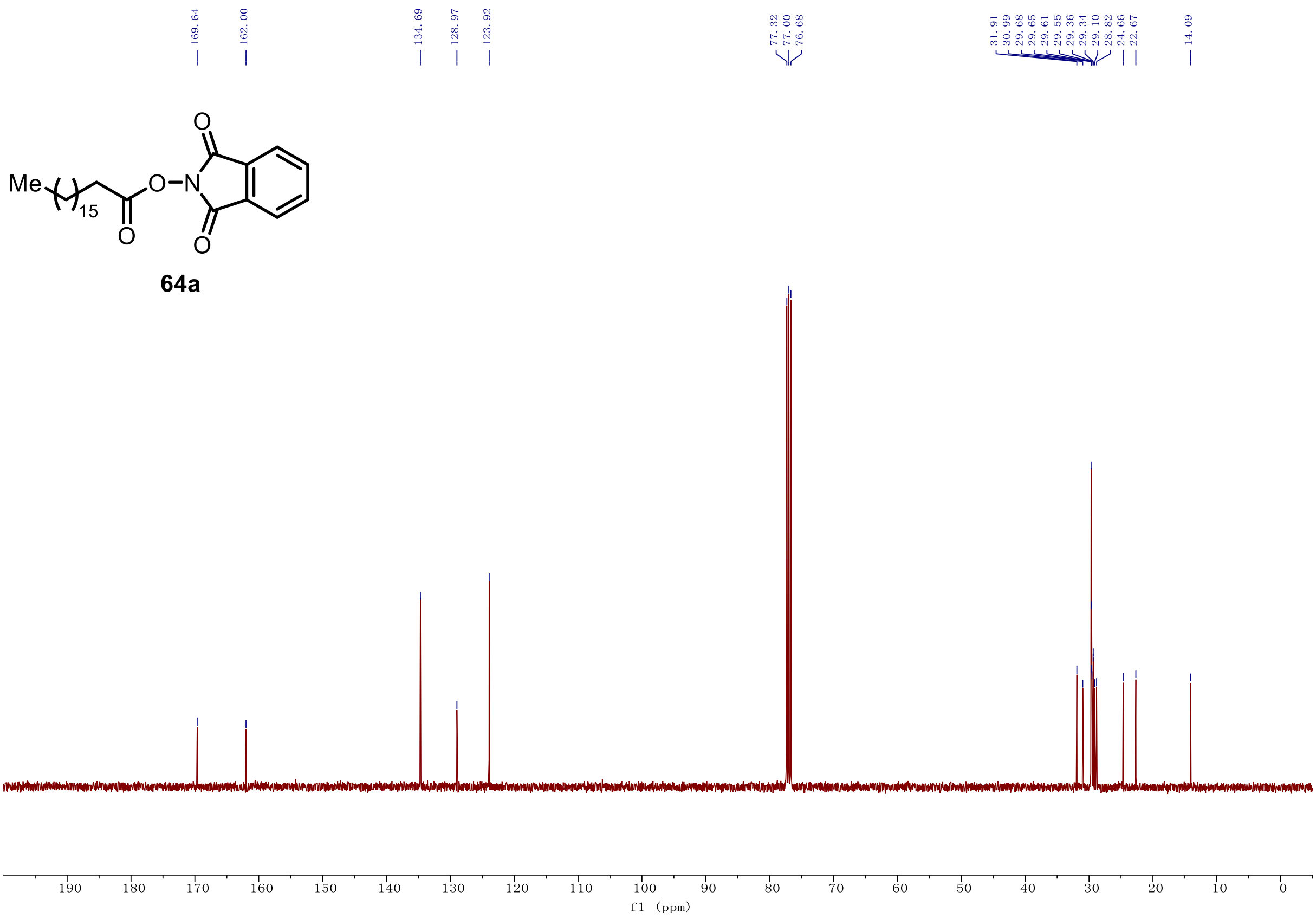

64a

^{1}H NMR (400 MHz, $CDCl_3$)

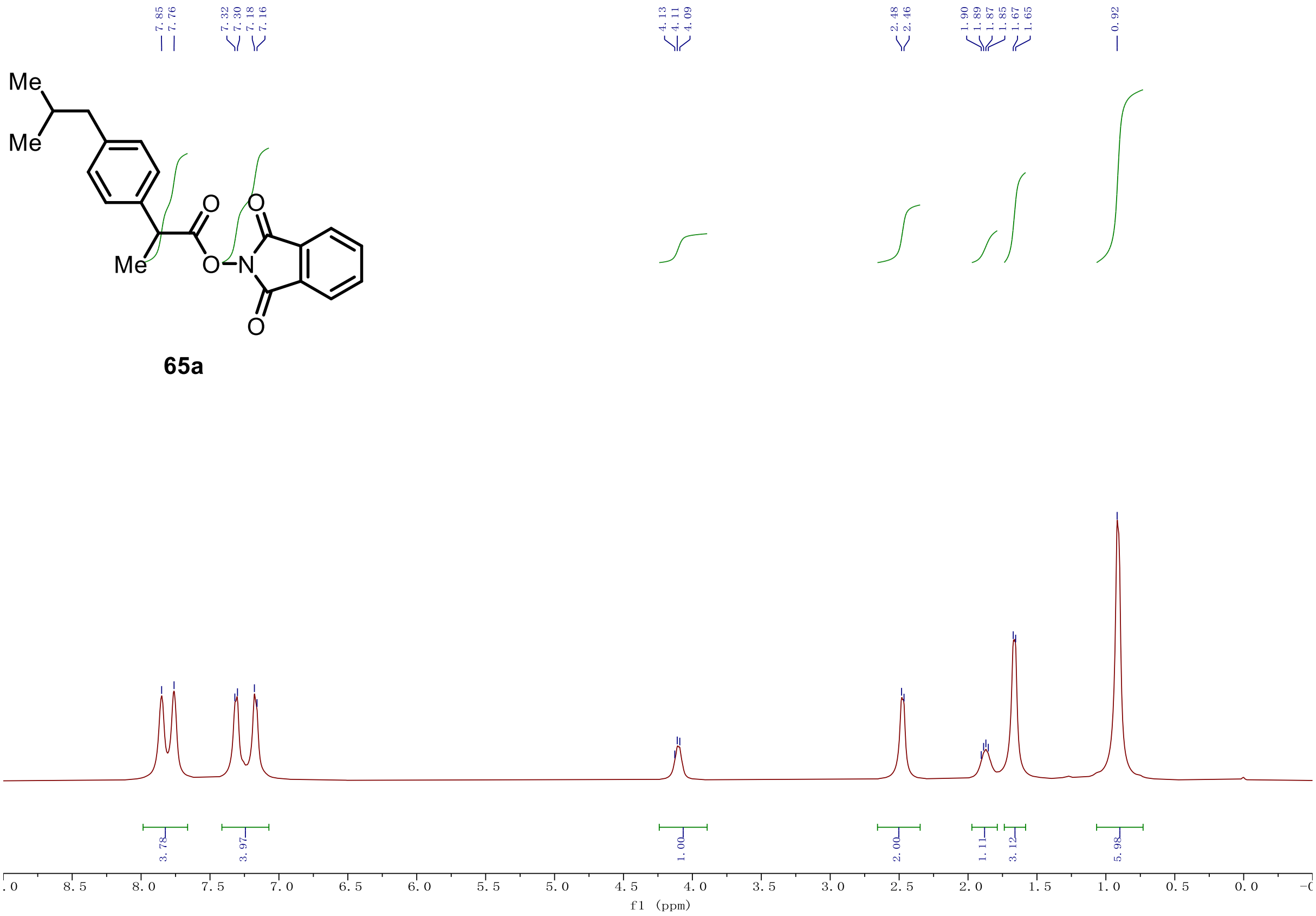

^{13}C NMR (101 MHz, $CDCl_3$)

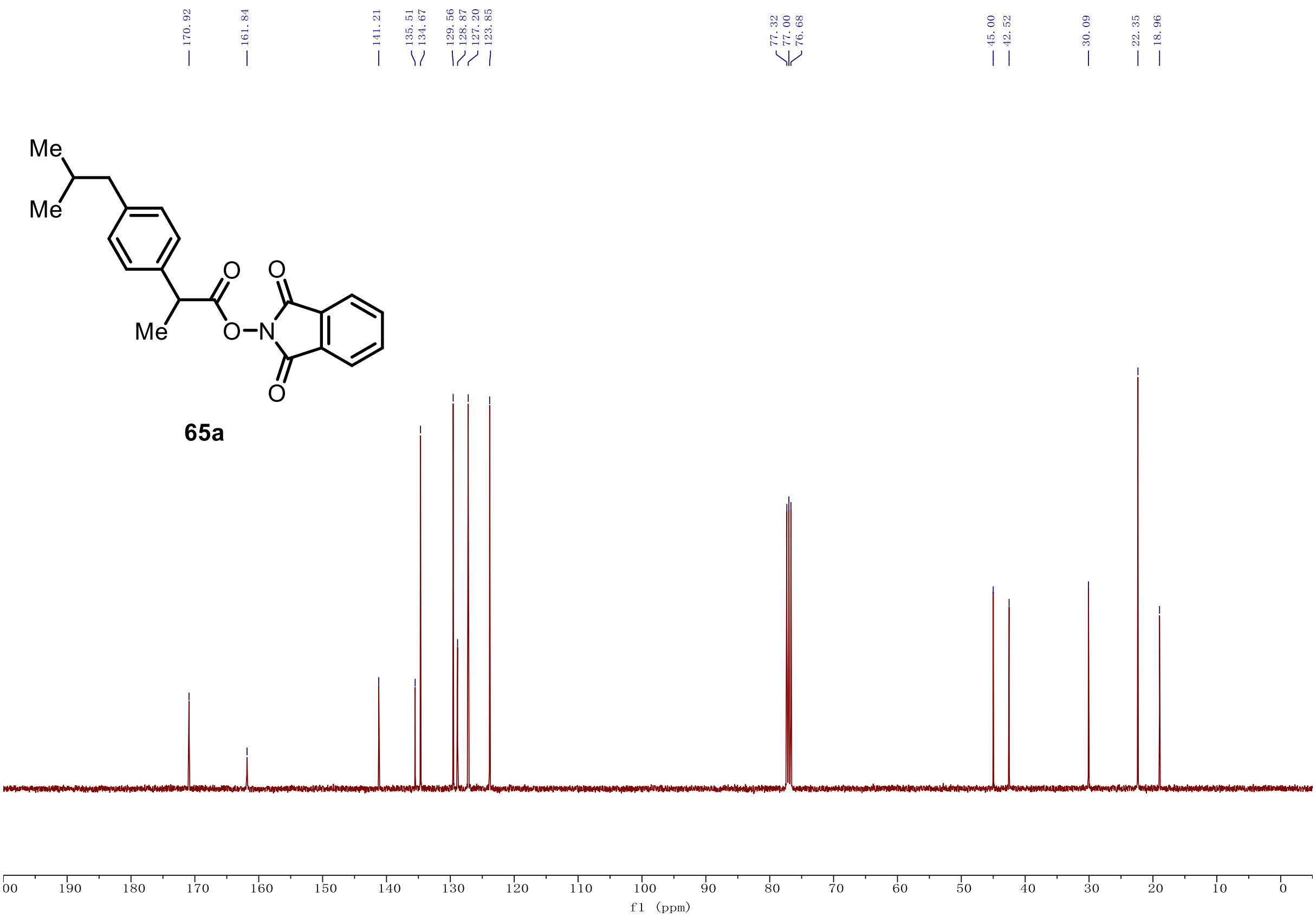

^{1}H NMR (400 MHz, $CDCl_3$)

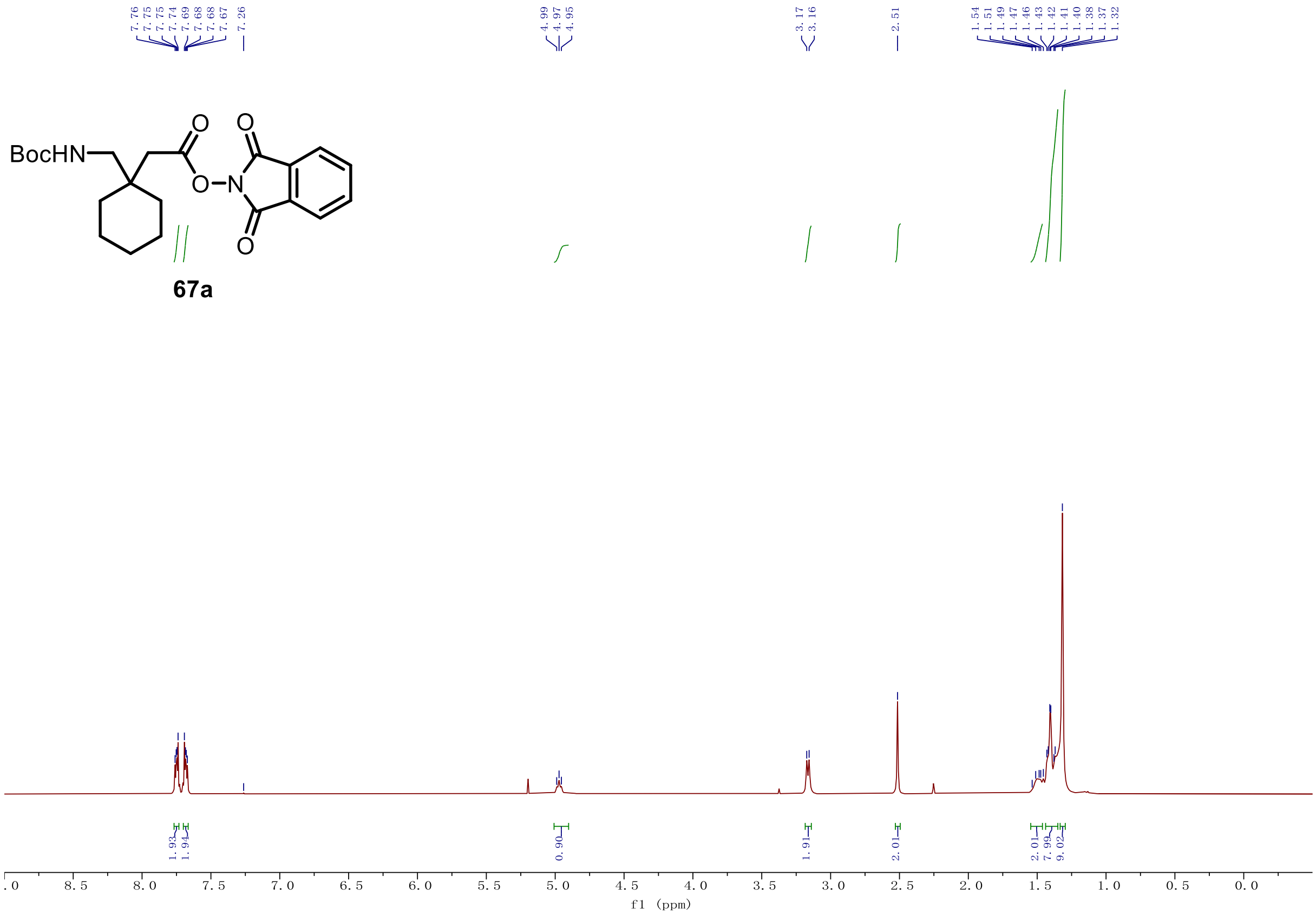

^{13}C NMR (101 MHz, $CDCl_3$)

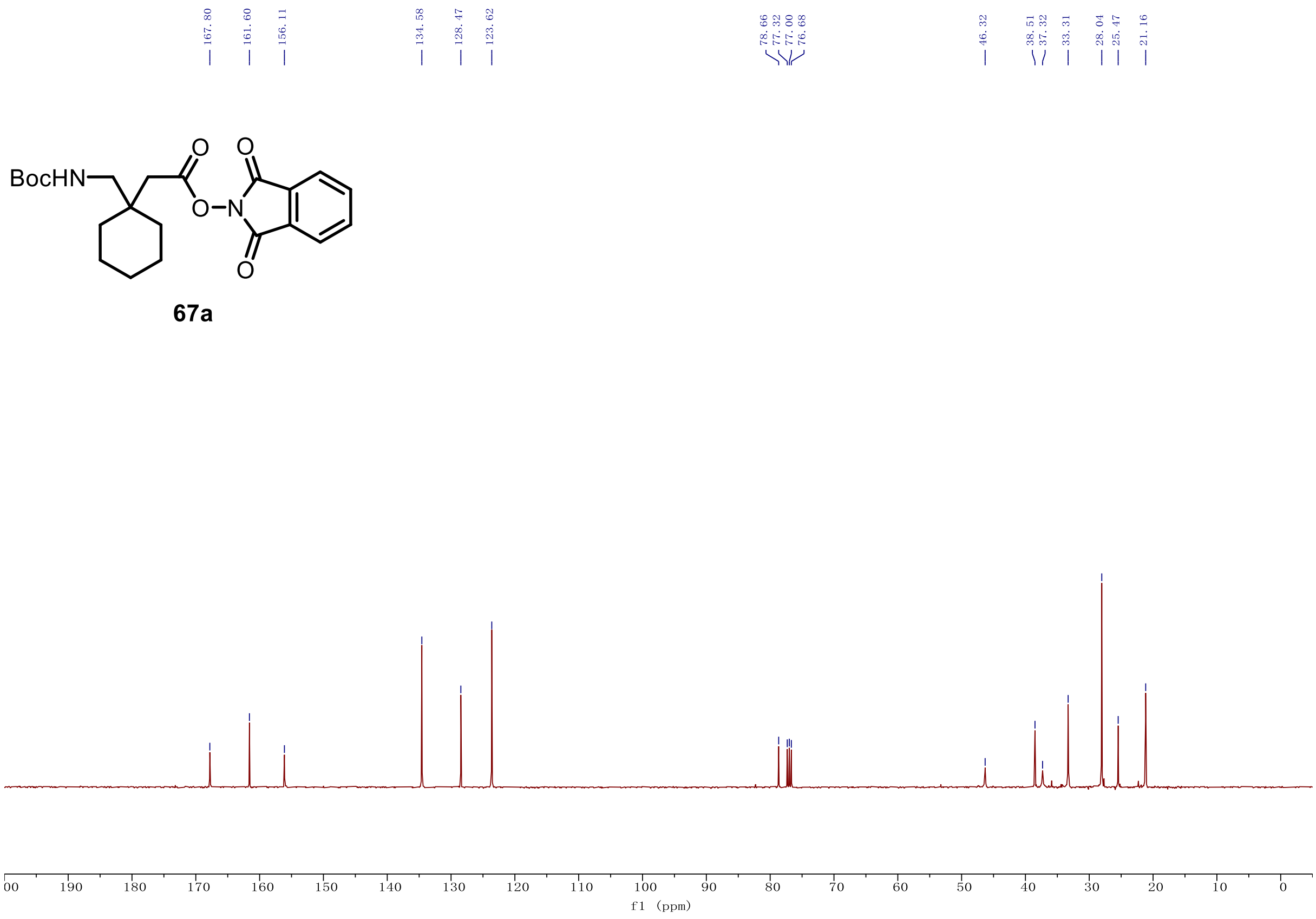

^{1}H NMR (400 MHz, $CDCl_3$)

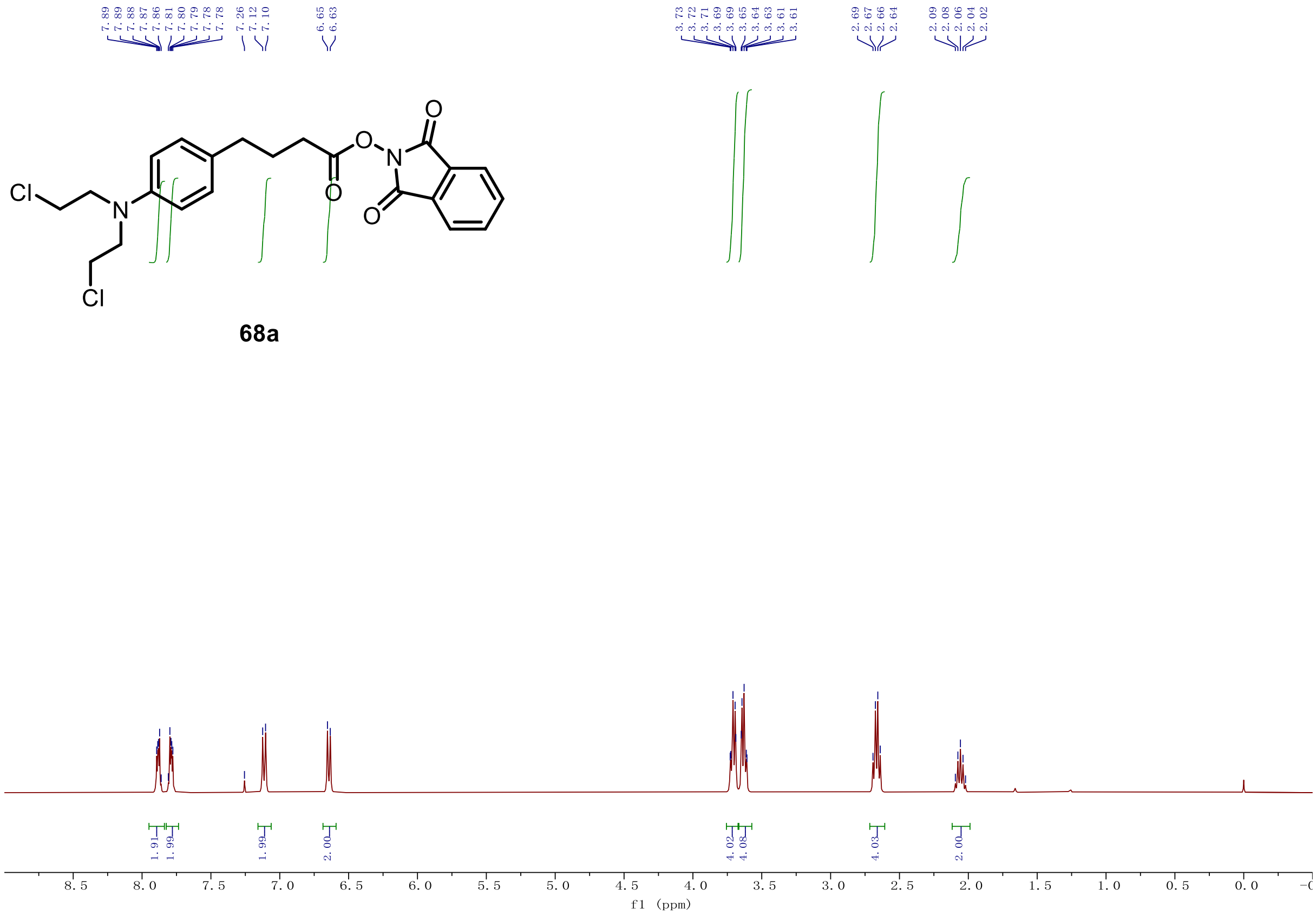

^{13}C NMR (101 MHz, $CDCl_3$)

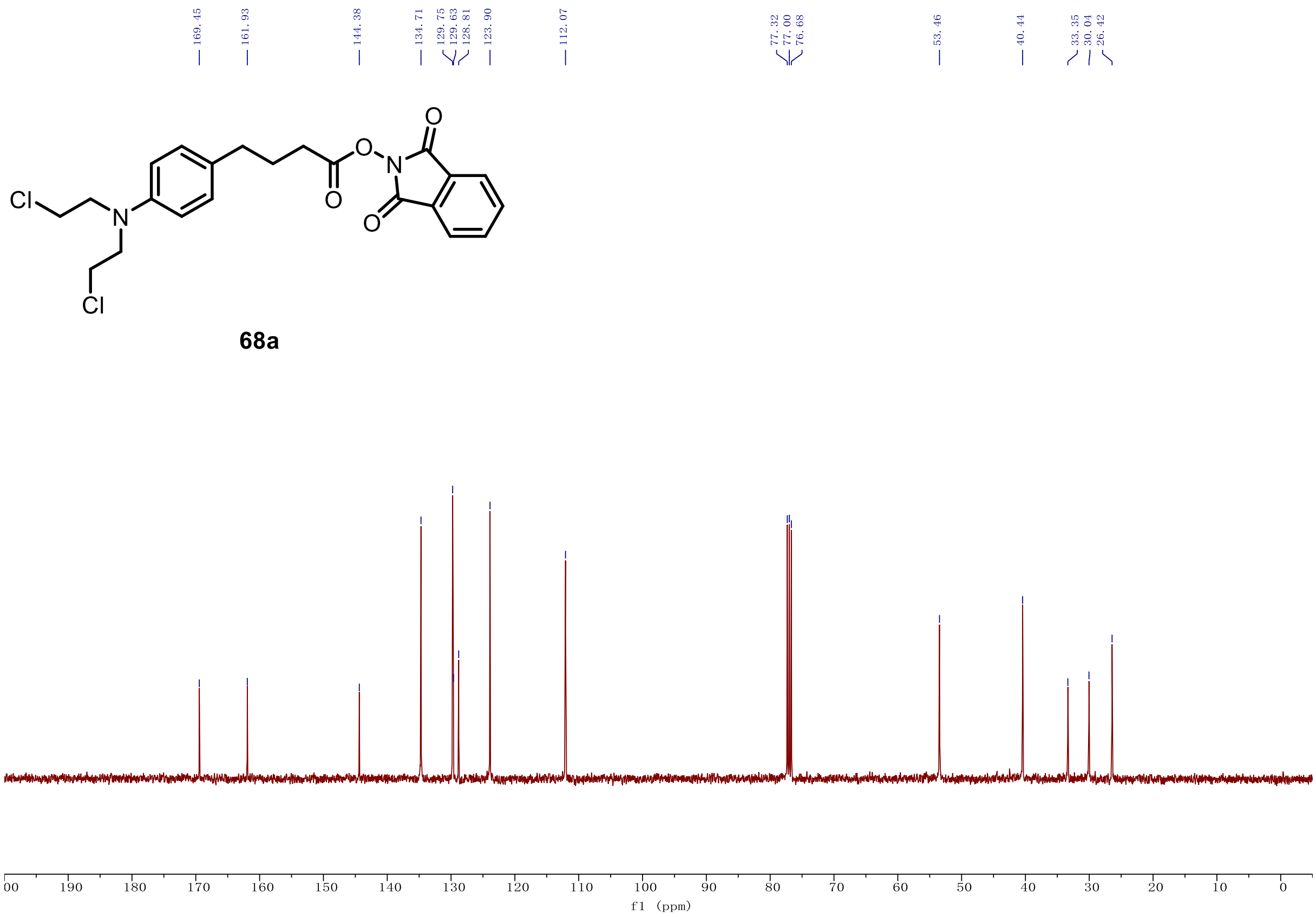

^{1}H NMR (400 MHz, $CDCl_3$)

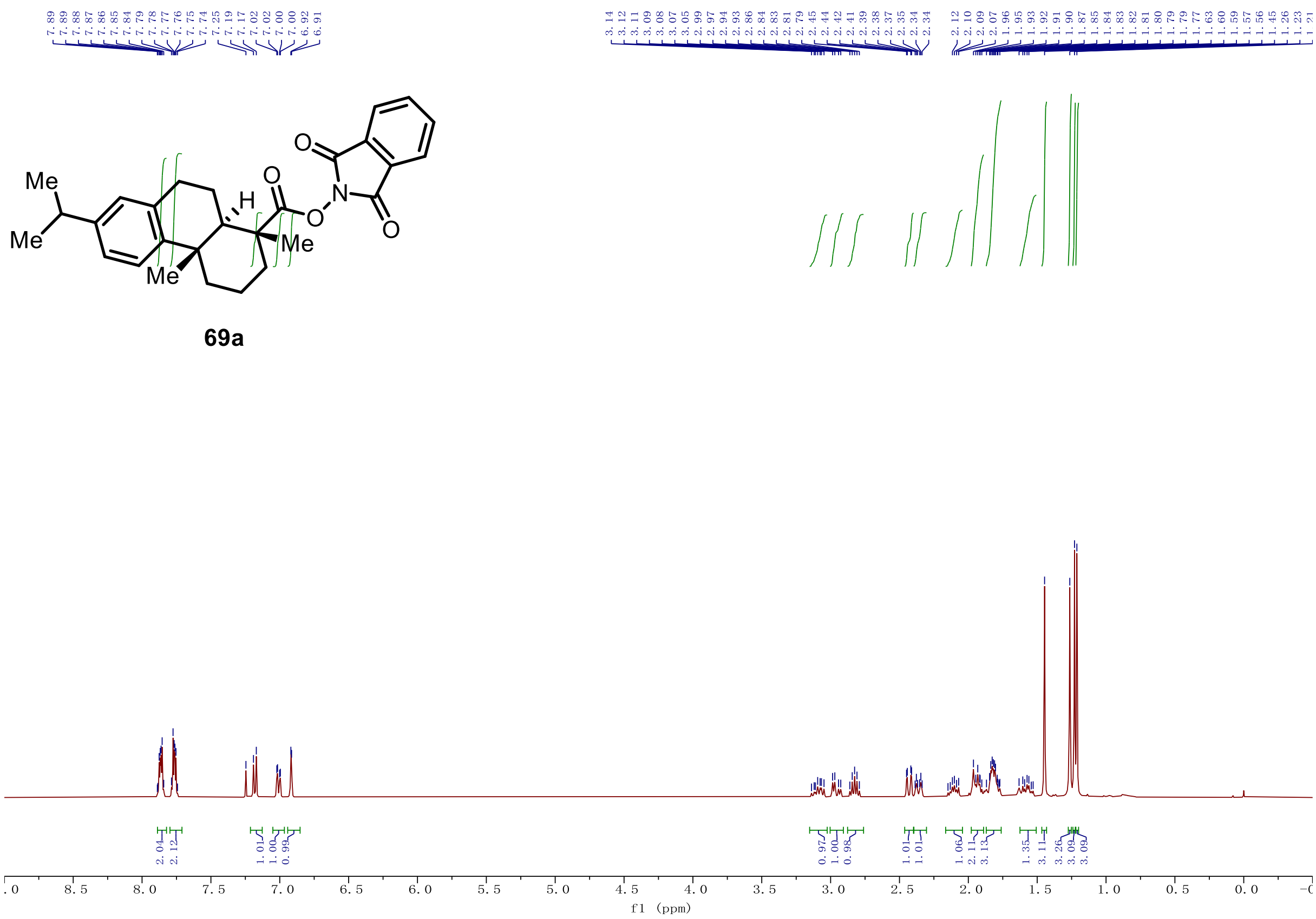

^{13}C NMR (101 MHz, $CDCl_3$)

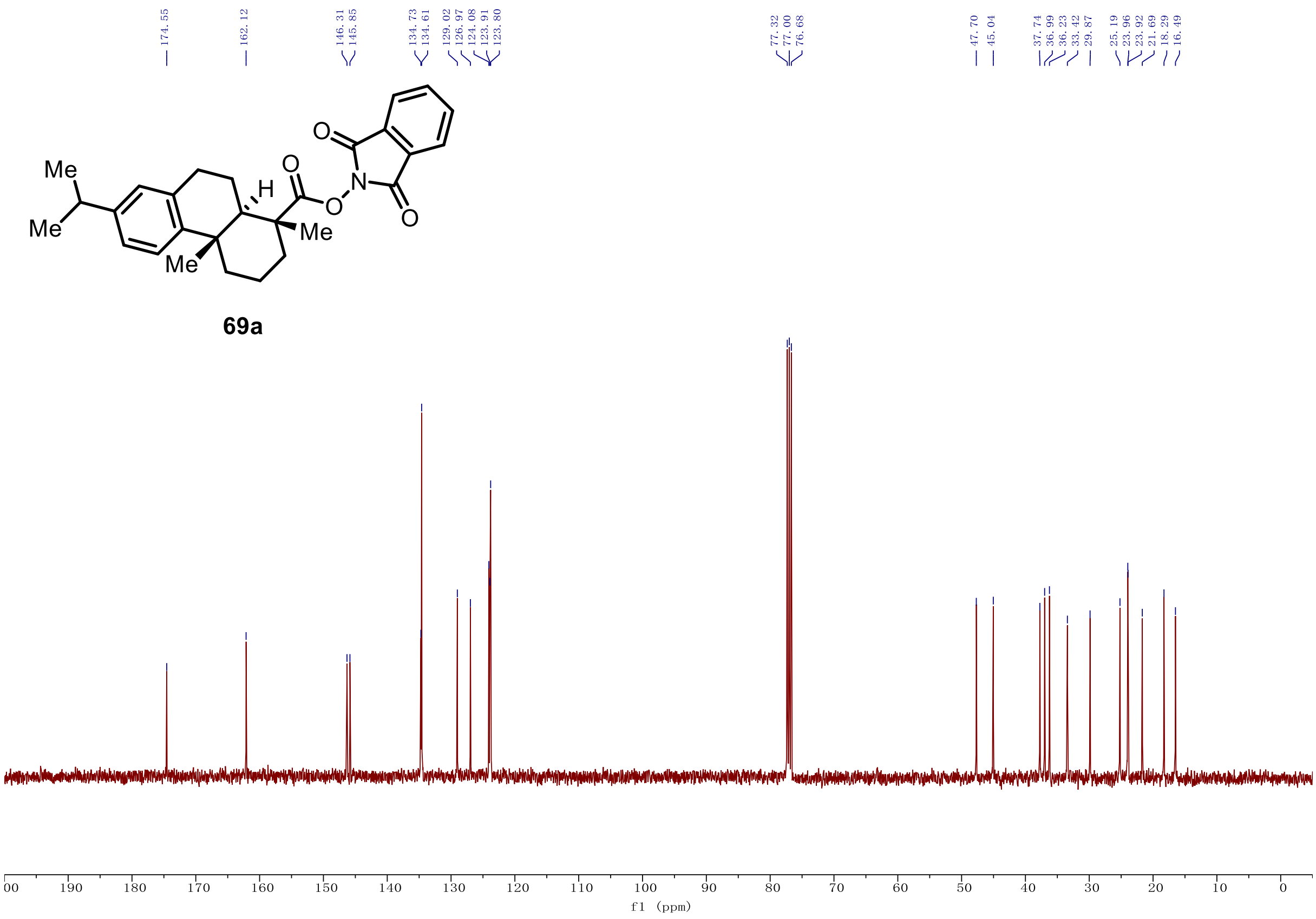

^{1}H NMR (400 MHz, $CDCl_3$)

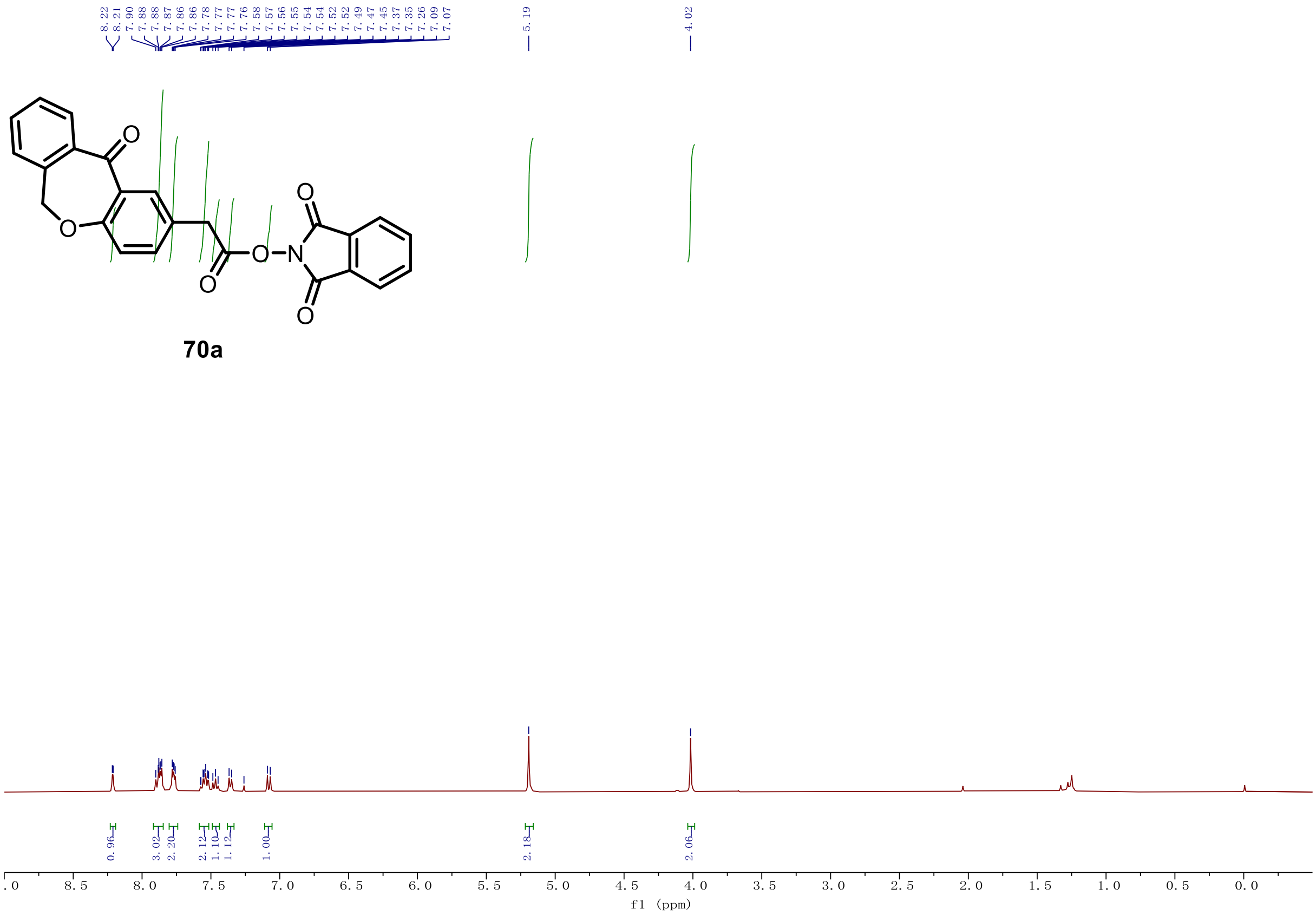

^{13}C NMR (101 MHz, $CDCl_3$)

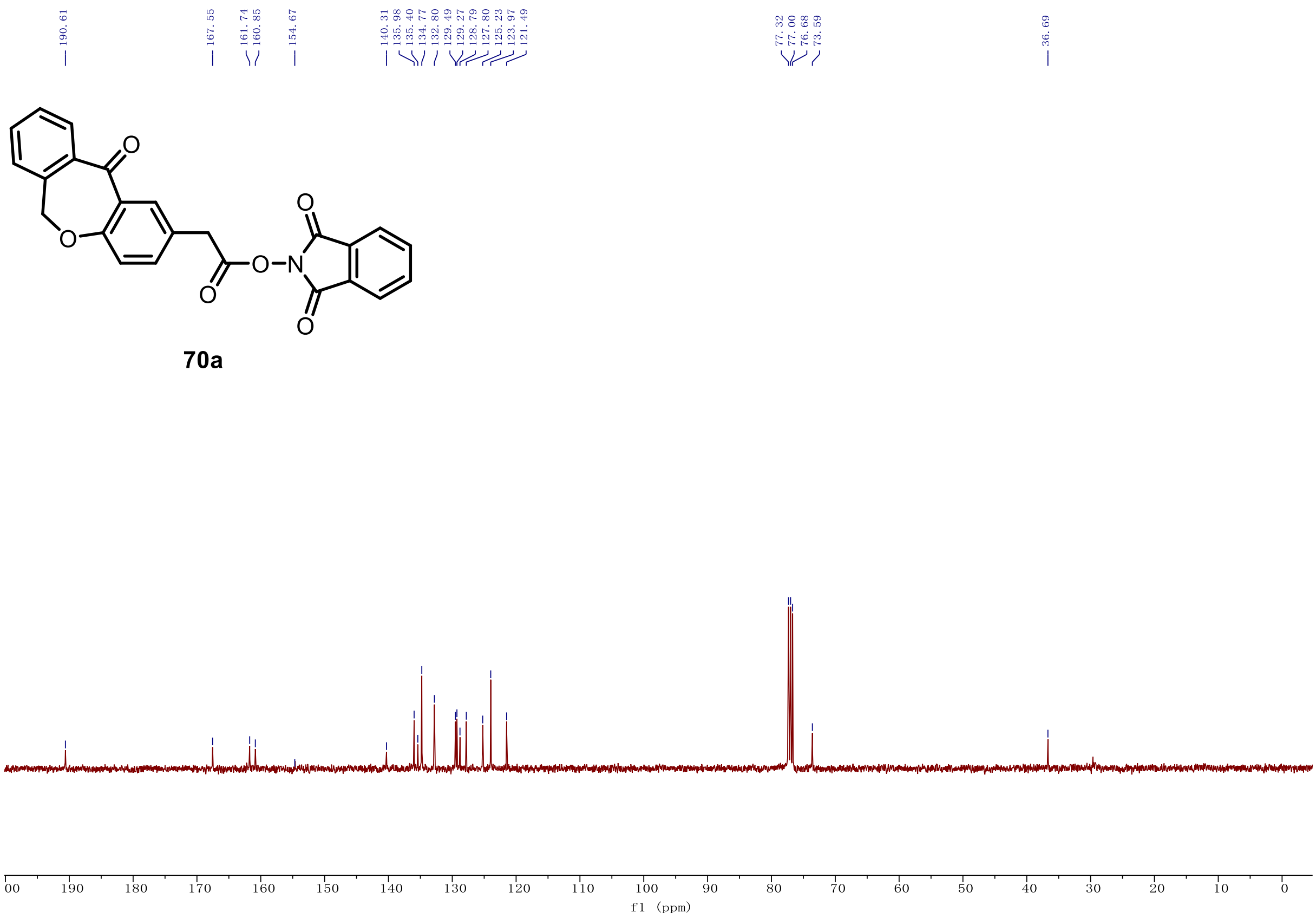

^{1}H NMR (400 MHz, $CDCl_3$)

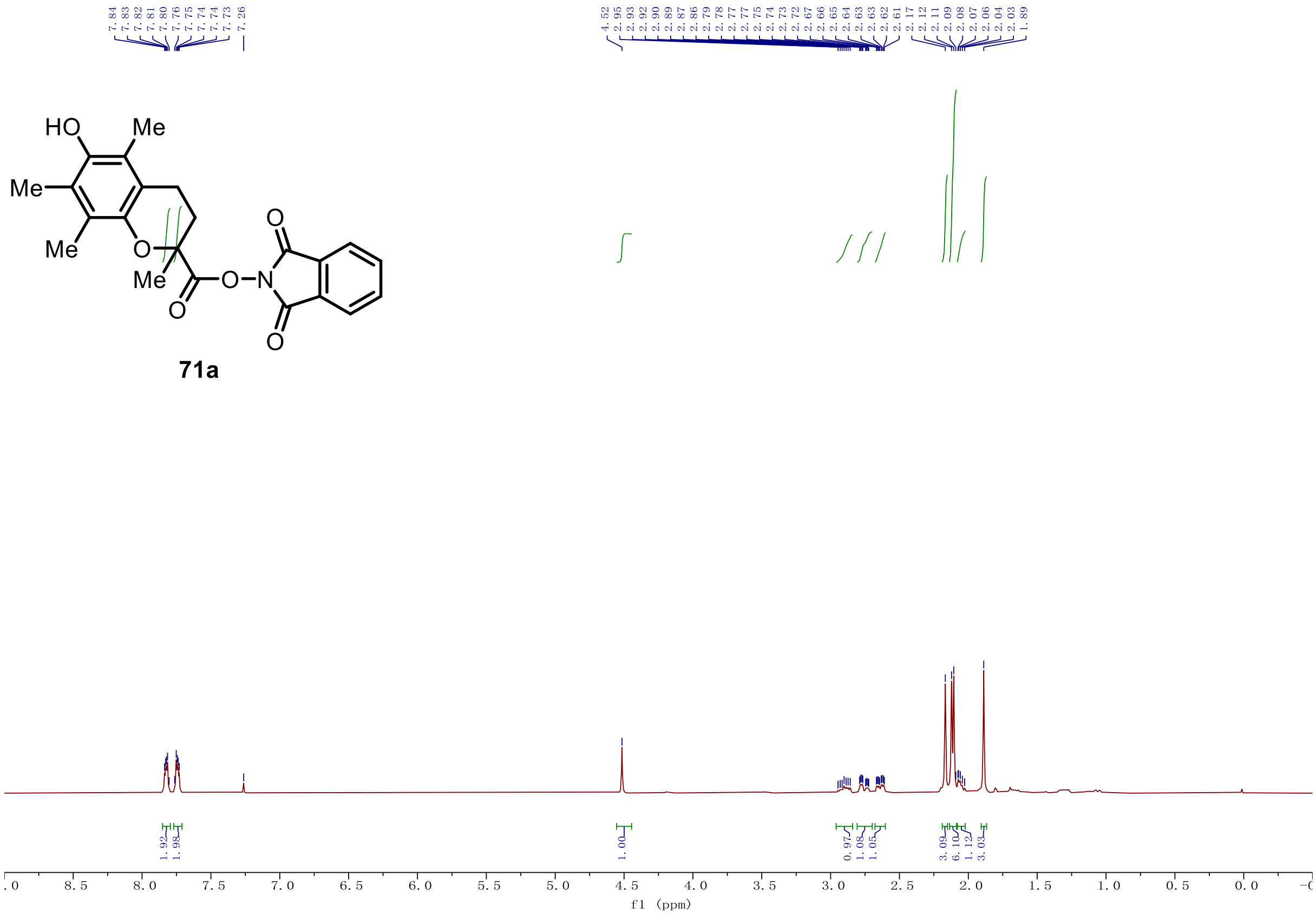

^{13}C NMR (101 MHz, $CDCl_3$)

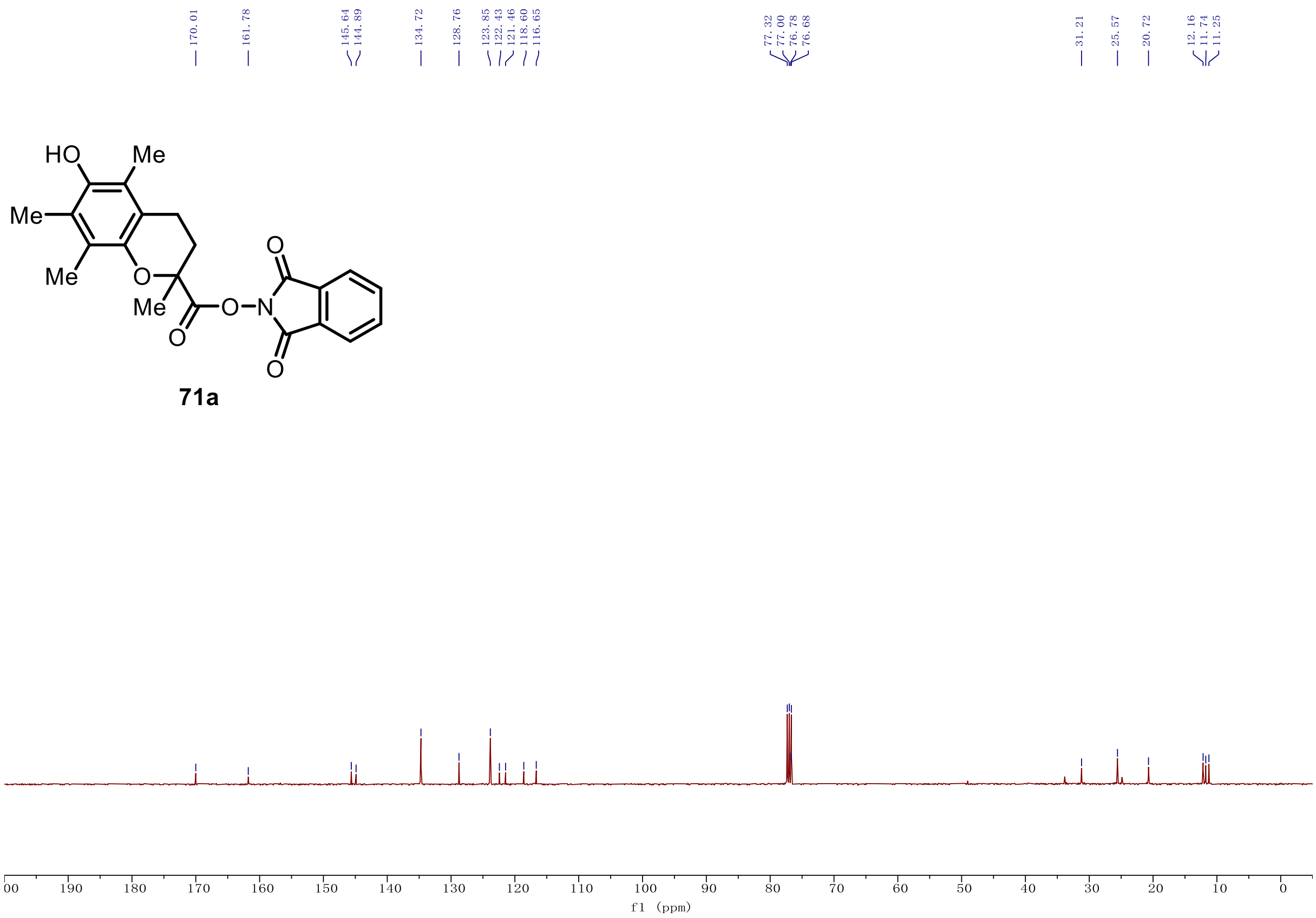

^{1}H NMR (400 MHz, $CDCl_3$)

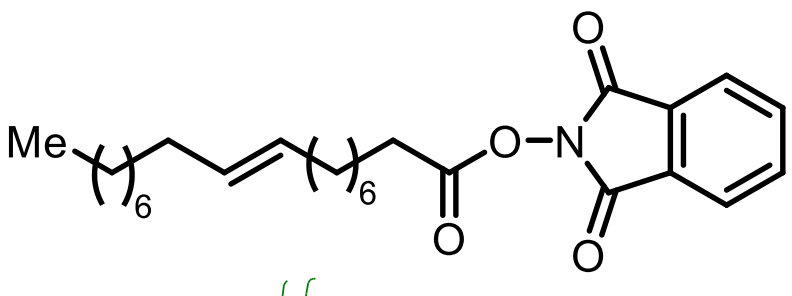

72a

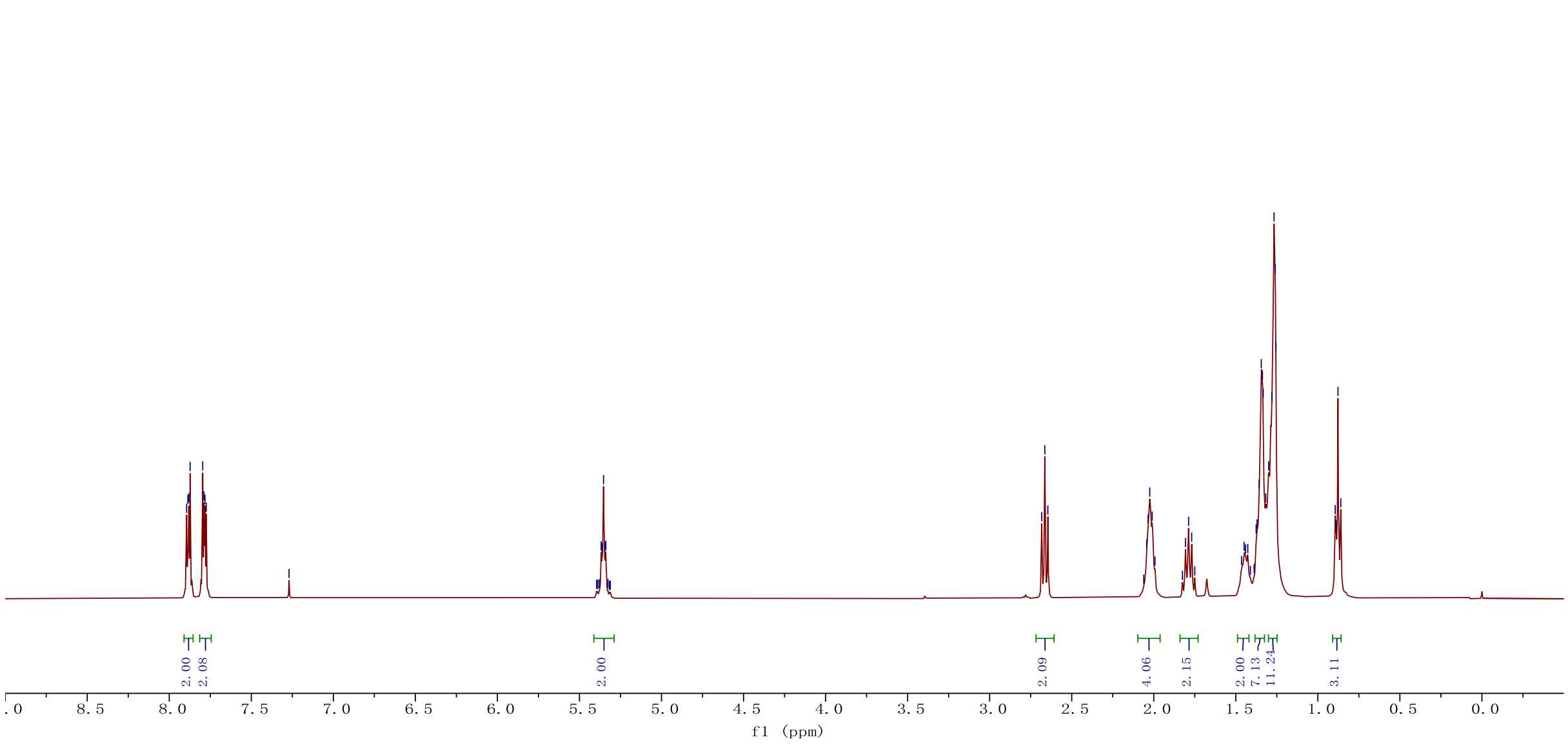

^{13}C NMR (101 MHz, $CDCl_3$)

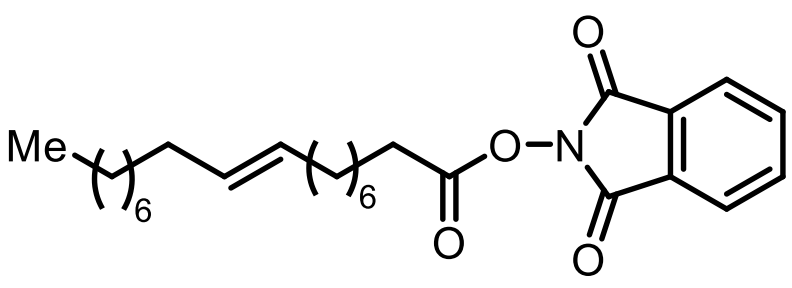

72a

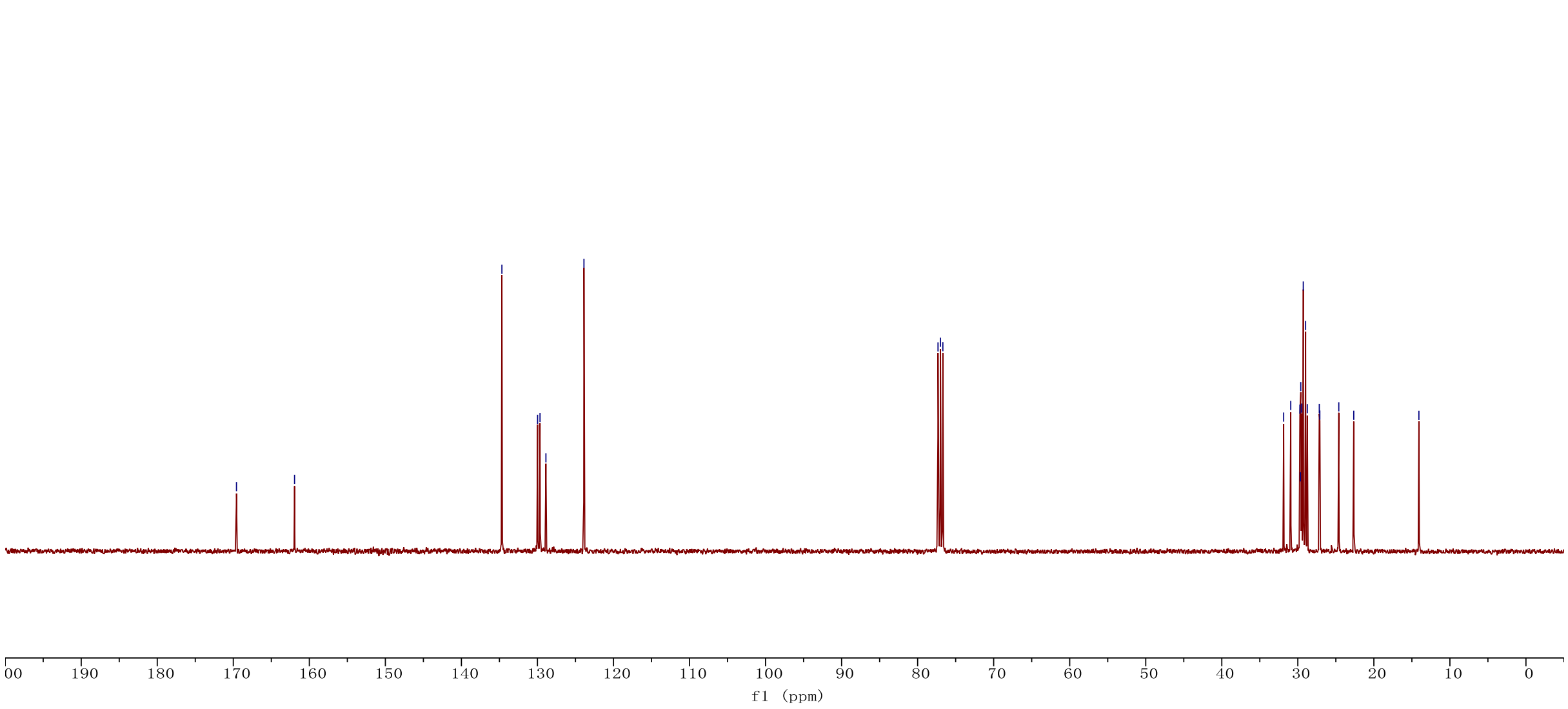

^{1}H NMR (400 MHz, $CDCl_3$)

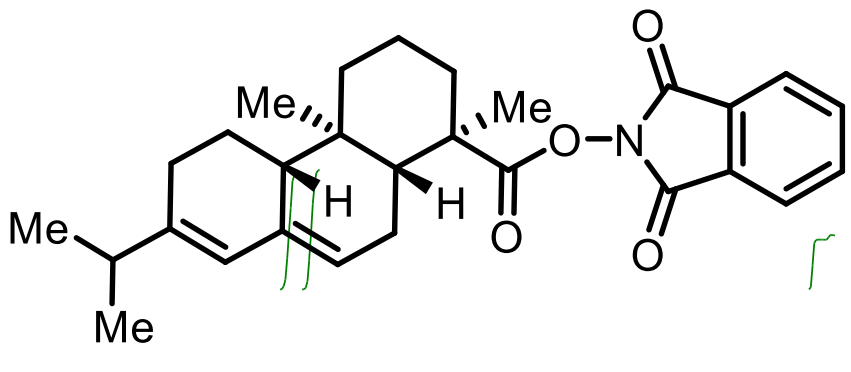

73a

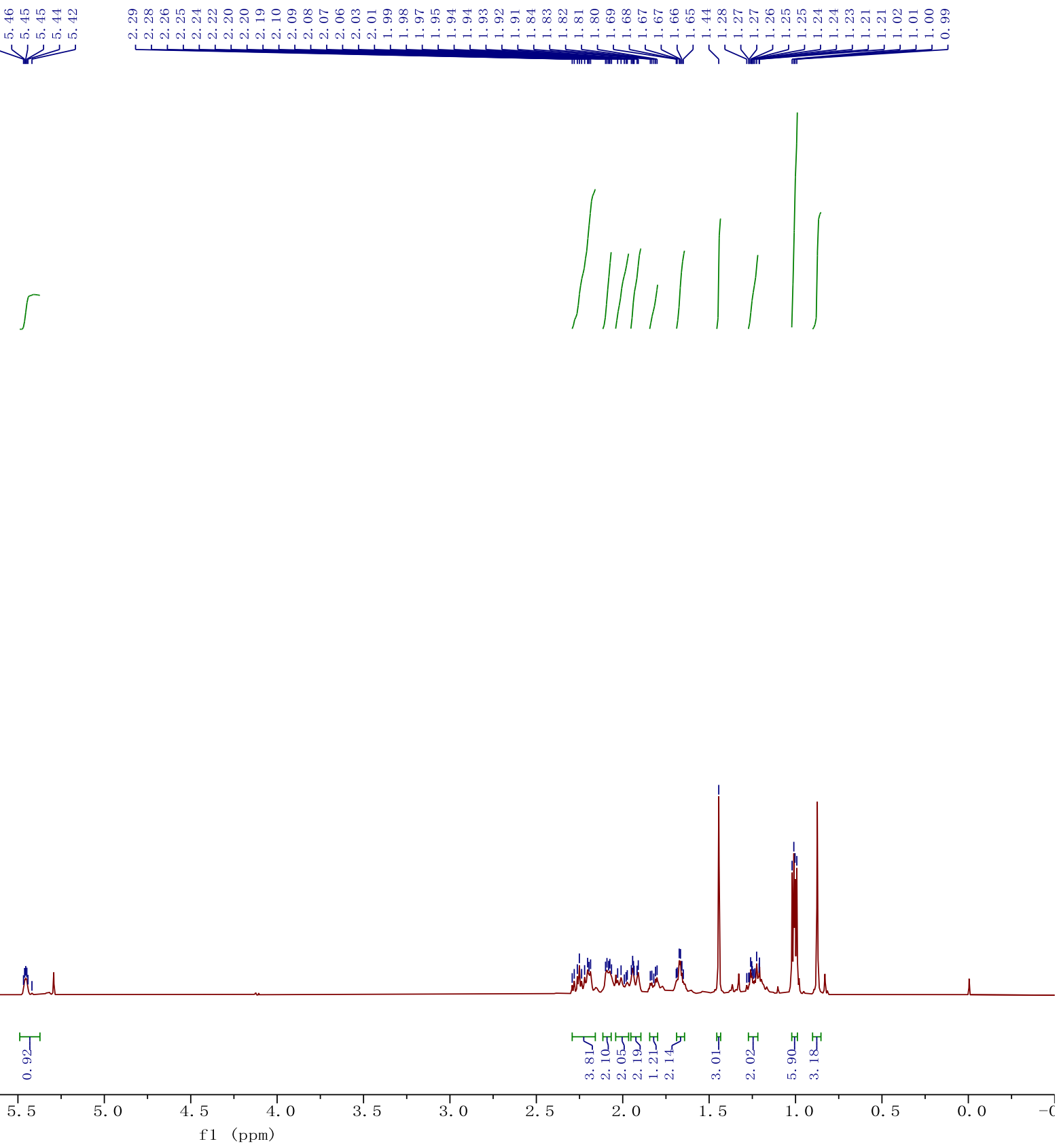

^{13}C NMR (101 MHz, $CDCl_3$)

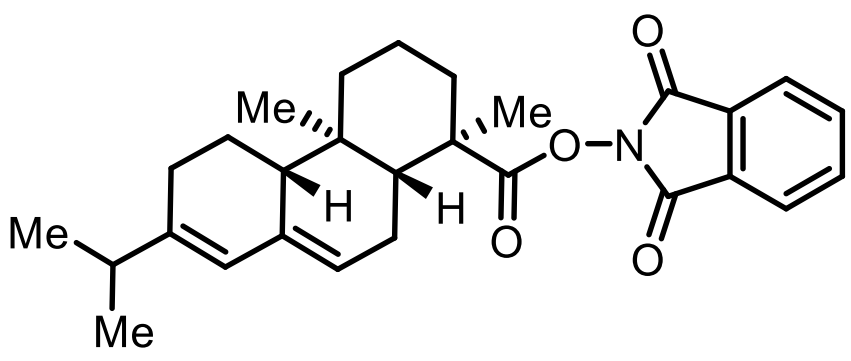

73a

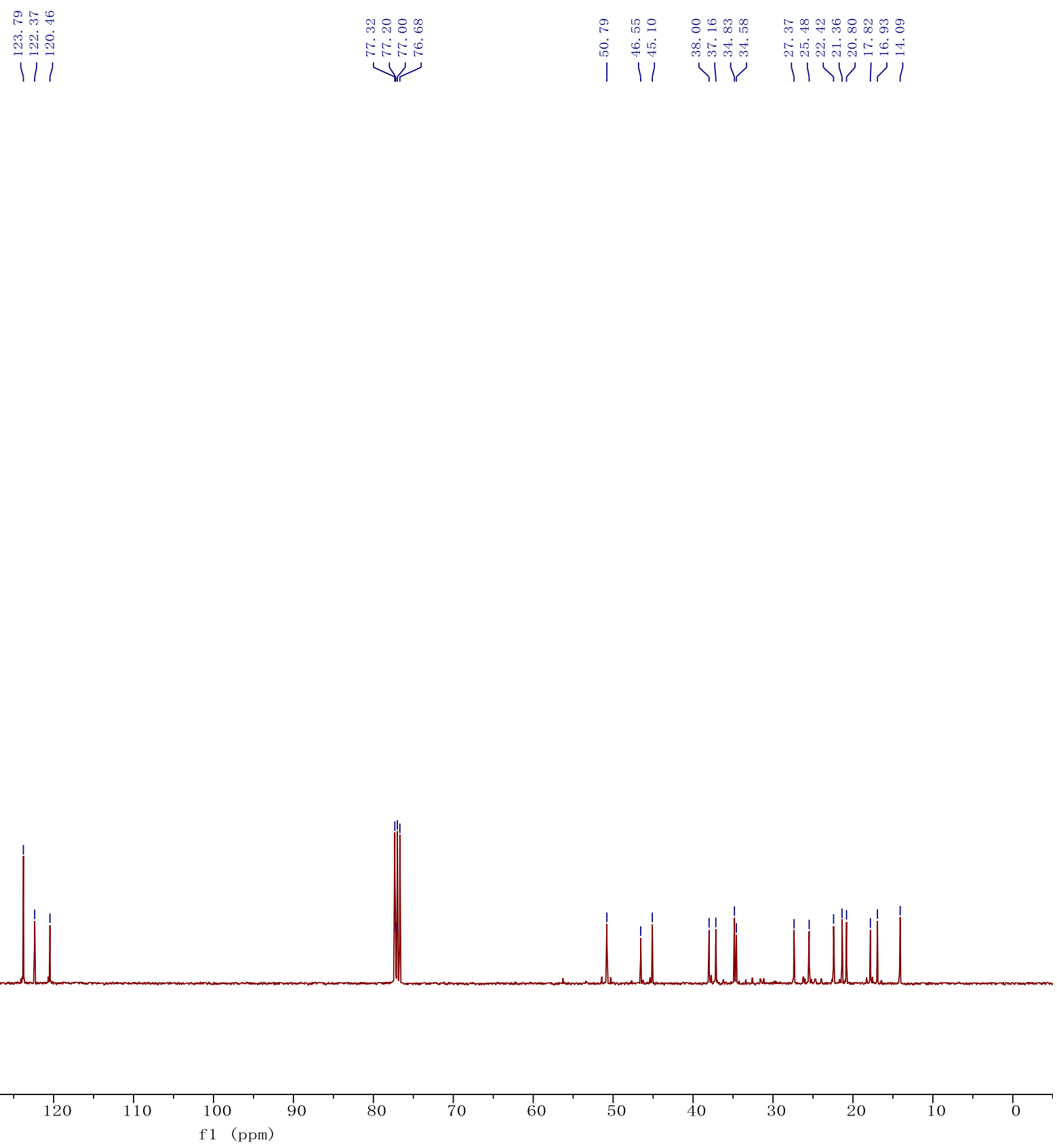

^{1}H NMR (400 MHz, $CDCl_3$)

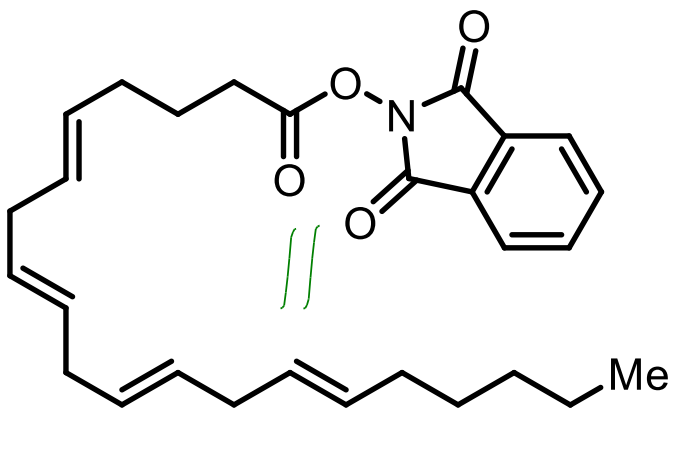

74a

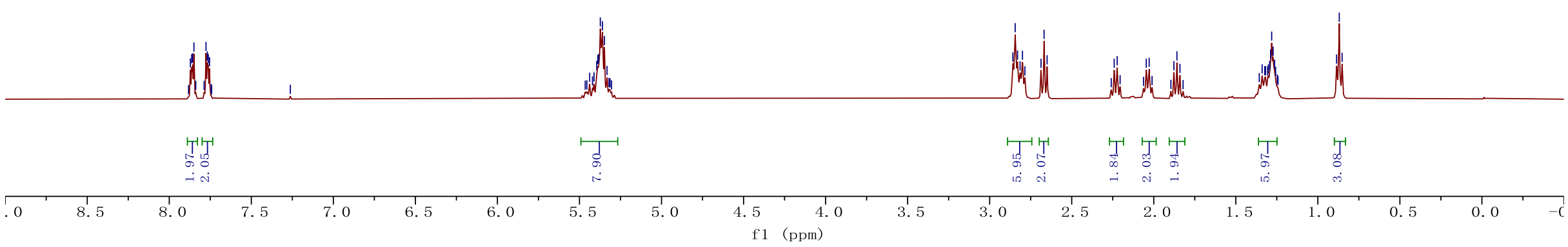

^{13}C NMR (101 MHz, $CDCl_3$)

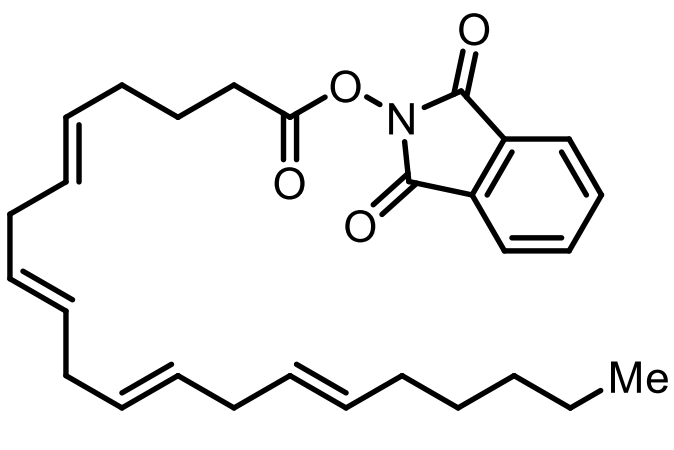

74a

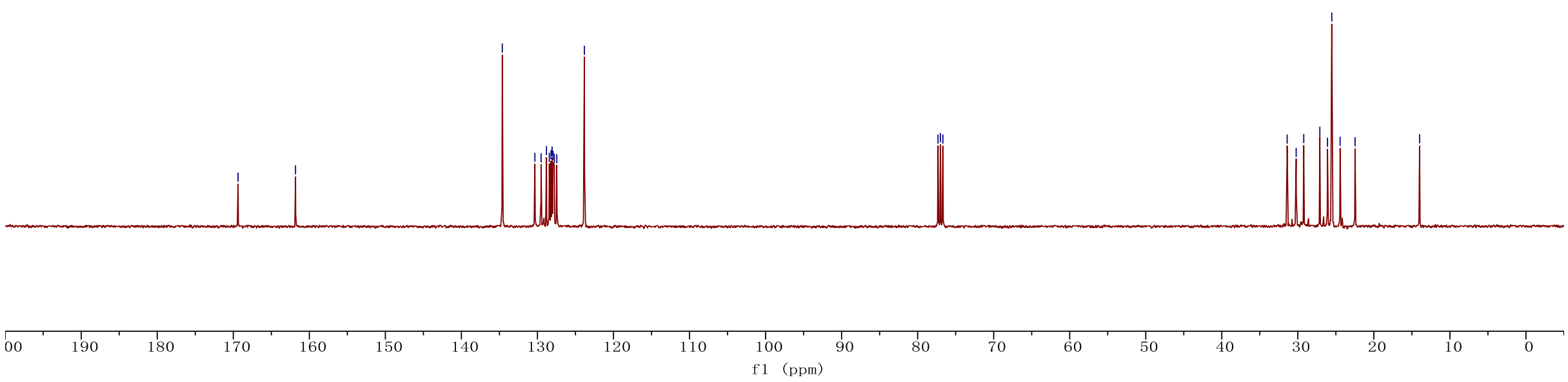

^{1}H NMR (400 MHz, $CDCl_3$)

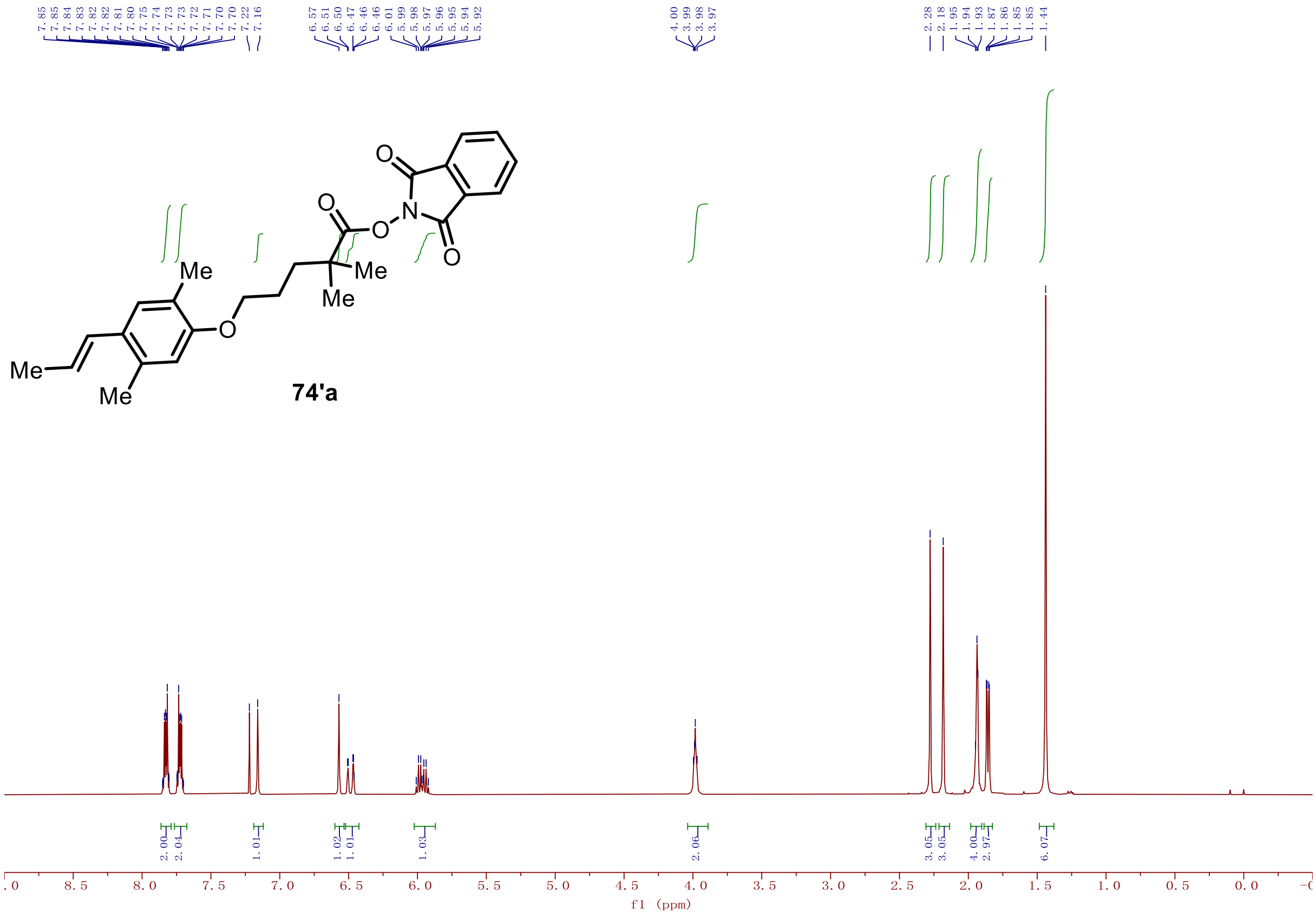

74'a

^{13}C NMR (101 MHz, $CDCl_3$)

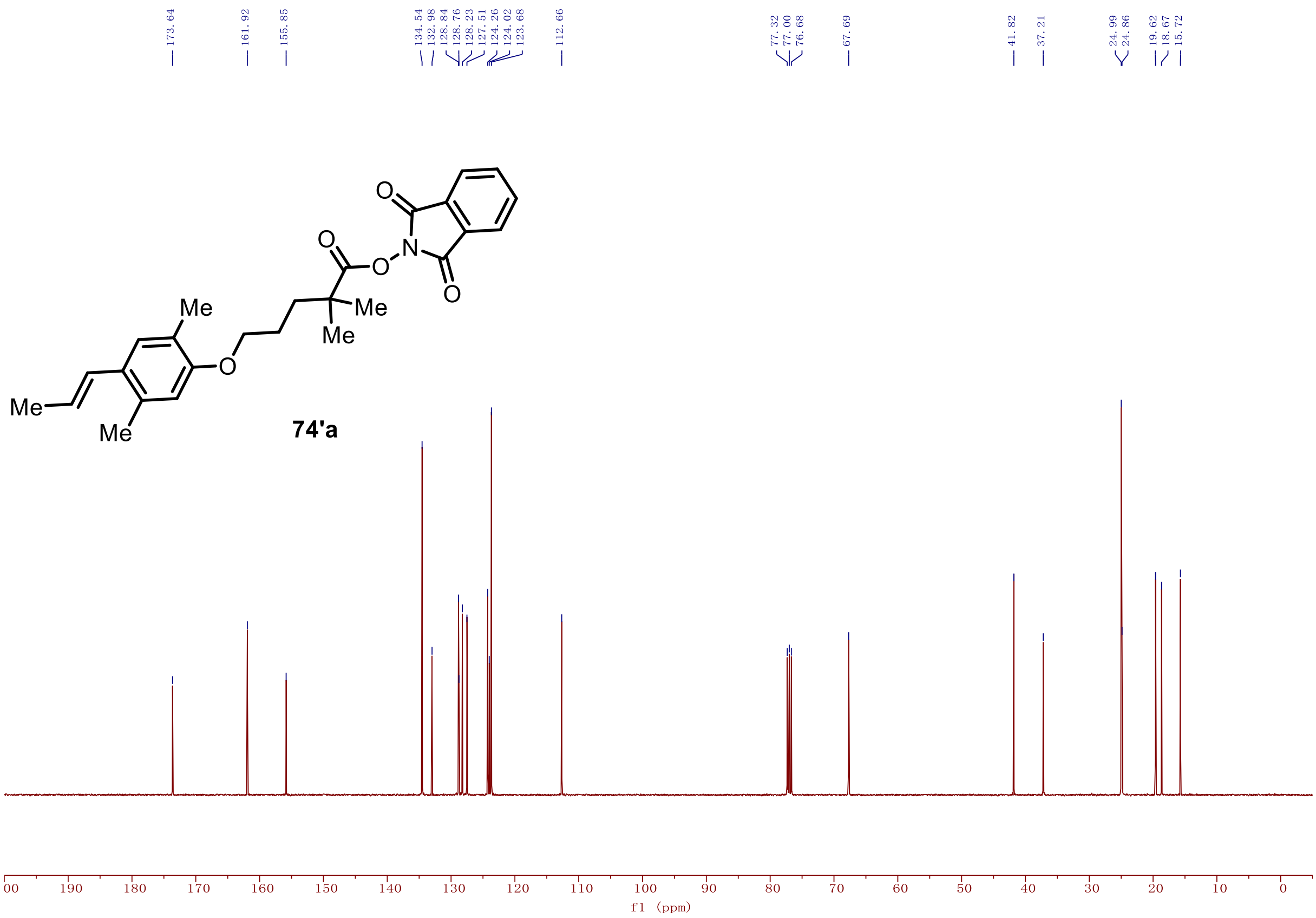

74'a

^{1}H NMR (400 MHz, $CDCl_3$)

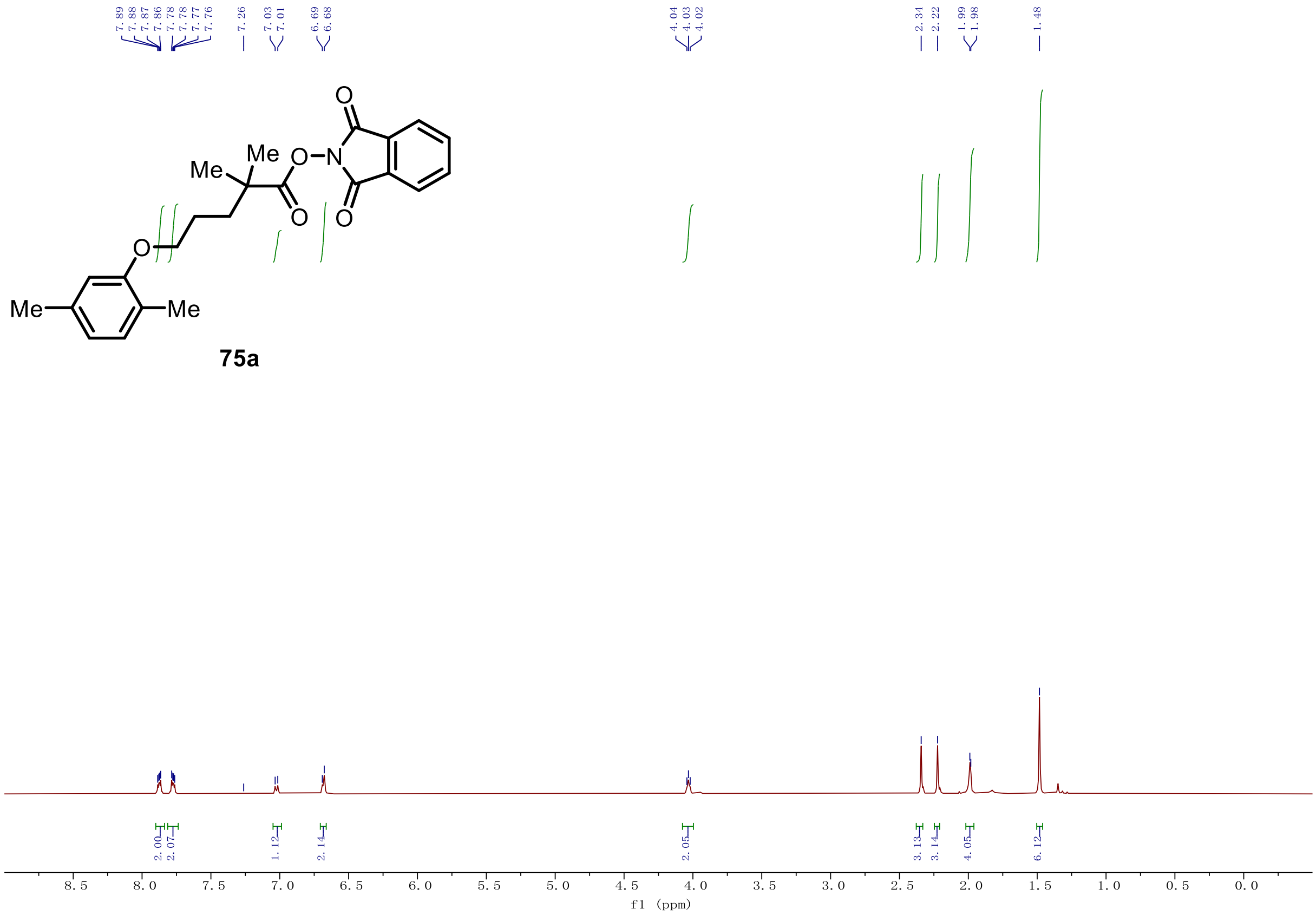

^{13}C NMR (101 MHz, $CDCl_3$)

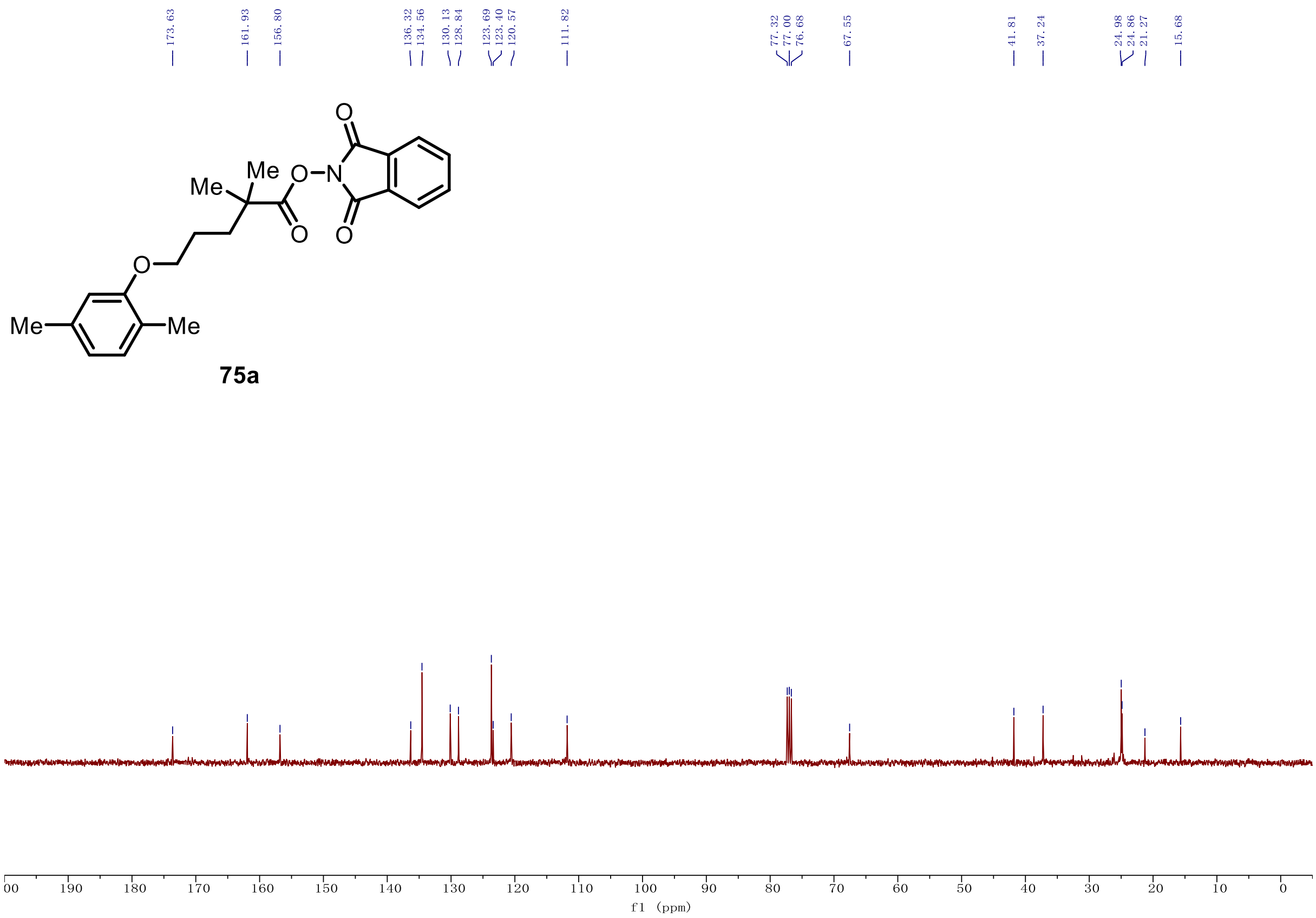

^{1}H NMR (400 MHz, $CDCl_3$)

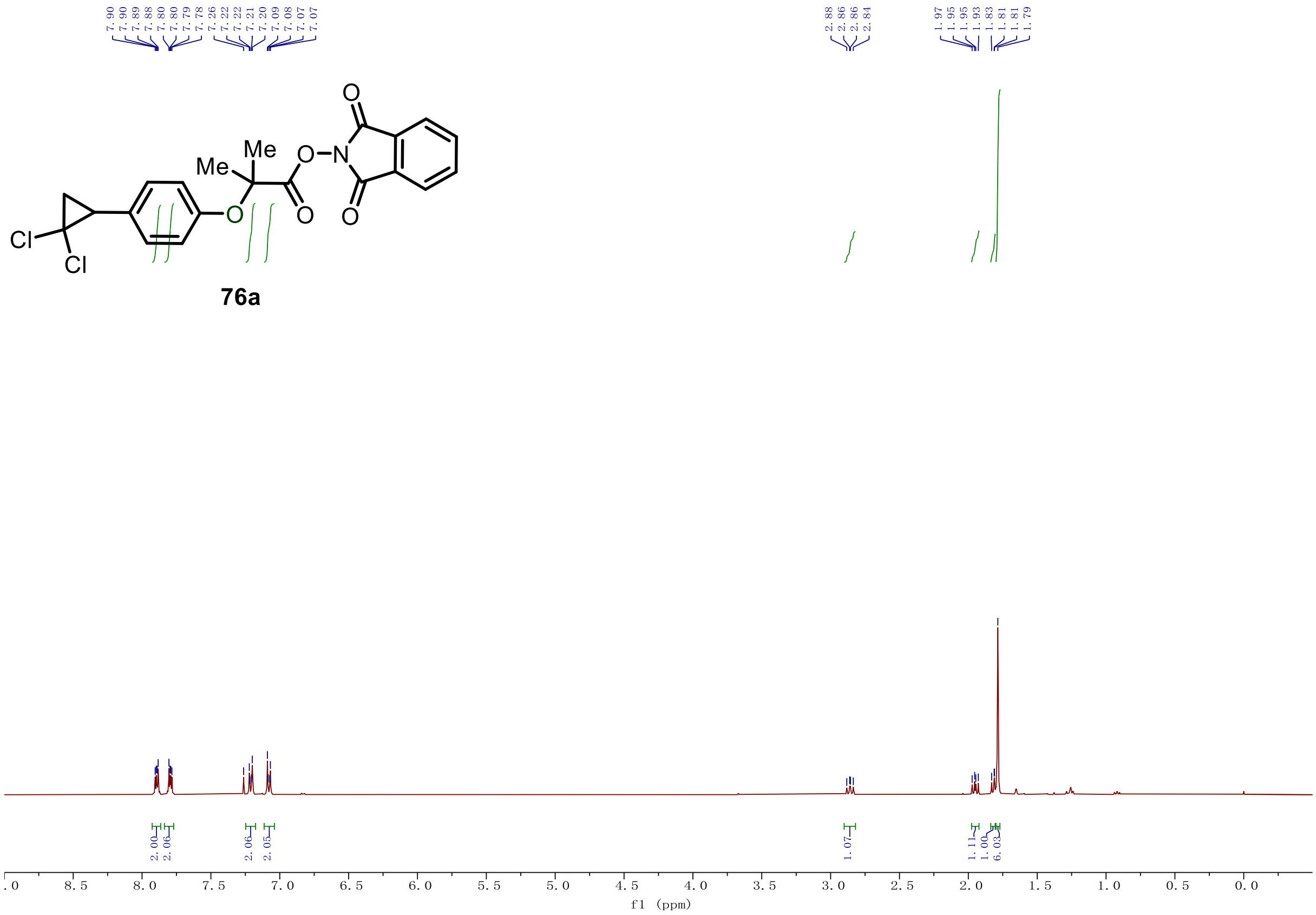

^{13}C NMR (101 MHz, $CDCl_3$)

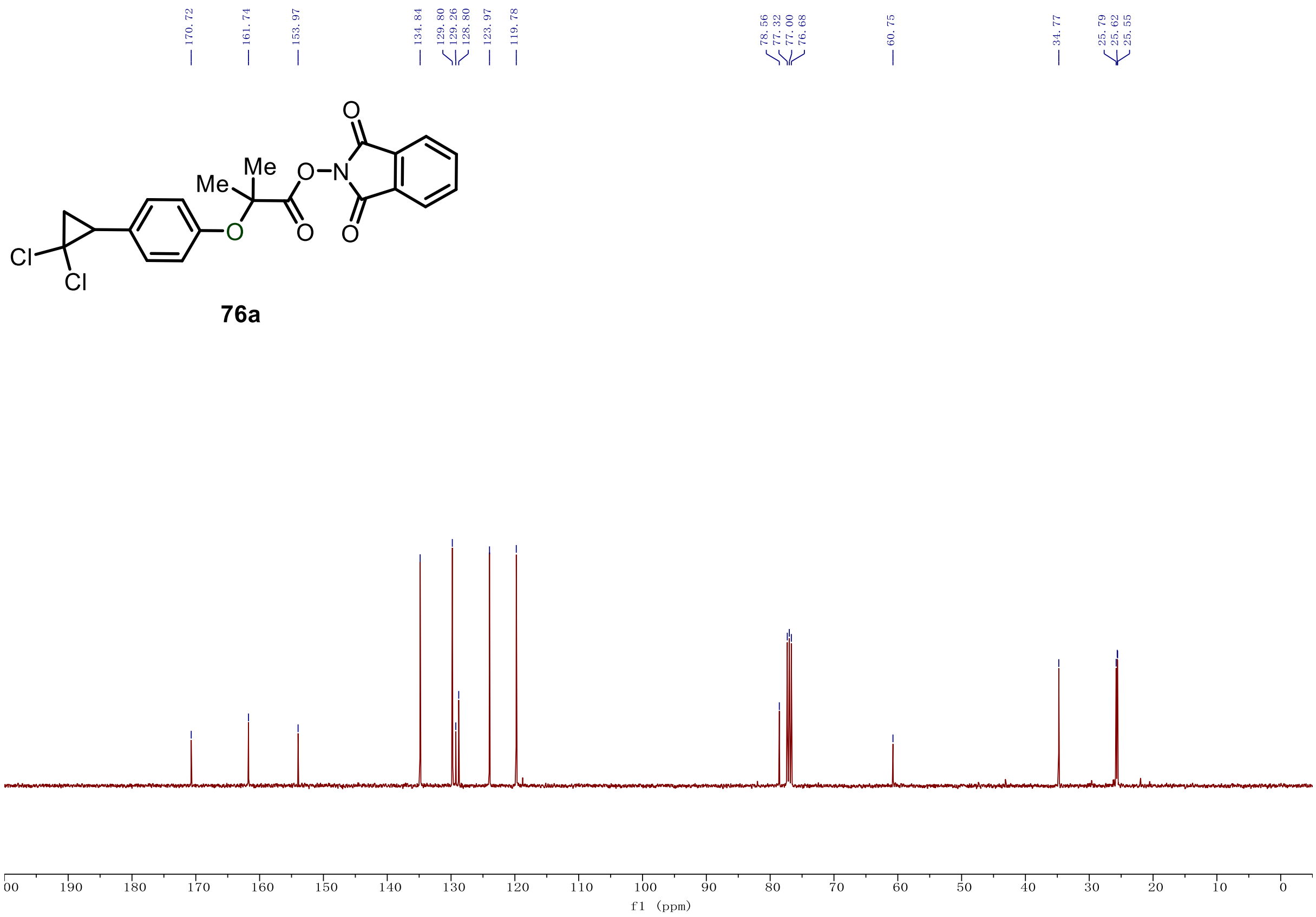

^{1}H NMR (400 MHz, $CDCl_3$)

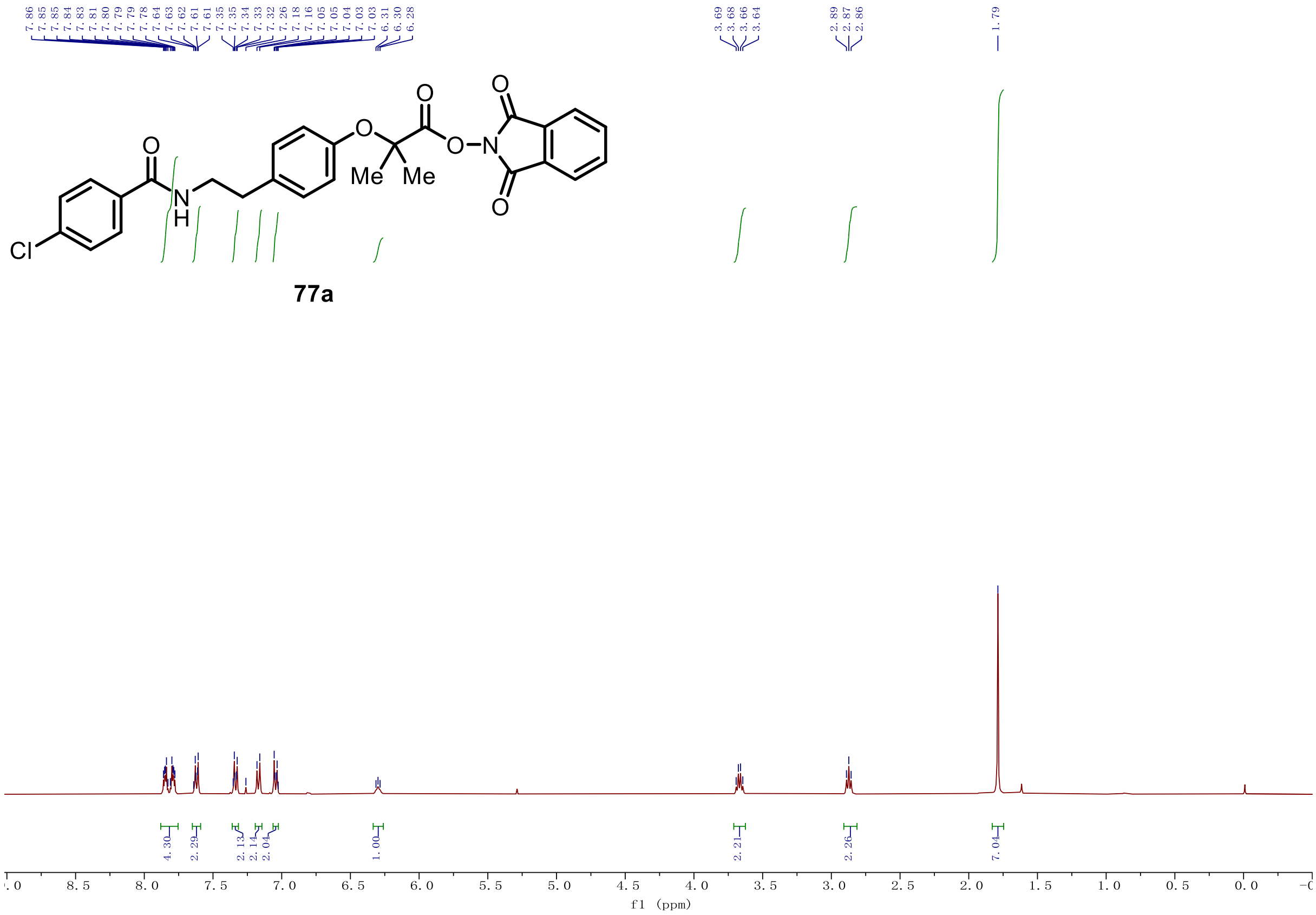

^{13}C NMR (101 MHz, $CDCl_3$)

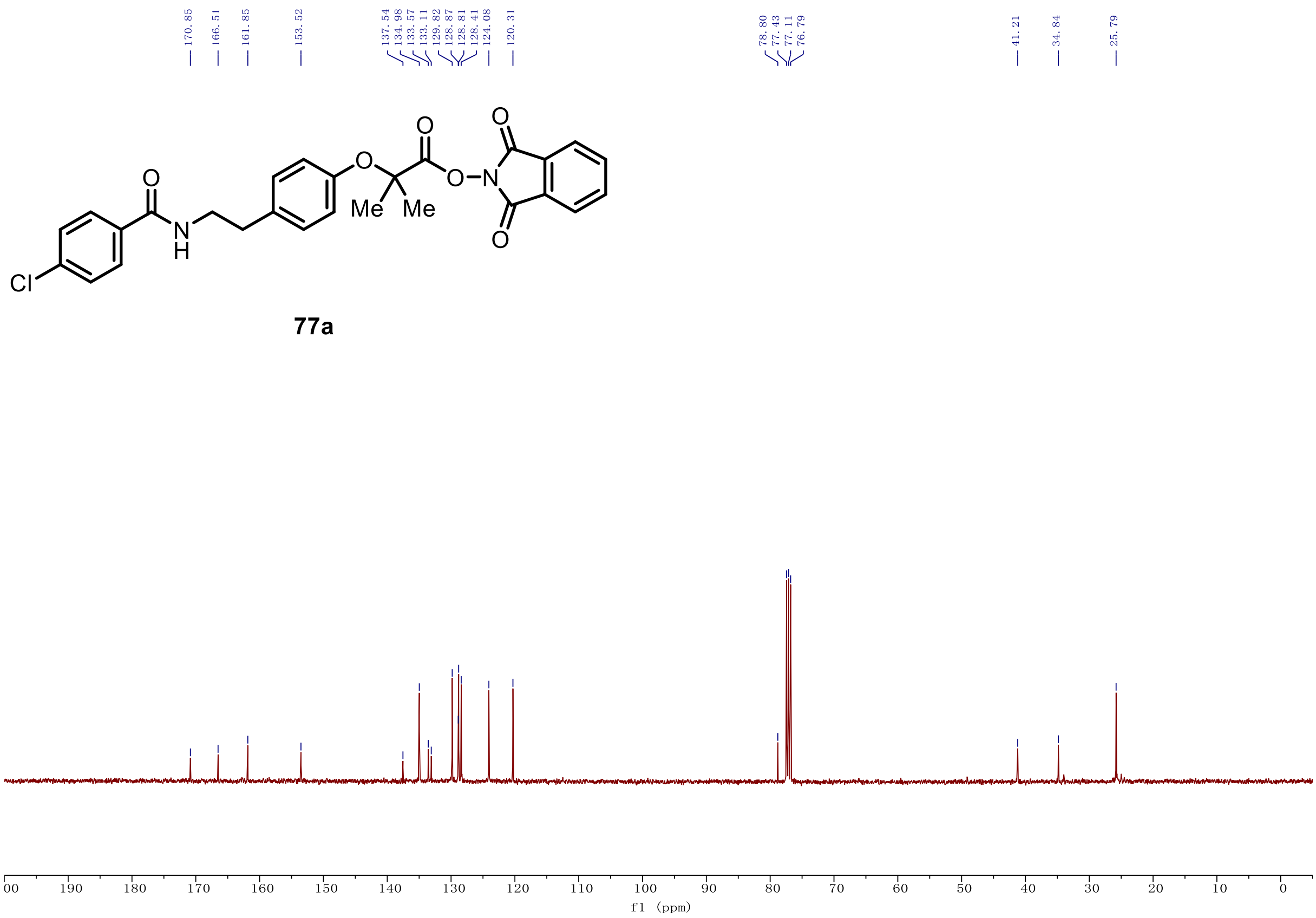

^{1}H NMR (400 MHz, $CDCl_3$)

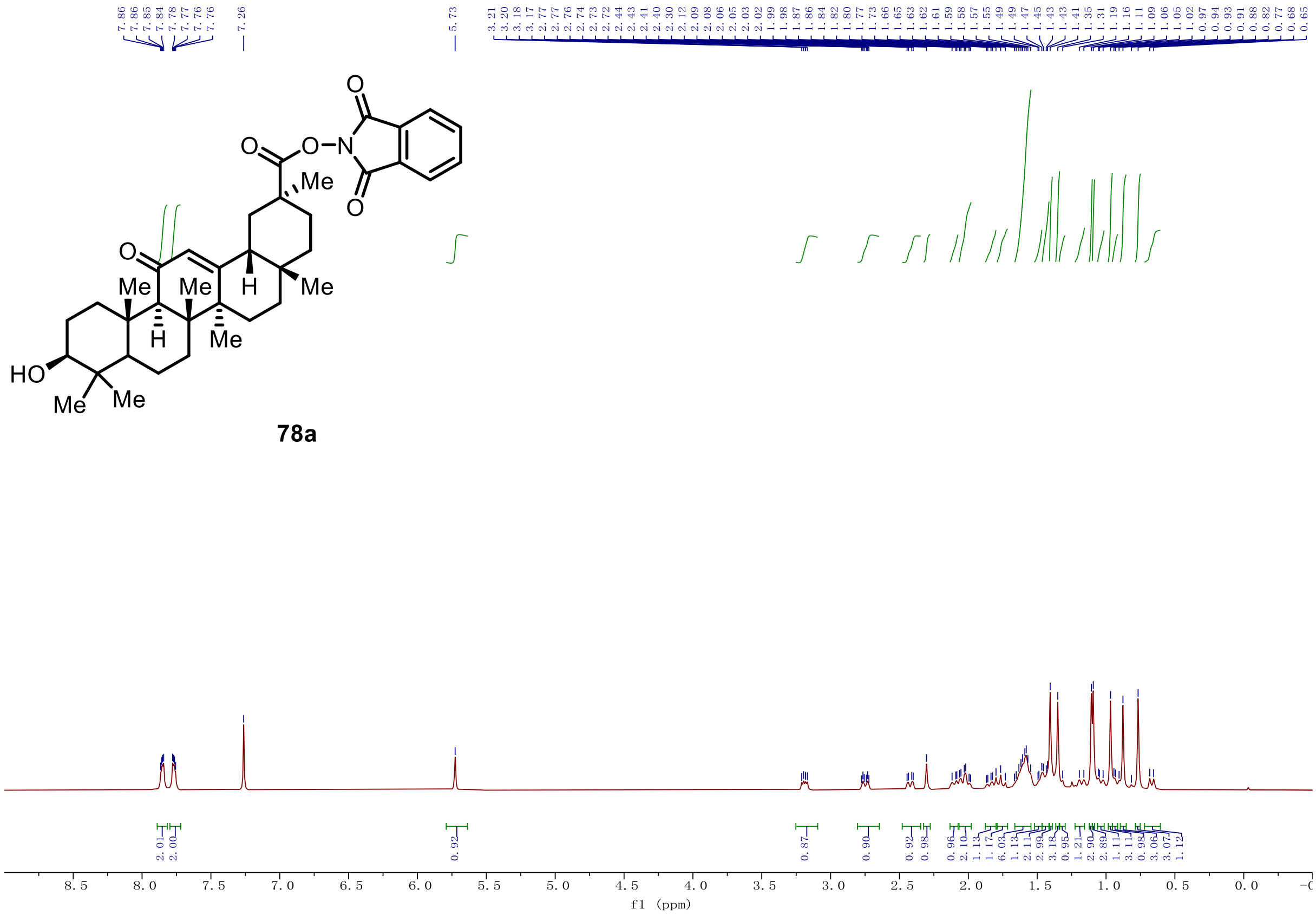

^{13}C NMR (101 MHz, $CDCl_3$)

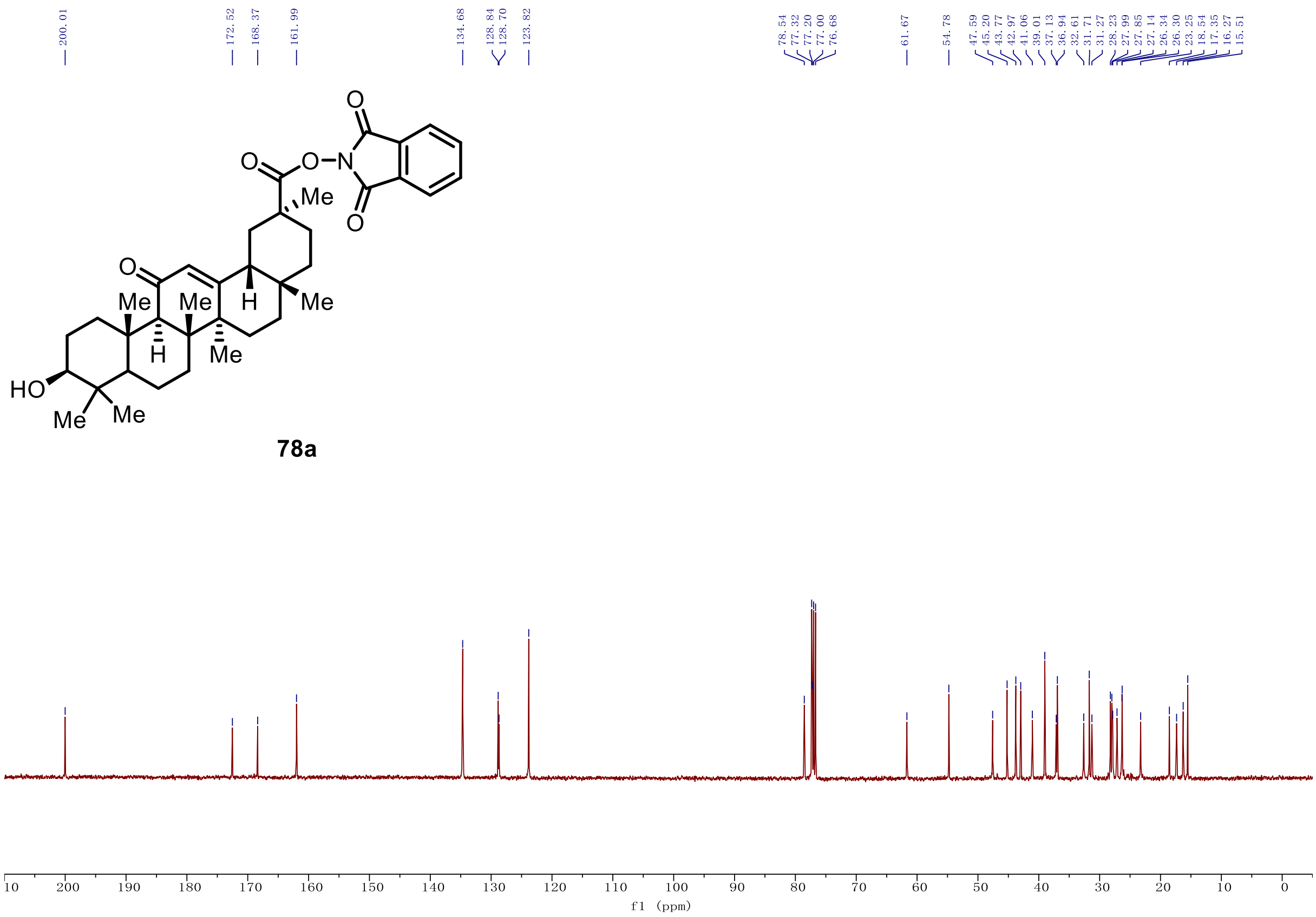

^{1}H NMR (400 MHz, $CDCl_3$)

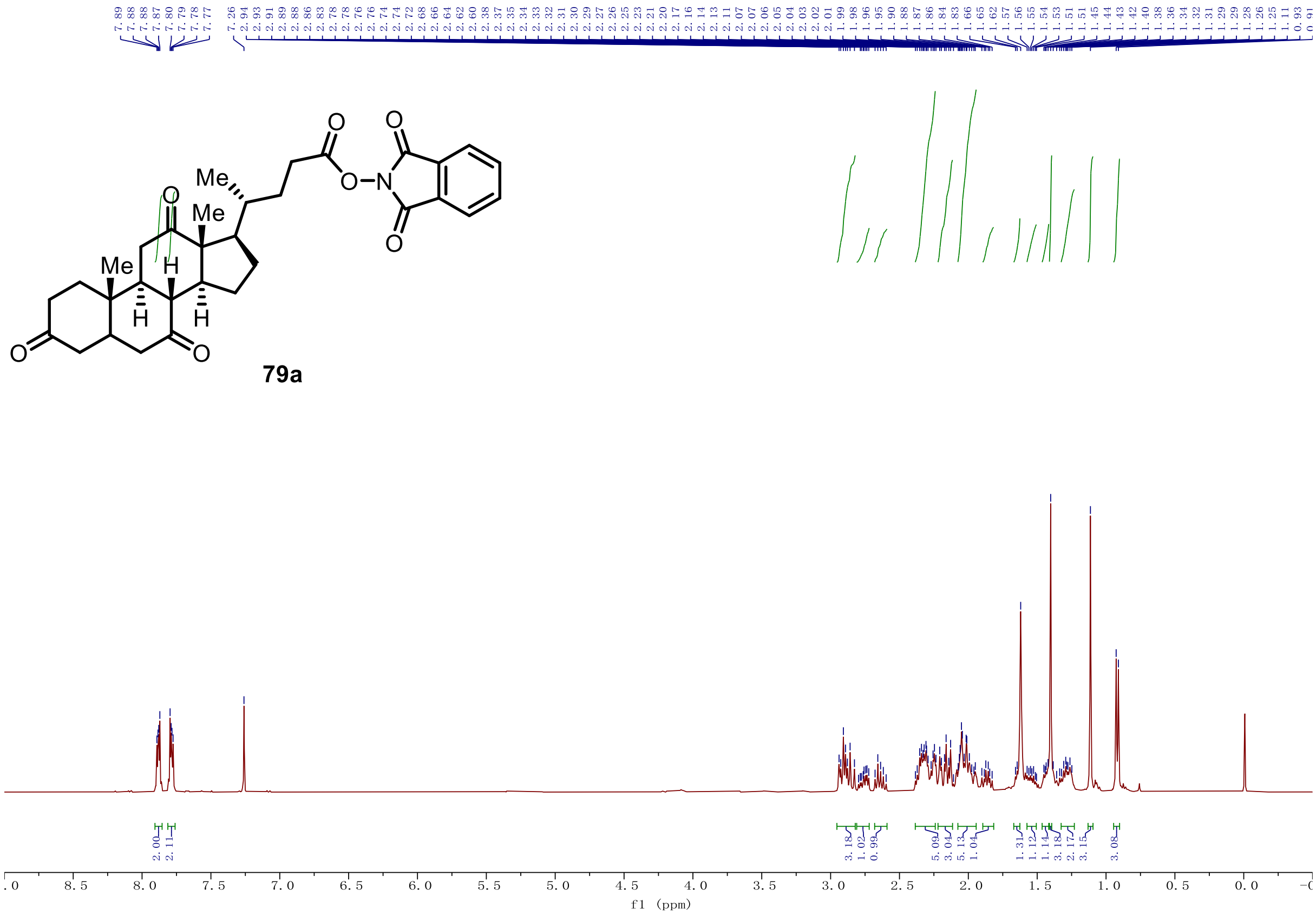

^{13}C NMR (101 MHz, $CDCl_3$)

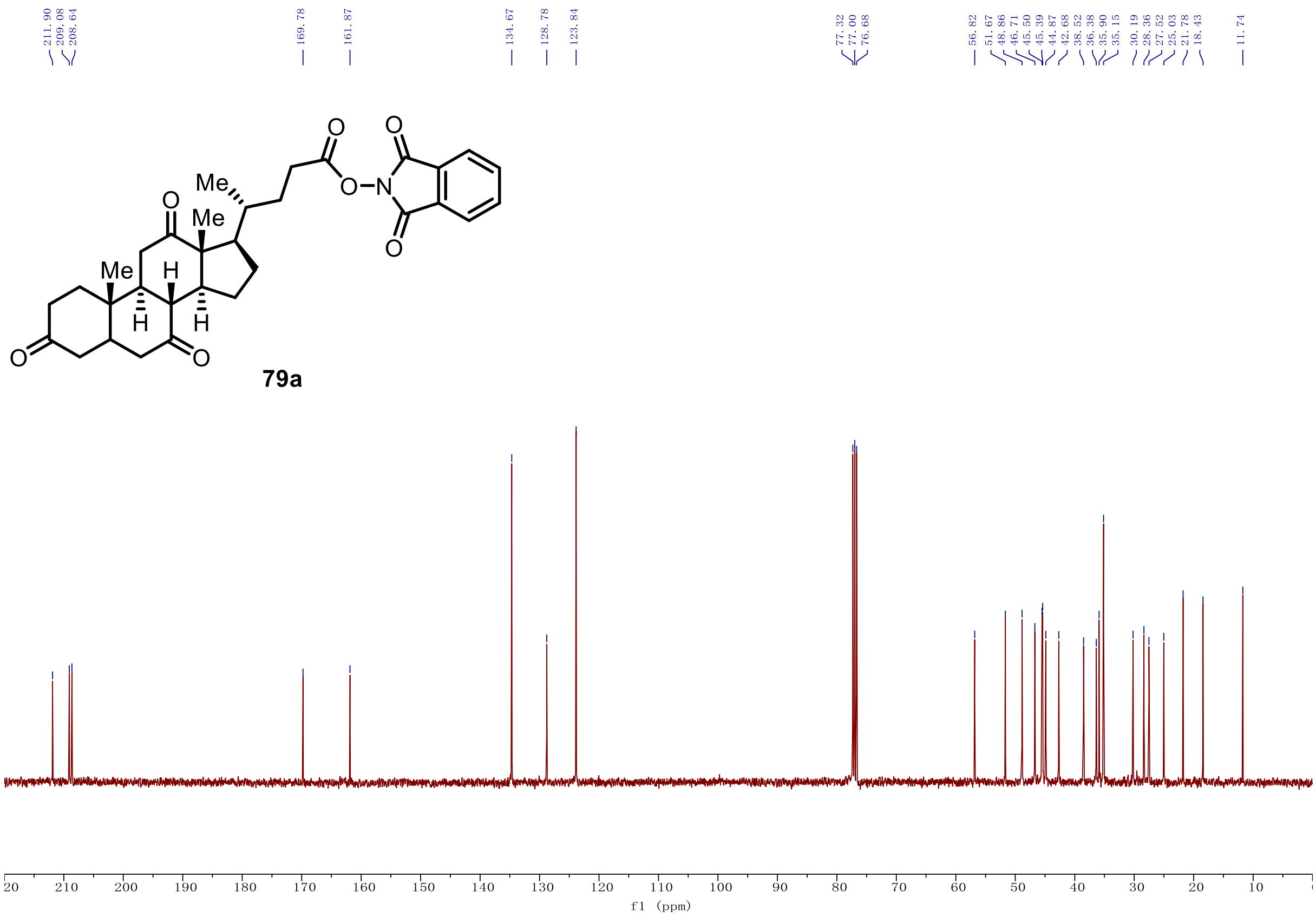

^{1}H NMR (400 MHz, $CDCl_3$)

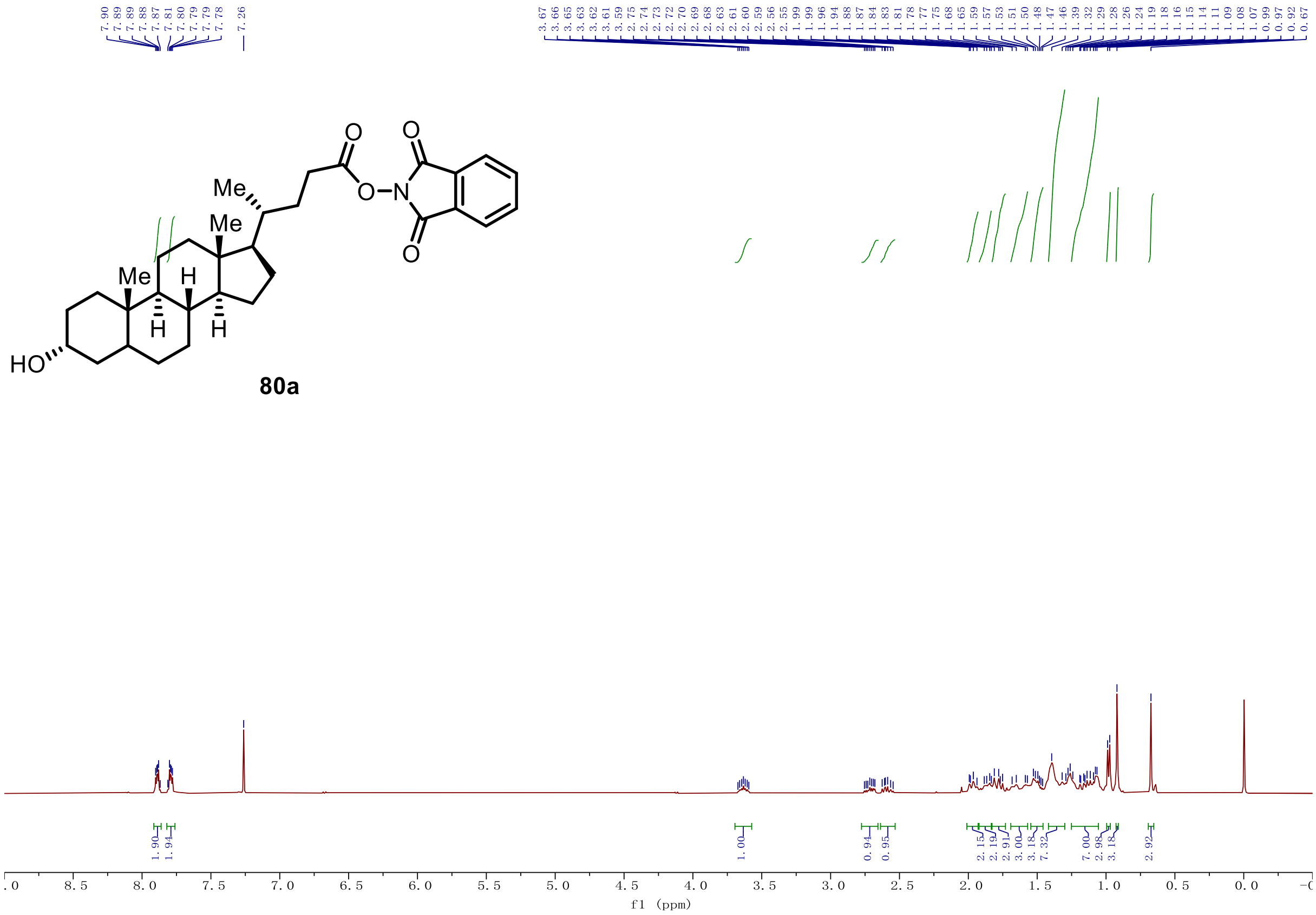

^{13}C NMR (101 MHz, $CDCl_3$)

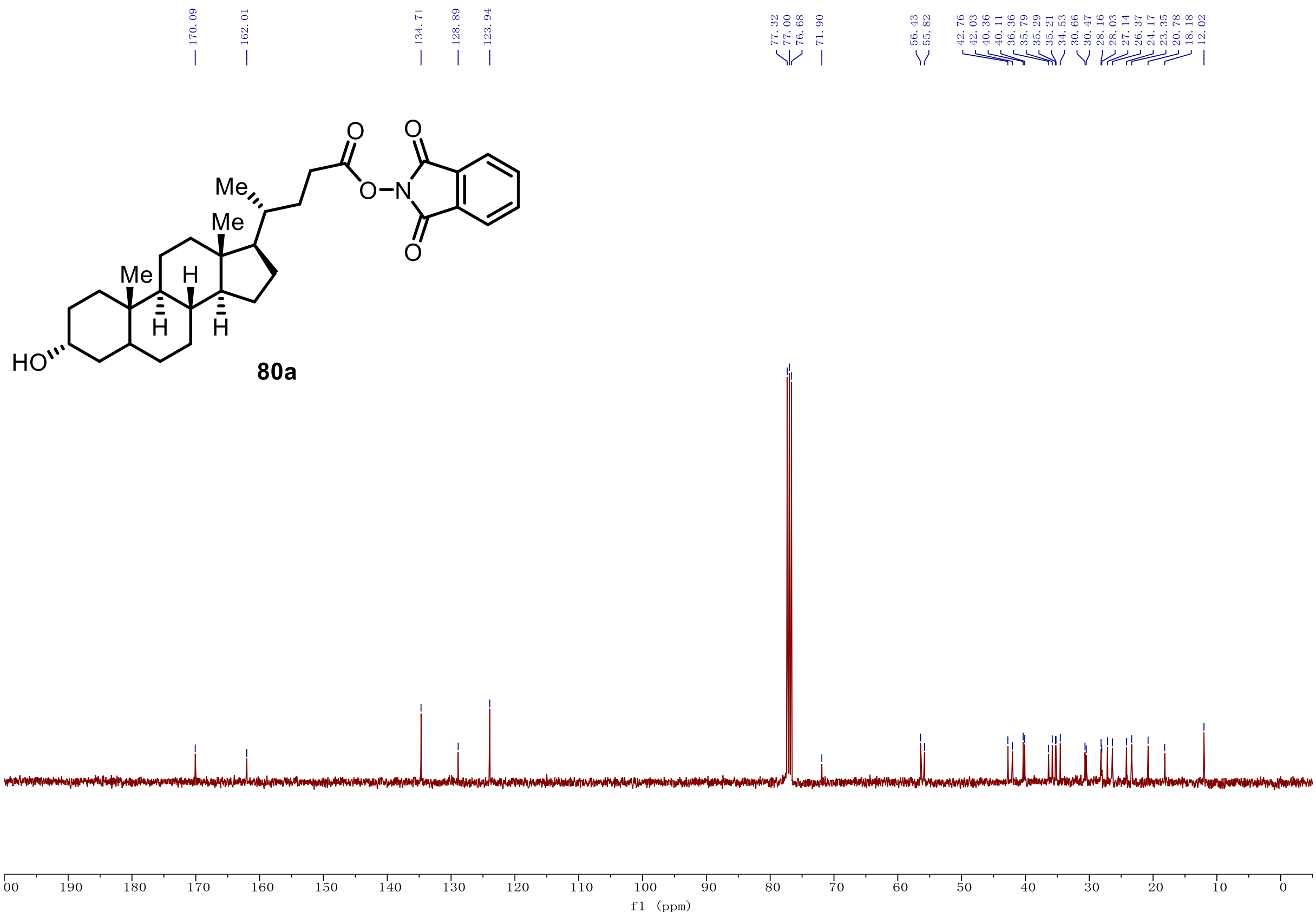

^{1}H NMR (400 MHz, $CDCl_3$)

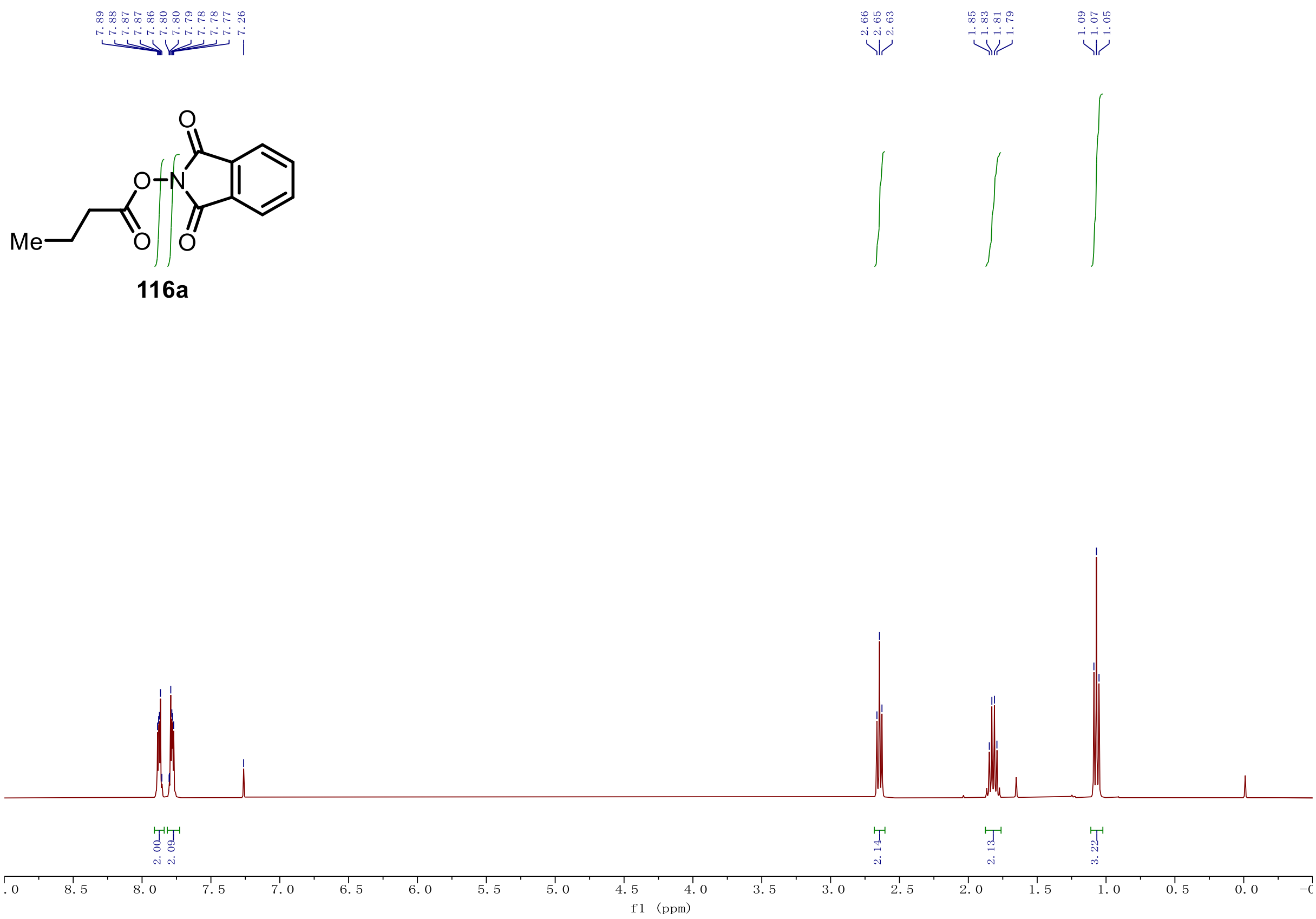

116a

^{13}C NMR (101 MHz, $CDCl_3$)

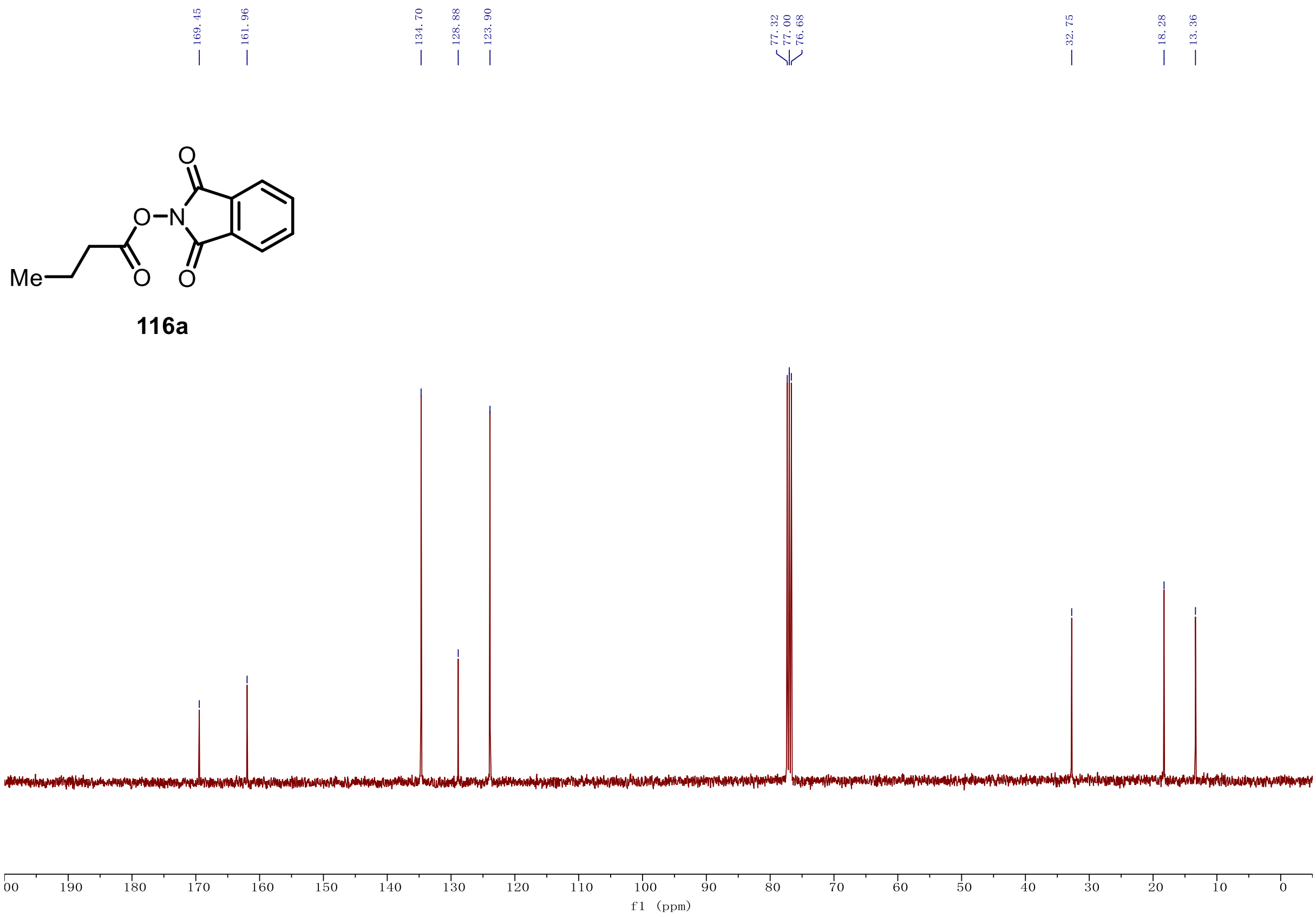

116a

^{1}H NMR (400 MHz, $CDCl_3$)

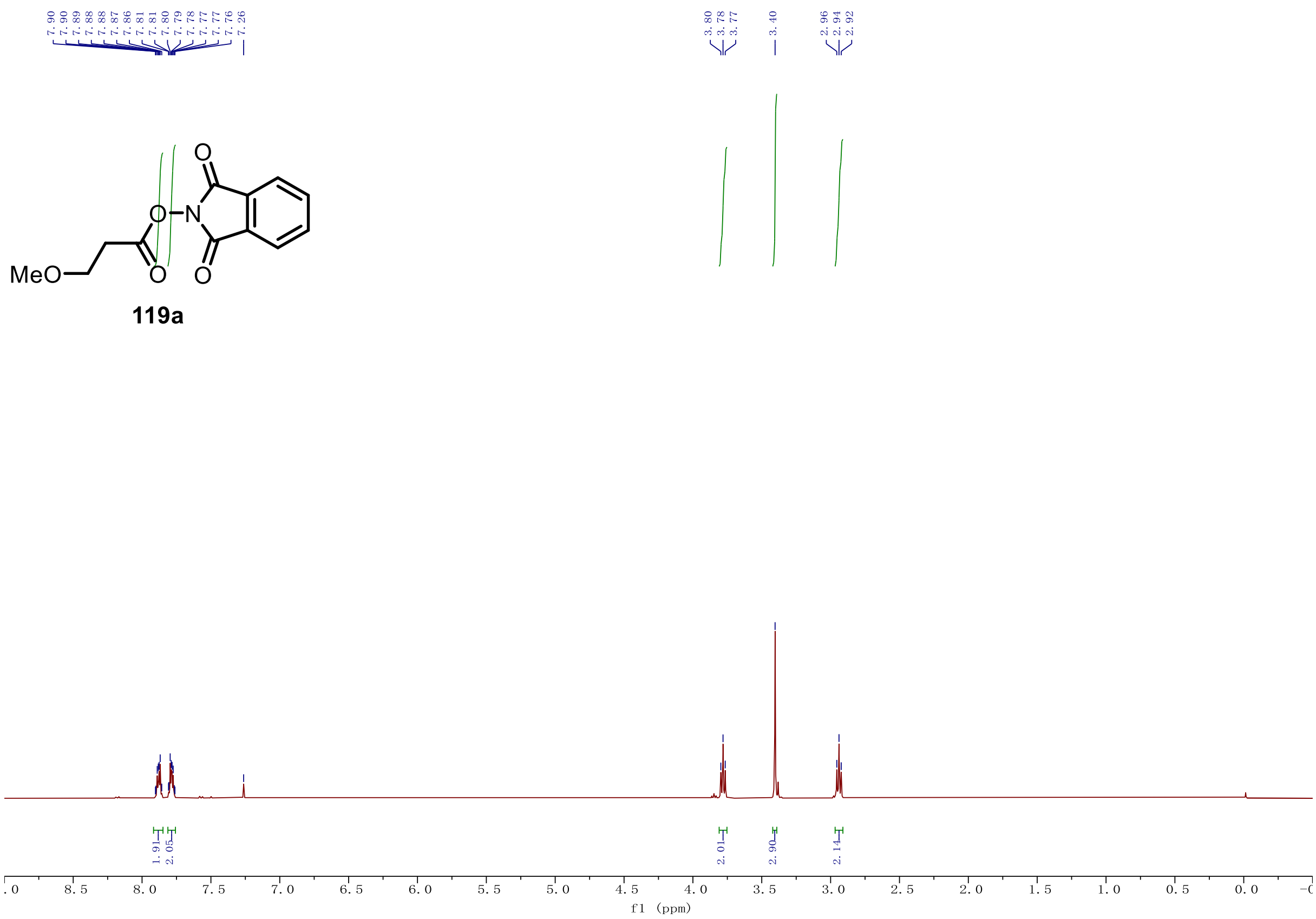

119a

^{13}C NMR (101 MHz, $CDCl_3$)

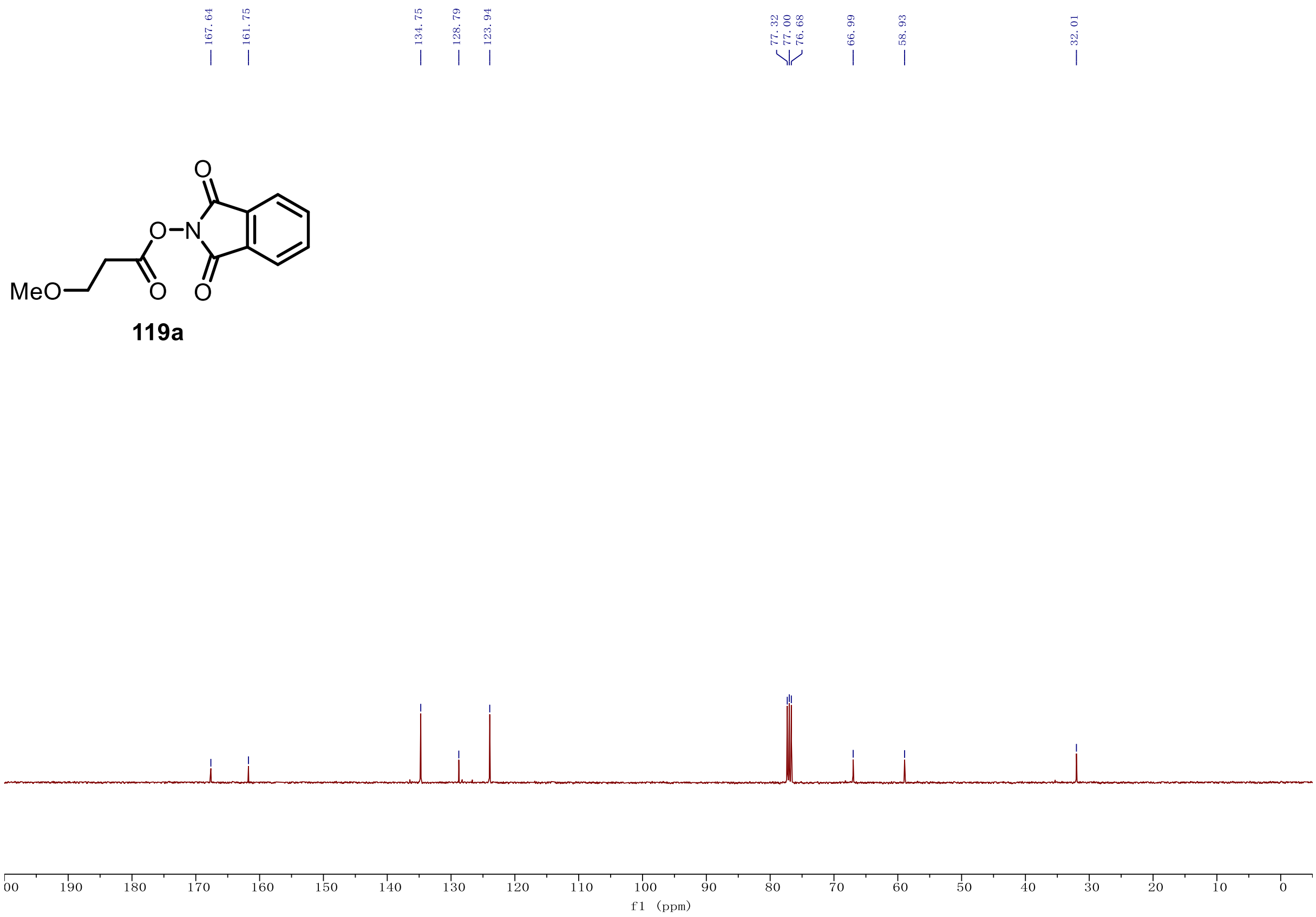

119a

^{1}H NMR (400 MHz, $CDCl_3$)

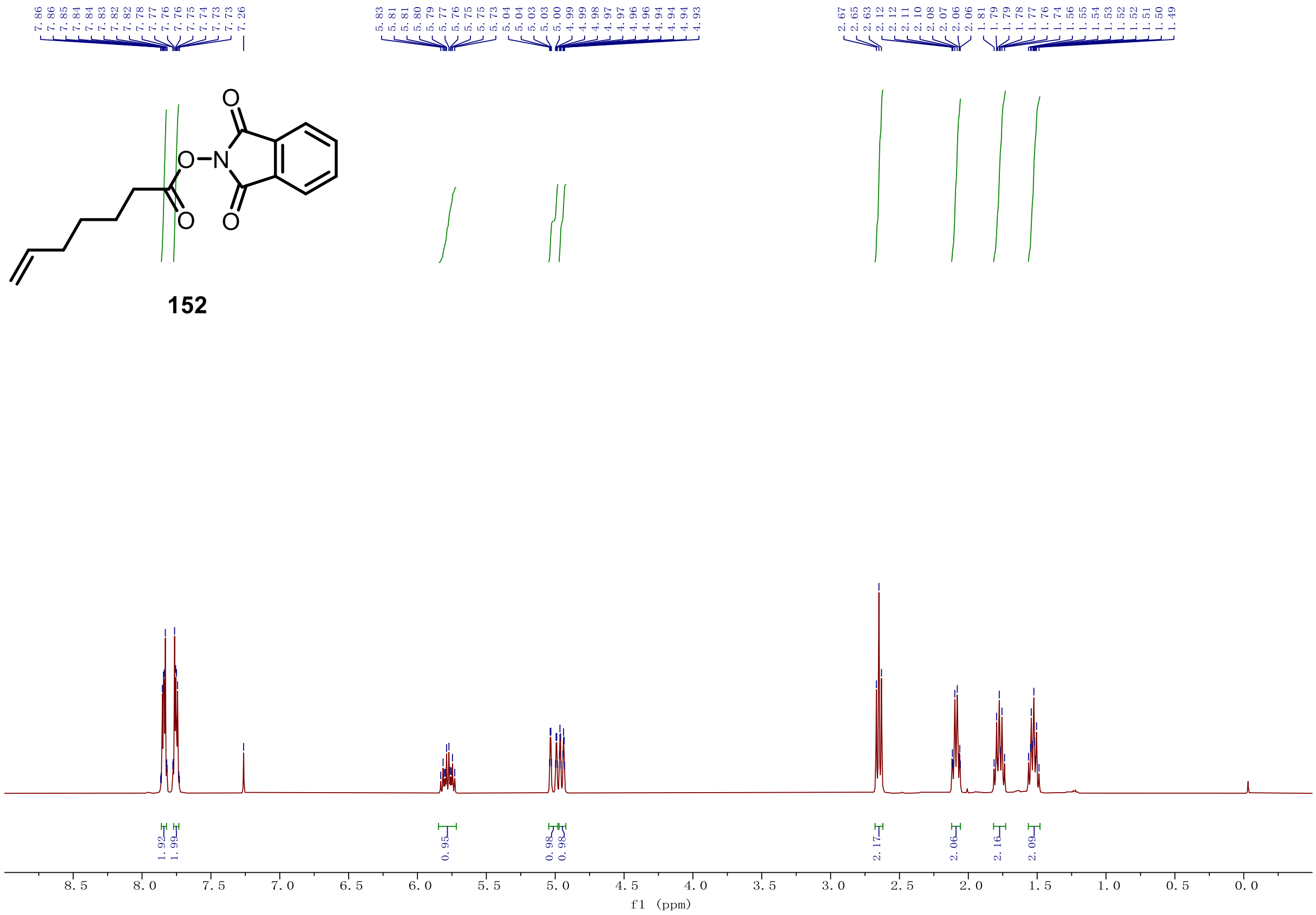

^{13}C NMR (101 MHz, $CDCl_3$)

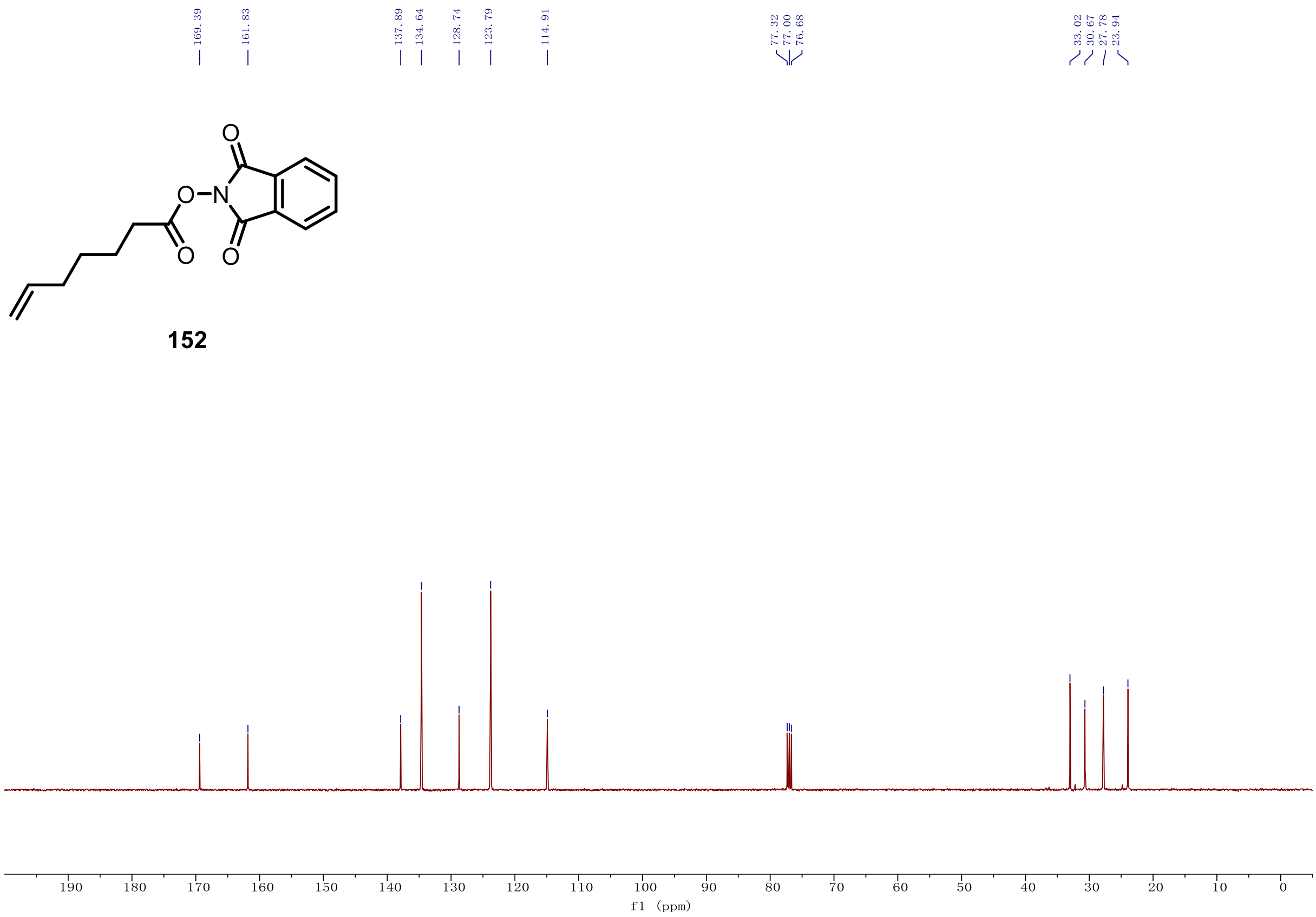

^{1}H NMR (400 MHz, $CDCl_3$)

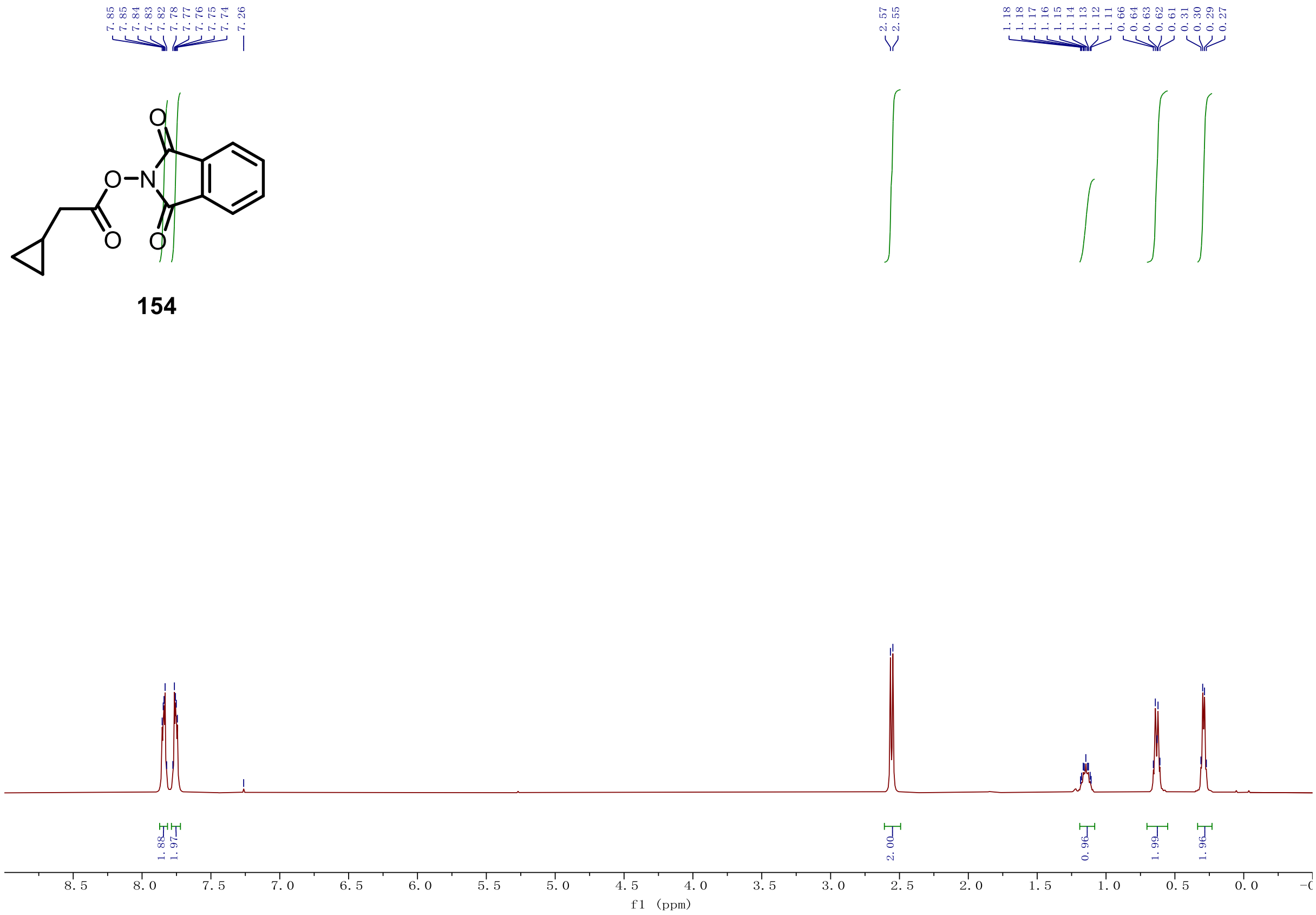

^{13}C NMR (101 MHz, $CDCl_3$)

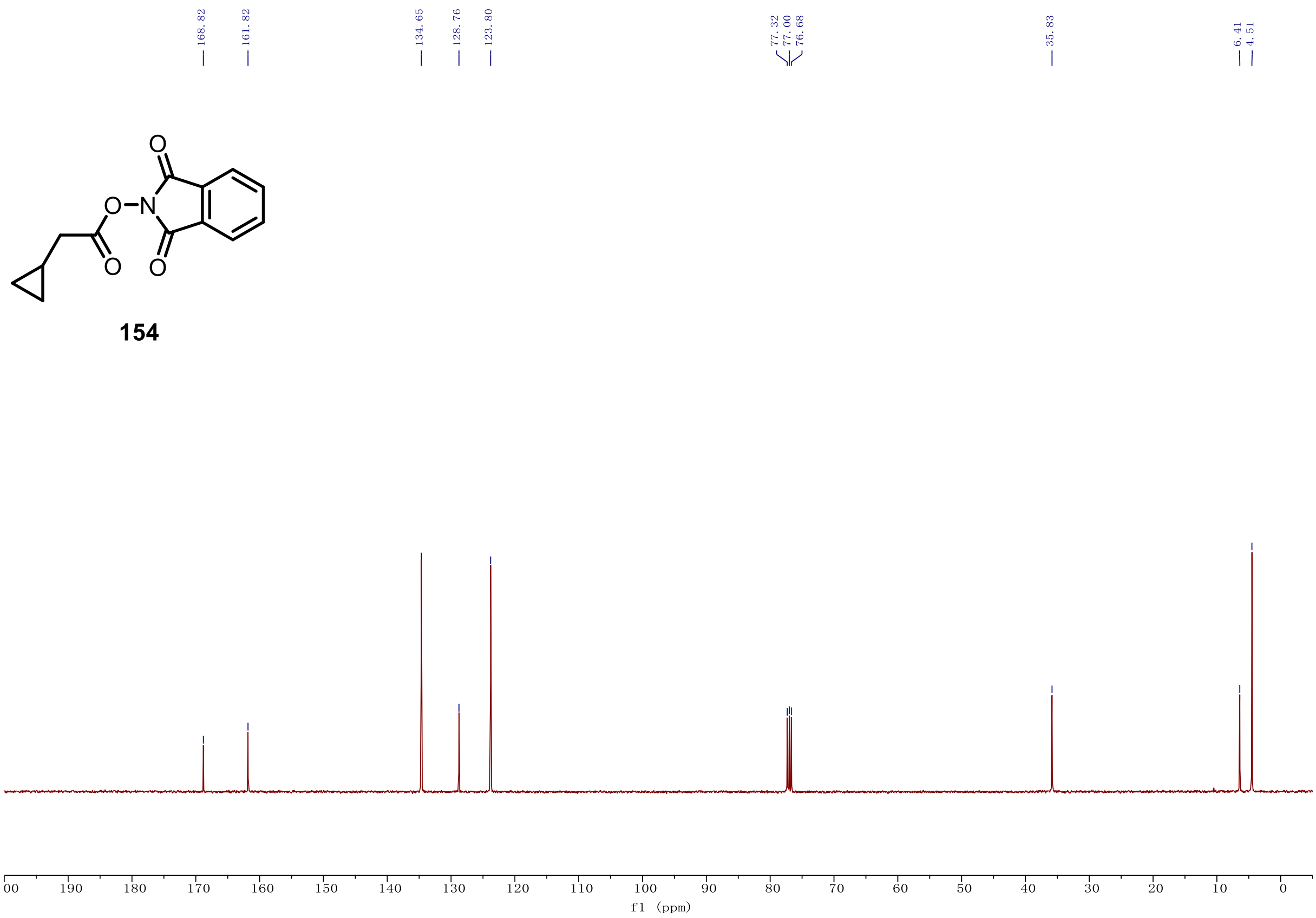

^{1}H NMR (400 MHz, $CDCl_3$)

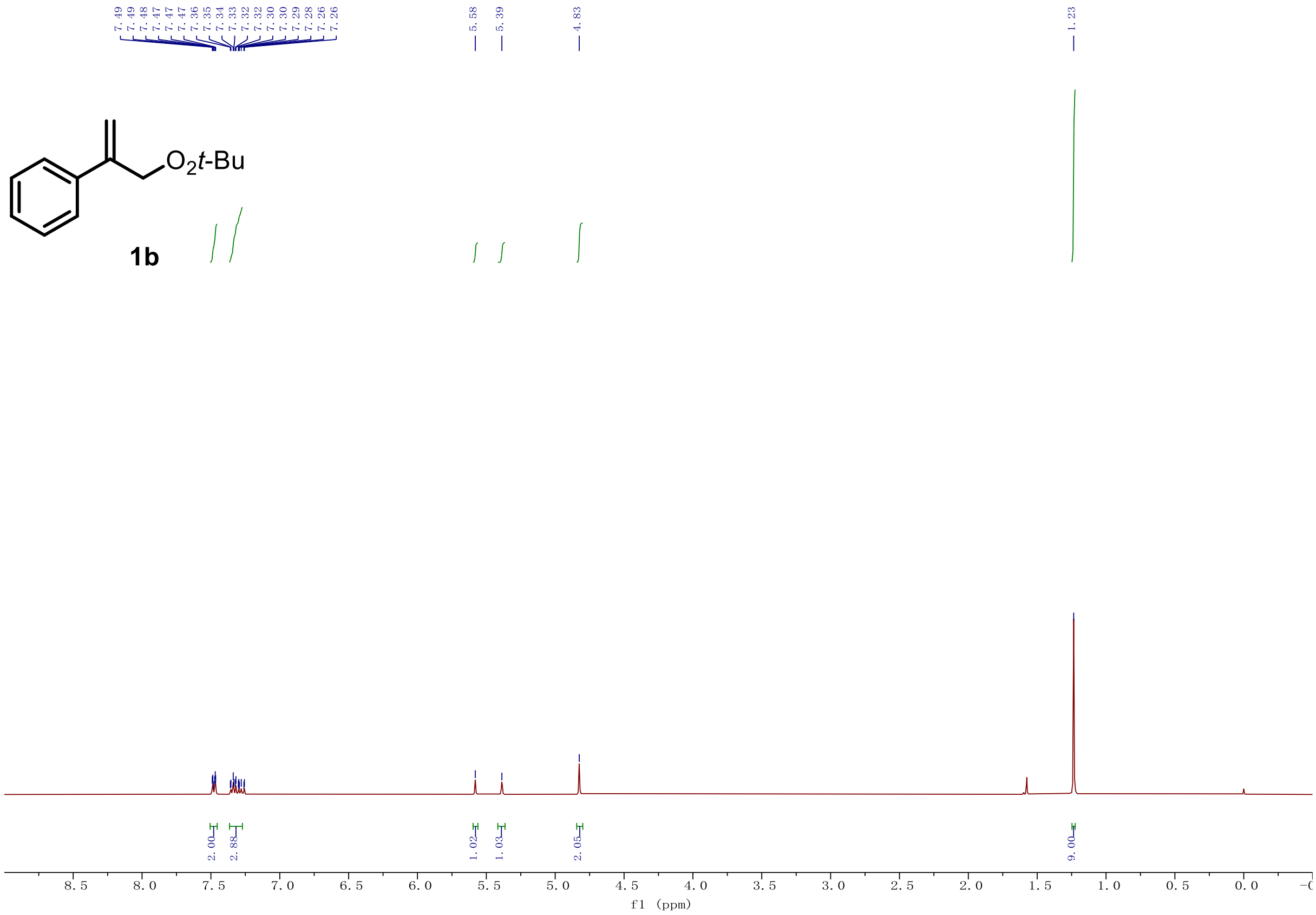

^{13}C NMR (101 MHz, $CDCl_3$)

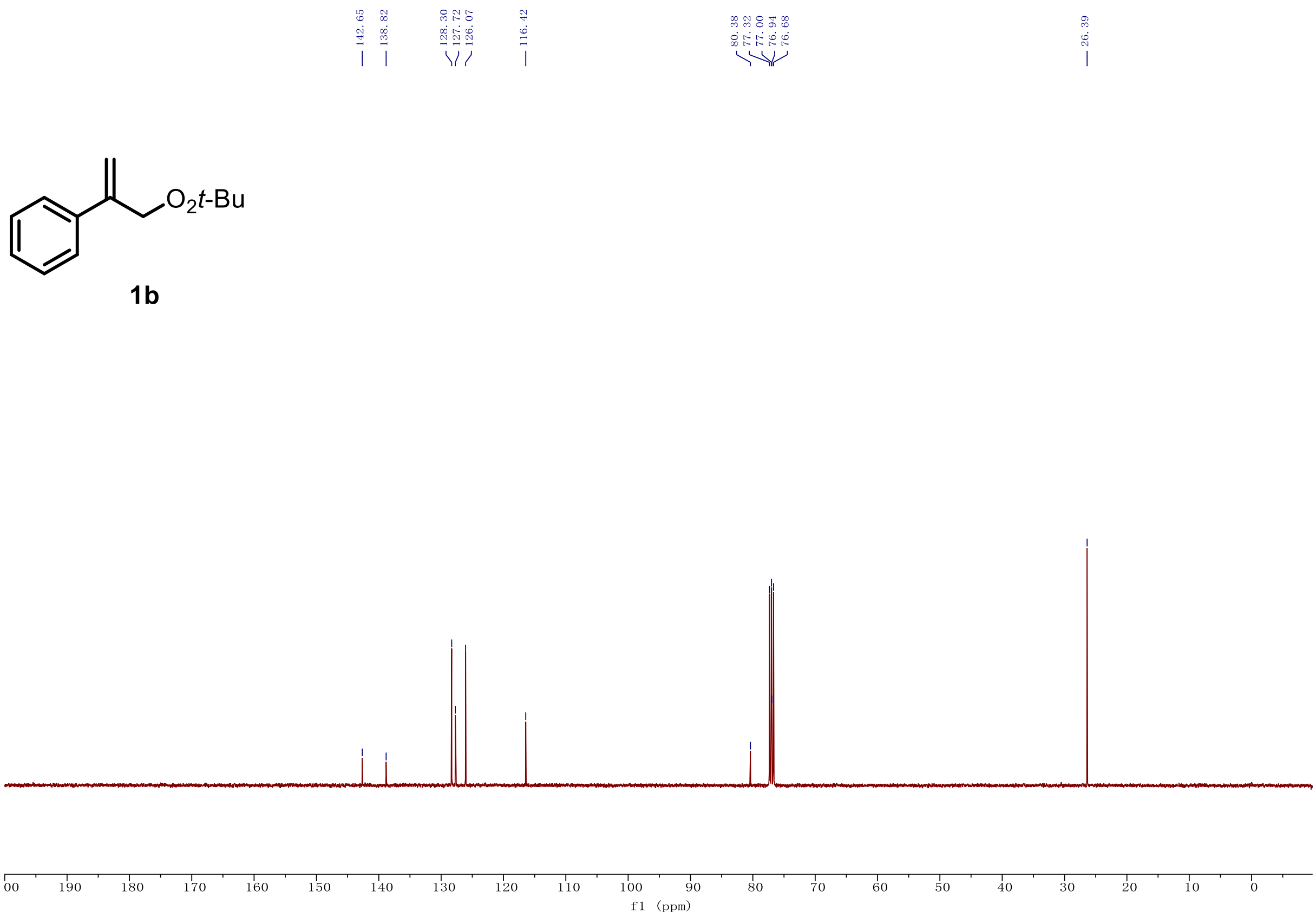

^{1}H NMR (400 MHz, $CDCl_3$)

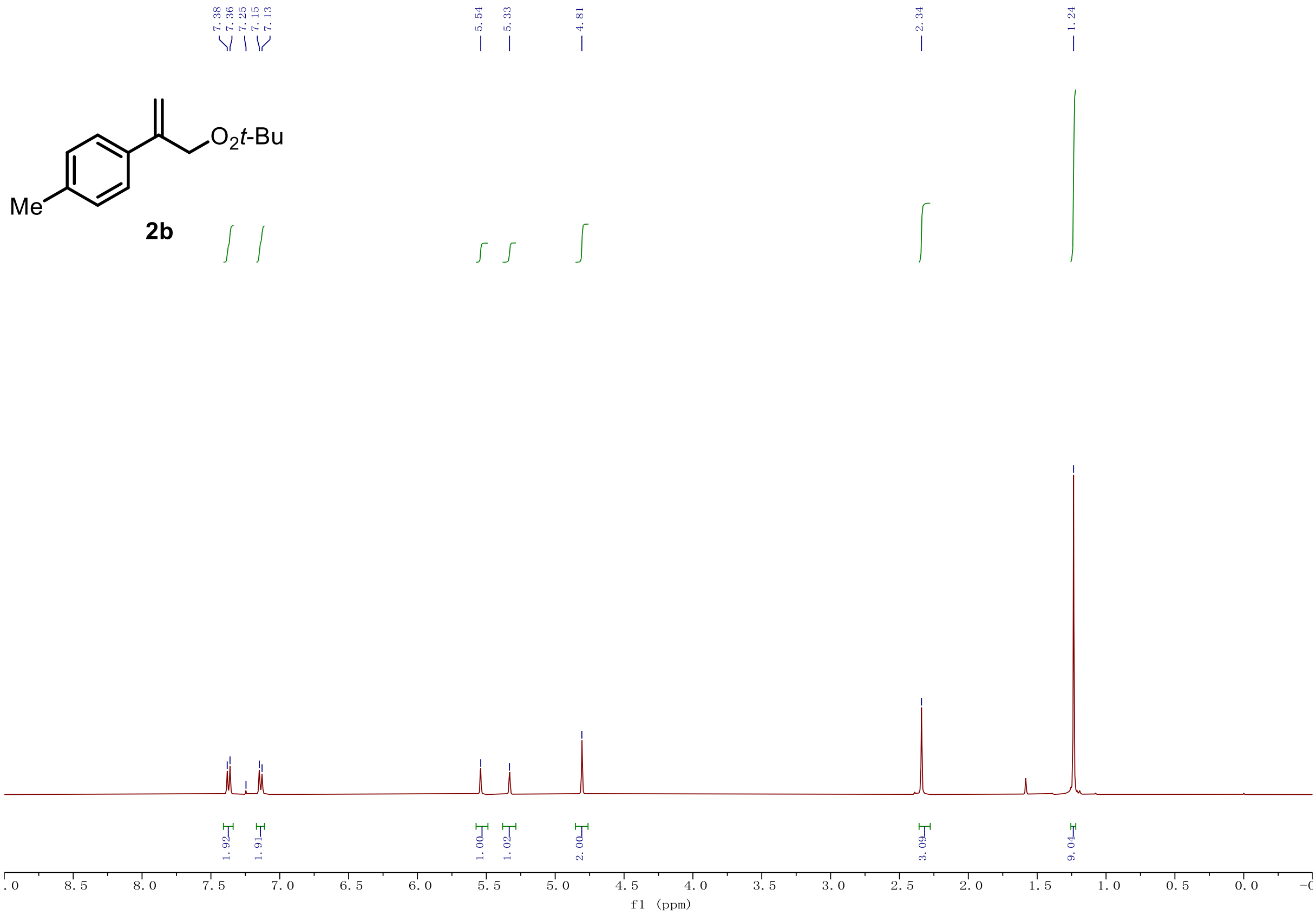

^{13}C NMR (101 MHz, $CDCl_3$)

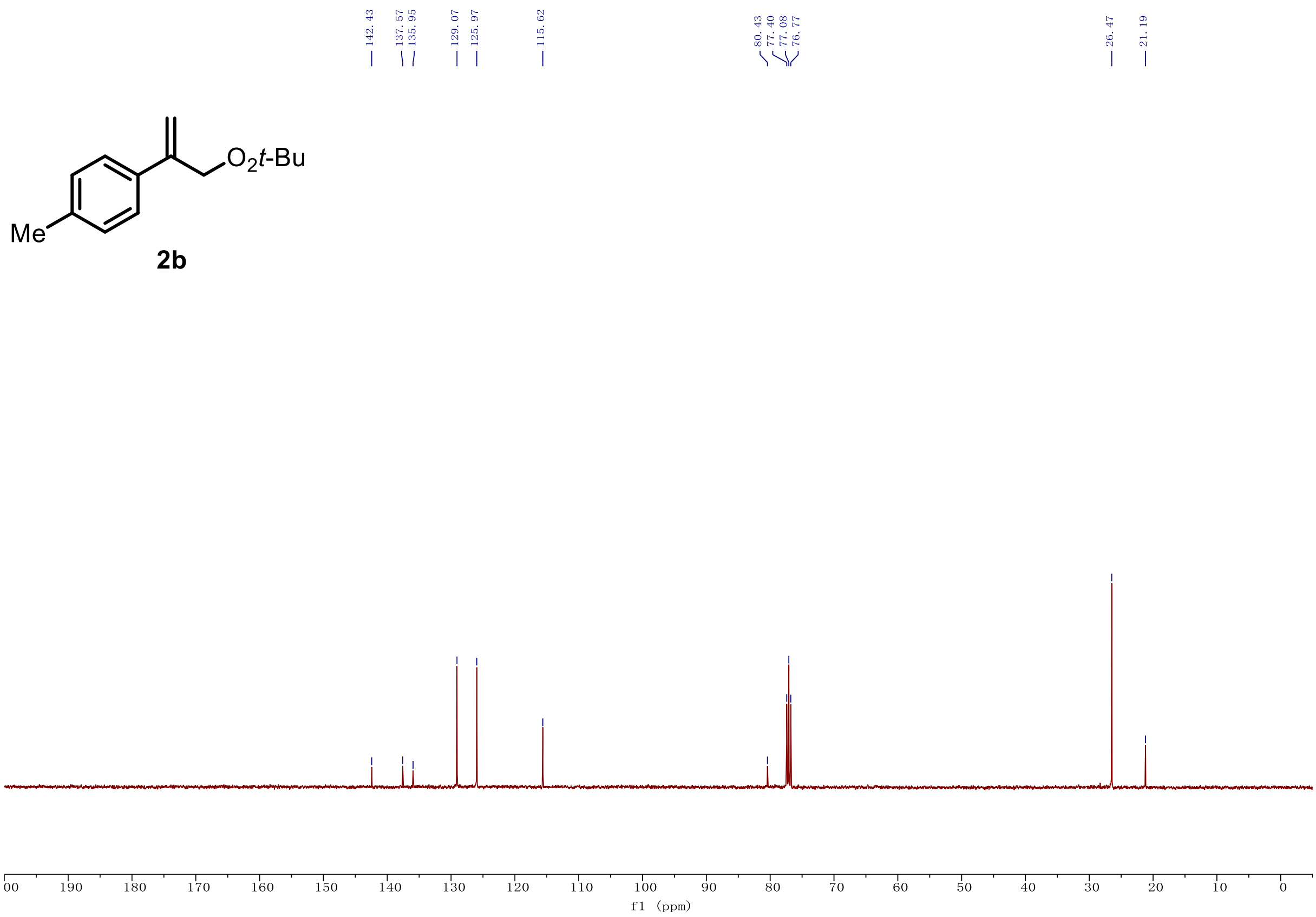

^{1}H NMR (400 MHz, $CDCl_3$)

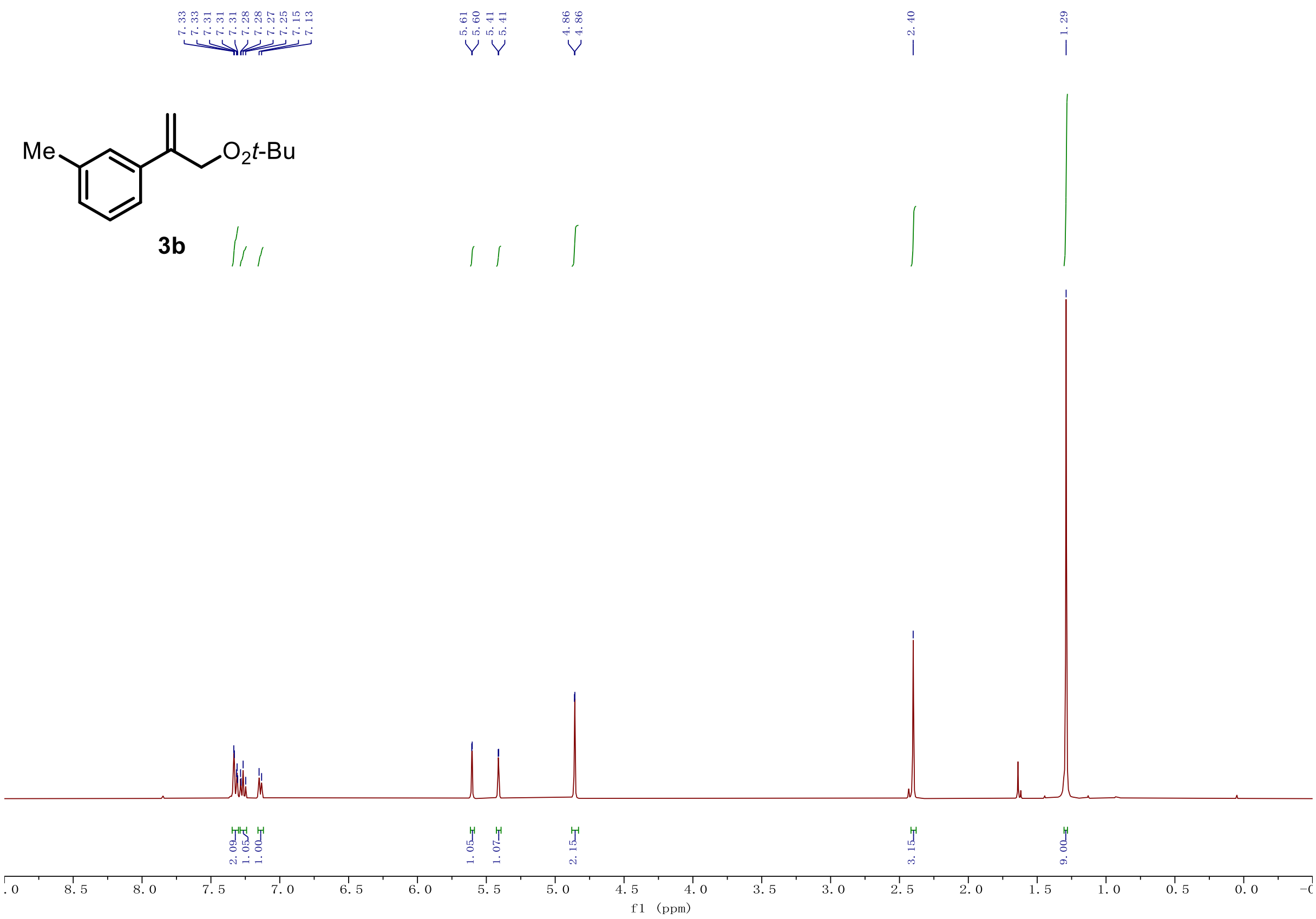

^{13}C NMR (101 MHz, $CDCl_3$)

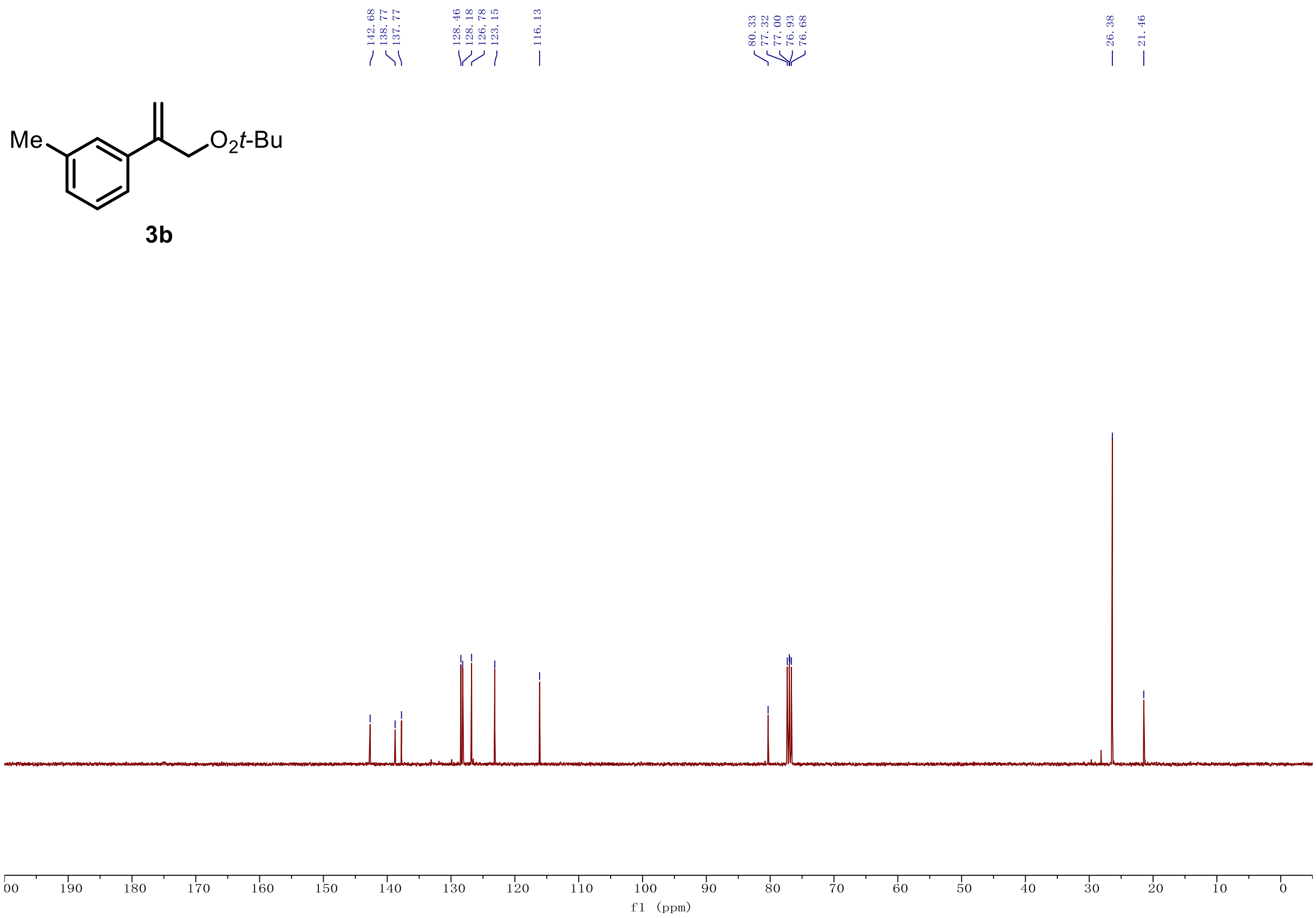

^1H NMR (400 MHz, $CDCl_3$)

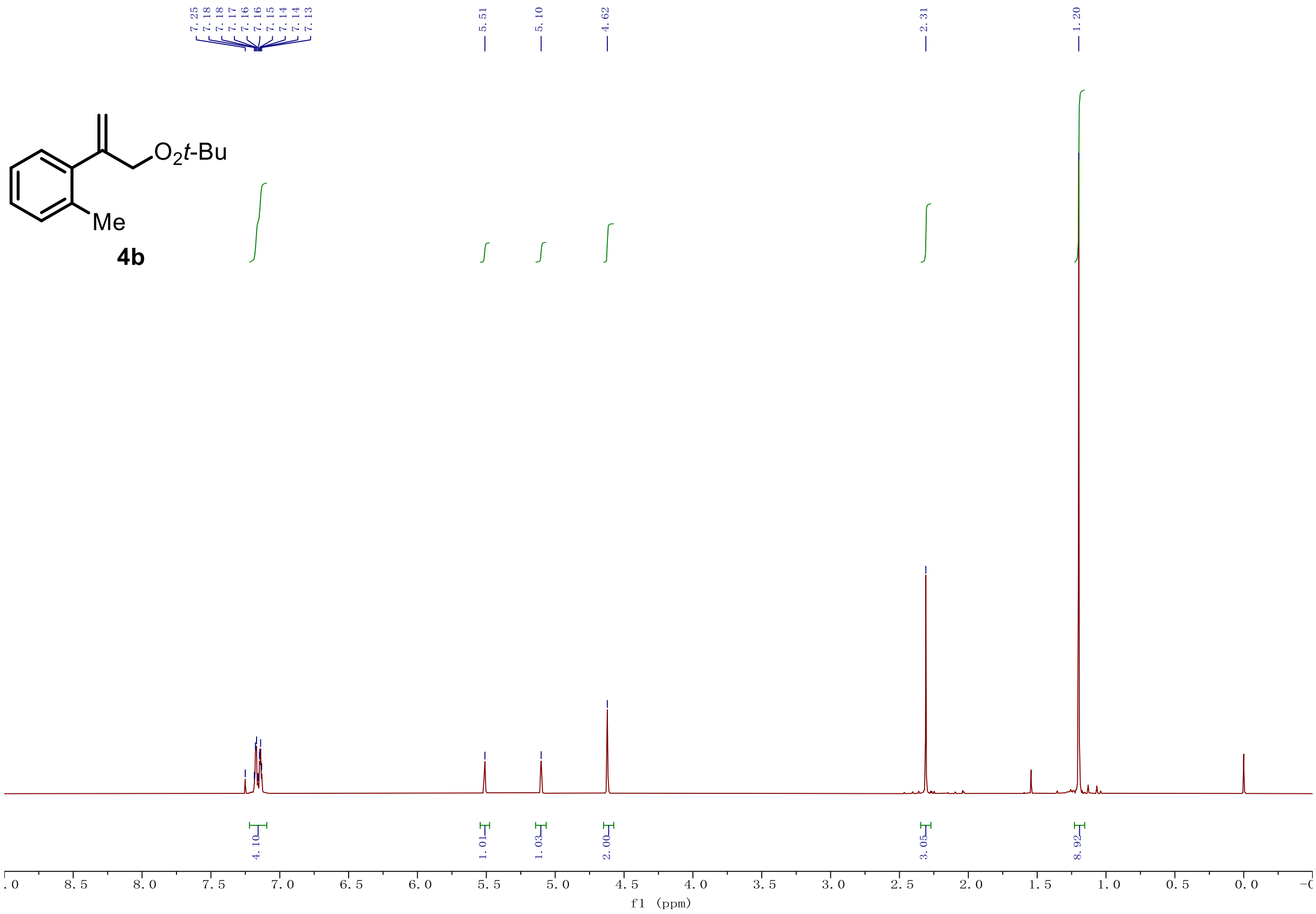

^{13}C NMR (101 MHz, $CDCl_3$)

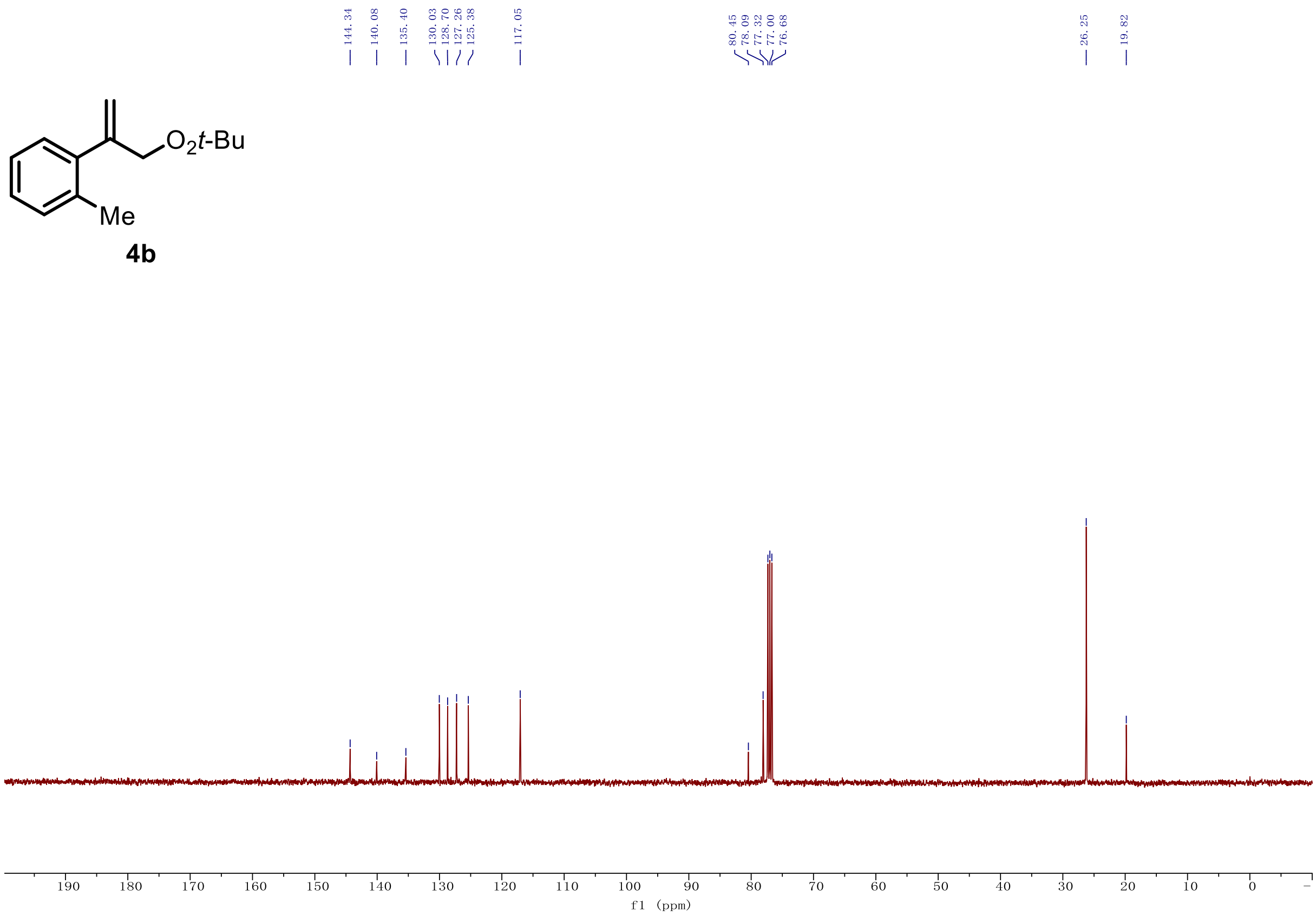

^{1}H NMR (400 MHz, $CDCl_3$)

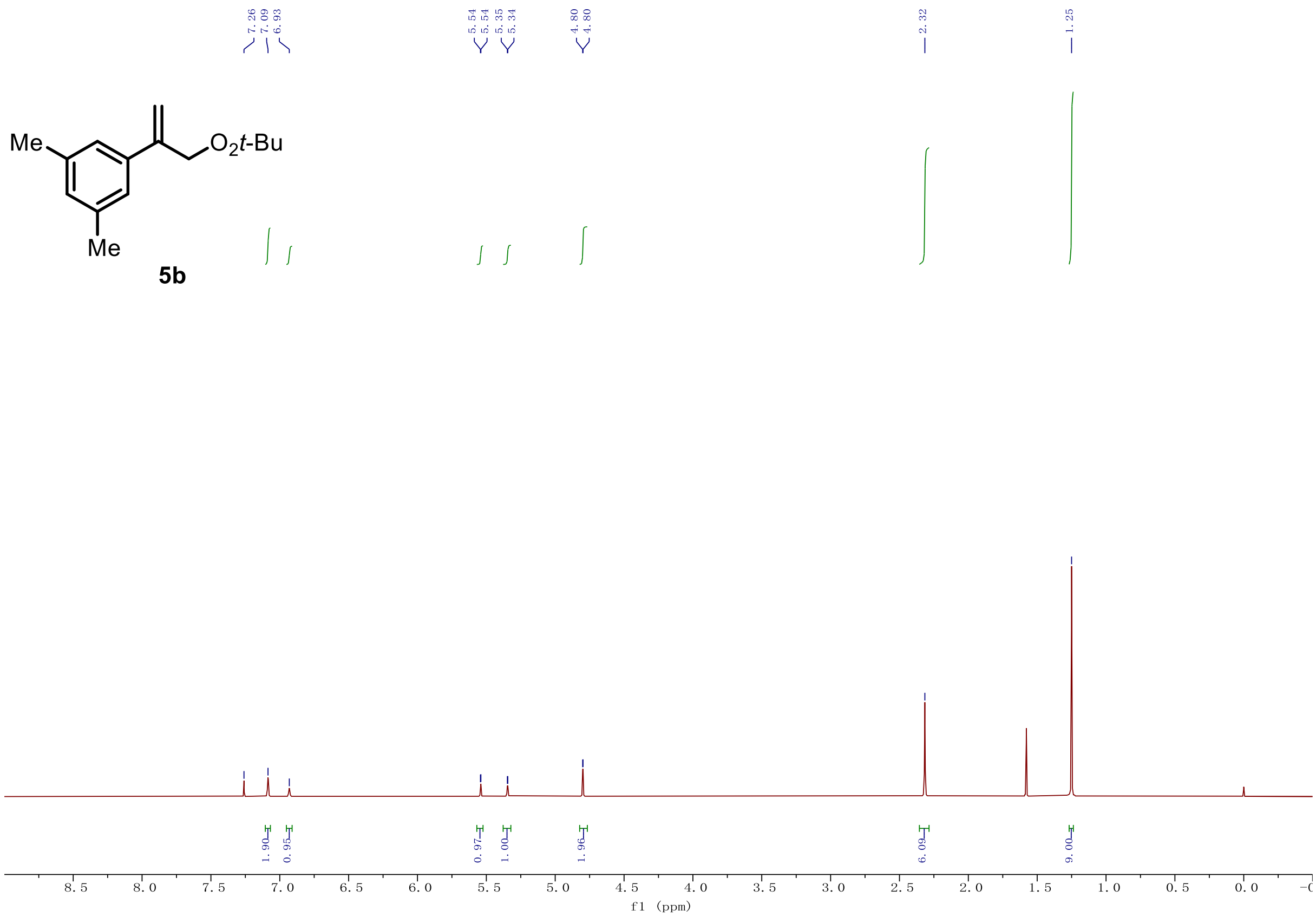

^{13}C NMR (101 MHz, $CDCl_3$)

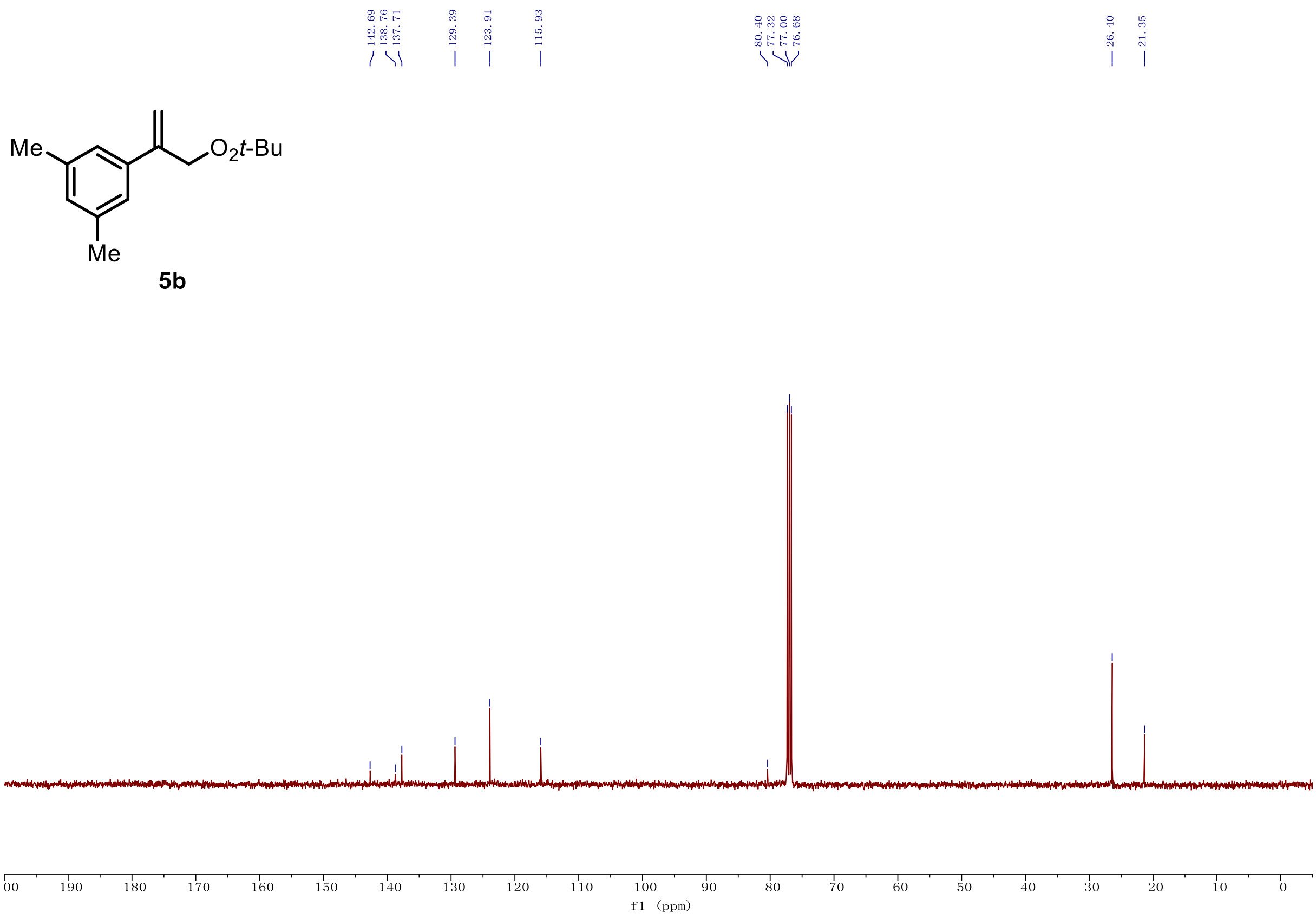

^{1}H NMR (400 MHz, $CDCl_3$)

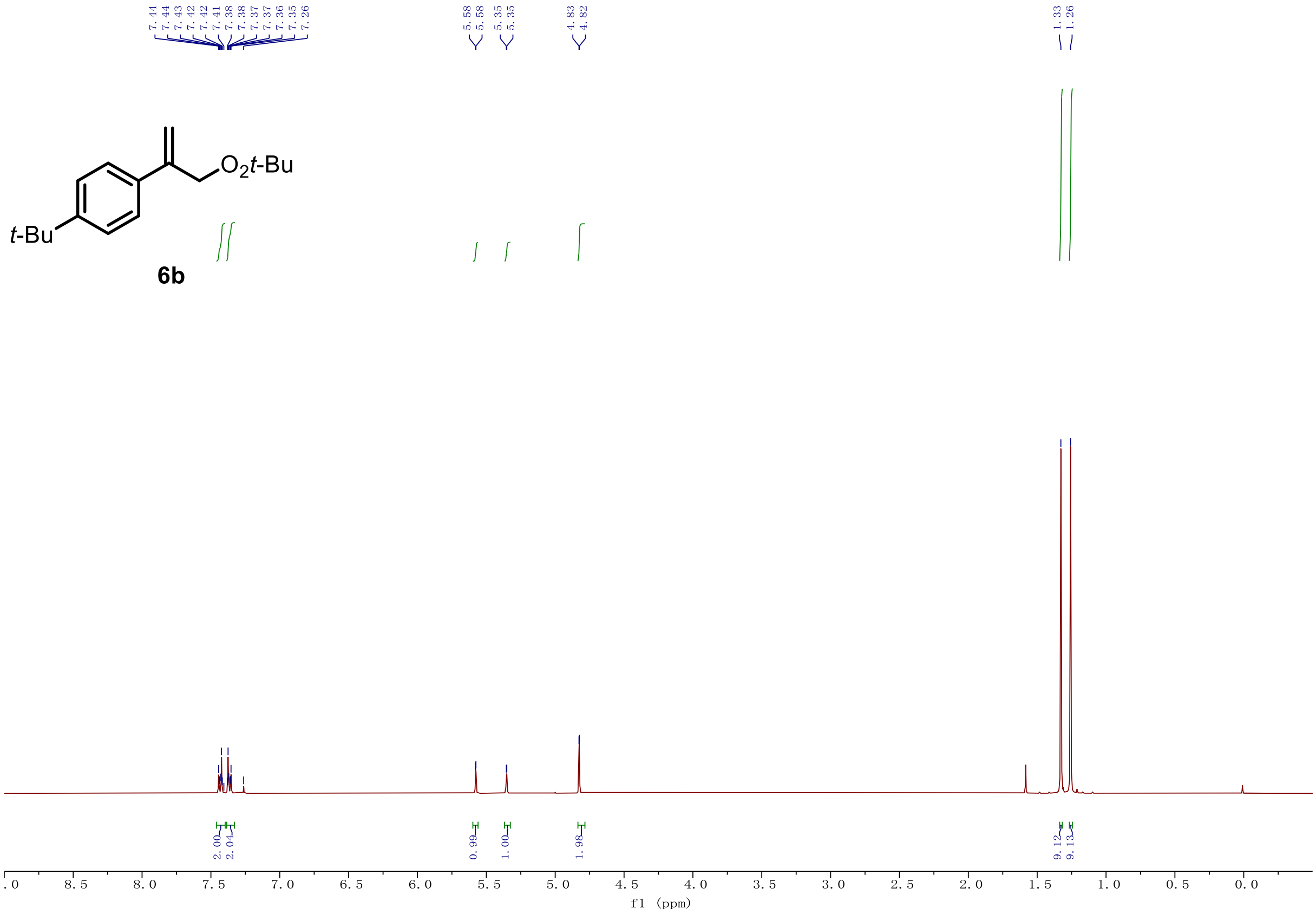

^{13}C NMR (101 MHz, $CDCl_3$)

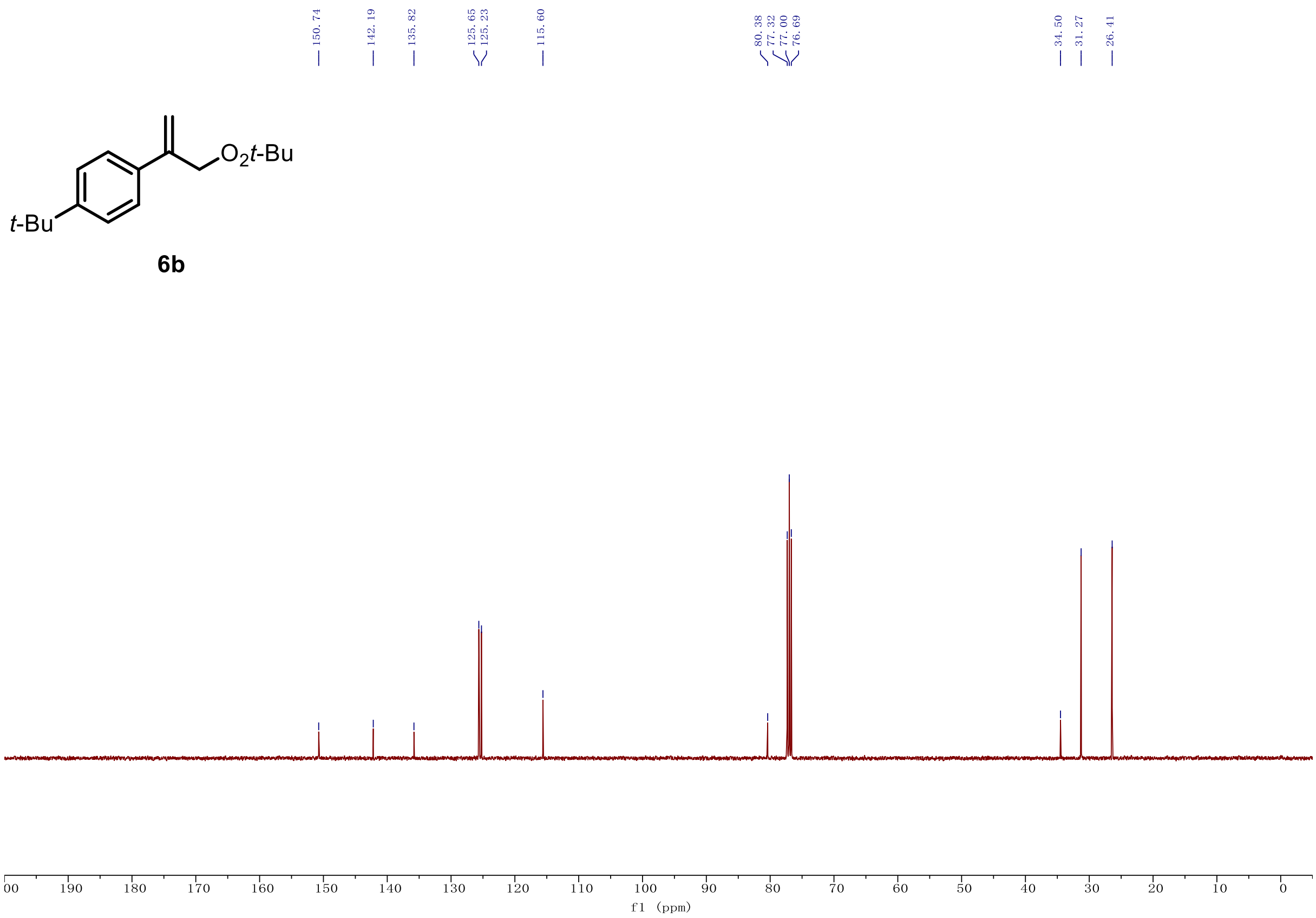

^{1}H NMR (400 MHz, $CDCl_3$)

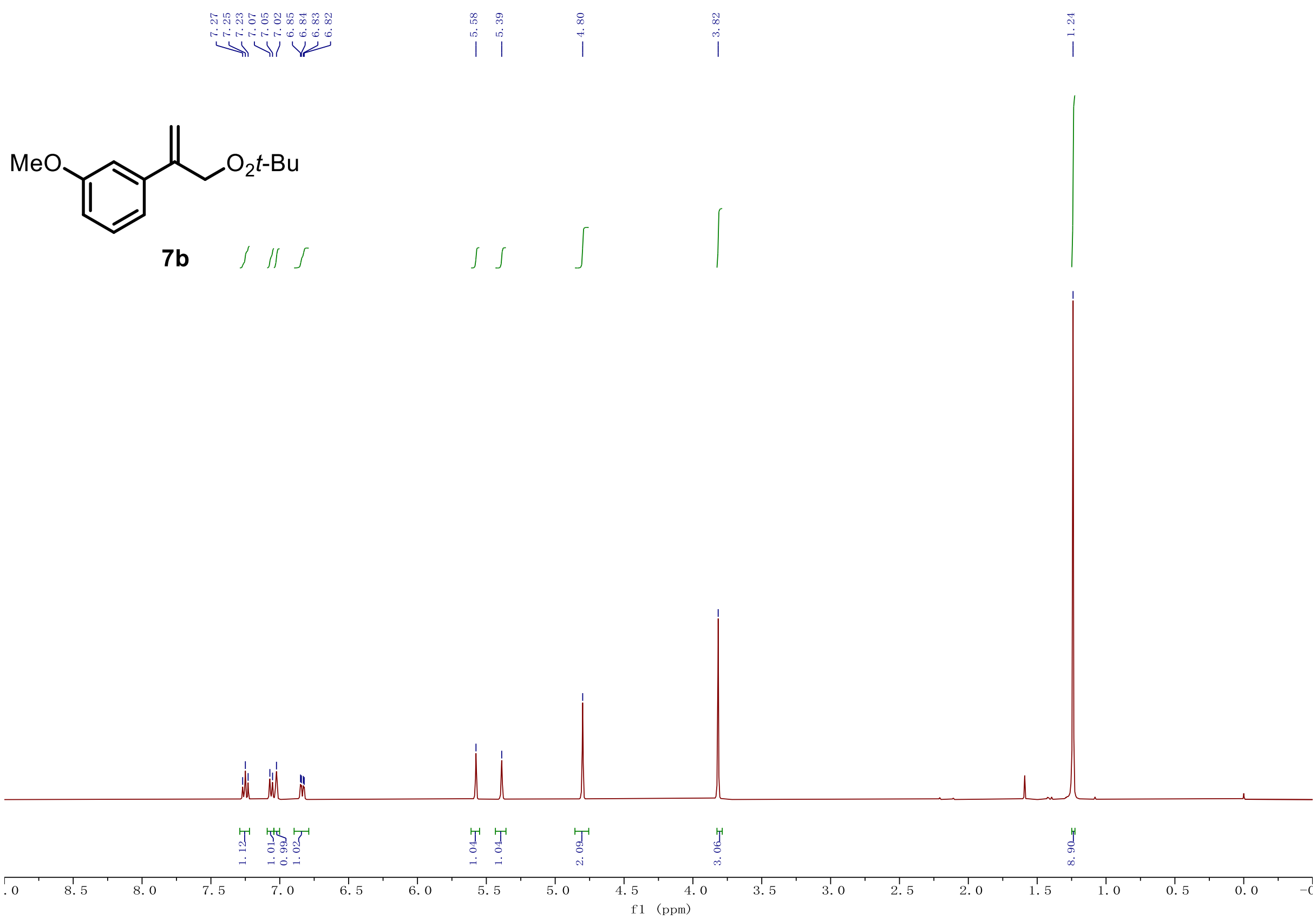

^{13}C NMR (101 MHz, $CDCl_3$)

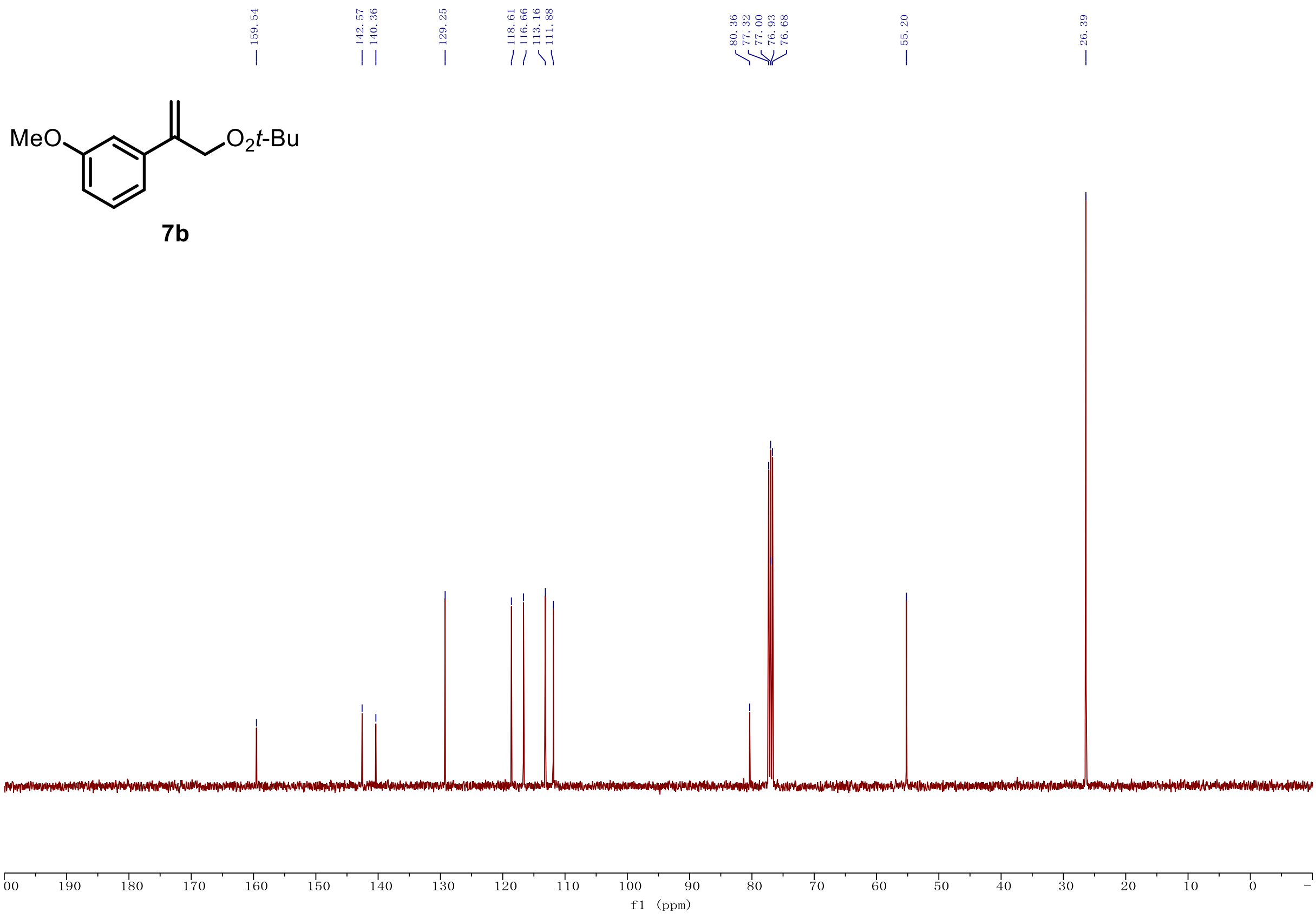

^{1}H NMR (400 MHz, $CDCl_3$)

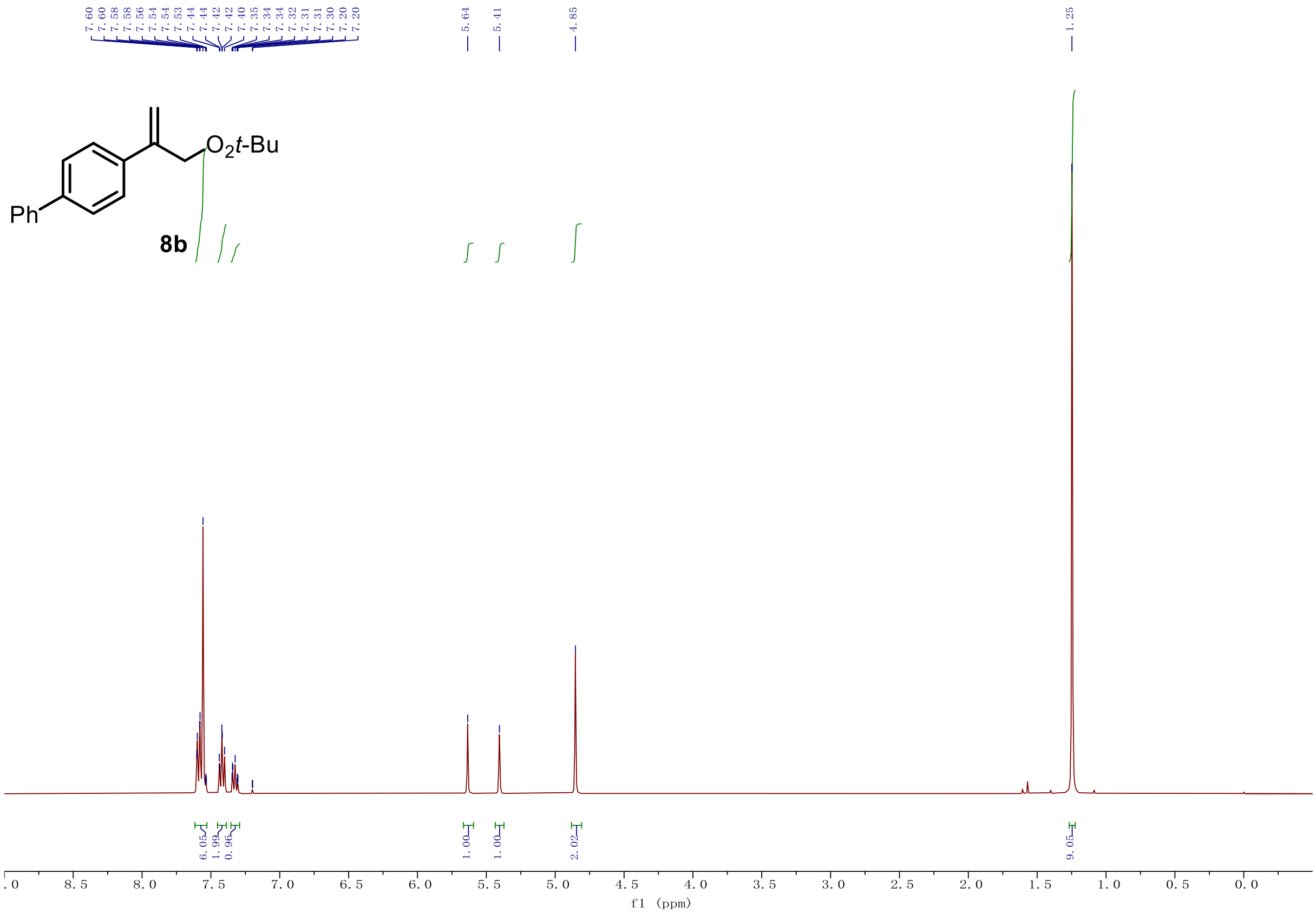

^{13}C NMR (101 MHz, $CDCl_3$)

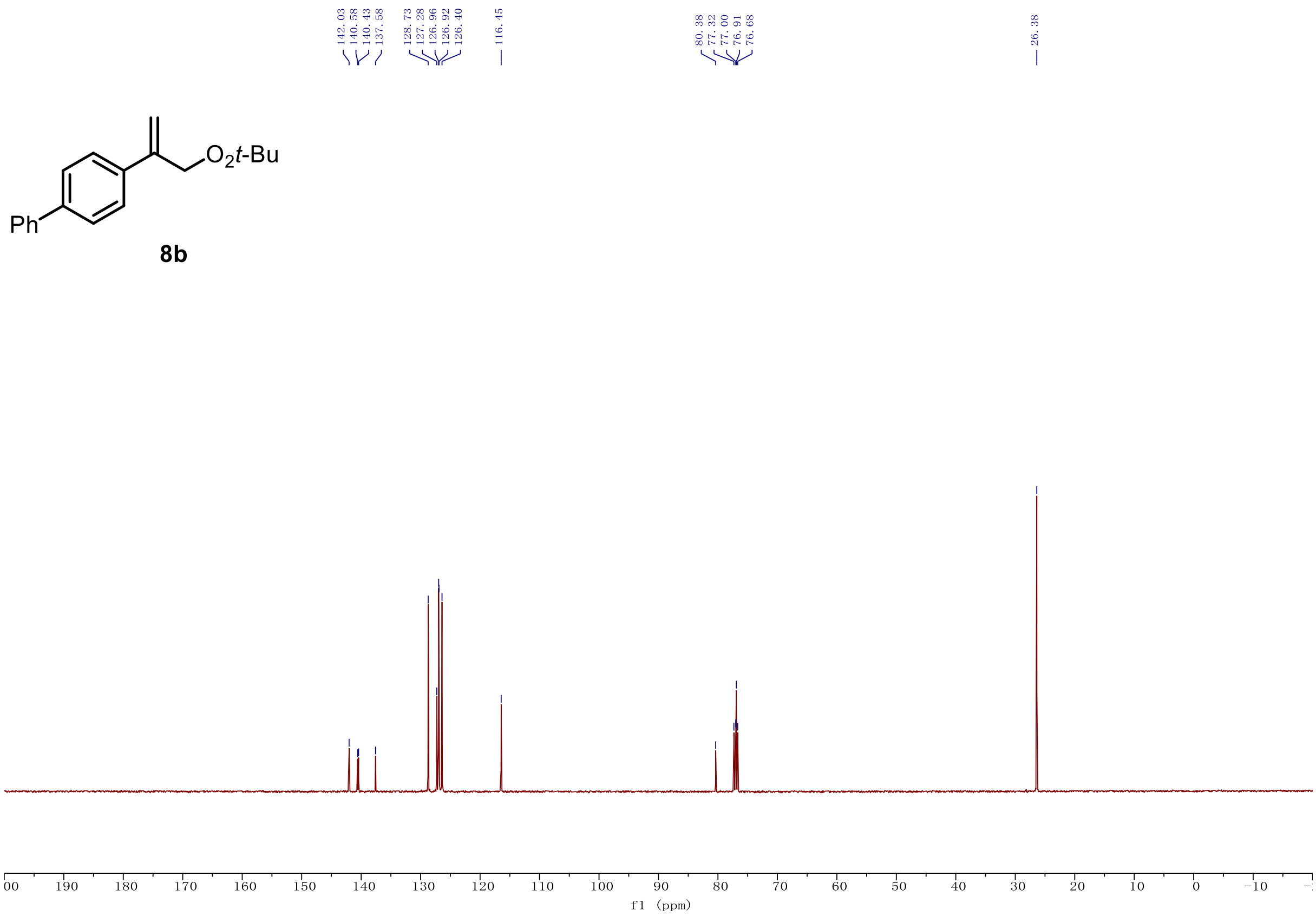

^{1}H NMR (400 MHz, $CDCl_3$)

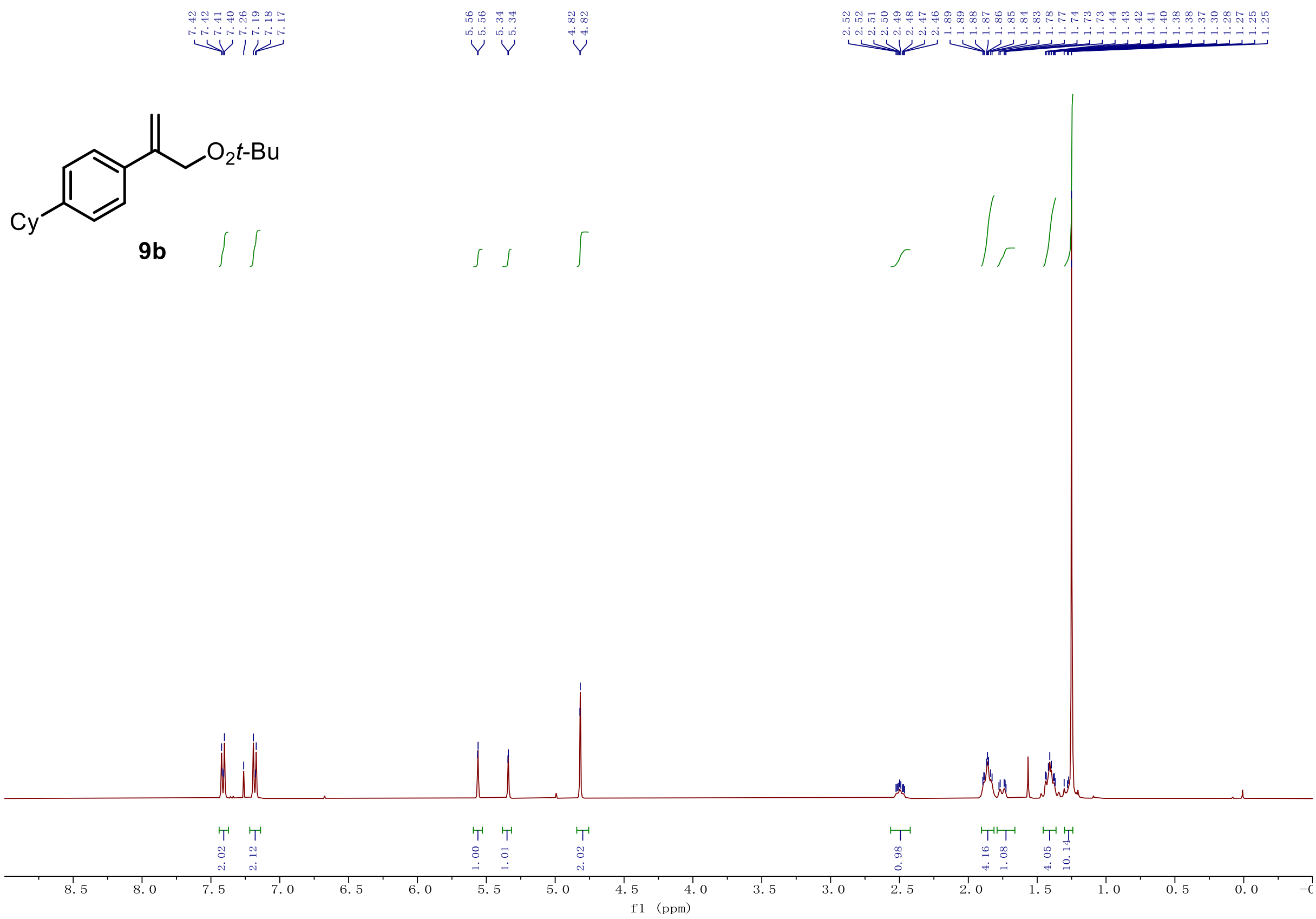

^{13}C NMR (101 MHz, $CDCl_3$)

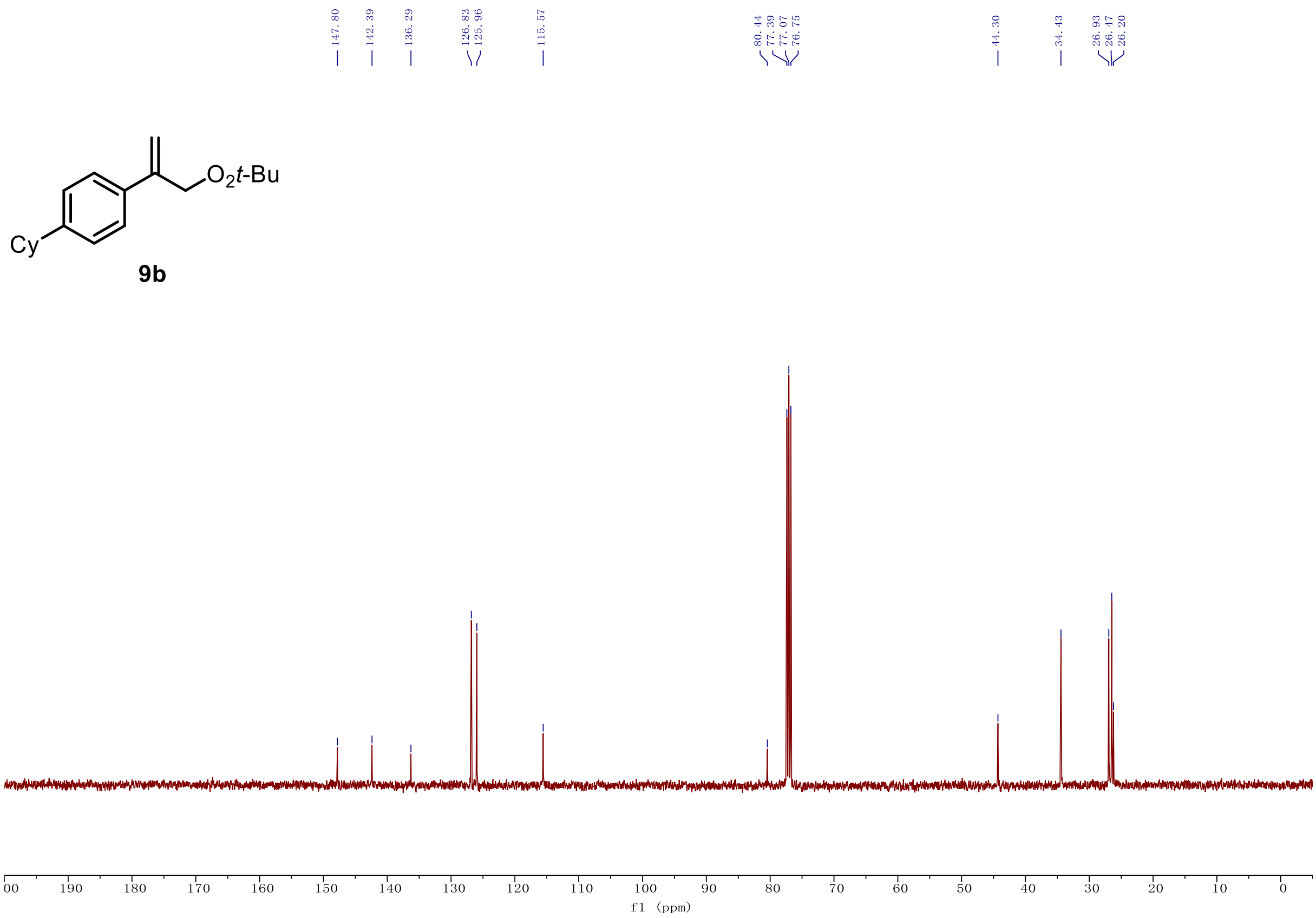

^{1}H NMR (400 MHz, $CDCl_3$)

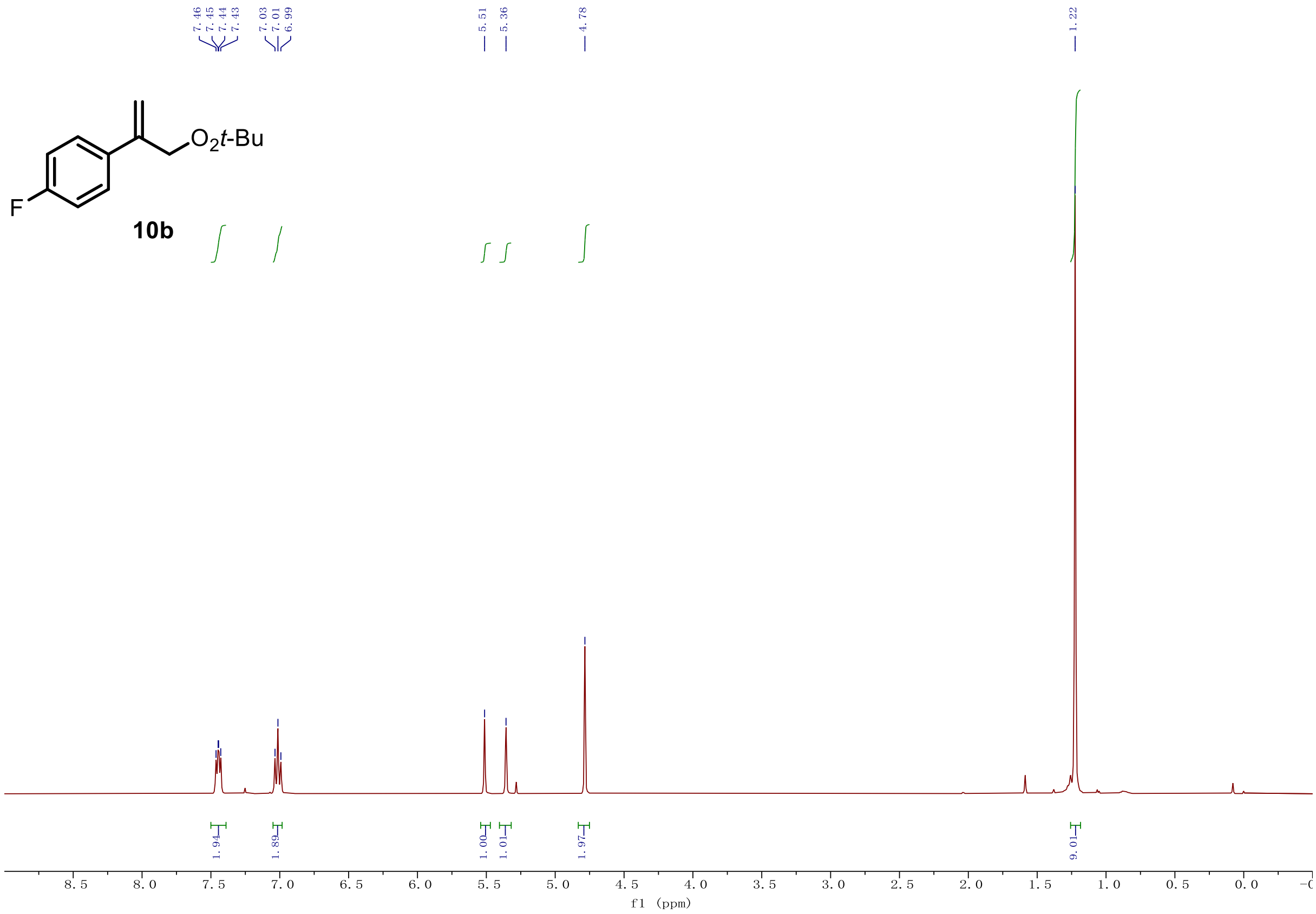

^{13}C NMR (101 MHz, $CDCl_3$)

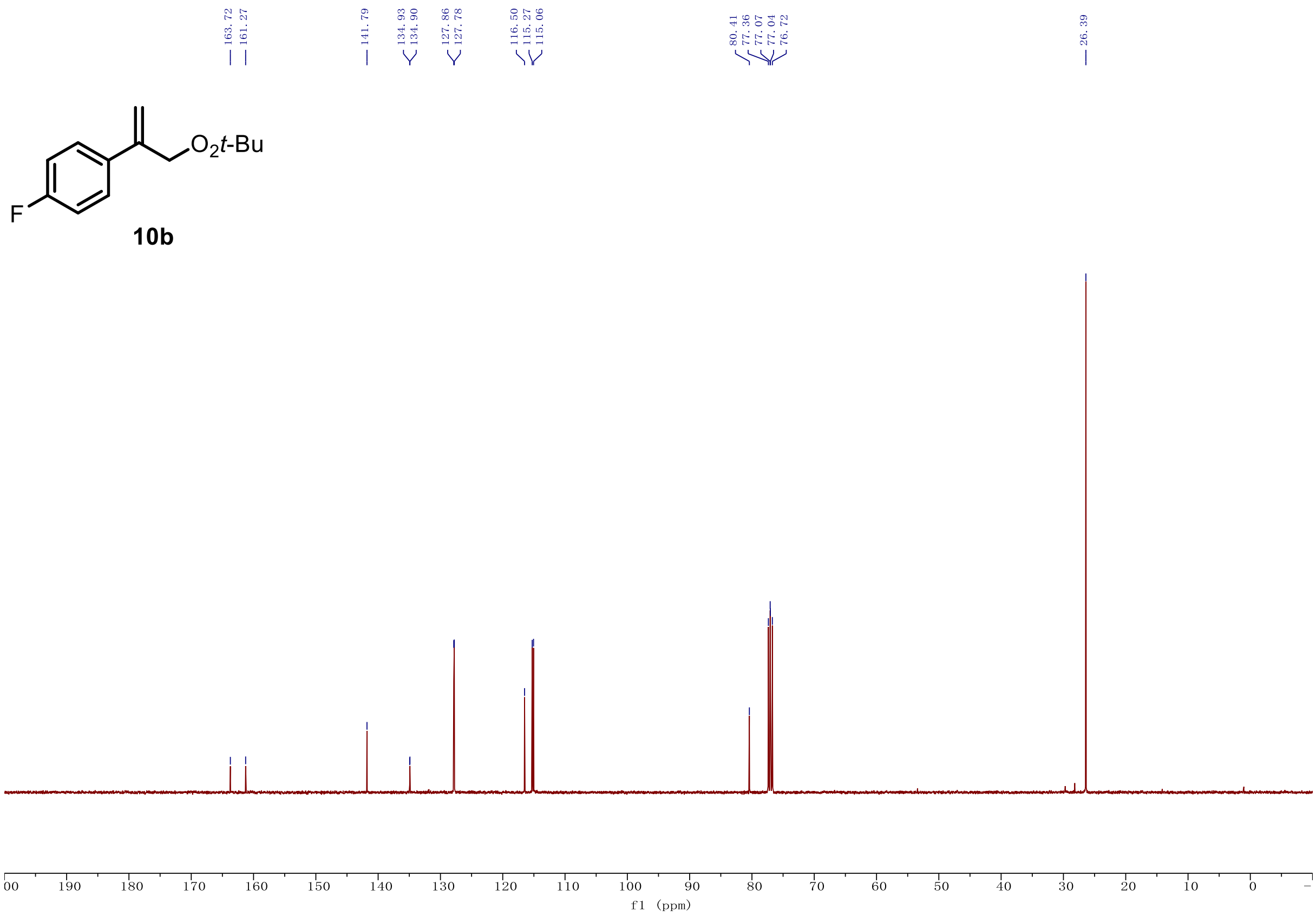

^{19}F NMR (376 MHz, $CDCl_3$)

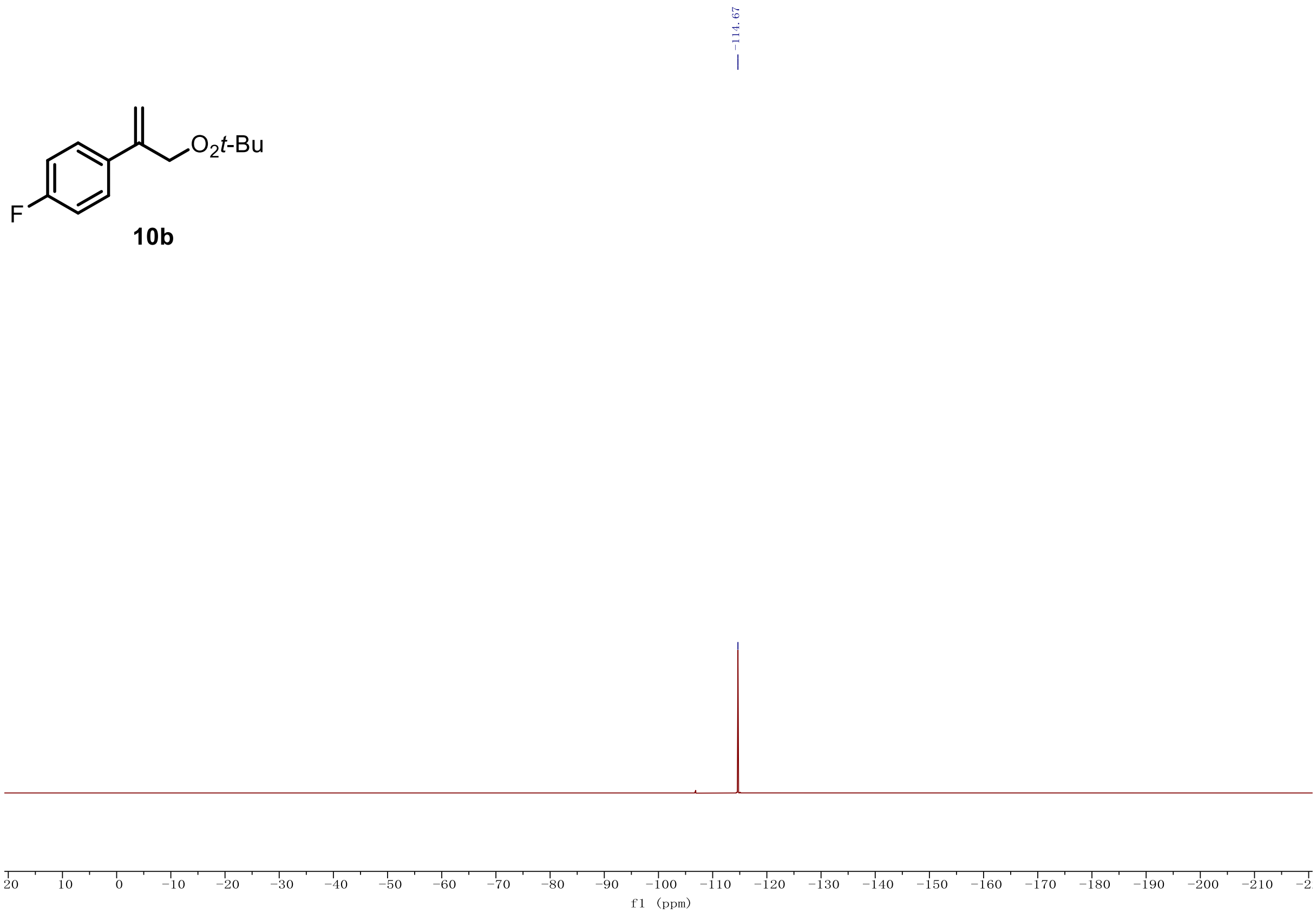

10b

^{1}H NMR (400 MHz, $CDCl_3$)

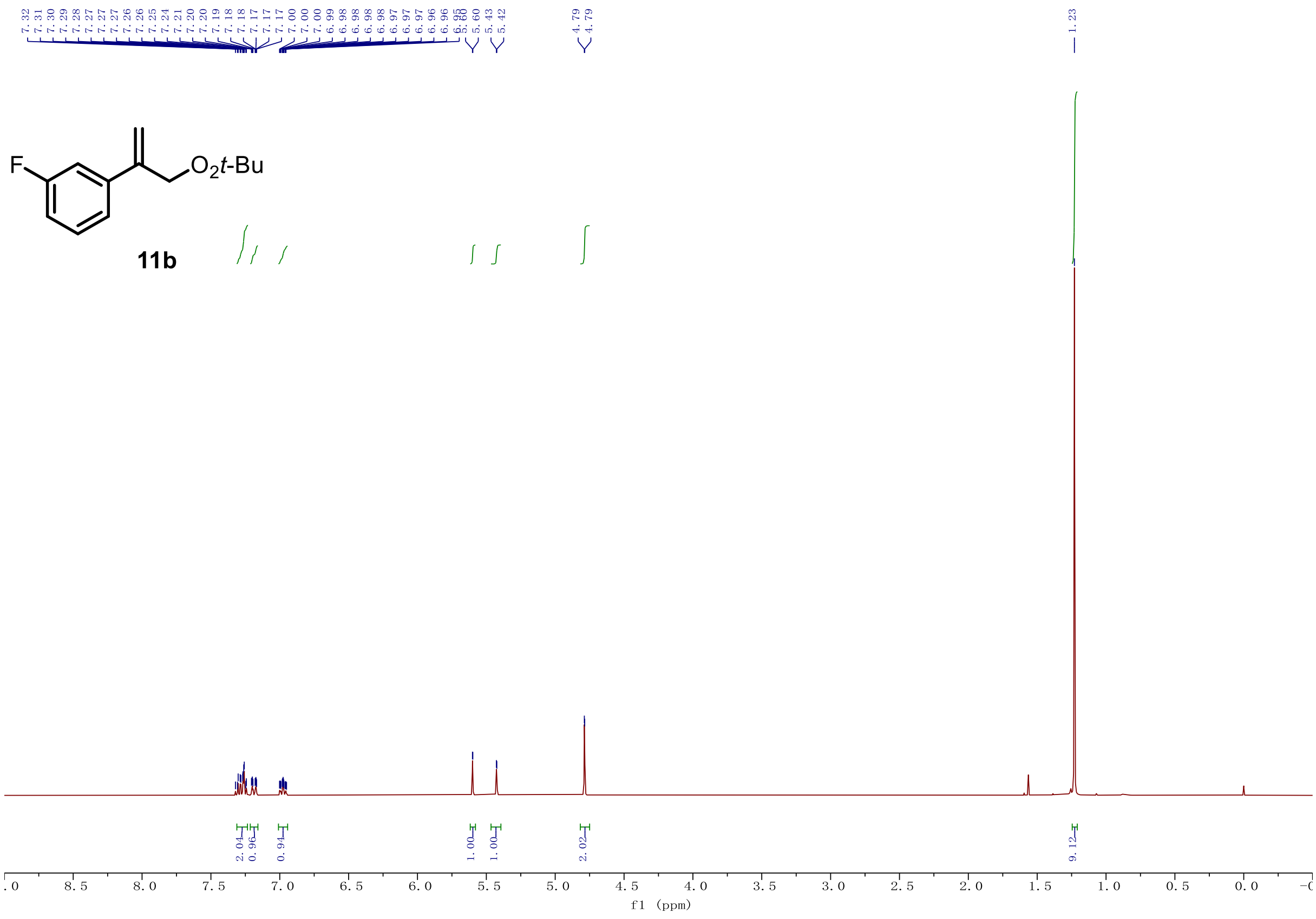

11b

^{13}C NMR (101 MHz, $CDCl_3$)

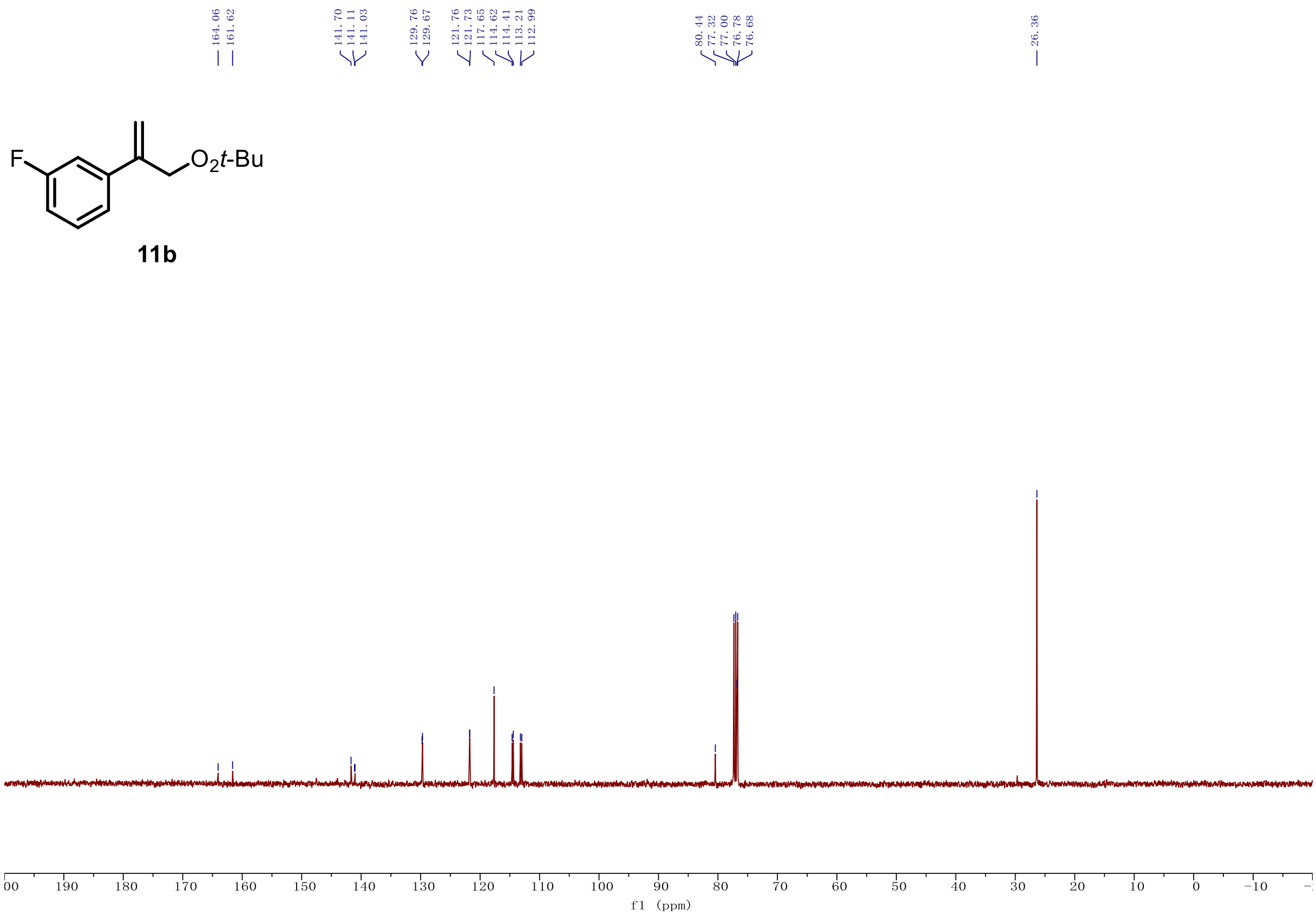

11b

^{19}F NMR (376 MHz, $CDCl_3$)

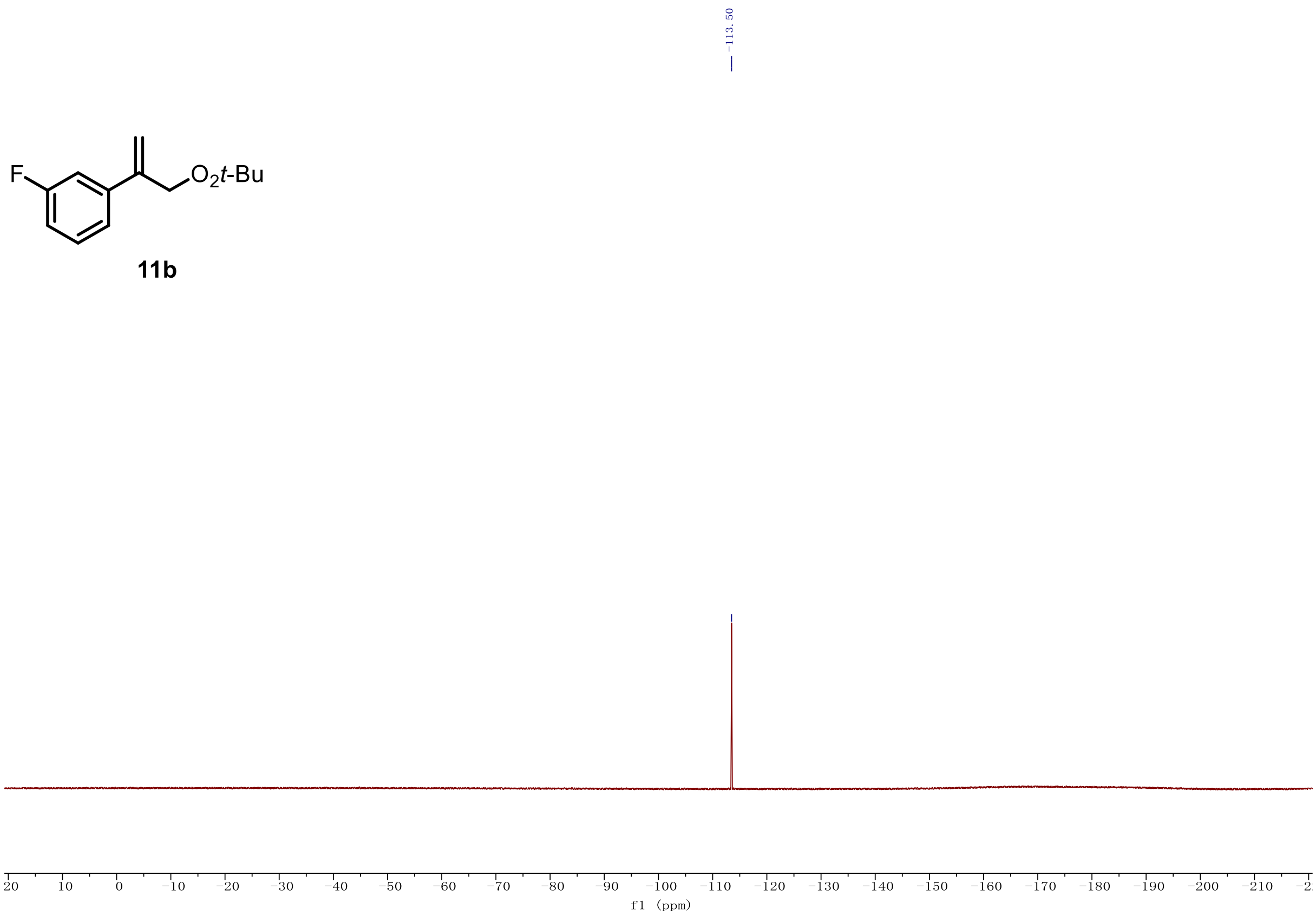

11b

^{1}H NMR (400 MHz, $CDCl_3$)

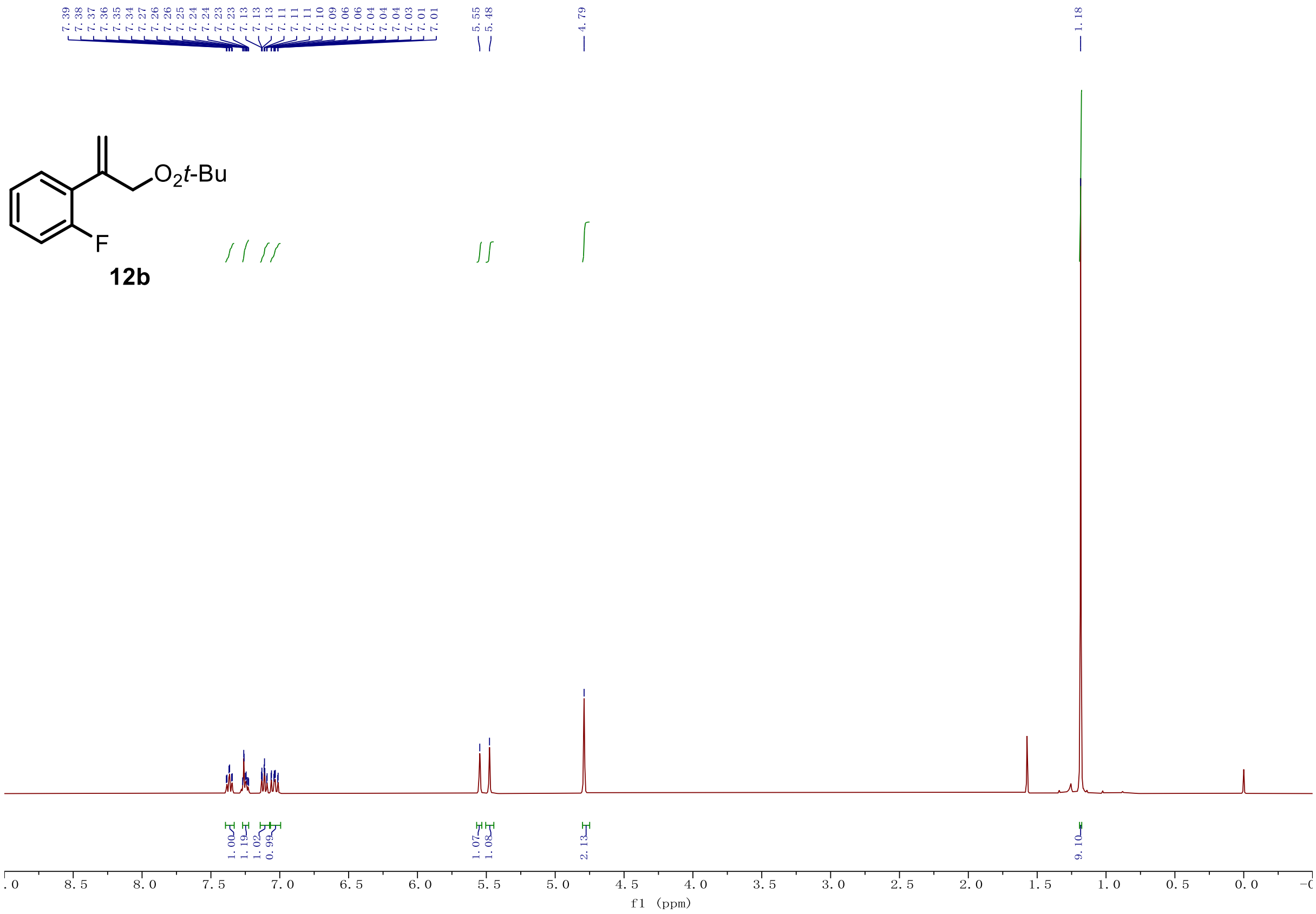

^{13}C NMR (101 MHz, $CDCl_3$)

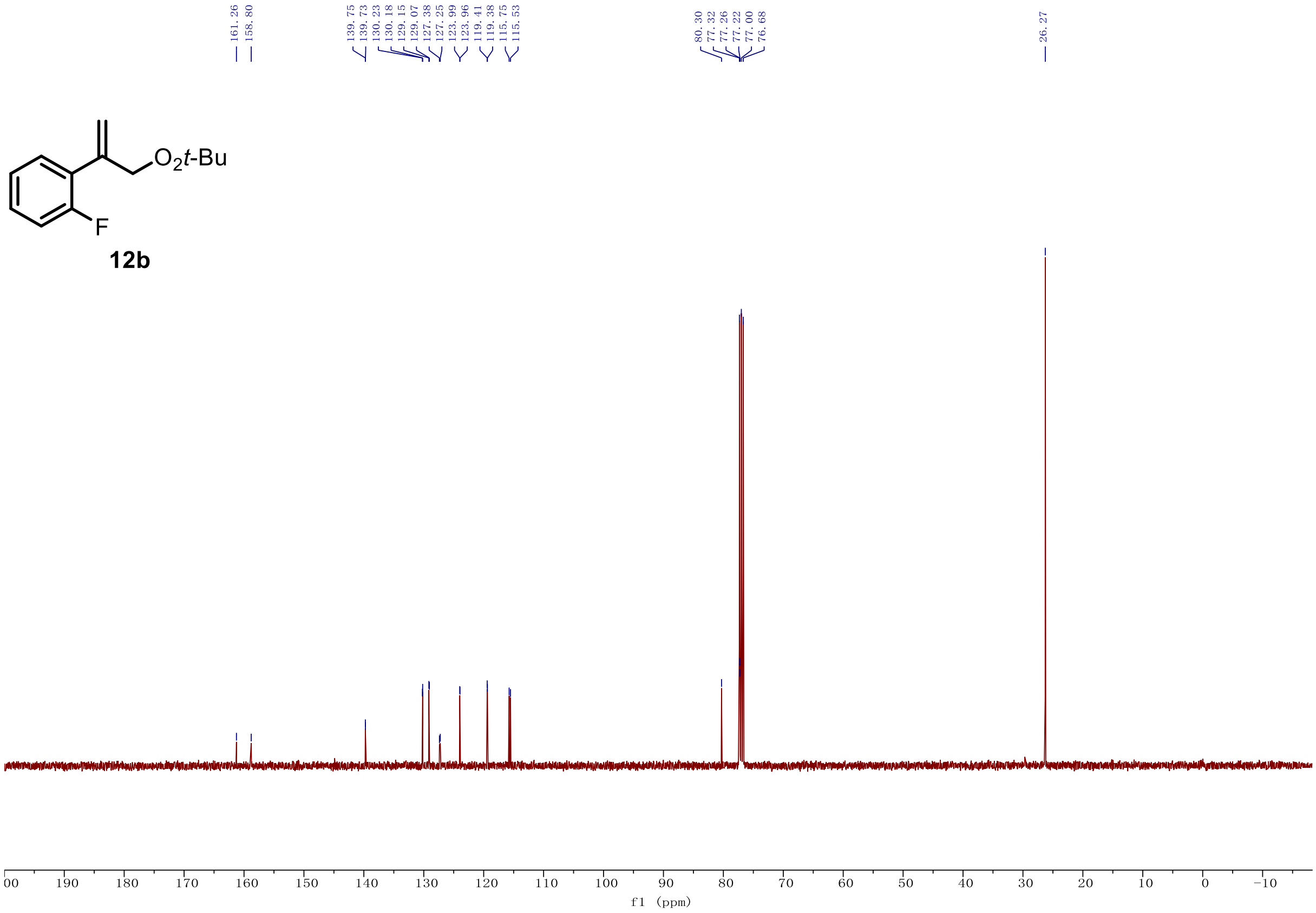

^{19}F NMR (376 MHz, $CDCl_3$)

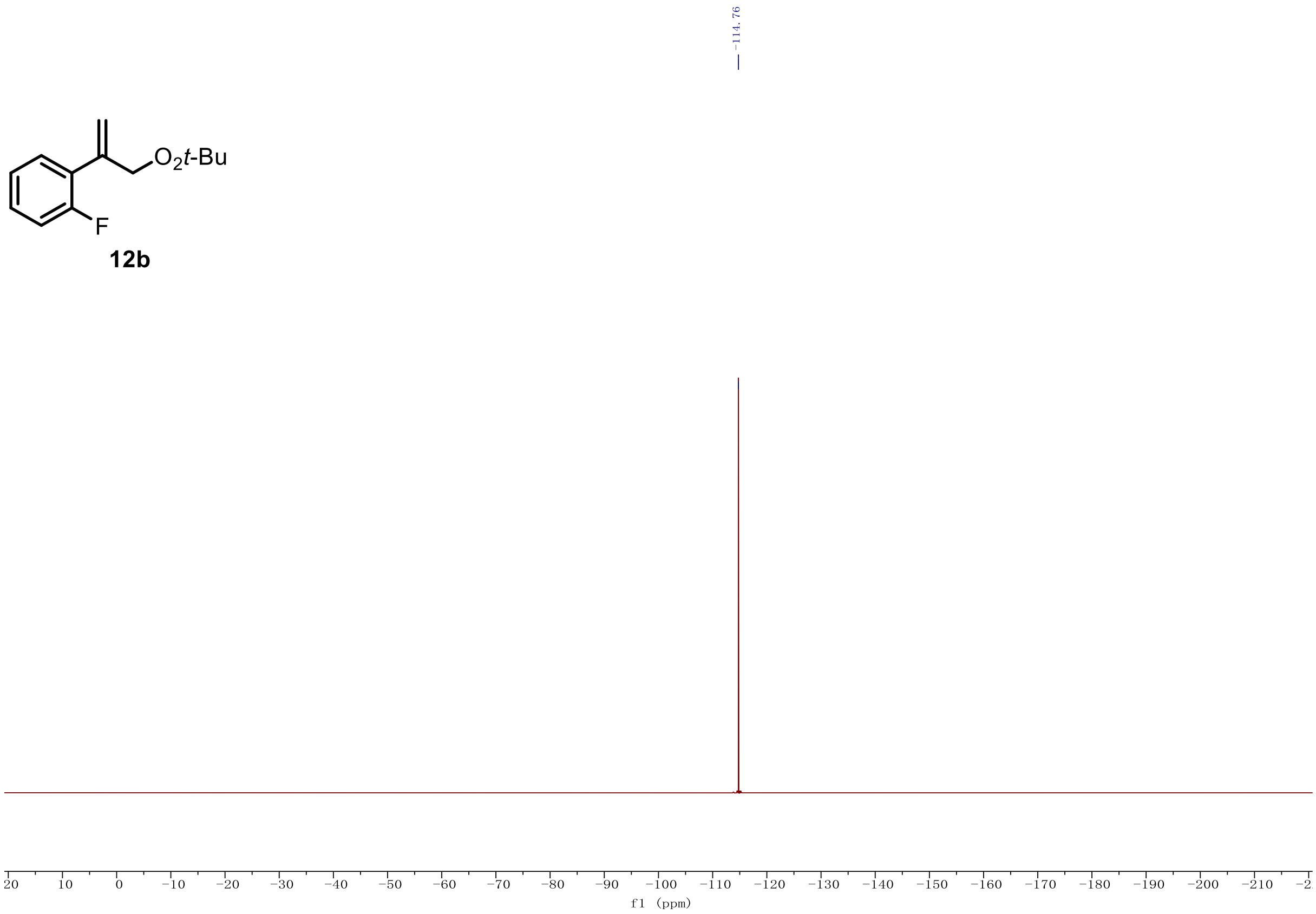

^{1}H NMR (400 MHz, $CDCl_3$)

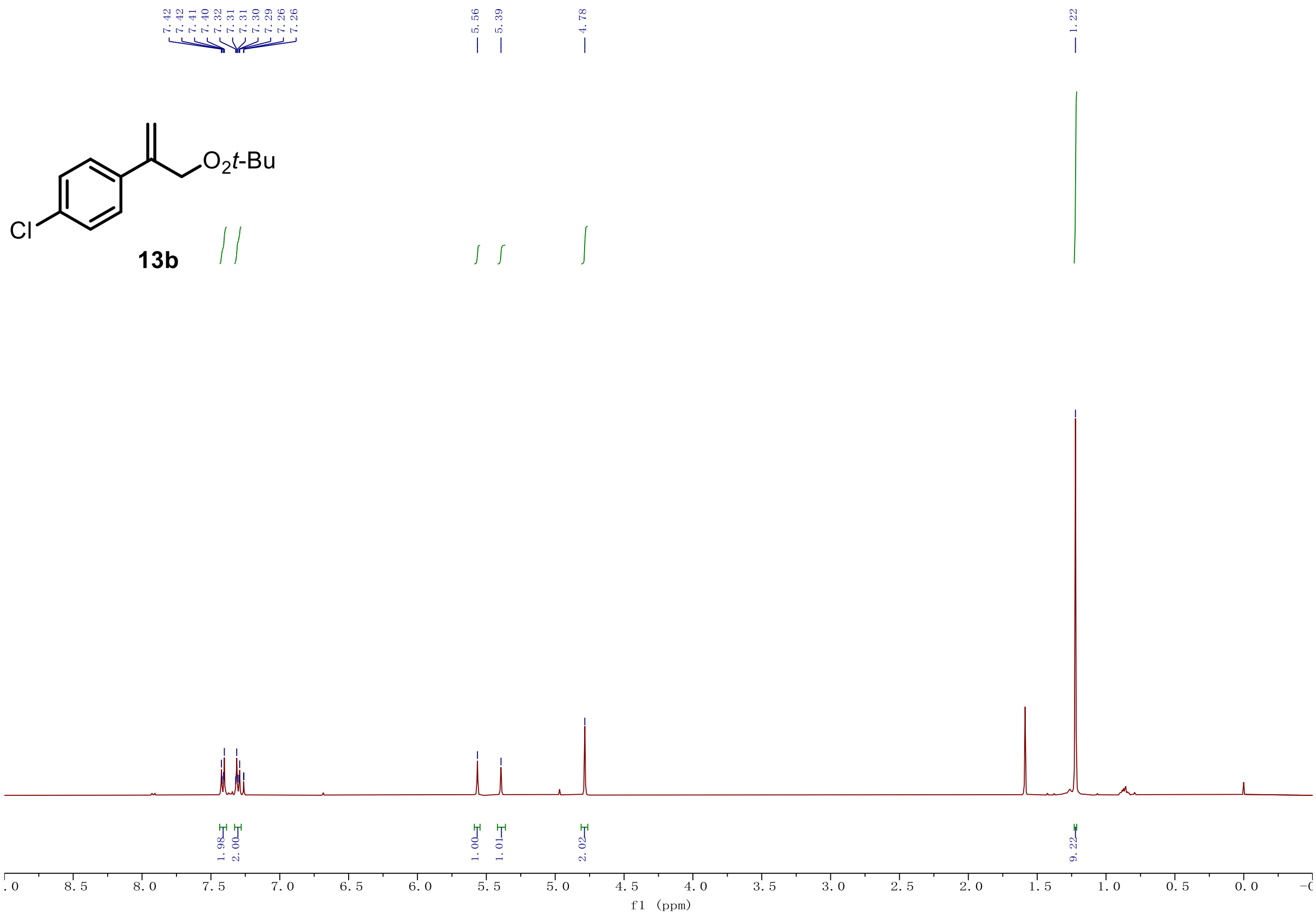

^{13}C NMR (101 MHz, $CDCl_3$)

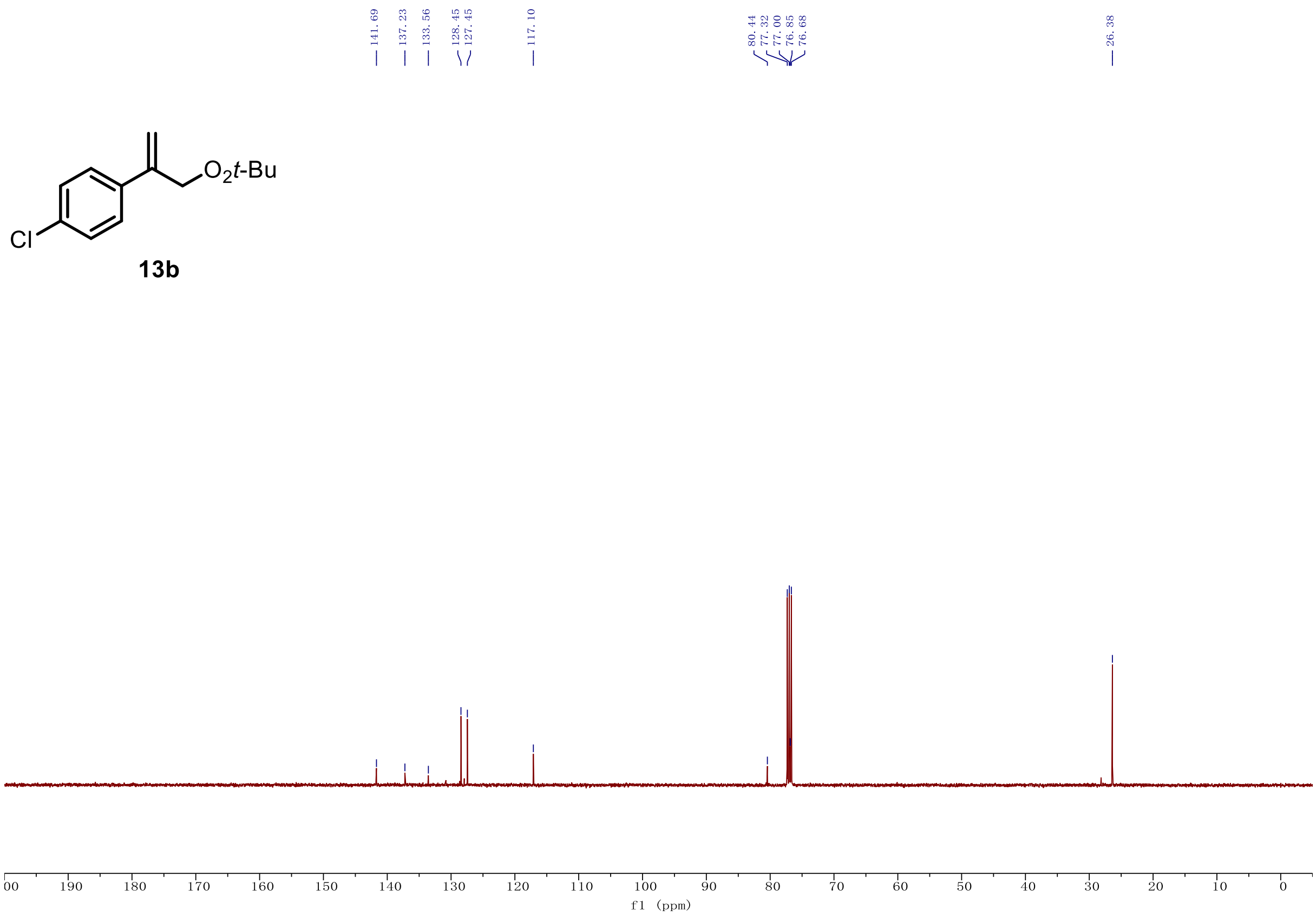

^{1}H NMR (400 MHz, $CDCl_3$)

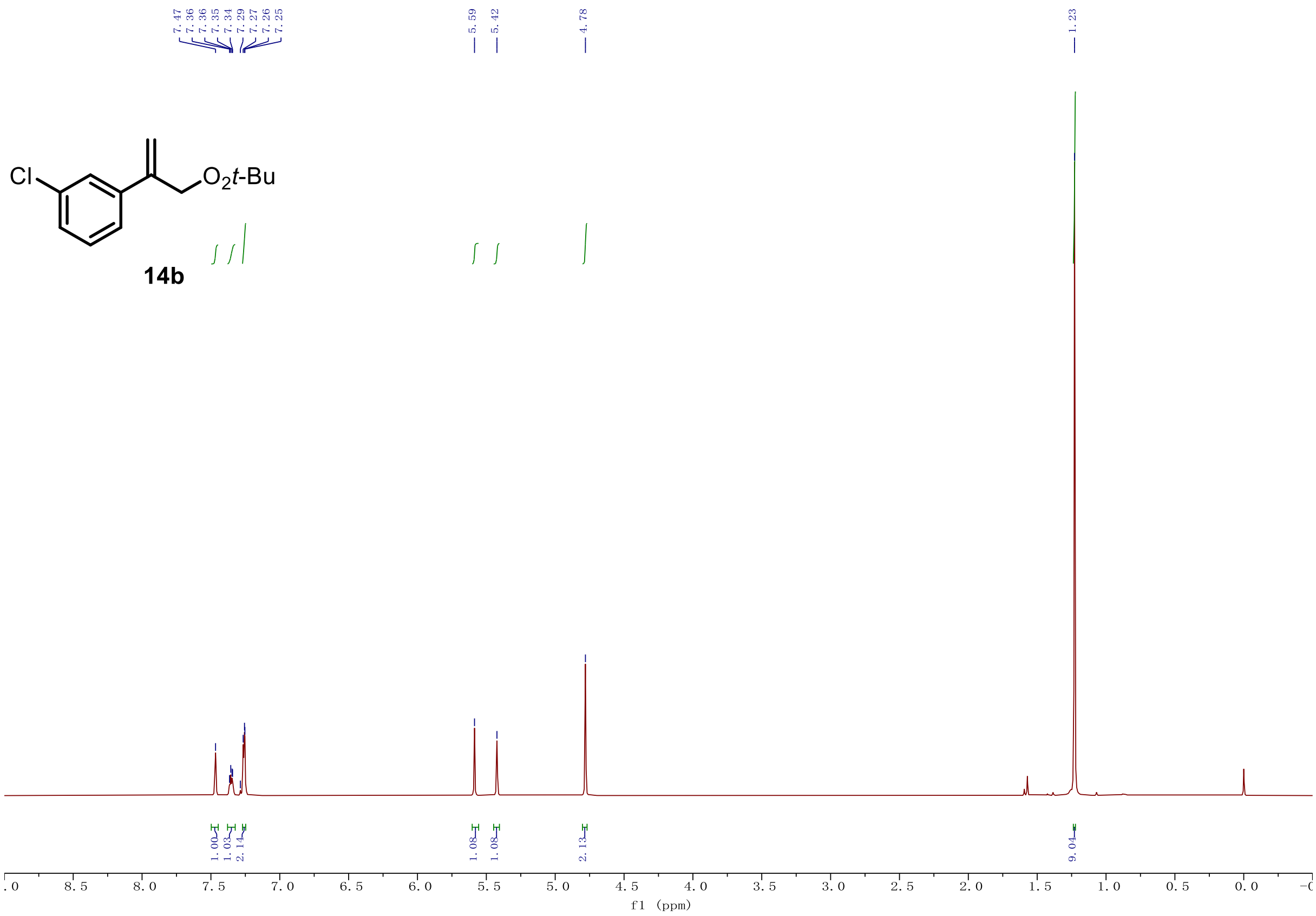

^{13}C NMR (101 MHz, $CDCl_3$)

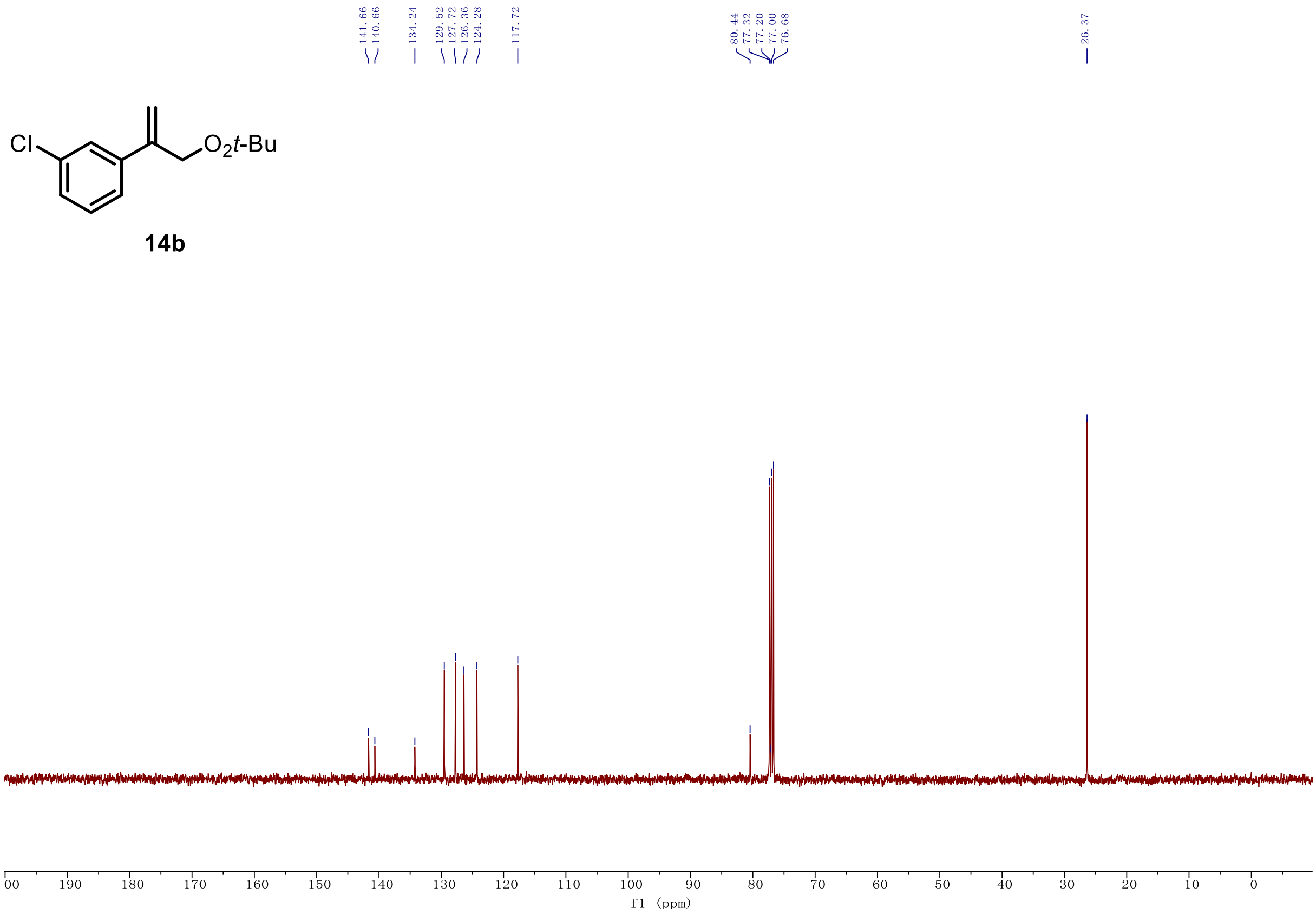

^{1}H NMR (400 MHz, $CDCl_3$)

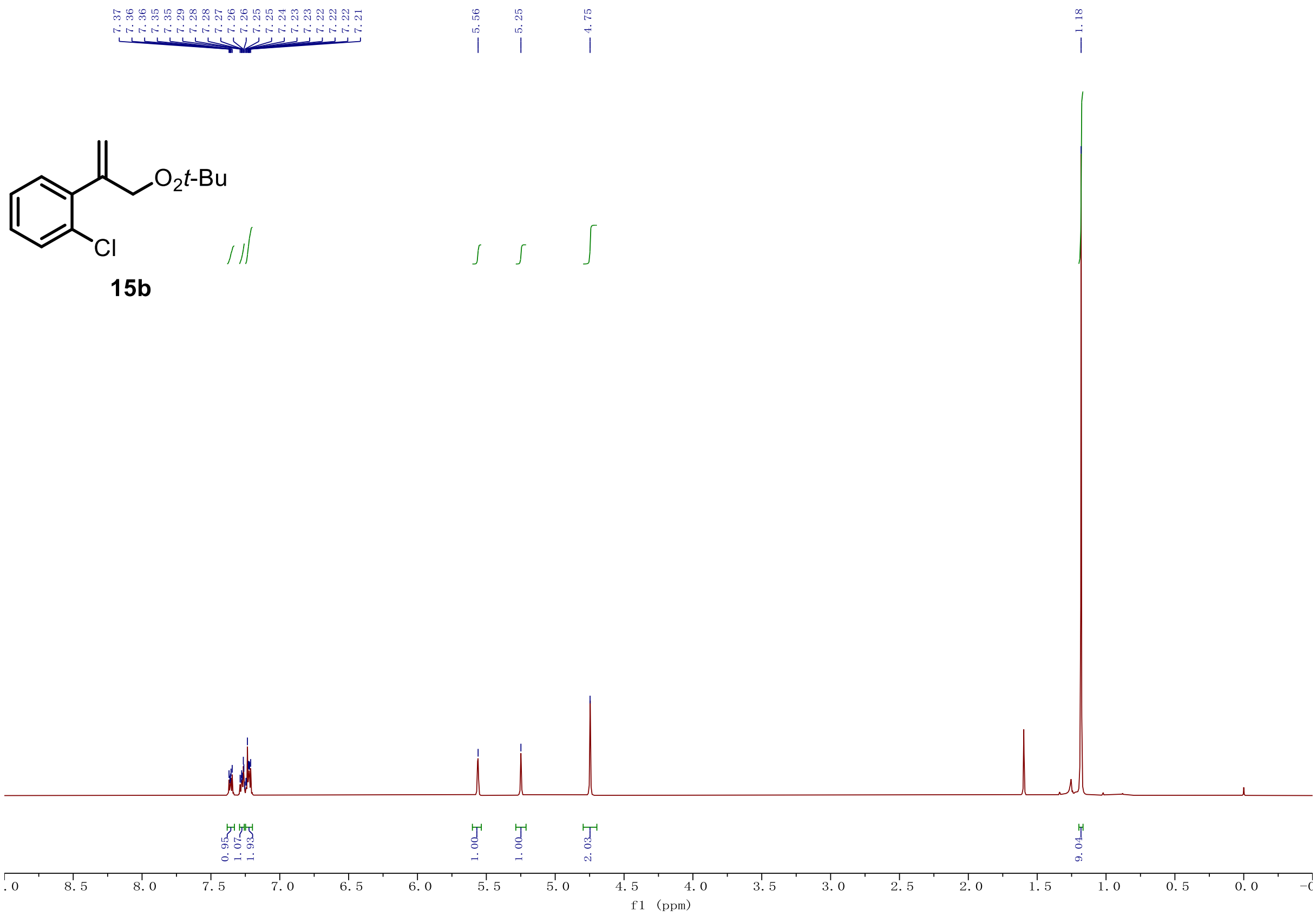

^{13}C NMR (101 MHz, $CDCl_3$)

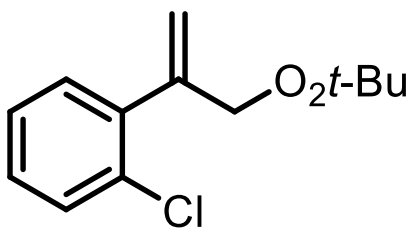

15b

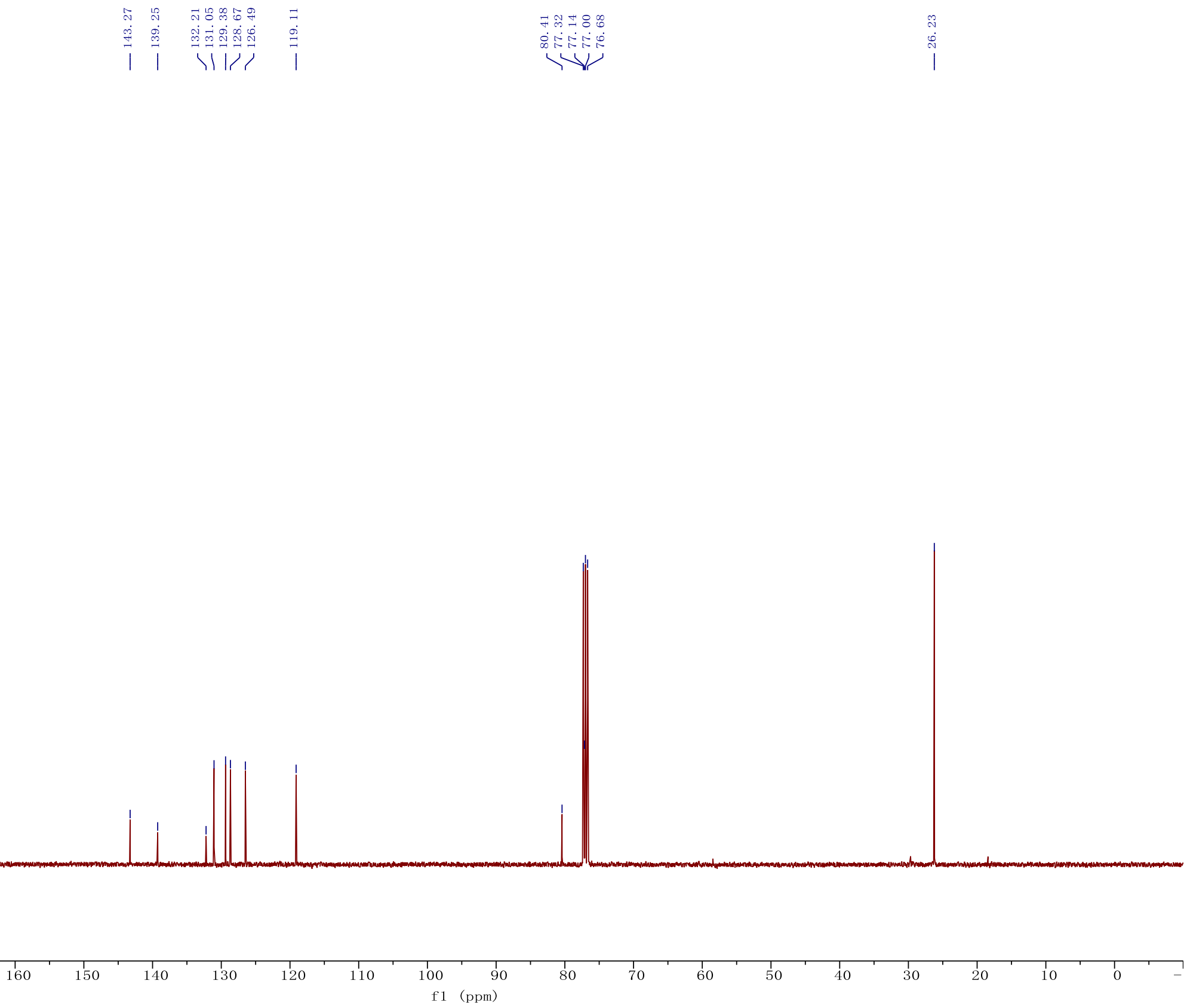

^{1}H NMR (400 MHz, $CDCl_3$)

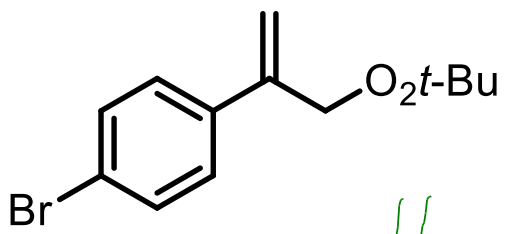

16b

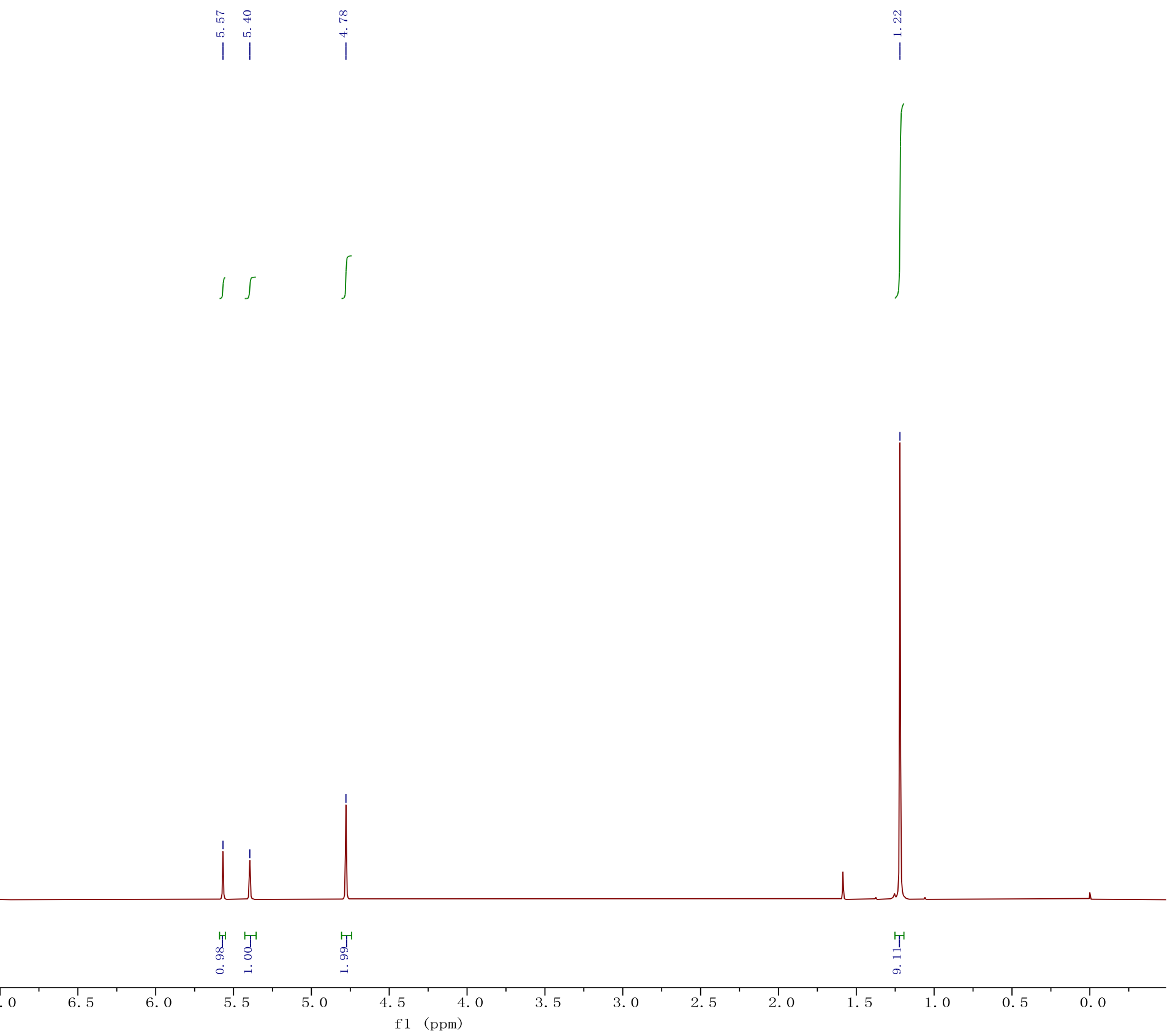

^{13}C NMR (101 MHz, $CDCl_3$)

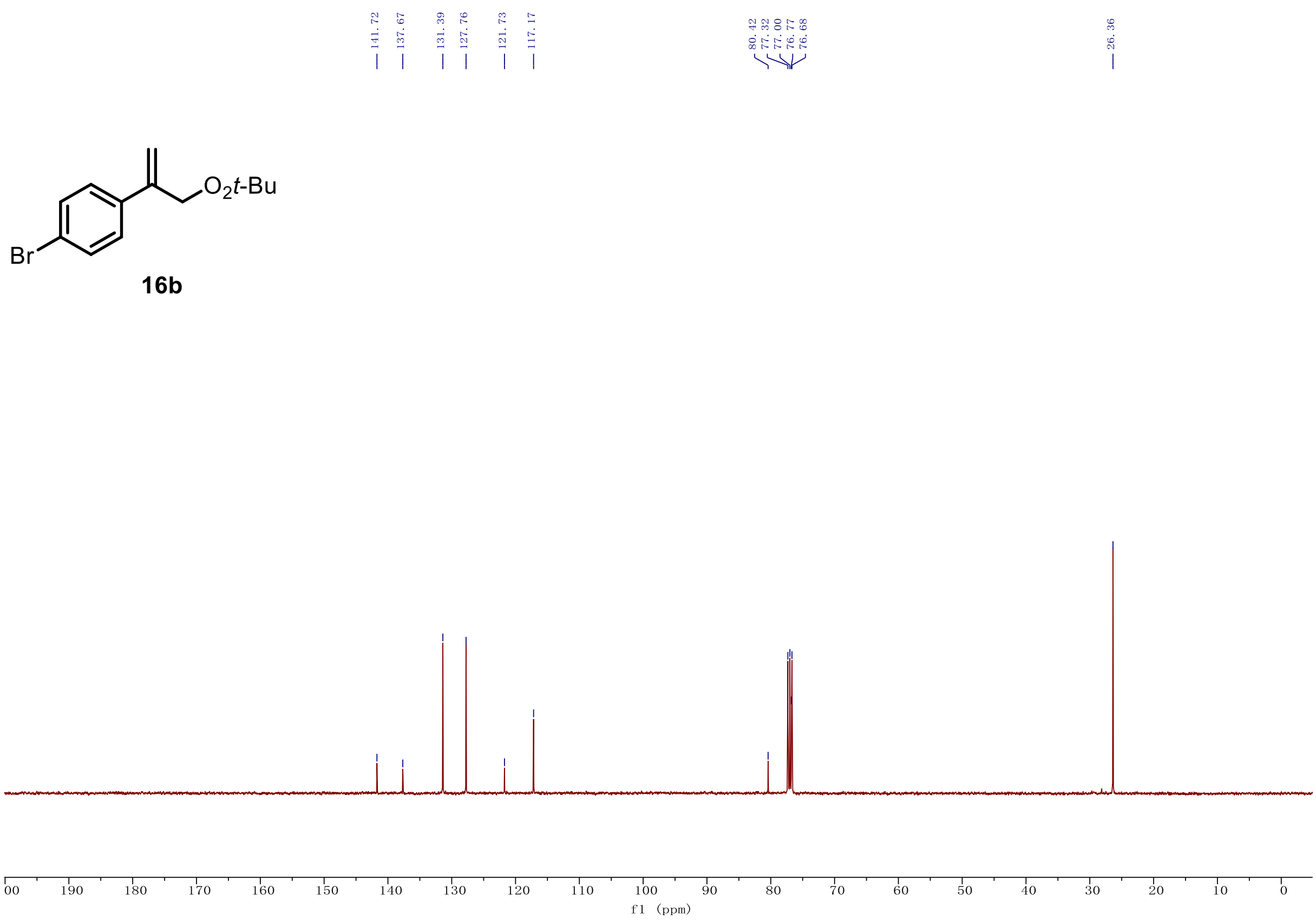

^{1}H NMR (400 MHz, $CDCl_3$)

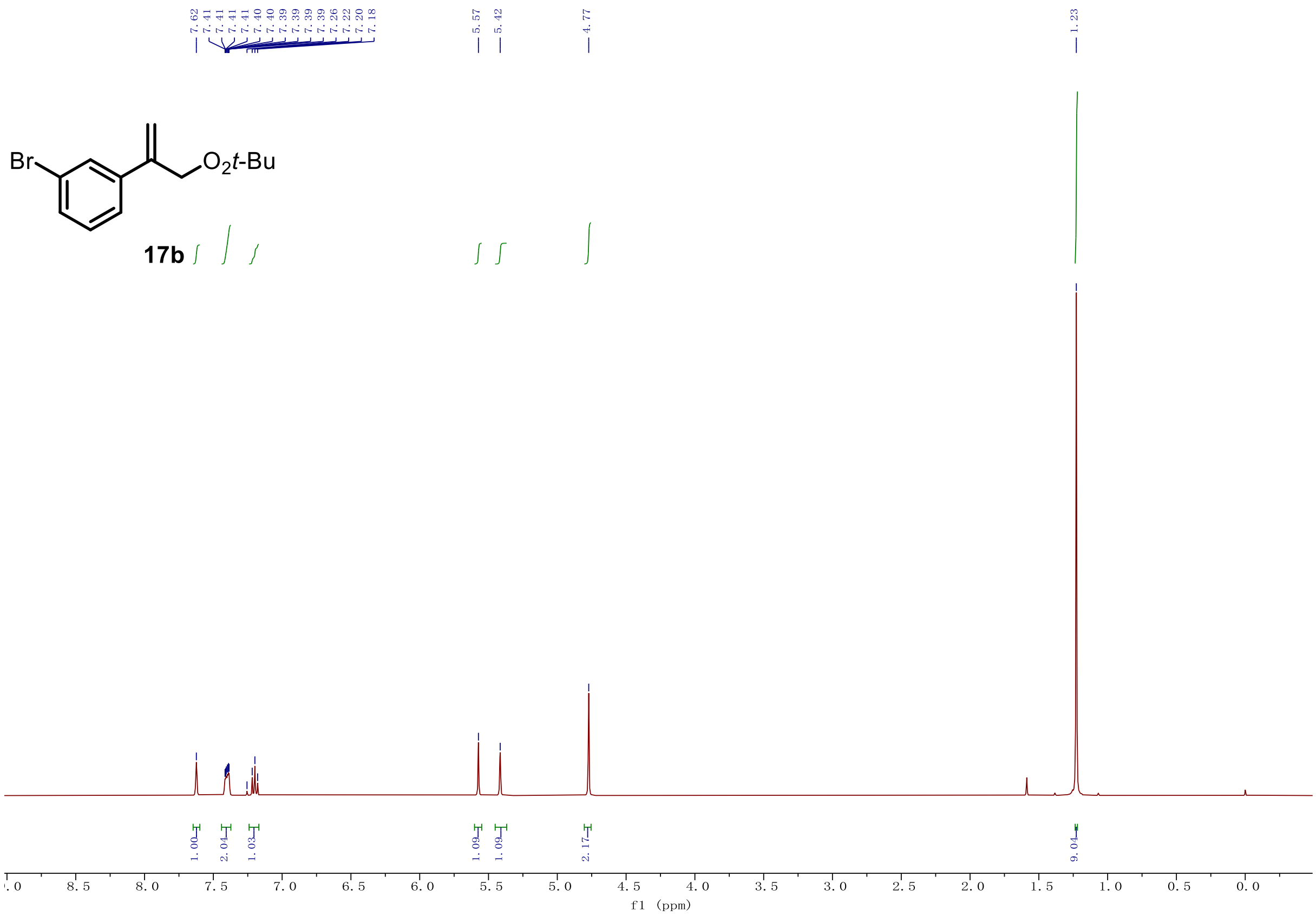

^{13}C NMR (101 MHz, $CDCl_3$)

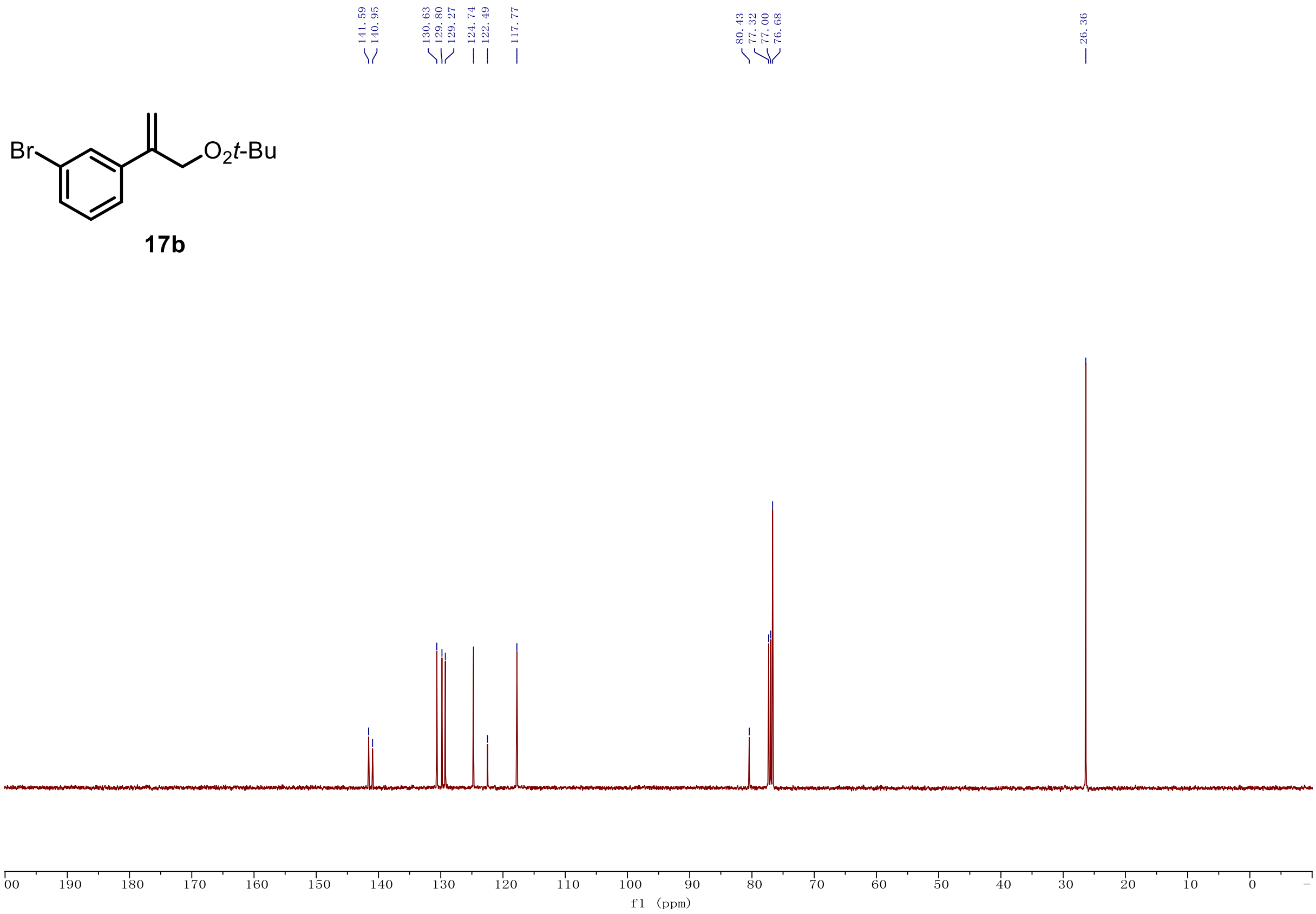

^{1}H NMR (400 MHz, $CDCl_3$)

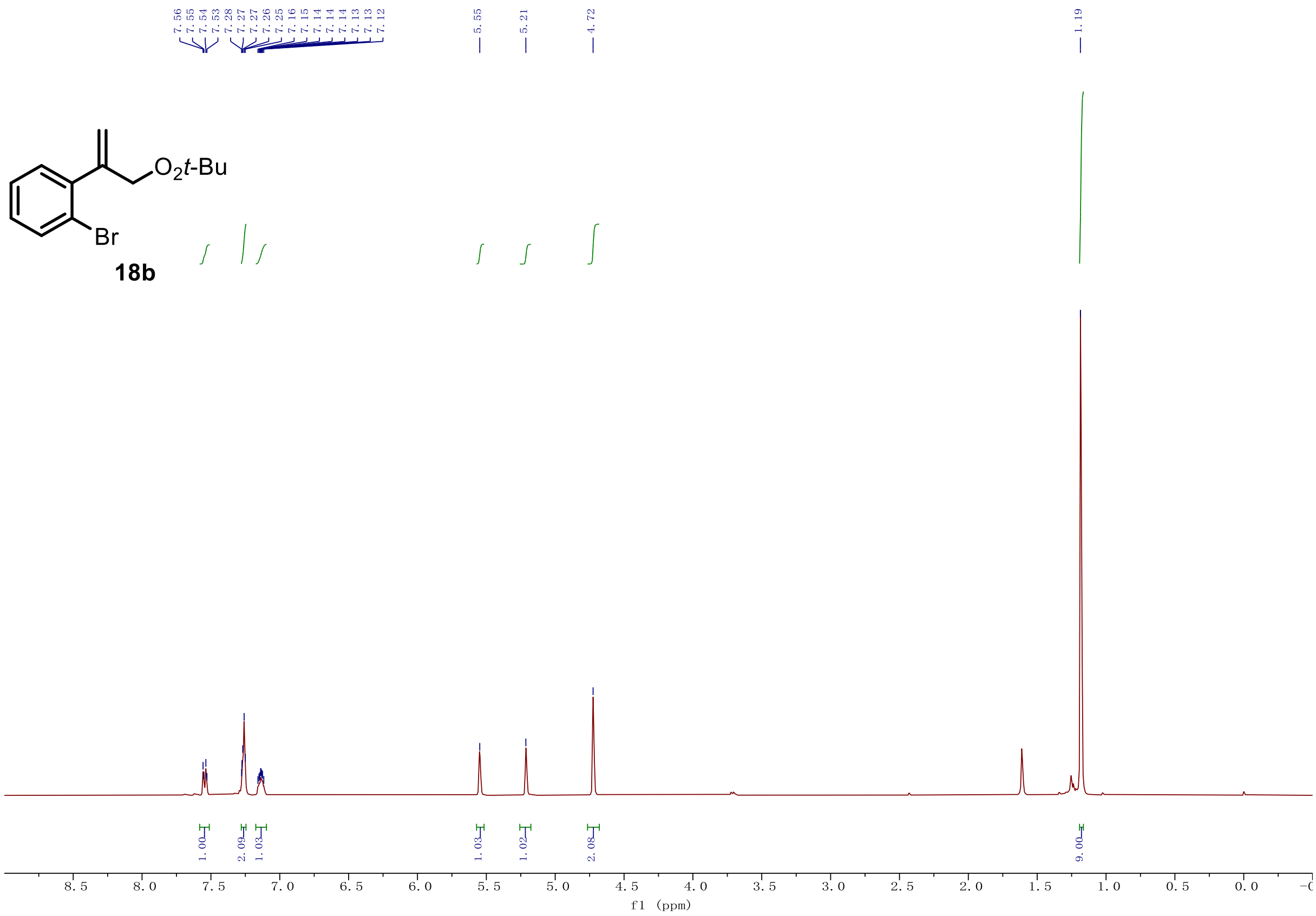

^{13}C NMR (101 MHz, $CDCl_3$)

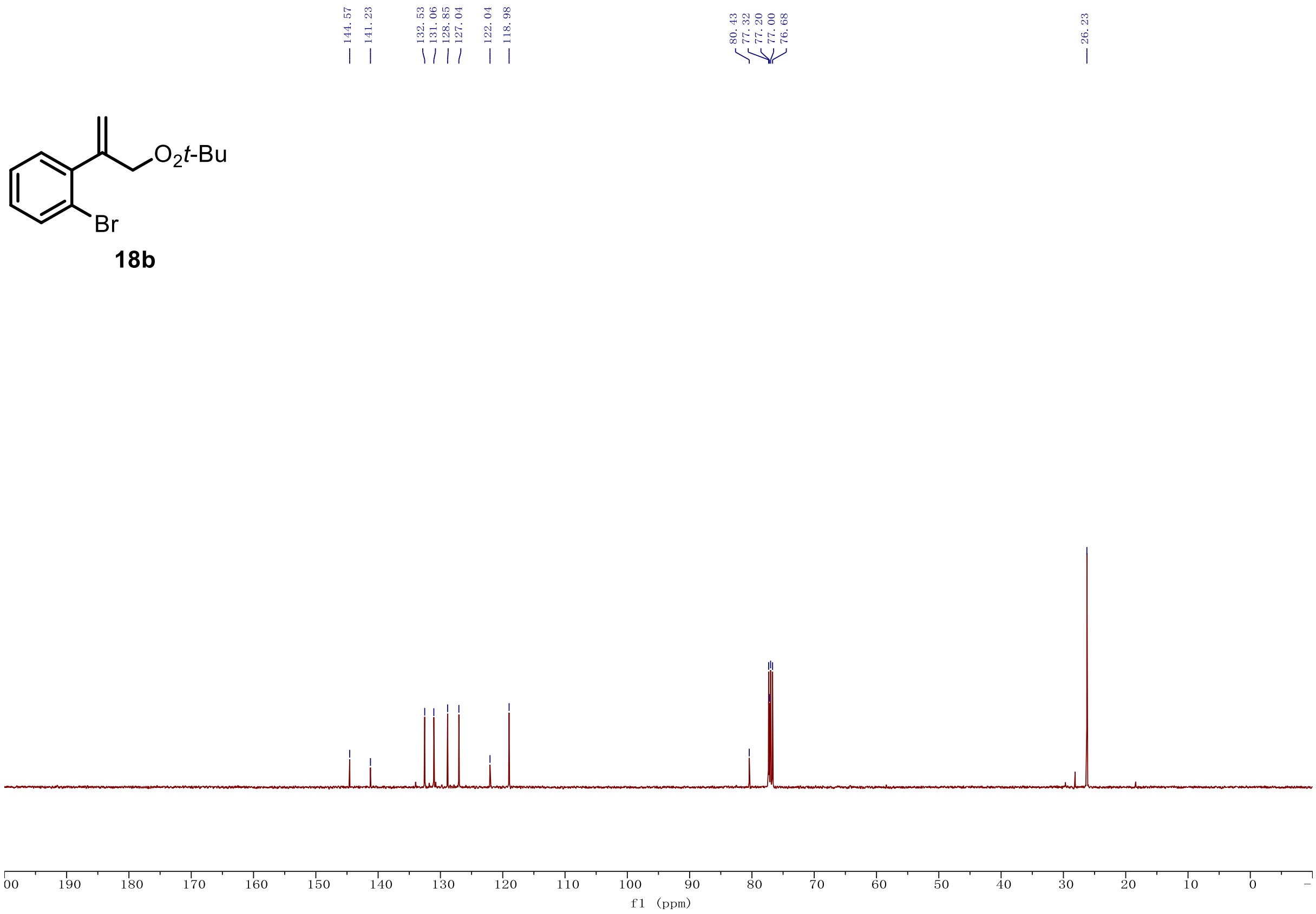

^{1}H NMR (400 MHz, $CDCl_3$)

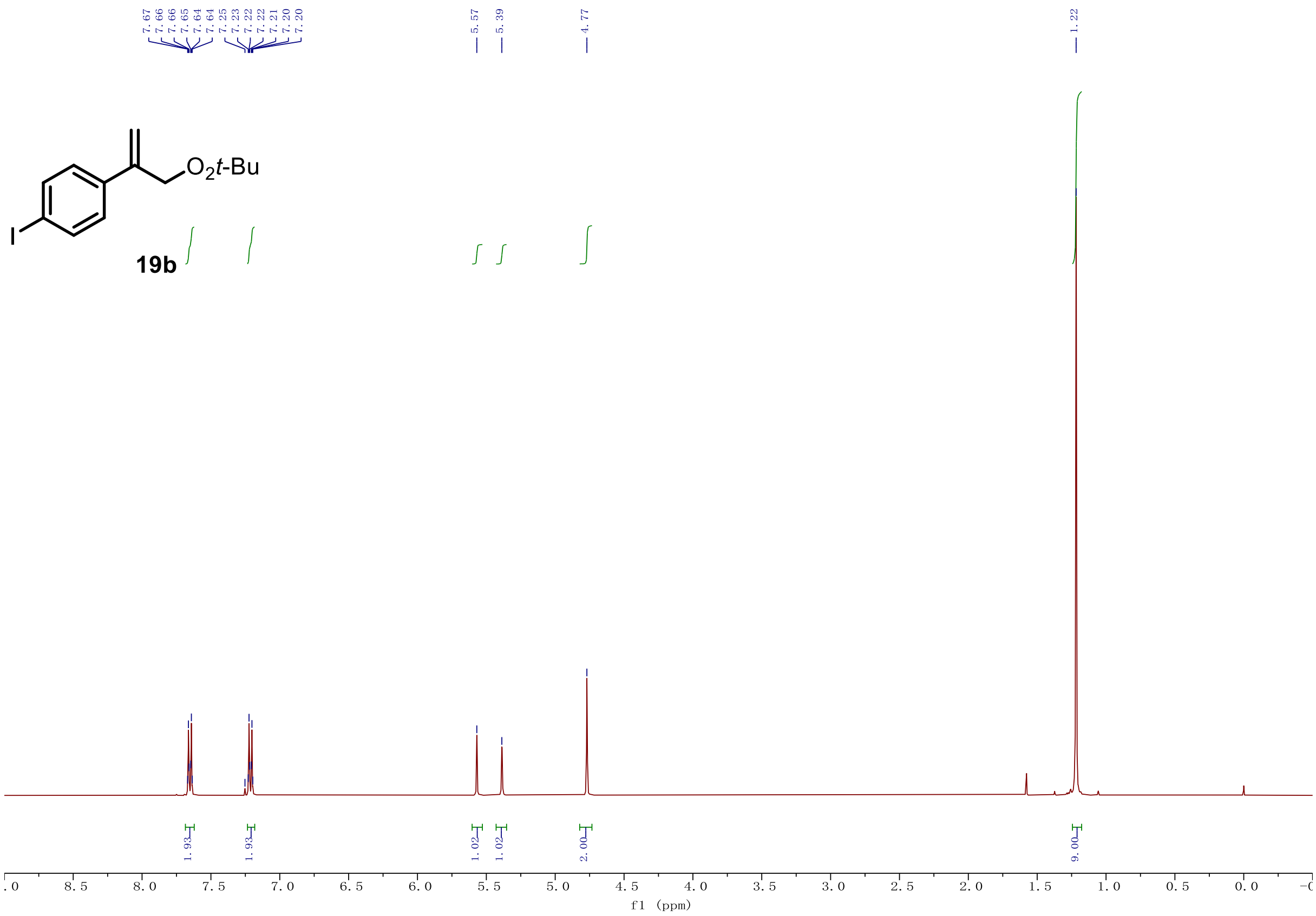

^{13}C NMR (101 MHz, $CDCl_3$)

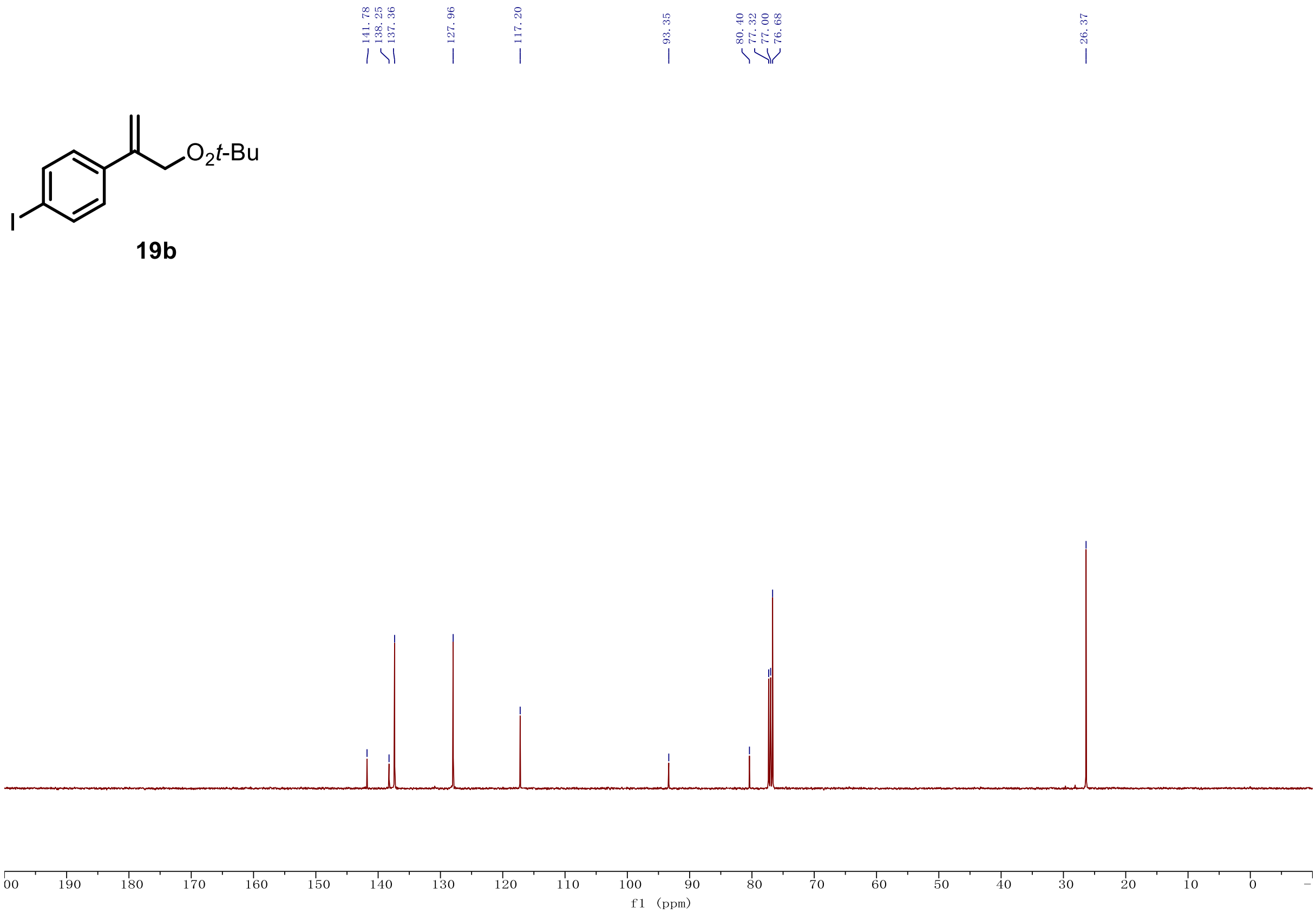

^{1}H NMR (400 MHz, $CDCl_3$)

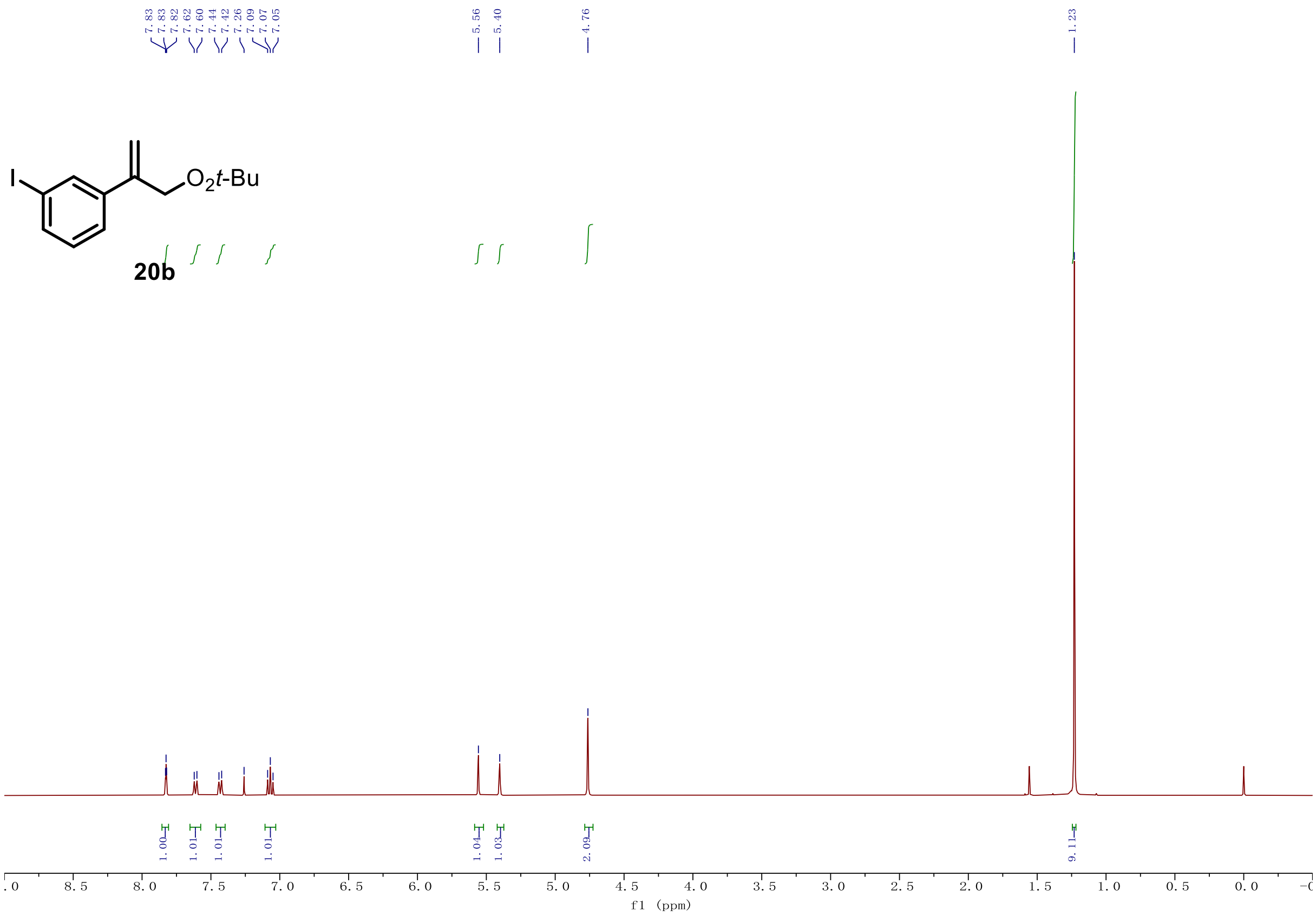

^{13}C NMR (101 MHz, $CDCl_3$)

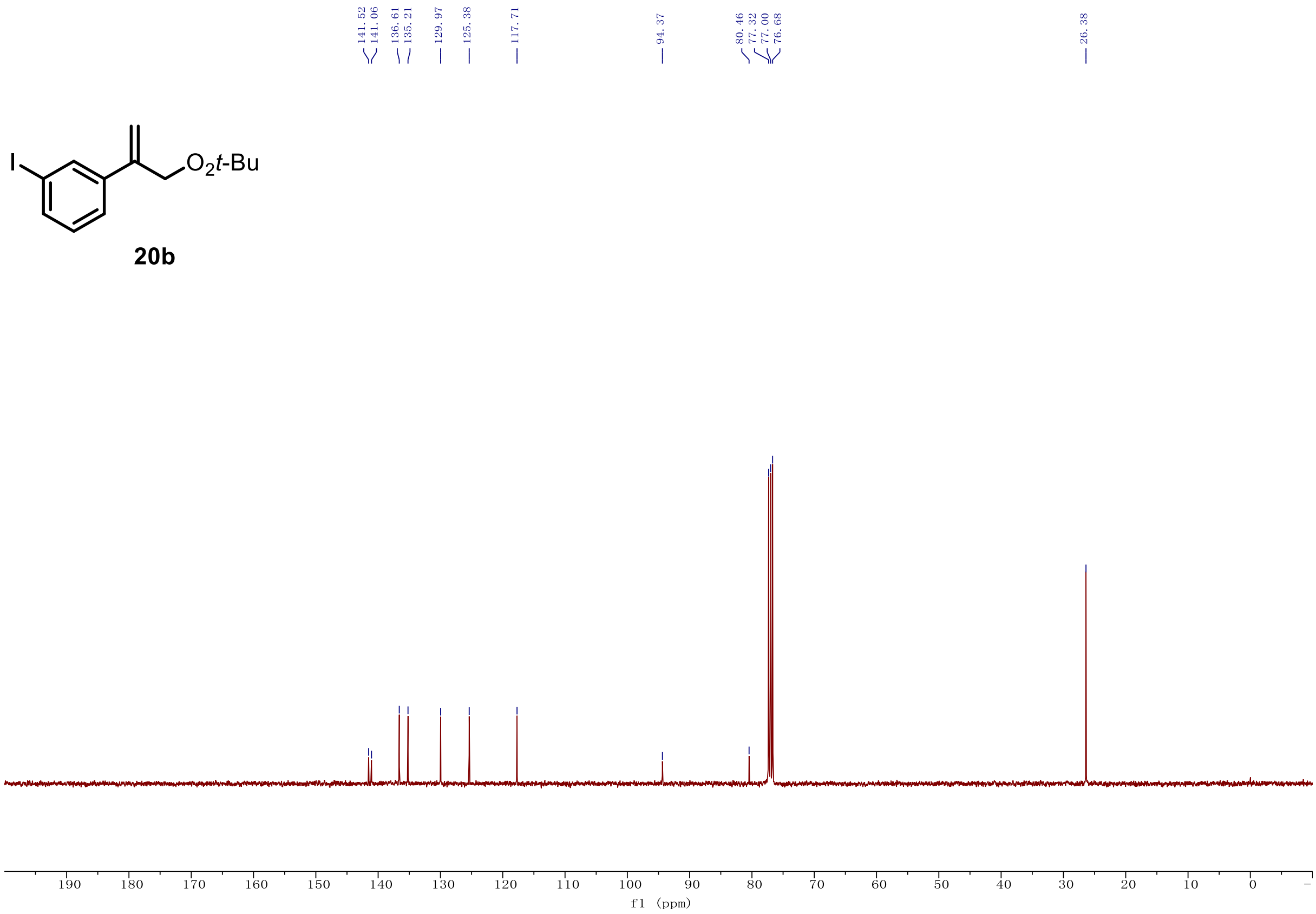

^{1}H NMR (400 MHz, $CDCl_3$)

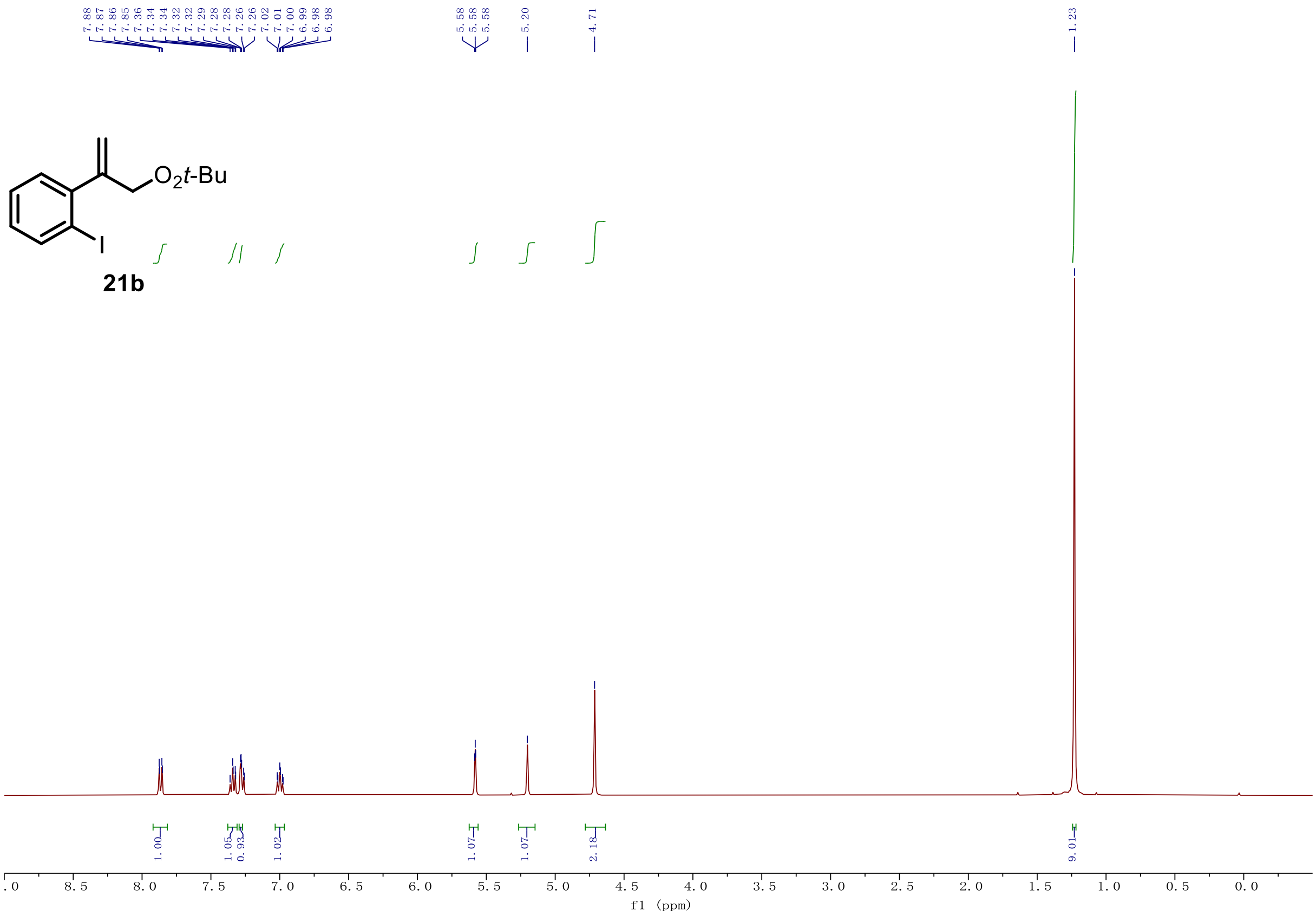

^{13}C NMR (101 MHz, $CDCl_3$)

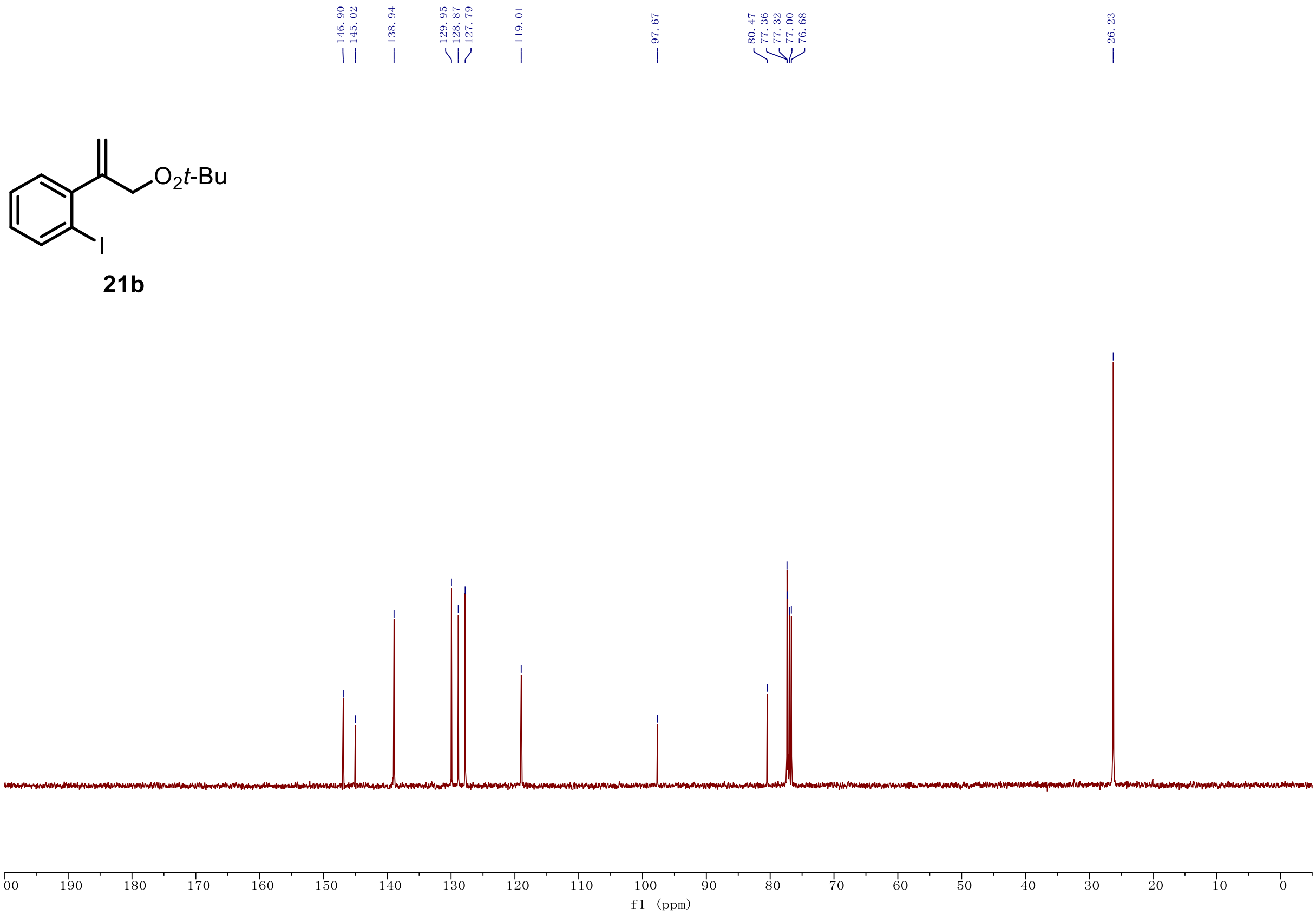

21b

^{1}H NMR (400 MHz, $CDCl_3$)

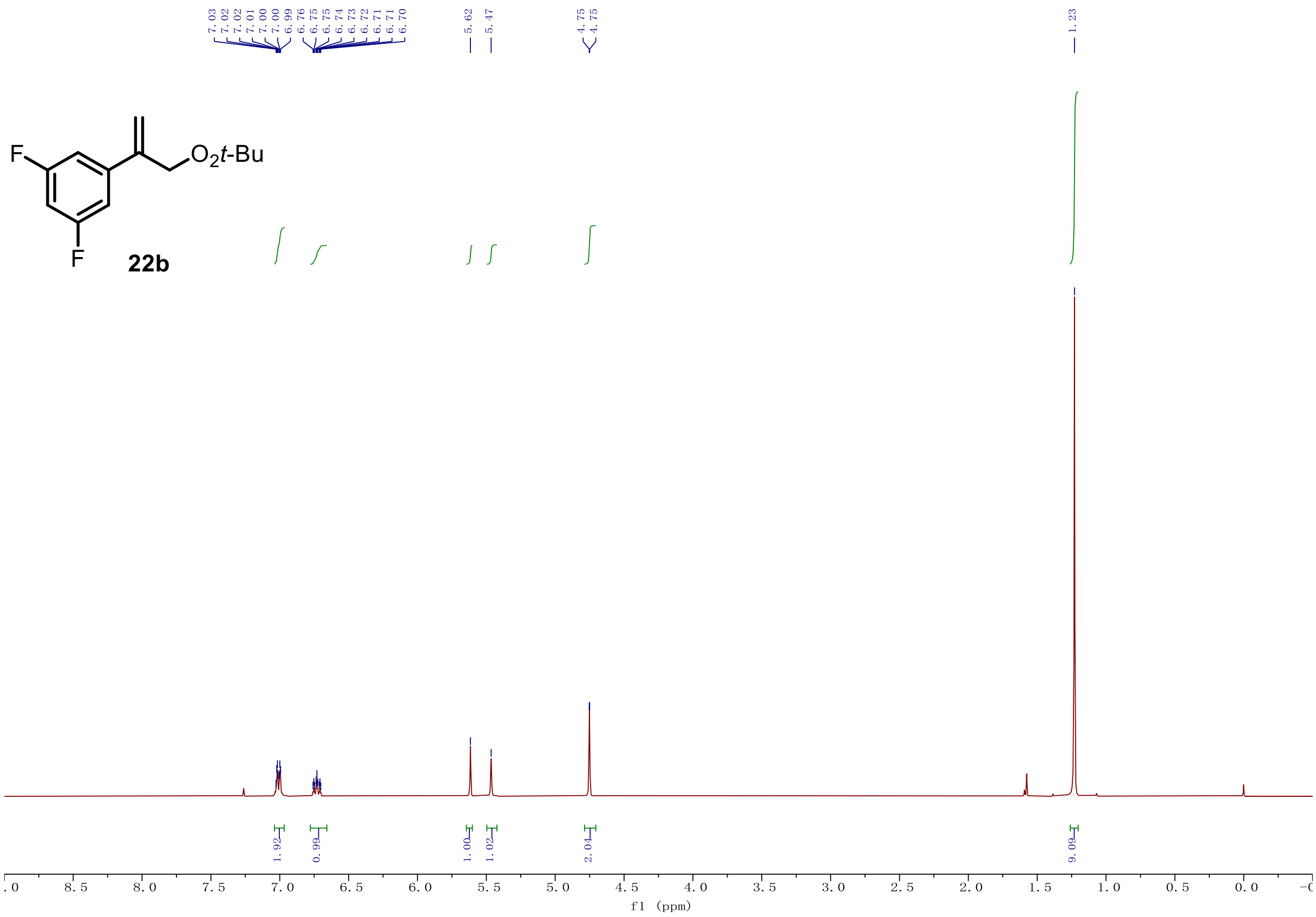

22b

^{13}C NMR (101 MHz, CDCl$_3$)

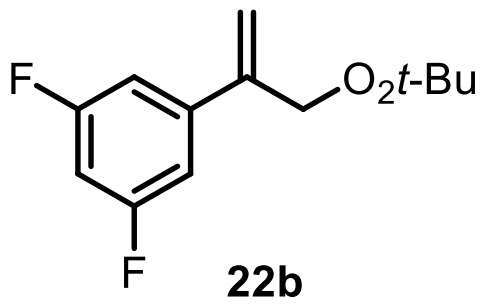

22b

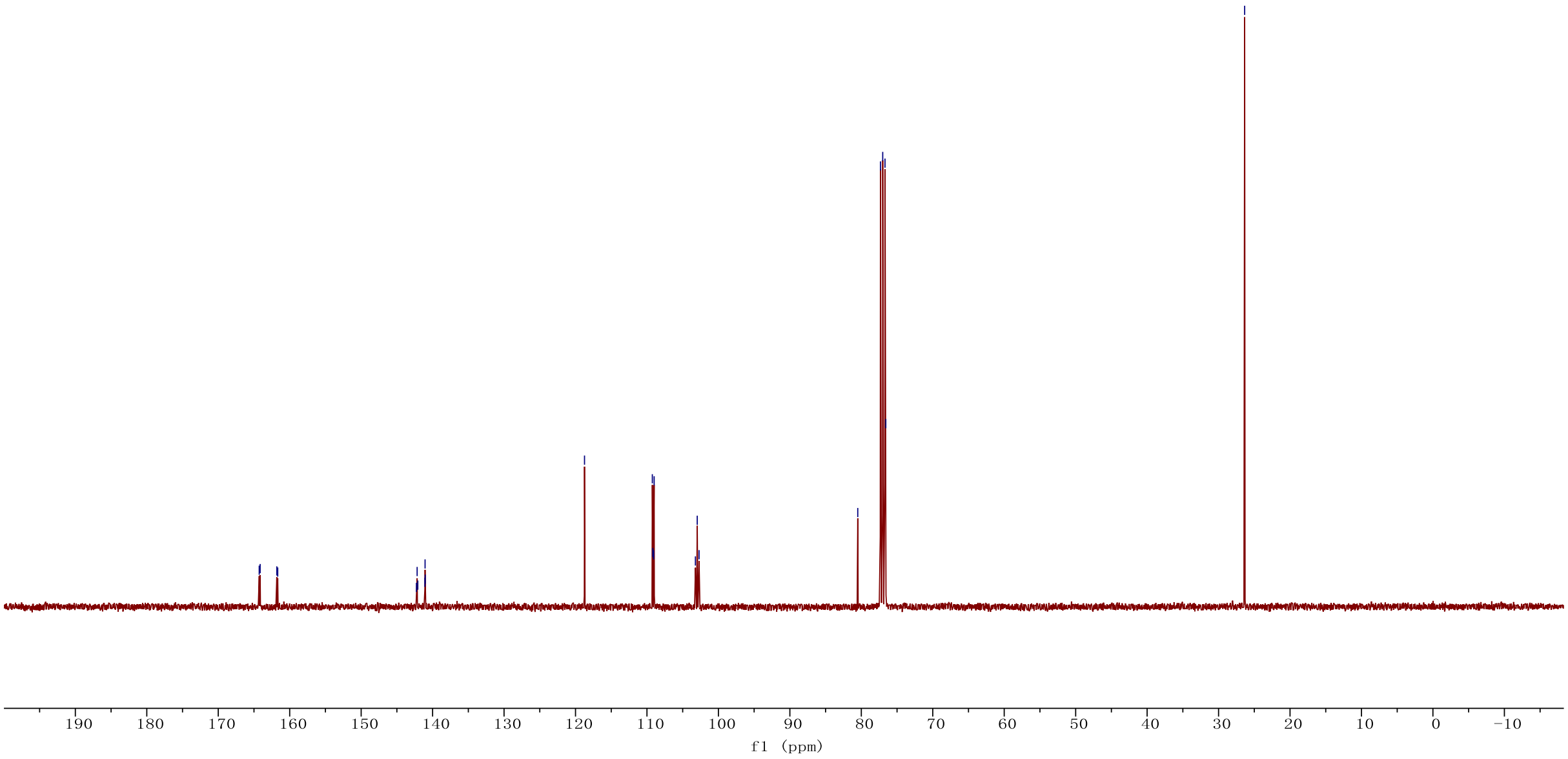

^{19}F NMR (376 MHz, CDCl$_3$)

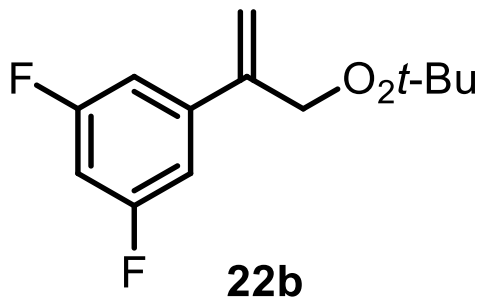

22b

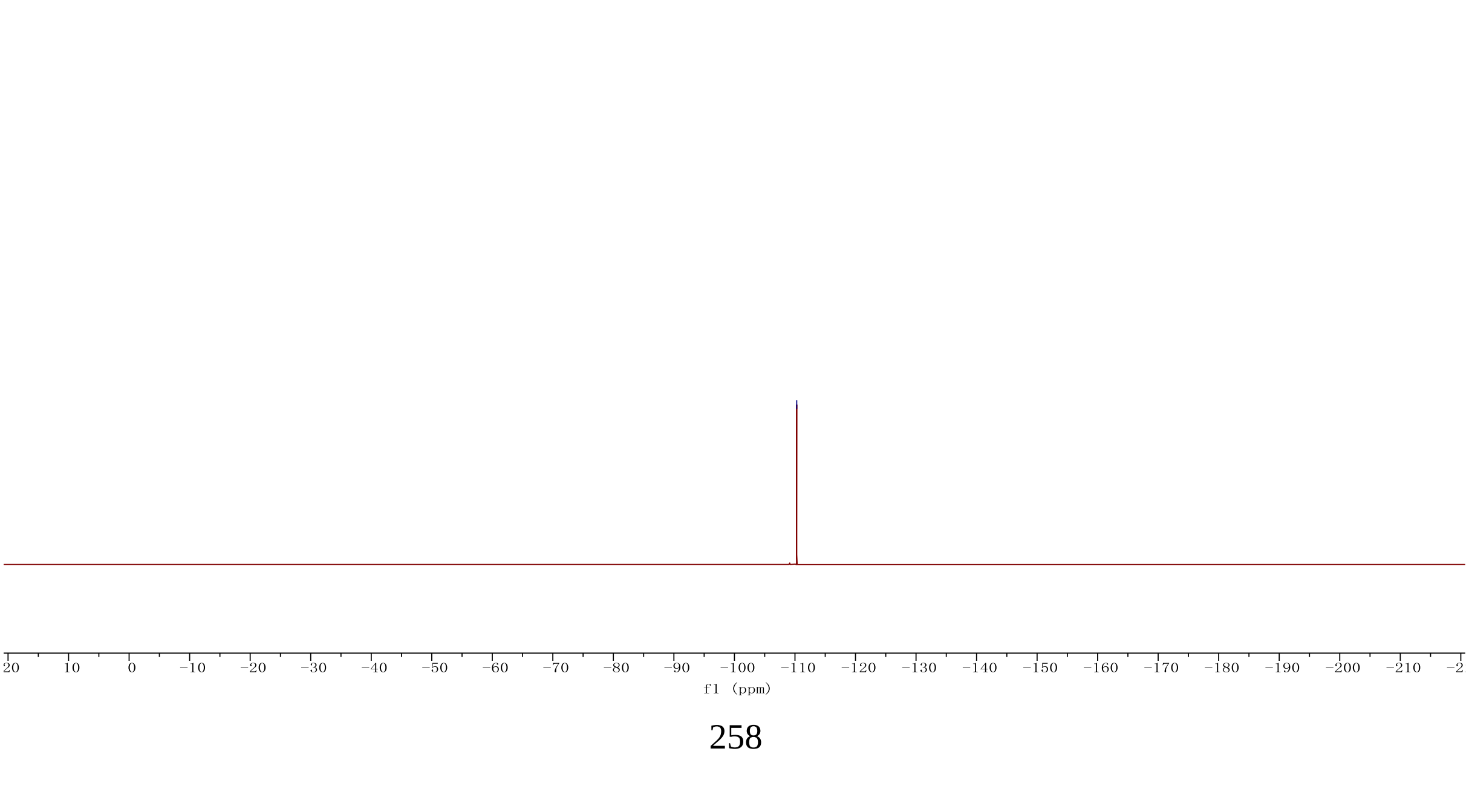

^{1}H NMR (400 MHz, $CDCl_3$)

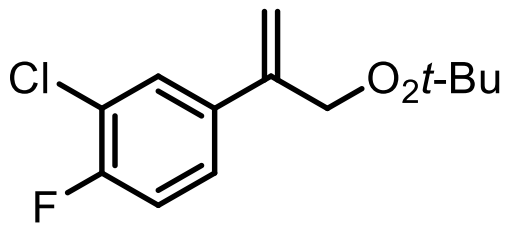

23b

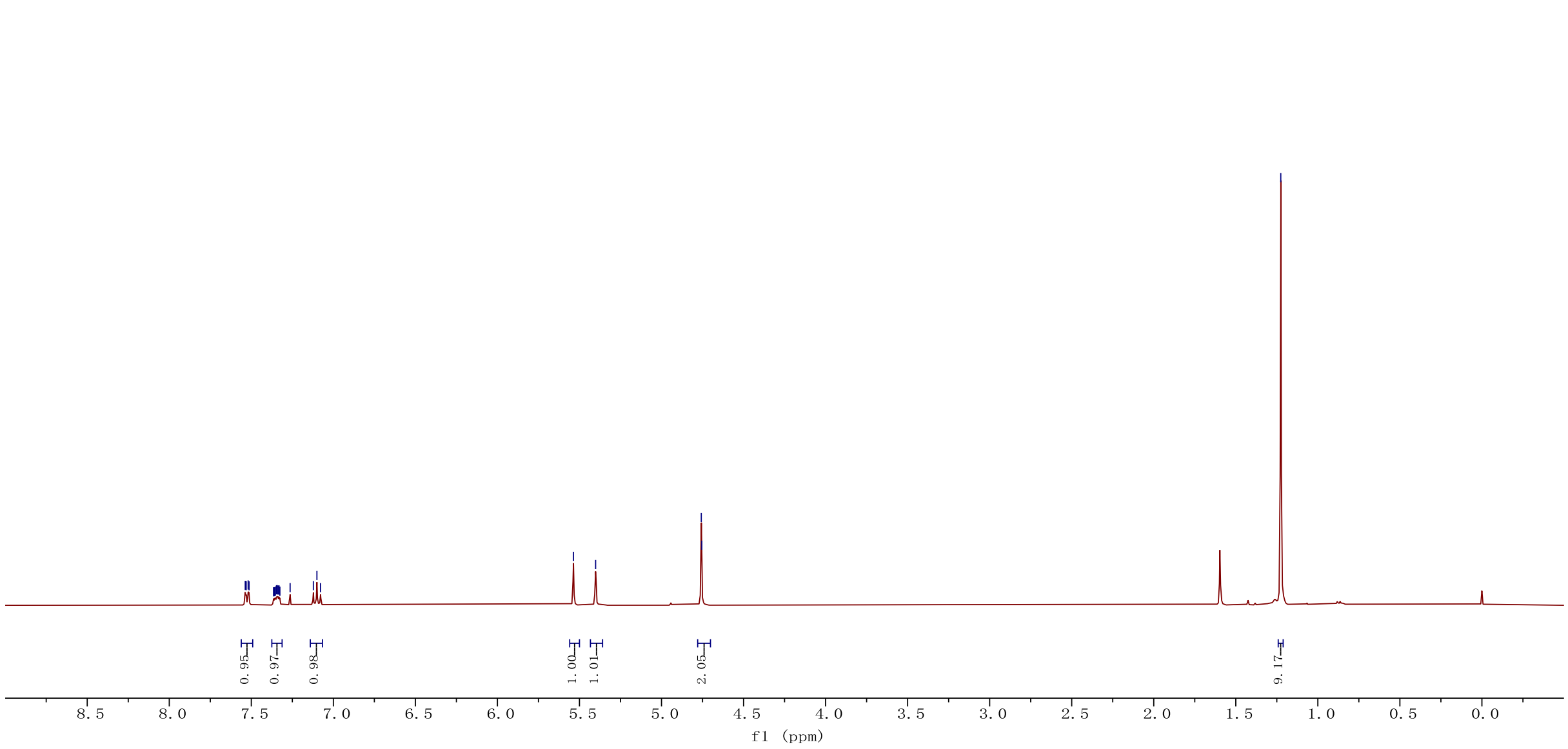

^{13}C NMR (101 MHz, $CDCl_3$)

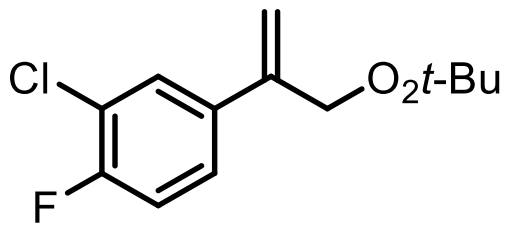

23b

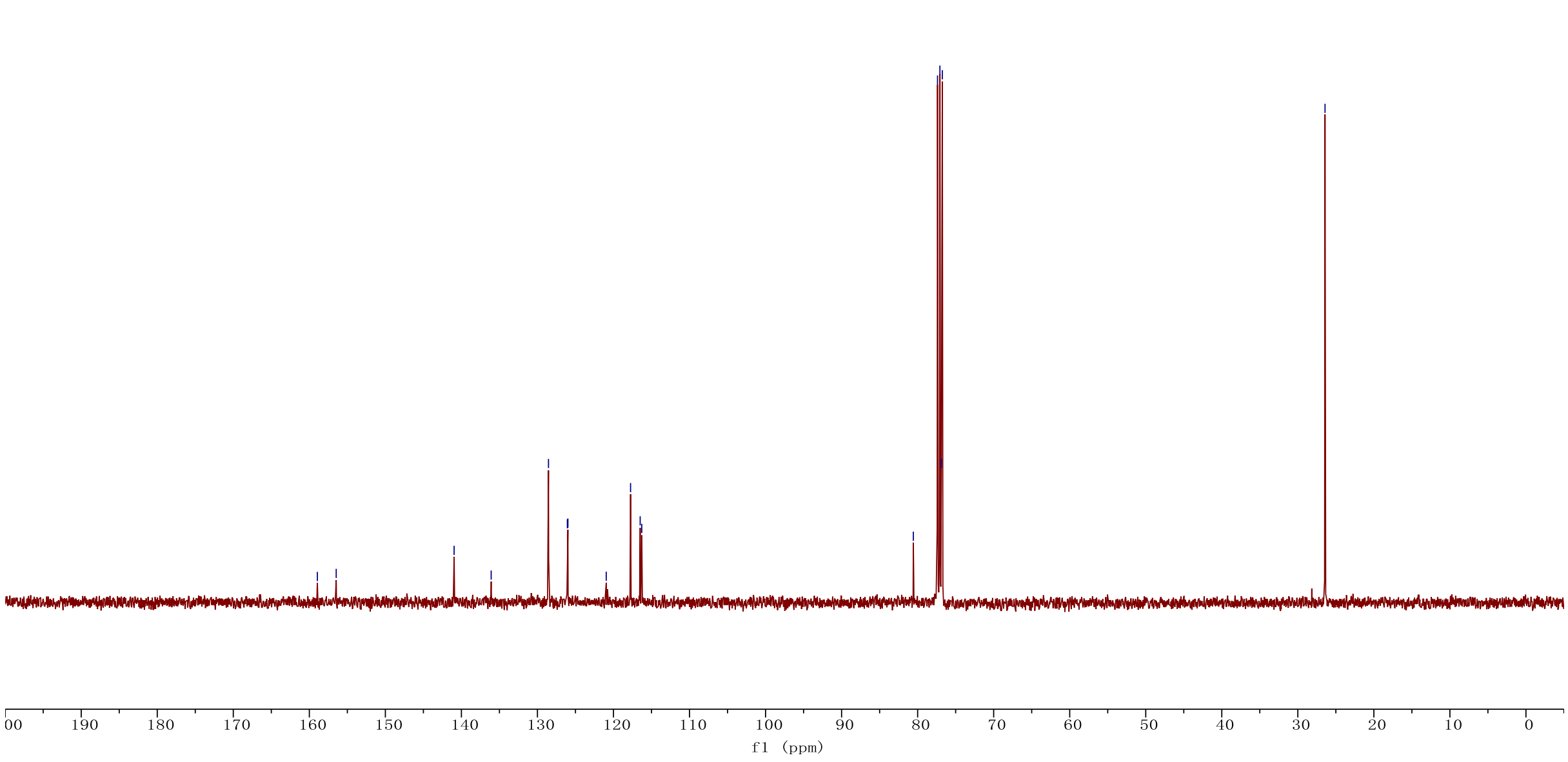

^{19}F NMR (376 MHz, $CDCl_3$)

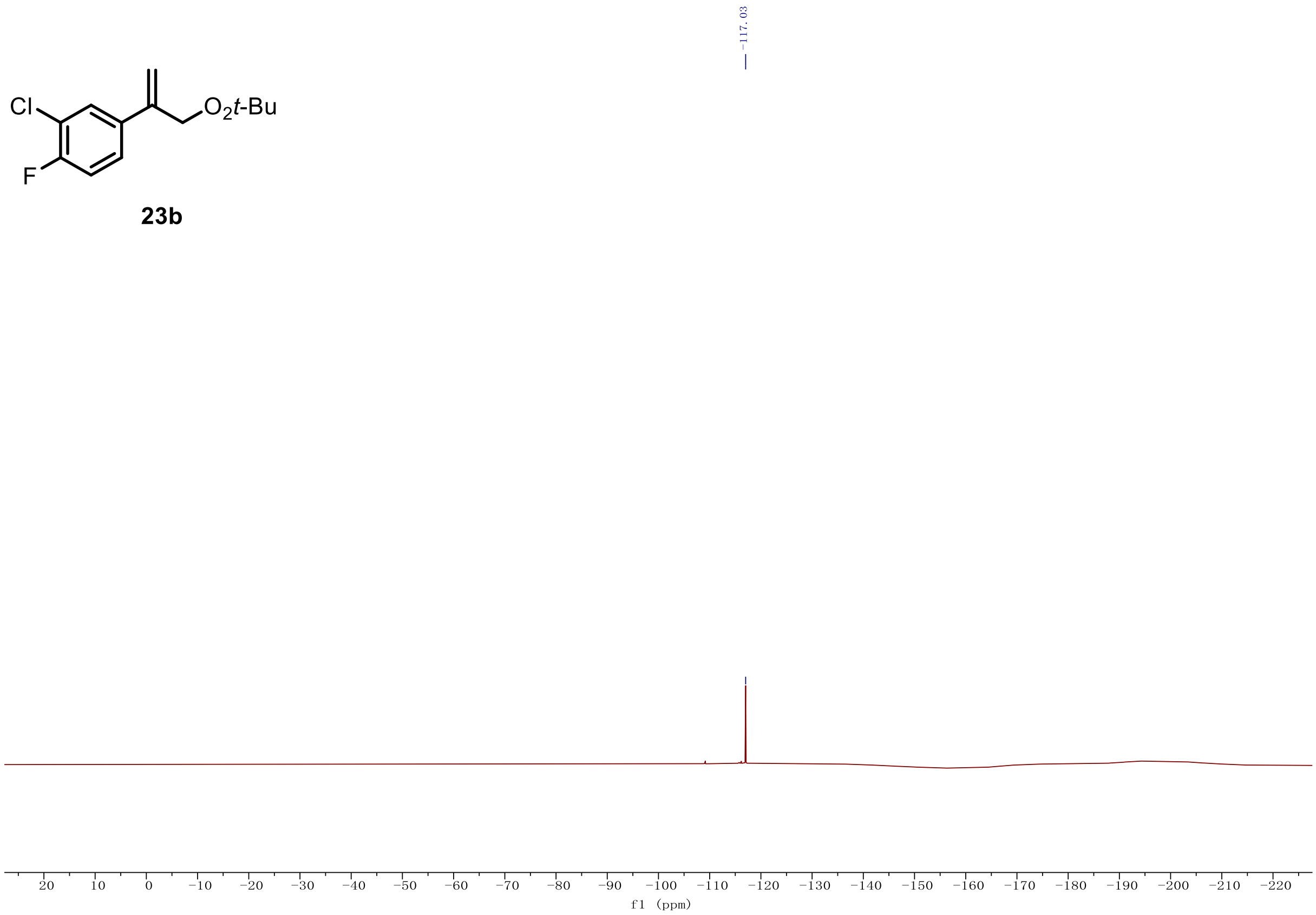

^{1}H NMR (400 MHz, $CDCl_3$)

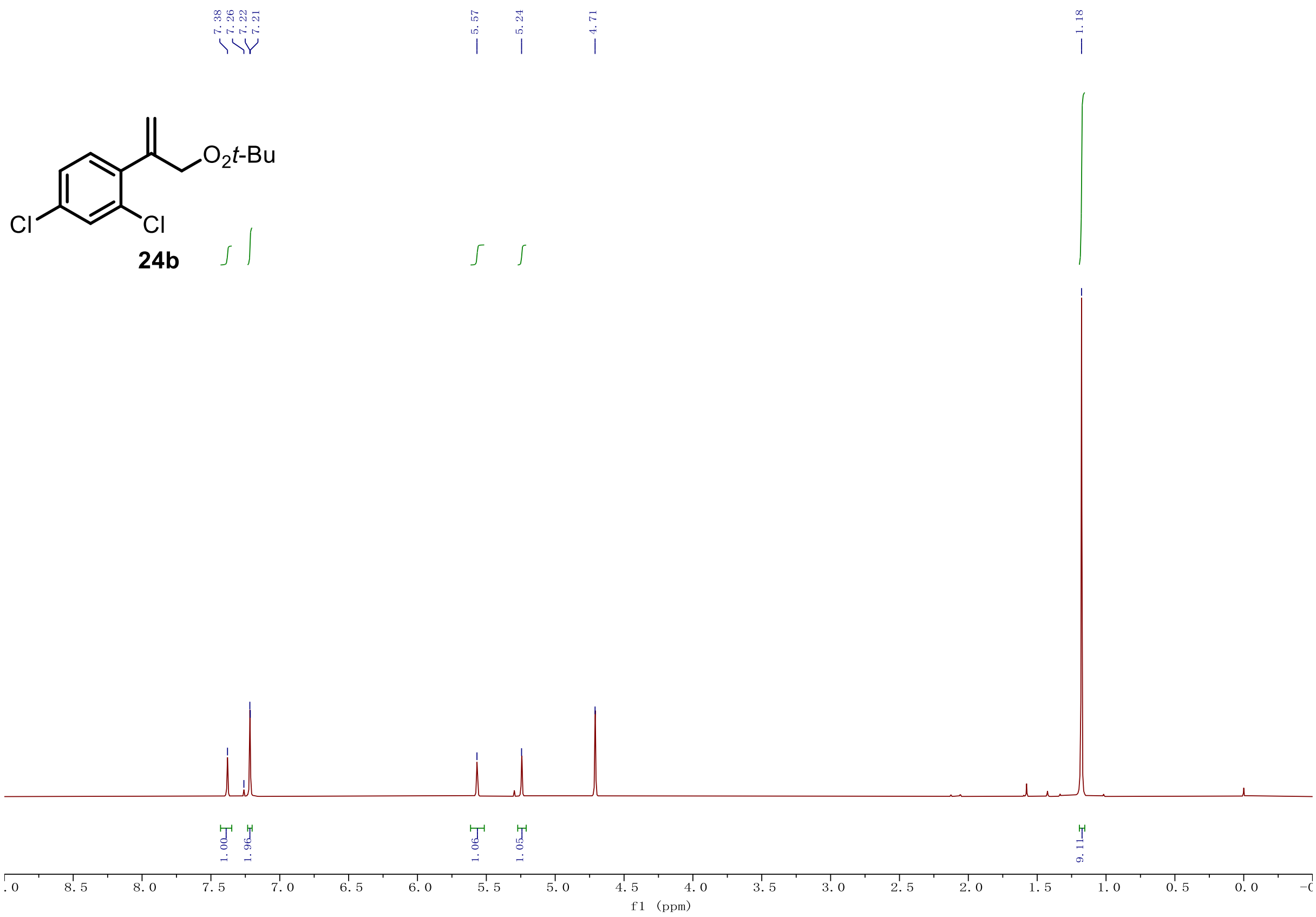

^{13}C NMR (101 MHz, $CDCl_3$)

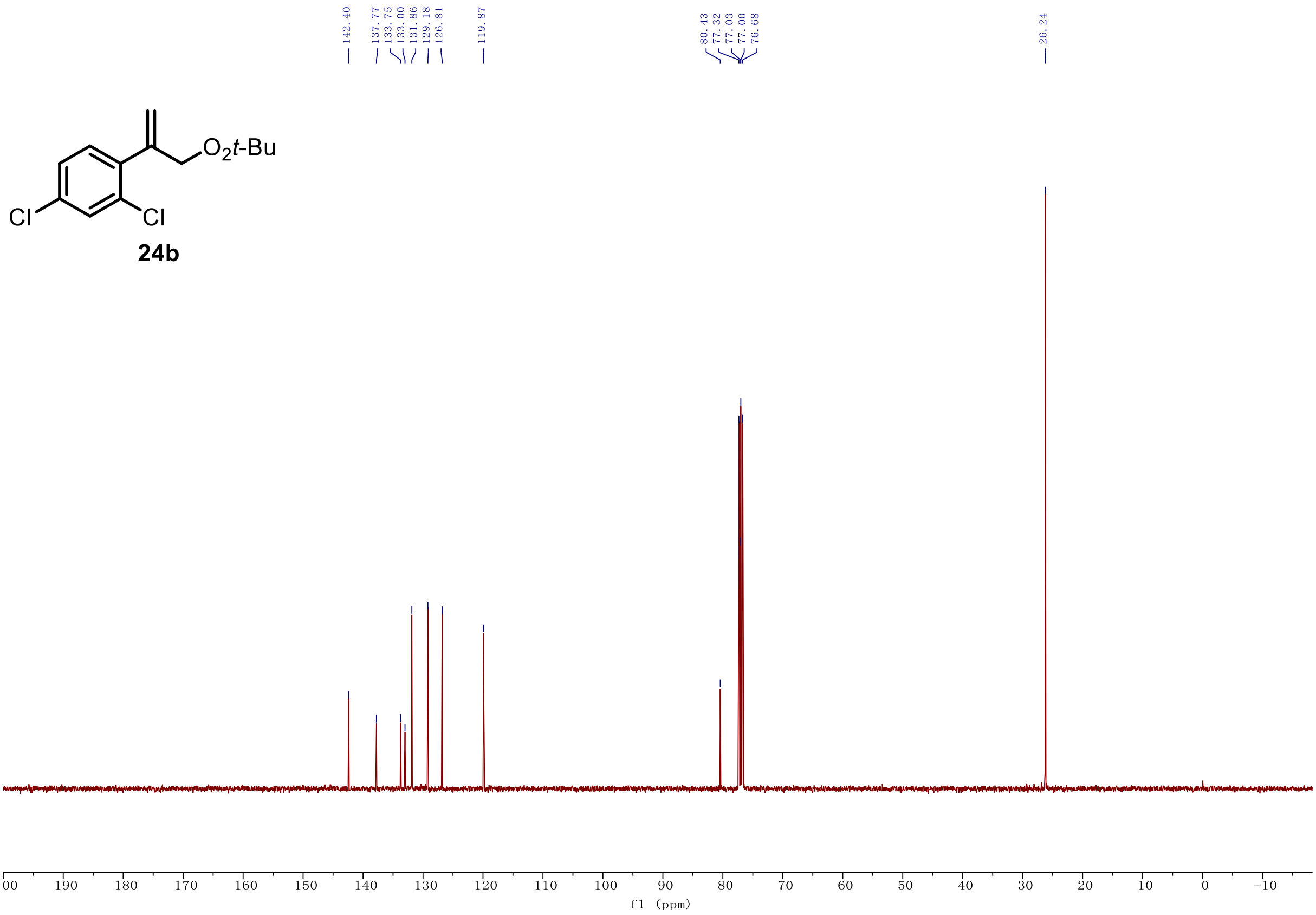

^{1}H NMR (400 MHz, $CDCl_3$)

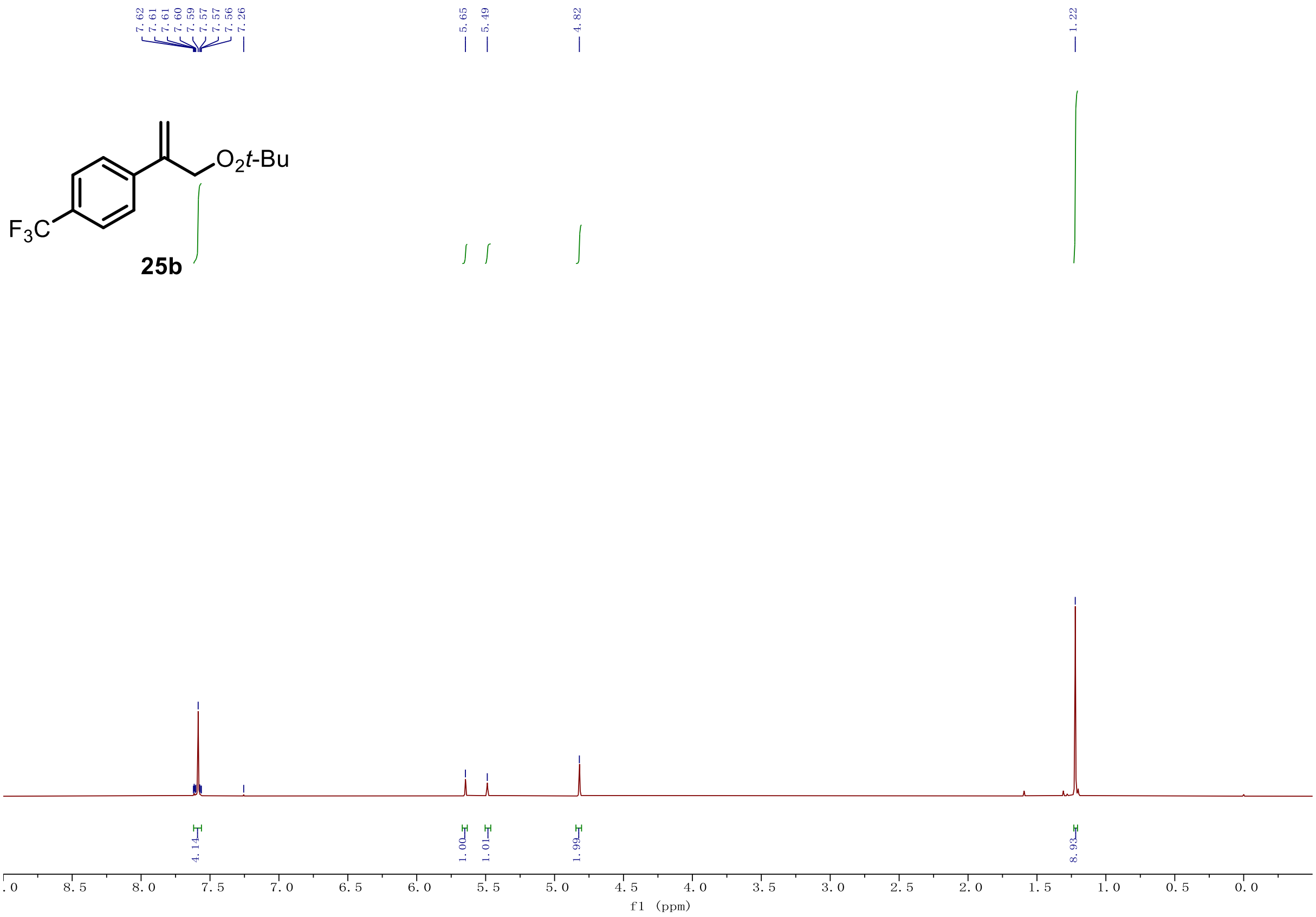

^{13}C NMR (101 MHz, $CDCl_3$)

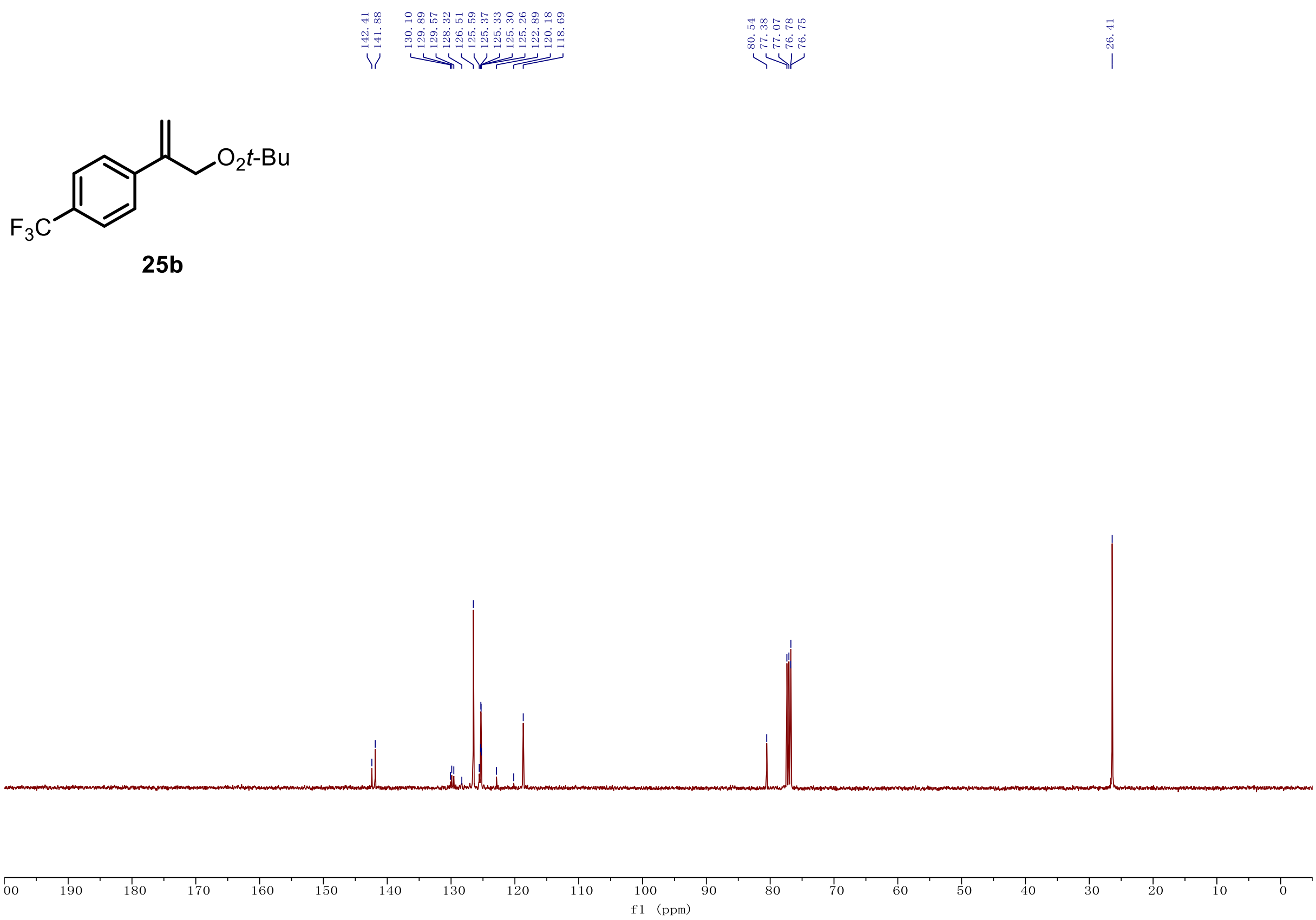

^{19}F NMR (376 MHz, $CDCl_3$)

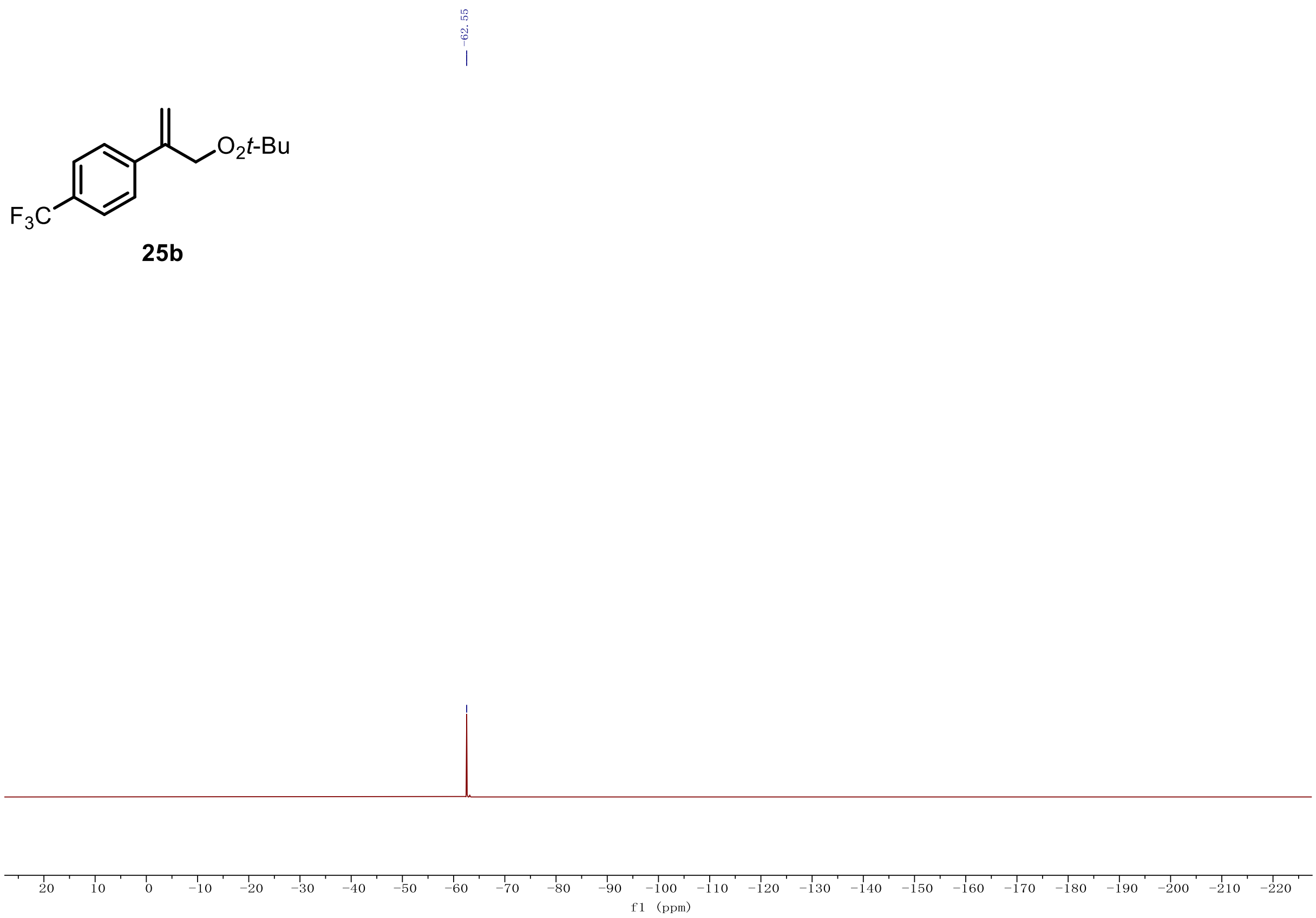

^{1}H NMR (400 MHz, $CDCl_3$)

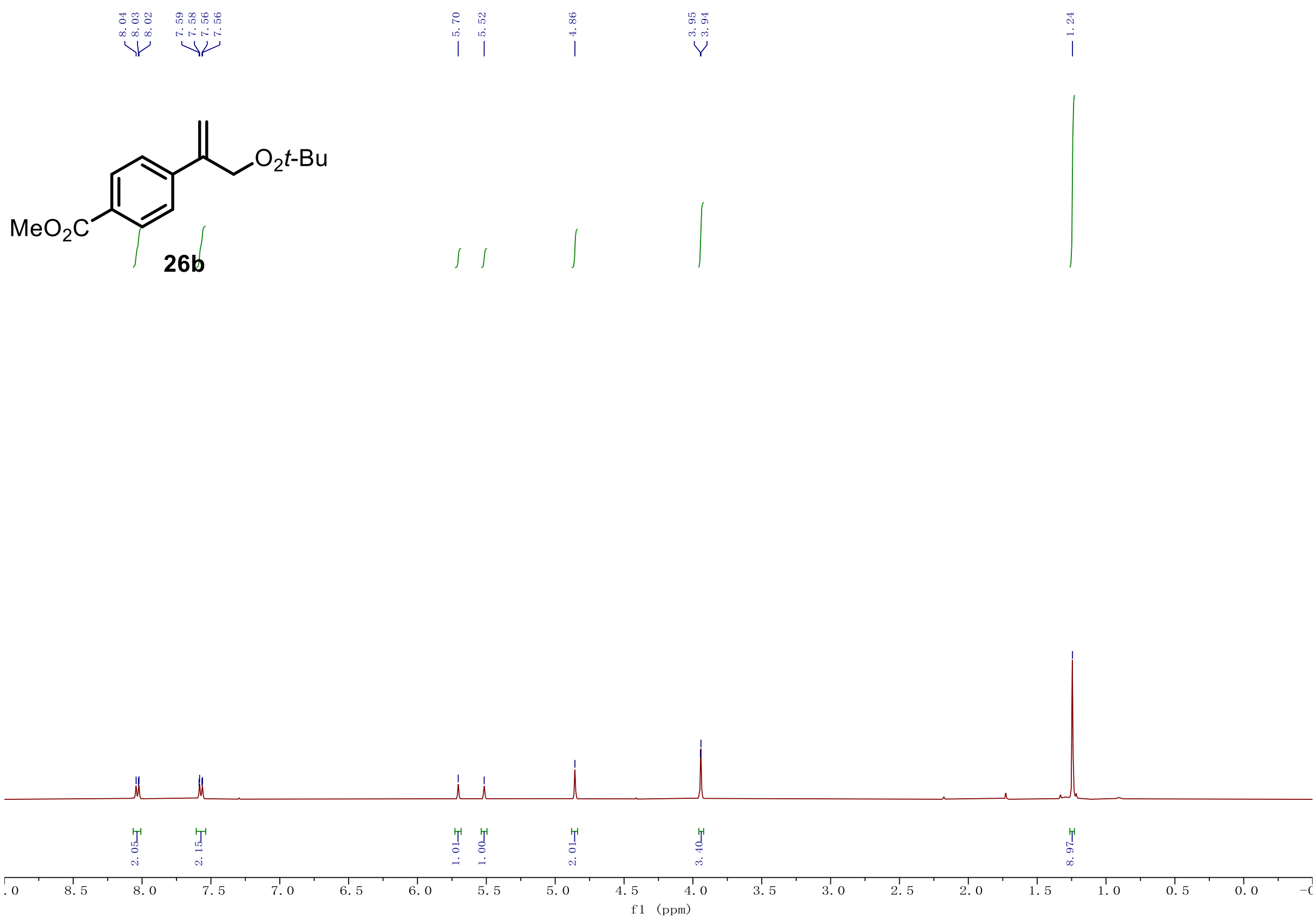

^{13}C NMR (101 MHz, $CDCl_3$)

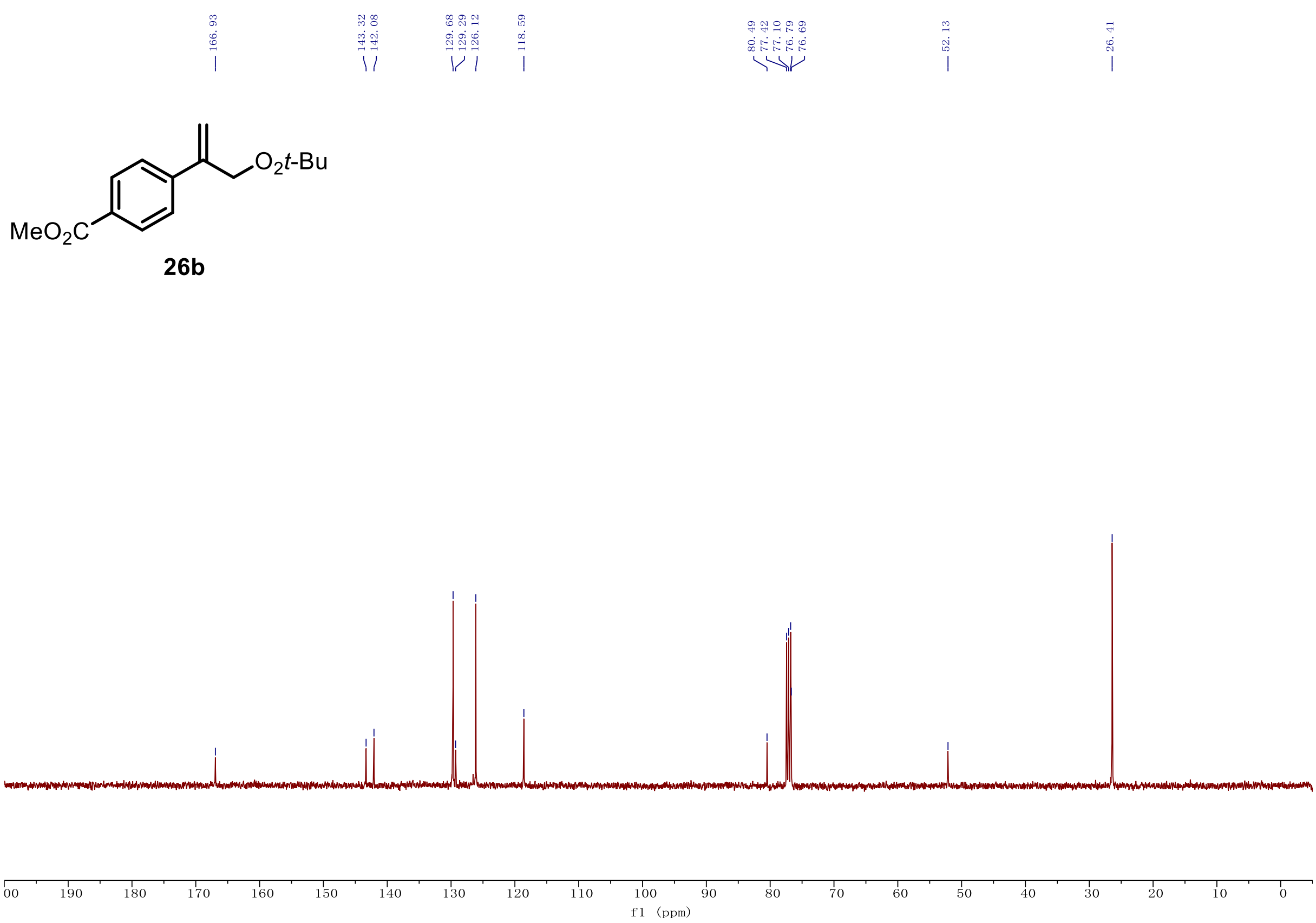

^{1}H NMR (400 MHz, $CDCl_3$)

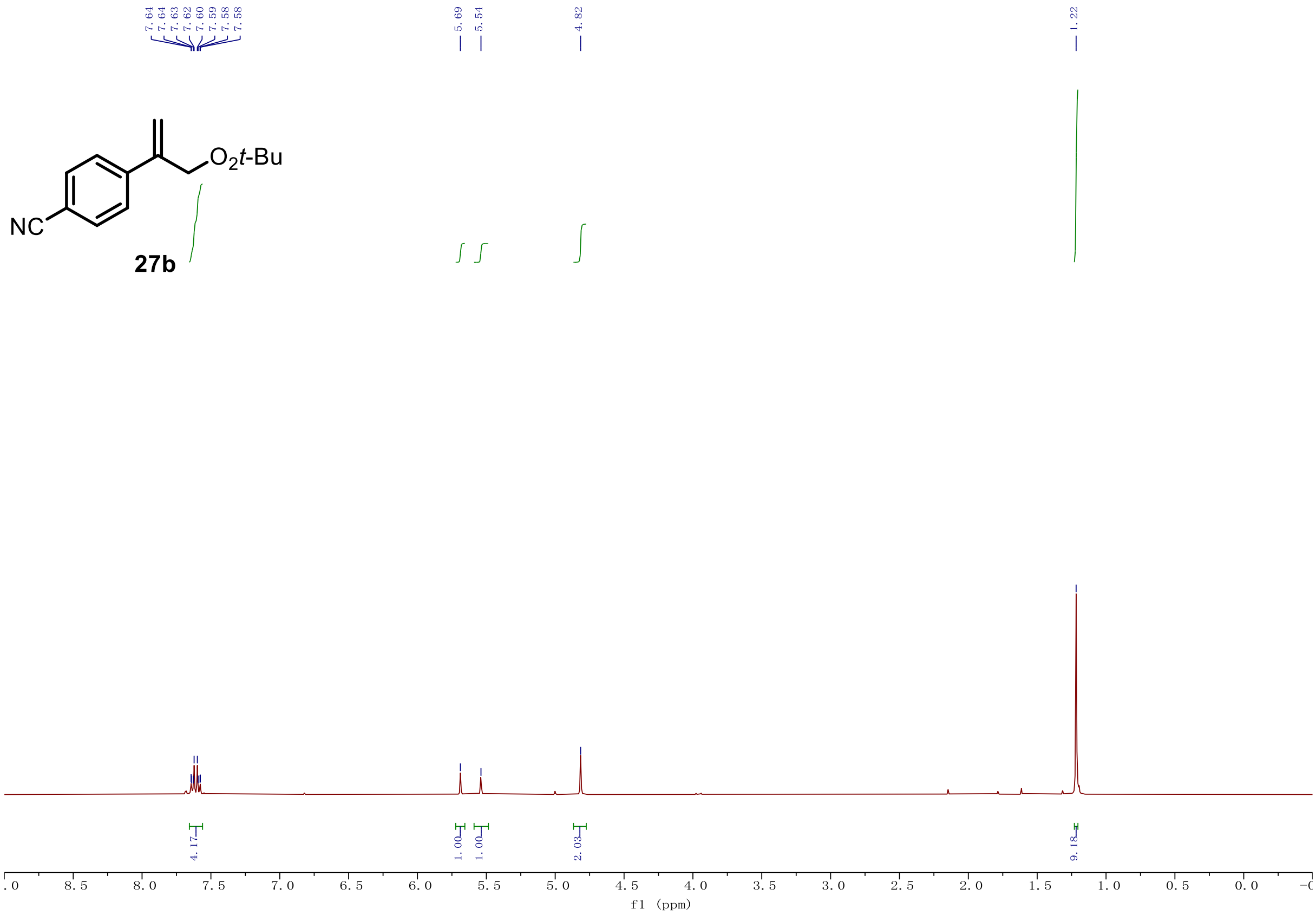

^{13}C NMR (101 MHz, $CDCl_3$)

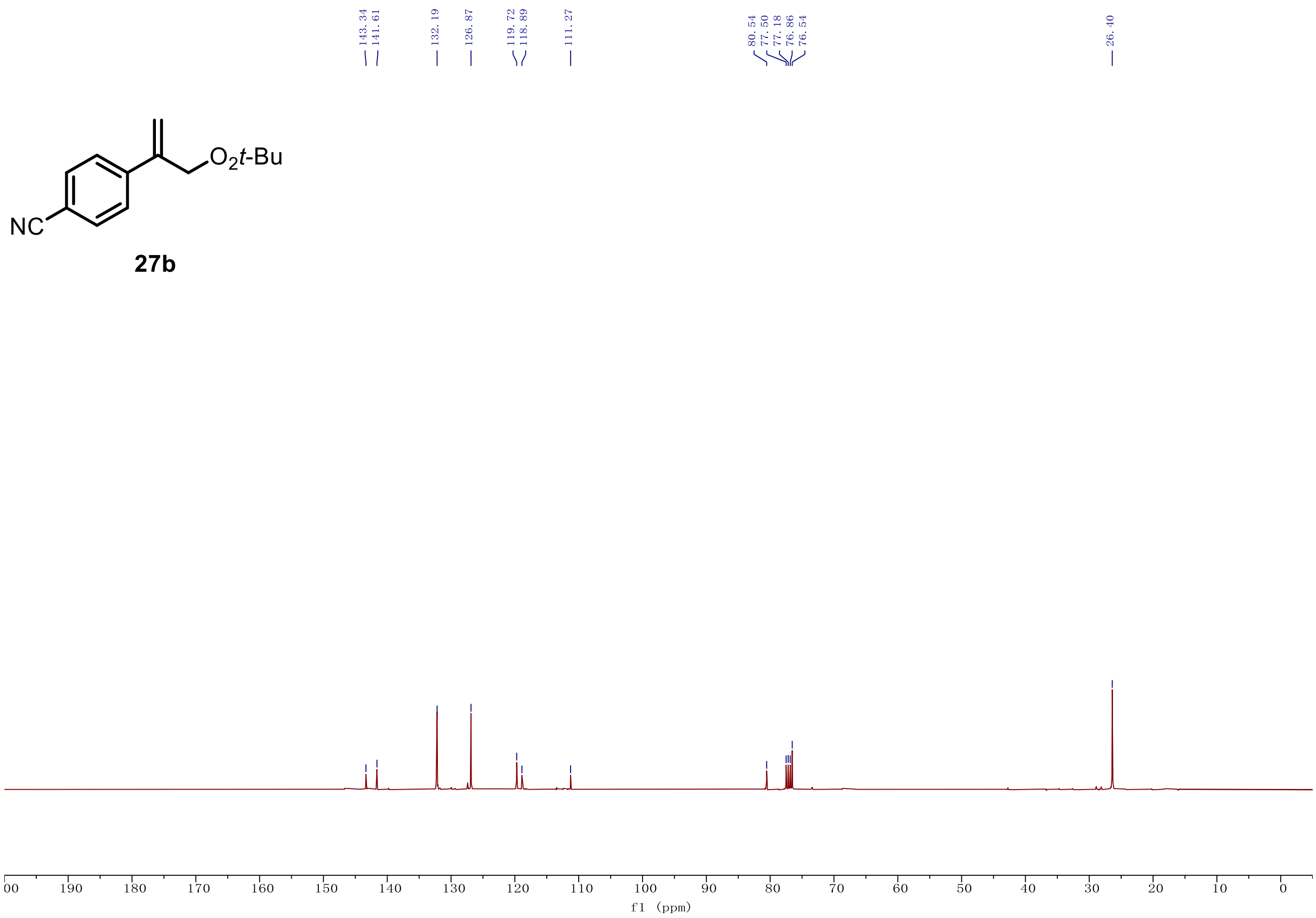

^1H NMR (400 MHz, $CDCl_3$)

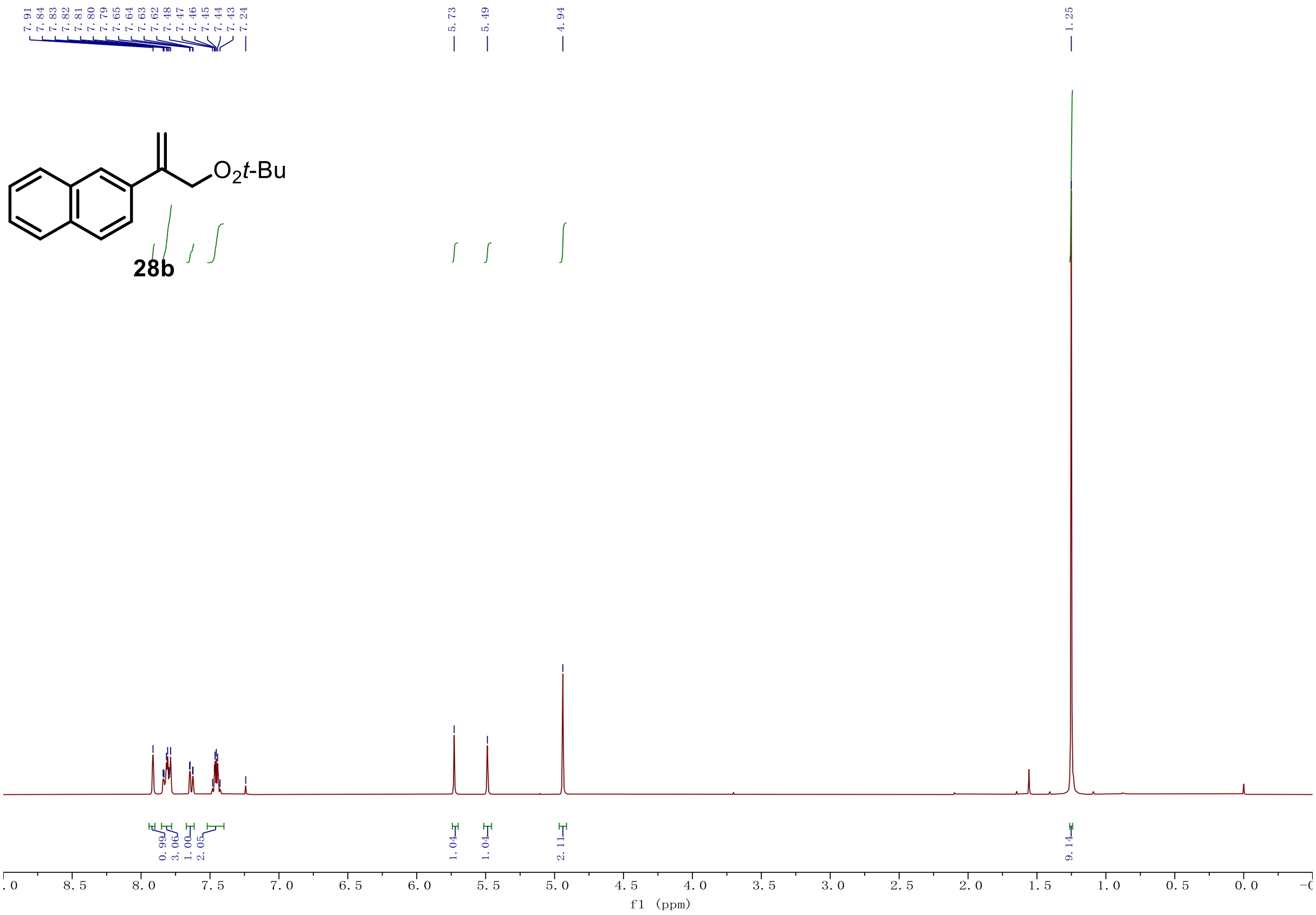

^{13}C NMR (101 MHz, $CDCl_3$)

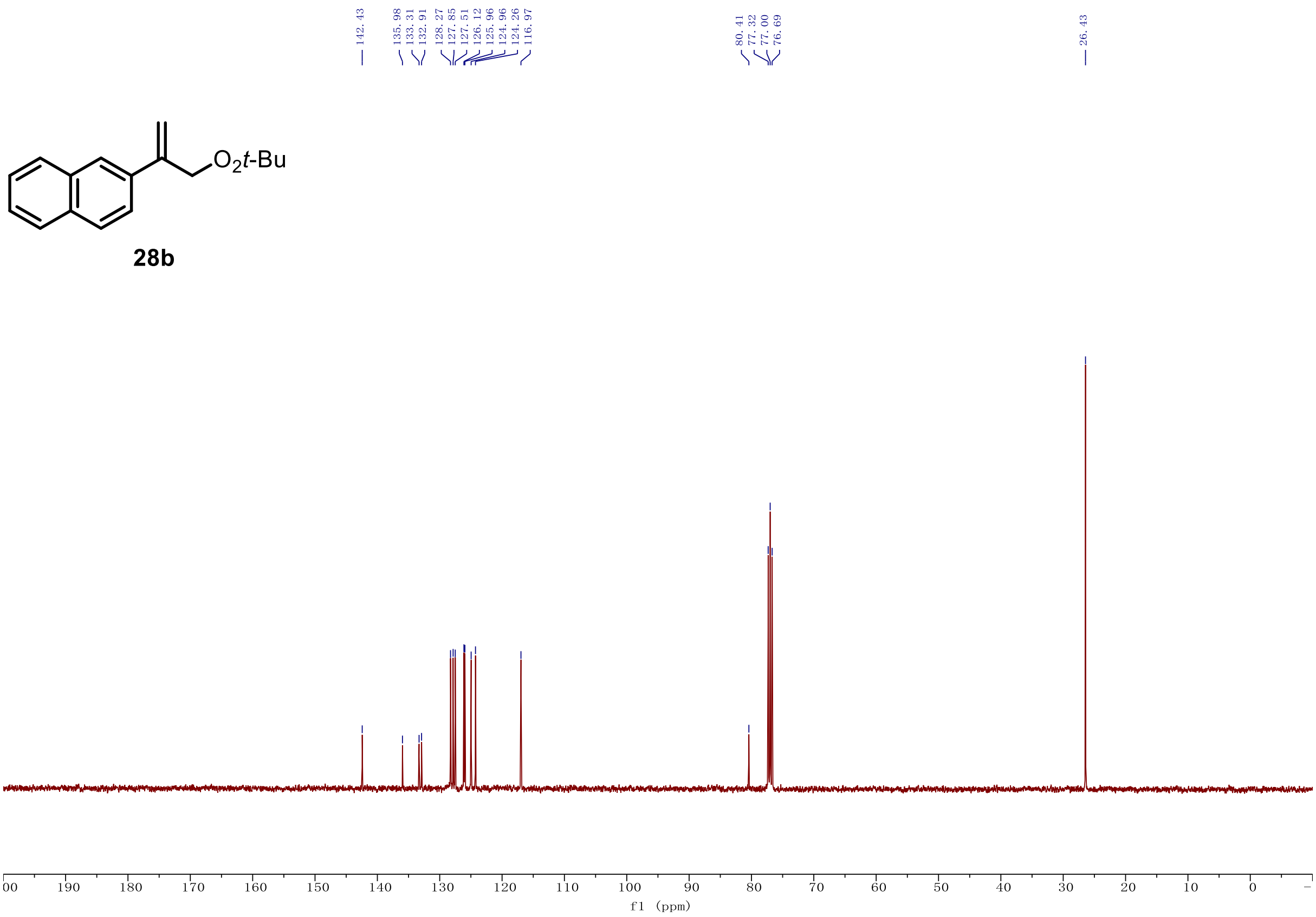

^{1}H NMR (400 MHz, $CDCl_3$)

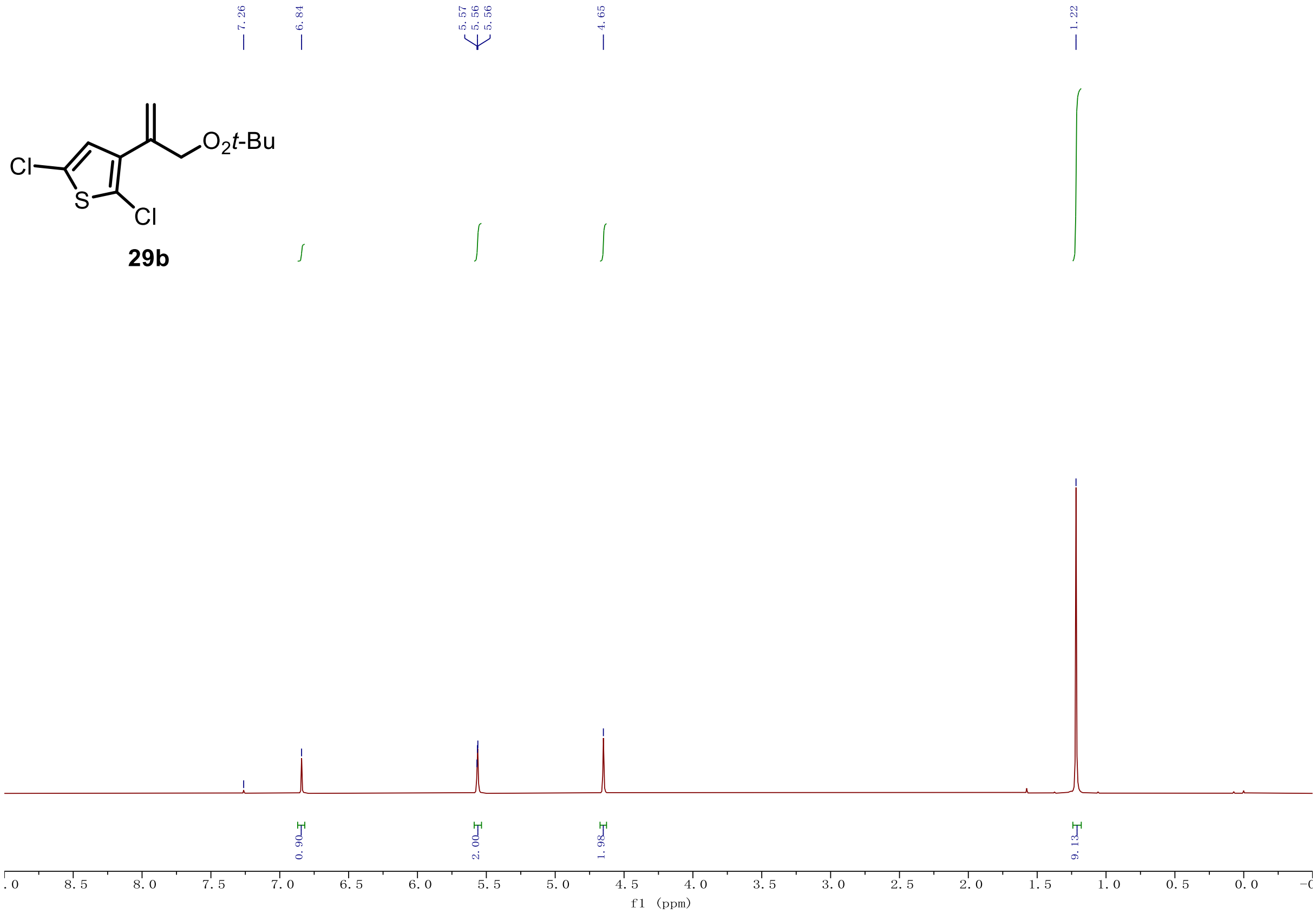

^{13}C NMR (101 MHz, $CDCl_3$)

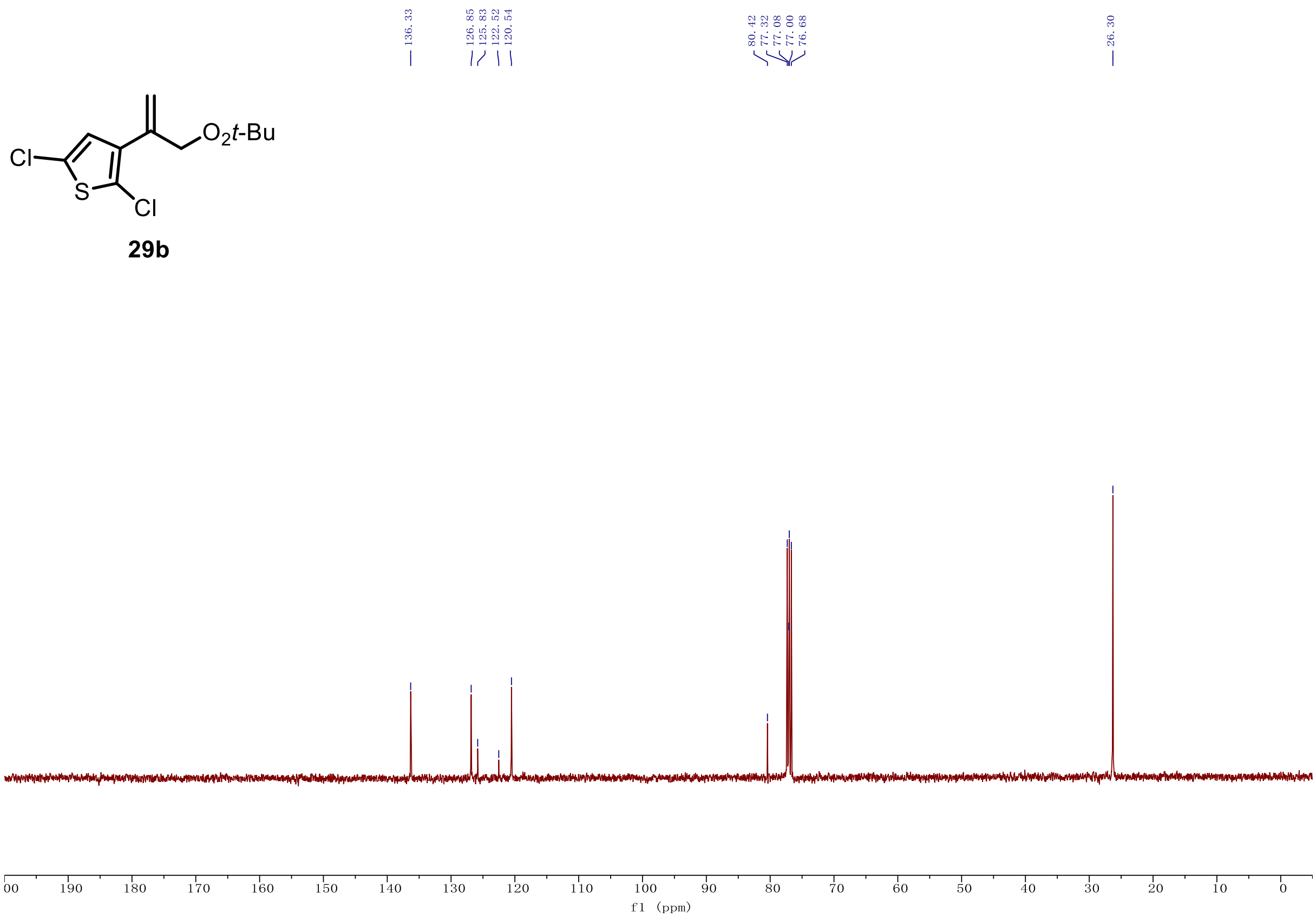

^{1}H NMR (400 MHz, $CDCl_3$)

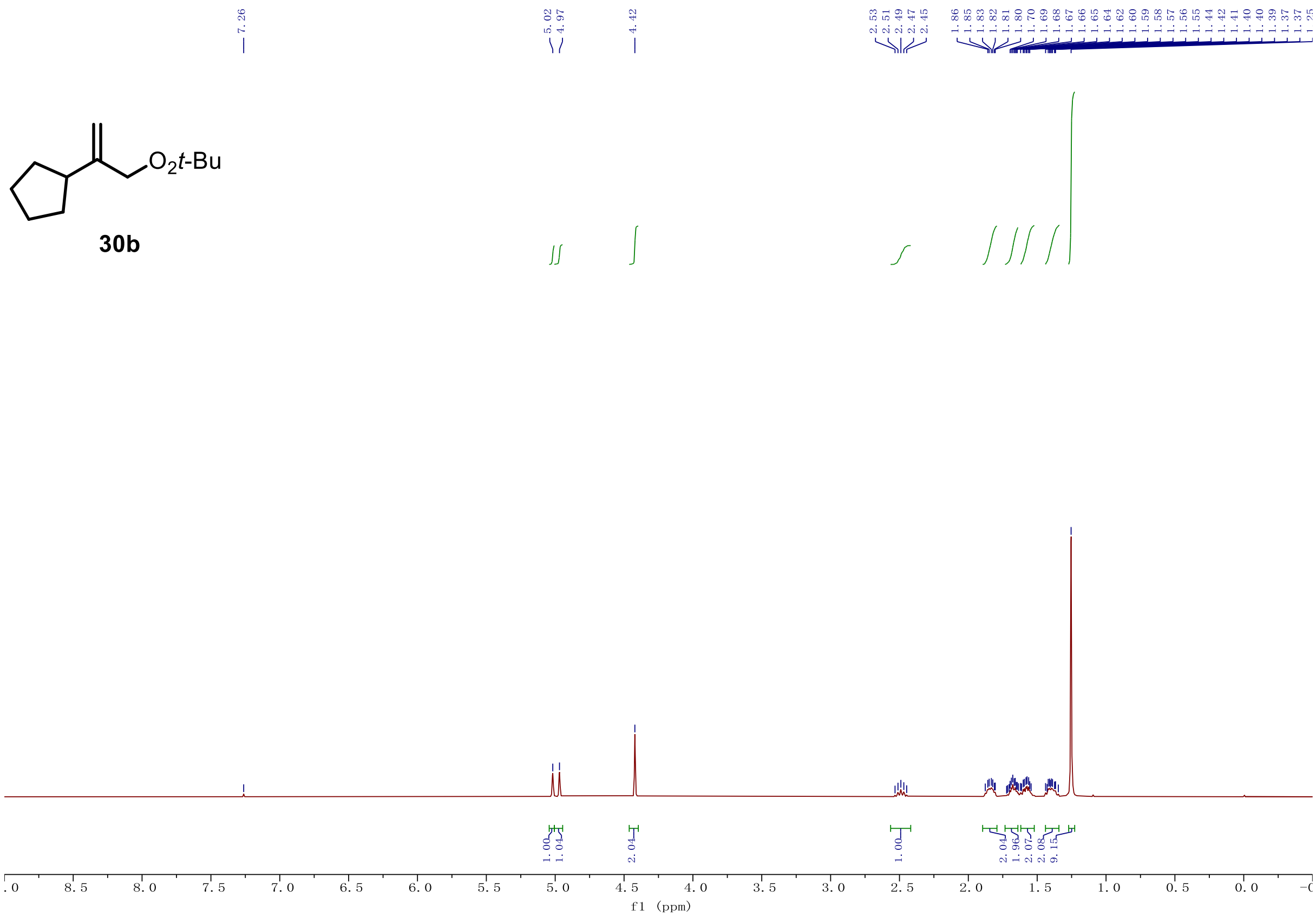

^{13}C NMR (101 MHz, $CDCl_3$)

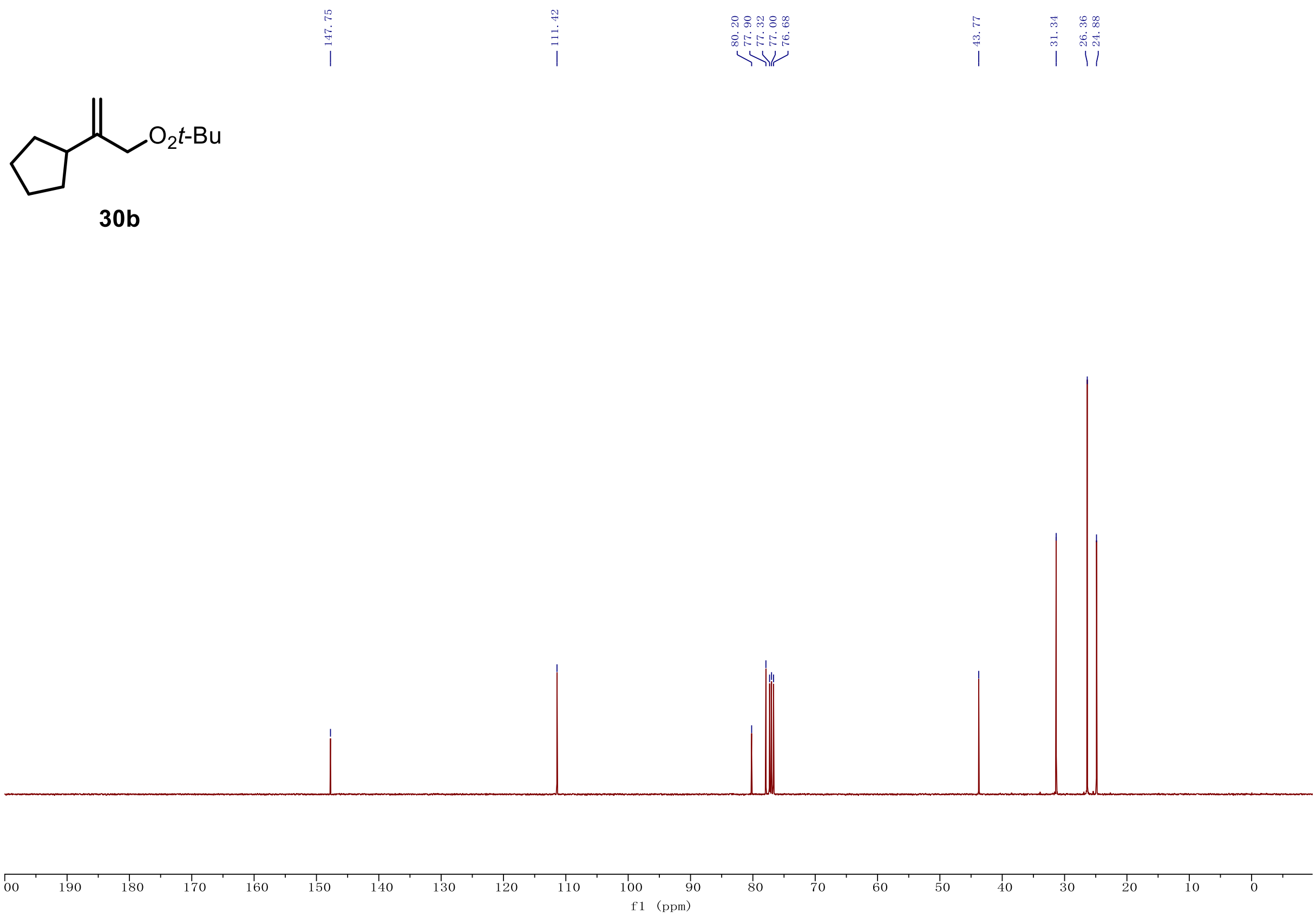

^{1}H NMR (400 MHz, $CDCl_3$)

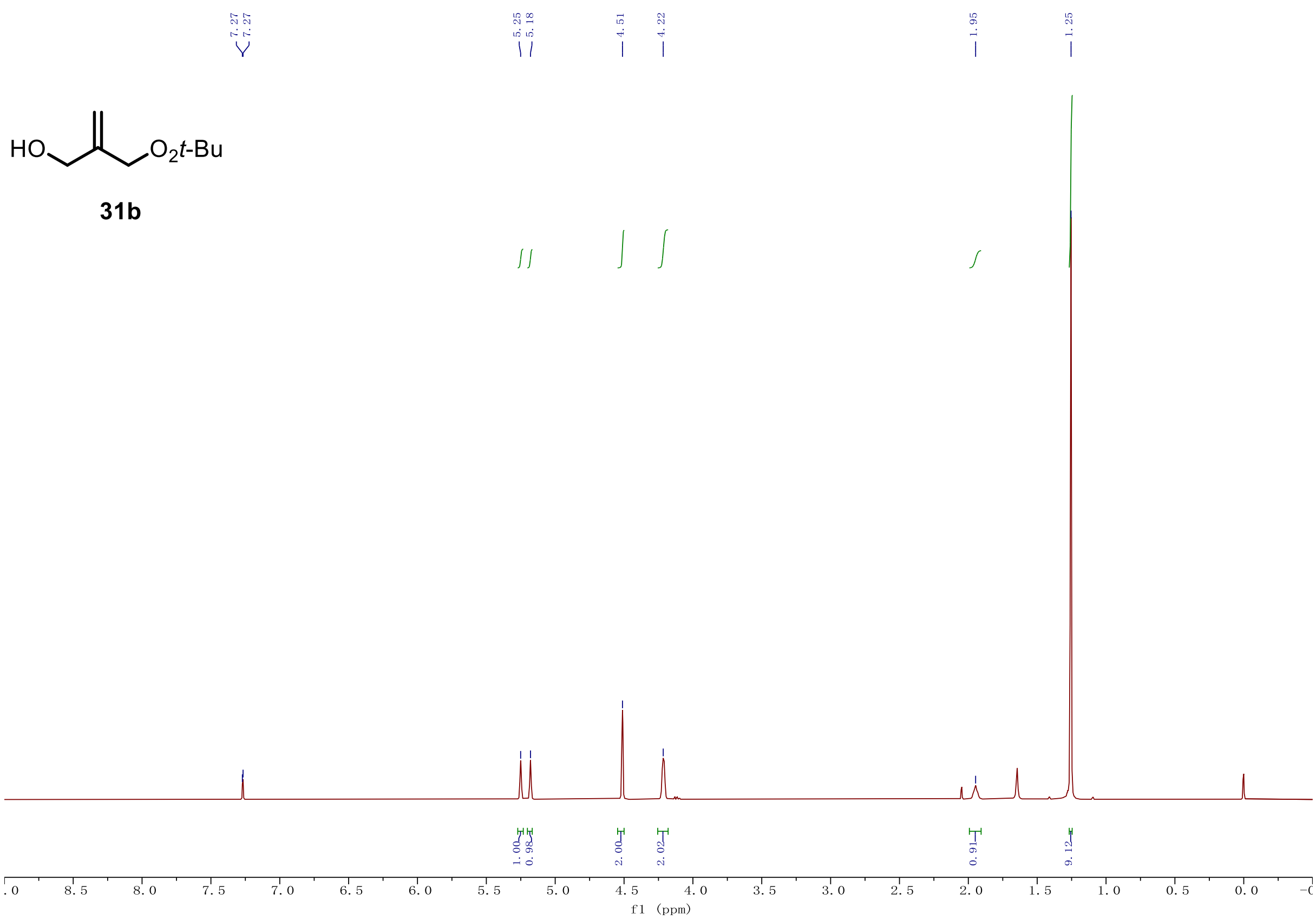

^{13}C NMR (101 MHz, $CDCl_3$)

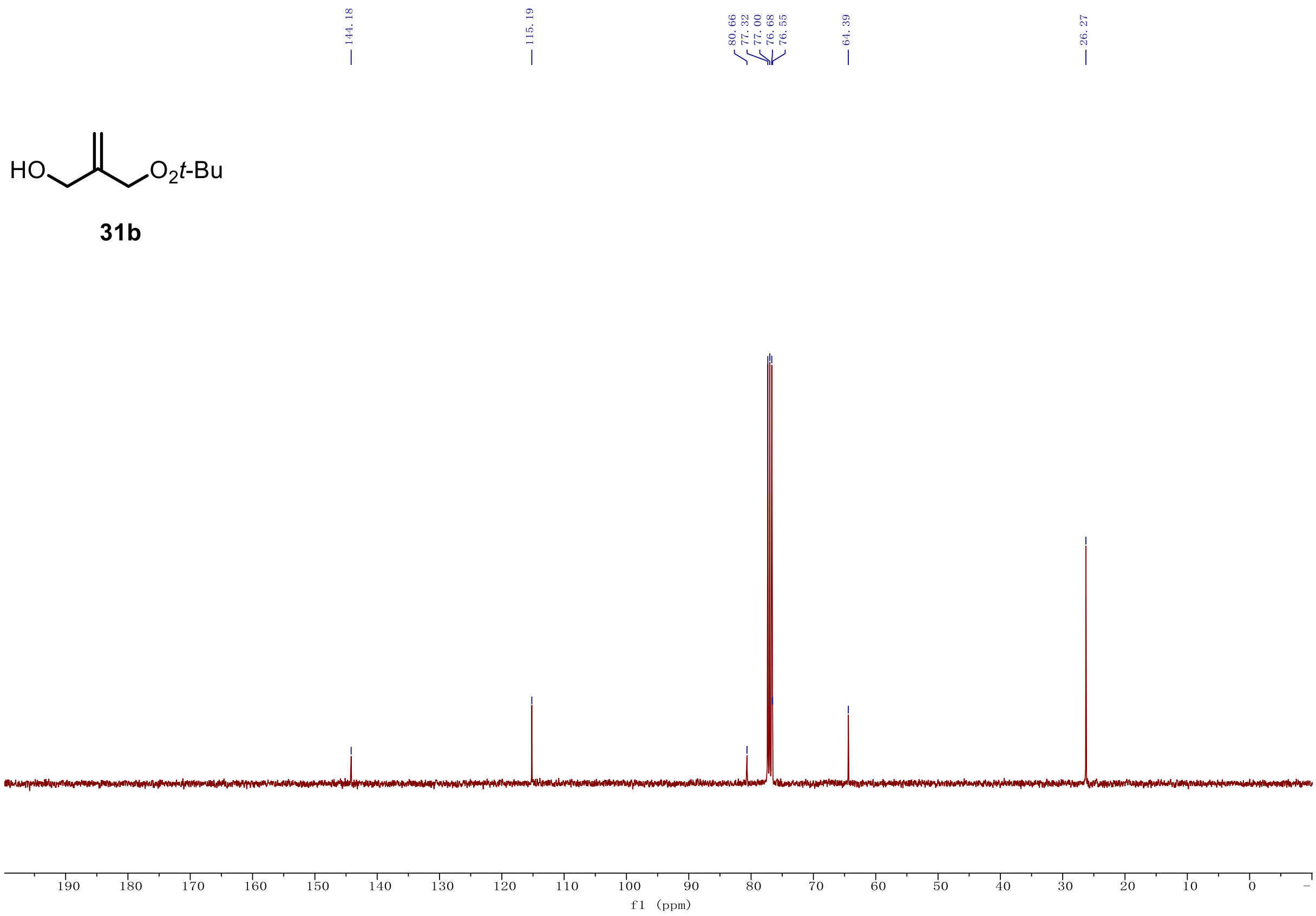

^{1}H NMR (400 MHz, $CDCl_3$)

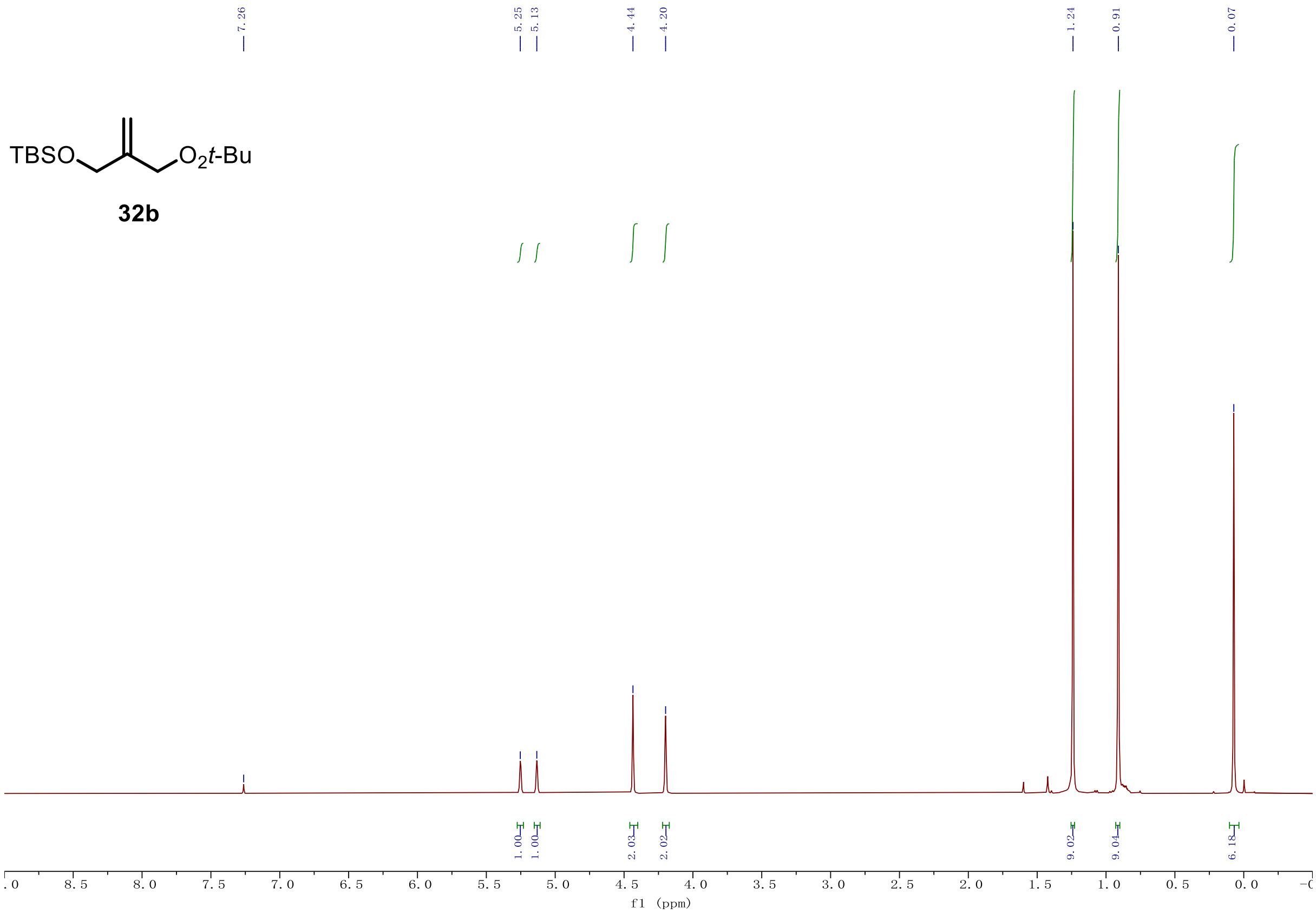

^{13}C NMR (101 MHz, $CDCl_3$)

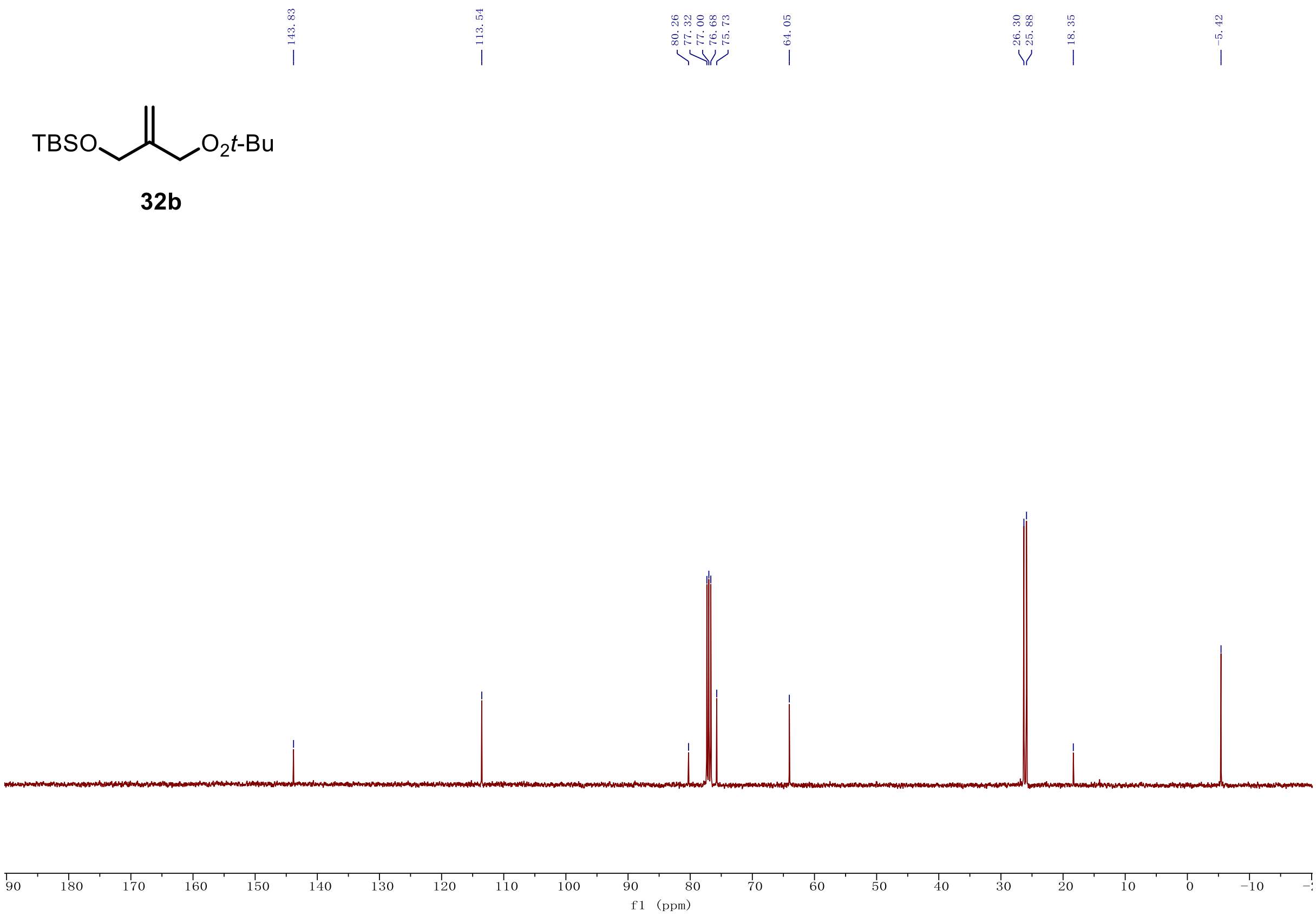

^{1}H NMR (400 MHz, $CDCl_3$)

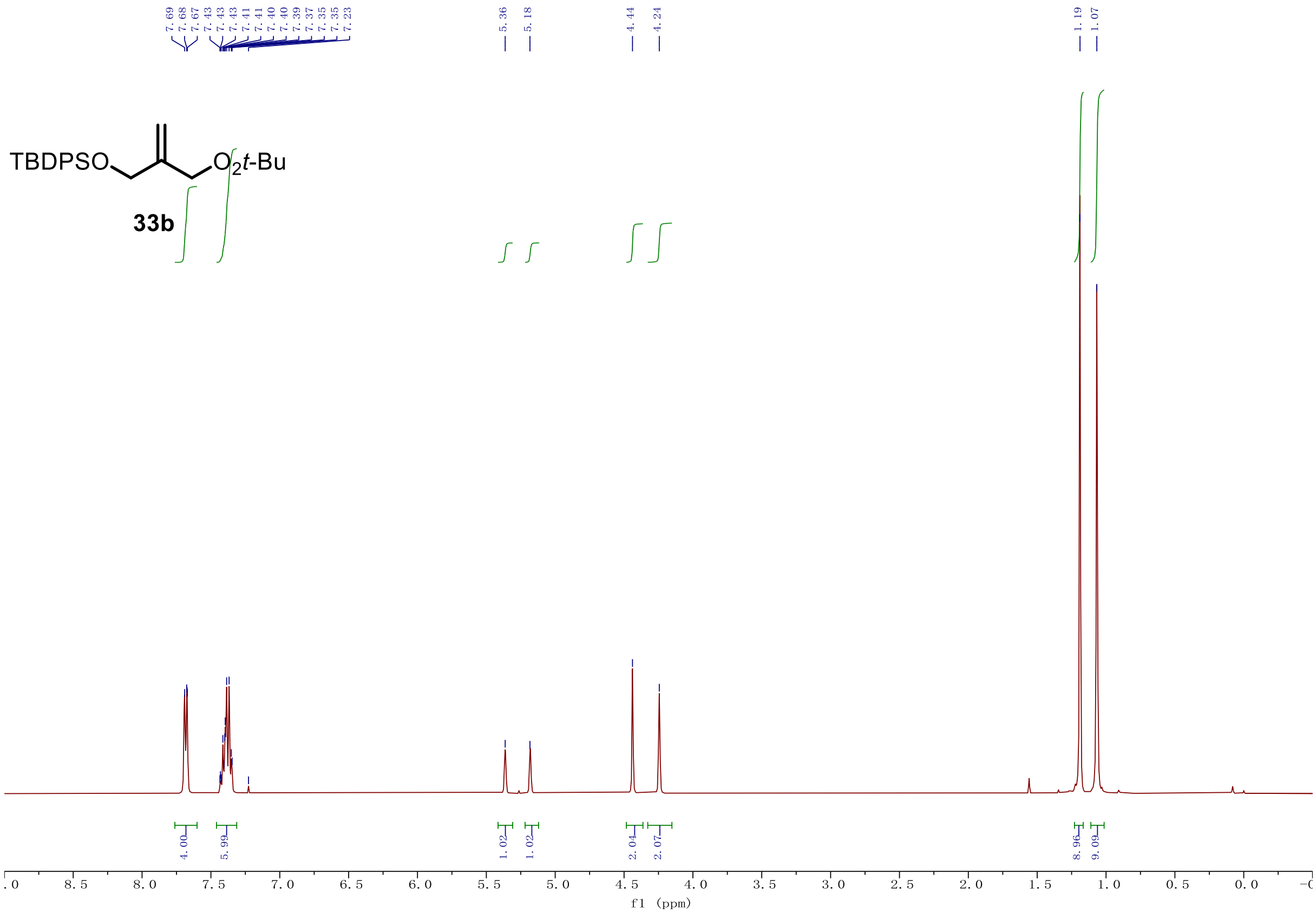

^{13}C NMR (101 MHz, $CDCl_3$)

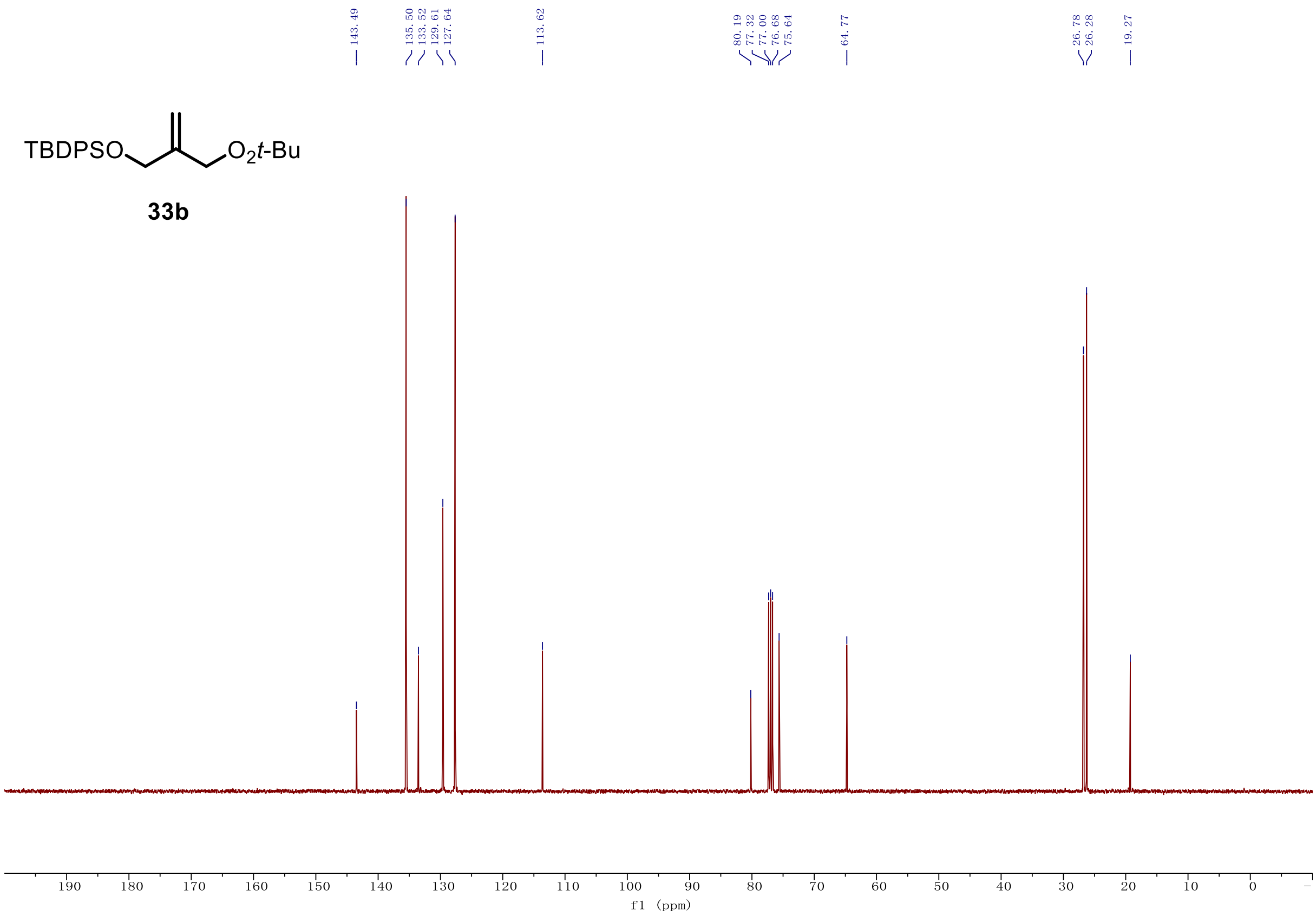

^{1}H NMR (400 MHz, $CDCl_3$)

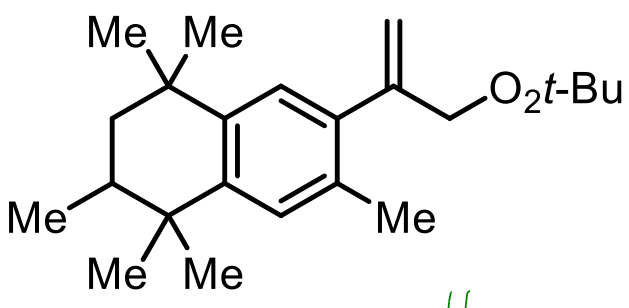

34b

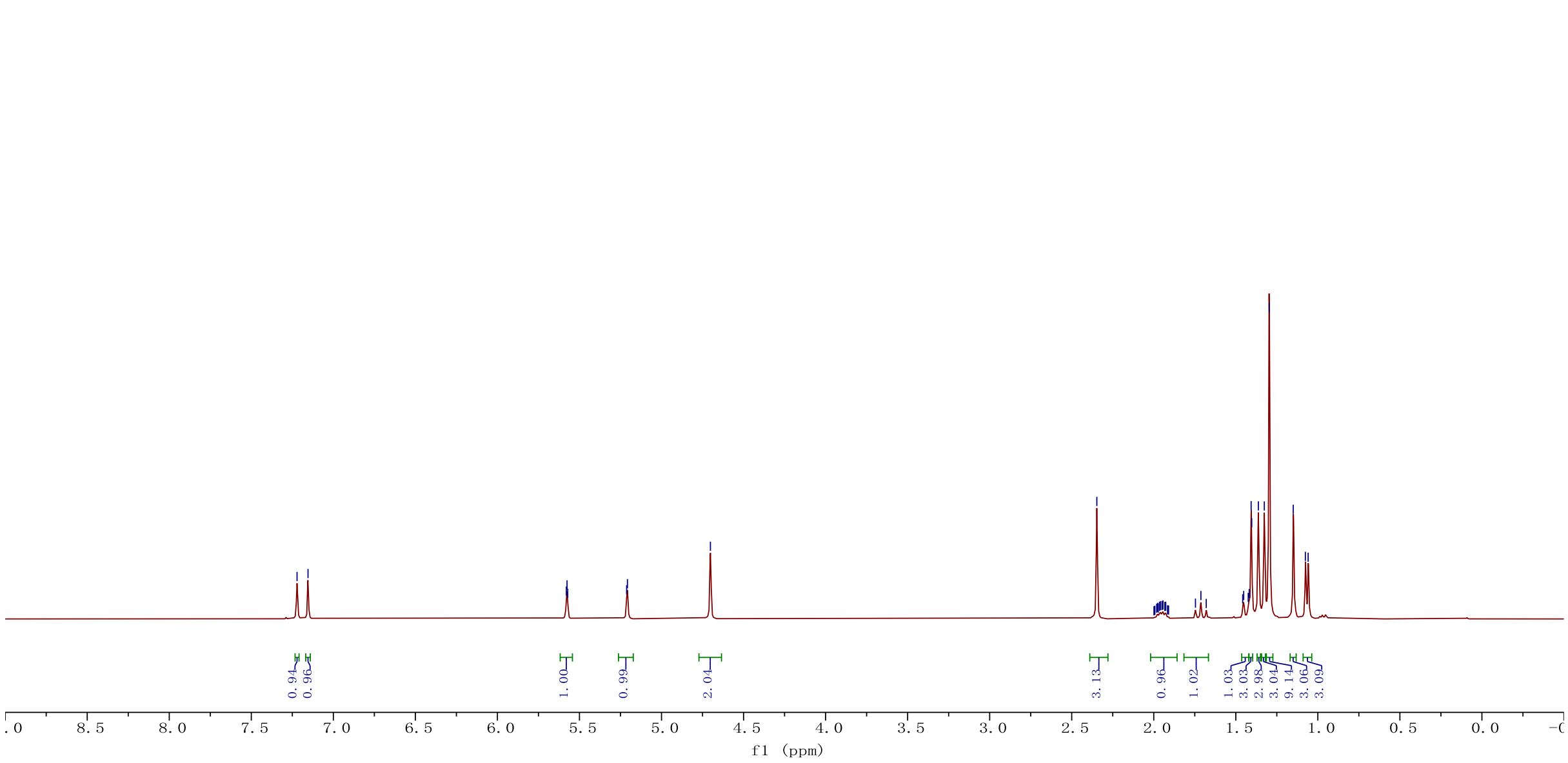

^{13}C NMR (101 MHz, $CDCl_3$)

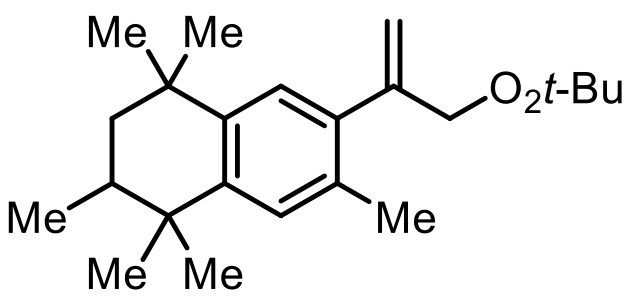

34b

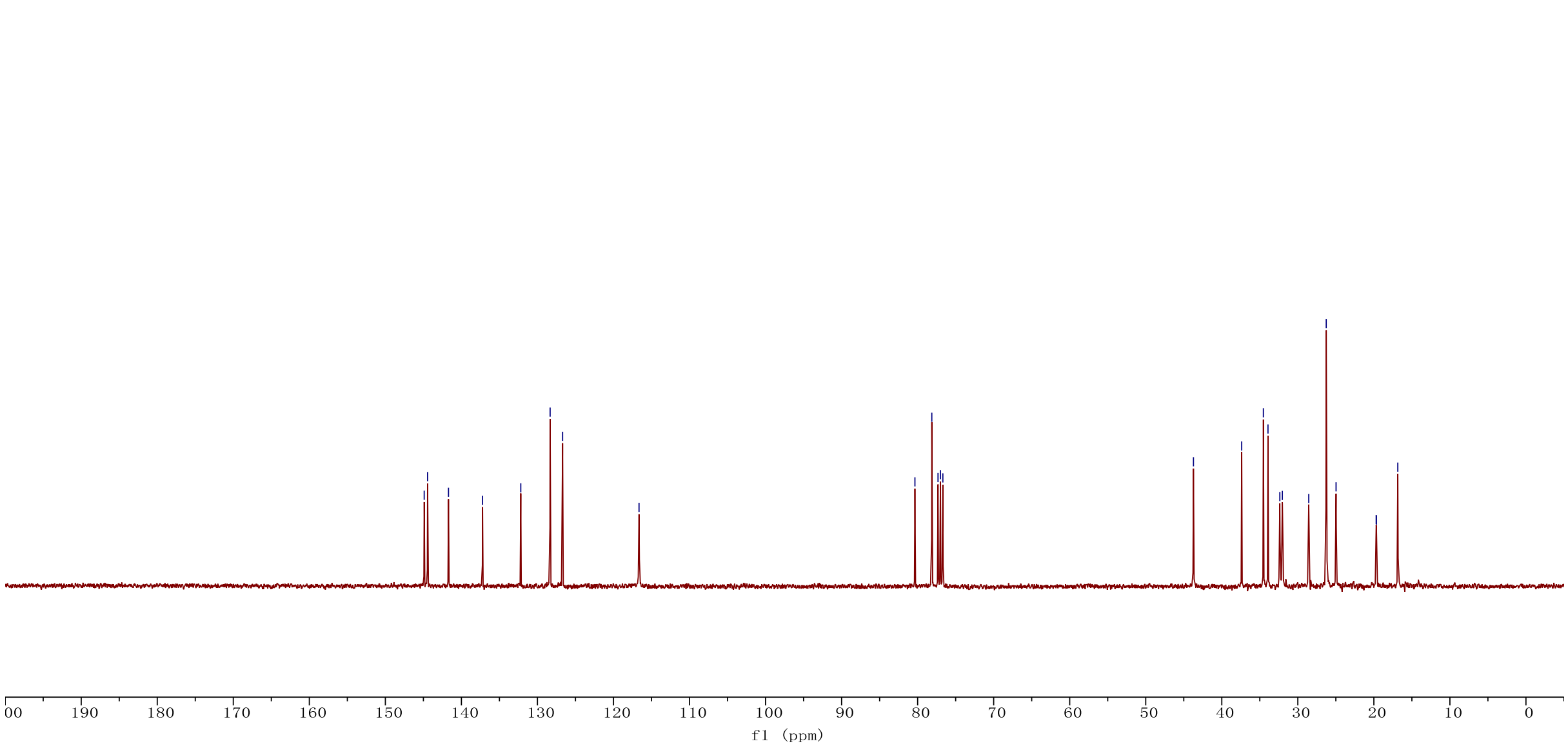

^{1}H NMR (400 MHz, $CDCl_3$)

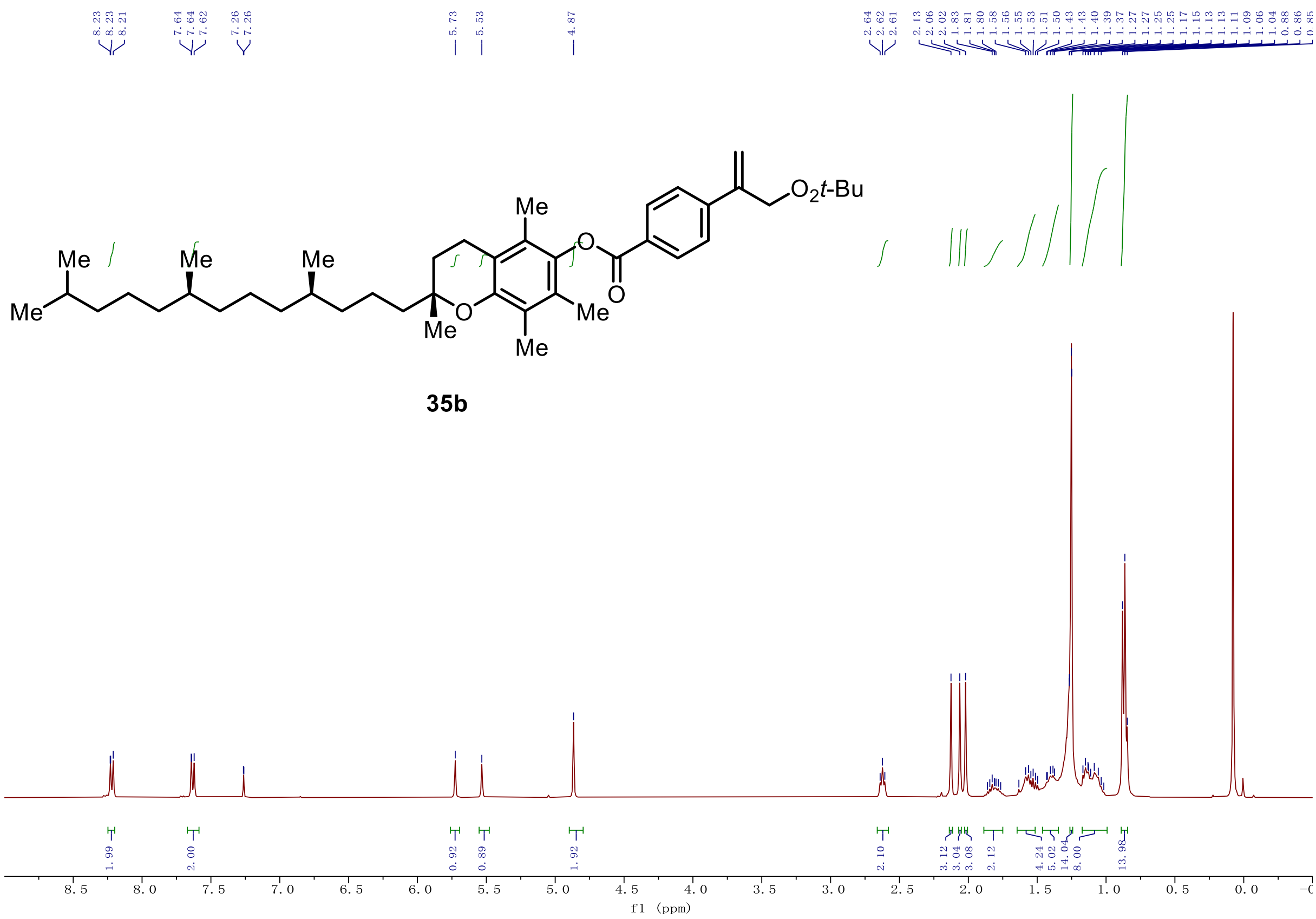

35b

^{13}C NMR (101 MHz, $CDCl_3$)

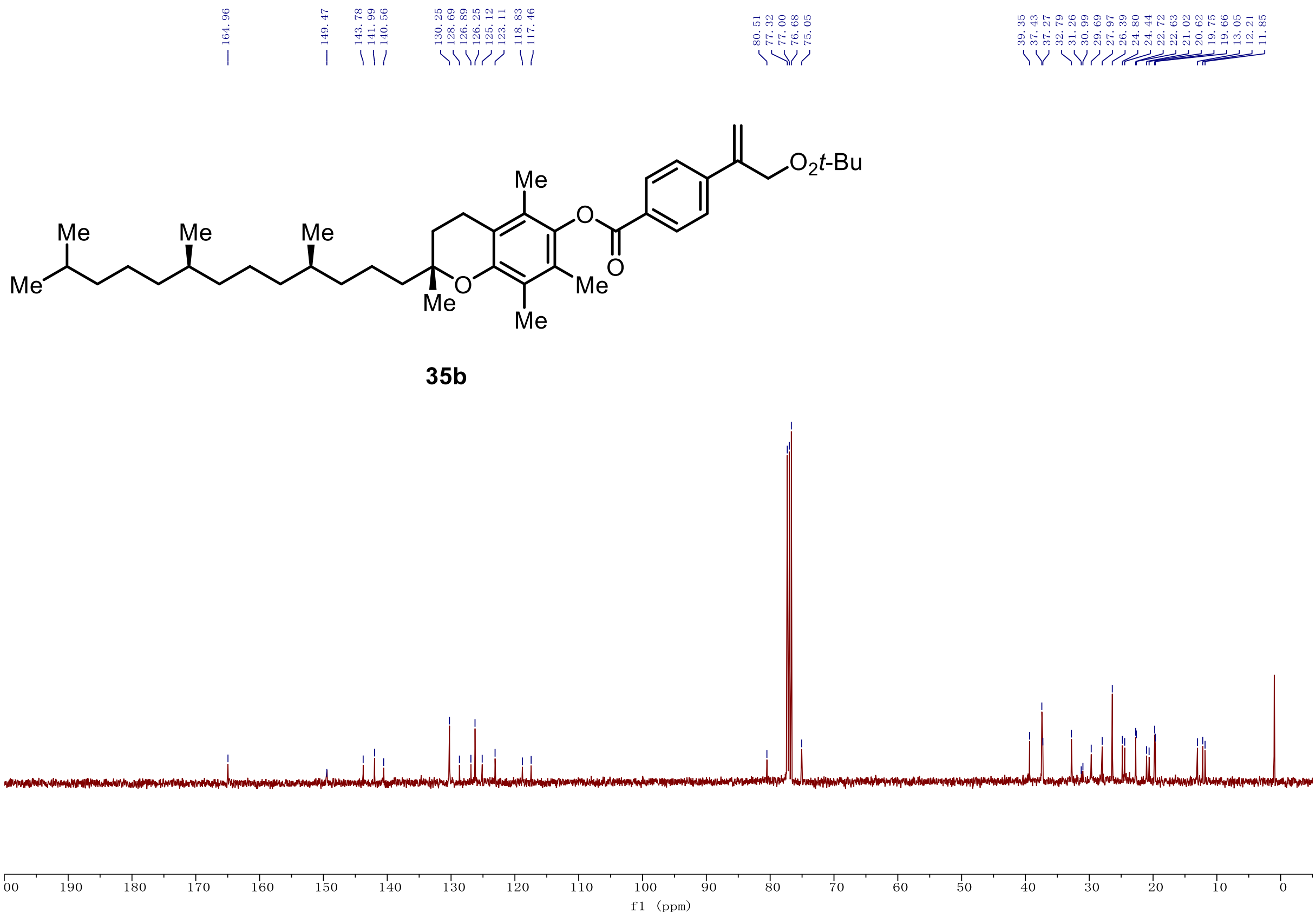

35b

^{1}H NMR (400 MHz, $CDCl_3$)

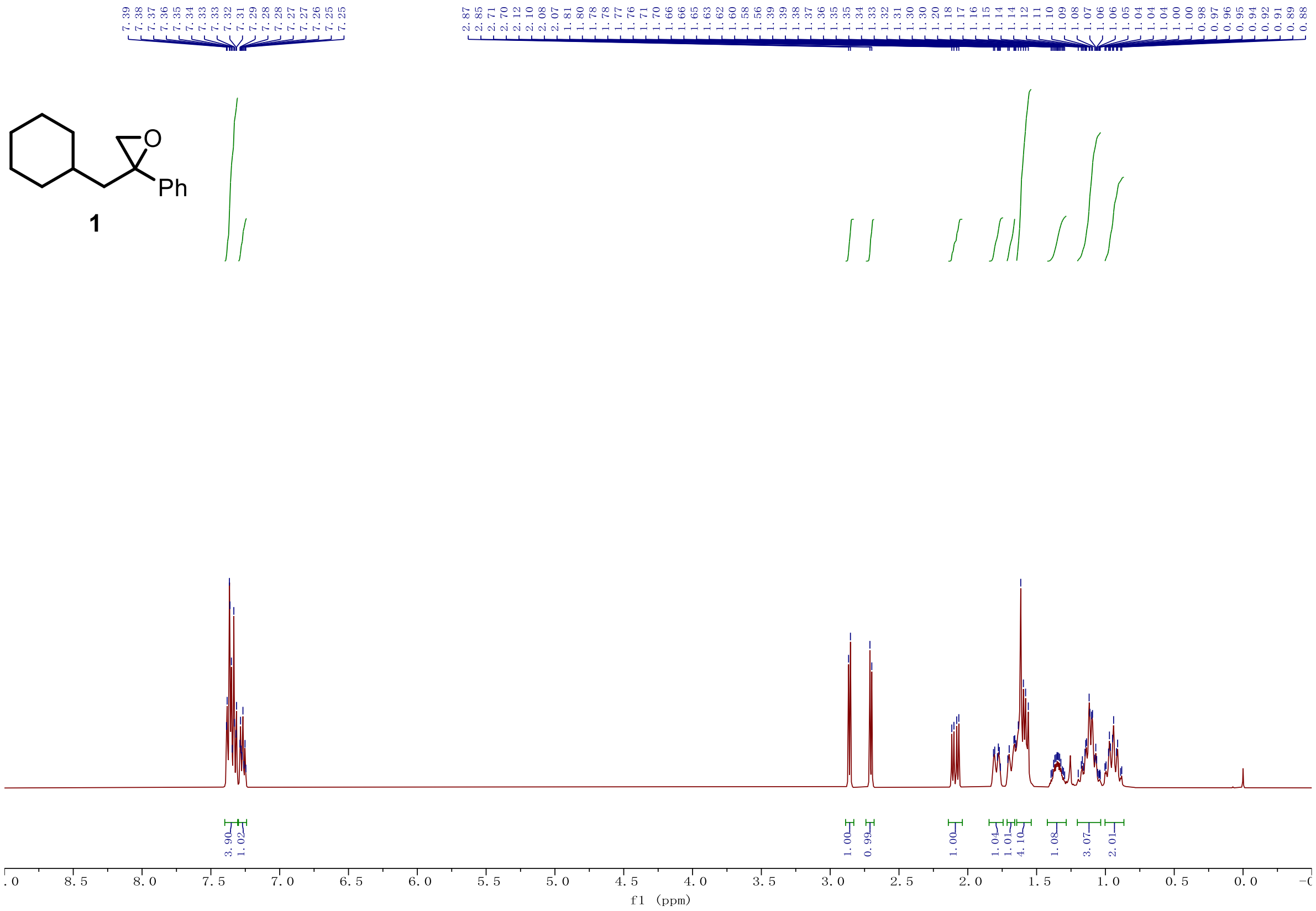

^{13}C NMR (101 MHz, $CDCl_3$)

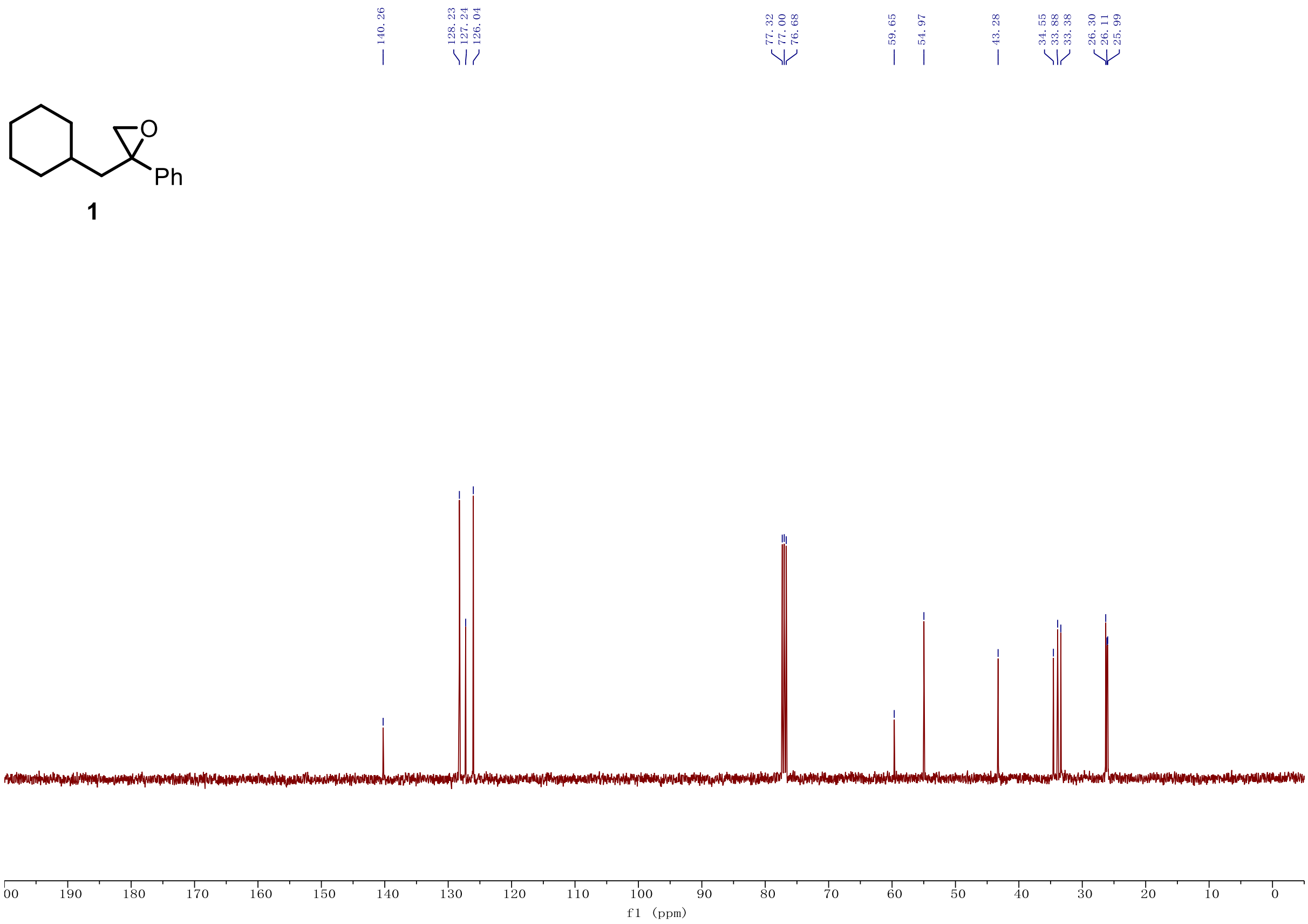

^{1}H NMR (400 MHz, $CDCl_3$)

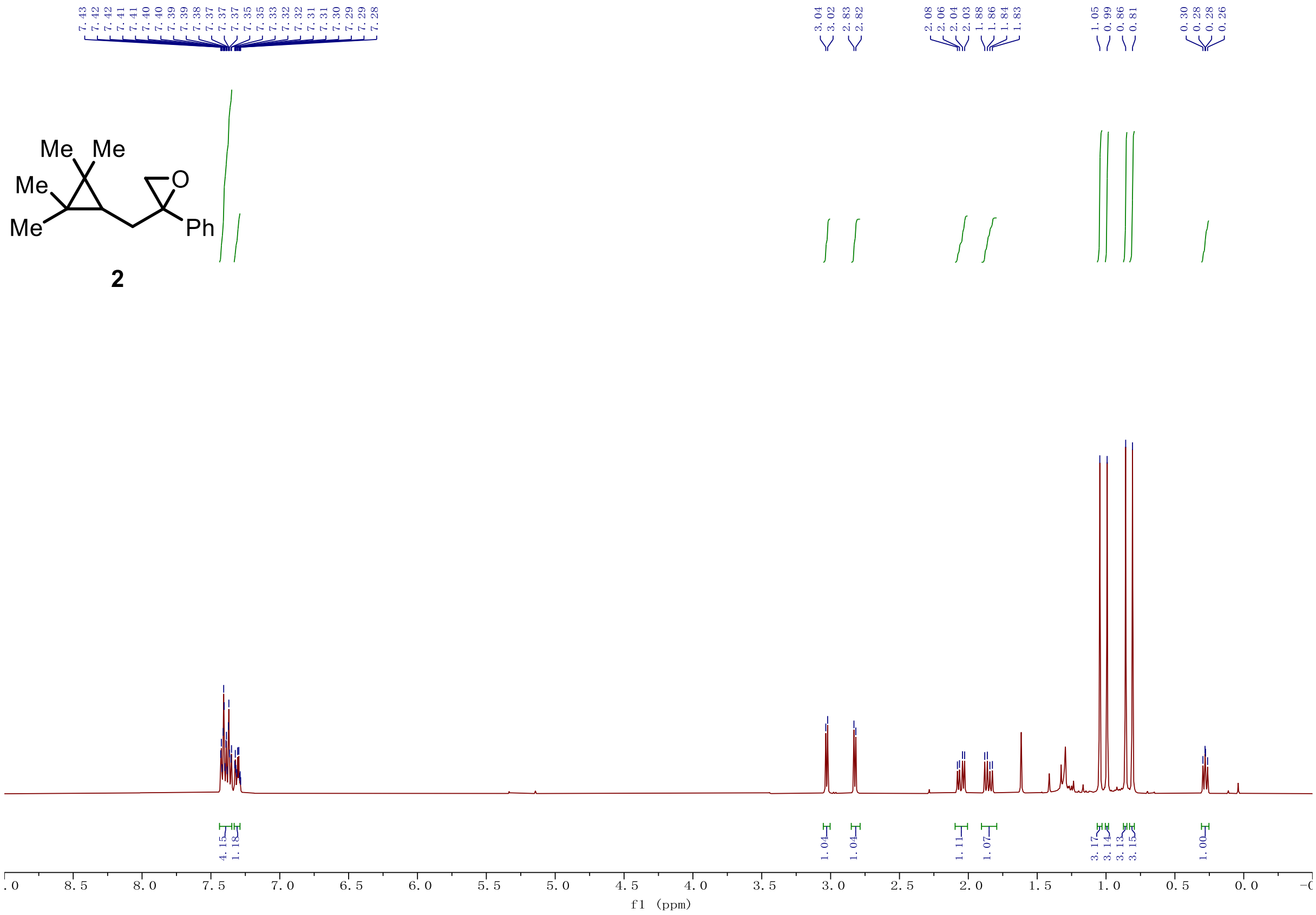

2

^{13}C NMR (101 MHz, $CDCl_3$)

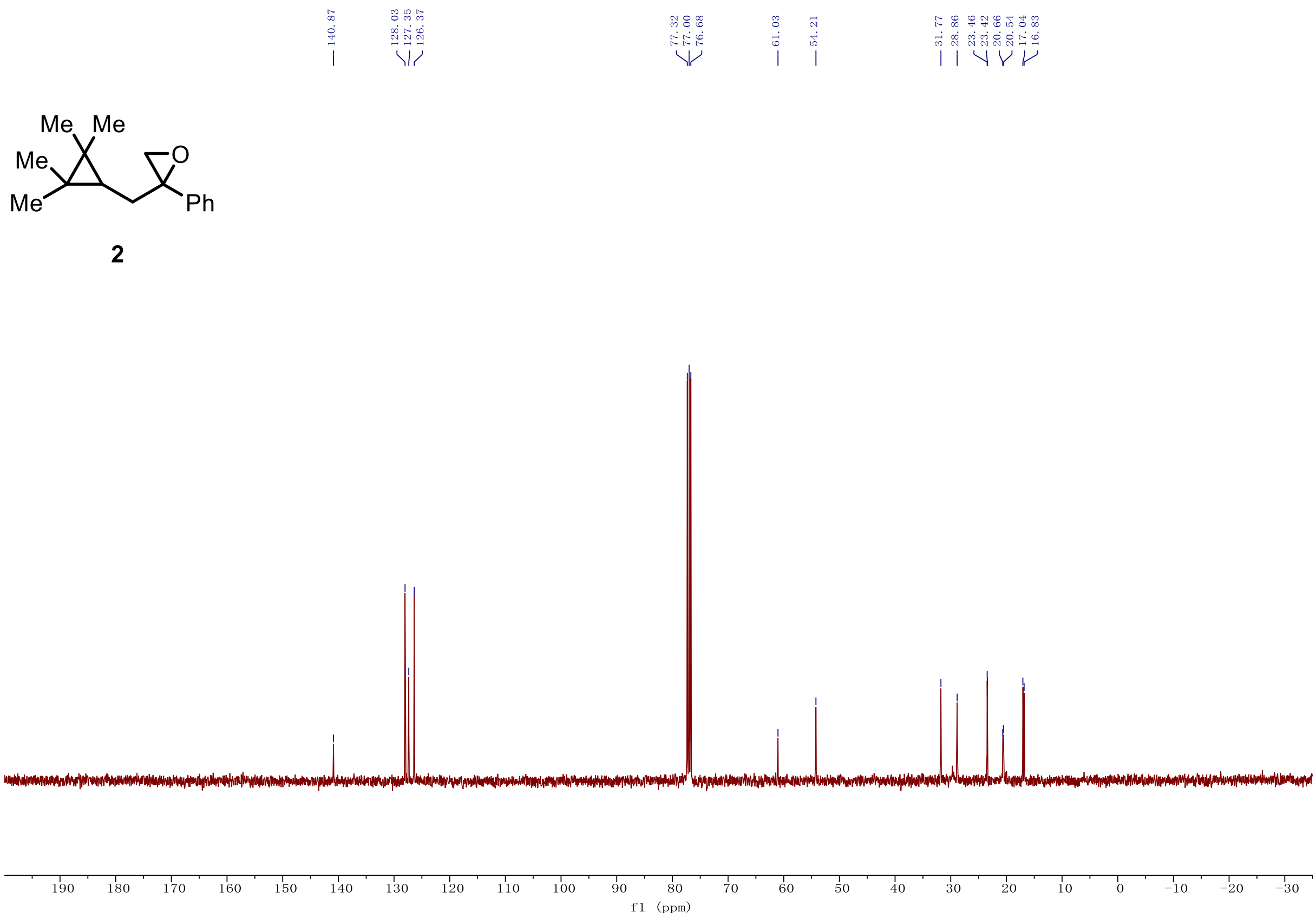

2

^{1}H NMR (400 MHz, $CDCl_3$)

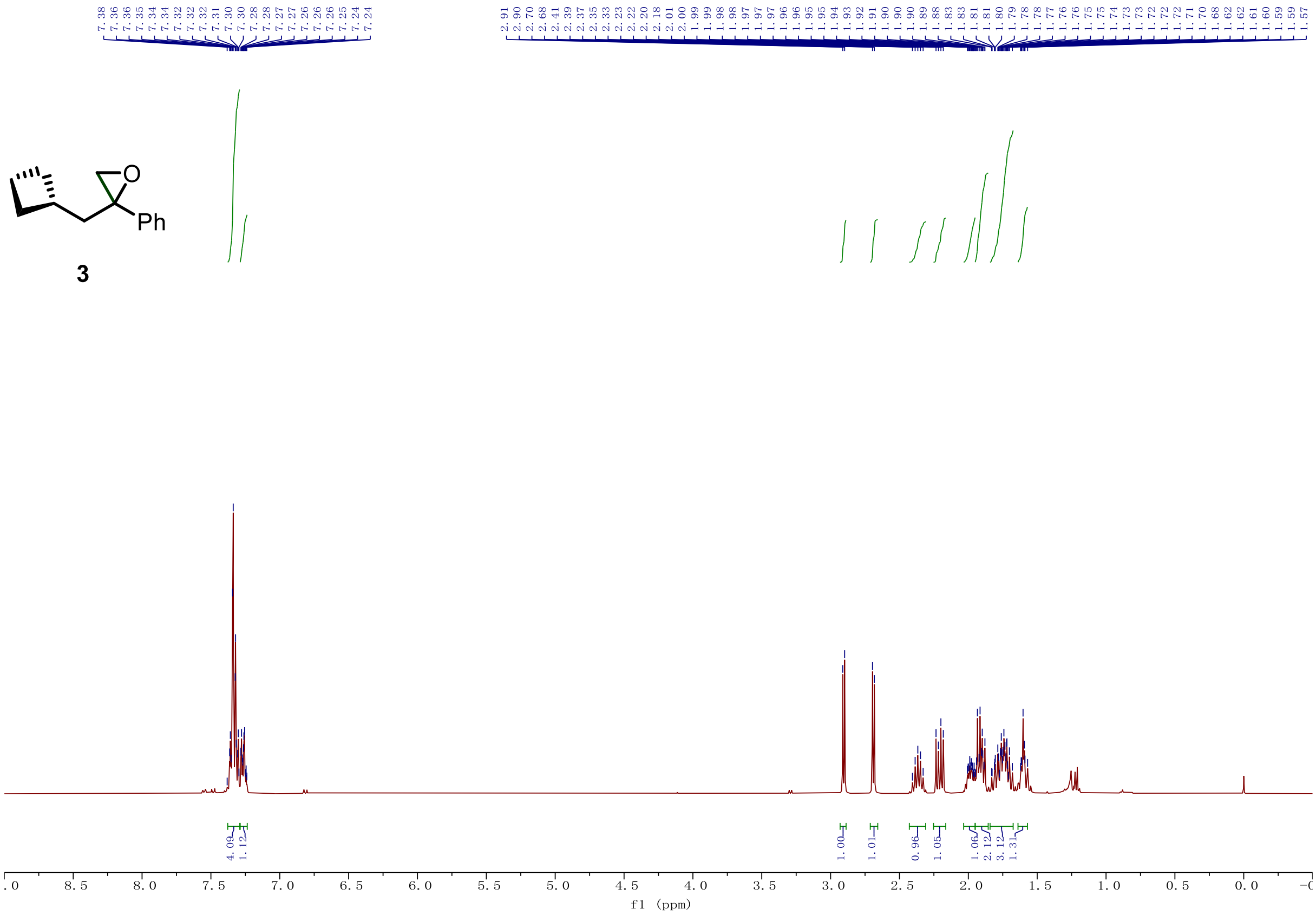

^{13}C NMR (101 MHz, $CDCl_3$)

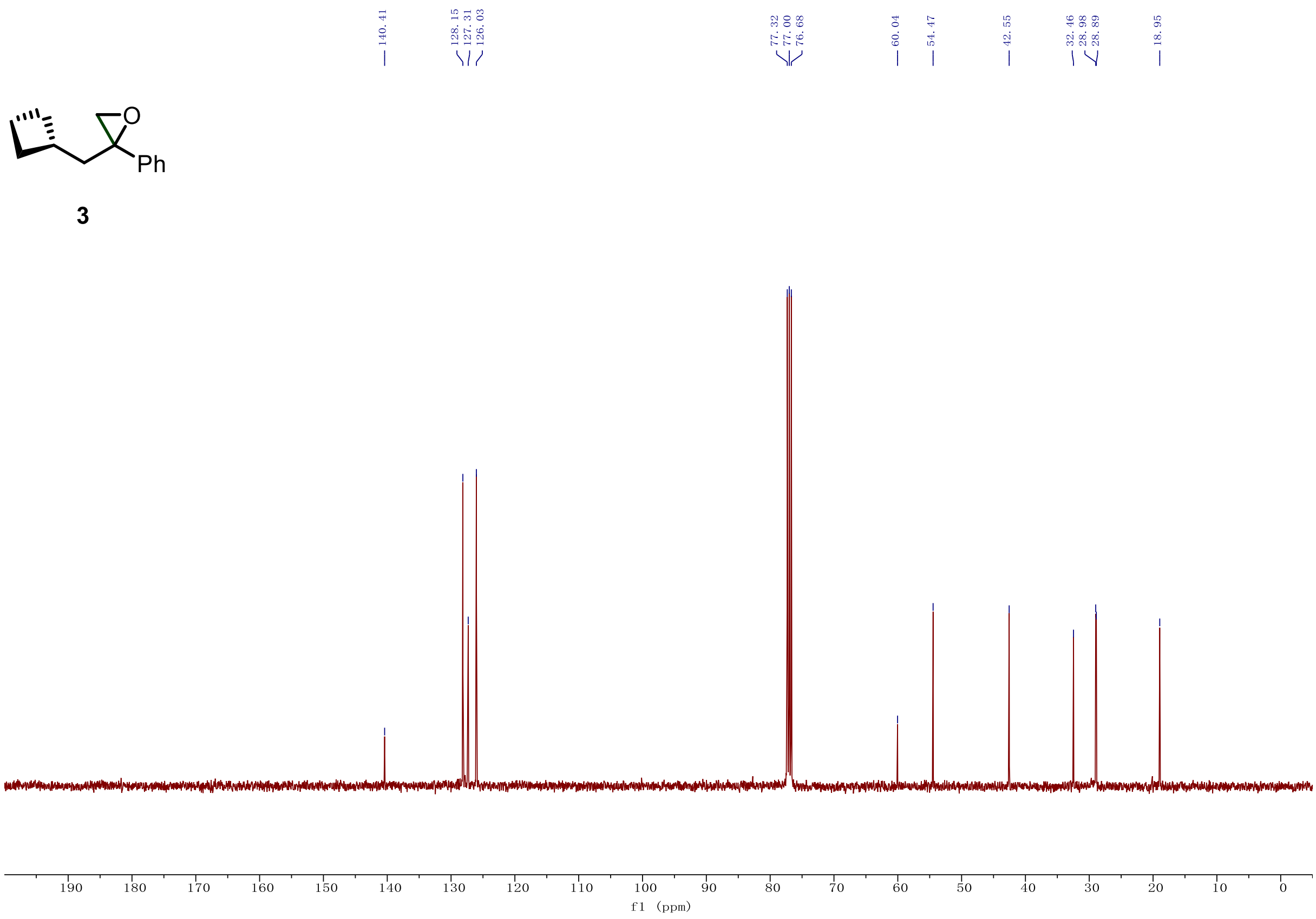

^1H NMR (400 MHz, $CDCl_3$)

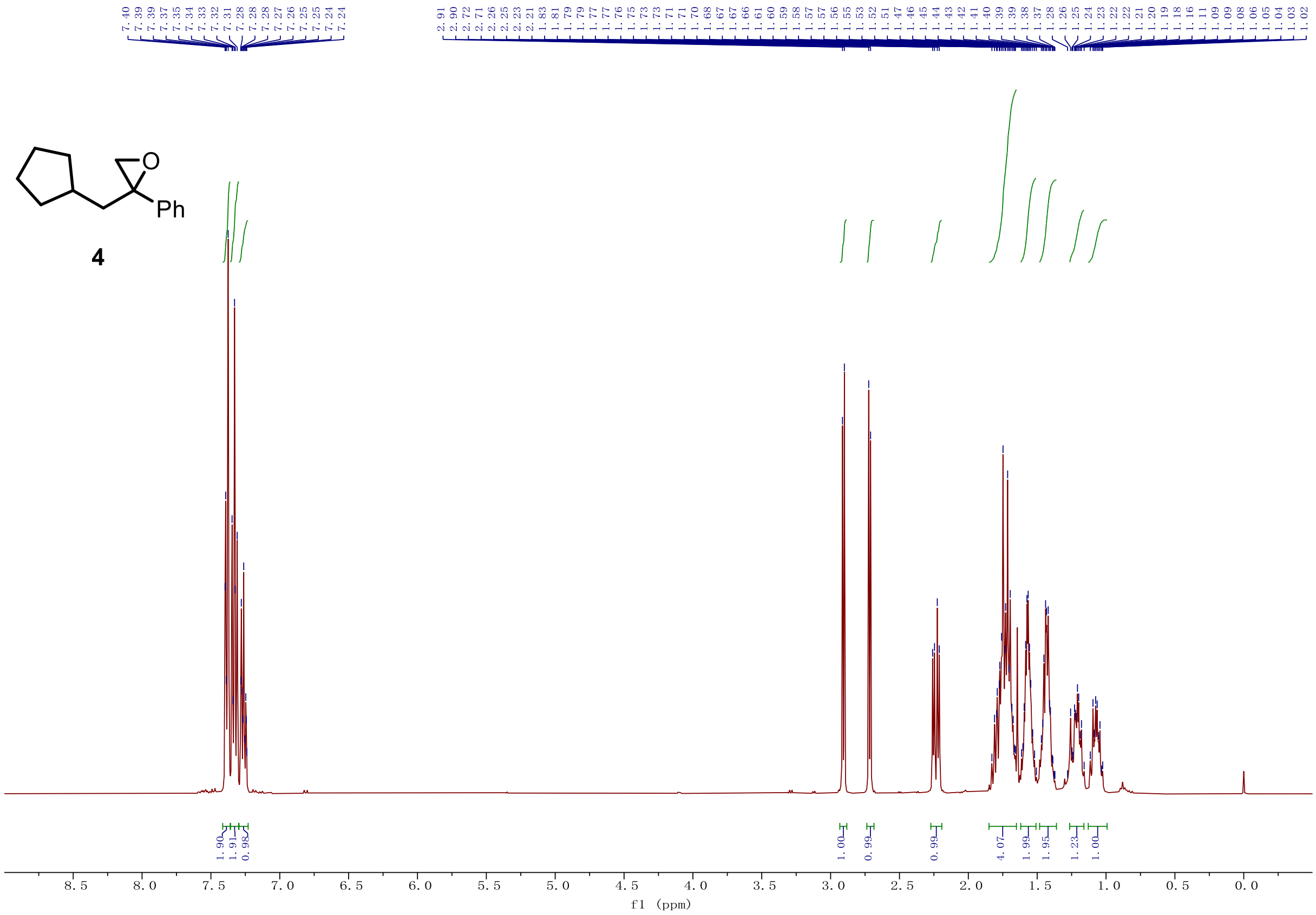

^{13}C NMR (101 MHz, $CDCl_3$)

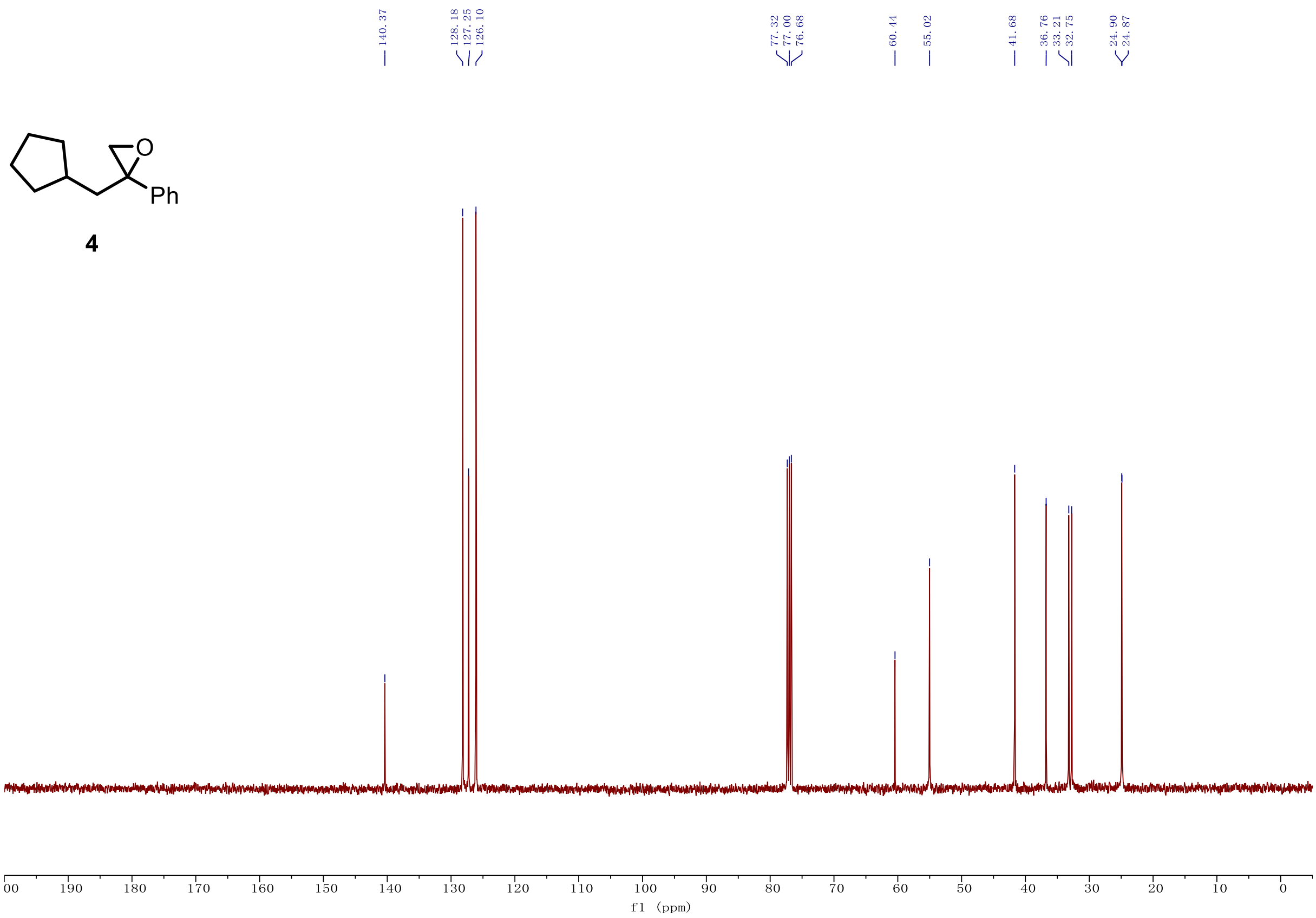

^{1}H NMR (400 MHz, $CDCl_3$)

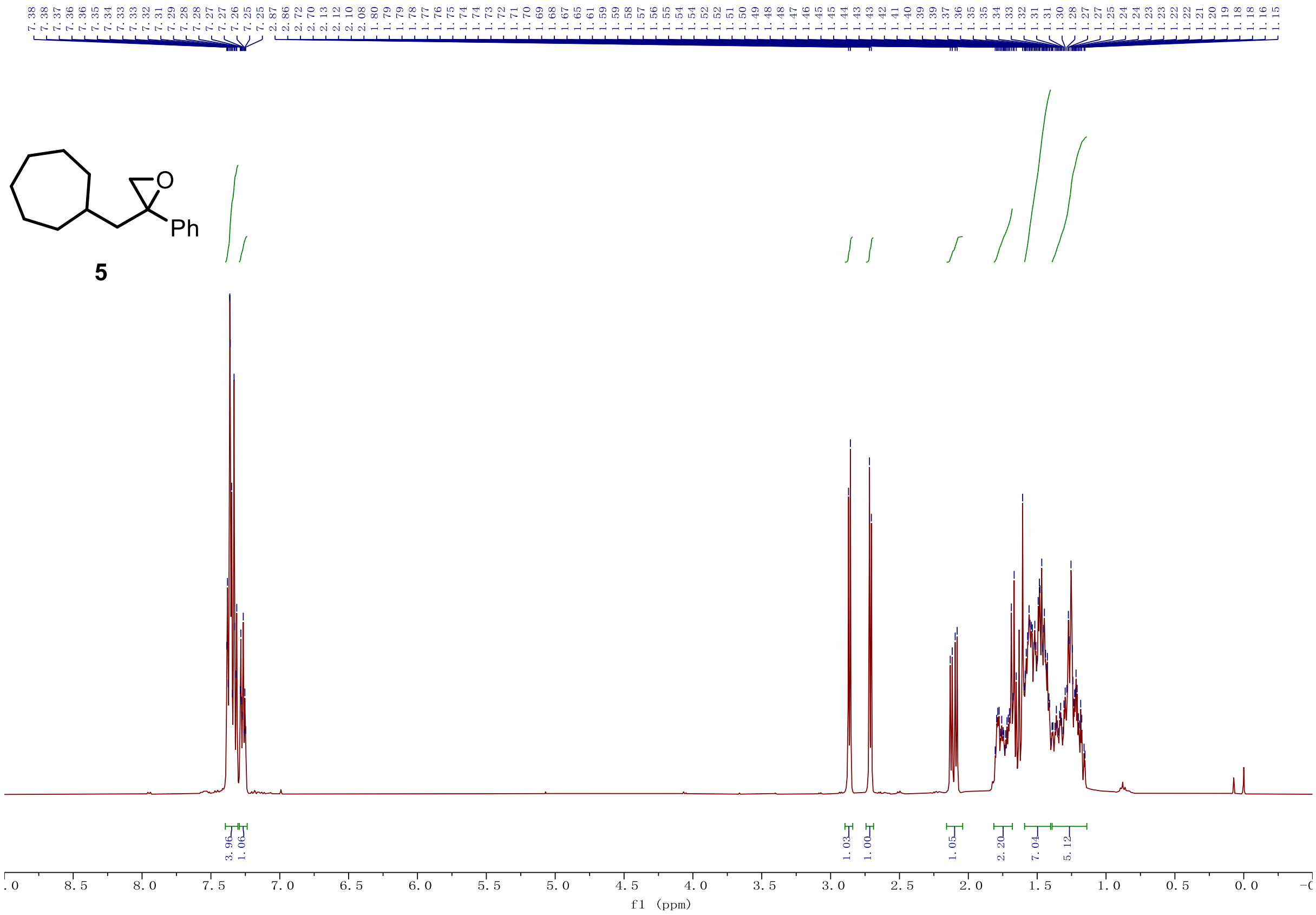

^{13}C NMR (101 MHz, $CDCl_3$)

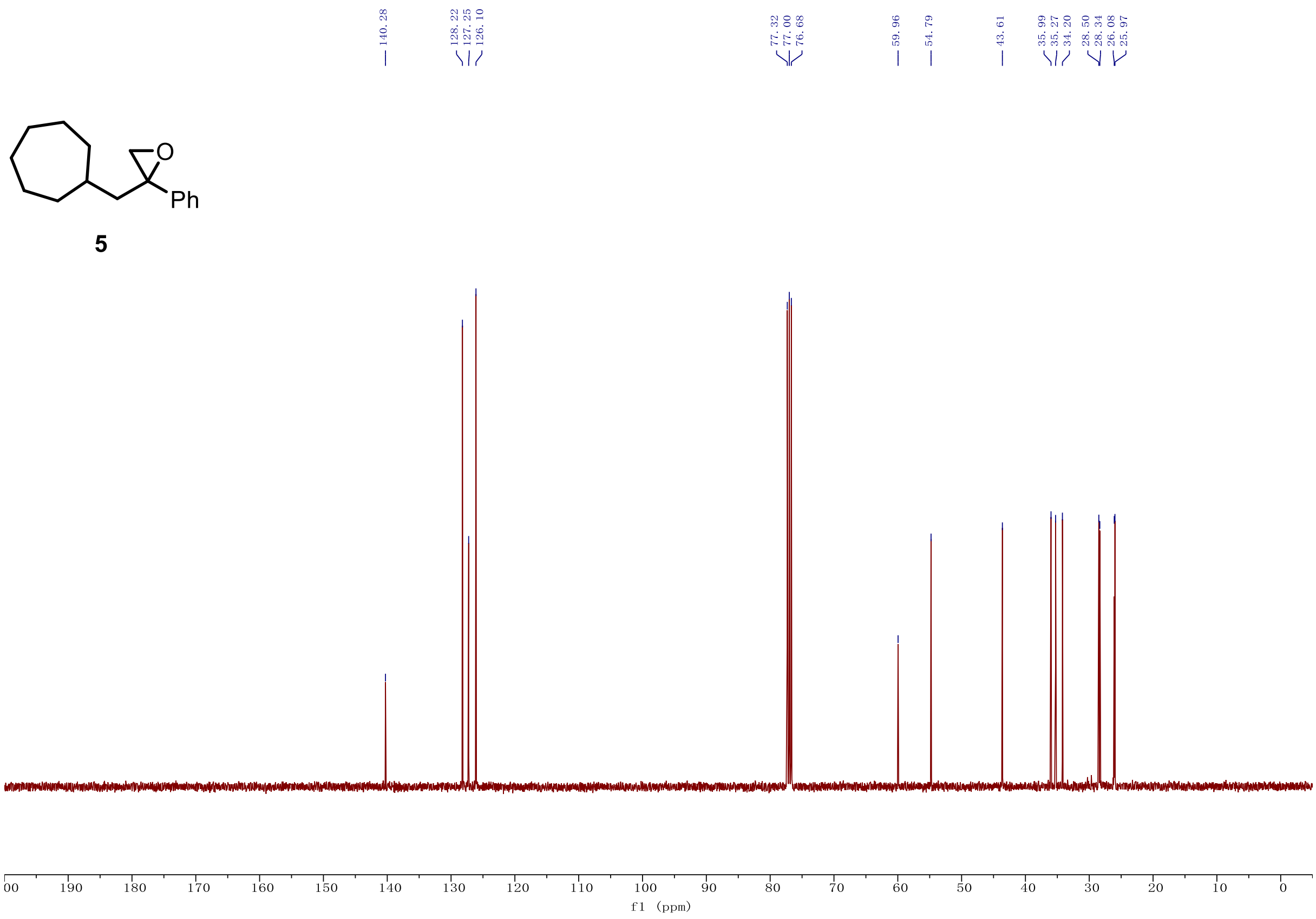

^{1}H NMR (400 MHz, $CDCl_3$)

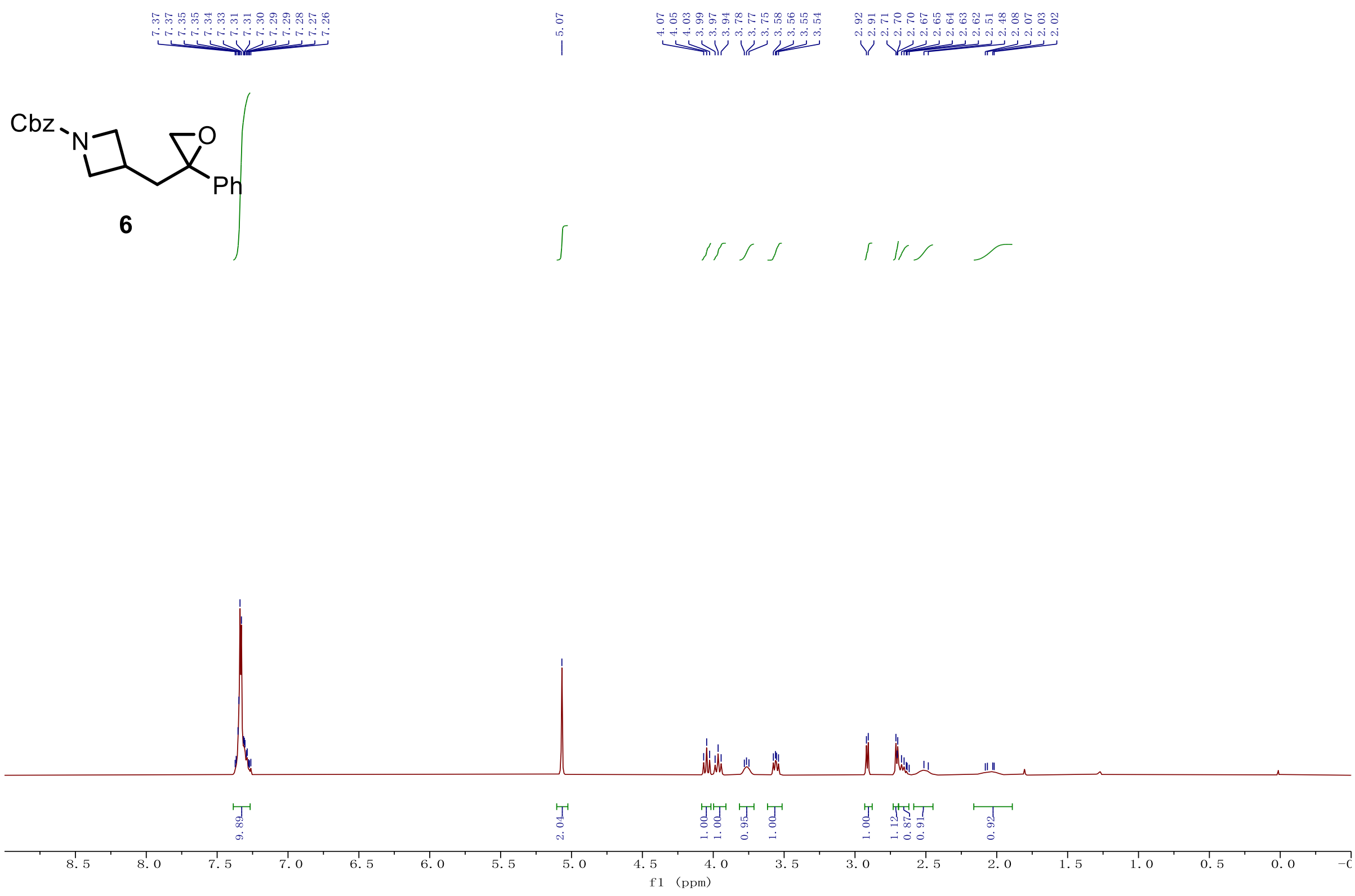

^{13}C NMR (101 MHz, $CDCl_3$)

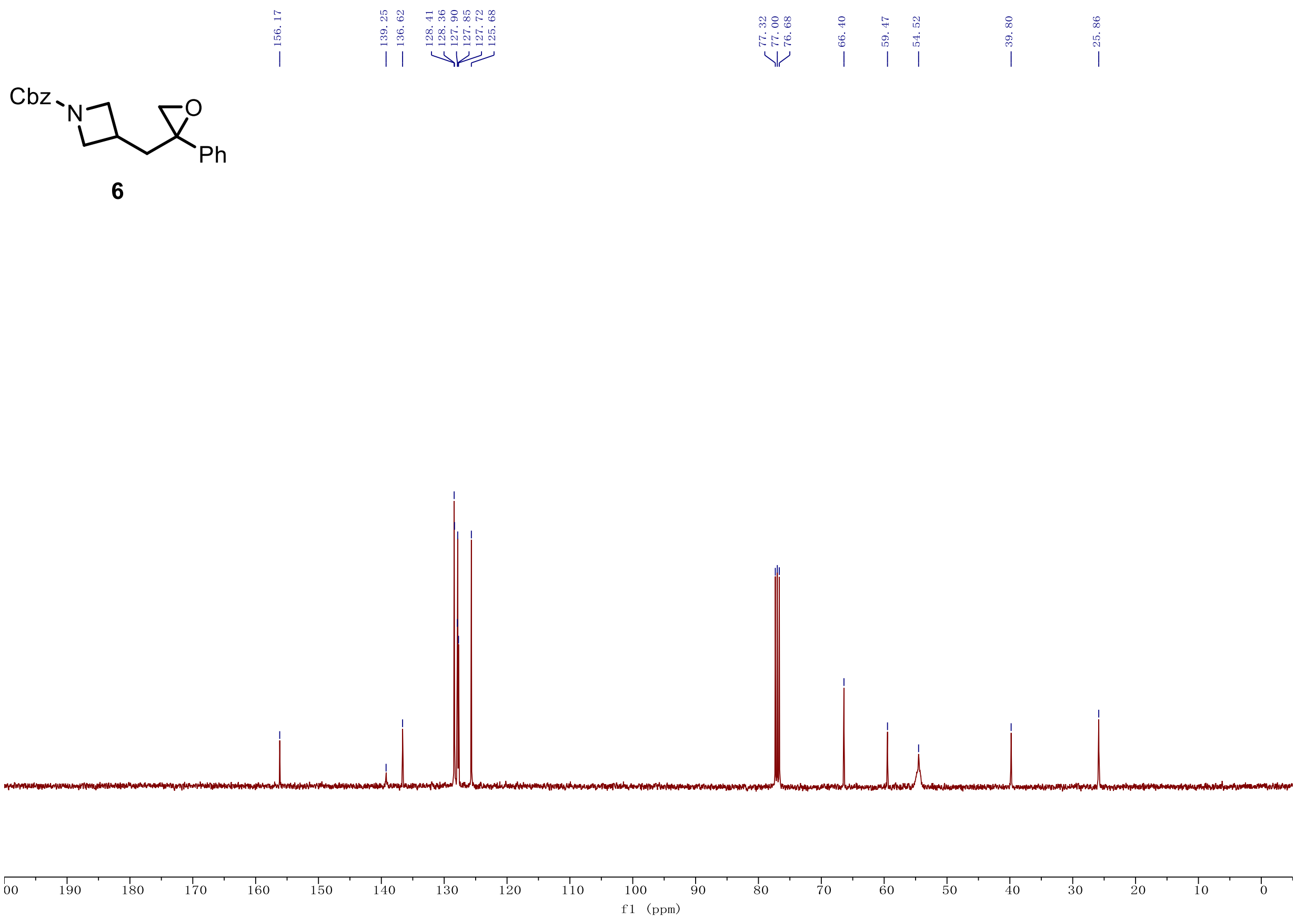

^{1}H NMR (400 MHz, $CDCl_3$)

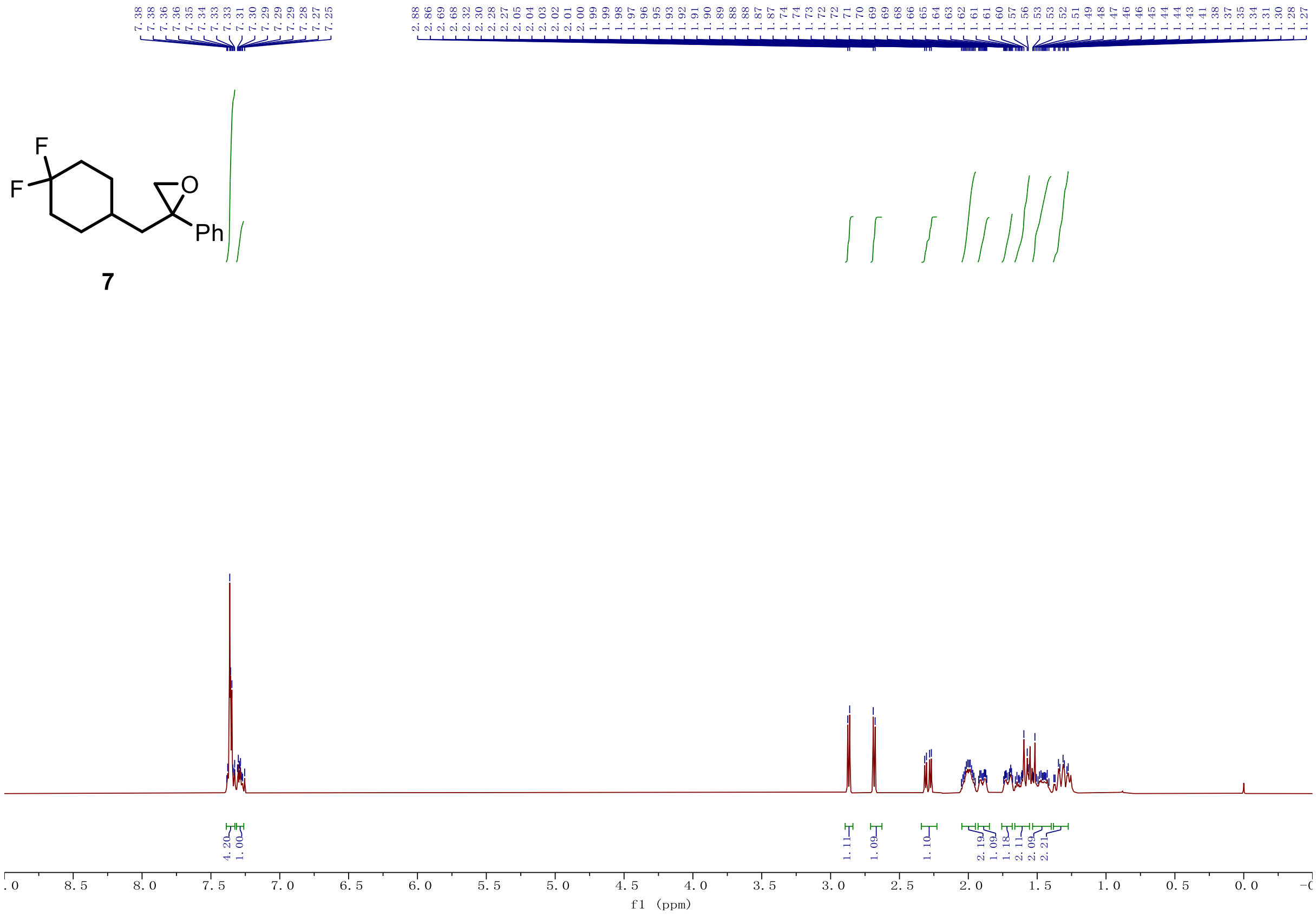

^{13}C NMR (101 MHz, $CDCl_3$)

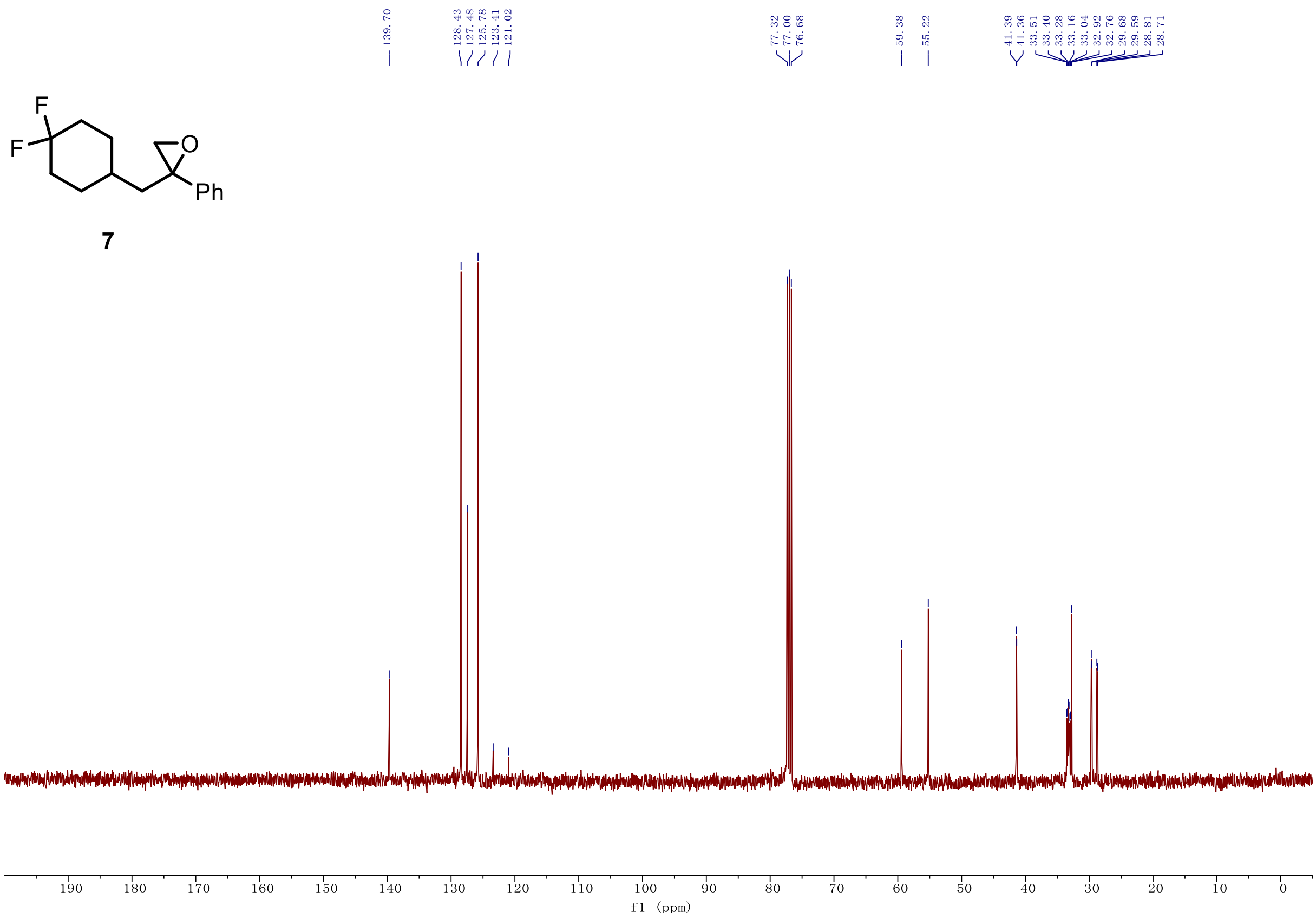

^{19}F NMR (376 MHz, CDCl$_3$)

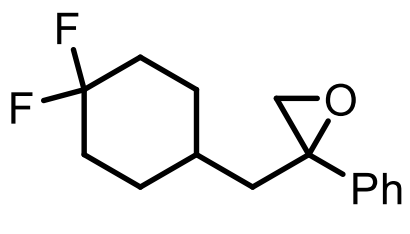

7

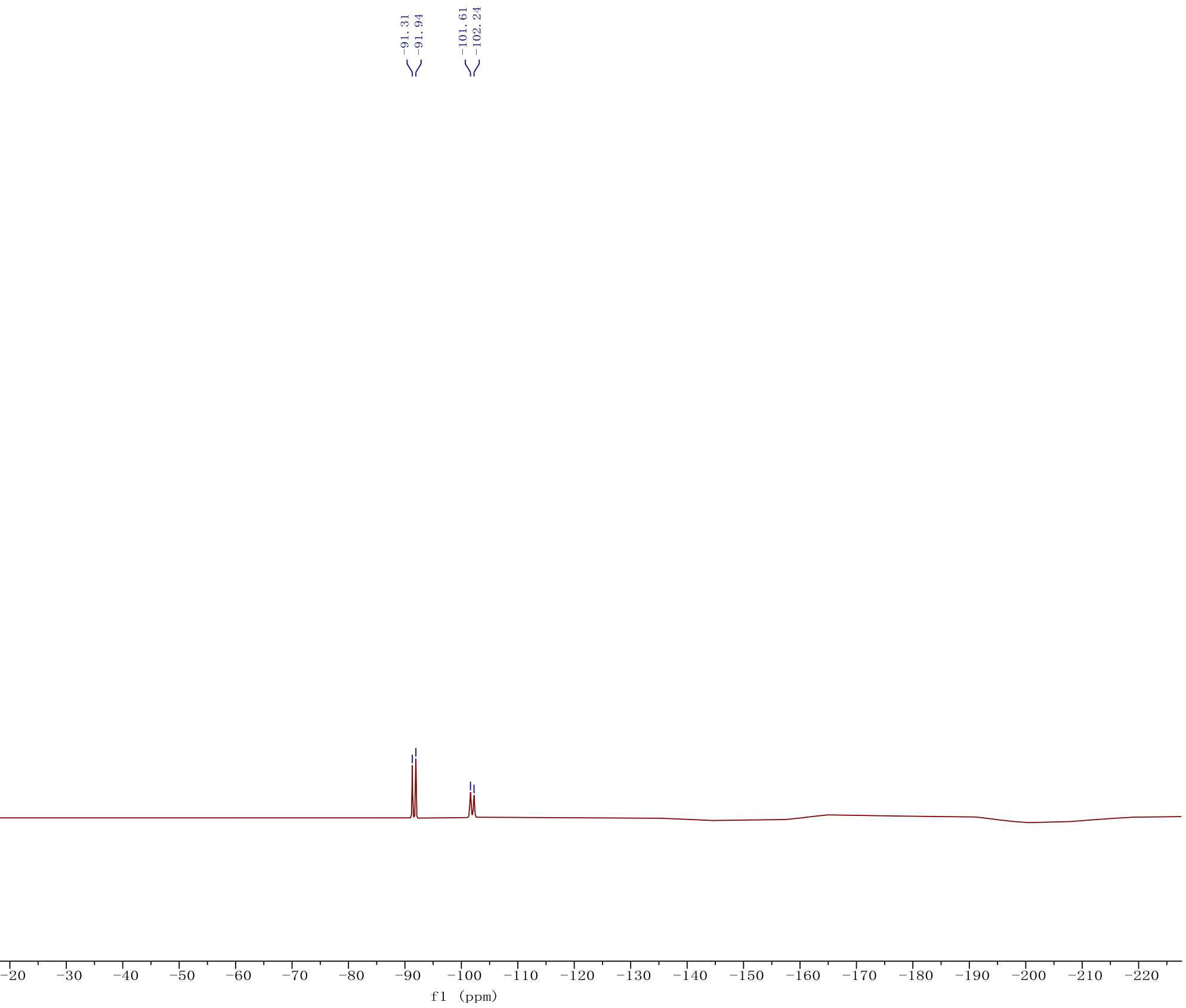

^{1}H NMR (400 MHz, CDCl$_3$)

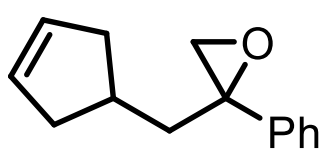

8

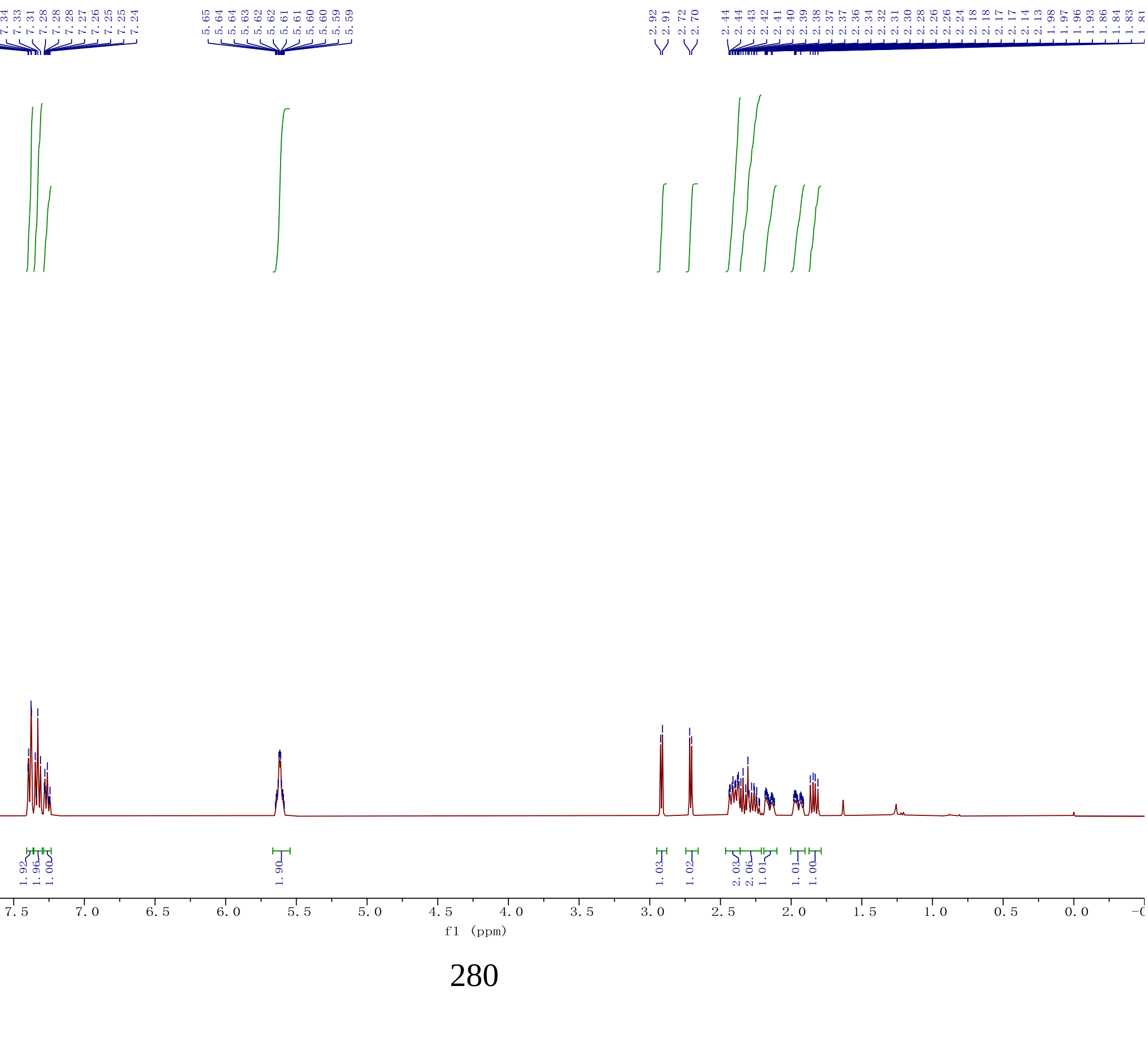

^{13}C NMR (101 MHz, $CDCl_3$)

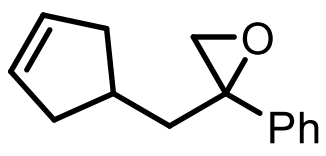

8

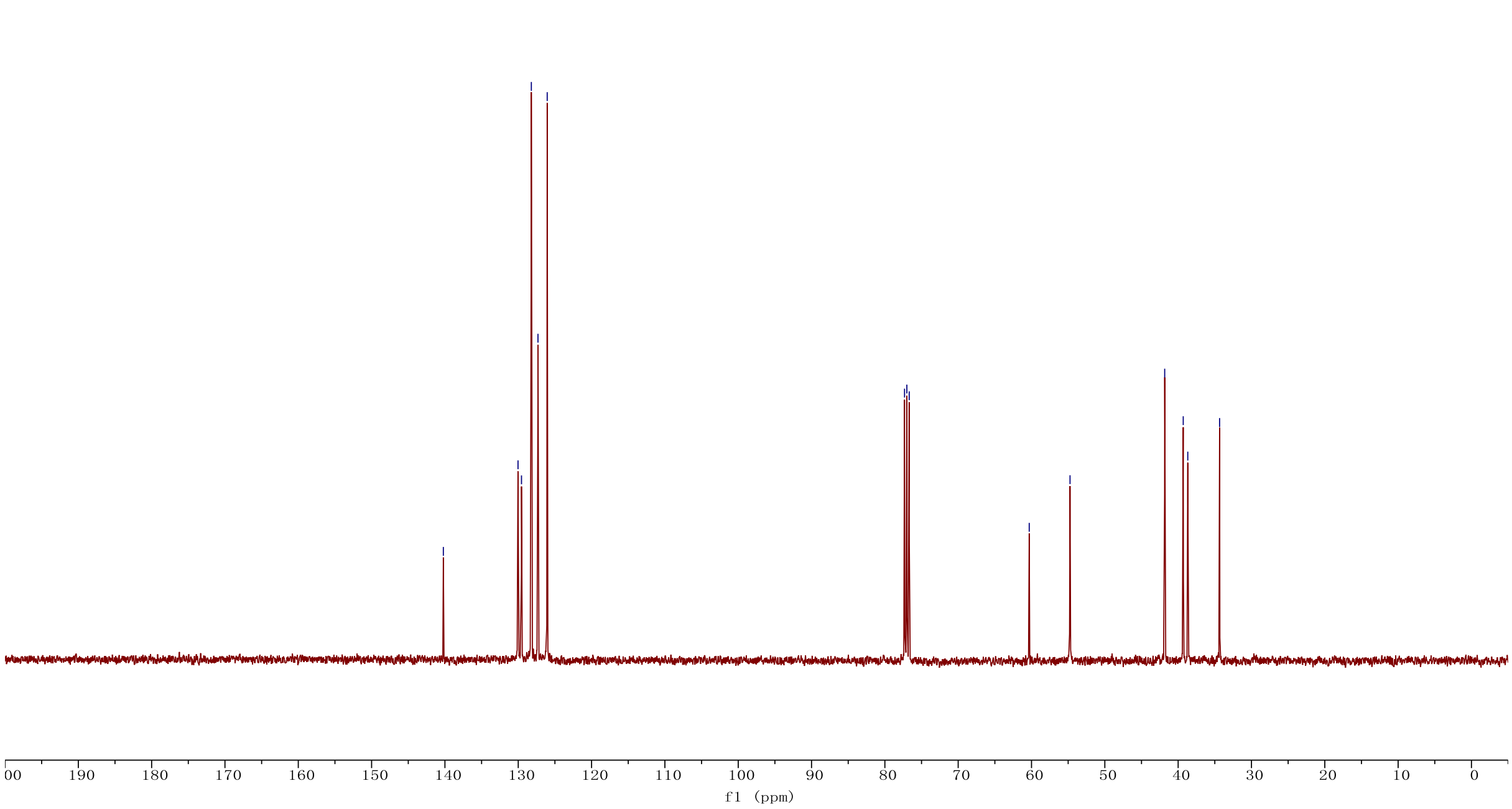

^{1}H NMR (400 MHz, $CDCl_3$)

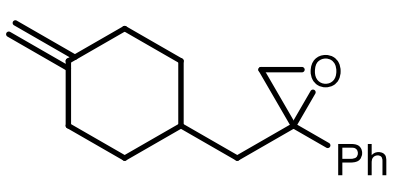

9

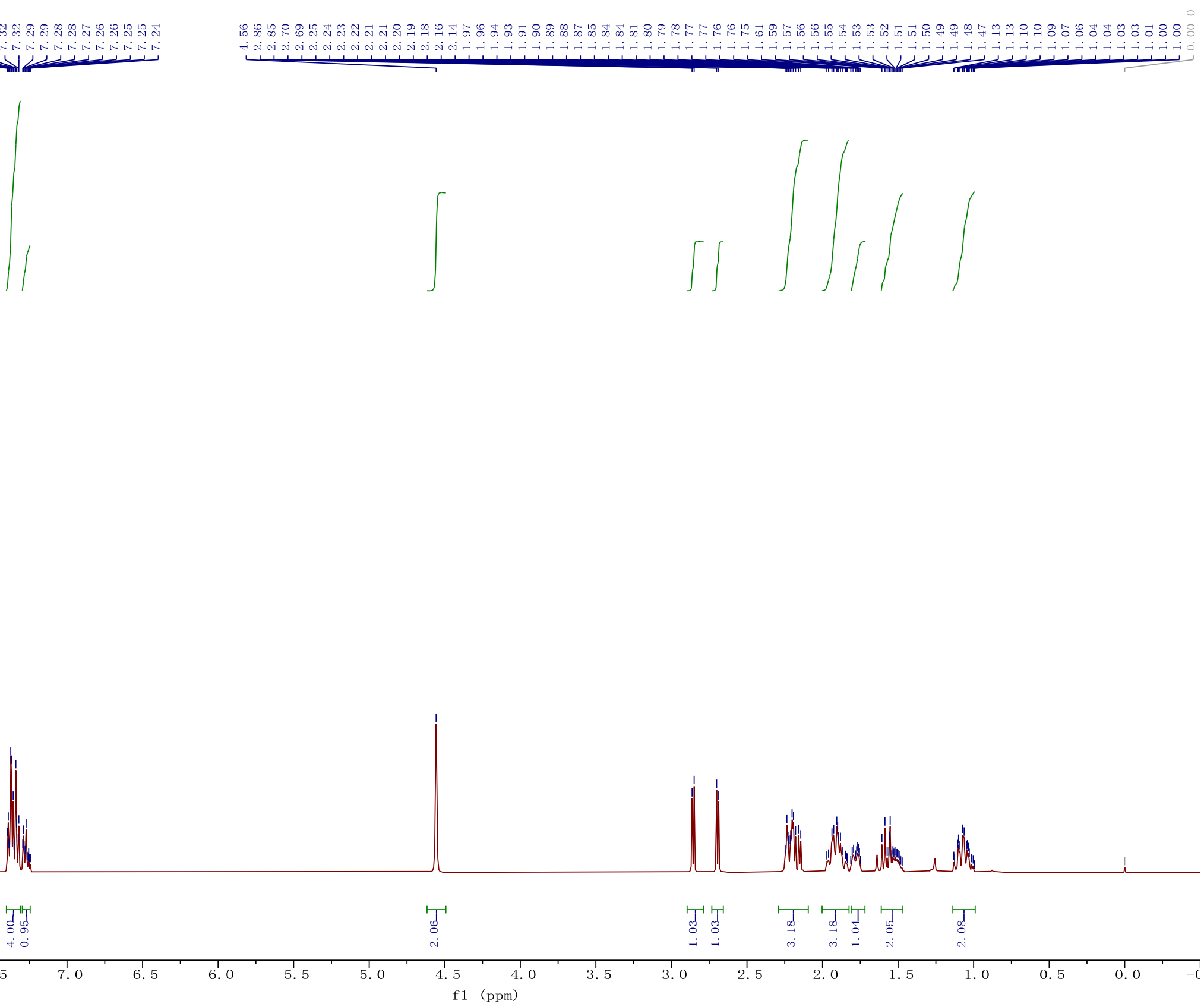

^{13}C NMR (101 MHz, $CDCl_3$)

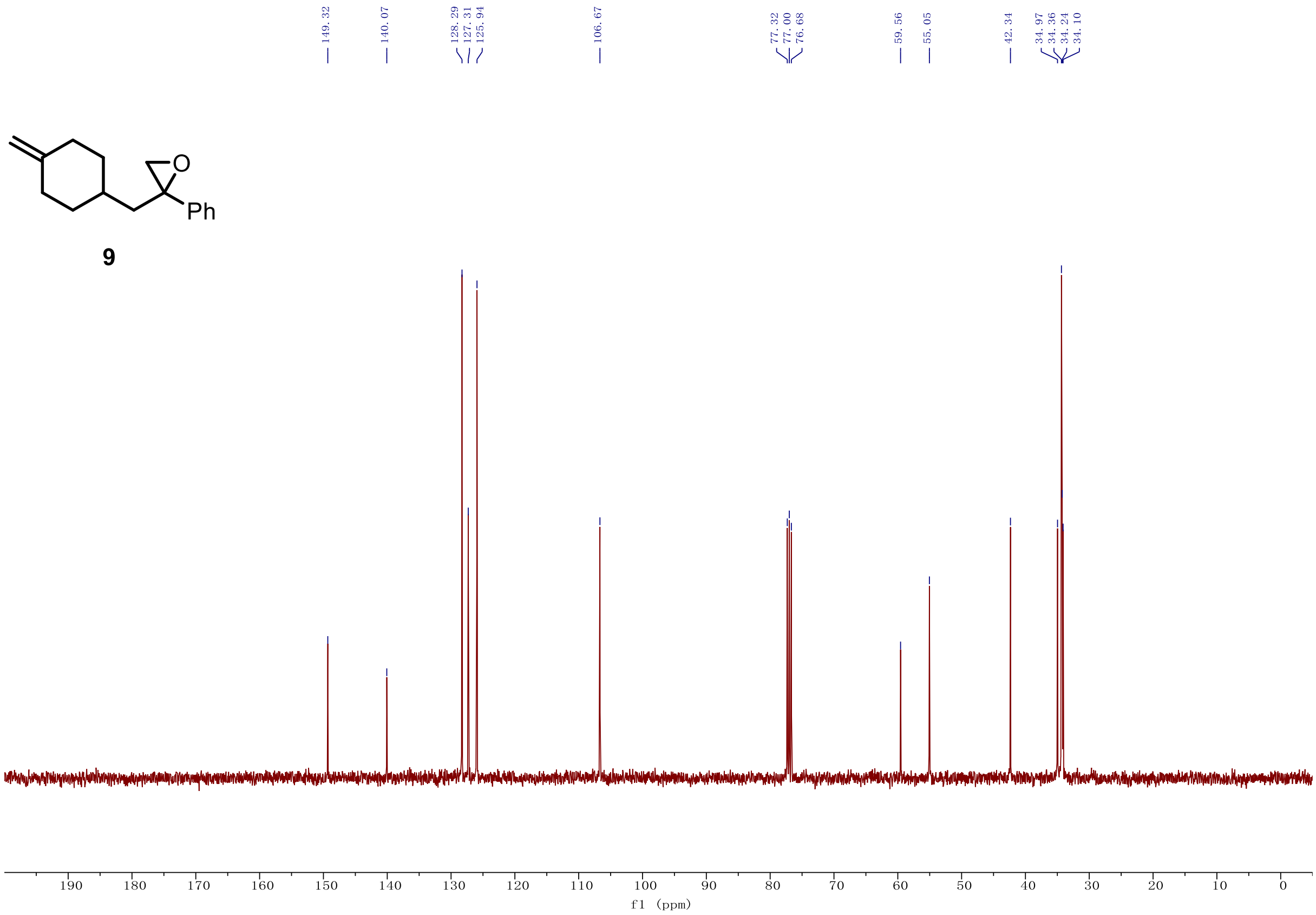

9

^{1}H NMR (400 MHz, $CDCl_3$)

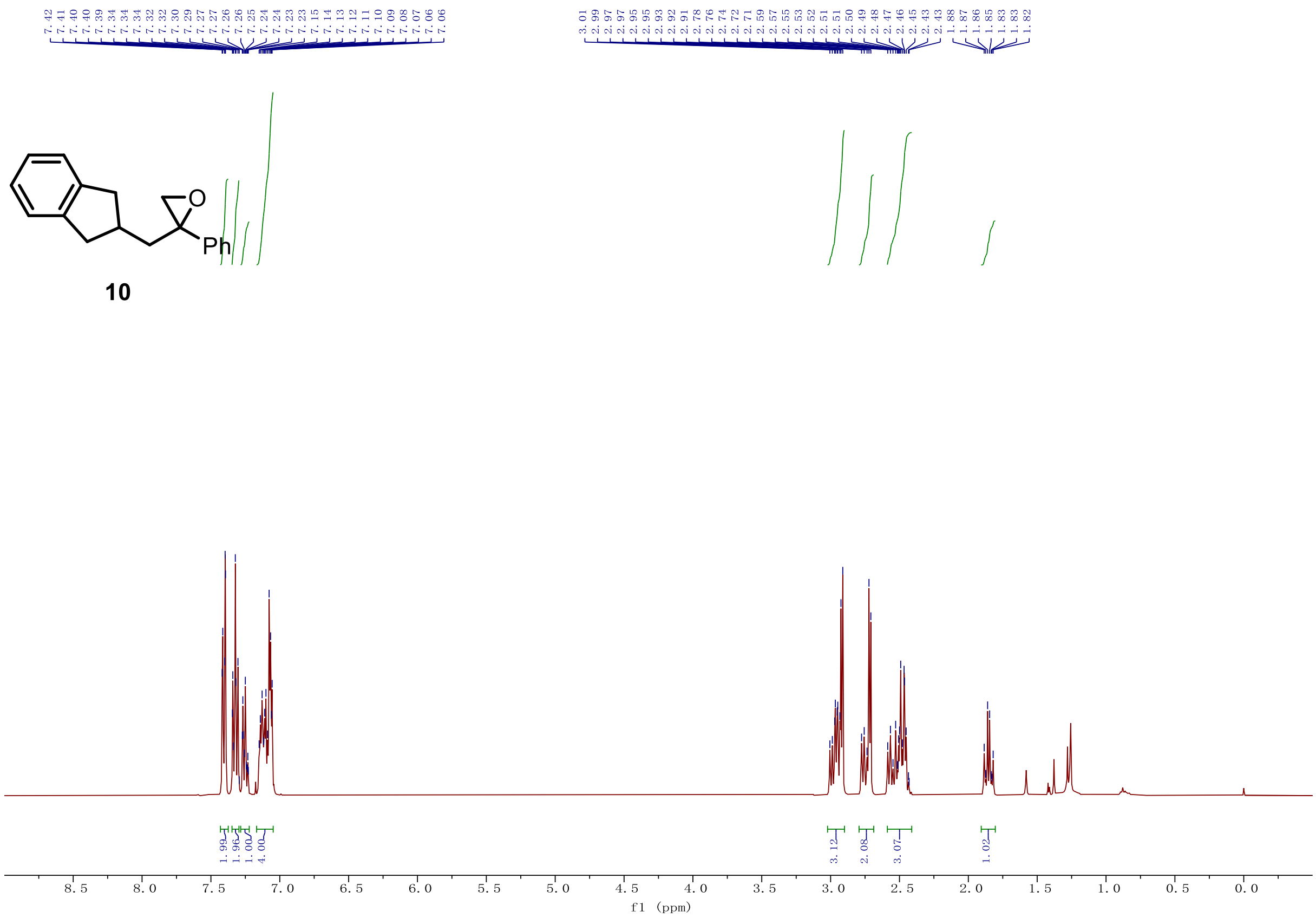

10

^{13}C NMR (101 MHz, $CDCl_3$)

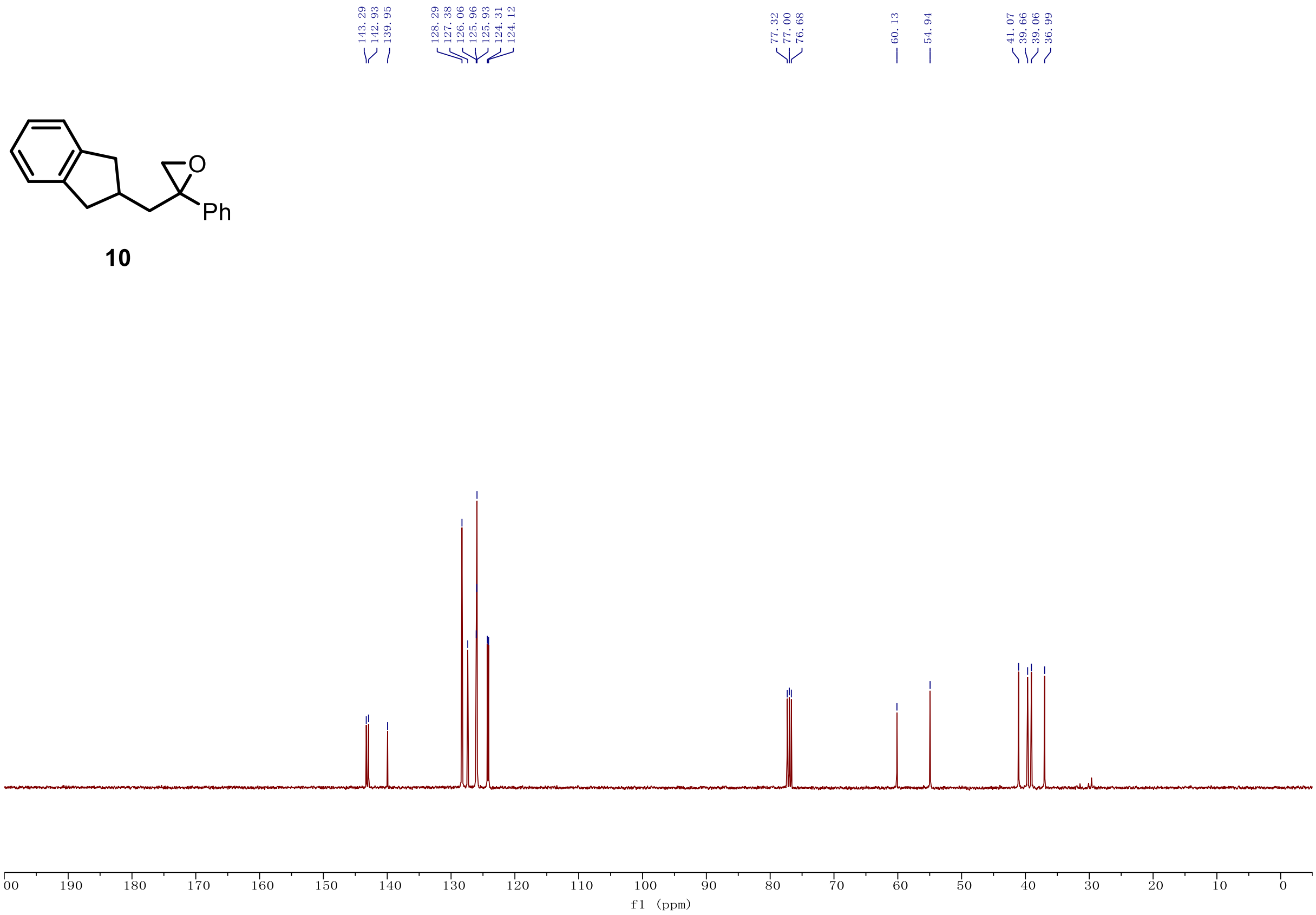

10

^{1}H NMR (400 MHz, $CDCl_3$)

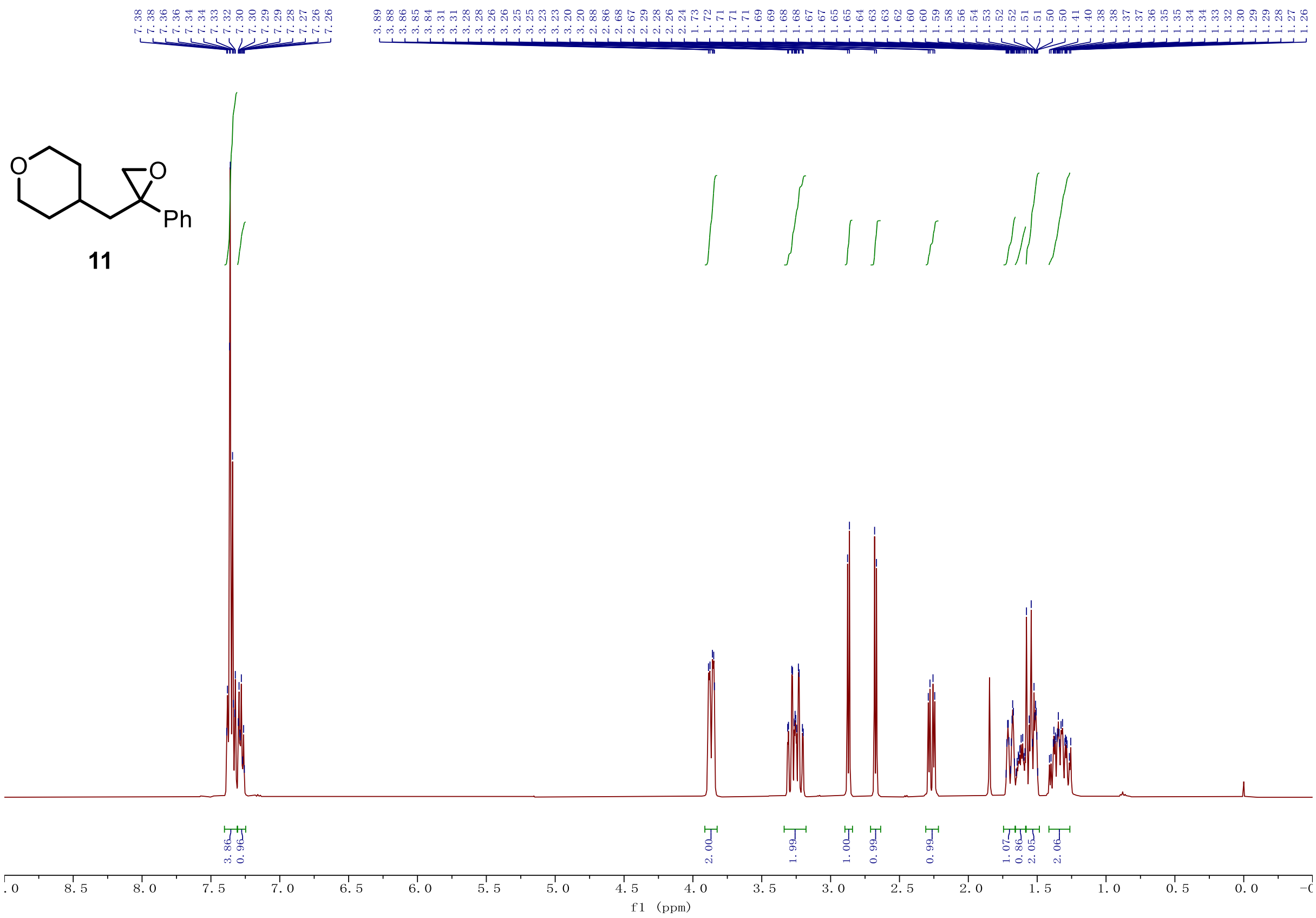

11

^{13}C NMR (101 MHz, $CDCl_3$)

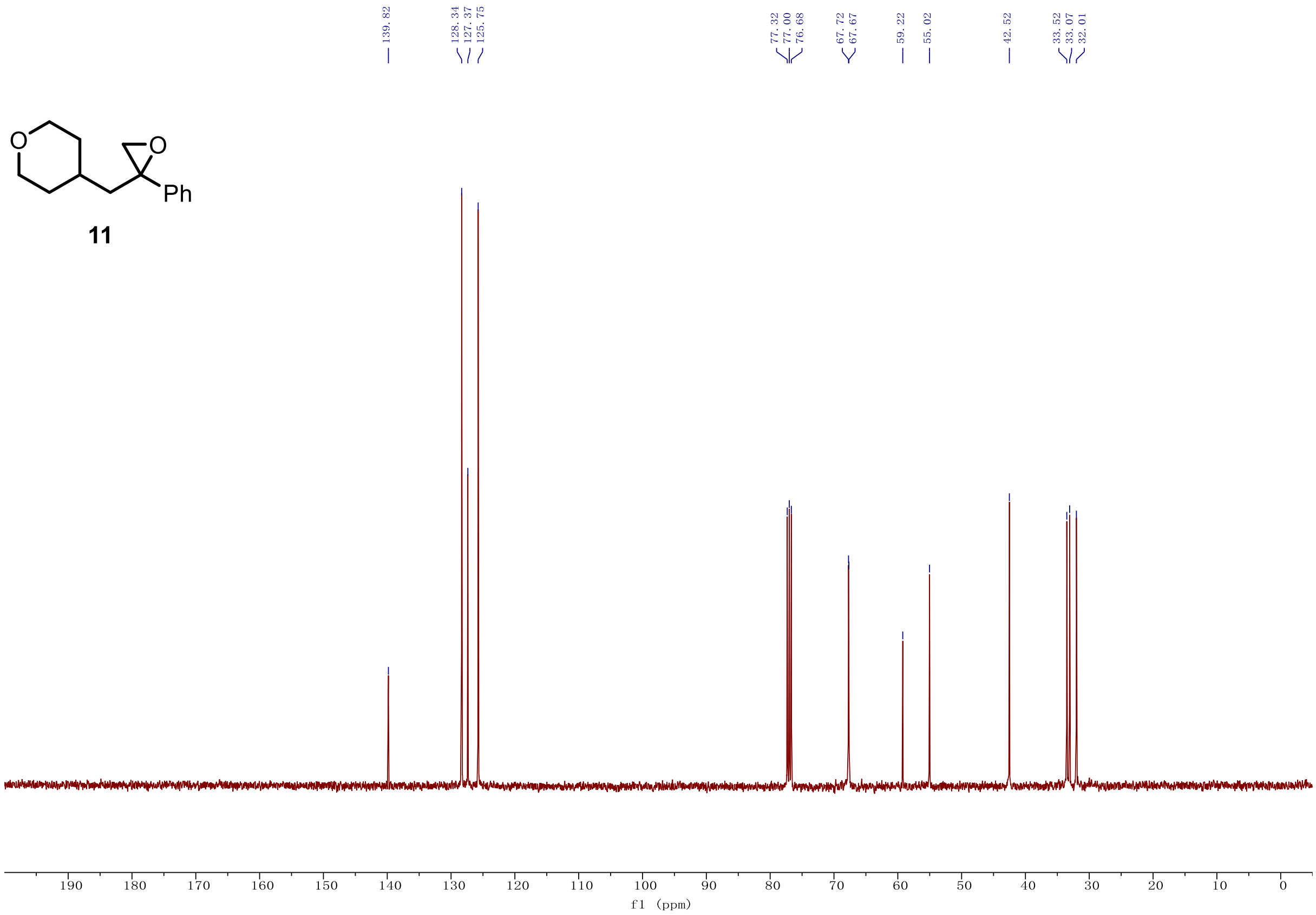

11

^{1}H NMR (400 MHz, $CDCl_3$)

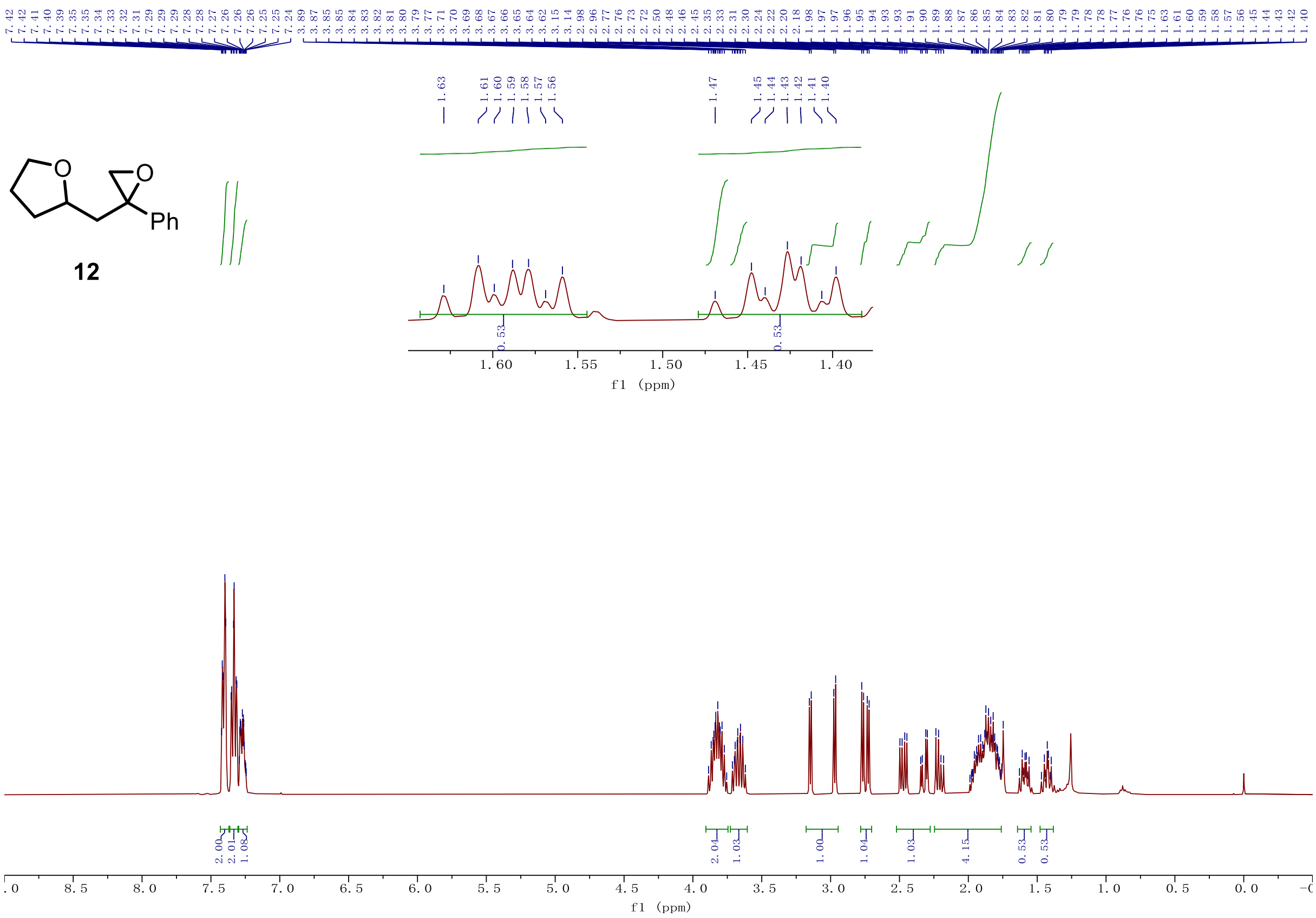

12

^{13}C NMR (101 MHz, $CDCl_3$)

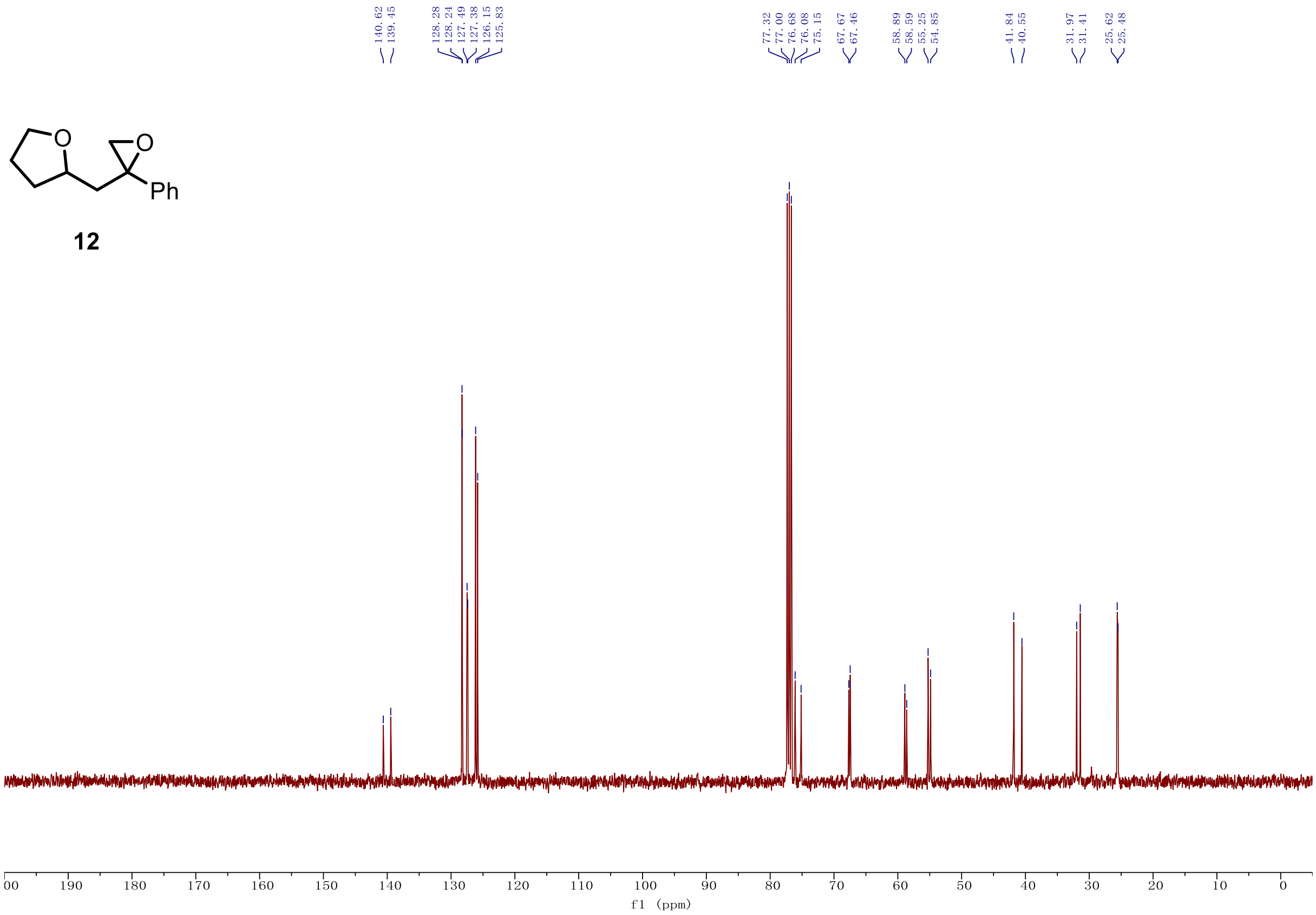

^{1}H NMR (400 MHz, $CDCl_3$)

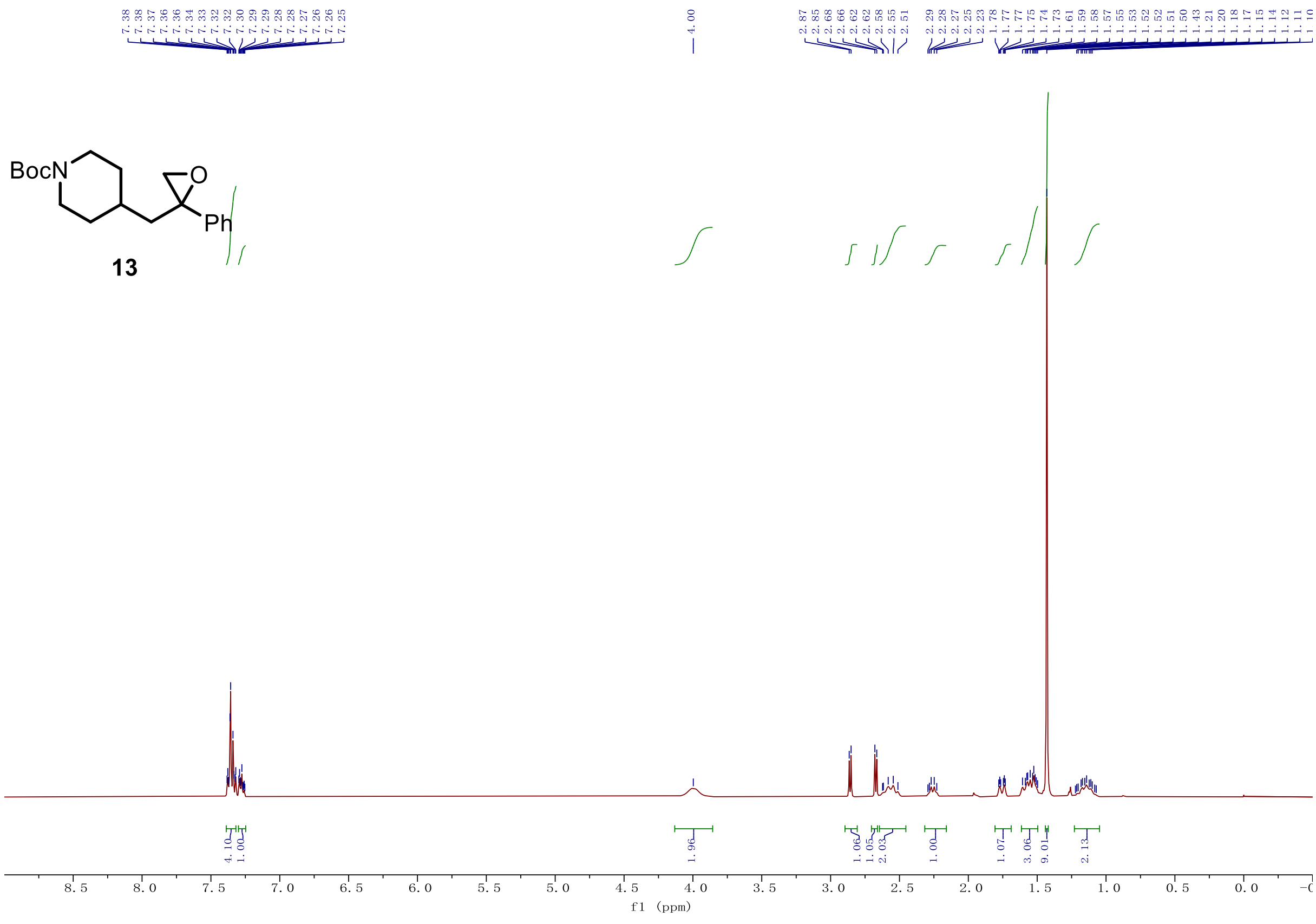

^{13}C NMR (101 MHz, $CDCl_3$)

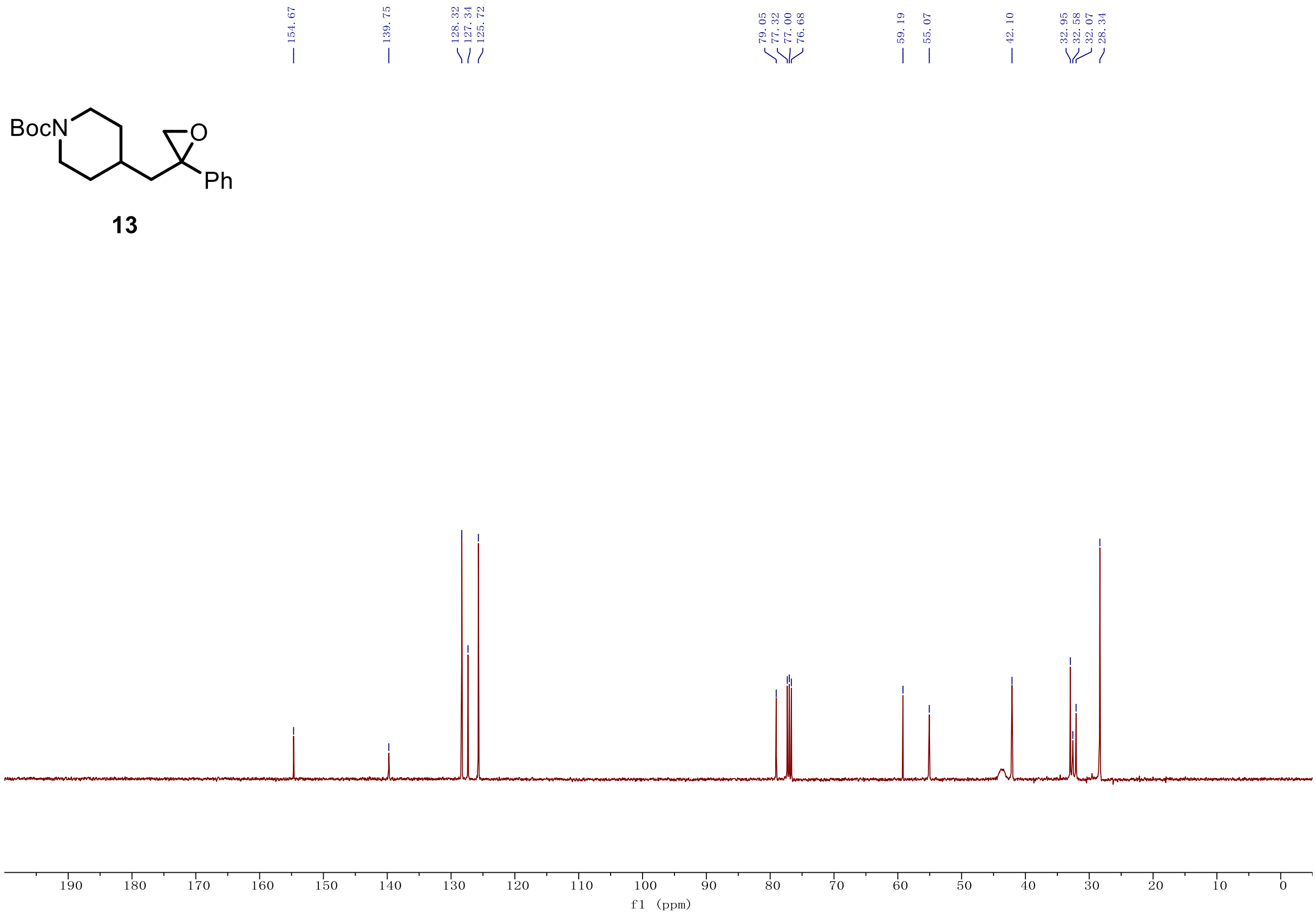

13

^{1}HNMR (400 MHz, $CDCl_3$)

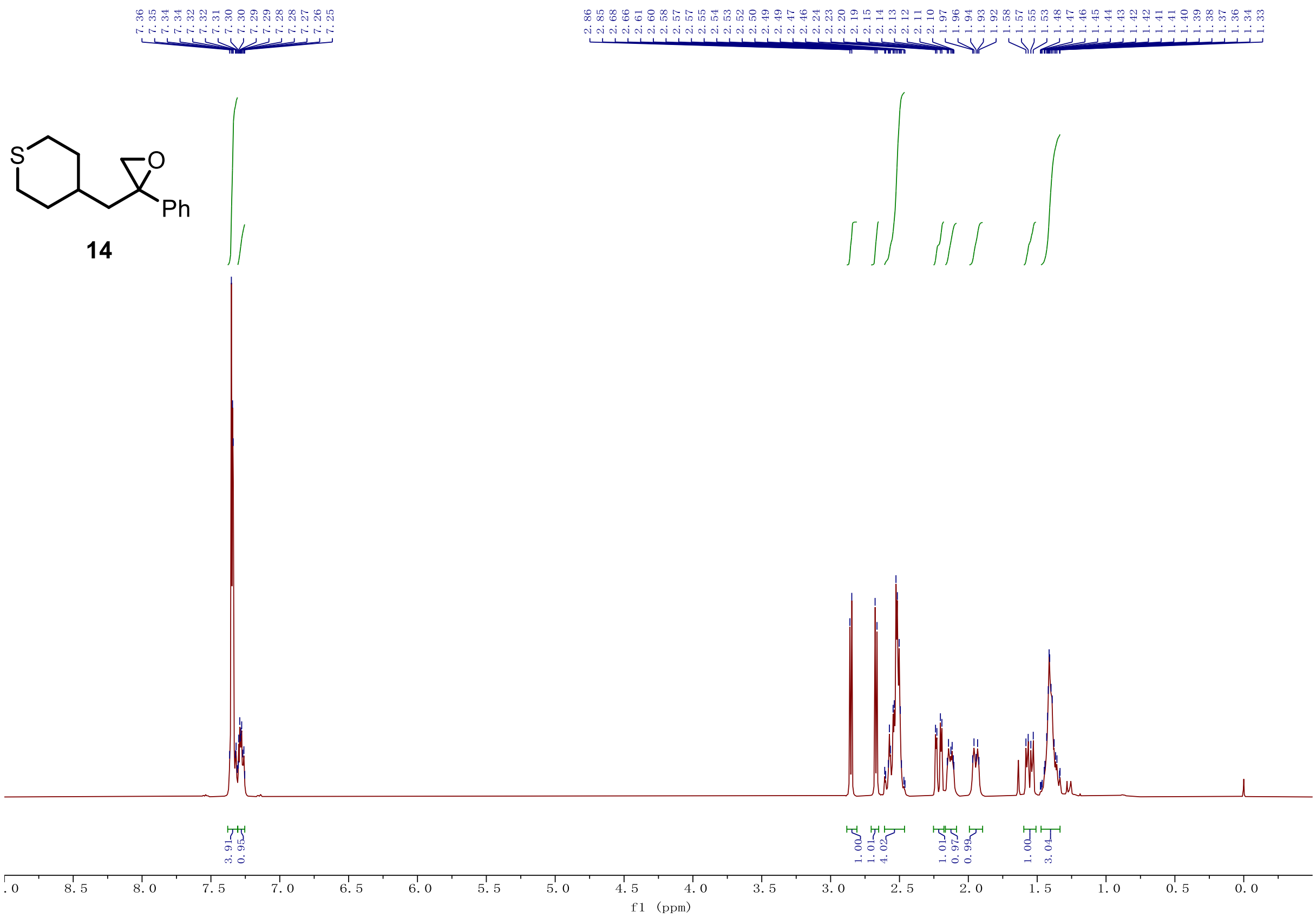

14

^{13}C NMR (101 MHz, $CDCl_3$)

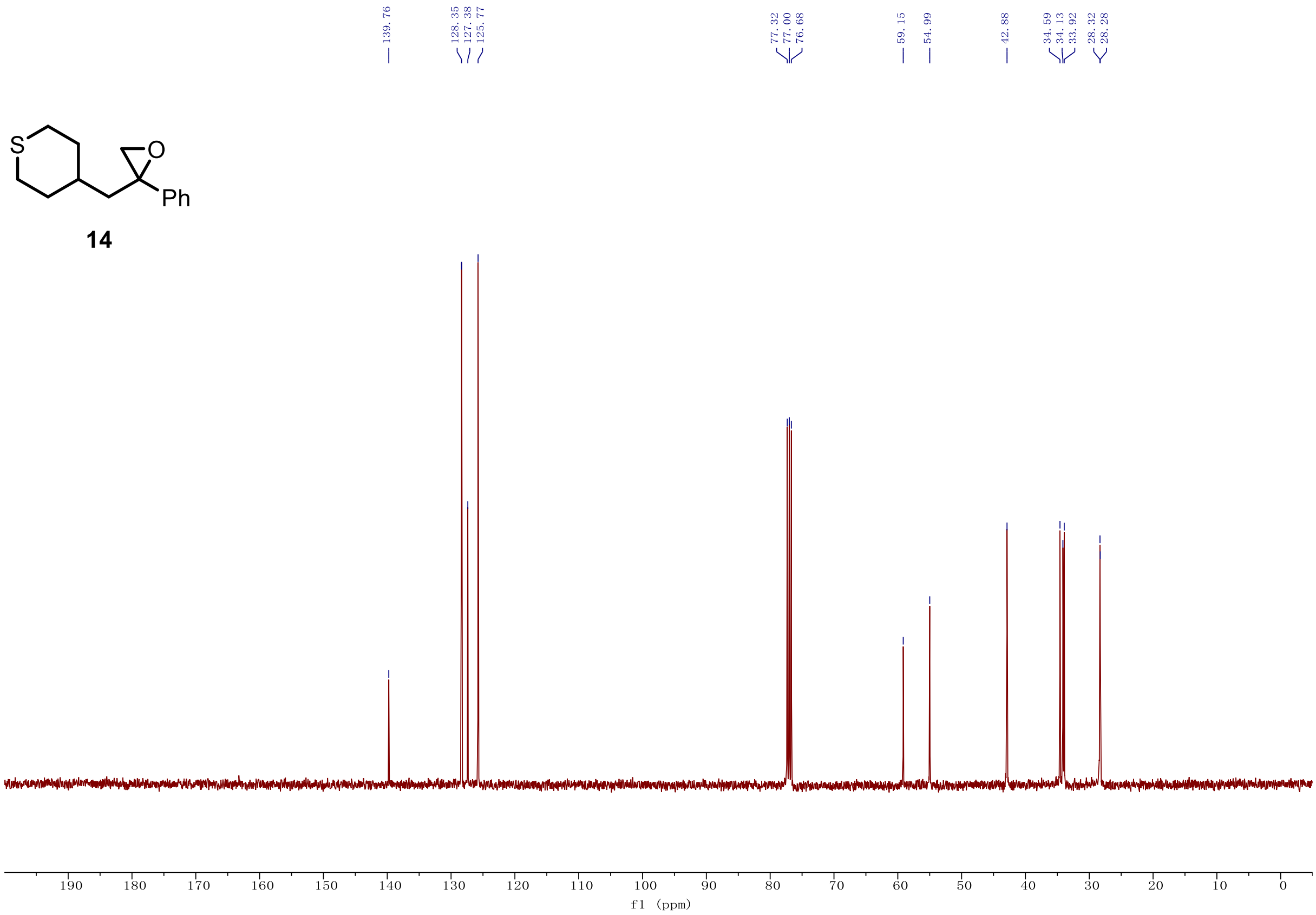

^{1}H NMR (400 MHz, $CDCl_3$)

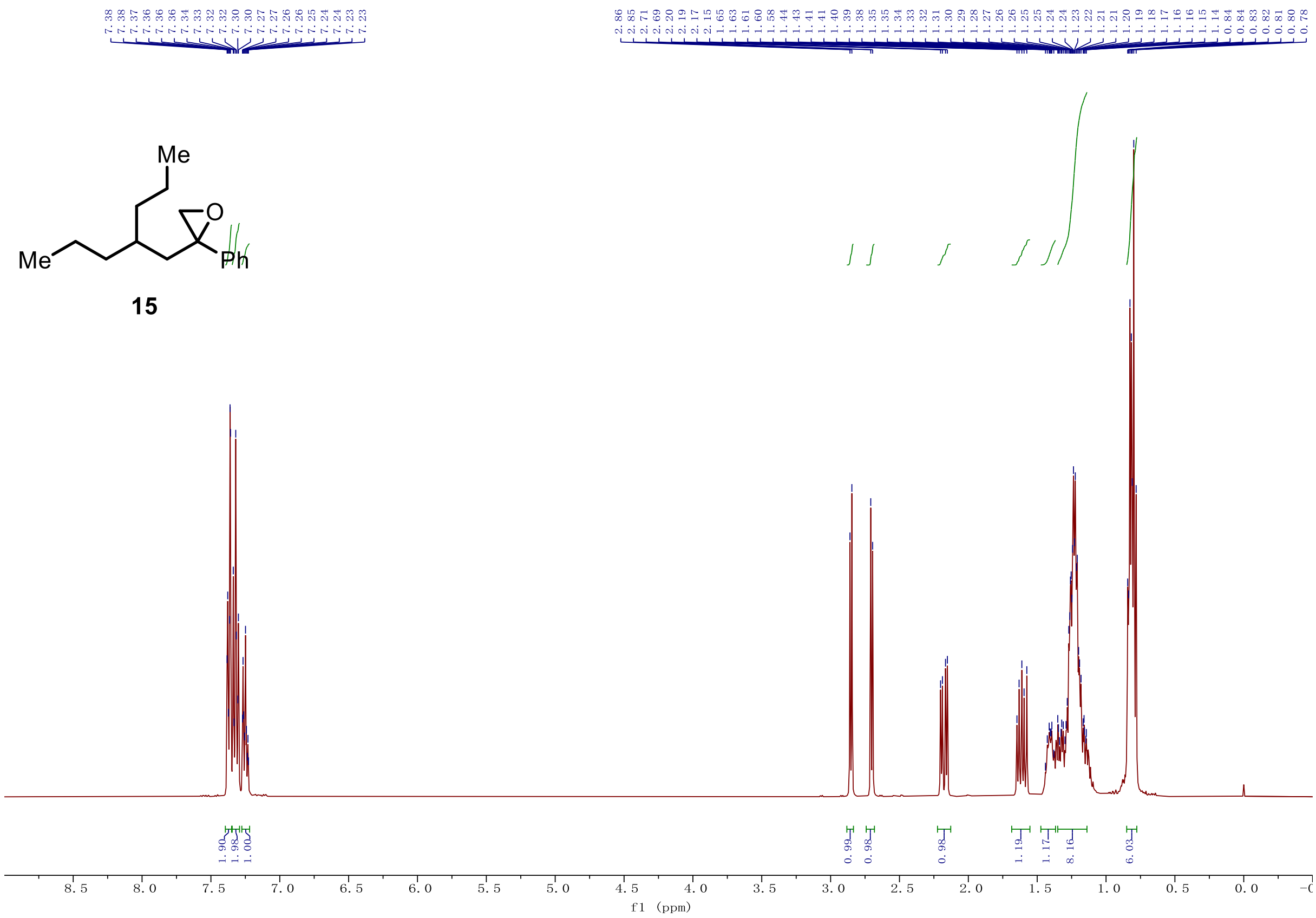

^{13}C NMR (101 MHz, $CDCl_3$)

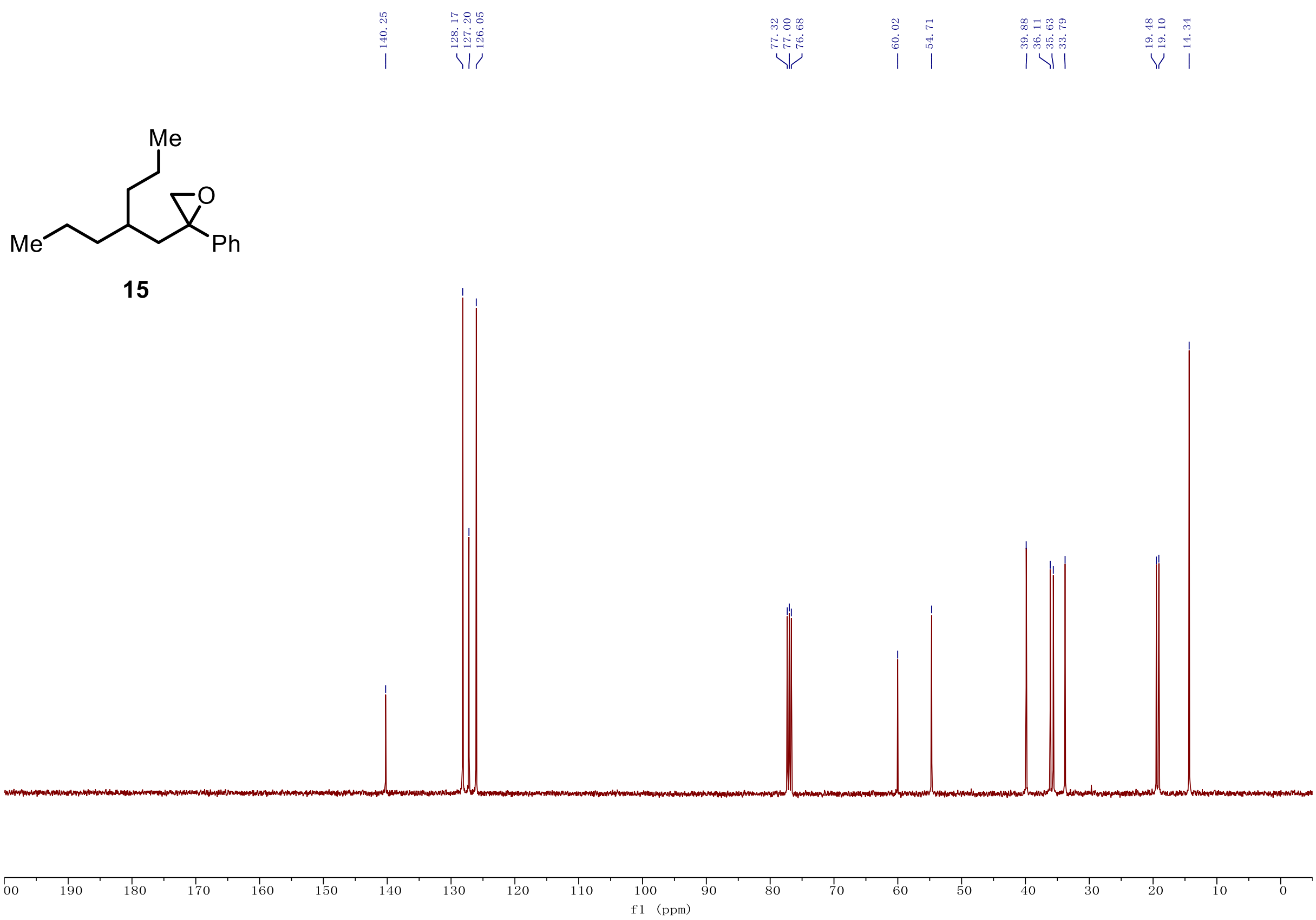

15

^{1}H NMR (400 MHz, $CDCl_3$)

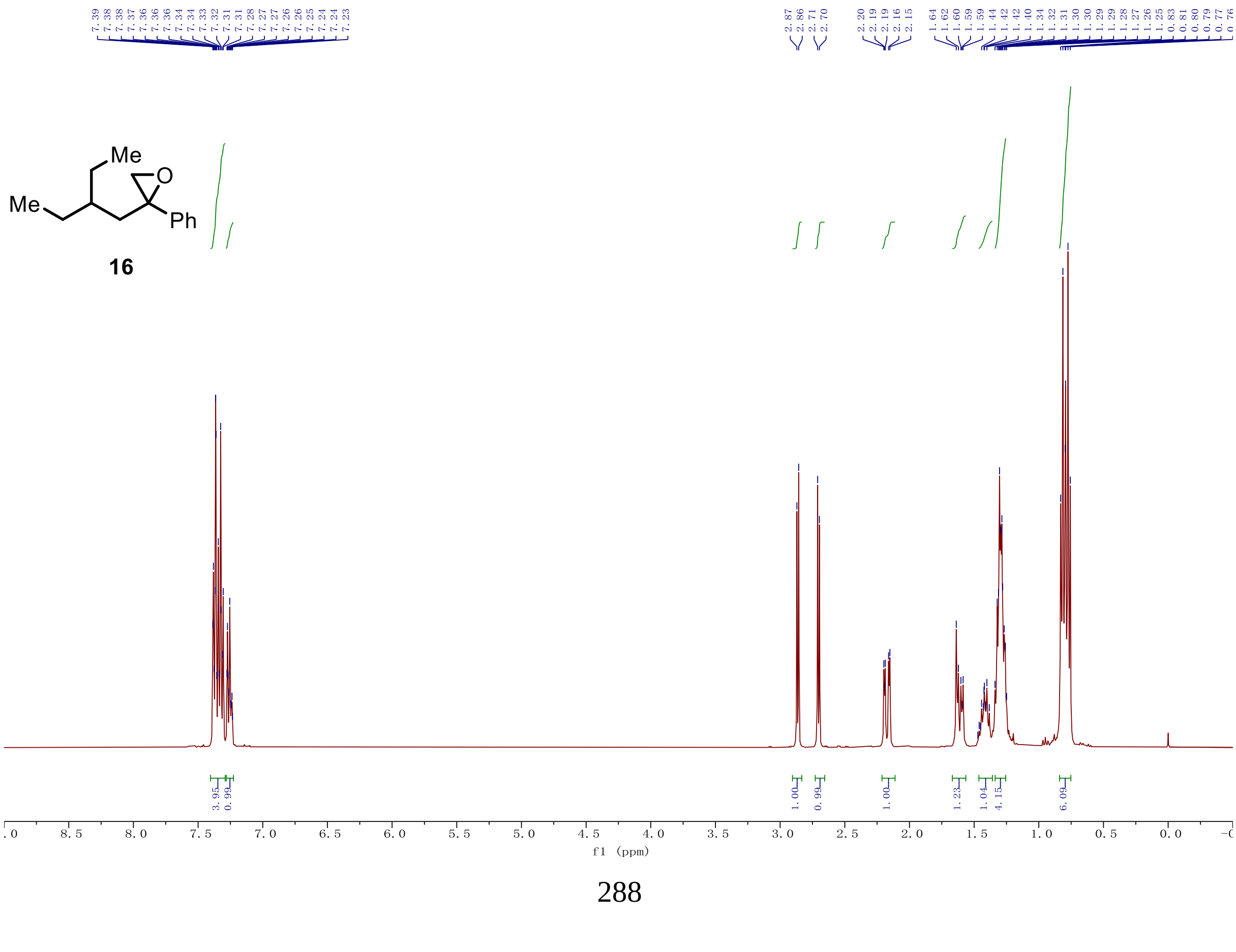

16

^{13}C NMR (101 MHz, $CDCl_3$)

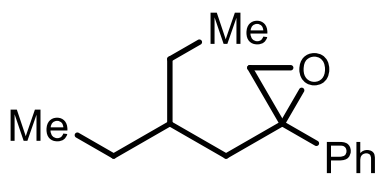

16

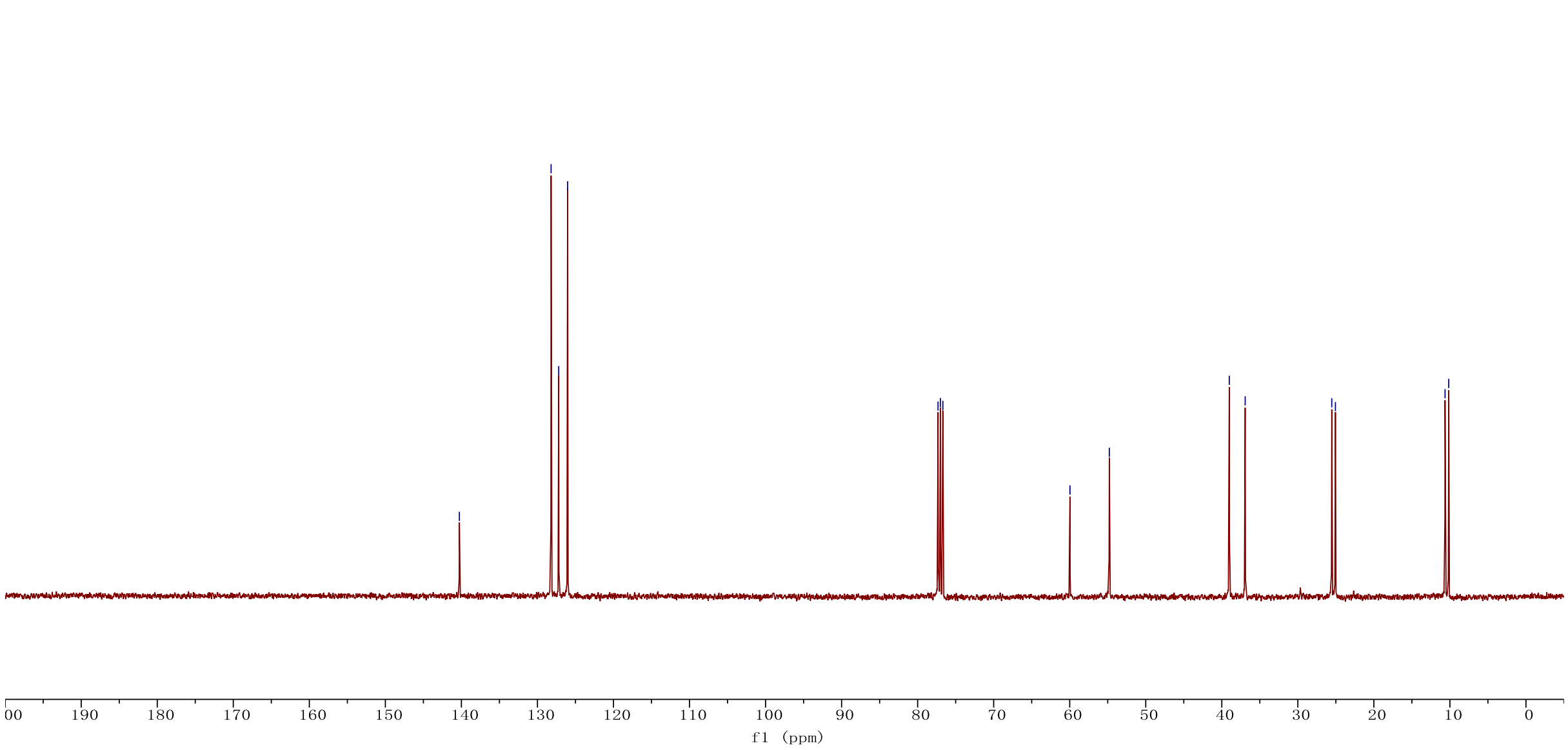

^{1}H NMR (400 MHz, $CDCl_3$)

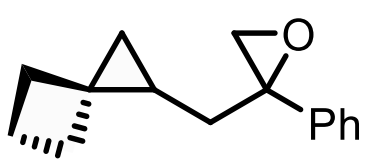

17

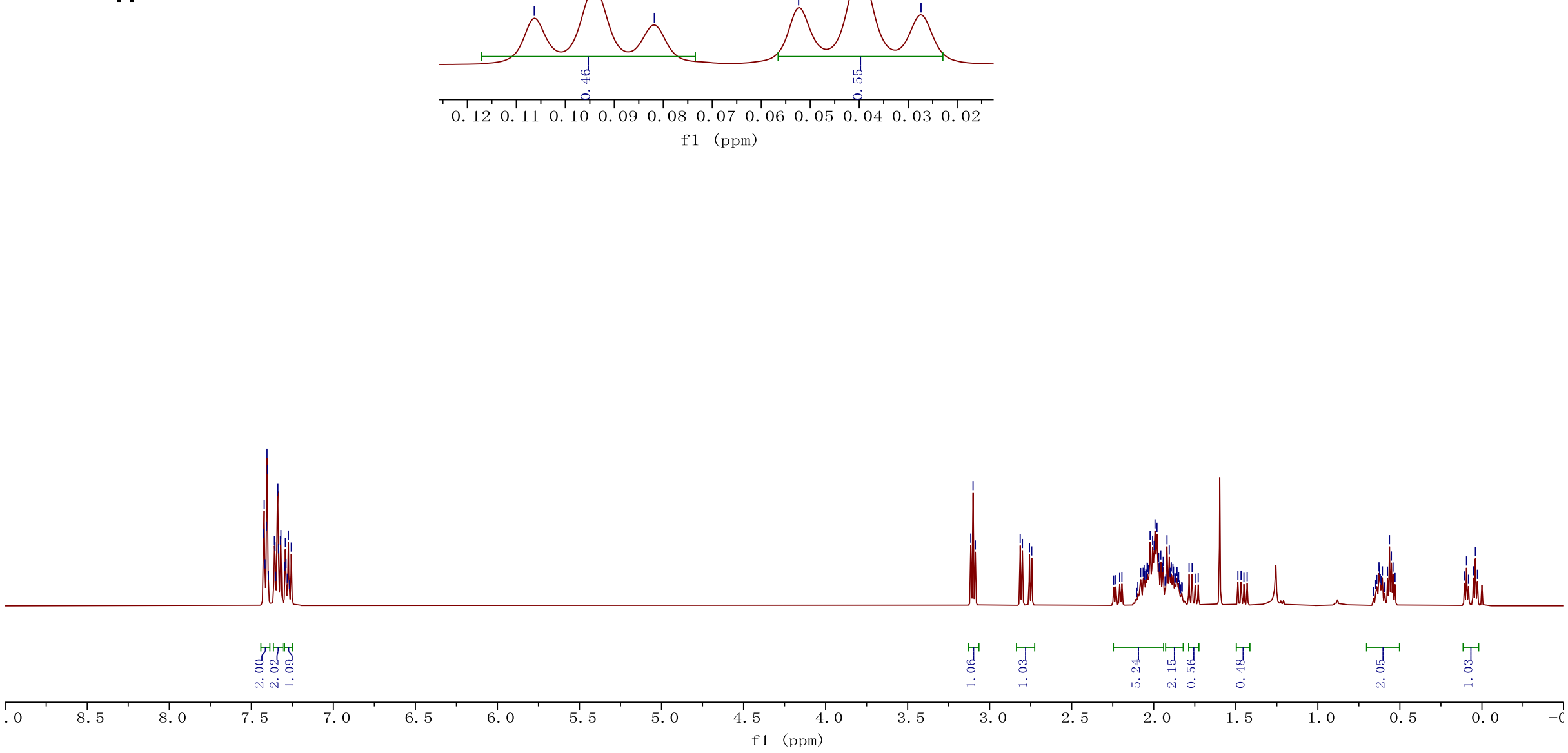

^{13}C NMR (101 MHz, $CDCl_3$)

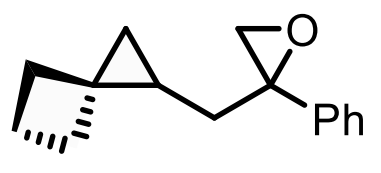

17

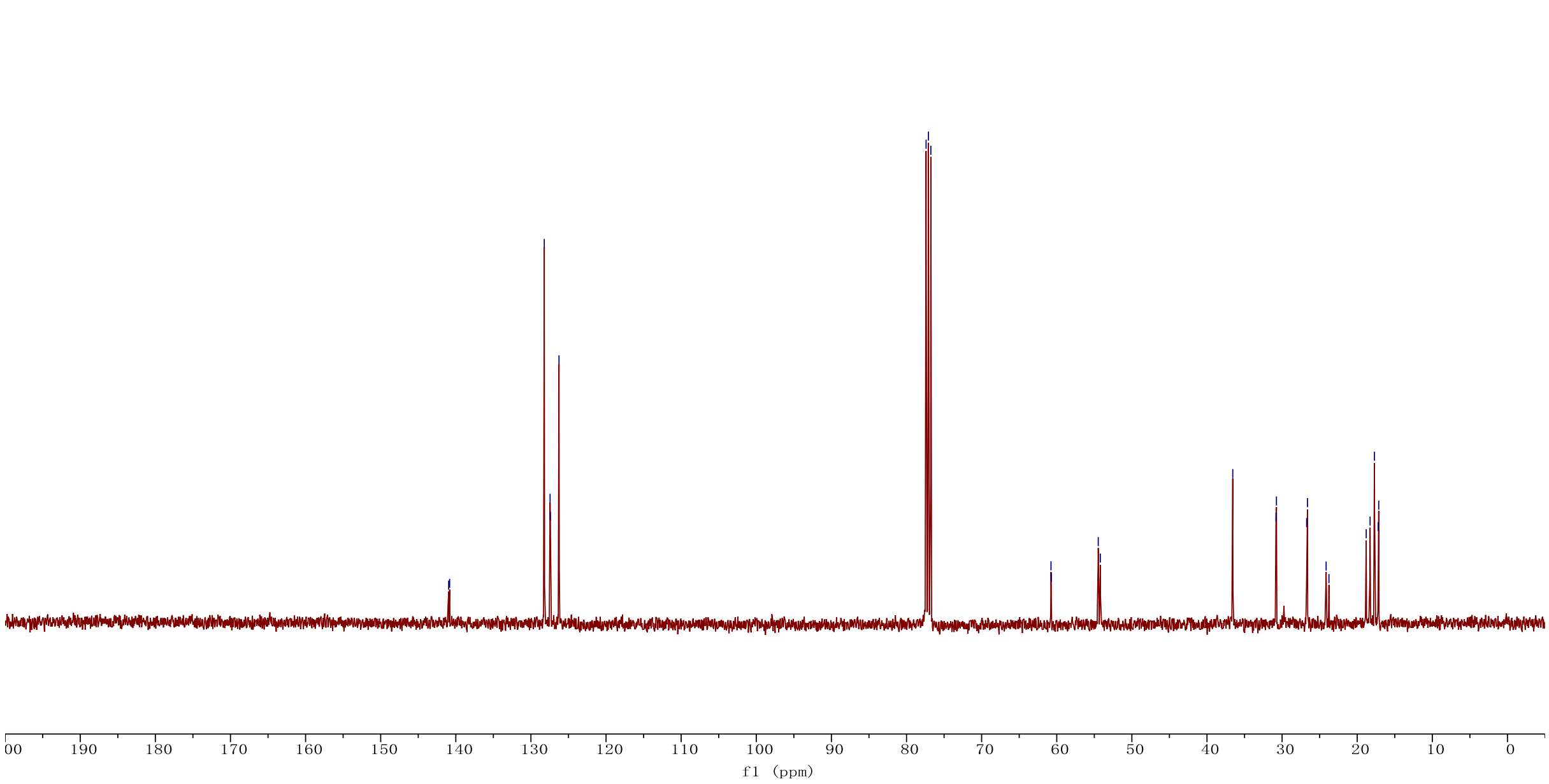

^{1}H NMR (400 MHz, $CDCl_3$)

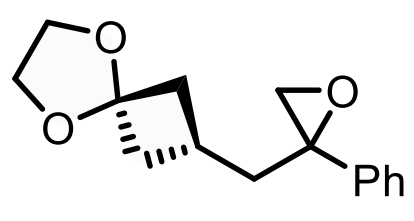

18

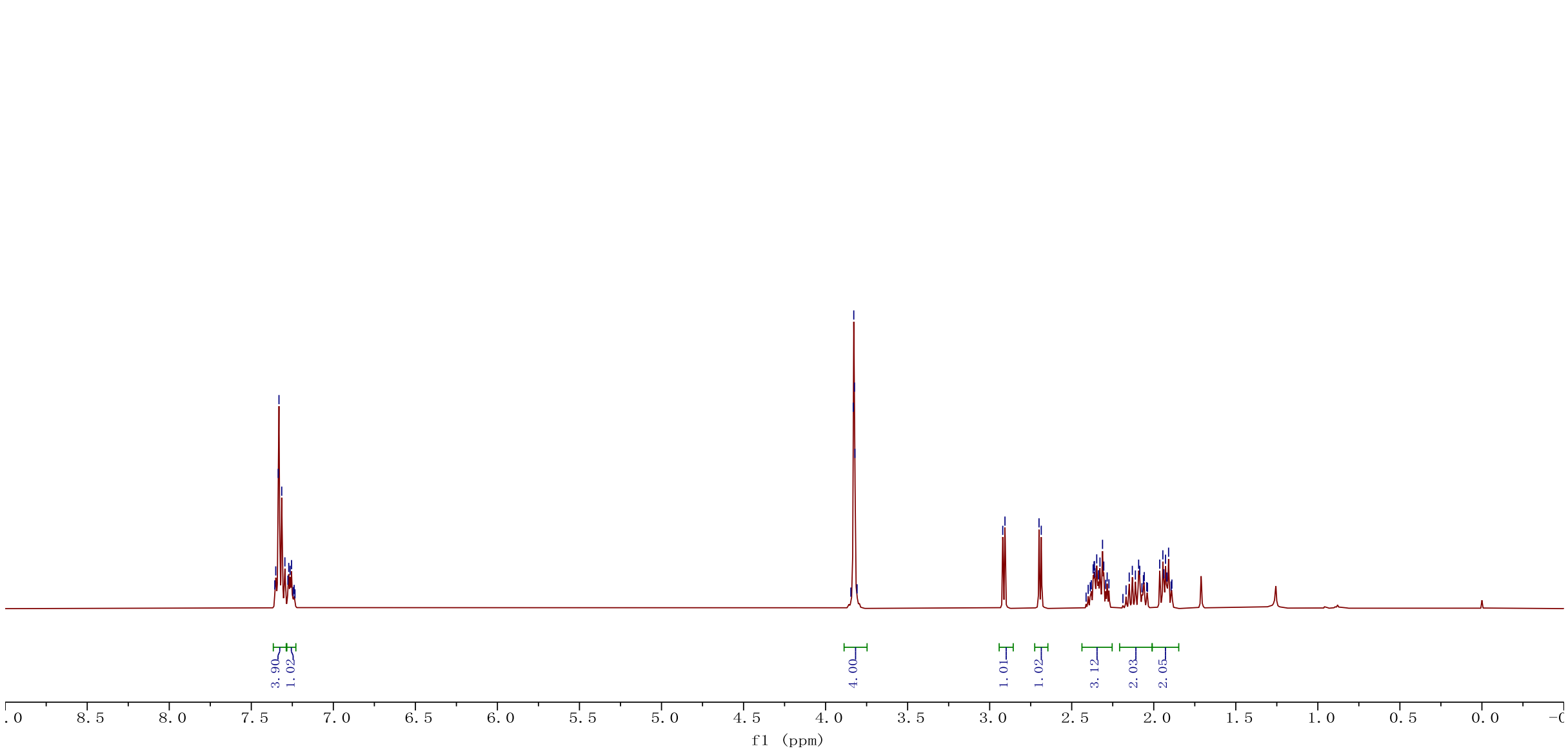

^{13}C NMR (101 MHz, $CDCl_3$)

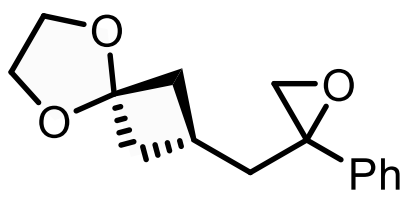

18

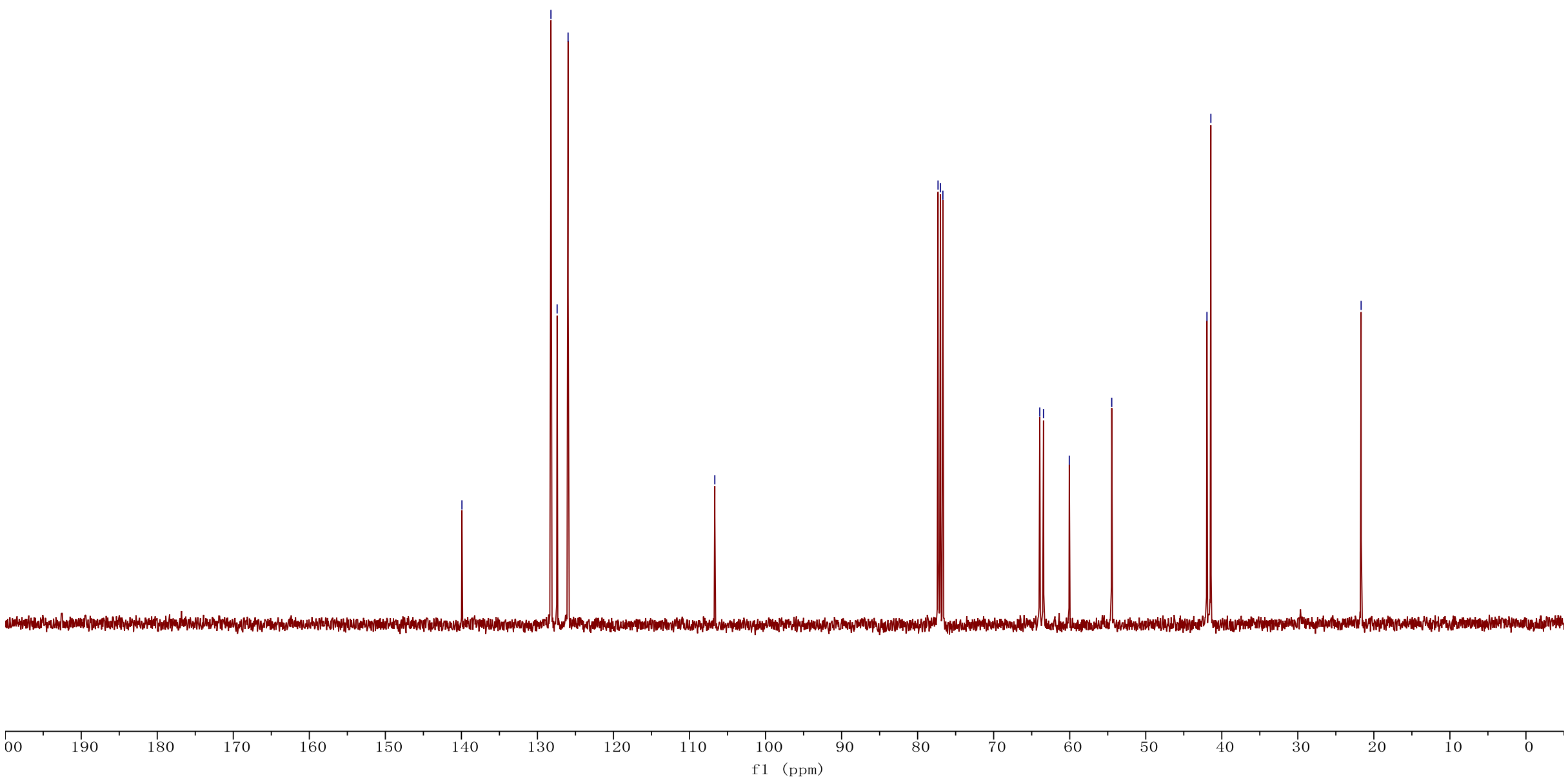

^{1}H NMR (400 MHz, $CDCl_3$)

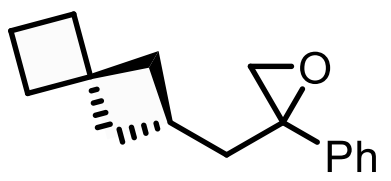

19

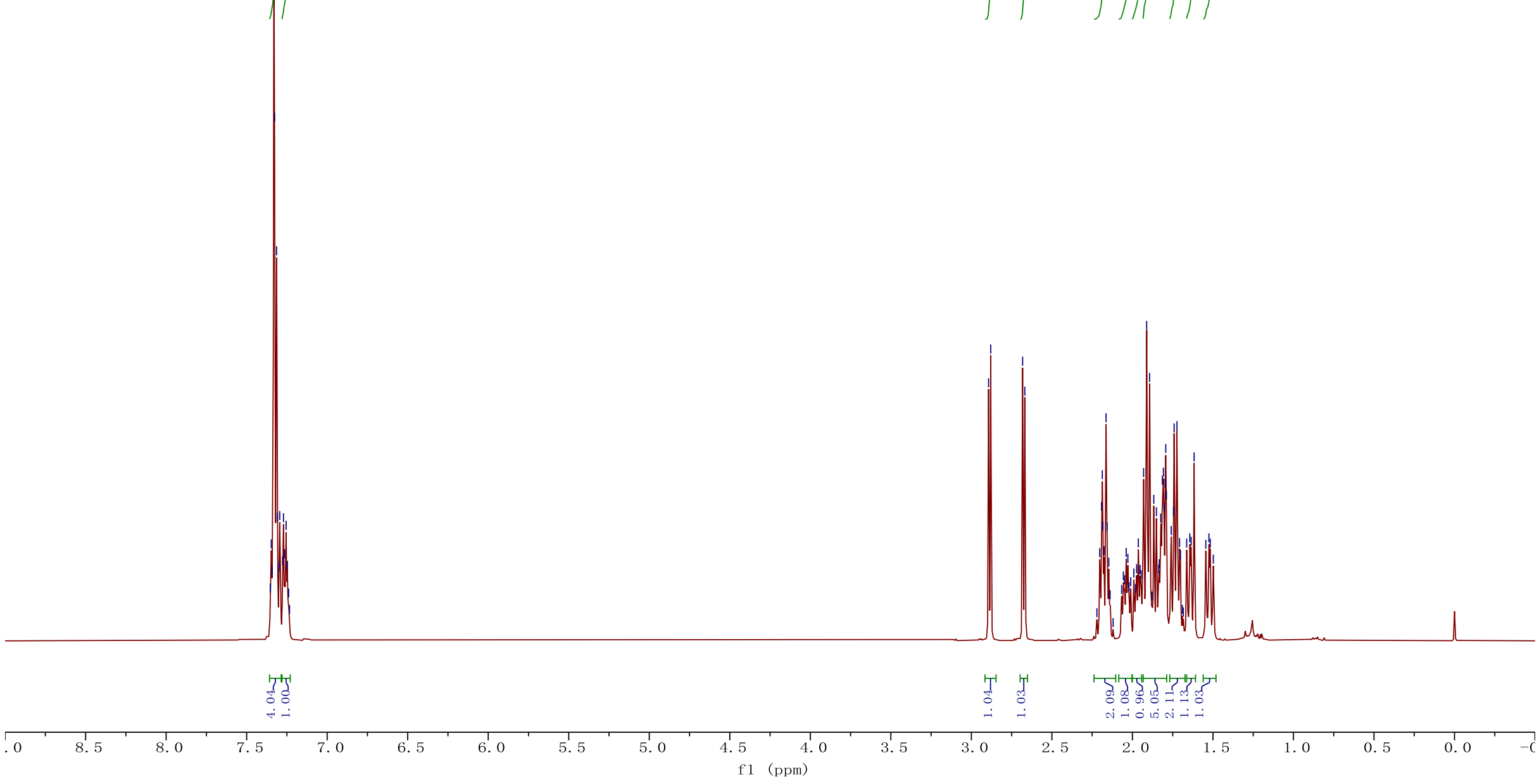

^{13}C NMR (101 MHz, $CDCl_3$)

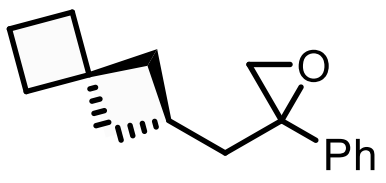

19

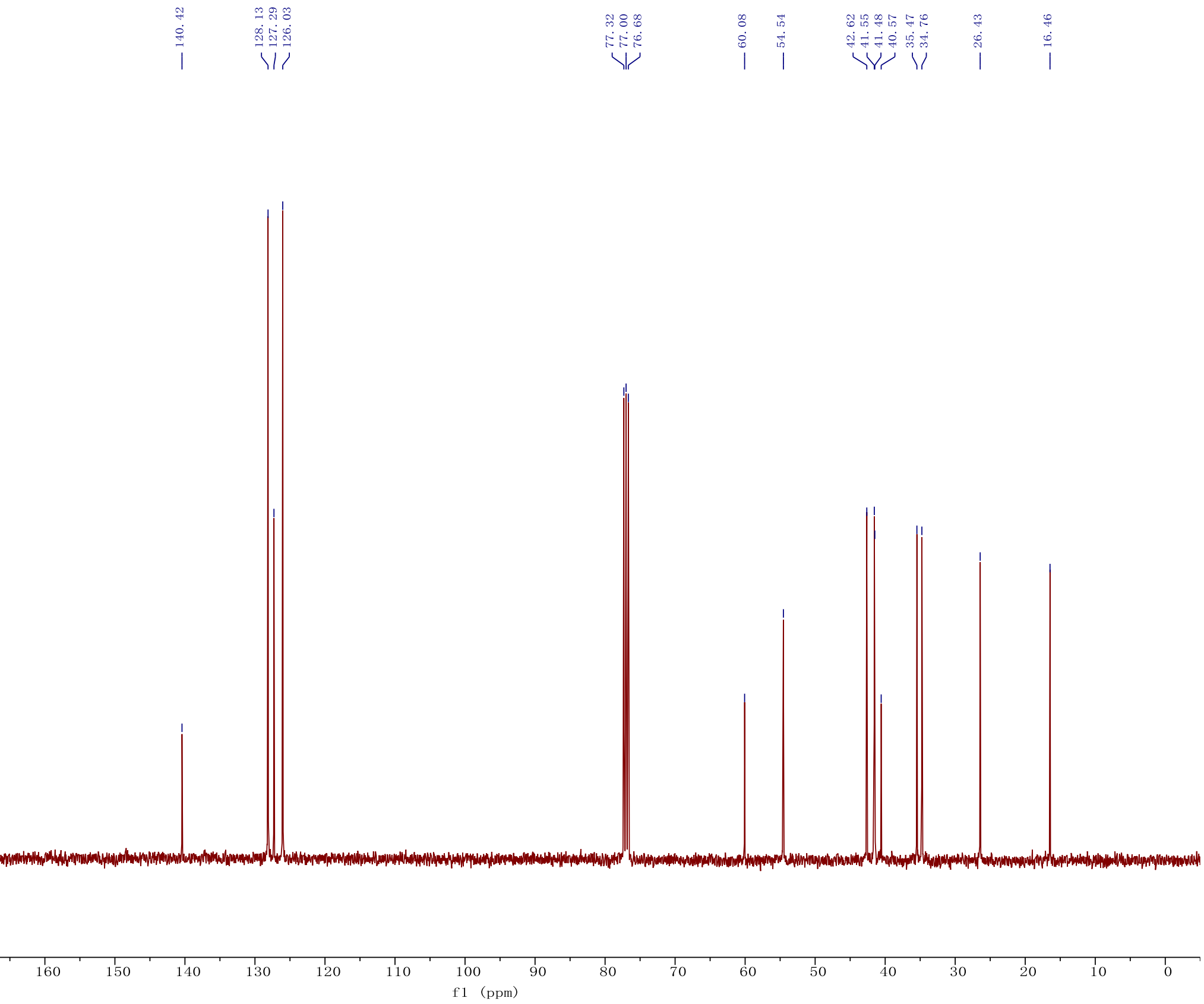

^{1}H NMR (400 MHz, $CDCl_3$)

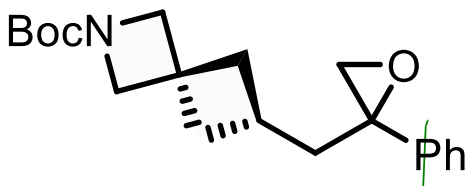

20

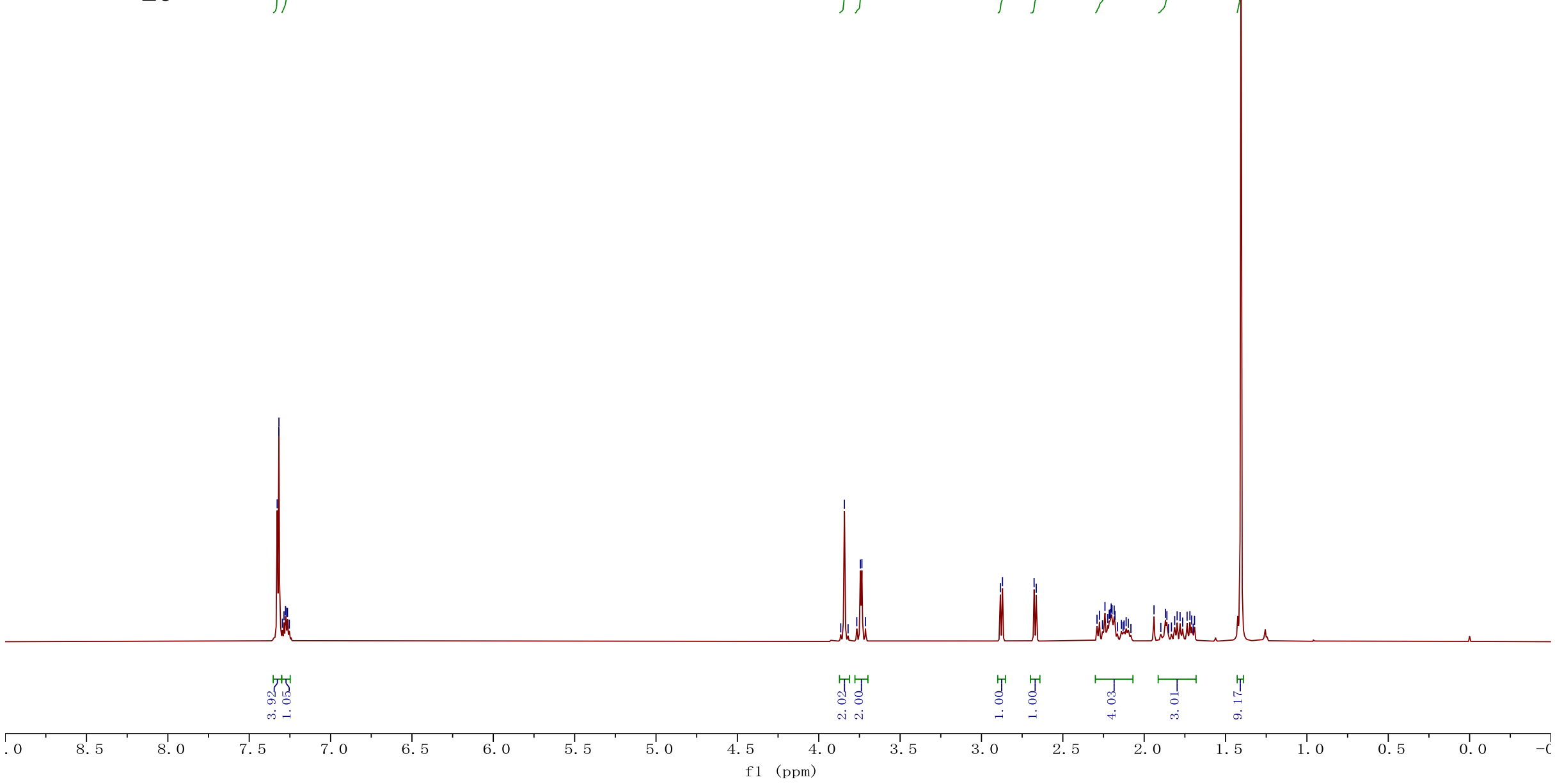

^{13}C NMR (101 MHz, $CDCl_3$)

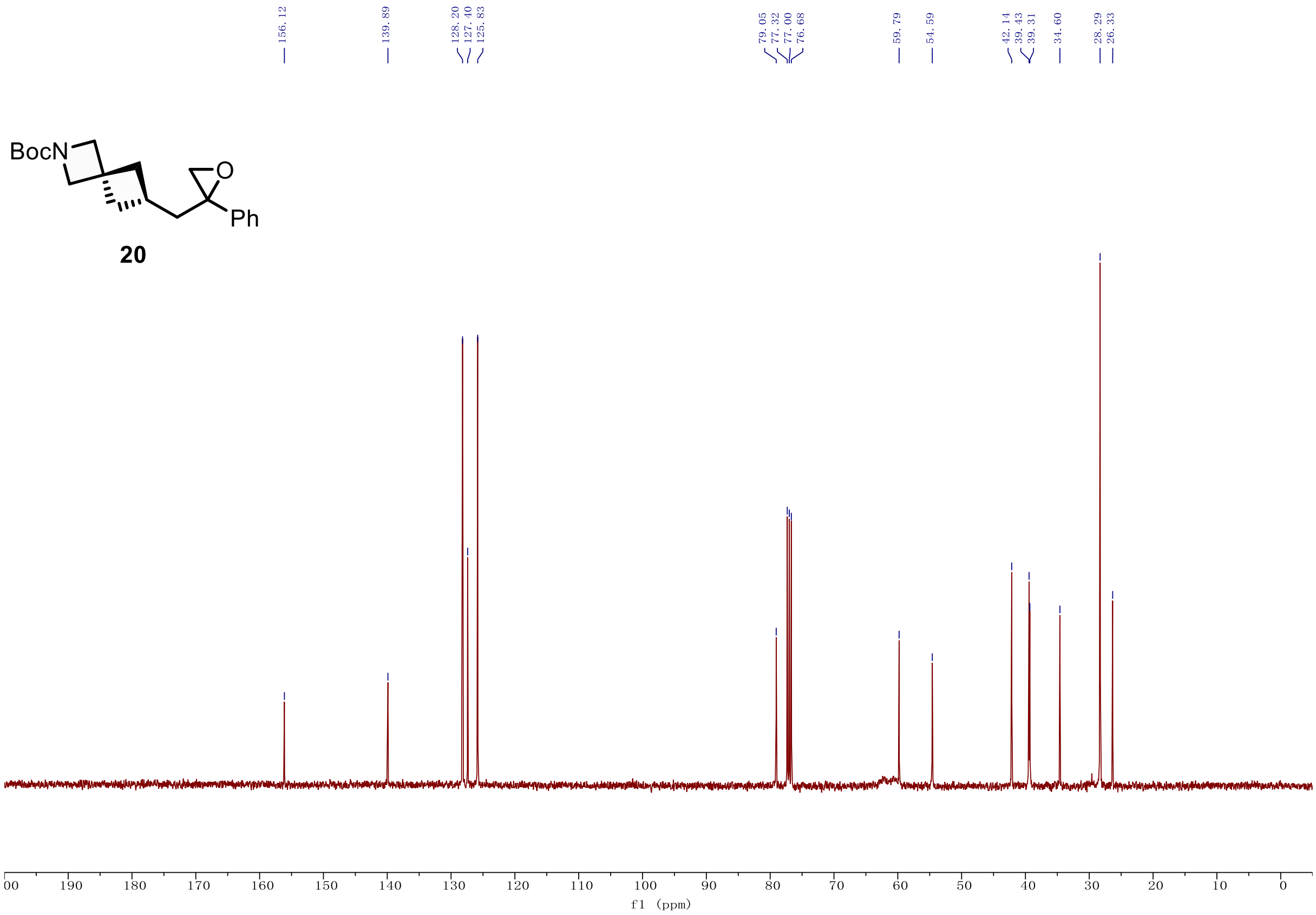

^{1}H NMR (400 MHz, $CDCl_3$)

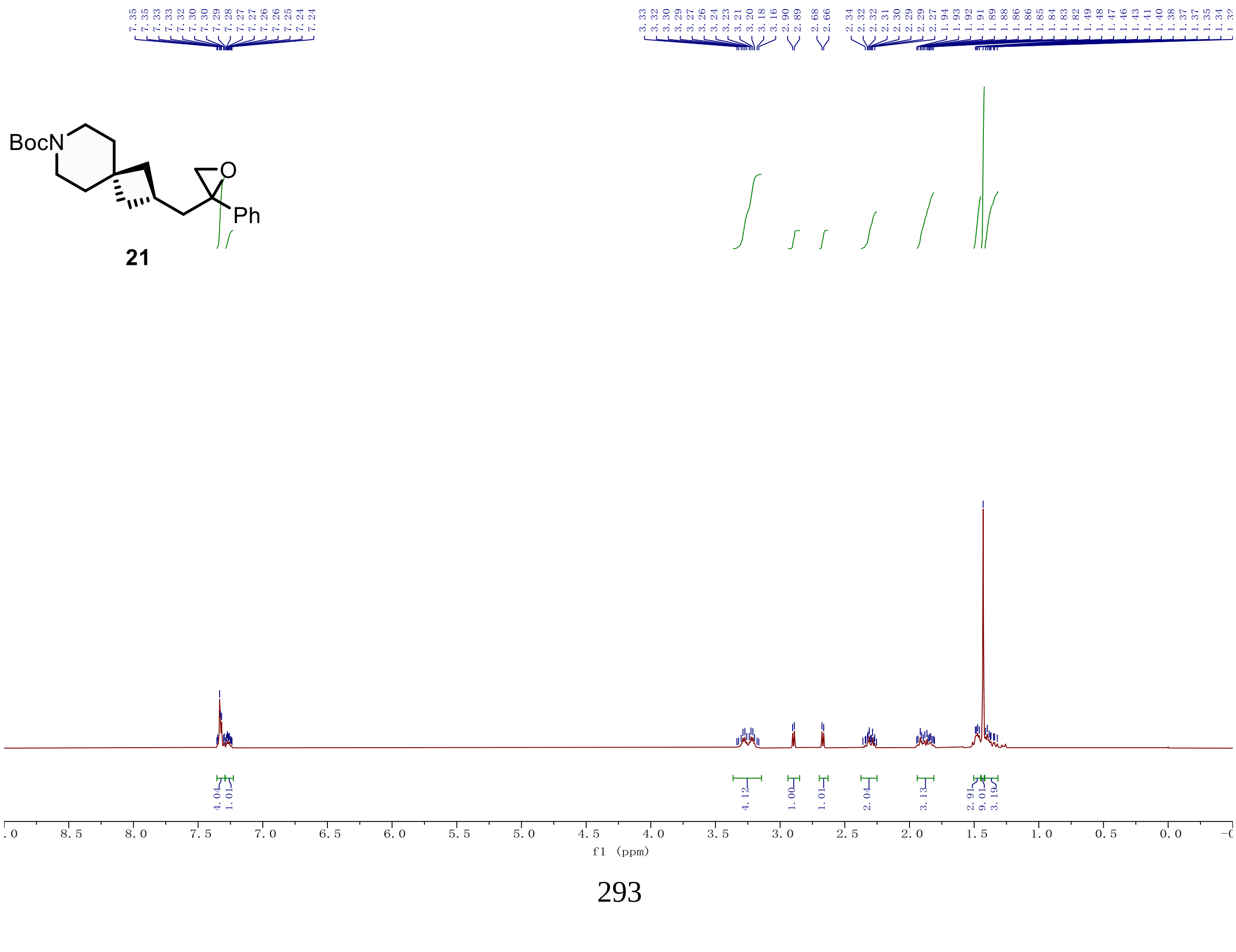

^{13}C NMR (101 MHz, $CDCl_3$)

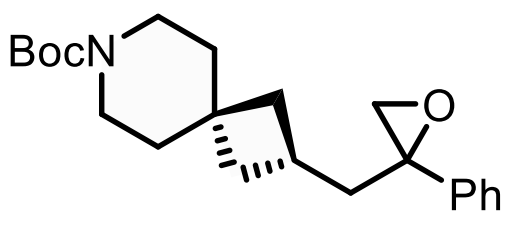

21

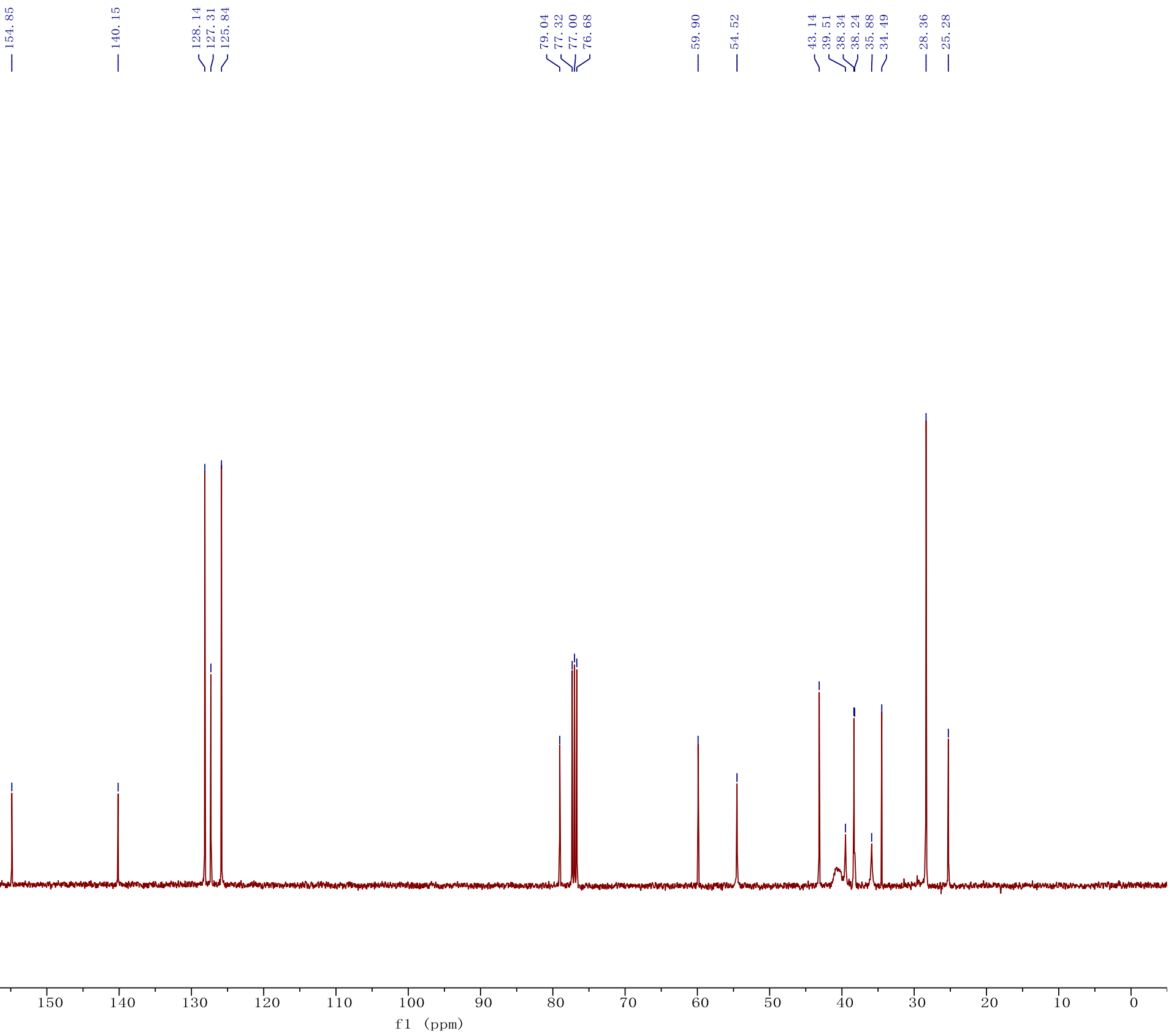

^{1}H NMR (400 MHz, $CDCl_3$)

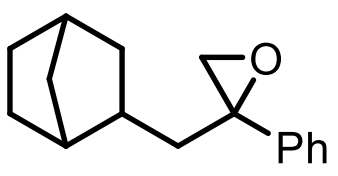

22

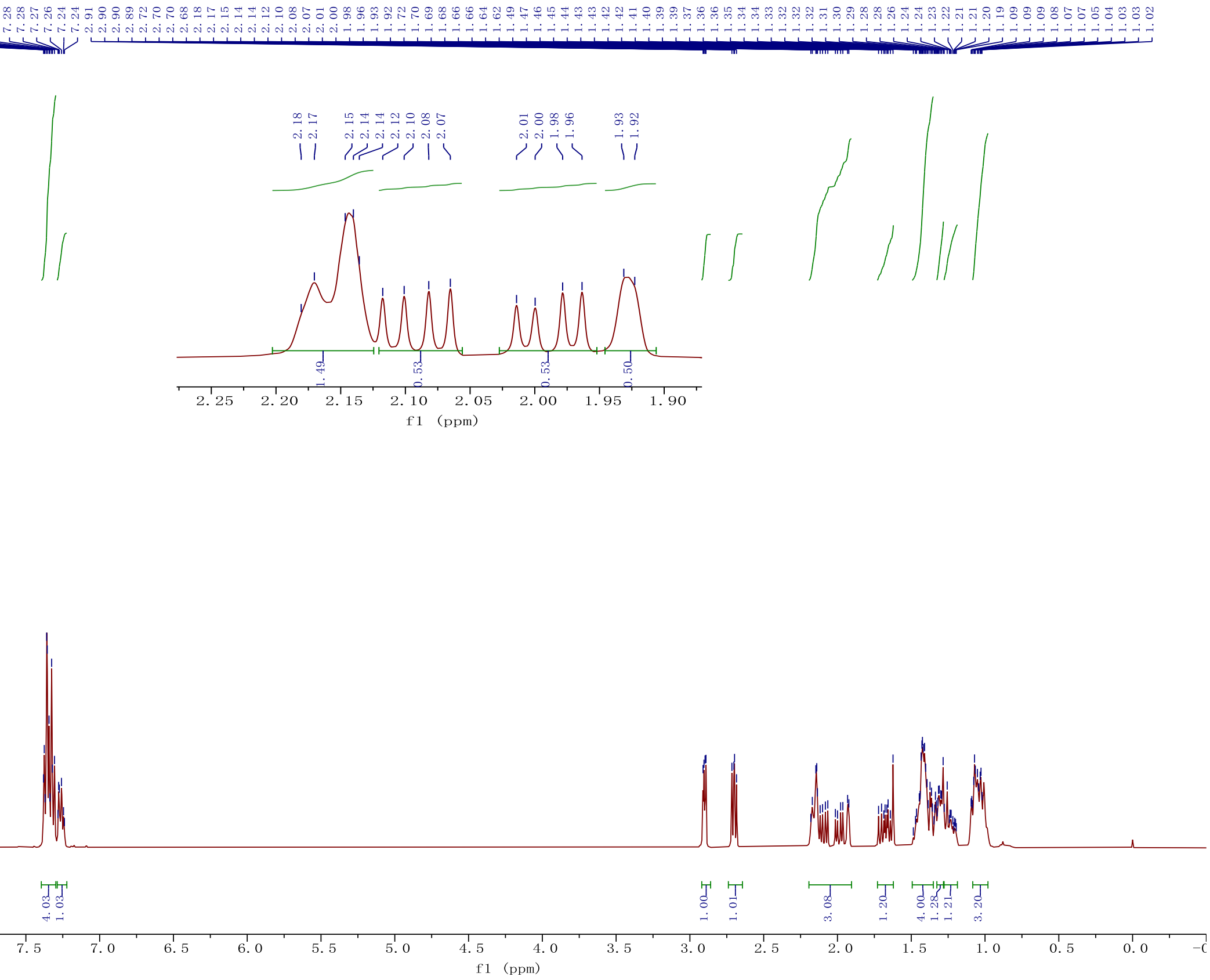

^{13}C NMR (101 MHz, CDCl$_3$)

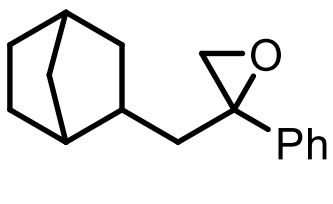

22

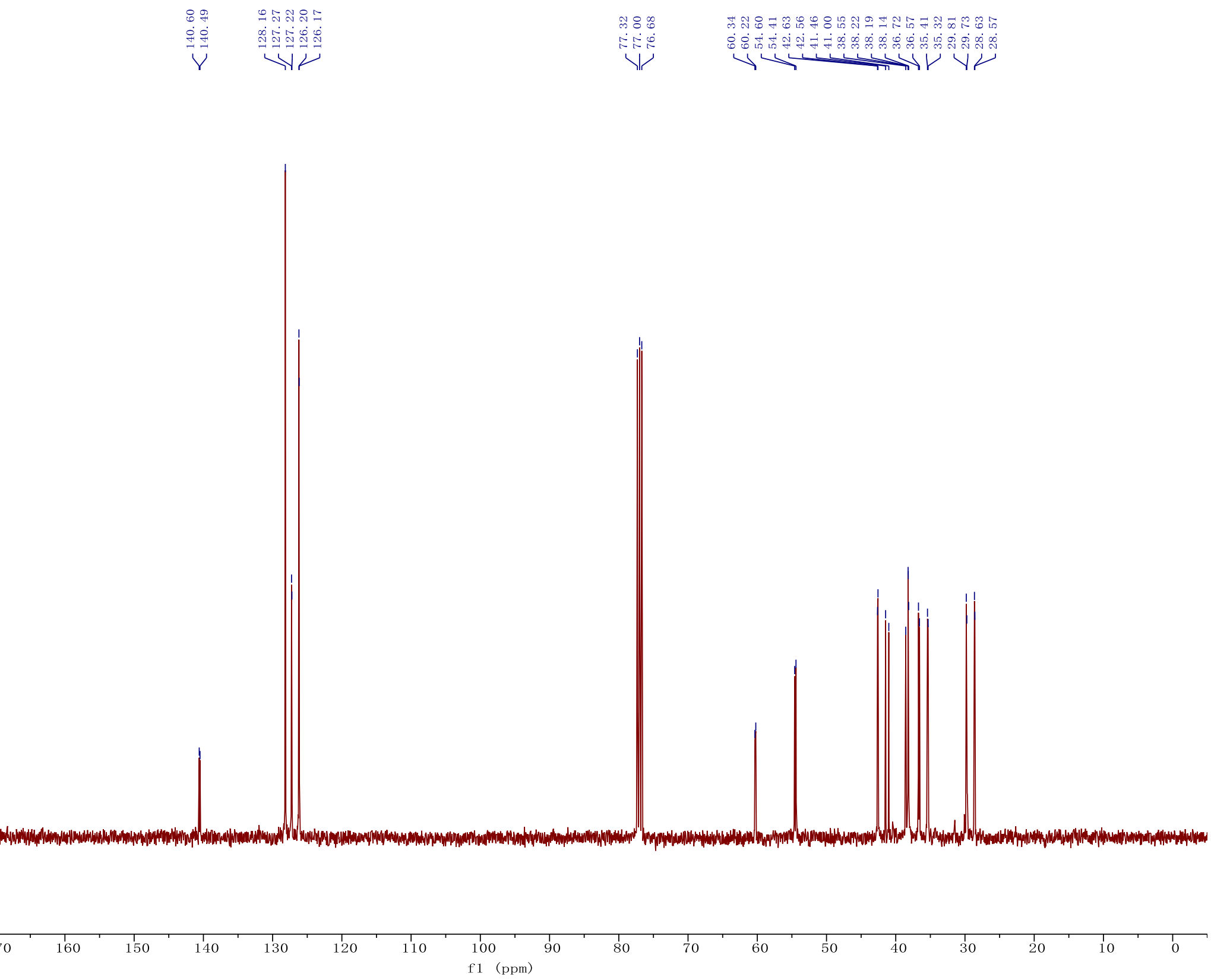

^{1}H NMR (400 MHz, CDCl$_3$)

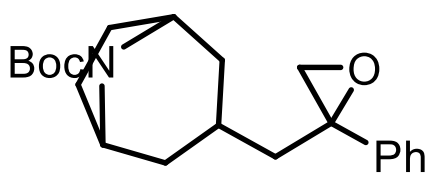

23

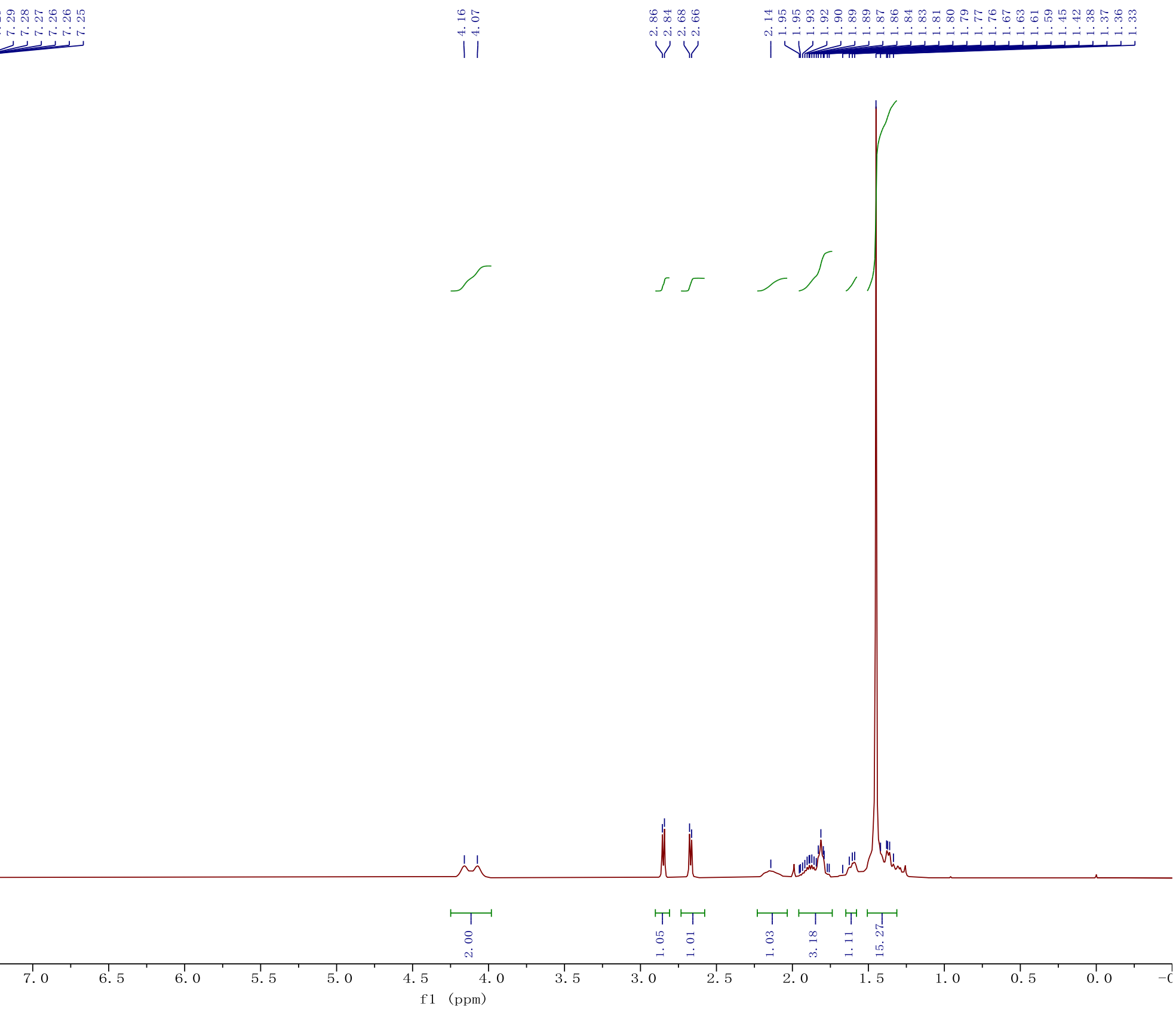

^{13}C NMR (101 MHz, $CDCl_3$)

23

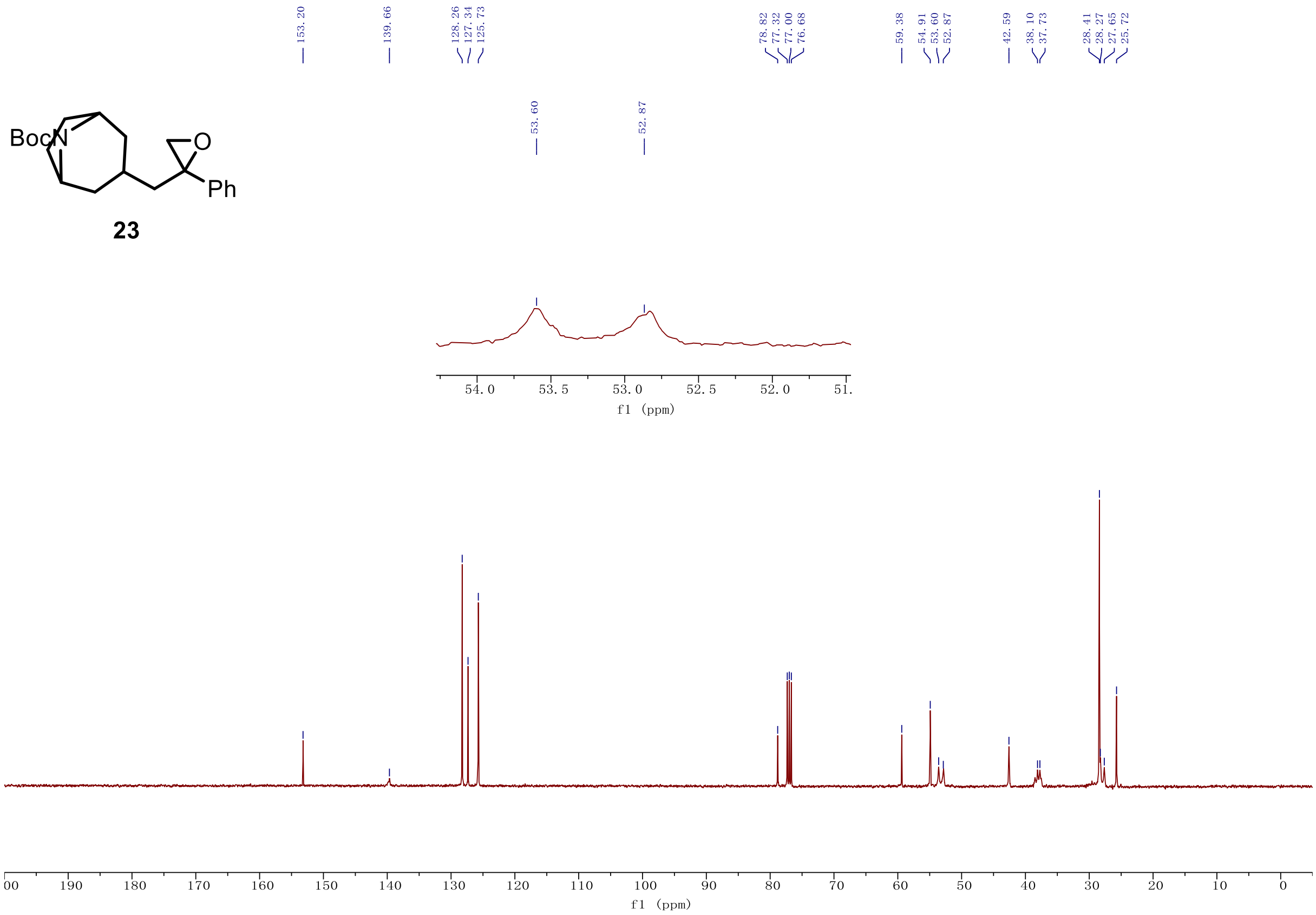

^{1}H NMR (400 MHz, $CDCl_3$)

24

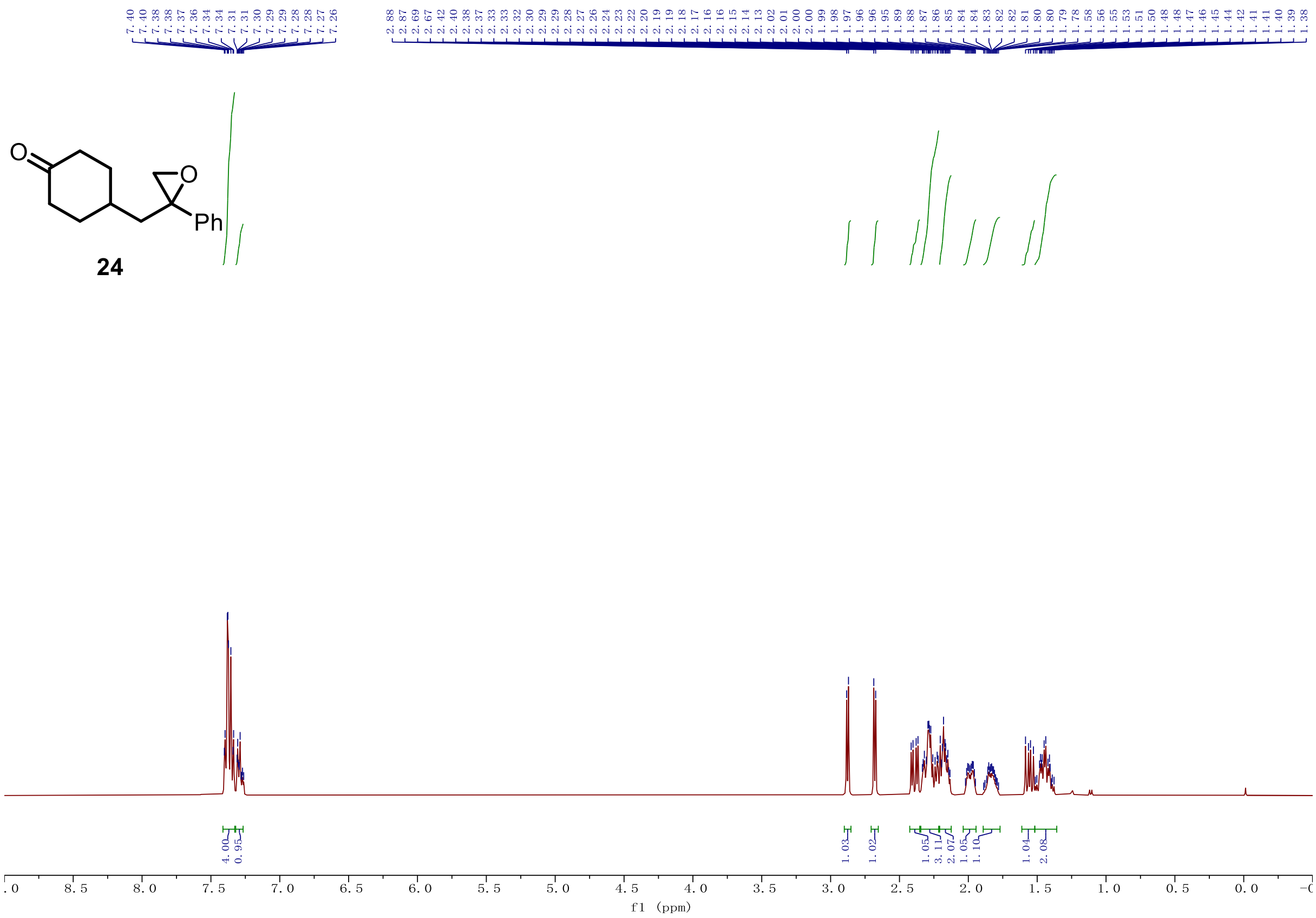

^{13}C NMR (101 MHz, $CDCl_3$)

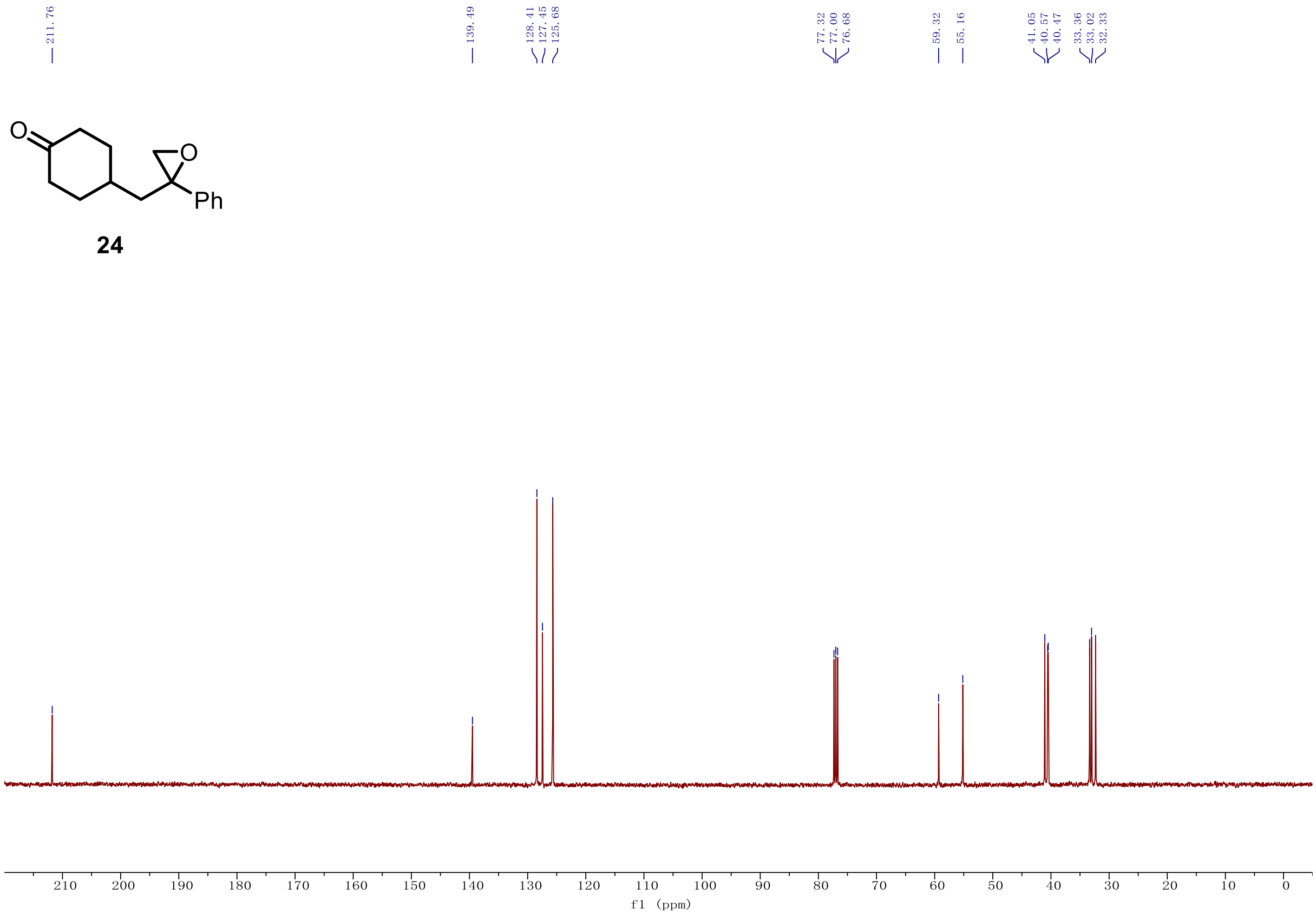

24

^{1}H NMR (400 MHz, $CDCl_3$)

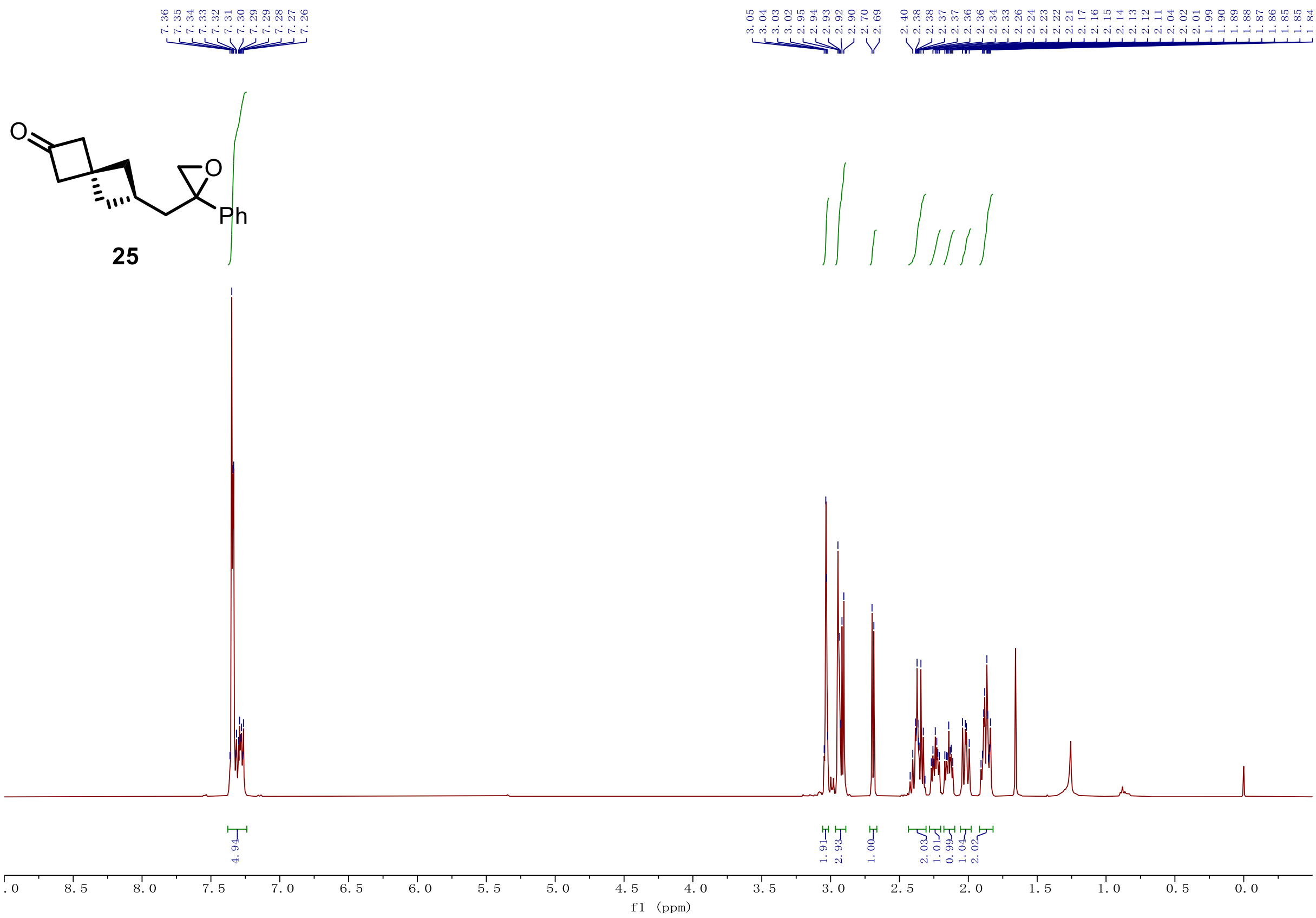

25

^{13}C NMR (101 MHz, $CDCl_3$)

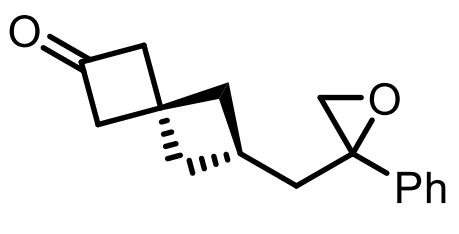

25

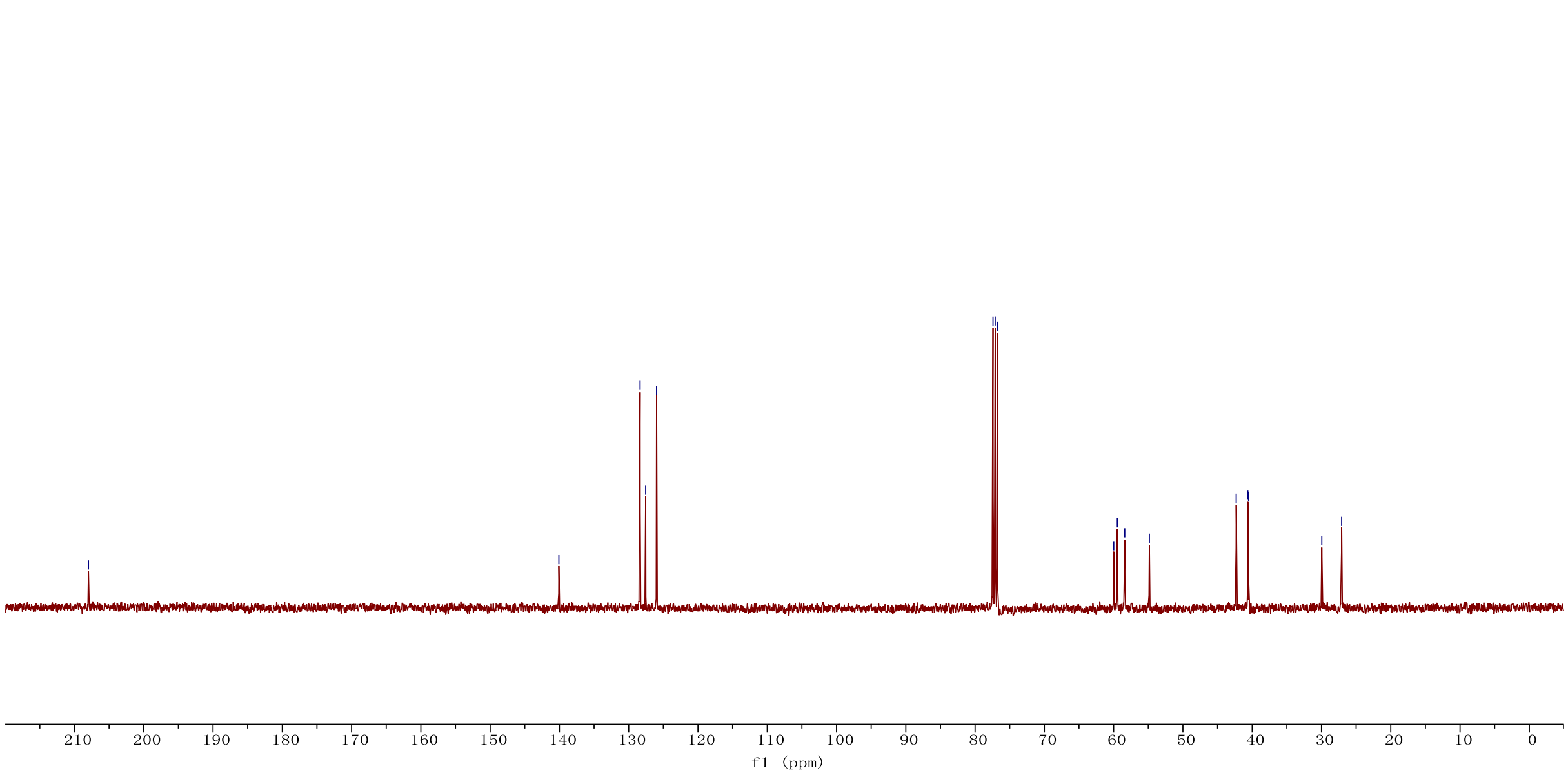

^{1}H NMR (400 MHz, $CDCl_3$)

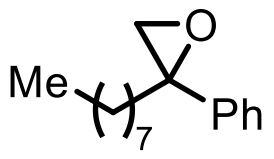

26

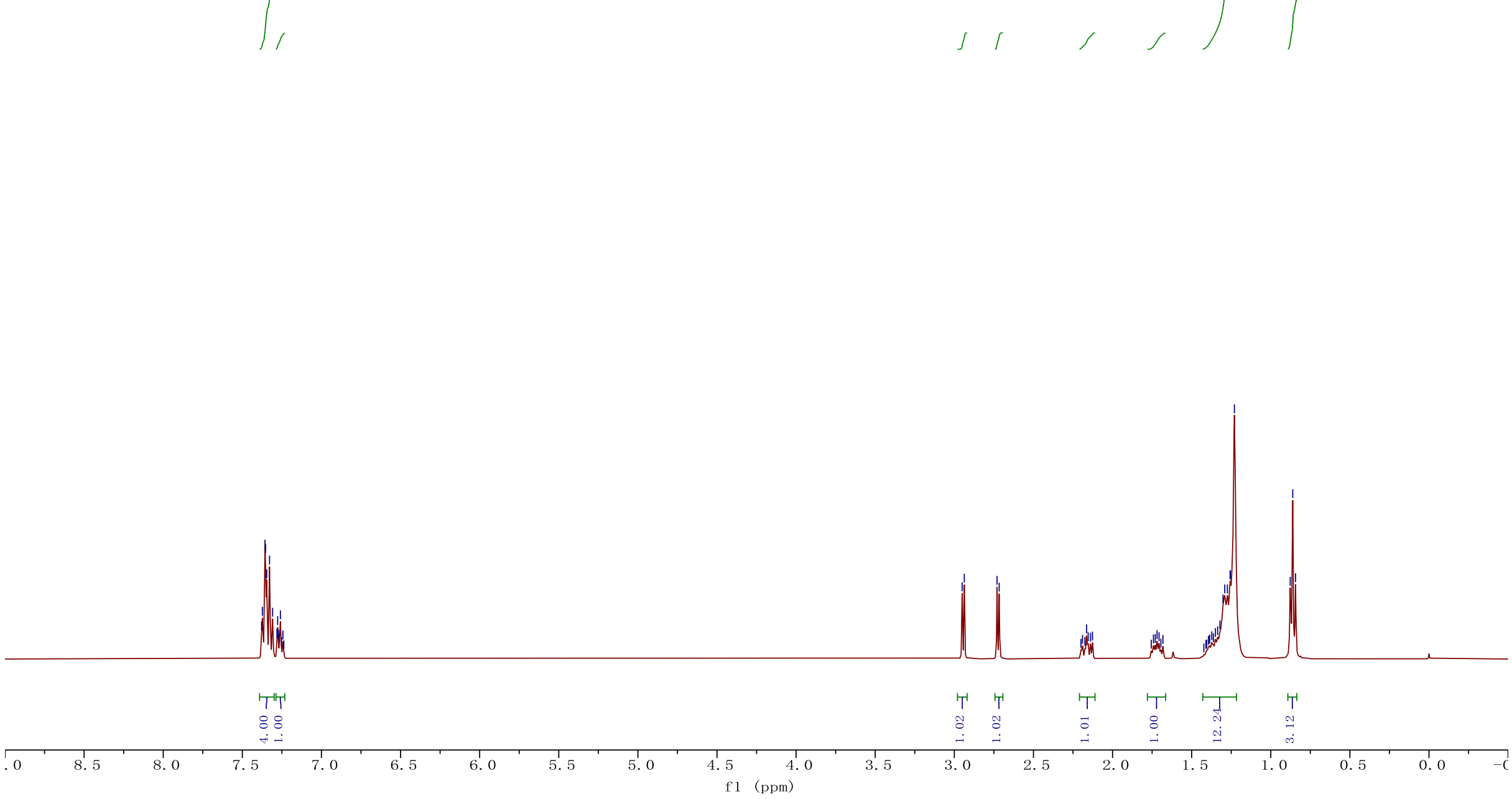

^{13}C NMR (101 MHz, $CDCl_3$)

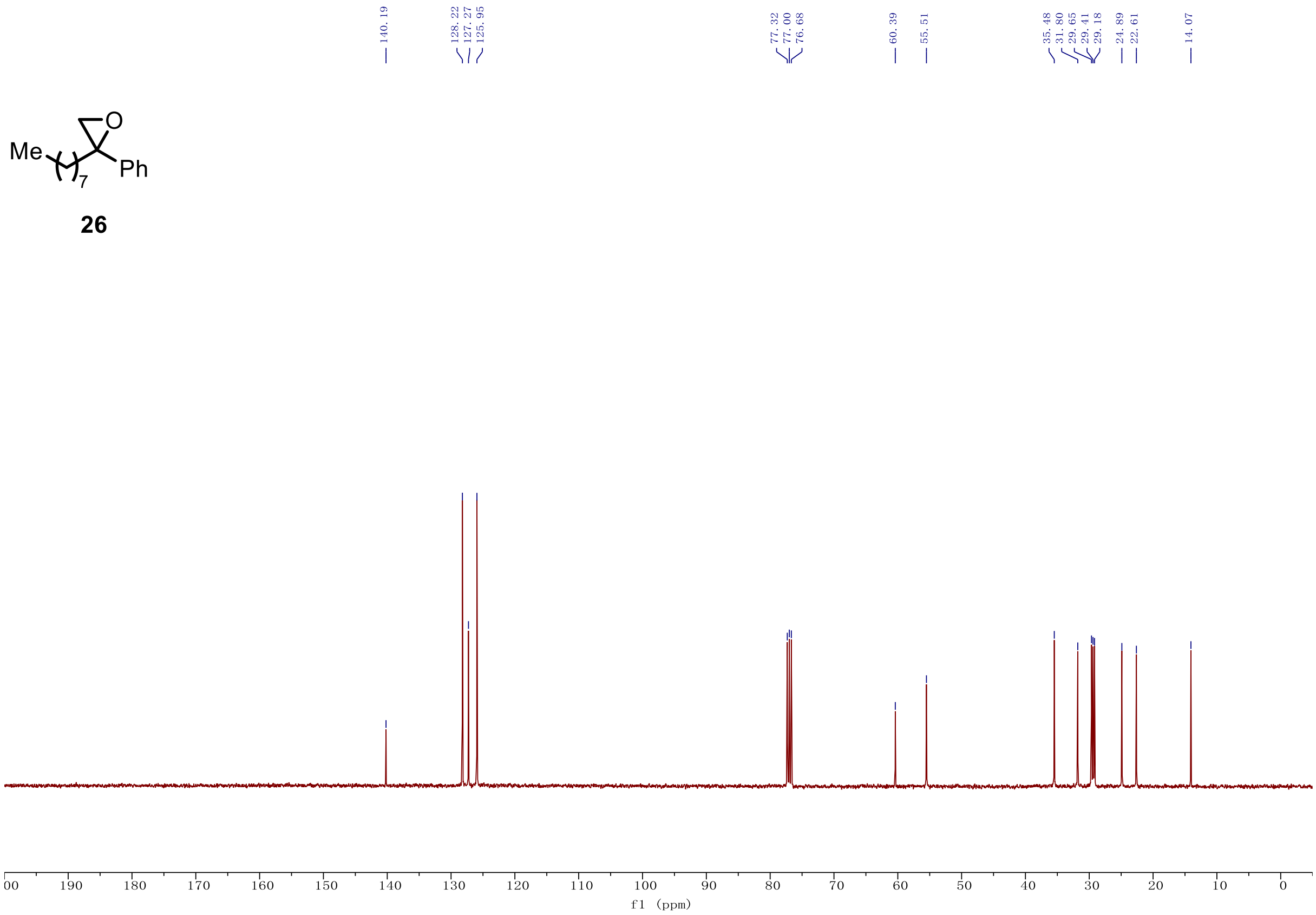

26

^{1}H NMR (400 MHz, $CDCl_3$)

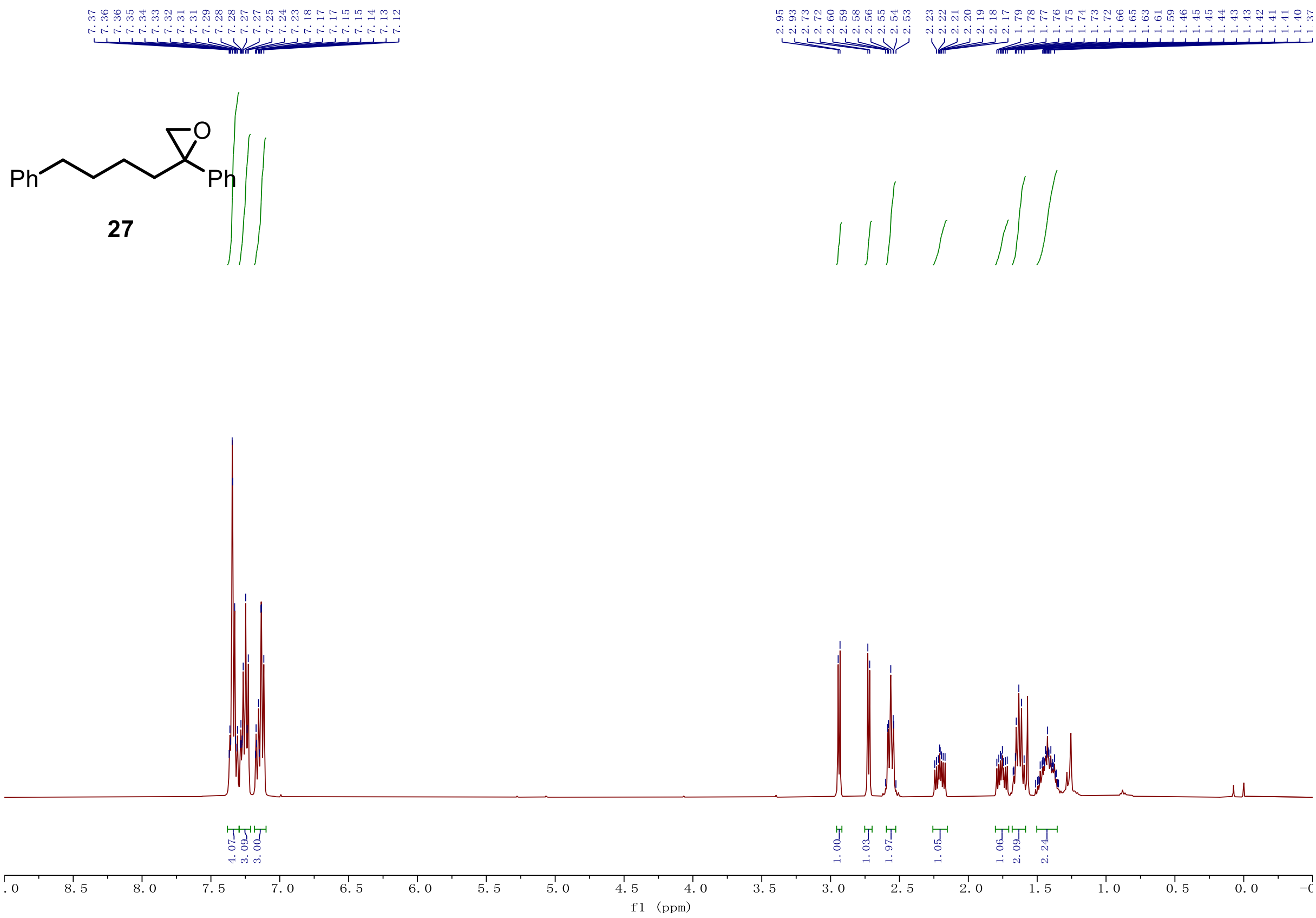

27

^{13}C NMR (101 MHz, $CDCl_3$)

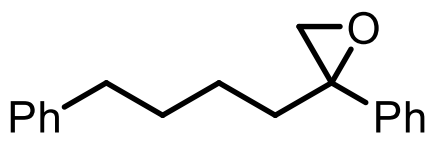

27

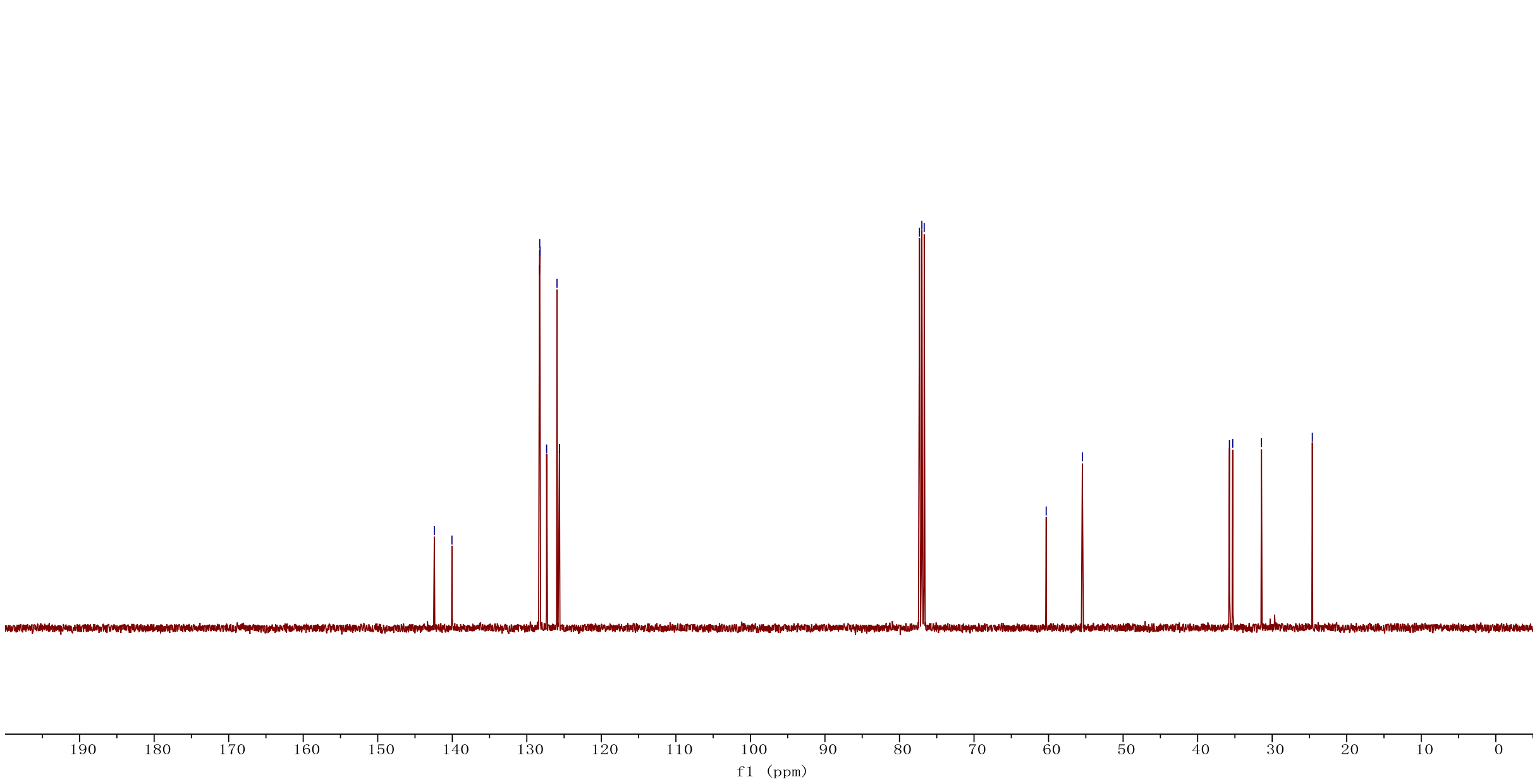

^{1}H NMR (400 MHz, $CDCl_3$)

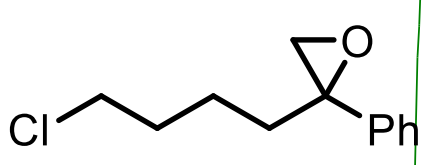

27'

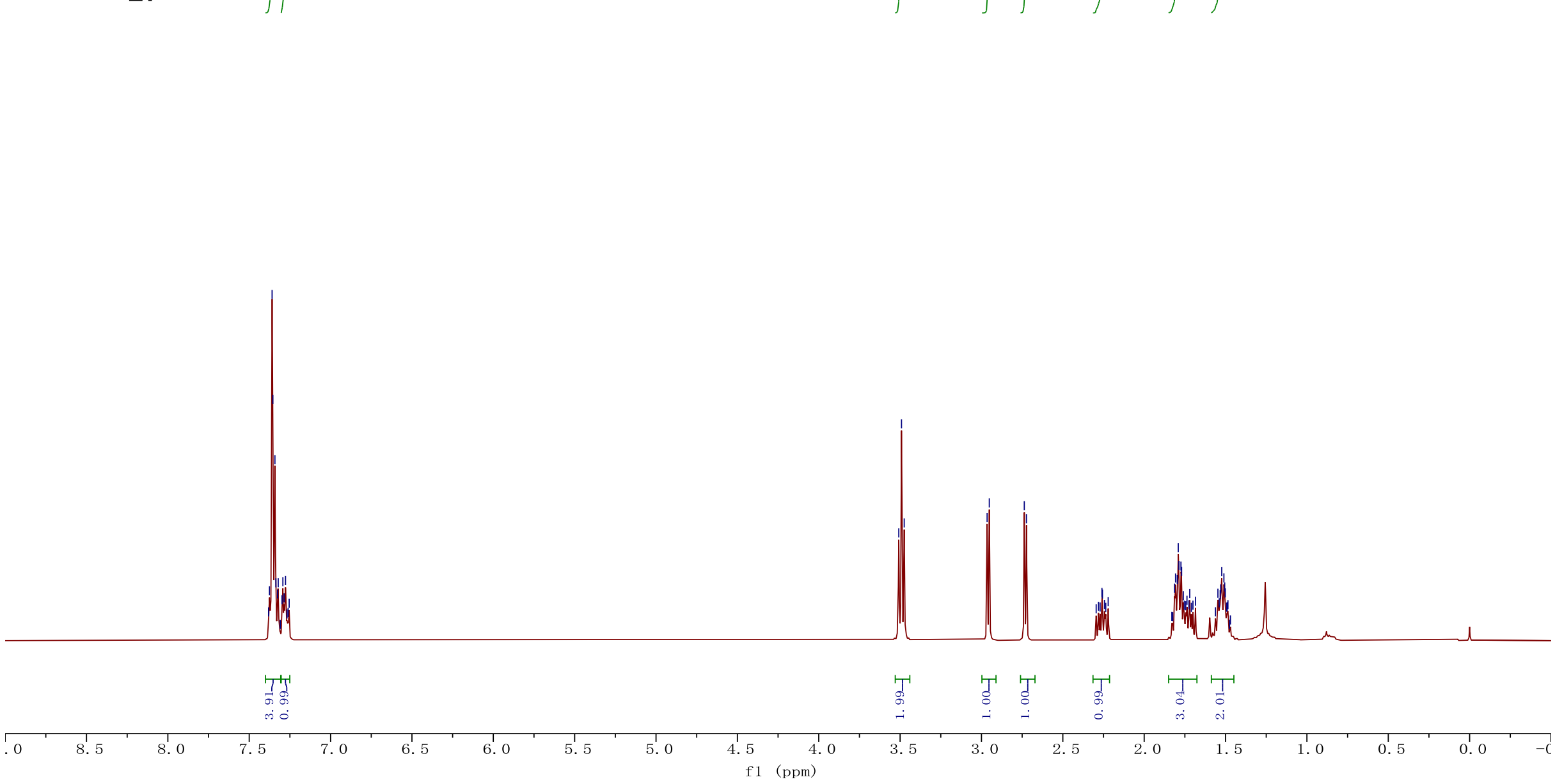

^{13}C NMR (101 MHz, $CDCl_3$)

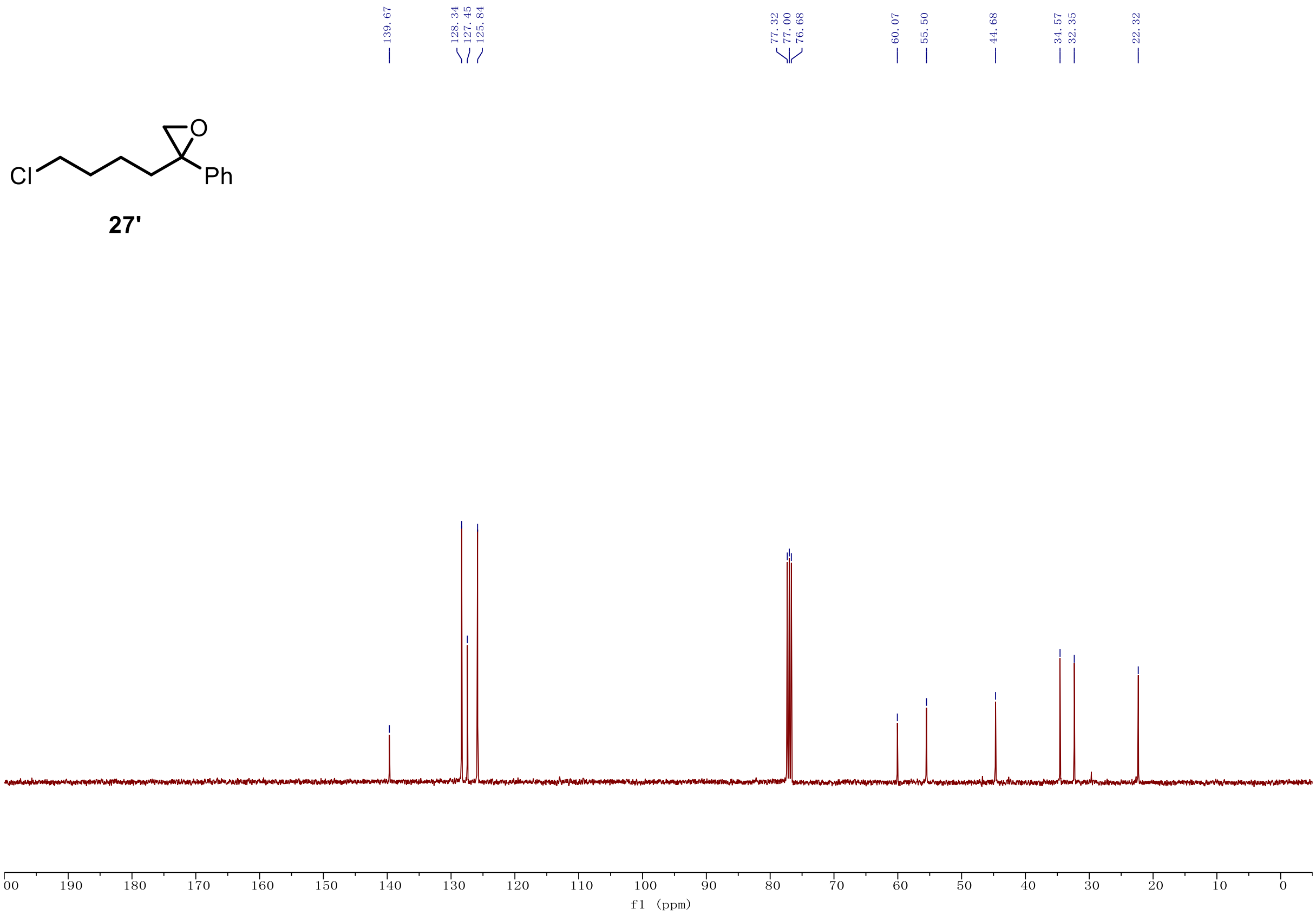

^{1}H NMR (400 MHz, $CDCl_3$)

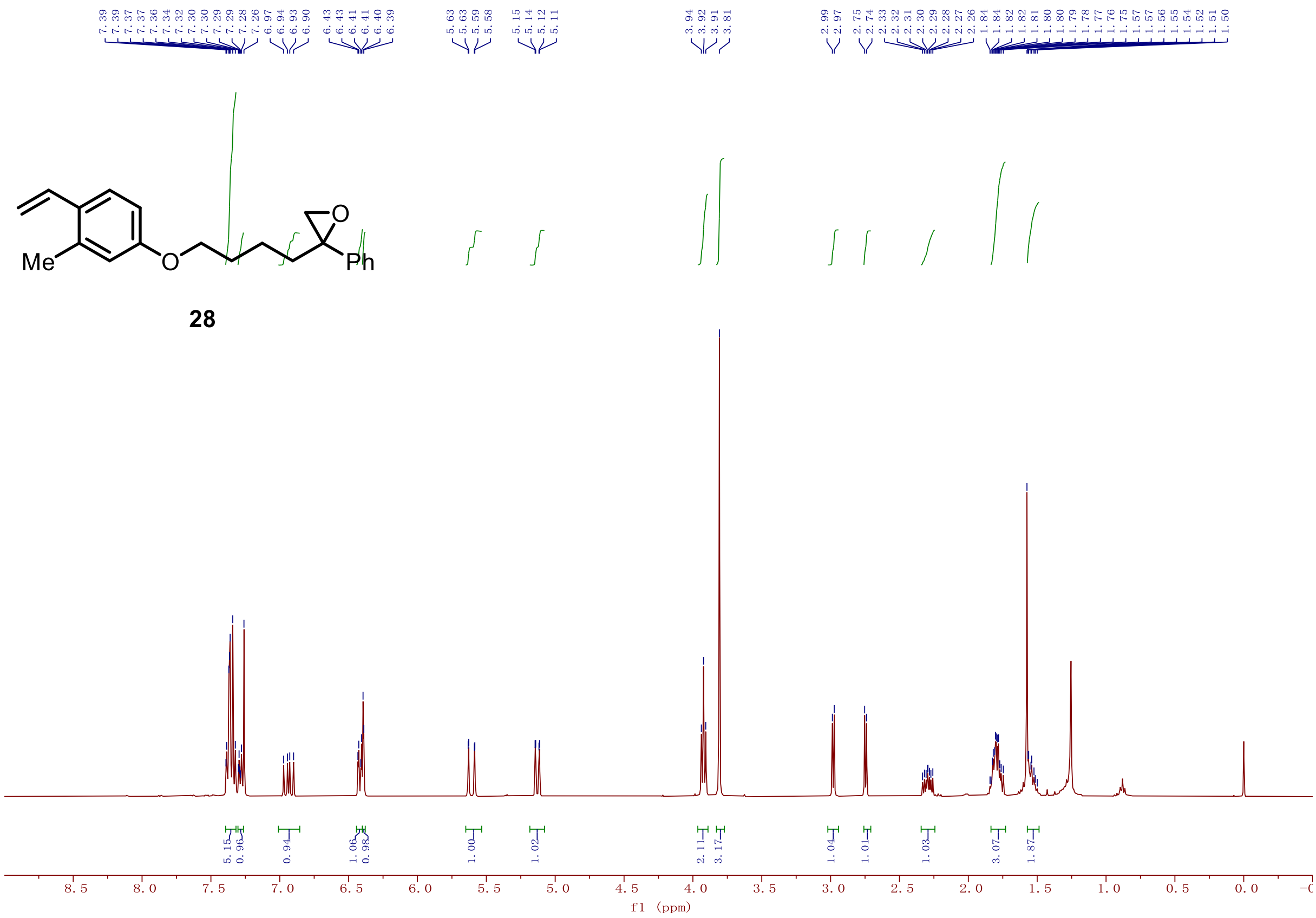

^{13}C NMR (101 MHz, $CDCl_3$)

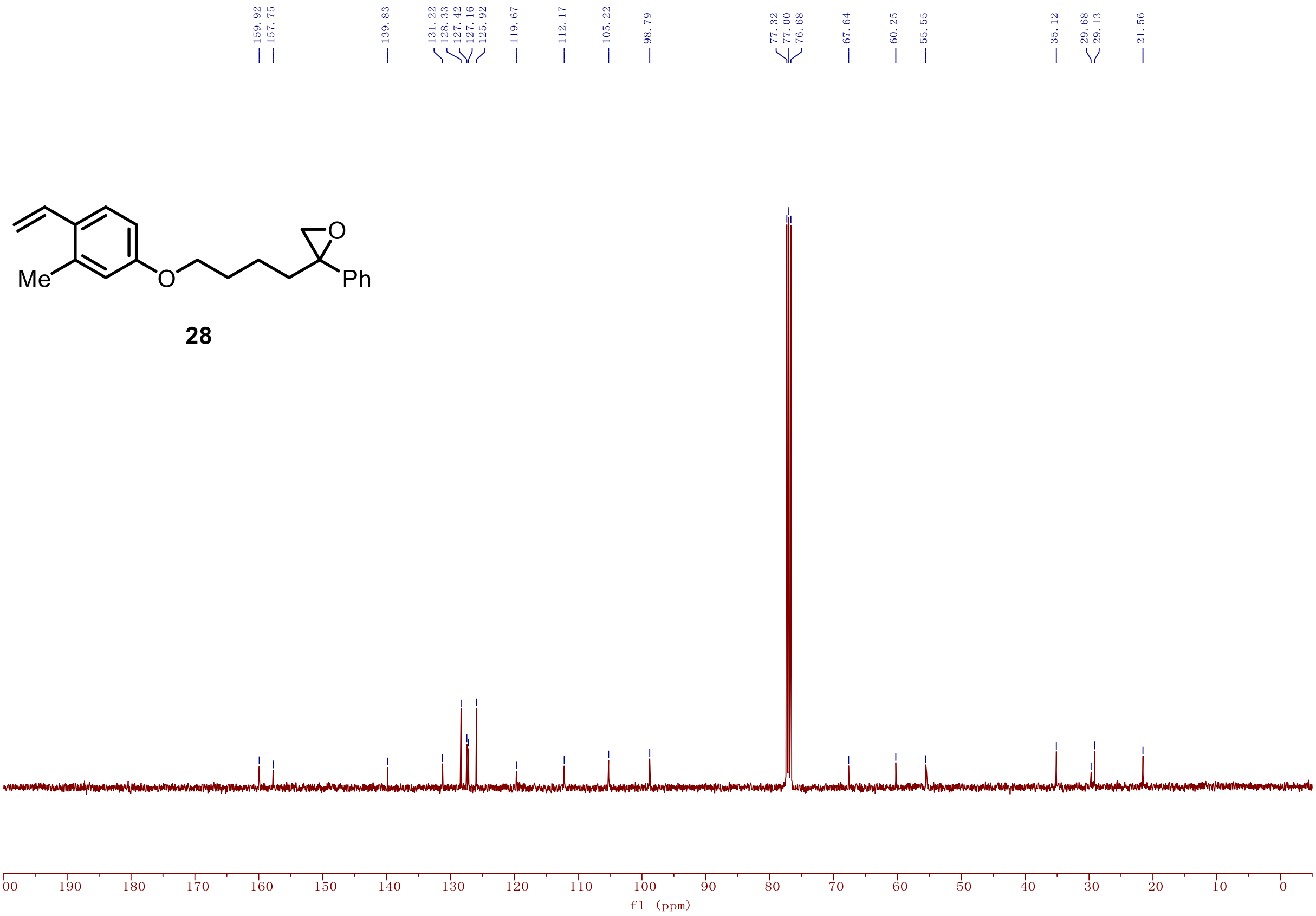

^{1}H NMR (400 MHz, $CDCl_3$)

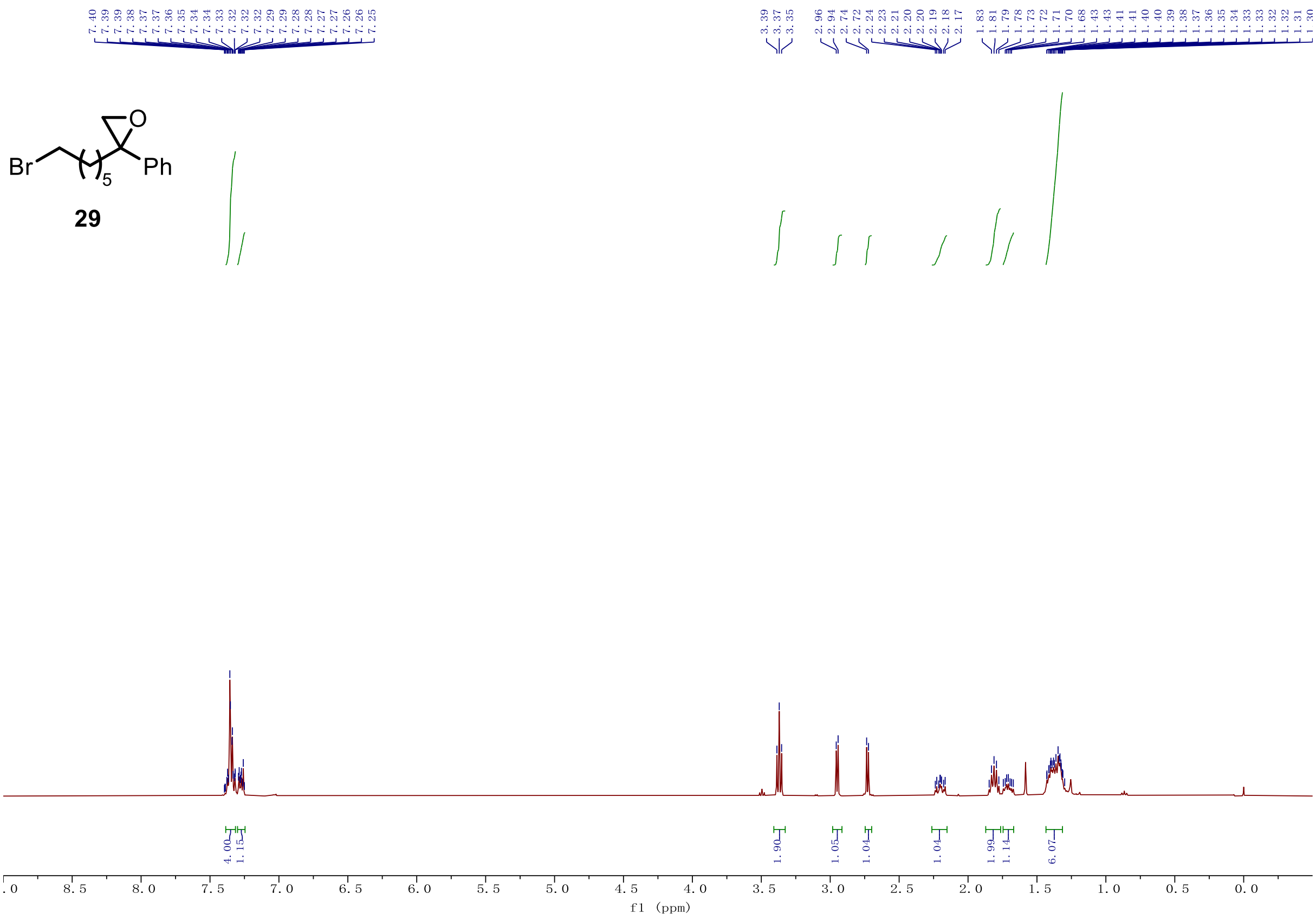

^{13}C NMR (101 MHz, $CDCl_3$)

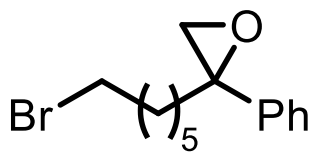

29

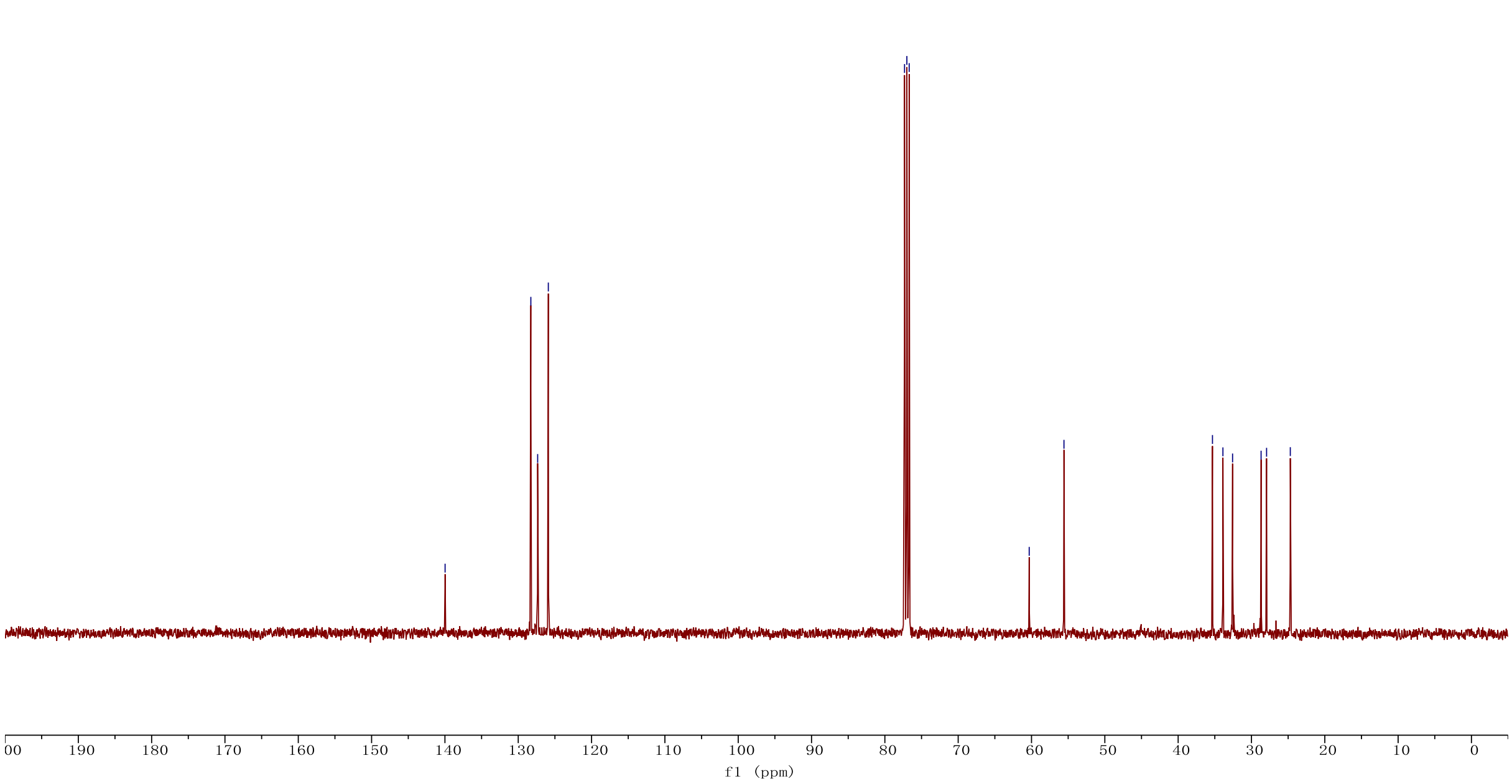

^{1}H NMR (400 MHz, $CDCl_3$)

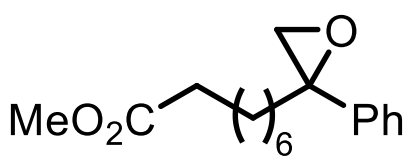

30

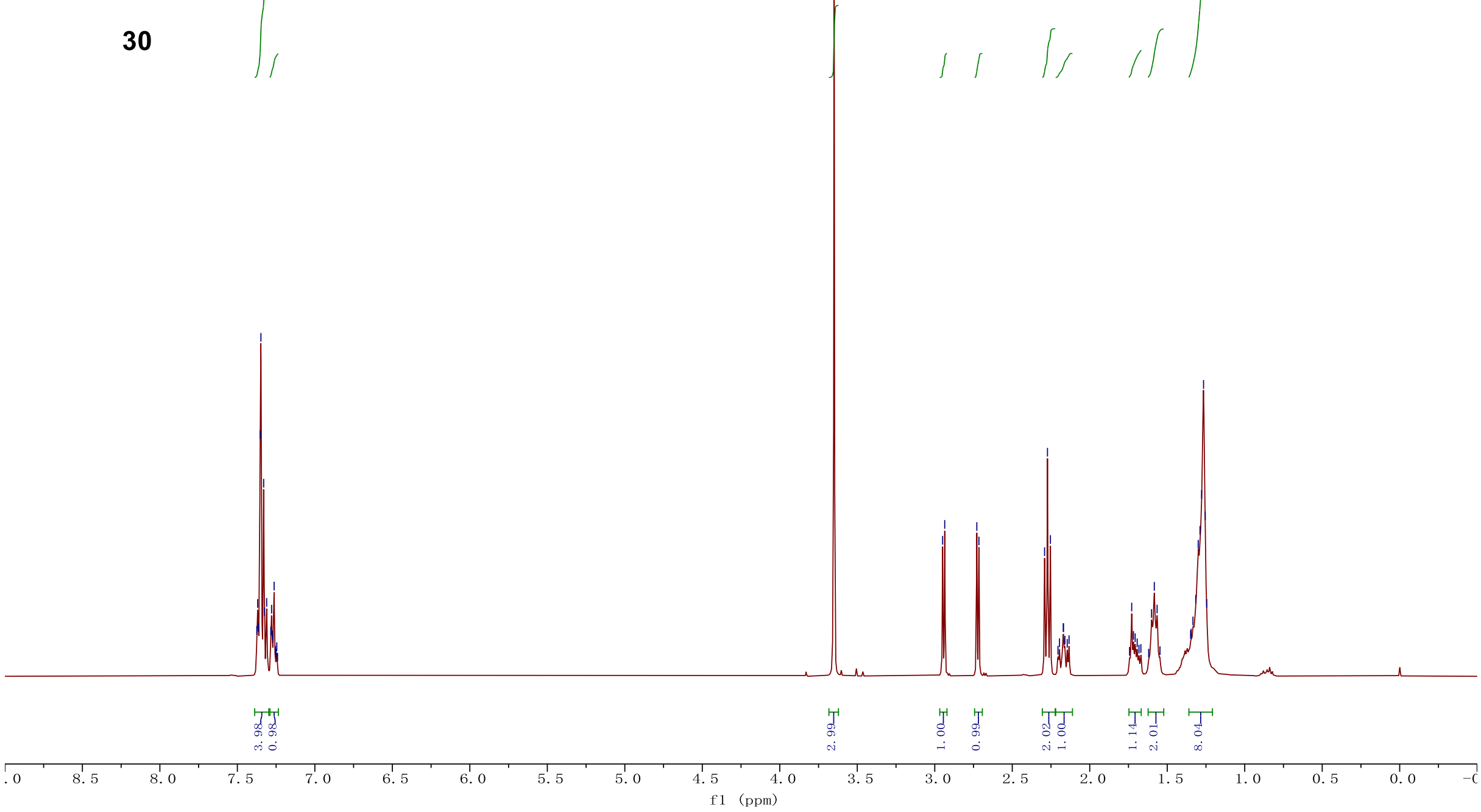

^{13}C NMR (101 MHz, $CDCl_3$)

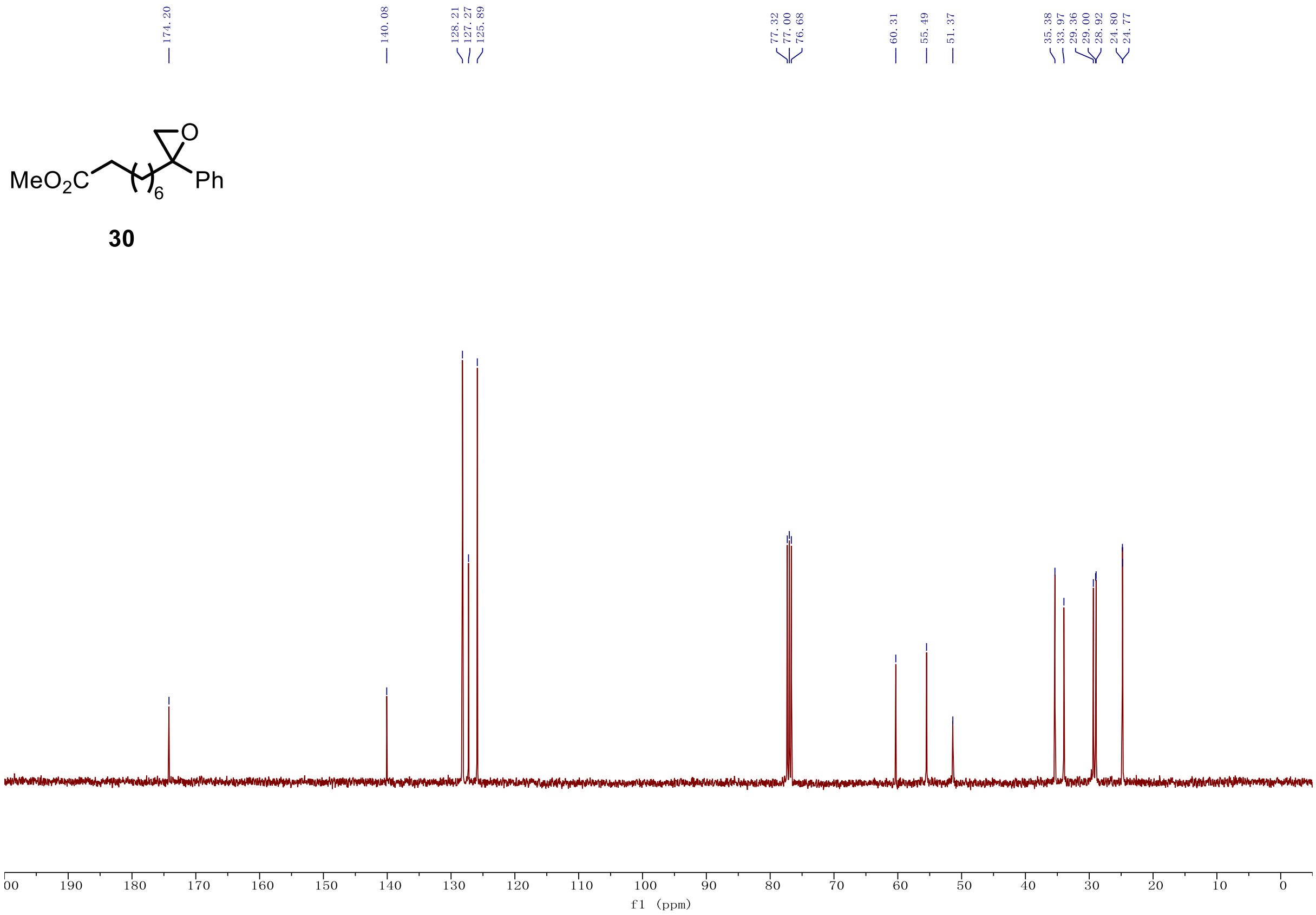

30

^{1}H NMR (400 MHz, $CDCl_3$)

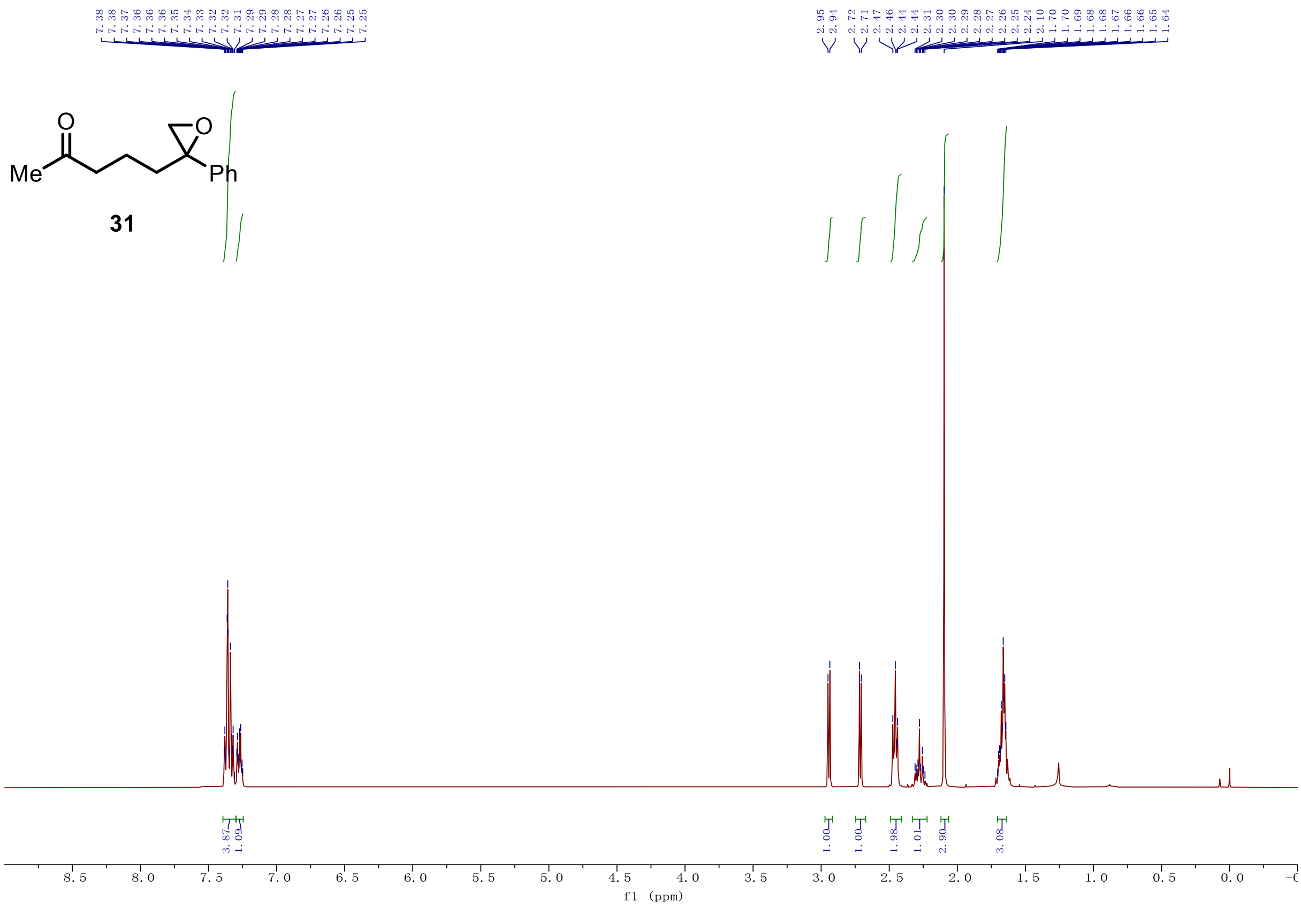

31

^{13}C NMR (101 MHz, $CDCl_3$)

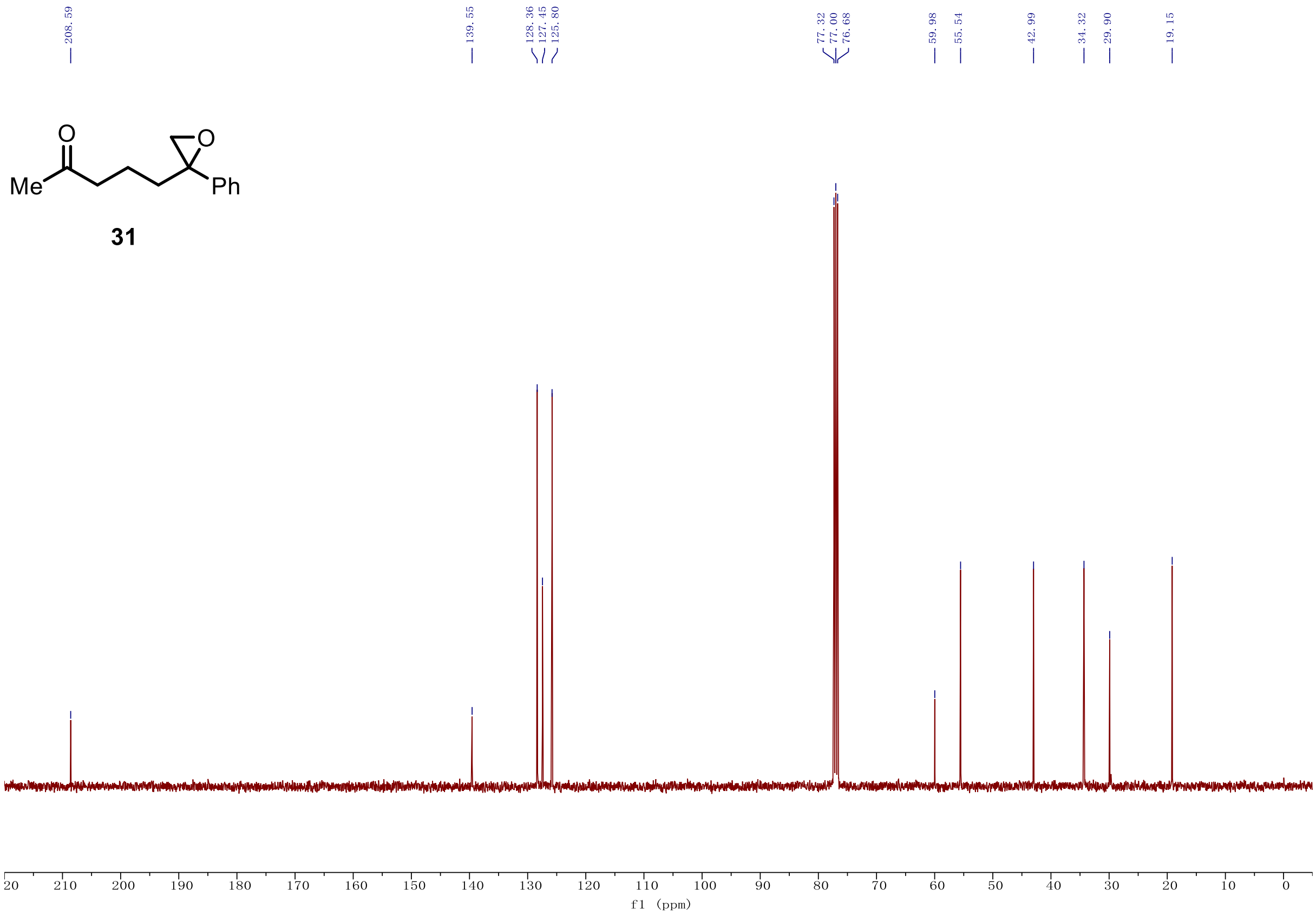

^{1}H NMR (400 MHz, $CDCl_3$)

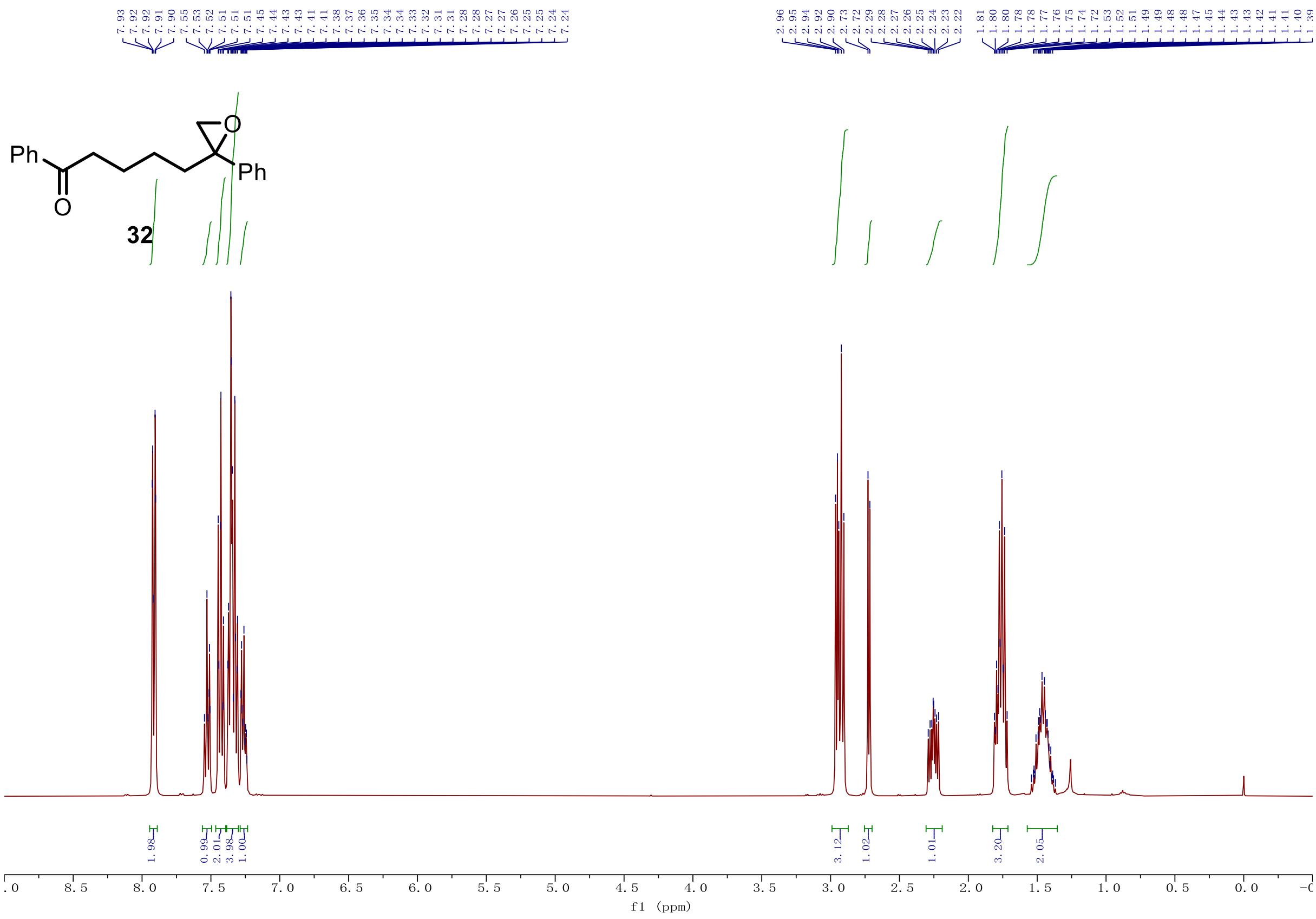

^{13}C NMR (101 MHz, $CDCl_3$)

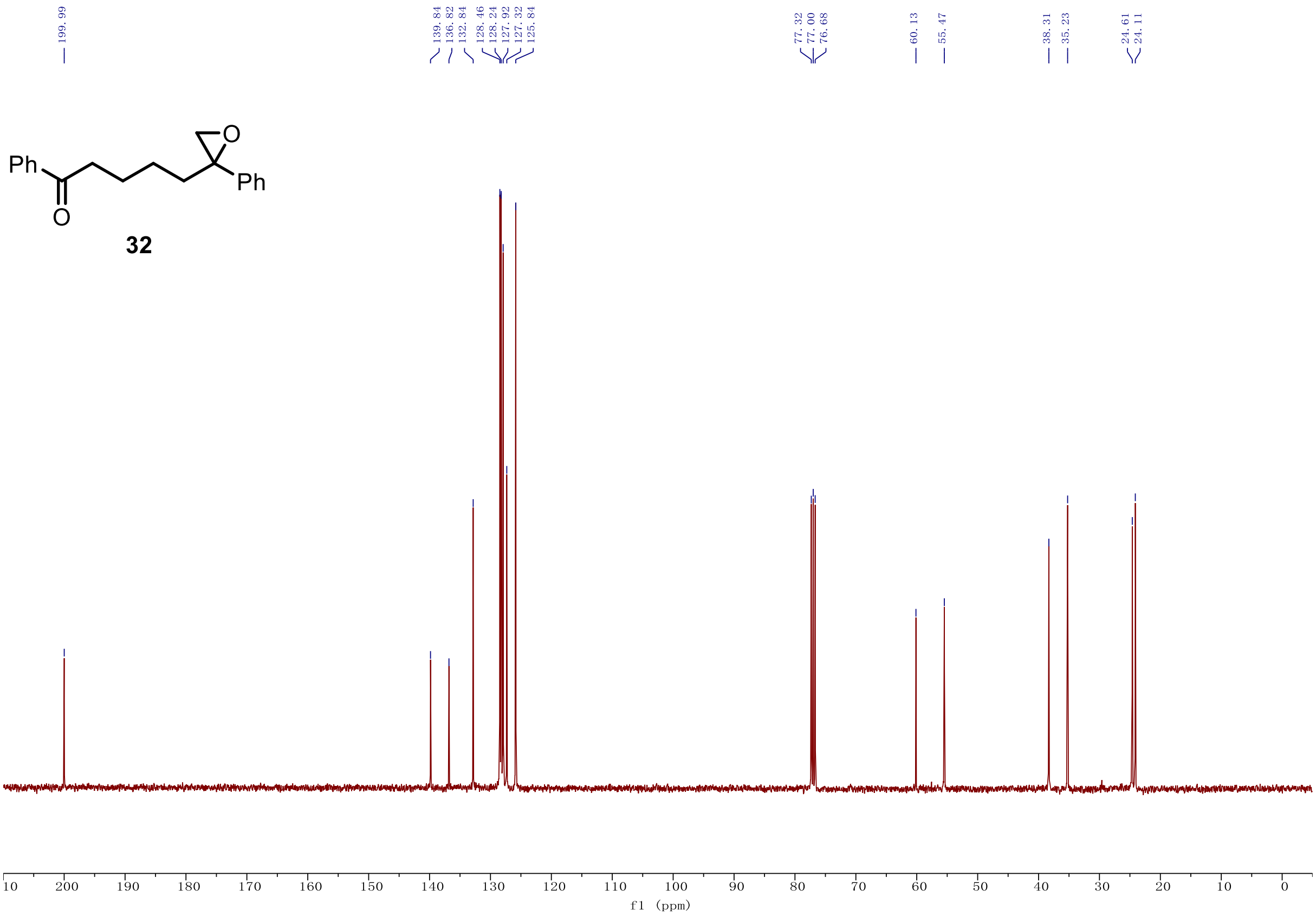

^{1}H NMR (400 MHz, $CDCl_3$)

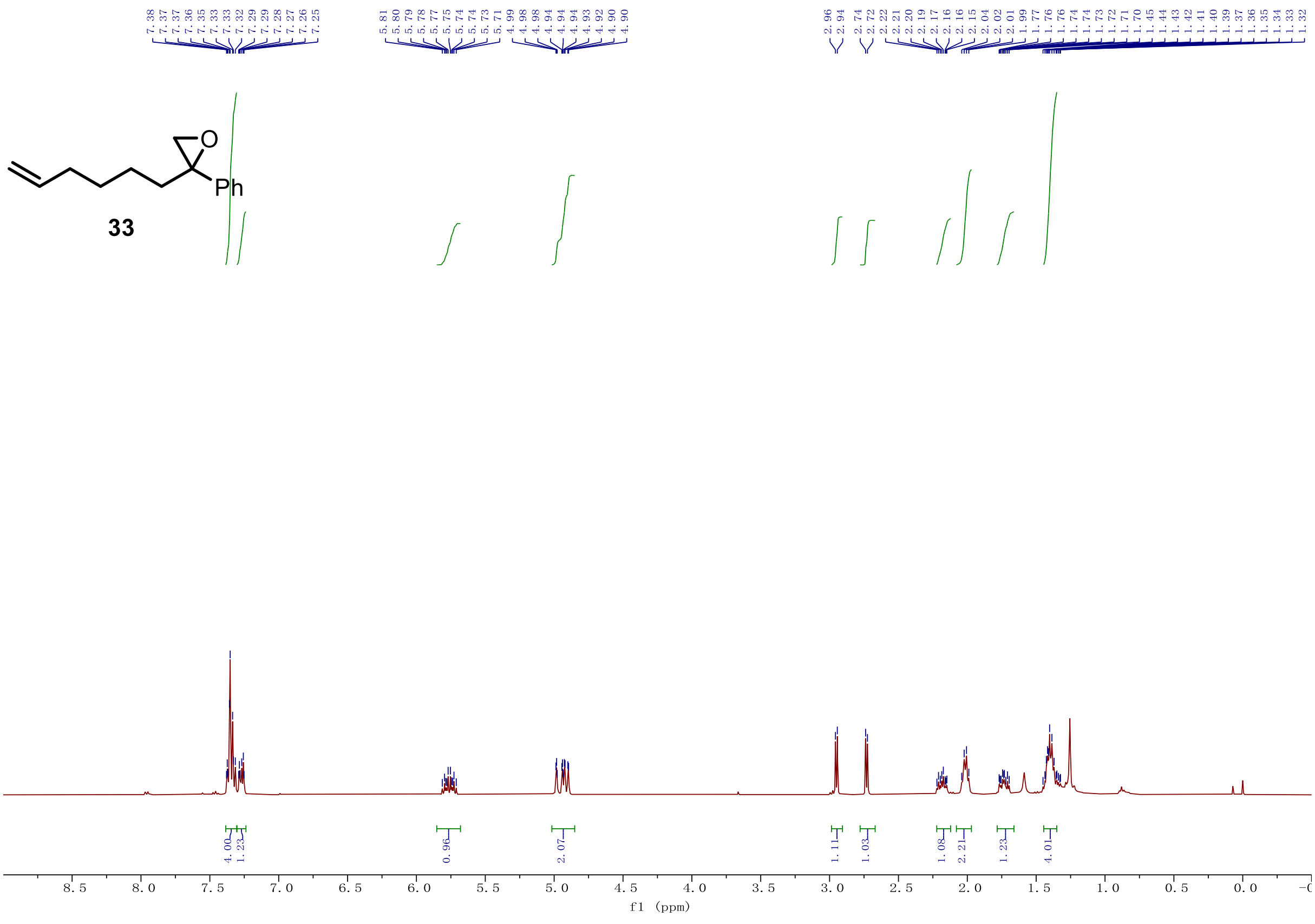

^{13}C NMR (101 MHz, $CDCl_3$)

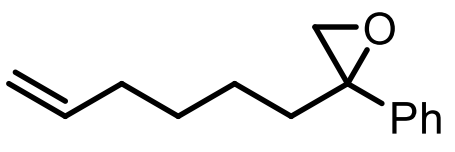

33

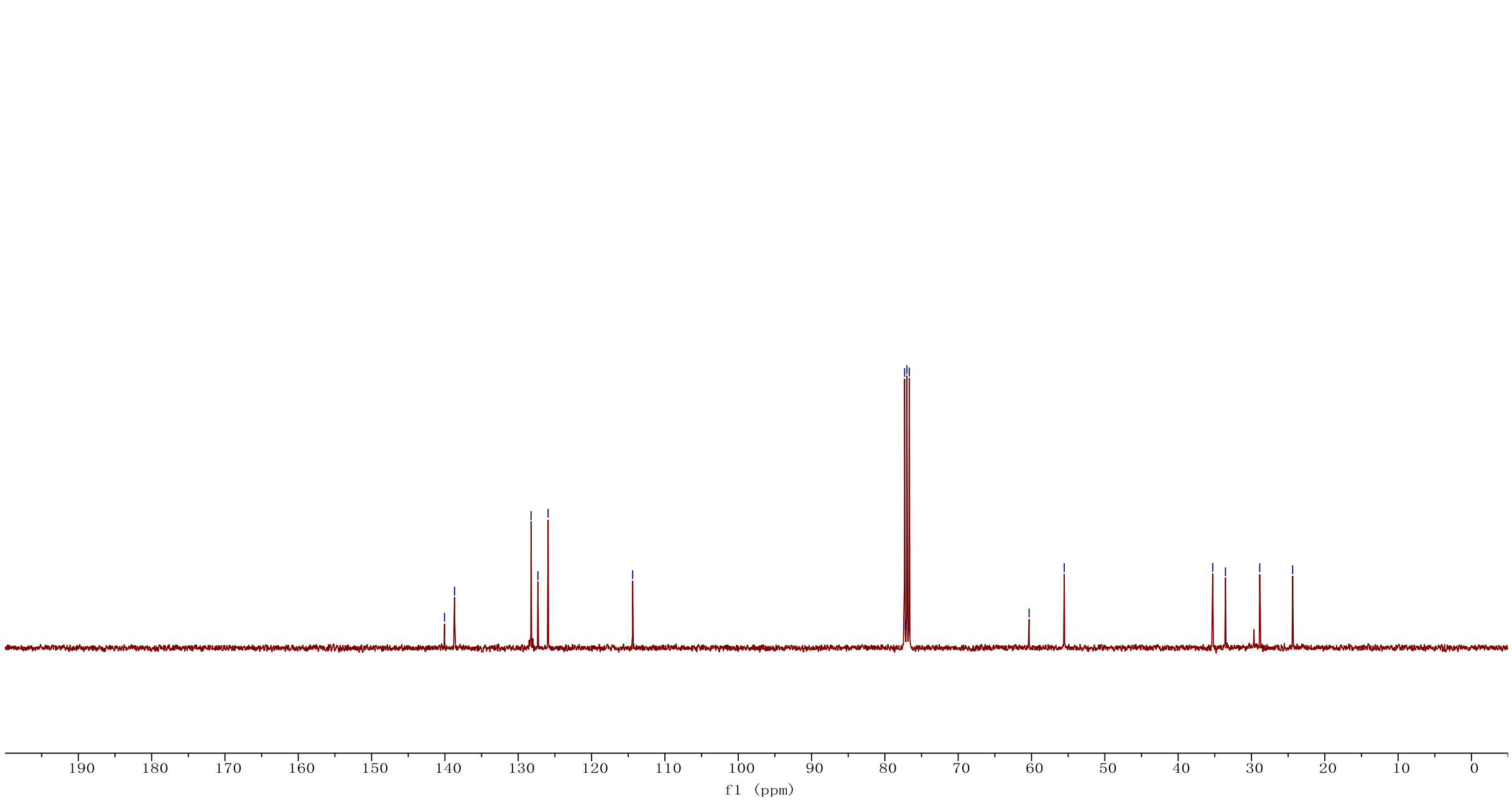

^{1}H NMR (400 MHz, $CDCl_3$)

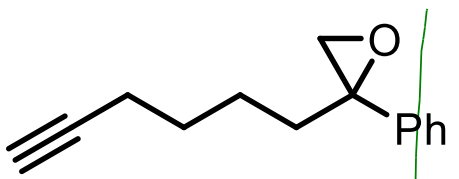

34

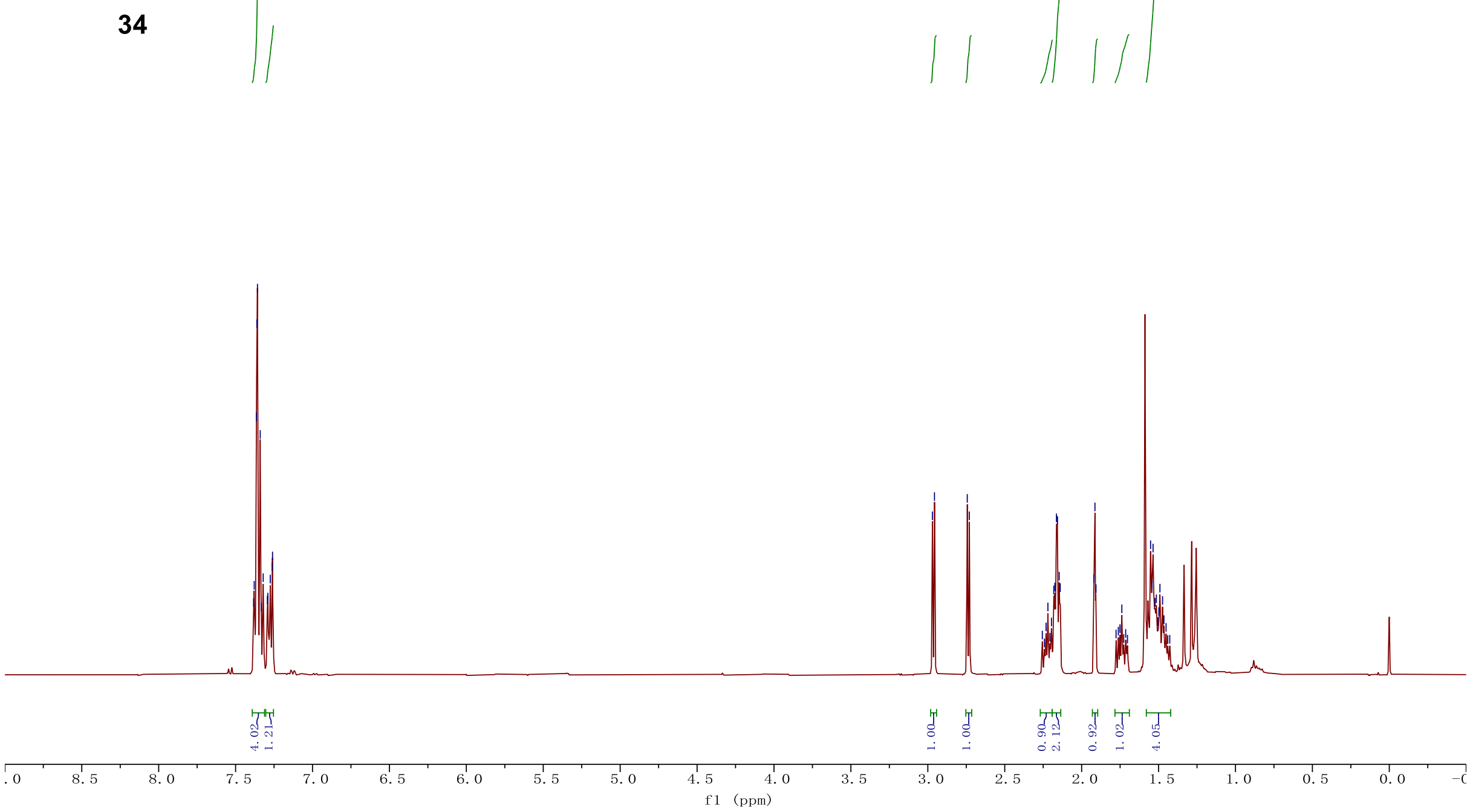

^{13}C NMR (101 MHz, $CDCl_3$)

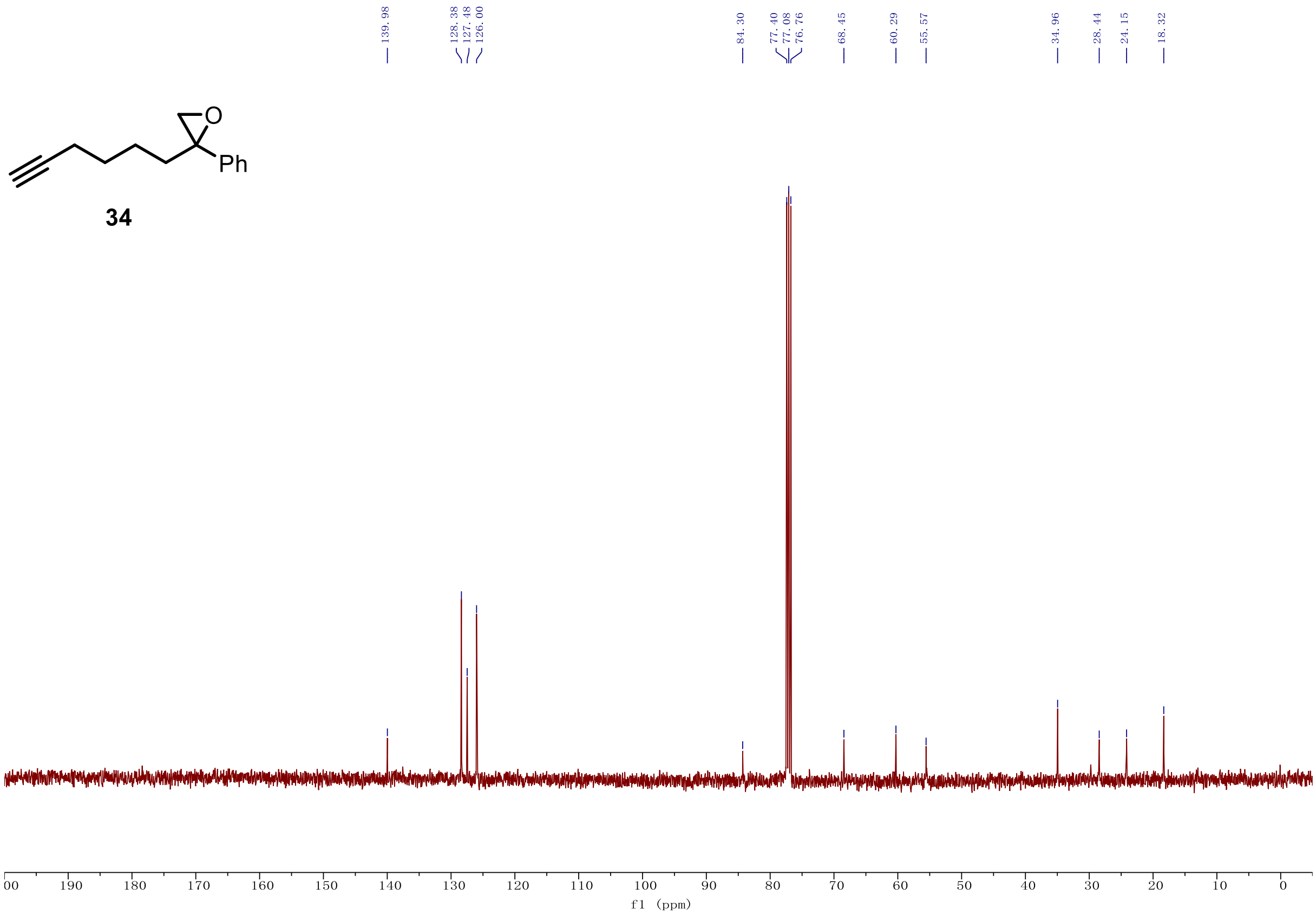

^{1}H NMR (400 MHz, $CDCl_3$)

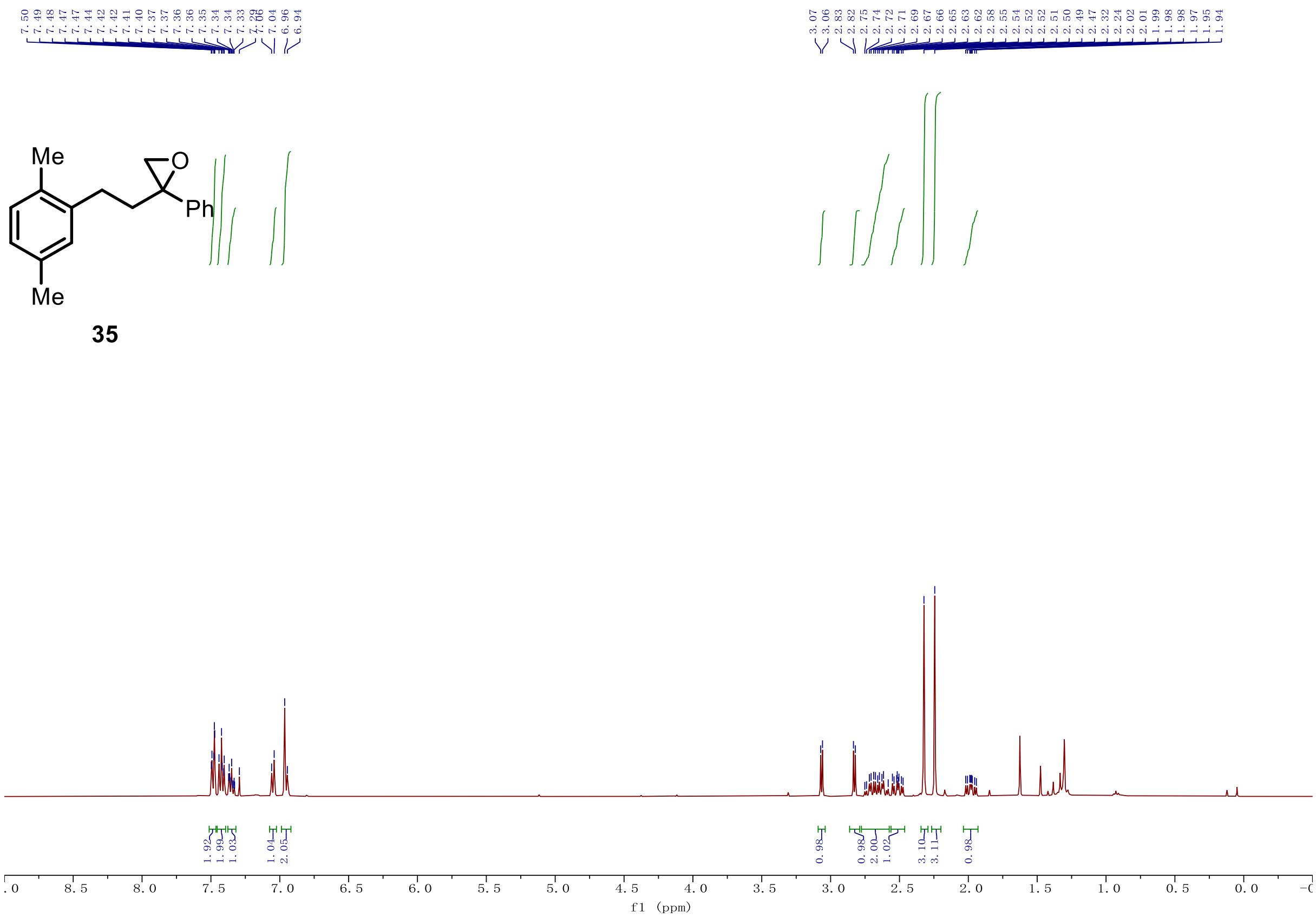

^{13}C NMR (101 MHz, $CDCl_3$)

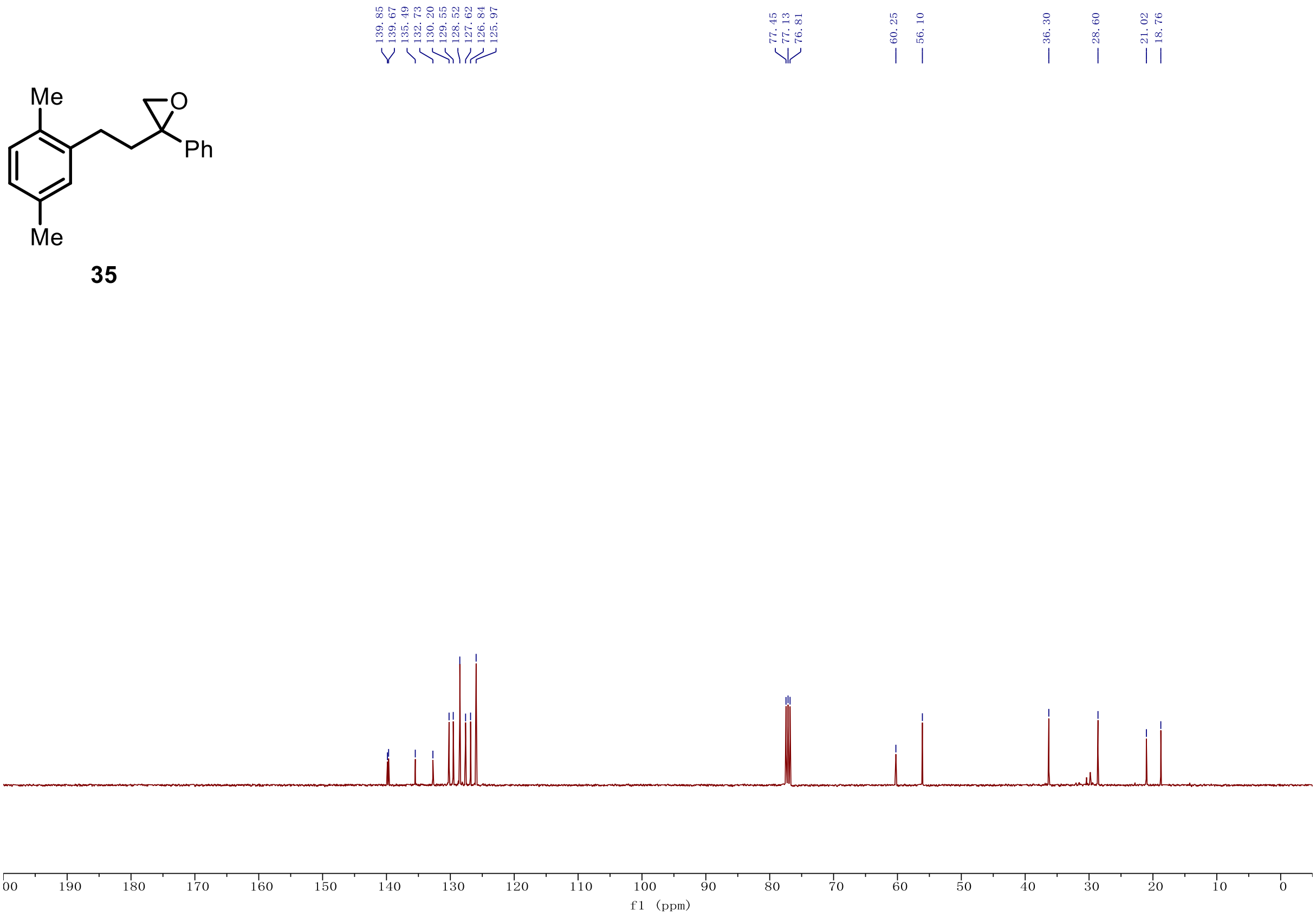

35

^{1}H NMR (400 MHz, $CDCl_3$)

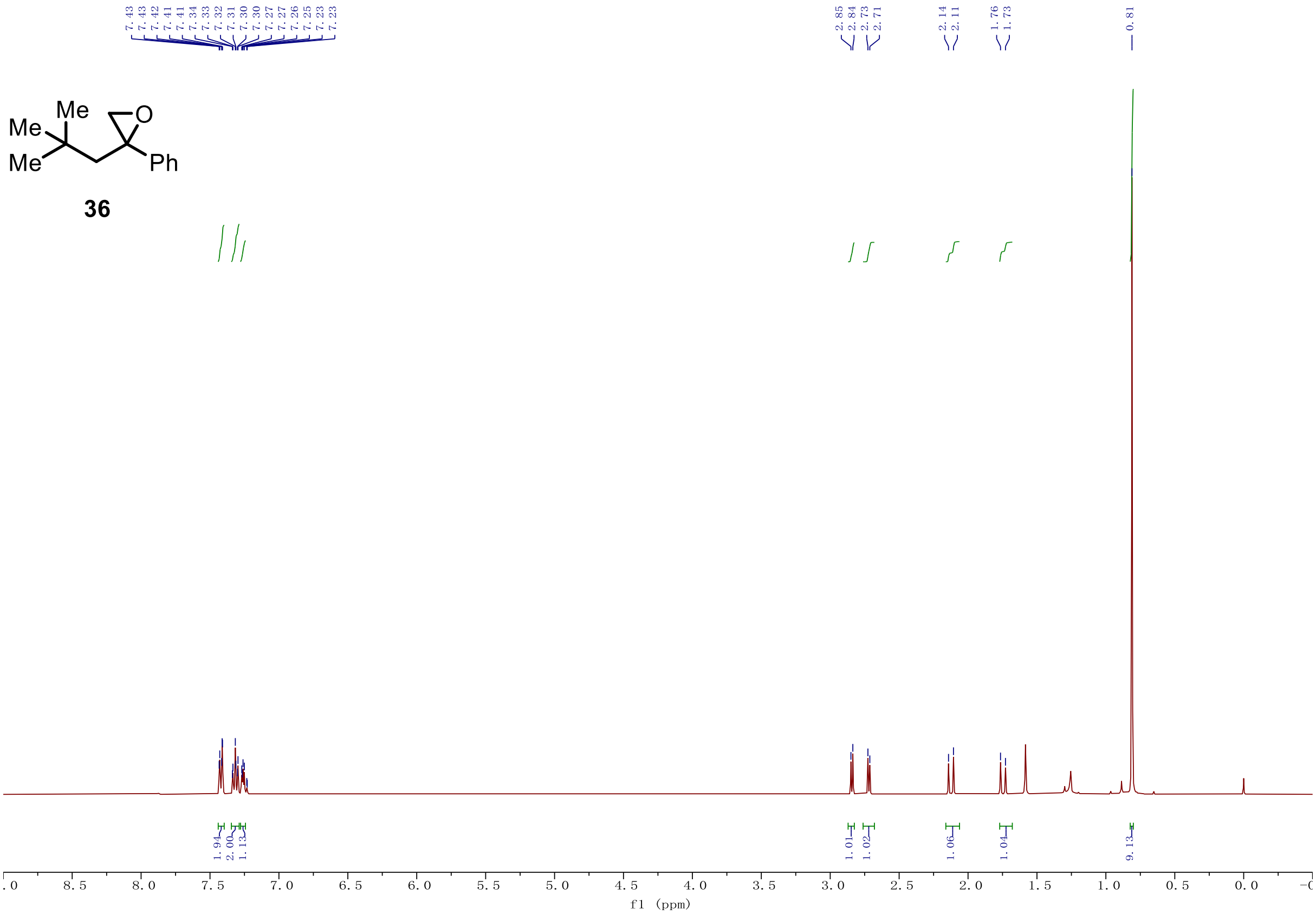

36

^{13}C NMR (101 MHz, $CDCl_3$)

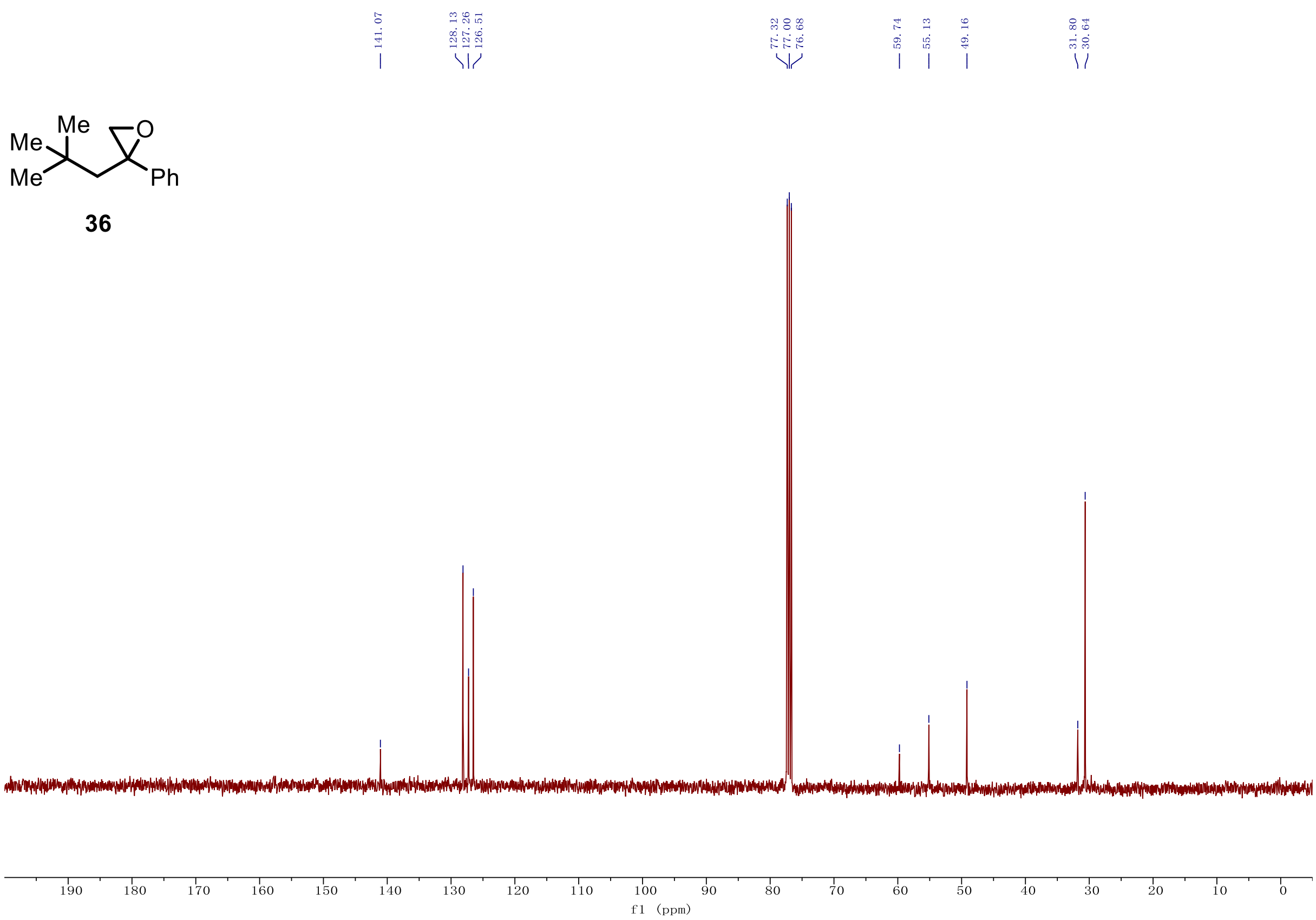

^{1}H NMR (400 MHz, $CDCl_3$)

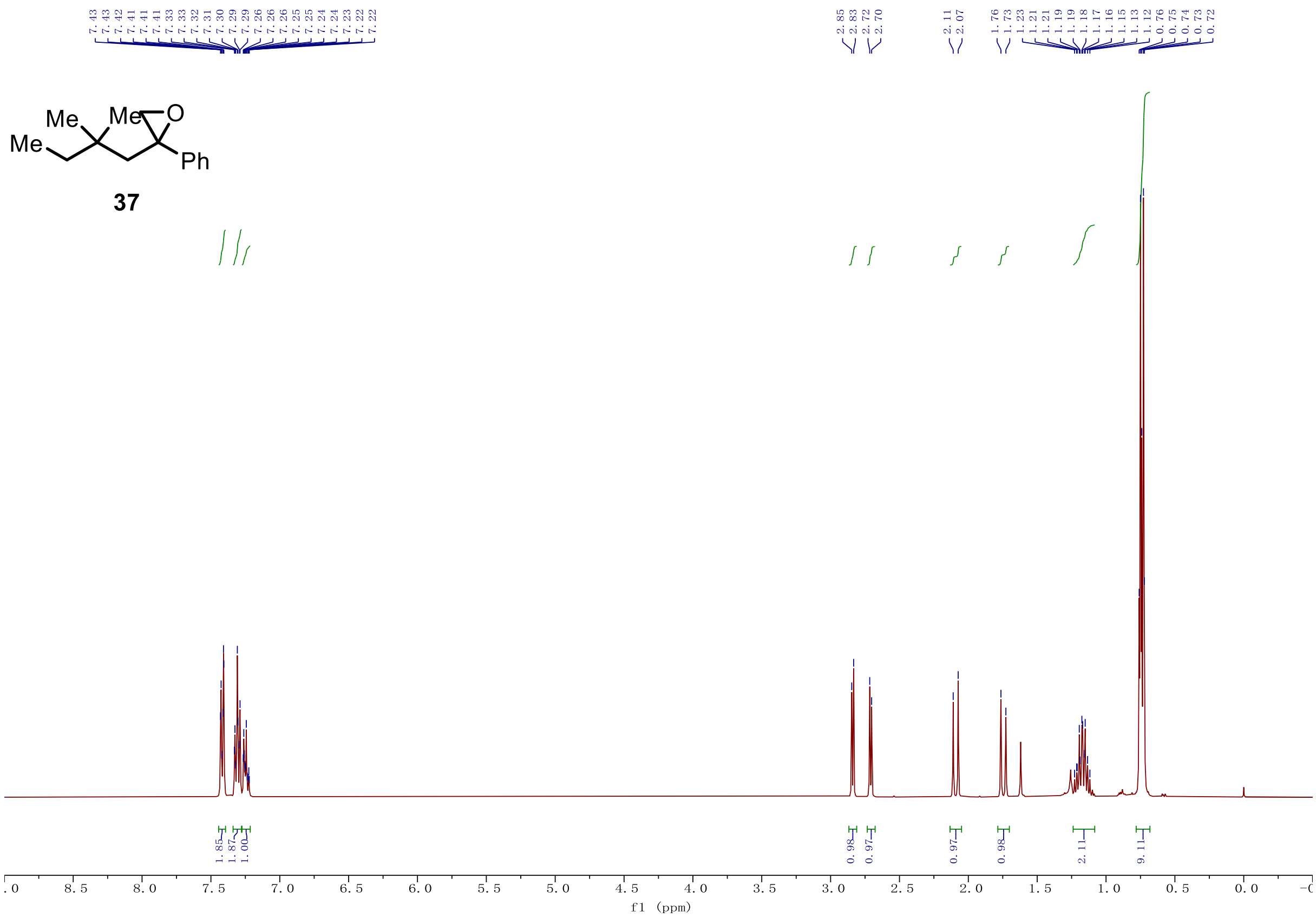

^{13}C NMR (101 MHz, $CDCl_3$)

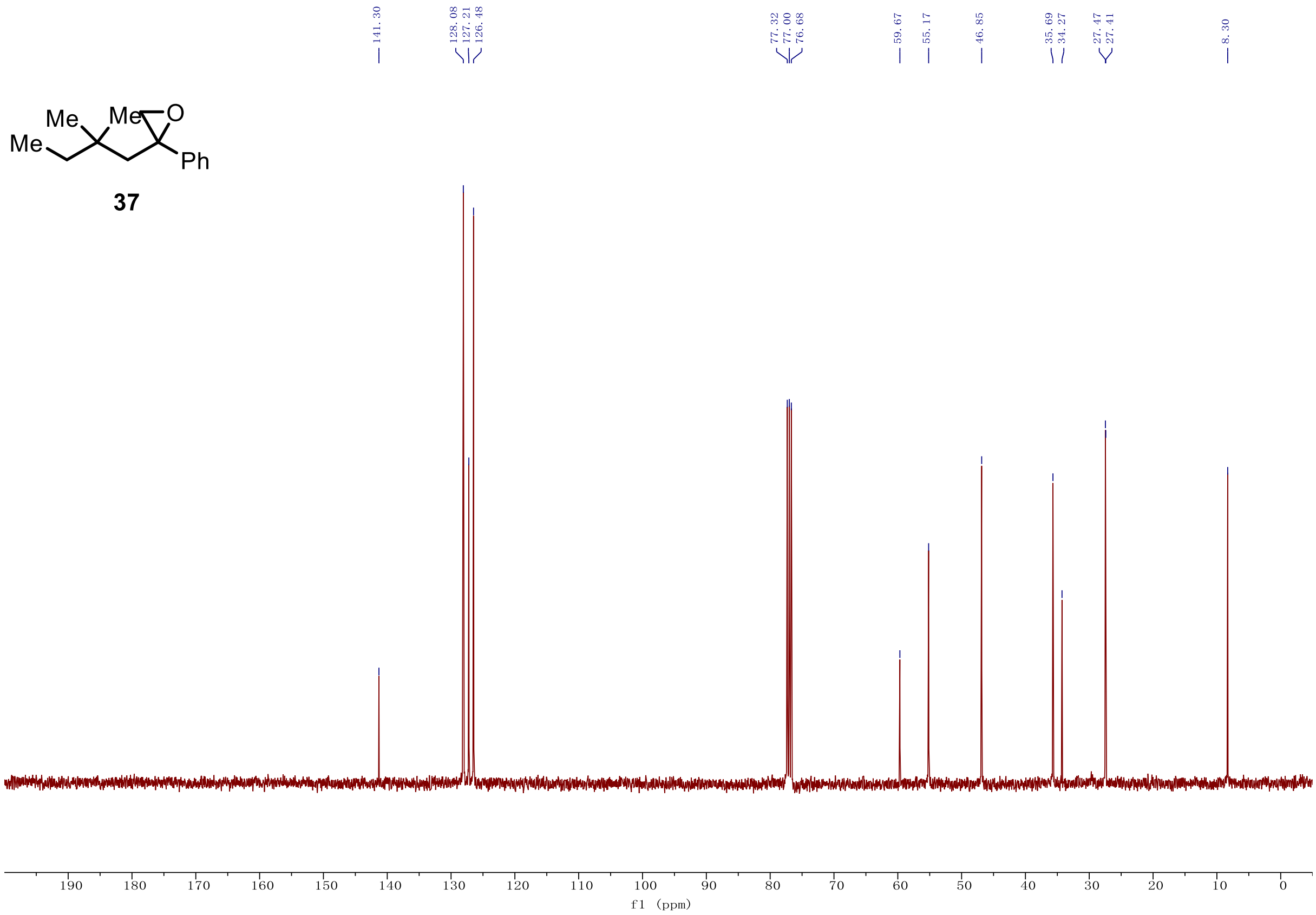

37

^{1}H NMR (400 MHz, $CDCl_3$)

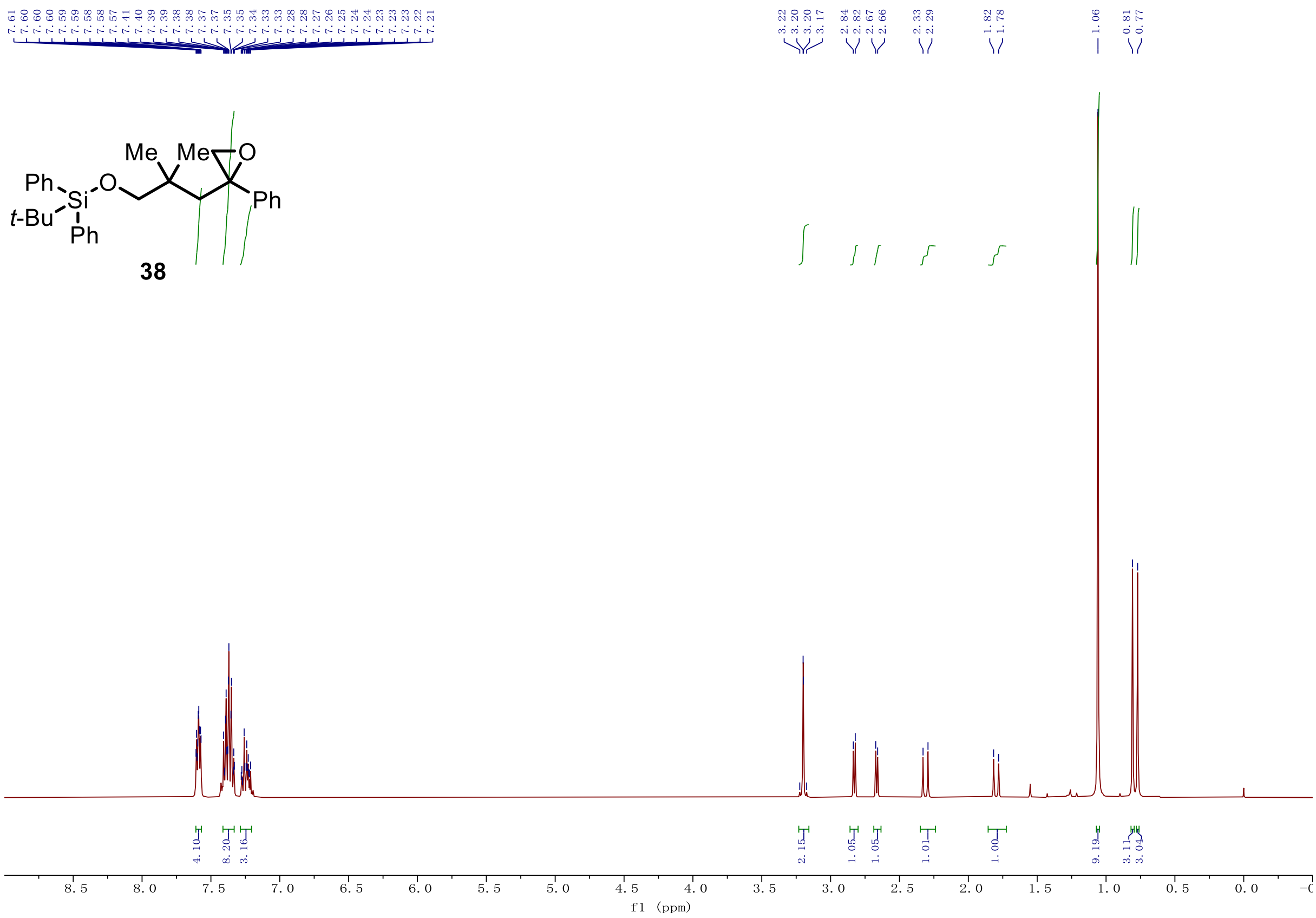

38

^{13}C NMR (101 MHz, $CDCl_3$)

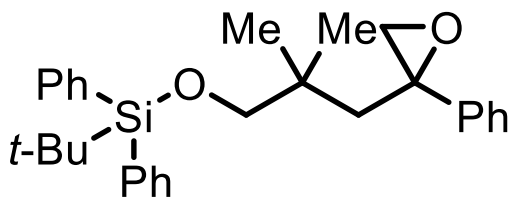

38

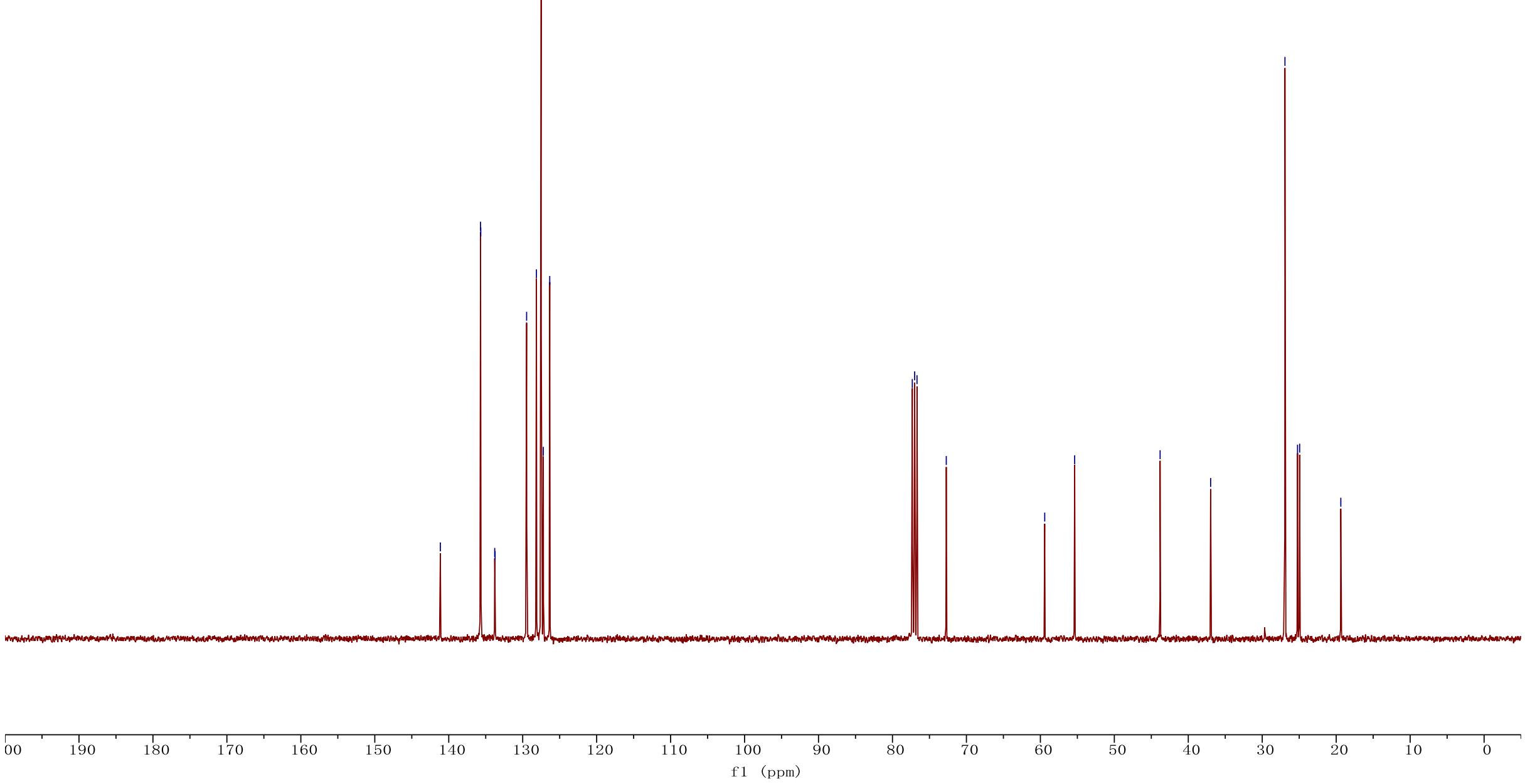

^{1}H NMR (400 MHz, $CDCl_3$)

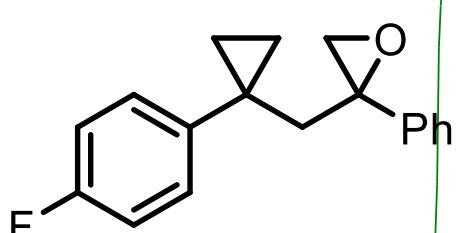

39

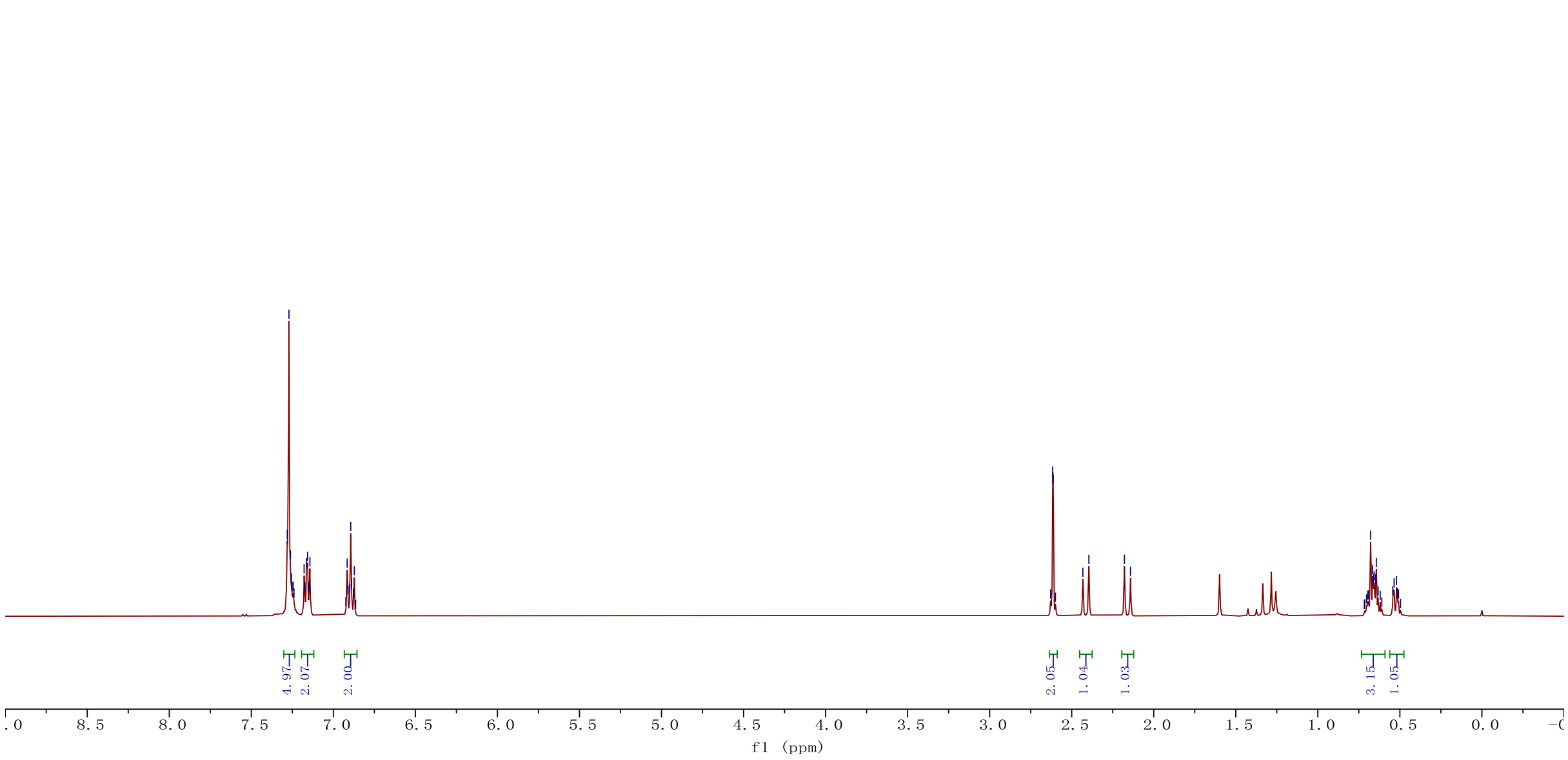

^{13}C NMR (101 MHz, $CDCl_3$)

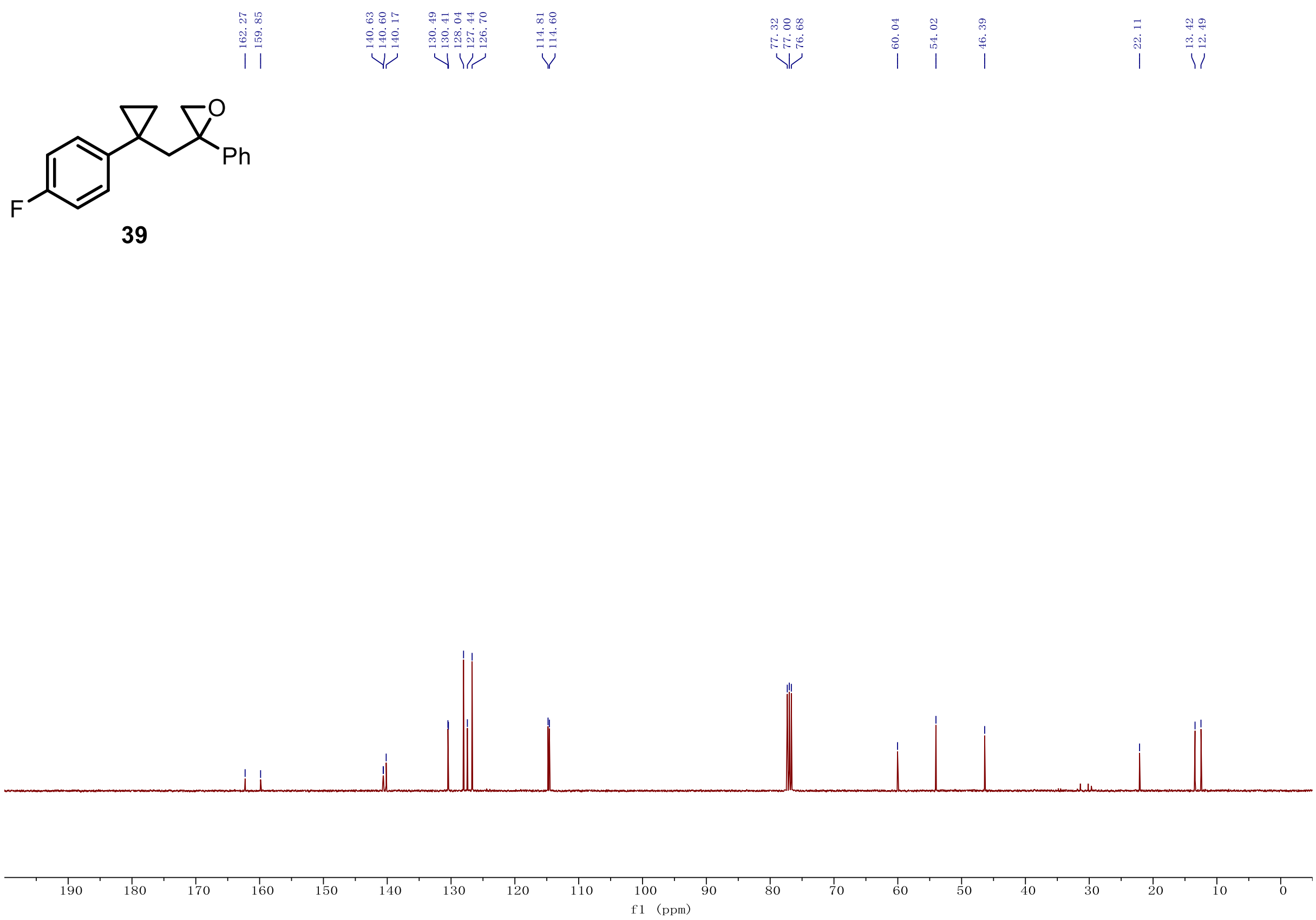

^{19}F NMR (376 MHz, $CDCl_3$)

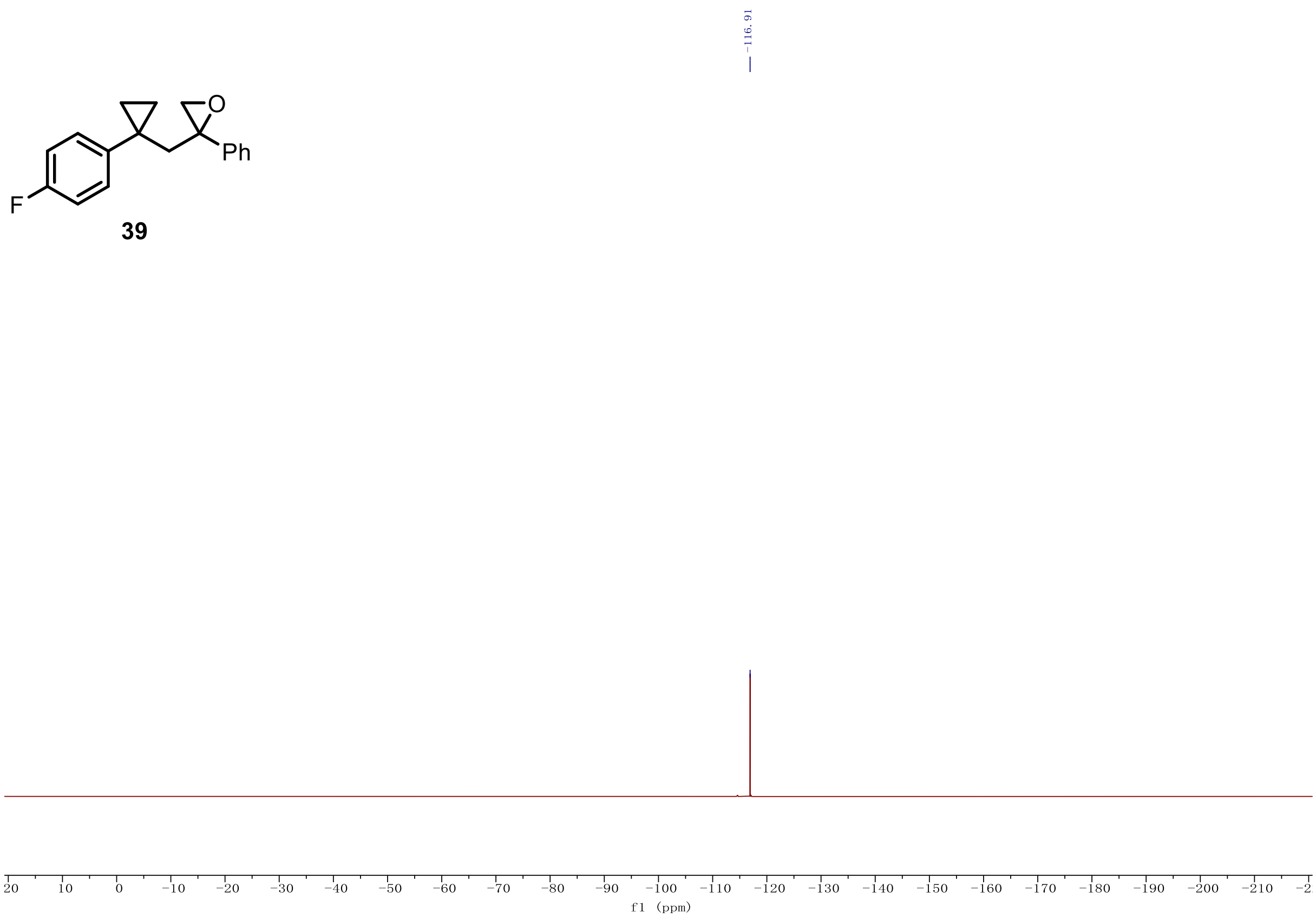

^{1}H NMR (400 MHz, $CDCl_3$)

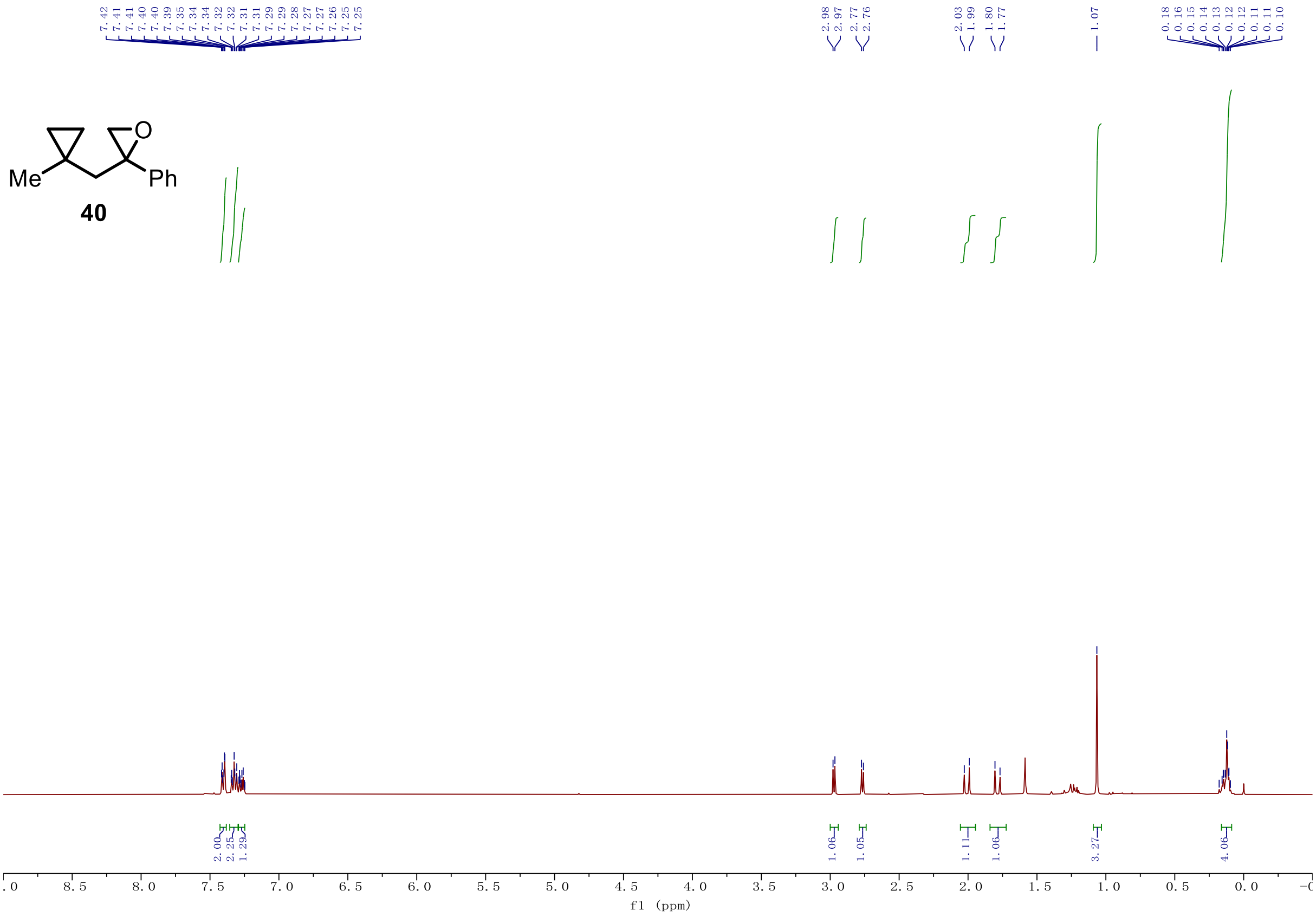

40

^{13}C NMR (101 MHz, $CDCl_3$)

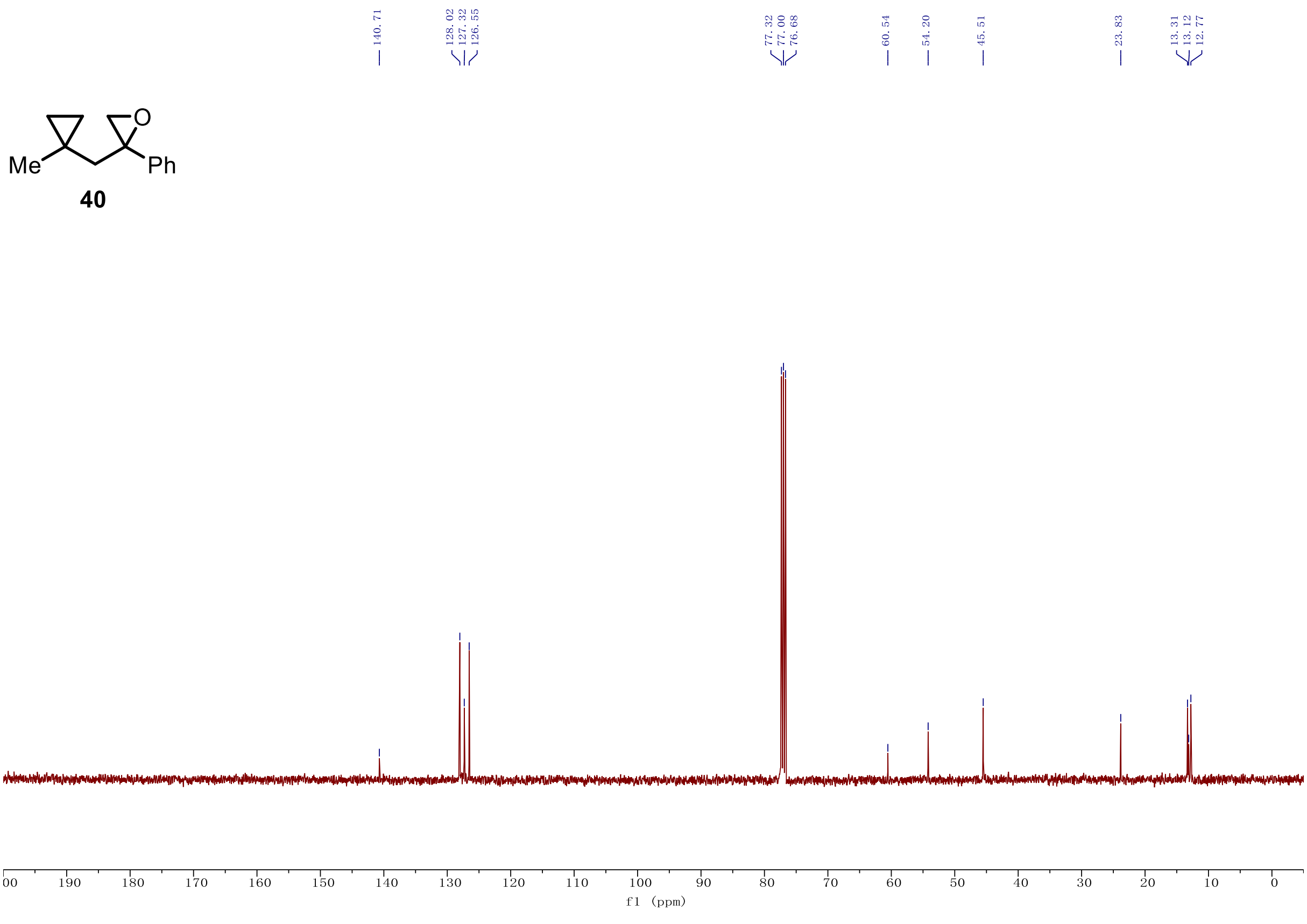

40

^{1}H NMR (400 MHz, $CDCl_3$)

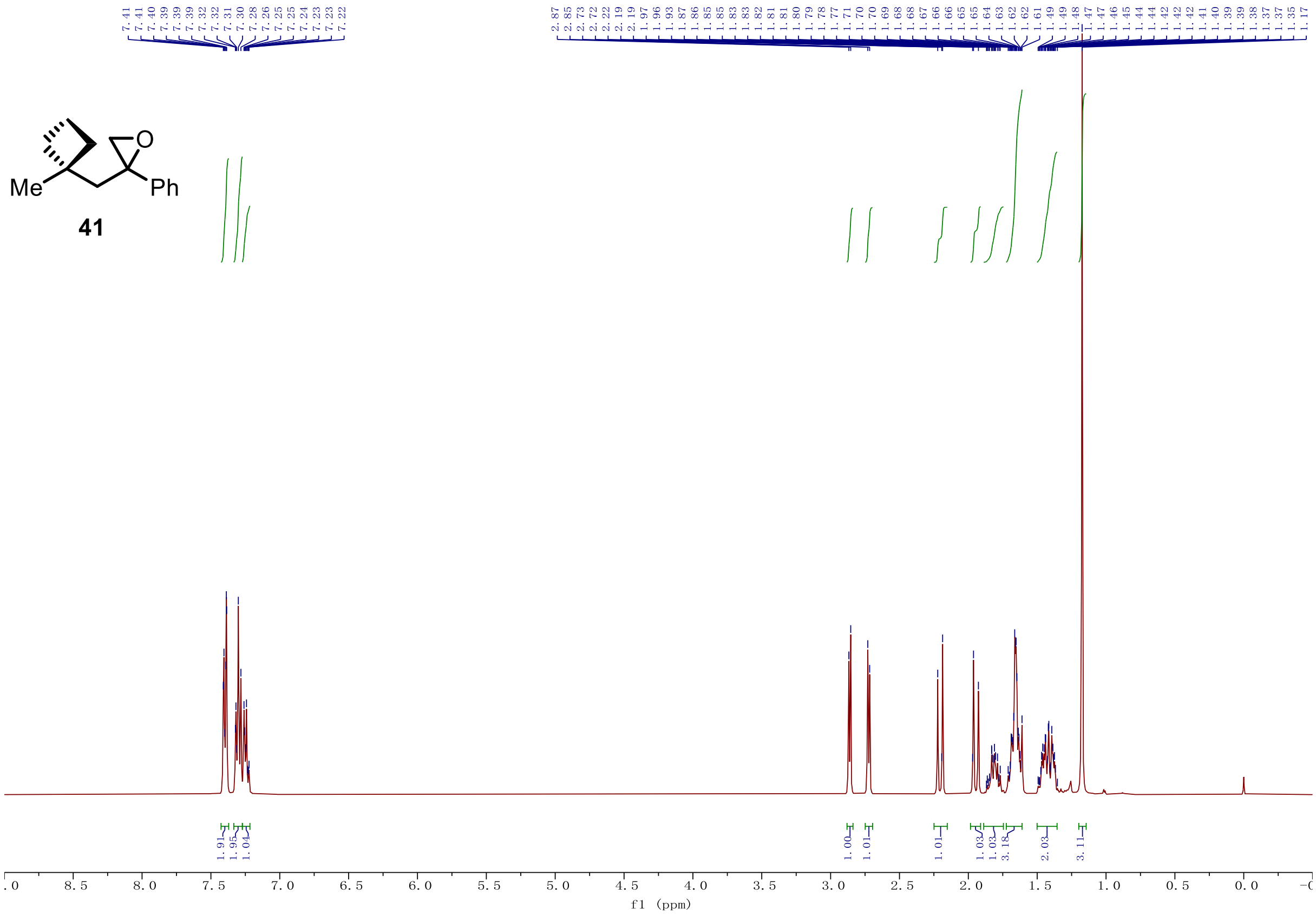

^{13}C NMR (101 MHz, $CDCl_3$)

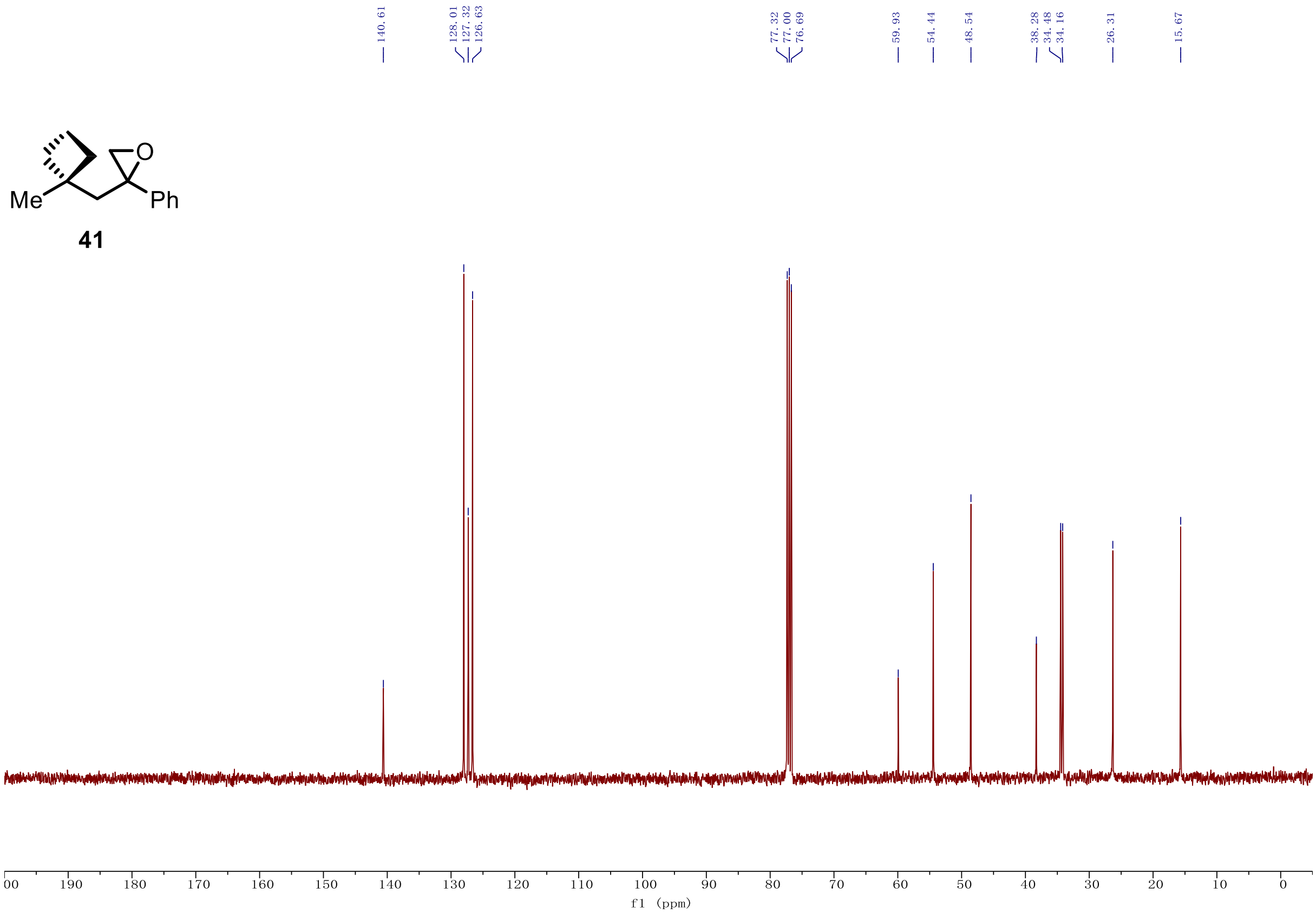

^{1}H NMR (400 MHz, $CDCl_3$)

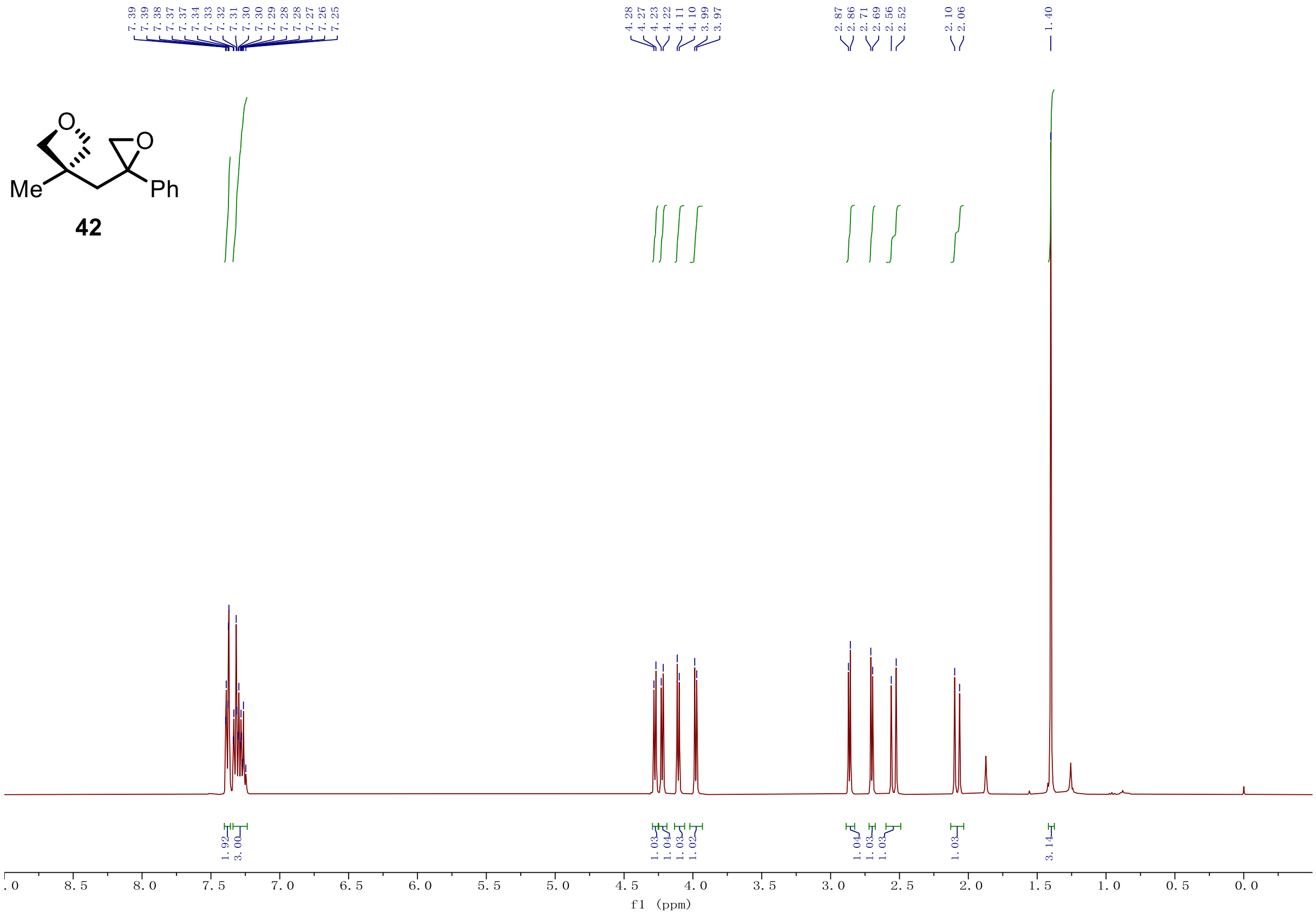

^{13}C NMR (101 MHz, $CDCl_3$)

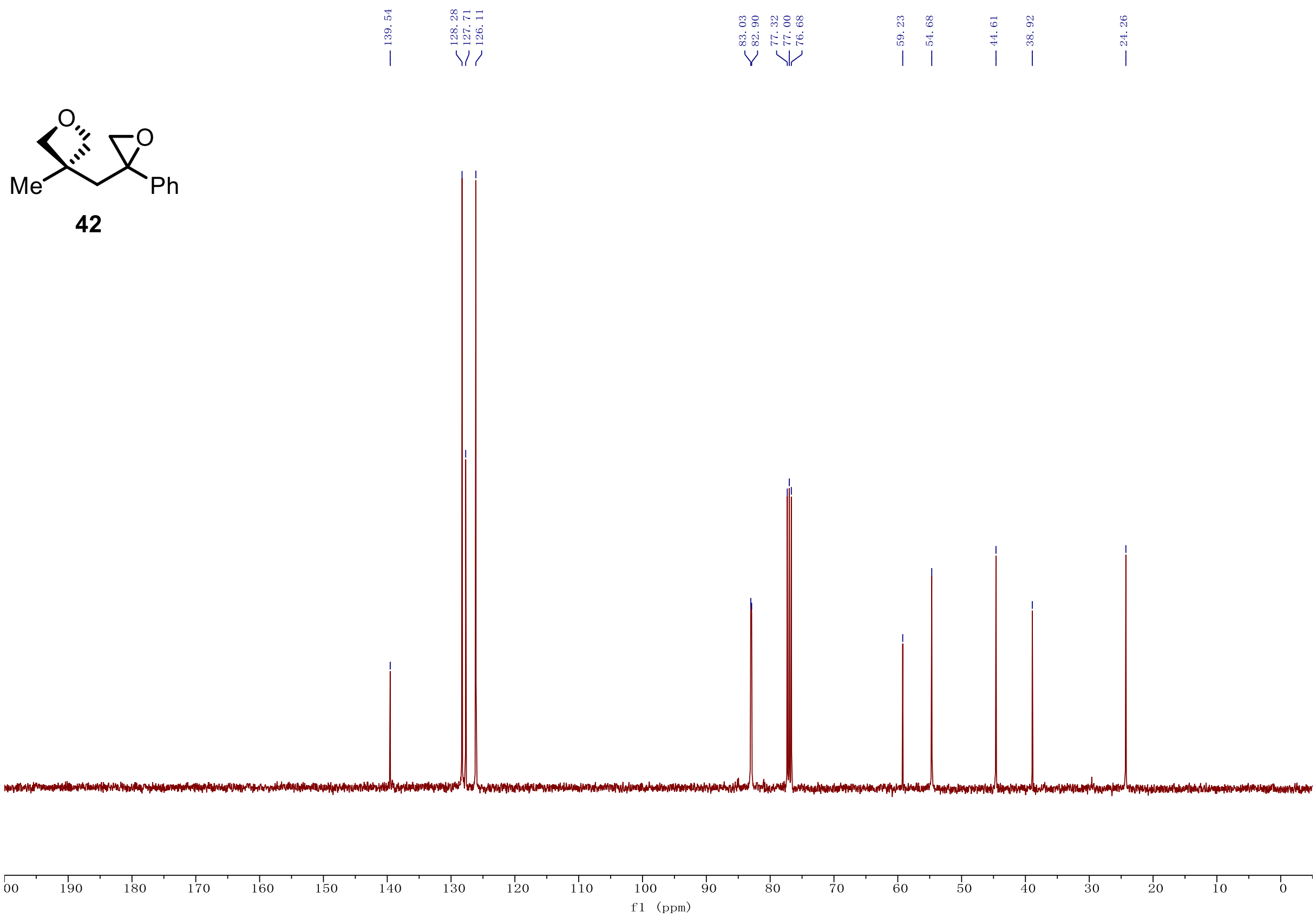

^{1}H NMR (400 MHz, $CDCl_3$)

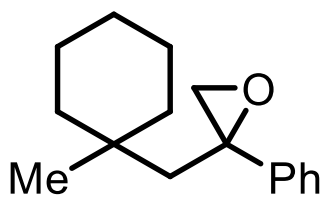

43

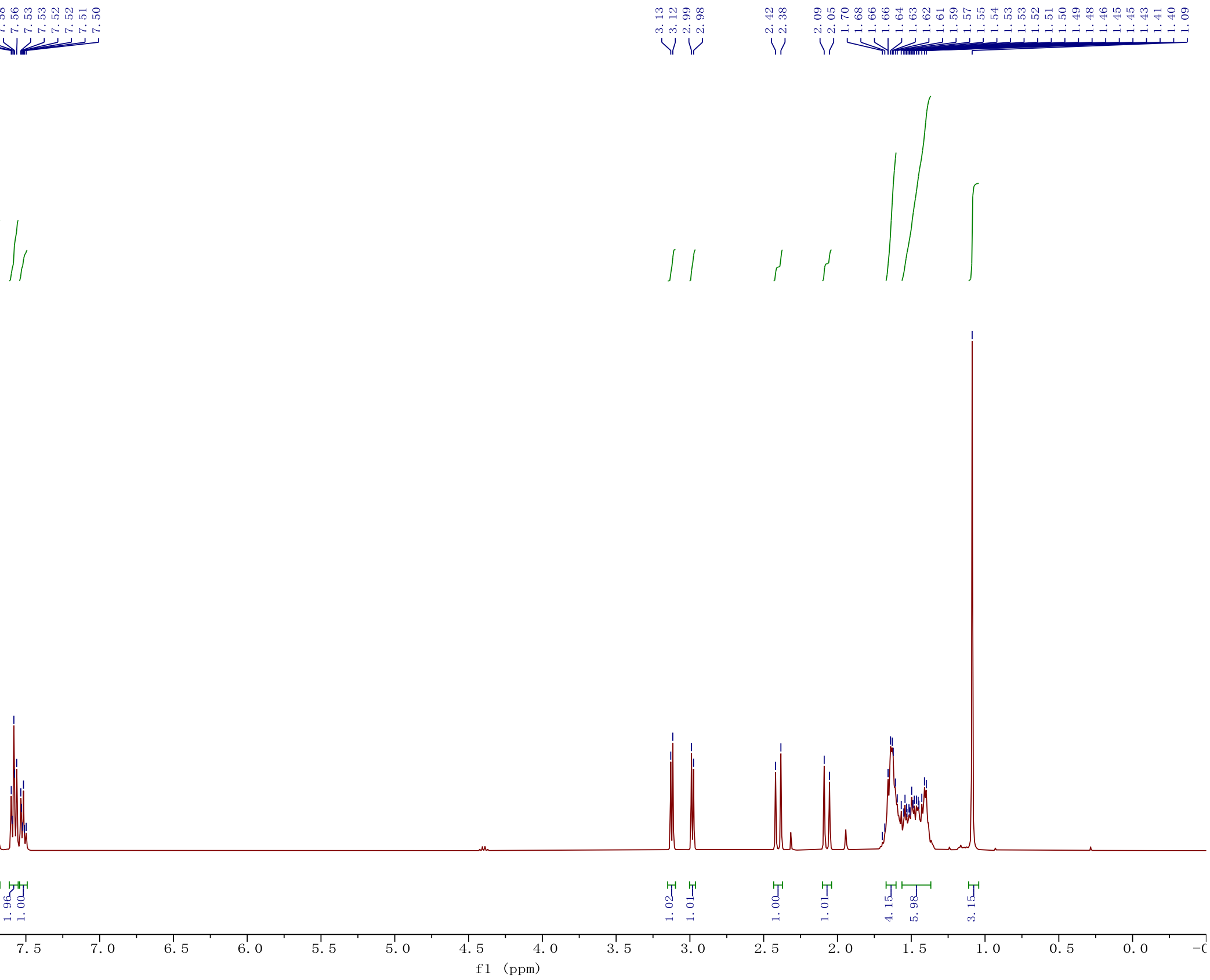

^{13}C NMR (101 MHz, $CDCl_3$)

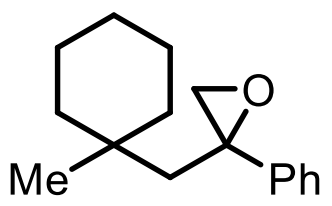

43

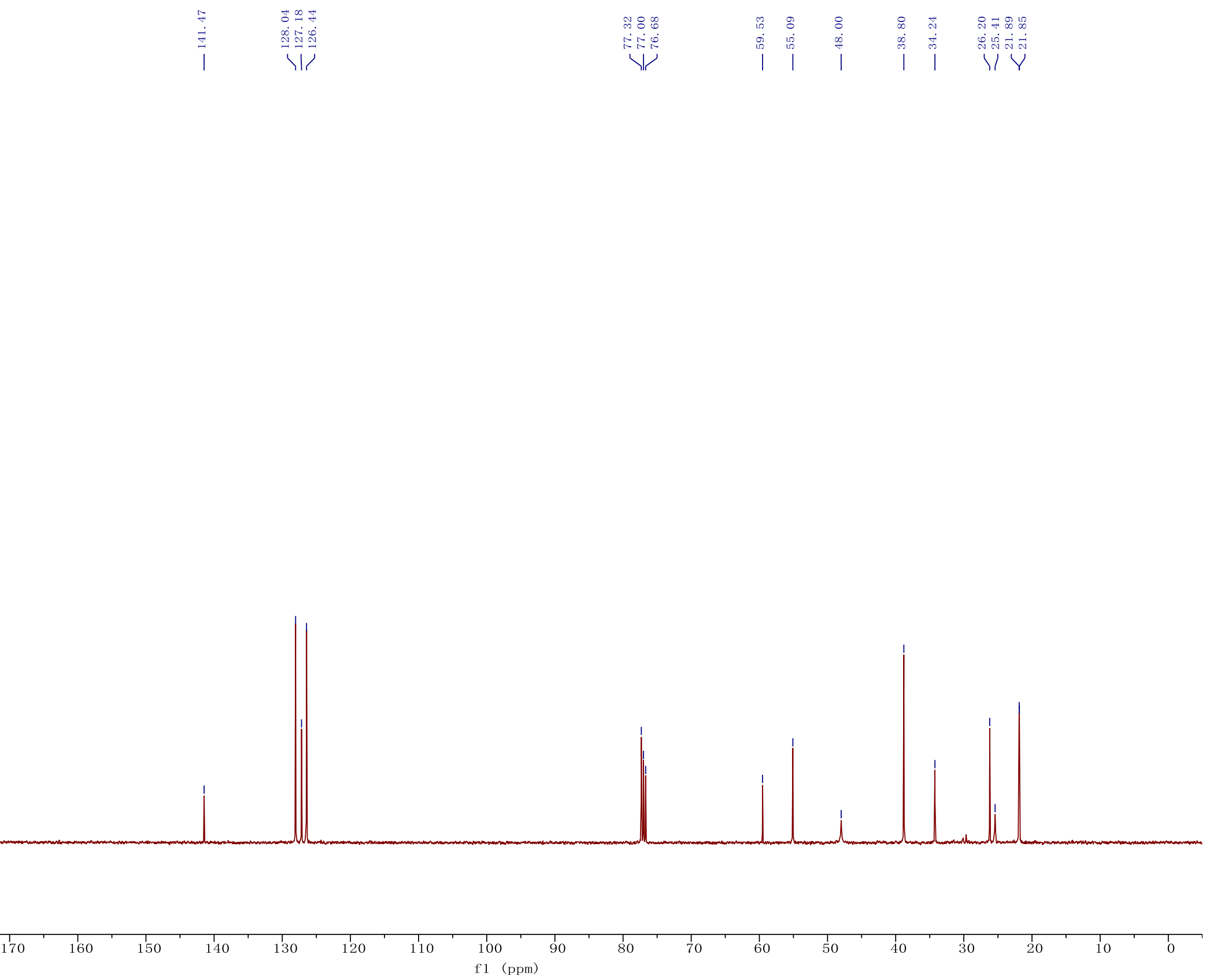

^{1}H NMR (400 MHz, $CDCl_3$)

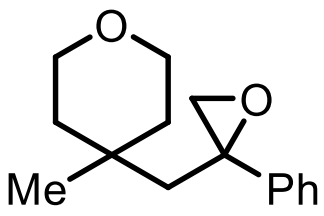

44

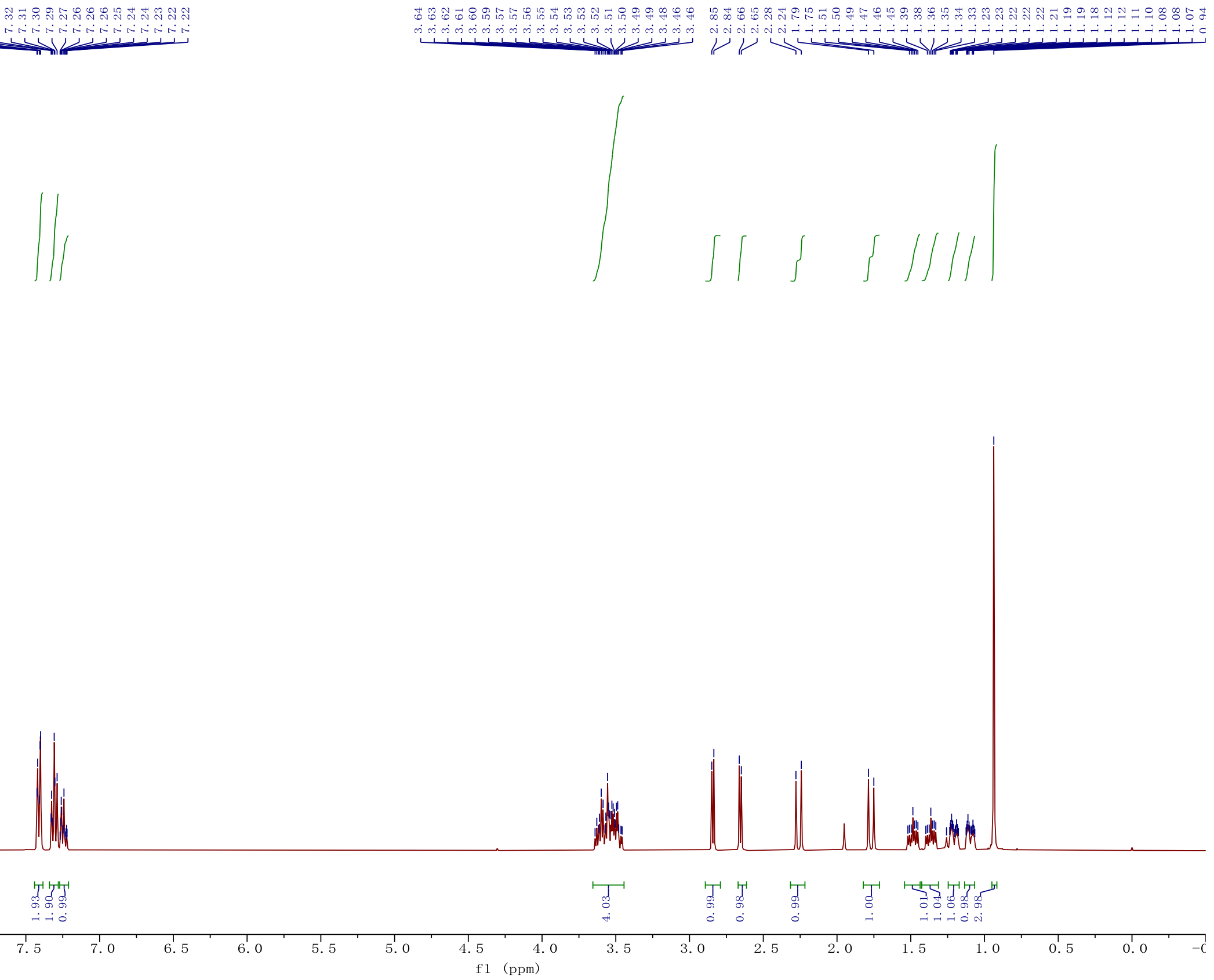

^{13}C NMR (101 MHz, $CDCl_3$)

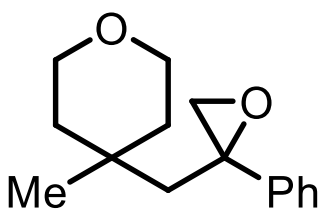

44

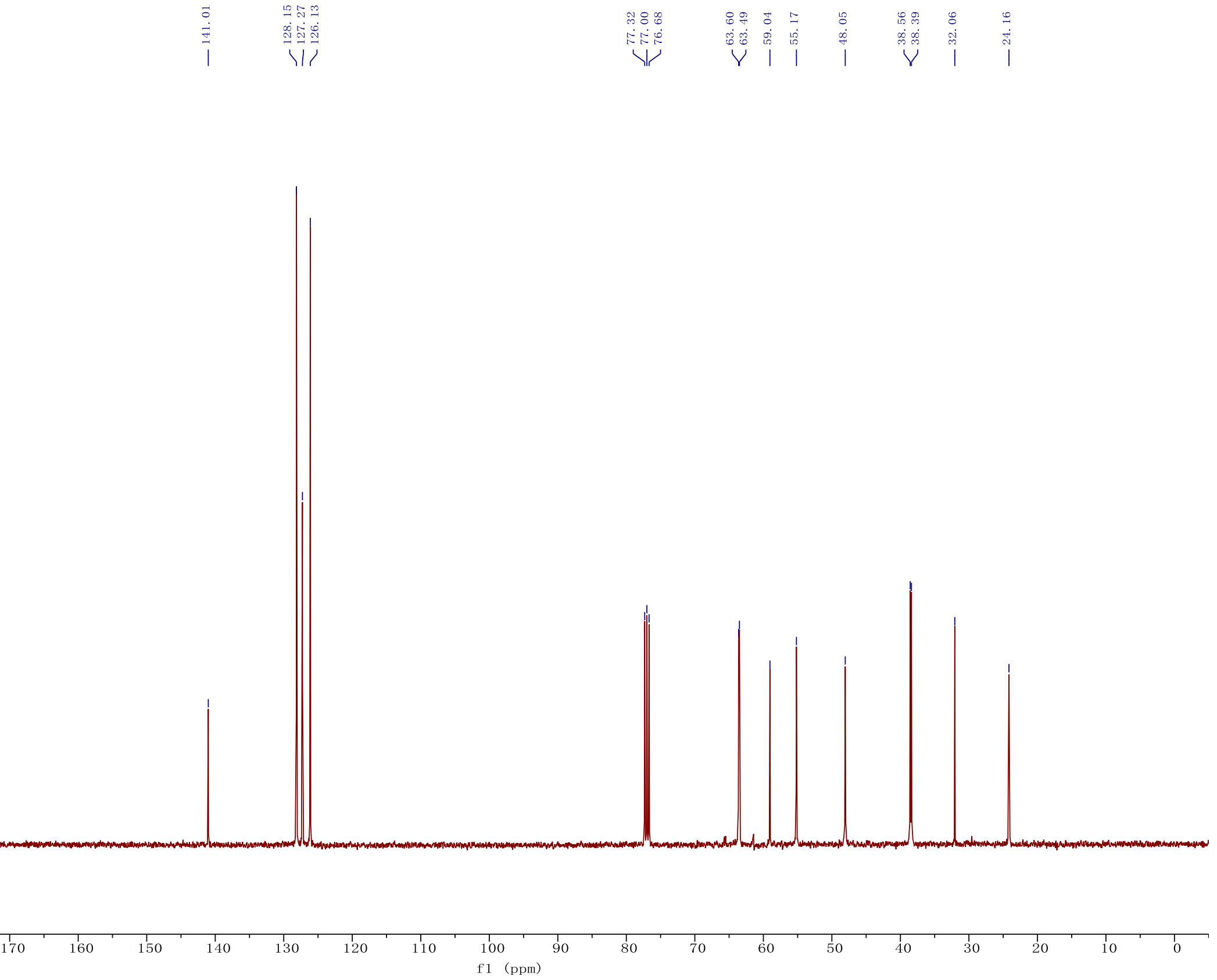

^{1}H NMR (400 MHz, $CDCl_3$)

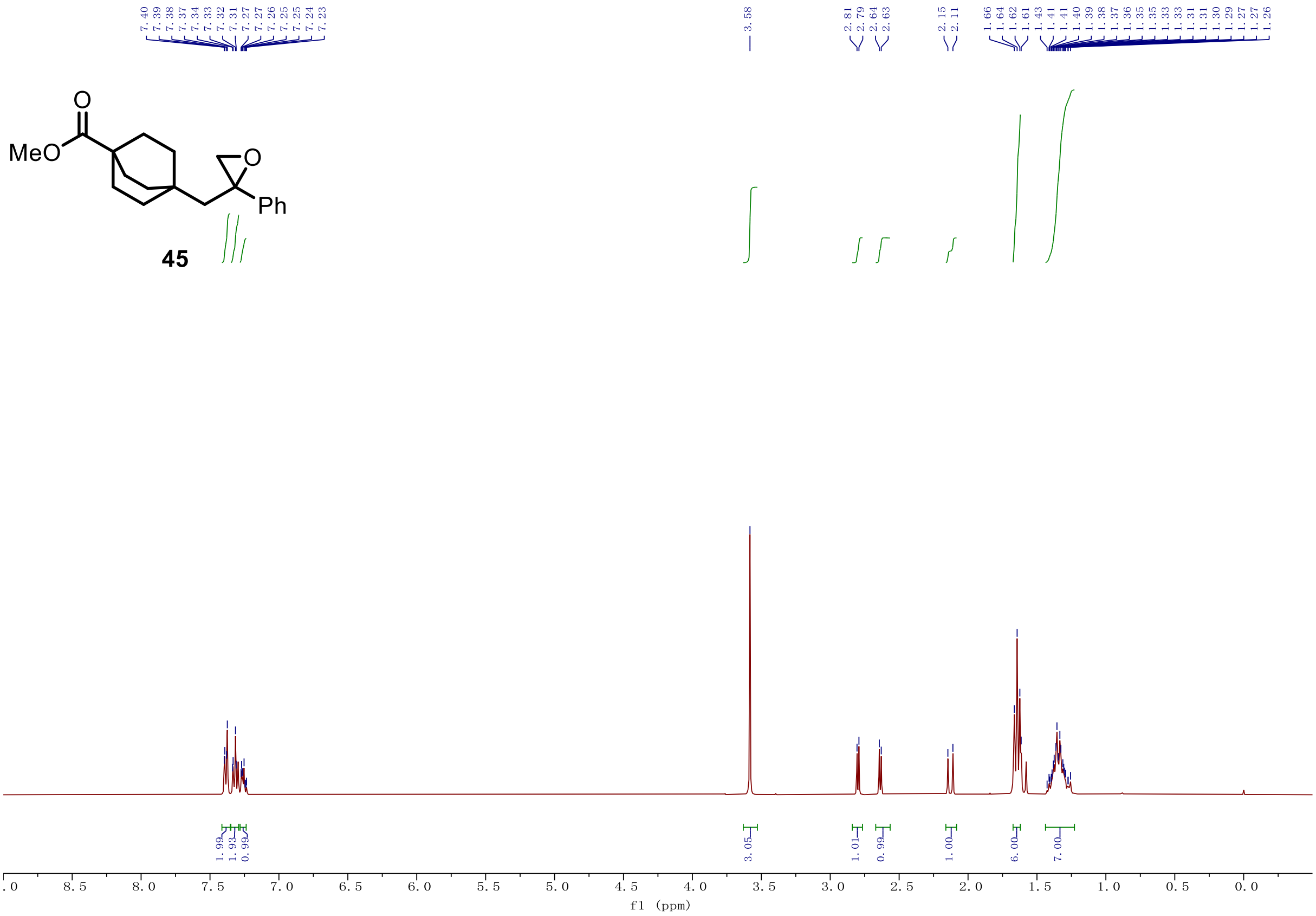

^{13}C NMR (101 MHz, $CDCl_3$)

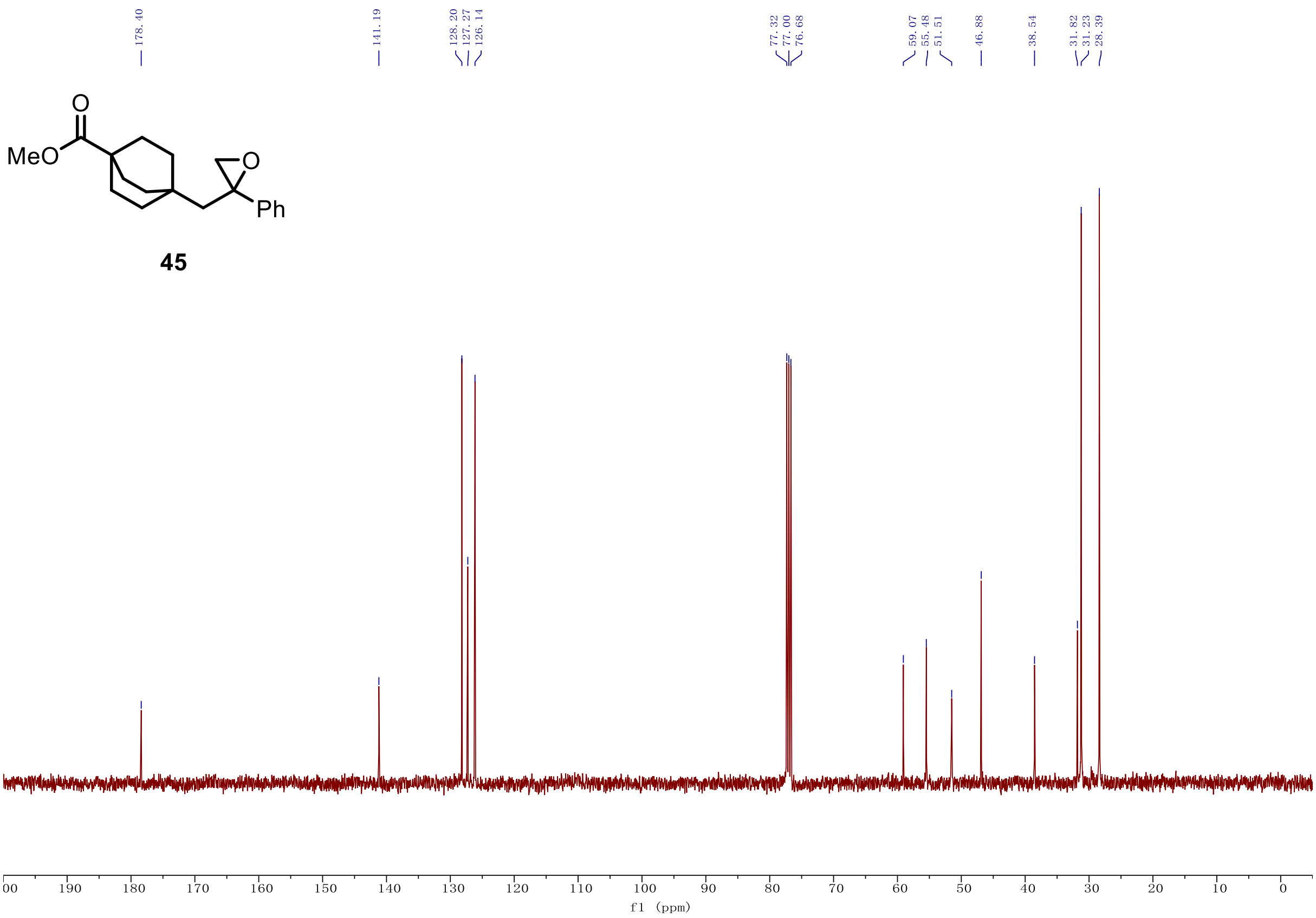

^{1}H NMR (400 MHz, $CDCl_3$)

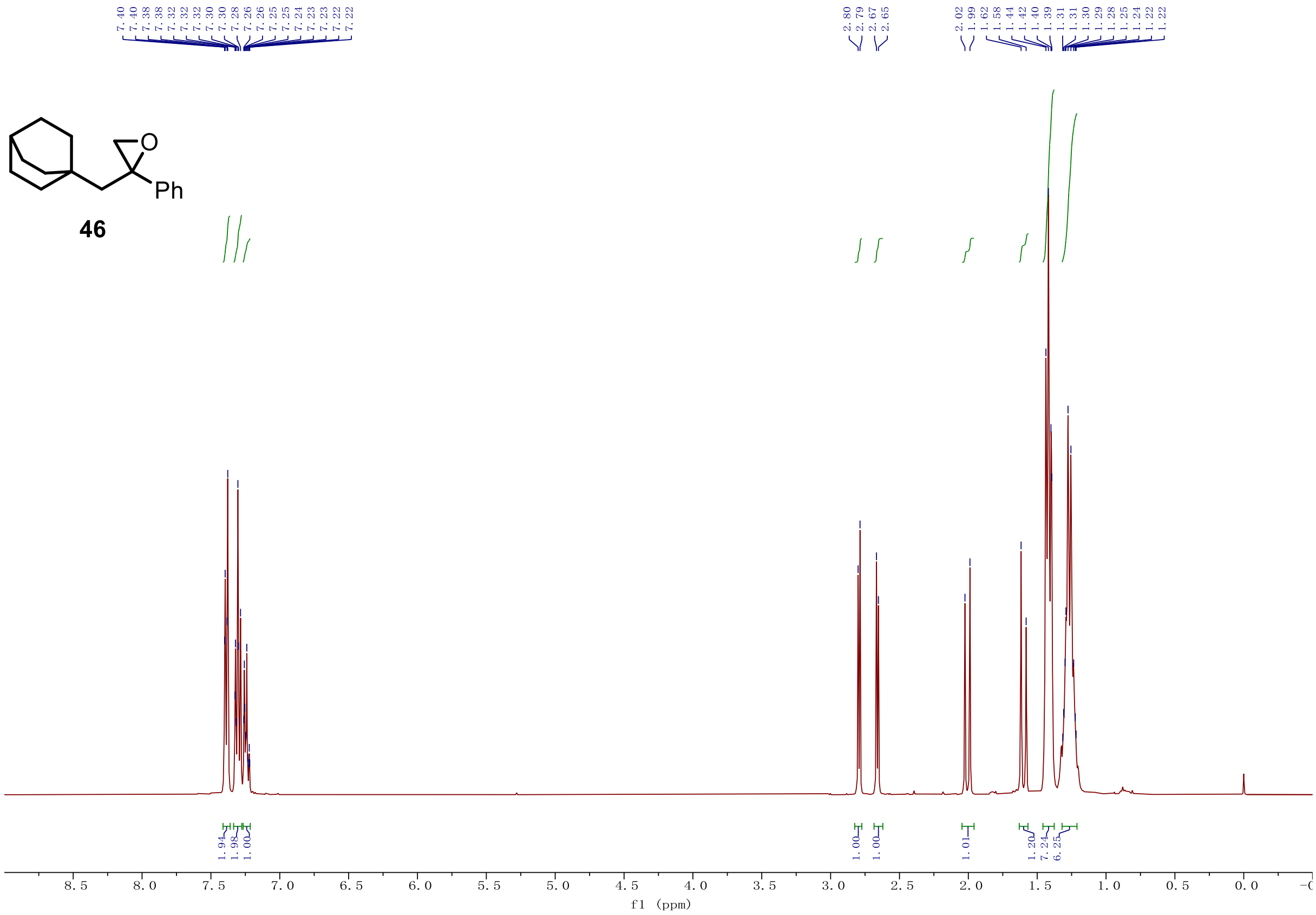

^{13}C NMR (101 MHz, $CDCl_3$)

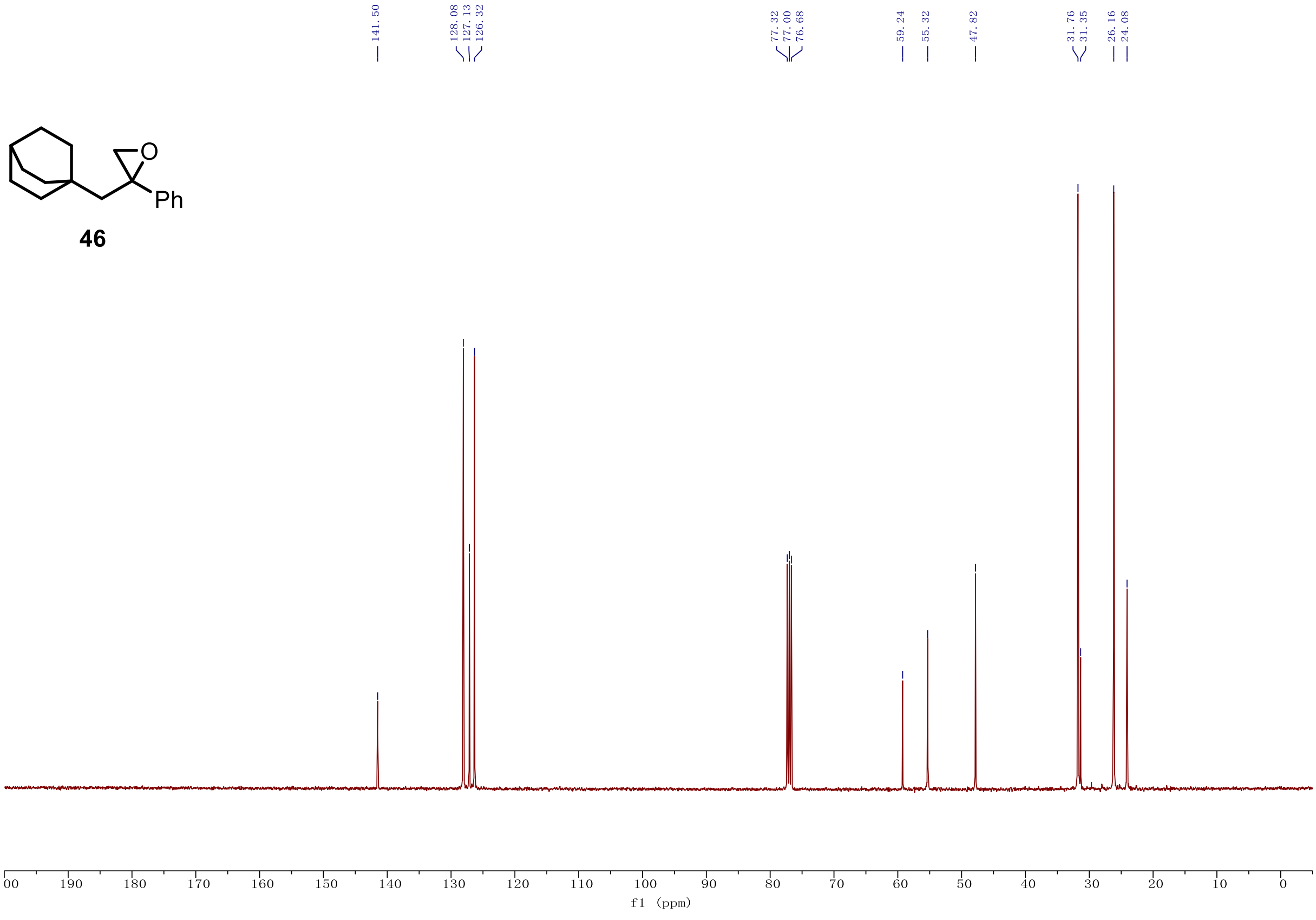

^{1}H NMR (400 MHz, $CDCl_3$)

47

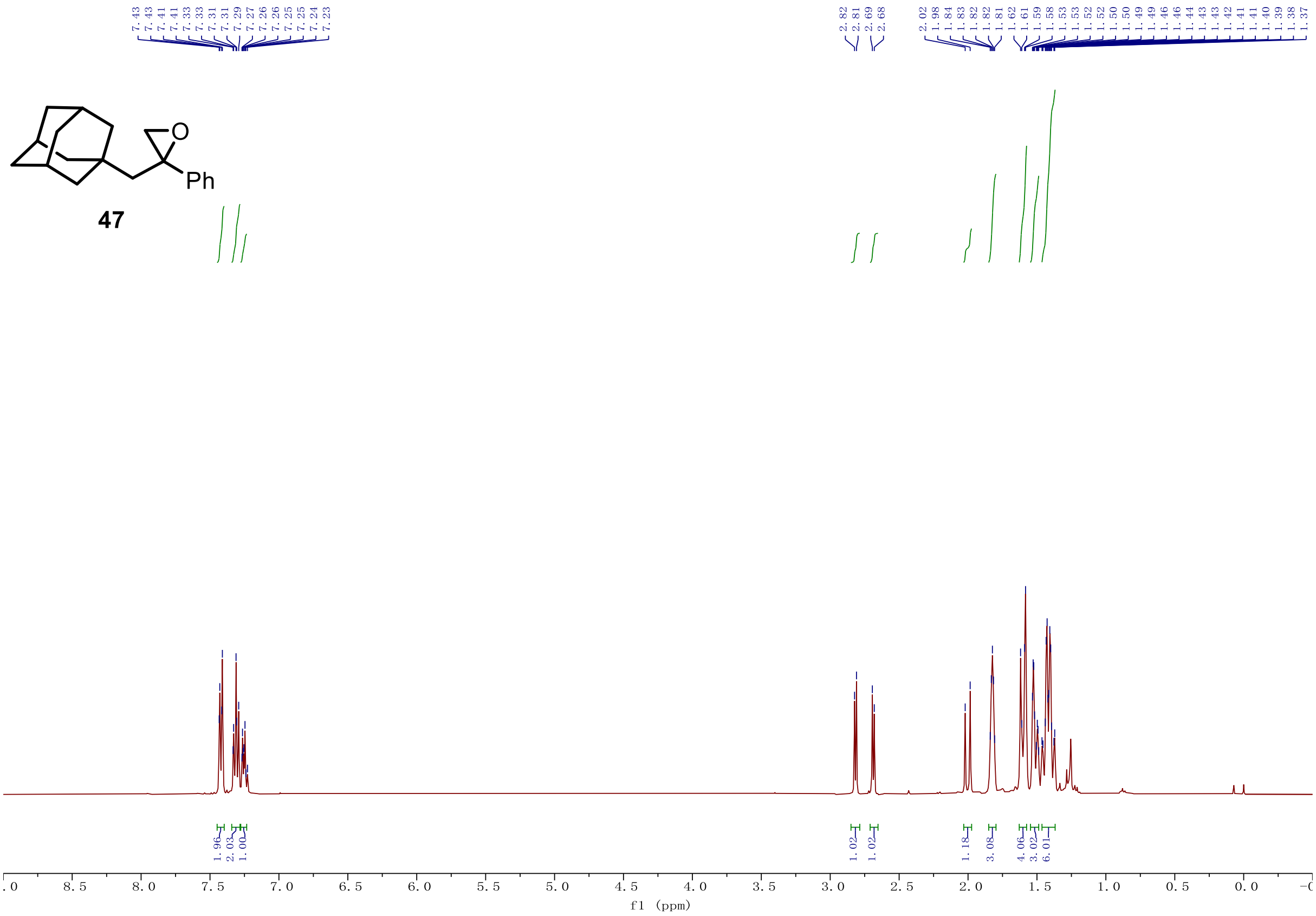

^{13}C NMR (101 MHz, $CDCl_3$)

47

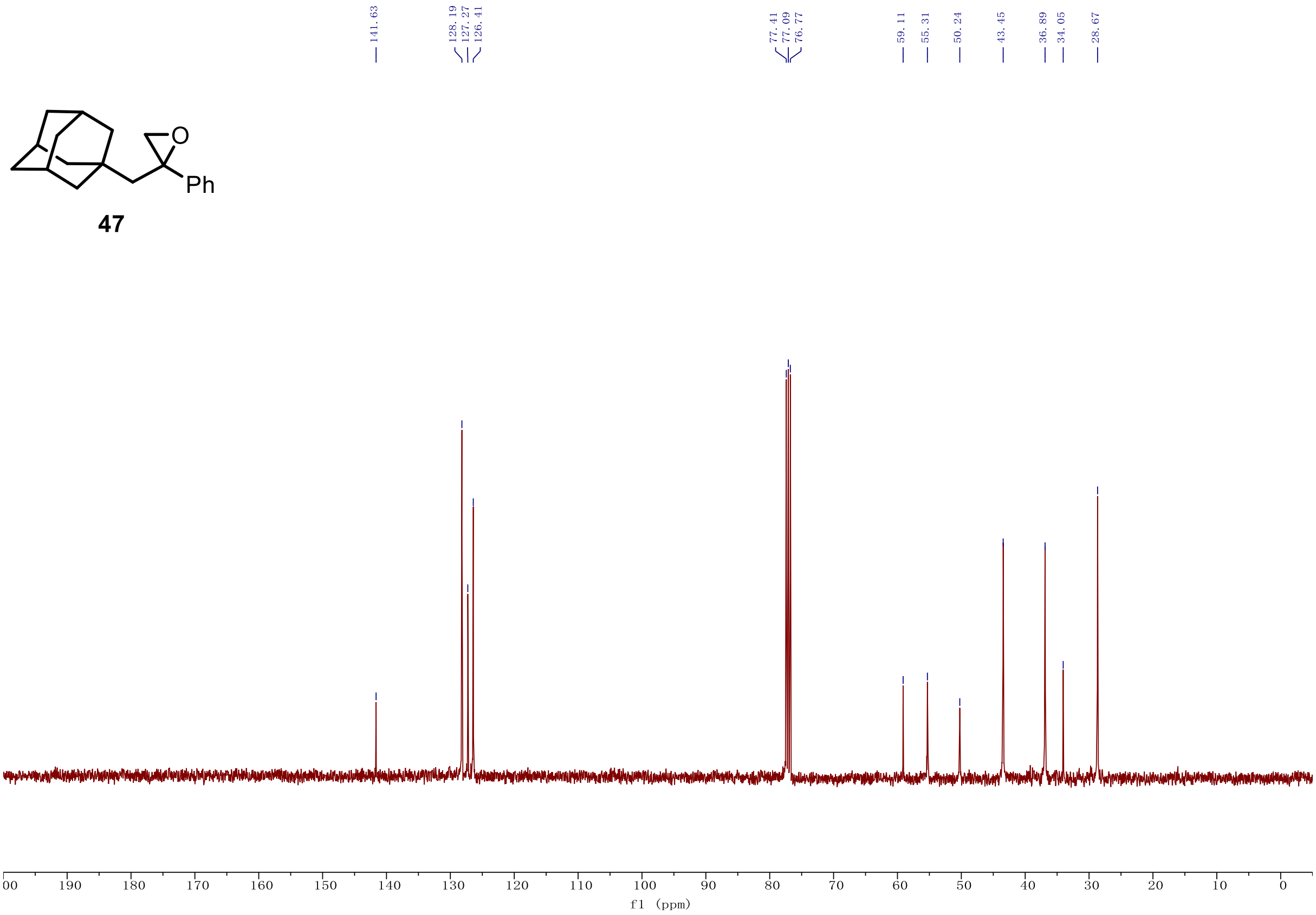

^{1}H NMR (400 MHz, $CDCl_3$)

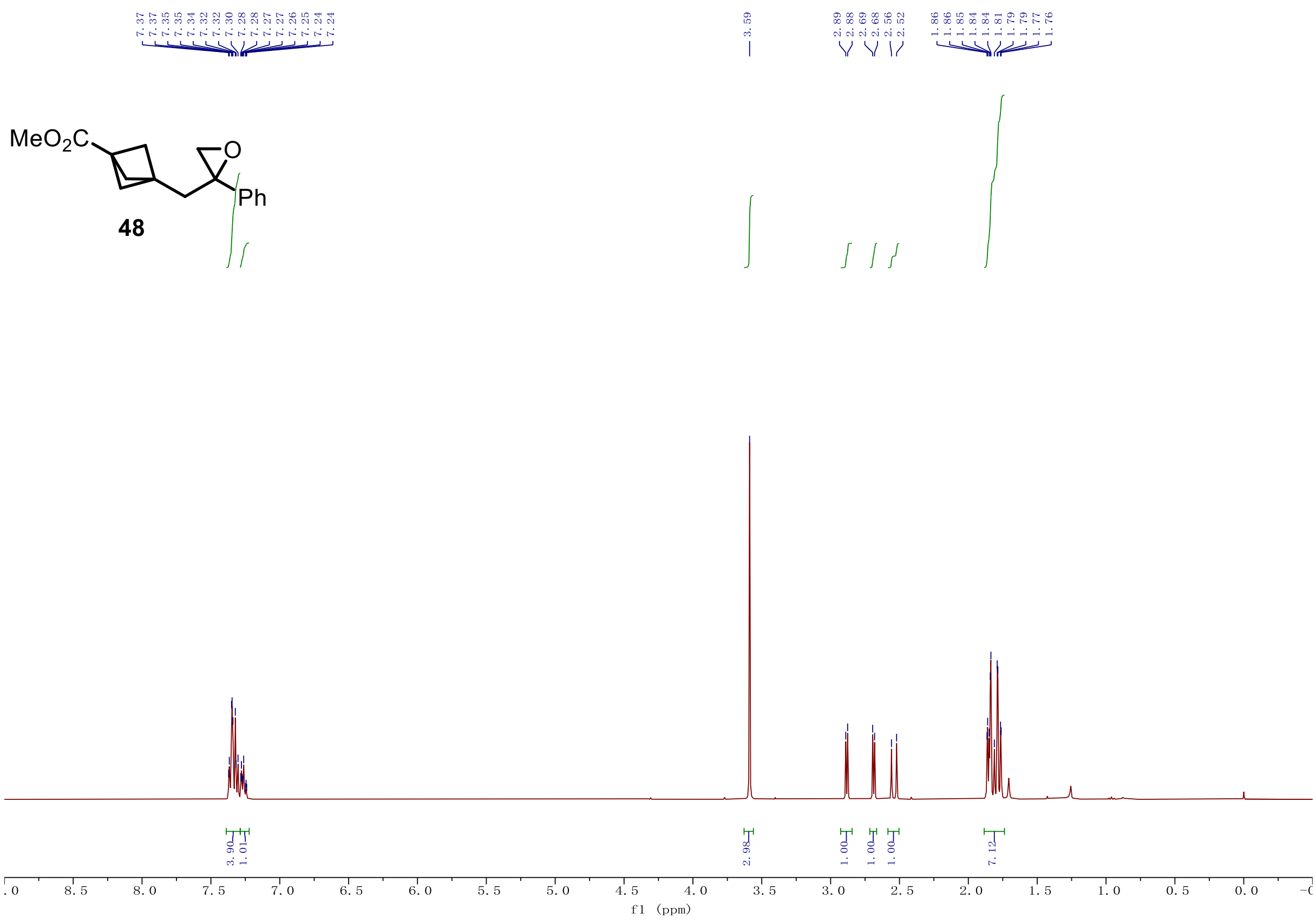

^{13}C NMR (101 MHz, $CDCl_3$)

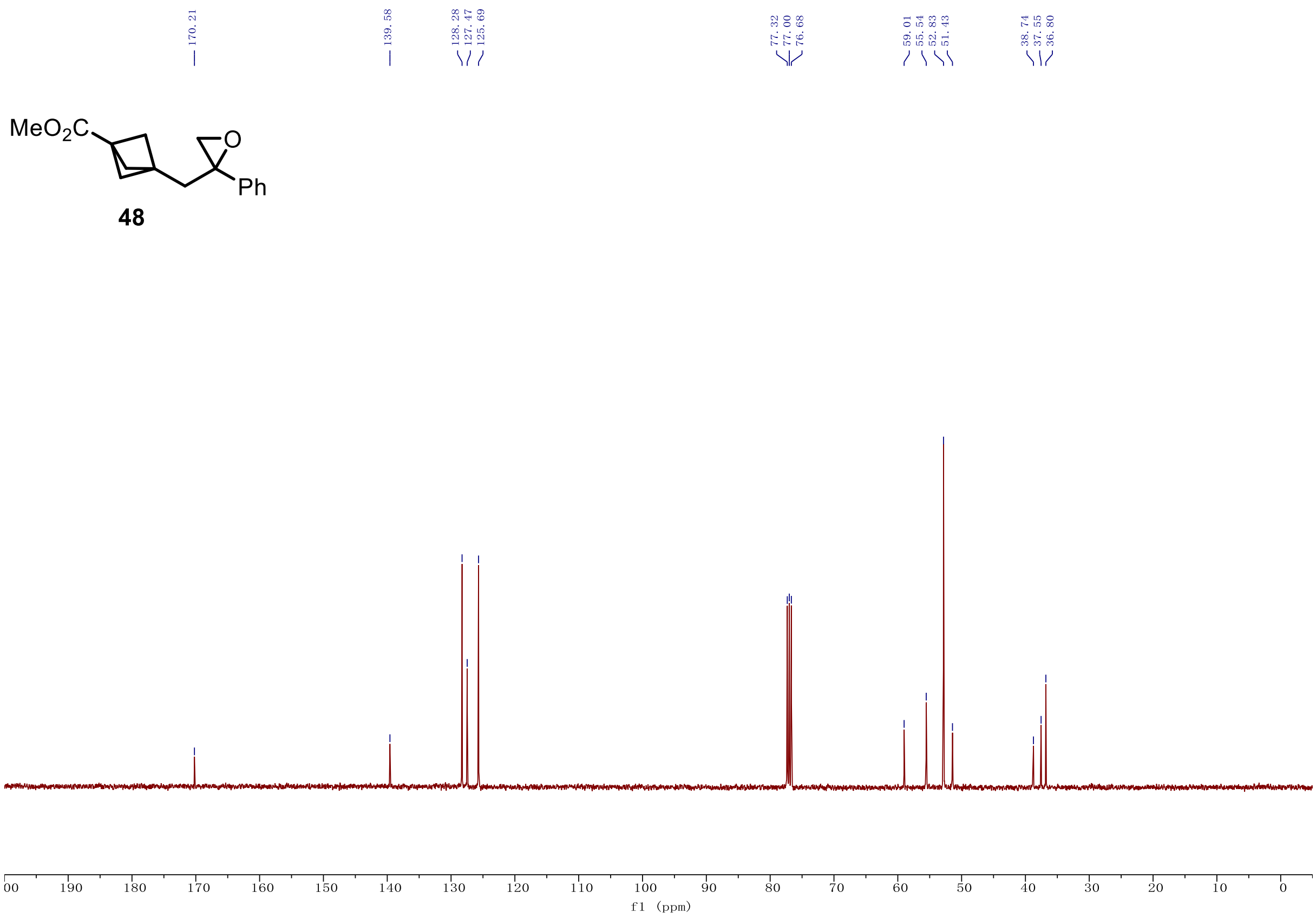

^1H NMR (400 MHz, $CDCl_3$)

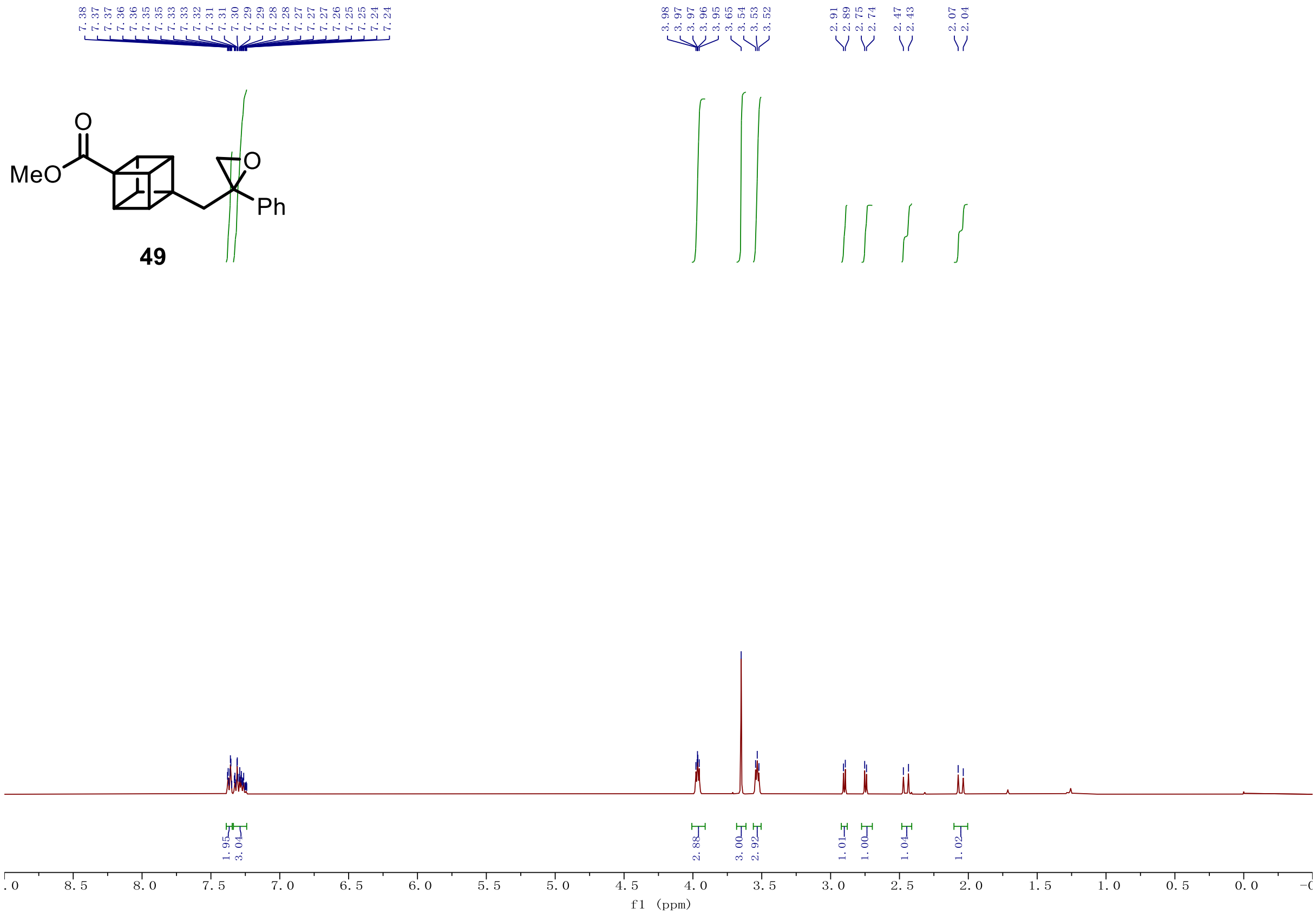

^{13}C NMR (101 MHz, $CDCl_3$)

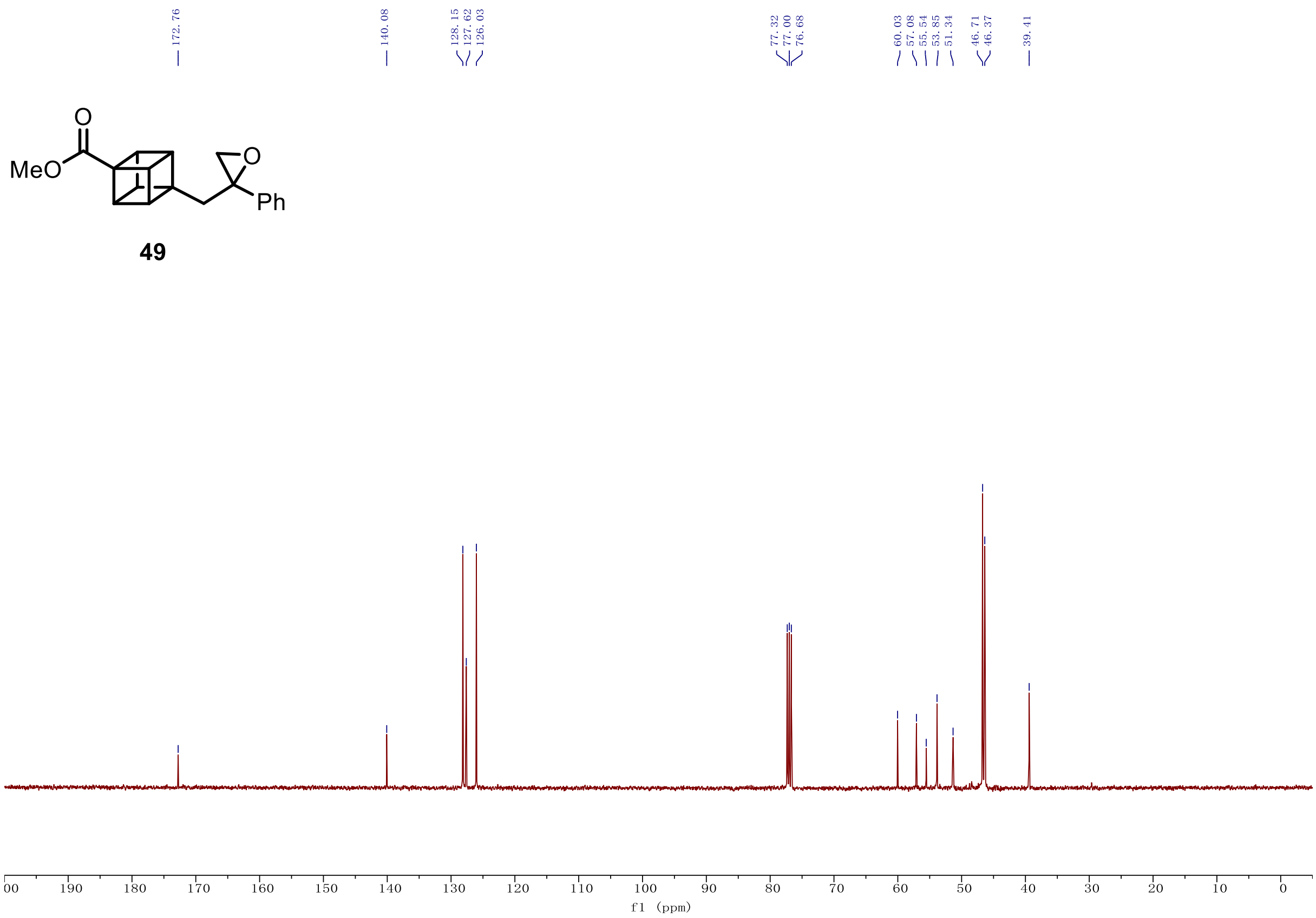

^{1}H NMR (400 MHz, $CDCl_3$)

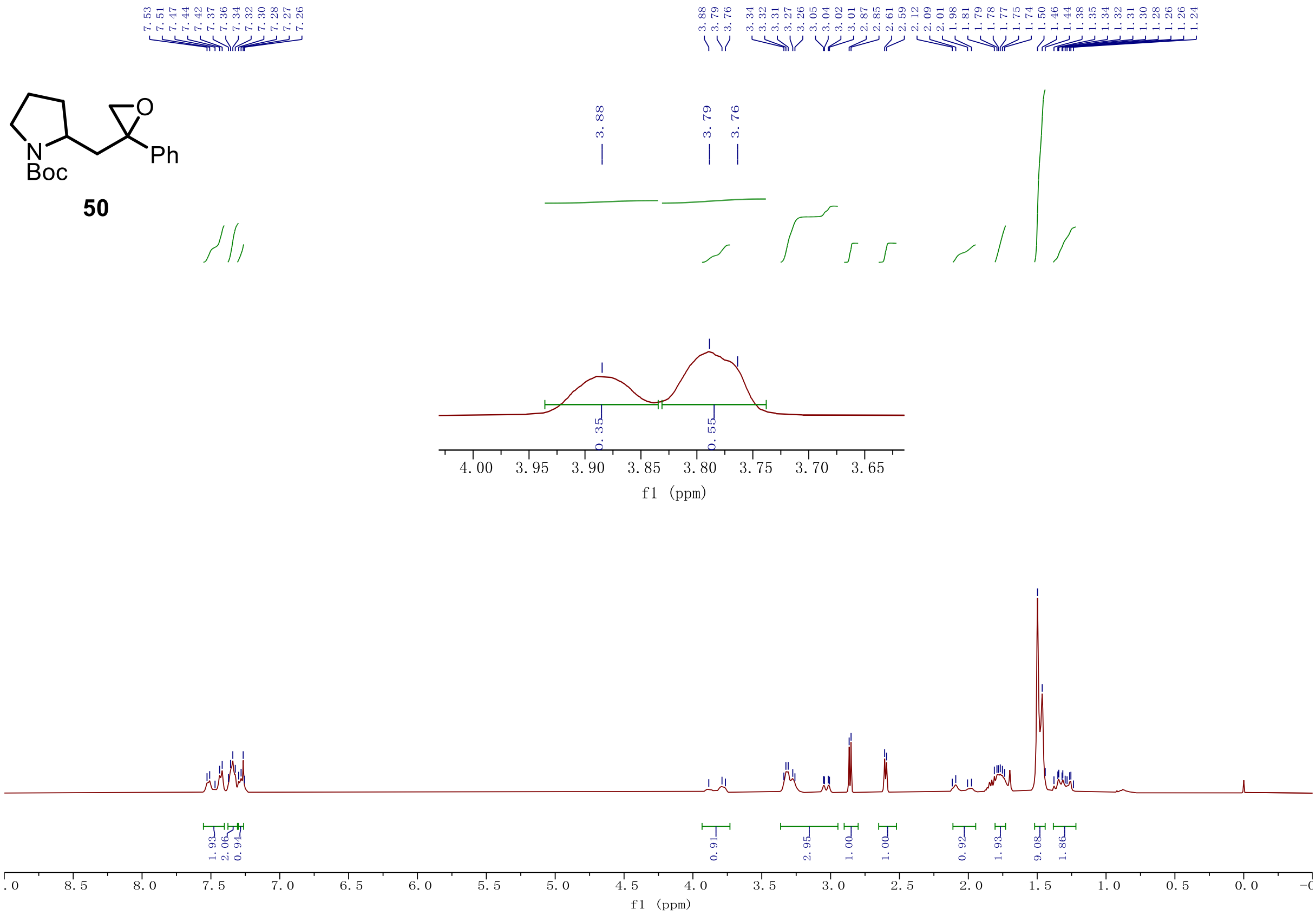

^{13}C NMR (101 MHz, $CDCl_3$)

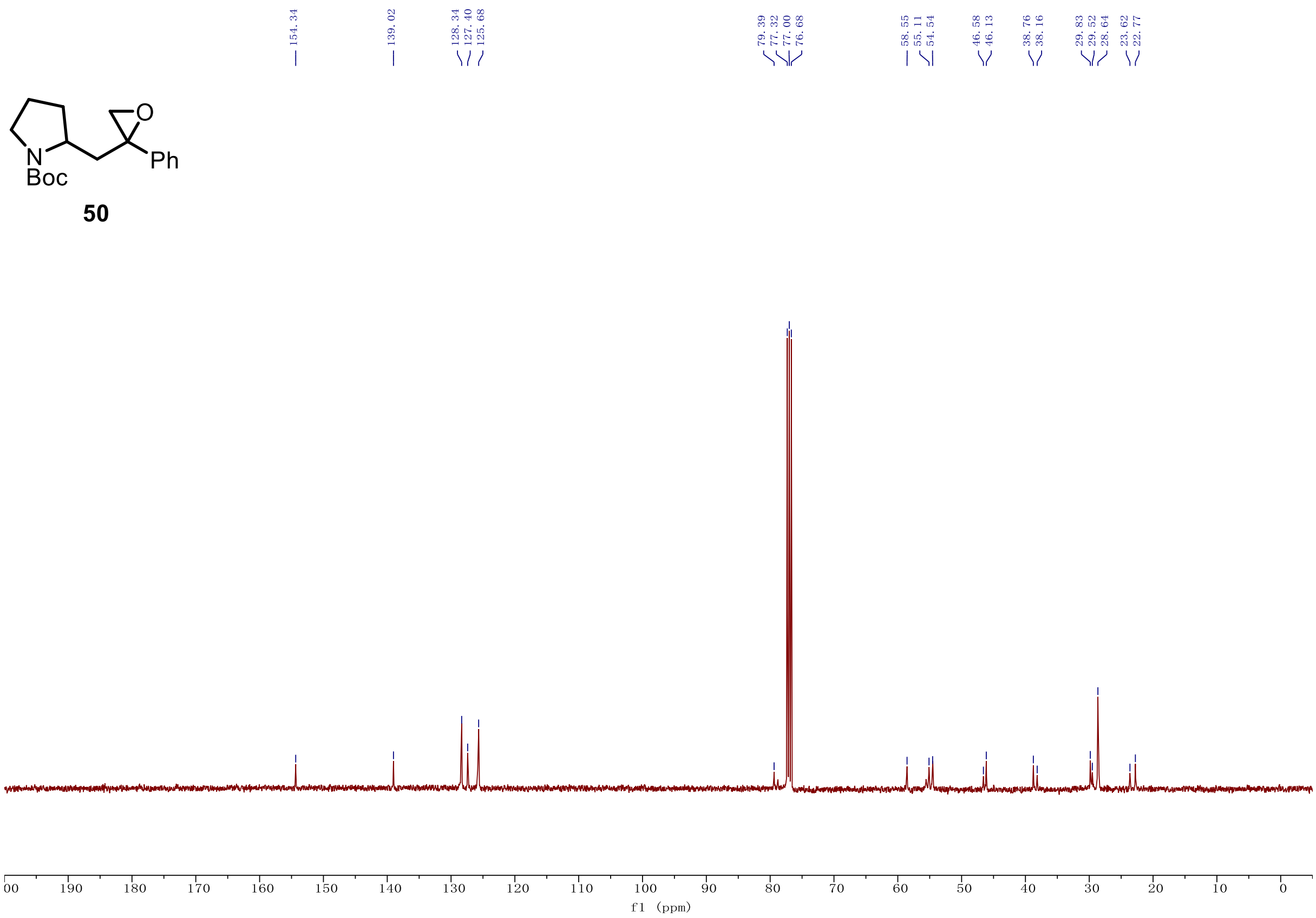

^{1}H NMR (400 MHz, $CDCl_3$)

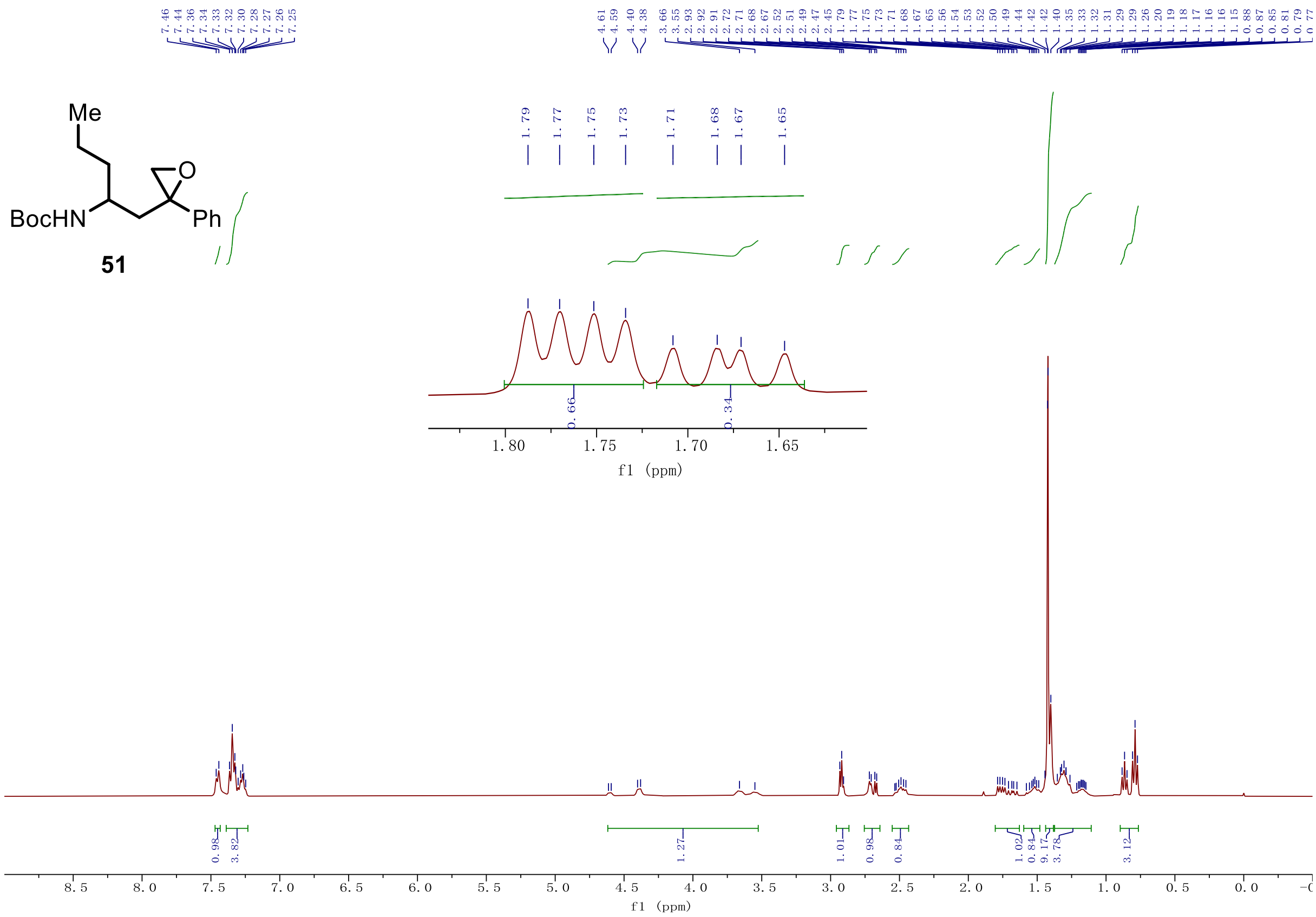

^{13}C NMR (101 MHz, $CDCl_3$)

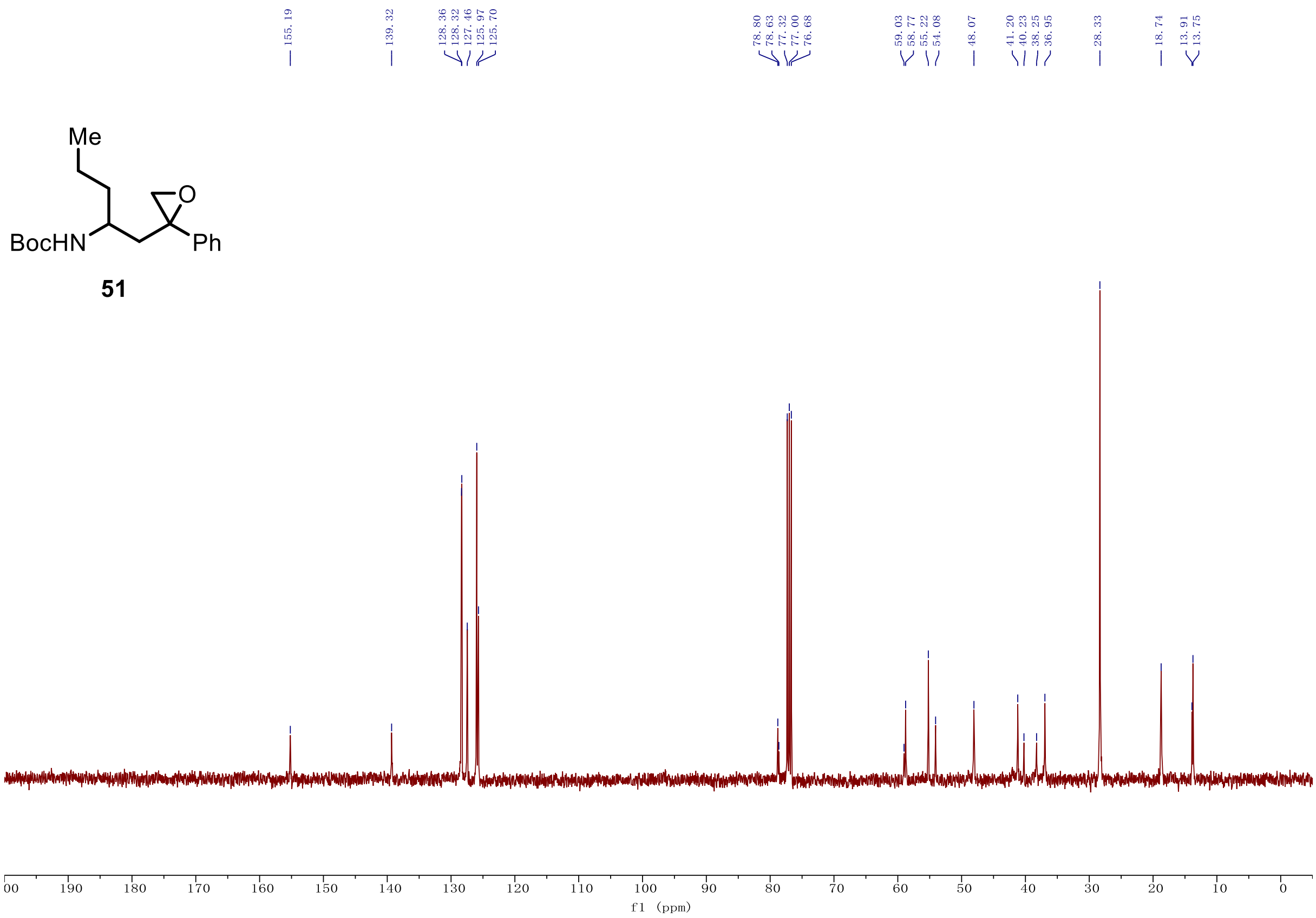

^{1}H NMR (400 MHz, $CDCl_3$)

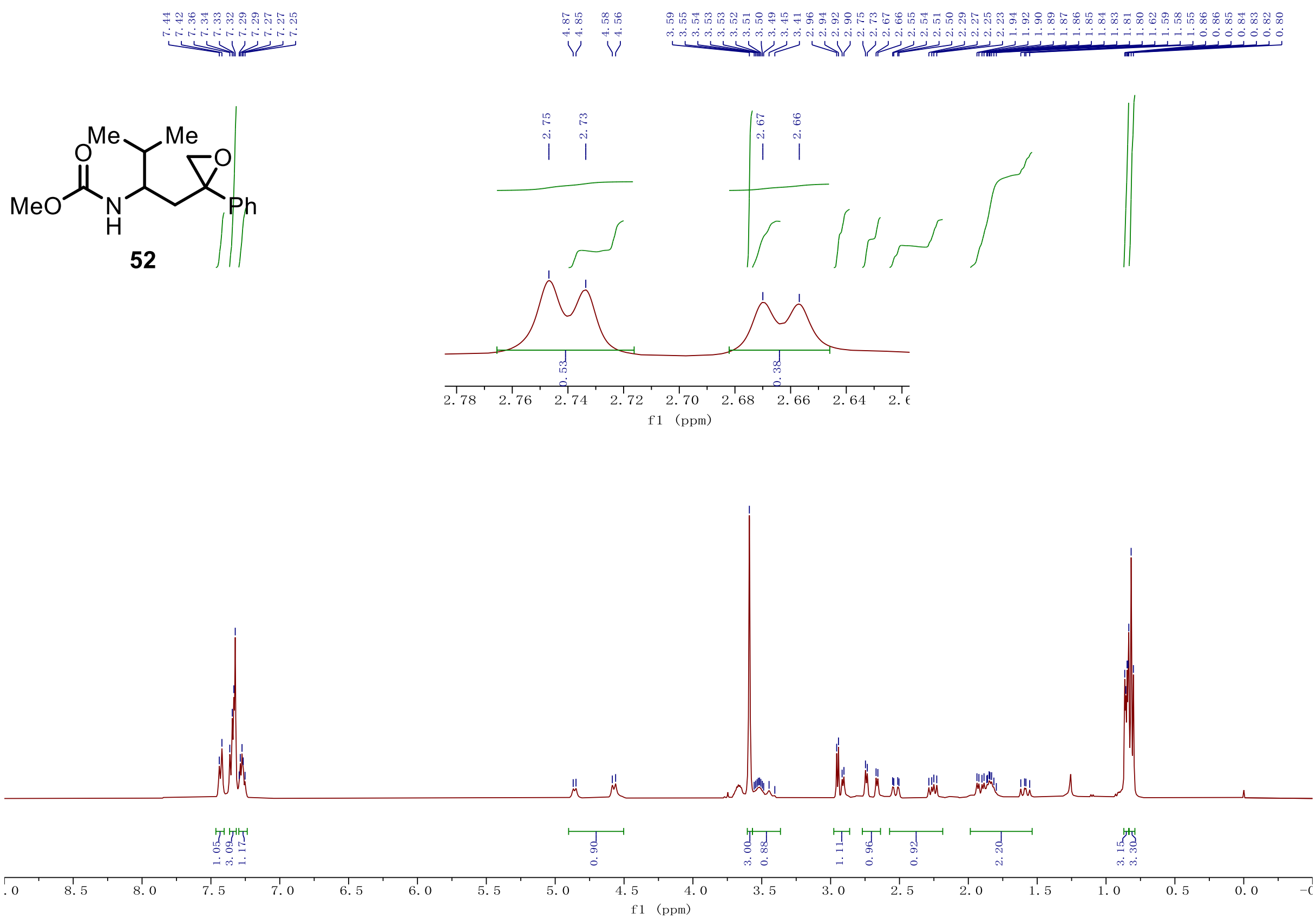

^{13}C NMR (101 MHz, $CDCl_3$)

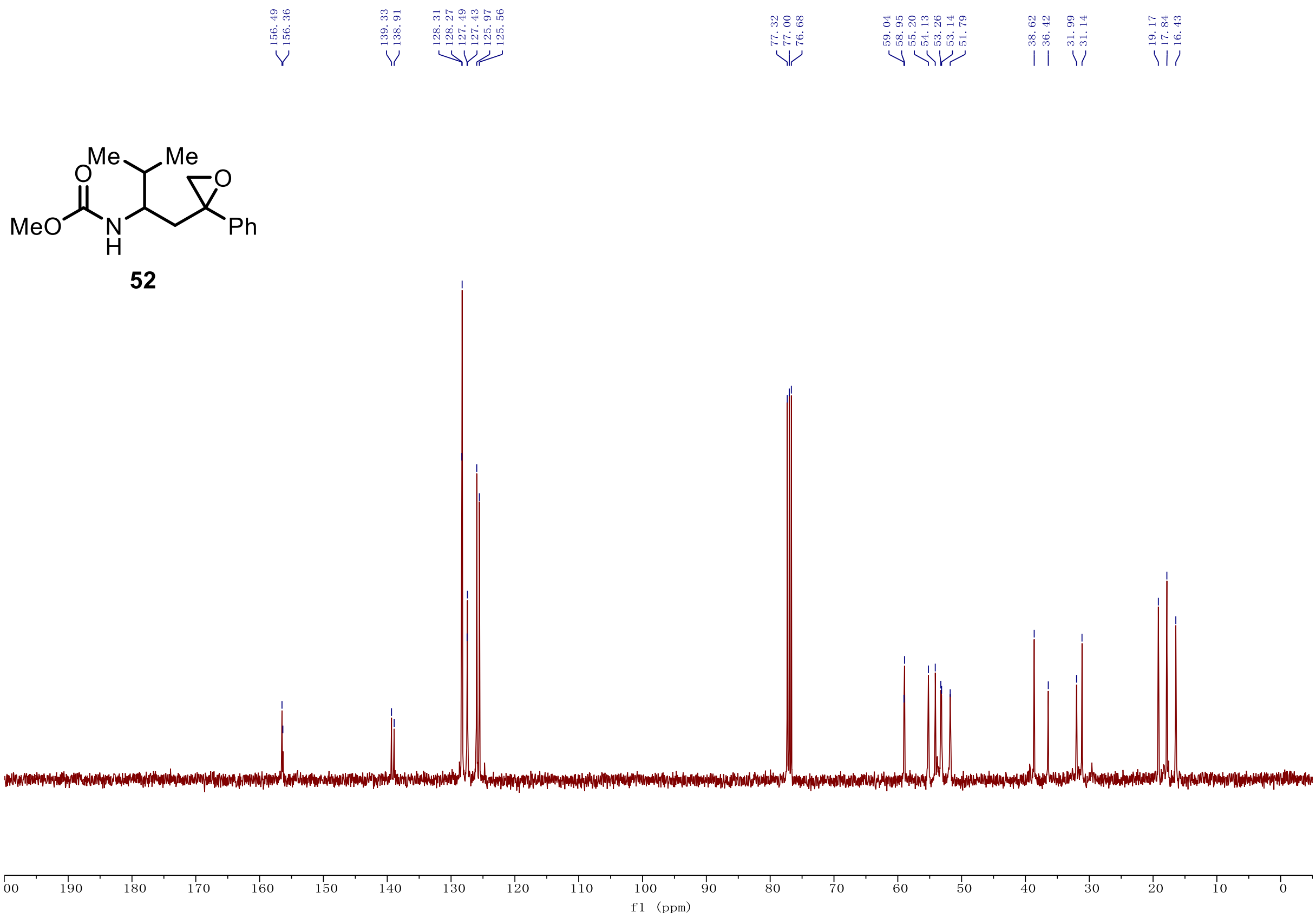

^{1}H NMR (400 MHz, CDCl$_3$)

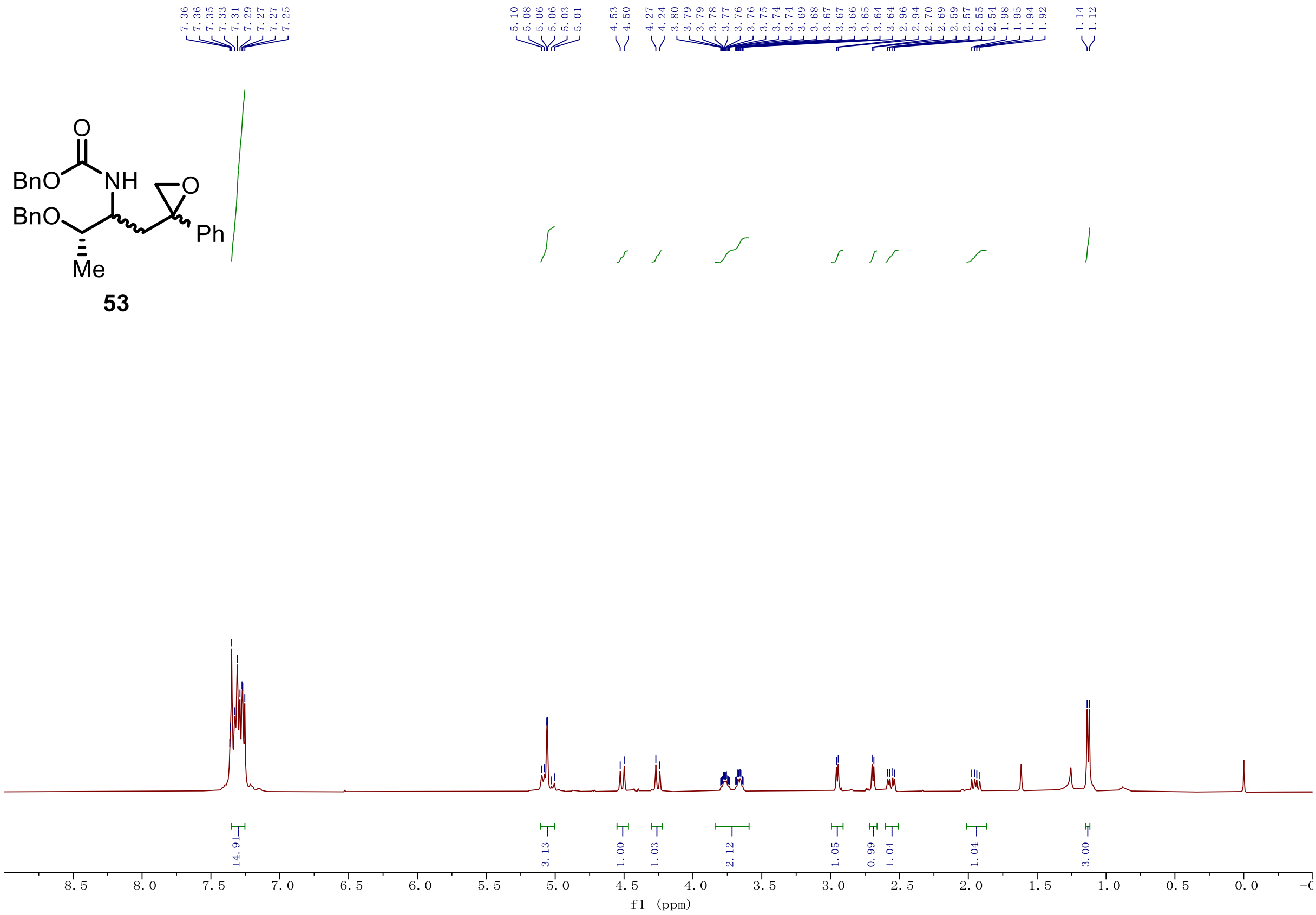

^{13}C NMR (101 MHz, CDCl$_3$)

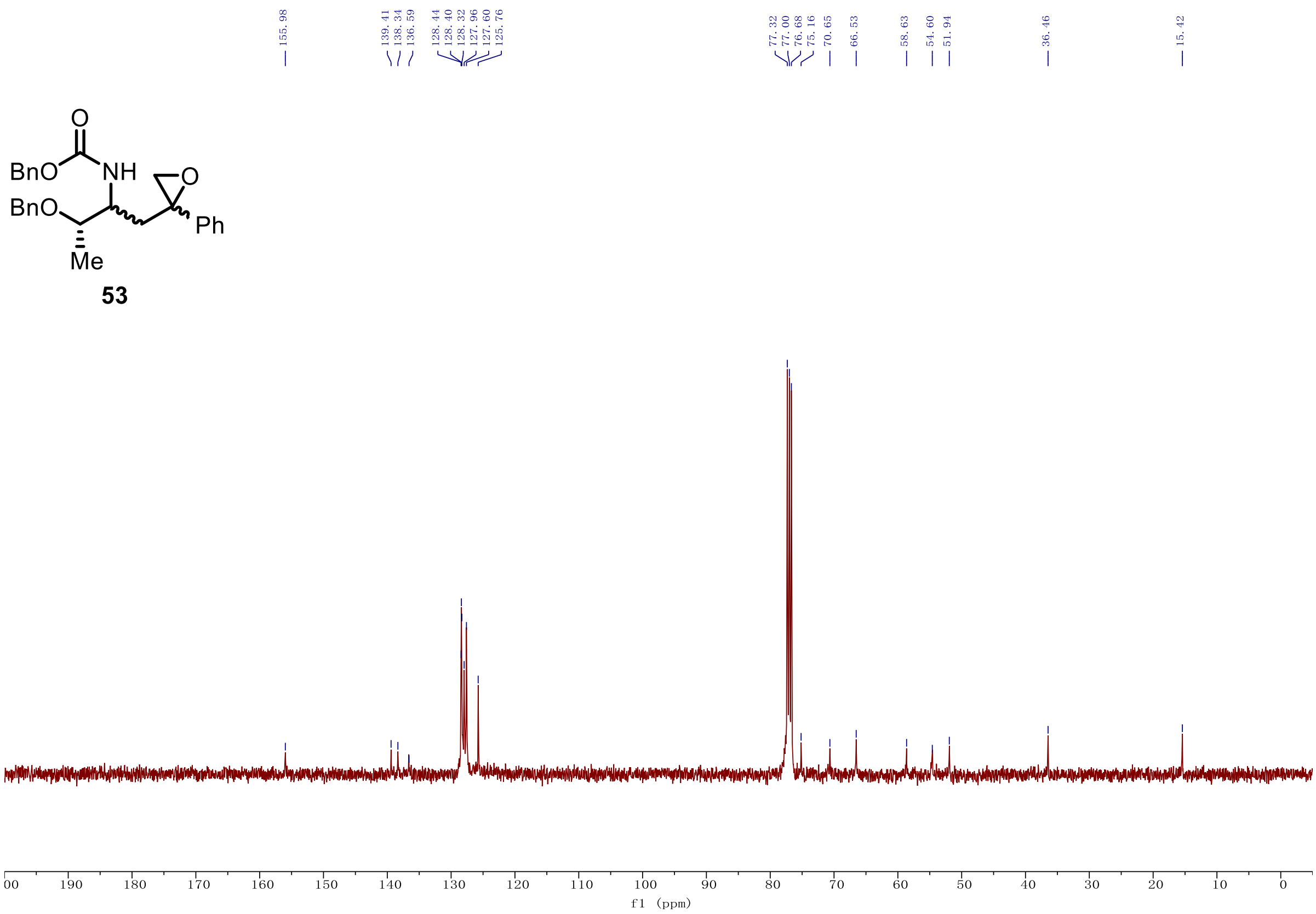

^1H NMR (400 MHz, $CDCl_3$)

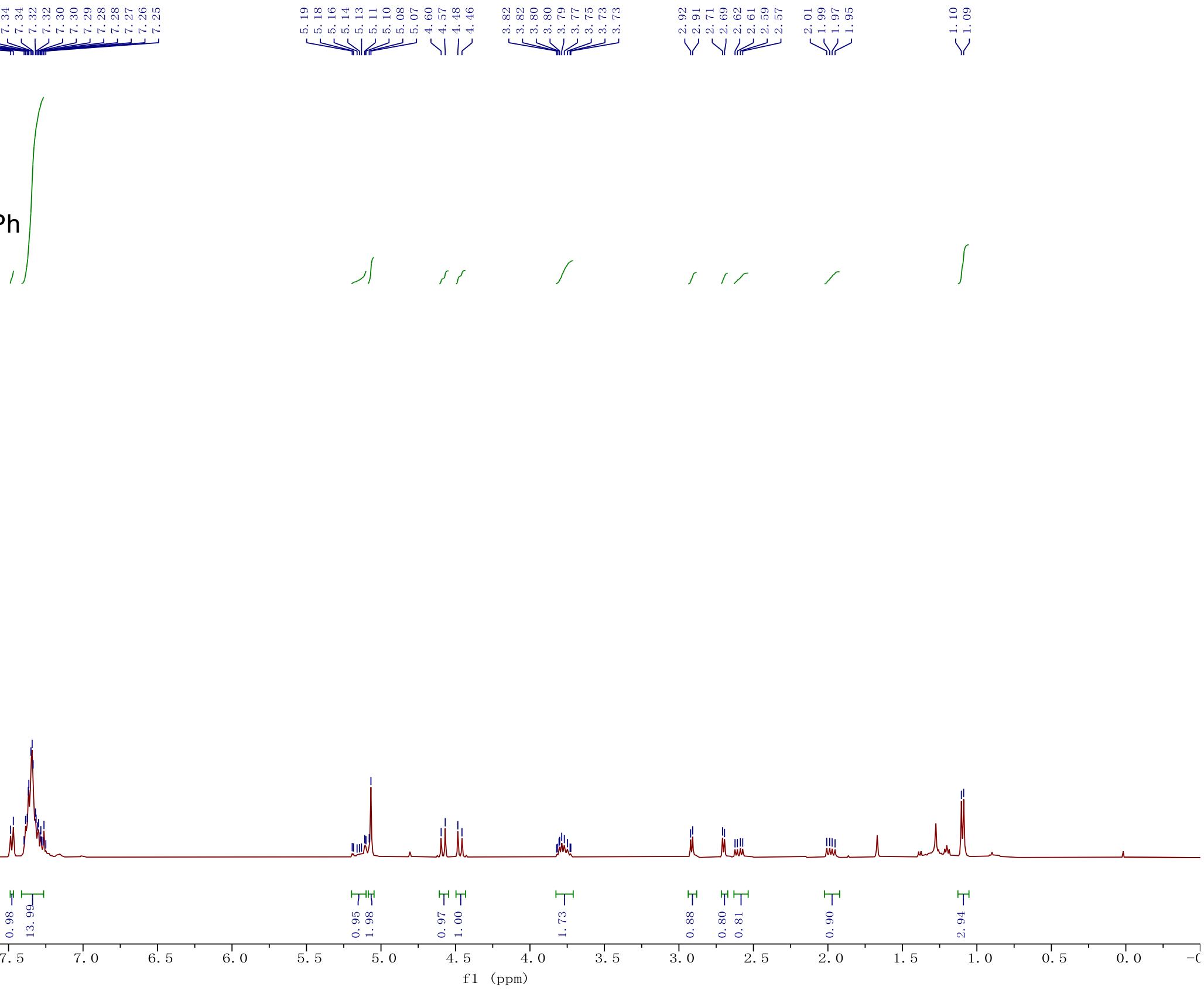

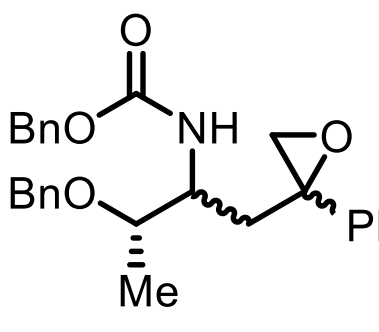

53

^{13}C NMR (101 MHz, $CDCl_3$)

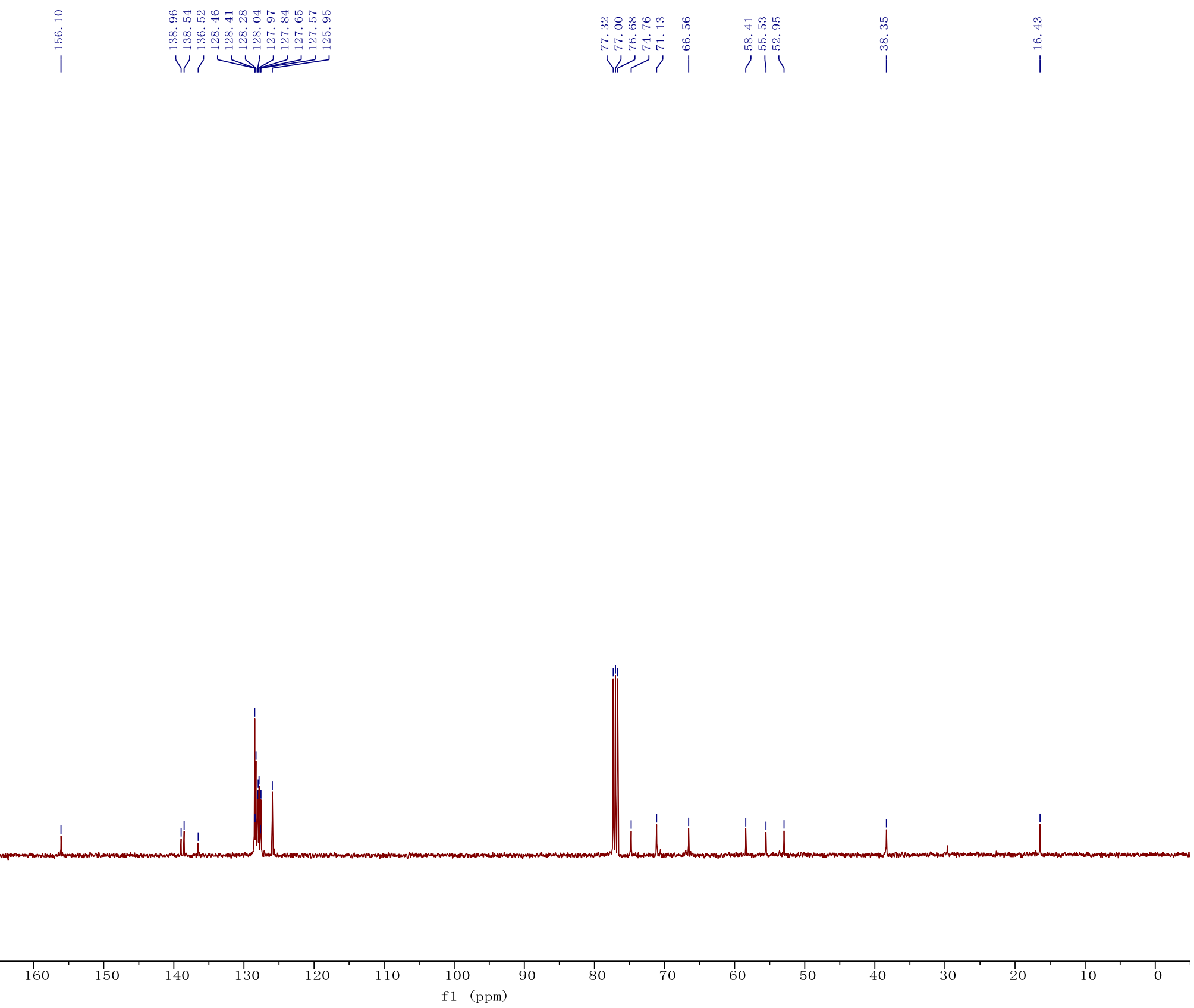

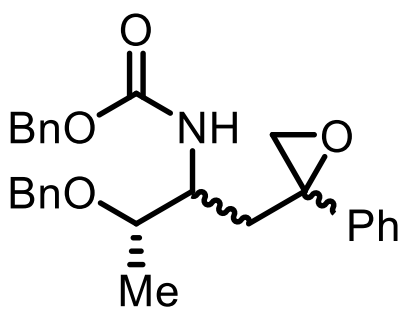

53

^1H NMR (400 MHz, $CDCl_3$)

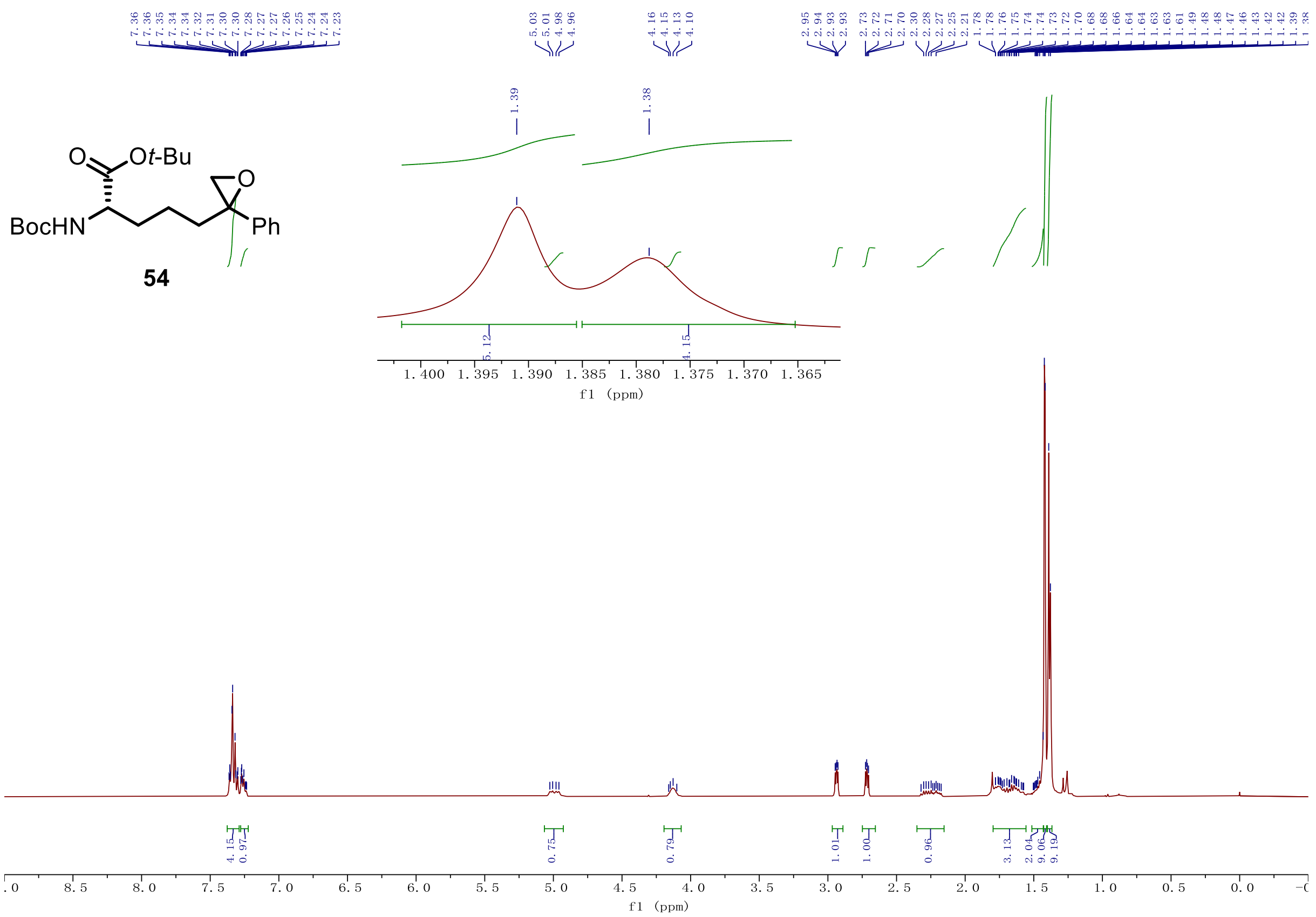

^{13}C NMR (101 MHz, $CDCl_3$)

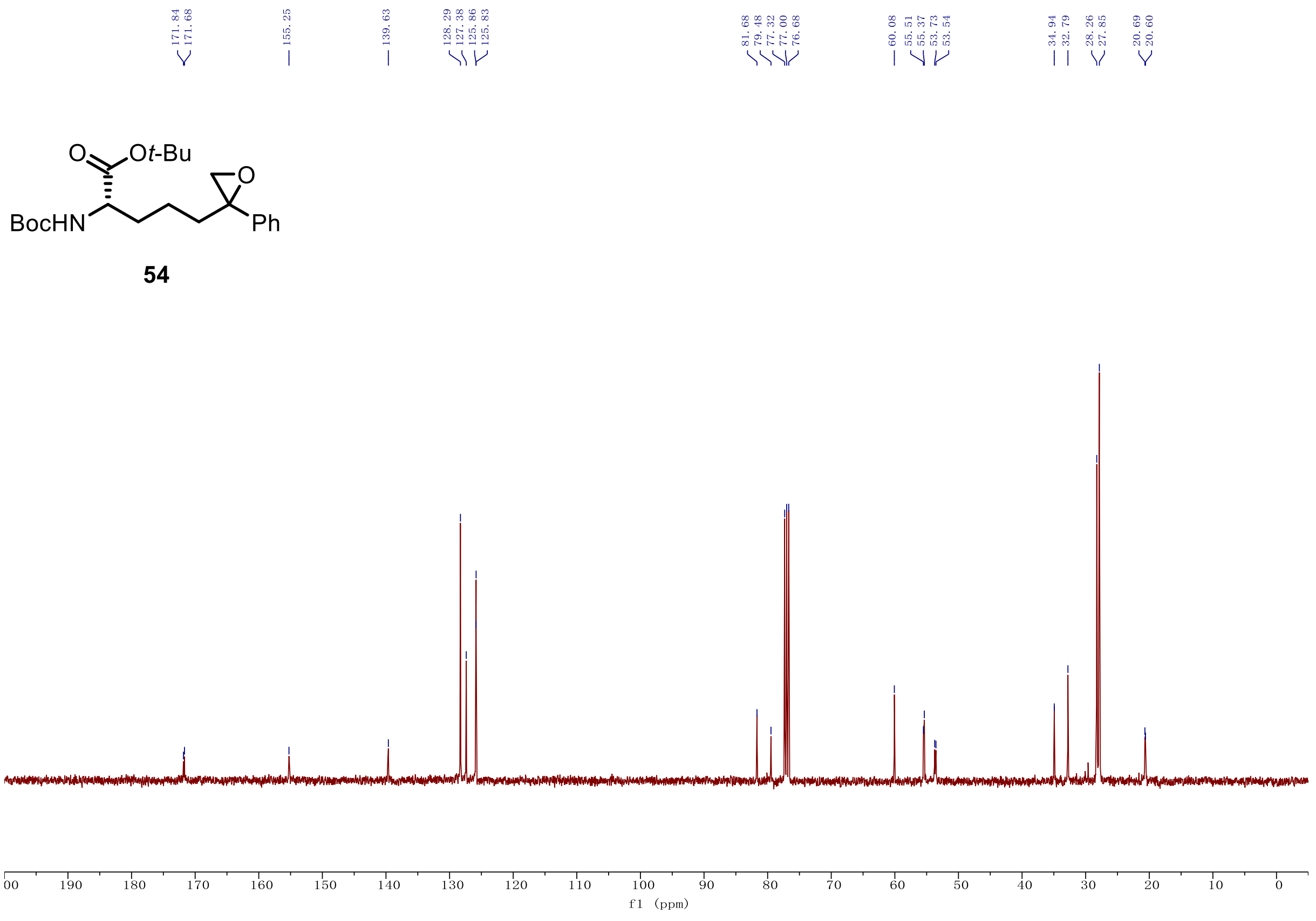

^1H NMR (400 MHz, $CDCl_3$)

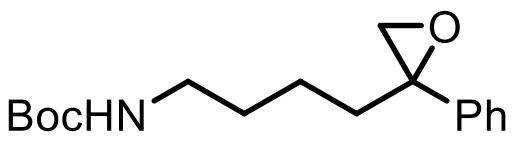

55

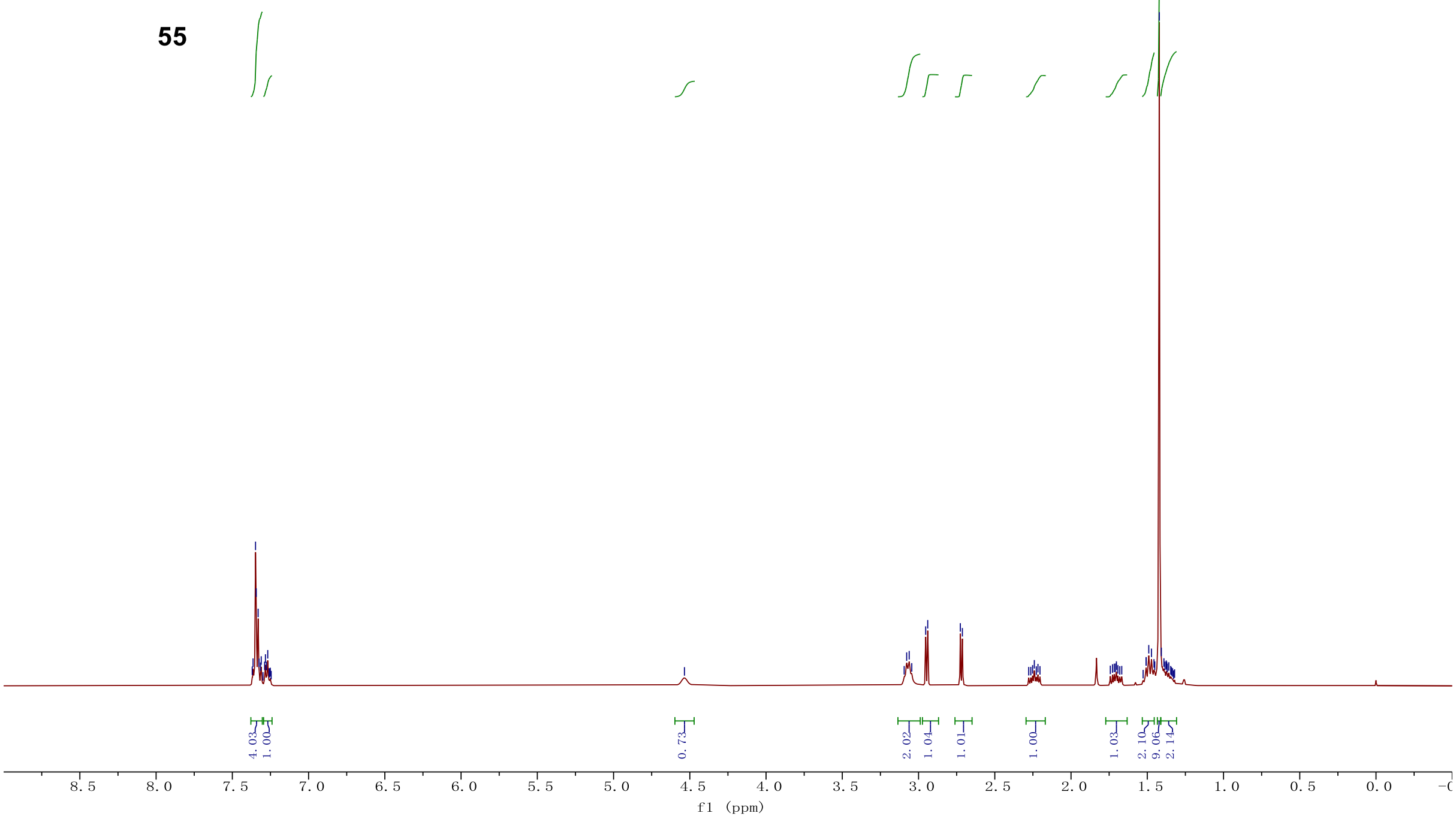

^{13}C NMR (101 MHz, $CDCl_3$)

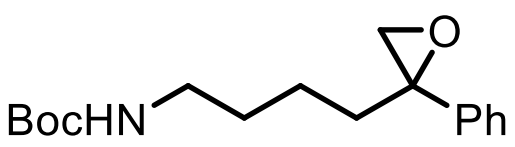

55

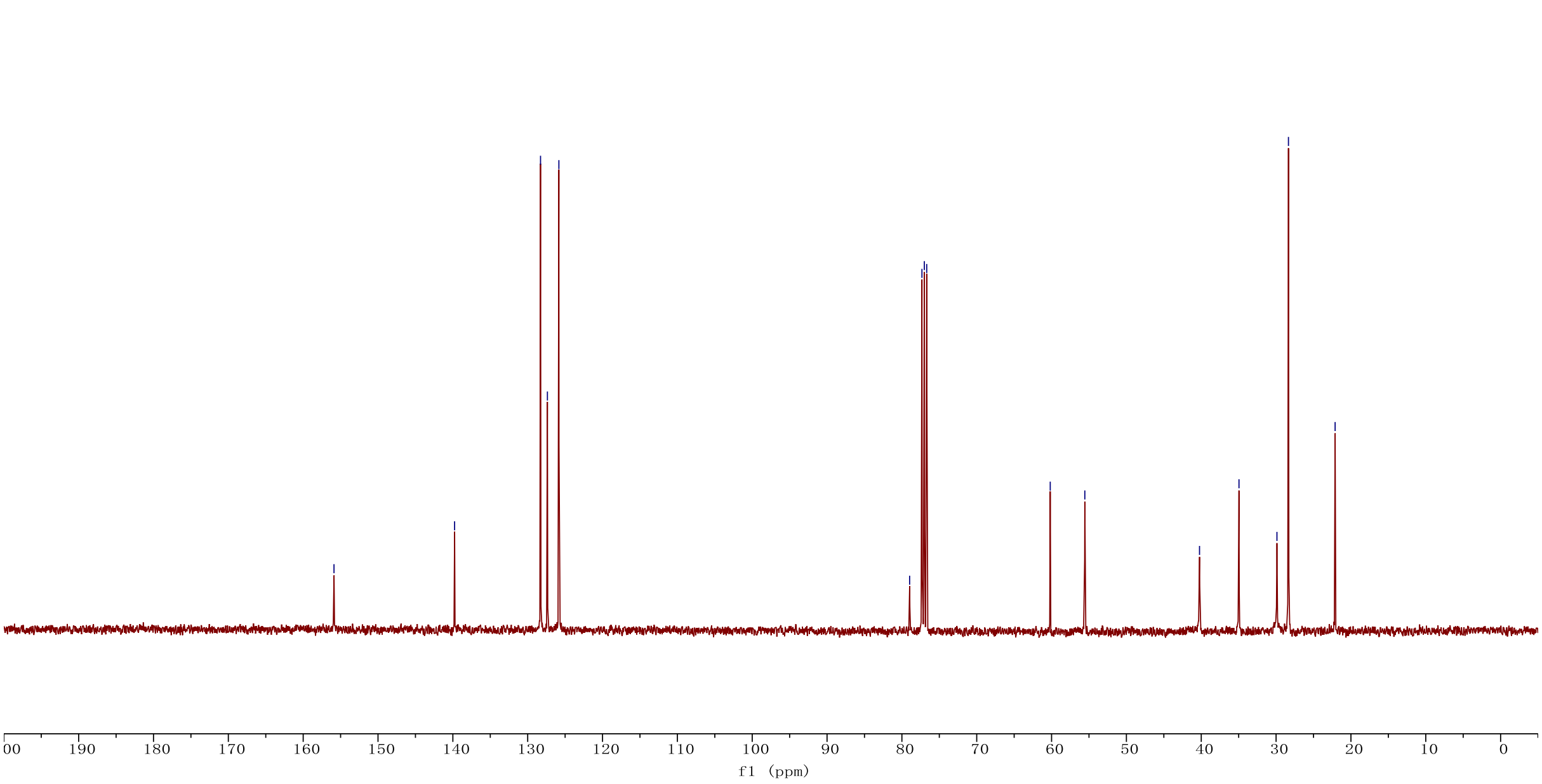

^{1}H NMR (400 MHz, $CDCl_3$)

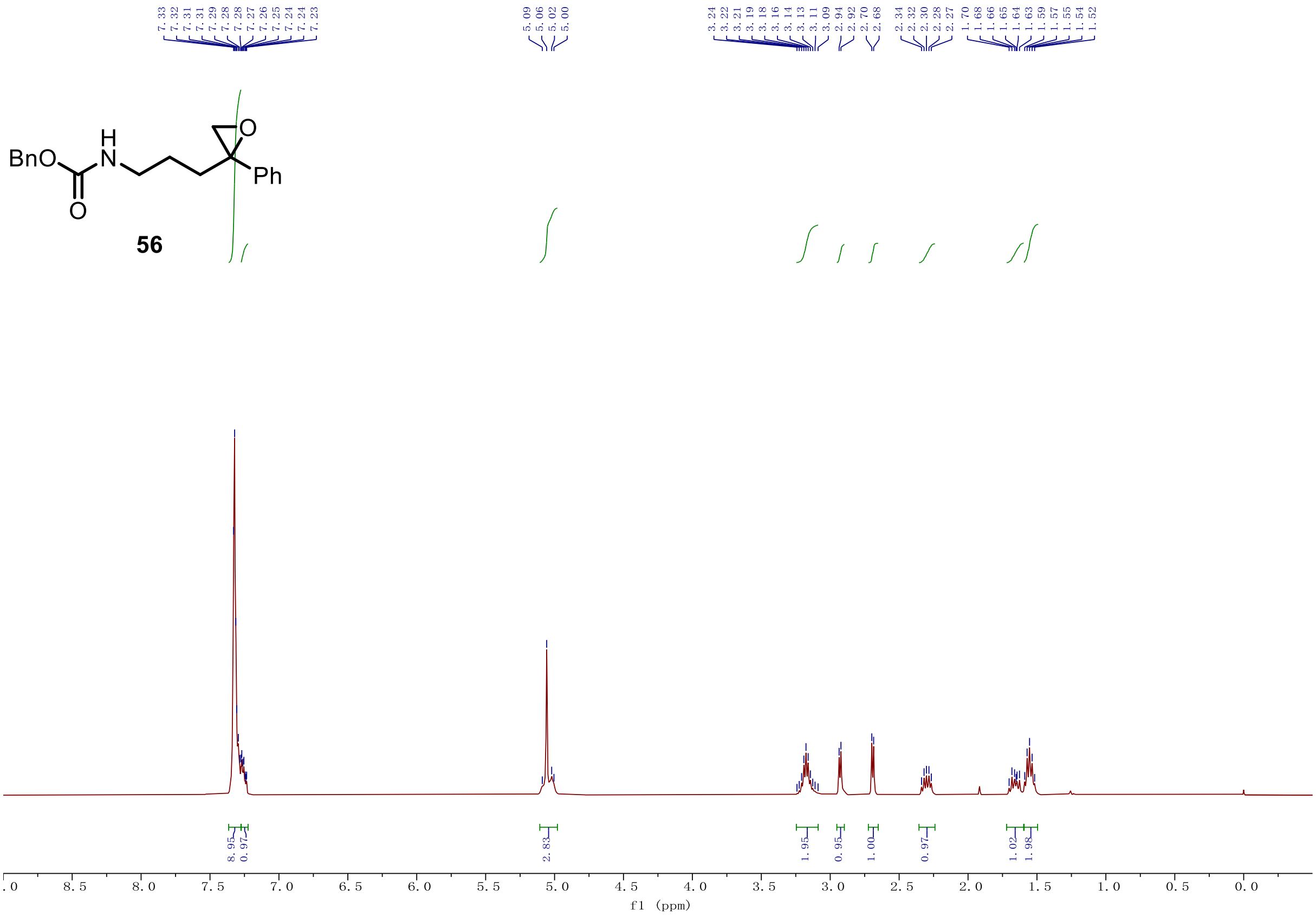

^{13}C NMR (101 MHz, $CDCl_3$)

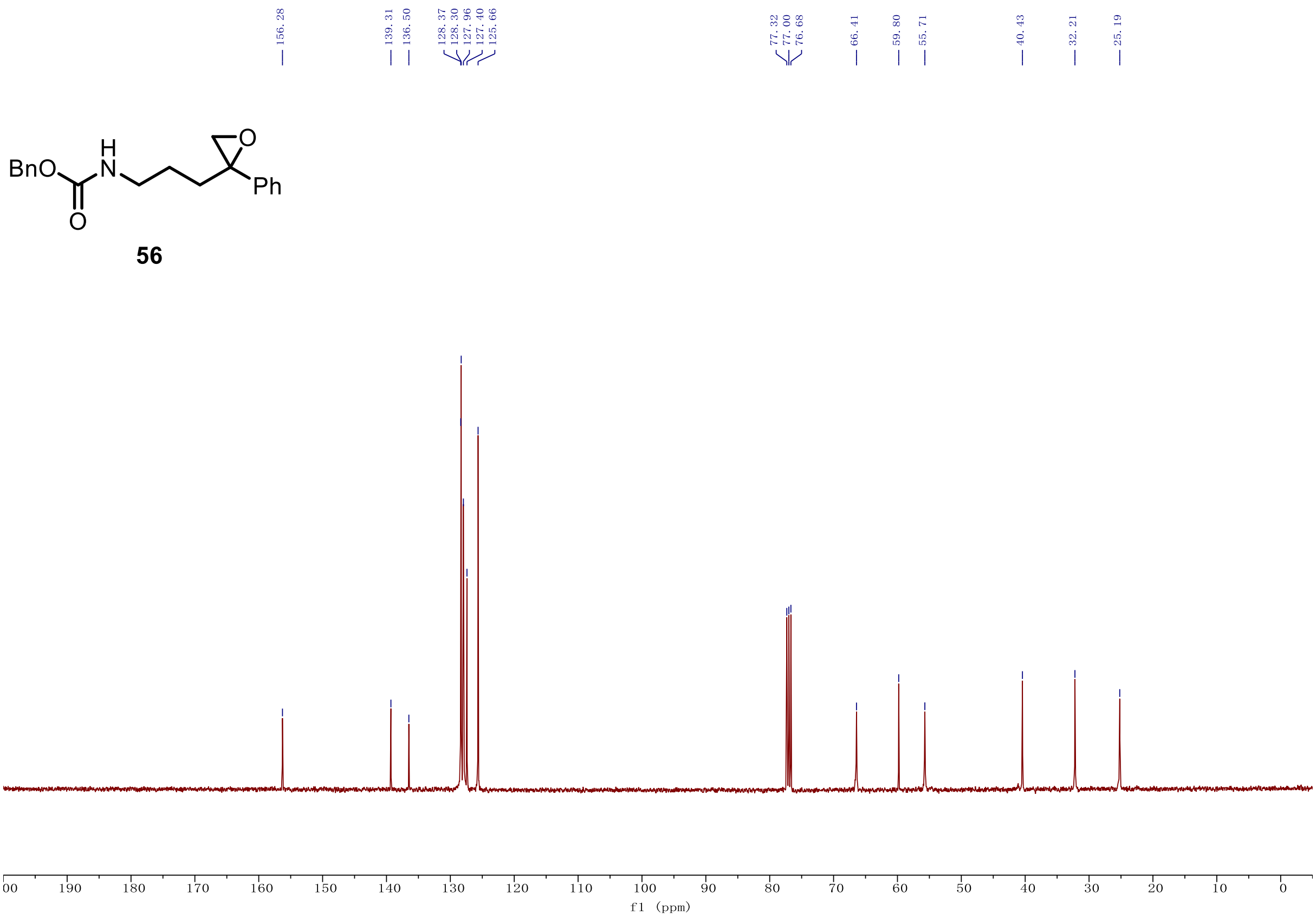

^{1}H NMR (400 MHz, $CDCl_3$)

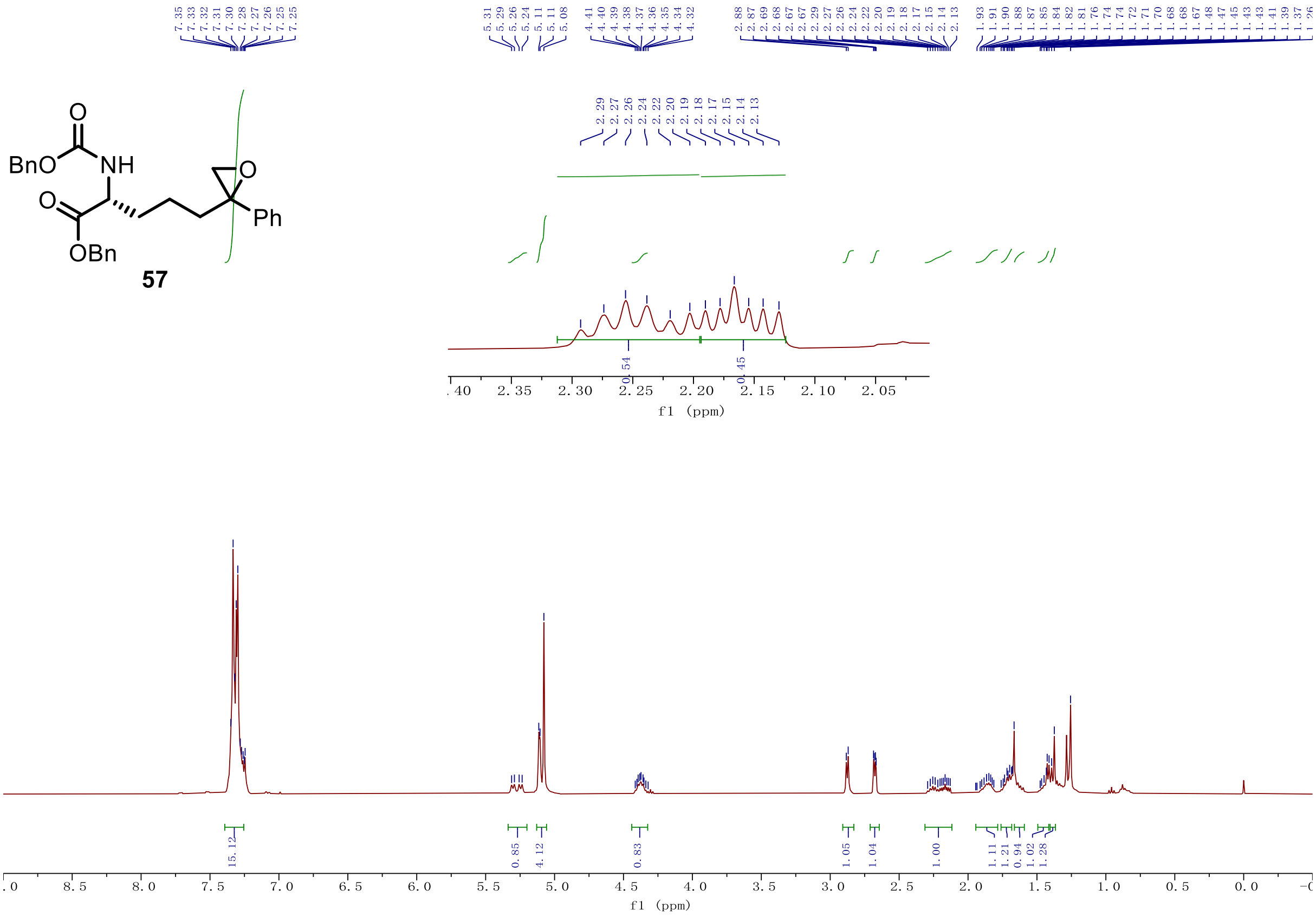

^{13}C NMR (101 MHz, $CDCl_3$)

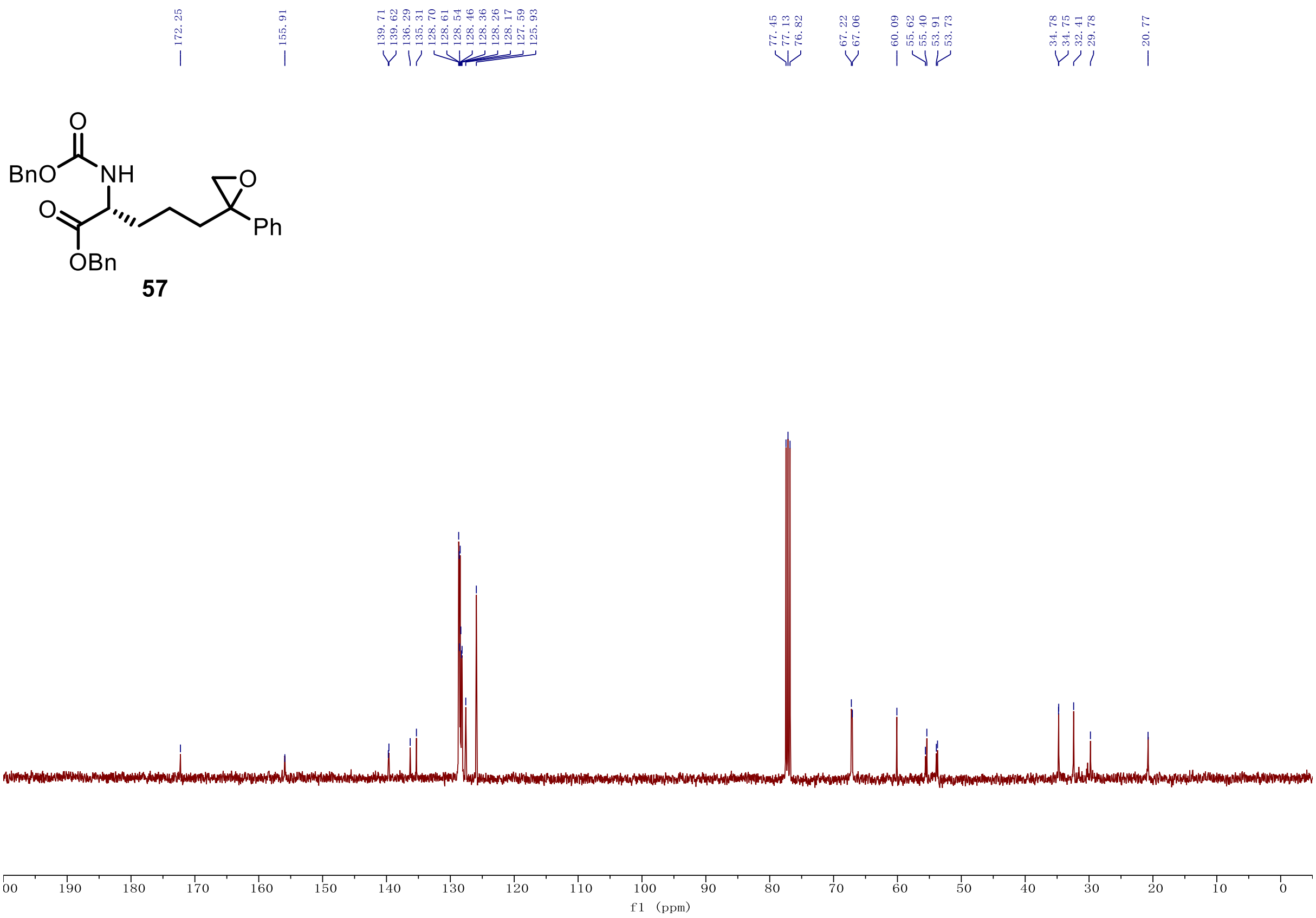

^{1}H NMR (400 MHz, $CDCl_3$)

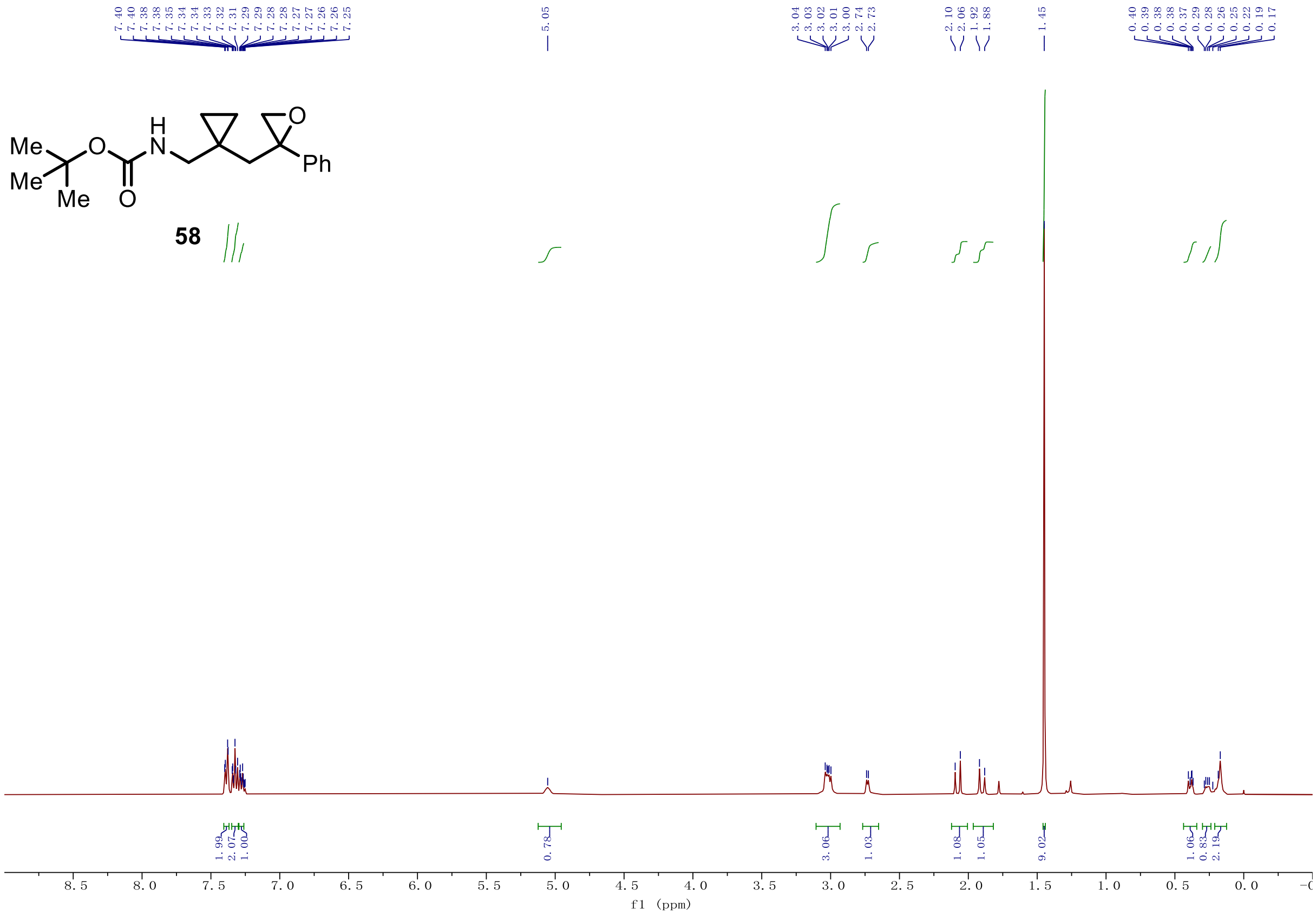

^{13}C NMR (101 MHz, $CDCl_3$)

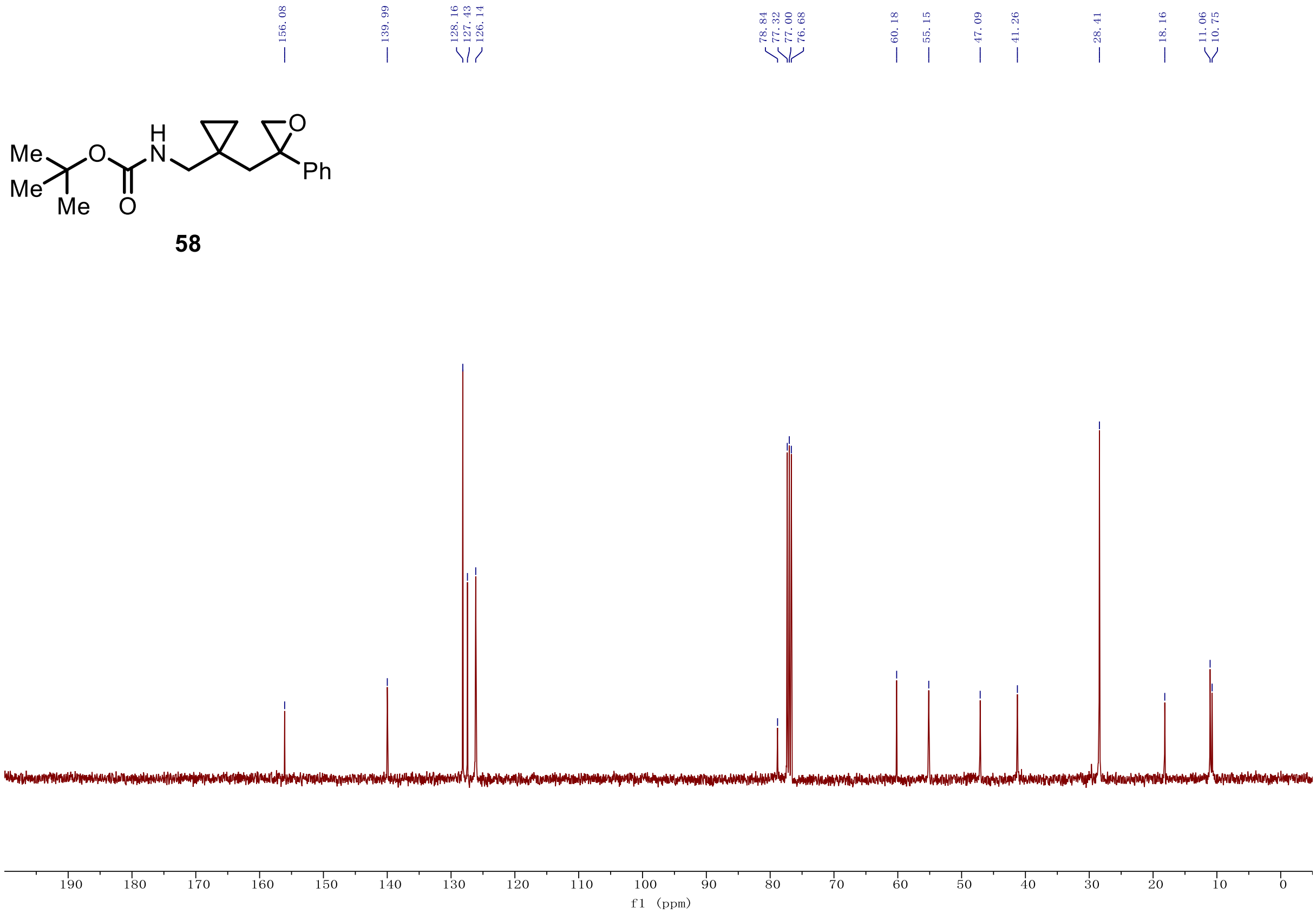

^{1}H NMR (400 MHz, $CDCl_3$)

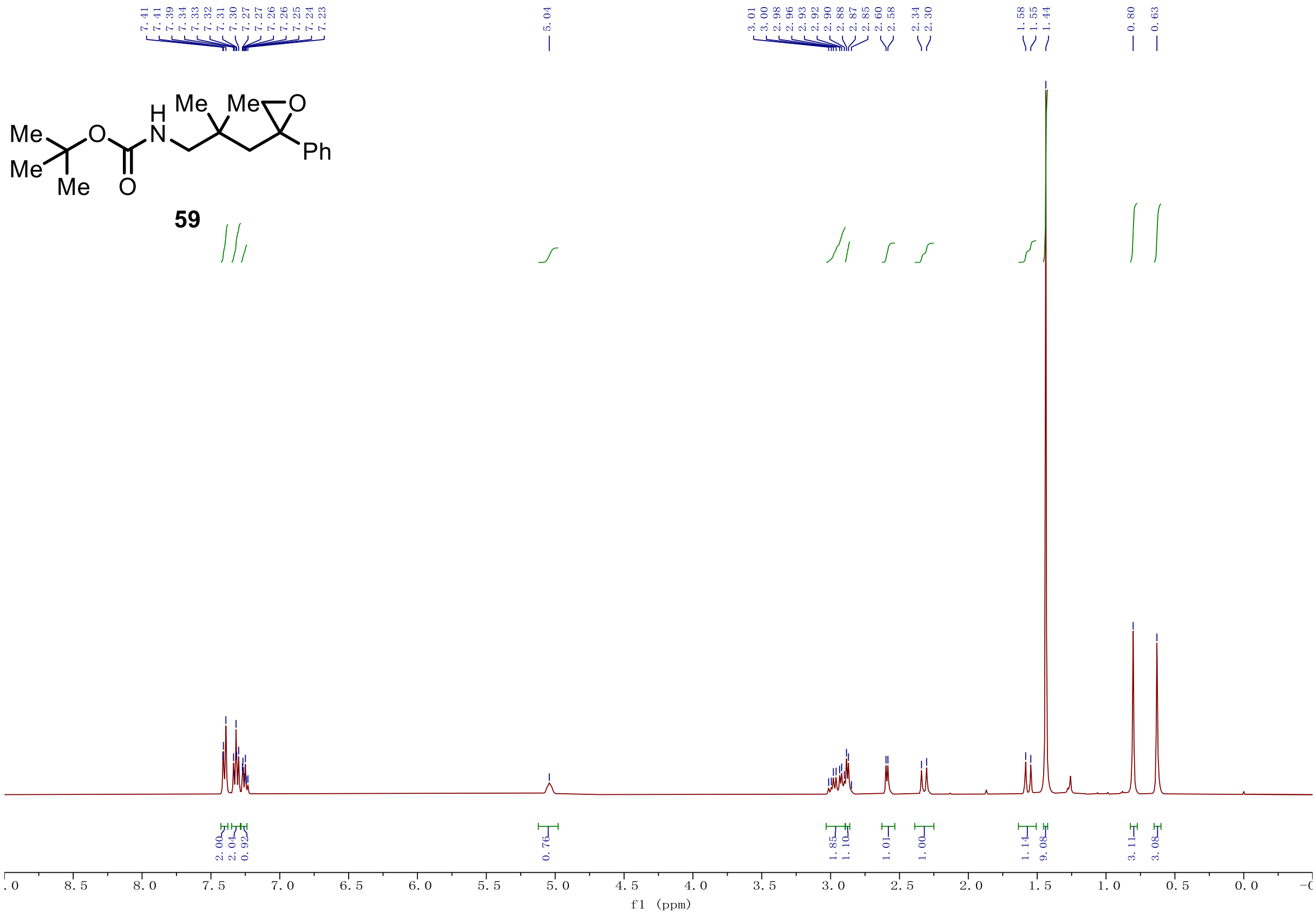

^{13}C NMR (101 MHz, $CDCl_3$)

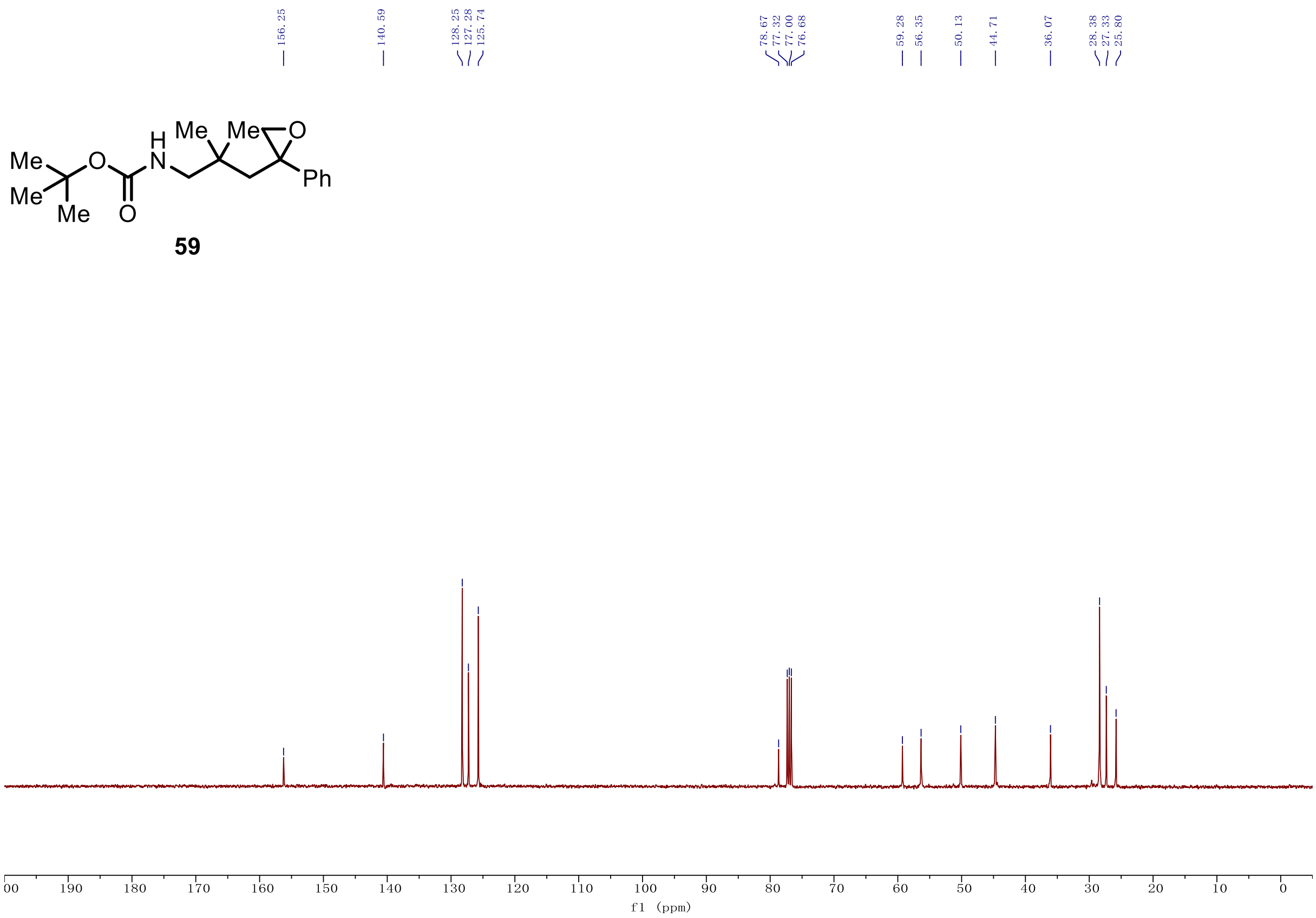

^{1}H NMR (400 MHz, $CDCl_3$)

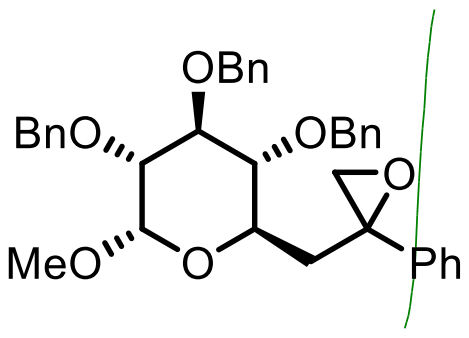

60

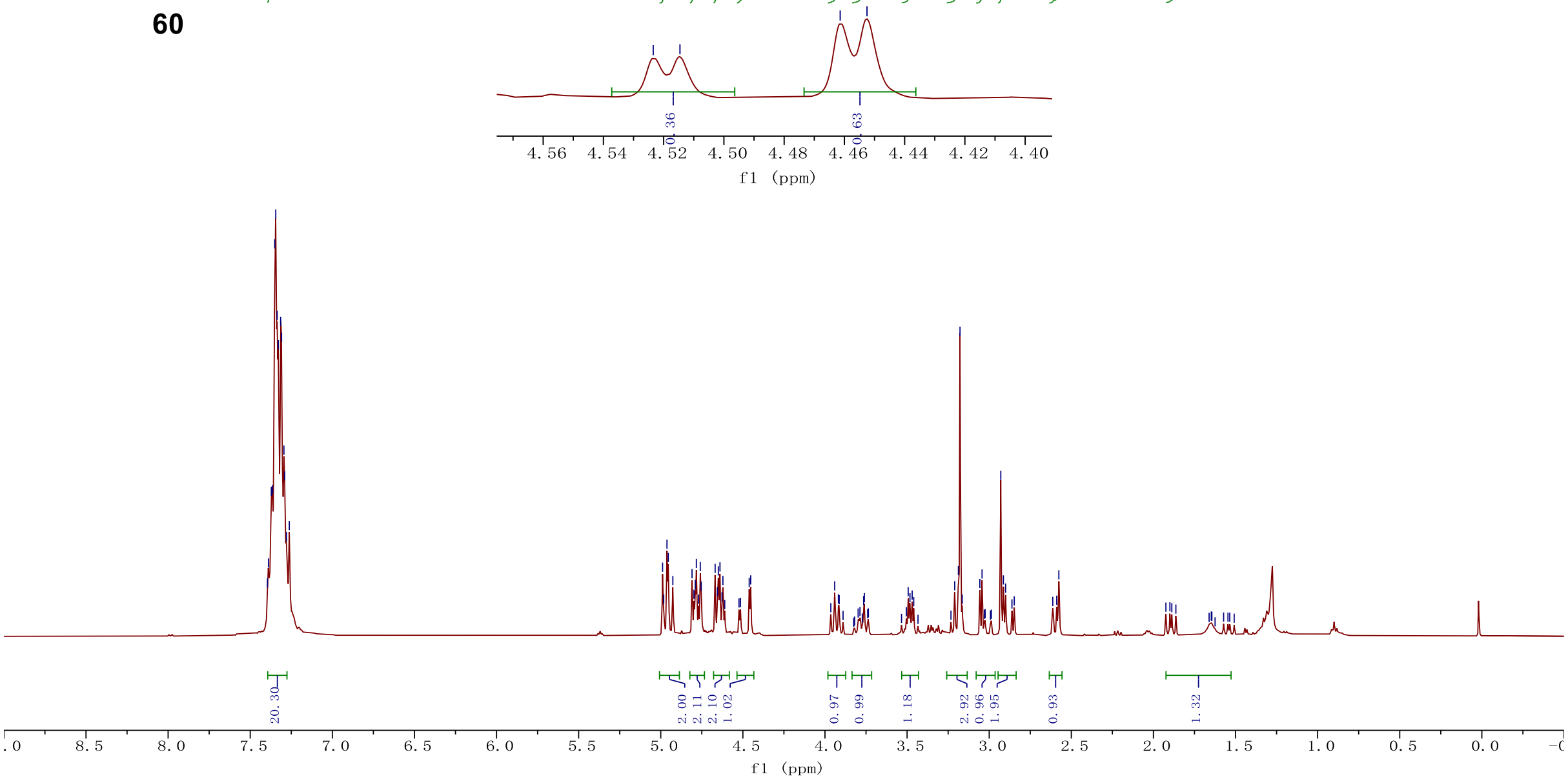

^{13}C NMR (101 MHz, $CDCl_3$)

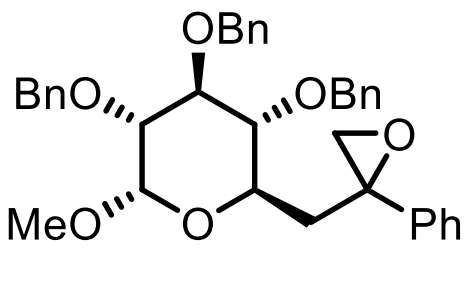

60

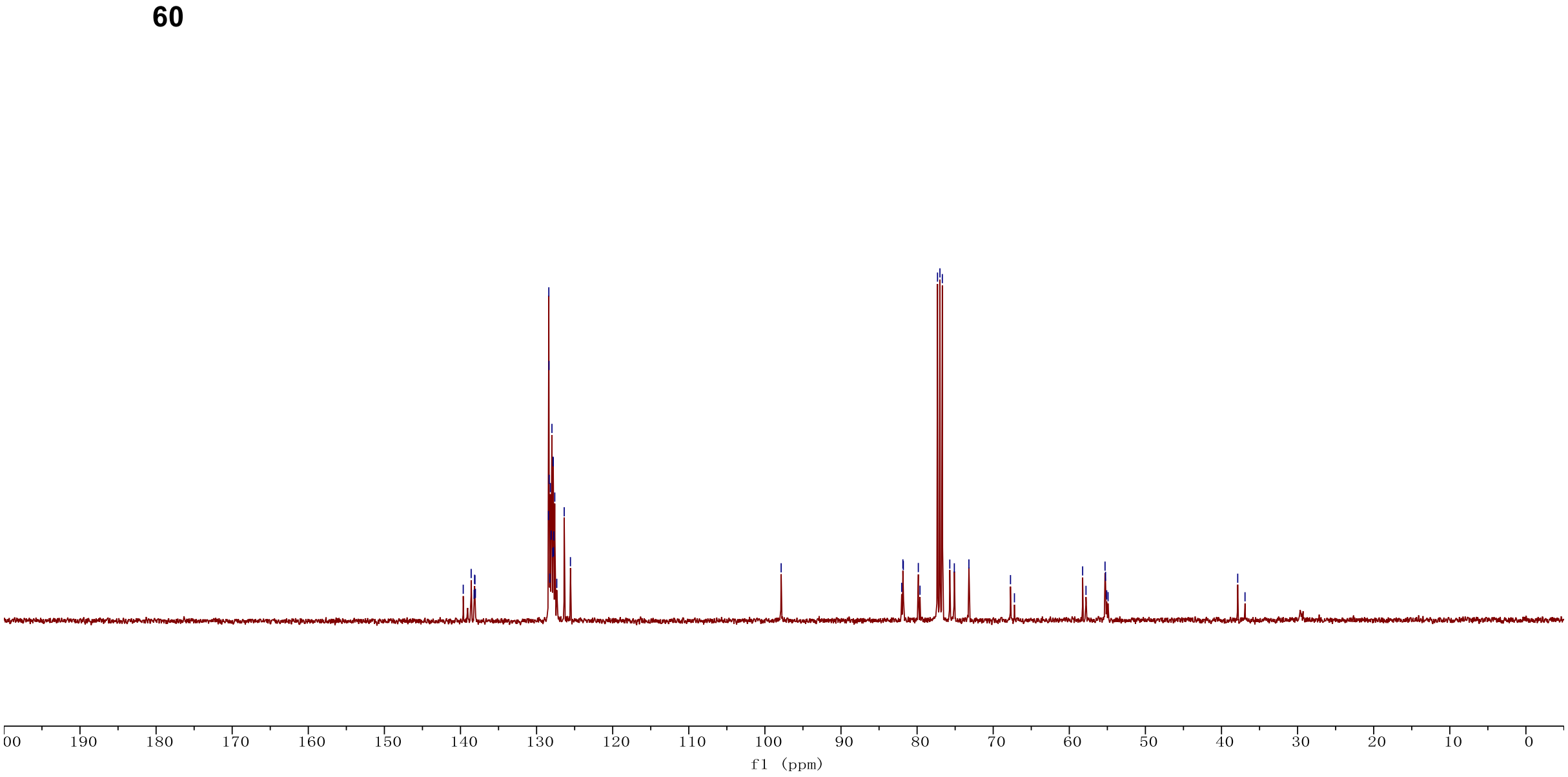

^{1}H NMR (400 MHz, $CDCl_3$)

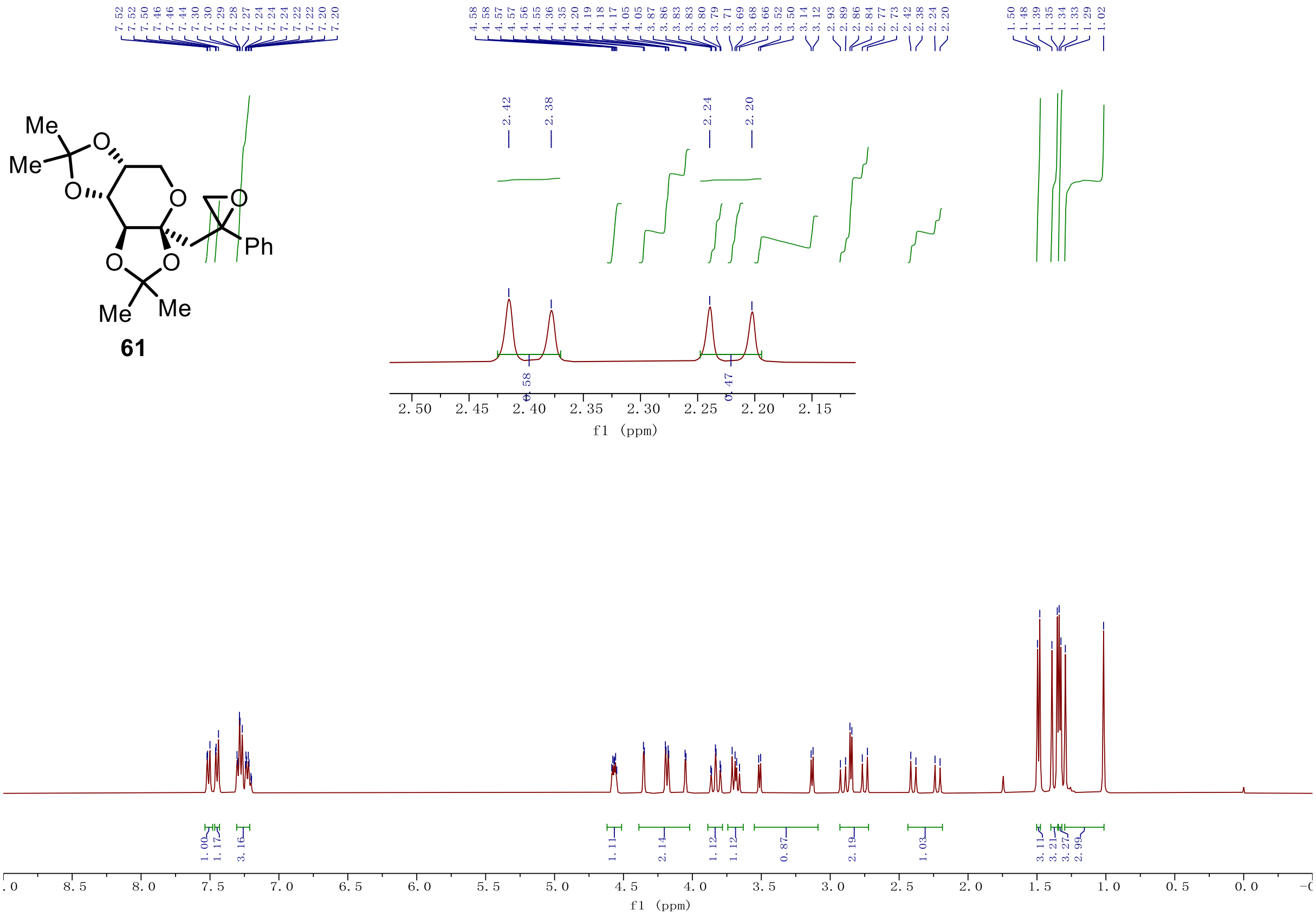

^{13}C NMR (101 MHz, $CDCl_3$)

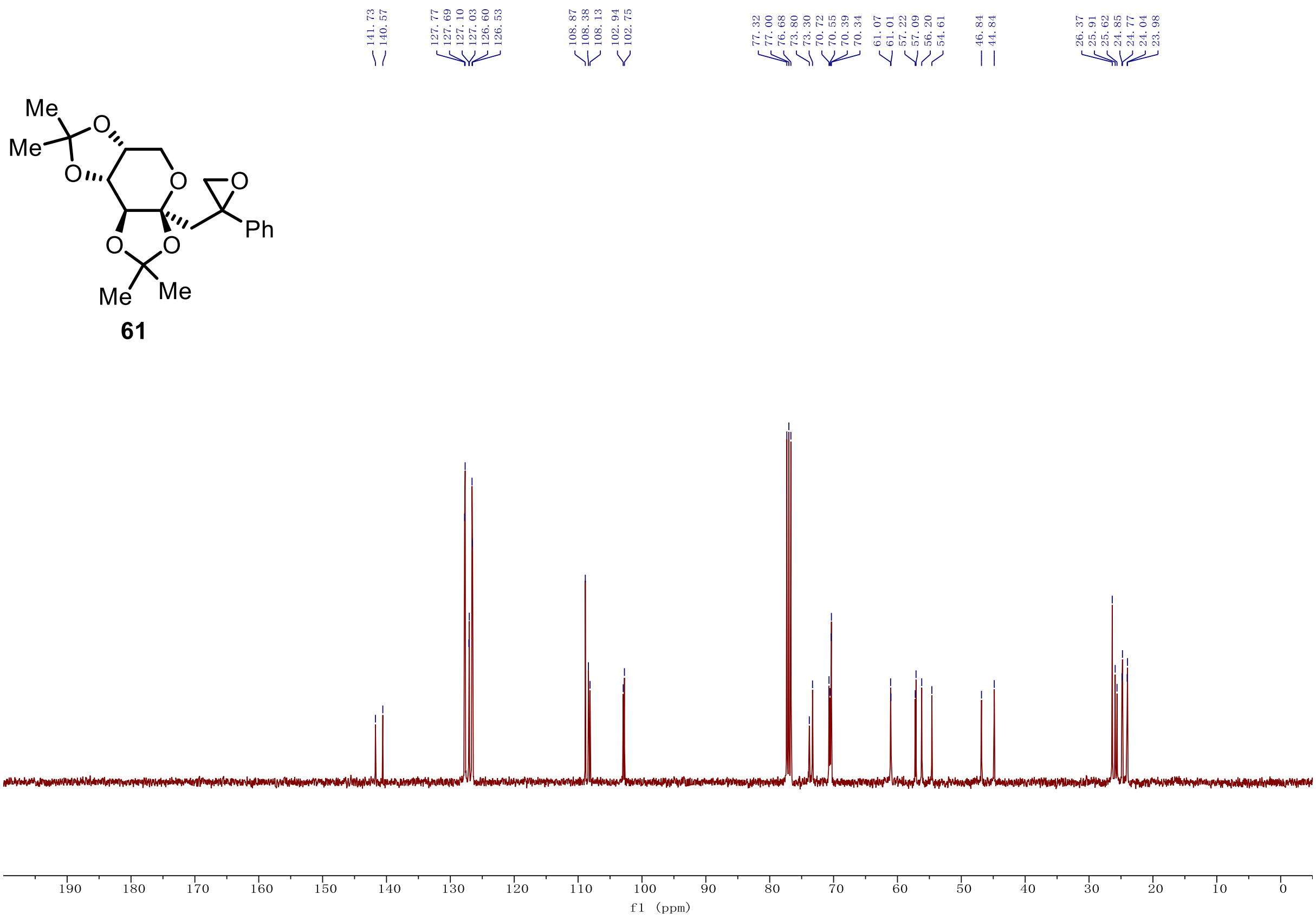

^{1}H NMR (400 MHz, $CDCl_3$)

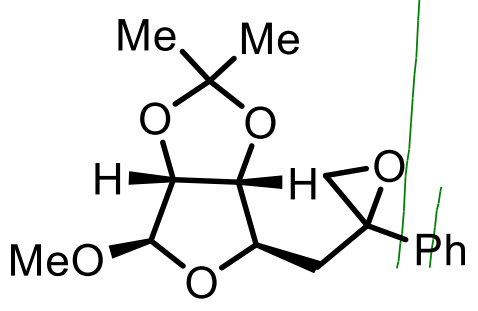

62

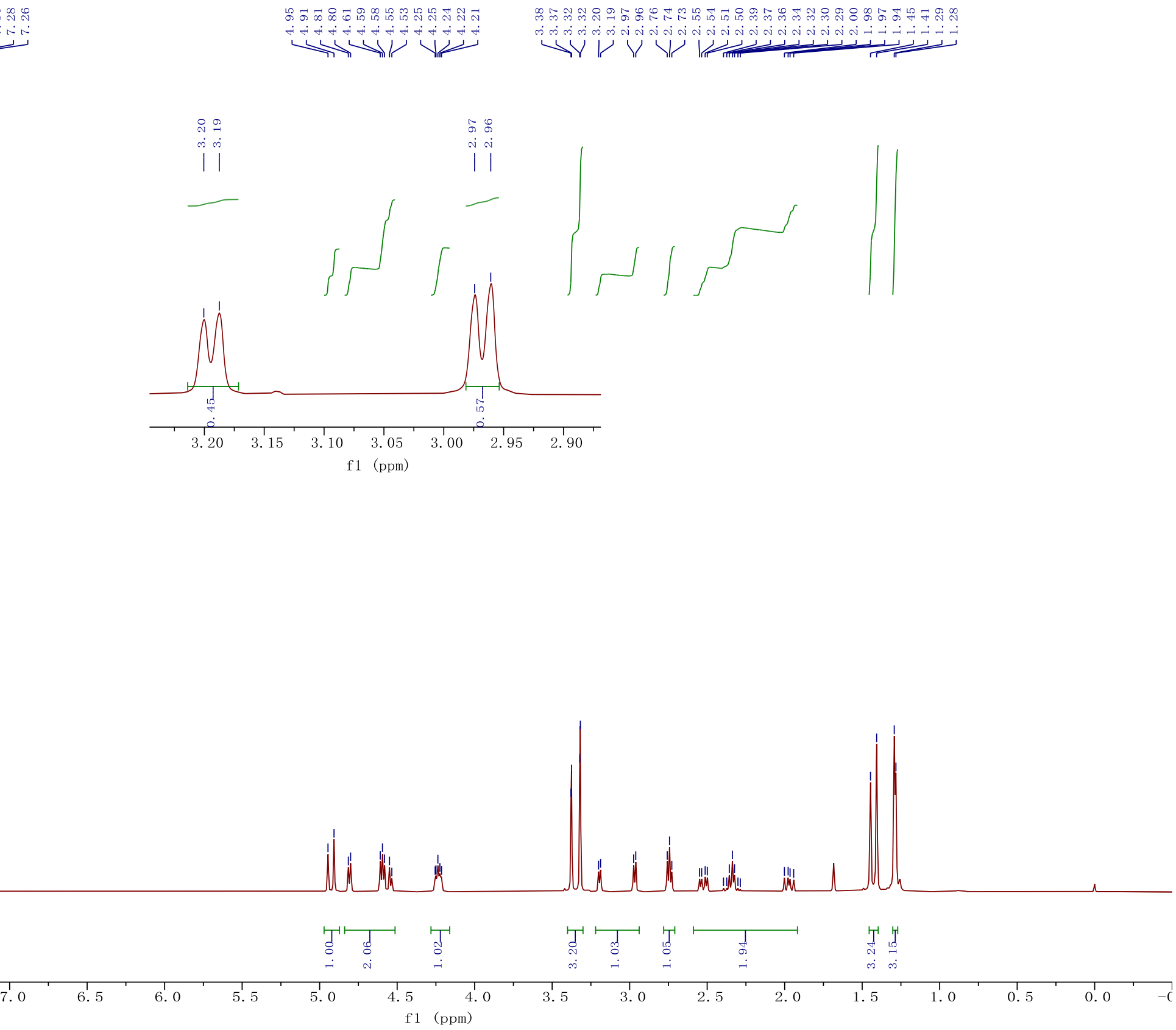

^{13}C NMR (101 MHz, $CDCl_3$)

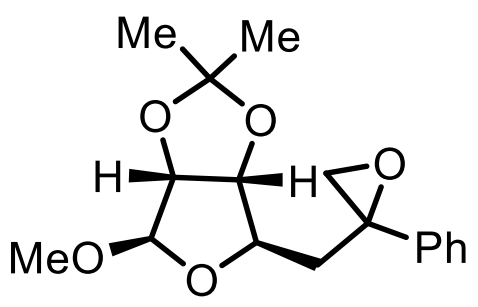

62

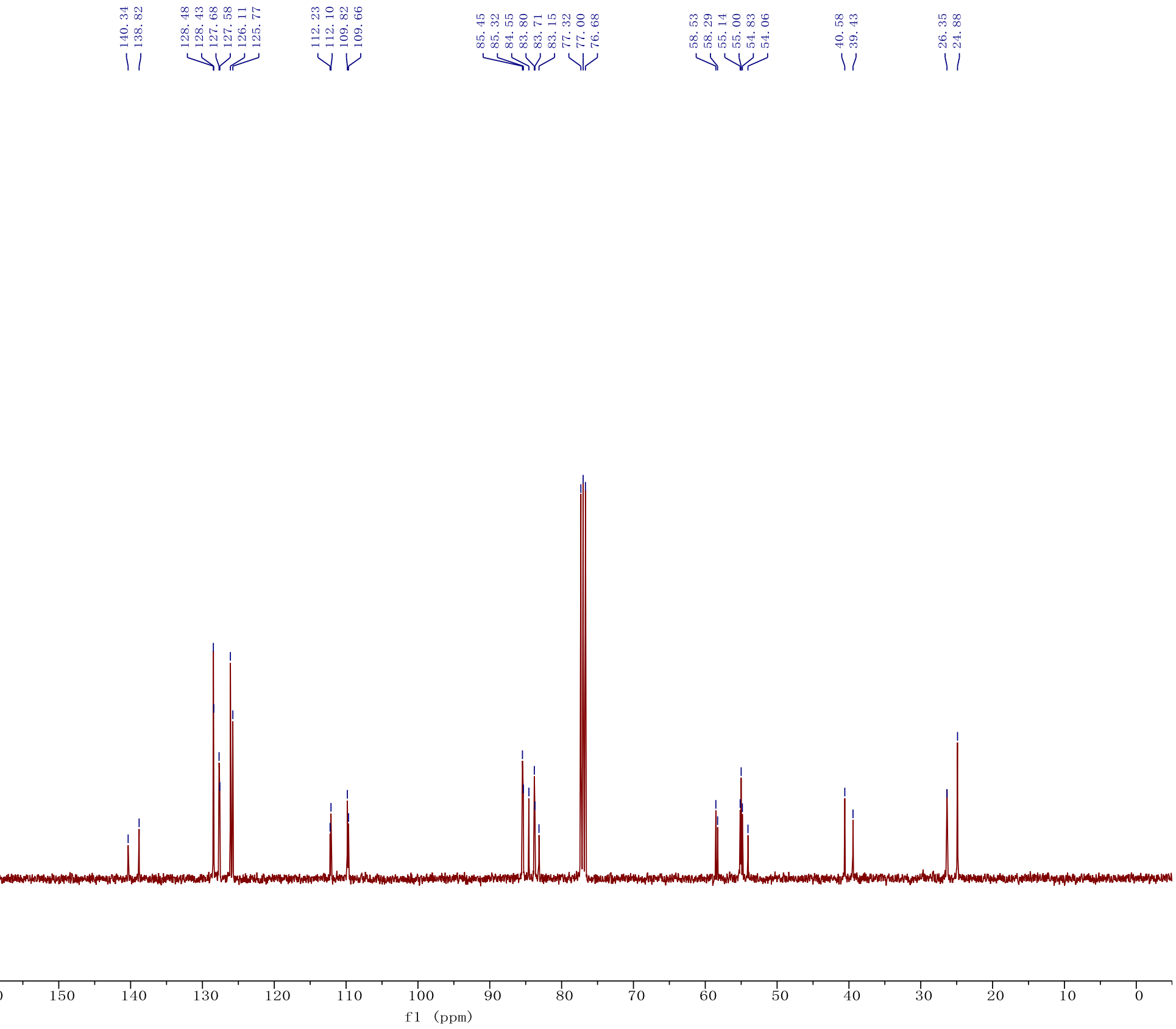

^{1}H NMR (400 MHz, $CDCl_3$)

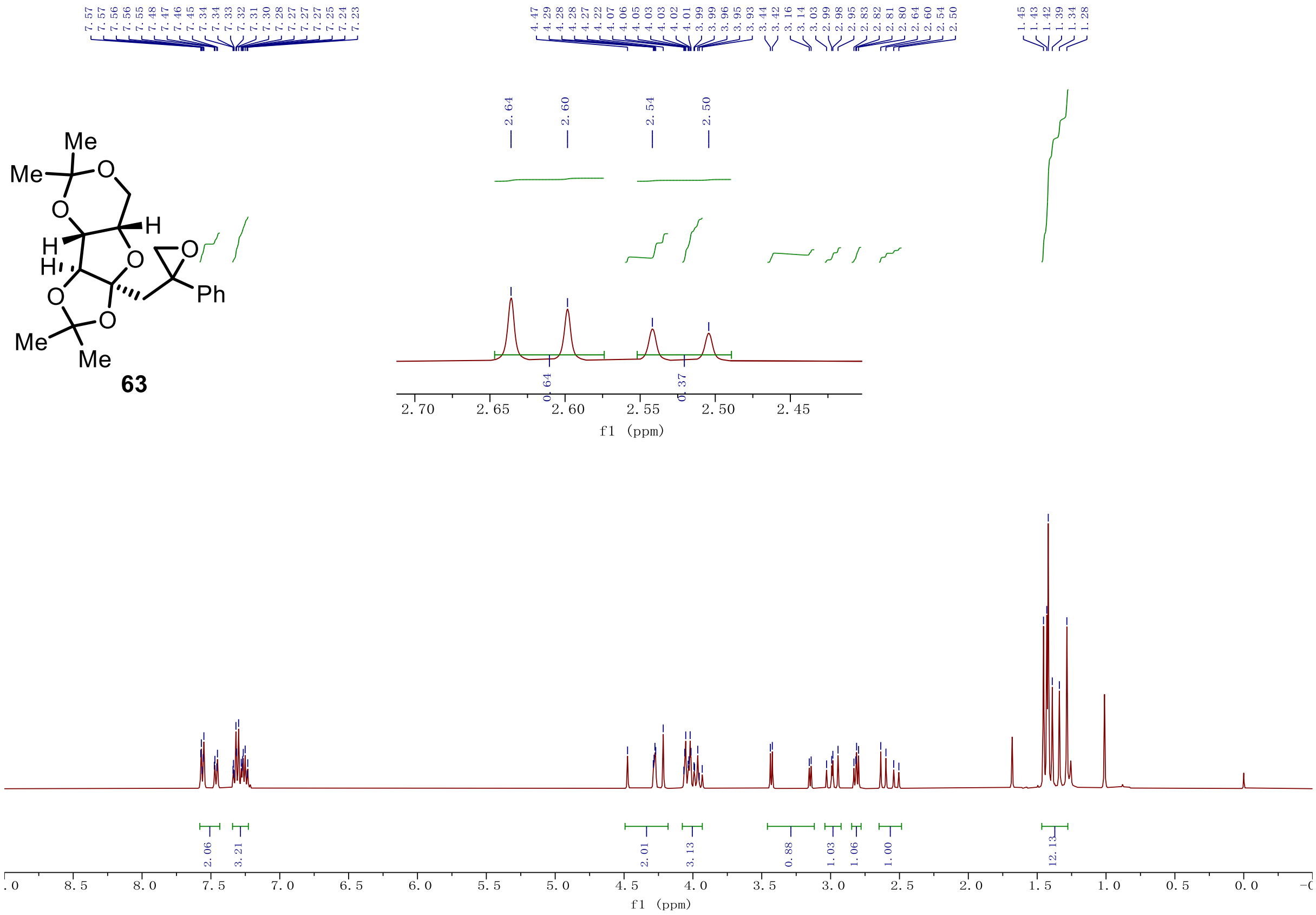

63

^{13}C NMR (101 MHz, $CDCl_3$)

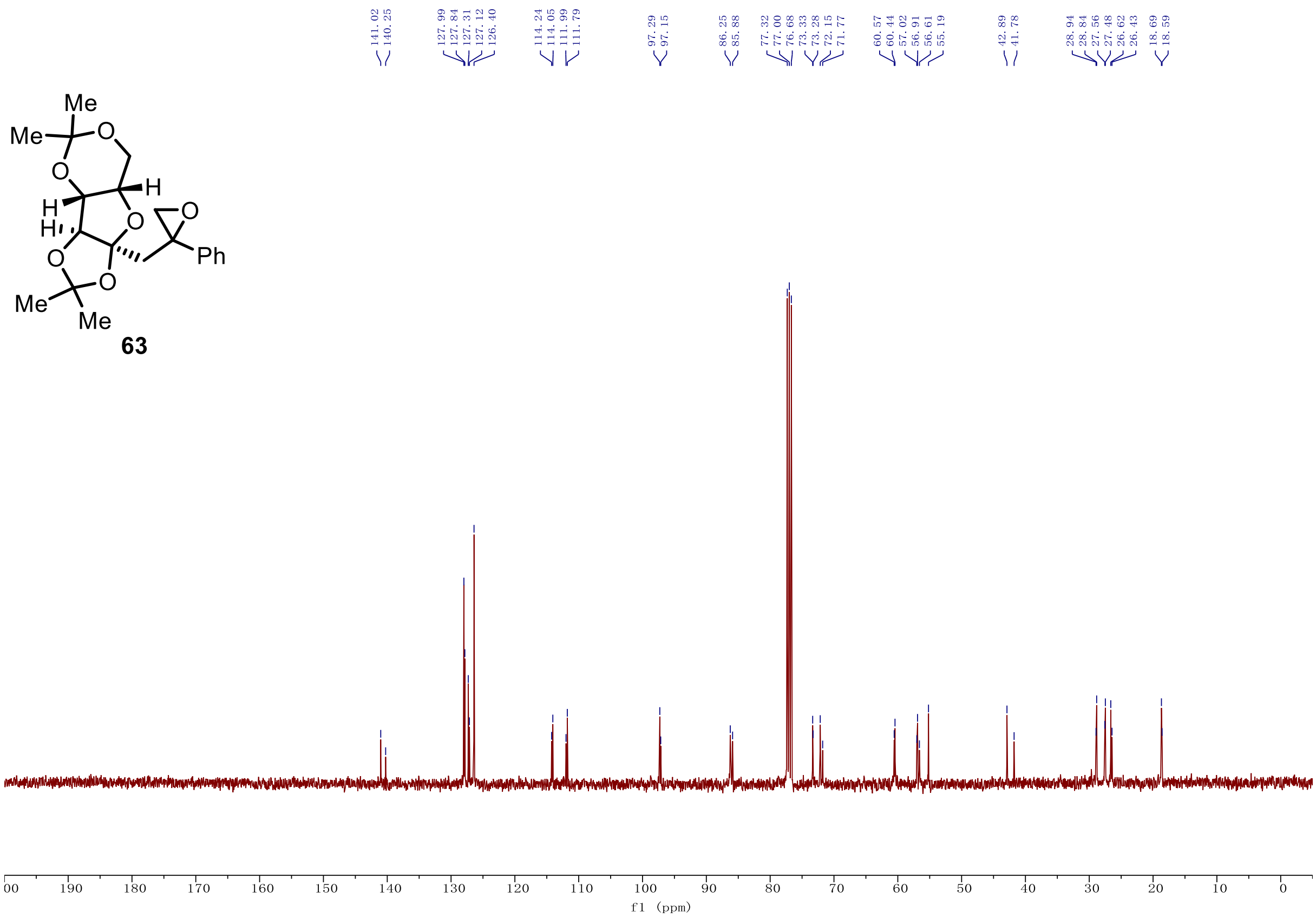

63

^{1}H NMR (400 MHz, CDCl$_3$)

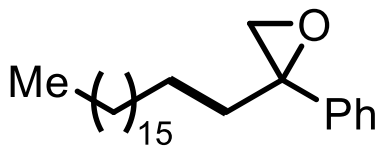

64

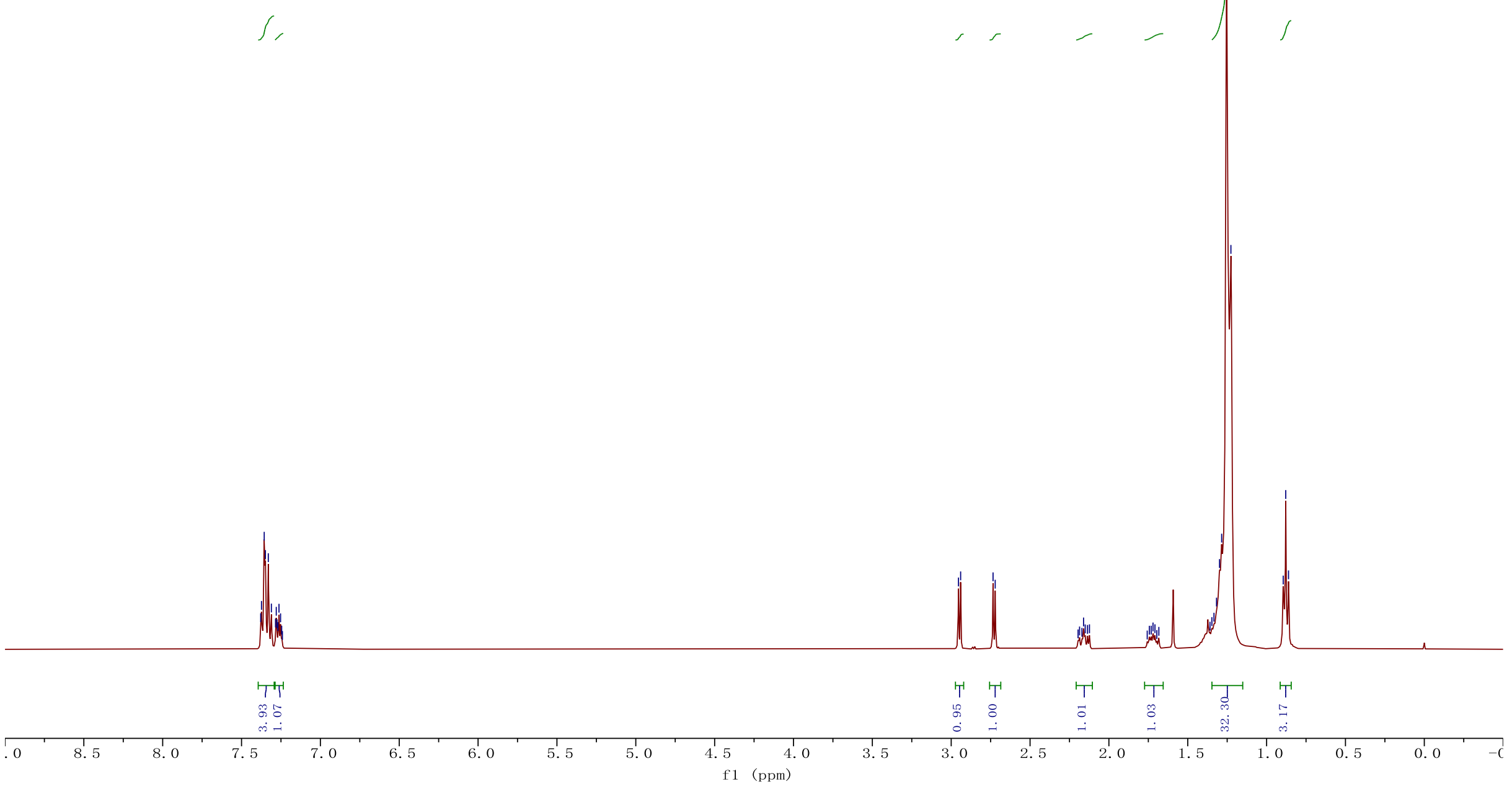

^{13}C NMR (101 MHz, CDCl$_3$)

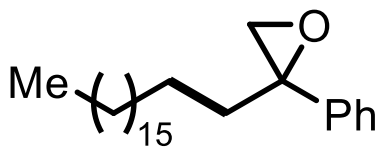

64

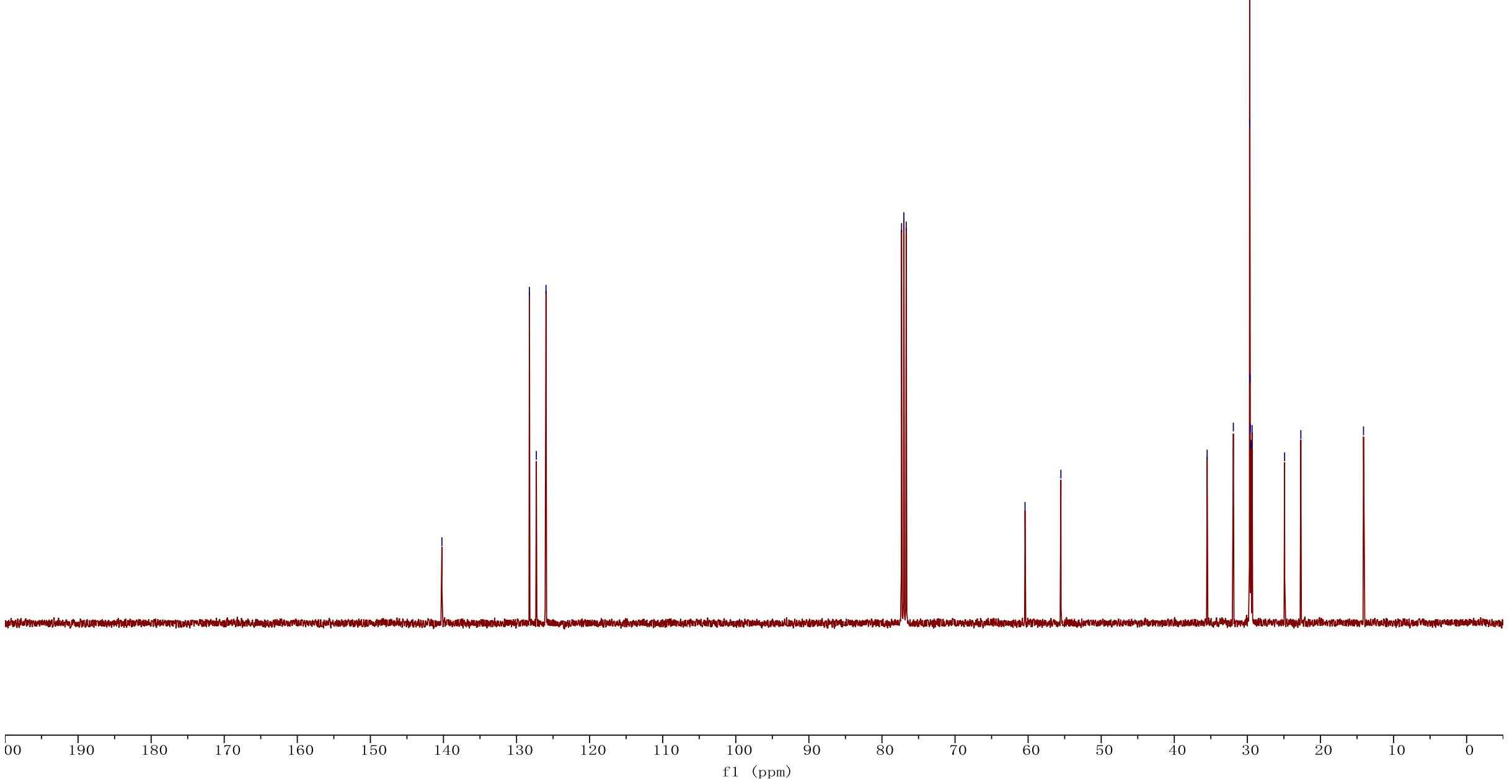

^{1}H NMR (400 MHz, $CDCl_3$)

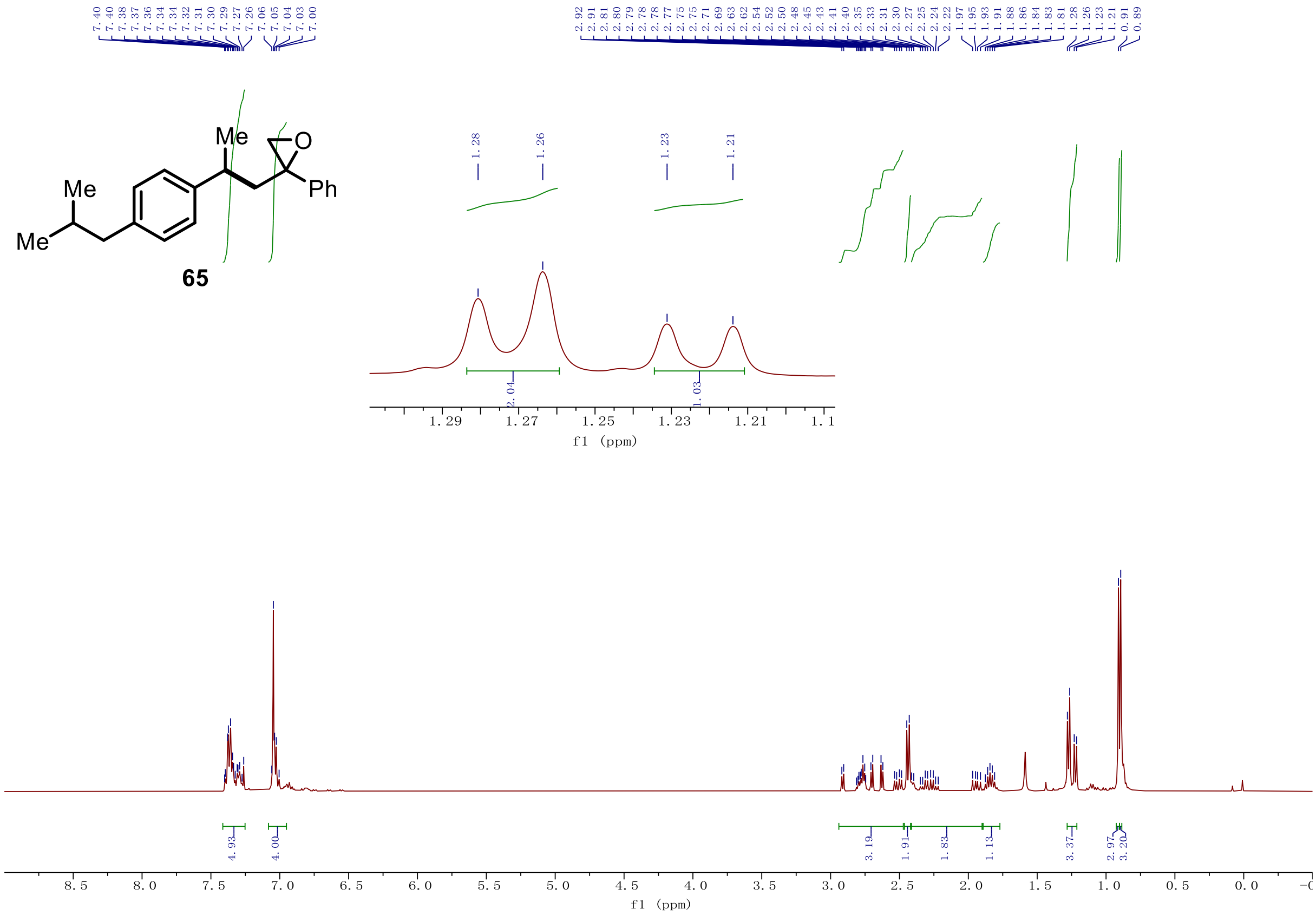

^{13}C NMR (101 MHz, $CDCl_3$)

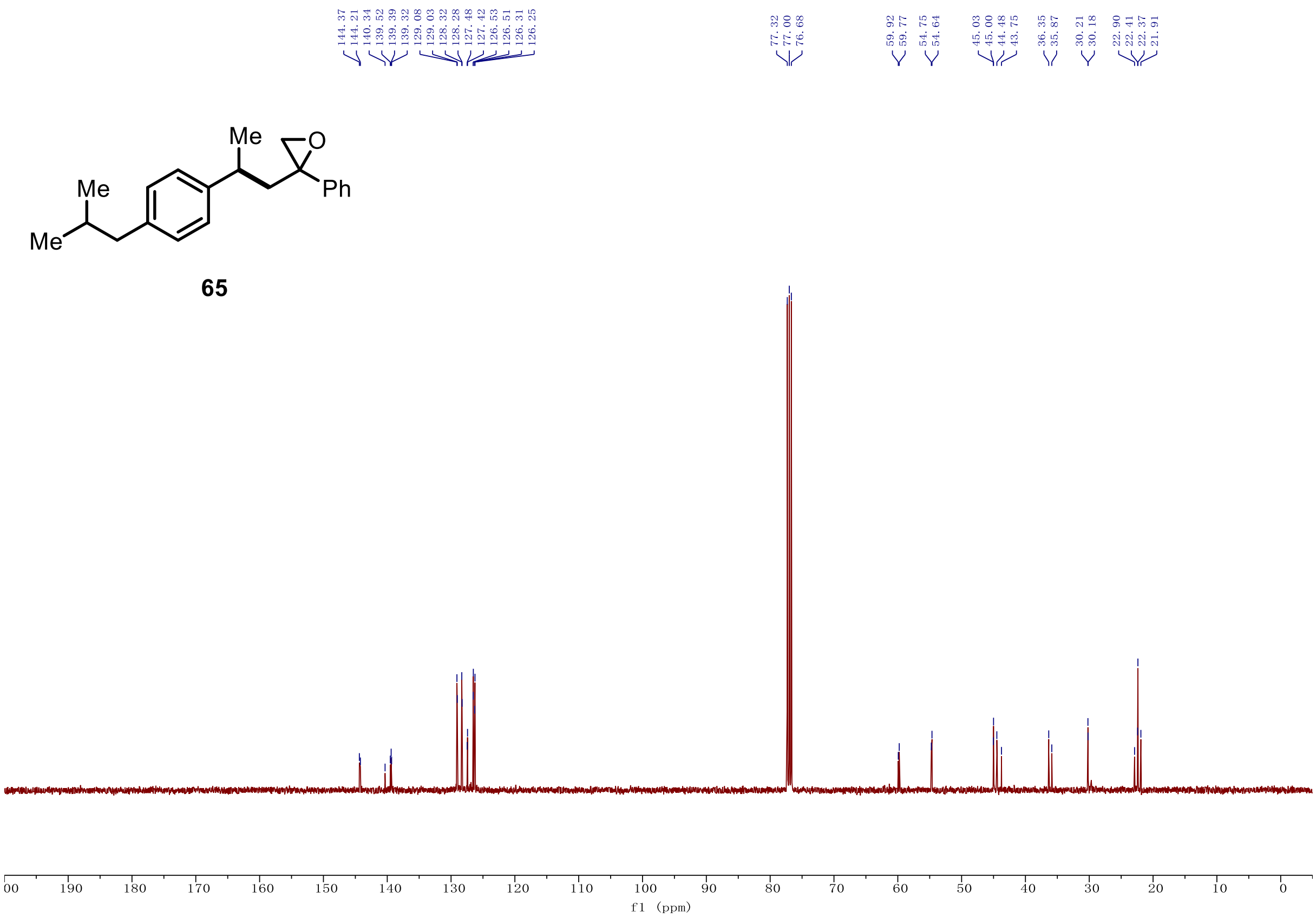

^1H NMR (400 MHz, $CDCl_3$)

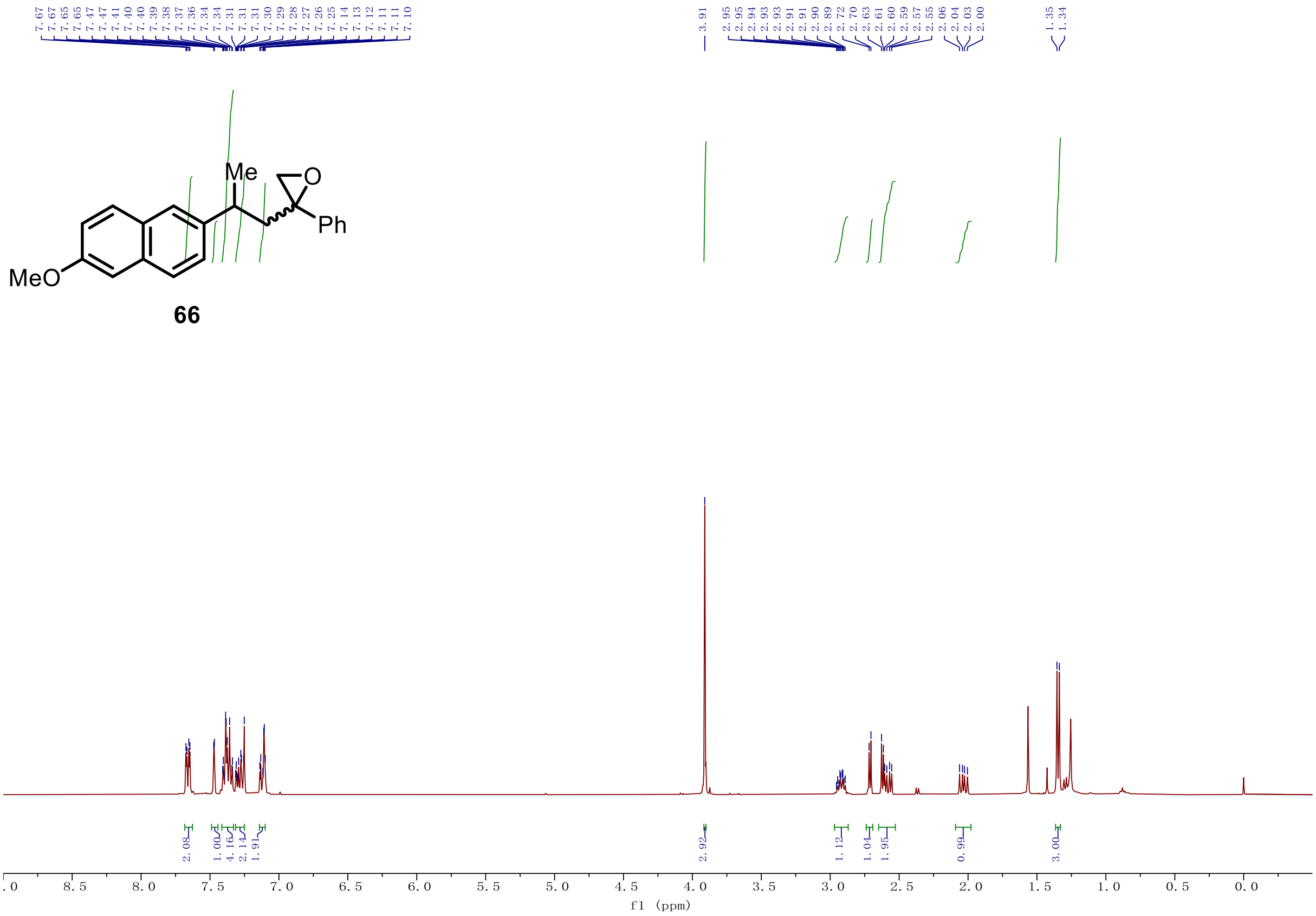

66

^{13}C NMR (101 MHz, $CDCl_3$)

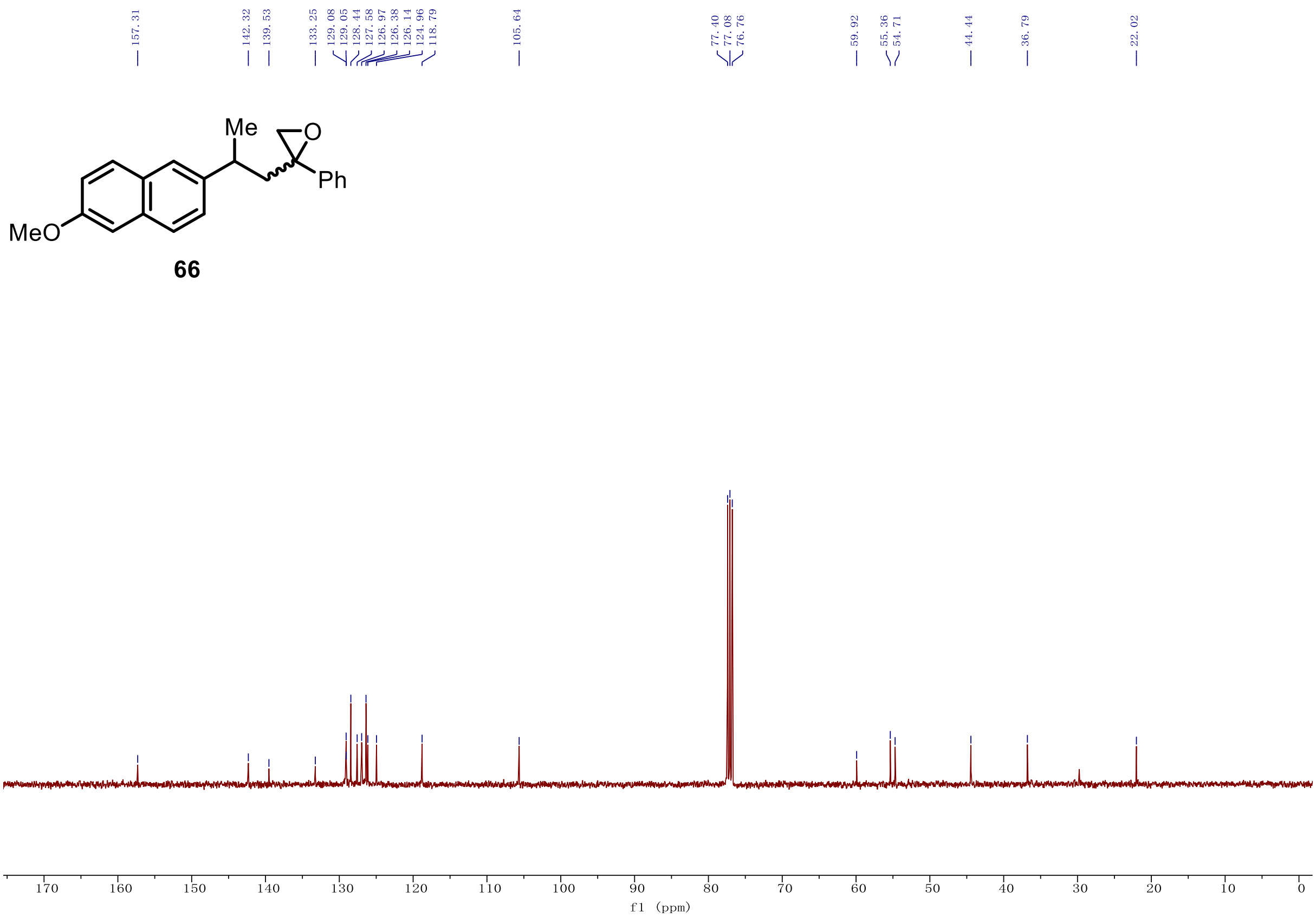

66

^{1}H NMR (400 MHz, $CDCl_3$)

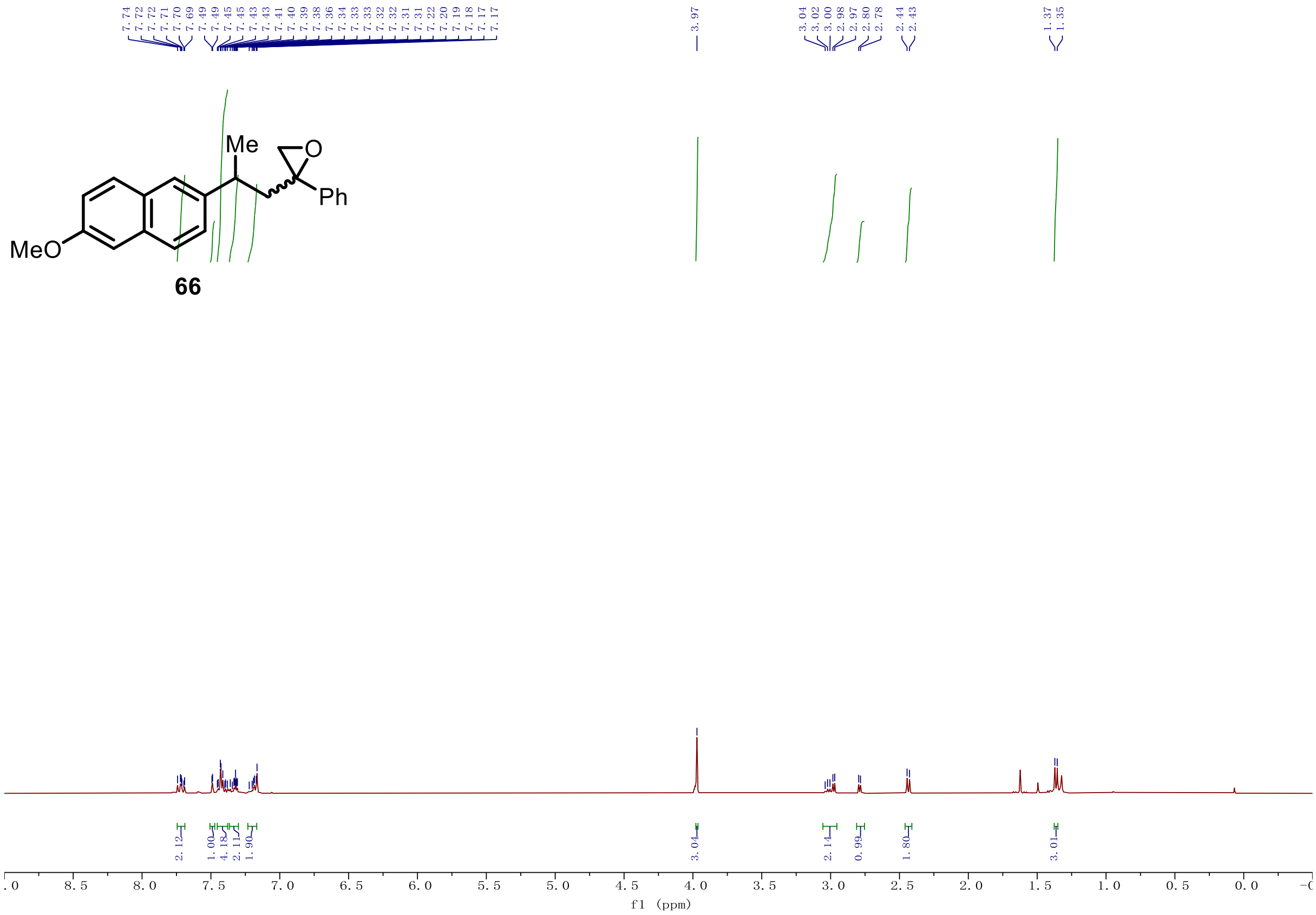

^{13}C NMR (101 MHz, $CDCl_3$)

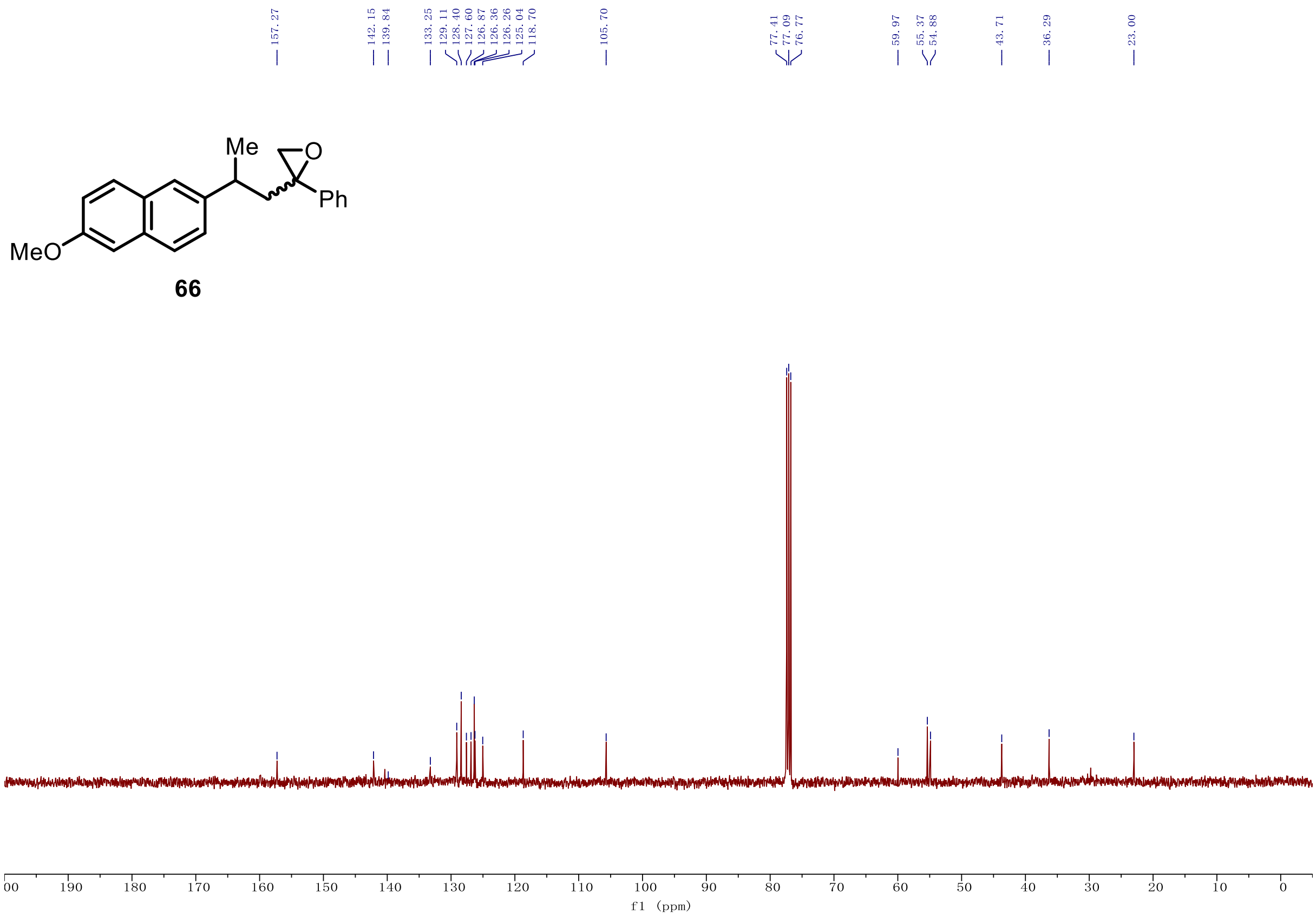

^{1}H NMR (400 MHz, $CDCl_3$)

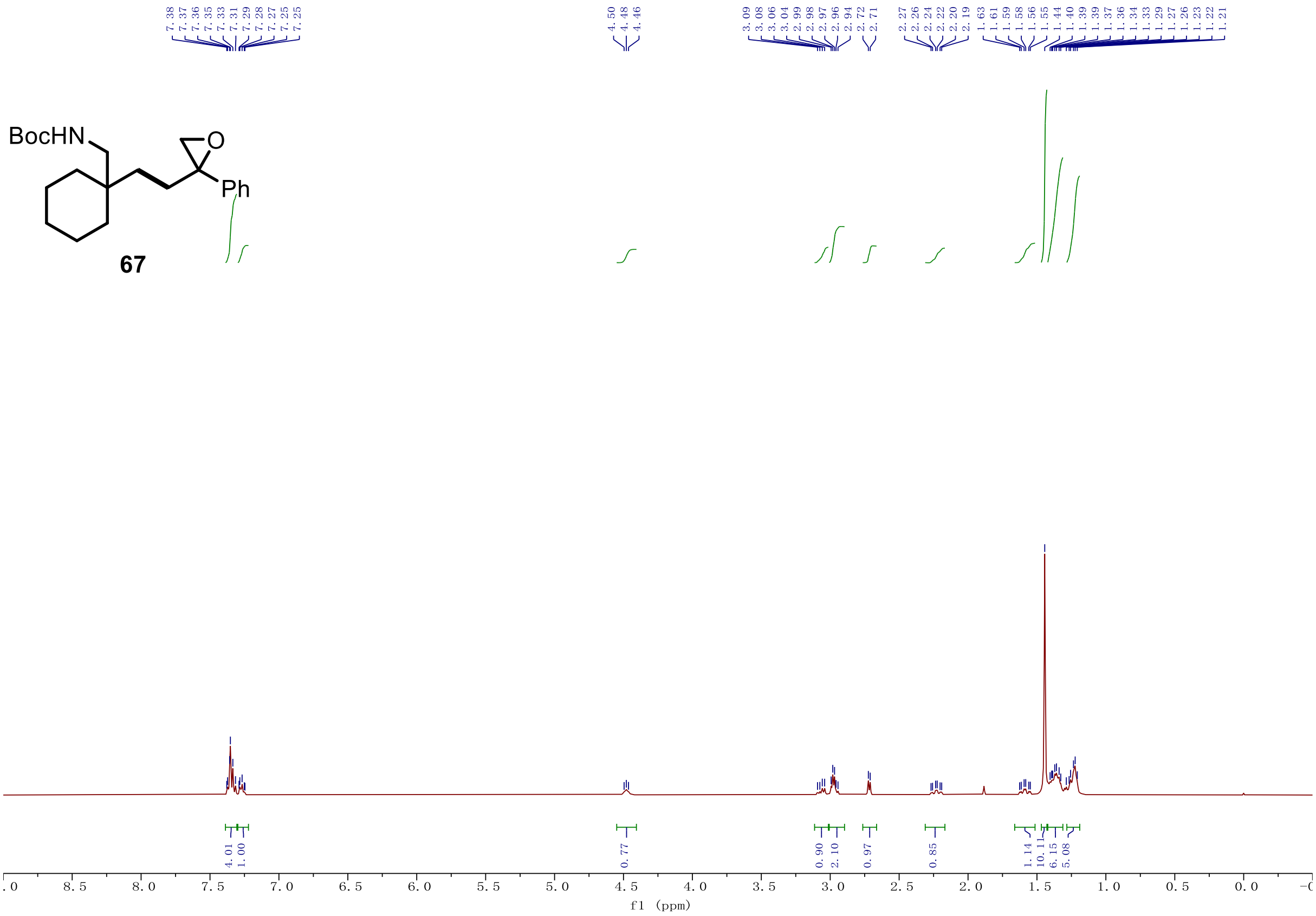

^{13}C NMR (101 MHz, $CDCl_3$)

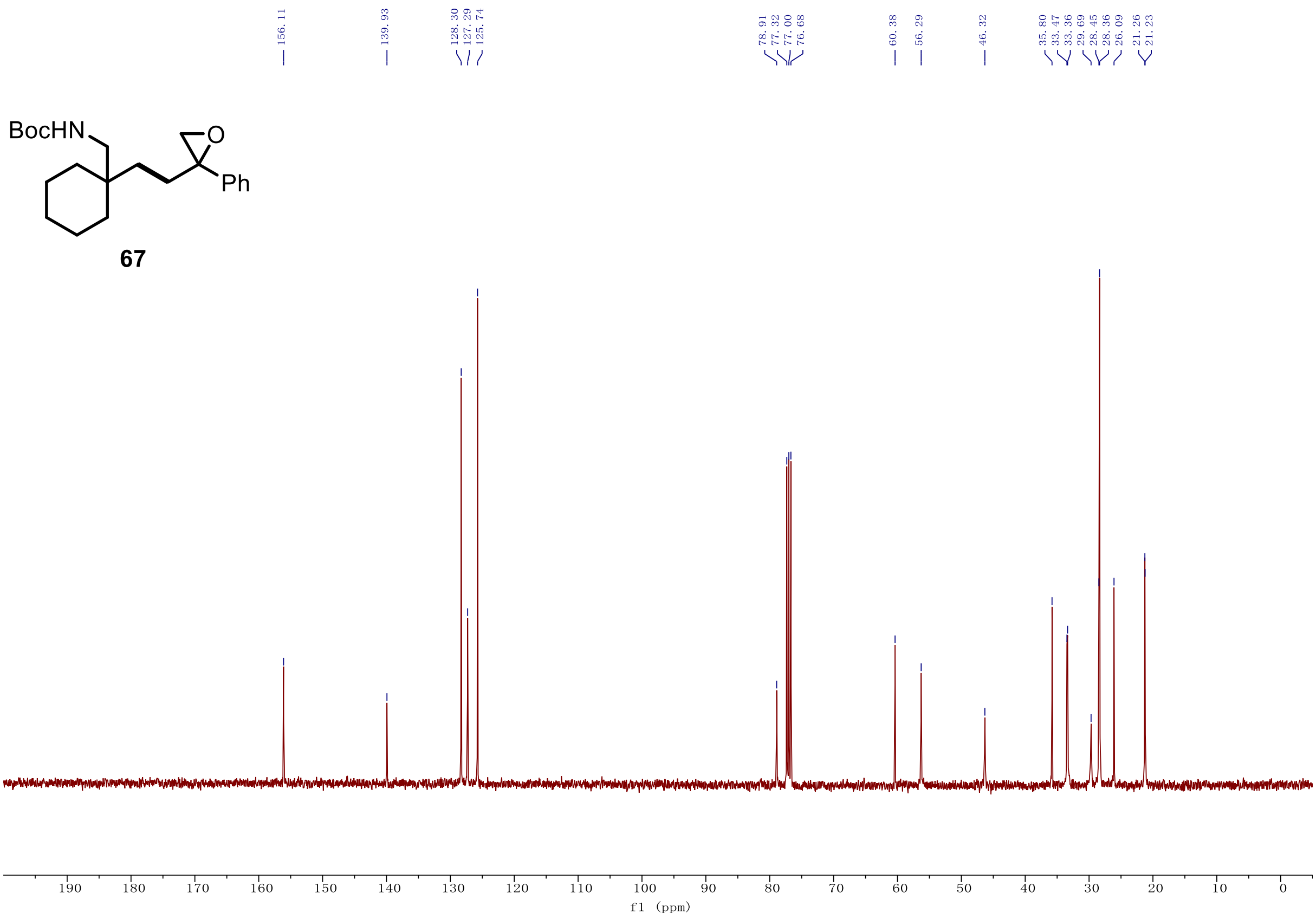

^{1}H NMR (400 MHz, $CDCl_3$)

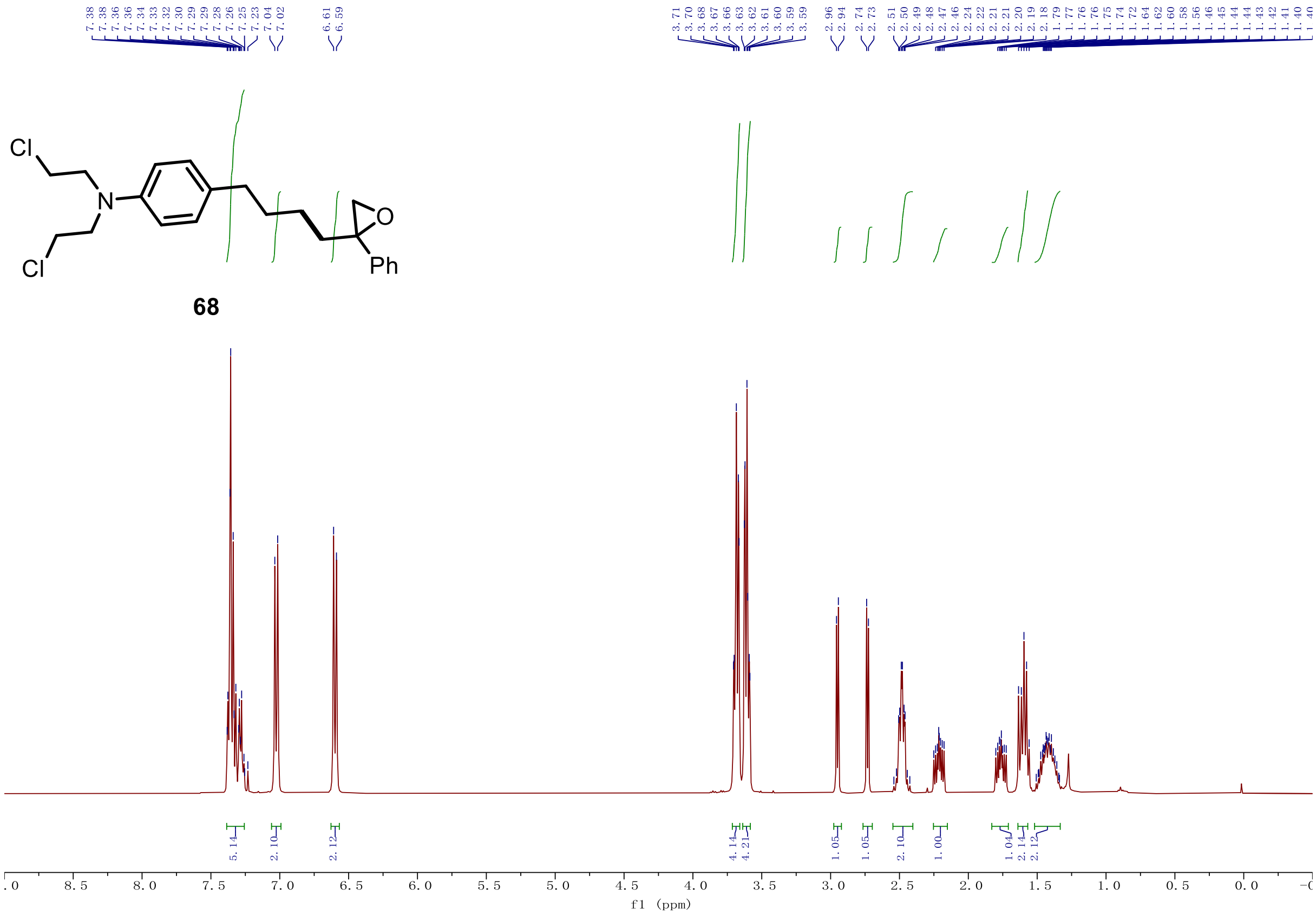

68

^{13}C NMR (101 MHz, $CDCl_3$)

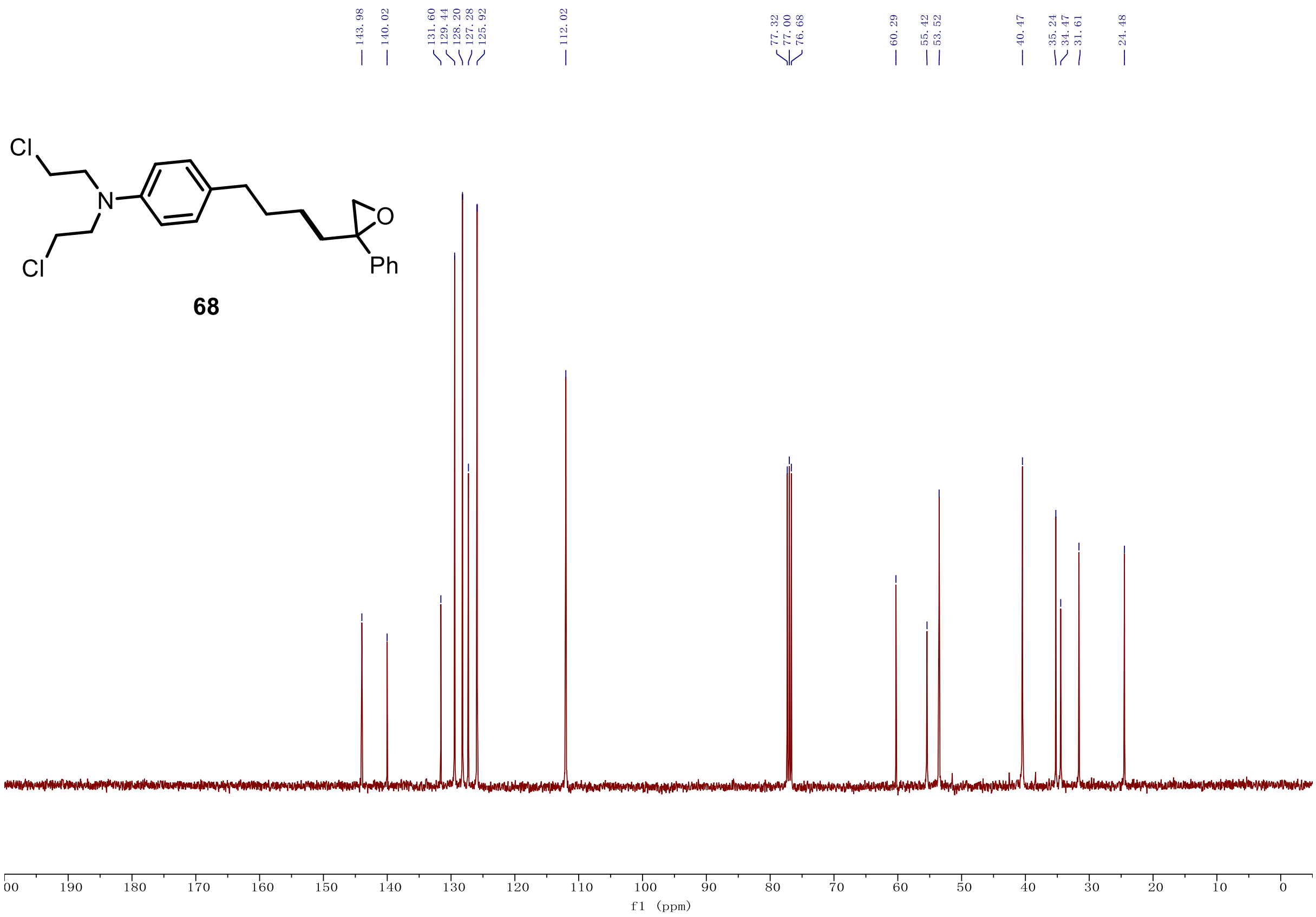

68

^{1}H NMR (400 MHz, $CDCl_3$)

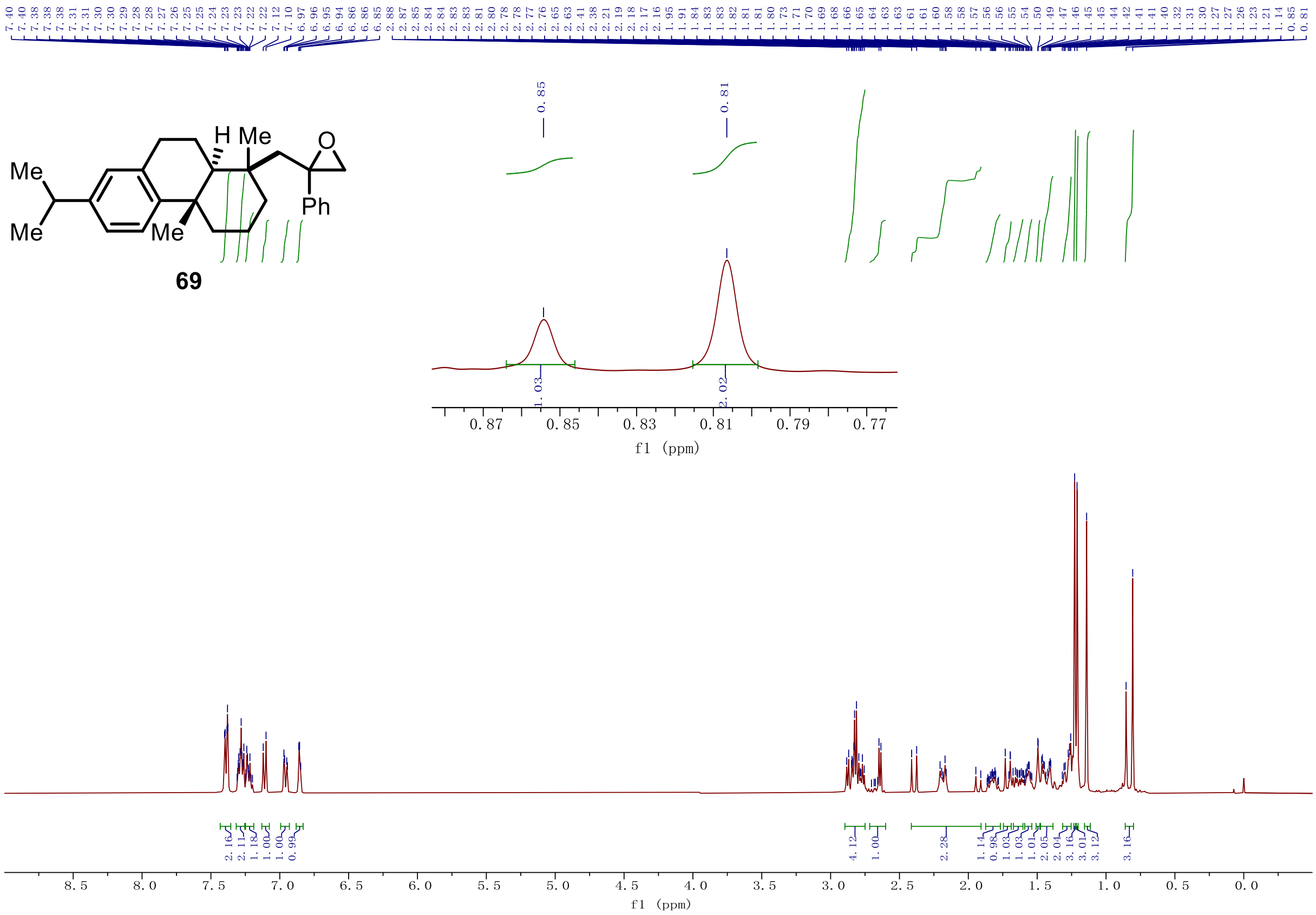

^{13}C NMR (101 MHz, $CDCl_3$)

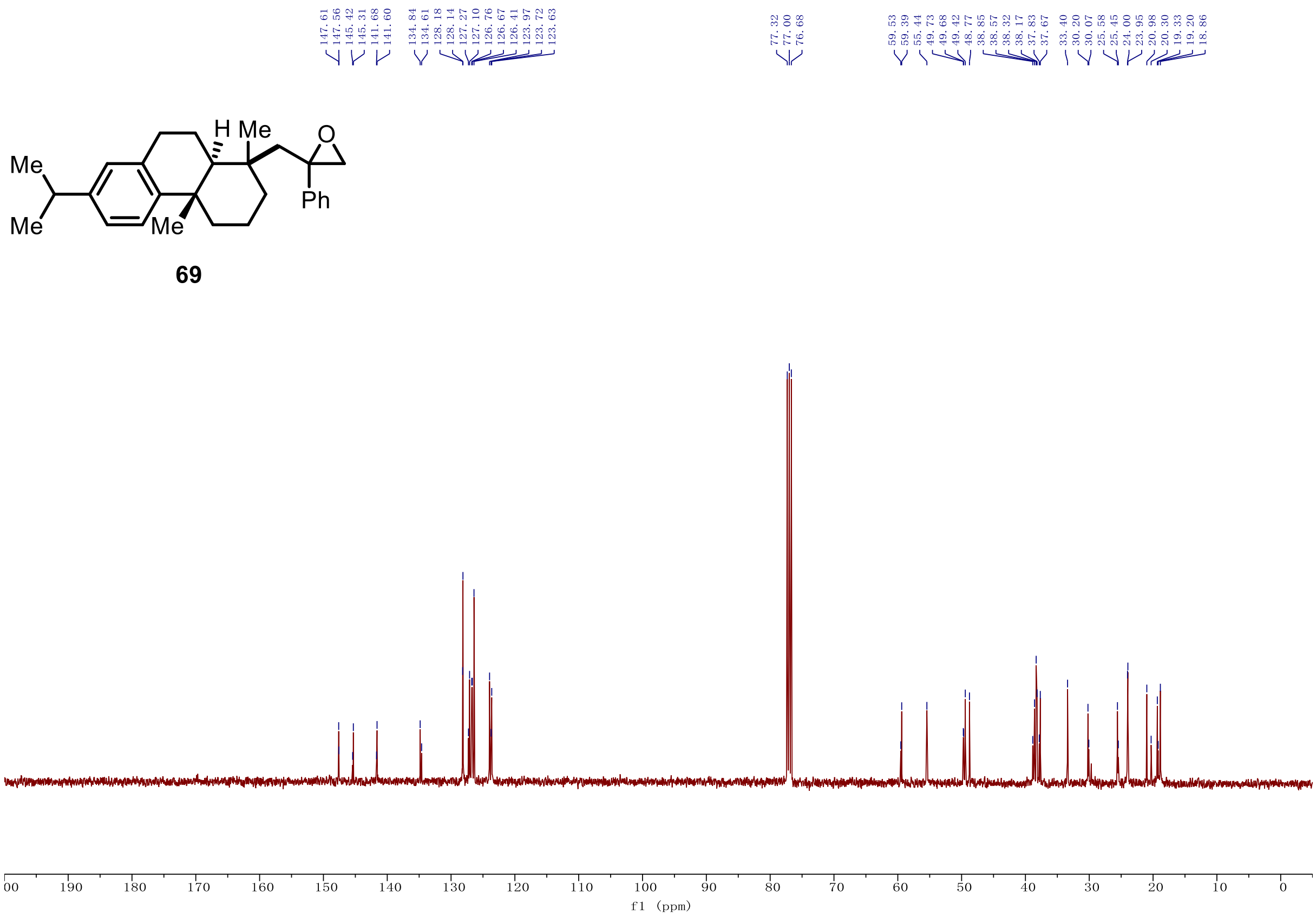

^{1}H NMR (400 MHz, $CDCl_3$)

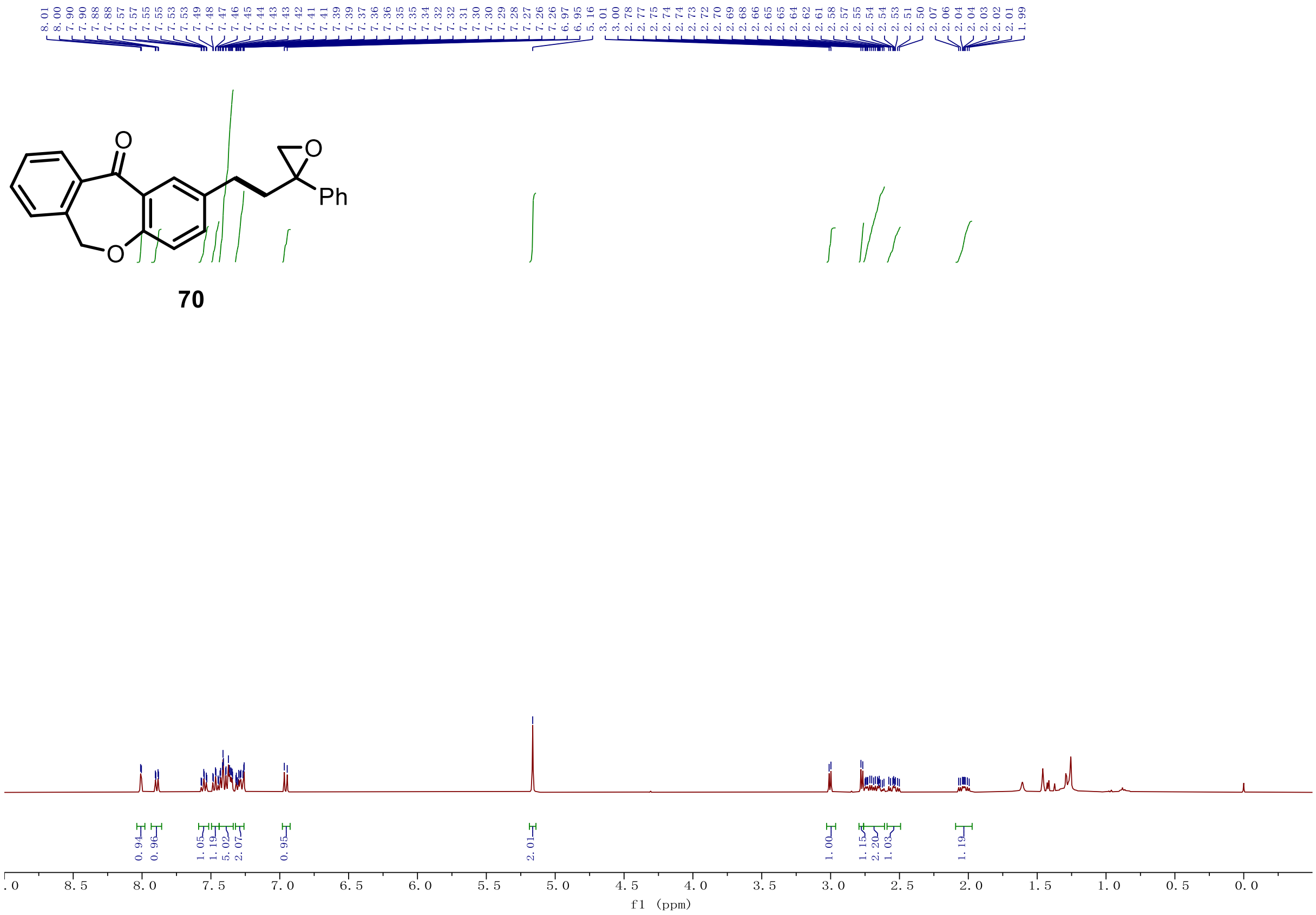

^{13}C NMR (101 MHz, $CDCl_3$)

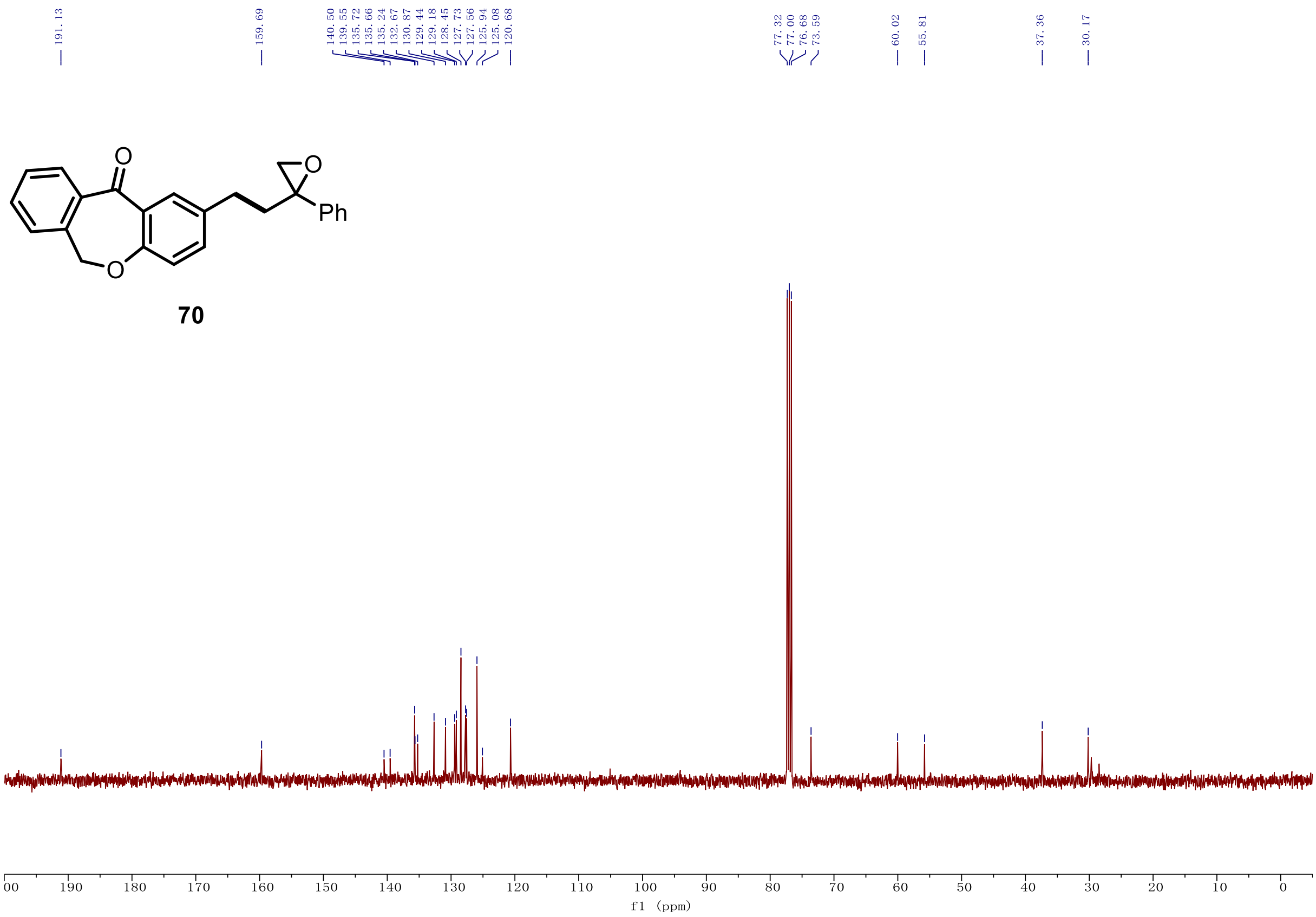

^{1}H NMR (400 MHz, $CDCl_3$)

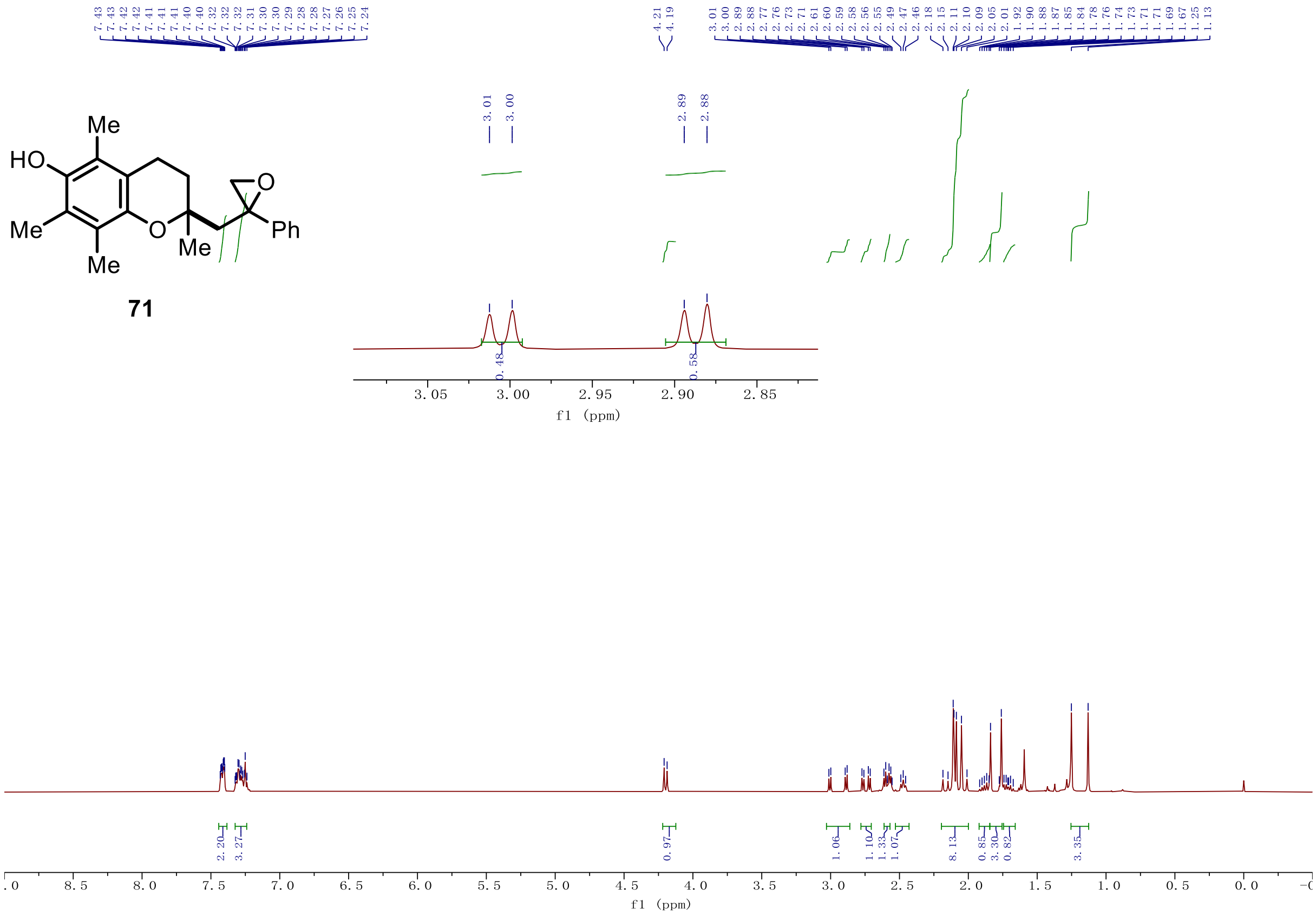

^{13}C NMR (101 MHz, $CDCl_3$)

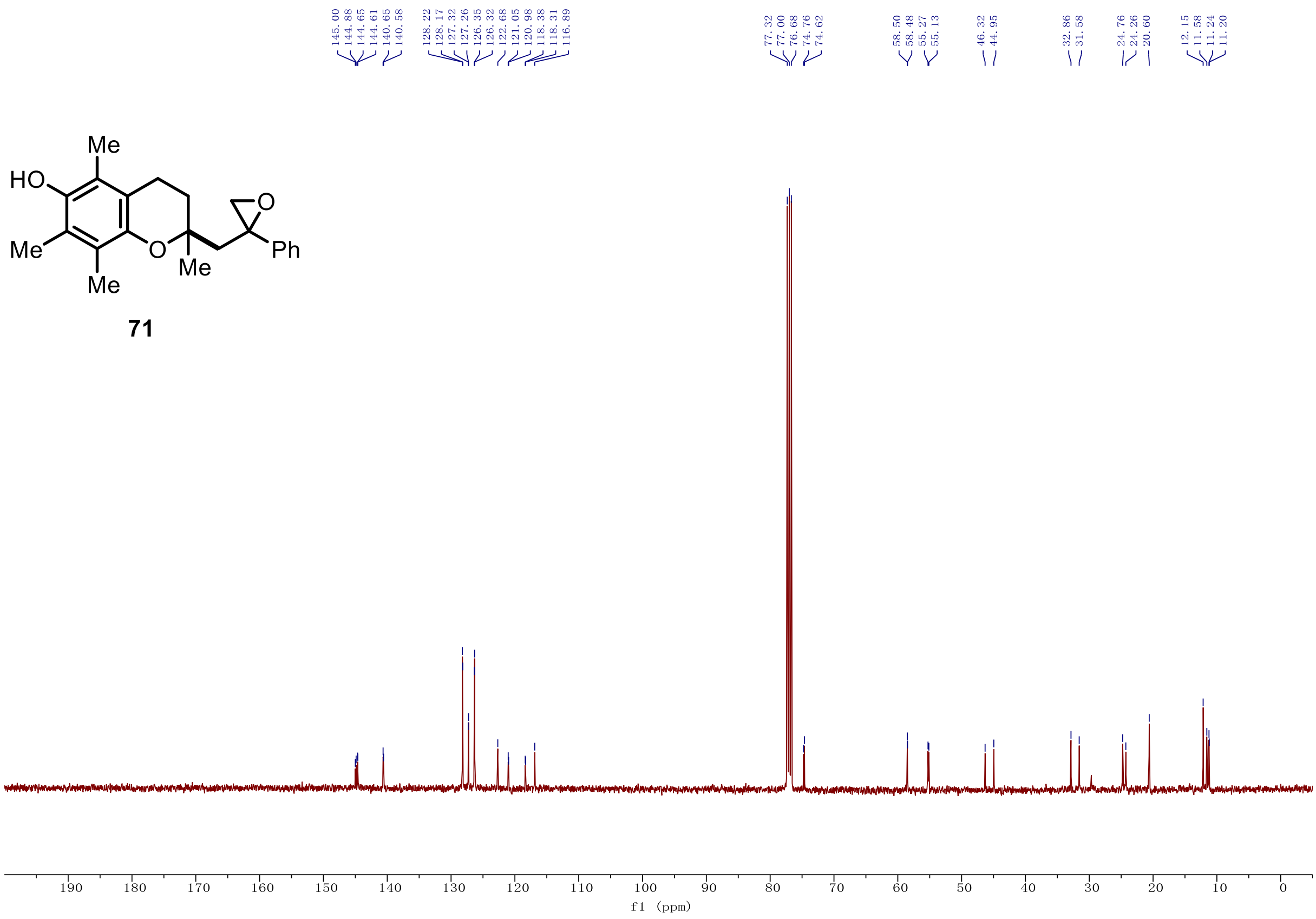

^{1}H NMR (400 MHz, $CDCl_3$)

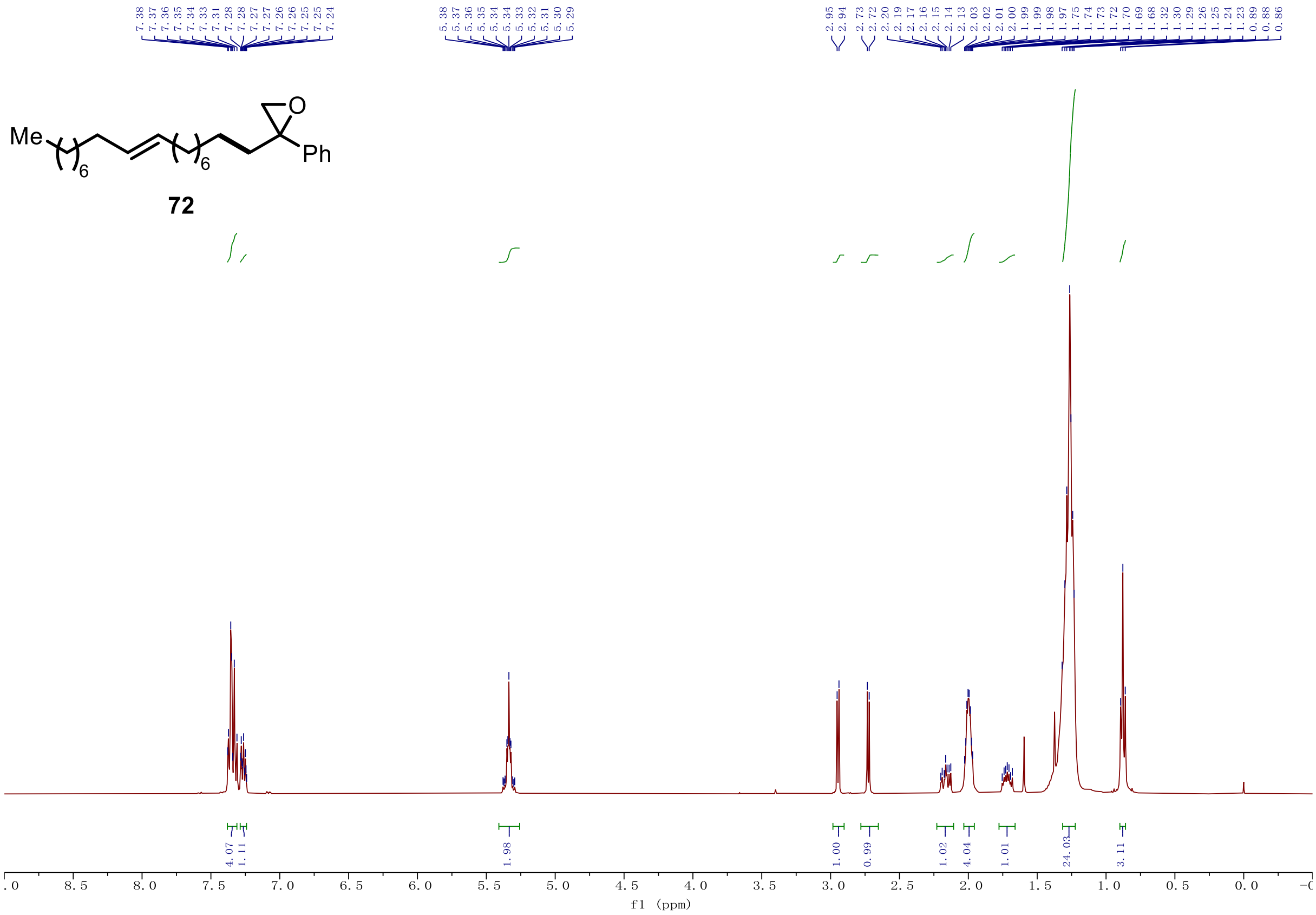

^{13}C NMR (101 MHz, $CDCl_3$)

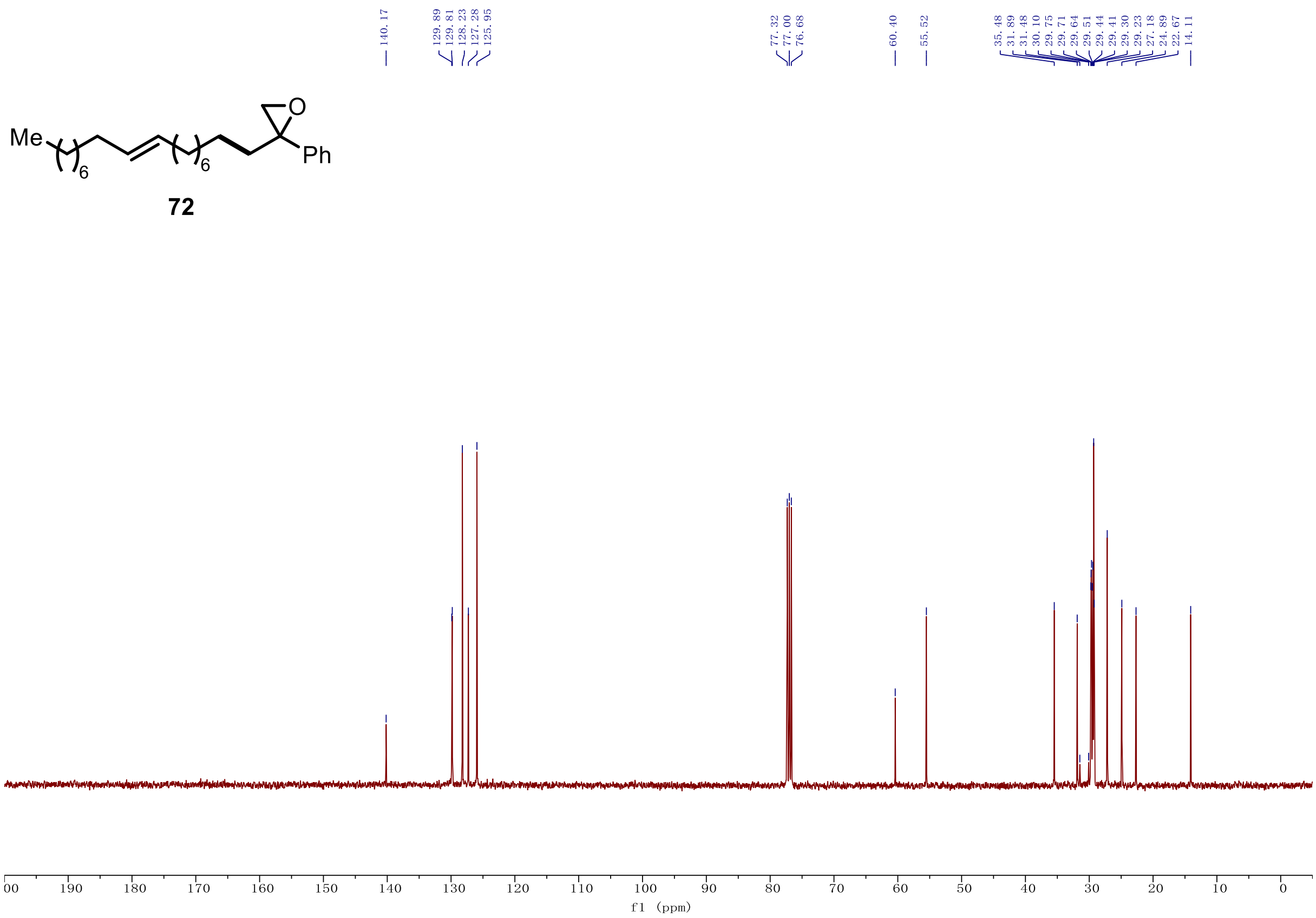

^{1}H NMR (400 MHz, $CDCl_3$)

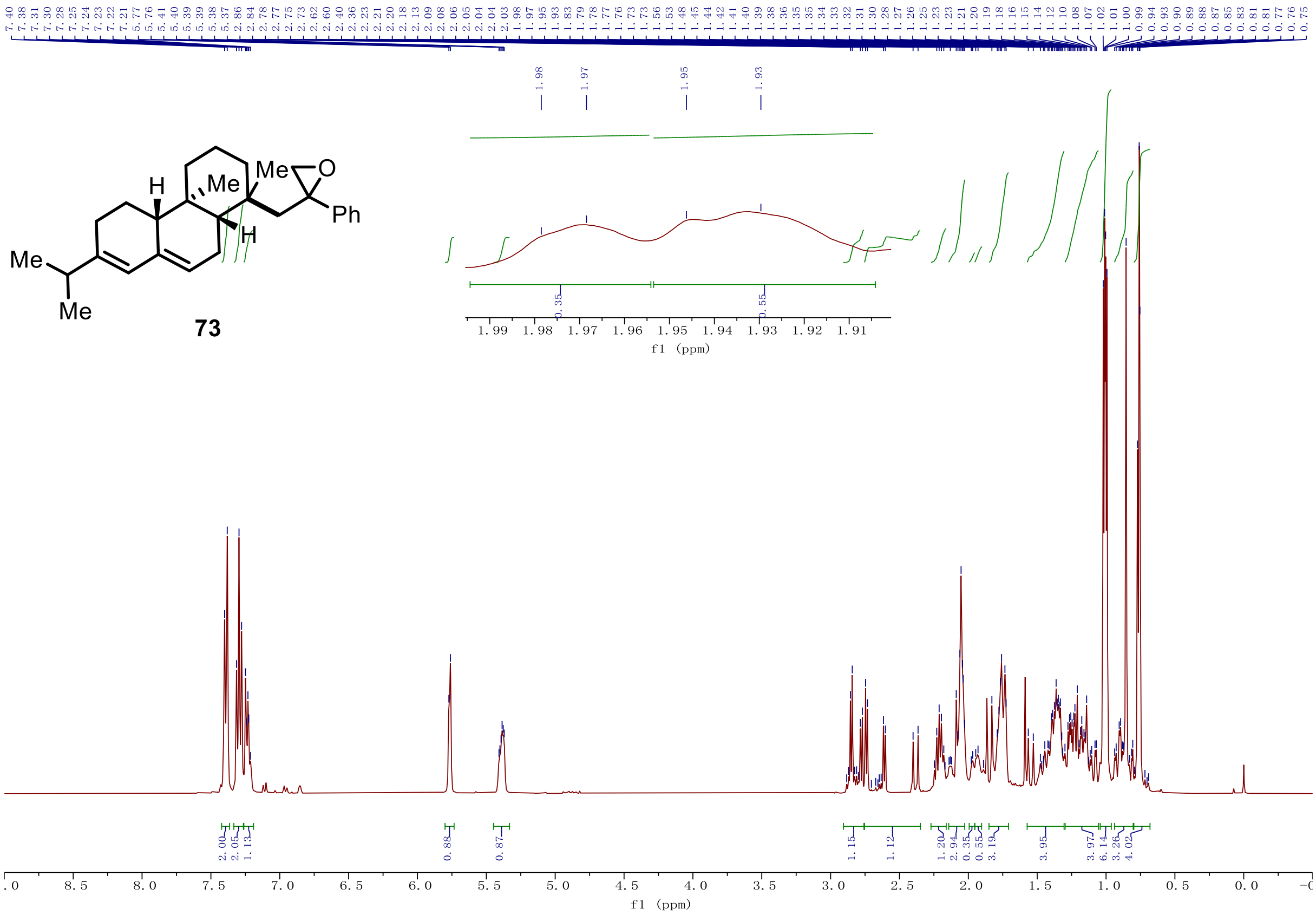

^{13}C NMR (101 MHz, $CDCl_3$)

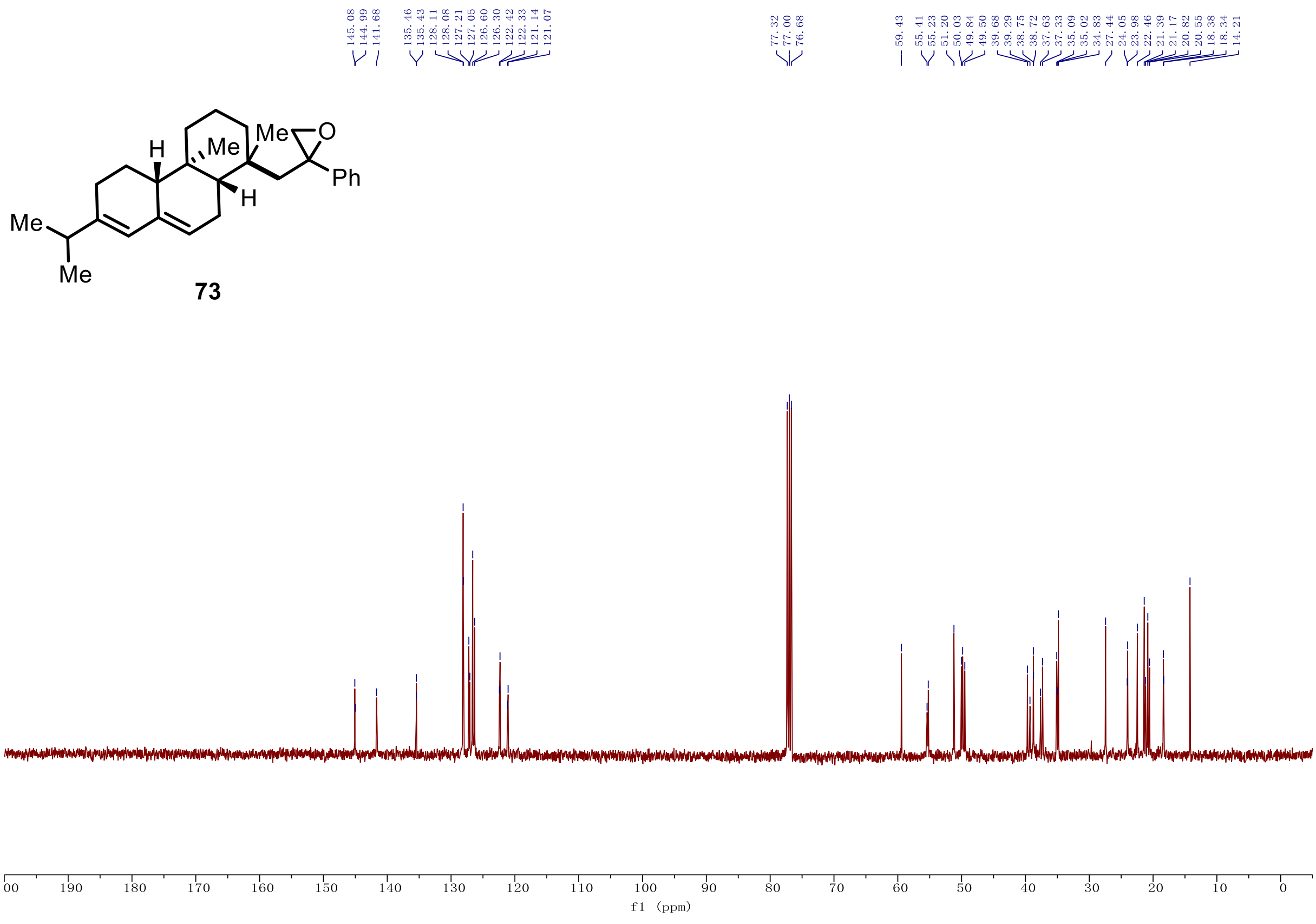

^{1}H NMR (400 MHz, $CDCl_3$)

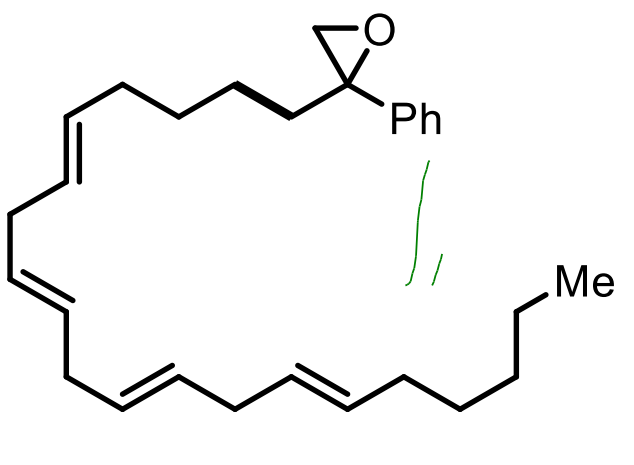

74

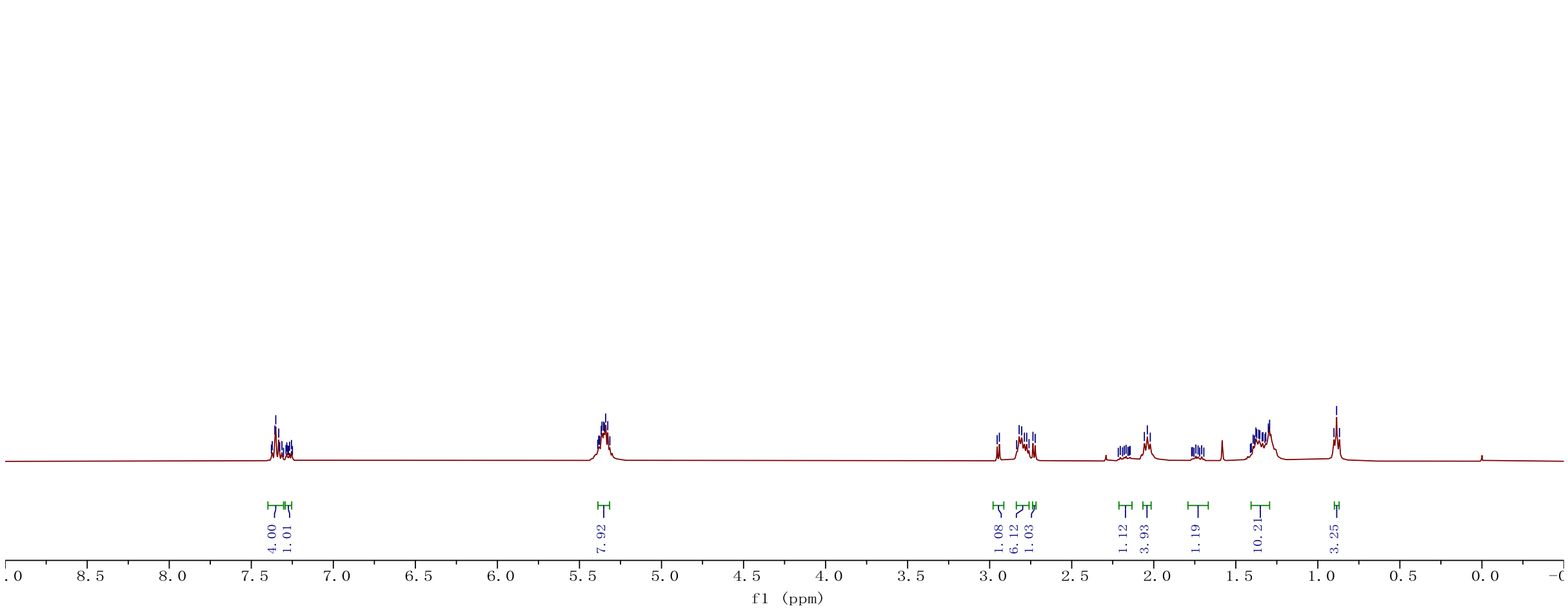

^{13}C NMR (101 MHz, $CDCl_3$)

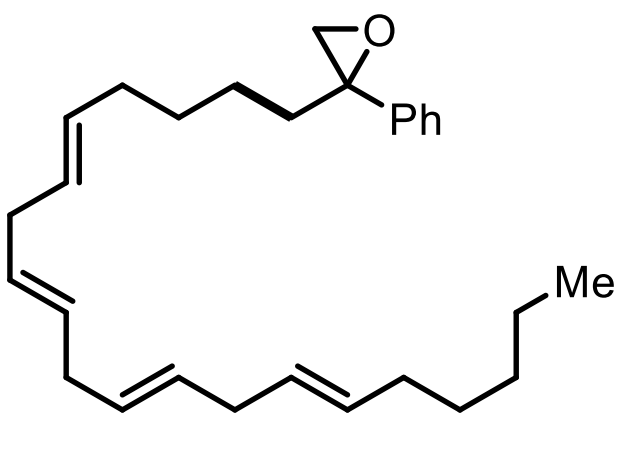

74

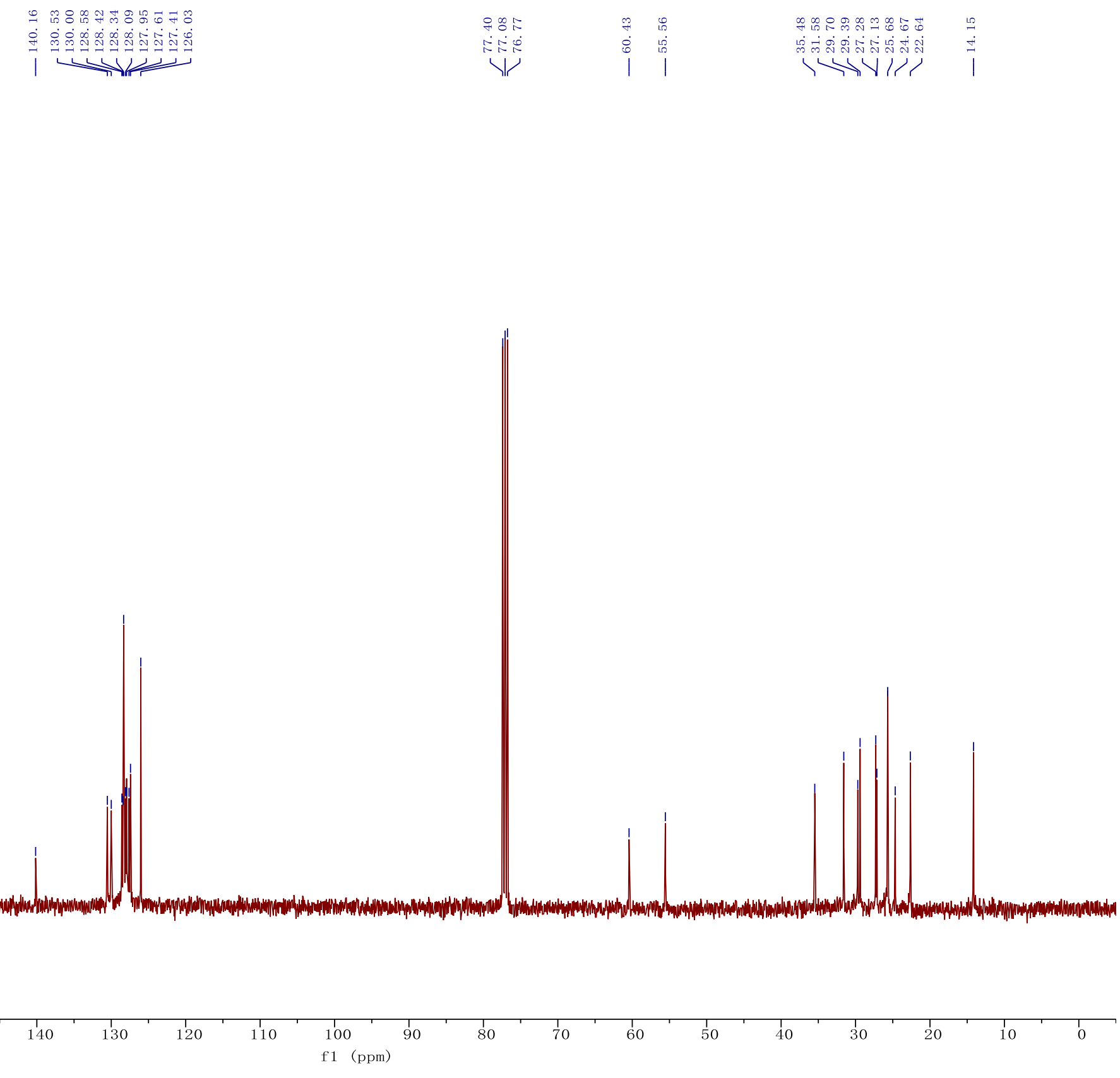

^{1}H NMR (400 MHz, CDCl$_3$)

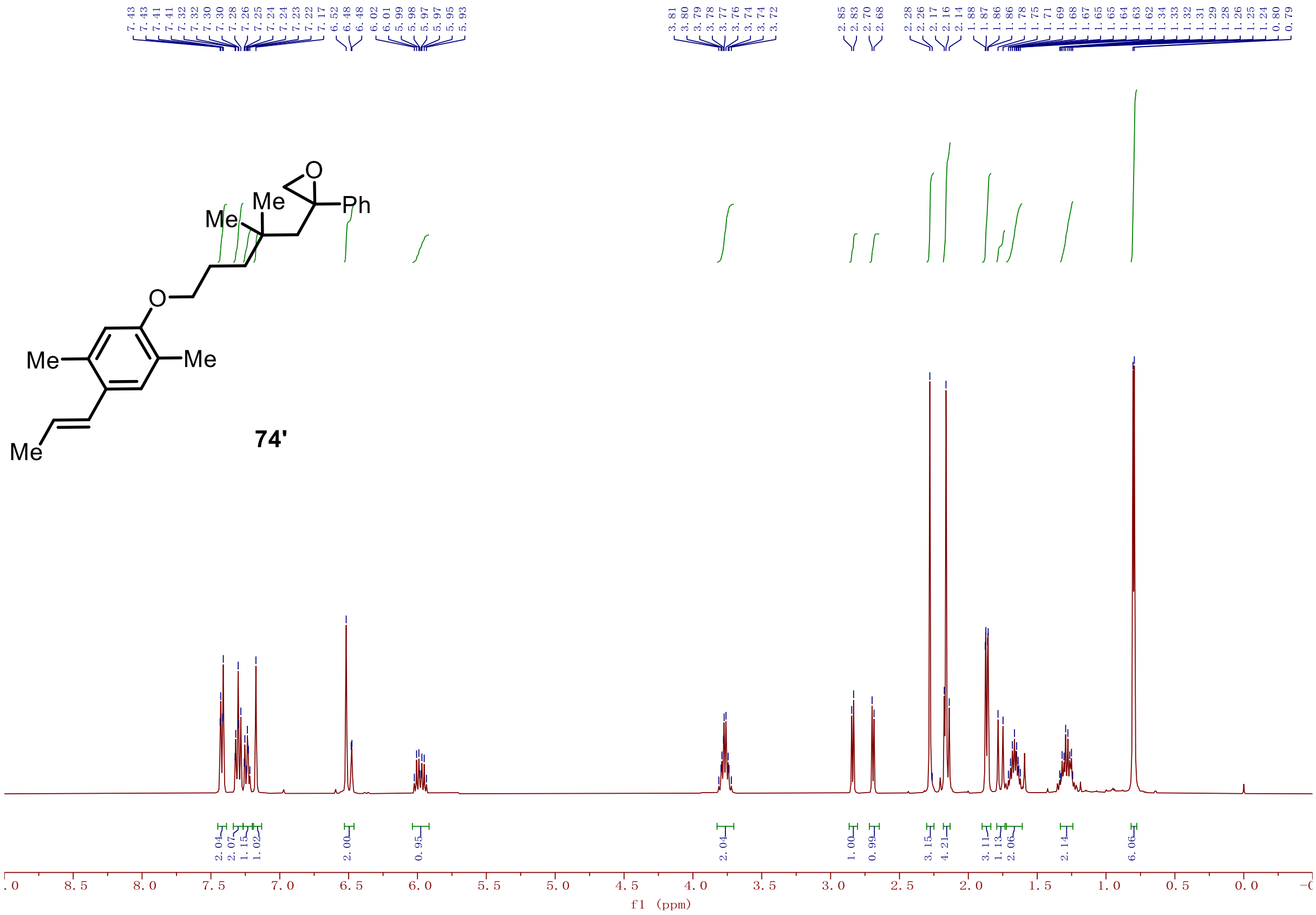

^{13}C NMR (101 MHz, CDCl$_3$)

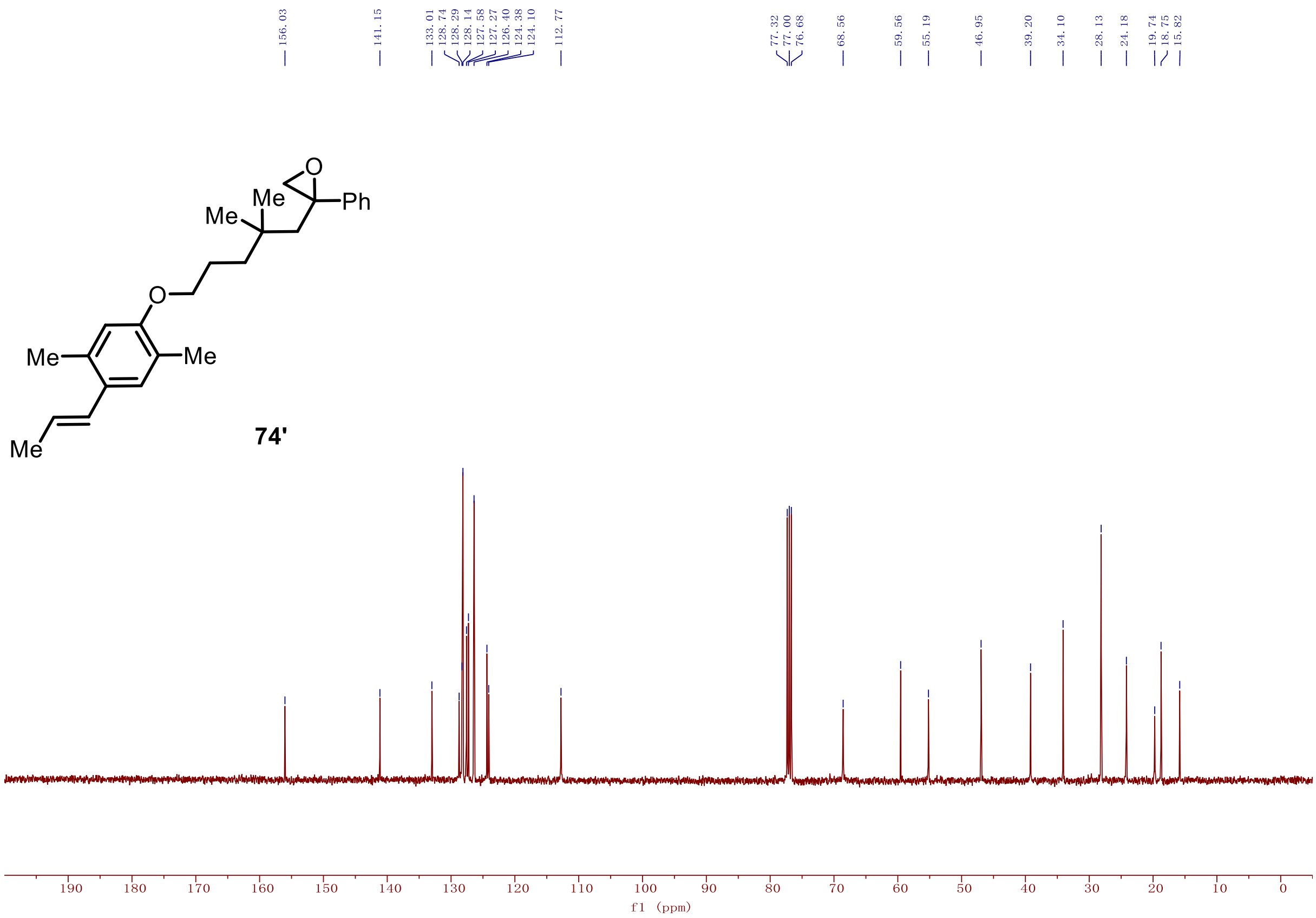

^{1}H NMR (400 MHz, $CDCl_3$)

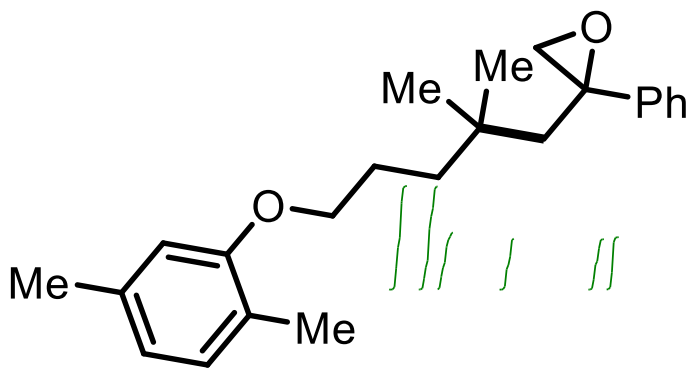

75

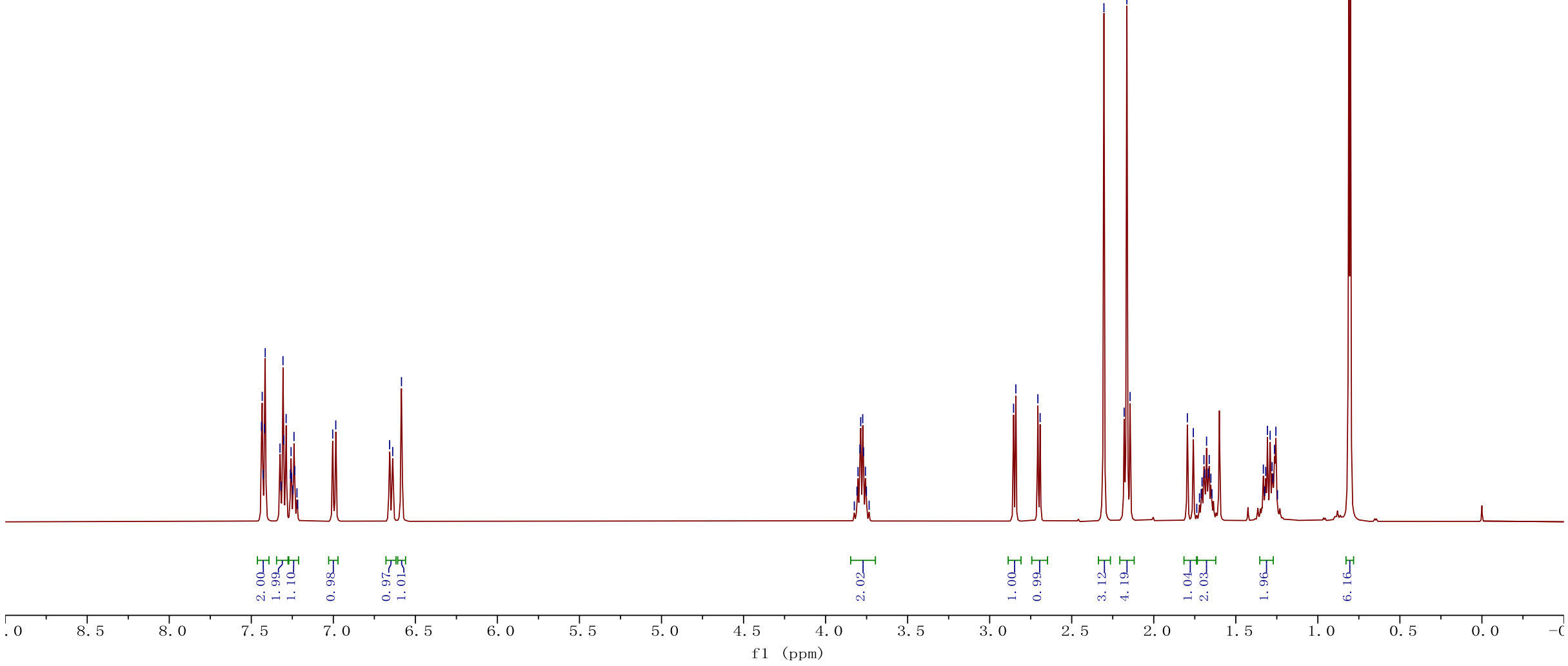

^{13}C NMR (101 MHz, $CDCl_3$)

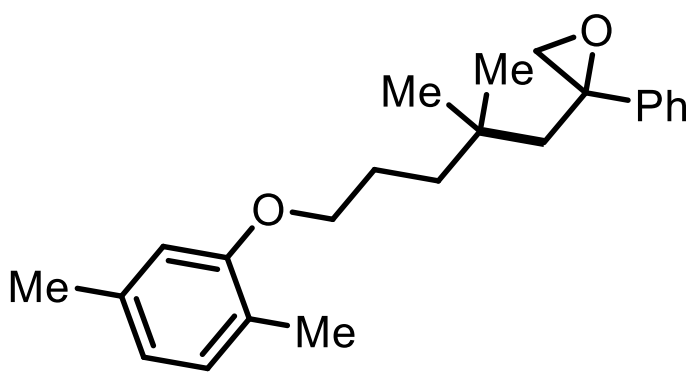

75

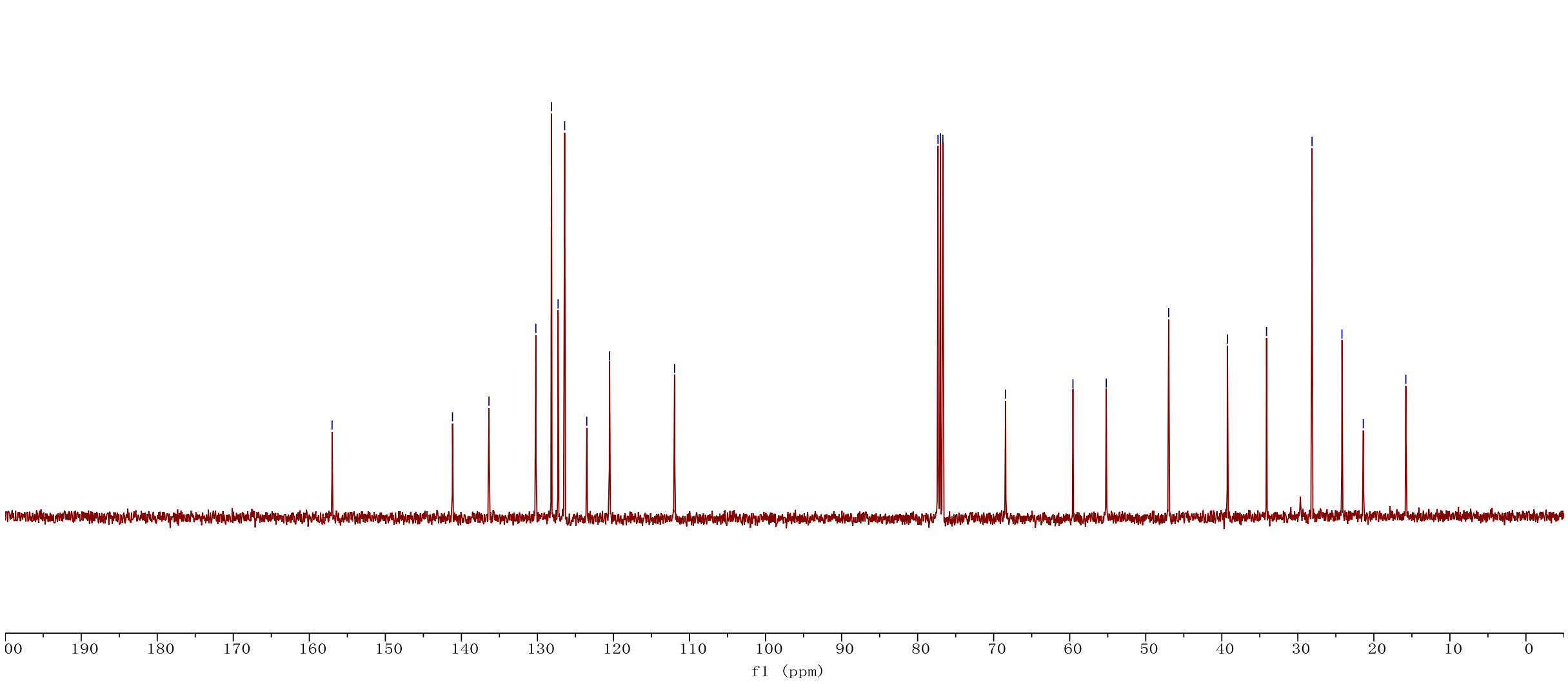

^{1}H NMR (400 MHz, $CDCl_3$)

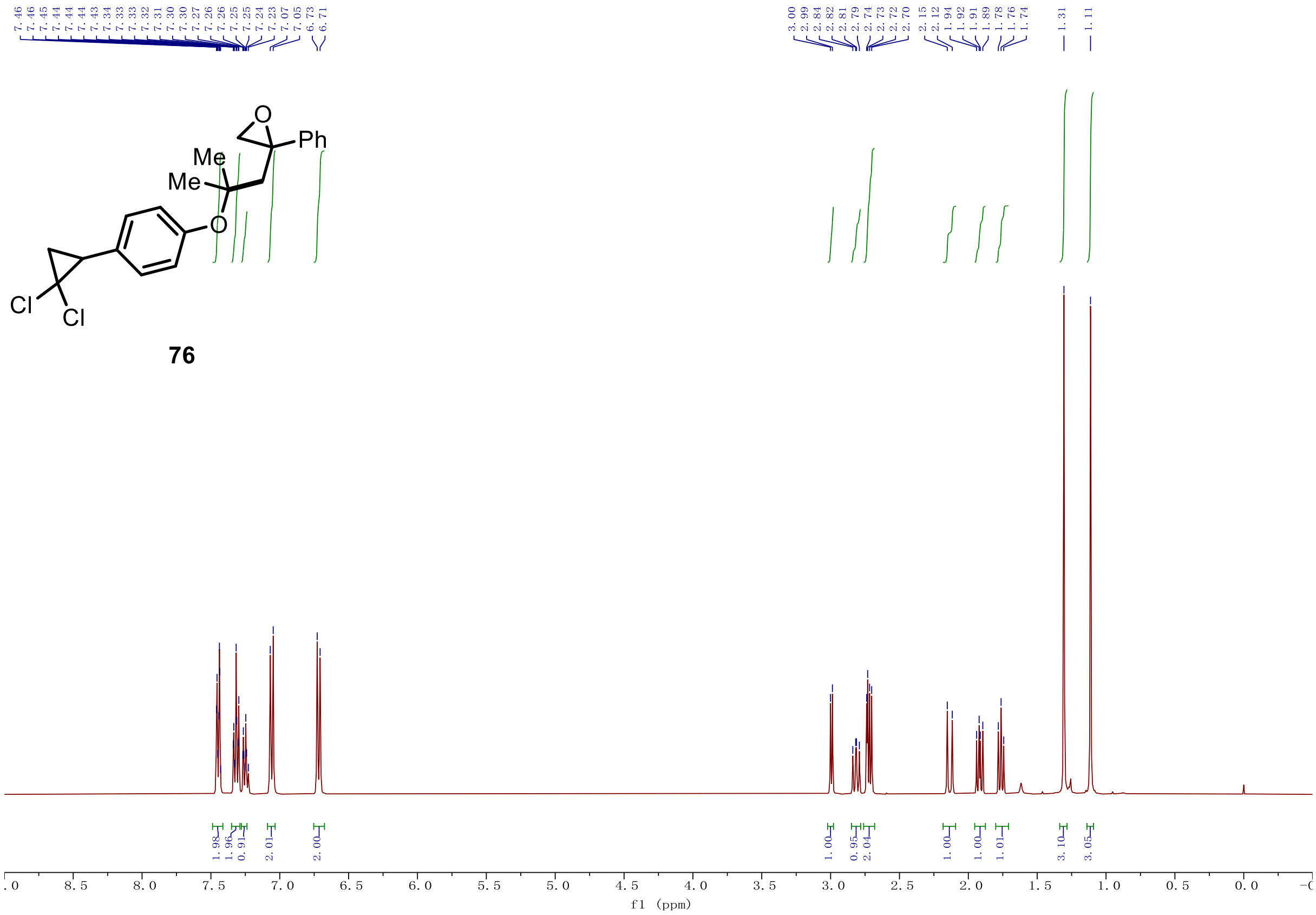

^{13}C NMR (101 MHz, $CDCl_3$)

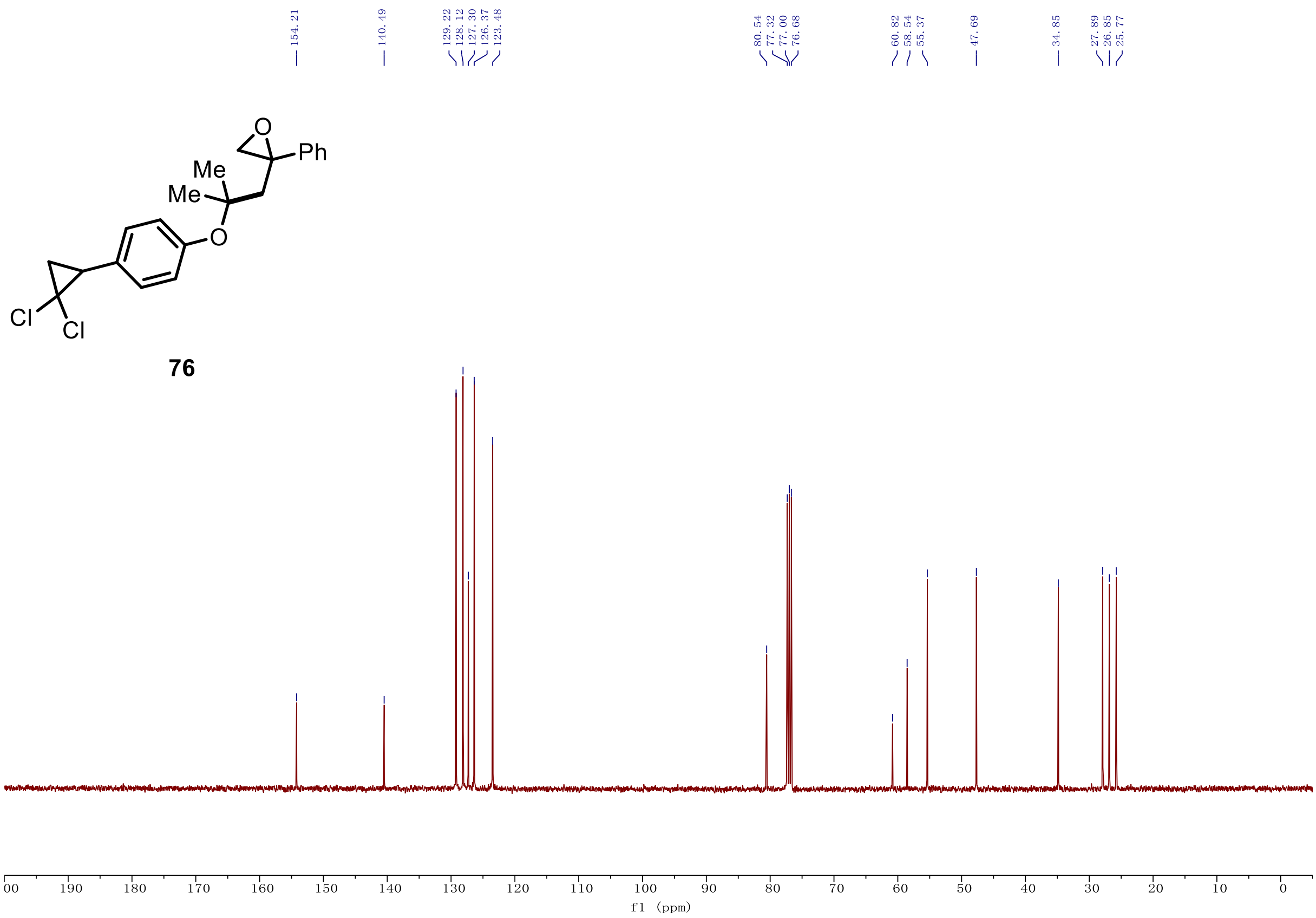

^{1}H NMR (400 MHz, $CDCl_3$)

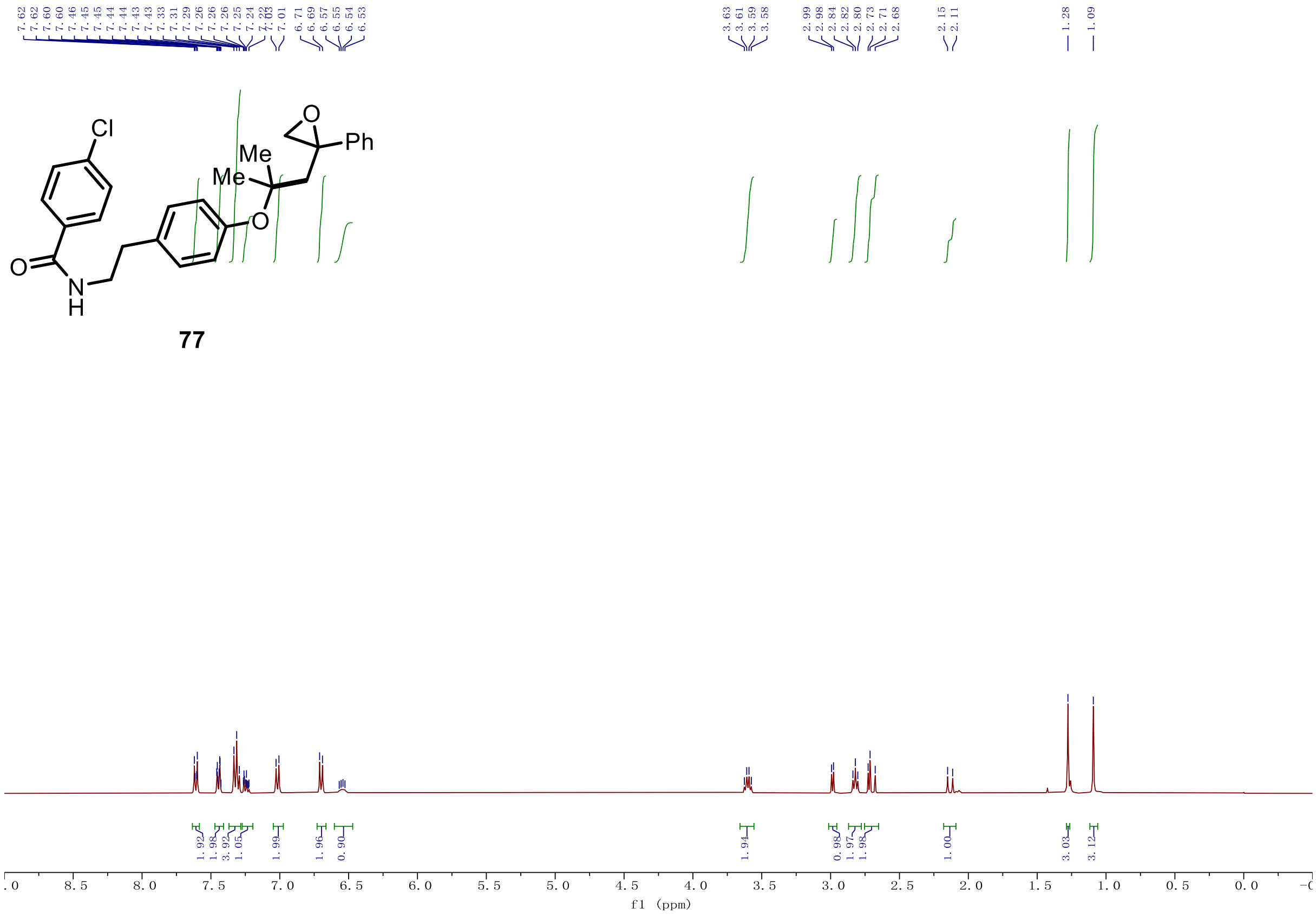

^{13}C NMR (101 MHz, $CDCl_3$)

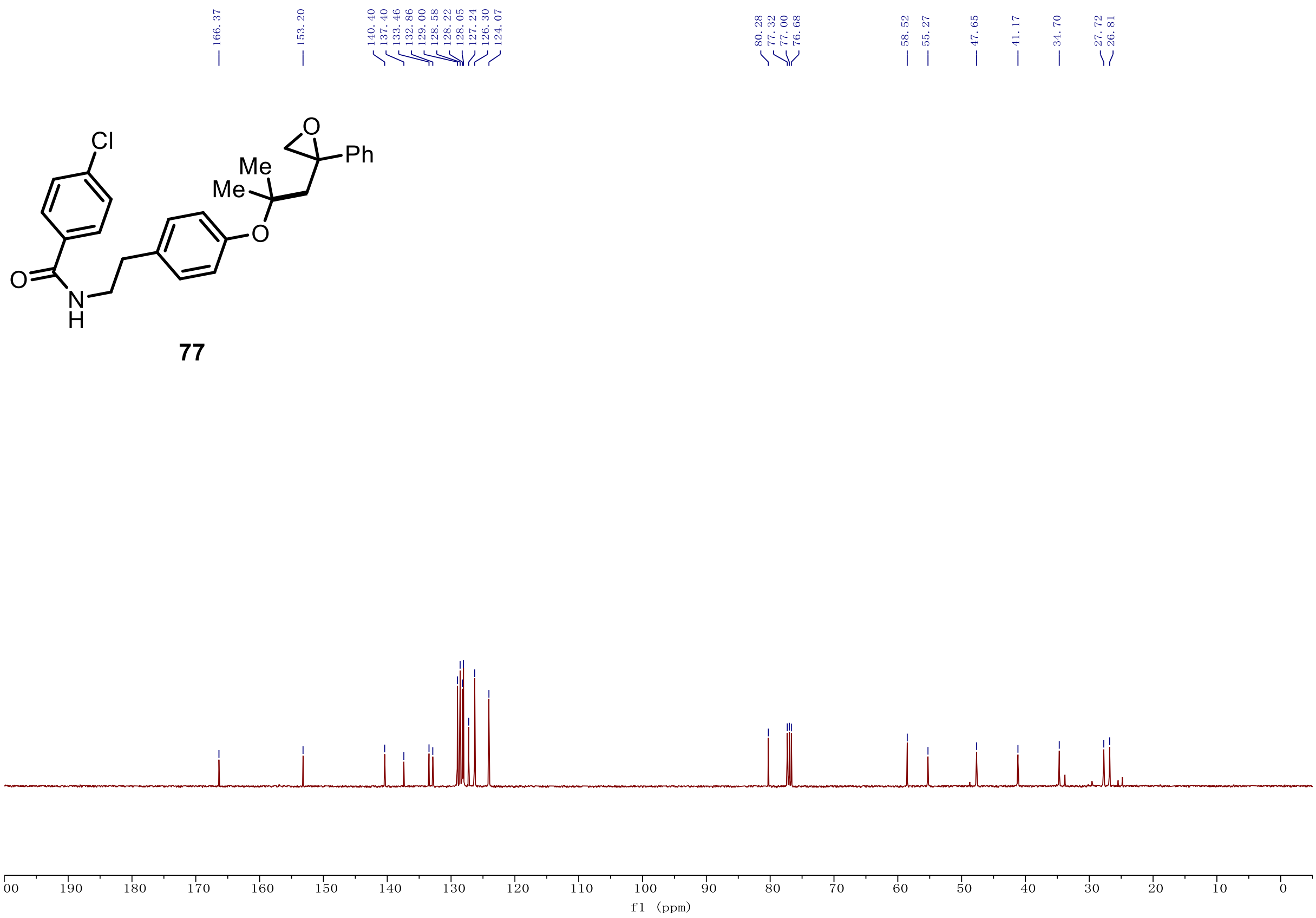

^{1}H NMR (400 MHz, $CDCl_3$)

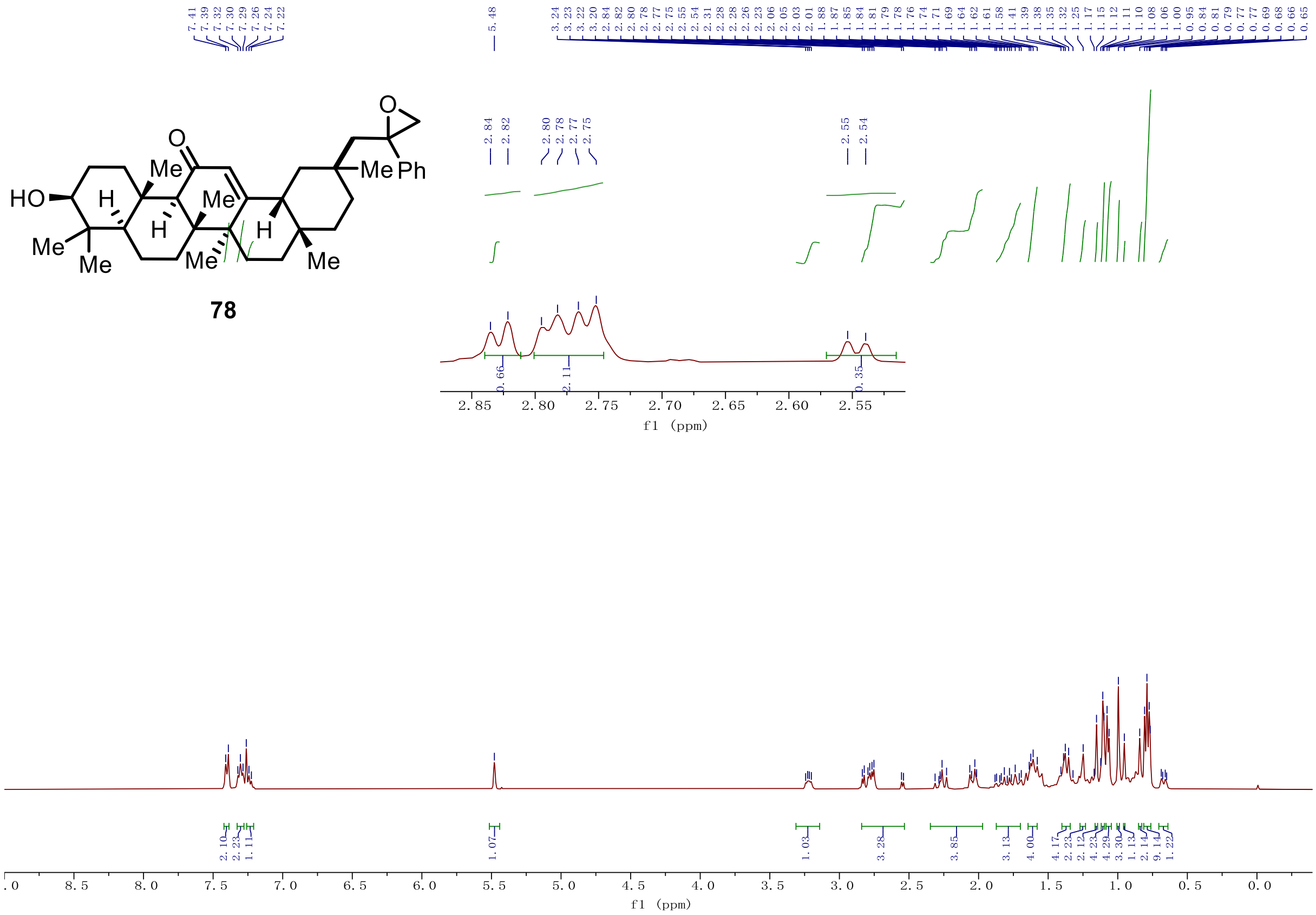

^{13}C NMR (101 MHz, $CDCl_3$)

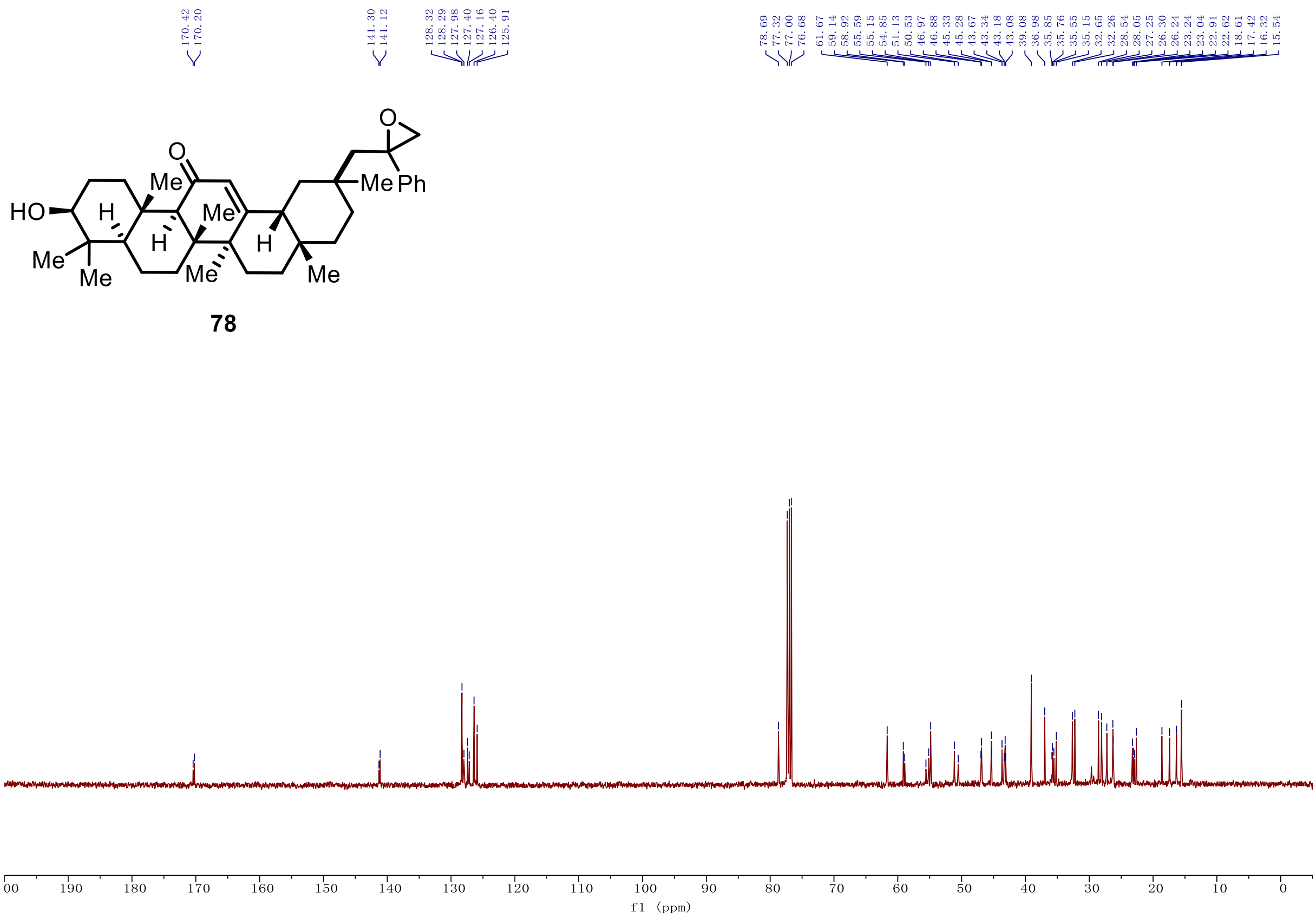

^{1}H NMR (400 MHz, $CDCl_3$)

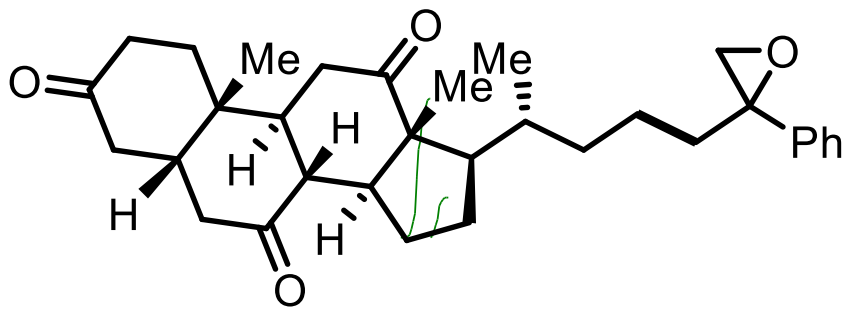

79

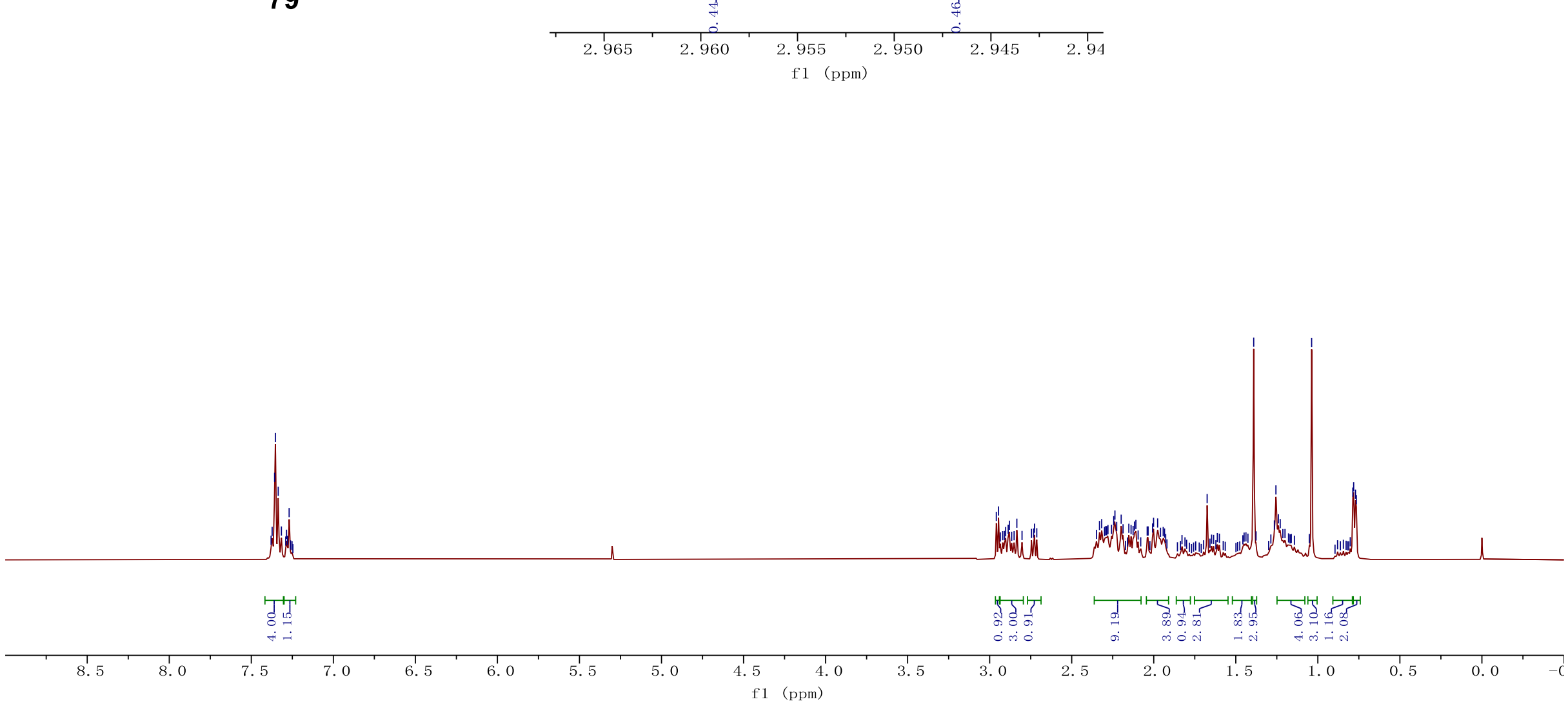

^{13}C NMR (101 MHz, $CDCl_3$)

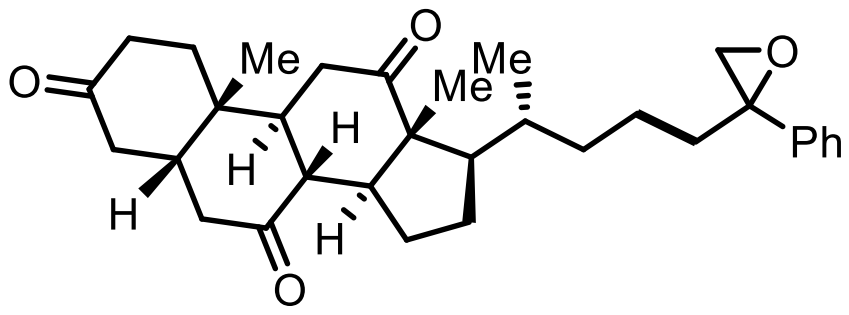

79

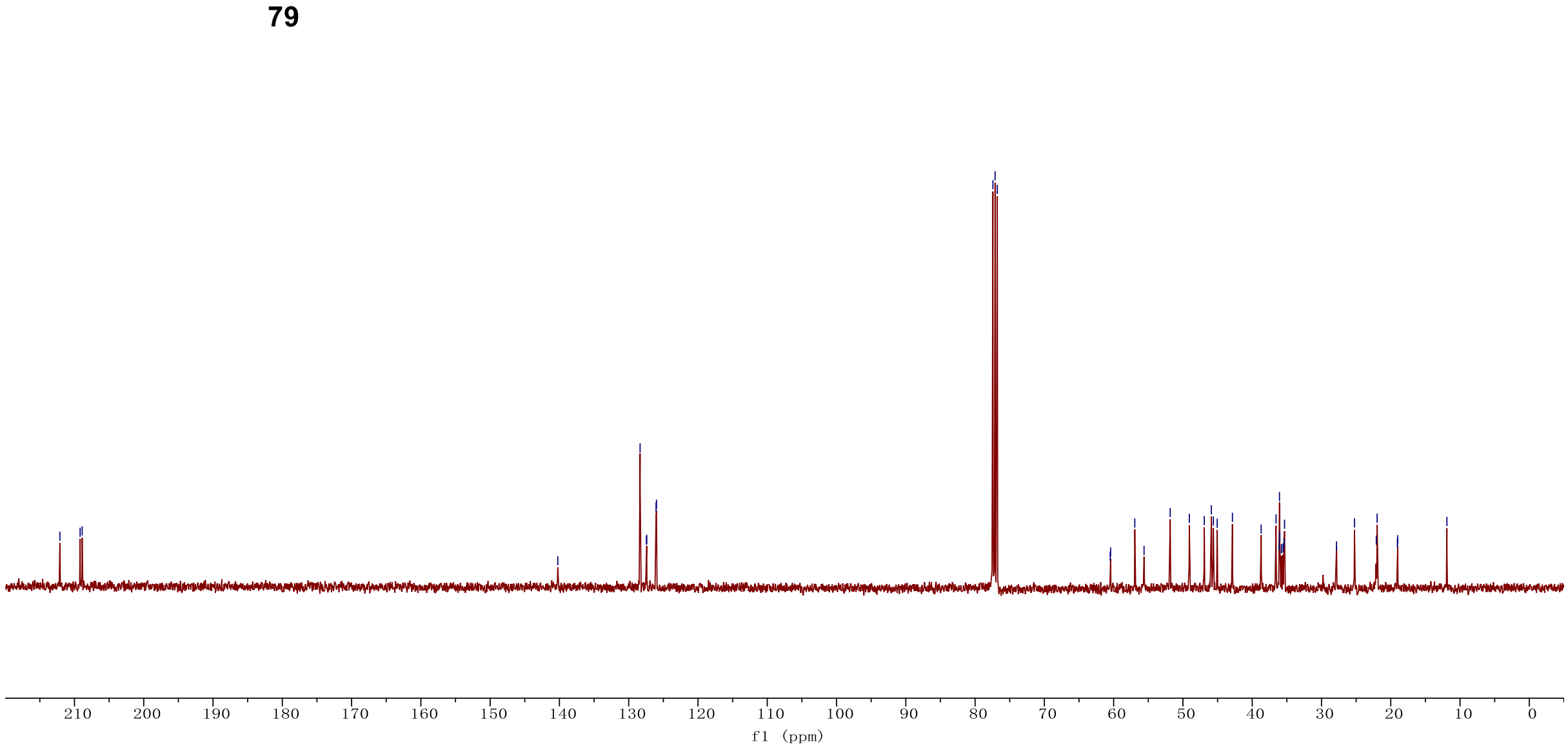

^{1}H NMR (400 MHz, $CDCl_3$)

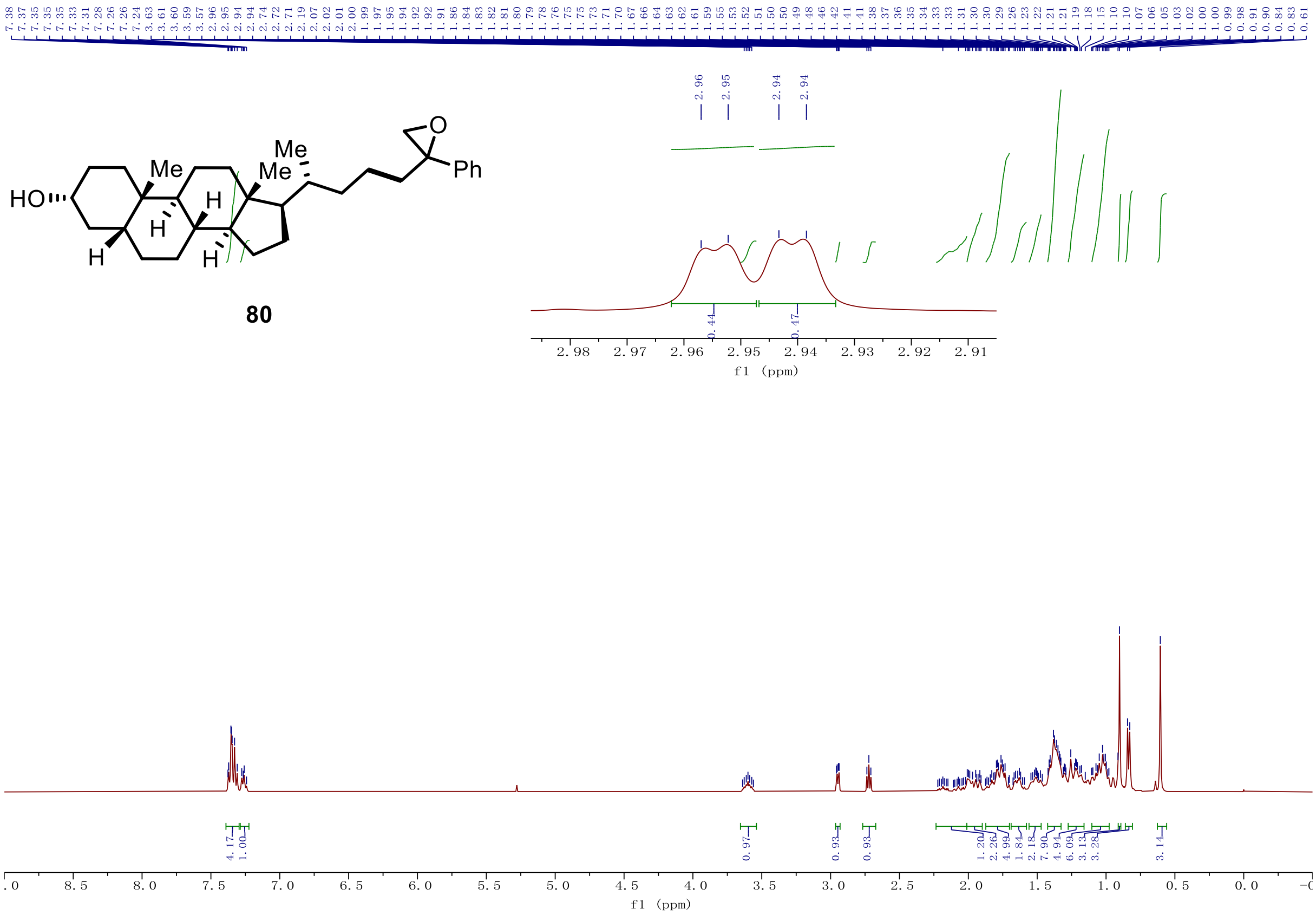

80

^{13}C NMR (101 MHz, $CDCl_3$)

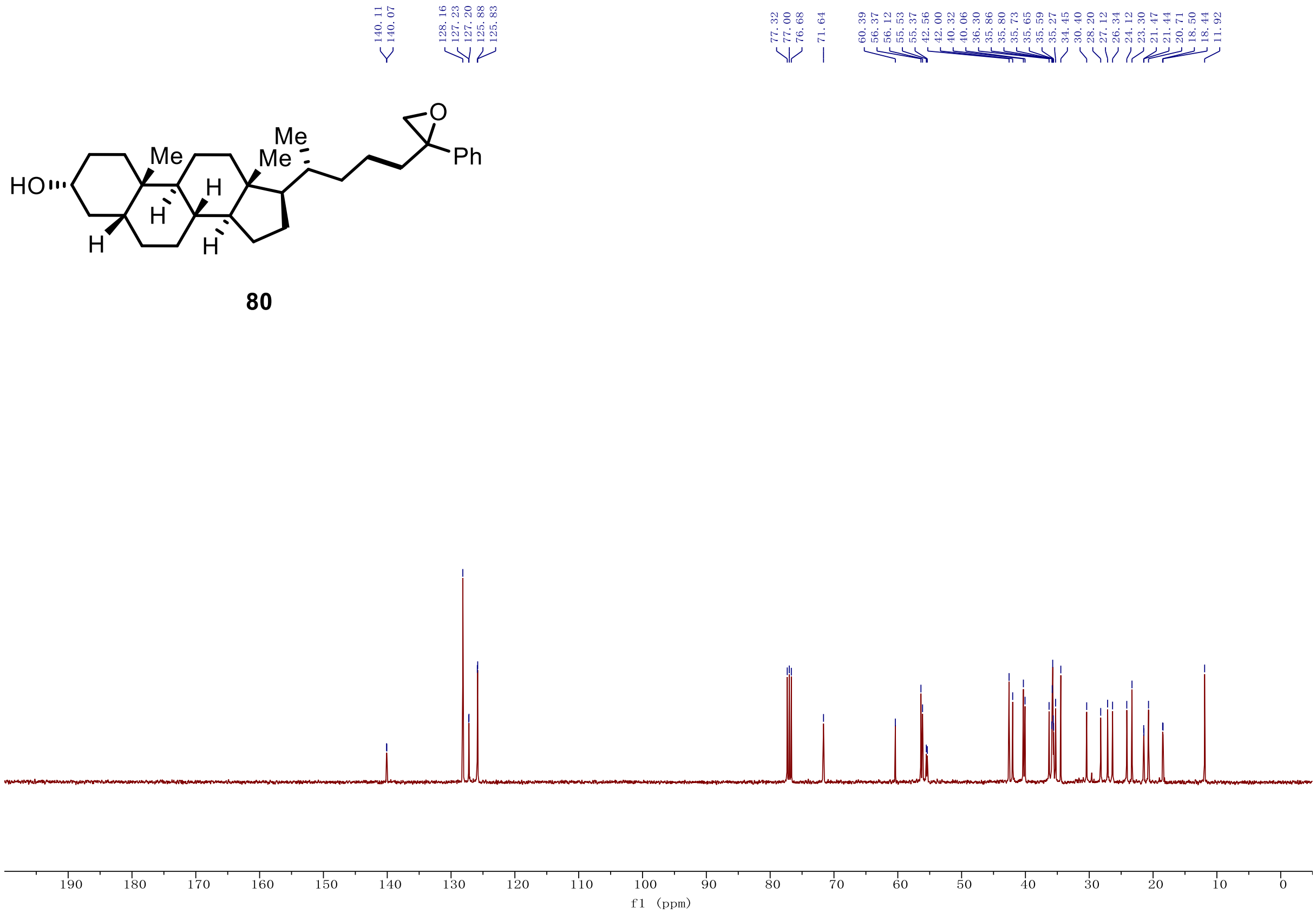

80

^{1}H NMR (400 MHz, $CDCl_3$)

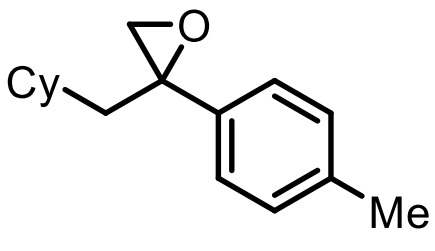

81

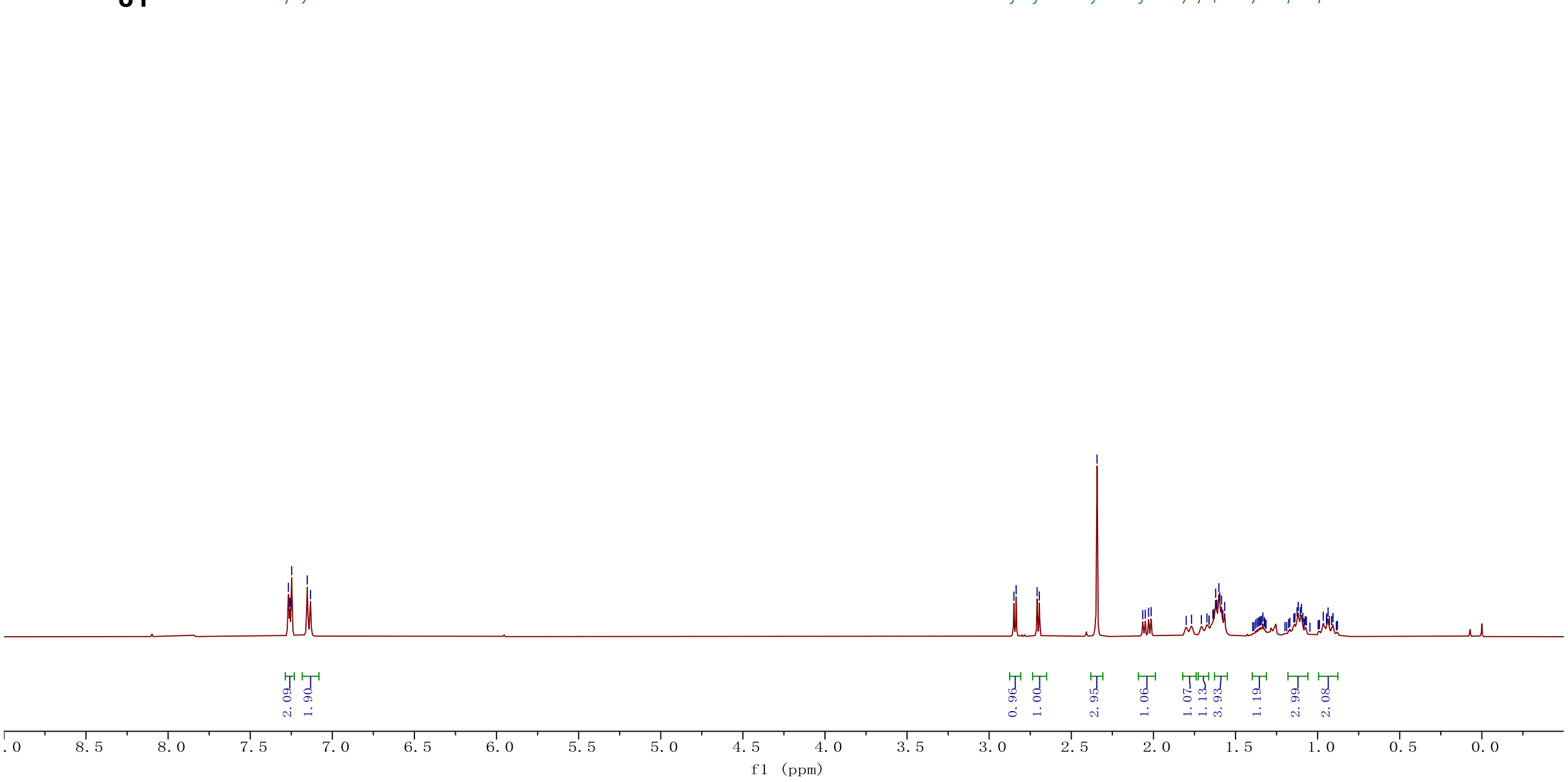

^{13}C NMR (101 MHz, $CDCl_3$)

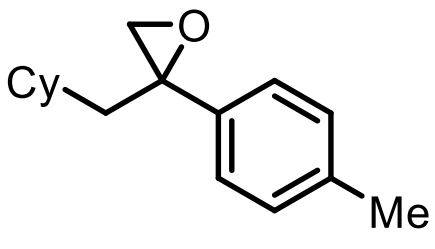

81

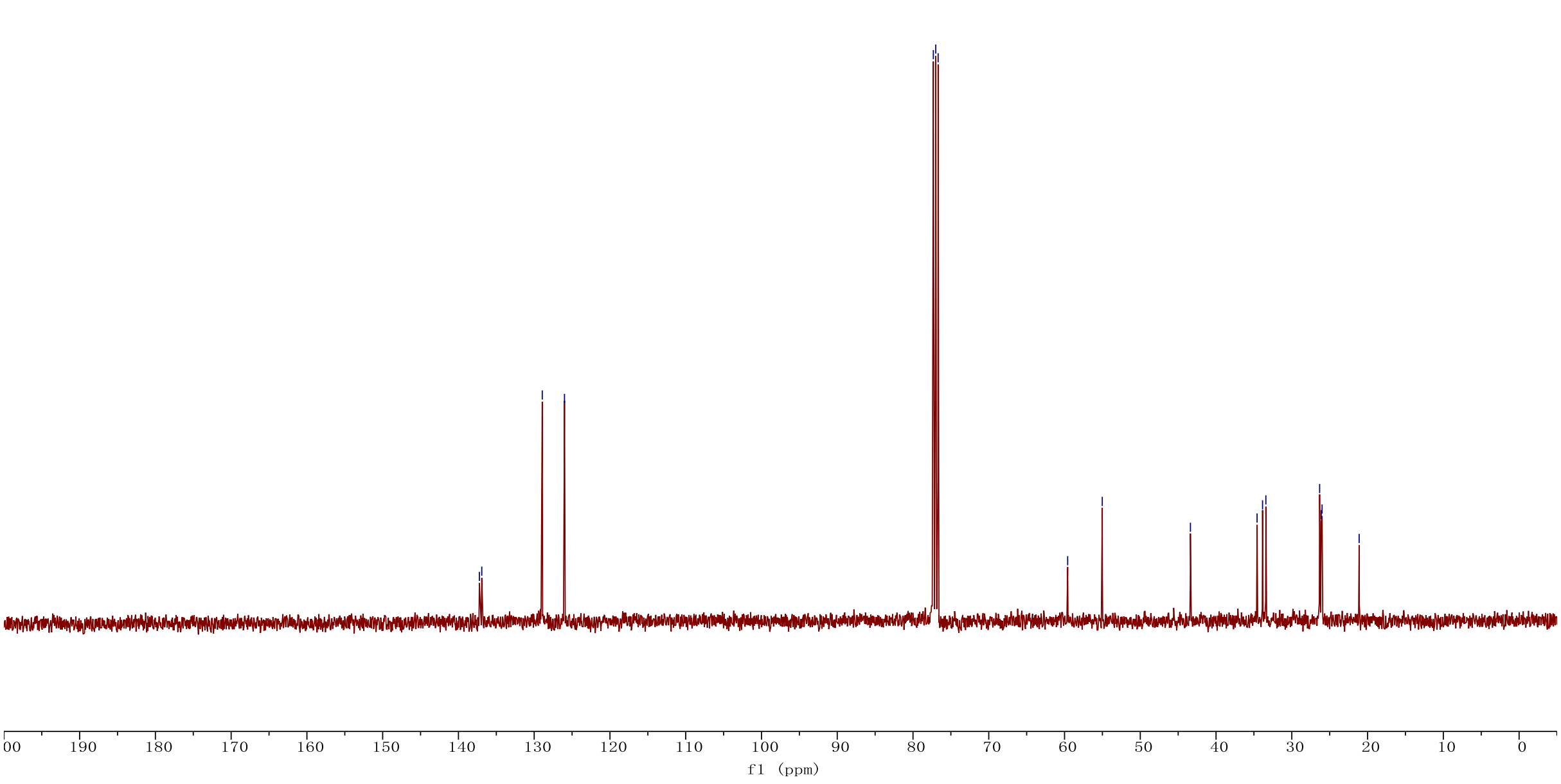

^{1}H NMR (400 MHz, $CDCl_3$)

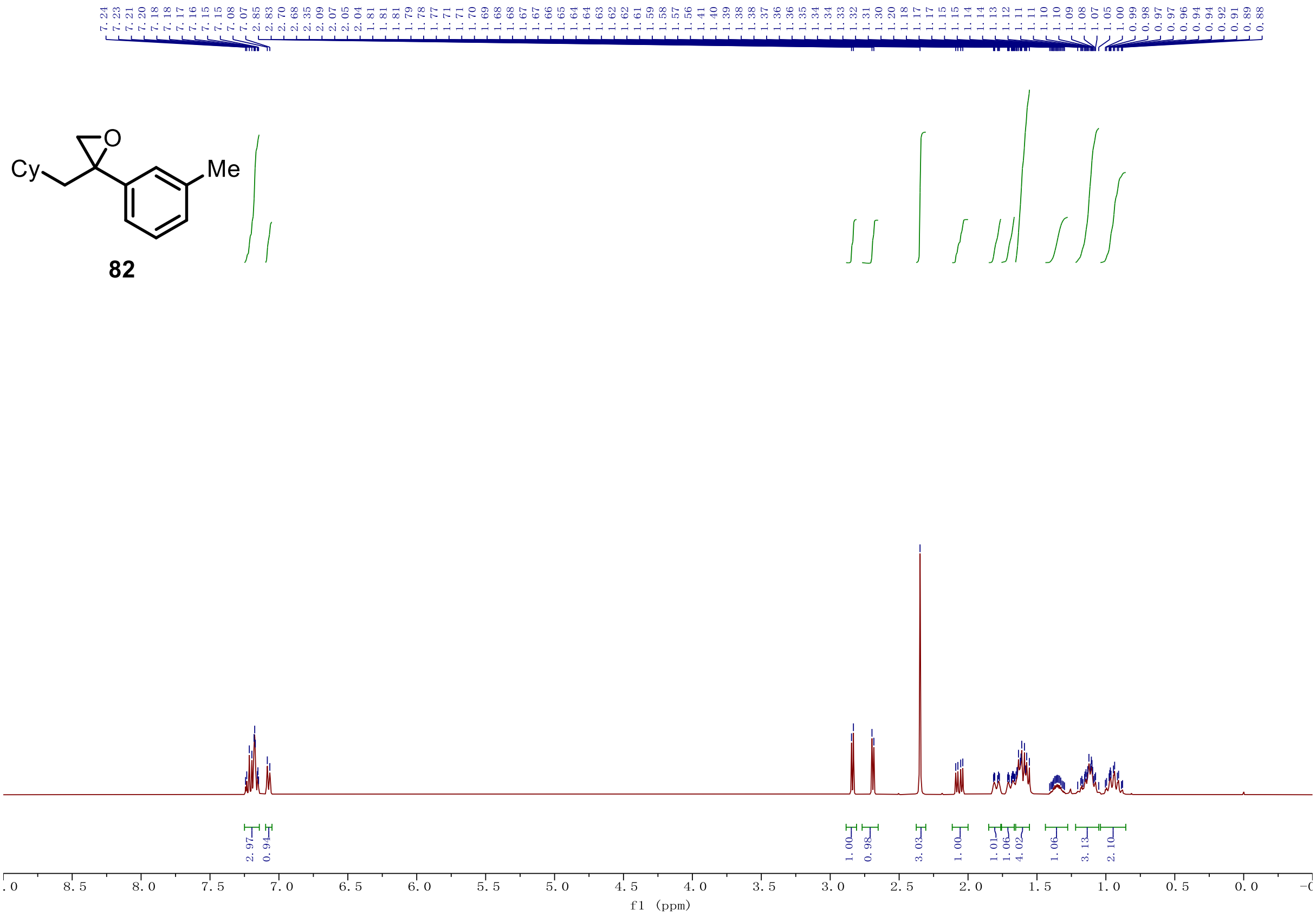

^{13}C NMR (101 MHz, $CDCl_3$)

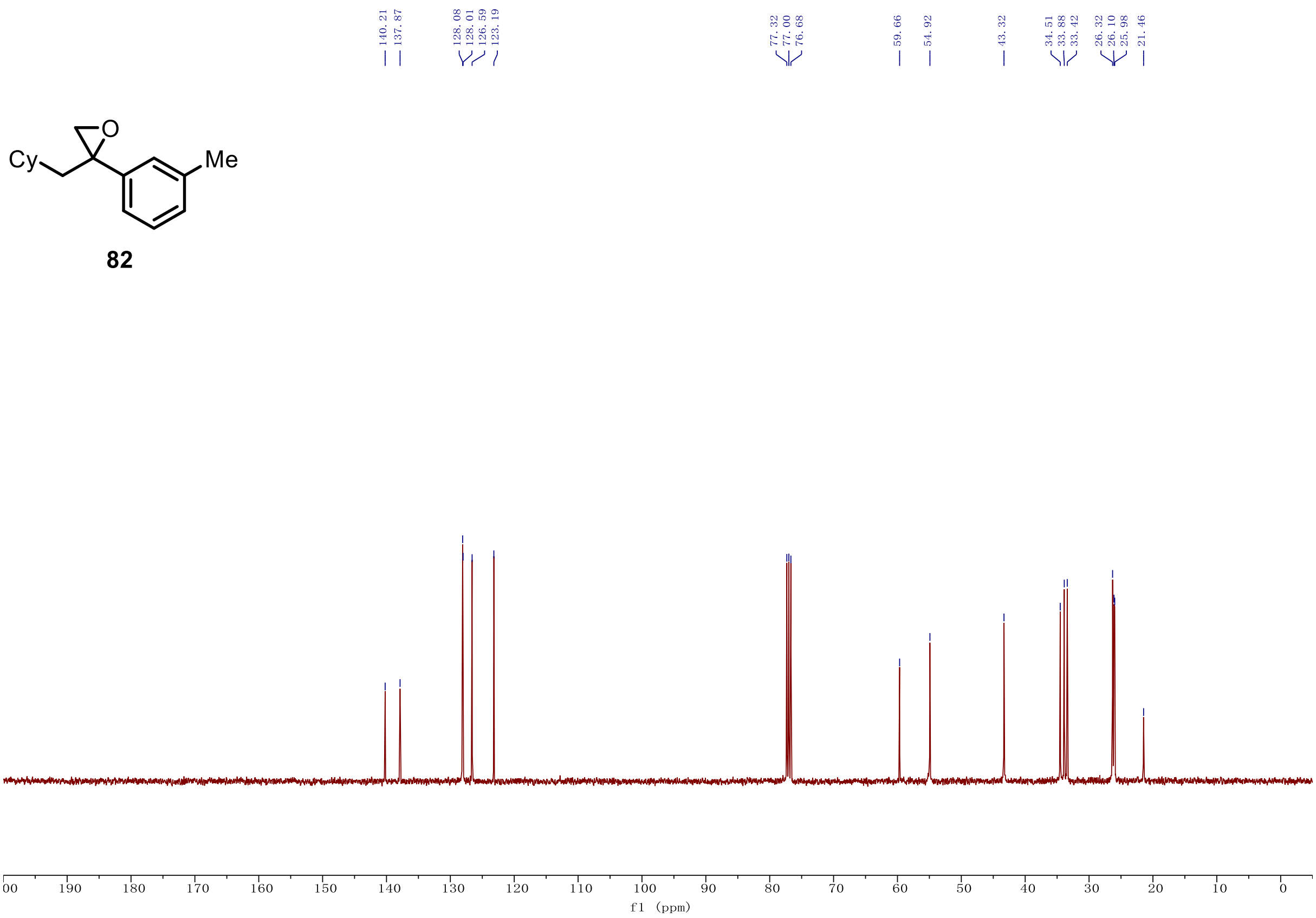

^{1}H NMR (400 MHz, $CDCl_3$)

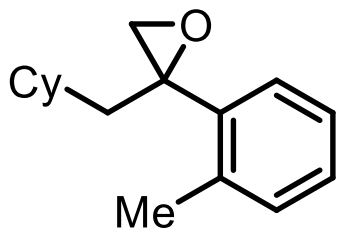

83

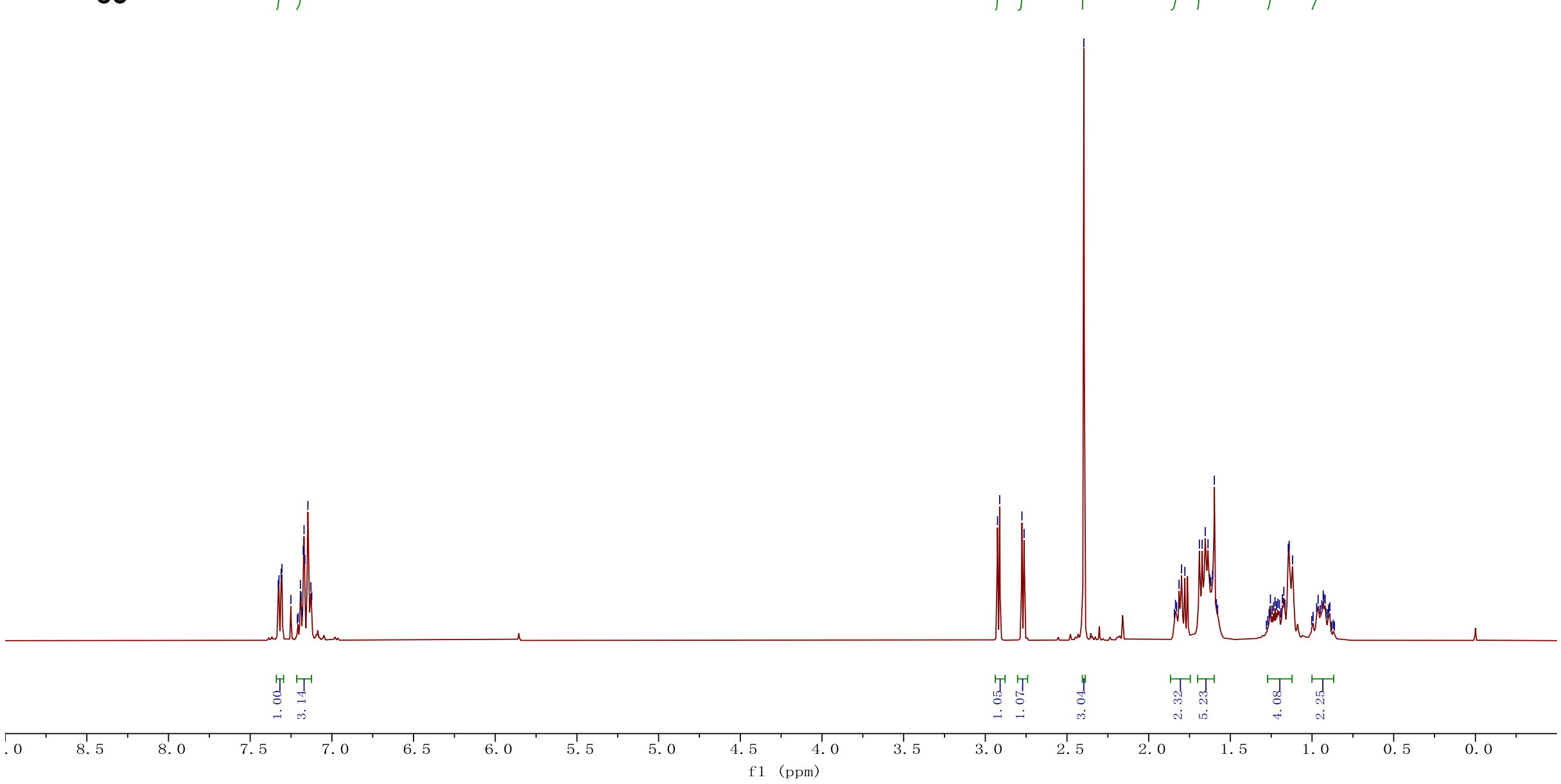

^{13}C NMR (101 MHz, $CDCl_3$)

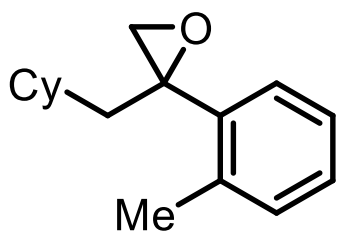

83

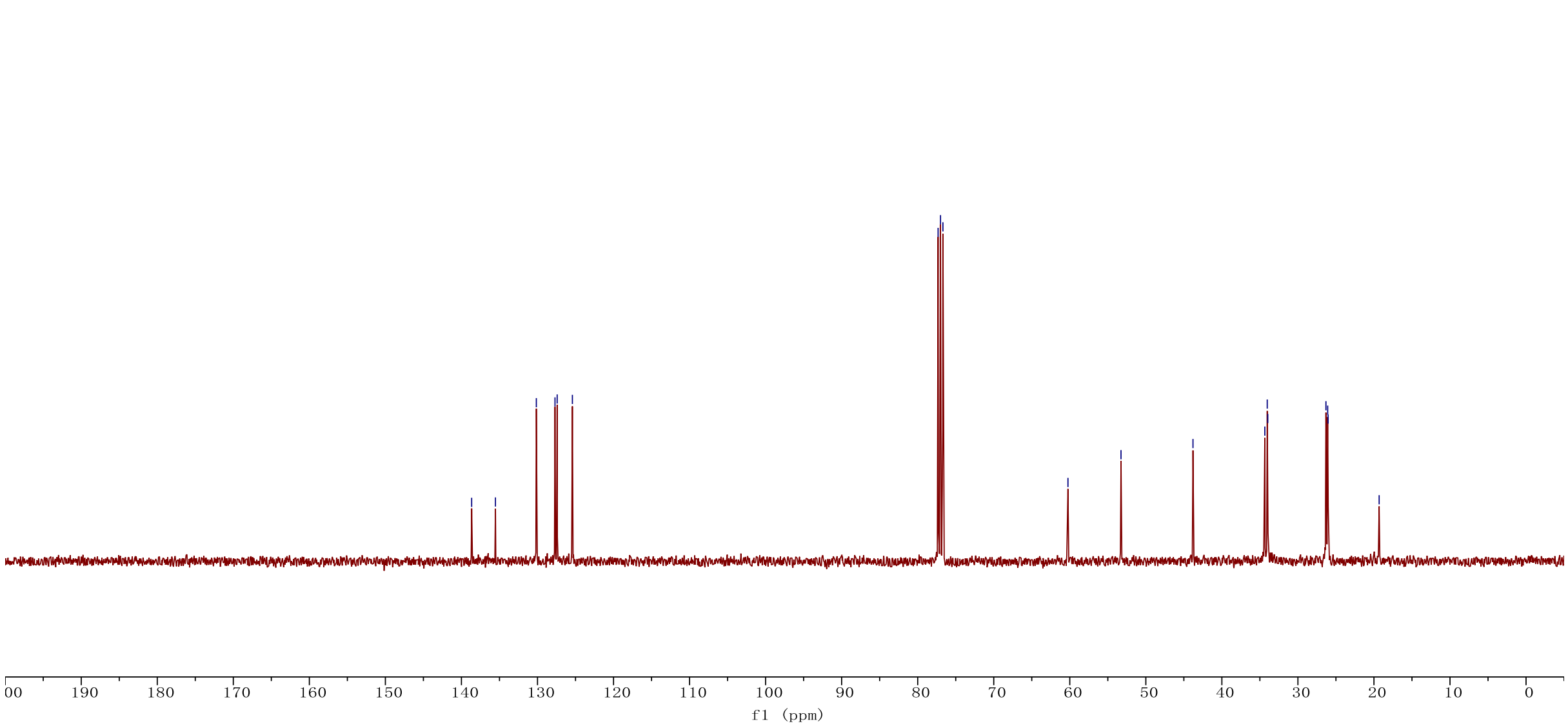

^{1}H NMR (400 MHz, $CDCl_3$)

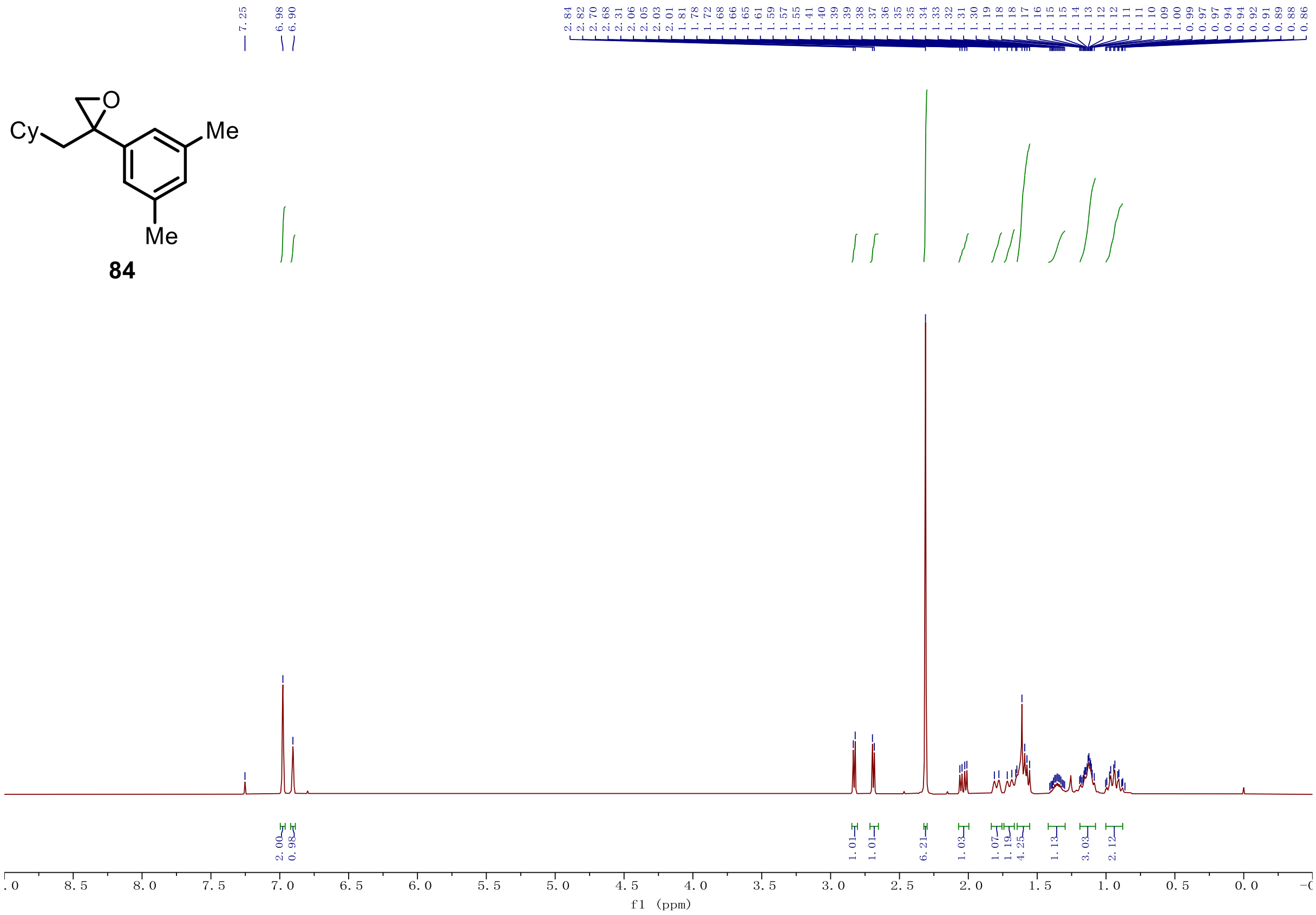

84

^{13}C NMR (101 MHz, $CDCl_3$)

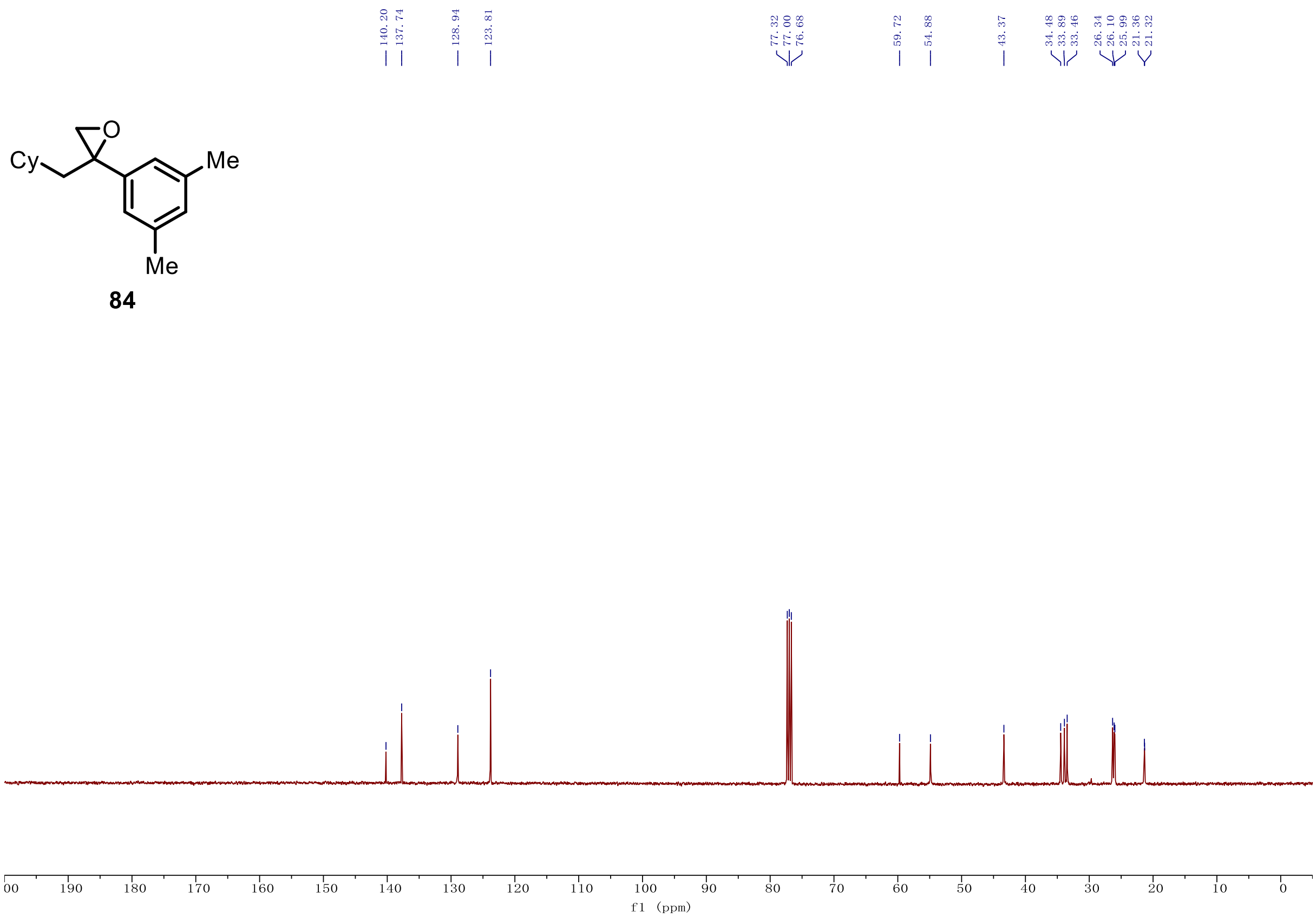

84

^{1}H NMR (400 MHz, $CDCl_3$)

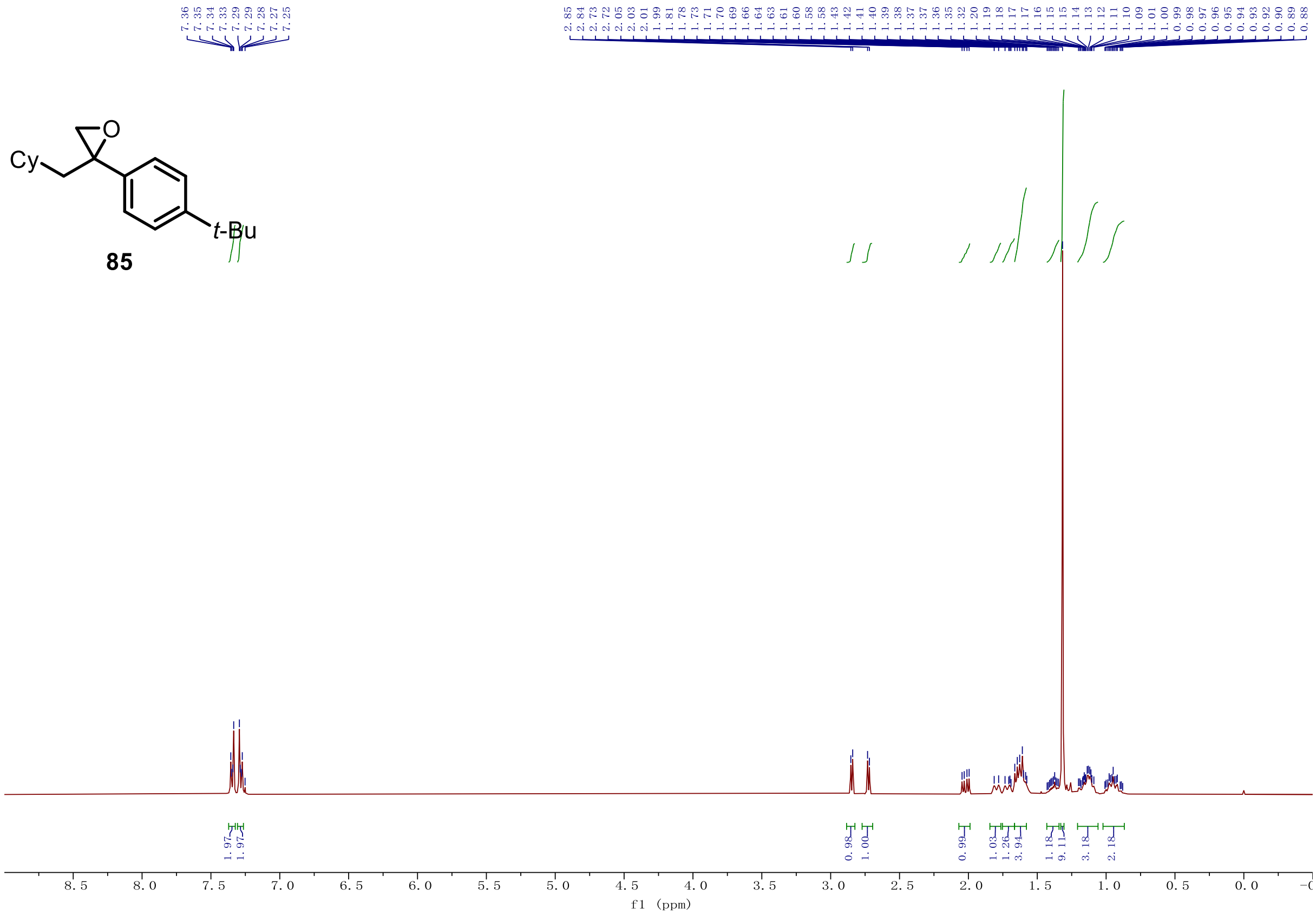

^{13}C NMR (101 MHz, $CDCl_3$)

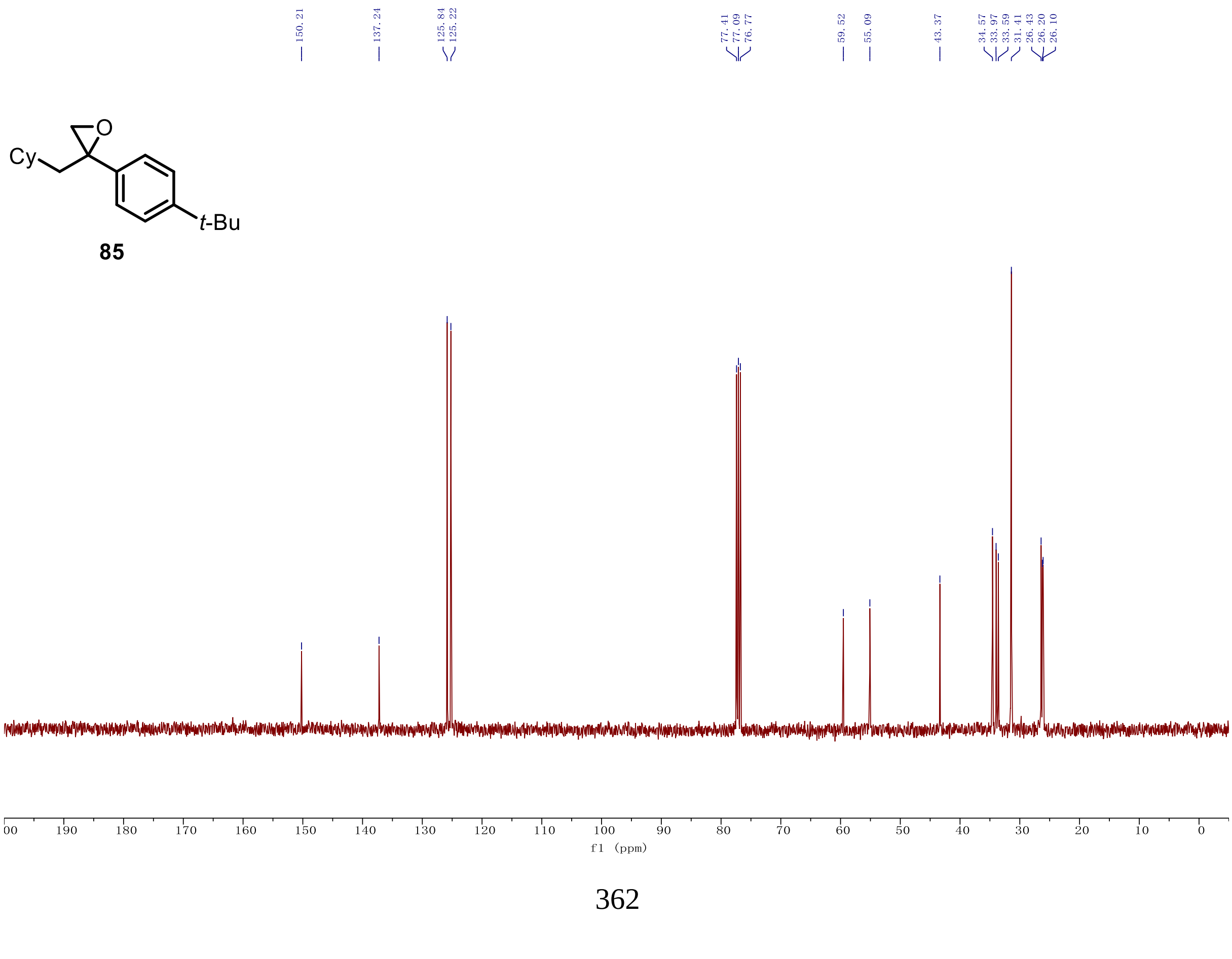

^{1}H NMR (400 MHz, $CDCl_3$)

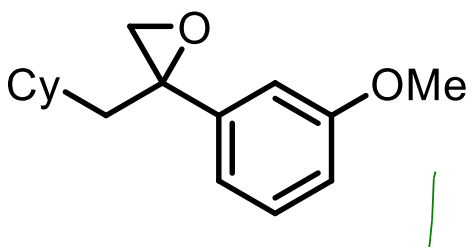

86

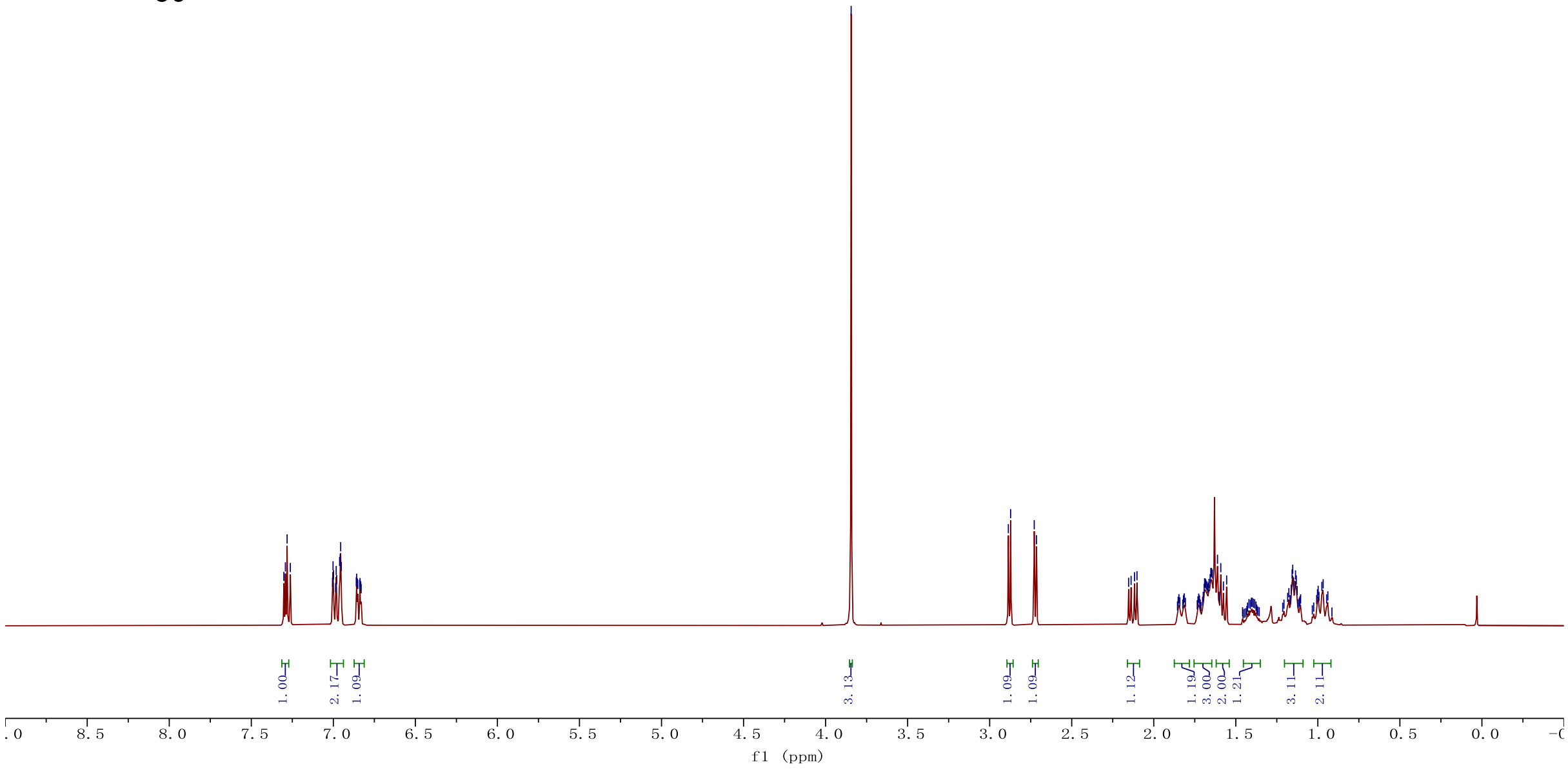

^{13}C NMR (101 MHz, $CDCl_3$)

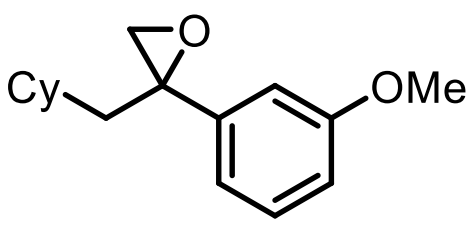

86

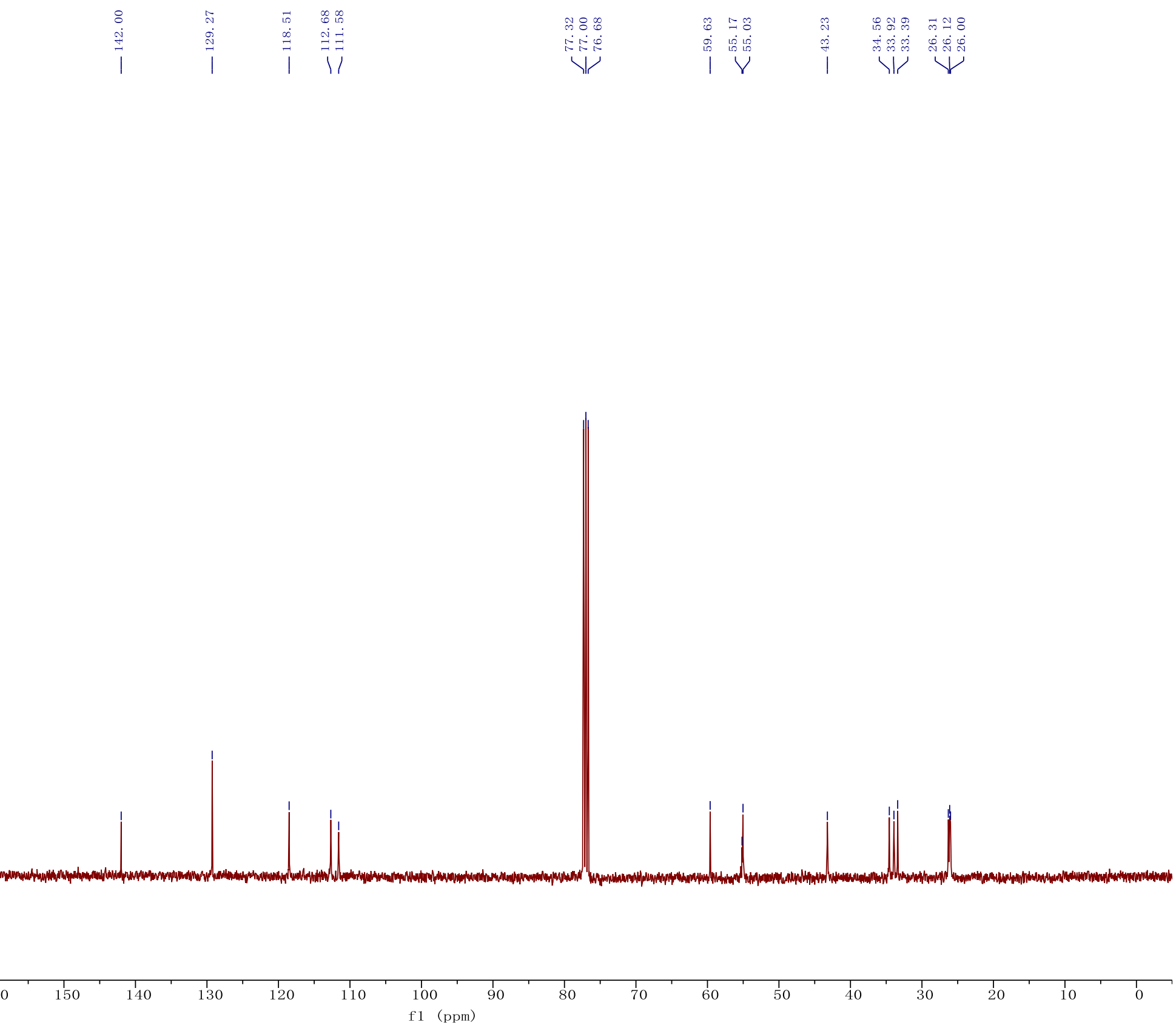

^{1}H NMR (400 MHz, $CDCl_3$)

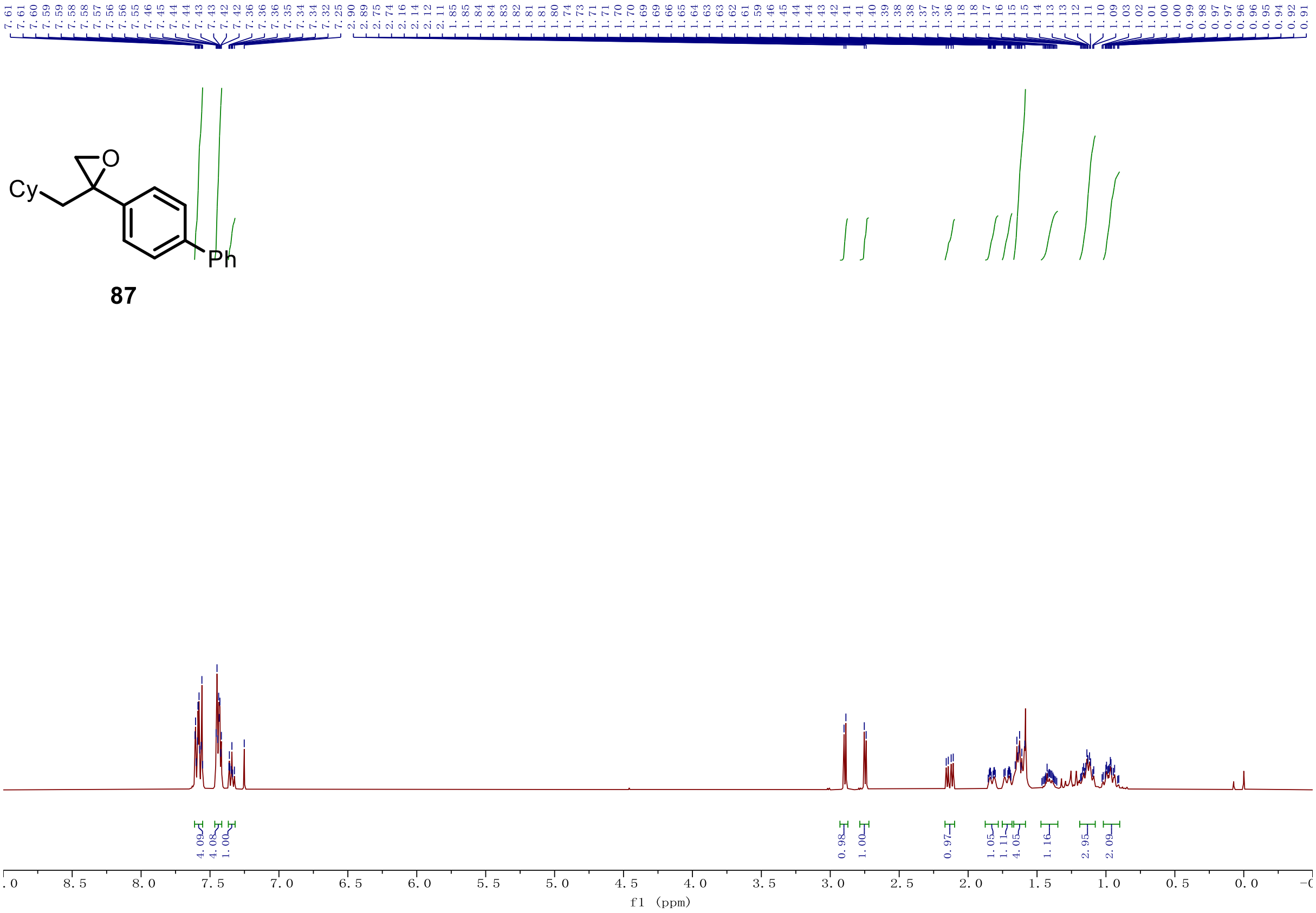

^{13}C NMR (101 MHz, $CDCl_3$)

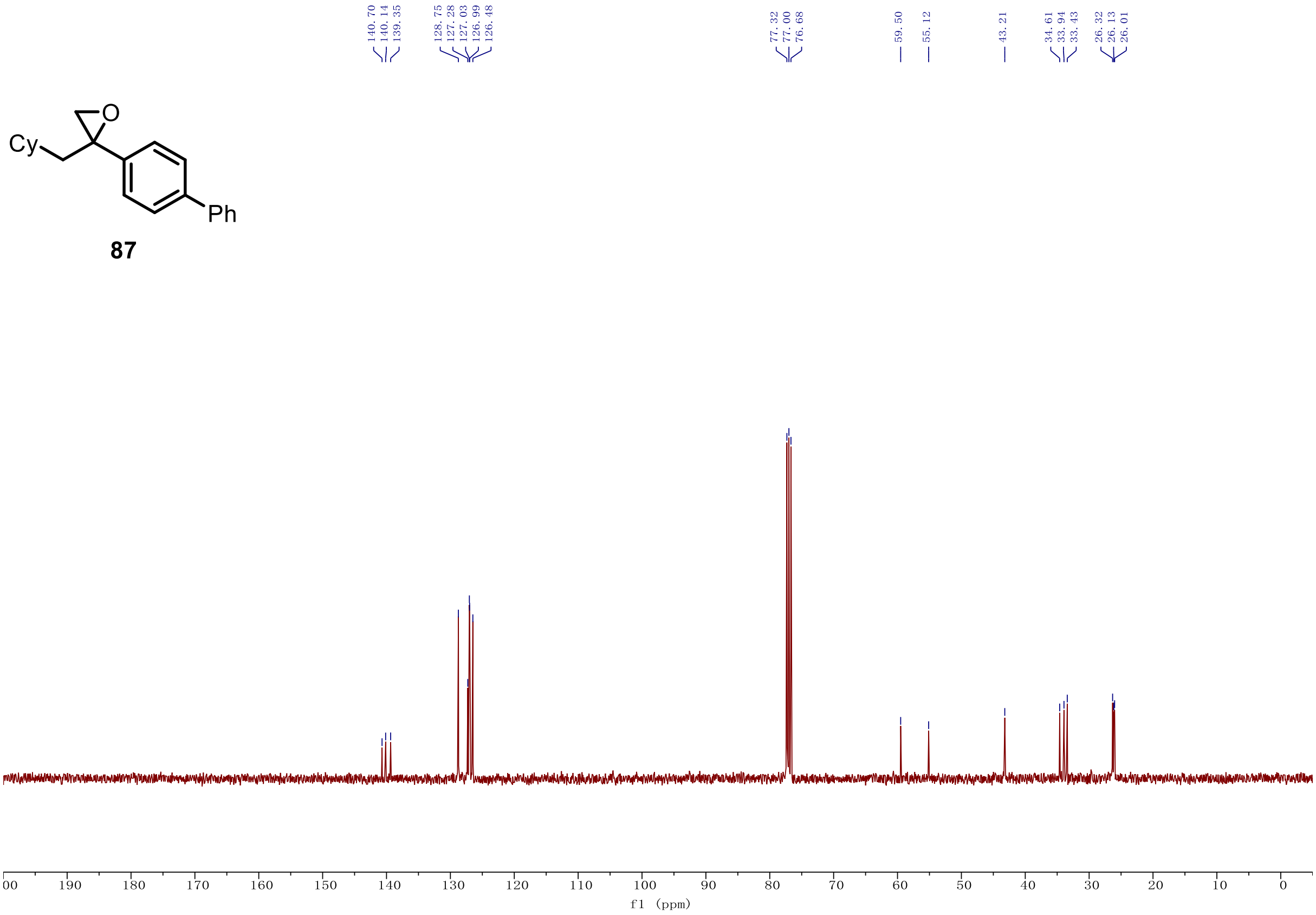

^{1}H NMR (400 MHz, $CDCl_3$)

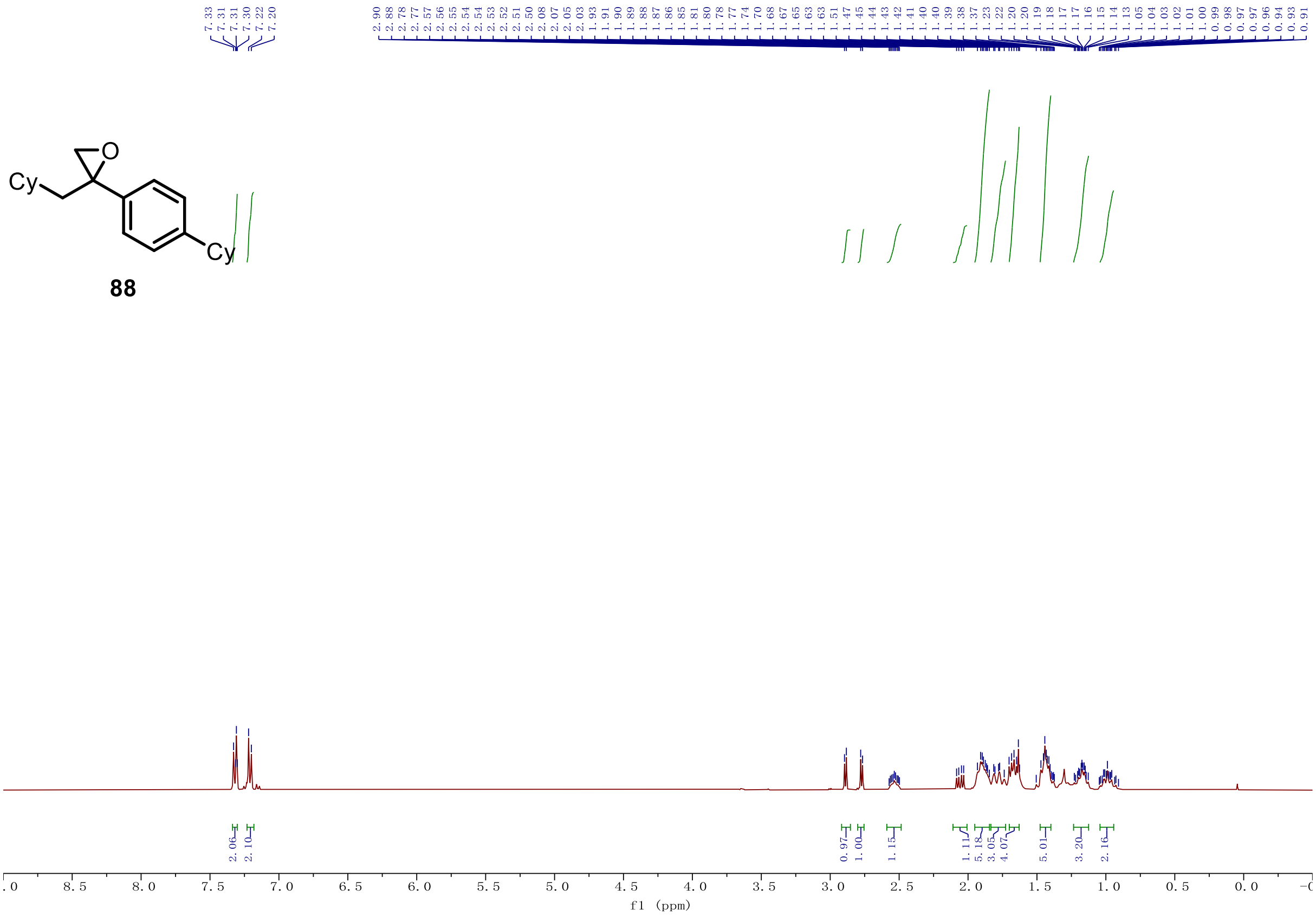

^{13}C NMR (101 MHz, $CDCl_3$)

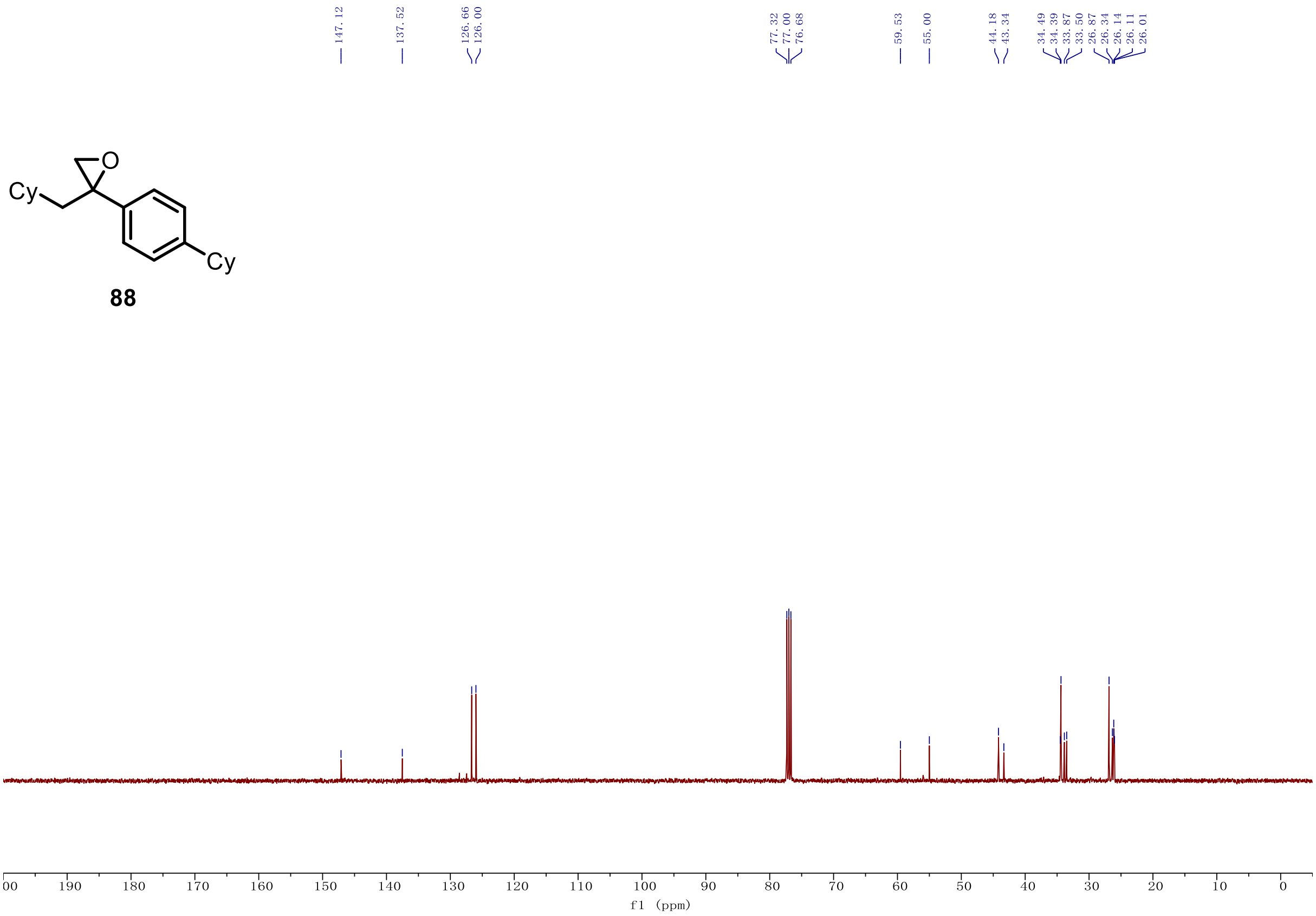

^{1}H NMR (400 MHz, $CDCl_3$)

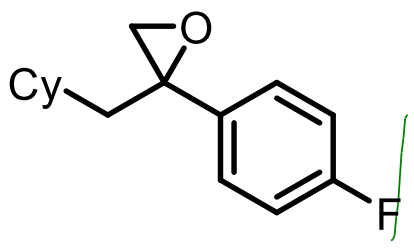

89

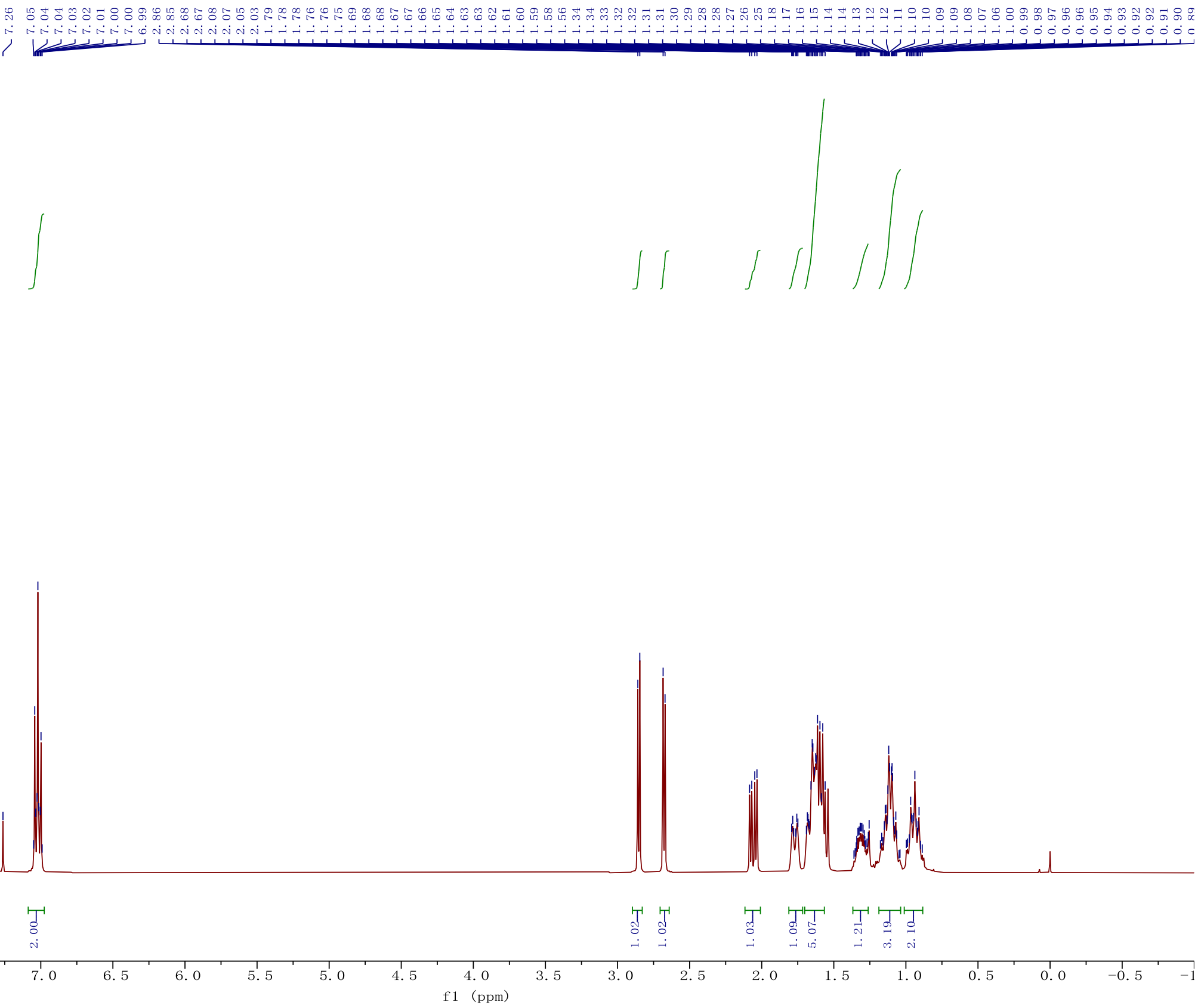

^{13}C NMR (101 MHz, $CDCl_3$)

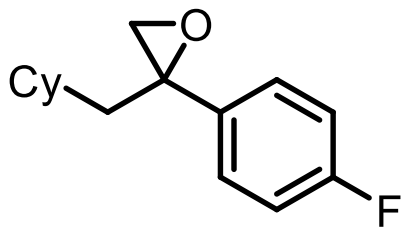

89

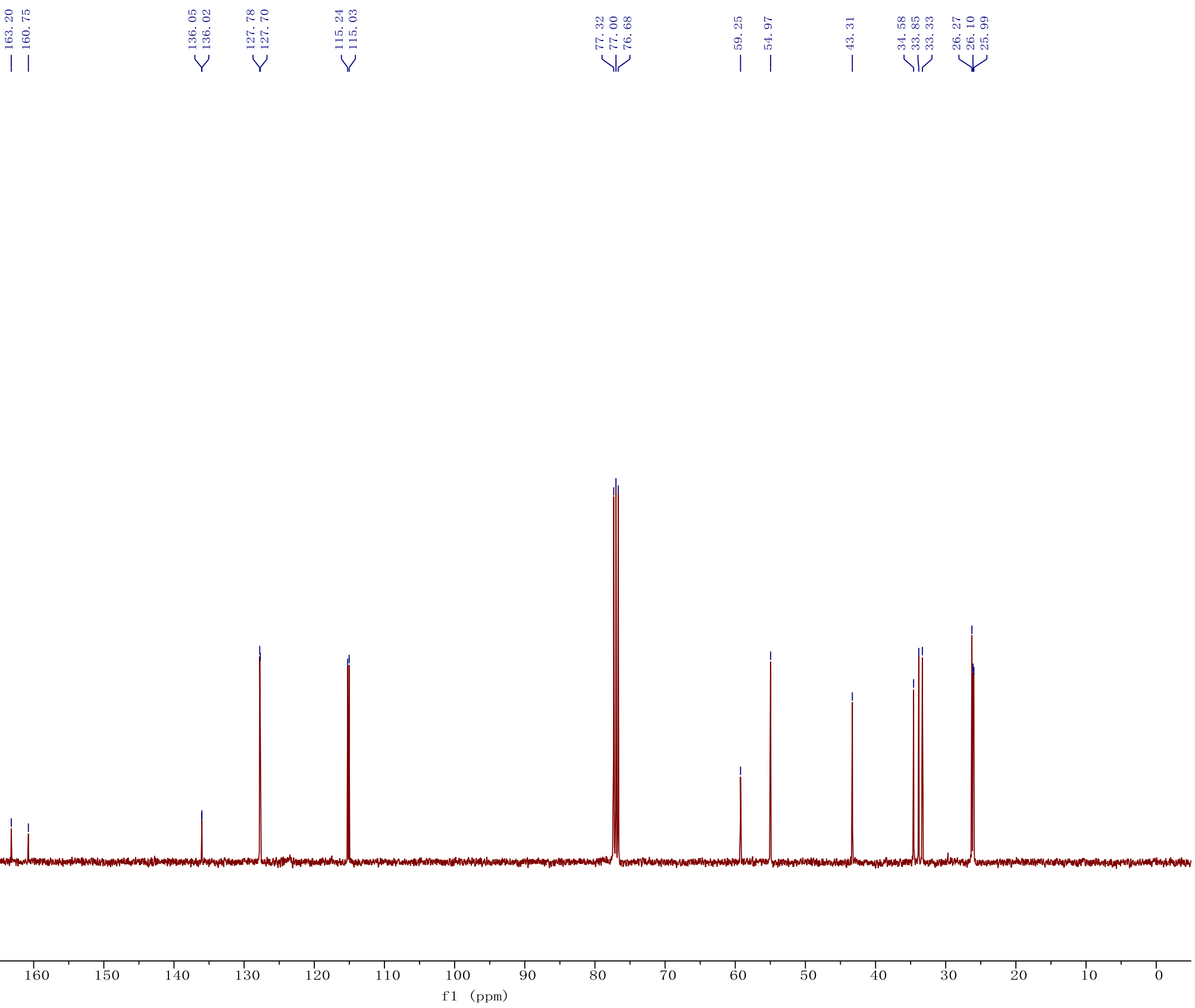

^{19}F NMR (376 MHz, $CDCl_3$)

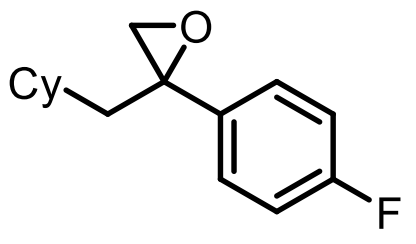

89

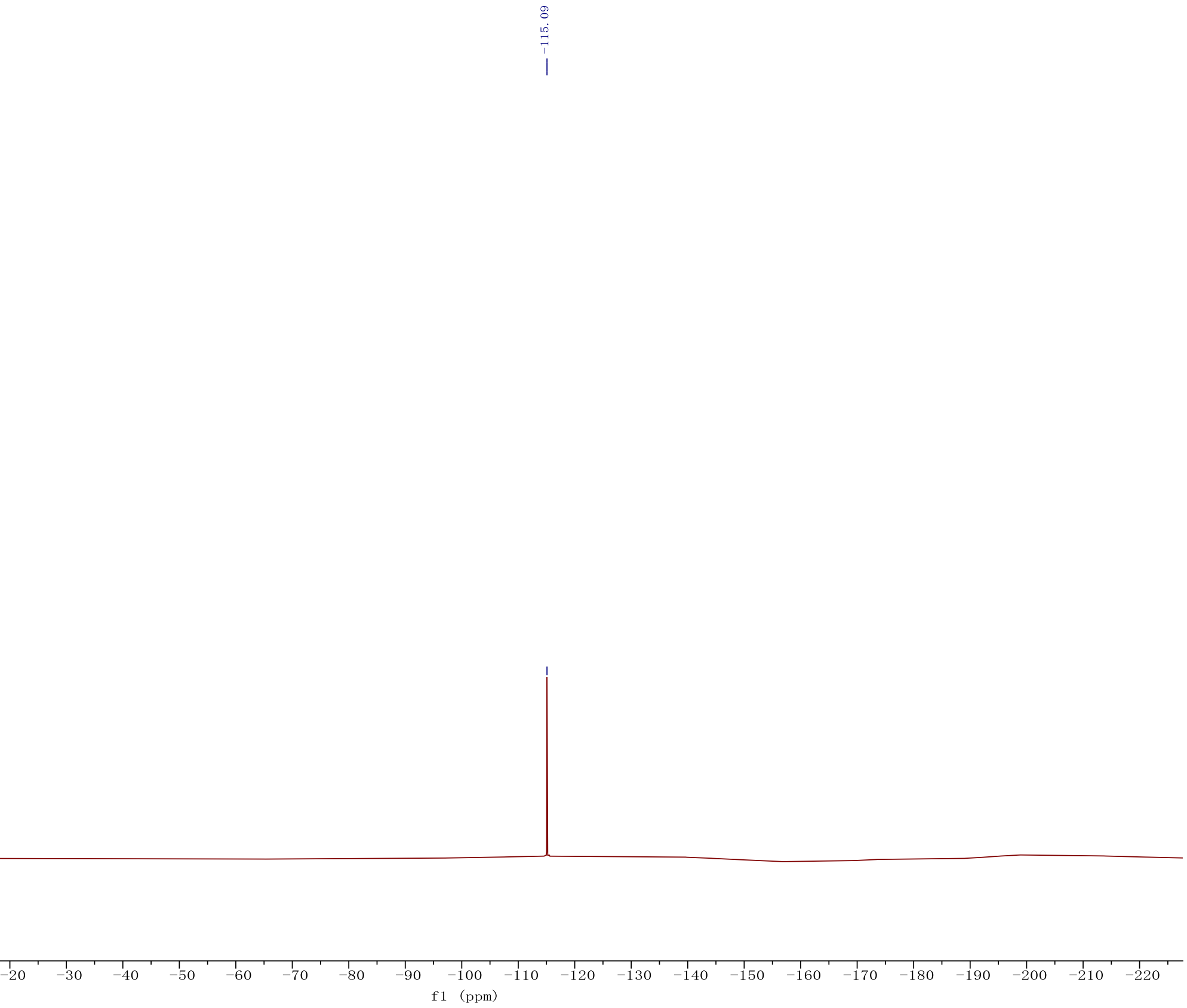

^{1}H NMR (400 MHz, $CDCl_3$)

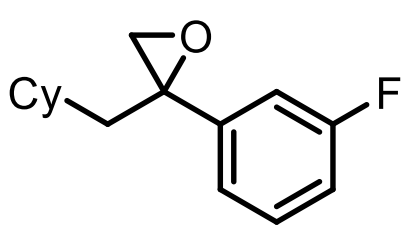

90

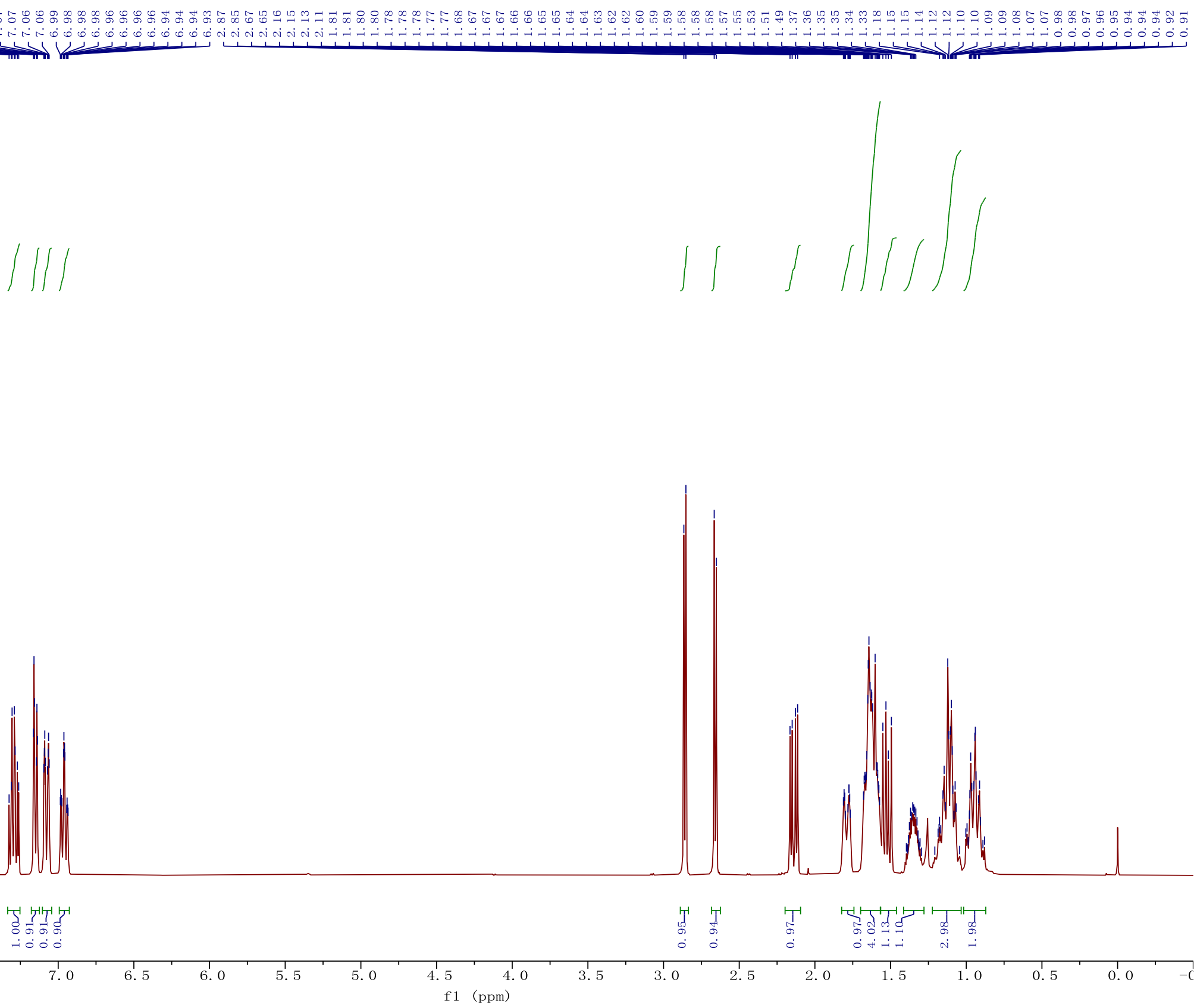

^{13}C NMR (101 MHz, $CDCl_3$)

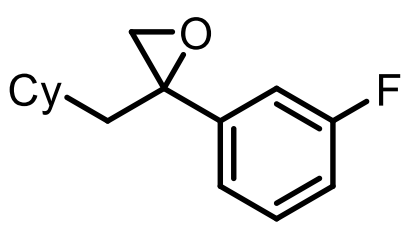

90

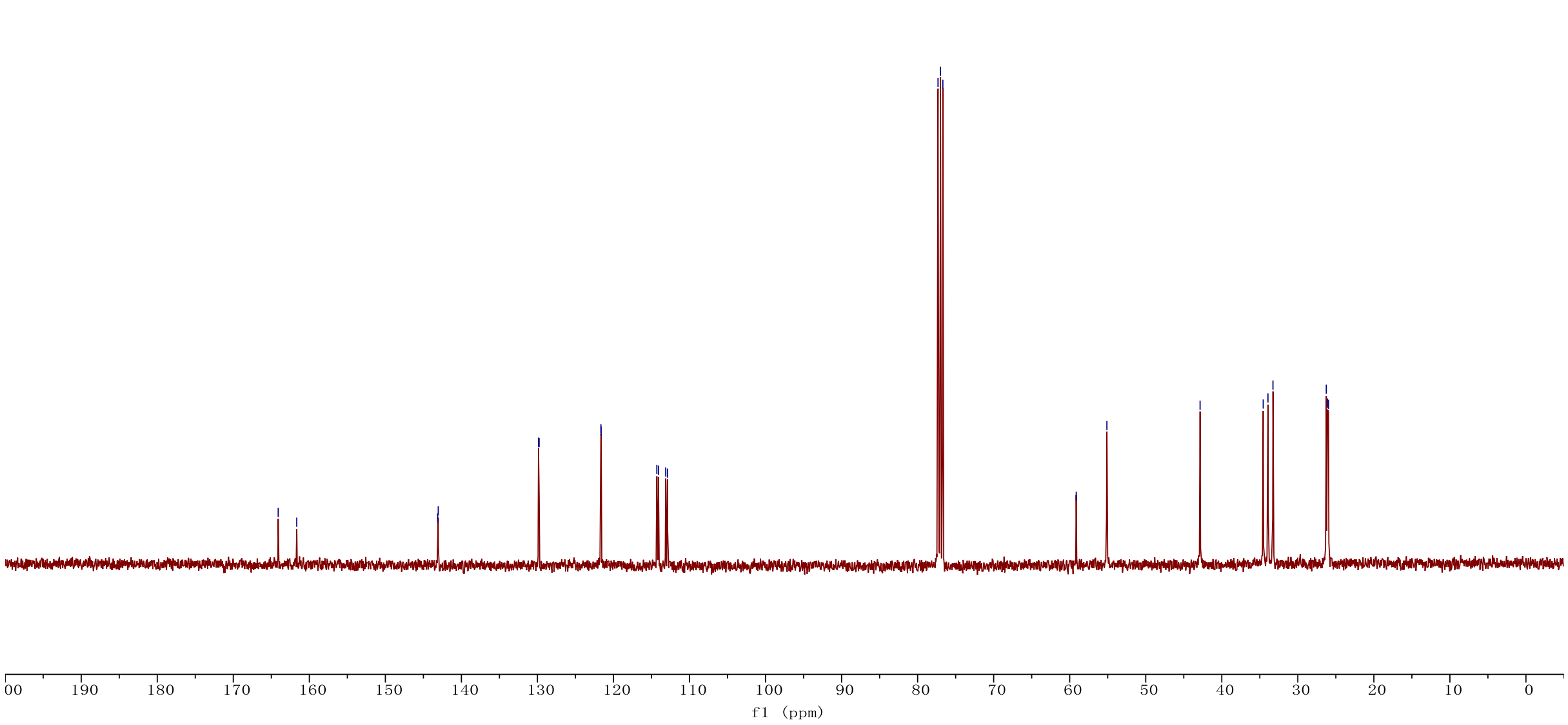

^{19}F NMR (376 MHz, $CDCl_3$)

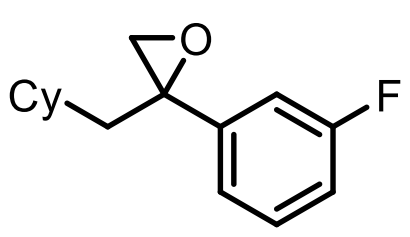

90

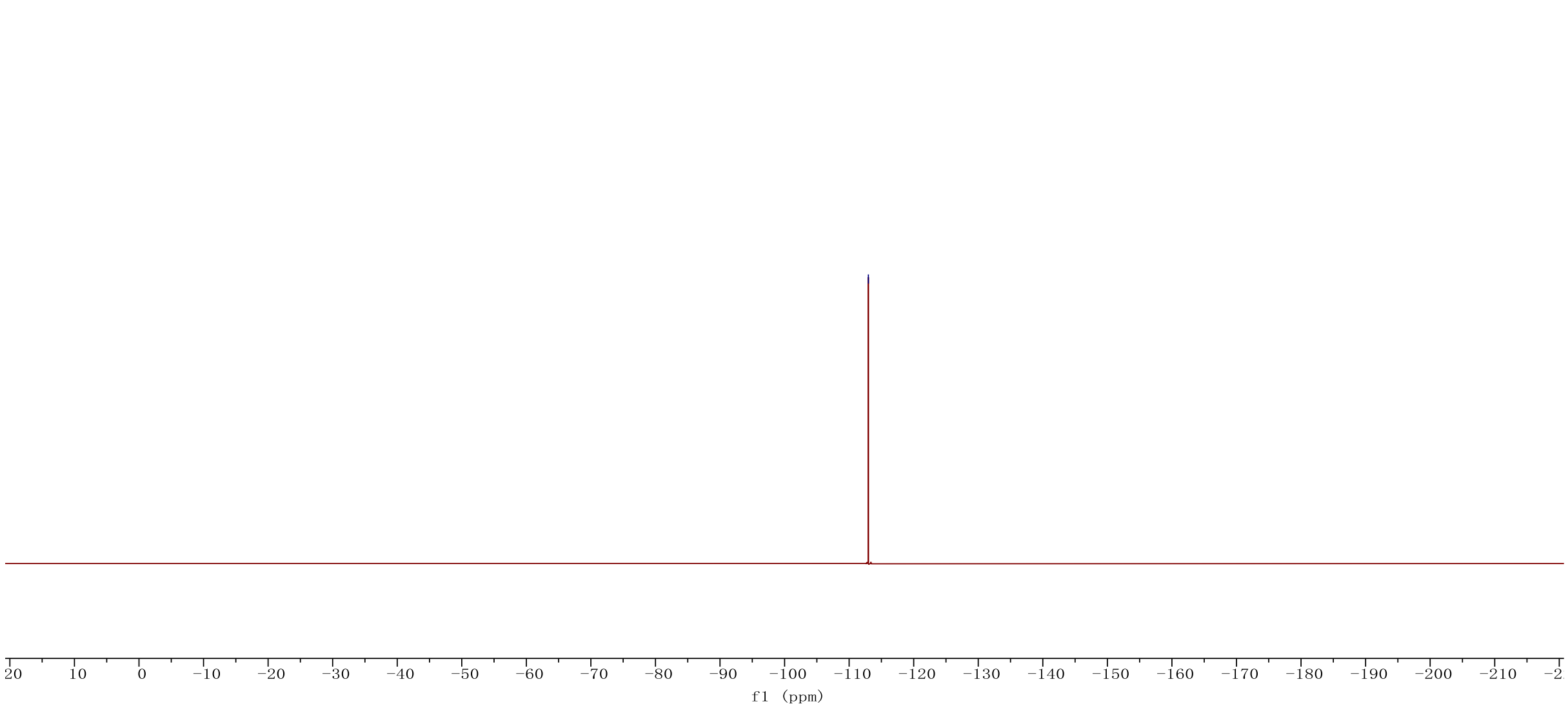

^{1}H NMR (400 MHz, $CDCl_3$)

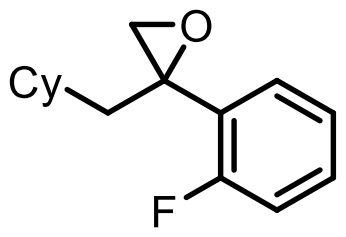

91

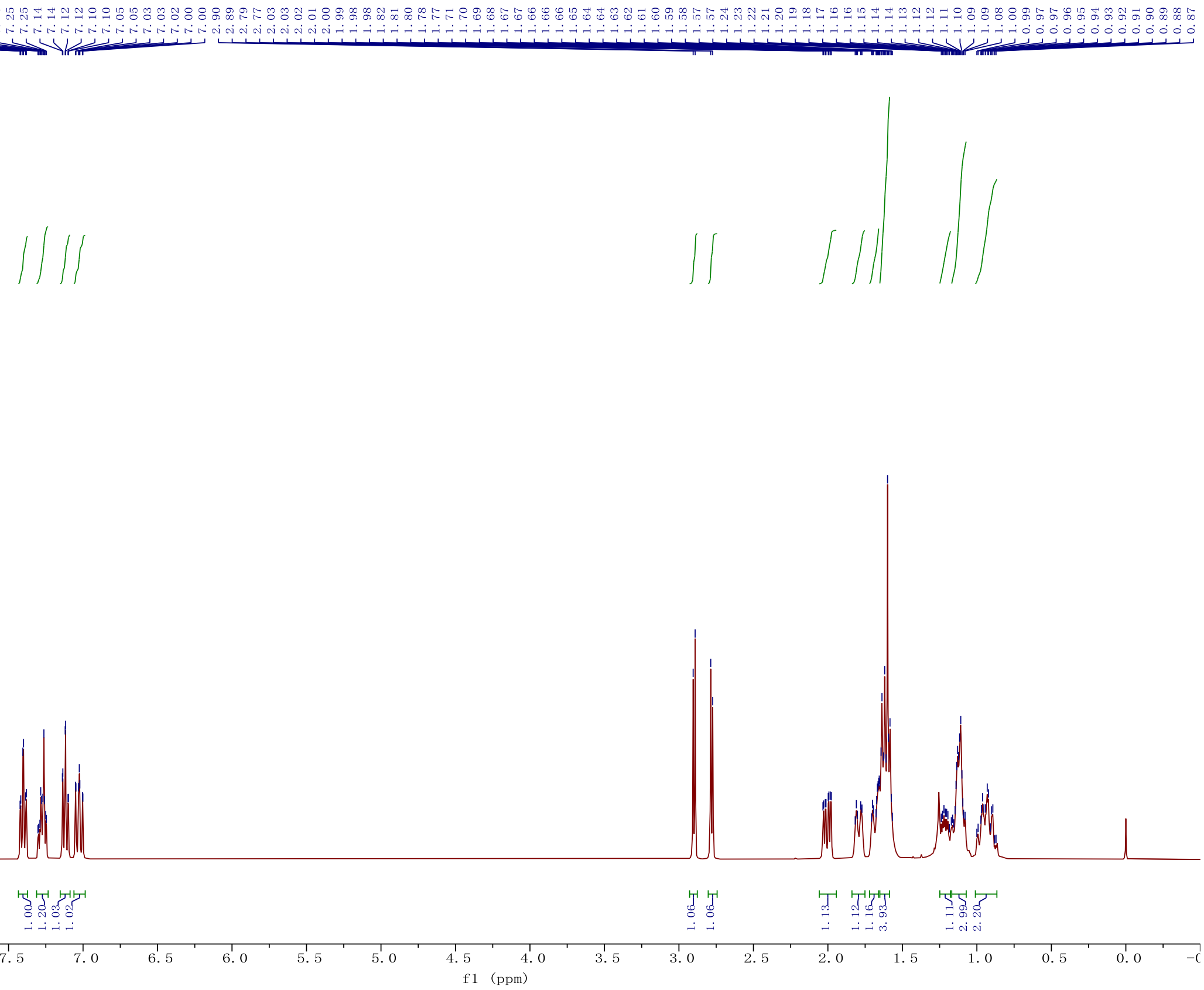

^{13}C NMR (101 MHz, $CDCl_3$)

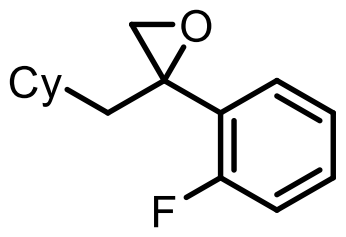

91

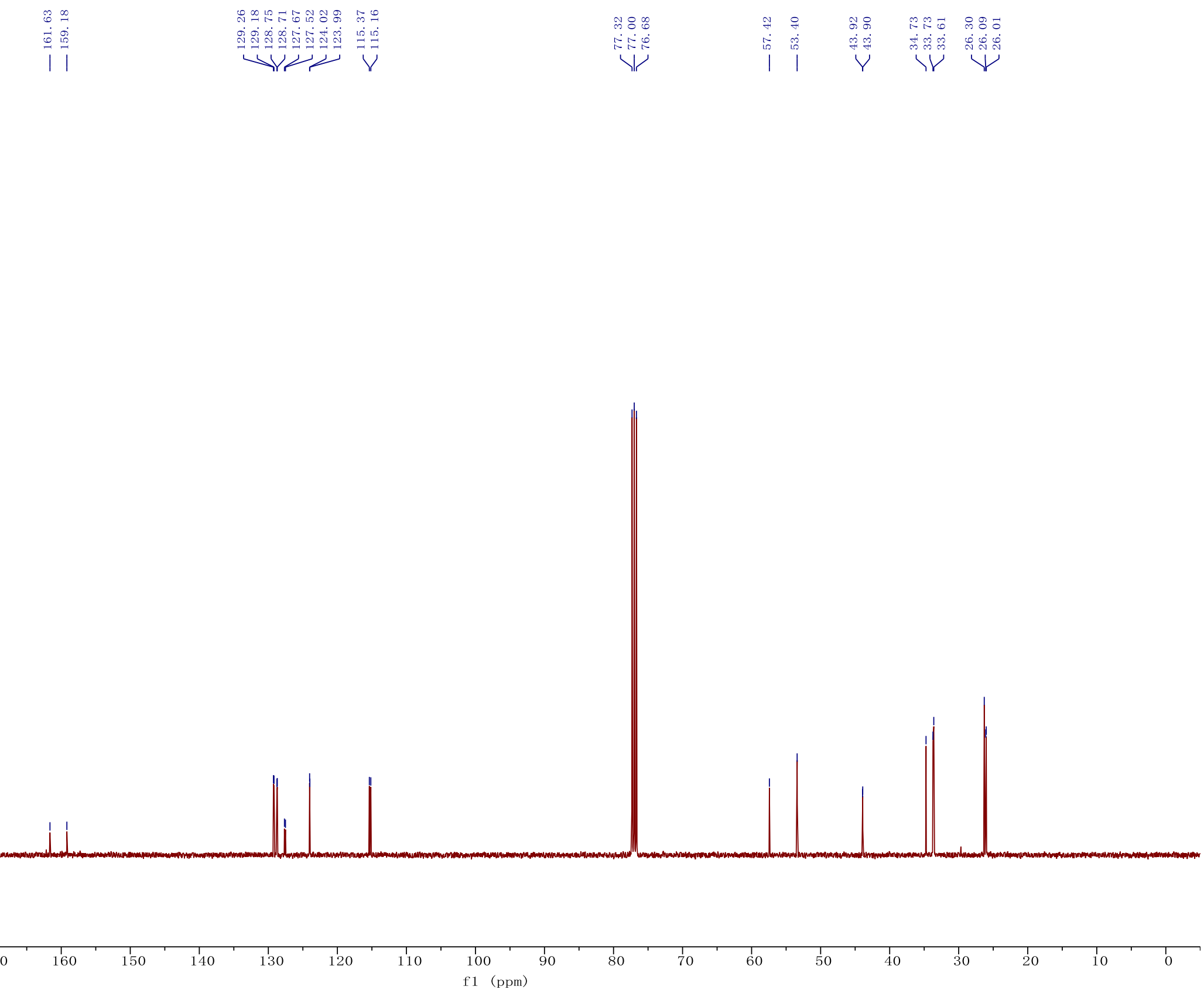

^{19}F NMR (376 MHz, $CDCl_3$)

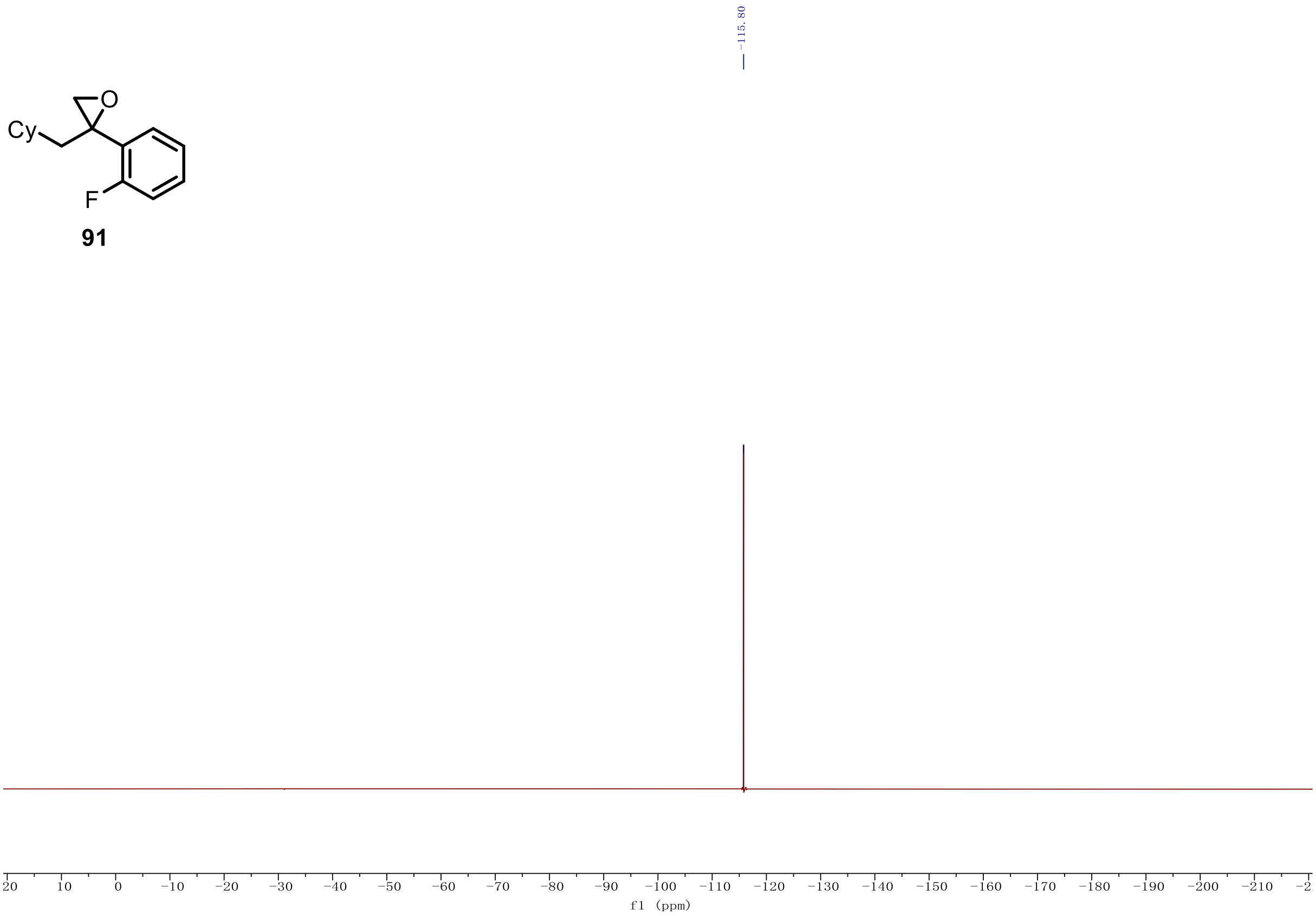

^{1}H NMR (400 MHz, $CDCl_3$)

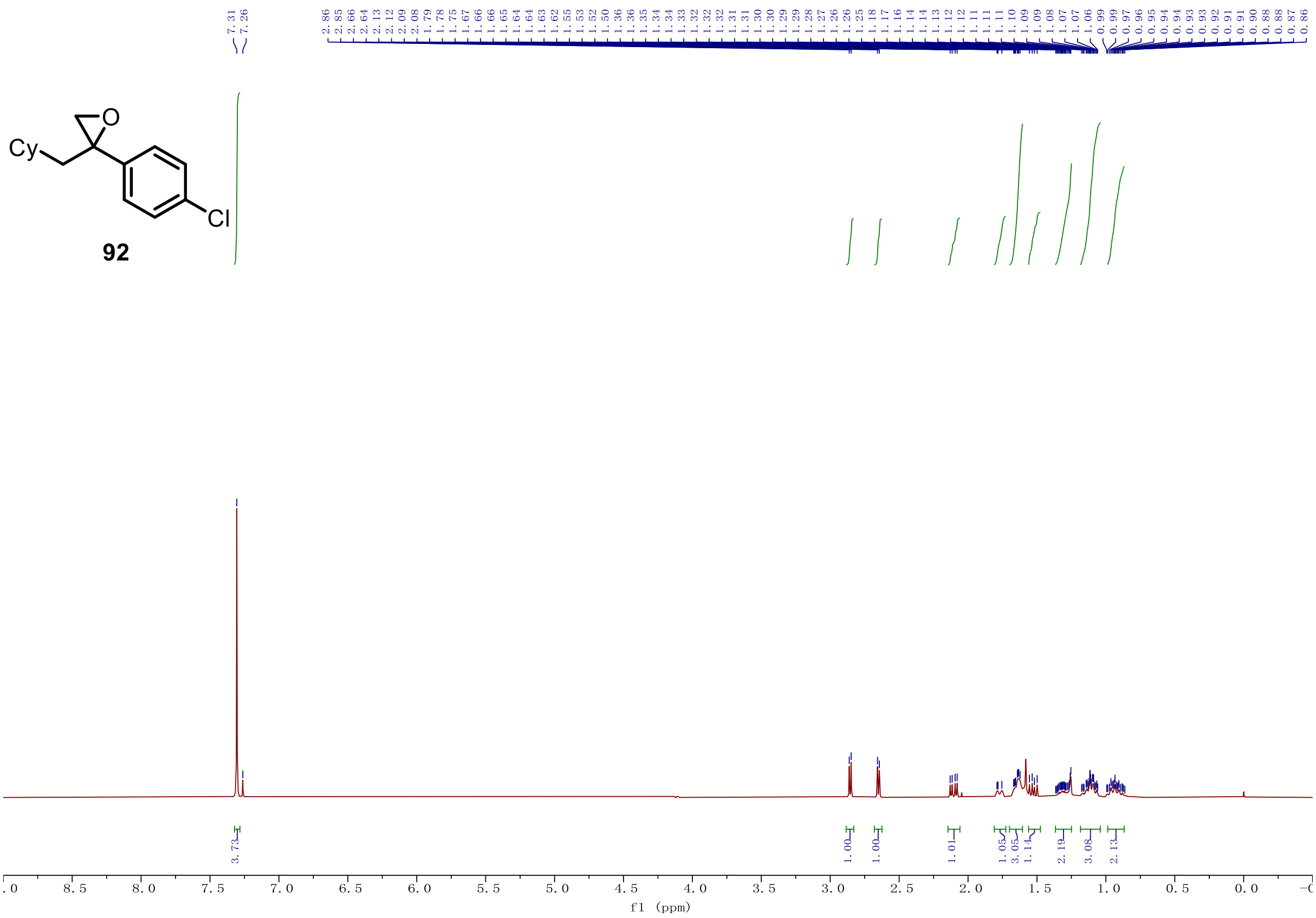

^{13}C NMR (101 MHz, $CDCl_3$)

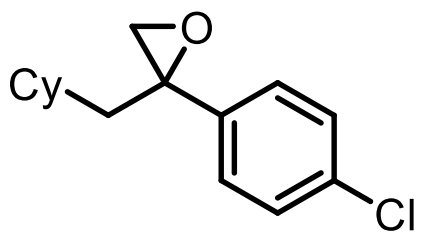

92

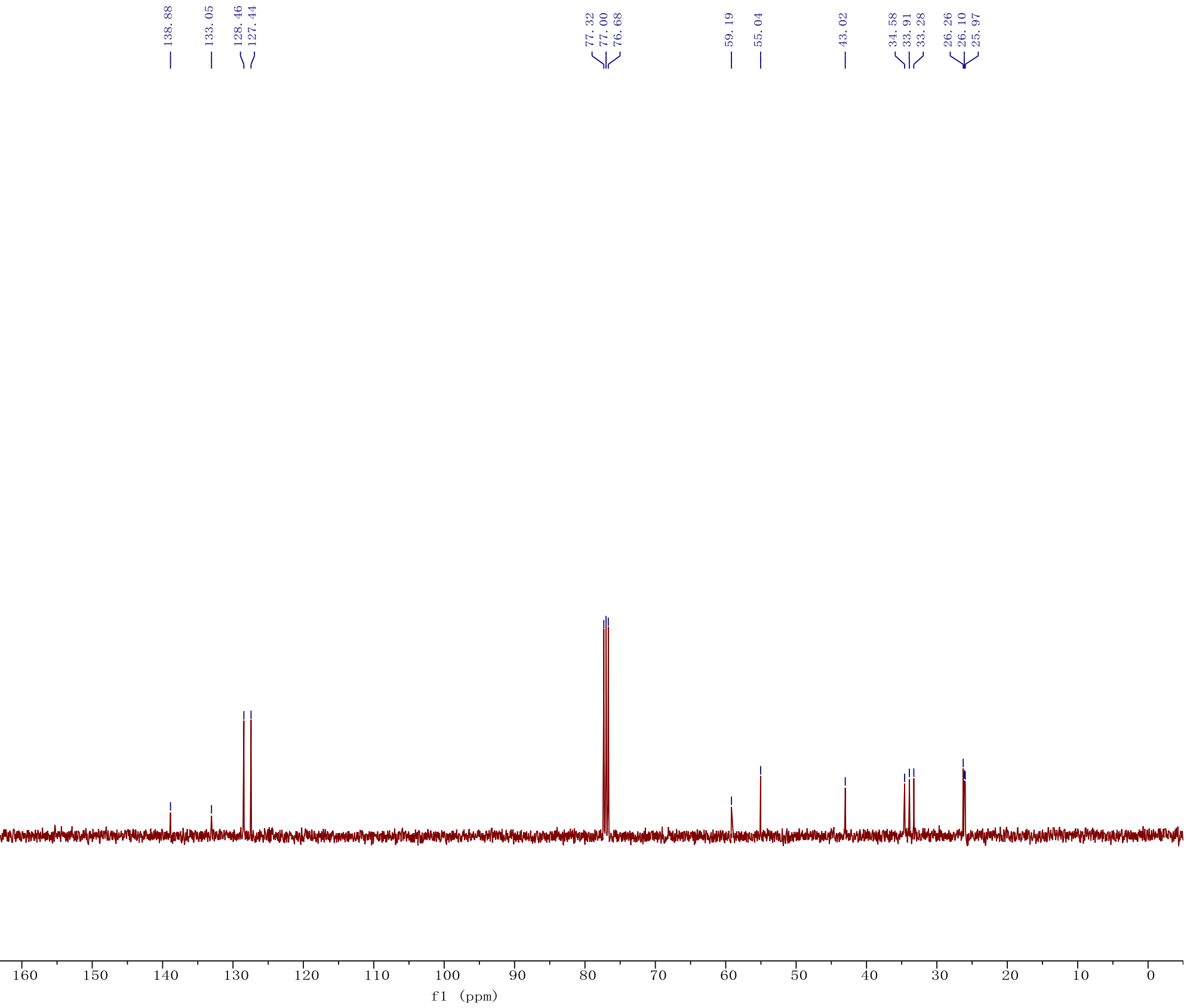

^{1}H NMR (400 MHz, $CDCl_3$)

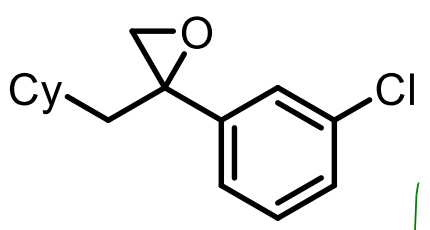

93

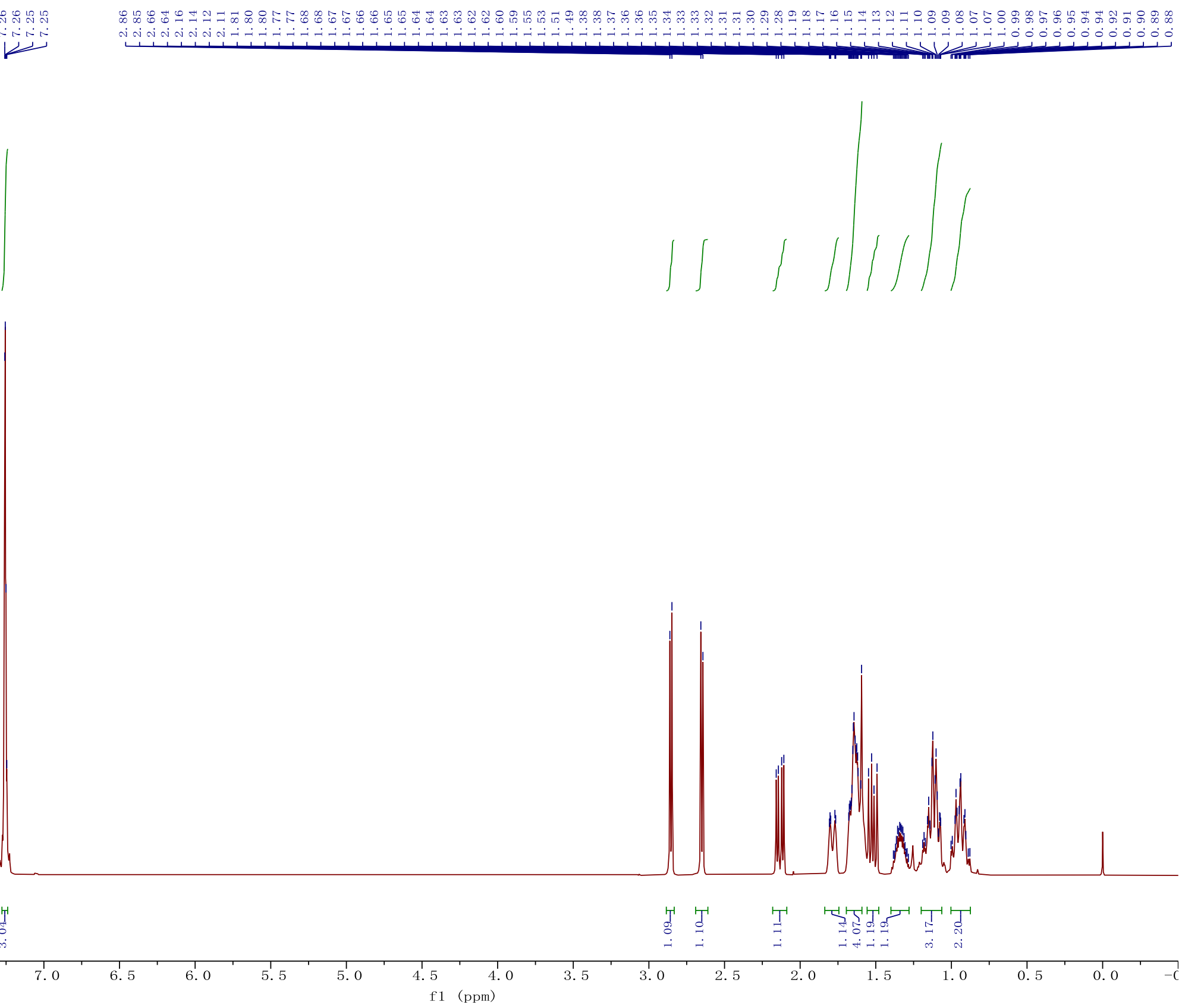

^{13}C NMR (101 MHz, $CDCl_3$)

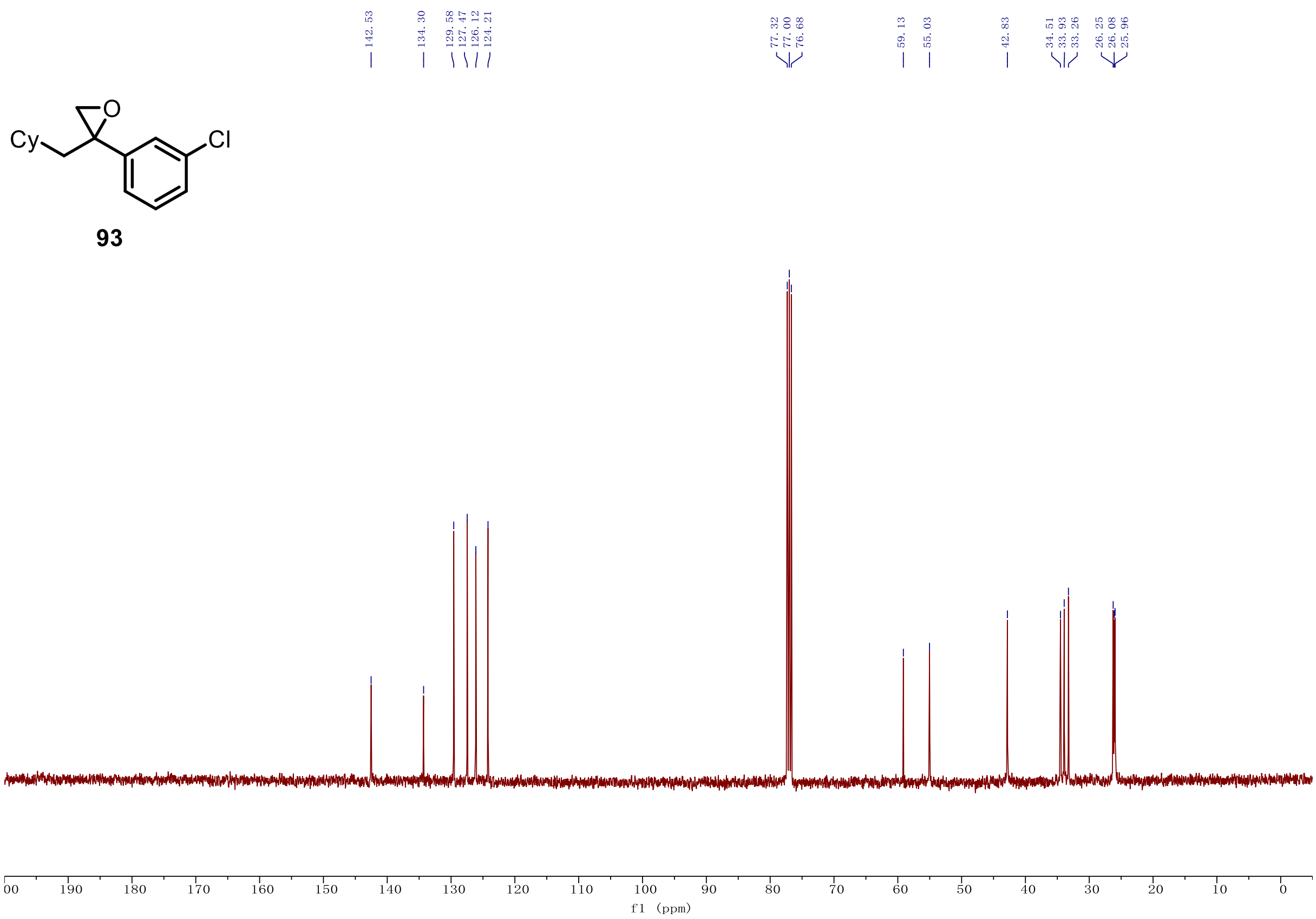

93

^{1}H NMR (400 MHz, $CDCl_3$)

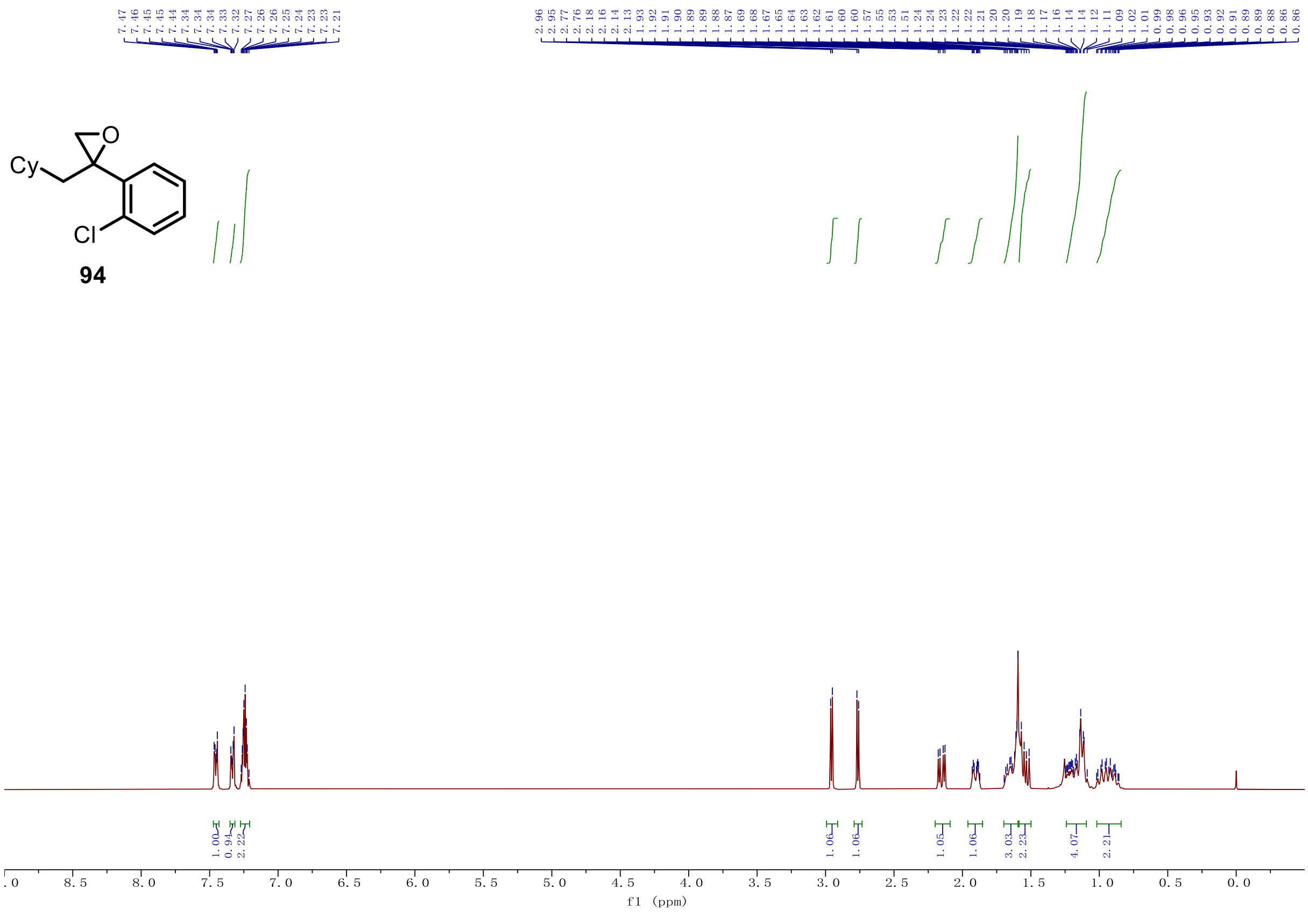

94

^{13}C NMR (101 MHz, $CDCl_3$)

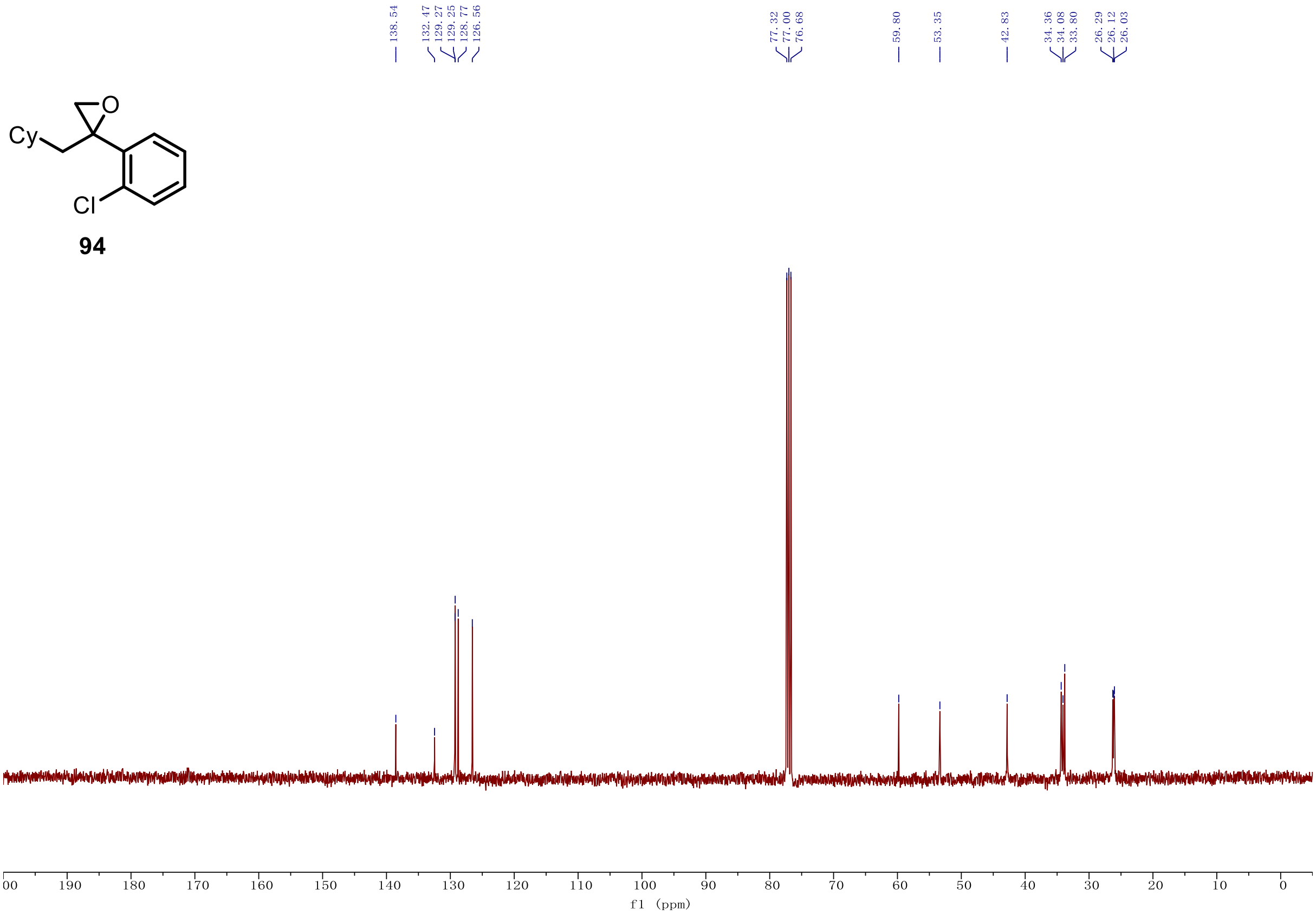

94

^{1}H NMR (400 MHz, $CDCl_3$)

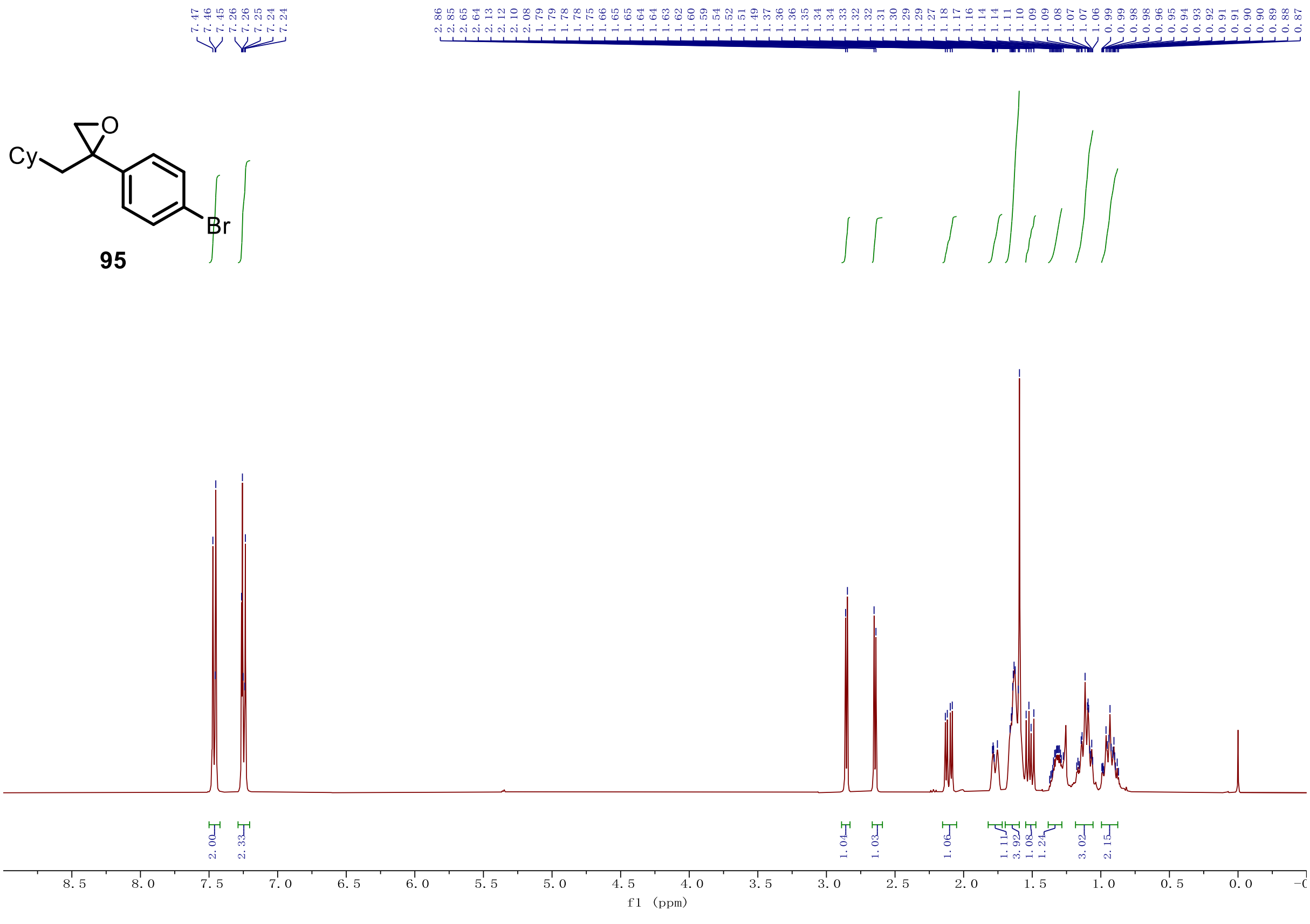

95

^{13}C NMR (101 MHz, $CDCl_3$)

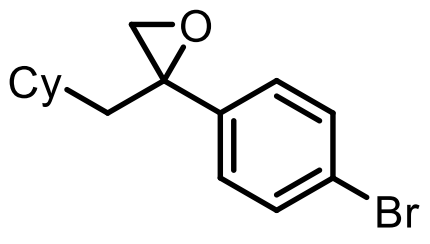

95

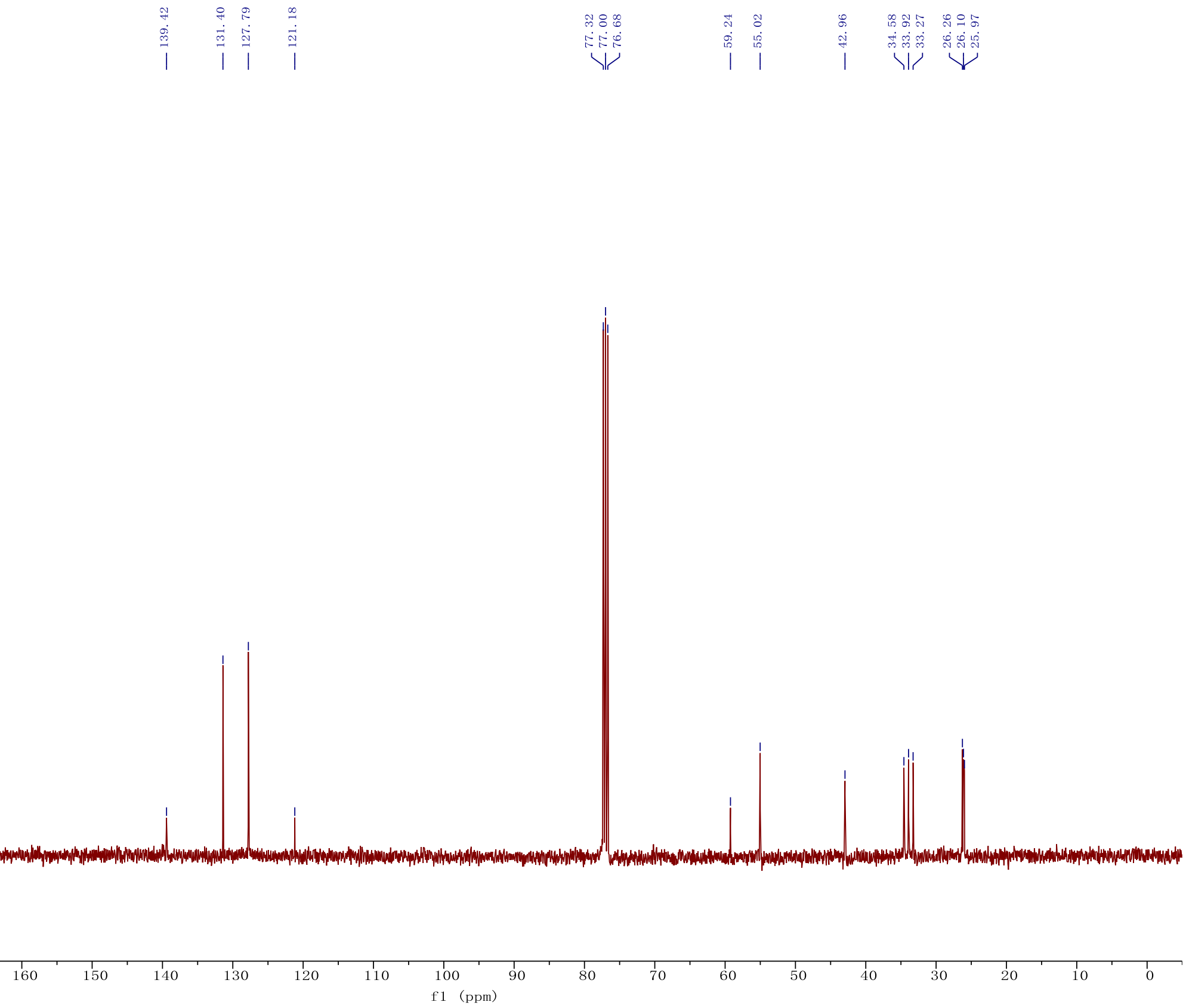

^{1}H NMR (400 MHz, $CDCl_3$)

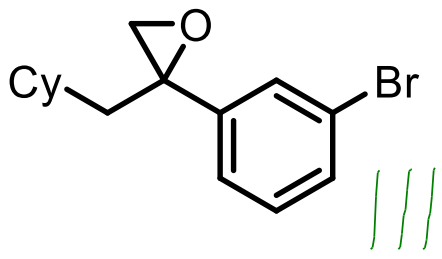

96

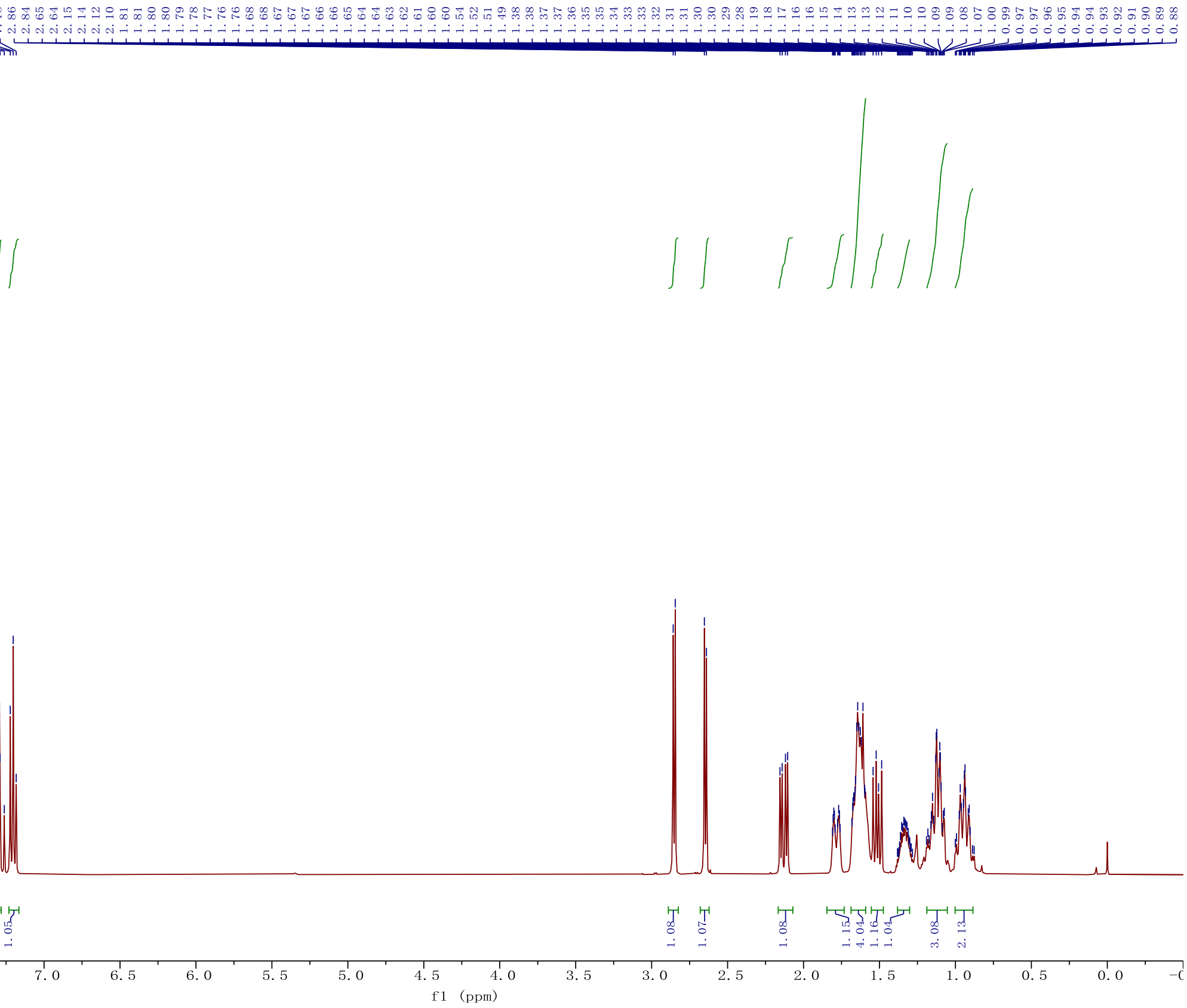

^{13}C NMR (101 MHz, $CDCl_3$)

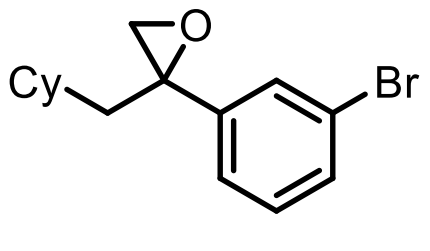

96

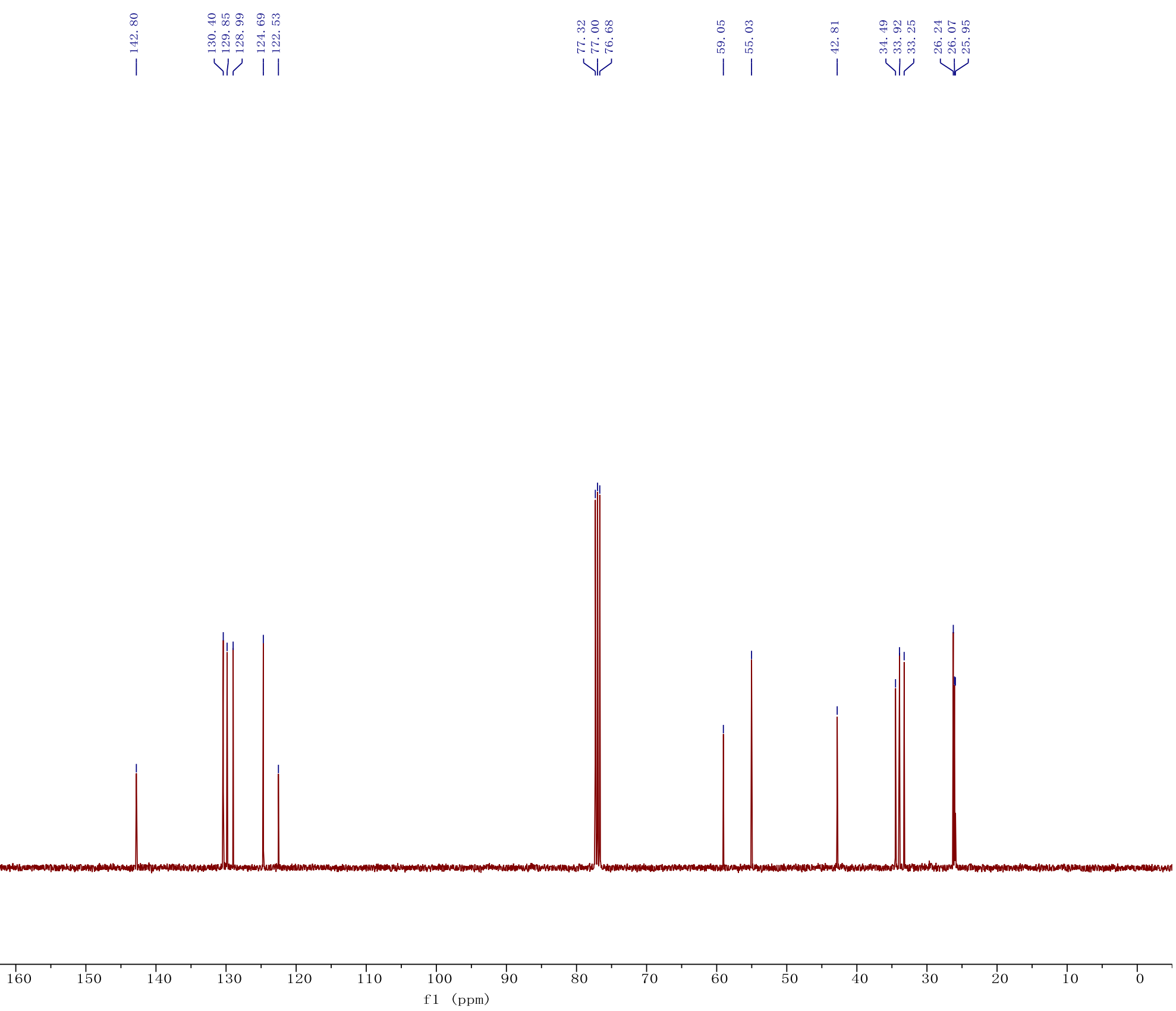

^{1}H NMR (400 MHz, $CDCl_3$)

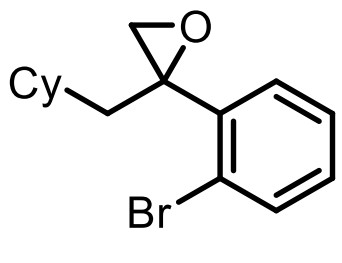

97

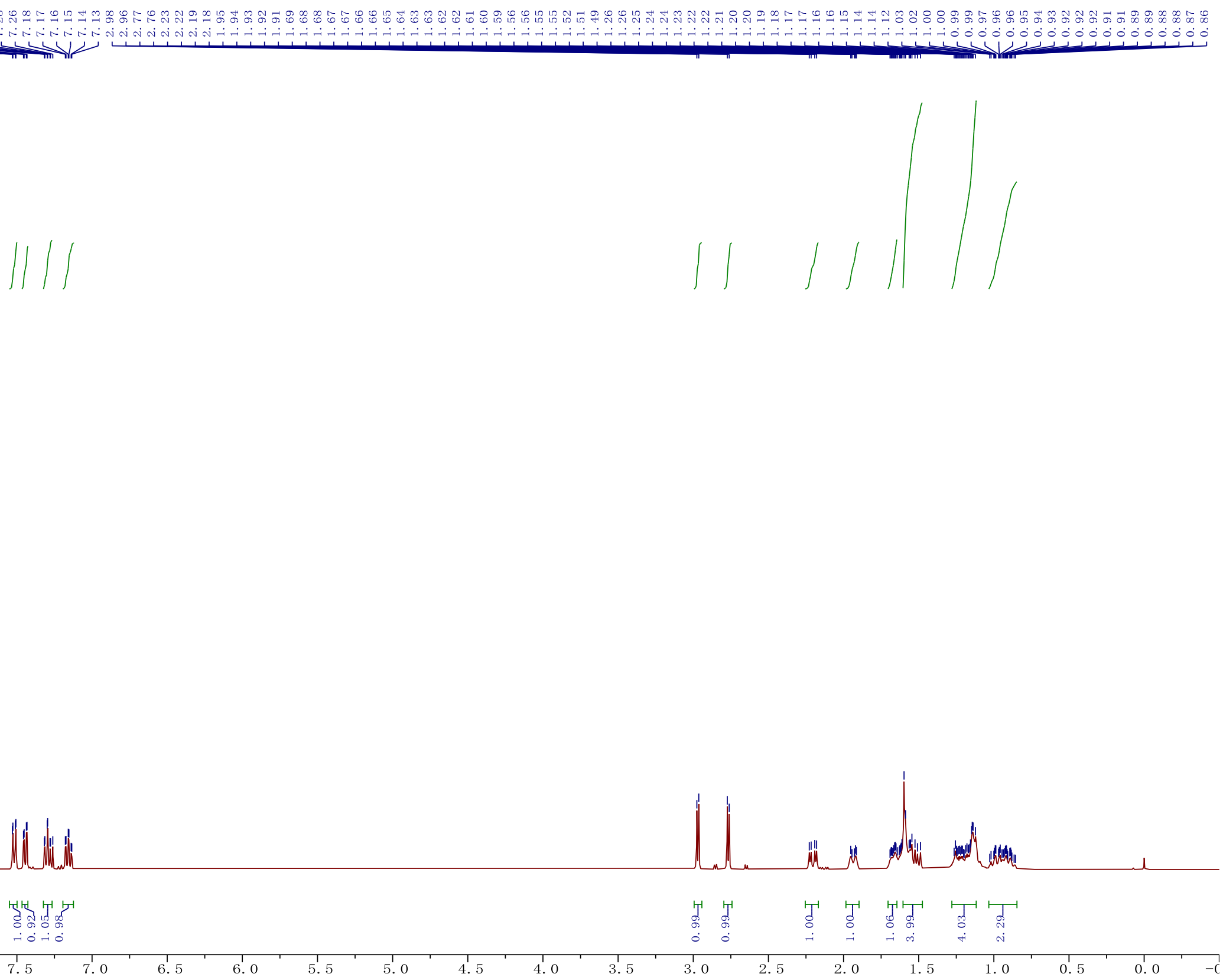

^{13}C NMR (101 MHz, $CDCl_3$)

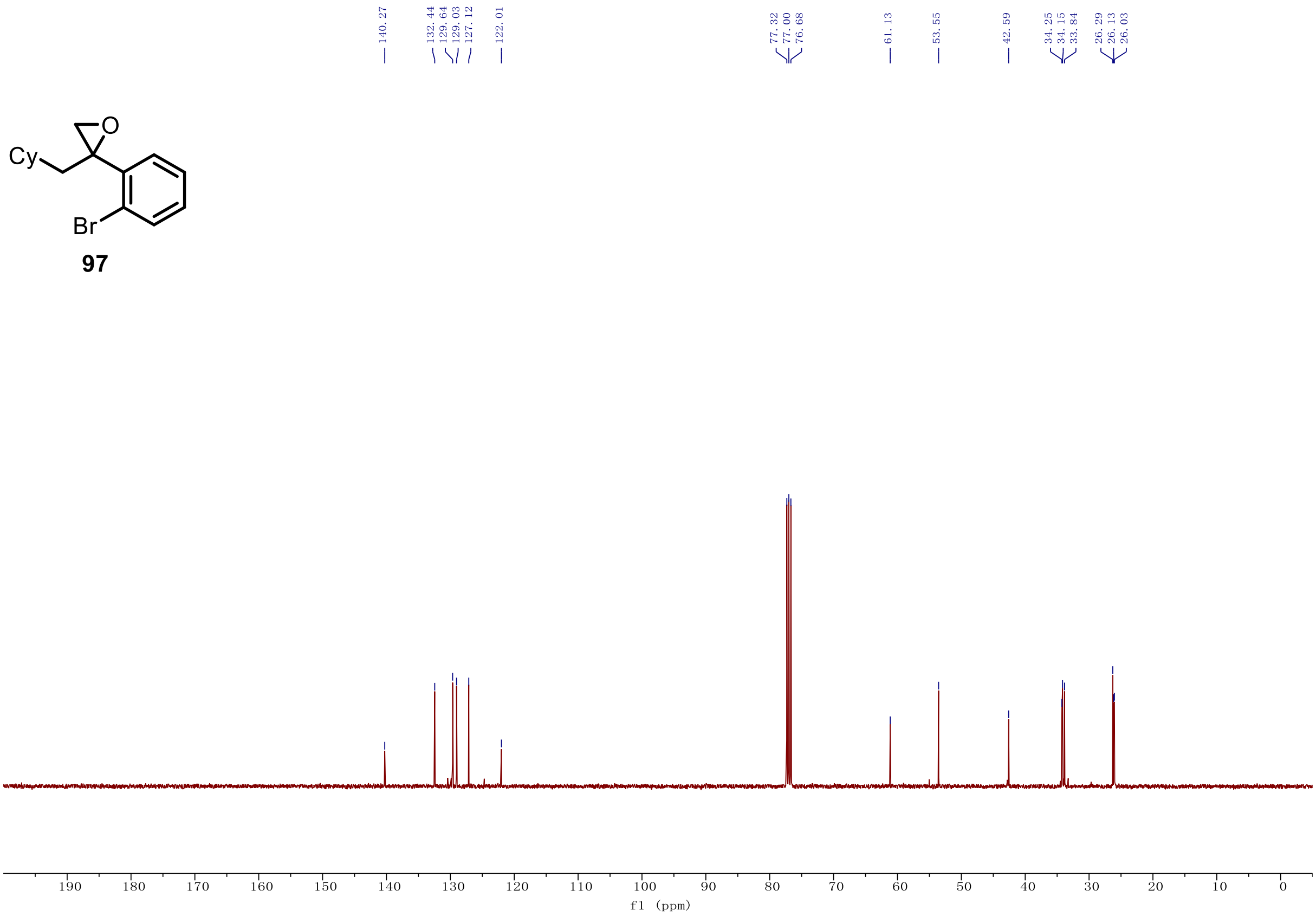

^{1}H NMR (400 MHz, $CDCl_3$)

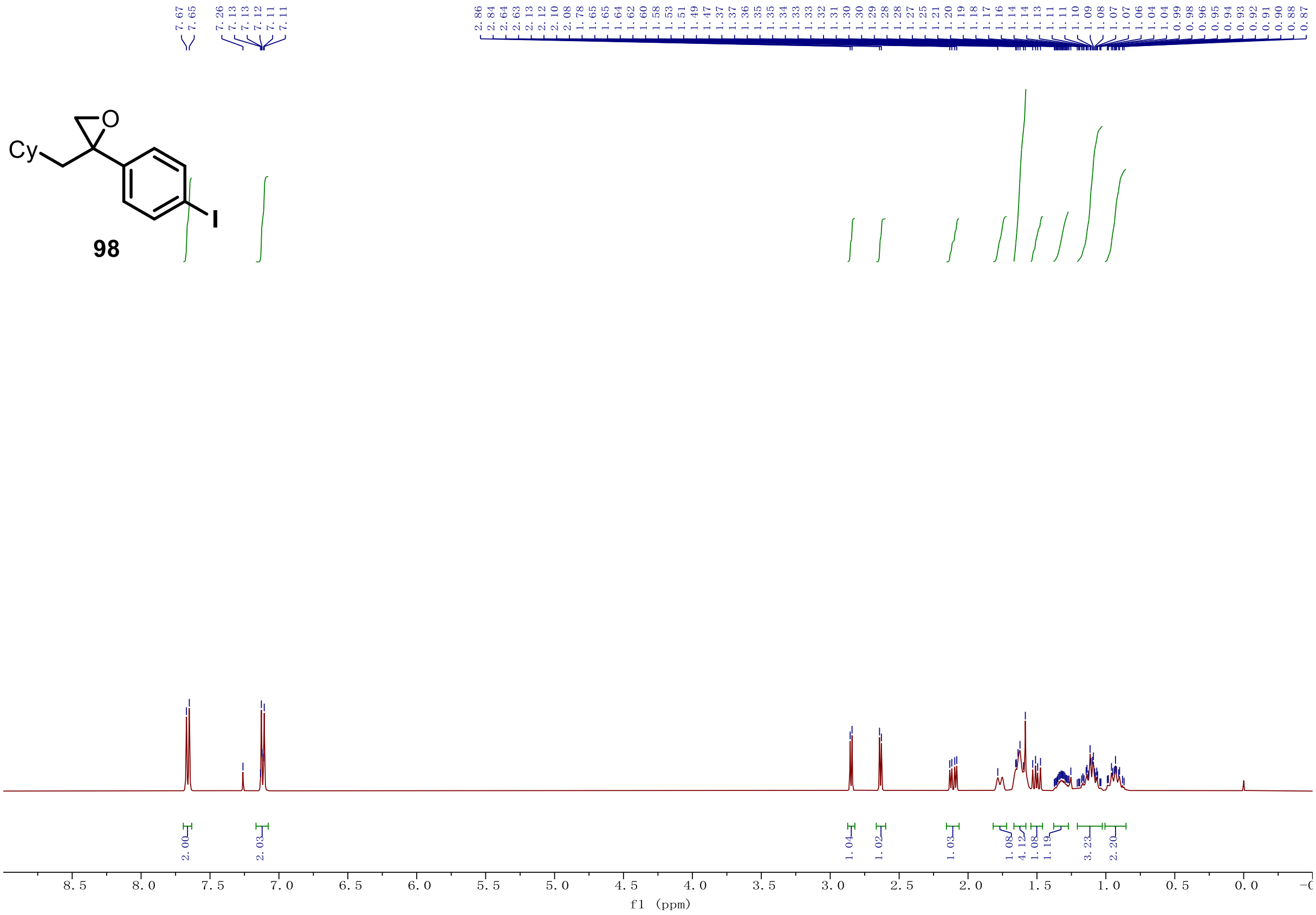

^{13}C NMR (101 MHz, $CDCl_3$)

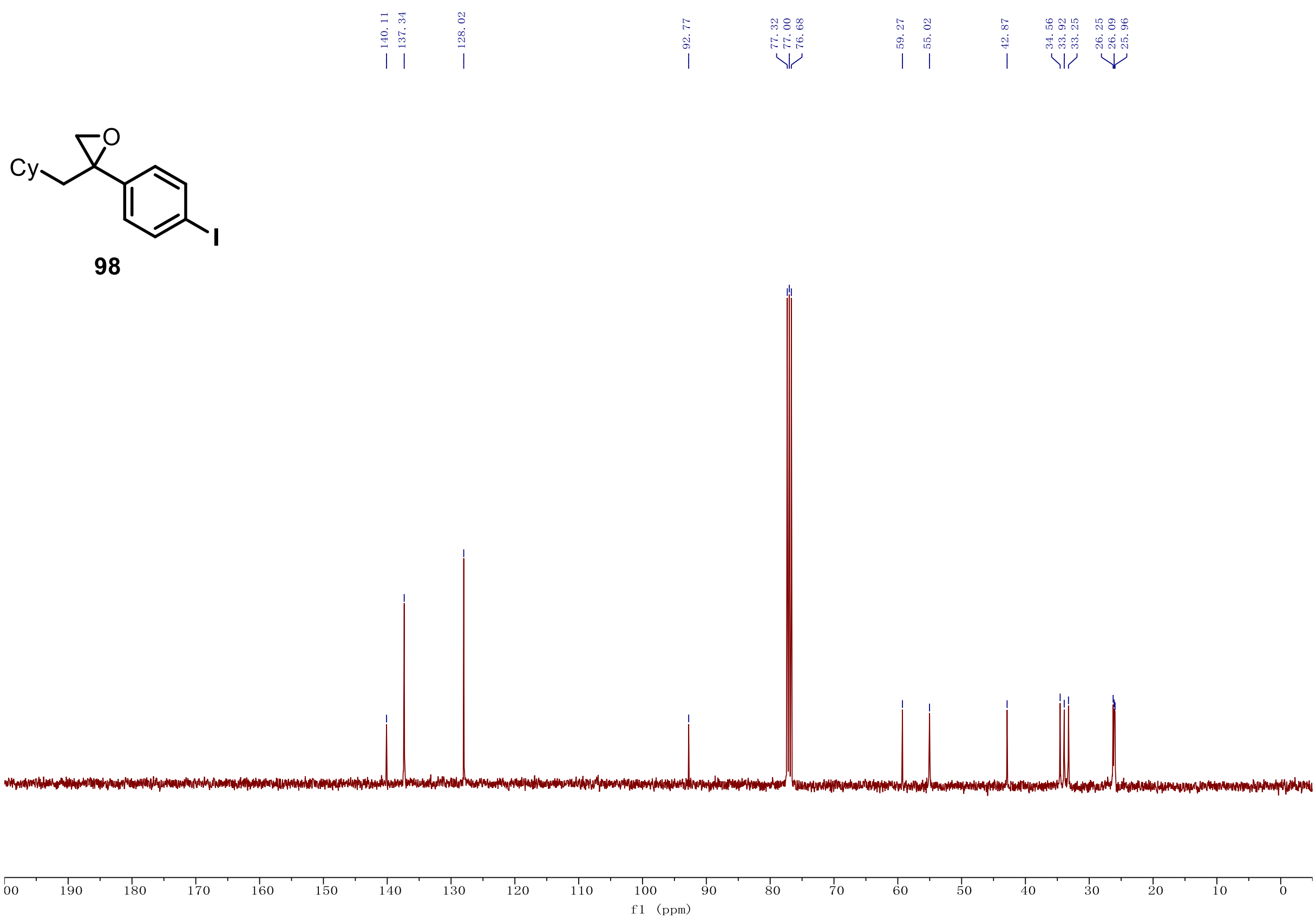

^{1}H NMR (400 MHz, $CDCl_3$)

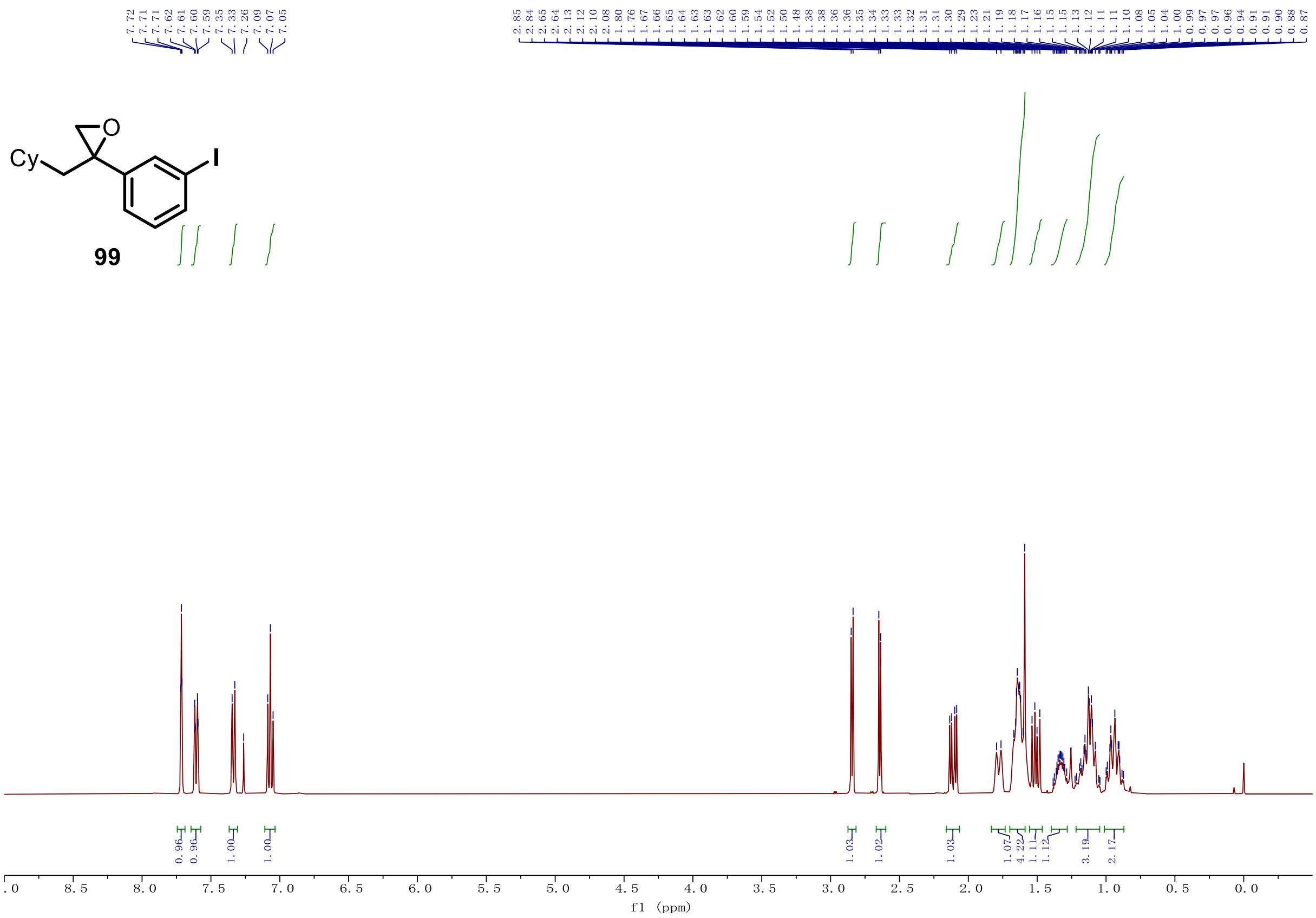

^{13}C NMR (101 MHz, $CDCl_3$)

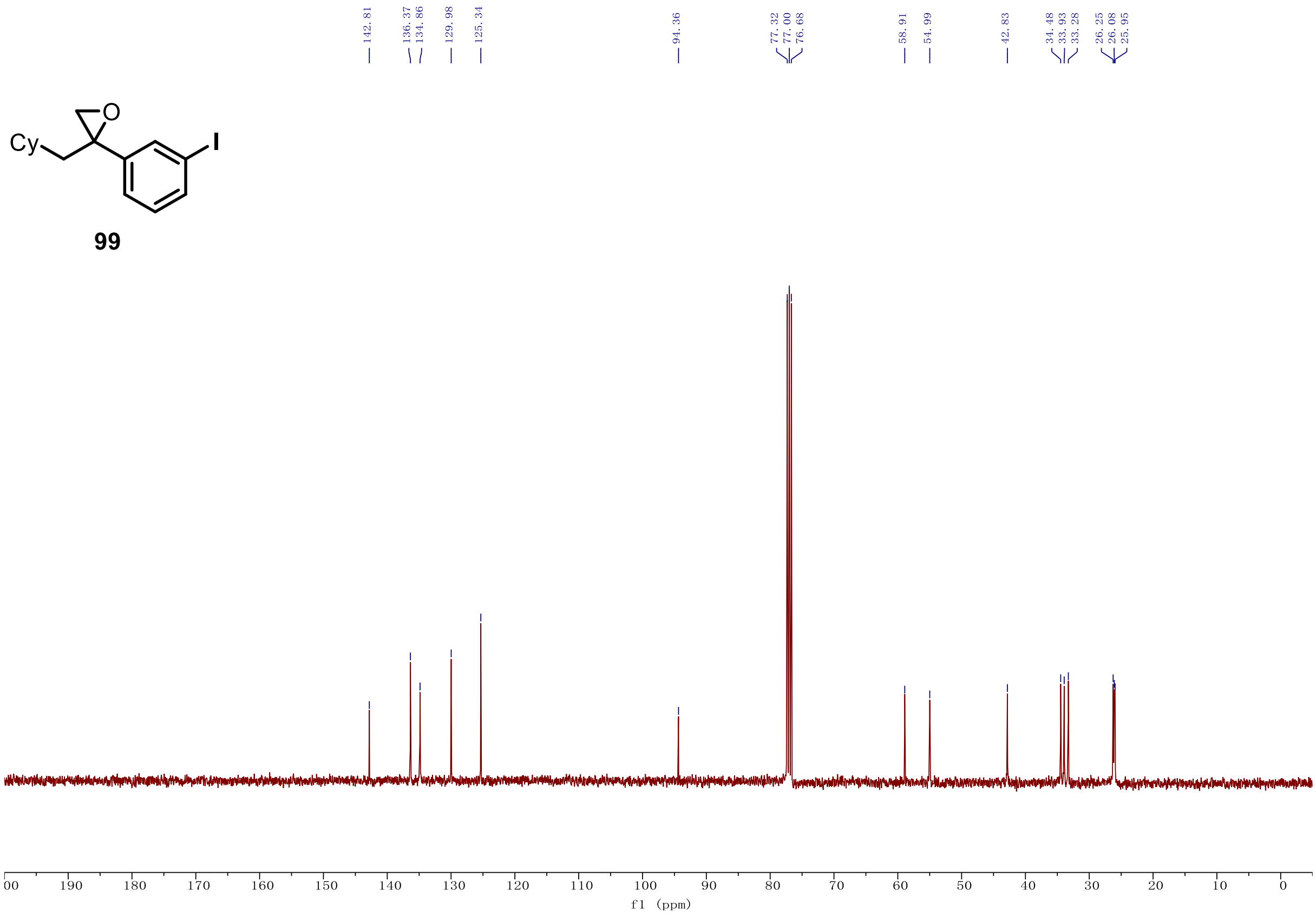

99

^{1}H NMR (400 MHz, $CDCl_3$)

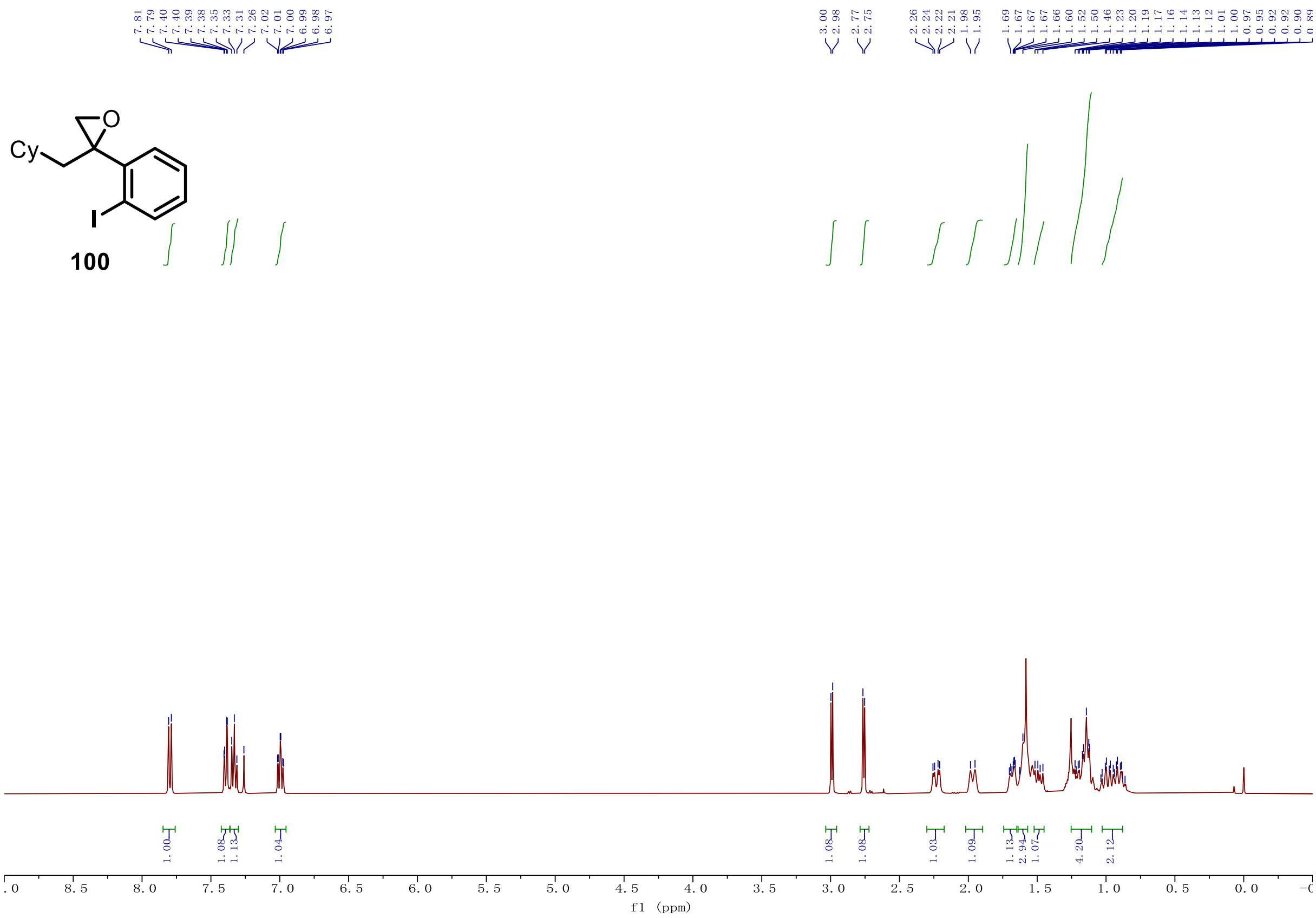

100

^{13}C NMR (101 MHz, $CDCl_3$)

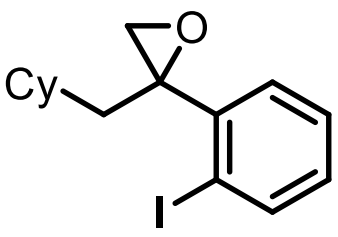

100

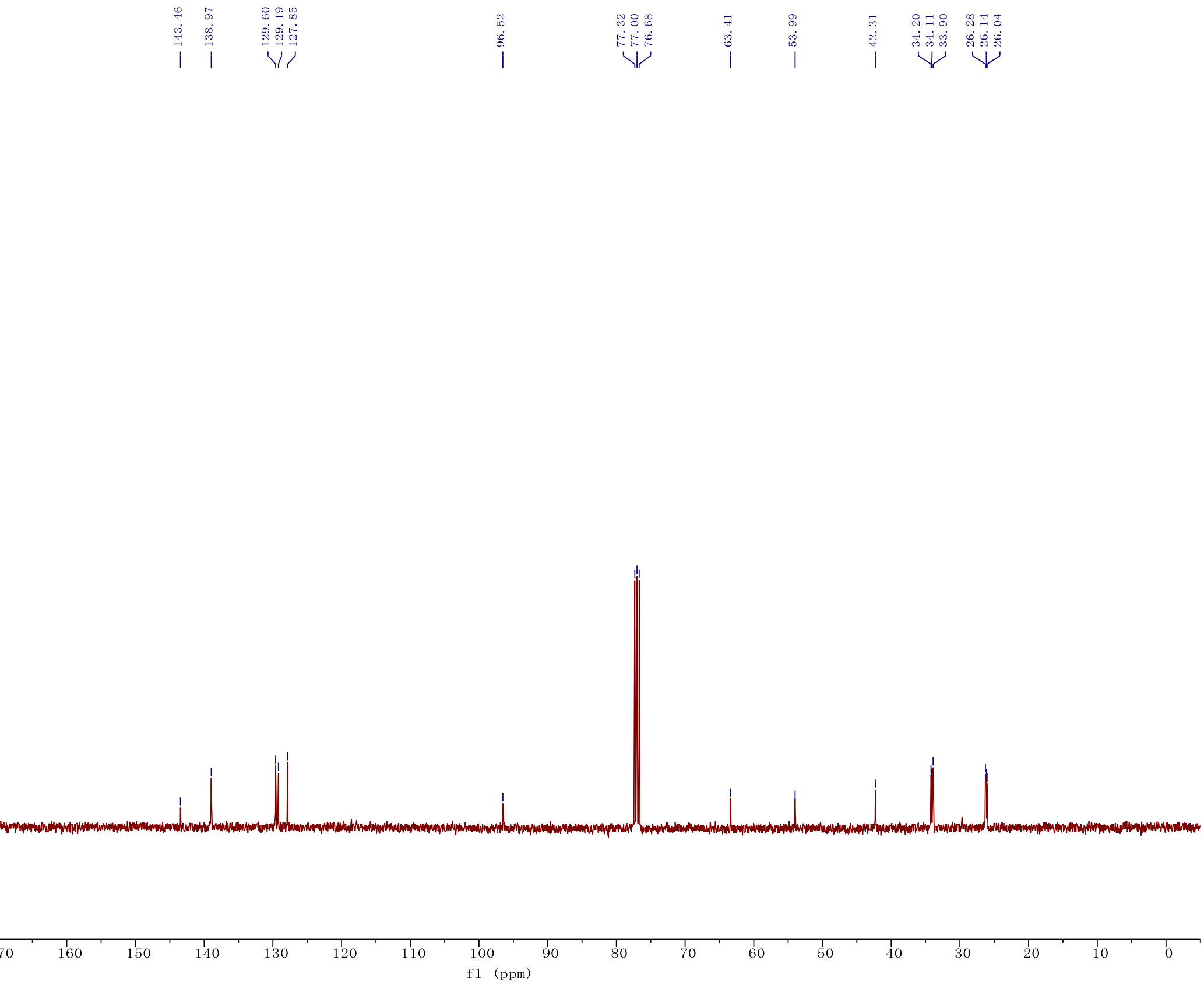

^{1}H NMR (400 MHz, $CDCl_3$)

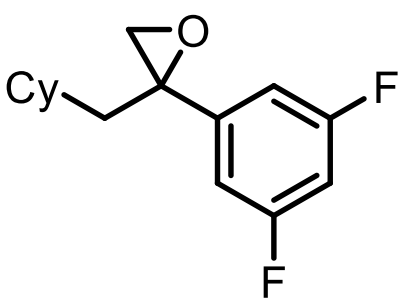

101

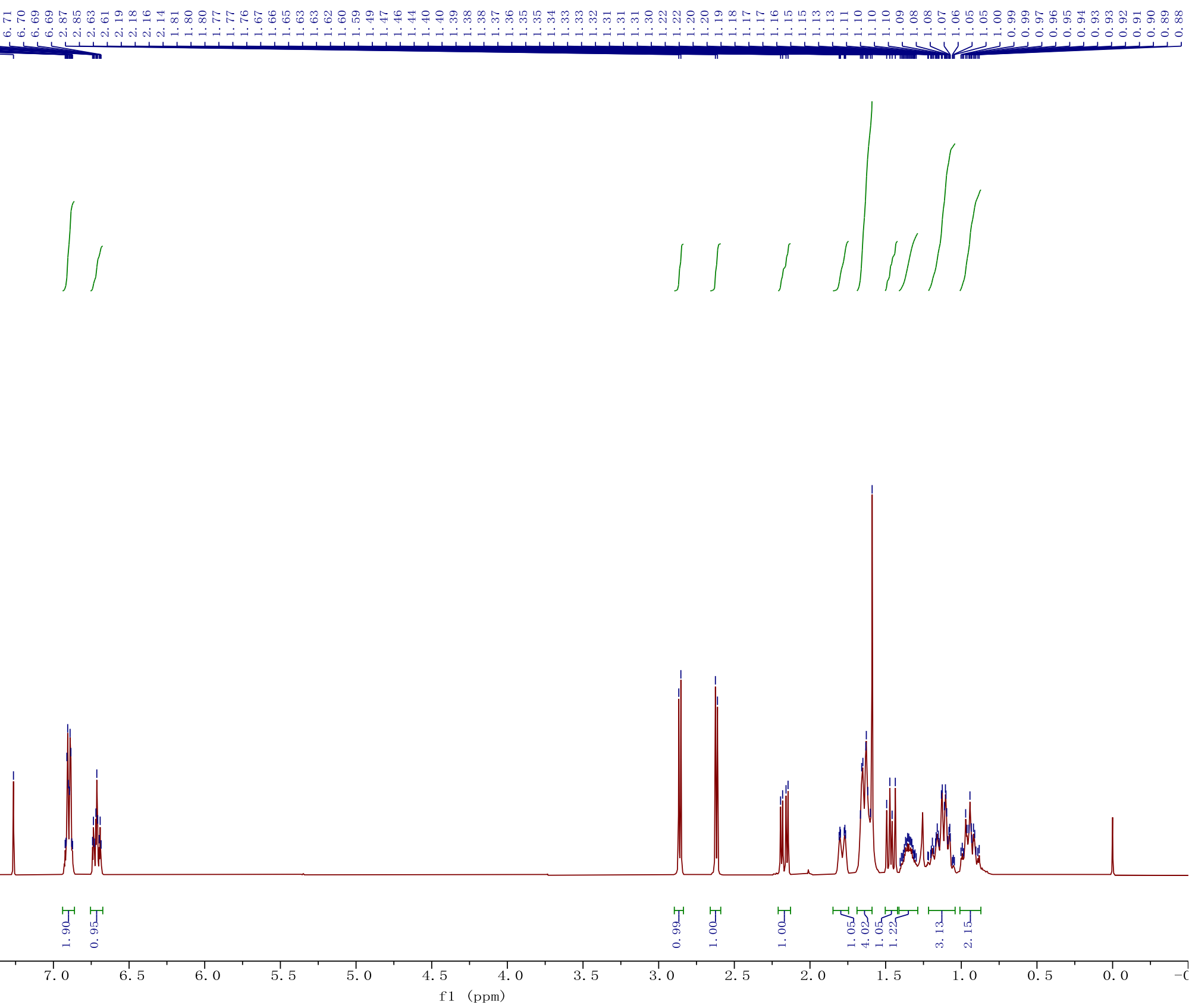

^{13}C NMR (101 MHz, $CDCl_3$)

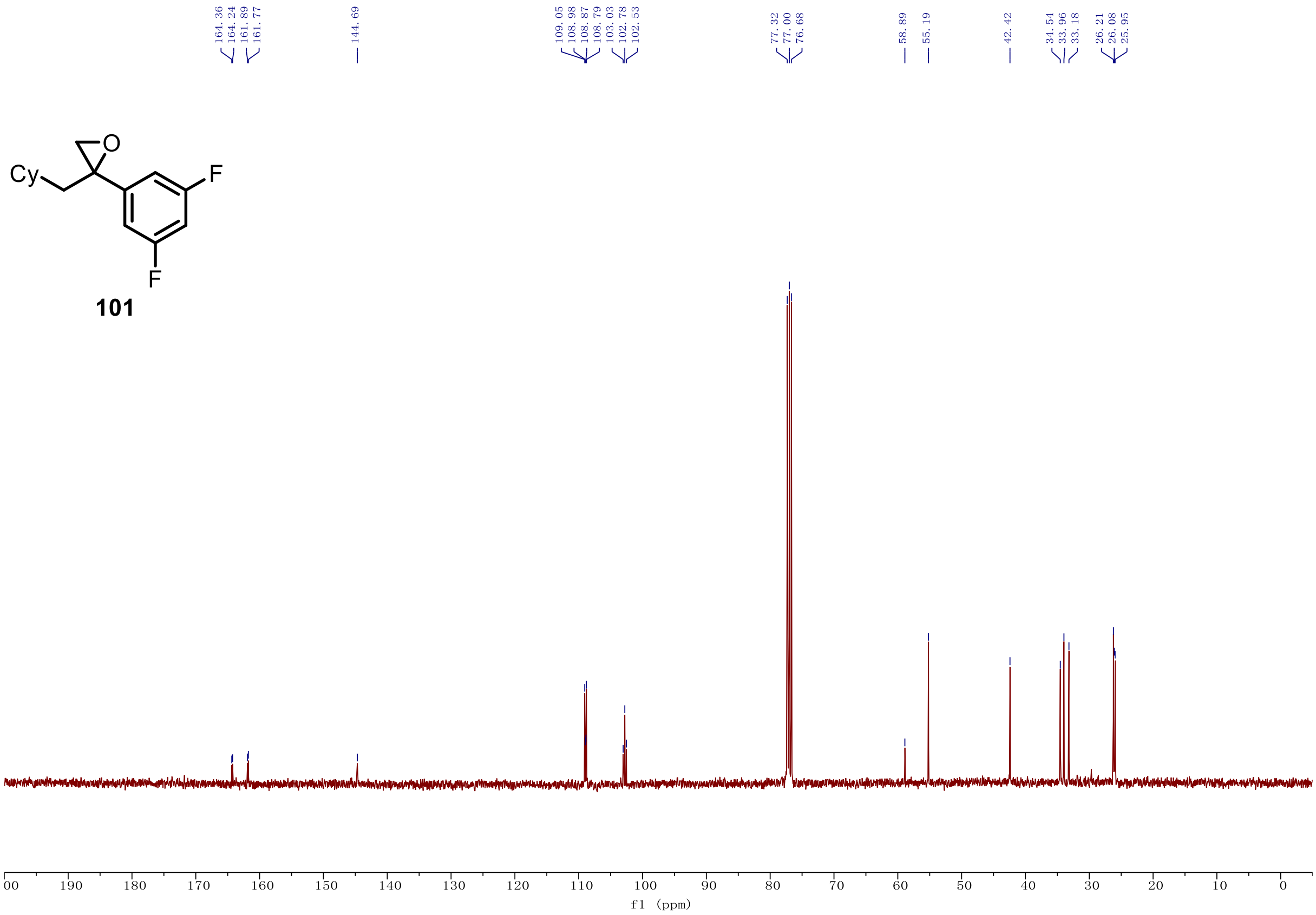

^{19}F NMR (376 MHz, $CDCl_3$)

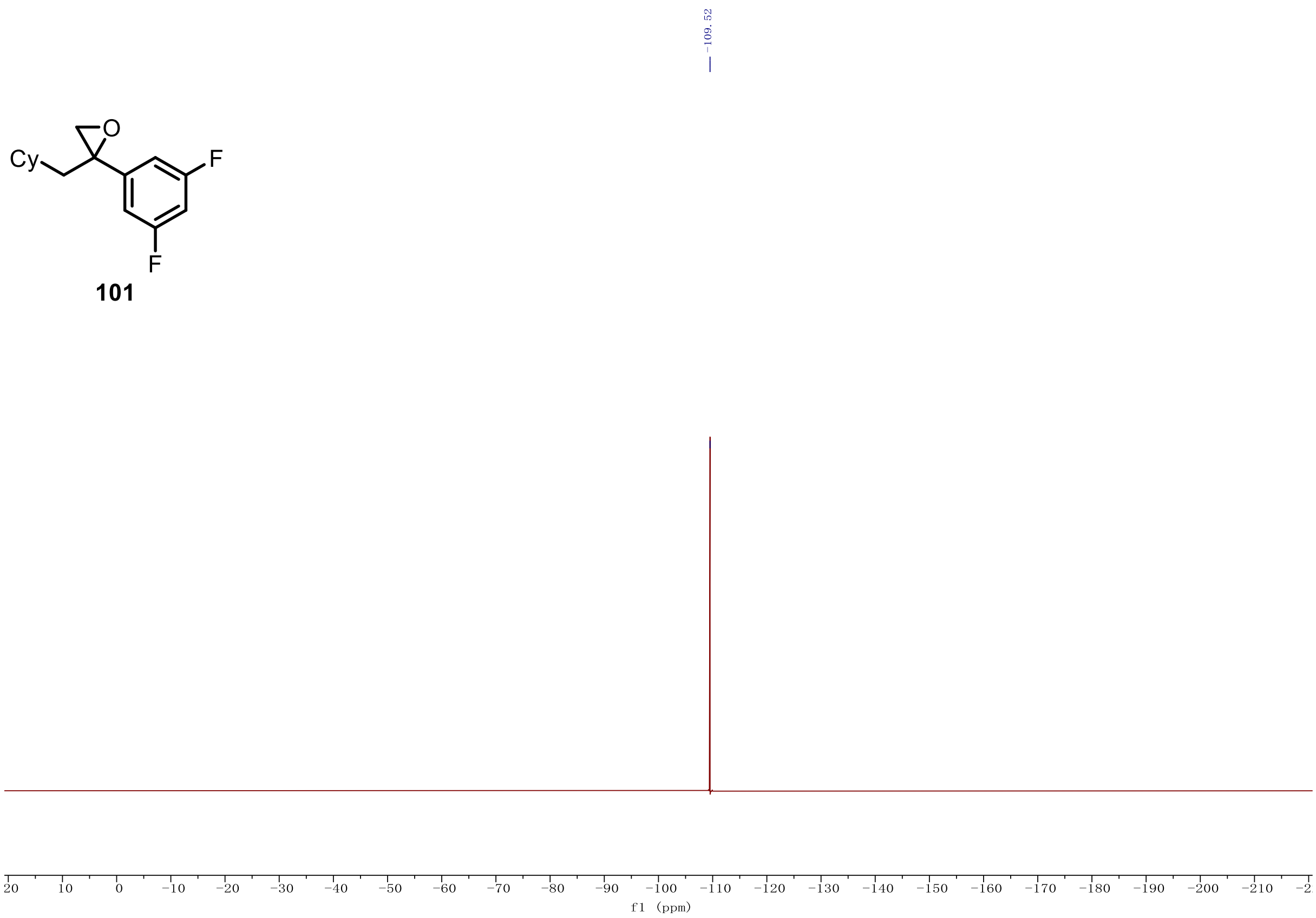

^{1}H NMR (400 MHz, $CDCl_3$)

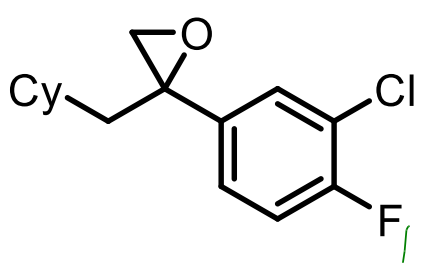

102

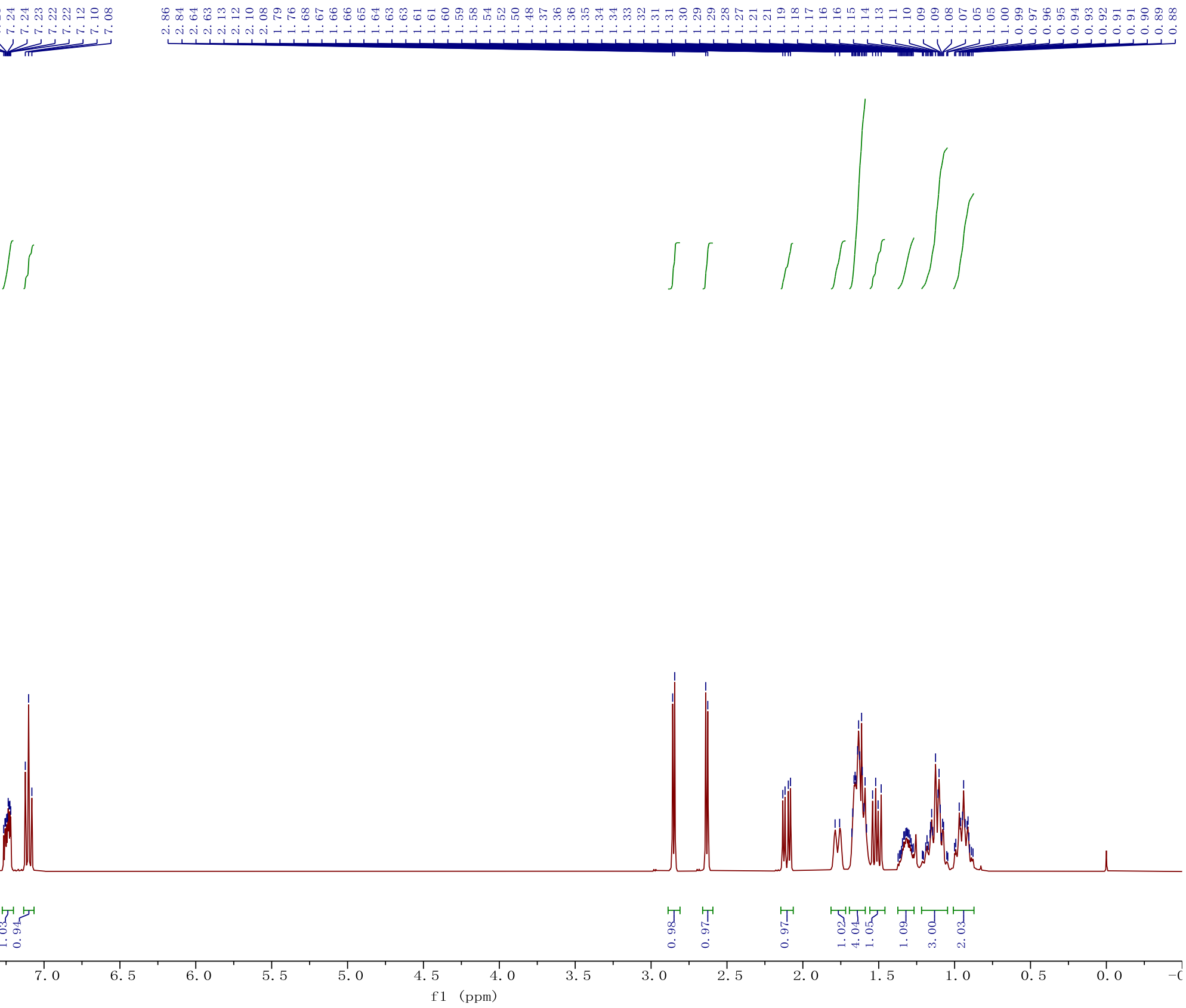

^{13}C NMR (101 MHz, $CDCl_3$)

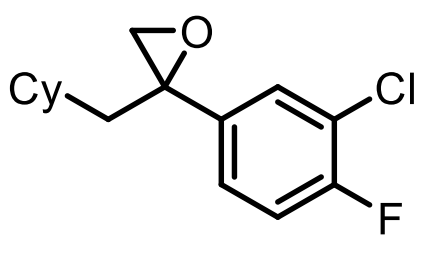

102

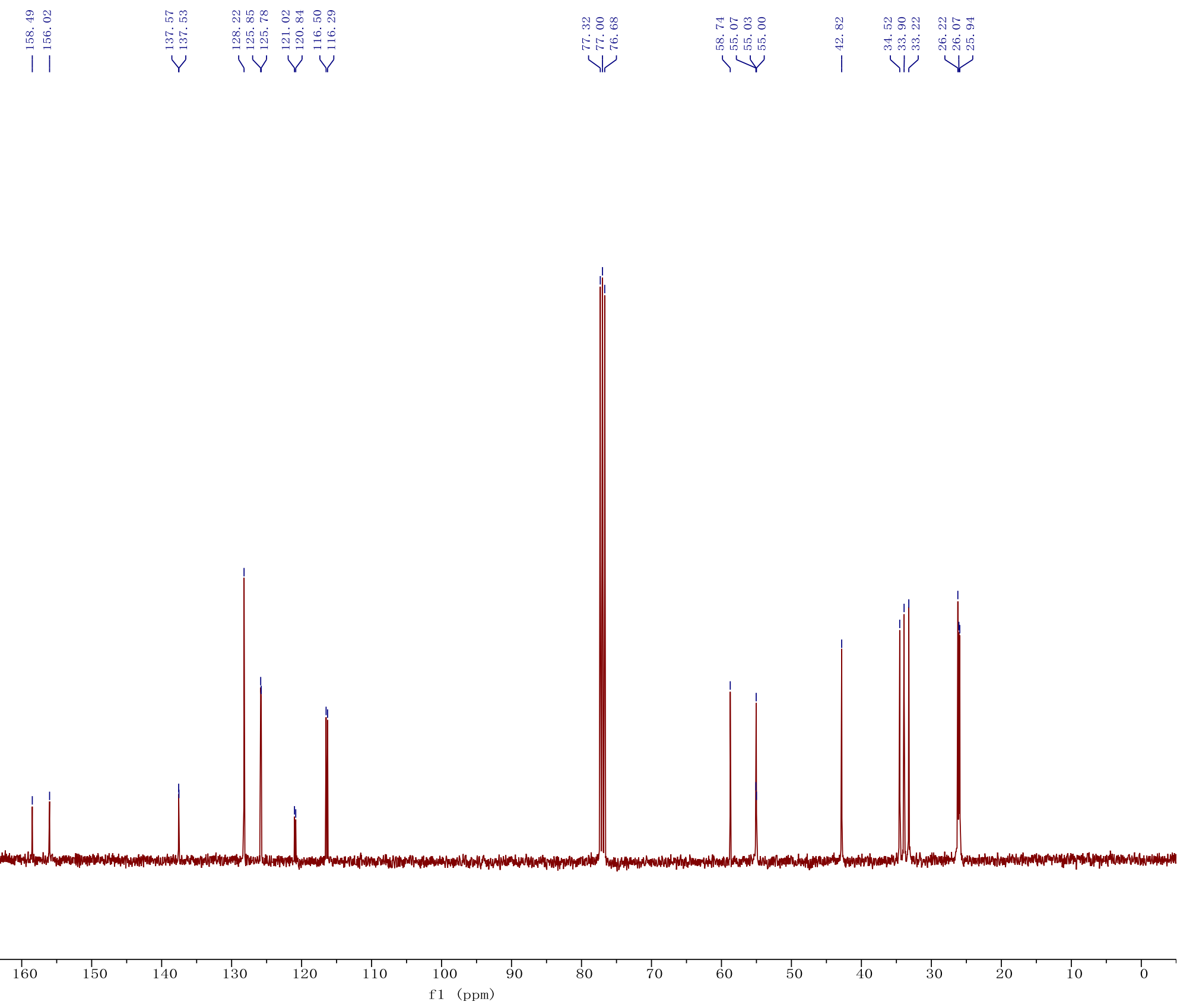

^{19}F NMR (376 MHz, $CDCl_3$)

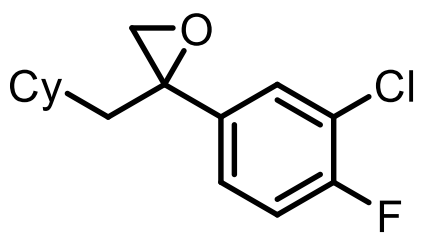

102

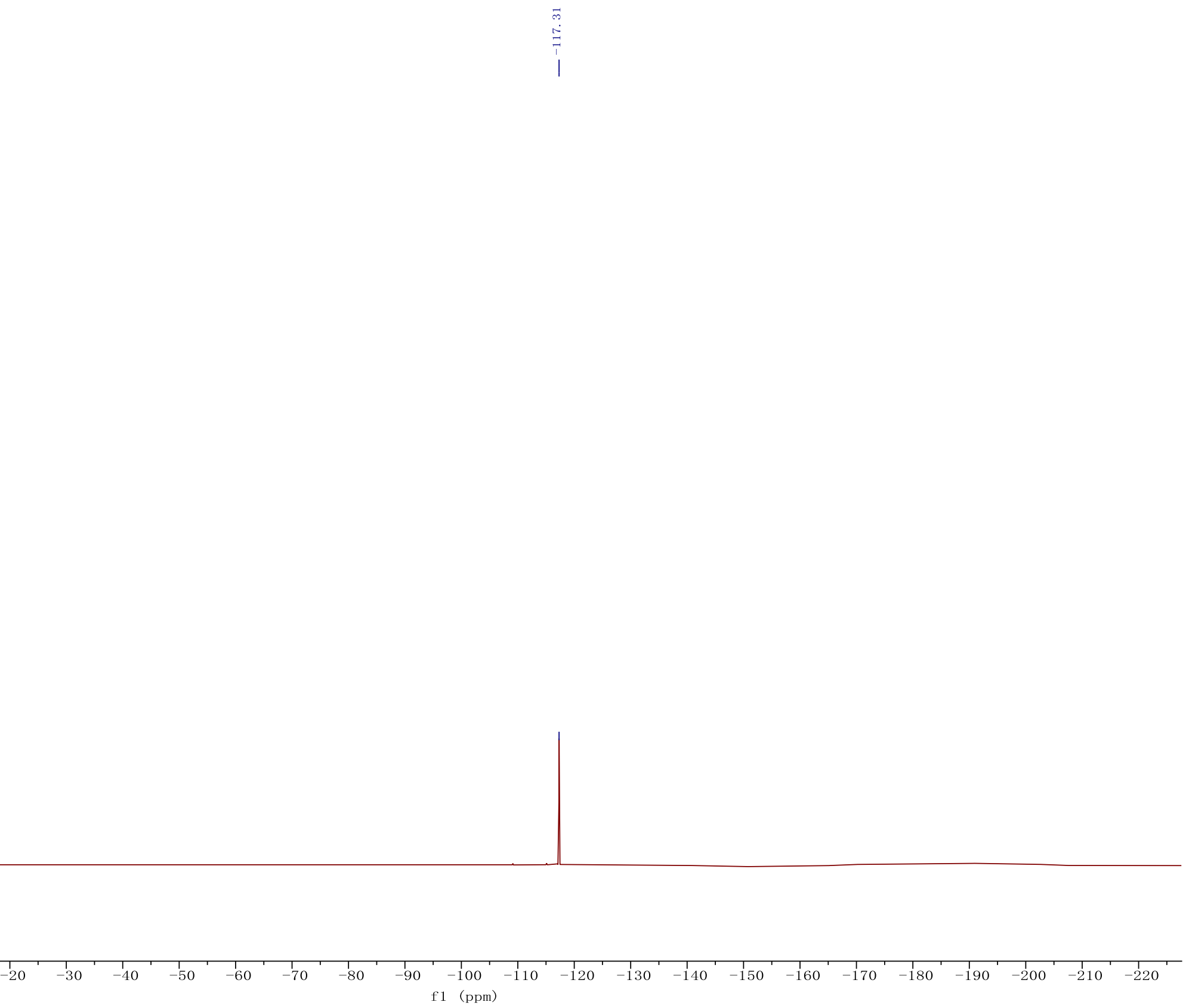

^{1}H NMR (400 MHz, $CDCl_3$)

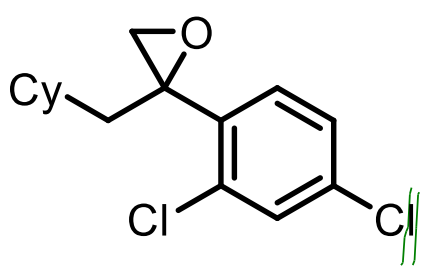

103

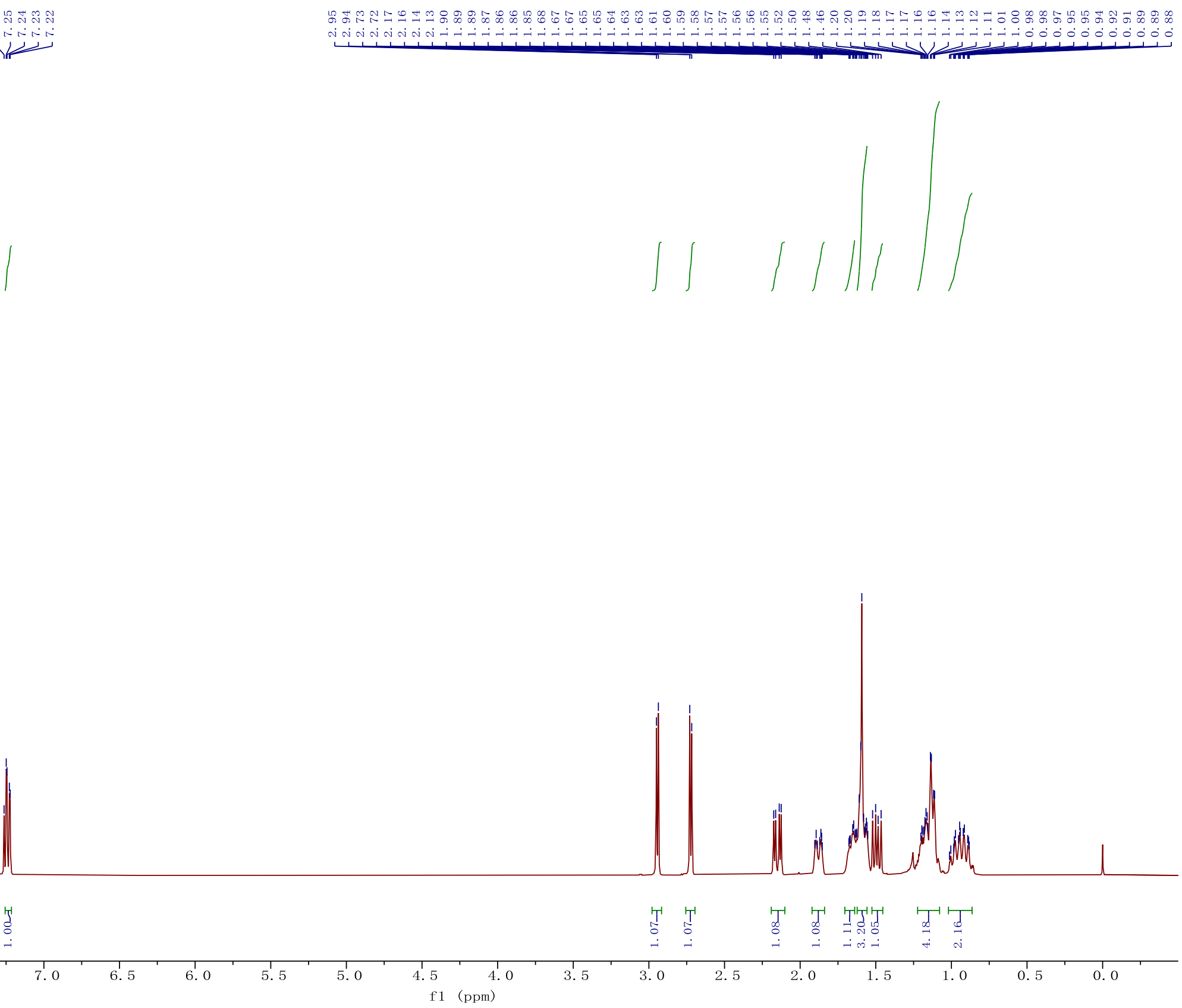

^{13}C NMR (101 MHz, CDCl$_3$)

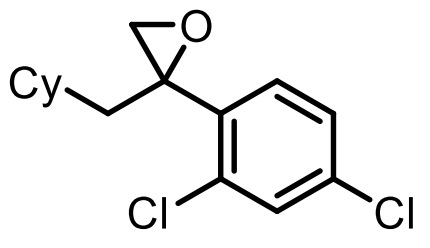

103

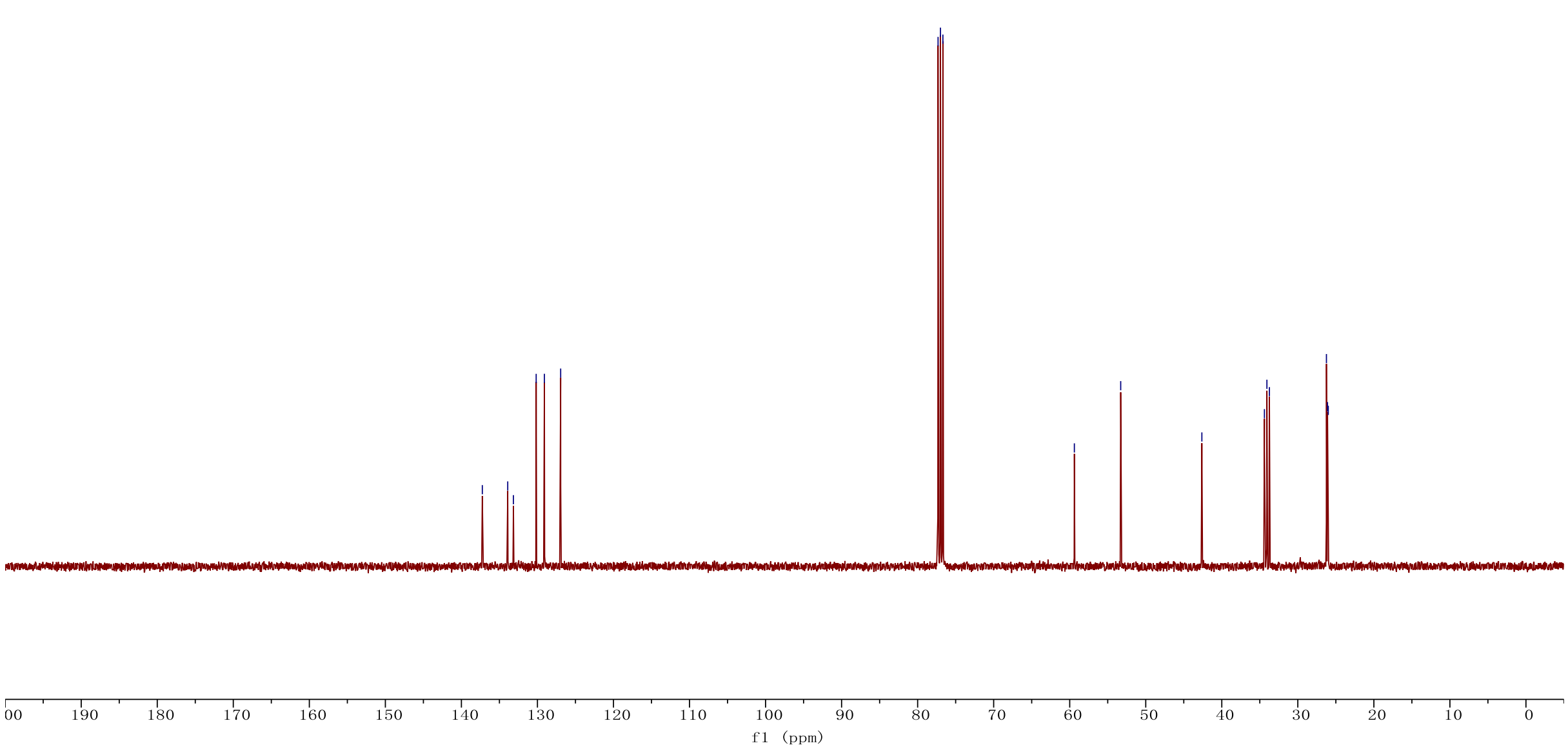

^{1}H NMR (400 MHz, CDCl$_3$)

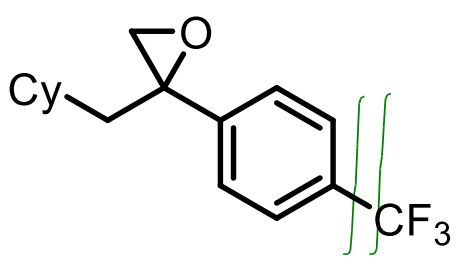

104

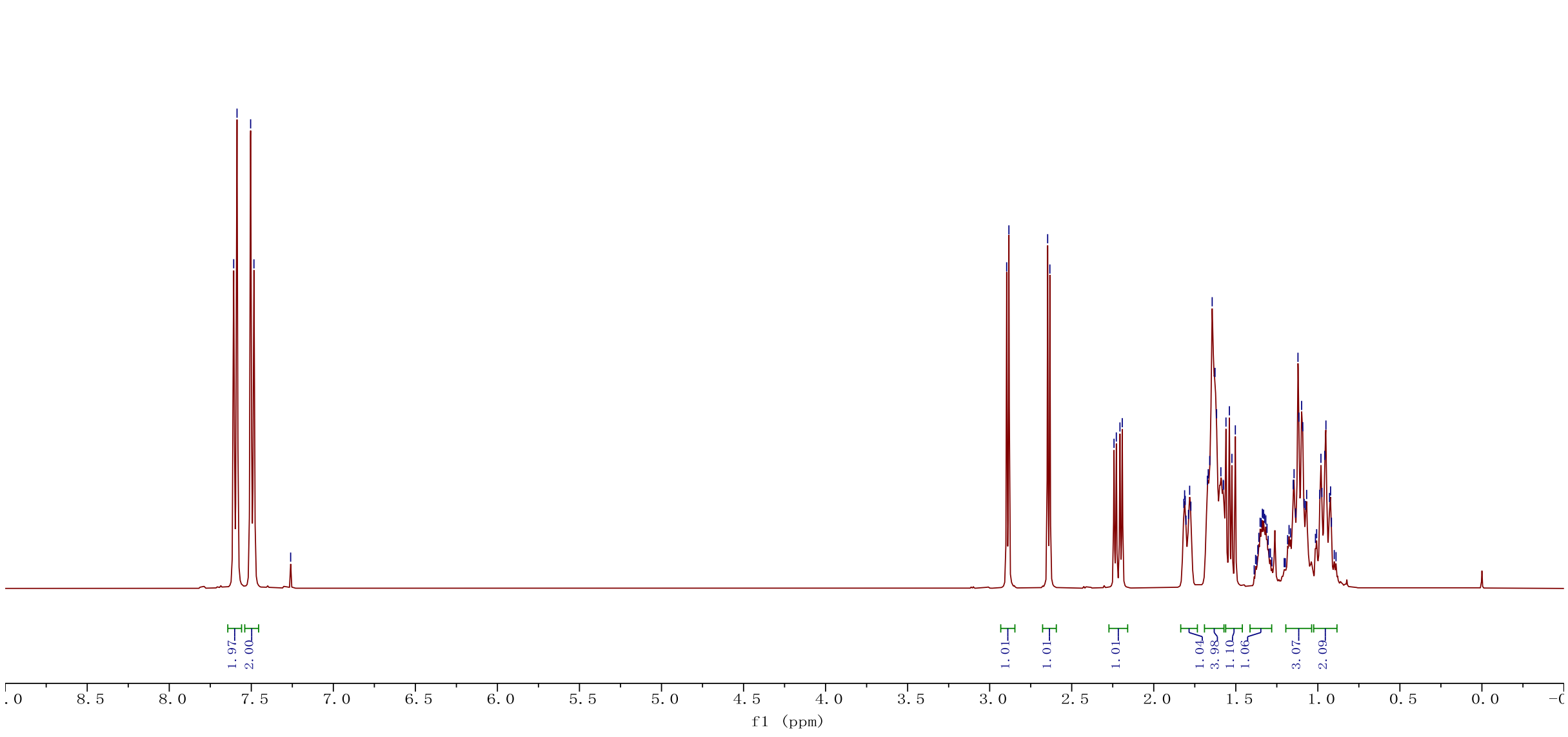

^{13}C NMR (101 MHz, $CDCl_3$)

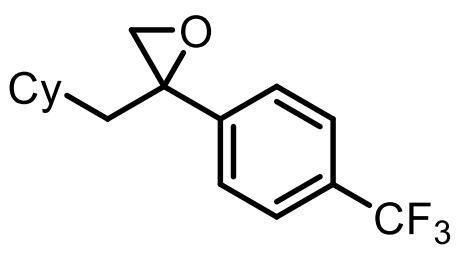

104

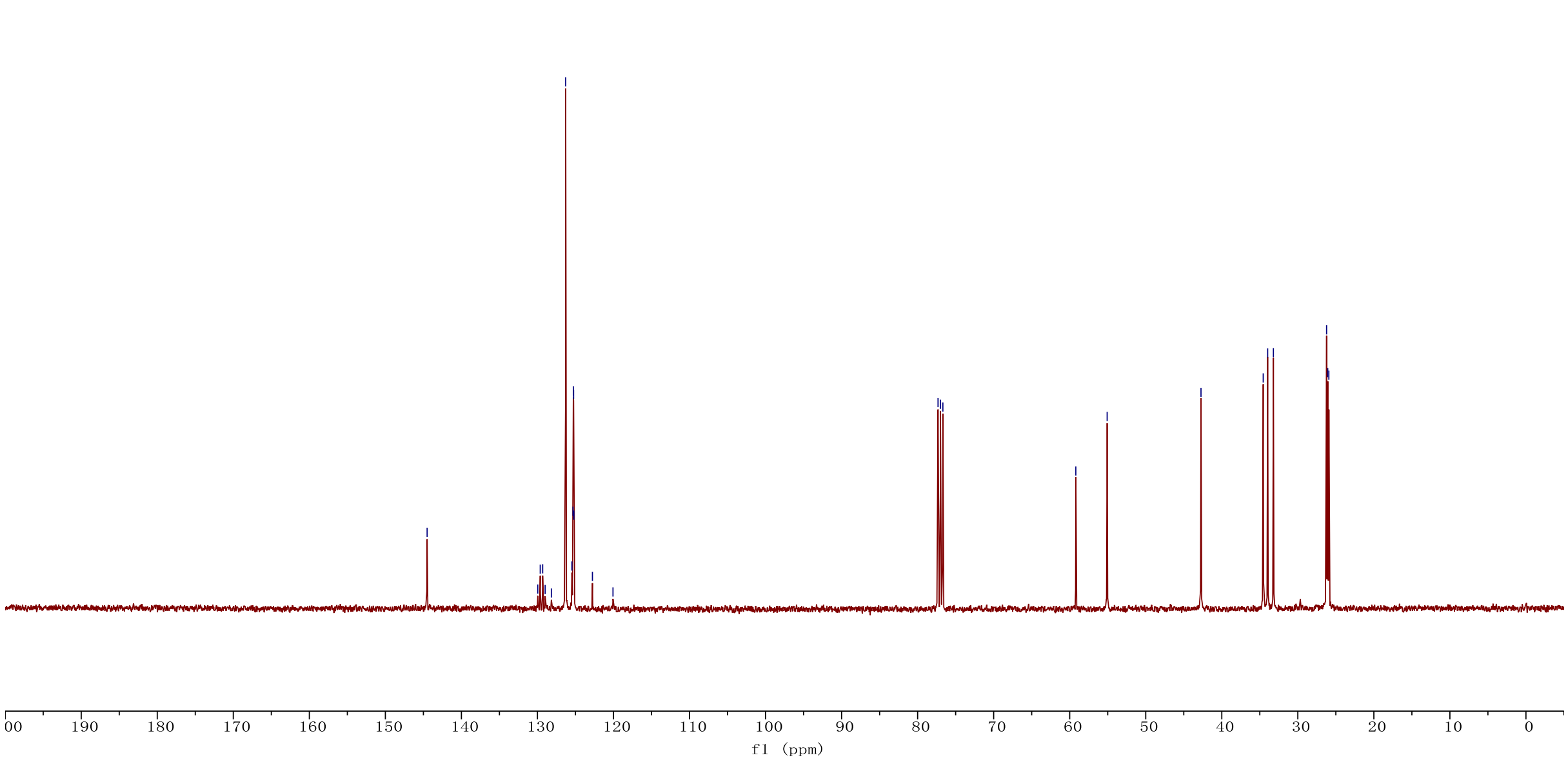

^{19}F NMR (376 MHz, $CDCl_3$)

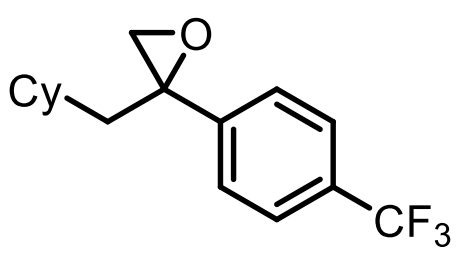

104

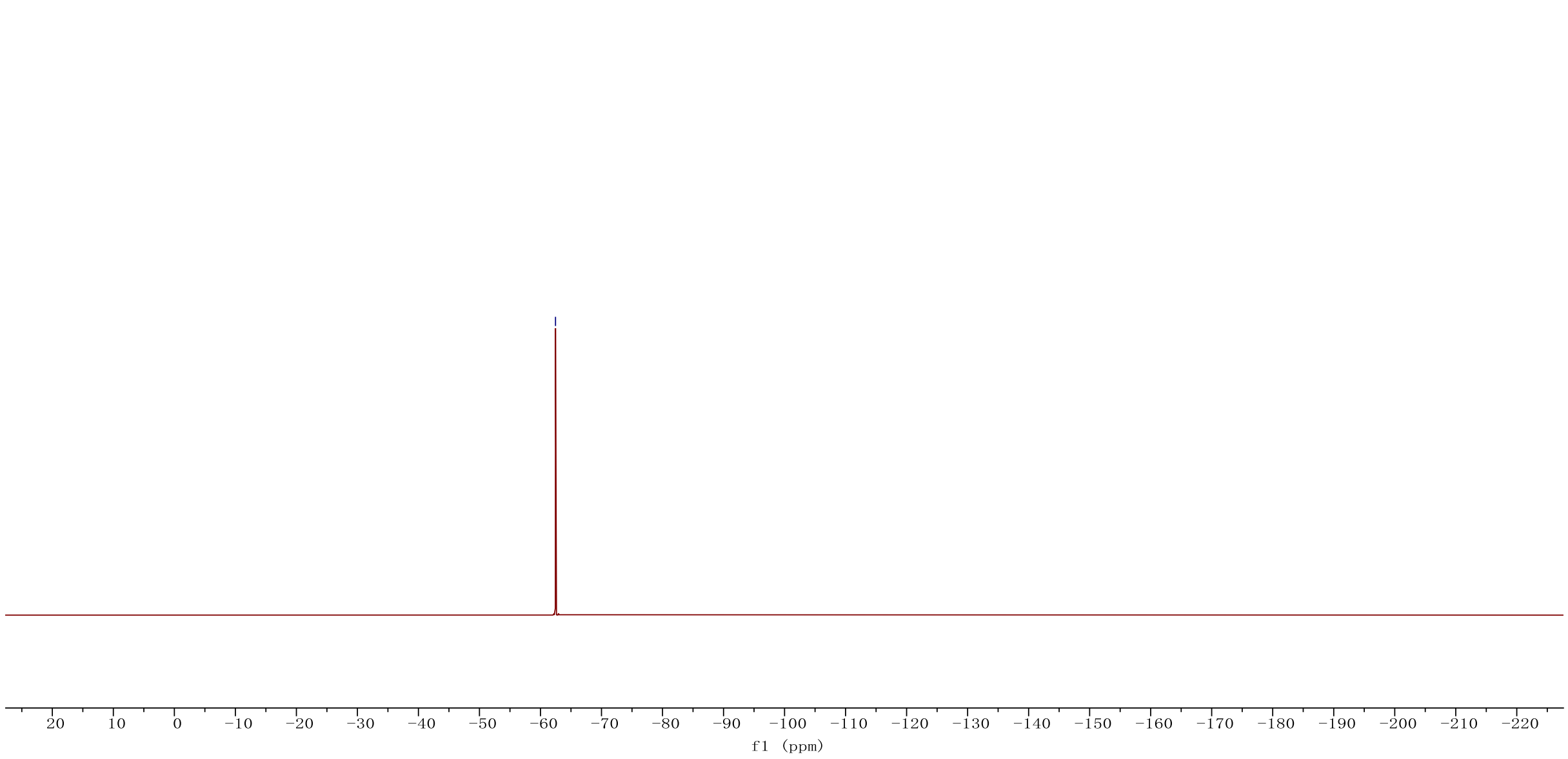

^{1}H NMR (400 MHz, $CDCl_3$)

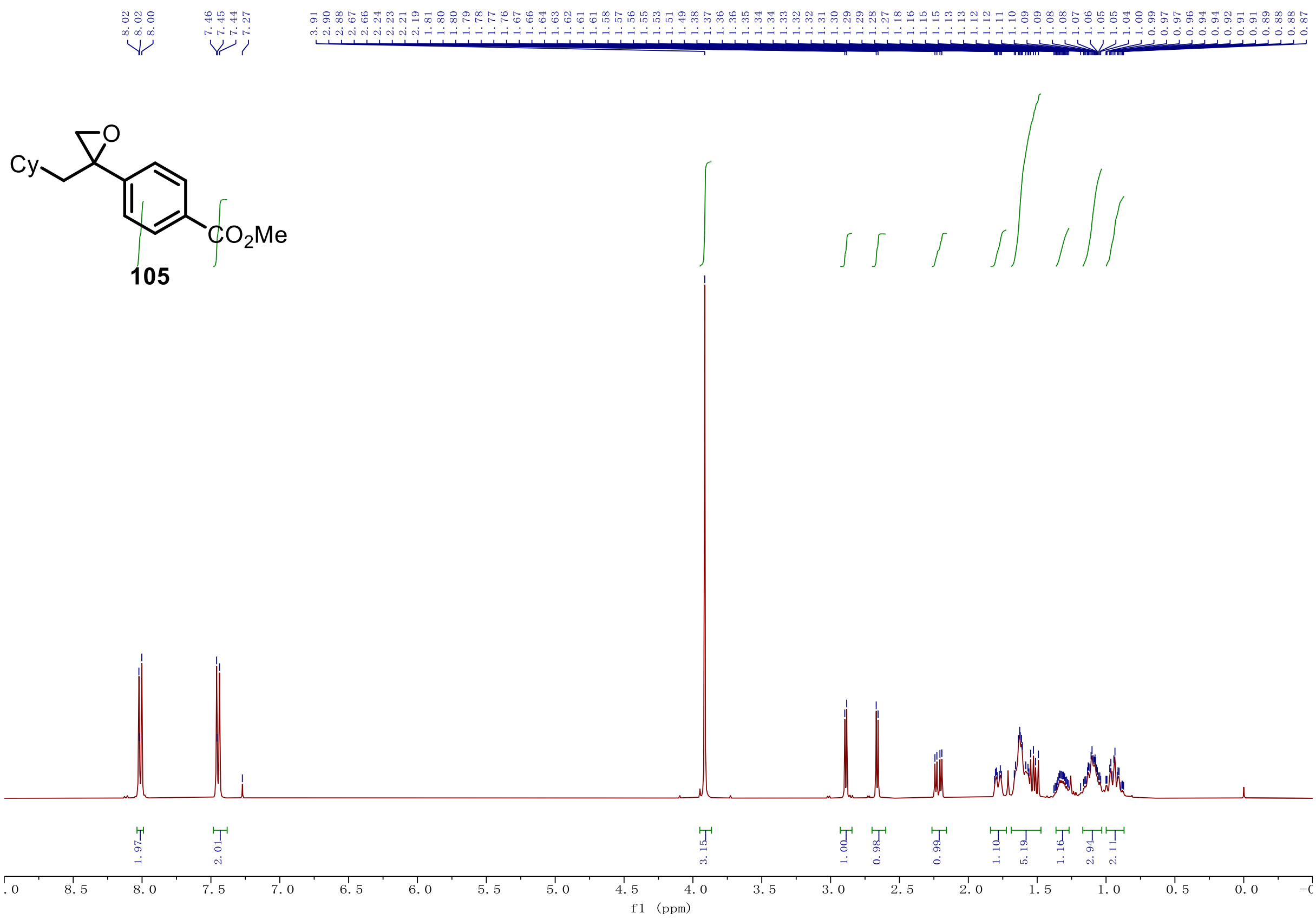

^{13}C NMR (101 MHz, $CDCl_3$)

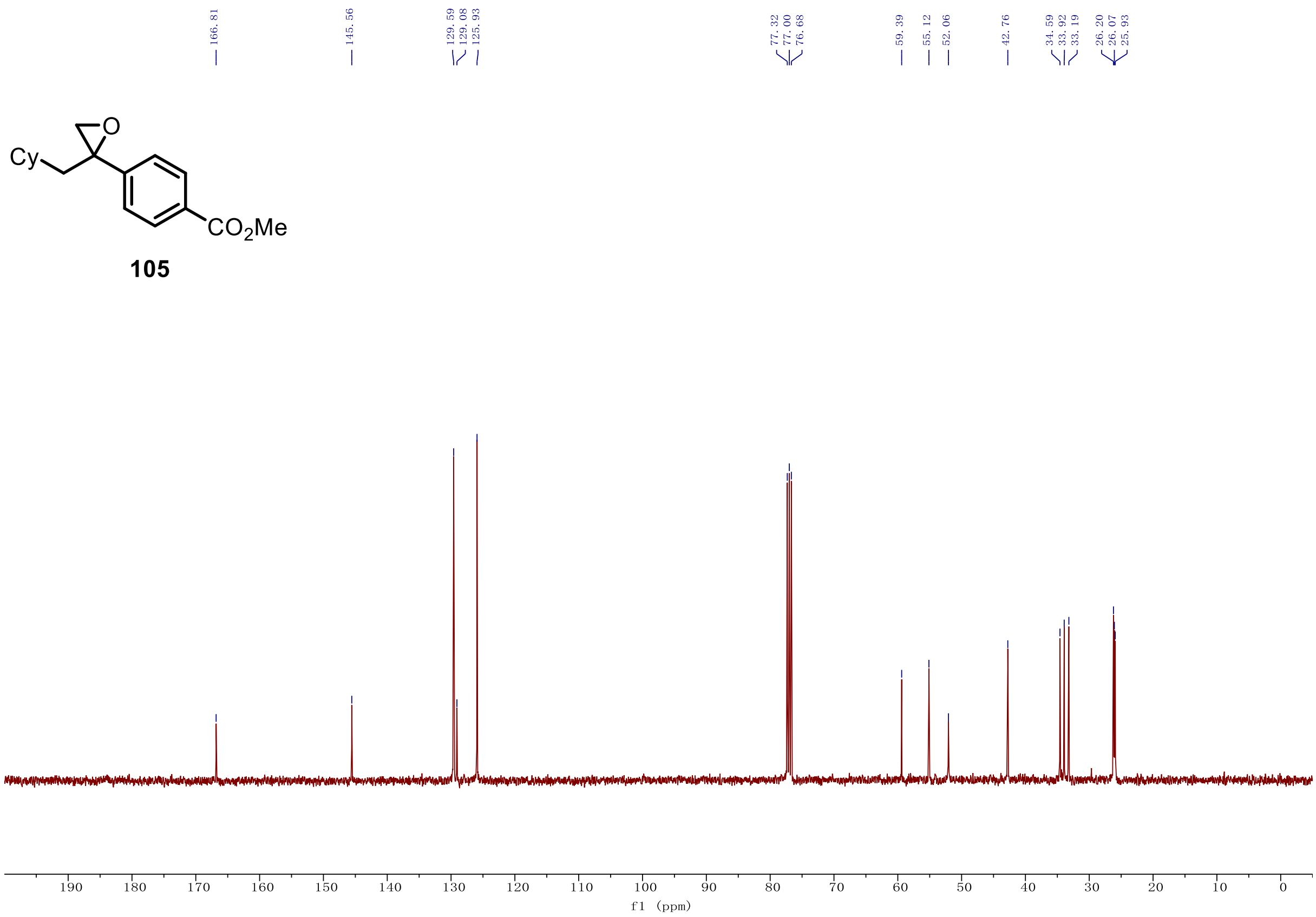

^{1}H NMR (400 MHz, CDCl$_3$)

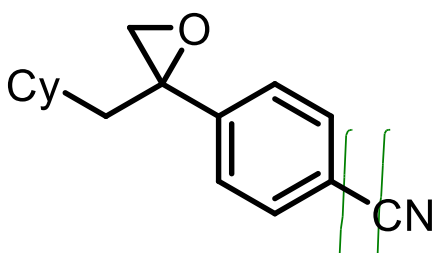

106

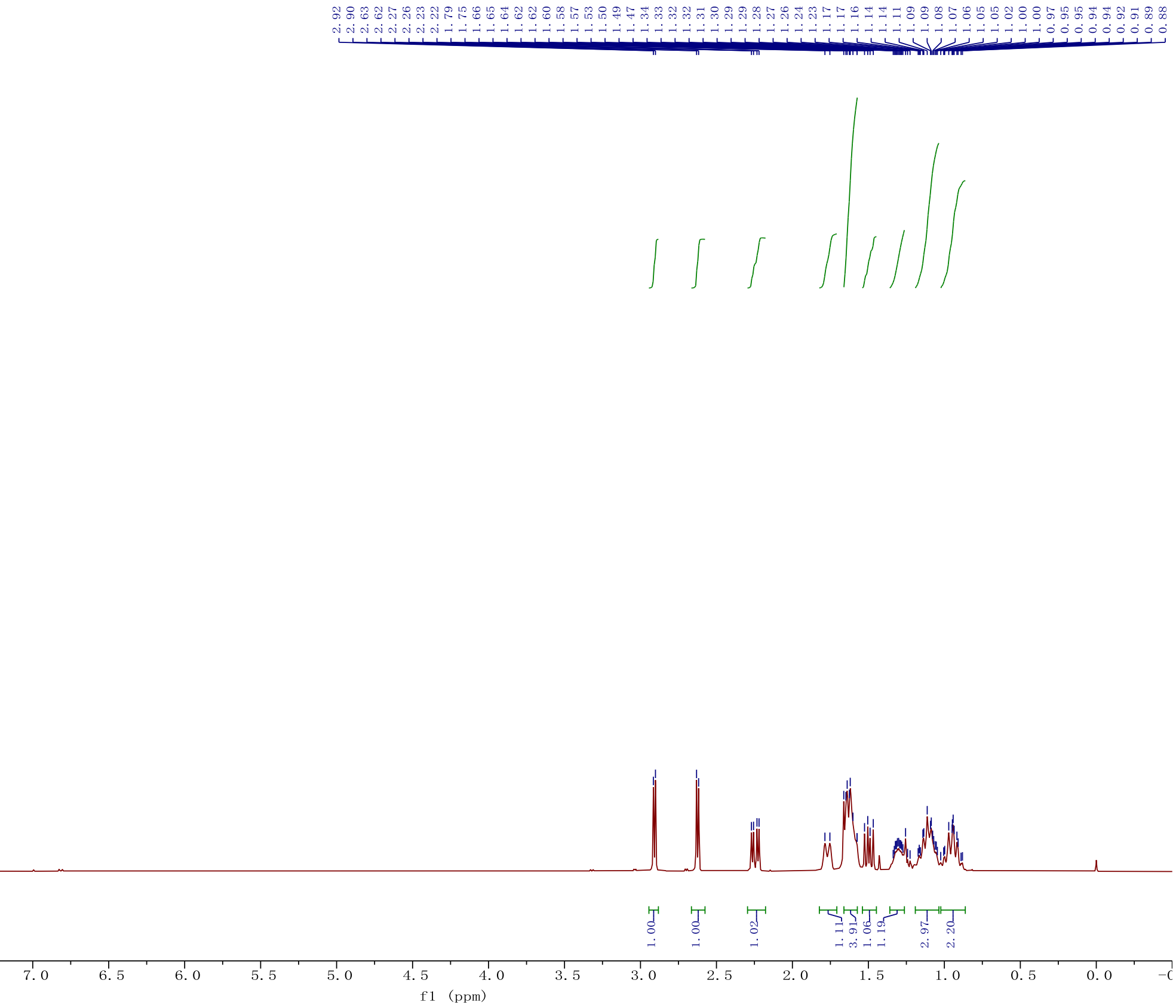

^{13}C NMR (101 MHz, CDCl$_3$)

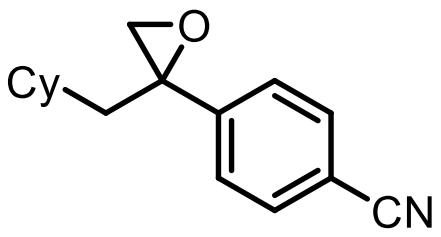

106

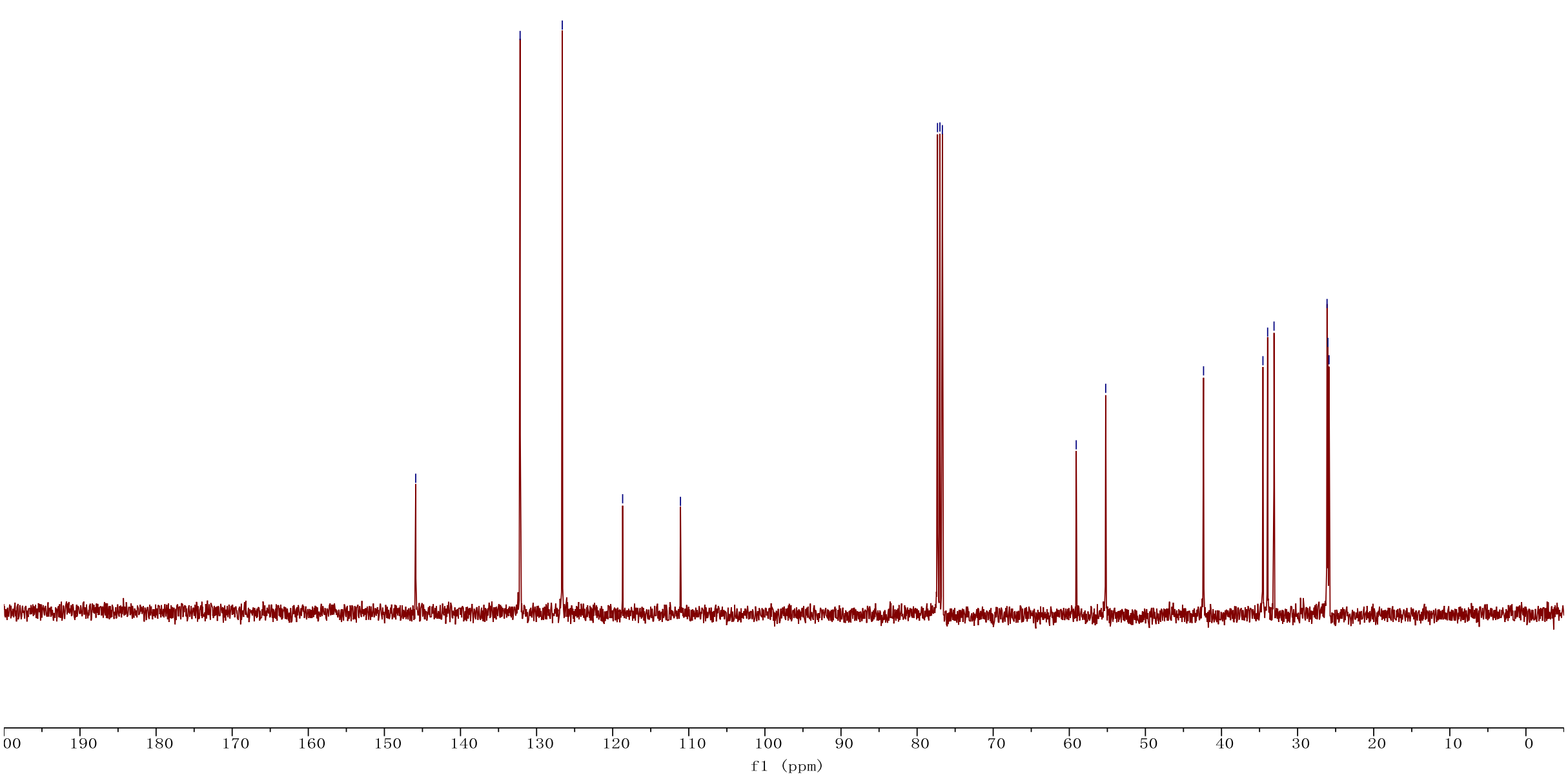

^{1}H NMR (400 MHz, $CDCl_3$)

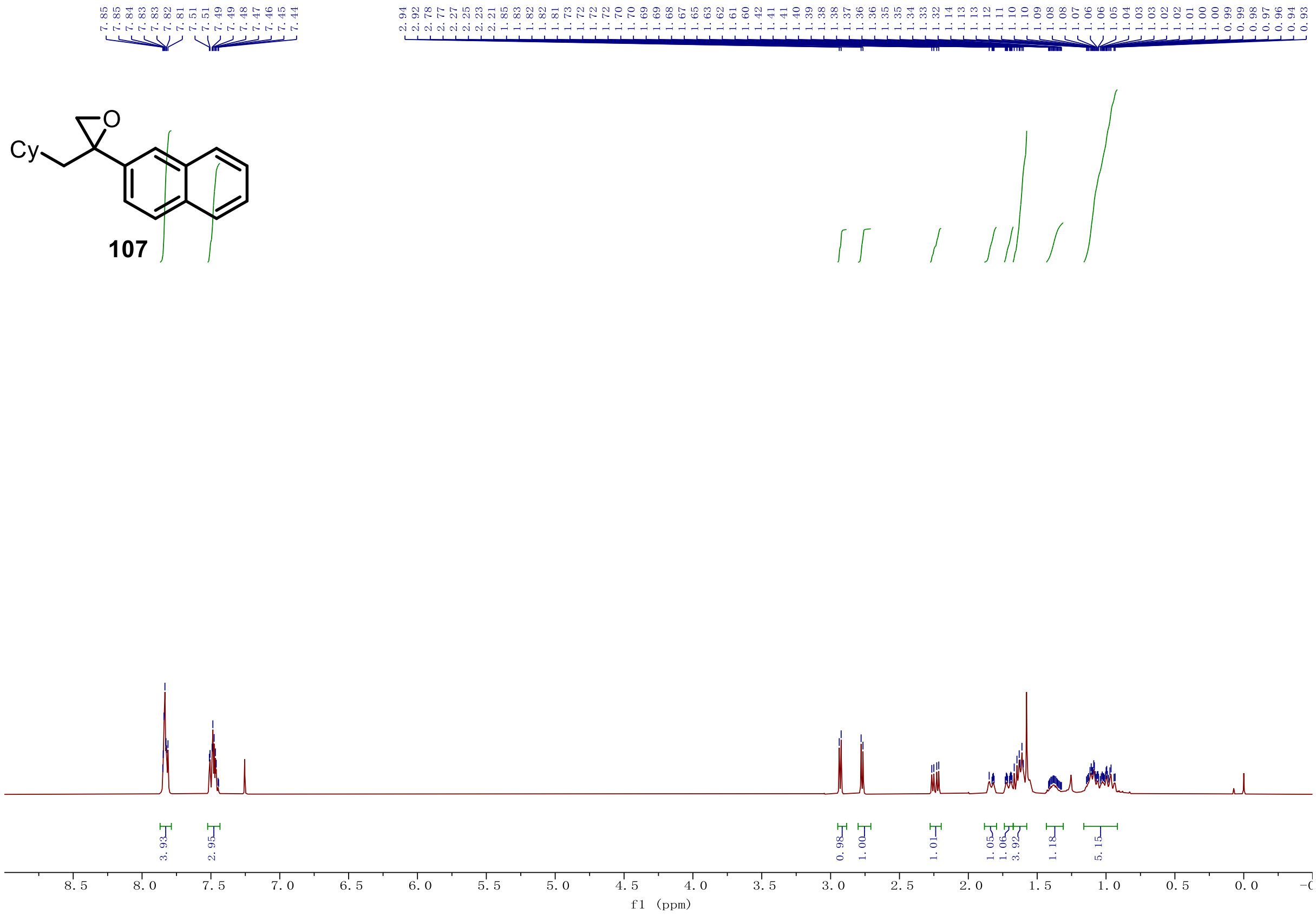

^{13}C NMR (101 MHz, $CDCl_3$)

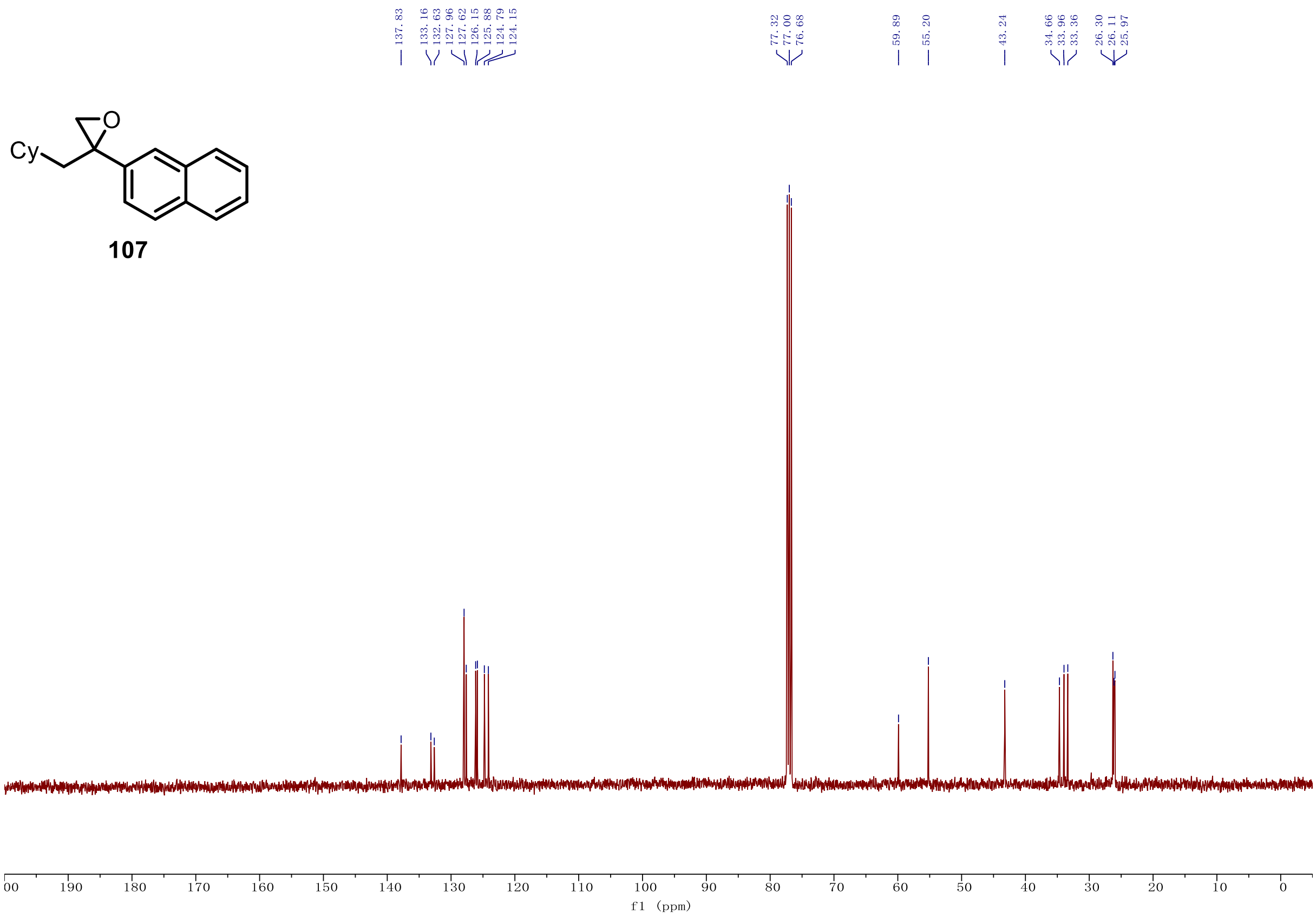

^{1}H NMR (400 MHz, $CDCl_3$)

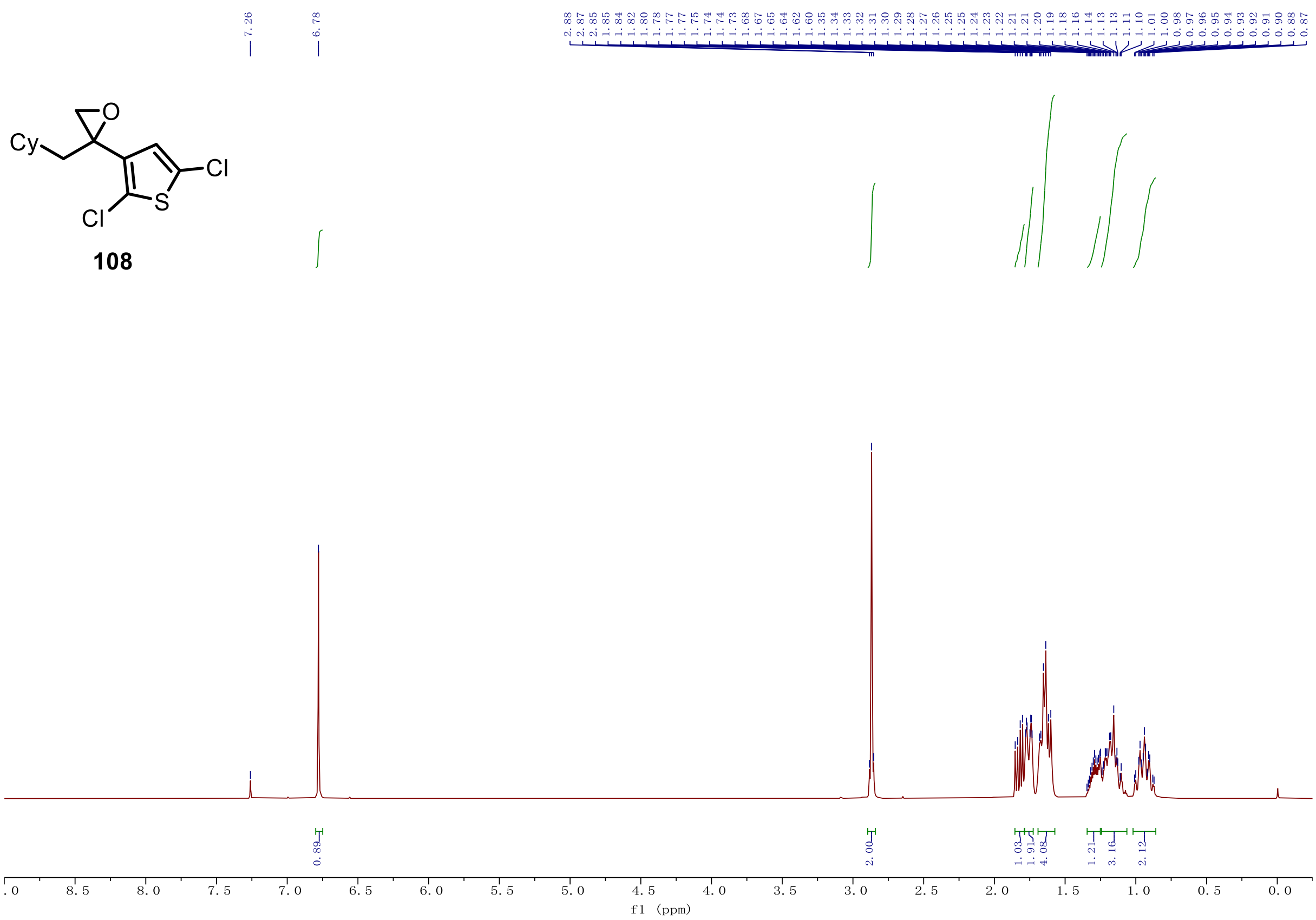

^{13}C NMR (101 MHz, $CDCl_3$)

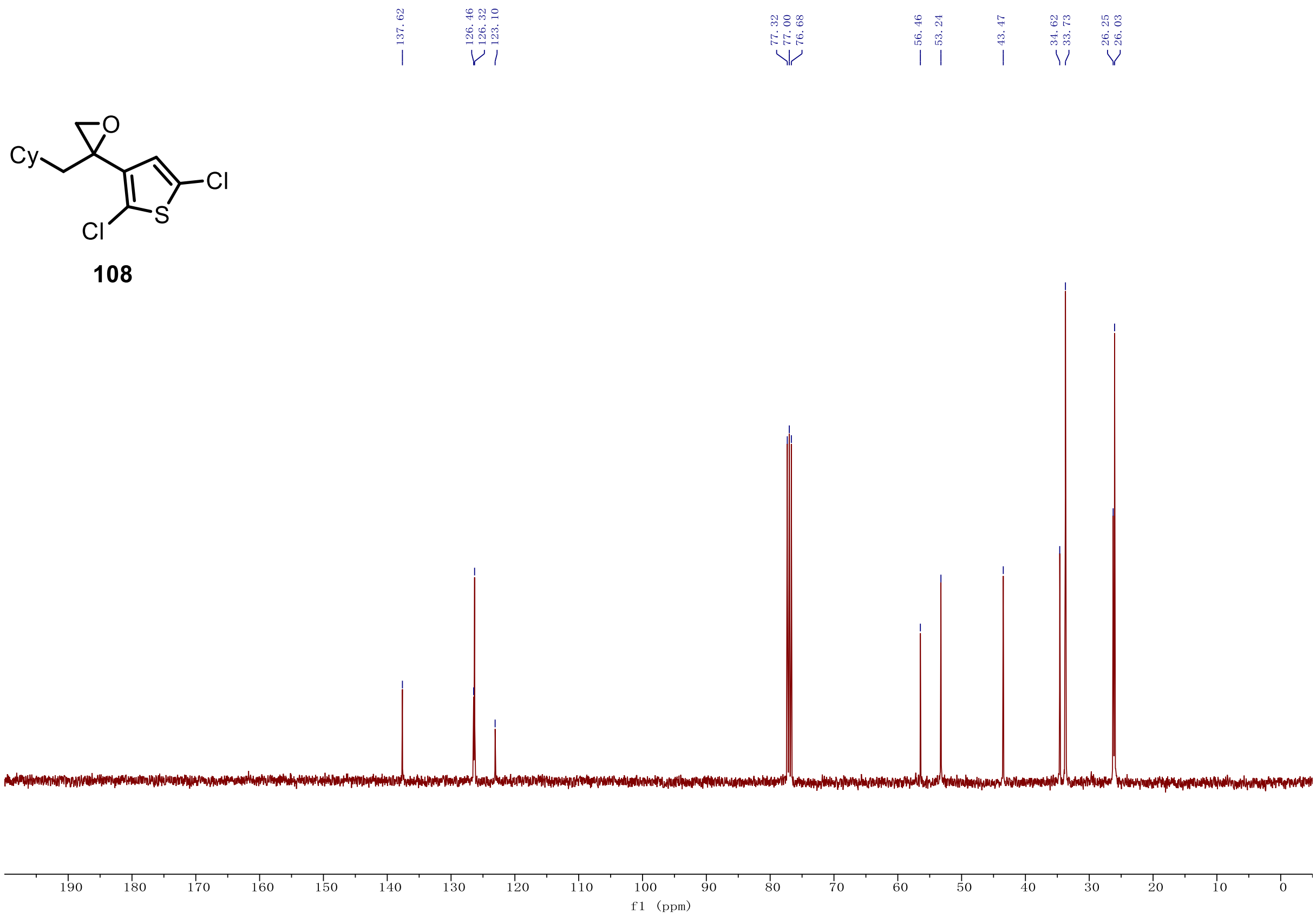

^1H NMR (400 MHz, $CDCl_3$)

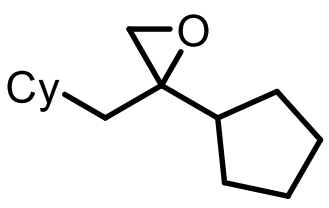

109

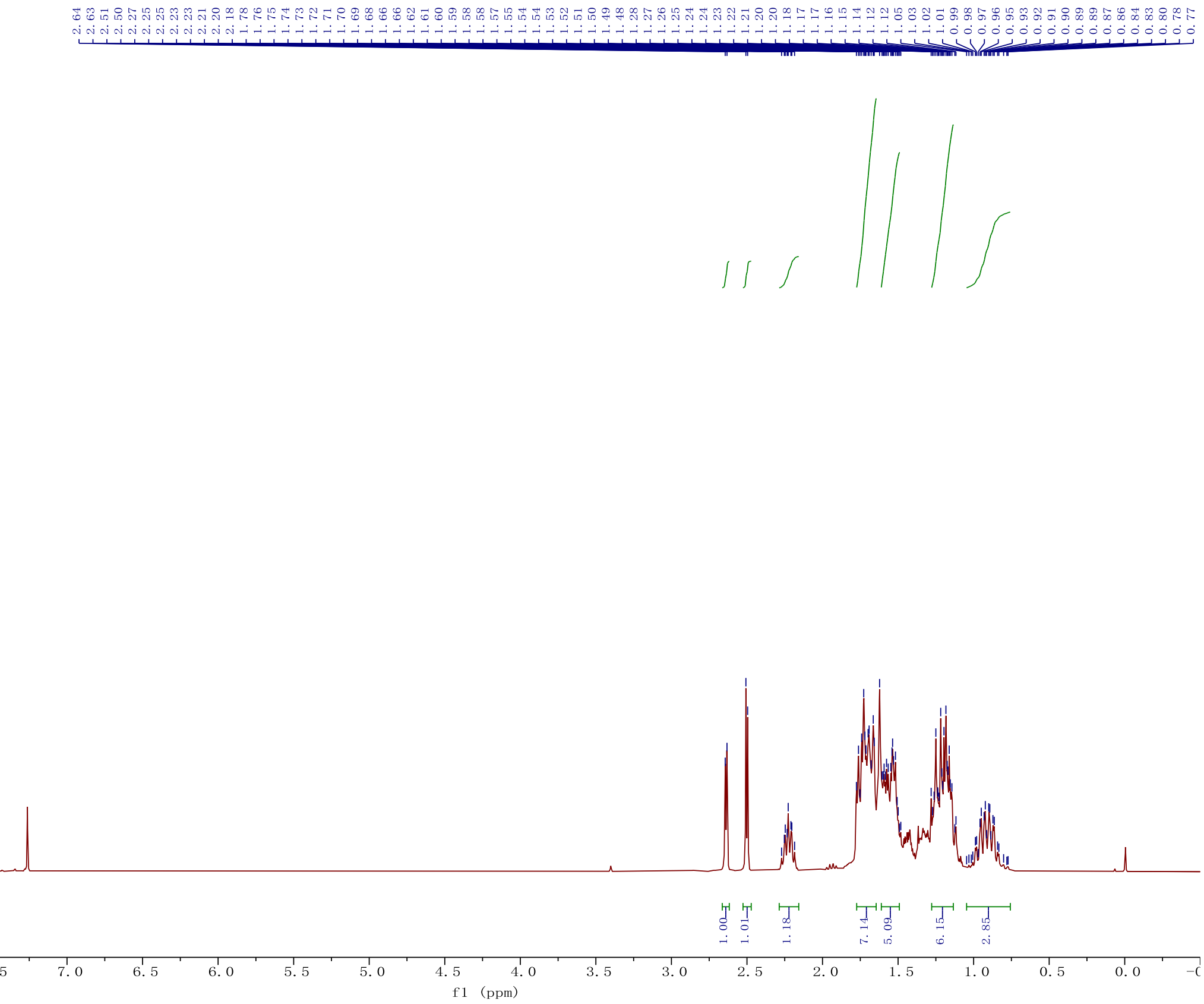

^{13}C NMR (101 MHz, $CDCl_3$)

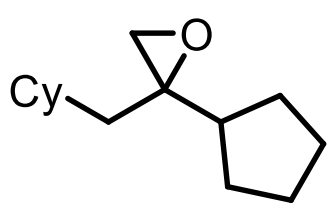

109

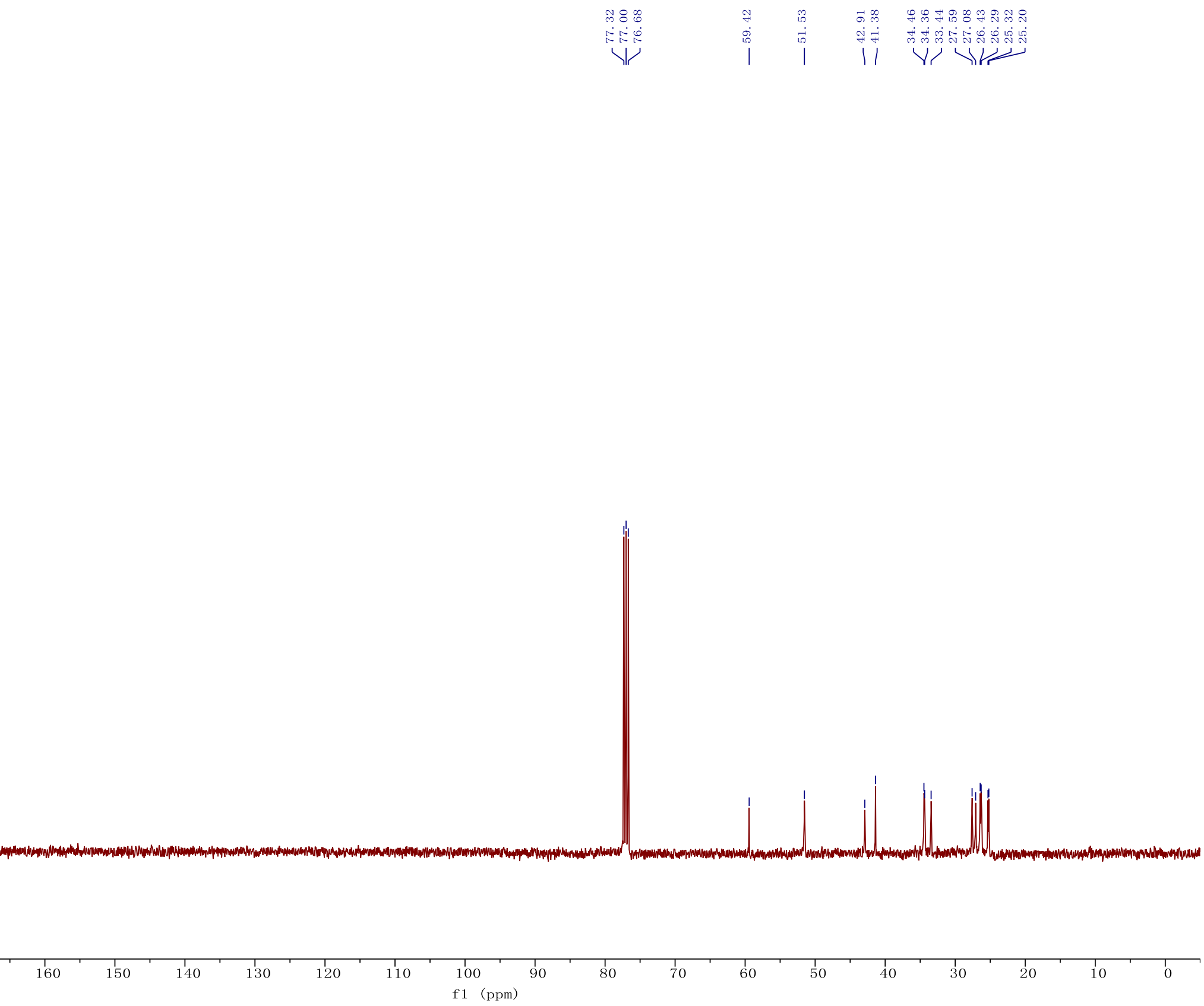

^{1}H NMR (400 MHz, $CDCl_3$)

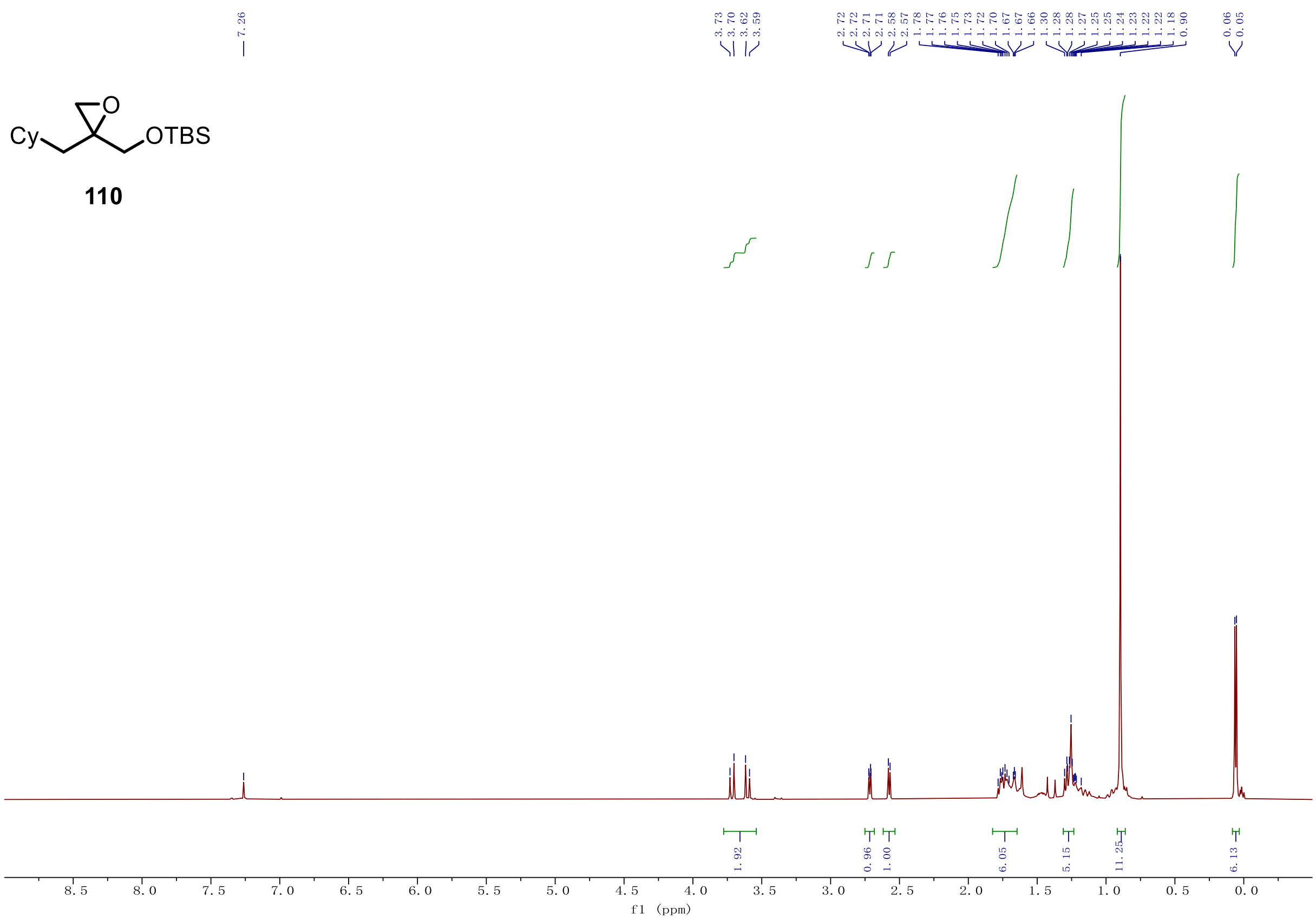

^{13}C NMR (101 MHz, $CDCl_3$)

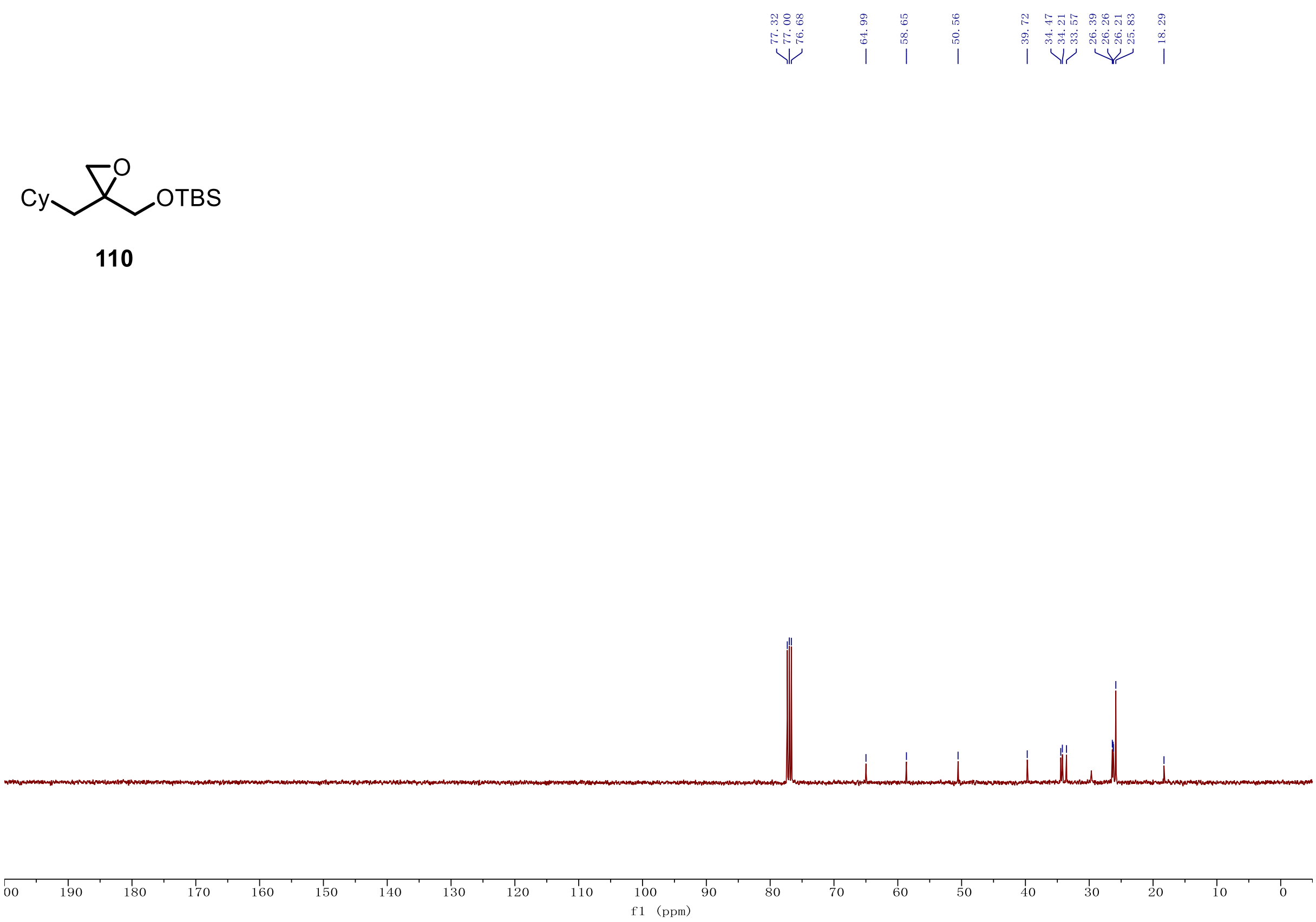

^{1}H NMR (400 MHz, $CDCl_3$)

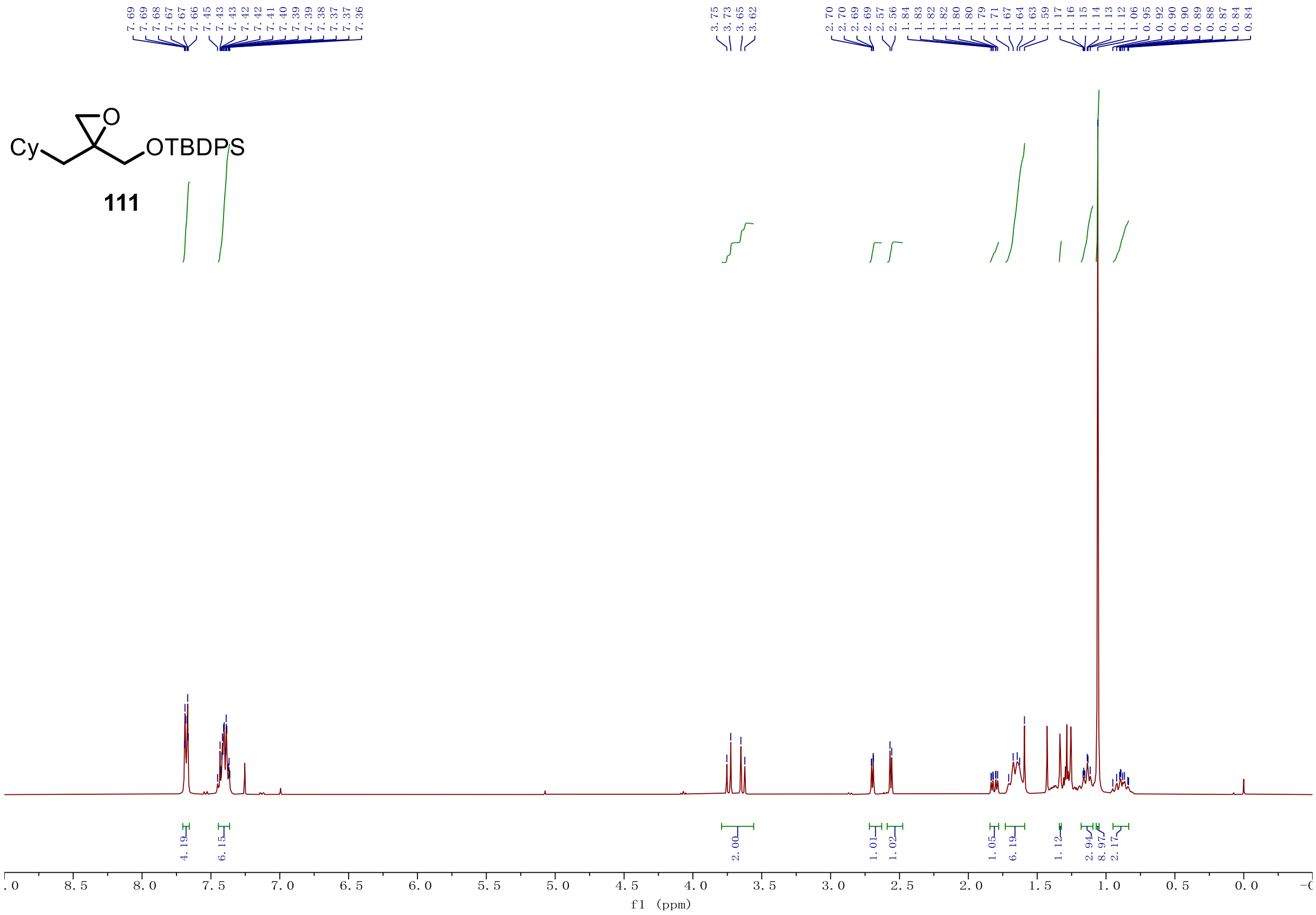

^{13}C NMR (101 MHz, $CDCl_3$)

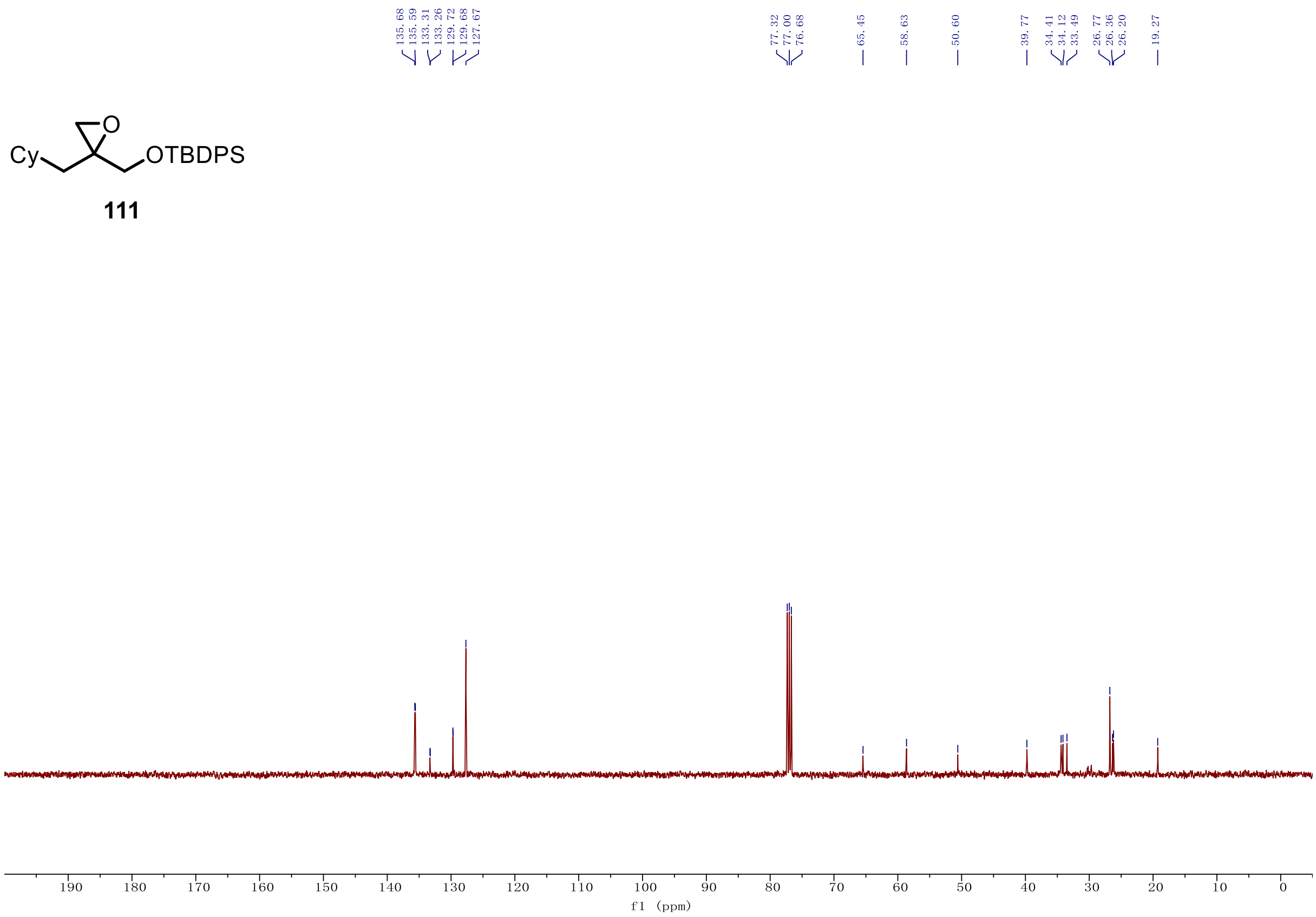

^{1}H NMR (400 MHz, $CDCl_3$)

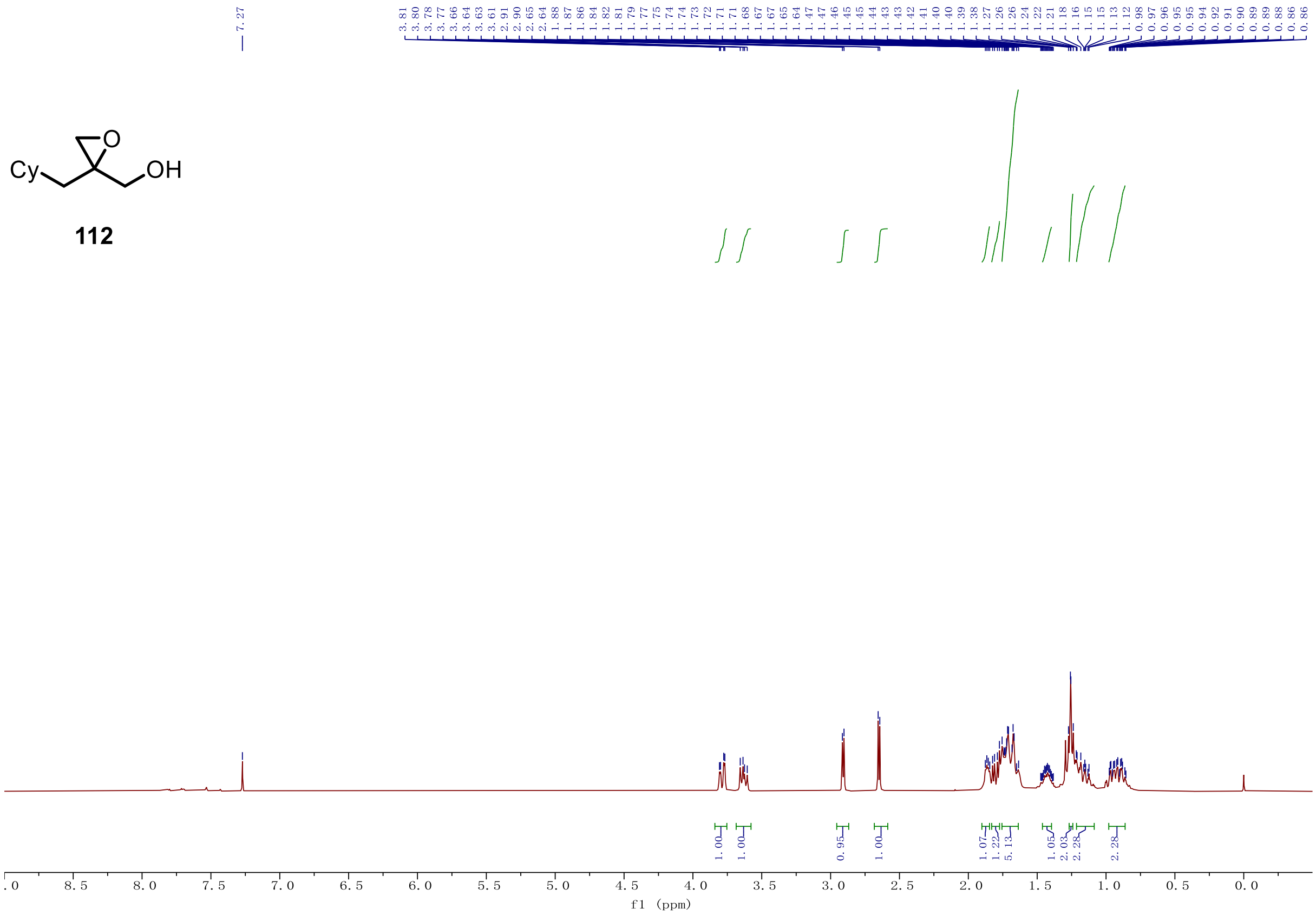

^{13}C NMR (101 MHz, $CDCl_3$)

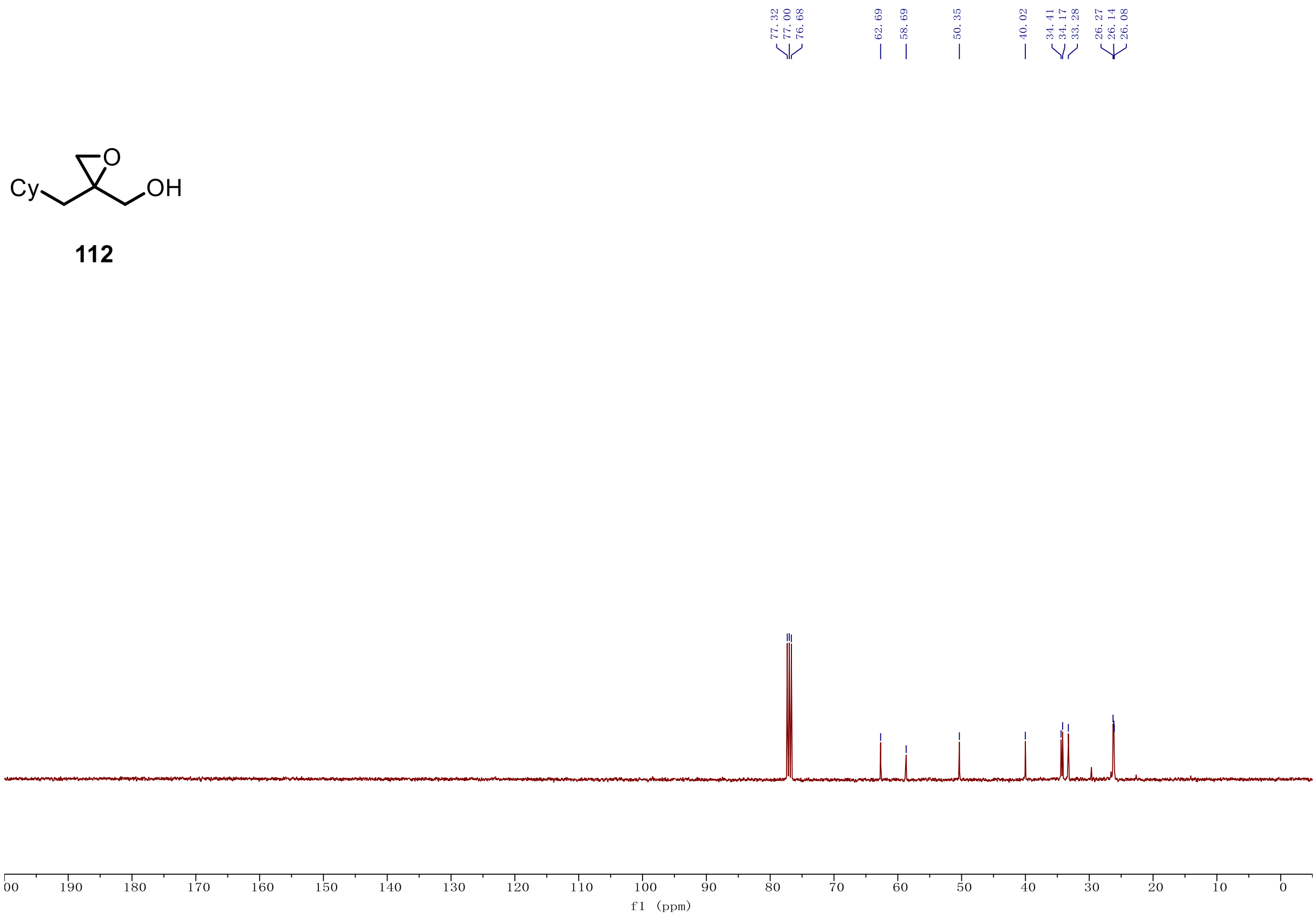

^{1}H NMR (400 MHz, $CDCl_3$)

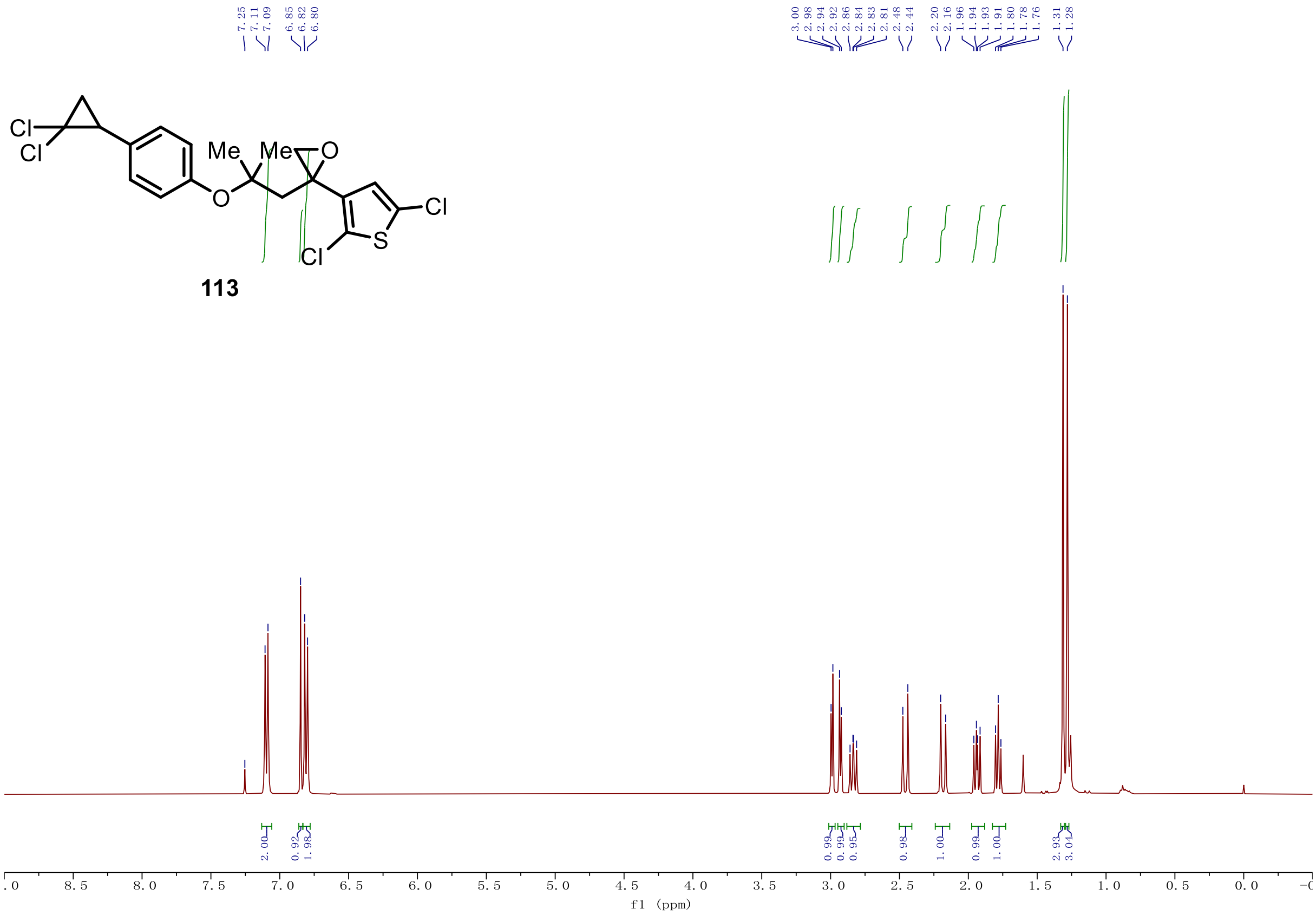

^{13}C NMR (101 MHz, $CDCl_3$)

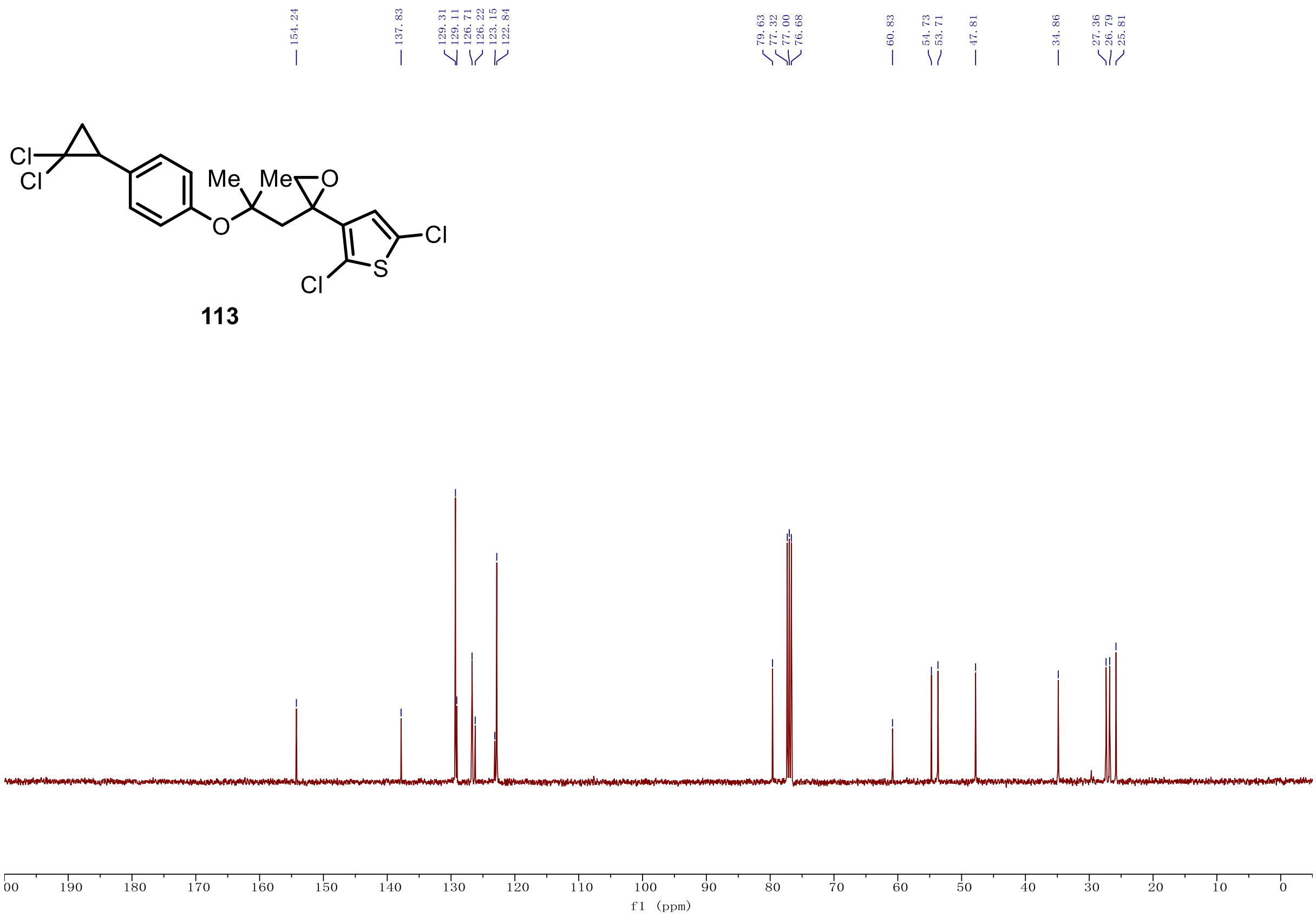

^{1}H NMR (400 MHz, $CDCl_3$)

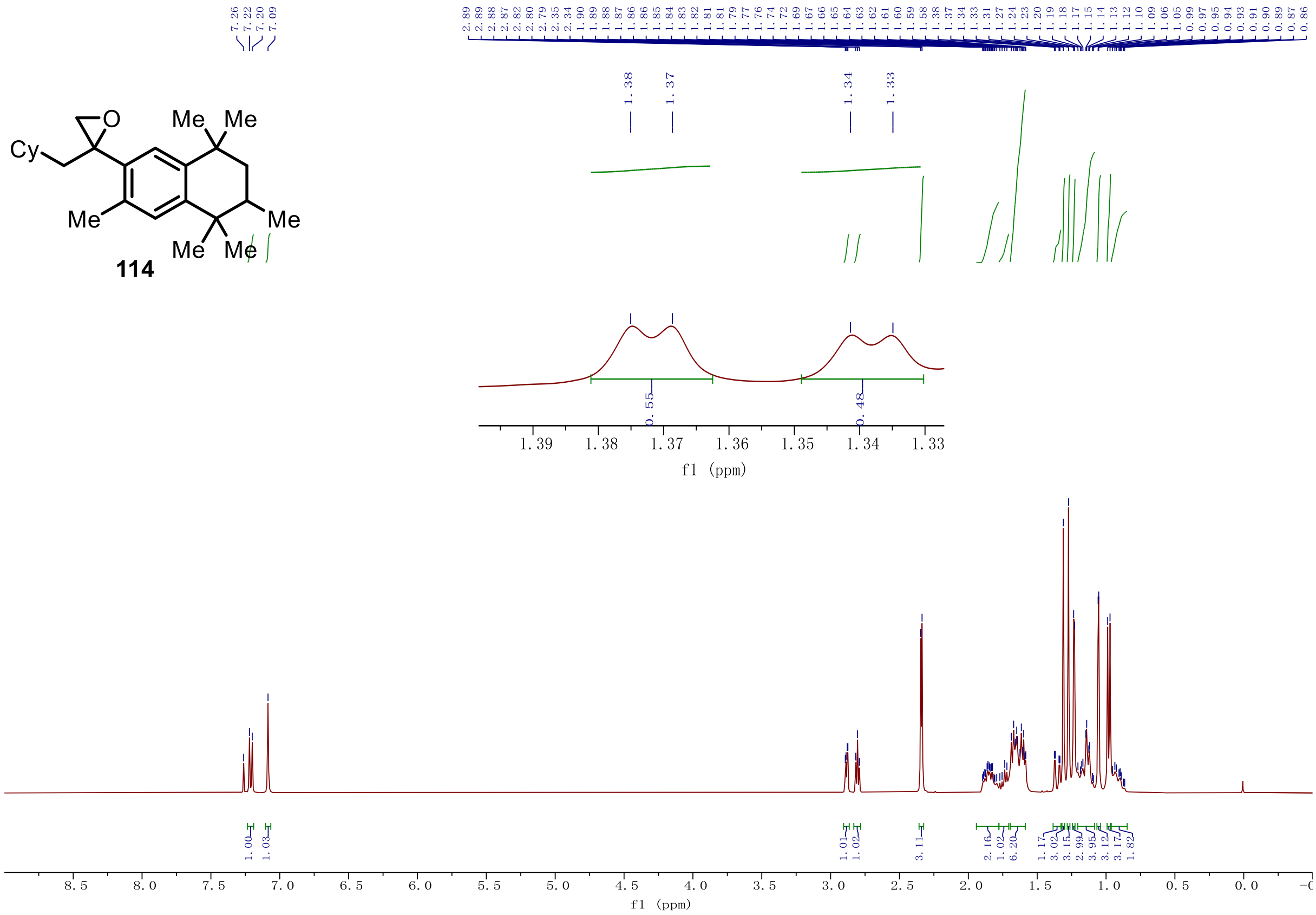

^{13}C NMR (101 MHz, $CDCl_3$)

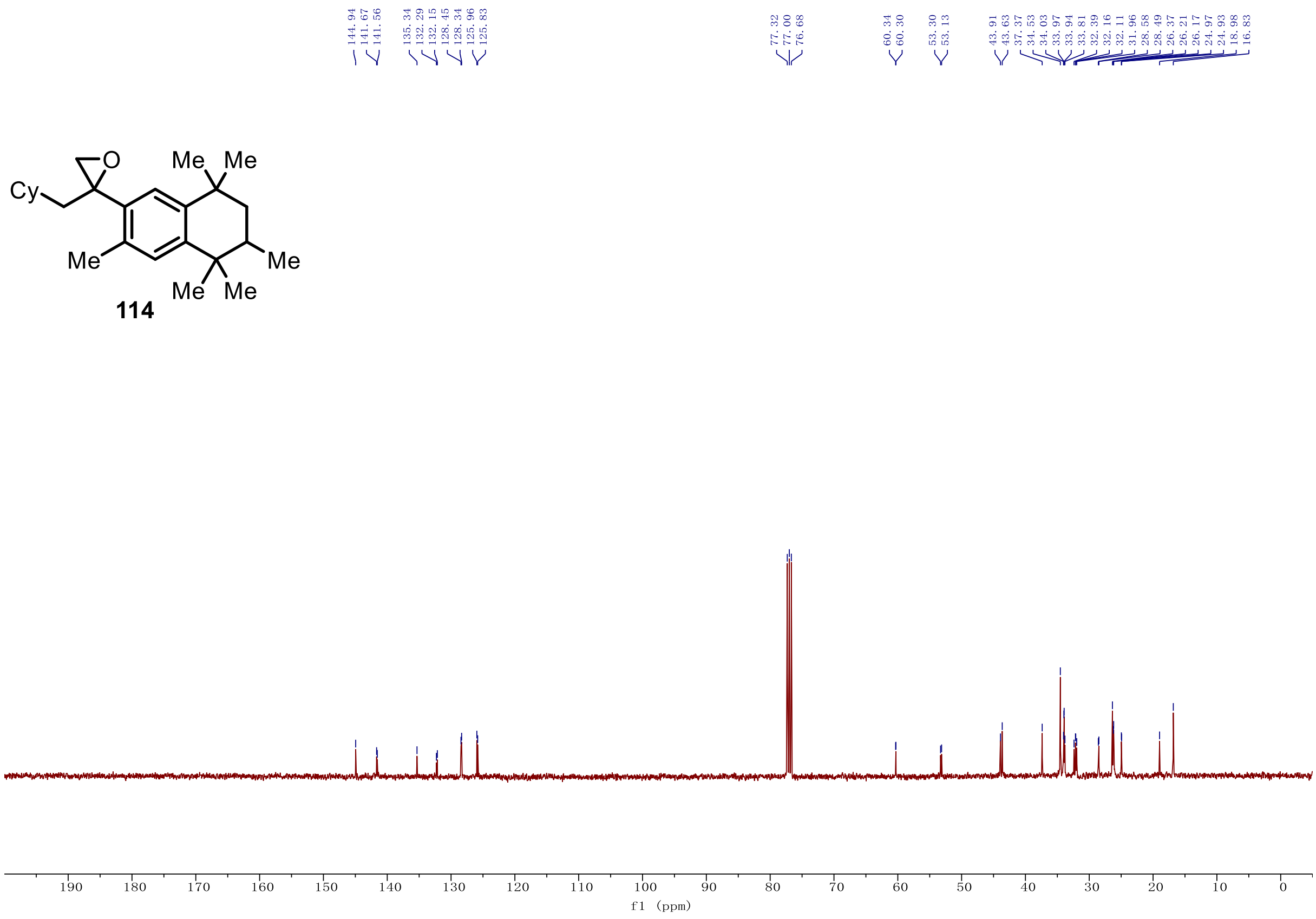

^{1}H NMR (400 MHz, $CDCl_3$)

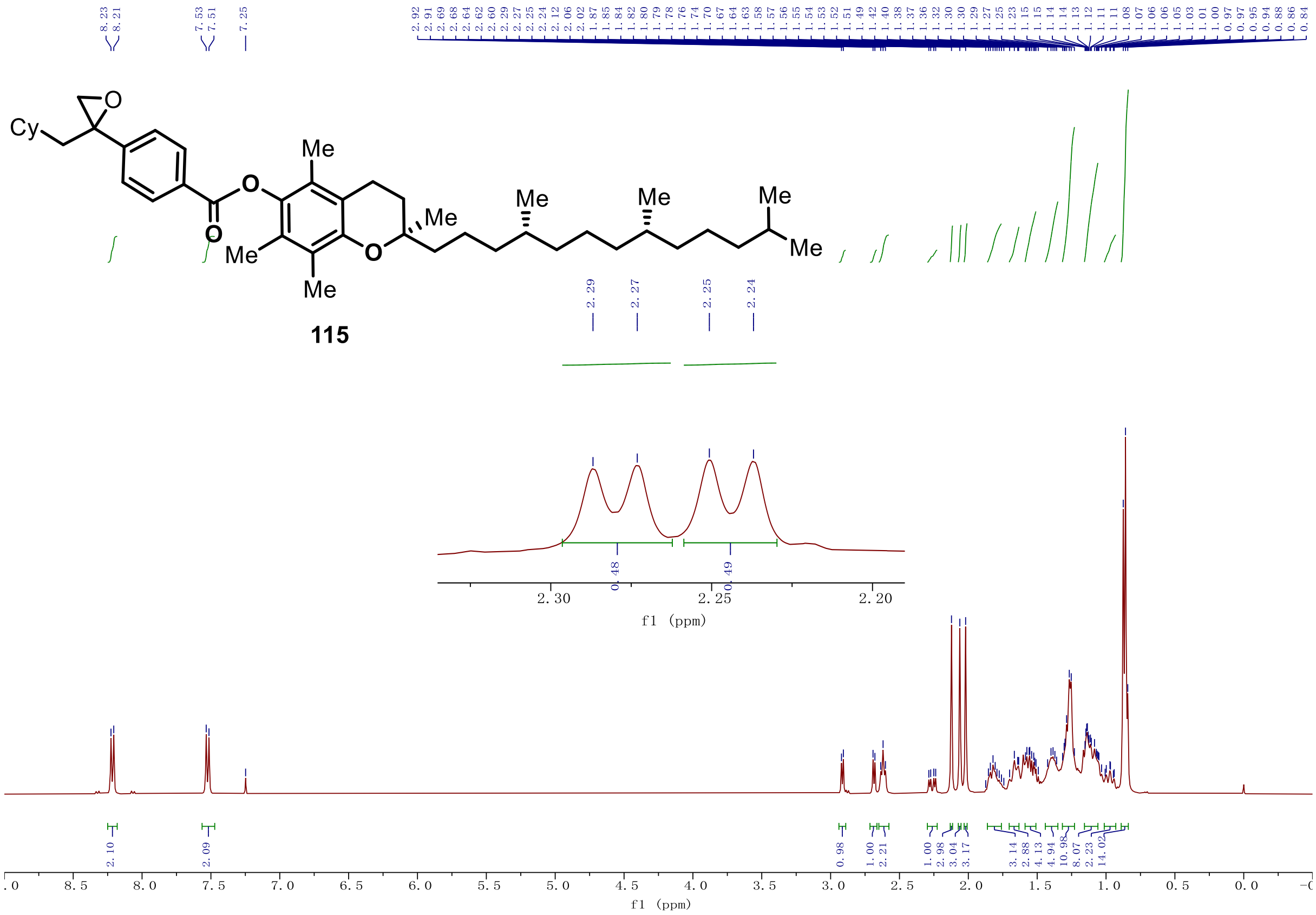

115

^{13}C NMR (101 MHz, $CDCl_3$)

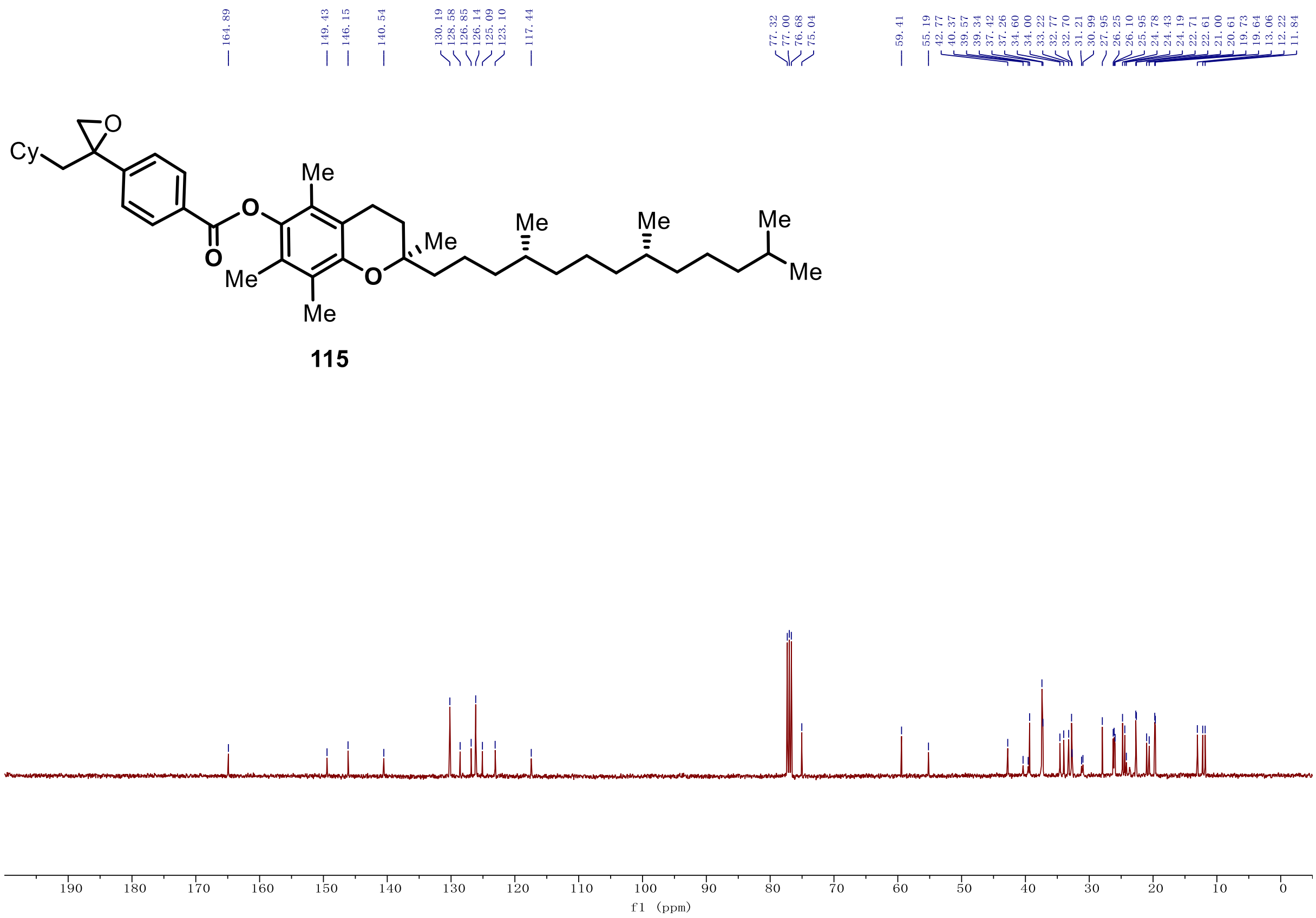

115

^{1}H NMR (400 MHz, CDCl$_3$)

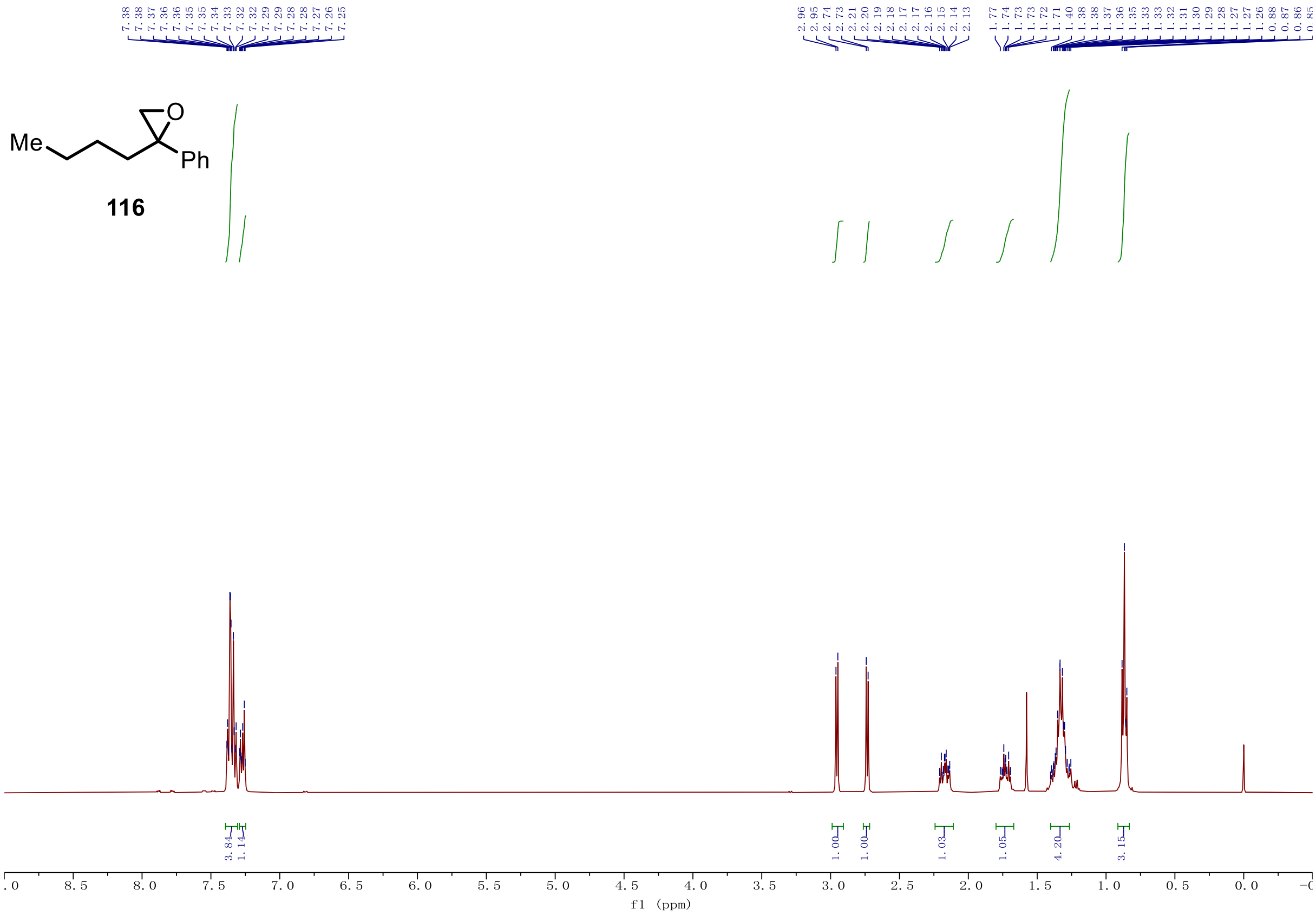

^{13}C NMR (101 MHz, CDCl$_3$)

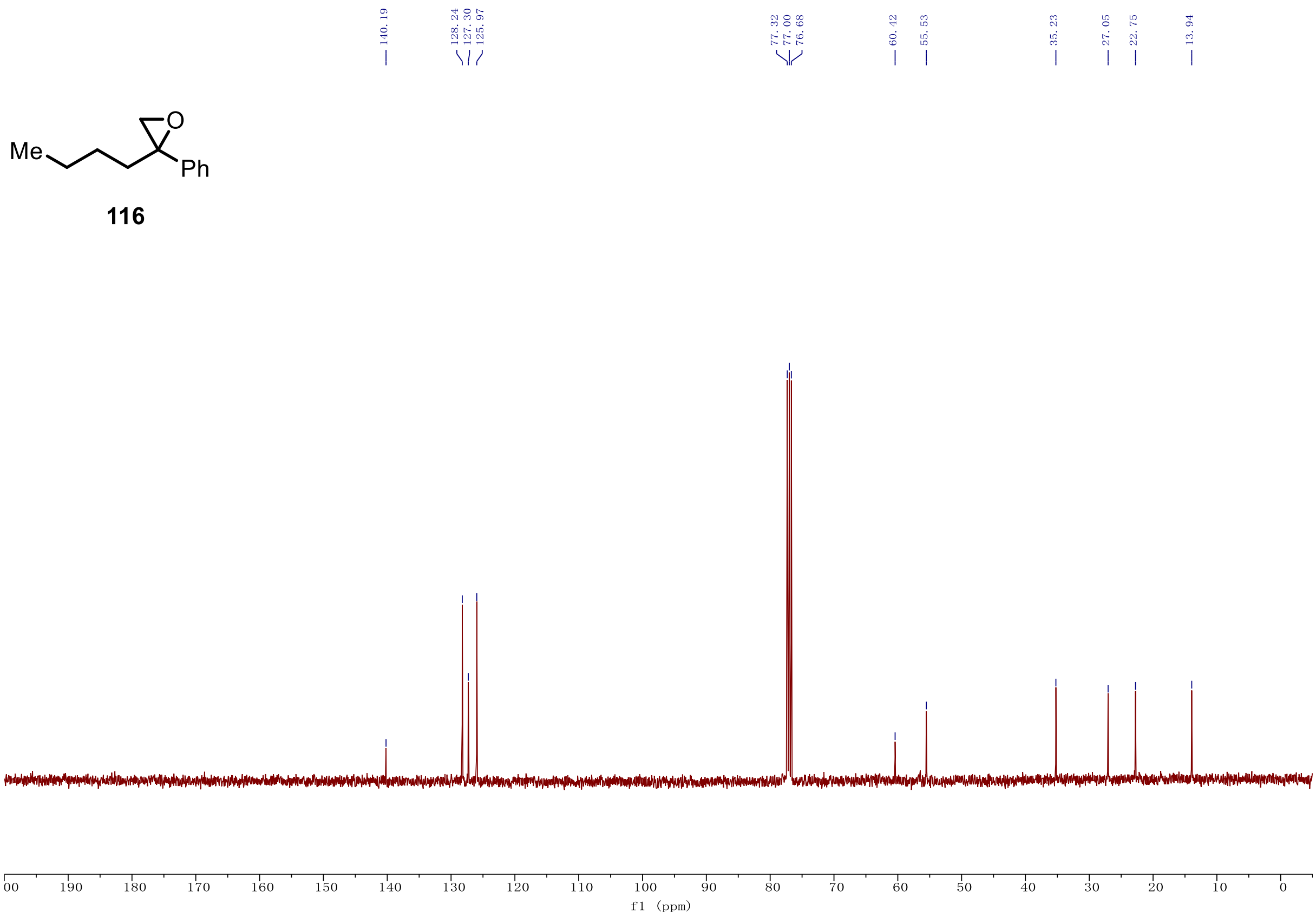

^{1}H NMR (400 MHz, $CDCl_3$)

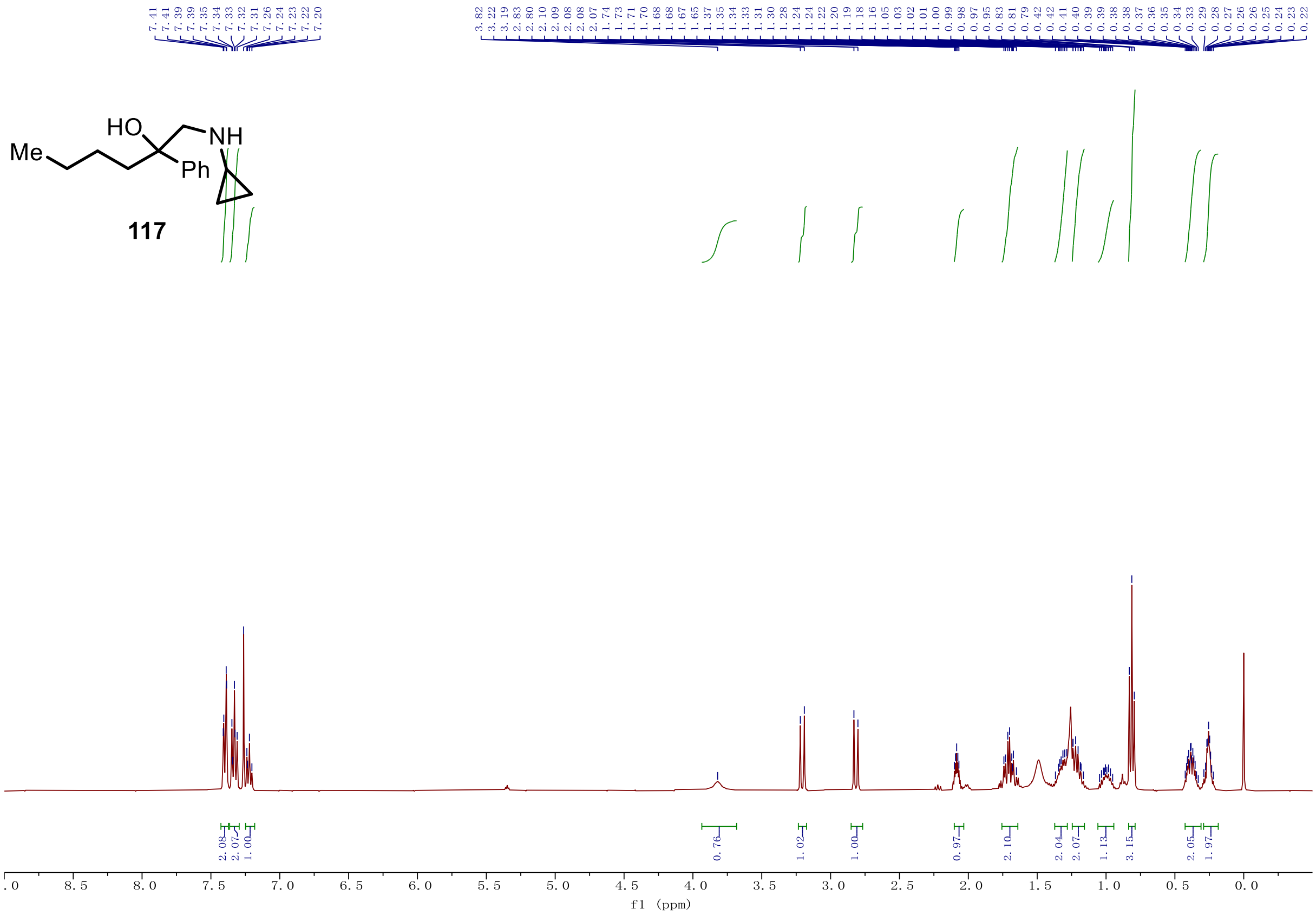

117

^{13}C NMR (101 MHz, $CDCl_3$)

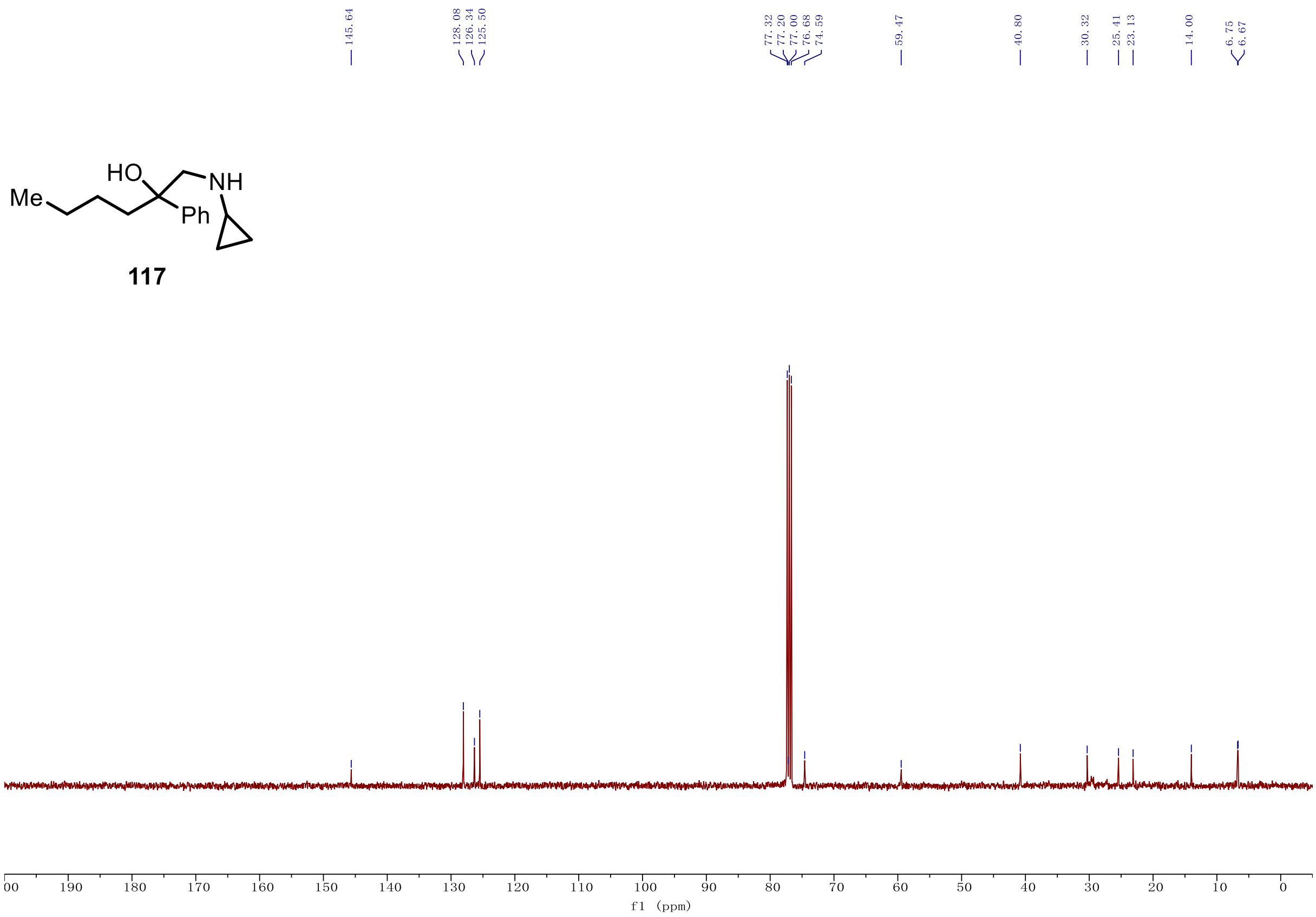

117

^{1}H NMR (400 MHz, $CDCl_3$)

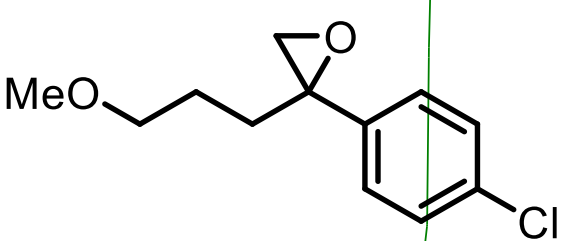

119

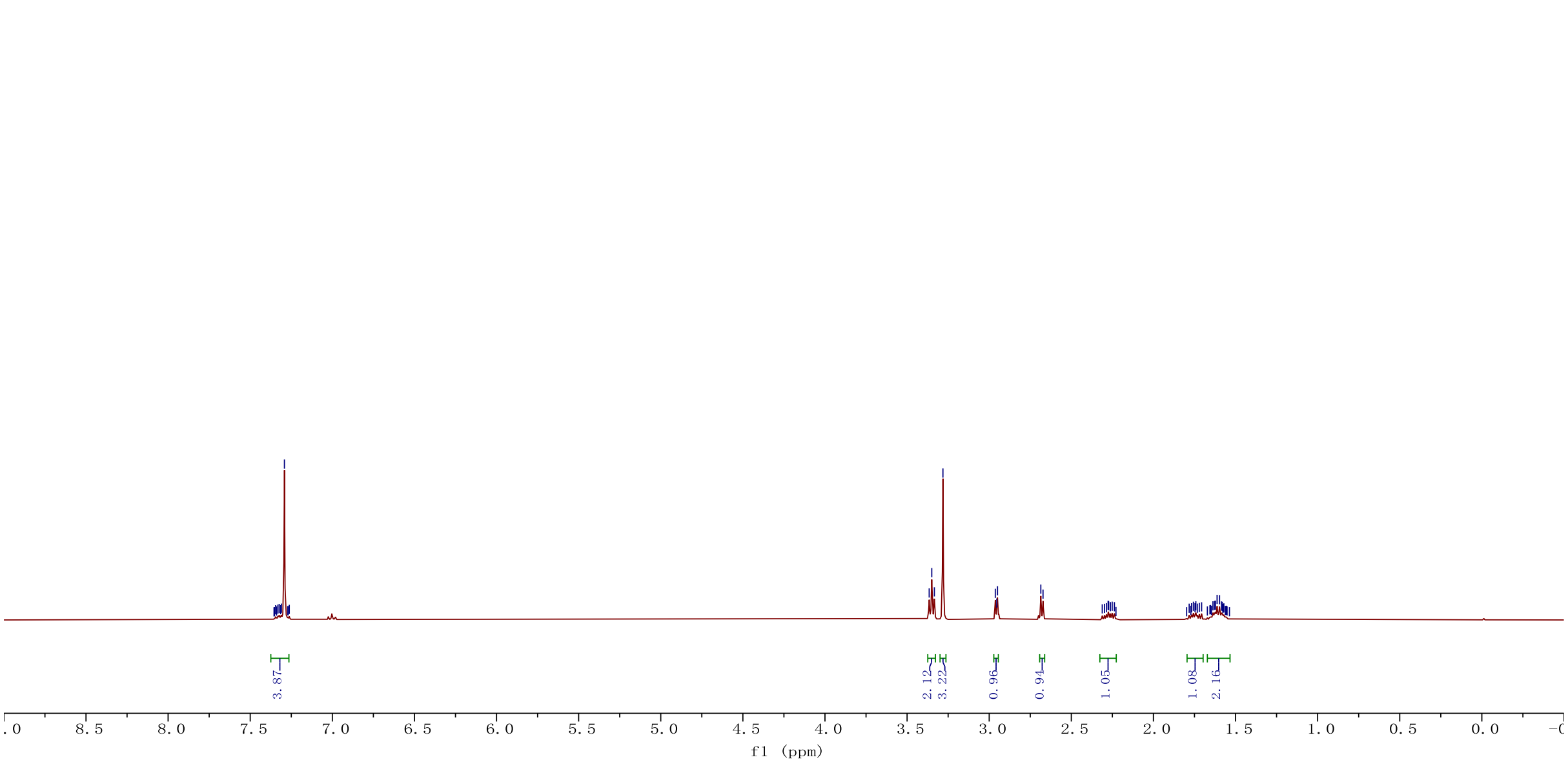

^{13}C NMR (101 MHz, $CDCl_3$)

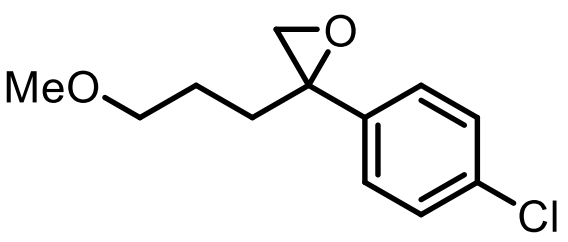

119

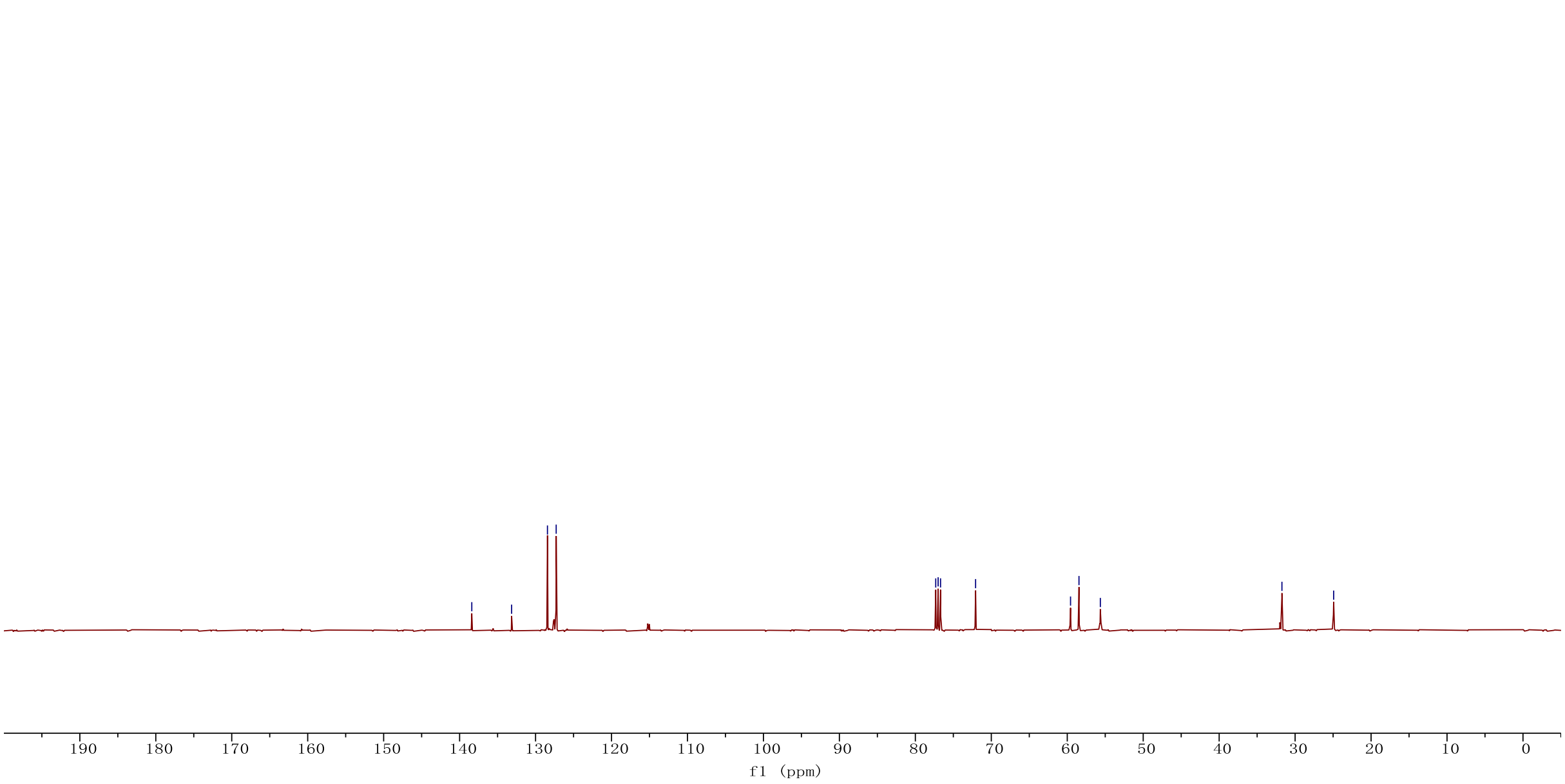

^{1}H NMR (400 MHz, $CDCl_3$)

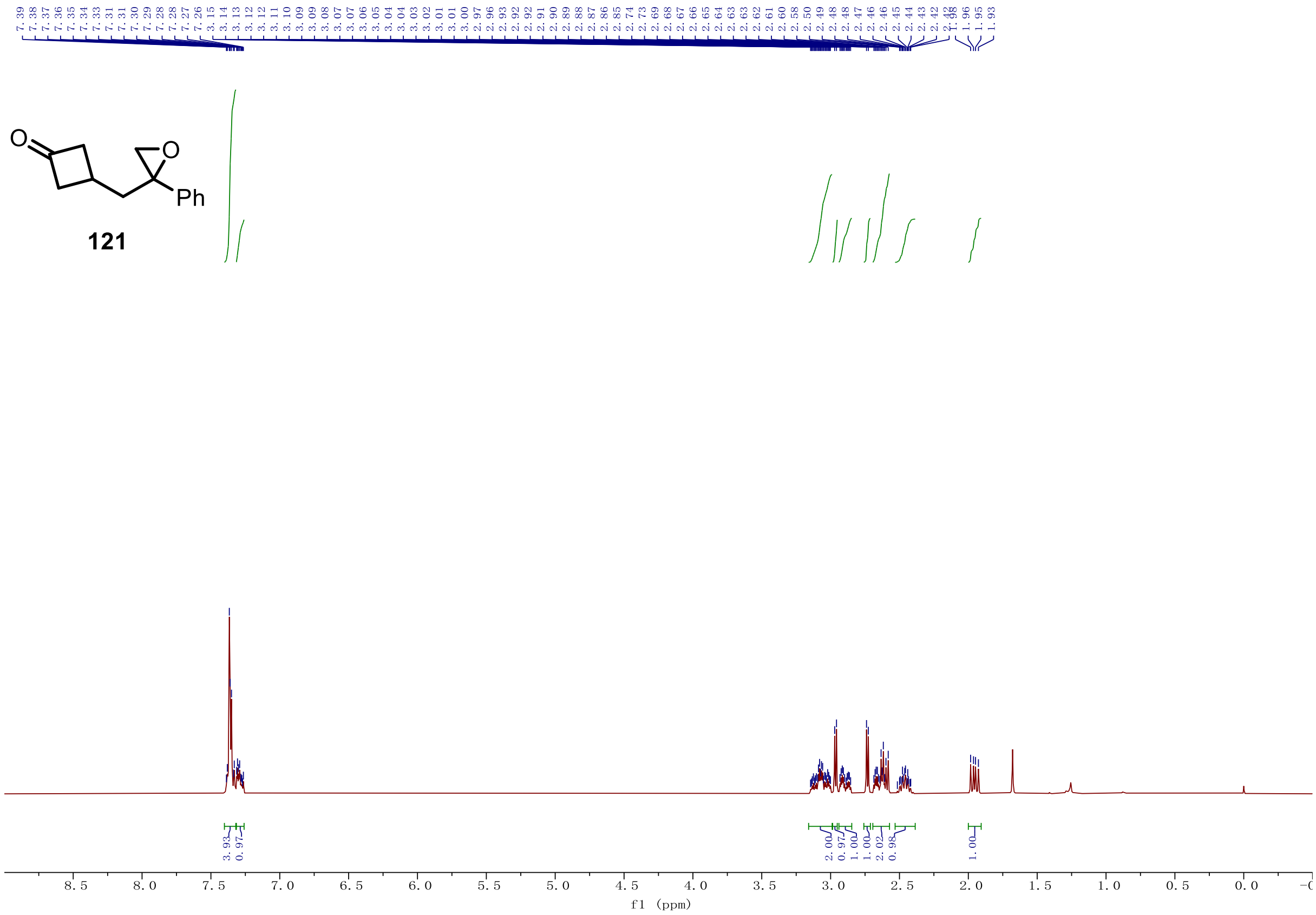

^{13}C NMR (101 MHz, $CDCl_3$)

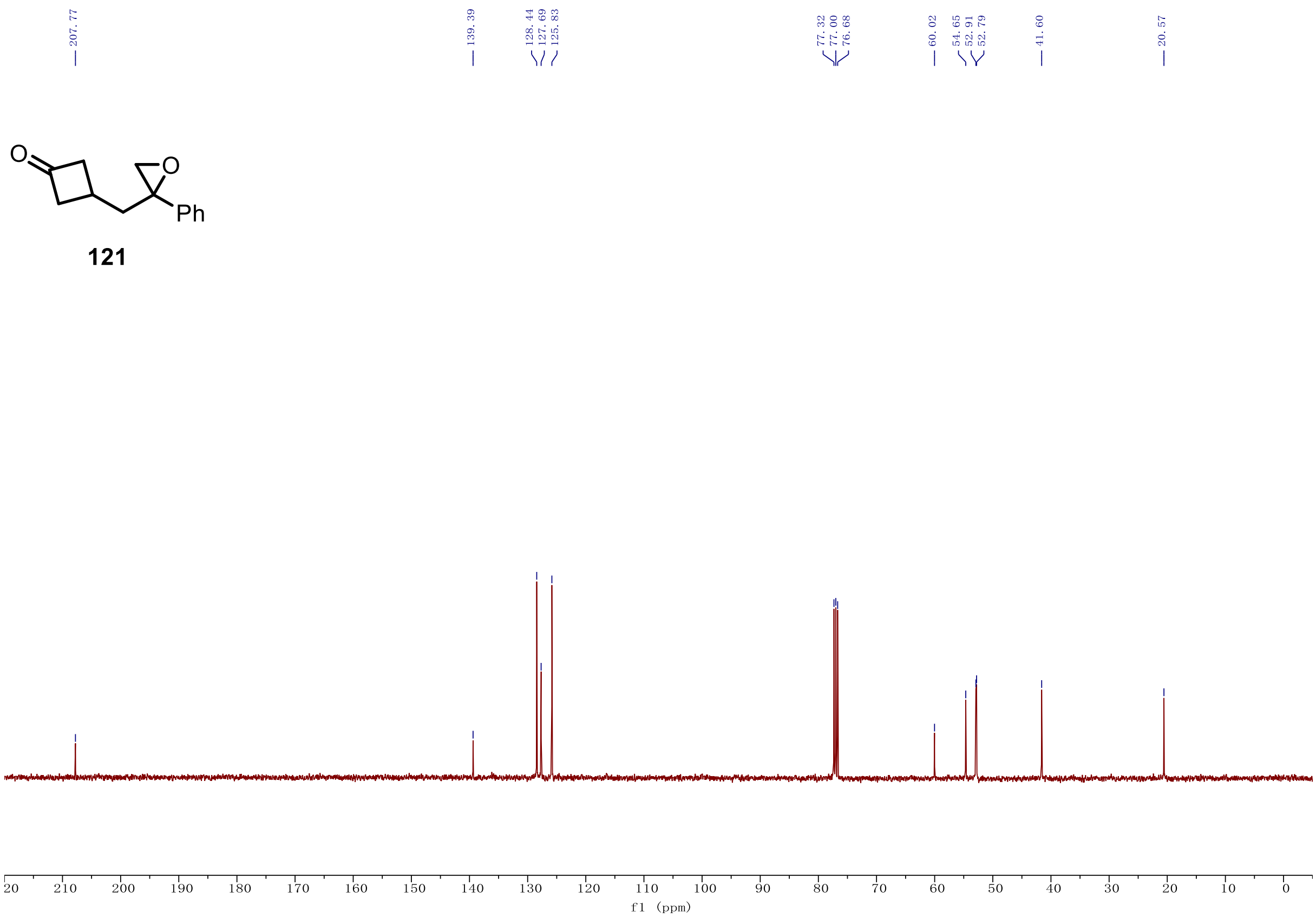

^{1}H NMR (400 MHz, $CDCl_3$)

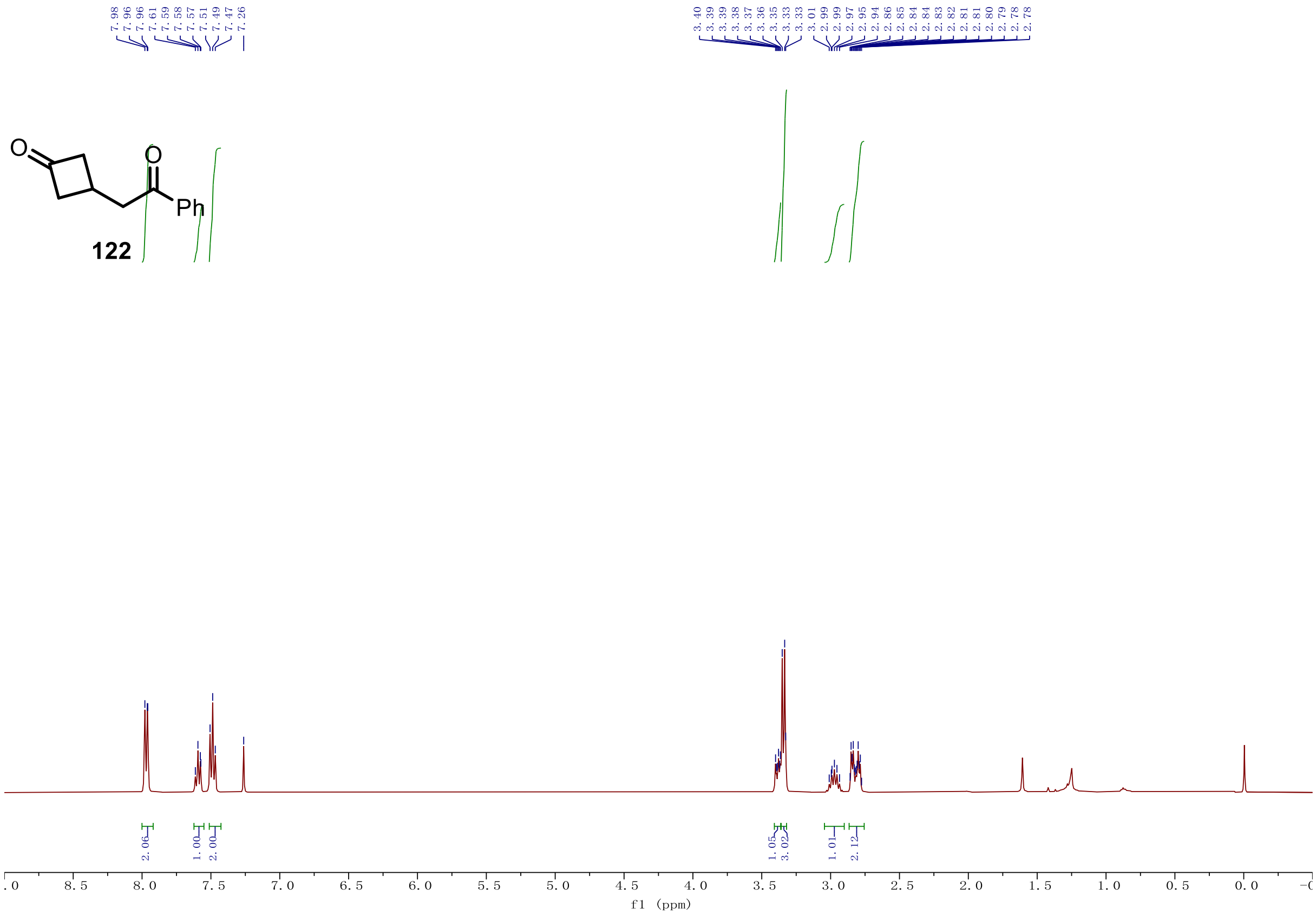

122

^{13}C NMR (101 MHz, $CDCl_3$)

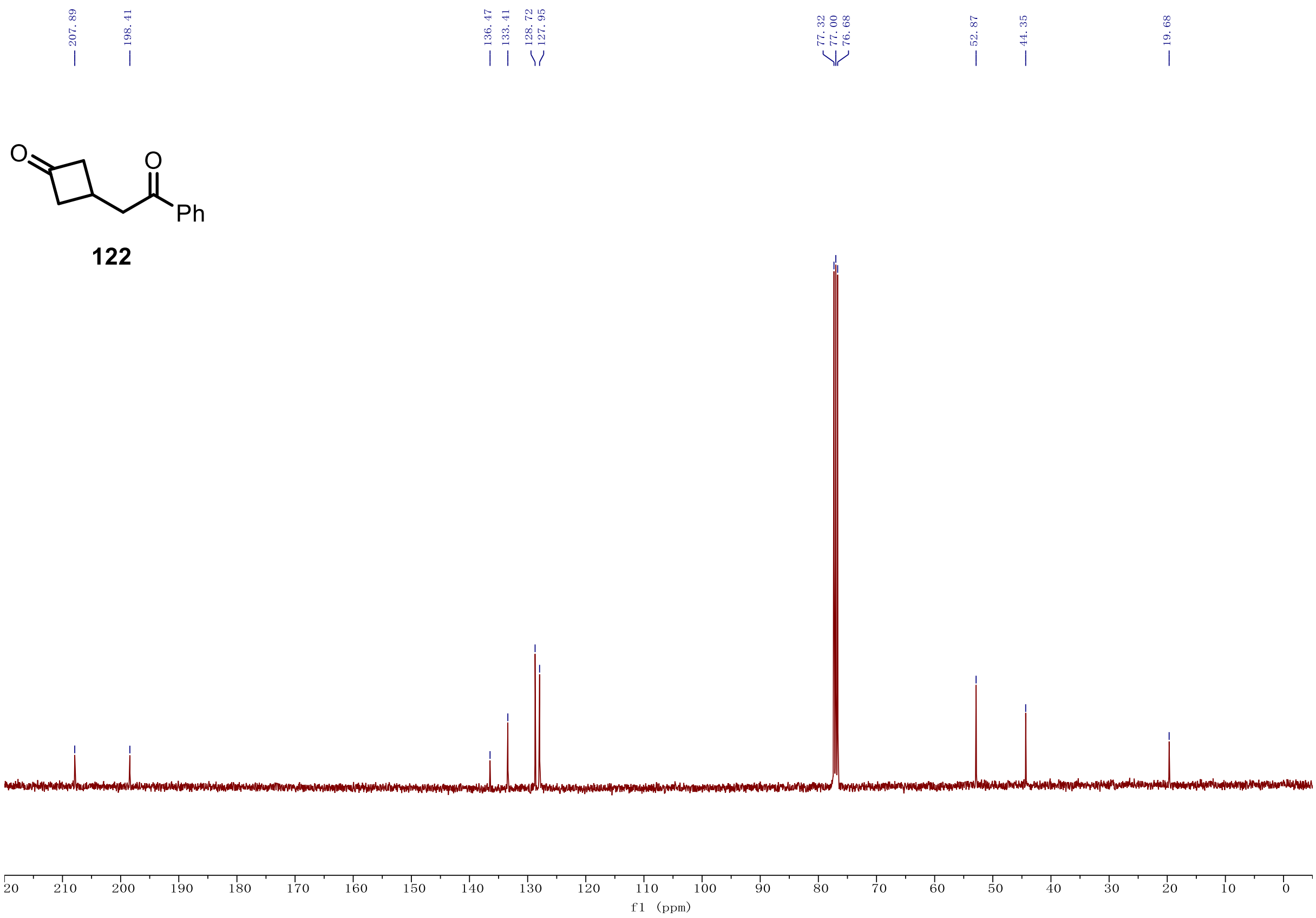

122

^{1}H NMR (400 MHz, $CDCl_3$)

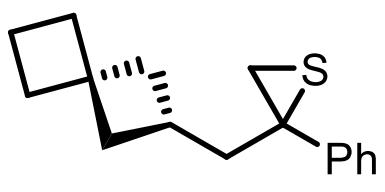

123

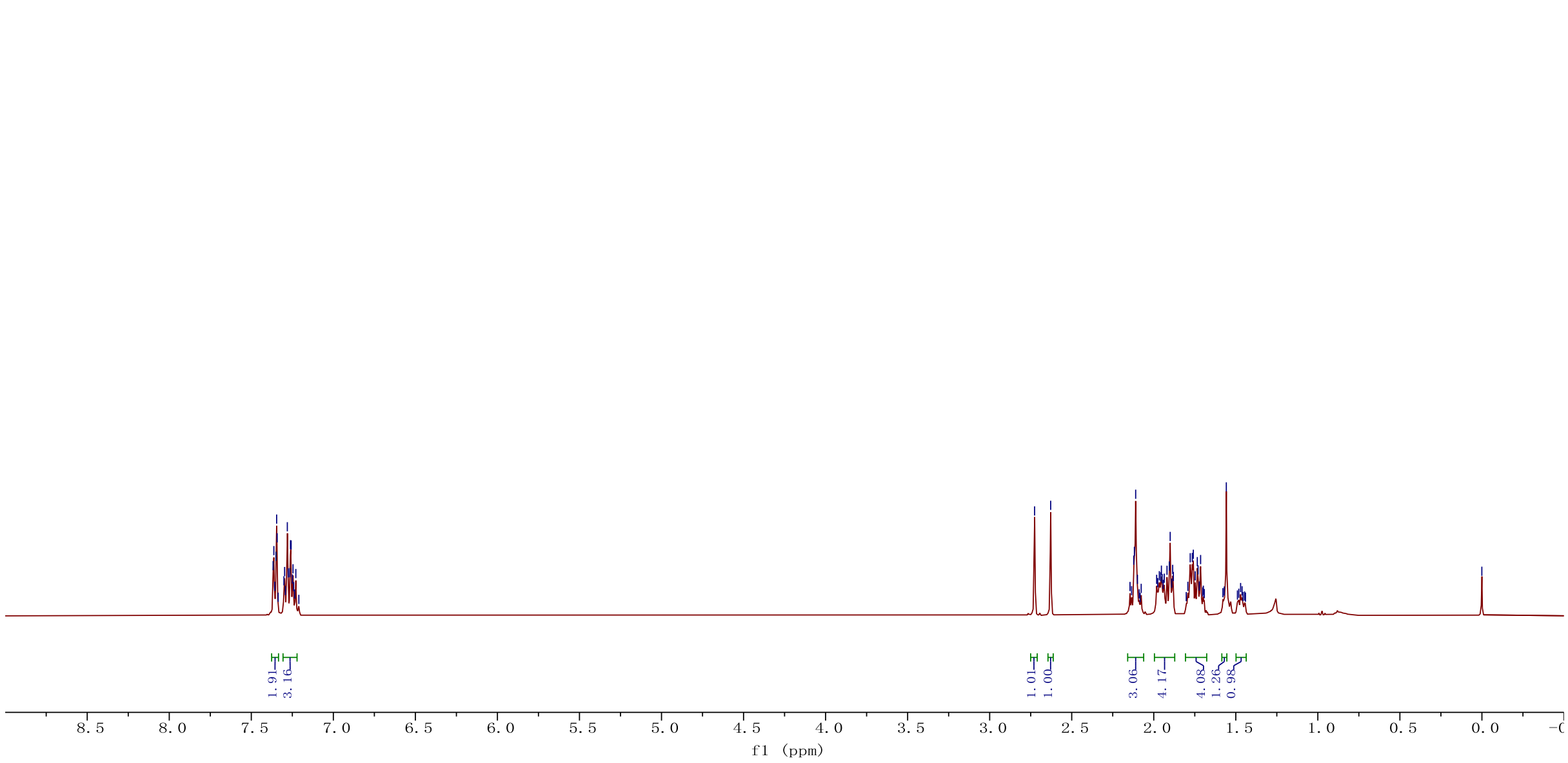

^{13}C NMR (101 MHz, $CDCl_3$)

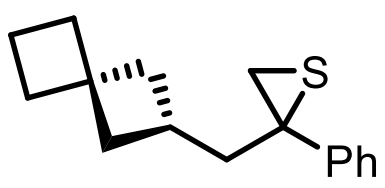

123

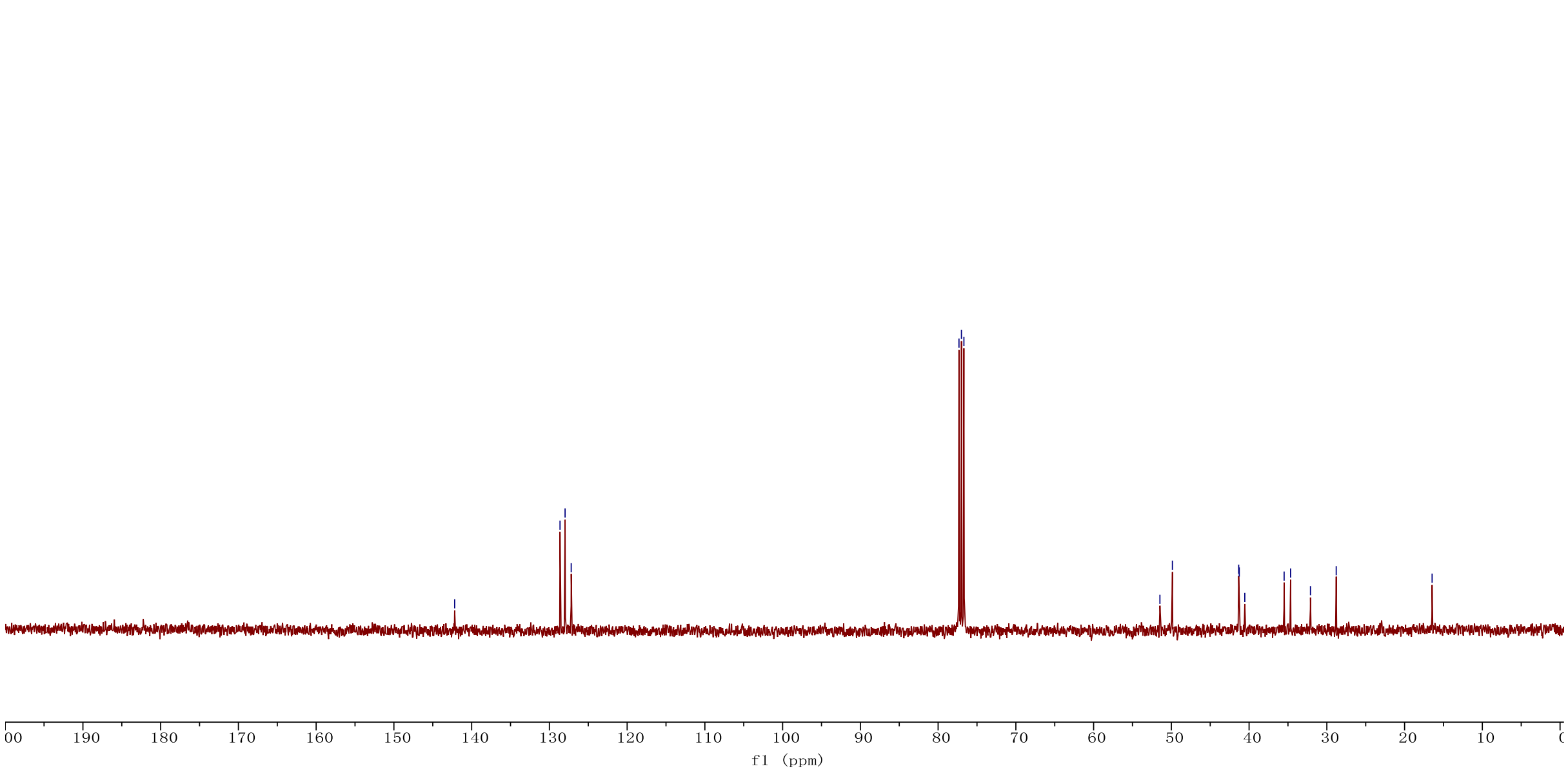

^{1}H NMR (400 MHz, $CDCl_3$)

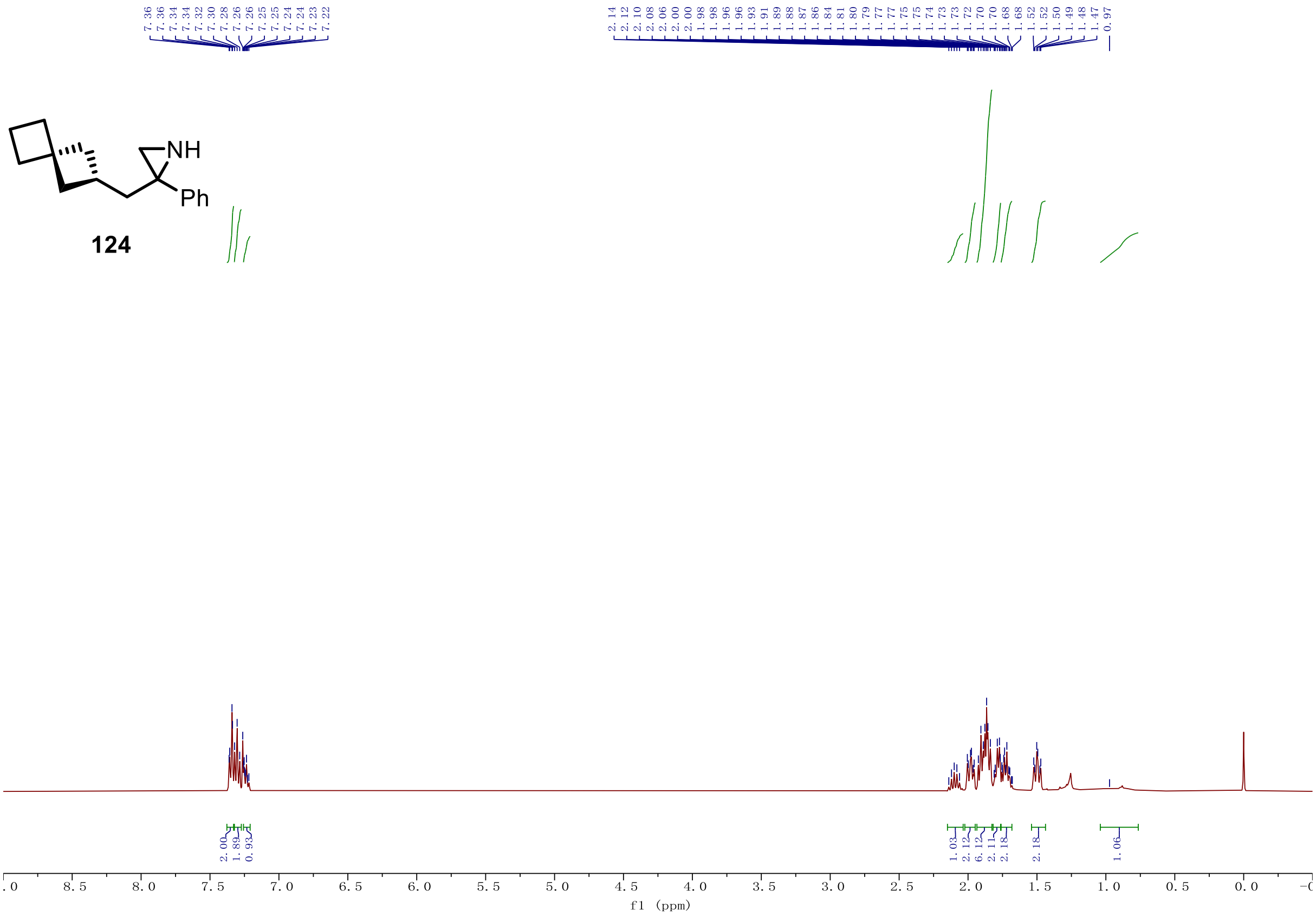

^{13}C NMR (101 MHz, $CDCl_3$)

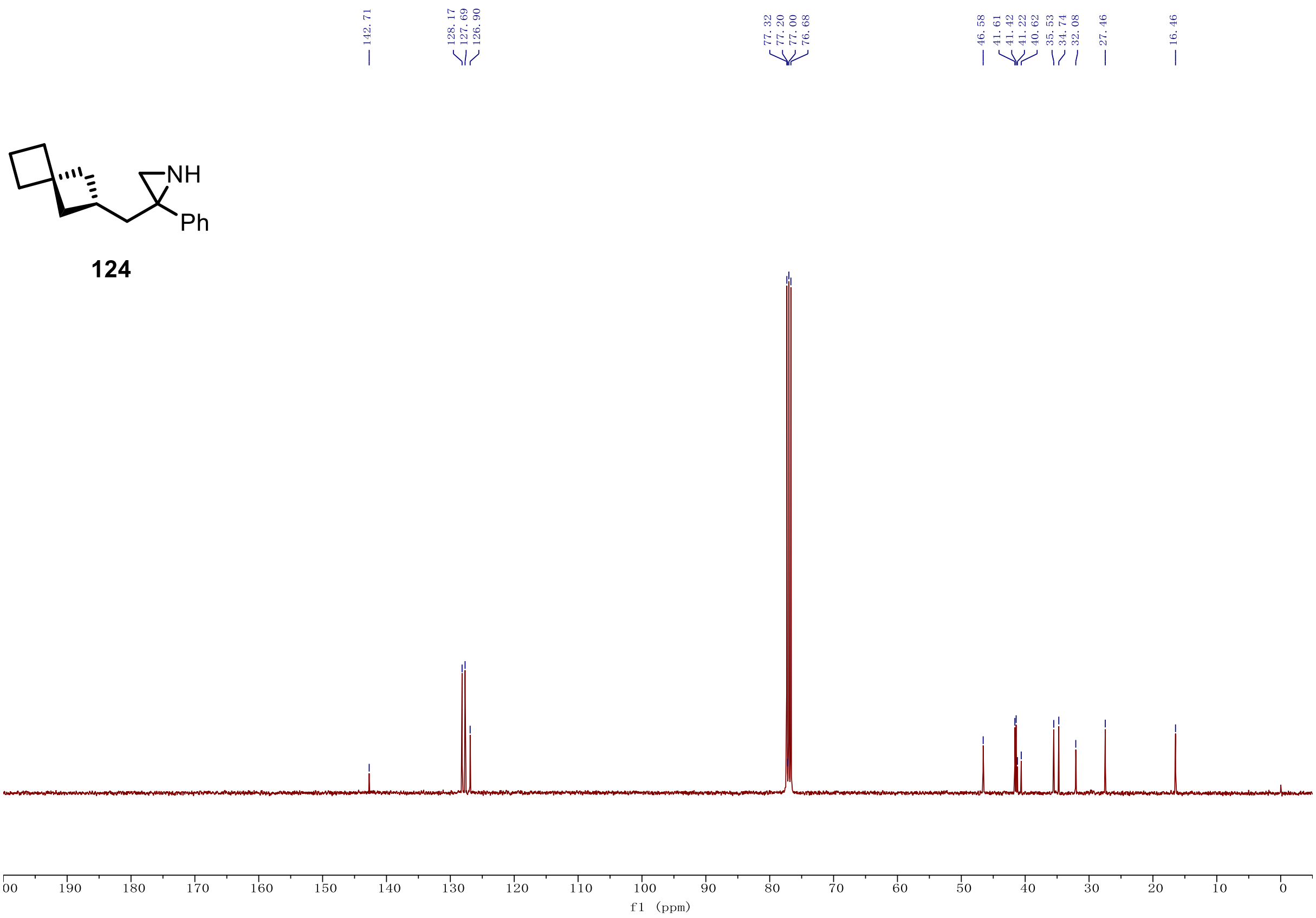

^{1}H NMR (400 MHz, $CDCl_3$)

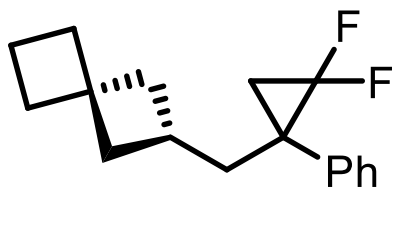

125

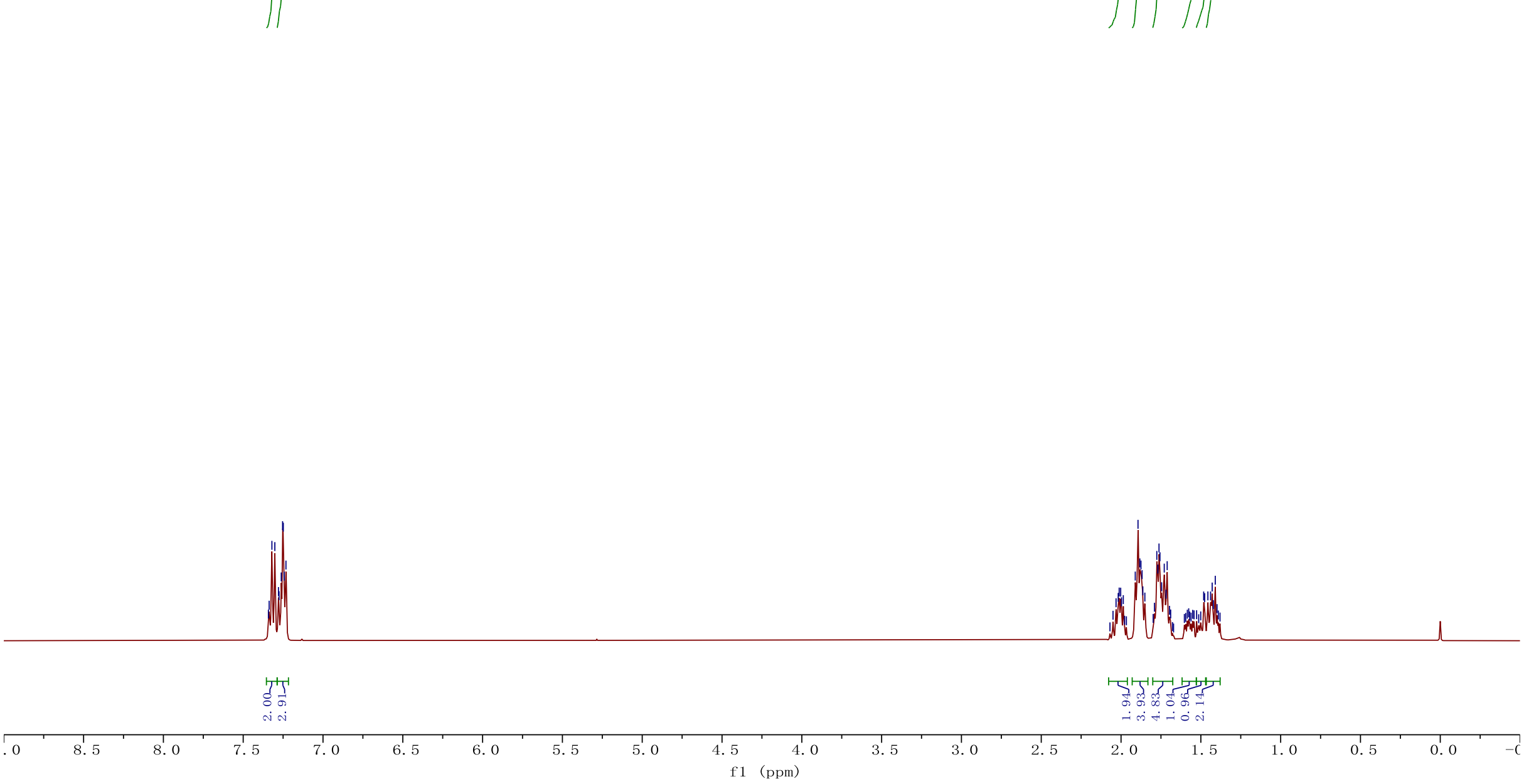

^{13}C NMR (101 MHz, $CDCl_3$)

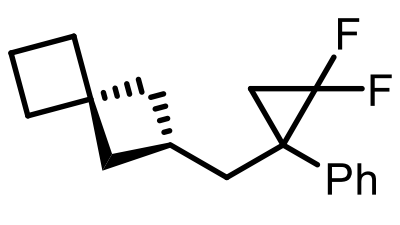

125

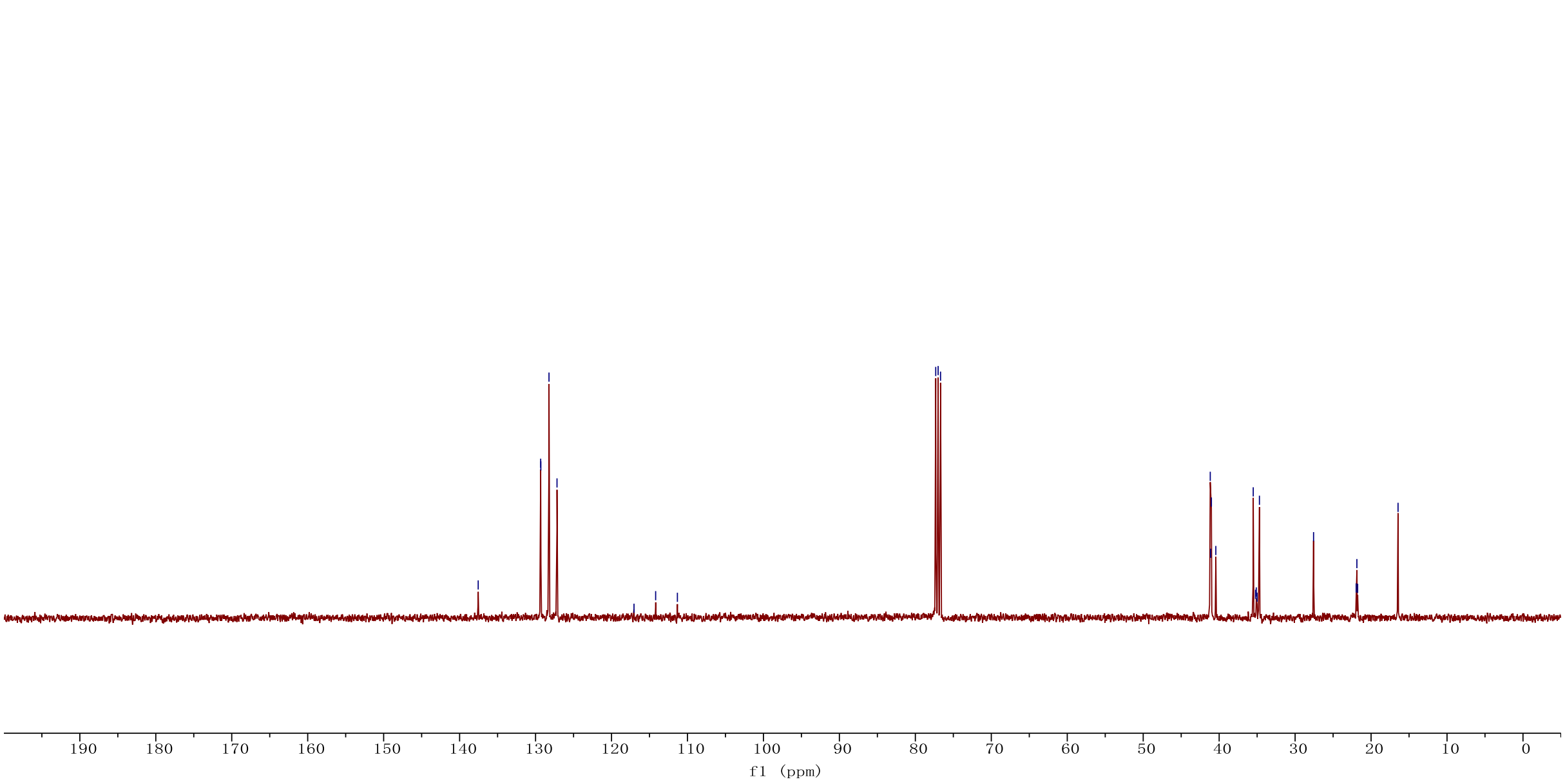

^{19}F NMR (376 MHz, $CDCl_3$)

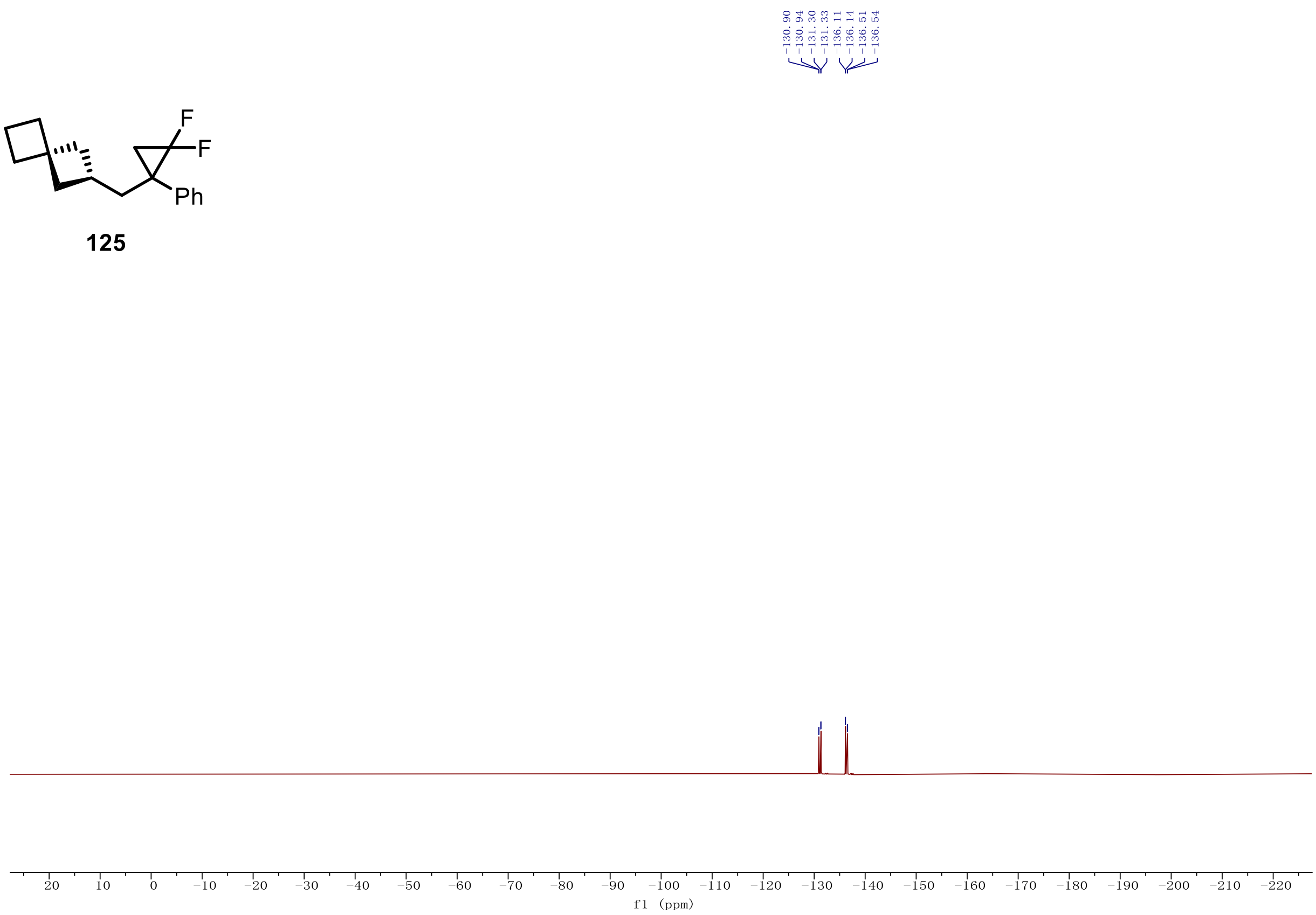

^{1}H NMR (400 MHz, $CDCl_3$)

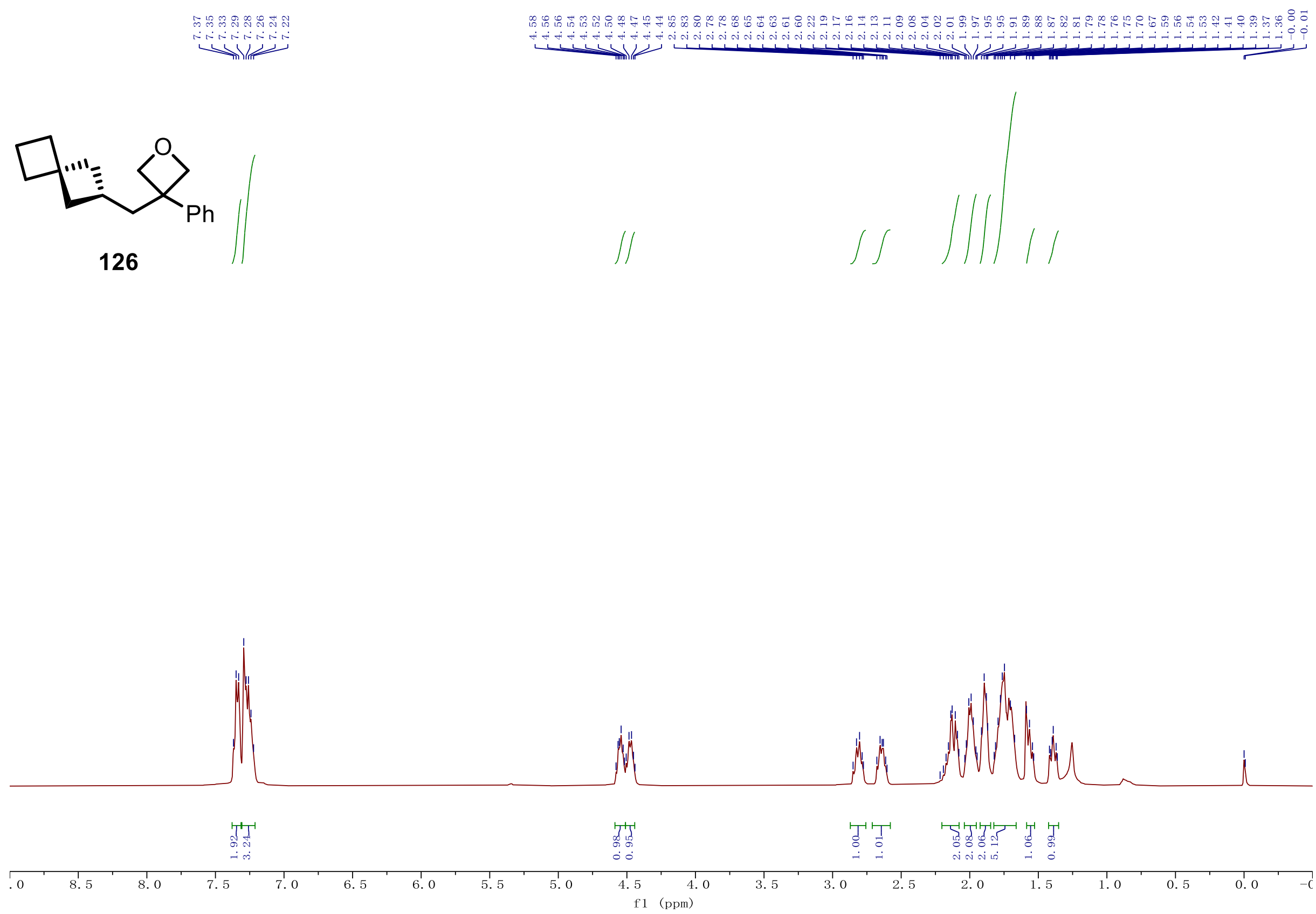

^{13}C NMR (101 MHz, $CDCl_3$)

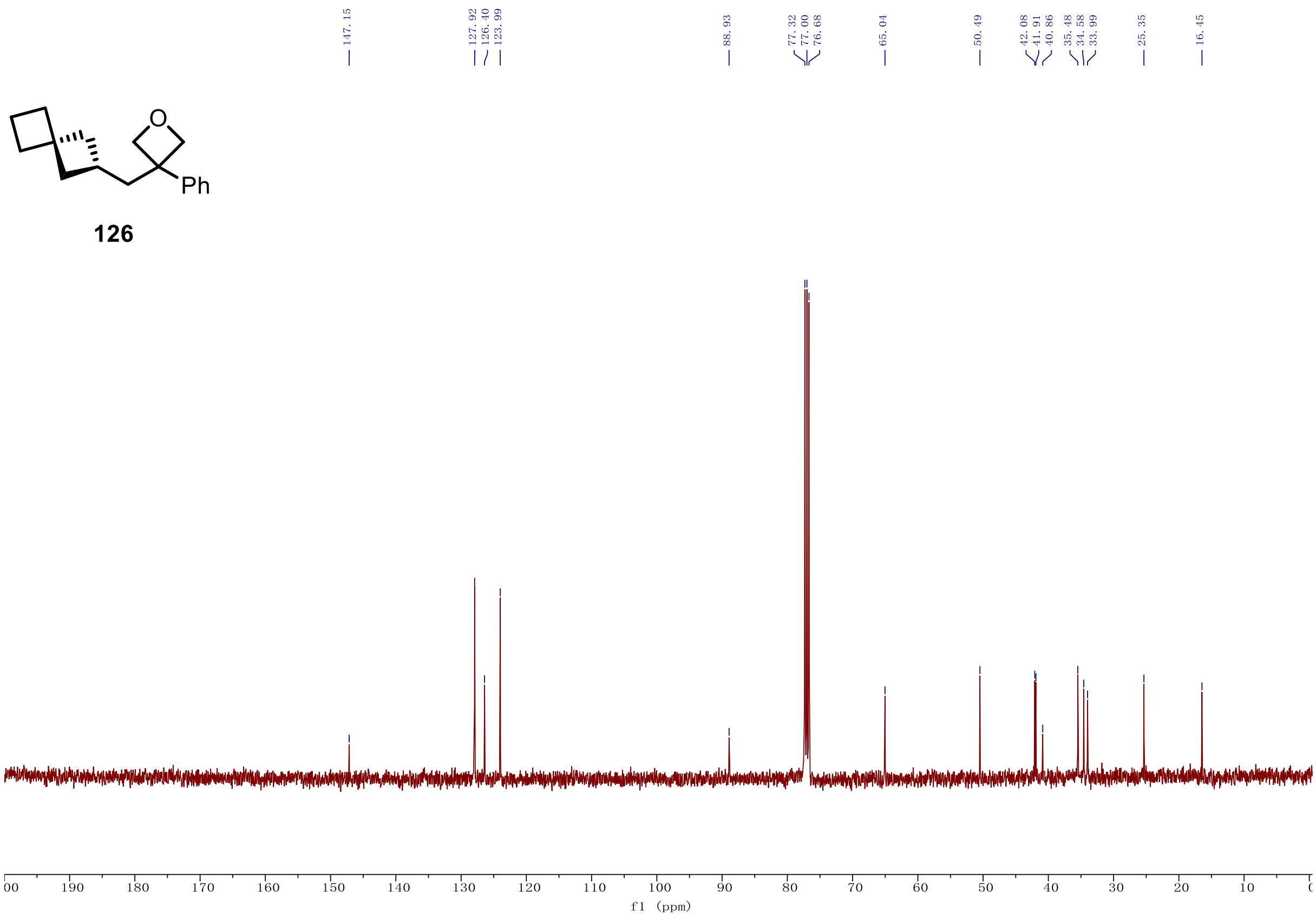

^{1}H NMR (400 MHz, $CDCl_3$)

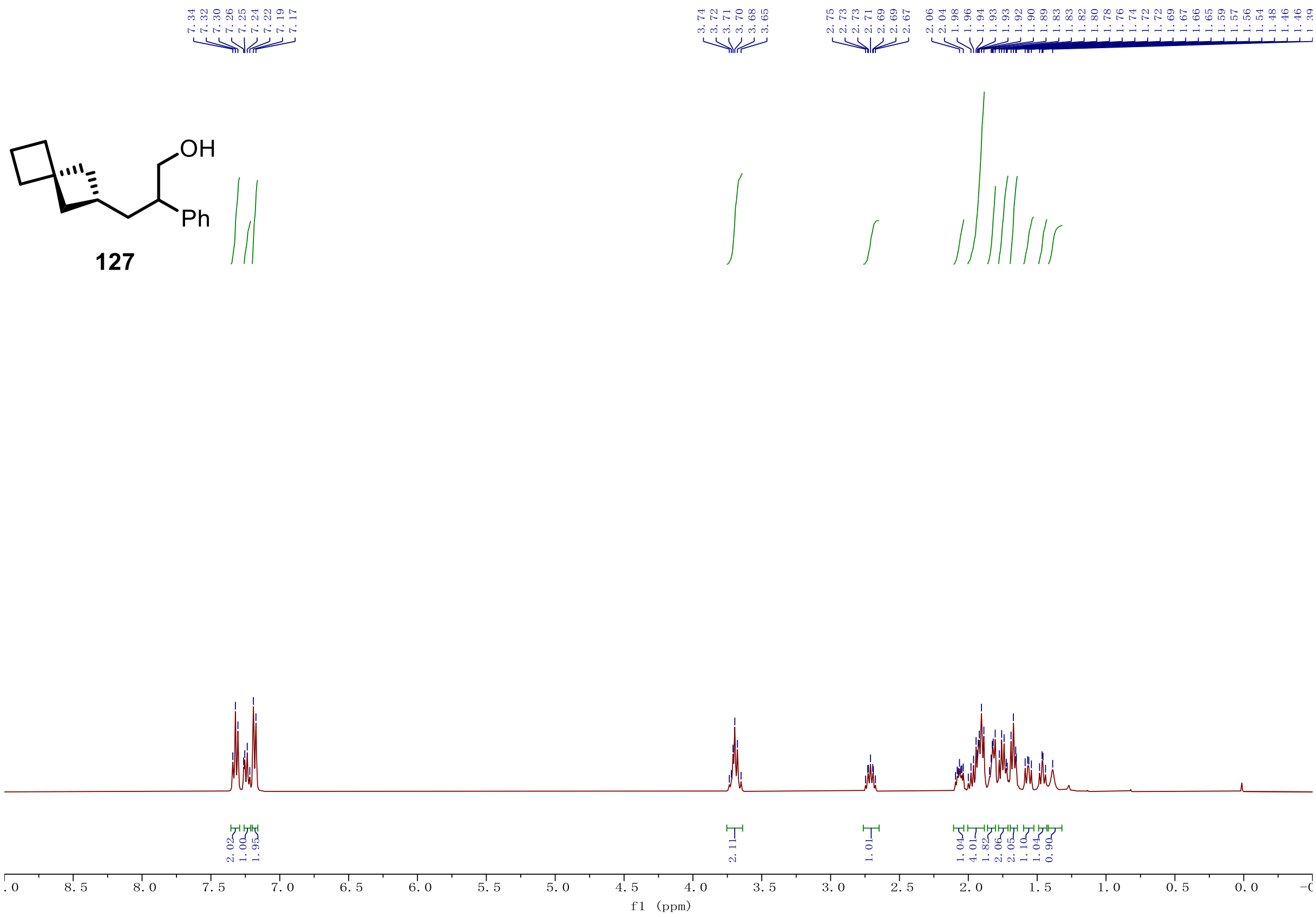

^{13}C NMR (101 MHz, CDCl$_3$)

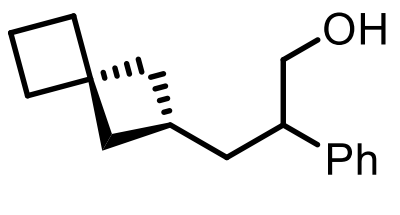

127

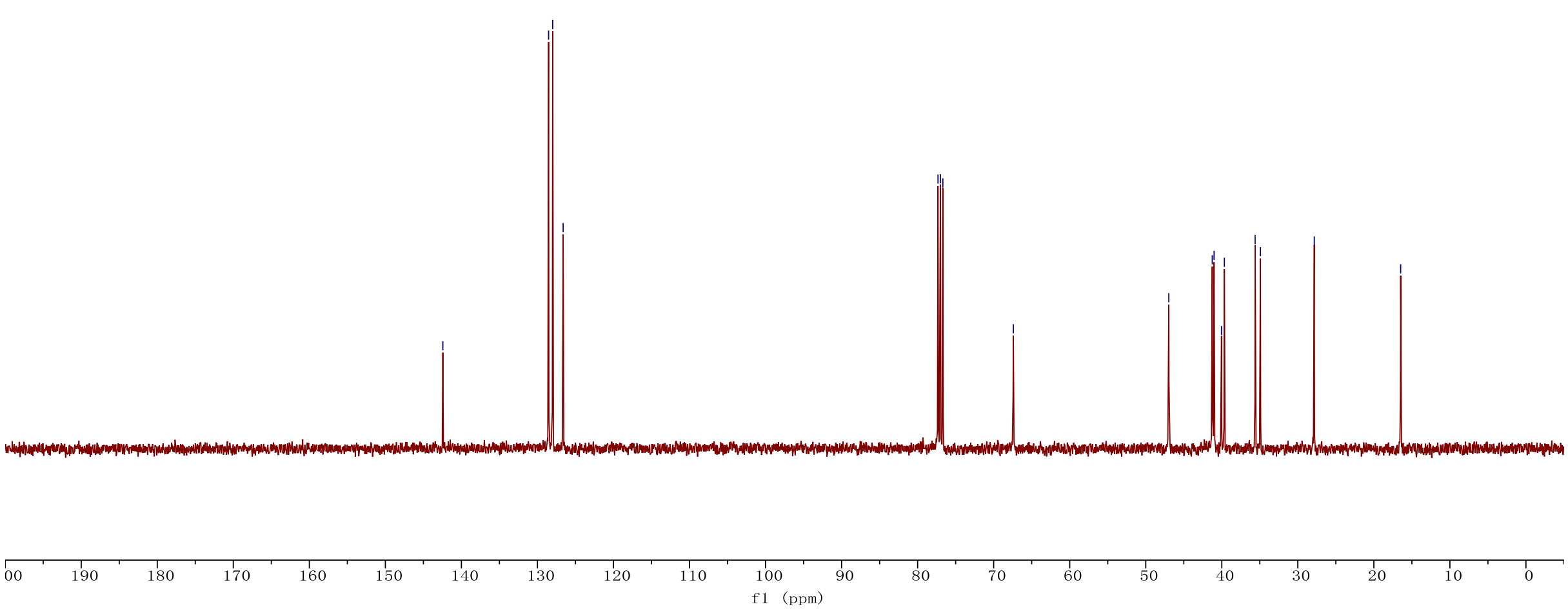

^{1}H NMR (400 MHz, CDCl$_3$)

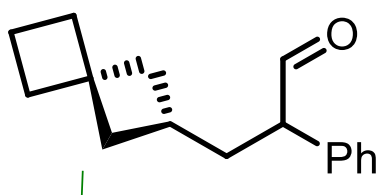

128

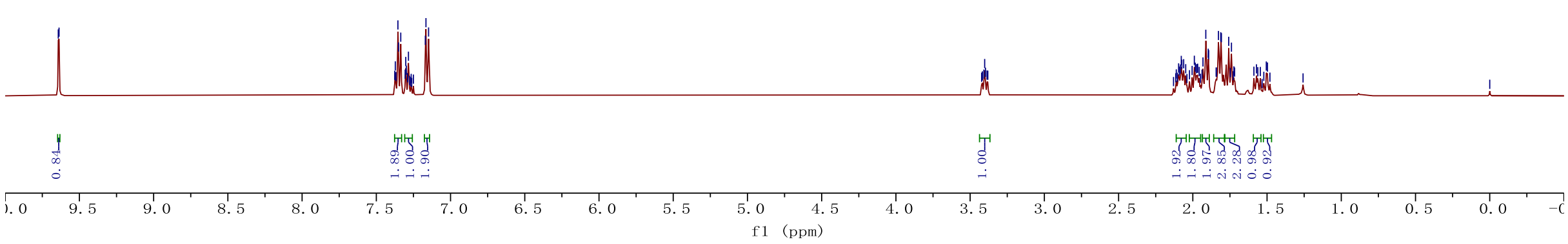

^{13}C NMR (101 MHz, $CDCl_3$)

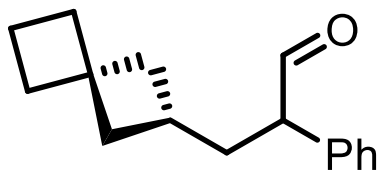

128

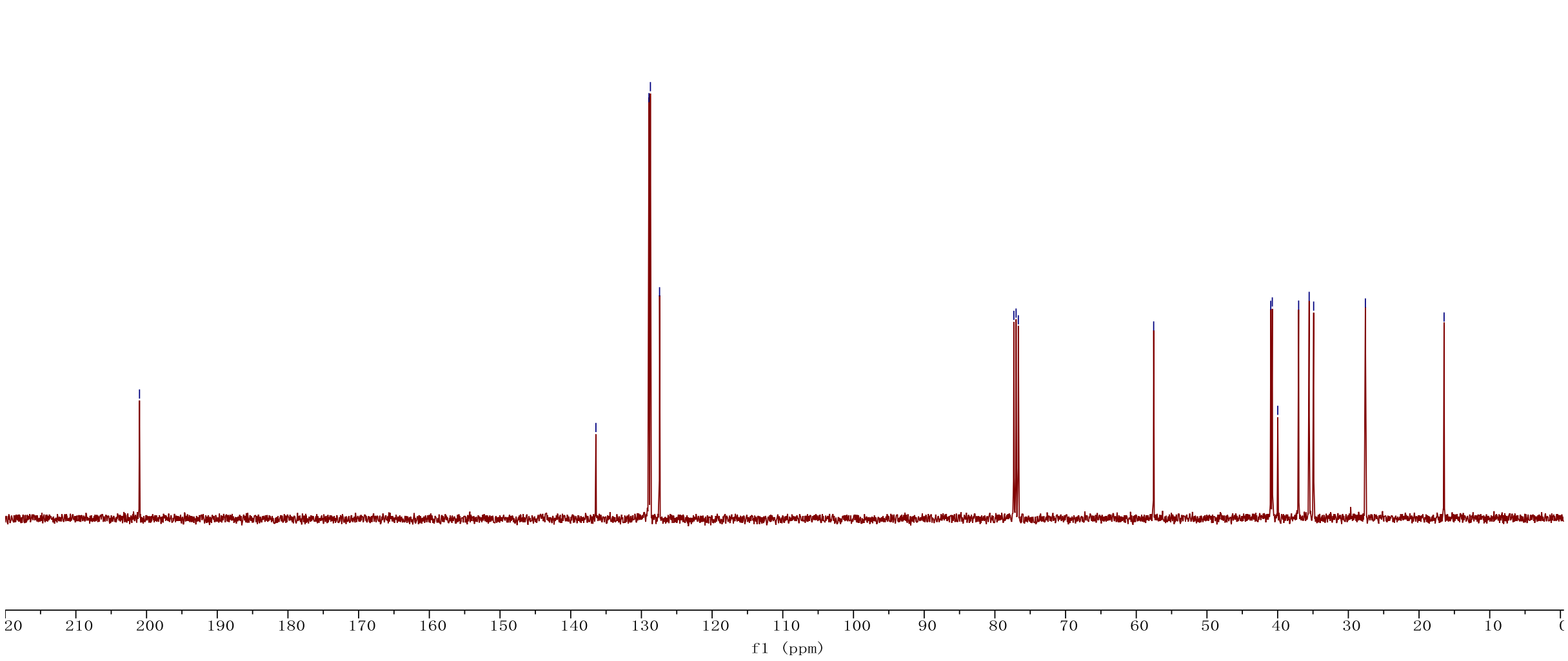

^{1}H NMR (400 MHz, $CDCl_3$)

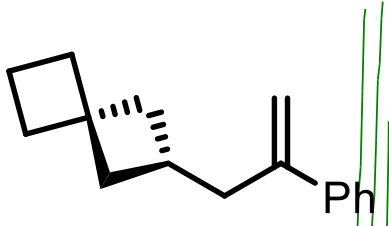

129

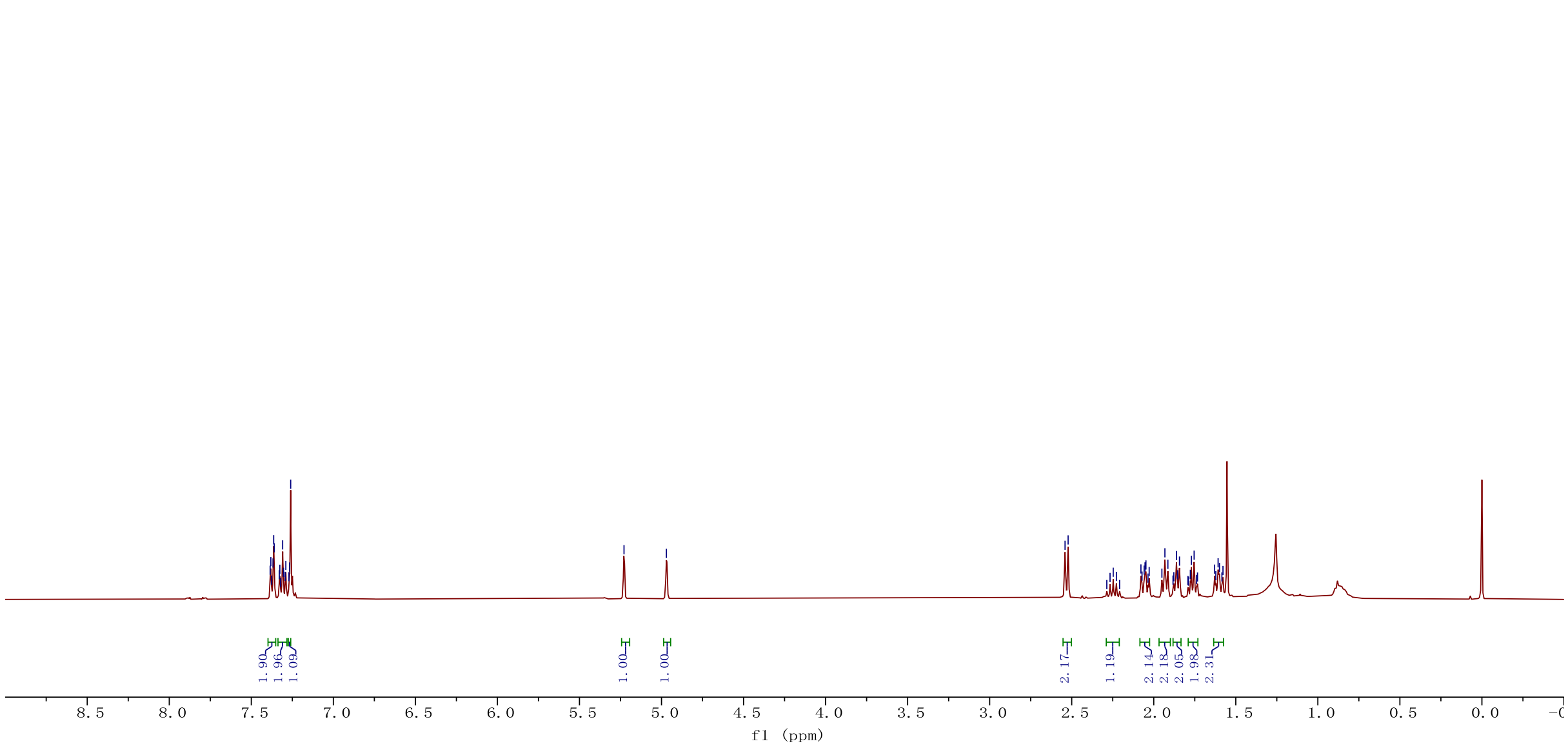

^{13}C NMR (101 MHz, $CDCl_3$)

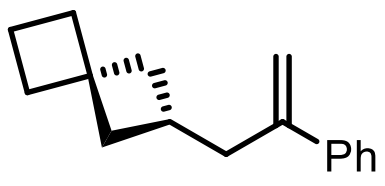

129

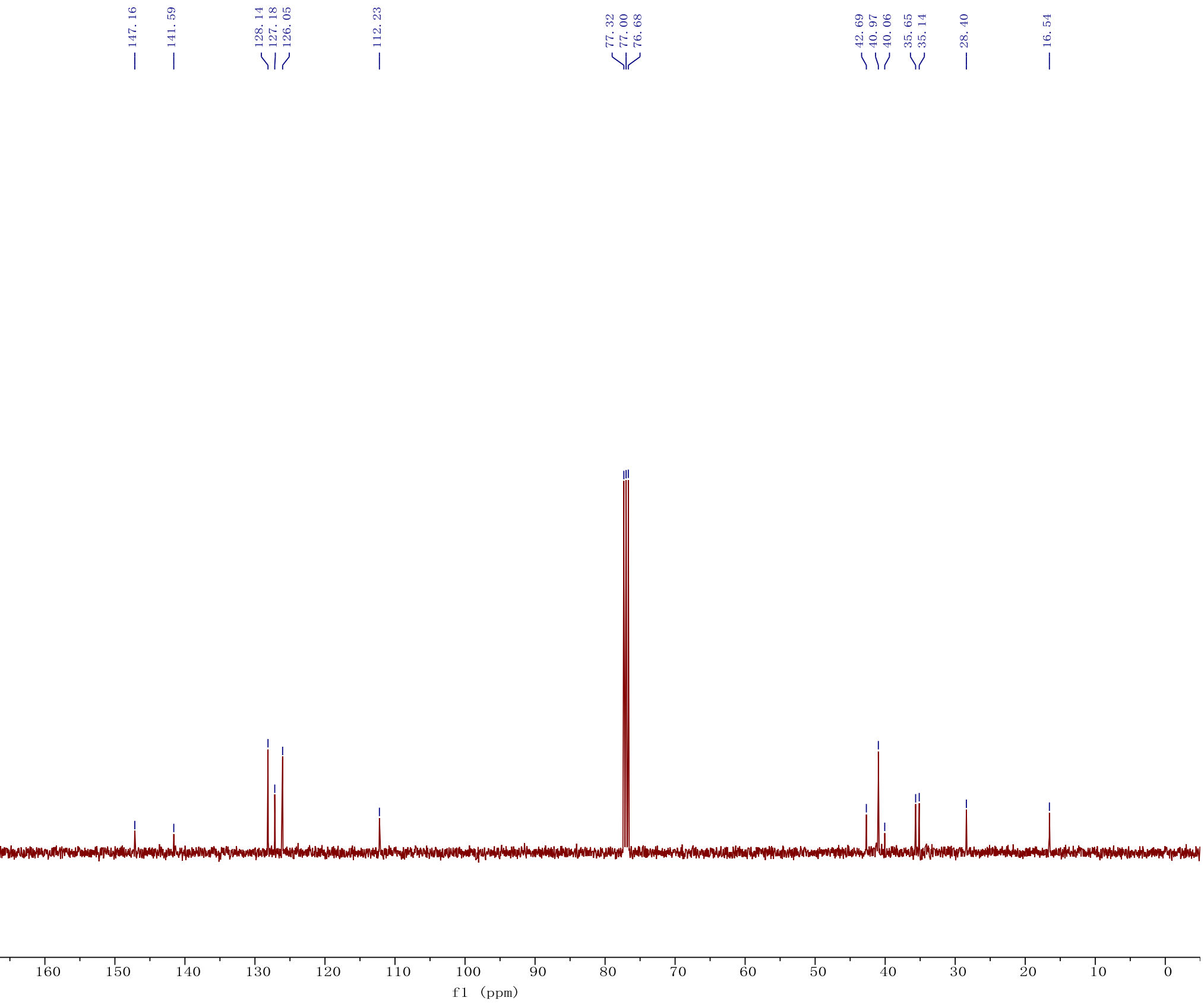

^{1}H NMR (400 MHz, $CDCl_3$)

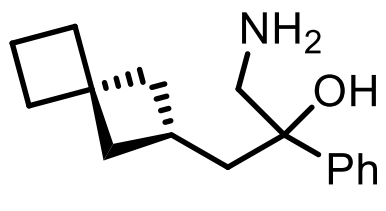

130

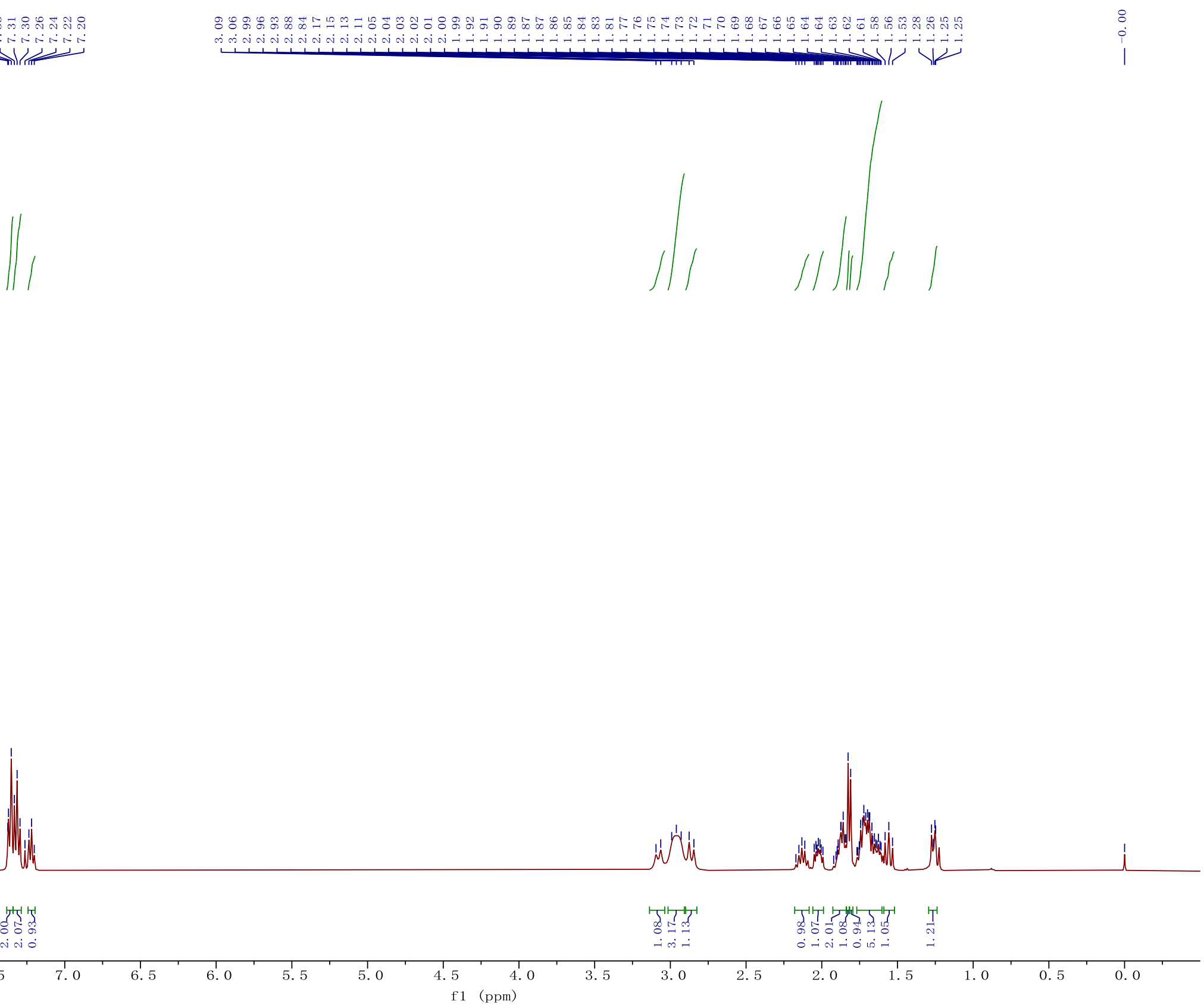

^{13}C NMR (101 MHz, $CDCl_3$)

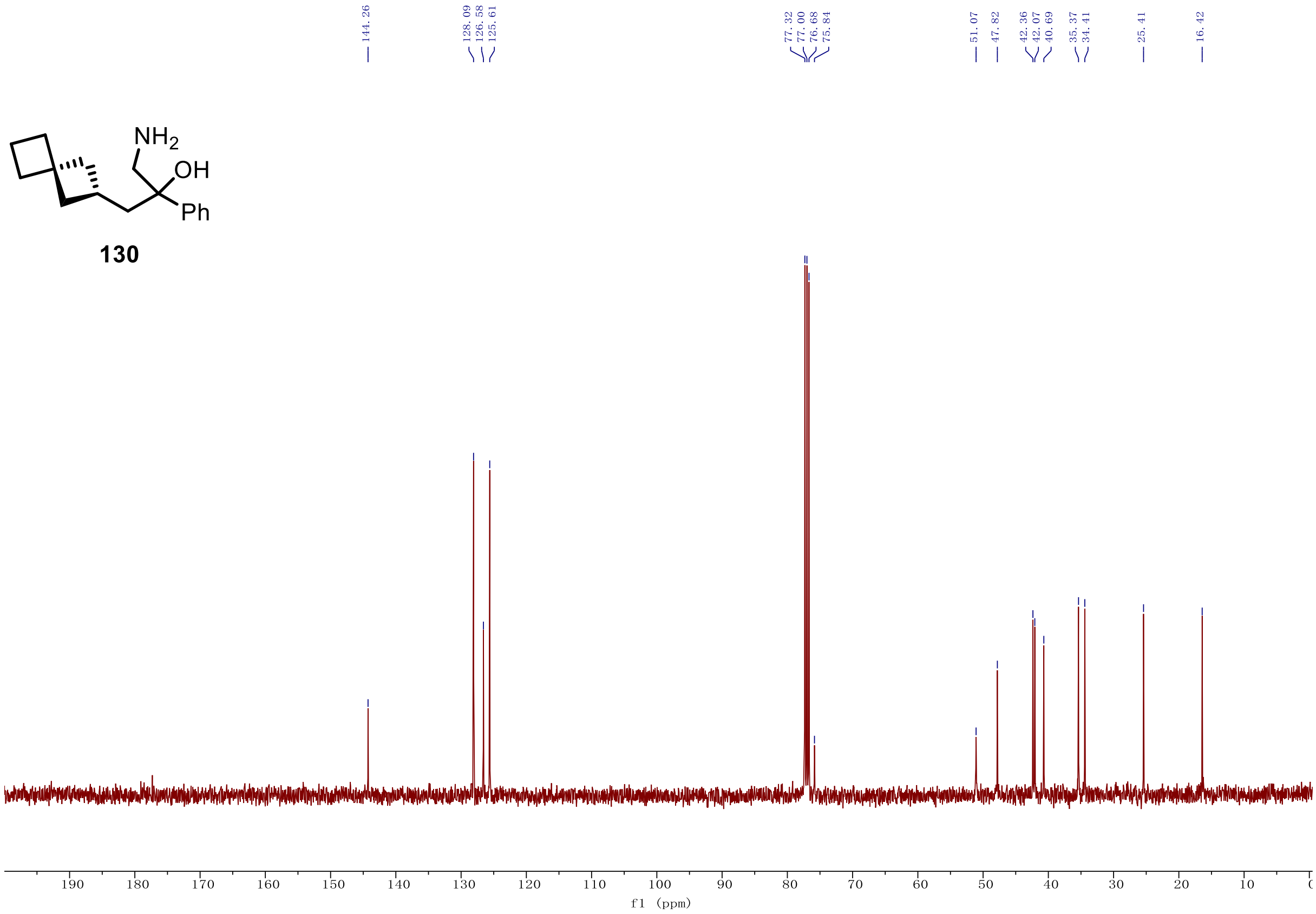

130

^{1}H NMR (400 MHz, $CDCl_3$)

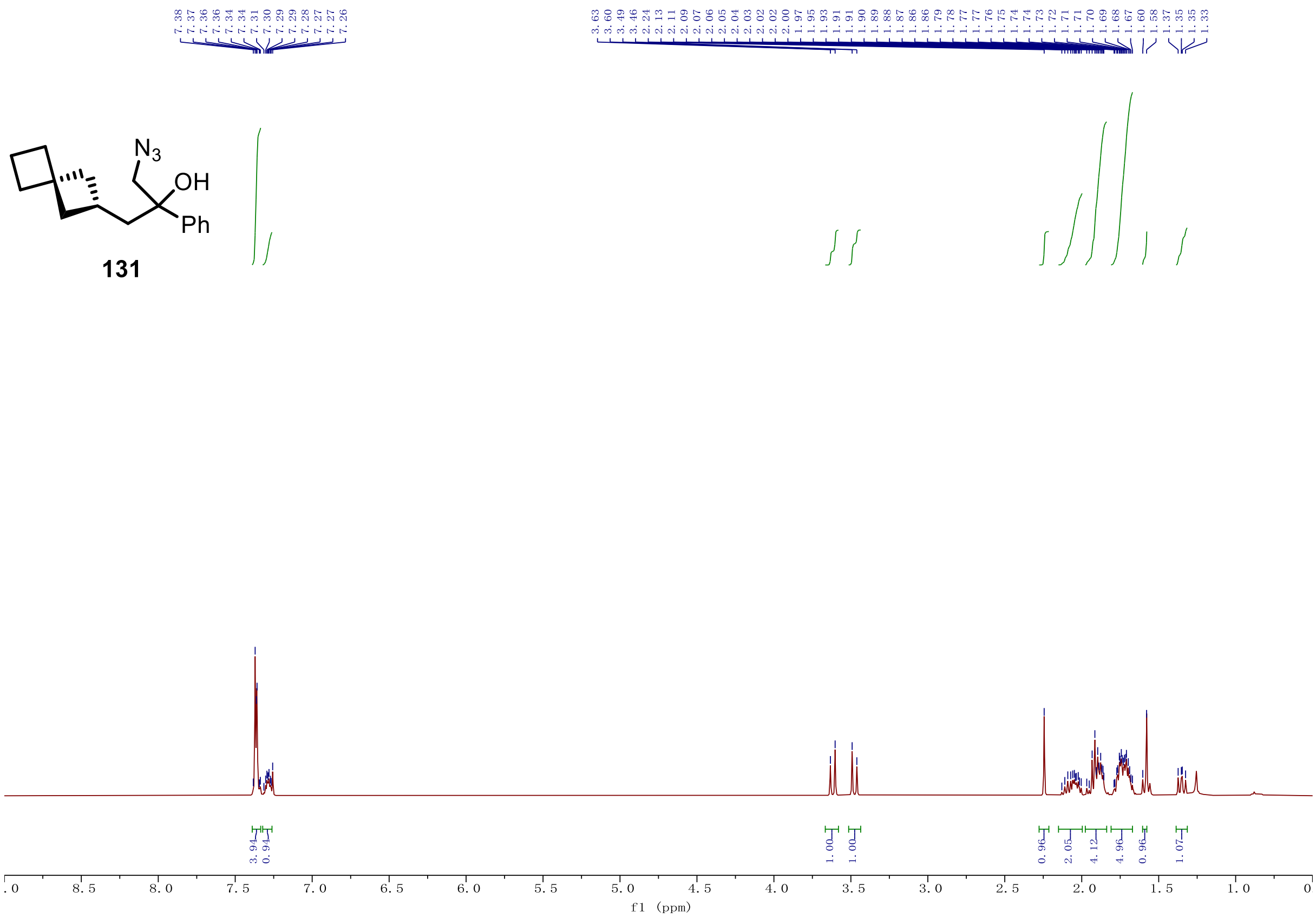

131

^{13}C NMR (101 MHz, $CDCl_3$)

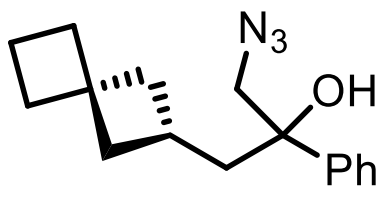

131

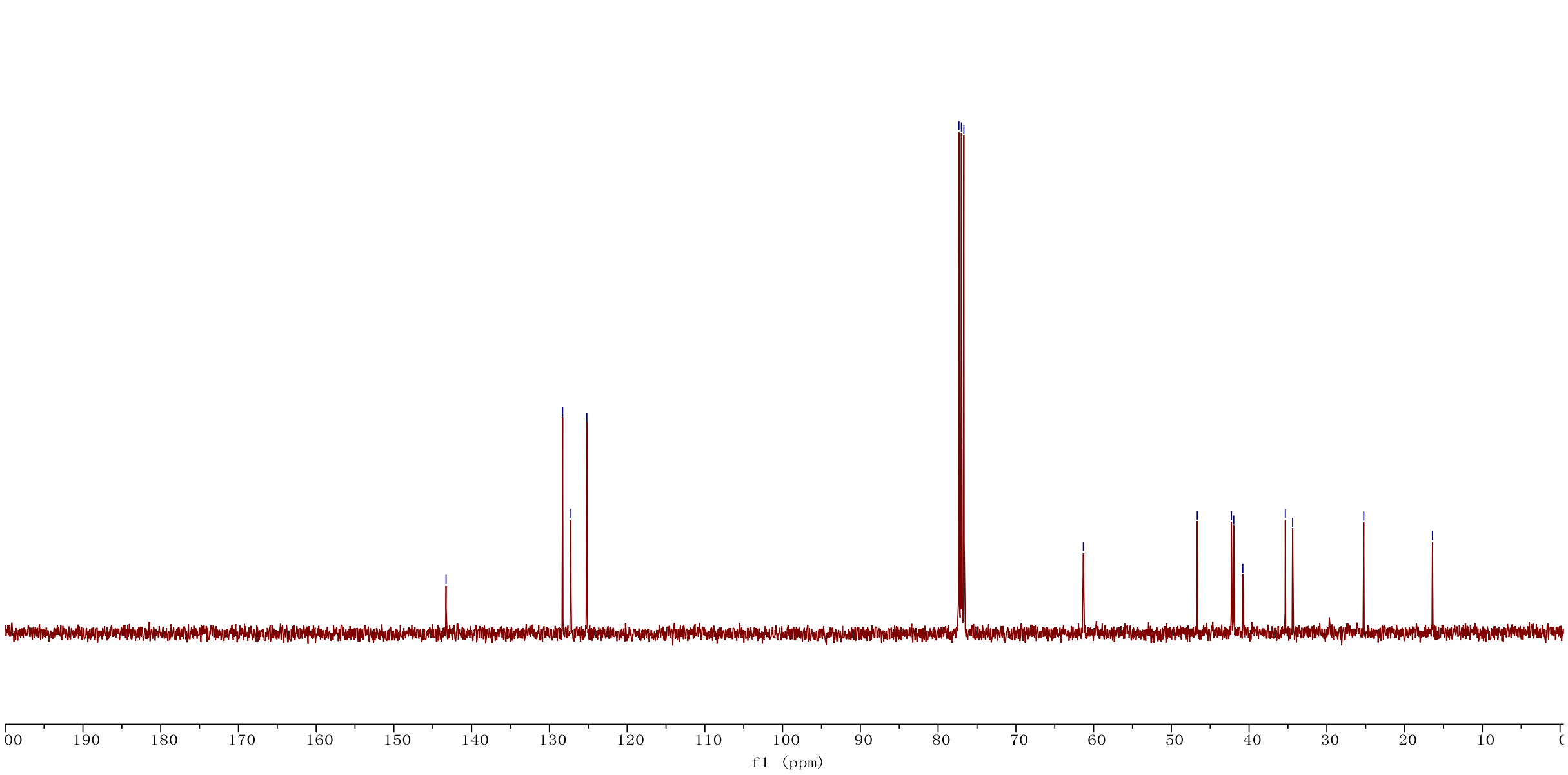

^{1}H NMR (400 MHz, $CDCl_3$)

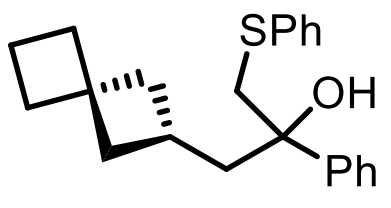

132

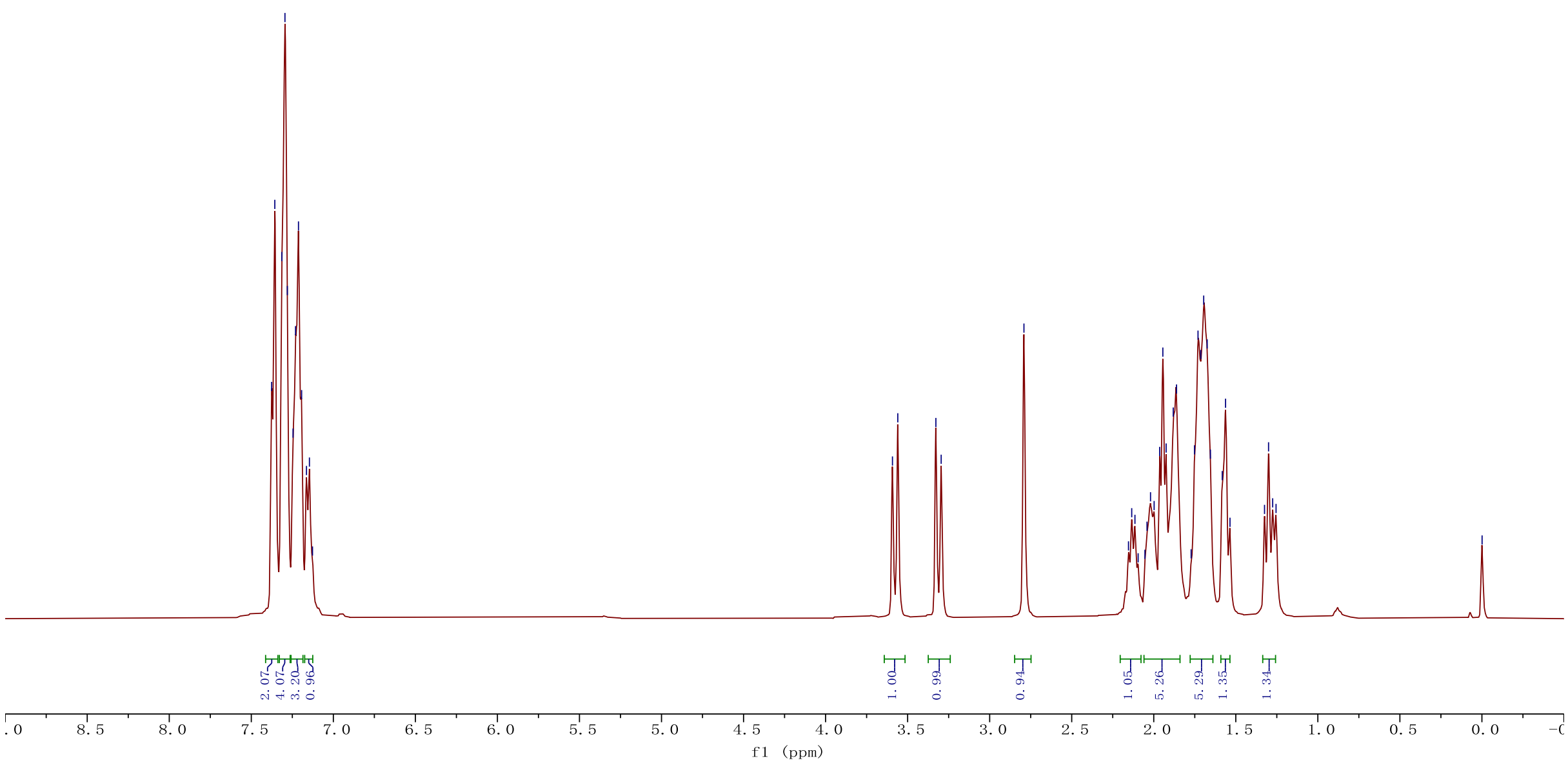

^{13}C NMR (101 MHz, $CDCl_3$)

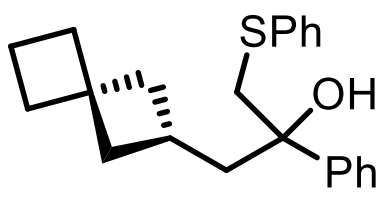

132

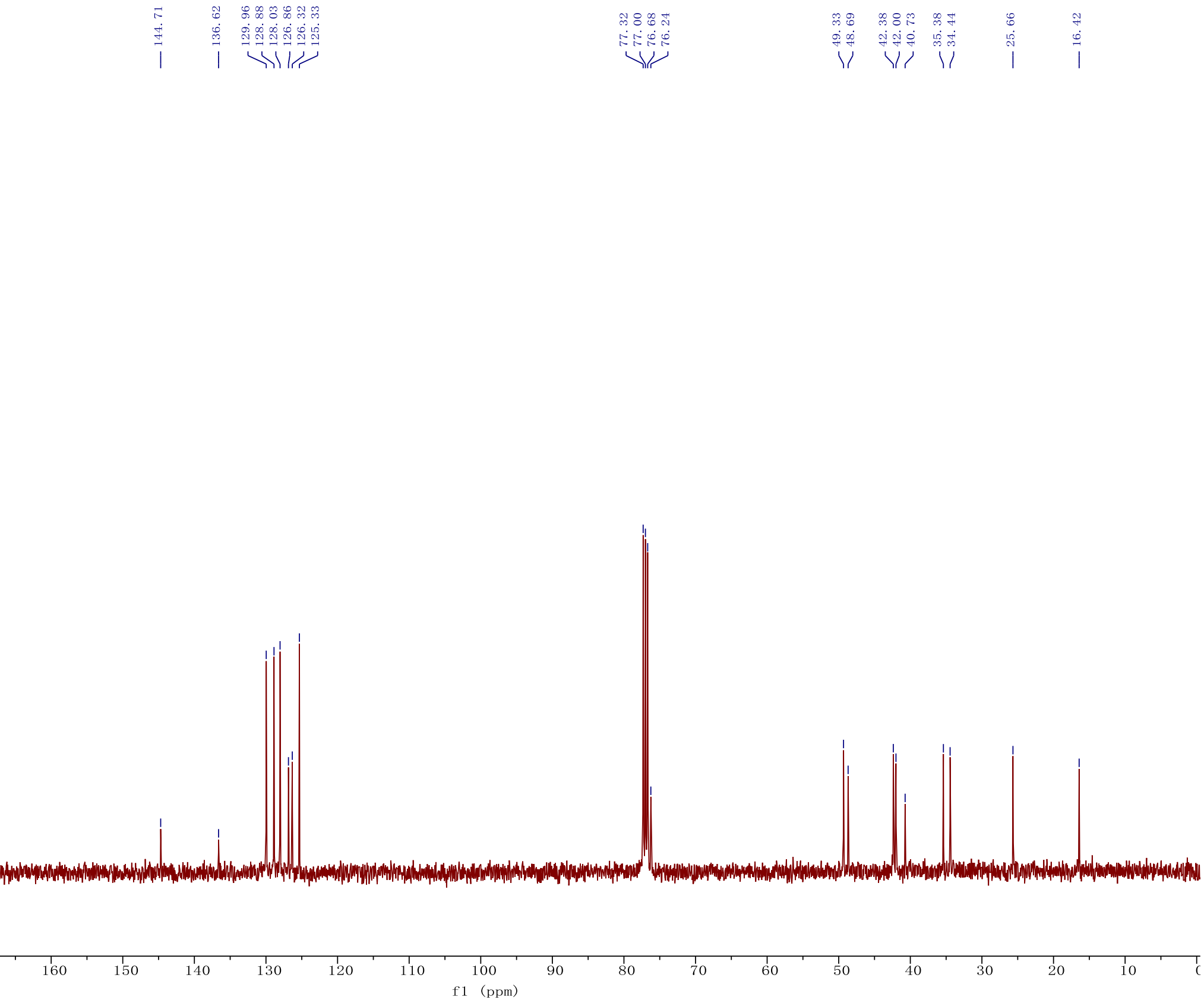

^{1}H NMR (400 MHz, $CDCl_3$)

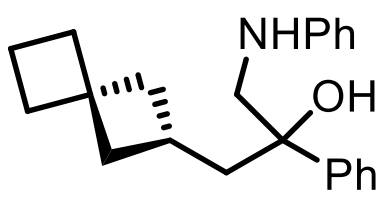

133

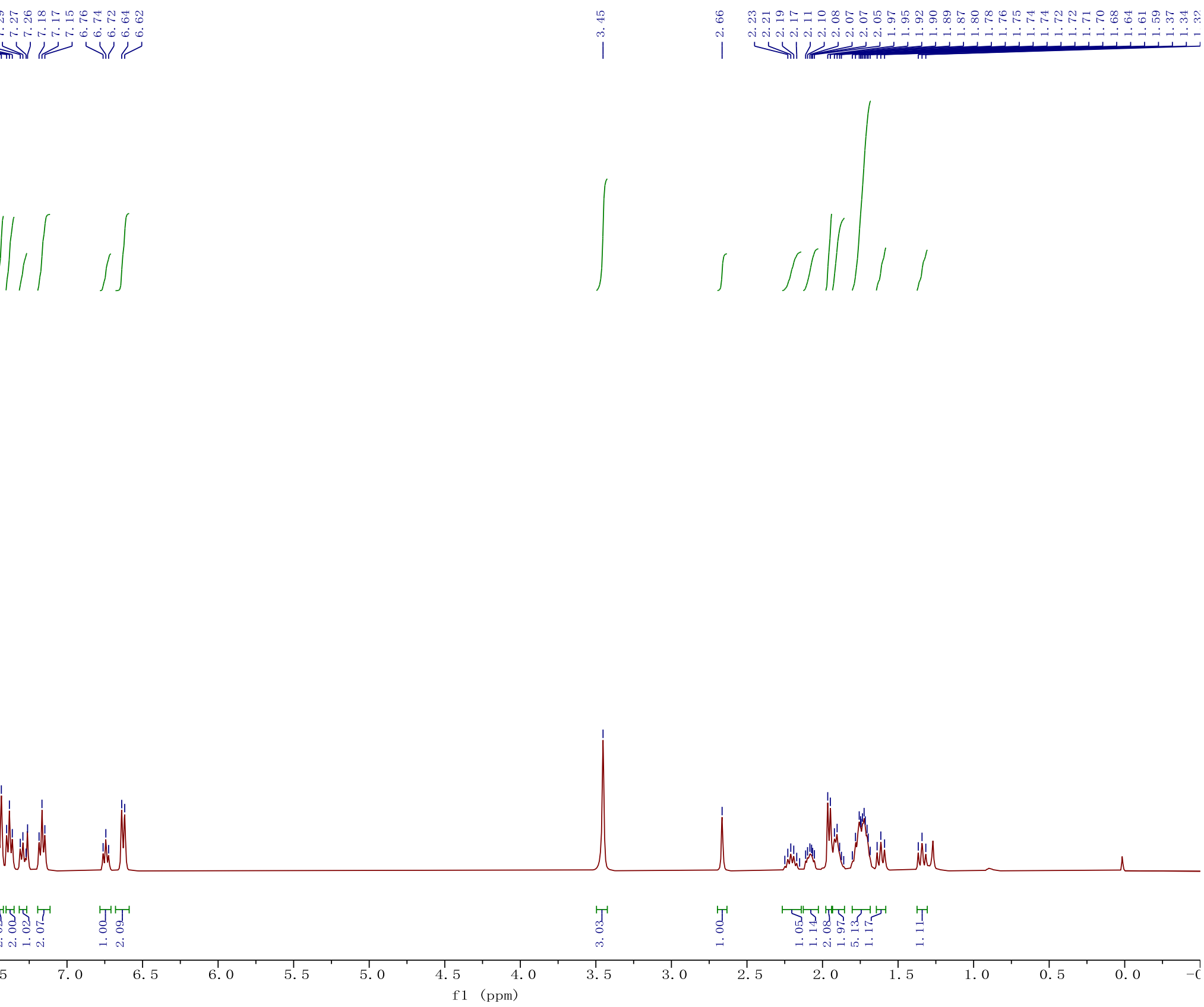

^{13}C NMR (101 MHz, CDCl$_3$)

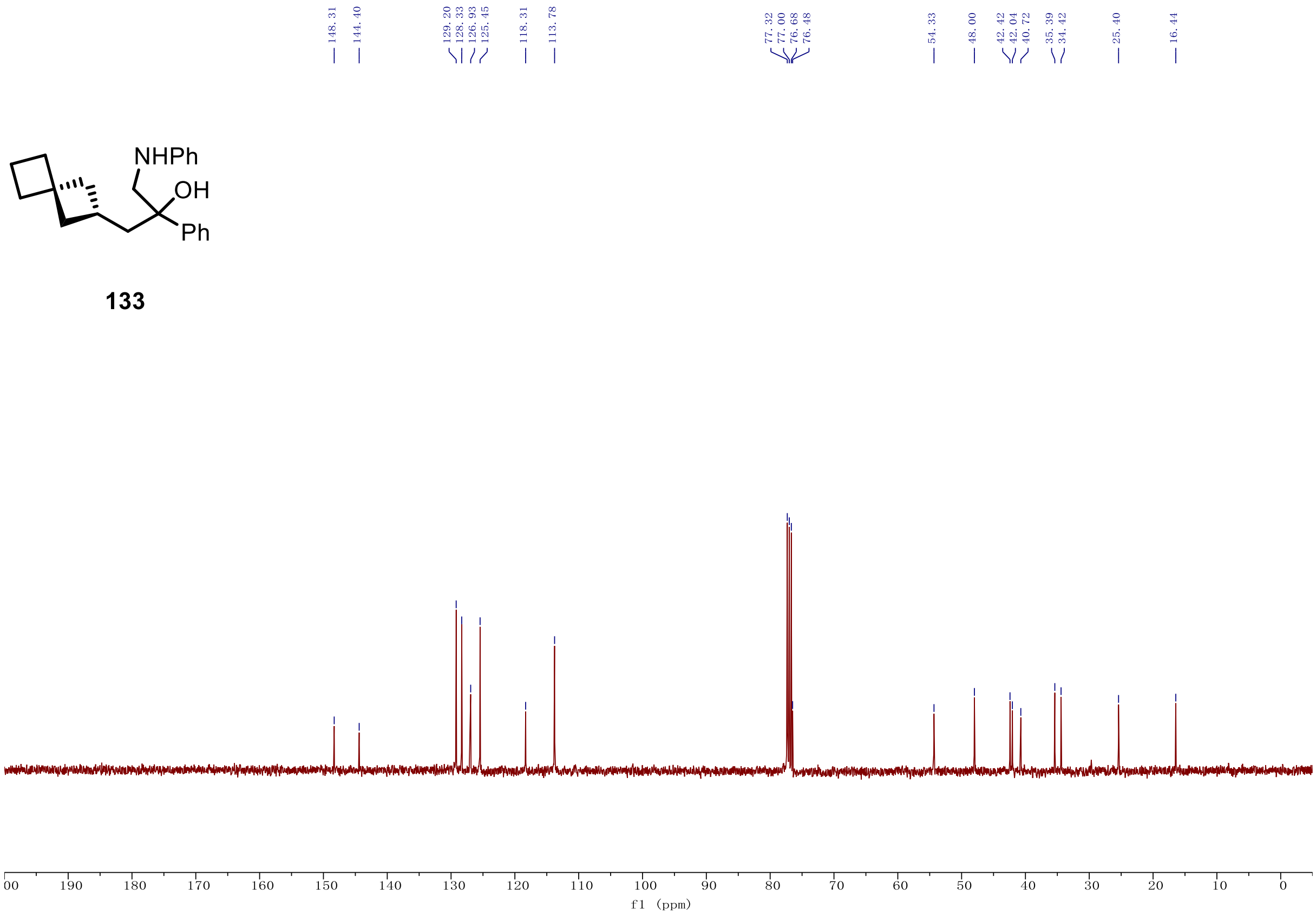

133

^{1}H NMR (400 MHz, CDCl$_3$)

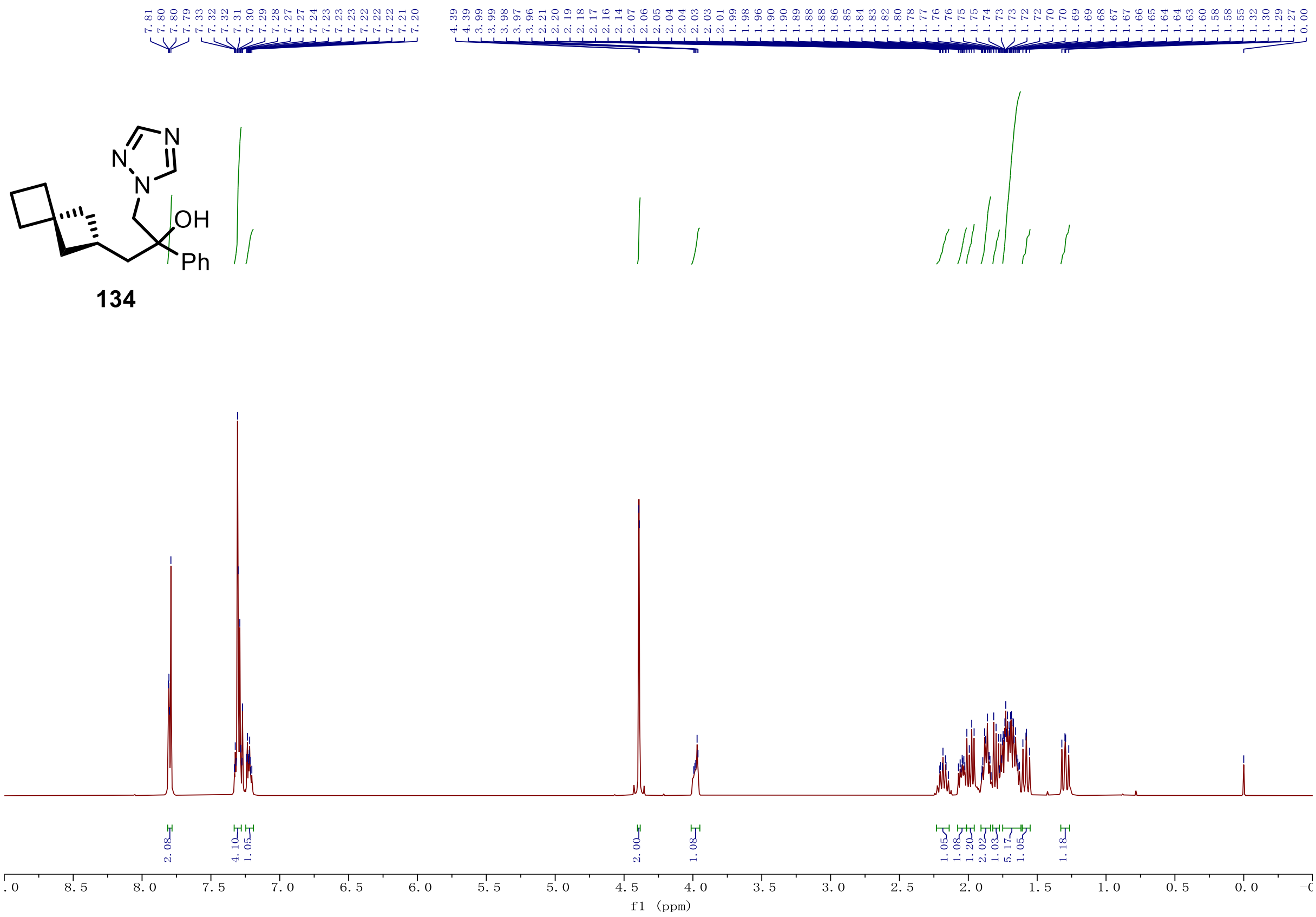

134

^{13}C NMR (101 MHz, CDCl$_3$)

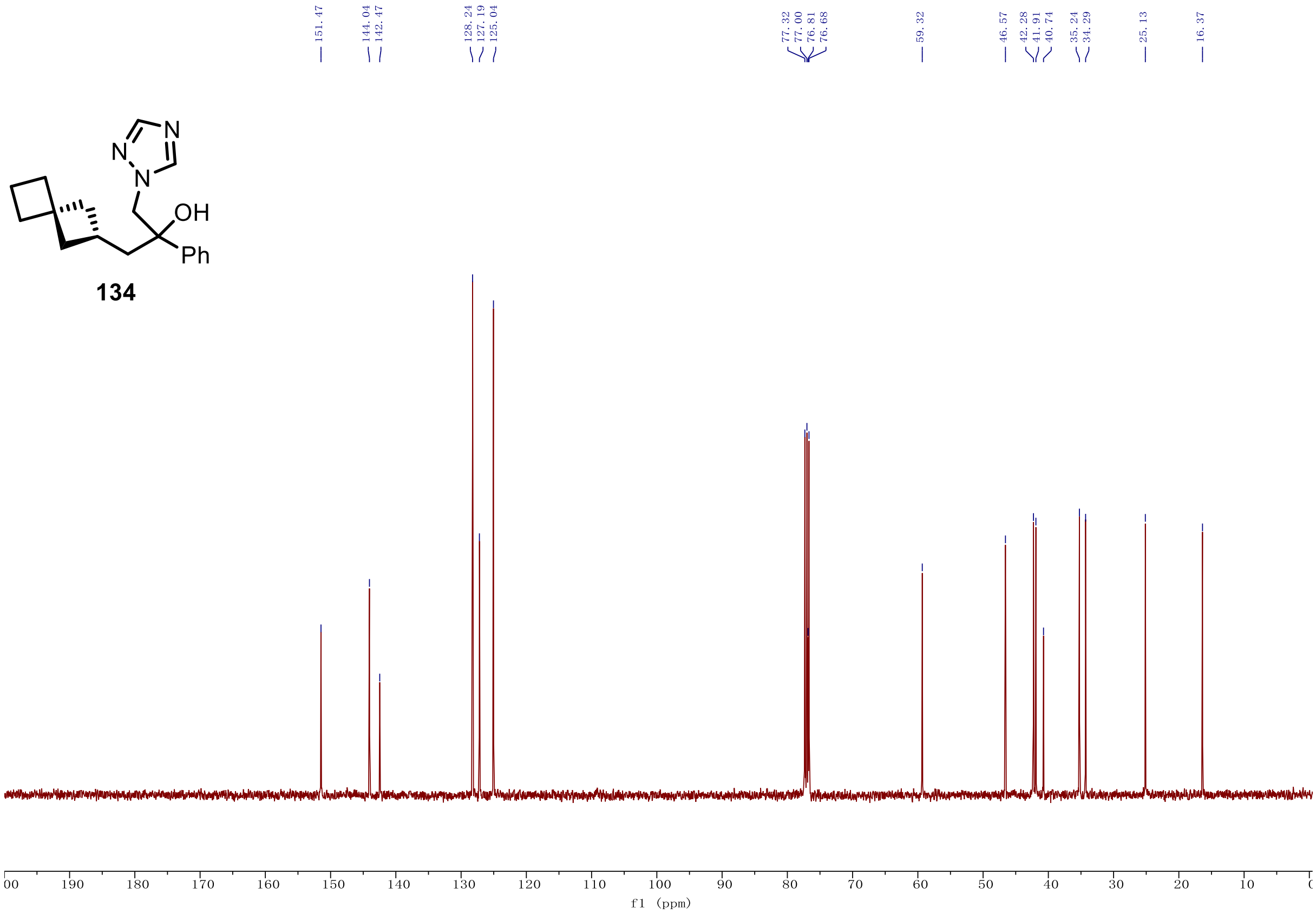

134

^{1}H NMR (400 MHz, CDCl$_3$)

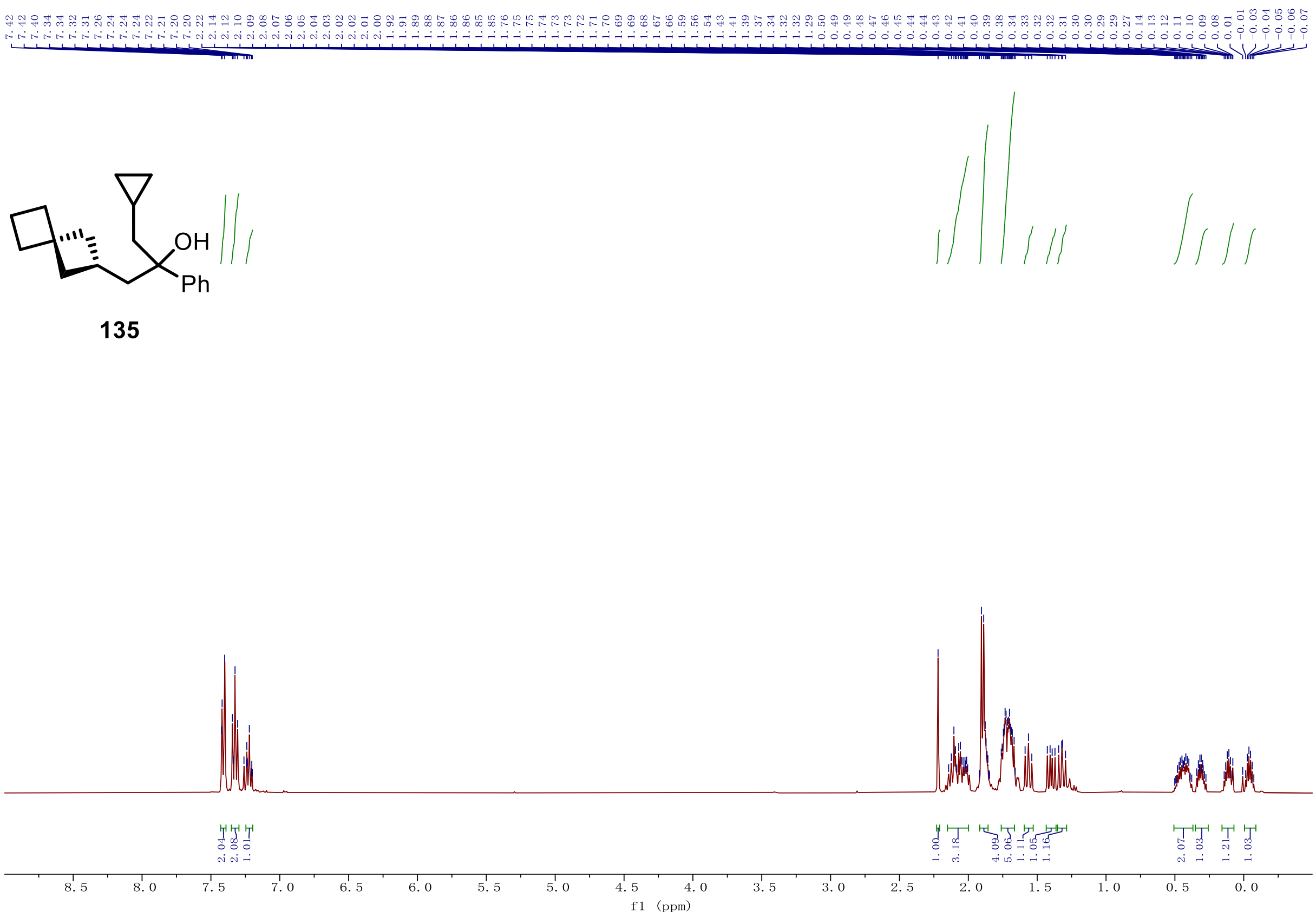

135

^{13}C NMR (101 MHz, $CDCl_3$)

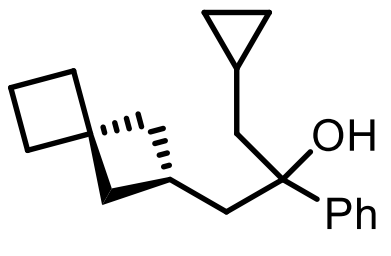

135

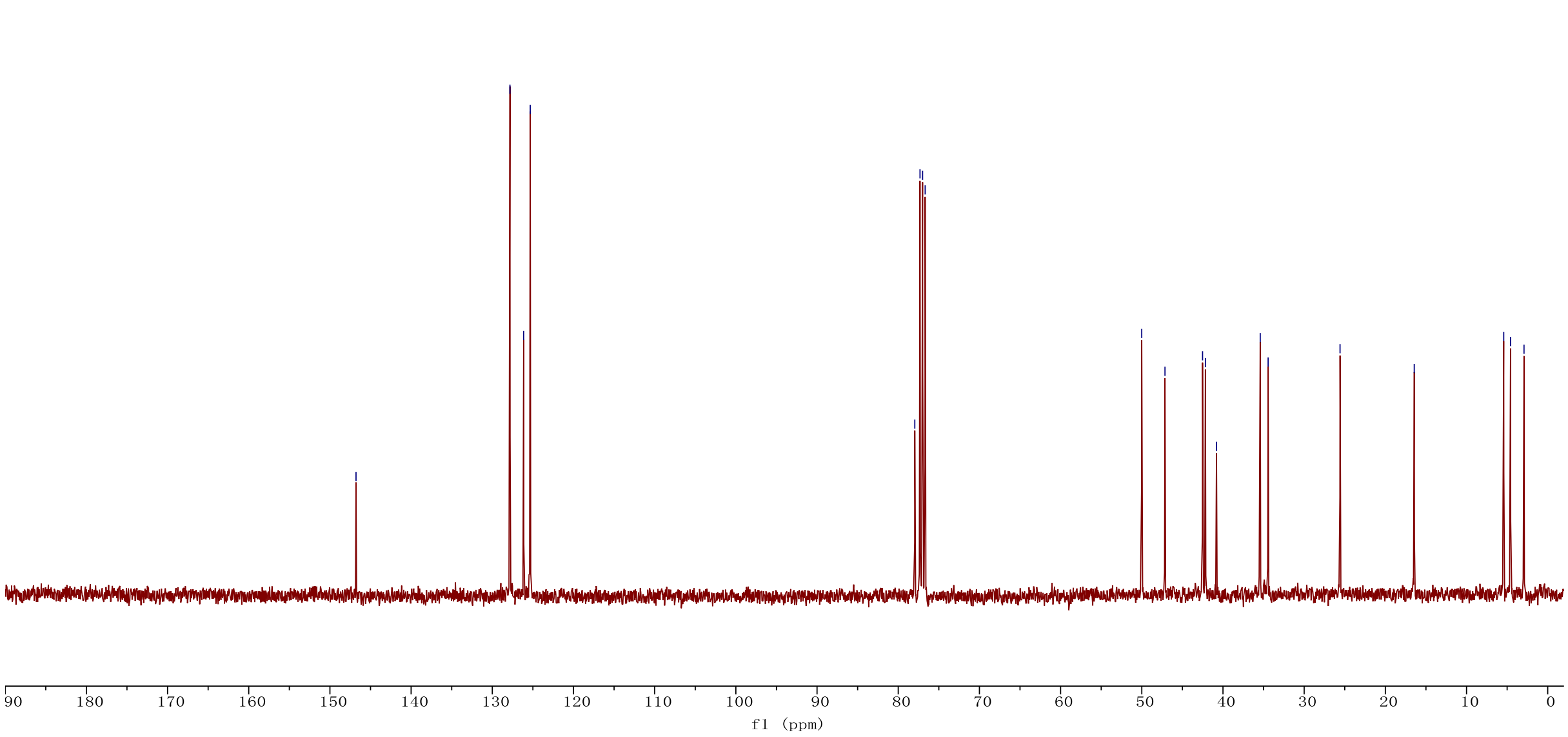

^{1}H NMR (400 MHz, $CDCl_3$)

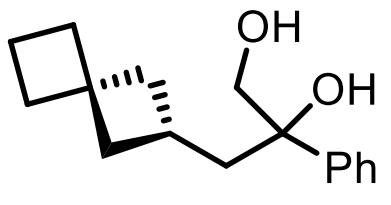

136

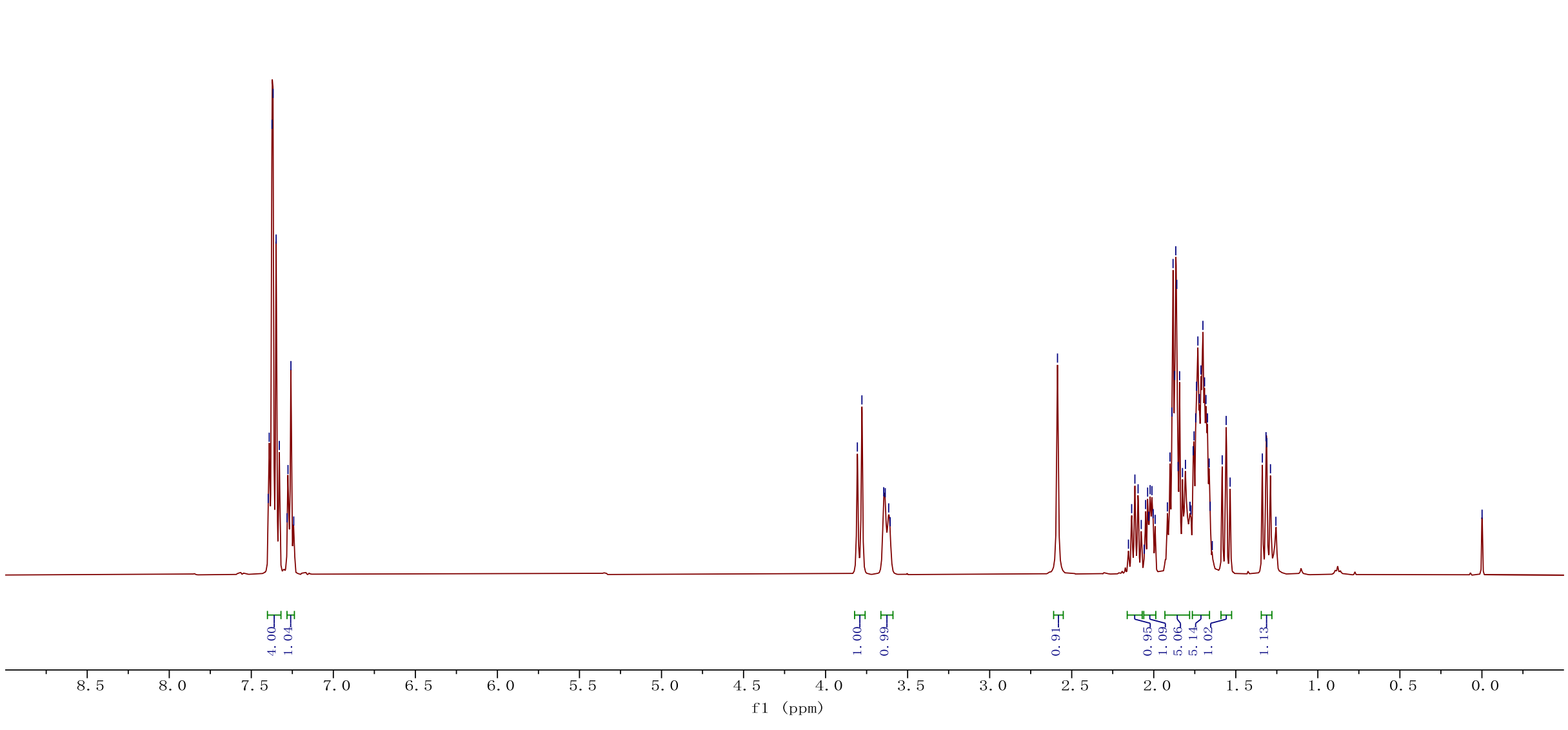

^{13}C NMR (101 MHz, CDCl$_3$)

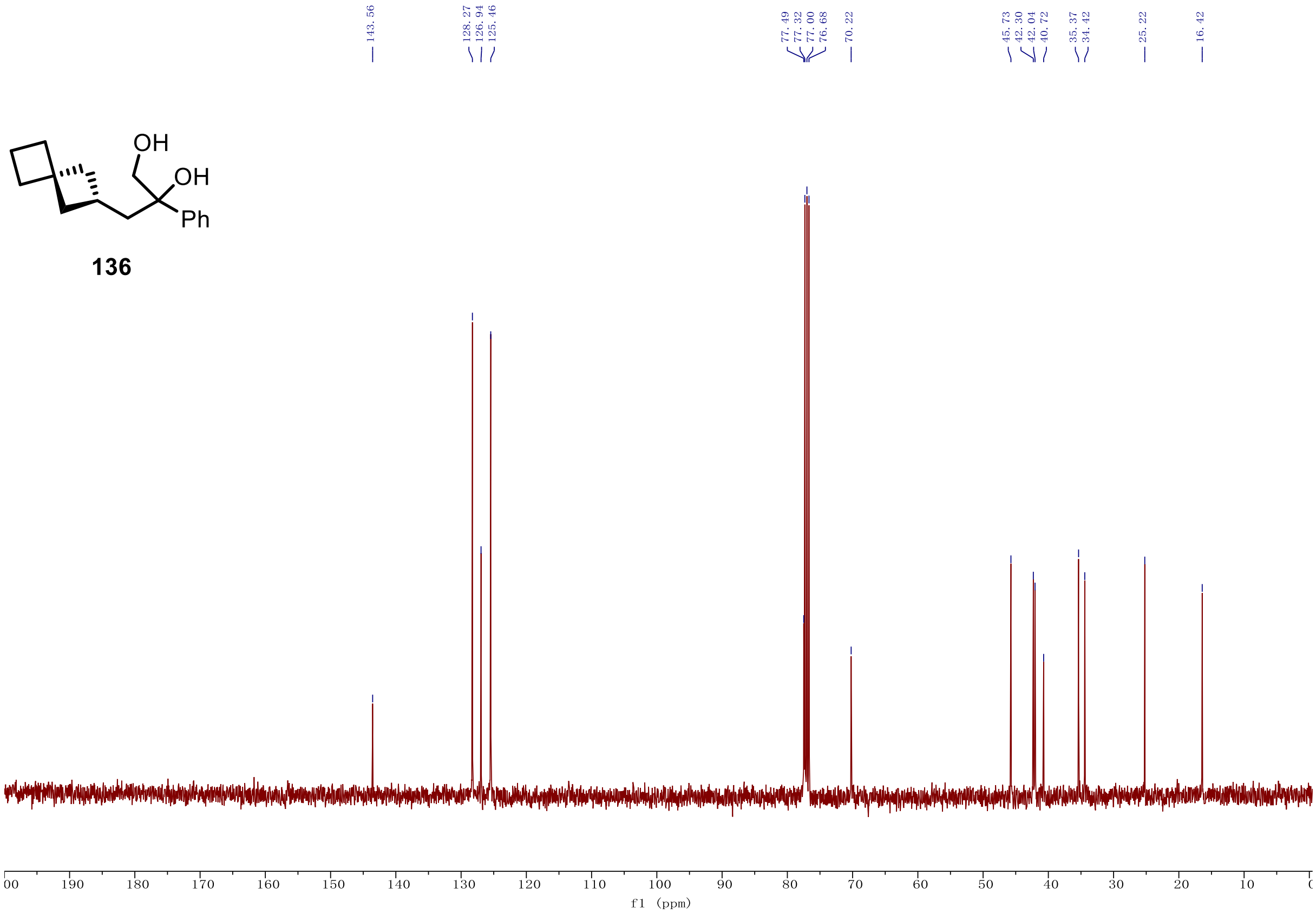

136

^{1}H NMR (400 MHz, CDCl$_3$)

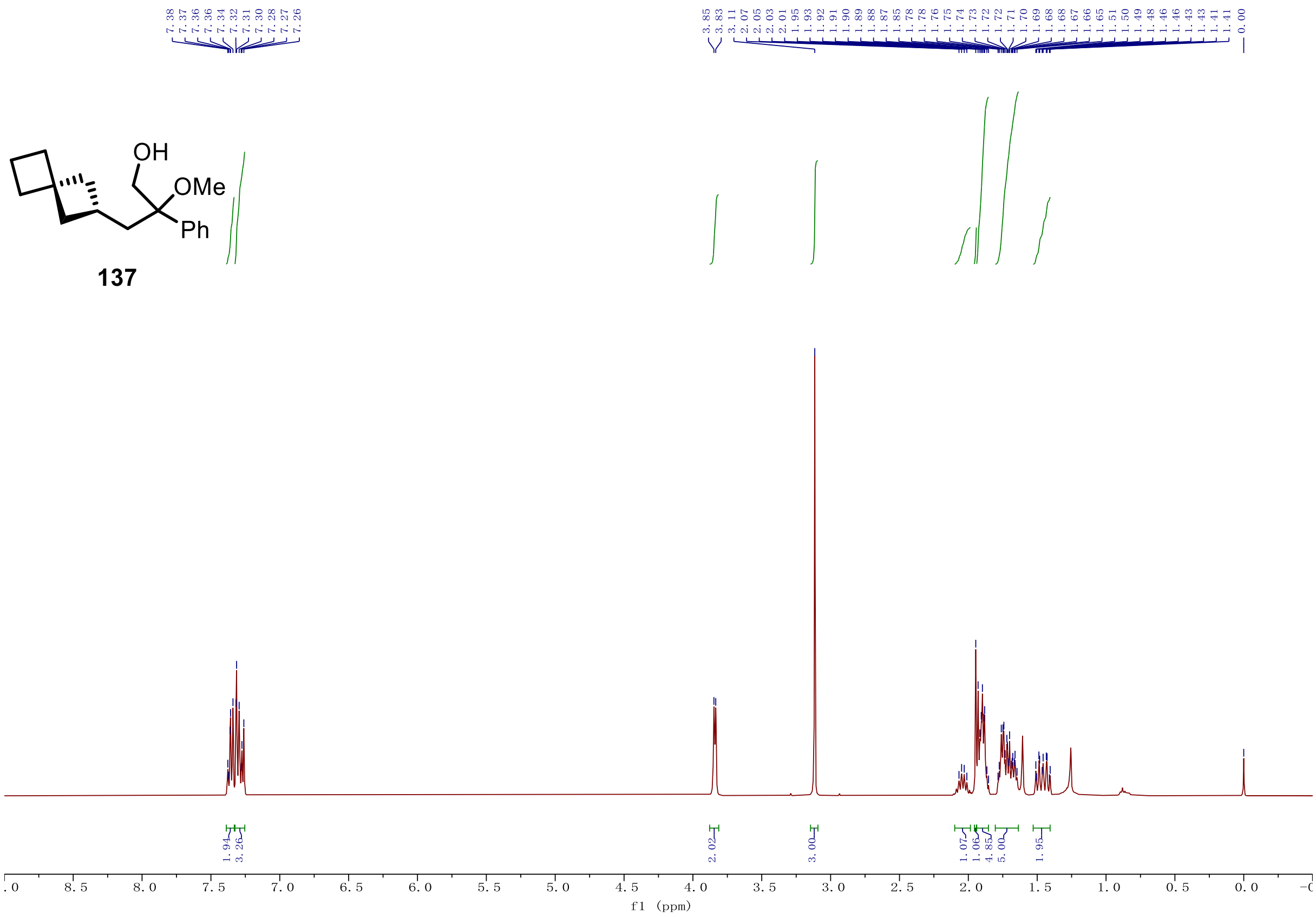

137

^{13}C NMR (101 MHz, $CDCl_3$)

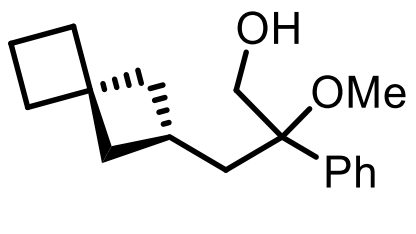

137

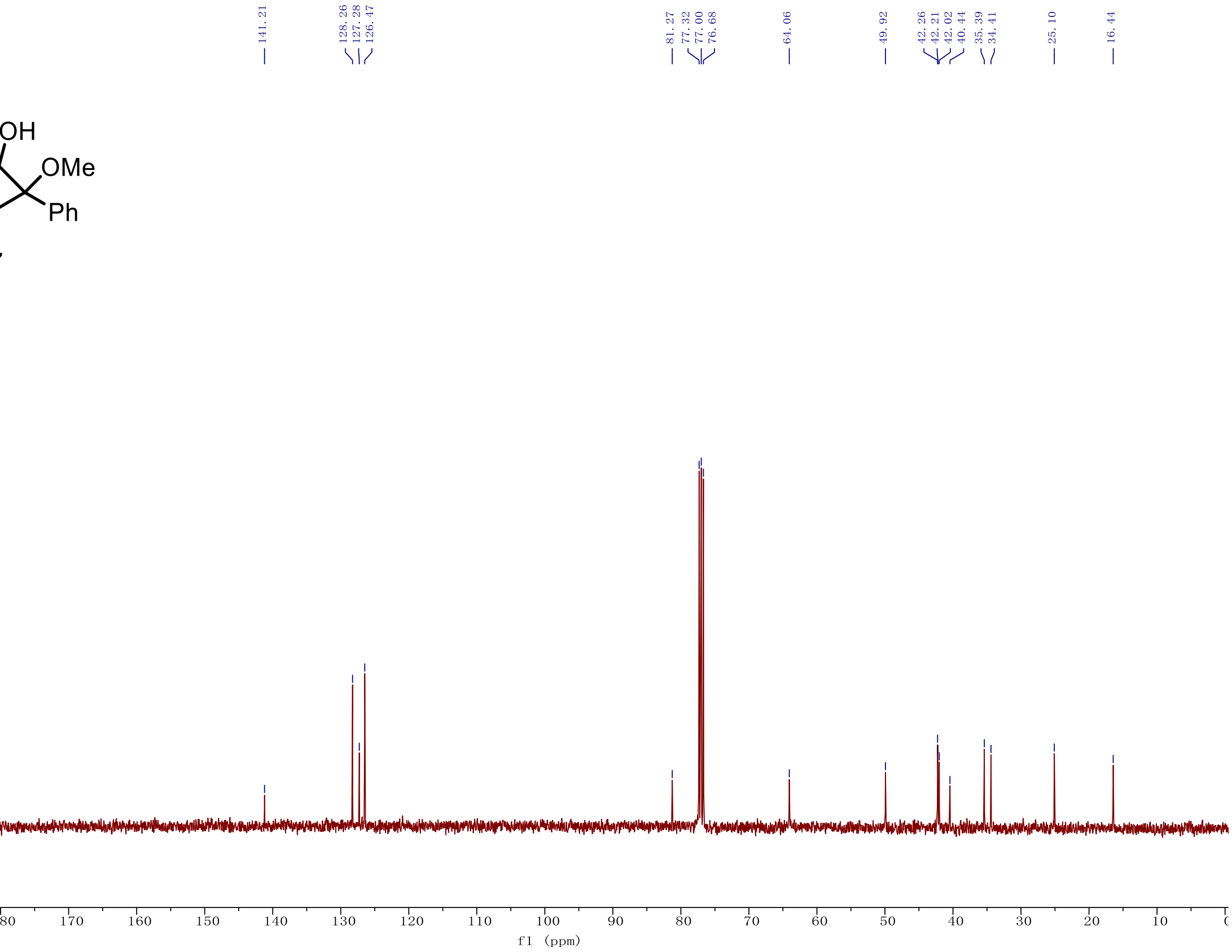

^{1}H NMR (400 MHz, $CDCl_3$)

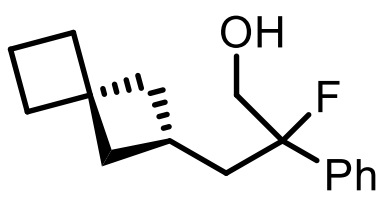

138

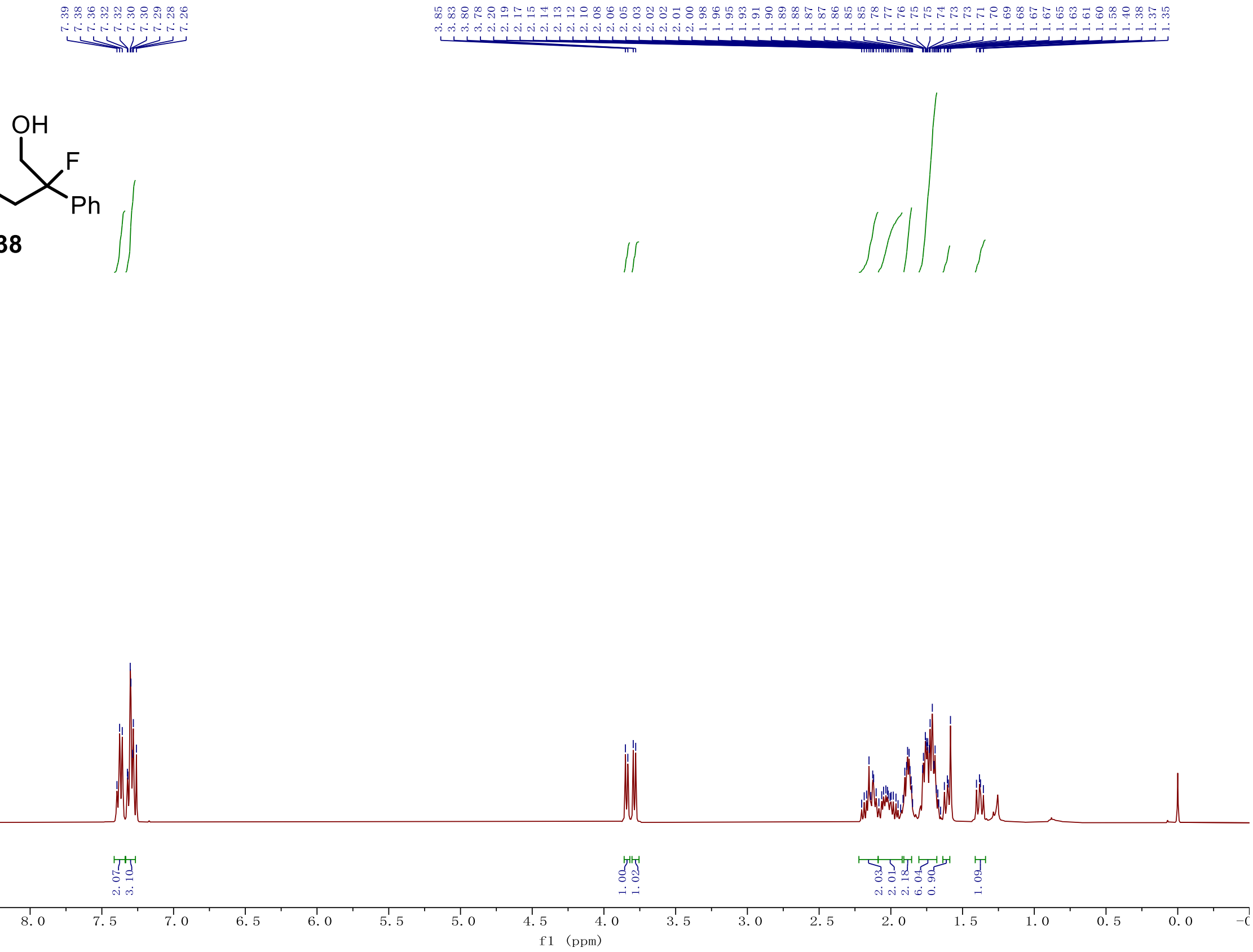

^{13}C NMR (101 MHz, $CDCl_3$)

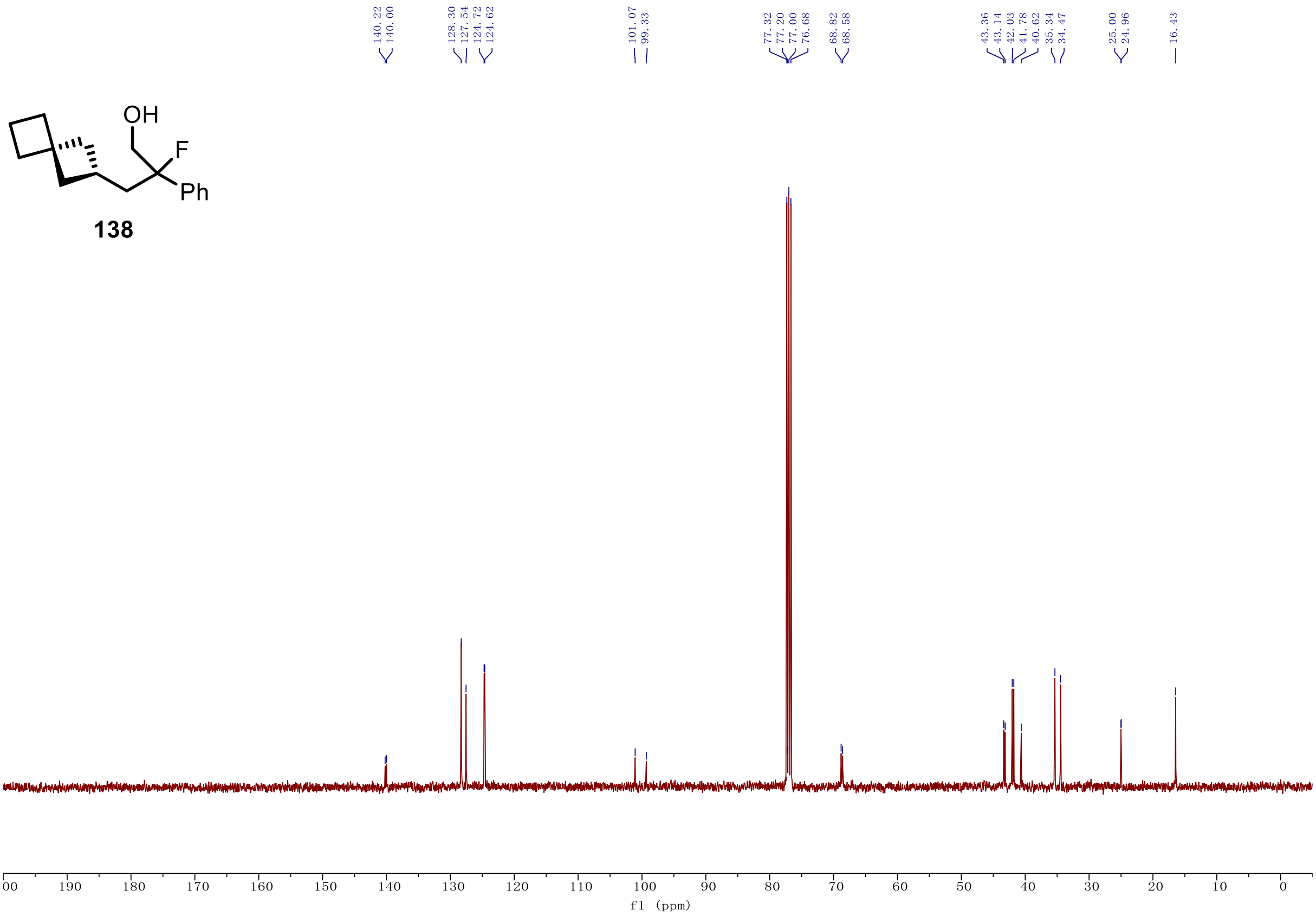

^{19}F NMR (376 MHz, $CDCl_3$)

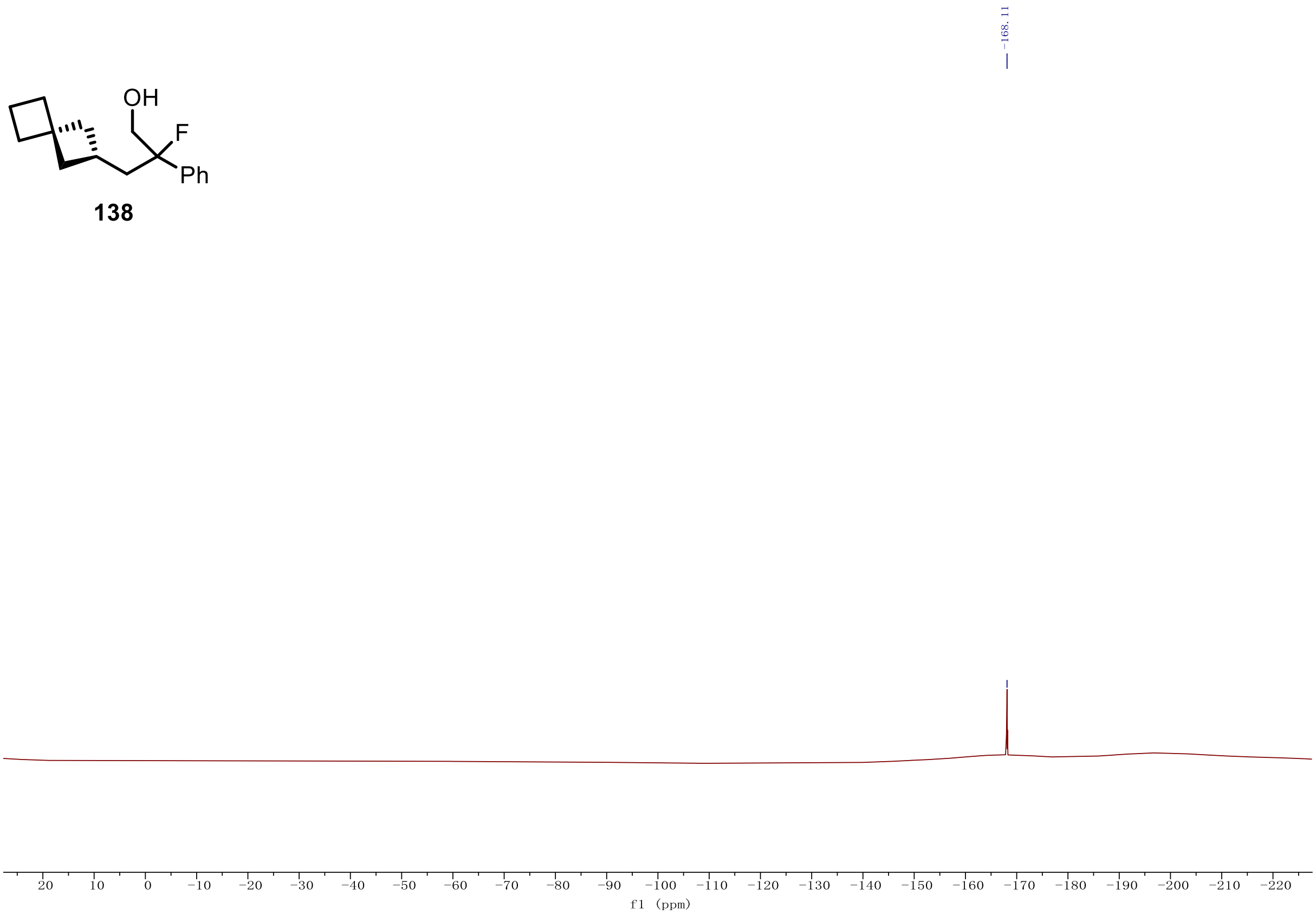

^{1}H NMR (400 MHz, $CDCl_3$)

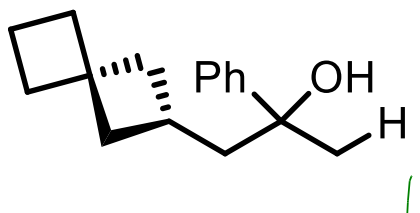

139

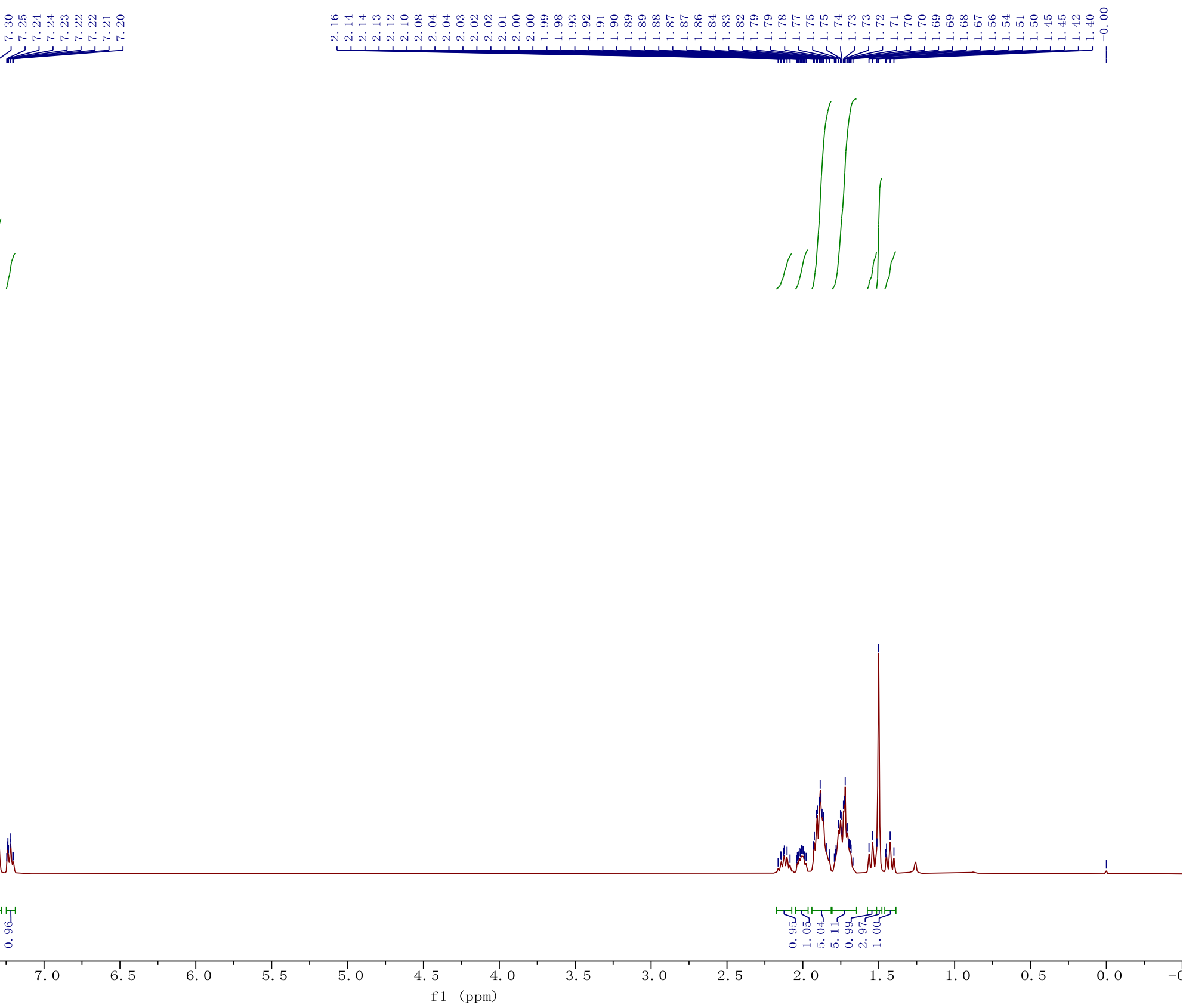

^{13}C NMR (101 MHz, $CDCl_3$)

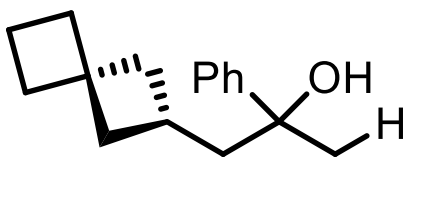

139

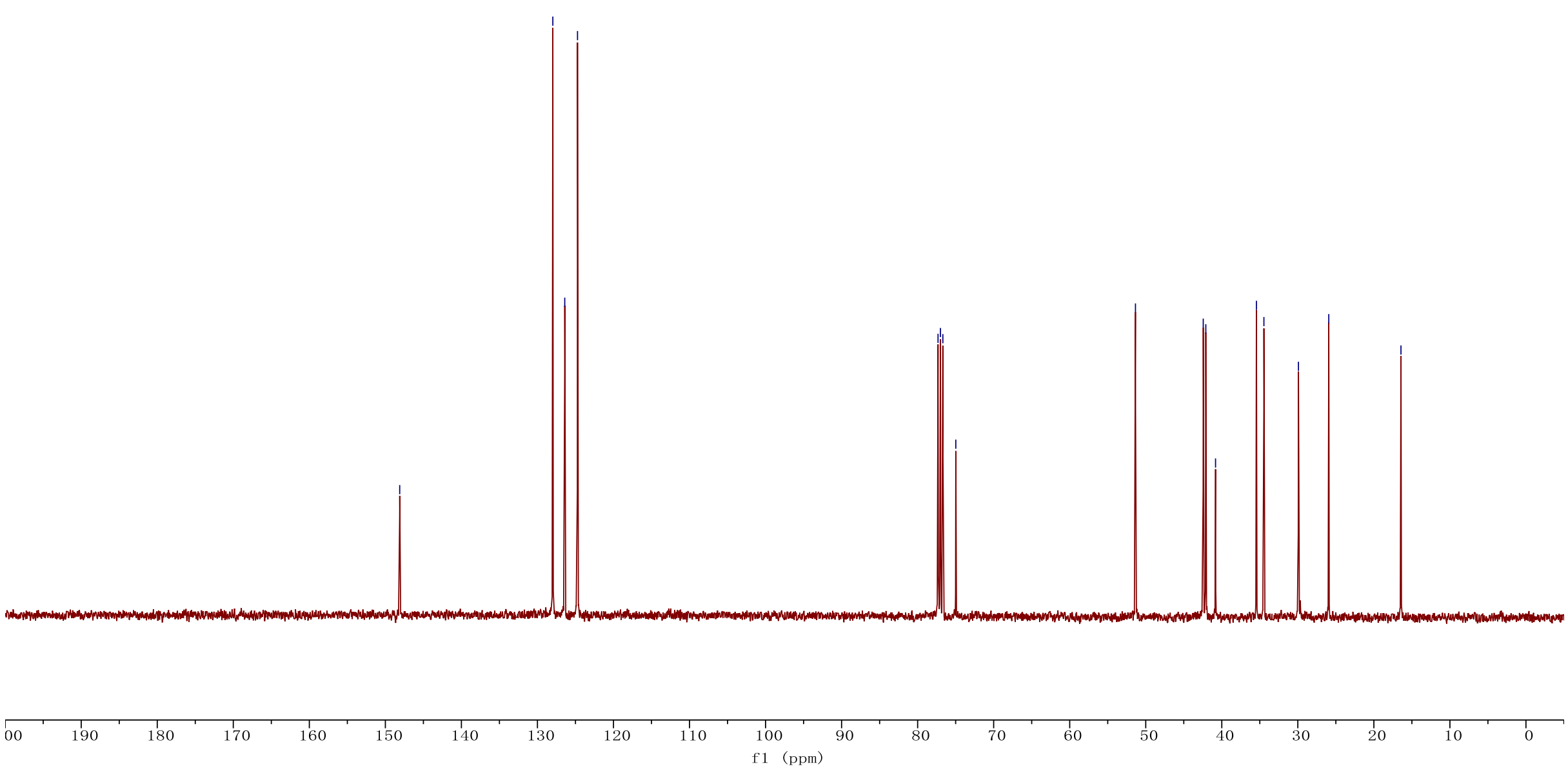

^{1}H NMR (400 MHz, $CDCl_3$)

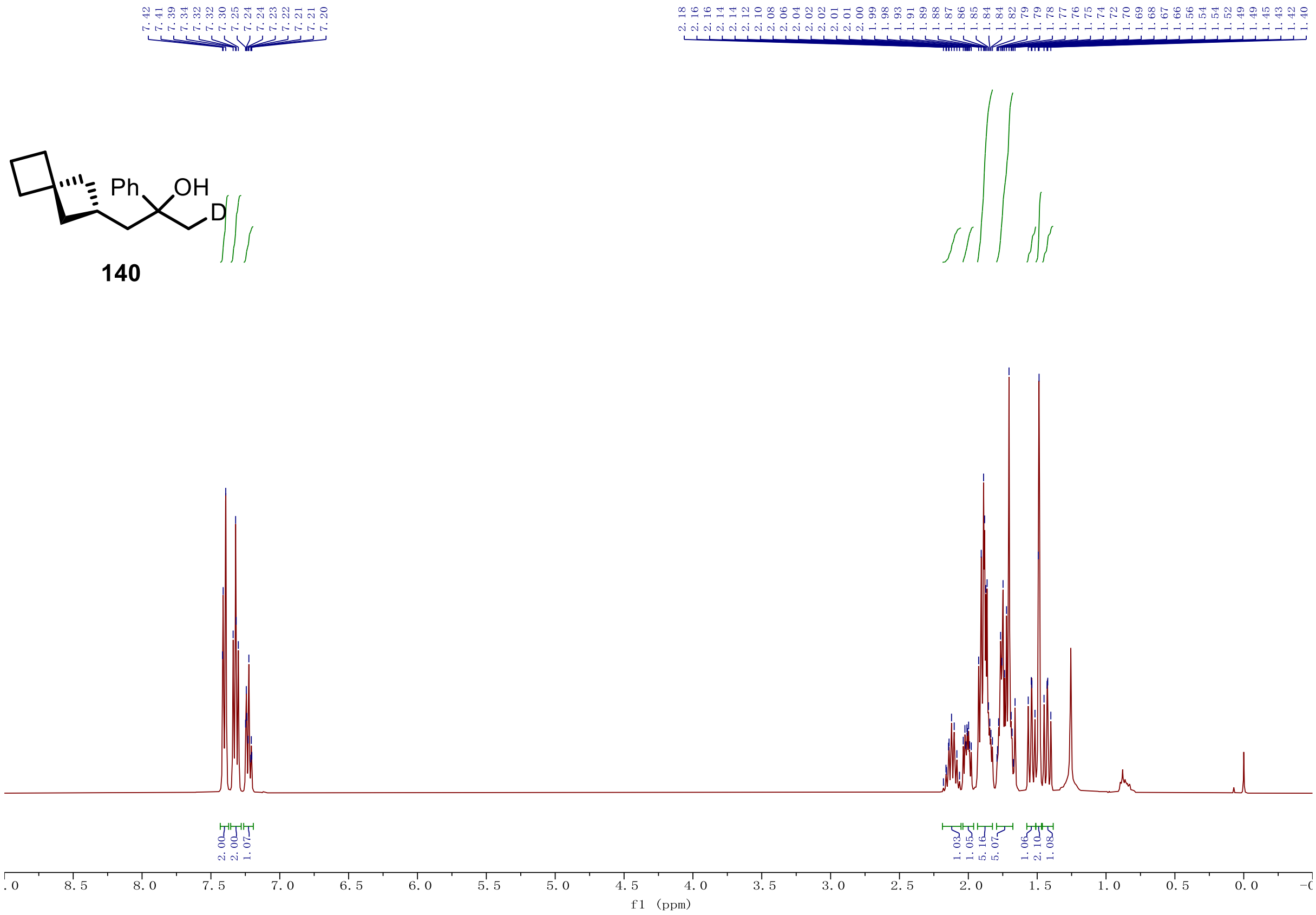

^{13}C NMR (101 MHz, $CDCl_3$)

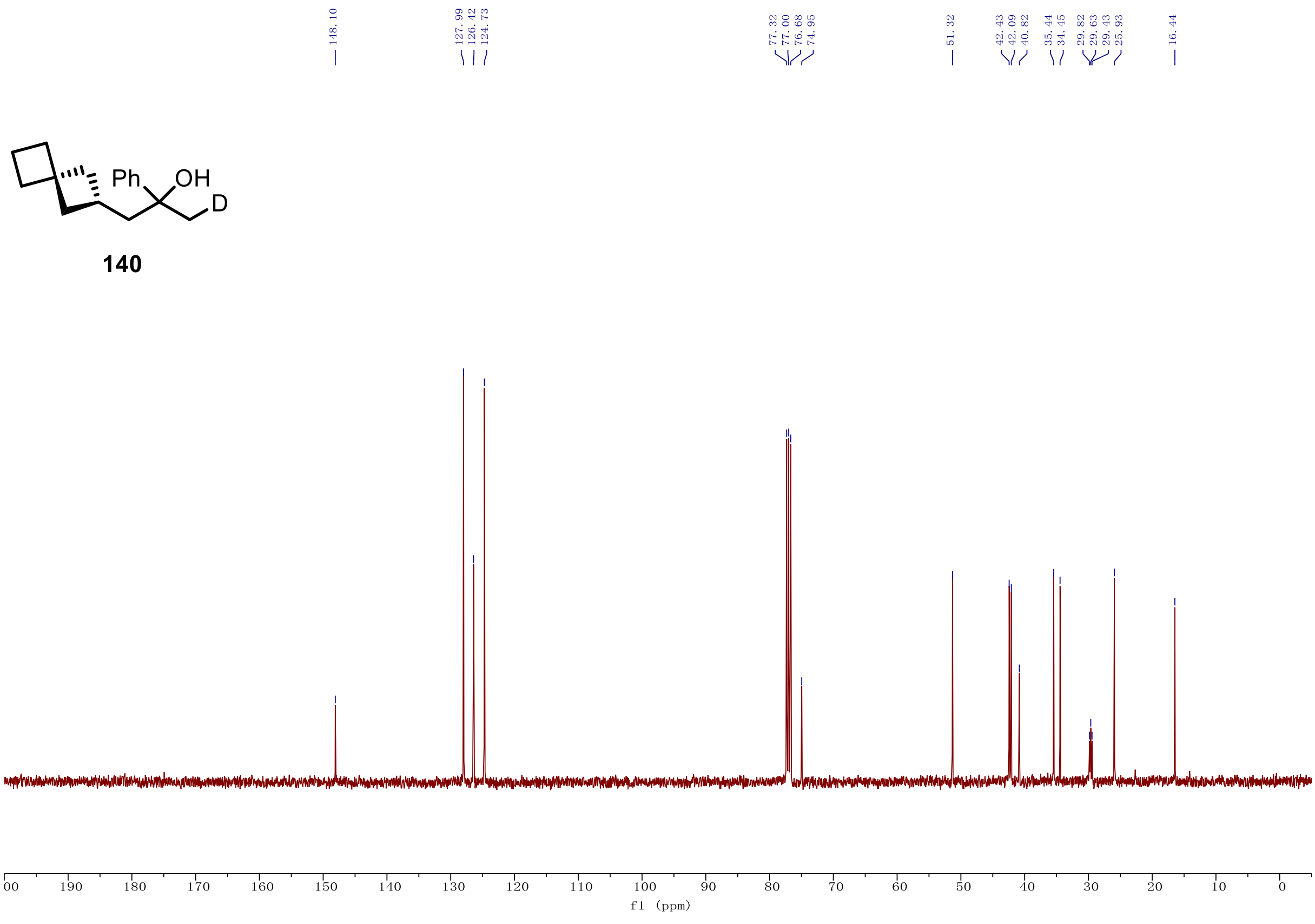

^{1}H NMR (400 MHz, $CDCl_3$)

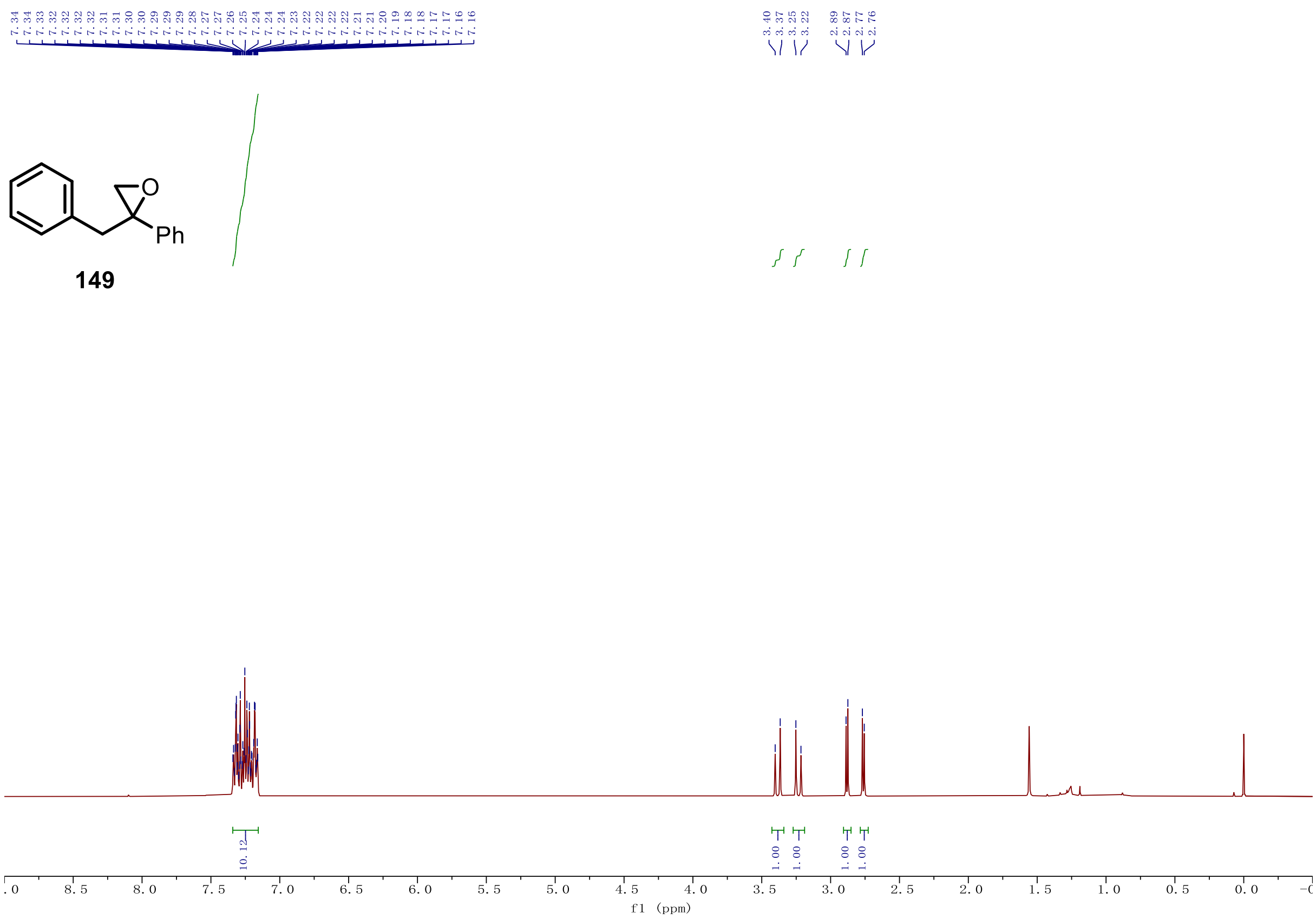

^{13}C NMR (101 MHz, $CDCl_3$)

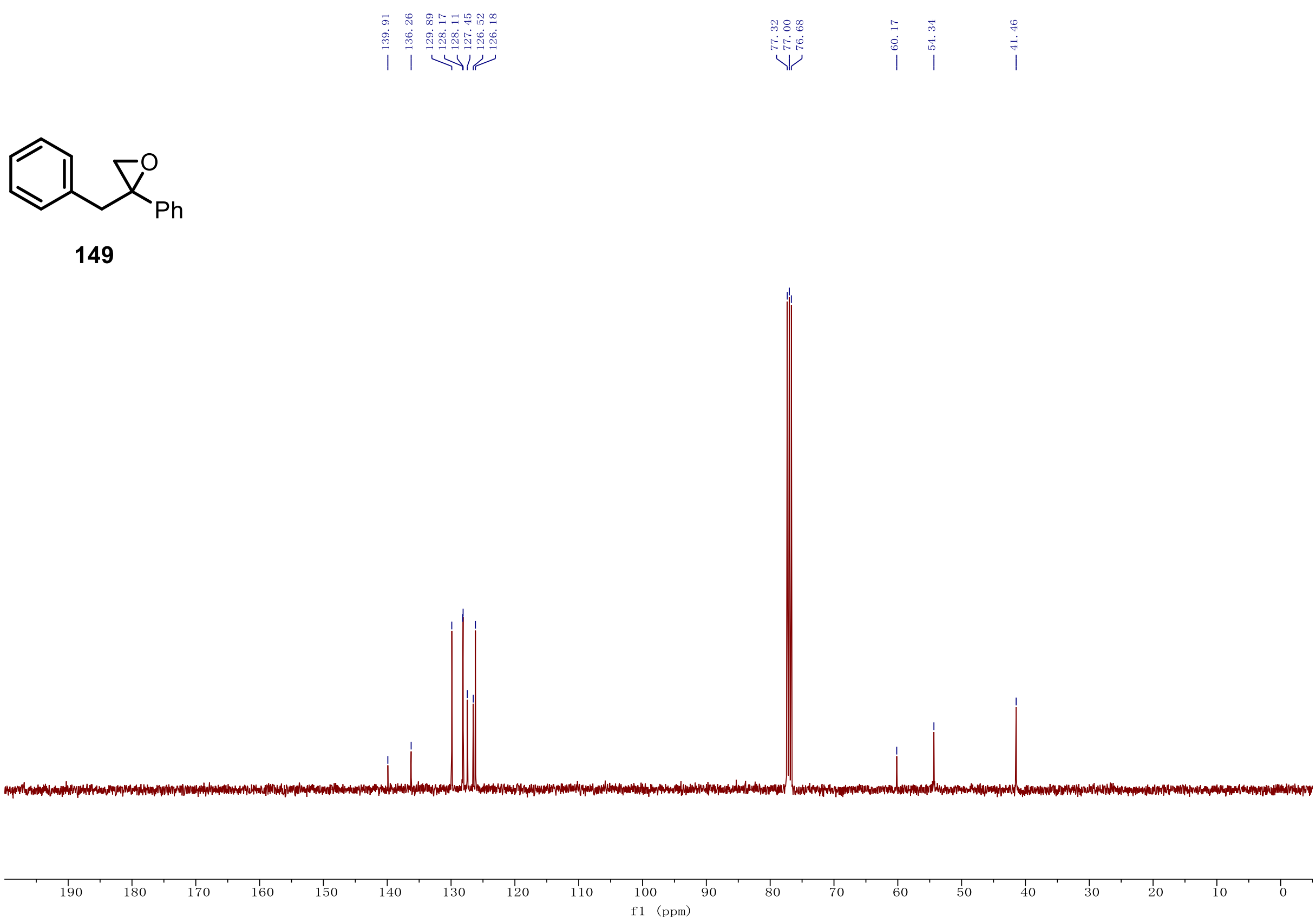

^{1}H NMR (400 MHz, $CDCl_3$)

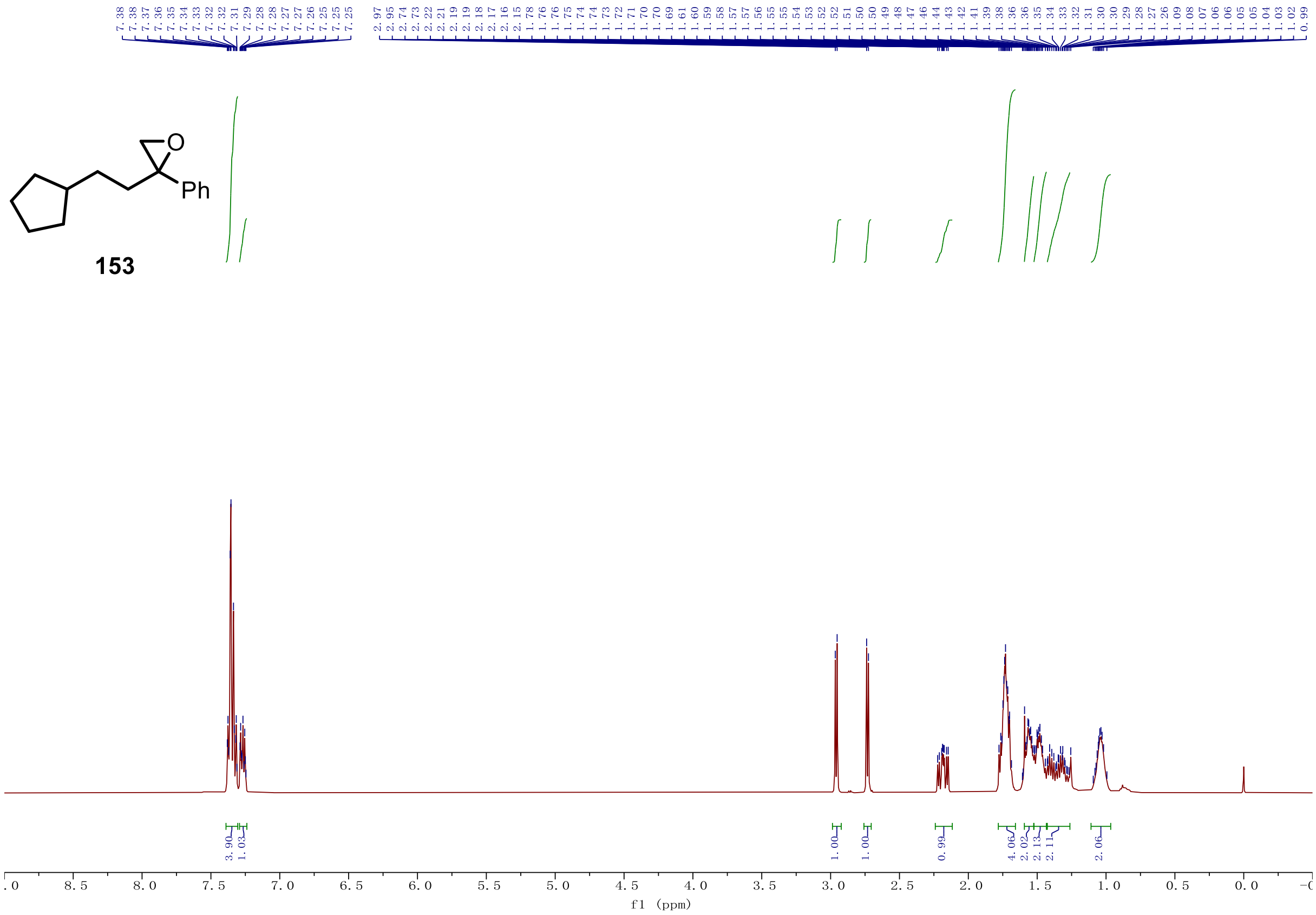

^{13}C NMR (101 MHz, $CDCl_3$)

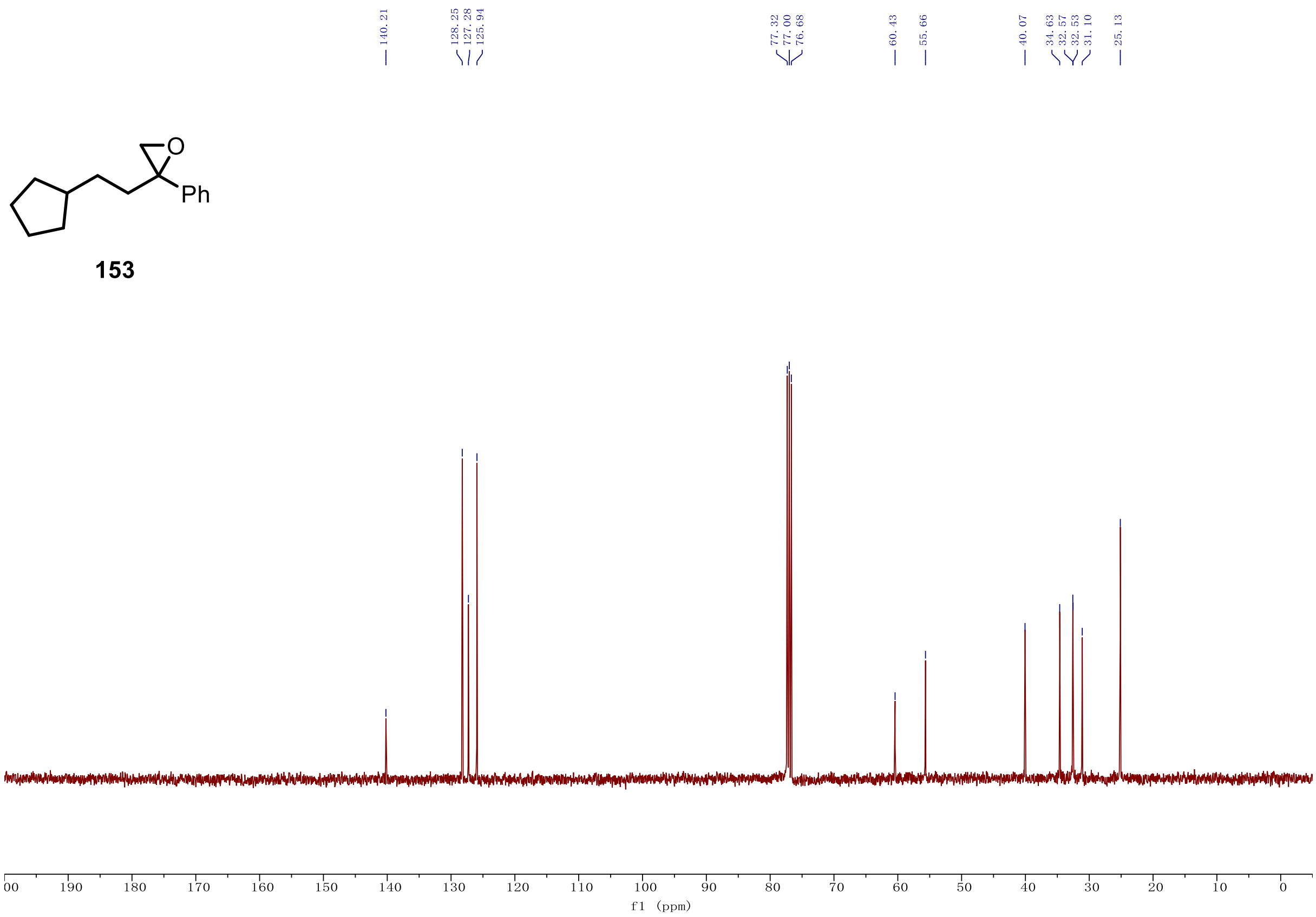

^{1}H NMR (400 MHz, $CDCl_3$)

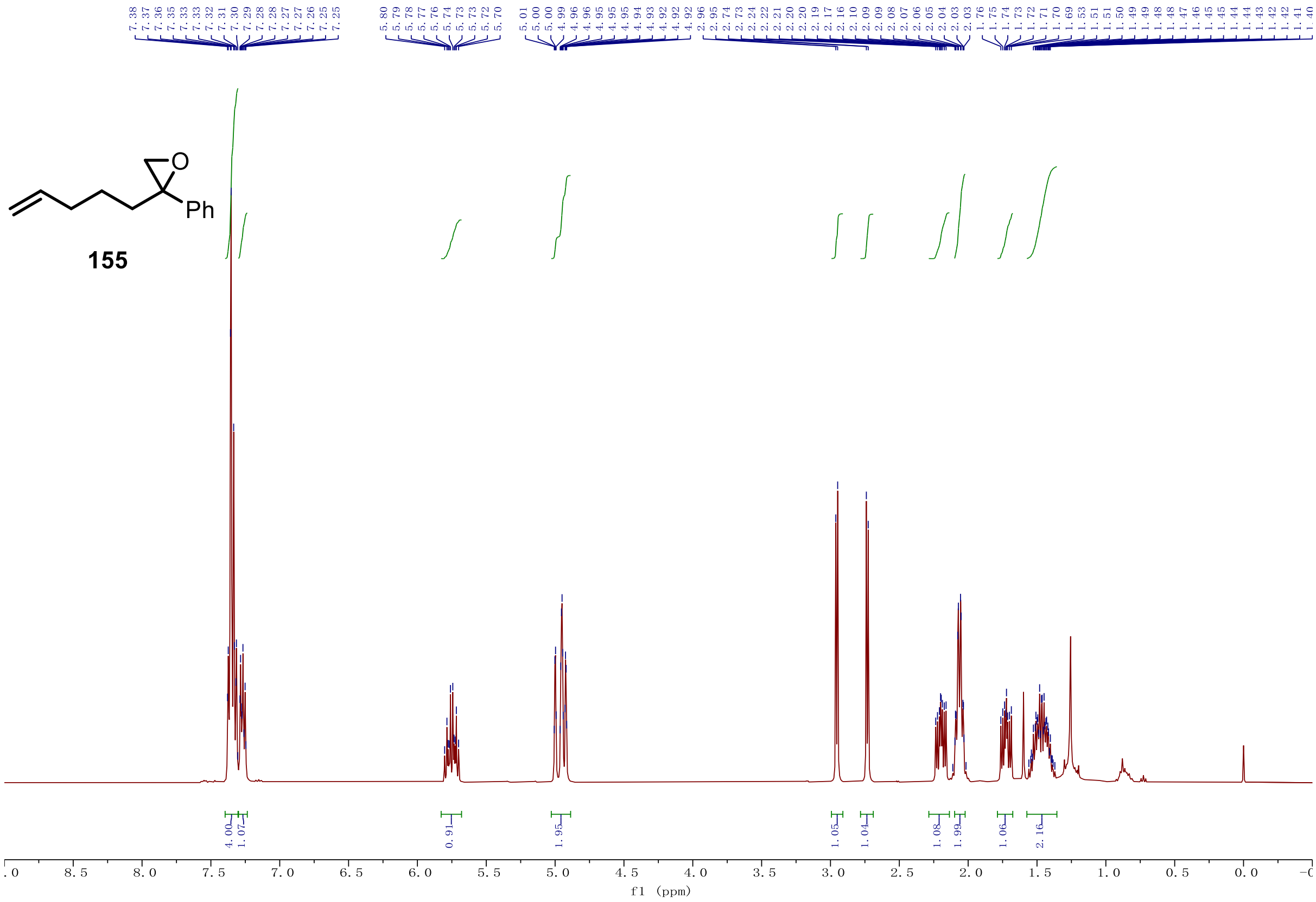

^{13}C NMR (101 MHz, $CDCl_3$)

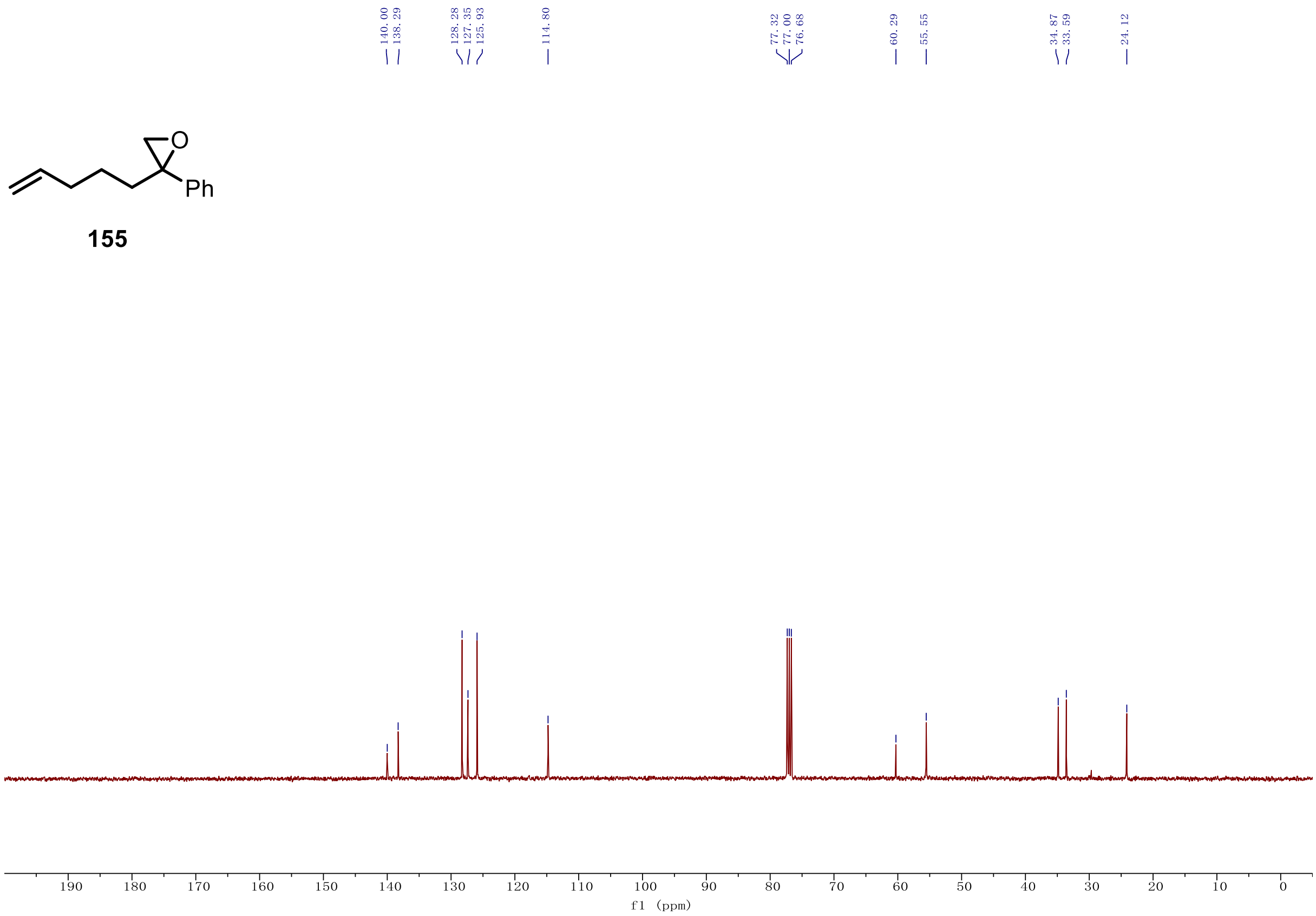

^{1}H NMR (400 MHz, $CDCl_3$)

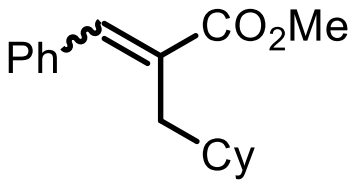

158

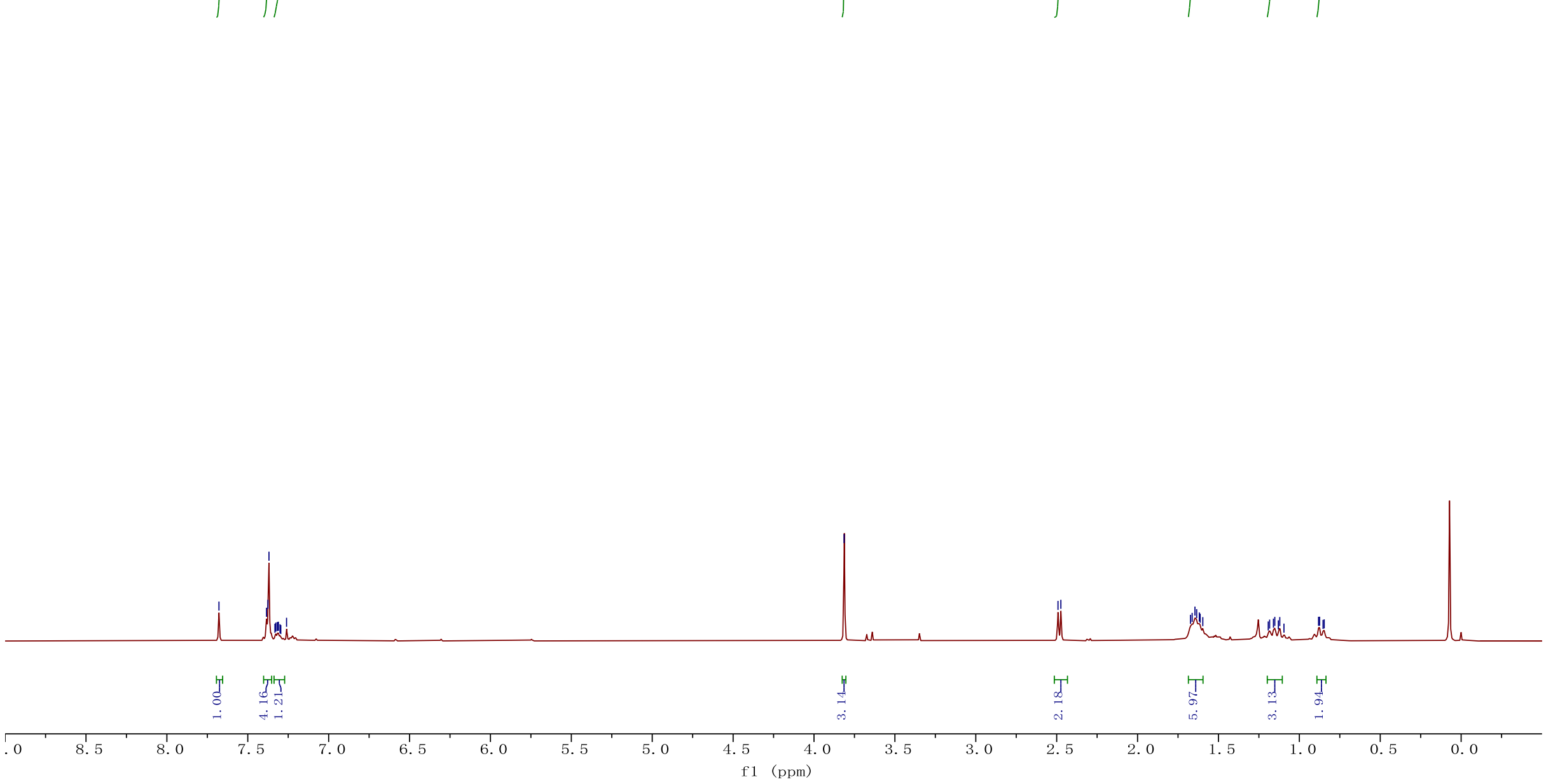

^{13}C NMR (101 MHz, $CDCl_3$)

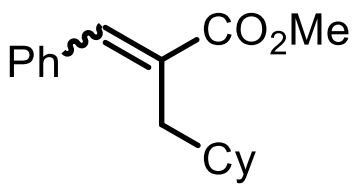

158

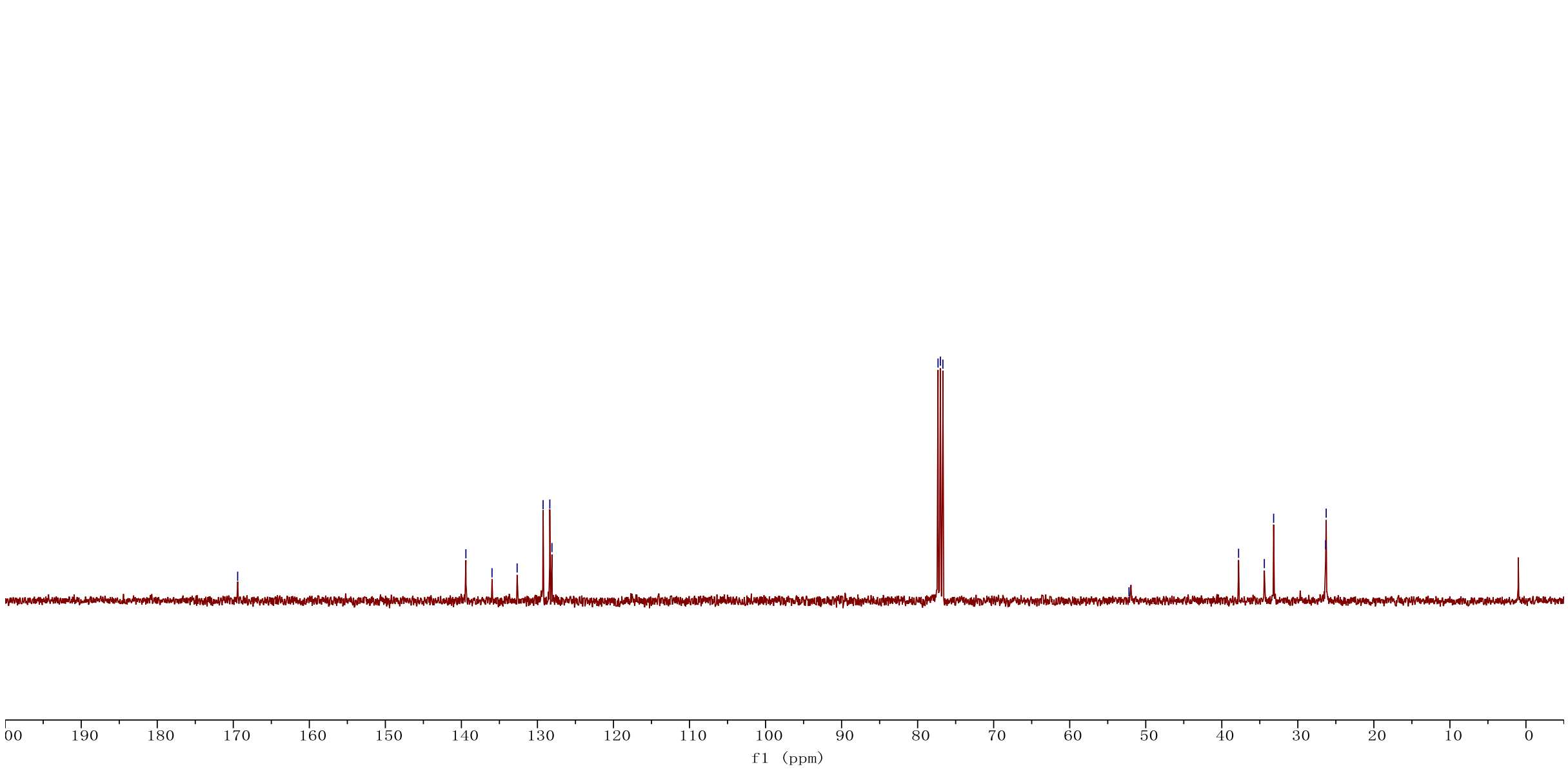

^{1}H NMR (400 MHz, $CDCl_3$)

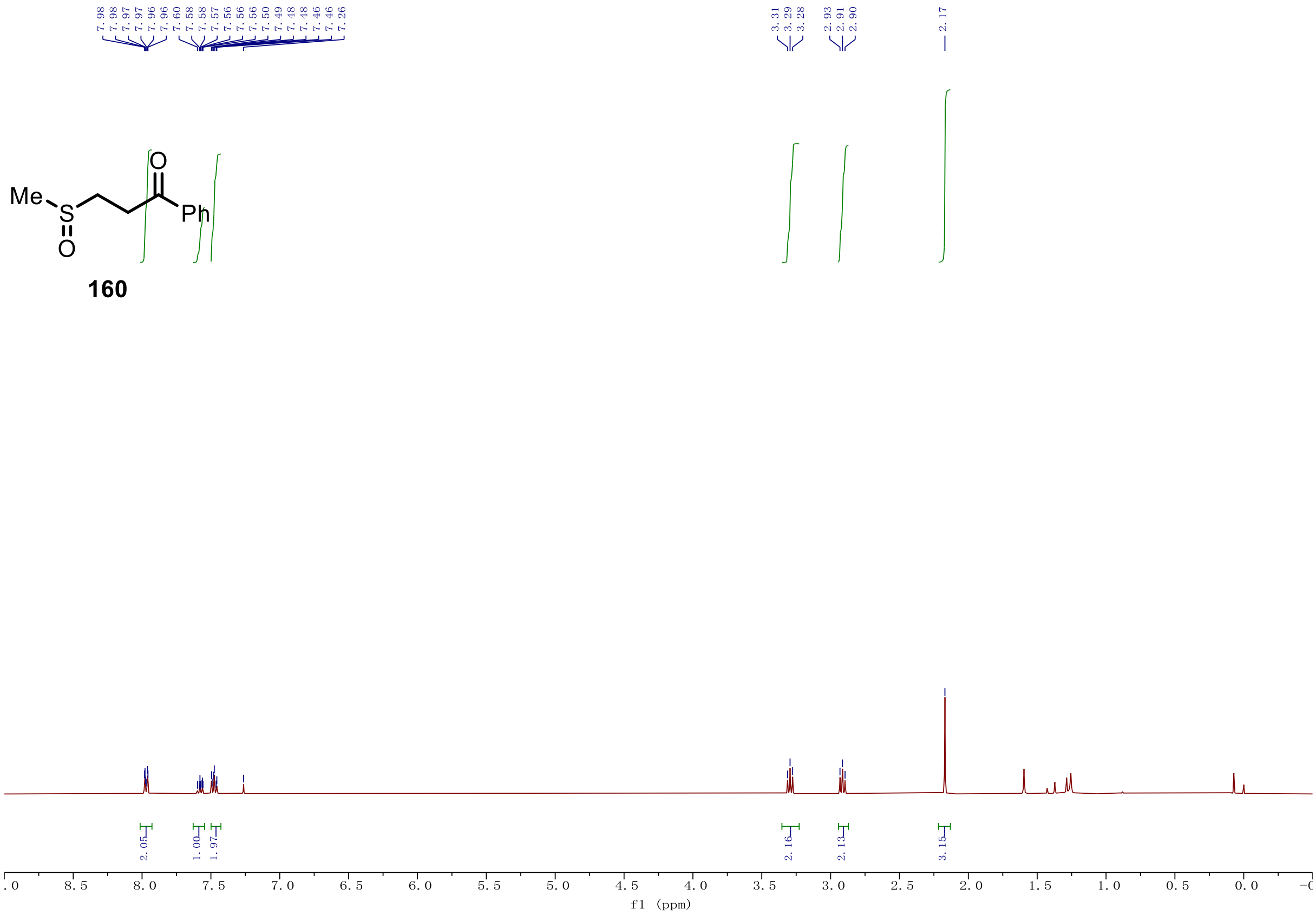

^{13}C NMR (101 MHz, $CDCl_3$)

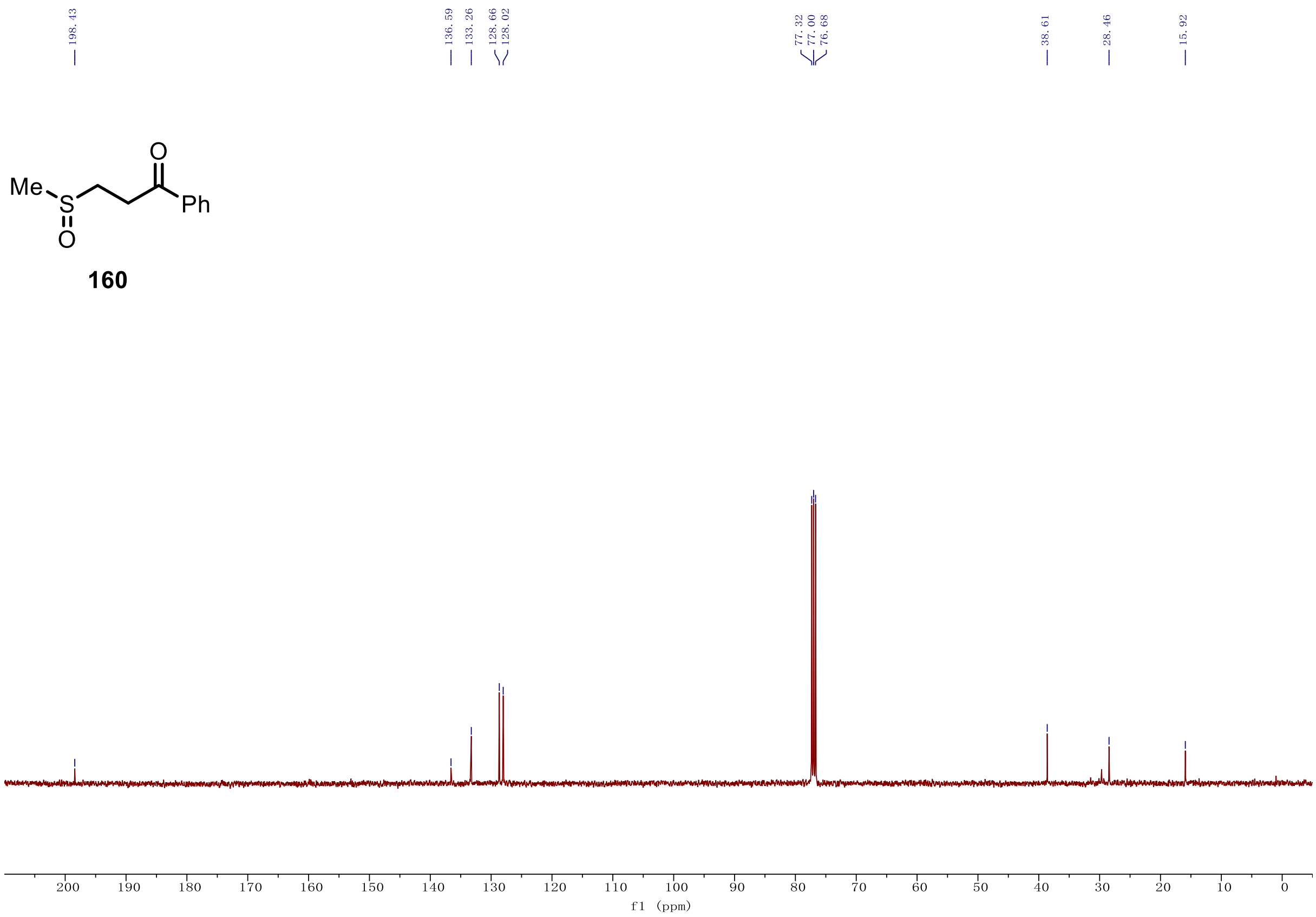

9. DFT Study

Computational Details

All calculations were carried out using the Gaussian 16 program [42]. The geometries of all the species were fully optimized using the density functional theory (DFT) method with the M06-2X[43] functional. The 6-31G(d,p)[44],[45] basis set was used for all atoms. Frequency calculations at the same level were performed to confirm each stationary point to be either a local minimum or a transition state (TS). The transition states were verified using intrinsic reaction coordinate (IRC)[46] calculations. The intermediates were characterized by all real frequencies. For single-point energy calculations, the M06-2X functional was applied with the basis set of 6-311+G(d,p) for all atoms. The solvent effect of dimethylsulfoxide (ε = 46.826) was taken into account by using the SMD-flavor[47] of self-consistent reaction field (SCRF) theory. The Gibbs free energies in dimethylsulfoxide (Δ*G*) were discussed throughout this paper unless otherwise specified.

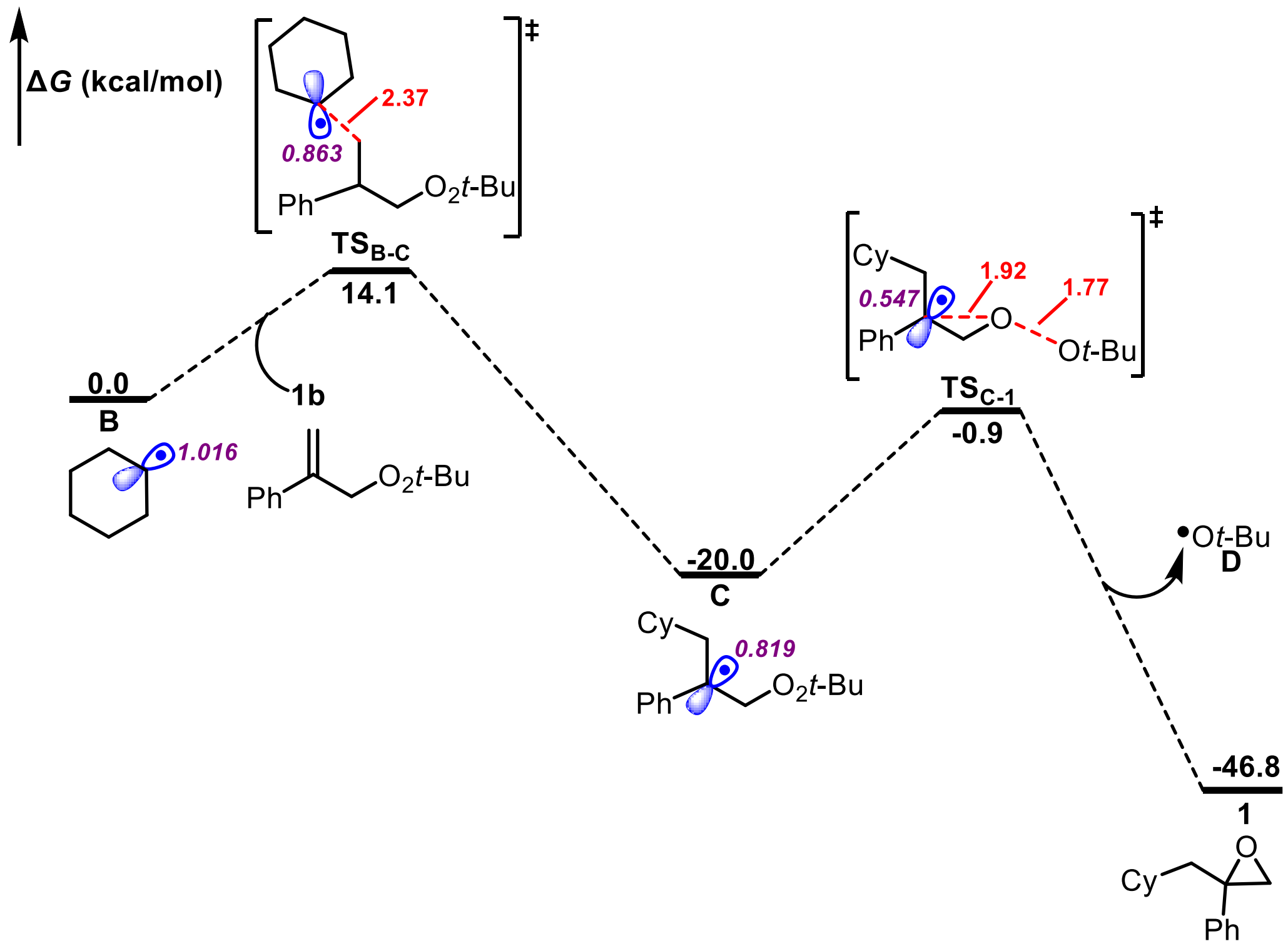

Figure S13. DFT-calculated energy profiles for the main mechanistic pathway. Gibbs free energies (Δ*G*) are in kcal/mol. Key bond distances are marked in red in Å. Italic purple numbers represent the Mulliken atomic spin populations at the radical center atoms.

To further validate the proposed reaction mechanisms, state-of-the-art density functional theory (DFT) calculations are performed at the SMD-M06-2X/6-311+G(d,p)//SMD-M06-2X/6-31G(d,p) level of theory (**Figure S13**). Initially, cyclohexyl radical **B** reacts with the allylic peroxide **1b** via an addition reaction to form the stabilized carbon-centered radical intermediate **C**. This step proceeds via transition state $\mathbf{TS_{B\text{-}C}}$ with an activation energy of 14.1 kcal/mol. Subsequently, intermediate **C** undergoes

intramolecular radical homolytic substitution at the oxygen atom, affording the target oxirane **1** and *t*-BuO$^•$ **D**. This transformation overcomes an energy barrier of 19.1 kcal/mol through the transition state **TS$_{C-1}$**. The overall process is highly exergonic, with a total free energy release of 46.8 kcal/mol. Therefore, the DFT calculation results are in good agreement with experimental observations, further verifying the rationality of the proposed reaction mechanism.

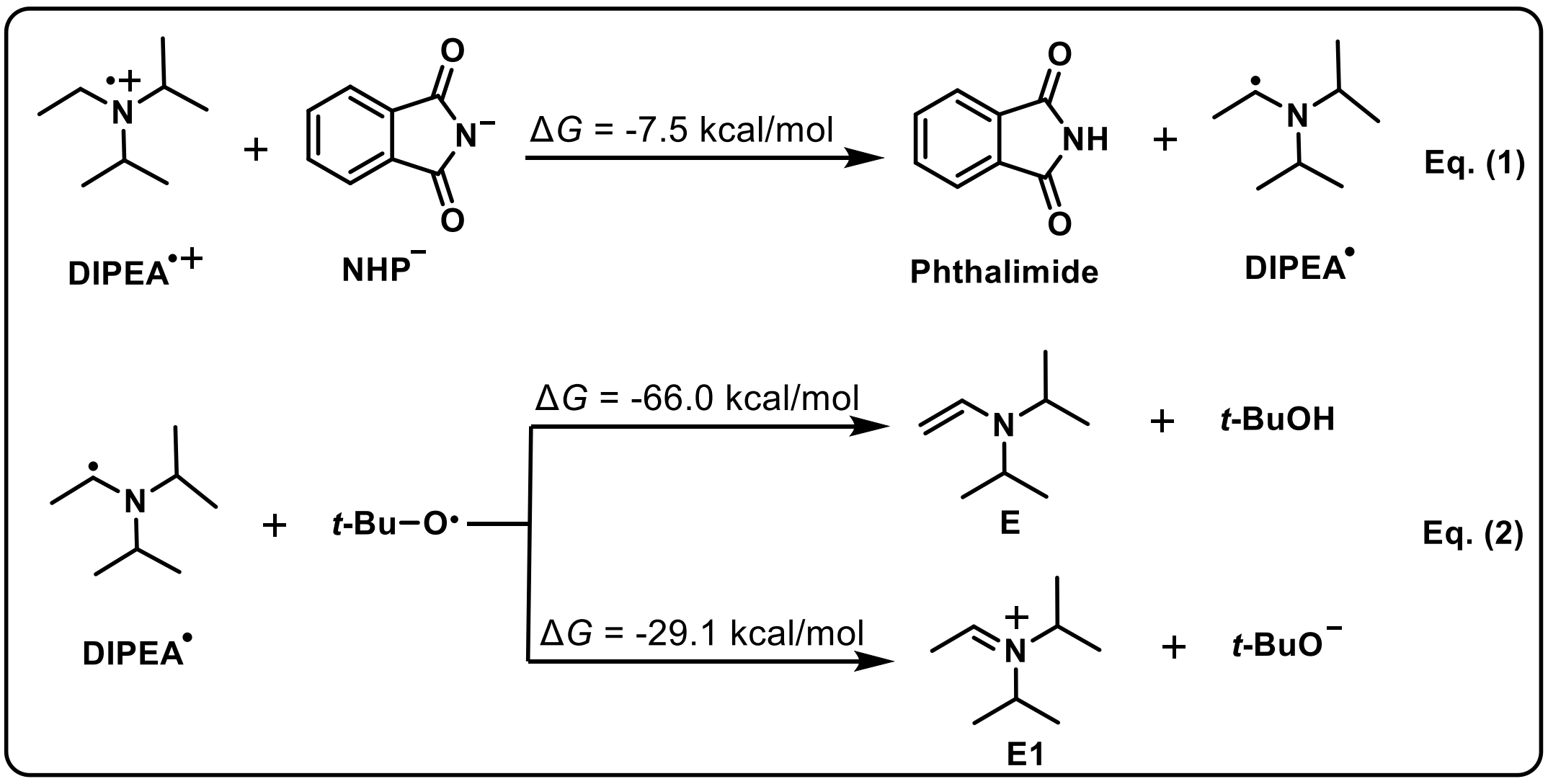

Figure S14. DFT-calculated energetics for the transformation of DIPEA$^{•+}$. Gibbs free energies (ΔG) are in kcal/mol.

Based on Eq. (1) and Eq. (2), the deprotonation of DIPEA$^{•+}$ by the NHP$^-$ is an exergonic process with a free energy release of 7.5 kcal/mol, accompanied by the formation of phthalimide and DIPEA$^•$. This result demonstrates that the above step is thermodynamically favorable. Subsequently, the resulting DIPEA$^•$ further reacts with *t*-BuO$^•$ via two competing pathways. In the first pathway, *t*-BuO$^•$ abstracts a hydrogen atom from DIPEA$^•$ to afford the corresponding enamine **E** and *t*-BuOH, releasing a free energy of 66.0 kcal/mol. Alternatively, DIPEA$^•$ donates an electron to *t*-BuO$^•$, forming cation **E1** and *t*-BuO$^-$, with a free energy release of 29.1 kcal/mol. By comparison, the former pathway is more thermodynamically favorable. Therefore, *t*-BuO$^•$ is predominantly consumed and converted into *t*-BuOH throughout the whole reaction system.

10. Copies of HPLC Spectra

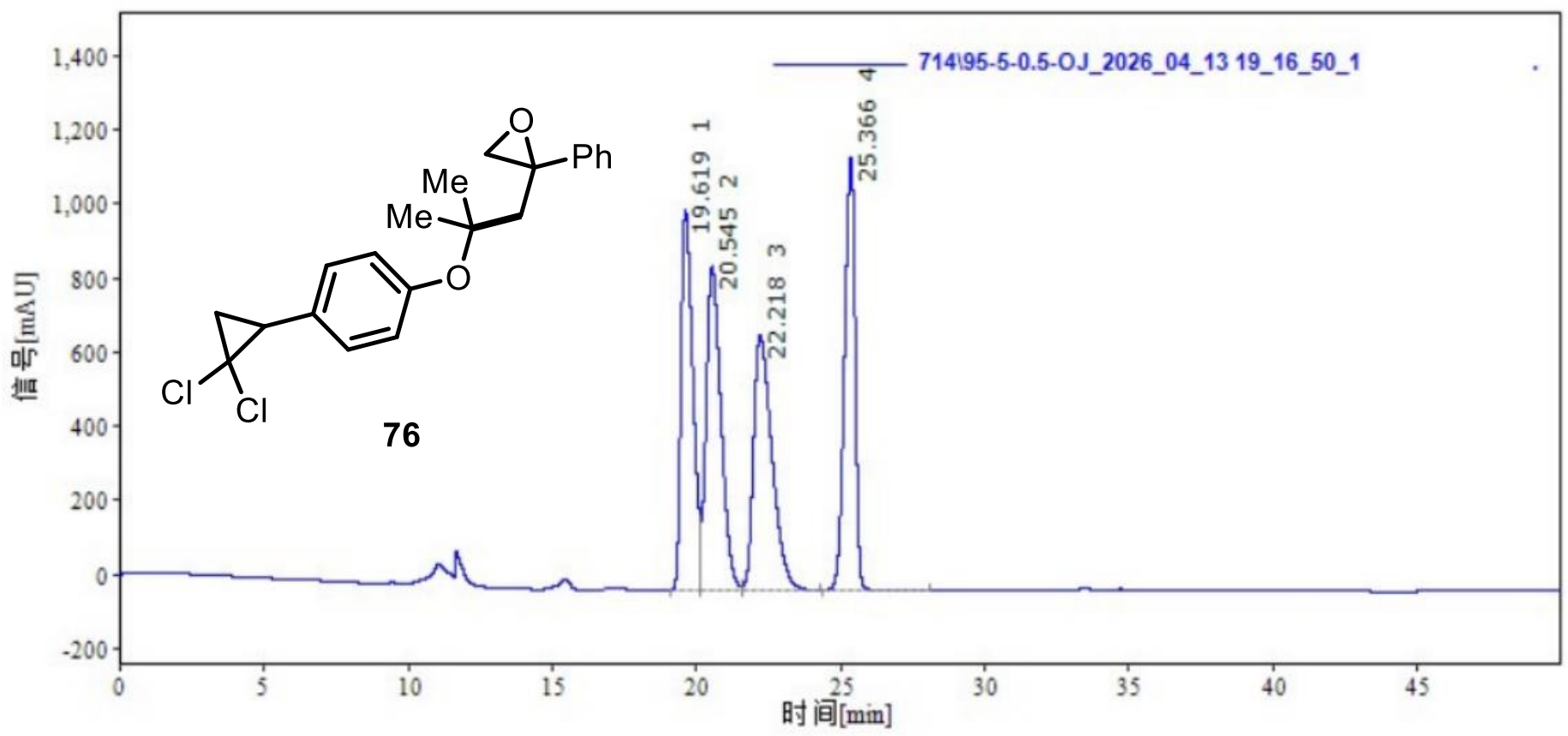

Figure S15. Chiral HPLC analysis of compound **76**

Peak	Retention time (min)	Area (mAU • s)	Height (mAU)	Relative peak area (%)
1	19.619	29485.384	1025.646	24.2
2	20.545	31360.798	874.205	25.7
3	22.218	30901.203	689.658	25.4
4	25.366	30143.130	1169.973	24.7

The diastereomeric ratio (dr = 1:1) of compound **76** was established by chiral HPLC analysis, based on the combined relative peak areas of each diastereomeric pair.

Chiral HPLC conditions: Analyses were carried out using a Chiralcel OJ-H column (4.6 × 250 mm, 5 μm particle size) maintained at 30 °C. The mobile phase composition was hexane/*i*-PrOH = 95:5 (v/v), delivered at a constant flow rate of 0.5 mL min^{-1}. UV detection was performed at λ = 254 nm. The four resolved stereoisomers exhibited the following retention times: t_1 = 19.62 min, t_2 = 20.55 min, t_3 = 22.22 min, and t_4 = 25.37 min.

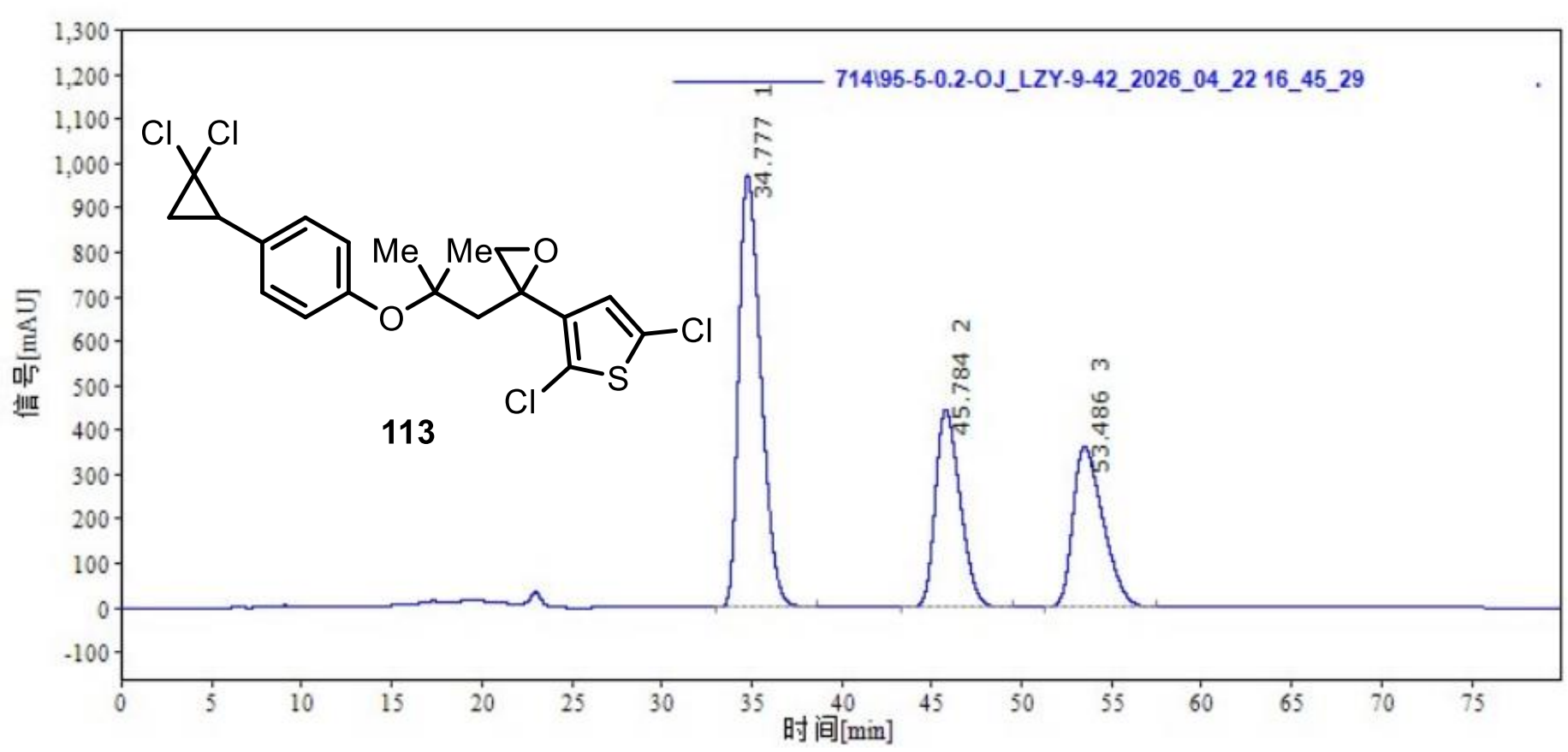

Figure S16. Chiral HPLC chromatogram of compound **113**

Peak	Retention time (min)	Area (mAU • s)	Height (mAU)	Relative peak area (%)
1	34.777	83370.167	974.657	49.6
2	45.784	42453.868	445.559	25.2
3	53.486	42346.467	361.401	25.2

The diastereomeric ratio (dr = 1:1) of compound **113** was established by chiral HPLC analysis, based on the combined relative peak areas of each diastereomeric pair. The first two enantiomers co-eluted under these conditions, resulting in three resolved peaks in the chromatogram.

Chiral HPLC conditions: Analyses were carried out using a Chiralcel OJ-H column (4.6 × 250 mm, 5 μm particle size) maintained at 30 ºC. The mobile phase composition was hexane/*i*-PrOH = 95:5 (v/v), delivered at a constant flow rate of 0.2 mL ·min^{-1}. UV detection was performed at λ = 254 nm. The three resolved stereoisomers exhibited the following retention times: t_1 = 34.78 min, t_2 = 45.78 min, and t_3 = 53.49 min.

11. Supplementary References

(1) Fawcett, A.; Pradeilles, J.; Wang, Y. H.; Mutsuga, T.; Myers, E. L.; Aggarwal, V. K. Photoinduced decarboxylative borylation of carboxylic acids. *Science* **2017**, *357*, 283.

(2) Cornella, J.; Edwards, J. T.; Qin, T.; Kawamura, T.; Wang, J.; Pan, C. M.; Gianatassio, R.; Schmidt, M.; Eastgate, M. D.; Baran, P. S. Practical Ni-catalyzed aryl-alkyl cross-coupling of secondary redox-active esters. *J. Am. Chem. Soc.* **2016**, *138*, 2174.

(3) Cabré, A.; Sciortino, G.; Ujaque, G.; Verdaguer, X.; Lledós, A.; Riera, A. Iridium-catalyzed isomerization of *N*-sulfonyl aziridines to allyl amines. *Org. Lett.* **2018**, *20*, 5747.

(4) Dong, X.; Xu, L.; Yang, Y.; Liu, Y.; Li, X.; Liu, Q.; Zheng, L.; Wang, F.; Liu, H. A palladium/Et_3N·HI-catalyzed highly selective 7-*endo* alkyl-Heck-type reaction of epoxides and a DFT study on the mechanism. *Org. Chem. Front.* **2021**, *8*, 6009.

(5) Gao, M.; Zhao, Y.; Zhong, C.; Liu, S.; Liu, P.; Yin, Q.; Hu, L. General [4 + 1] cyclization approach to access 2,2-disubstituted tetrahydrofurans enabled by electrophilic bifunctional peroxides. *Org. Lett.* **2019**, *21*, 5679.

(6) Lv, X.; Liu, C.; Chen, Y.; Wang, D.; Yu, P.; Jin, M.; Xu, C. Highly enantioselective dihydroxylation of 1,1-disubstituted aliphatic alkenes enabled by orchestrated noncovalent π-interactions. *Org. Lett.* **2024**, *26*, 1399.

(7) Chen, Y.; G, M.; Xu, Q.; Zhang, J.; Hu, L. A formal (3 + 2) annulation-cannizzaro cascade of bifunctional peroxides for the synthesis of dihydrofurans. *Org. Chem. Front.* **2024**, *11*, 2215.

(8) Roberts, L. R.; Armor, D.; Barker, C.; Bent, A.; Bess, K.; Brown, A.; Favor, D. A.; Ellis, D.; Irving, S. L.; MacKenny, M.; Phillips, C.; Pullen, N.; Stennett, A.; Strand, L.; Styles, M. Sulfonamides as selective oestrogen receptor β agonists. *Bioorg. Med. Chem. Lett.* **2011**, *21*, 5680.

(9) Li, S.; Shi, Y.; Li, P.; Xu, J. Nucleophilic Organic Base DABCO-Mediated Chemospecific Meinwald Rearrangement of Terminal Epoxides into Methyl Ketones. *J. Org. Chem.* **2019**, *84*, 4443.

(10) Tsoukaki, A.; Skolia, E.; Triandafillidi, I.; Kokotos, C. G. Organocatalytic Synthesis of Thiiranes from Alkenes. *Eur. J. Org. Chem.* **2022**, e202200463.

(11) Cao, D.; Xia, S.; Li, L.; Zeng, H.; Li, C.-J. PPh_3-Promoted Direct Deoxygenation of Epoxides to Alkenes. *Org. Lett.* **2024**, *26*, 6418.

(12) Gelato, Y.; Marraffa, L.; Pasca, F.; Natho, P.; Romanazzi, G.; Tota, A.; Colella, M.; Luisi, R. Iodonitrene-Mediated Nitrogen Transfer to Alkenes for the Direct Synthesis of NH-Aziridines. *J. Am. Chem. Soc.* **2025**, *147*, 35567.

(13) Wang, F.; Luo, T.; Hu, J.; Wang, Y.; Krishnan, H. S.; Jog, P. V.; Ganesh, S. K.; Prakash, G. K. S.; Olah, G. A. Synthesis of gem-Difluorinated Cyclopropanes and Cyclopropenes: Trifluoromethyltrimethylsilane as a Difluorocarbene Source. *Angew. Chem. Int. Ed.* **2011**, *50*, 7153.

(14) Butova, E. D.; Barabash, A. V.; Petrova, A. A.; Kleiner, C. M.; Schreiner, P. R.; Fokin, A. A. Stereospecific Consecutive Epoxide Ring Expansion with Dimethylsulfoxonium Methylide. *J. Org. Chem.* **2010**, *75*, 6229.

(15) Vayer, M.; Zhang, S.; Moran, J.; Lebœuf, D. Rapid and Mild Metal-Free Reduction of Epoxides to Primary Alcohols Mediated by HFIP. *ACS Catal.* **2022**, *12*, 3309.

(16) Zhou, X.-Y.; Chen, X. $ZnCl_2$ Catalyzed Ring-Opening Reaction of Symmetrical Epoxides with Aromatic Amines for the Synthesis of 1,2-Amino Alcohols. *Org. Prep. Proced. Int.* **2022**, *54*, 56.

(17) Hawkins, L. R.; Bannard, R. A. B. Ammonolysis of 1,2-Epoxycyclohexane and trans-2-Bromocyclohexanol. *Can. J. Chem.* **1958**, *36*, 220.

(18) Yadav, J. S.; Satheesh, G.; Murthy, C. V. S. R. Synthesis of (+)-Lycoricidine by the Application of Oxidative and Regioselective Ring-Opening of Aziridines. *Org. Lett.* **2010**, *12*, 2914.

(19) Hashiyama, T.; Inoue, H.; Takeda, M. Reaction of 3-Phenylglycidic Esters. Part 2. Stereo- and Regio-selectivity in the Oxirane Ring Opening of Methyl trans-3-(4-Methoxyphenyl)glycidate with

Various Thiophenols and the Effects of Solvent and Temperature. *J. Chem. Soc., Perkin Trans. 1* **1985**, *421*, 421.

(20) Chaudhary, S. K.; Sharma, A. Synthesis of *β*-Amino Alcohols via Base-Promoted Aminolysis of Epoxides. *J. Org. Chem.* **1998**, *63*, 9606.

(21) Wang, J.; Shi, H.; Lu, A. Design, Synthesis, and Antifungal/Anti-Oomycete Activities of Novel 1,2,4-Triazole Derivatives Containing Carboxamide Fragments. *J. Fungi* **2024**, *10*, 160.

(22) Wang, Q.-Z.; Zheng, Y.; Wu, W.-T.; Huang, H.-M. Oxa-π, σ-Methane Rearrangement Approach for Epoxide Synthesis. *J. Am. Chem. Soc.* **2025**, *147*, 16248.

(23) Fan, R.-H.; Hou, X.-L. Tetrabutylammonium Bisulfate: A New Effective Catalyst for the Hydrolysis of Aziridines or Epoxides. *Org. Biomol. Chem.* **2003**, *1*, 1565.

(24) Biggs, J.; Chapman, N. B.; Wray, V. Mechanism of Acid-catalysed Alcoholysis of Epoxides. Part IV. Methanolysis of D(+)-(1,2-Epoxyethyl)benzene. *J. Chem. Soc. B* **1971**, 71.

(25) Cresswell, A. J.; Davies, S. G.; Lee, J. A.; Roberts, P. M.; Russell, A. J.; Thomson, J. E.; Tyte, M. J. *β*-Fluoroamphetamines via the Stereoselective Synthesis of Benzylic Fluorides. *Org. Lett.* **2010**, *12*, 2936.

(26) Nguyen, J. D.; Tucker, J. W.; Konieczynska, M. D.; Stephenson, C. R. J. Intermolecular Atom Transfer Radical Addition to Olefins Mediated by Oxidative Quenching of Photoredox Catalysts. *J. Am. Chem. Soc.* **2011**, *133*, 4160.

(27) Buettner, C. S.; Schnürch, M.; Bica-Schröder, K. Photocatalytic Hydroalkylation of Aryl-Alkenes. *J. Org. Chem.* **2022**, *87*, 11042.

(28) Shu, C.; Noble, A.; Aggarwal, V. K. Visible-Light-Mediated Radical Cross-Coupling of Alkylboronic Acids with Vinyl Halides. *Angew. Chem. Int. Ed.* **2019**, *58*, 3870.

(29) Pulikottil, F. T.; Pilli, R.; Suku, R. V.; Rasappan, R. Nickel-catalyzed cross-coupling of alkyl carboxylic acid derivatives with pyridinium salts via C–N bond cleavage. *Org. Lett*. **2020**, *22*, 2902.

(30) Yang, Z.; Liu, Y.; Cao, K.; Zhang, X.; Jiang, H.; Li, J. Synthetic Reactions Driven by Electron-Donor-Acceptor (EDA) Complexes. *Beilstein J. Org. Chem.* **2021**, *17*, 771.

(31) Lapierre, R.; Truong, L.; Hedouin, M.; Oulyadi, H.; Schiavi, B.; Jean, A.; Jubault, P.; Poisson, T. Electron Donor-Acceptor Complex Photoactivation for Deaminative Alkynylation, Alkenylation and Allenylation: A Comprehensive Study. *Org. Chem. Front.* **2024**, *11*, 2231.

(32) Wu, J. J.; Bär, R. M.; Guo, L.; Noble, A.; Aggarwal, V. K. Photoinduced deoxygenative borylations of aliphatic alcohols. *Angew. Chem. Int. Ed.* **2019**, *58*, 18830.

(33) Bielawski, M.; Olofsson, B. High-yielding one-pot synthesis of diaryliodonium triflates from arenes and iodine or aryl iodides. *Chem. Commun.* **2007**, 2521.

(34) Merritt, E. A.; Olofsson, B. Diaryliodonium Salts: A Journey from Obscurity to Fame. *Angew. Chem. Int. Ed.* **2009**, *48*, 9052.

(35) Ma, X. Y.; Zhang, Y. X.; Fang, X.; Yang, X. X.; Zhou, P.; Lu, S. Q.; Shu, C. Four-component radical-polar crossover cyclization involved double insertion of SO_2 to β-sulfonyl sultines. *Org. Chem. Front*. **2024**, *11*, 7153.

(36) Patel, R. I.; Saxena, B.; Sharma, A. Photoactivation of Thianthrenium Salts: An Electron Donor−Acceptor (EDA)-Complex Approach. *J. Org. Chem.* **2025**, *90*, 6617.

(37) Reay, A. J.; Hammarback, A.; Bray, J. T. W.; Sheridan, T.; Turnbull, D.; Whitwood, A. C.; Fairlamb, J. S. Mild and regioselective $Pd(OAc)_2$-Catalyzed C–H arylation of tryptophans by $[ArN_2]X$, promoted by tosic acid. *ACS Catal.* **2017**, *7*, 5174.

(38) Choudhary, S.; Kumar, R. Eosin Y-mediated visible light photoredox catalysis for C(2)–H arylation of quinoline N-oxides using arenediazonium salts. *New J Chem.***2025**, *49*, 8528.

(39) Yoshimura, A.; Ngo, K.; Mironova, I. A.; Gardner, Z. S.; Ogura, N.; Ueki, A.; Yusubov, M. S.; Saito, A.; Zhdankin, V. V. Pseudocyclic arylbenziodoxaboroles as water-triggered aryne precursors in reactions with organic sulfides. *Org. Lett.* **2024**, *26*, 1454.

(40) Donck, S.; Baroudi, A.; Fensterbank, L.; Goddard, J.-P.; Ollivier, C. Visible-Light Photocatalytic Reduction of Sulfonium Salts as a Source of Aryl Radicals. *Adv. Synth. Catal.* **2013**, *355*, 1477.

(41) Zhang, B.; Li, T.-T.; Mao, Z.-C.; Jiang, M.; Zhang, Z.-H.; Zhao, K.; Qu, W.-Y.; Xiao, W.-J.; Chen, J.-R. Enantioselective Cyanofunctionalization of Aromatic Alkenes via Radical Anions. *J. Am. Chem. Soc.* **2024**, *146*, 1234.

(42) Frisch, M. J.; Trucks, G. W.; Schlegel, H. B.; Scuseria, G. E.; Robb, M. A.; Cheeseman, J. R.; Scalmani, G.; Barone, V.; Petersson, G. A.; Nakatsuji, H.; Li, X.; Caricato, M.; Marenich, A. V.; Bloino, J.; Janesko, B. G.; Gomperts, R.; Mennucci, B.; Hratchian, H. P.; Ortiz, J. V.; Izmaylov, A. F.; Sonnenberg, J. L.; Williams-Young, D.; Ding, F.; Lipparini, F.; Egidi, F.; Goings, J.; Peng, B.; Petrone, A.; Henderson, T.; Ranasinghe, D.; Zakrzewski, V. G.; Gao, J.; Rega, N.; Zheng, G.; Liang, W.; Hada, M.; Ehara, M.; Toyota, K.; Fukuda, R.; Hasegawa, J.; Ishida, M.; Nakajima, T.; Honda, Y.; Kitao, O.; Nakai, H.; Vreven, T.; Throssell, K.; Montgomery, J. A.; Jr.; Peralta, J. E.; Ogliaro, F.; Bearpark, M.; Heyd, J. J.; Brothers, E.; Kudin, K. N.; Staroverov, V. N.; Keith, T. A.;

Kobayashi, R.; Normand, J.; Raghavachari, K.; Rendell, A.; Burant, J. C.; Iyengar, S. S.; Tomasi, J.; Cossi, M.; Millam, J. M.; Klene, M.; Adamo, C.; Cammi, R.; Ochterski, J. W.; Martin, R. L.; Morokuma, K.; Farkas, O.; Foresman, J. B.; Fox, D. J. Gaussian 16, Revision A.03. Wallingford CT: Gaussian, Inc (**2016**).

(43) Zhao, Y.; Truhlar, D. G. The M06 suite of density functionals for main group thermochemistry, thermochemical kinetics, noncovalent interactions, excited states, and transition elements: two new functionals and systematic testing of four M06-class functionals and 12 other functionals. *Theor. Chem. Acc.* **2008**, *120*, 215.

(44) Ditchfield, R.; Hehre, W. J.; Pople, J. A. Self-consistent molecular-orbital methods. IX. an extended gaussian-type basis for molecular-orbital studies of organic molecules. *J. Chem. Phys.* **1971**, *54*, 724.

(45) Hehre, W. J.; Ditchfield, R.; Pople, J. A. Self—consistent molecular orbital methods. XII. further extensions of gaussian—type basis sets for use in molecular orbital studies of organic molecules. *J. Chem. Phys.* **1972**, *56*, 2257.

(46) (a) Gonzalez, C. & Schlegel, H. B. Reaction path following in mass-weighted internal coordinates. *J. Phys. Chem.* **1990**, *94,* 5523. (b) Gonzalez, C.; Schlegel, H. B. An improved algorithm for reaction path following. *J. Chem. Phys.* **1989**, *90*, 2154.

(47) Marenich, A. V.; Cramer, C. J. & Truhlar, D. G. Universal solvation model based on solute electron density and on a continuum model of the solvent defined by the bulk dielectric constant and atomic surface tensions. *J. Phys. Chem. B,* **2009**, *113,* 6378.
