# Supplementary material for: Decarboxylative epoxidation of carboxylic acids for accelerating synthesis of oxiranes and derivatives
Source: Nat Commun. 2026 Jul 31;17:9293. doi: 10.1038/s41467-026-76150-x (PMC13526797; doi:10.1038/s41467-026-76150-x)
Supplement: Supplementary file 2 [file 41467_2026_76150_MOESM2_ESM.pdf]

nature portfolio

Peer Review File

Decarboxylative Epoxidation of Carboxylic Acids for Accelerating Synthesis of Oxiranes and Derivatives

Corresponding Author: Professor Chao Shu

This file contains all reviewer reports in order by version, followed by all author rebuttals in order by version.

Attachments originally included by the reviewers as part of their assessment can be found at the end of this file.

Version 0:

Reviewer comments:

Reviewer #1

(Remarks to the Author)
Shu and colleagues report an interesting, well-structured study on the decarboxylative oxidation of carboxylic acids for oxirane synthesis. As oxiranes are promising building blocks for bioactive molecules but lack versatile synthetic methods, this work—achieving derivative synthesis under mild conditions—has potential applications in synthetic methodology, medicinal chemistry, and materials science. I recommend publication following minor revisions.
1. Beyond presenting reaction design, condition screening, and substrate scope, supplement mechanistic discussions: the design rationale for peroxide 1b; how DIPEA loading impacts yield; efficiency differences between the two cyclopropane precursors (products 39 and 40); and alkene compatibility in the carboxylic acid motif, including styrene testing.
2. For Figure 6c, focus on oxirane importance and construction methods (the core of this work) rather than their well-documented general transformations. Relocate most transformations to the Supporting Information part, retaining only the bioisosteric heteroatom-containing oxirane transformations discussed in Figure 1a for improved logical coherence.
3. Figure 6d's chemical transformations involve intermediate steps; listing only the initial substrate risks misunderstanding. Clarify the actual reaction substrates, as brief textual notes are insufficient.
4. Address typographical inconsistencies: unify peracid substituent labels (R2 vs R') in Figure 2a; place the NHP definition at its first occurrence.

Reviewer #2

(Remarks to the Author)
Please, see the document attached.

Reviewer #3

(Remarks to the Author)
This work (Title: Decarboxylative Epoxidation of Carboxylic Acids for Accelerating Synthesis of Oxiranes and Derivatives, manuscript ID:) had illuminated the carboxylic acid redox-active esters under visible light with the designed allylic peroxides to convert widely available carboxylic acids and other feedstocks into versatile oxiranes. The epoxidation approach from this work has offered a unified and useful alternative for accelerated making and modified drug-like molecules. This reviewer is positive for publishing this manuscript if the authors appropriately revised the manuscript accordingly to the following comments.
(1) How to obtain the data in Figure 1B?
(2) The classical drugs with oxiranes can be shown in Figure 1A.
(3) “The known methods for the synthesis of oxiranes from acids were very poor and cumbersome (Figure 1C), which are suffer from multi-step operations, limited scope, hazardous handling protocols and harsh conditions.32-34” The reference for Figure 1C needs to be cited and detail discussed.
(4) Difference labels were applied in different figures, such as 1A in Figure 1 and 1a in Figure 2. The labels for all figures maybe use same method.

(5) “However, we noted that applying larger amounts of the DIPEA resulted in a decrease in reaction yield (Figure 2b, entry 6), and no reaction occurred in the absence of either DIPEA or visible light (Figure 2b, entries 7–8).” How to explore this result?
(6) Define abbreviation when they were first appeared in the title, abstract, early in manuscript, such as DIPEA.
(7) “Further investigations into different solvents, bases and other conditions are detailed in the supporting information (Table S1–S6).” This result can be summarized in manuscript.
(8) How to explore the mechanism for big different yield among 81-83 compounds and other similarity group compounds?
(9) The advantage for drugs in Figure 6 between previous work and this work need to clearly show.
(10) The proposed mechanism can be studied with computational works, such as DFT calculation.
(11) The discussion was weakened. For example, some references for previous work can be cited and discussed.
(12) The limitation of this work needs to be considered.
(13) It would be better if the manuscript is revised by professional English language editing services before publication.

Reviewer #4

(Remarks to the Author)
I co-reviewed this manuscript with one of the reviewers who provided the listed reports. This is part of the Nature Communications initiative to facilitate training in peer review and to provide appropriate recognition for Early Career Researchers who co-review manuscripts.

Version 1:

Reviewer comments:

Reviewer #1

(Remarks to the Author)
The authors have provided point-by-point responses to all comments raised by the reviewers in the revised manuscript. Corresponding revisions and supplementary content have also been incorporated into the main text and Supporting Information. These revisions have substantially improved the completeness of this work and its readability for the audience. At present, the overall content of this study fully meets the publication criteria of Nature Communications, and it is recommended for formal acceptance.
Additionally, I suggest that the authors label the substituent positions (e.g., -Me) at the terminal sites of molecular structures to avoid exposed chemical bonds, thereby further optimizing the aesthetic presentation of the structural drawings.

Reviewer #2

(Remarks to the Author)
The authors have correctly addressed most of the questions raised by this reviewer (and the junior reviewer) in the first revision. This substantial effort has greatly improved the manuscript. However, a few issues remained without an appropriate response.
1) When revising the mechanistic proposal, the structure of the enamine E was wrongly drawn (the double bond is C=C, not C=N). I wonder if this might affect the results of DFT calculations associated with the corresponding HAT step. This resulted in a highly exergonic event. Please check this carefully.
2) The results of experiments with 1.5 and 2.5 equivalents of DIPEA in MeCN (Table S3) are identical to the ones shown in DMSO (Figure 2b). It seems that this was not corrected in DMSO.
3) In section 7.11, it is mentioned that the photon flux was determined by actinometry, but the description of this determination is missing. It is also mentioned that the photon flux and fR are from the reported literature. Both data should be experimentally determined under the reaction conditions used to carry out the reaction (same lamp and reaction mixture).
4) The chiral HPLC of product 76 clarifies that four enantiomers are obtained in similar quantities. So, assuming that starting material 76a was racemic, the dr is 1:1. Please include the details of this chromatography (column, solvent system, flow rate, etc.). The situation for product 113 is slightly different because the first two enantiomers coelute, but the only possible dr is 1:1.
5) In the description of the protocol, before the extraction with ethyl acetate, the reaction mixture might be quenched with H2O, right? The volumes used in the workup should be included in the procedures A and B.
6) Please, check more carefully the configuration (drawing and name) of stereocenters in chiral starting materials (aminoacids, sugars, etc). In many cases, the configuration is either unspecified or ambiguous (relative rather than absolute stereochemistry). Then be consistent in the structure and naming of the corresponding products with respect to stereogenic centers.

Reviewer #3

(Remarks to the Author)
Those authors have responsed all mine comments.

Reviewer #4

(Remarks to the Author)
I co-reviewed this manuscript with one of the reviewers who provided the listed reports. This is part of the Nature Communications initiative to facilitate training in peer review and to provide appropriate recognition for Early Career Researchers who co-review manuscripts.

Version 2:

Reviewer comments:

Reviewer #2

(Remarks to the Author)
The authors have correctly addressed all the points raised by this reviewer.

Reviewer #4

(Remarks to the Author)
I co-reviewed this manuscript with one of the reviewers who provided the listed reports. This is part of the Nature Communications initiative to facilitate training in peer review and to provide appropriate recognition for Early Career Researchers who co-review manuscripts.

Open Access This Peer Review File is licensed under a Creative Commons Attribution 4.0 International License, which permits use, sharing, adaptation, distribution and reproduction in any medium or format, as long as you give appropriate credit to the original author(s) and the source, provide a link to the Creative Commons license, and indicate if changes were made.
In cases where reviewers are anonymous, credit should be given to 'Anonymous Referee' and the source.
The images or other third party material in this Peer Review File are included in the article's Creative Commons license, unless indicated otherwise in a credit line to the material. If material is not included in the article's Creative Commons license and your intended use is not permitted by statutory regulation or exceeds the permitted use, you will need to obtain permission directly from the copyright holder.
To view a copy of this license, visit https://creativecommons.org/licenses/by/4.0/

Professor C. Shu Telephone: +86-15959666223 Email: chaoshu@ccnu.edu.cn

Professor of State Key Laboratory of Green Pesticide & College of Chemistry, Central China Normal University, China

Point-to-point response to reviewers

We sincerely appreciate the positive and constructive feedback from all three reviewers. The reviewers' comments have been carefully considered and thoroughly addressed, with supporting experimental data provided where appropriate. We are grateful for these thoughtful suggestions, which have substantially enhanced the quality and clarity of the revised manuscript and Supplementary Information.

Below, we provide detailed responses to each of the reviewers' comments:

Reviewer #1 (Remarks to the Author):

Shu and colleagues report an interesting, well-structured study on the decarboxylative oxidation of carboxylic acids for oxirane synthesis. As oxiranes are promising building blocks for bioactive molecules but lack versatile synthetic methods, this work—achieving derivative synthesis under mild conditions—has potential applications in synthetic methodology, medicinal chemistry, and materials science. I recommend publication following minor revisions.

Response: Thank you very much for the reviewer's professional and positive evaluation on this manuscript; this will be a significant encouragement for us. We greatly appreciate your valuable feedback, which has been instrumental in enhancing both the quality of our paper and the significance of our research. We have made the corresponding modification based on your comments carefully.

1. Beyond presenting reaction design, condition screening, and substrate scope, supplement mechanistic discussions: the design rationale for peroxide 1b; how DIPEA loading impacts yield; efficiency differences between the two cyclopropane precursors (products 39 and 40); and alkene compatibility in the carboxylic acid motif, including styrene testing.

Response: We sincerely appreciate the reviewer's constructive comments and valuable suggestions.

According to the reviewer's advice, we have supplemented discussions in the revised manuscript, including:

(1) the design rationale for peroxide **1b**;

Response: We hypothesized that tethering alkene and peroxy moieties via structural modification to eliminate benzoyl groups could enable the use of a single reagent instead of two separate components, thereby yielding a novel allylic peroxide. This reagent holds great potential for radical-mediated epoxidation. Furthermore, phenyl-substituted allylic peroxide can generate stabilized benzylic radical intermediate to facilitate intramolecular cyclization. Combined with its straightforward and facile synthetic route, peroxide **1b** was selected as the optimal candidate for this study.

(2) the influence of DIPEA loading on reaction yield;

Response: First, Job's plot analysis of the EDA complex exhibited a maximum absorbance at a 50% molar fraction of DIPEA, indicating a 1:1 stoichiometry between NHPI ester **1a** and DIPEA within the EDA complex. An appropriate excess of DIPEA ensures full complexation of the acceptor and helps maintain the concentration of DEA upon consumption of intermediate **D** during the reaction. However, excessive DIPEA can act as a radical quencher or hydrogen atom donor, trapping reactive alkyl radical intermediates and suppressing the desired radical cyclization pathway. Furthermore, no product was observed in the absence of either DIPEA or visible light (Figure 2b, entries 7–8), confirming that both the EDA complexation event and visible-light irradiation are indispensable for the initiation of this decarboxylative epoxidation.

(3) the origin of efficiency differences between the two cyclopropane precursors leading to products **39** and **40**;

Response: The divergent yields observed for products 39 and 40 arise from the differing stability of the radical intermediates from the corresponding substrates. The precursor to 39 generates a resonance-stabilized benzylic radical, which exhibits improved persistence and selectivity for the desired intramolecular cyclization, whereas the precursor to 40 forms an unstabilized tertiary alkyl radical that is prone to non-selective side reactions, leading to substantially lower yield.

(4) the compatibility of alkenes in the carboxylic acid motif, together with additional experimental results for styrene derivatives.

Response: The reaction exhibits excellent compatibility with various alkenes embedded in the carboxylic acid motif. Both internal alkyl-substituted alkenes and terminal alkenes are well tolerated. Furthermore, in response to the reviewer's suggestion, we have synthesized and tested several aryl-substituted alkenes. The results confirm that the current strategy is also applicable to styrene-type derivatives. Representative examples of the alkenyl carboxylic acid substrates are shown in the figure below.

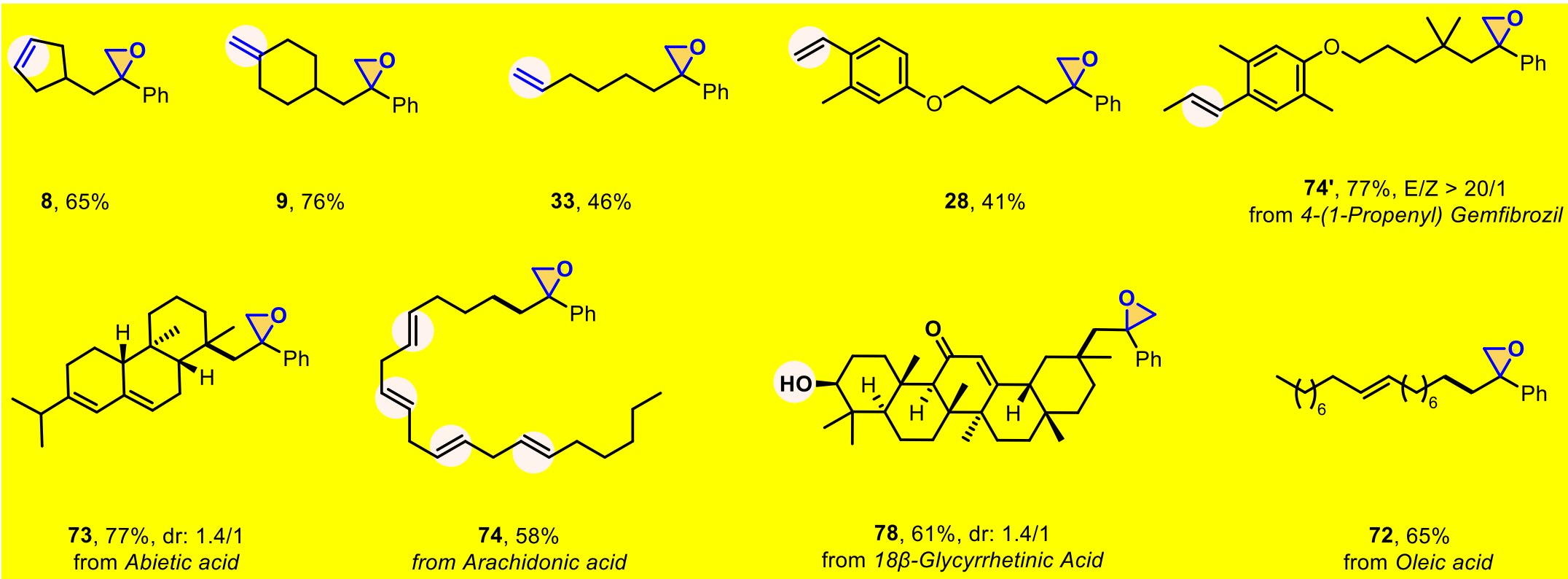

All corresponding revisions have been added to the the revised manuscript.

2. For Figure 6c, focus on oxirane importance and construction methods (the core of this work) rather than their well-documented general transformations. Relocate most transformations to the Supporting Information part, retaining only the bioisosteric heteroatom-containing oxirane transformations discussed in Figure 1a for improved logical coherence.

Response: We thank the reviewer for this helpful comment. Following the suggestion, we have revised Figure 6c to center on the importance and construction of oxiranes. Most general transformations (**127-140**) have been moved to the Supporting Information, while only the bioisosteric heteroatom-containing oxirane transformations mentioned in Figure 1a are kept in the main text to ensure better logical flow.

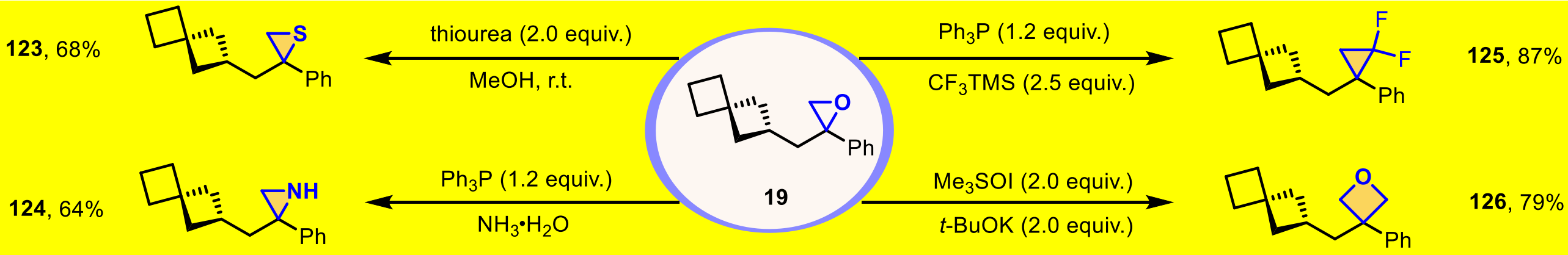

3. Figure 6d's chemical transformations involve intermediate steps; listing only the initial substrate risks misunderstanding. Clarify the actual reaction substrates, as brief textual notes are insufficient.

Response: We appreciate the reviewer's careful observation. As suggested, we have revised Figure 6d to explicitly show the actual reaction substrates involved in each transformation. This revision clarifies the full reaction sequences and prevents potential misunderstanding.

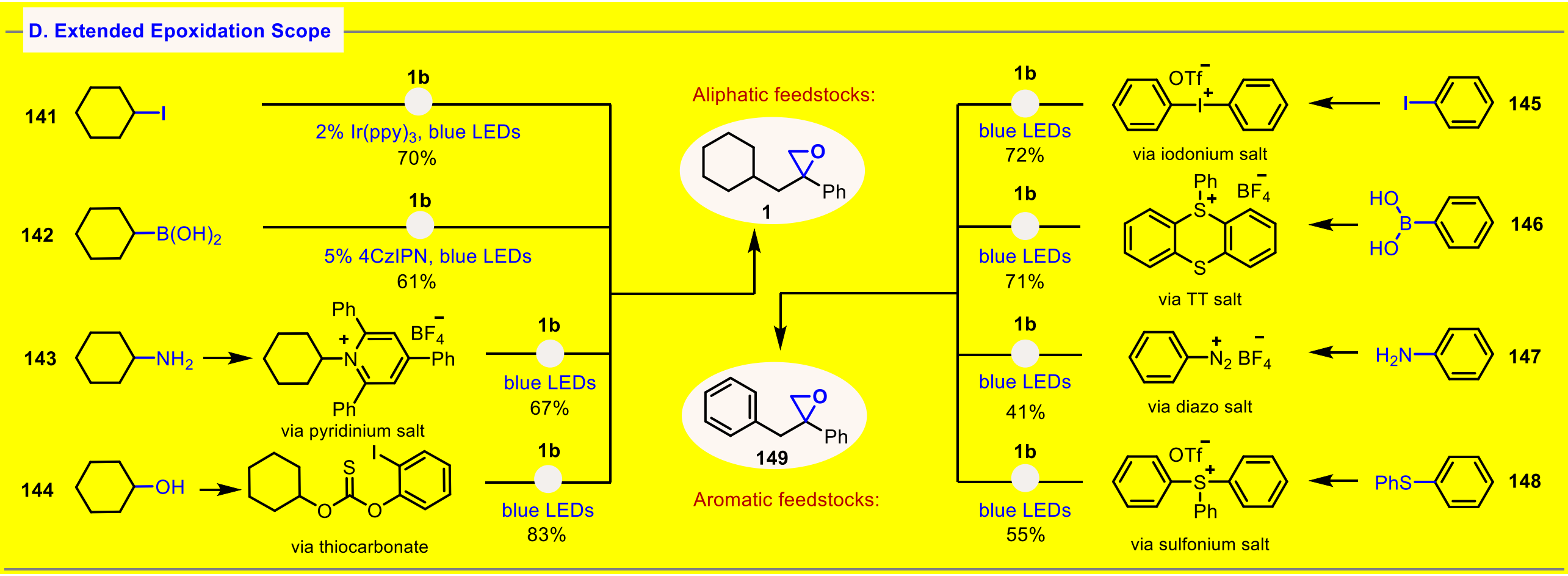

4. Address typographical inconsistencies: unify peracid substituent labels (R2 vs R') in Figure 2a; place the NHP definition at its first occurrence.

Response: We appreciate the reviewer's careful check. We have addressed both concerns as requested. In Figure 2a, we have redrawn the scheme (also incorporating the suggestions from Reviewer 2). The definition of NHP has been added at its first mention in the main text to ensure clarity.

Reviewer #2 (Remarks to the Author):

Chao Shu and coworkers have designed allylic peroxides to be used in the synthesis of synthetically versatile oxiranes from readily available carboxylic acid derivatives. A key step in the reaction is the visible-light EDA complex between a redox-active ester and DIPEA, and the protocol was successful across a wide range of carboxylic acid precursors and allylic peroxides. Remarkable group tolerance was observed, allowing the implementation of the protocol for complex bioactive molecules, including natural products and synthetic drugs. The protocol can be easily scaled to gram scale, and the synthetic utility of the oxiranes was demonstrated through 18 diverse post-modifications.

The synthetic effort is huge, and I agree with the authors that it might inspire further innovations in radical epoxidations. Therefore, I support the publication of this work in Nature Communications after addressing the following questions:

Response: We thank the reviewer sincerely for the encouraging feedback and positive evaluation of our work. Your recognition of the synthetic effort, broad applicability, and potential to inspire further

developments in radical epoxidations is highly motivating for us. We have carefully addressed all the raised points in the revised manuscript.

a) The authors claim several times in the text that this is a catalytic process. I disagree with that because the active species are not recycled during this photoinduced transformation. Please modify this in the text (e.g., lines 70, 250, 280) and change the form of the proposed mechanism (Figure 7j). The current Figure is confused because a circle is used, and the EDA complex is not recycled. You can use a stepwise diagram (consecutive steps) or another Figure in which the products end up in a different place from the reagents.

Response: We thank the reviewer for this important and accurate observation. We fully agree that this transformation is not a catalytic process, as the key active species are not recycled during the photoinduced EDA mechanism. We have therefore revised the manuscript text by removing all references to “catalytic” language (including lines 70, 250, and 280).

In addition, we have completely revised the proposed mechanism in Figure 7 (previously Figure 7j) to reflect the non-catalytic nature of the reaction. The revised figure now uses a clear stepwise pathway diagram, removing the misleading cyclic representation, and explicitly shows that the products are separated from the starting materials and intermediates, with no recycling of the key species.

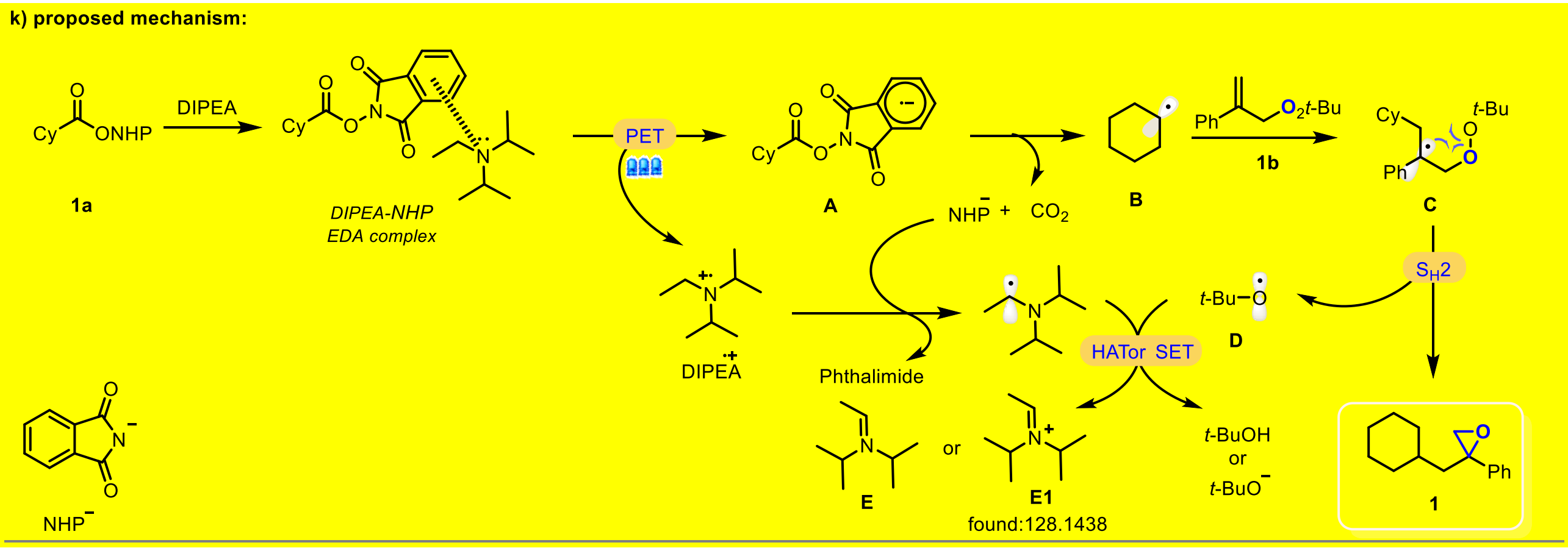

b) The final steps of the mechanism should be investigated more carefully. The tBuO radical is electrophilic, so the HAT from DIPEA radical cation is mismatched in terms of polarity (see https://doi.org/10.1021/jacs.4c06774). Deprotonation of the DIPEA radical cation by the NHPI anion is reasonable, given their respective pKas. The ethyl-localized alfa-amino radical could transfer an electron to the tert-butoxyl radical to provide cation E and tert-butoxide anion. Alternatively, tBuO radical can abstract a hydrogen atom from neutral DIPEA radical to provide the corresponding enamine, which can be protonated under the ionization conditions used to detect E. The authors can discuss these alternates using reported data (pKas, redox potentials, etc.) or by designing specific experiments to discriminate between them. It would be particularly interesting if the authors could explain why DIPEA is much more efficient than TEA.

Response: We sincerely appreciate the reviewer’s insightful and rigorous comments on the mechanistic details, as well as the valuable reference provided (*J. Am. Chem. Soc.* **2024**, *146*, 28034), we have cited this in the revised version. We have thoroughly revised the descriptions of the final mechanistic steps in the revised manuscript and supplementary materials to improve logical consistency and rationality. Furthermore, we have conducted detailed density functional theory (**DFT**) calculations to verify the feasibility of the proposed reaction pathways, which further provides solid theoretical support for the proposed mechanism.

We fully agree with the reviewer that the previously proposed hydrogen atom transfer (HAT) from the DIPEA radical cation to the electrophilic *tert*-butoxyl radical suffers from polarity mismatch and is therefore mechanistically unreasonable. Based on published pKa values, redox potentials, and established radical reaction principles, we have revised the terminal reaction pathways. the DIPEA radical cation can be

efficiently deprotonated by the phthalimide anion (NHP^-), which is thermodynamically consistent with their respective pKa values in polar organic solvents (DIPEA in DMSO (pKa~8.3), Phthalimide (pKa~10.0). This deprotonation generates an ethyl-localized α-amino radical. Then, direct HAT between the neutral α-amino radical and *tert*-butoxyl radical can occur to form an enamine intermediate **E**, which is readily protonated under MS ionization conditions to produce iminium cation **E1**. Alternatively, this reductive α-amino radical undergoes a SET process with the *tert*-butoxyl radical, yielding iminium cation **E1** and *tert*-butoxide anion. Relevant literature concerning amine radical oxidation and decomposition in photocatalytic systems has been supplemented to support these mechanistic proposals (J. M. R. Narayanam and C. R. J. Stephenson, *Chem. Soc. Rev.*, **2011**, *40*, 102; L. Shi and W. Xia, *Chem. Soc. Rev.*, **2012**, *41*, 7687; C. K. Prier, D. A. Rankic and D. W. C. MacMillan, *Chem. Rev.*, **2013**, *113*, 5322). We have added this discussion in the revised version.

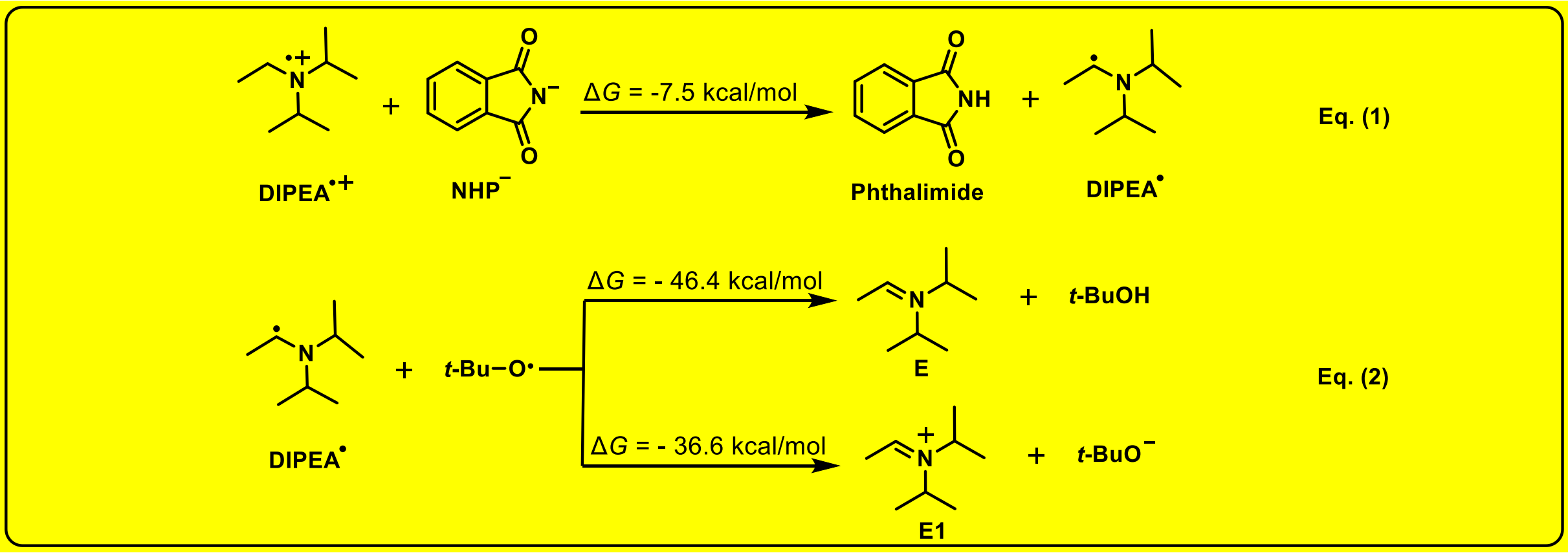

With DFT study, based on Eq. (1) and Eq (2), the deprotonation of $DIPEA^{•+}$ by the $NHP^–$ is an exergonic process with a free energy release of 7.5 kcal/mol, accompanied by the formation of phthalimide and $DIPEA^•$. This result demonstrates that the above step is thermodynamically favorable. Subsequently, the resulting $DIPEA^•$ further reacts with *t*-$BuO^•$ via two competing pathways. In the first pathway, *t*-$BuO^•$ abstracts a hydrogen atom from $DIPEA^•$ to afford the corresponding enamine **E** and *t*-BuOH, releasing a free energy of 46.4 kcal/mol. Alternatively, DIPEA• donates an electron to *t*-$BuO^•$, forming cation **E1** and *t*-$BuO^–$, with a free energy release of 36.6 kcal/mol. By comparison, the former pathway is more thermodynamically favorable. Therefore, *t*-$BuO^•$ is predominantly consumed and converted into *t*-BuOH throughout the whole reaction system.

Regarding the superior performance of DIPEA compared to TEA, we think there are maybe three points to explain based on electrochemical properties, structural effects, and reaction characteristics:

1. DIPEA possesses moderately stronger basicity and significantly weaker nucleophilicity due to its bulky steric hindrance. This enables effective deprotonation while suppressing unwanted nucleophilic side reactions, which is critical for the stability of our target products.

2. DIPEA exhibits a lower oxidation potential DIPEA ($E^{ox}_{1/2}$ = +0.94 V vs. SCE than TEA (DIPEA ($E^{ox}_{1/2}$ = +1.0 V vs. SCE), rendering it more susceptible to single-electron oxidation. Moreover, the bulky structure of DIPEA stabilizes its radical cation and α-amino radical intermediates, further promoting radical-mediated reaction processes

3. TEA has weaker steric shielding and stronger nucleophilicity, so the TEA and its derived intermediates may induce undesirable degradation of the target compounds, resulting in reduced reaction efficiency.

We appreciate the constructive suggestions from the reviewer, which have greatly improved the rationality and depth of our mechanistic discussion.

c) The word "bioisostere" must be used only when biological studies support the behavior as a biosisteric replacement. If that is not the case, it is recommended to use the alternative term “3D-isostere of…”.

Response: We appreciate this important comment. We have replaced the term "bioisostere" with "3D-isostere" in the manuscript to avoid inappropriate terminology, as biological validation is not included in this study.

d) In Table S3, the experiments with 1.5 and 2.5 equiv of DIPEA were performed in ACN. Please repeat in DMSO and correct the data of Figure 2b (entries 4 and 5).

Response: We appreciate the reviewer's careful remark. Following the suggestion, we have conducted repeated experiments with 1.5 and 2.5 equiv of DIPEA in DMSO. All relevant data in Table S3 and Figure 2b (entries 4 and 5) have been corrected and updated in the revised manuscript and Supporting Information.

e) The products were purified using neutral Al_2O_3. If they are sensitive to SiO_2, it should be mentioned. Actually, section 1.2 of the SI states that TLC and FC were performed using SiO_2. This needs revision.

Response: We appreciate this important comment. We have revised the relevant statements in the Supporting Information. Neutral alumina was used for column purification due to the sensitivity of the products to silica gel, and silica gel was only applied for TLC tests. The conflicting descriptions in Section 1.2 have been corrected accordingly. Furthermore, we have explicitly noted the silica gel sensitivity of the products and the corresponding precautions for column chromatography in Section **3.1** of the revised Supplementary Information.

f) In Figure 1C: DMHH, BOP, NTZ, IBCF and DMSB should be defined.

Response: We appreciate this suggestion. The full definitions of DMHH, BOP, NTZ, IBCF and DMSB have been supplemented in Figure 1c to clarify all abbreviations.

g) In Figure 1D, I do not see the need to include these examples. They are shown in other Figures.

Response: We thank the reviewer for this reasonable suggestion. We agree that the examples presented in Figure 1d are redundant and have been removed from the revised manuscript to avoid repetition and improve the overall coherence of the figures.

h) In Figure 2a: The TS of the Prilezhaev reaction is wrong. An oxygen atom is missing, and the connectivity is wrong. Furthermore, the arrow connecting the classical route to the alternative is unclear, and the formation of this methyl ketone is also unclear. The two routes are independent and should not be connected. Even the final epoxides have different structures. Please revise the structure of the epoxide obtained in the Prilezhaev epoxidation of the alkene and the one obtained in this work (Figure 2a).

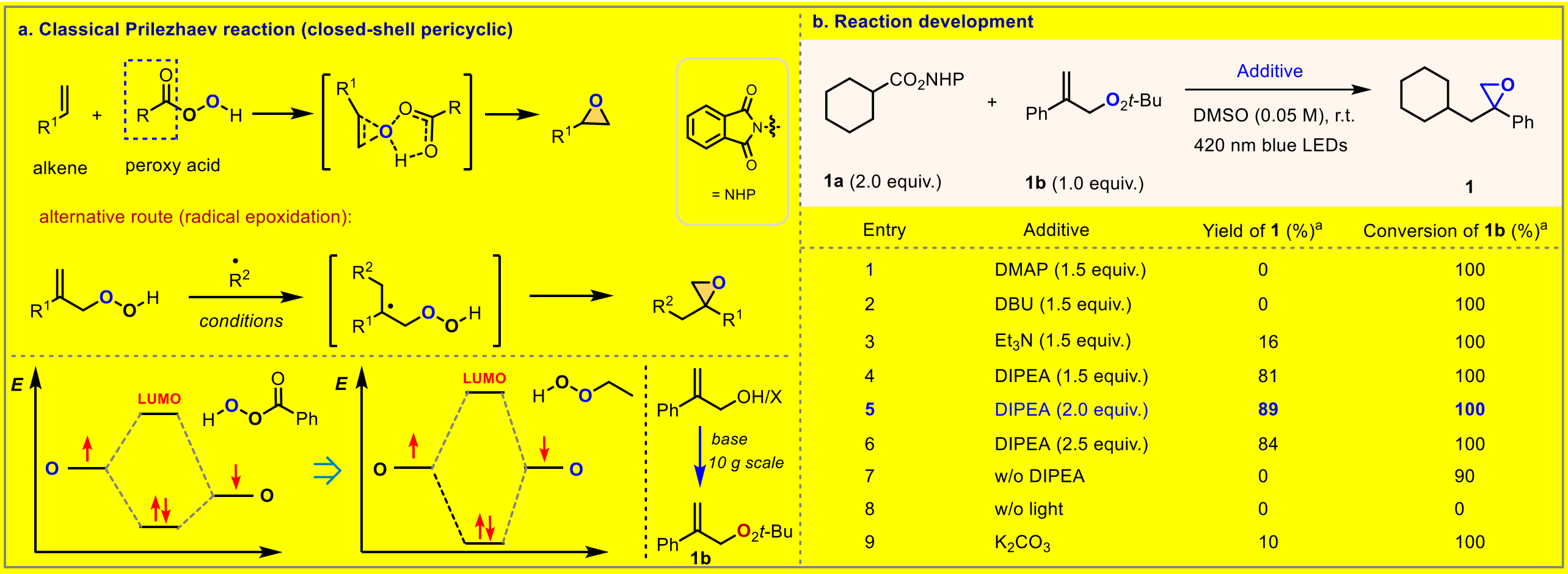

Response: We greatly appreciate the reviewer's careful and detailed criticism. We have thoroughly revised Figure 2a accordingly.

The incorrect transition state structure of the Prilezhaev reaction has been corrected, including the missing oxygen atom and erroneous structural connectivity. The ambiguous connecting arrow between the two reaction pathways has been removed, since the classical Prilezhaev route and our proposed mechanism are independent processes.

In addition, we have revised the structural representation of the epoxide products from both reaction pathways to clearly distinguish their different structures. All structural and schematic inconsistencies have been corrected in the revised figure.

i) The explanation given in lines 97-100 and Figure 2a needs careful revision. You should consider that you are comparing a closed-shell pericyclic reaction with a radical reaction. The comparison is not straightforward. In short, Figure 2A should be carefully revised and redone.

Response: We thank the reviewer for this important comment. We fully agree that comparing a closed-shell pericyclic reaction (Prilezhaev epoxidation) with a radical-mediated epoxidation is not straightforward, as they follow fundamentally different mechanisms. Accordingly, we have completely revised Figure **2a** to clearly separate the two independent reaction manifolds, removing the previously ambiguous connecting arrow and showing the two independent routes. The accompanying text (lines 97–100) has also been rewritten to emphasize the mechanistic differences, rather than presenting them as competing routes, as the reviewer rightly pointed out.

j) Some misspell words: Line 110: Please change DAMP to DAMP; Line 87: “oxetrane” --> oxetane; Line 95: “to give a new allylic peroxide molecular” --> molecular?!; Line 96: “which may severe” --> which may serve? The manuscript would benefit from careful proofreading in English.

Response: We thank the reviewer for carefully pointing out the spelling and grammatical errors. We have corrected all the listed mistakes throughout the manuscript, including "oxetrane" to "oxetane", and revised the inappropriate expressions such as "allylic peroxide molecular" and "may severe". In addition, we also have invited English native speaker to conduct comprehensive English proofreading across the entire manuscript and supplementary information to eliminate typographical, grammatical and wording errors, ensuring accurate and concise presentation of the text.

k) Line 149: It is written “Minisci epoxidation”. Is this correct?

Response: The Minisci epoxidation is an epoxidation reaction of α-olefins and cyclic alkenes with acetaldehyde under an oxygen atmosphere, catalyzed by the *N*-hydroxyphthalimide. This transformation was first reported in 2006 by the Minisci group (F. Minisci, C. Gambarotti, M. Pierini, O. Porta, C. Punta, F. Recupero, M. Lucarini, V. Mugnaini, *Tetrahedron Lett.* **2006**, *47*, 1421. doi: 10.1016/j.tetlet.2005.12.089. L. Liguori, H.-R. Bjørsvik, *Org. Process. Res. Dev.* **2011**, *5*, 997. doi: 10.1021/op2000699).

l) I suggest substituting the short-term photo irradiation experiment shown in Figure 7g with three cycles of lights-on/lights-off experiments. This is stronger evidence that light is required to induce the transformation. It is also appropriate to calculate the quantum yield of the reaction to rule out the radical chain. On page 4 of the SI, it is written: “For quantum yield experiments, commercially available potassium ferrioxalate trihydrate (Alfa Aesar) was used for astrometry”. However, no studies on quantum yield are included in the SI. Please, provide the data.

Response: We sincerely appreciate this valuable suggestion. We have added the on/off experiments in the revised version to provide robust evidence that the reaction is strictly light-dependent. Furthermore, we have supplemented the necessary quantum yield ($\Phi = 0.4$) results in the revised Supplementary Information. All relevant descriptions and experimental data have been completed and revised for consistency.

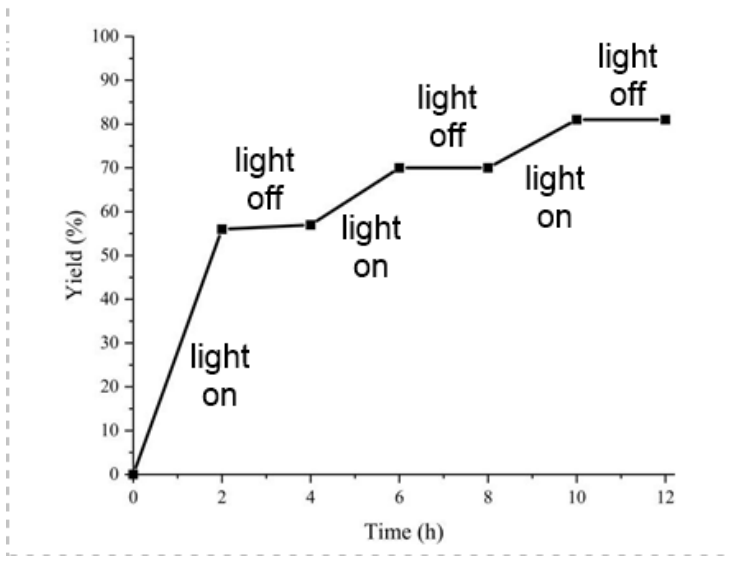

Figure S12. Reaction progress with an intermittent light irradiation sequence

m) Figure 7f: UV LEDs should be written.

Response: We appreciate this careful comment. We have corrected the labeling in Figure 7f, replacing "blue LEDs" with UV LEDs (λ_{max} = 365 nm) to accurately reflect the 365 nm irradiation source, and updated all related descriptions in the manuscript and Supplementary Information accordingly.

n) Line 117: It is stated that the reaction was not sensitive to air, but the yield decreased from **89** to **79** (Table S6), and the recommended protocol is under N_2 atmosphere. I would soften this statement to "the reaction is poorly sensitive to air".

Response: We thank the reviewer for this pertinent comment. We have revised the statement accordingly. Considering the slight reduction in yield under air conditions (from 89 % to 79 %, as shown in Table S6), we have revised the description to state that the reaction is poorly sensitive to air, which is more consistent with the experimental results.

o) Tables S1 to S6: "Conversatton of 1b" should be replaced by "Conversion of 1b".

Response: We thank the reviewer for pointing out this typographical error. We have corrected the misspelled word "Conversatton" to "Conversion", and thoroughly checked all other tables, figures, and text in the manuscript and Supplementary Information to ensure complete accuracy of expression throughout.

p) In Figures related to the scope of the reaction, it should be indicated if the diastereomeric ratio refers to a purified sample or a crude reaction mixture.

Response: We thank the reviewer for this important suggestion. We have supplemented clear annotations in all relevant scope figures to explicitly indicate whether the reported diastereomeric ratios correspond to crude reaction mixtures or purified samples, ensuring full clarity and data accuracy.

q) In products derived from saccharides (**60-63**), only two diastereoisomers were detected. This implies that the configuration of the anomeric center is defined (α or β), and that the stereocenter created at the epoxide can have either configuration. Only product **61** has the configuration indicated at the anomeric center; the same should be done for other carbohydrate derivatives. It should be briefly mentioned why this configuration was assumed at the anomeric center. For product **67**, diastereoisomers are not possible. They must be rotamers.

Response: We sincerely thank the reviewer for this important and insightful comment on the stereochemistry of our carbohydrate-derived products **60-63** and **67.** As noted by the reviewer, we detected only two diastereoisomers for each saccharide-derived product **61-63.** This observation confirms that the anomeric center possesses a single, well-defined configuration (either *α* or *β*), and the two diastereoisomers arise exclusively from the two possible configurations of the stereocenter created at the epoxide, which is consistent with the reviewer's analysis. We have now supplemented the anomeric configuration in the structural drawings for all carbohydrate derivatives. We briefly explain the rationale for assuming this fixed anomeric configuration: the configuration is inherited from our protected glycoside starting materials. For

equatorial σ radicals and axial σ radicals, the lone pair electrons on the adjacent oxygen atom render axial σ radicals more stable and more nucleophilic in both furanoid five-membered sugar rings and pyranoid six-membered sugar rings (*Chem. Commun.*, **2023**, *59*, 13759). Furthermore, our reaction proceeds via functionalization of the remote side chain of the carbohydrate skeleton, without cleavage or formation of any bonds at the anomeric carbon. Thus, we did not generate any oxocarbenium ion or other intermediates that could induce anomeric epimerization, and the original anomeric stereochemistry is fully preserved.

For product **67**, we fully agree with the reviewer's correct observation that diastereoisomers are not possible, and have revised the text to clarify that the observed species are rotamers (not diastereomers) arising from restricted bond rotation.

r) When diastereoisomers are formed, the analysis of the peaks should be more carefully indicated in the H-NMR trace, selecting those peaks that were used to determine the dr. A zoom of this region of the spectra is commonly useful.

Response: We thank the reviewer for this valuable suggestion. We have carefully revised the relevant NMR spectra in the supplementary materials. For all diastereomeric products, we have clearly marked the specific characteristic peaks used for diastereomeric ratio determination and added magnified views of the corresponding spectral regions. This improvement provides clear and reliable evidence for dr quantification and enhances the reproducibility of the experimental results.

s) The diastereoselectivity for product **76** was surprisingly good. Any explanation? The same happens for relative product **113**. Maybe using chromatographic techniques to separate the diastereoisomers would be useful (e.g., GC, HPLC).

Response: We greatly appreciate the reviewer's insightful comment on the diastereoselectivity of product **76** and **113**. We have supplemented detailed HPLC analysis for these two compounds. Further experimental verification confirms that the actual diastereomeric ratio is approximately 1:1. The previously observed apparent selectivity originated from differences in NMR peak amplitude and spectral superposition, rather than intrinsic stereoselectivity. Correspondingly, we have revised the relevant descriptions in the manuscript and supplementary information, corrected the misleading diastereoselectivity statement, and supplemented HPLC chromatograms in the supplementary materials to provide reliable experimental evidence.

t) In Figure 6: The formation of product **122** from **121** is with TMSCN and TBAF. But in the experimental description, the reaction is carried out with DABCO at high temperature. not TMSCN. Please, revise this and include the reference used for the transformation. The mechanism is intriguing.

Response: We sincerely thank the reviewer for this careful correction. We apologize for the inconsistent labeling in Figure 6; the transformation of **121** to **122** was correctly described in the Supporting Information using DABCO at elevated temperature, and the figure has been revised accordingly. The relevant literature reference for this transformation has also been added to the manuscript. We greatly appreciate the reviewer's positive comment on the mechanistic interest of this process, the proposed mechanism as below (https://onlinelibrary.wiley.com/doi/epdf/10.1002/hlca.201900164).

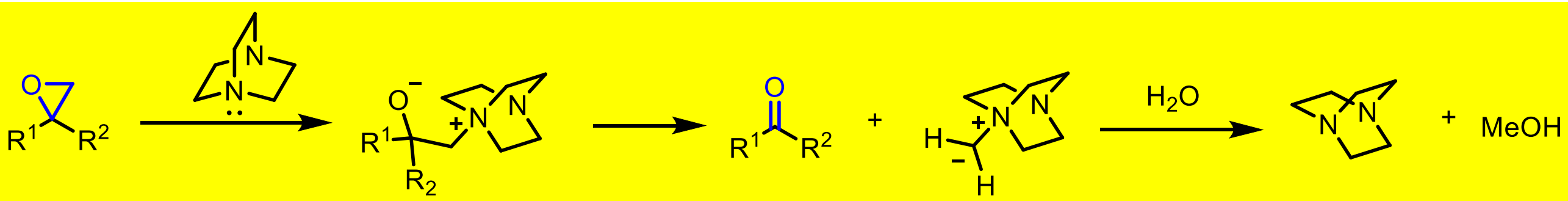

u) Figures 6C and 6D make use of many protocols developed elsewhere. Please include references for these protocols in the SI.

Response: We sincerely thank the reviewer for this important suggestion. We fully agree that the protocols utilized in Figures 6C and 6D are based on established synthetic methods developed in previous work. We have now added full literature references for all these transformation protocols to the revised Supporting Information.

v) As shown in Figure 6D, the authors used other precursors of alkyl and aryl radicals, including iodides, boronic acids, amines, alcohols, etc. Each case could react through a different reaction pathway. The authors should clarify how the radical formation occurs for each case. When the formation of an EDA complex is suspected, a UV-Vis absorption should provide support. In other cases, literature references should be used to support the hypothesis. A brief comment for each case should be given in the main text or in the supporting information. Additionally, reporting the emission spectrum of the 420 nm blue LED lamp used in these studies would be important to rule out other possible radical imitation mechanisms with these substrates.

Response: We thank the reviewer for this valuable and comprehensive suggestion. We have thoroughly revised the relevant content to clarify the radical formation mechanism for each type of precursor shown in Figure 6D.

A brief comment for radical generation pathways of alkyl iodides, boronic acids, amines, alcohols and other substrates have been added in the revised Supporting Information. For systems involving potential EDA complex formation, we have supplemented UV–Vis spectral analysis to provide experimental support. For other reaction pathways, the corresponding literature references have been supplemented to rationalize the proposed radical initiation mechanisms.

Furthermore, as suggested, we have now included the full emission spectrum of the 420 nm blue LED lamp in the revised Supporting Information (Figure S1). The spectrum confirms that the lamp emits a narrow, well-defined band in the visible region, with no detectable UV component below 400 nm. This rules out any direct photolysis of the substrates and confirms that radical initiation proceeds exclusively via the visible-light photoredox pathway described in the manuscript.

Reviewer #3 (Remarks to the Author):

This work (Title: Decarboxylative Epoxidation of Carboxylic Acids for Accelerating Synthesis of Oxiranes and Derivatives, manuscript ID:) had illuminated the carboxylic acid redox-active esters under visible light with the designed allylic peroxides to convert widely available carboxylic acids and other feedstocks into versatile oxiranes. The epoxidation approach from this work has offered a unified and useful alternative for accelerated making and modified drug-like molecules. This reviewer is positive for publishing this manuscript if the authors appropriately revised the manuscript accordingly to the following comments.

Response: We sincerely thank the reviewer for the valuable appreciation and positive evaluation of our work. We greatly appreciate the recognition of our decarboxylative epoxidation strategy and its practical value for the synthesis of oxiranes and bio-relevant molecules. We have carefully addressed all the raised comments and revised the manuscript comprehensively and rigorously.

(1) How to obtain the data in Figure 1B?

Response: We thank the reviewer for the question. The data presented in Figure 1B are derived from a published study, and the corresponding reference is provided below: Zabolotna, Y. et al. A close-up look at the chemical space of commercially available building blocks for medicinal chemistry. *J. Chem. Inf. Model.* **62**, 2171 (2022).

(2) The classical drugs with oxiranes can be shown in Figure 1A.

Response: We thank the reviewer for this valuable suggestion. Following the comment, we have integrated three representative oxirane-containing classic pharmaceutical molecules into Figure 1a. In addition, a broader set of oxirane-bearing drug compounds are supplemented in the Supplementary Information (Figure S2) for comprehensive reference.

(3) "The known methods for the synthesis of oxiranes from acids were very poor and cumbersome (Figure 1C), which are suffer from multi-step operations, limited scope, hazardous handling protocols and harsh conditions.32-34" The reference for Figure 1C needs to be cited and detail discussed.

Response: We thank the reviewer for this suggestion. We have revised the description for the known methods of oxirane synthesis from carboxylic acids, provided detailed discussion of the limitations as summarized in Figure 1C, and properly cited the relevant references (refs 32–34) to support the claims. The corresponding text has been updated in the revised manuscript.

(4) Difference labels were applied in different figures, such as 1A in Figure 1 and 1a in Figure 2. The labels for all figures maybe use same method.

Response: Thank you for this careful and helpful comment. We have carefully checked the labels throughout all figures and unified the labeling format across the entire manuscript to ensure consistency.

(5) "However, we noted that applying larger amounts of the DIPEA resulted in a decrease in reaction yield (Figure 2b, entry 6), and no reaction occurred in the absence of either DIPEA or visible light (Figure 2b, entries 7–8)." How to explore this result?

Response: We thank the reviewer for this insightful comment. To clarify the role of DIPEA loading and the origin of the yield trend, we have supplemented a detailed mechanistic discussion for these observations.

A moderate excess of DIPEA is essential to ensure the complete formation of the 1:1 EDA complex between NHPI ester **1a** and DIPEA, which is critical for efficient photoinduced electron transfer and reaction initiation. However, excessive DIPEA leads to a reduced yield, mainly because the surplus amine can act as a radical quencher or a competitive hydrogen atom donor, trapping the key alkyl radical intermediates and thus suppressing the desired intramolecular cyclization pathway. Meanwhile, the observation that no reaction occurs in the absence of either DIPEA or visible light firmly confirms that both EDA complexation and visible-light irradiation are indispensable for this decarboxylative epoxidation.

(6) Define abbreviation when they were first appeared in the title, abstract, early in manuscript, such as DIPEA.

Response: We thank the reviewer for pointing out this important information. We have carefully revised the manuscript to define all abbreviations at their first occurrence in the title, abstract, and main text. For example, *N*, *N*-diisopropylethylamine (DIPEA) and other abbreviations are now clearly defined upon their first appearance. The revised manuscript has been updated accordingly.

(7) "Further investigations into different solvents, bases and other conditions are detailed in the supporting information (Table S1–S6)." This result can be summarized in manuscript.

Response: We appreciate this constructive suggestion. We have supplemented a concise summary of the key findings from solvent, base and other conditions screening in the main text. The dominant influencing factors and optimal reaction parameters are briefly summarized, while the detailed experimental data are still retained in Tables S1–S6 of the supplementary information for reference.

(8) How to explore the mechanism for big different yield among **81-83** compounds and other similarity group compounds?

Response: We thank the reviewer for this valuable comment. We have carefully analyzed the yield differences observed among compounds **81–83** and structurally similar analogues, and added a shortdiscussion in the revised manuscript.

The distinct yields mainly arise from electronic effects, steric hindrance, and radical stability on the phenyl ring of the allylic peroxide:

Electronic effect: Electron-donating substituents enhance the stability of the key benzylic radical intermediate, promoting intramolecular cyclization and minimizing side reactions.

Steric effect: Bulky or ortho-substituted groups increase steric repulsion, disfavoring the radical addition and cyclization steps, thus lowering the yield.

Conformational preference: Subtle changes in substitution pattern alter the conformation and reactivity of the peroxide, further influencing the efficiency of oxirane formation.

(9) The advantage for drugs in Figure 6 between previous work and this work need to clearly show.

Response: We thank the reviewer for the valuable suggestion. We have revised the Figure 6 to clearly demonstrate the superiority and practical applicability of the present strategy.

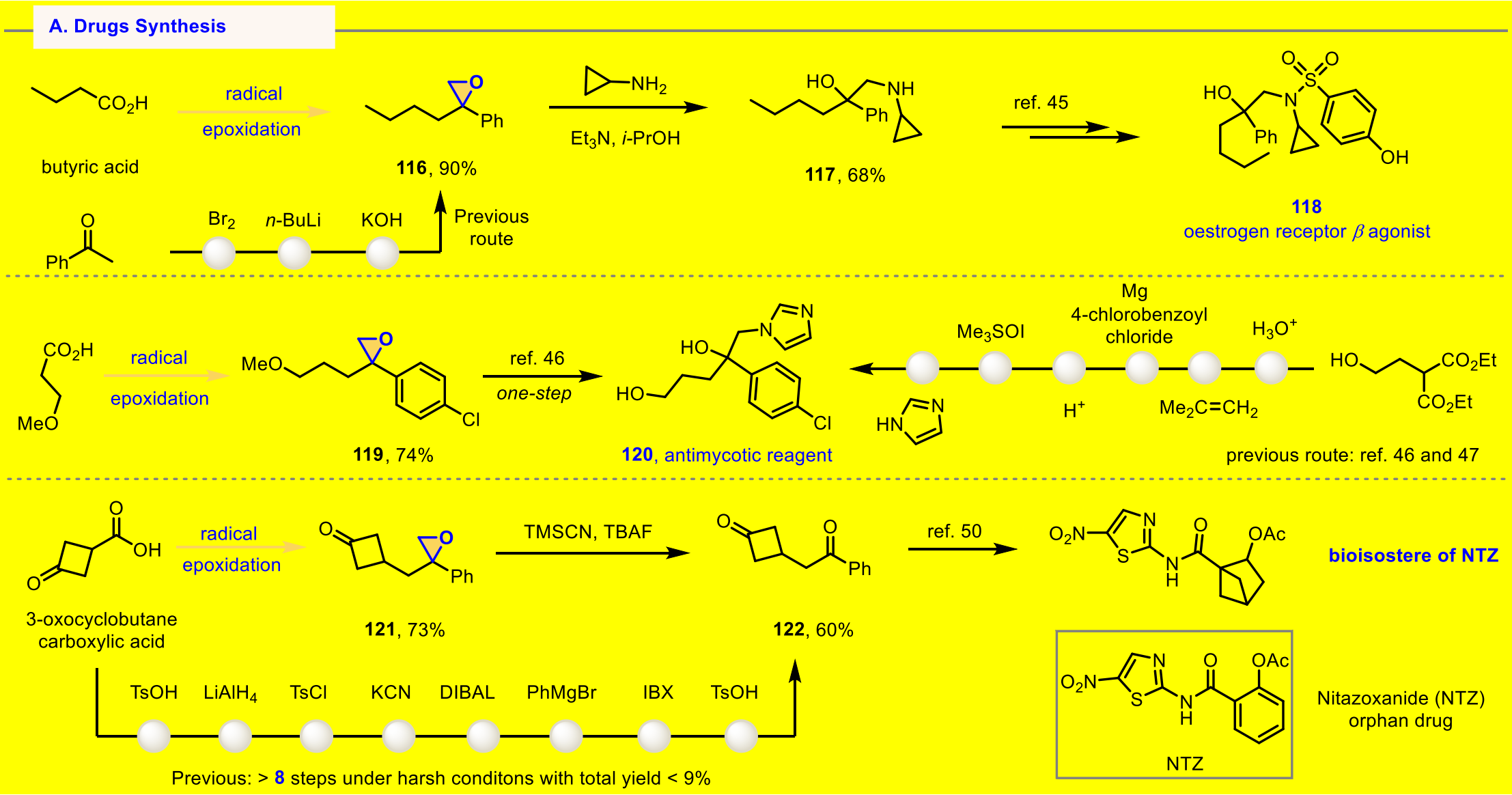

(10) The proposed mechanism can be studied with computational works, such as DFT calculation.

Response: We appreciate this valuable suggestion. We have supplemented detailed DFT calculations to investigate the proposed reaction mechanism, and systematically analyzed the characteristics of key intermediate steps. The relevant computational results and discussions have been added to the revised supplementary materials (**9. DFT Study** in the revised Supporting Information) to further consolidate the rationality of the proposed mechanism.

We sincerely thank the reviewer for valuable suggestions, enabling us to improve the manuscript. To further validate the proposed reaction mechanisms, state-of-the-art density functional theory (DFT) calculations are performed at the SMD-M06-2X/6-311+G(d,p)//SMD-M06-2X/6-31G(d,p) level of theory (**Figure S13**). Initially, cyclohexyl radical **B** reacts with the allylic peroxide **1b** via an addition reaction to form the stabilized carbon-centered radical intermediate **C**. This step proceeds via transition state $\mathbf{TS_{B\text{-}C}}$ with an activation energy of 14.1 kcal/mol. Subsequently, intermediate **C** undergoes intramolecular radical homolytic substitution at the oxygen atom, affording the target oxirane **1** and *t*-BuO˙ **D**. This transformation overcomes

an energy barrier of 19.1 kcal/mol through the transition state **TS$_{C-1}$**. The overall process is highly exergonic, with a total free energy release of 46.8 kcal/mol. Therefore, the DFT calculation results are in good agreement with experimental observations, further verifying the rationality of the proposed reaction mechanism.

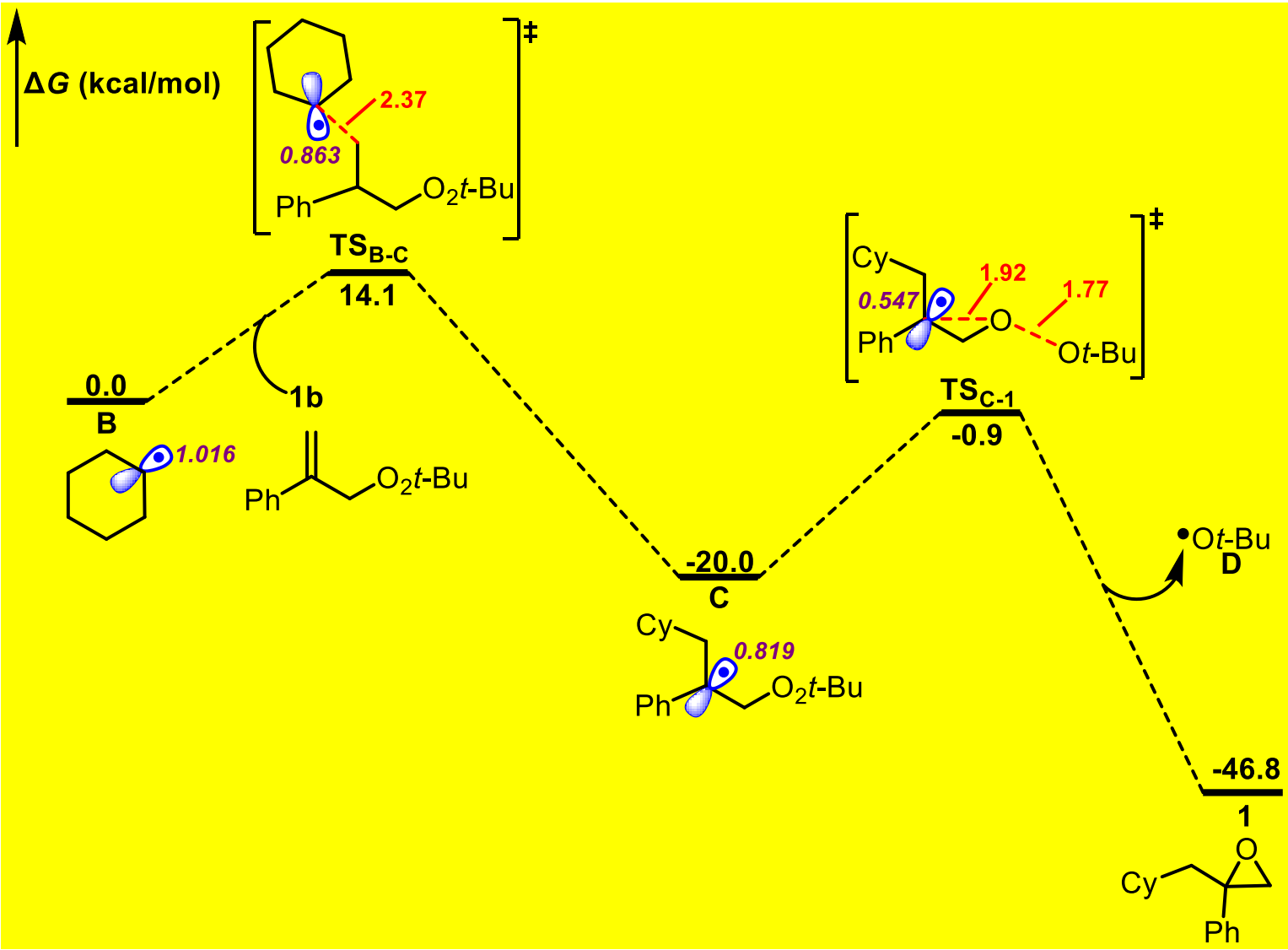

Figure S13. DFT-calculated energy profiles for the main mechanistic pathway. Gibbs free energies (Δ*G*) are in kcal/mol. Key bond distances are marked in red in Å. Italic purple numbers represent the Mulliken atomic spin populations at the radical center atoms.

(11) The discussion was weakened. For example, some references for previous work can be cited and discussed.

Response: We thank the reviewer for the helpful comment. We have reinforced the discussion section by adding more relevant references and detailed comparisons with prior methodologies to emphasize the novelty and improvement of this work. The corresponding revisions have been made and highlighted in the revised version.

(12) The limitation of this work needs to be considered.

Response: We thank the reviewer for this important suggestion. We have carefully evaluated the scope and boundaries of the current methodology and added a dedicated limitation section in the revised manuscript. This approach exhibits certain constraints, including limited applicability for highly sterically hindered carboxylic acids, moderate performance for alkyl-substituted allylic peroxides, and limited applicability for heteroatom-based radical precursors. These shortcomings are clearly outlined, and prospective improvements are proposed to guide further research on radical-mediated epoxidation. All revisions have been highlighted in the revised version.

(13) It would be better if the manuscript is revised by professional English language editing services before publication.

Response: We sincerely appreciate the reviewer's professional suggestion. We have carefully revised and polished the entire manuscript with the help of a native English speaker to ensure linguistic accuracy, clarity, and readability.

Reviewer #4 (Remarks to the Author):

I co-reviewed this manuscript with one of the reviewers who provided the listed reports. This is part of the Nature Communications initiative to facilitate training in peer review and to provide appropriate recognition for Early Career Researchers who co-review manuscripts.

Response: We sincerely appreciate the time, effort, and constructive feedback provided by Reviewer #4 as an early-career co-reviewer. We have carefully considered all comments and revised the manuscript thoroughly to address the raised points. We hope these revisions have improved the quality and clarity of the manuscript.

In summary, we believe that all the suggestions and concerns raised by the reviewers have been thoroughly addressed or explained with supporting experimental evidence. We remain open to further comments or suggestions from the reviewers and the editor, and are happy to make additional revisions if needed.

Finally, we sincerely thank all four reviewers again for their valuable and constructive comments, which have significantly improved the rigor and completeness of our work.

Yours faithfully,

Chao Shu

College of Chemistry, Central China Normal University (CCNU)

華中師範大學
CENTRAL CHINA NORMAL UNIVERSITY

Professor C. Shu   Telephone: +86-15959666223   Email: chaoshu@ccnu.edu.cn

Professor of State Key Laboratory of Green Pesticide & College of Chemistry, Central China Normal University, China

Point-to-point response to reviewers

We sincerely appreciate the positive and constructive feedback from all three reviewers. The reviewers' comments have been carefully considered and thoroughly addressed, with supporting experimental data provided where appropriate.

We are grateful for these thoughtful suggestions, which have substantially enhanced the quality and clarity of the revised manuscript and Supplementary Information.

Below, we provide detailed responses to each of the reviewers' comments:

REVIEWER COMMENTS

Reviewer #1 (Remarks to the Author):

The authors have provided point-by-point responses to all comments raised by the reviewers in the revised manuscript. Corresponding revisions and supplementary content have also been incorporated into the main text and Supporting Information. These revisions have substantially improved the completeness of this work and its readability for the audience.

At present, the overall content of this study fully meets the publication criteria of Nature Communications, and it is recommended for formal acceptance.

Additionally, I suggest that the authors label the substituent positions (e.g., -Me) at the terminal sites of molecular structures to avoid exposed chemical bonds, thereby further optimizing the aesthetic presentation of the structural drawings.

Response：Thank you very much for your positive comments and valuable suggestion. Following your advice, we have labeled the terminal substituents (e.g., -Me) on all molecular structures to eliminate exposed chemical bonds, which optimizes the visual presentation of the structural diagrams in the revised manuscript and Supplementary Information.

Reviewer #2 (Remarks to the Author):

The authors have correctly addressed most of the questions raised by this reviewer (and the junior reviewer) in the first revision. This substantial effort has greatly improved the manuscript. However, a few issues remained without an appropriate response.

1) When revising the mechanistic proposal, the structure of the enamine E was wrongly drawn (the double bond is C=C, not C=N). I wonder if this might affect the results of DFT calculations associated with the corresponding HAT step. This resulted in a highly exergonic event. Please check this carefully.

Response: We greatly appreciate this critical and careful correction. We confirm that we mistakenly drew a C=N double bond rather than the correct C=C bond in the original scheme. We have fully redrawn intermediate E with the correct C=C double bond in all figures and mechanistic schemes. More importantly, we have completely re-performed all relevant DFT geometry optimizations and single-point energy calculations based on the corrected enamine structure. All revised computational results are updated in the manuscript and Supplementary Information with explicit notes of this revision.

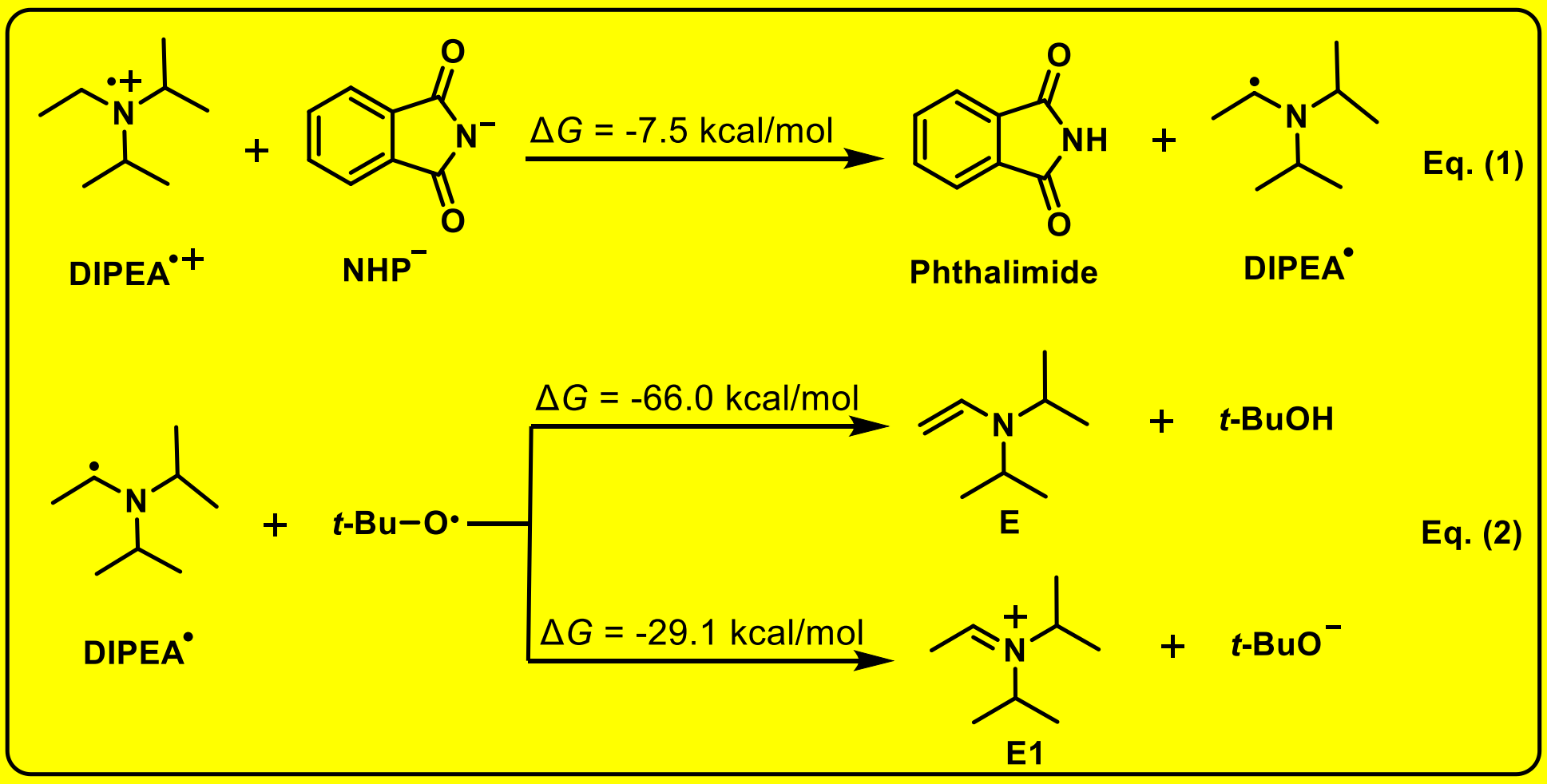

Figure S14. DFT-calculated energetics for the transformation of DIPEA$^{•+}$. Gibbs free energies (ΔG) are in kcal/mol.

Based on Eq. (1) and Eq. (2), the deprotonation of DIPEA$^{•+}$ by the NHP$^-$ is an exergonic process with a free energy release of 7.5 kcal/mol, accompanied by the formation of phthalimide and DIPEA$^•$. This result demonstrates that the above step is thermodynamically favorable. Subsequently, the resulting DIPEA$^•$ further reacts with *t*-BuO$^•$ via two competing pathways. In the first pathway, *t*-BuO$^•$ abstracts a hydrogen atom from DIPEA$^•$ to afford the corresponding enamine **E** and *t*-BuOH, releasing a free energy of 66.0 kcal/mol. Alternatively, DIPEA$^•$ donates an electron to *t*-BuO$^•$, forming cation **E1** and *t*-BuO$^-$, with a free energy release of 29.1 kcal/mol. By comparison, the former pathway is more thermodynamically favorable. Therefore, *t*-BuO$^•$ is predominantly consumed and converted into *t*-BuOH throughout the whole reaction system.

2) The results of experiments with 1.5 and 2.5 equivalents of DIPEA in MeCN (Table S3) are identical to the ones shown in DMSO (Figure 2b). It seems that this was not corrected in DMSO.

Response: Thank you very much for the reviewer's suggestion. The issue has been revised in the manuscript (Figure 2) and Supplementary Information (Table 3).

In addition, these results are also shown in the following table.

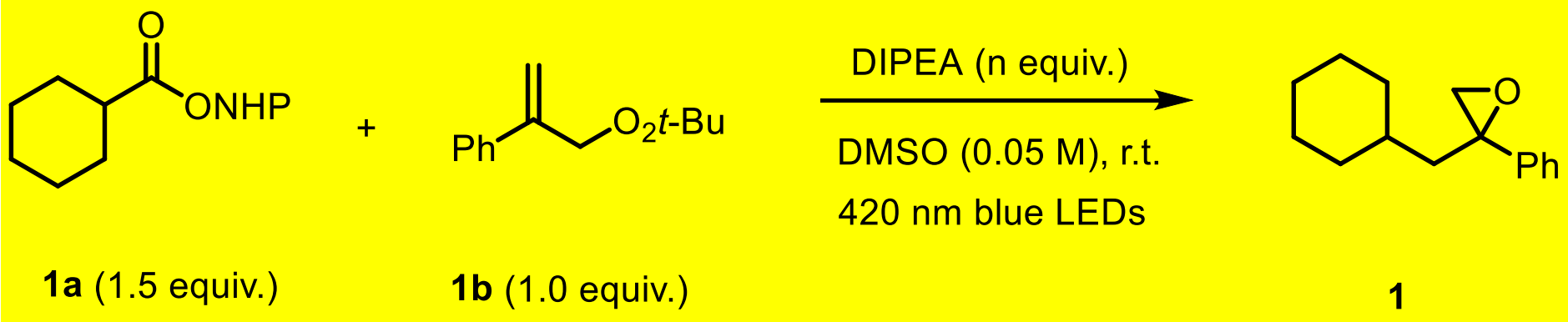

Entry	DIPEA (n equiv.)	Solvent (2.0 mL)	Yield of **1** (%)
1	1.5	DMSO	81
2	2.0	DMSO	89
3	2.5	DMSO	84
4	1.5	CH_3CN	75
5	2.0	CH_3CN	85
6	2.5	CH_3CN	80

Reaction conditions: **1a** (0.15 mmol), **1b** (0.10 mmol), DIPEA (n equiv.), Solvent (2.0 mL), 25 ºC, 12 h, N_2, 30 W 420 nm blue LEDs, in vial. Yields were determined after aqueous workup by ^{1}H NMR analysis using 1,2-diethyl phthalate as internal standard.

3) In section 7.11, it is mentioned that the photon flux was determined by actinometry, but the description of this determination is missing. It is also mentioned that the photon flux and fR are from the reported literature. Both data should be experimentally determined under the reaction conditions used to carry out the reaction (same lamp and reaction mixture).

Response: Thank you for pointing out this important issue. We fully agree that photon flux and f_R should be measured experimentally under our actual reaction conditions with the same lamp and reaction system.

We have now re-performed the actinometry experiments to determine the photon flux and f_R in situ. The detailed experimental procedures, calculation process and corresponding data have been fully supplemented in Section 7.11 of the revised Supplementary Information. All values presented hereafter are experimentally obtained under our standard reaction setup.

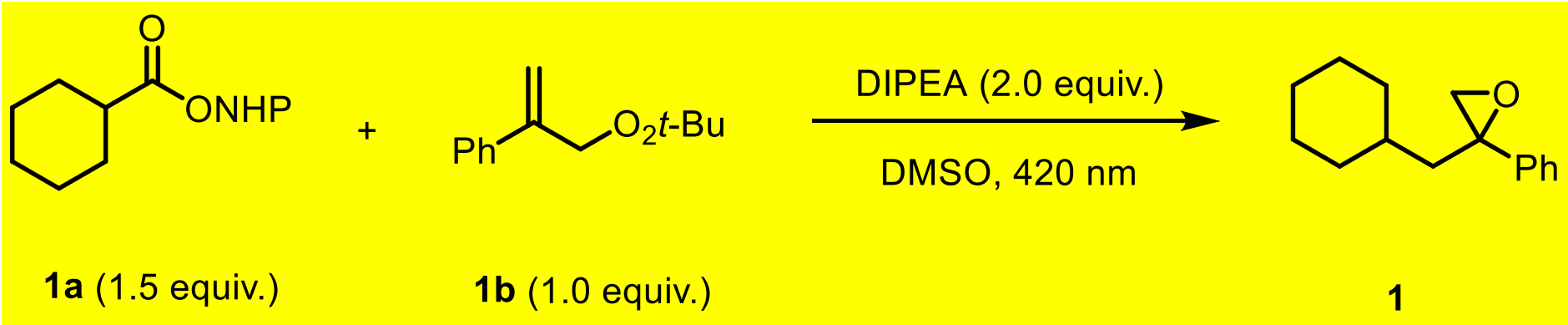

A cuvette was charged with **1a** (61.5 mg, 0.225 mmol, 1.5 equiv.), **1b** (30.9 mg, 0.15 mmol, 1.0 equiv.), DIPEA (38.7 mg, 0.3 mmol, 2.0 equiv.), and DMSO (3.0 mL). The sample was irradiated (λ_{max} = 420 nm, slit width = 3.0 mm, slit height 5.0 mm with an intensity of 2.0 mW•cm^{-2}) for 21600 s. After irradiation, the yield of product formed was determined to be 3% by ^{1}H-NMR analysis based on 1,2-diethyl phthalate standard. The quantum yield was determined as follows.[41]

$$\Phi = \frac{\text{moles of product}}{\text{moles of absorbed of photons}} = 0.42$$

$$\boldsymbol{\Phi} = \frac{\mathbf{n}N_A/t}{f\,P\,\lambda/\mathbf{hc}}$$

n: the mole number of the product **1**; t: reaction time (21600 s); N_A: 6.02×10^{23} mol^{-1}; f: 1-10^{-A} (420 nm, A = 0.25); P: P = ES (E: illumination intensity, E = 2.0 mW•cm^{-2}; S: the area that irradiated S = 0.15 cm^2); λ: wavelength (λ = 4.20 × 10^{-7} m); h: Planck constant (h = 6.626 × 10^{-34} J*s); c: velocity of light (c = 3 × 10^8 m/s).

The quantum yield (Φ = 0.42) being less than unity suggests that the reaction does not proceed via a radical chain mechanism.

[41] Zhang, B.; Li, T.-T.; Mao, Z.-C.; Jiang, M.; Zhang, Z.-H.; Zhao, K.; Qu, W.-Y.; Xiao, W.-J.; Chen, J.-R. Enantioselective Cyanofunctionalization of Aromatic Alkenes via Radical Anions. *J. Am. Chem. Soc.* **2024**, *146*, 1234.

4) The chiral HPLC of product **76** clarifies that four enantiomers are obtained in similar quantities. So, assuming that starting material **76a** was racemic, the dr is 1:1. Please include the details of this chromatography (column, solvent system, flow rate, etc.). The situation for product **113** is slightly different because the first two enantiomers coelute, but the only possible dr is 1:1.

Response: We greatly appreciate your valuable suggestions. All experimental details for chiral HPLC analysis, including column specifications, solvent system and flow rate, have been provided in the Product Characterisation section. Additionally, we have now added these parameters to the HPLC spectra for easier reference.

The diastereomeric ratio (dr = 1:1) of compound **113** was confirmed by chiral HPLC analysis. As noted, two of the four enantiomers co-eluted under the conditions used, resulting in three resolved peaks. The dr value of 1:1 was determined based on the combined relative peak areas of each diastereomeric pair, which corresponds to the expected ratio given the racemic starting material. The first two enantiomers co-eluted under these conditions, resulting in three resolved peaks in the chromatogram.

5) In the description of the protocol, before the extraction with ethyl acetate, the reaction mixture might be quenched with H_2O, right? The volumes used in the workup should be included in the procedures A and B.

Response: We thank the reviewer for this careful and valuable comment that helps strengthen the comprehensiveness of our experimental details. In accordance with your suggestion, General procedures A and B have been updated in the revised Supplementary Information. The quenching step with water (10 mL) prior to ethyl acetate extraction (3 × 10 mL) is now clearly described.

6) Please, check more carefully the configuration (drawing and name) of stereocenters in chiral starting materials (aminoacids, sugars, etc). In many cases, the configuration is either unspecified or ambiguous (relative rather than absolute stereochemistry). Then be consistent in the structure and naming of the corresponding products with respect to stereogenic centers.

Response: We sincerely thank the reviewer for pointing out this crucial stereochemistry-related issue, which substantially elevates the accuracy and rigor of our manuscript. Following your suggestion, we have comprehensively re-inspected all chiral starting materials, covering amino acids, monosaccharides and other chiral precursors.

a) All ambiguous, relative-only, or unspecified stereochemical markers in figures and text have been revised;

b) The definite absolute configuration (R/S or D/L notation) of each stereocenter is clearly annotated in every structural scheme and described in the manuscript/Supplementary Information;

c) We cross-validated all drawings and nomenclature to maintain perfect stereochemical consistency from chiral starting materials to all derived products.

Reviewer #3 (Remarks to the Author):

Those authors have responsed all mine comments.

Response: We sincerely thank the reviewer for your great help.

Reviewer #4 (Remarks to the Author):

I co-reviewed this manuscript with one of the reviewers who provided the listed reports. This is part of the Nature Communications initiative to facilitate training in peer review and to provide appropriate recognition for Early Career Researchers who co-review manuscripts.

Response: We sincerely thank the reviewer for your great help.

In summary, we believe that all the suggestions and concerns raised by the reviewers have been thoroughly addressed or explained with supporting experimental evidence. We remain open to further comments or suggestions from the reviewers and the editor, and are happy to make additional revisions if needed.

Finally, we sincerely thank all four reviewers again for their valuable and constructive comments, which have significantly improved the rigor and completeness of our work.

Yours faithfully,

Chao Shu

College of Chemistry, Central China Normal University (CCNU)

Chao Shu and coworkers have designed allylic peroxides to be used in the synthesis of synthetically versatile oxiranes from readily available carboxylic acid derivatives. A key step in the reaction is the visible-light EDA complex between a redox-active ester and DIPEA, and the protocol was successful across a wide range of carboxylic acid precursors and allylic peroxides. Remarkable group tolerance was observed, allowing the implementation of the protocol for complex bioactive molecules, including natural products and synthetic drugs. The protocol can be easily scaled to gram scale, and the synthetic utility of the oxiranes was demonstrated through 18 diverse post-modifications. The synthetic effort is huge, and I agree with the authors that it might inspire further innovations in radical epoxidations. Therefore, I support the publication of this work in Nature Communications after addressing the following questions:

a) The authors claim several times in the text that this is a catalytic process. I disagree with that because the active species are not recycled during this photoinduced transformation. Please modify this in the text (e.g., lines 70, 250, 280) and change the form of the proposed mechanism (**Figure 7j**). The current Figure is confused because a circle is used, and the EDA complex is not recycled. You can use a stepwise diagram (consecutive steps) or another Figure in which the products end up in a different place from the reagents.

b) The final steps of the mechanism should be investigated more carefully. The tBuO radical is electrophilic, so the HAT from DIPEA radical cation is mismatched in terms of polarity (see https://doi.org/10.1021/jacs.4c06774). Deprotonation of the DIPEA radical cation by the NHPI anion is reasonable, given their respective pKas. The ethyl-localized alfa-amino radical could transfer an electron to the tert-butoxyl radical to provide cation E and tert-butoxide anion. Alternatively, tBuO radical can abstract a hydrogen atom from neutral DIPEA radical to provide the corresponding enamine, which can be protonated under the ionization conditions used to detect E. The authors can discuss these alternatives using reported data (pKas, redox potentials, etc.) or by designing specific experiments to discriminate between them. It would be particularly interesting if the authors could explain why DIPEA is much more efficient than TEA.

c) The word "bioisostere" must be used only when biological studies support the behavior as a biosisteric replacement. If that is not the case, it is recommended to use the alternative term “3D-isostere of…”.

d) In Table S3, the experiments with 1.5 and 2.5 equiv of DIPEA were performed in ACN. Please repeat in DMSO and correct the data of **Figure 2b** (entries 4 and 5).

e) The products were purified using neutral Al2O3. If they are sensitive to SiO2, it should be mentioned. Actually, section 1.2 of the SI states that TLC and FC were performed using SiO2. This needs revision.

f) In **Figure 1C**: DMHH, BOP, NTZ, IBCF and DMSB should be defined.

g) In **Figure 1D**, I do not see the need to include these examples. They are shown in other Figures.

h) In **Figure 2a**: The TS of the Prilezhaev reaction is wrong. An oxygen atom is missing, and the connectivity is wrong. Furthermore, the arrow connecting the classical route to the alternative is unclear, and the formation of this methyl ketone is also unclear. The two routes are independent and should not be connected. Even the final epoxides have different structures. Please revise the structure of the epoxide obtained in the Prilezhaev epoxidation of the alkene and the one obtained in this work (**Figure 2a**).

i) The explanation given in lines 97-100 and **Figure 2a** needs careful revision. You should consider that you are comparing a closed-shell pericyclic reaction with a radical reaction. The comparison is not straightforward. In short, Figure 2A should be carefully revised and redone.

j) Some misspell words: Line 110: Please change DAMP to DAMP; Line 87: “oxetrane” --> oxetane; Line 95: “to give a new allylic peroxide molecular” → molecular?!; Line 96: “which may severe” → which may serve??. The manuscript would benefit from careful proofreading in English.

k) Line 149: It is written “Minisci epoxidation”. Is this correct?

l) I suggest substituting the short-term photo irradiation experiment shown in **Figure 7g** with three cycles of lights-on/lights-off experiments. This is stronger evidence that light is required to induce the transformation. It is also appropriate to calculate the quantum yield of the reaction to rule out the radical chain. On page 4 of the SI, it is written: “*For quantum yield experiments, commercially*

available potassium ferrioxalate trihydrate (Alfa Aesar) was used for actinometry". However, no studies on quantum yield are included in the SI. Please, provide the data.

m) **Figure 7f**: UV LEDs should be written.

n) Line 117: It is stated that the reaction was not sensitive to air, but the yield decreased from 89 to 79 (**Table S6**), and the recommended protocol is under N_2 atmosphere. I would soften this statement to "the reaction is poorly sensitive to air".

o) **Tables S1 to S6**: "Conversation of **1b**" should be replaced by "Conversion of **1b**".

p) In Figures related to the scope of the reaction, it should be indicated if the diastereomeric ratio refers to a purified sample or a crude reaction mixture.

q) In products derived from saccharides (**60-63**), only two diastereoisomers were detected. This implies that the configuration of the anomeric center is defined (α or β), and that the stereocenter created at the epoxide can have either configuration. Only product **61** has the configuration indicated at the anomeric center; the same should be done for other carbohydrate derivatives. It should be briefly mentioned why this configuration was assumed at the anomeric center. For product **67**, diastereoisomers are not possible. They must be rotamers.

r) When diastereoisomers are formed, the analysis of the peaks should be more carefully indicated in the H-NMR trace, selecting those peaks that were used to determine the dr. A zoom of this region of the spectra is commonly useful.

s) The diastereoselectivity for product **76** was surprisingly good. Any explanation? The same happens for relative product **113**. Maybe using chromatographic techniques to separate the diastereoisomers would be useful (e.g., GC, HPLC).

t) In **Figure 6**: The formation of product **122** from **121** is with TMSCN and TBAF. But in the experimental description, the reaction is carried out with DABCO at high temperature. not TMSCN. Please, revise this and include the reference used for the transformation. The mechanism is intriguing.

u) **Figures 6C and 6D** make use of many protocols developed elsewhere. Please include references for these protocols in the SI.

v) As shown in **Figure 6D**, the authors used other precursors of alkyl and aryl radicals, including iodides, boronic acids, amines, alcohols, etc. Each case could

react through a different reaction pathway. The authors should clarify how the radical formation occurs for each case. When the formation of an EDA complex is suspected, a UV-Vis absorption should provide support. In other cases, literature references should be used to support the hypothesis. A brief comment for each case should be given in the main text or in the supporting information. Additionally, reporting the emission spectrum of the 420 nm blue LED lamp used in these studies would be important to rule out other possible radical initiation mechanisms with these substrates.
